# Reappraisal of the DNA phosphorothioate modification machinery: uncovering neglected functional modalities and identification of new counter-invader defense systems

**DOI:** 10.1093/nar/gkad1213

**Published:** 2024-01-02

**Authors:** Siuli Rakesh, L Aravind, Arunkumar Krishnan

**Affiliations:** Department of Biological Sciences, Indian Institute of Science Education and Research Berhampur (IISER Berhampur), Berhampur 760010, India; National Center for Biotechnology Information (NCBI), National Library of Medicine (NLM), National Institutes of Health (NIH), Bethesda, MD 20894, USA; Department of Biological Sciences, Indian Institute of Science Education and Research Berhampur (IISER Berhampur), Berhampur 760010, India

## Abstract

The DndABCDE systems catalysing the unusual phosphorothioate (PT) DNA backbone modification, and the DndFGH systems, which restrict invasive DNA, have enigmatic and paradoxical features. Using comparative genomics and sequence-structure analyses, we show that the DndABCDE module is commonly functionally decoupled from the DndFGH module. However, the modification gene-neighborhoods encode other nucleases, potentially acting as the actual restriction components or suicide effectors limiting propagation of the selfish elements. The modification module's core consists of a coevolving gene-pair encoding the DNA-scanning apparatus – a DndD/CxC-clade ABC ATPase and DndE with two ribbon-helix-helix (MetJ/Arc) DNA-binding domains. Diversification of DndE’s DNA-binding interface suggests a multiplicity of target specificities. Additionally, many systems feature DNA cytosine methylase genes instead of PT modification, indicating the DndDE core can recruit other nucleobase modifications. We show that DndFGH is a distinct counter-invader system with several previously uncharacterized domains, including a nucleotide kinase. These likely trigger its restriction endonuclease domain in response to multiple stimuli, like nucleotides, while blocking protective modifications by invader methylases. Remarkably, different DndH variants contain a HerA/FtsK ATPase domain acquired from multiple sources, including cellular genome-segregation systems and mobile elements. Thus, we uncovered novel HerA/FtsK-dependent defense systems that might intercept invasive DNA during replication, conjugation, or packaging.

## Graphical Abstract

**Graphical Abstract:**
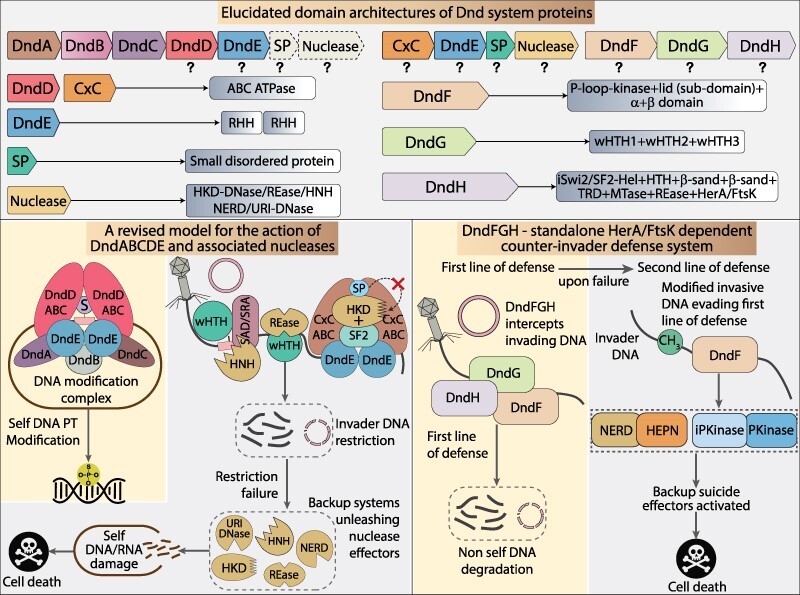

## Introduction

Discrimination of self-vs-nonself by recognizing epigenetic modifications of nucleobases in DNA or the DNA backbone is the mainstay of numerous prokaryotic immune systems that target the nucleic acids of invading selfish elements (i.e. bacteriophages, plasmids, and conjugative transposons) (1–7). The common mode of action for the most prevalent of these systems, the restriction-modification (R-M) systems, involves modifying self-DNA to differentiate it from the unmodified (non-self) DNA of the invader, thereby targeting it for enzymatic degradation (3–8). While the majority of R-M systems modify nucleobases (e.g. cytosine C-5, cytosine N4 and adenine N6 methylation) (3,9–11), the Dnd system, which is sporadically distributed across bacteria and archaea, instead modifies the DNA backbone. Through a multi-step enzymatic process, this system introduces the unique phosphorothioate (PT) modification, in which sulfur replaces the non-bridging oxygen in the DNA phosphate backbone (12–15). The Dnd system is characterized by two distinct operonic associations: the five-gene ‘DndABCDE’ and the three-gene ‘DndFGH’ (also known as DptFGH) clusters, which respectively mediate self-DNA modification and the restriction of invader DNA (12–16).

The DndABCDE operon codes for the components of a complex that includes: (i) DndA, a pyridoxal 5′-phosphate (PLP)-dependent cysteine desulfurase that extracts sulfur from cysteine, acting as a sulfur donor (17). (ii) DndC belongs to the PP-loop ATP pyrophosphatase family and features a 4Fe–4S iron–sulfur center coordinated with three cysteines. Recent research has claimed that DndC binds S-adenosylmethionine (S-AdoMet), which is used for a radical SAM reaction, via its 4Fe-4S cluster (15,18). However, it should be noted that DndC is evolutionarily unrelated to the radical SAM superfamily of enzymes, and the use of S-AdoMet as a substrate is unprecedented in the PP-loop superfamily. DndC possesses a distinct dyad of cysteines, which are likely to receive the sulfur abstracted from the donor by DndA and transfer it into the DNA backbone. The exact interplay between the demonstrated ATP-pyrophosphatase activity of DndC (e.g. in adenylation of the target) and the proposed S-AdoMet substrate in the transfer of the sulfur necessitates further investigation. (iii) DndB, formerly classified within the ParB superfamily of enzymes, is characterized by members exhibiting both nuclease and NTPase activity. (19). Based on wet-lab studies, it has been claimed that DndB functions as an ATP-dependent negative transcriptional regulator, preventing excessive PT incorporation across the self-genome (20,21). However, the possibility of DndB having an additional regulatory role as an NTPase that plays a direct role in limiting PT incorporation or as a nuclease that facilitates access to DNA for the modification machinery, or as a co-effector protecting the system through nucleotide-degradation cannot be ruled out.

The remaining two genes, DndD and DndE, always occur as a linked gene dyad; previous studies have often mischaracterized DndD as an AAA+ ATPase (14,22) and DndE as an uncharacterized globular α+β domain capable of nonspecific nuclease activity independent of PT (23). However, we had previously shown that DndD is an ABC-type ATPase belonging to the coiled-coil assemblage of the ABC superfamily, and DndE is a DNA-binding protein containing two RHH repeats (24). In our earlier study, we also identified a previously unknown sister clade to DndD, named the CxC ABC-ATPase, based on the presence of a ‘CxC’ type Zn-hook at the apex of the coiled-coil, which is absent in DndD. Like DndD, CxC ABC-ATPases are universally linked to DndE, although these DndEs show substantial sequence diversity compared to their counterparts linked to the canonical DndD. The CxC-ABC ATPases are at the core of anti-invader conflict systems that encompass the recently described PbeABCD systems (25). The CxC-DndE pair is also coupled in conserved gene neighborhoods to other genes suggestive of restriction and DNA modification activities, hinting at additional biochemical diversity in the Dnd system (24).

Earlier studies on the proposed three-gene restriction module DndFGH suggested that it is capable of preferentially binding to PT-modified consensus sites in self-DNA, such as 5′-GPTAAC-3′ or 5′-GPTTTC-3′, preventing it from targeting nearby unmodified consensus sites in the genome (16). This binding to PT-modified self-DNA is also said to reduce the ATPase, translocase, and nicking activities of DndFGH, thereby protecting self-DNA (16). The fact that only 10–15% of the GAAC/GTTC consensus sites in the self-genome were identified as PT-modified (using single-molecule real-time (SMRT) sequencing analysis) leaves the open question of how other unmodified consensus sites are left untargeted by DndFGH (26). Previous studies have shown that the deletion of any gene within the conserved DndFGH cluster significantly impairs their restriction ability, whereas the deletion of modification genes results in phage induction, upregulation of DNA repair pathways, and SOS response genes (27). However, these prior studies do not present a coherent picture of the action of the DndFGH complex with respect to its domain architecture and evolutionary history. Recent structural studies suggested that DndF contains a P-loop NTPase domain at its N-terminus, DndG has an HTH domain in its C-terminus, and DndH comprises a REase coupled to a RecA-like domain at its C-terminus (16). However, our initial analysis indicated that these provide an inaccurate account of the domain architectures of the large DndFGH complex, thereby leading to a poor understanding of its biochemistry and mode of action.

Thus, preliminary investigations suggested that there are several unsettled questions regarding the Dnd system as a whole: (i) Is there a phyletic decoupling of DndABCDE and DndFGH, and how do the individual modules function when they occur by themselves? (ii) Why is the modification component often linked to other endonucleases, and do they play a restriction role on their own, if any? (iii) What are the roles of the previously unrecognized domains in the functions of the DndFGH complex? (iv) How do these domains dissociate from PT-modified self-DNA to target and degrade unmodified invasive DNA? In this study, we address these questions through in-depth sequence-structure and comparative genomics analyses, with special attention to previously unrecognized and unannotated domains of the Dnd system.

Consequently, we show that the extended phyletic spread strongly supports the decoupling of the modification and proposed restriction components of the Dnd system in numerous bacterial and archaeal taxa. This indicates the absence of strong selective pressure to retain them together, raising questions about their functional ties. Our systematic delineation of all the domains encoded by DndFGH, along with careful analysis of their conserved features, provides strong evidence that they function as a distinctive prokaryotic immune system on their own. DndFGH independently encompasses domains for restriction, DNA-binding, and ATP-dependent translocase activities. Notably, we show that DndH/DptH has ‘captured’ HerA/FtsK-like DNA translocase domains from diverse cellular and mobile element genomes. Reconstruction of gene neighborhoods also reveals a variety of endonuclease domains coupled to the Dnd modification module in numerous taxa. This suggests potential alternative restriction capabilities in this system. Together, we present a revised understanding of the functions of DndABCDE, the DndFGH and other systems sharing certain diagnostic domains with them.

## Materials and methods

### Sequence analysis

Sequence profile searches were conducted against the NCBI non-redundant (nr) database using the PSI-BLAST (28) (RRID: SCR_001010) and JACKHMMER programs (29) (RRID: SCR_005305), creating PSSM and HMM-based profiles at each iteration. Distant and borderline homologs retrieved from these iterative searches, displaying subtle sequence synapomorphies (shared derived characters) with the query, were used as separate search seeds for independent BLASTP (30) (RRID: SCR_001010) searches against the NCBI NR database. The BLASTCLUST program (ftp://ftp.ncbi.nih.gov/blast/documents/blastclust.html) (RRID:SCR_016641; version: 2.2.26) was employed to cluster the dataset into homolog groups and remove nearly identical sequences. The length of pairwise alignments (L) and the bit-score (S) were adjusted based on the desired level of clustering. To detect and verify remote homologs, sensitive HMM-based profile-profile searches were performed using the HHpred program (31,32) (RRID: SCR_010276). Profile-profile searches were conducted against HMMs derived from PDB (33), Pfam models (34) and a custom database of diverse domains maintained by the Aravind group. HHblits (35) against UniRef30 were used as the default method for MSA generation in HHpred, along with other default parameters. Multiple sequence alignments (MSA) were constructed using Kalign (V2) (36) (RRID: SCR_011810) and MAFFT (37), with manual adjustments based on profile-profile searches against PDB, structural alignments, and predicted 3D structure models (see below).

### Structure analysis

Structure similarity searches were performed using the DaliLite program (38) (RRID: SCR_003047) against the PDB database, clustered at 75% sequence identity. The JPred (V4) program (39) (RRID: SCR_016504) was utilized to predict secondary structures from the generated multiple sequence alignments (MSA). Each MSA served as a reference model to obtain a 3D structure using AlphaFold2 (AF2) (40,41). The predicted secondary structure boundaries from Jpred4 were compared with the AF2 models for verification. AF2-predicted 3D structures were generated separately for each domain identified in this study and used for additional structure similarity searches with the DaliLite program against the PDB database. Structure similarity trees were constructed based on *Z*-scores obtained from an all-vs-all search of the compared structures using average linkage clustering. The figures showing structural visualization and 3D structure rendering were created using PyMOL (http://www.pymol.org) (RRID: SCR_000305).

### Comparative genomics and phylogenetic analysis

Complete taxonomic lineages for all identified sequences in this study were obtained from the NCBI taxonomy database. Contextual information from prokaryotic gene neighborhoods was extracted using a Perl script that retrieves the upstream and downstream genes of the query or anchor gene of interest. The script utilizes GenBank genome files corresponding to unique GenBank genome assemblies (GCA IDs) to retrieve the gene neighborhoods. The protein products were clustered using BLASTCLUST to identify conserved gene neighborhoods based on conservation across different taxa. Each cluster was annotated separately using the aforementioned sequence-structure analysis, and their domain architectures were delineated. Smaller or divergent clusters that remained unclustered as singlets were merged with larger clusters if there was supporting evidence, such as shared sequence motifs, shared structural synapomorphies, reciprocal BLAST search results, and/or shared genome context associations. The annotated cluster file was then mapped to the corresponding genes to reconstruct the overall gene neighborhoods. Additional filters were applied to identify valid neighborhoods for further analysis, including nucleotide distance constraints (typically 100 nucleotides), conservation of gene directionality within the neighborhood, and presence in more than one phylum.

Phylogenetic relationships were inferred using an approximate maximum likelihood (ML) method implemented in the FastTree program (42). Local support values were estimated accordingly. To improve the accuracy of topology, the number of rounds of minimum-evolution subtree-prune-regraft (SPR) moves in FastTree was increased to 4 (-spr 4). The options ‘-mlacc’ and ‘-slownni’ were used to make the ML nearest-neighbor interchanges (NNIs) more exhaustive. Phylogenetic tree topologies were also derived using ML methods based on the edge-linked partition model implemented in the IQ-TREE software (43,44). Branch supports were obtained using the ultrafast bootstrap method (1000 replicates) in IQ-TREE (45). Relationships and clustering of protein families and subfamilies were also determined by computing an all-against-all pairwise sequence similarities as implemented in the CLANS software (46).

### Entropy analysis

Position-wise Shannon entropy (H) for a given multiple-sequence alignment was calculated using the following equation:


\begin{eqnarray*}H = \mathop \sum \limits_{i = 1}^M {P}_i{\log }_2{P}_i\end{eqnarray*}

where *P* is the fraction of residues of amino acid type, *i* and *M* is the number of amino acid types. The Shannon entropy value for the *i*th position in the alignment ranges from 0 (only one residue at that position) to 4.32 (all 20 residues equally represented at that position) (47–49). Analysis of the entropy values which were thus derived was performed using the R language.

## Results and discussion

### Multi-pronged sequence-structure-based searches revise and extend the phyletic spread of the DndABCDE and DndFGH systems

To comprehensively determine the phyletic patterns of all eight Dnd genes of both the modification (DndABCDE) and restriction (DndFGH) modules and revise the previous appraisals of the same, we performed iterative sequence searches using the PSI-BLAST and JACKHMMER programs against the NCBI *nr* database. We did this using all specific domains and proteins previously characterized in the Dnd system as search seeds. All searches were run until convergence or stopped when PSSMs and HMM profiles built in subsequent iterations recovered generic homologous sequences (see methods). The sequences obtained through these searches were clustered using BLASTCLUST, and one representative from each cluster was utilized as seeds for additional searches. Sequences retrieved with borderline statistical significance (e-values between 0.01 and 0.001) that displayed subtle sequence features shared with the query were utilized as seeds for reciprocal searches against the NCBI nr database. These searches broadened the horizon of retrieved homologs of each of the Dnd system proteins. The procedure was repeated until no more clearly delineable members of the family could be added to the collection. For example, a search initiated with DndE exemplar WP_011991365.1 recovered divergent members, and these subsequently served as starting points to recover even more distant versions of DndE (see sections below). Similarly, searches initiated with the previously characterized version of DndF (Genbank accession WP_228949113.1) recovered numerous previously unidentified versions of DndF, and further transitive searches recovered additional DndFs that displayed an array of additional domain fusions in their N-termini (See sections below). This also recapitulated our earlier identification of the CxC type ABC-ATPases, which, when compared to its sister clade DndD, displays considerable sequence heterogeneity and differs from the former in possessing a Zn-hook typified by a CxC motif at the apex of the coiled-coil (24).

We then systematically confirmed through three steps: (i) clustering or aligning the newly identified and divergent candidates with the canonical or previously classified members of the family and scanning them for sequence synapomorphies to confirm membership. (ii) looking for conserved secondary structural elements through sensitive HMM-HMM-based alignments against the PDB database using the HHpred and directly aligning the three-dimensional structures predicted using AlphaFold2 (AF2); (iii) extracting conserved gene neighborhood information for all newly identified candidates and comparing them to known Dnd systems (see methods). All sequences confirmed using the above steps were utilized for further analysis, while those that did not qualify were discarded as false positives. We then made a phyletic assessment based on the unique NCBI tax IDs at the species level rather than the number of genomes in which the Dnd genes were found. Thereby, we avoided the inflation of counts due to multiple genome assemblies in the database for each taxon.

#### Phyletic patterns of dnd genes

**Figure 1.**
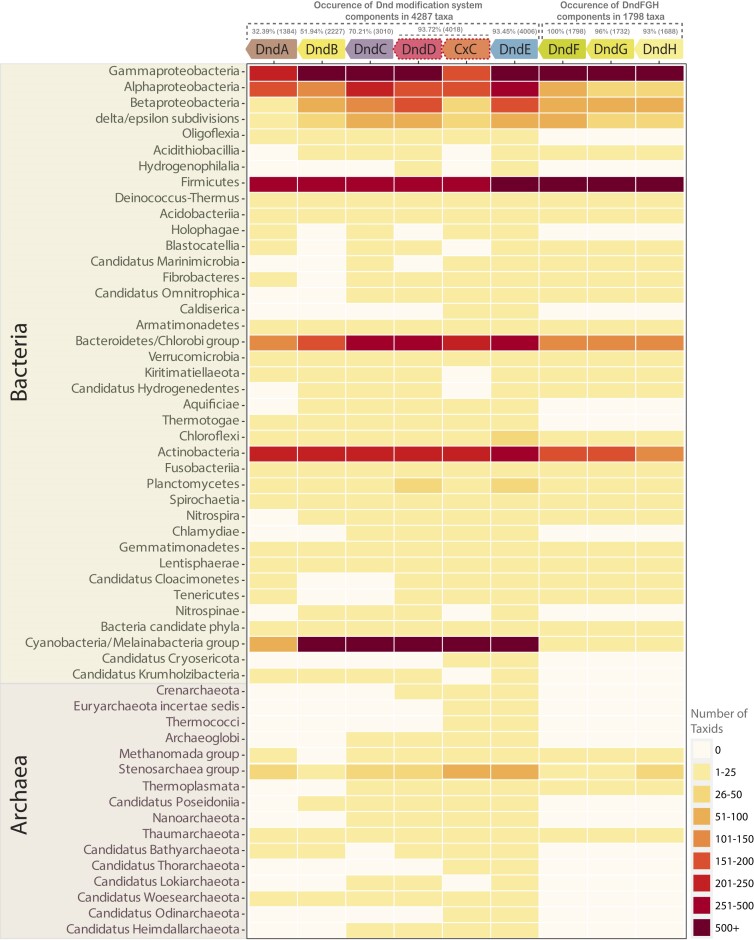
Updated phyletic distribution of Dnd systems. The heatmap shows the overall phyletic distribution of Dnd genes where the boxes are coloured based on the number of unique NCBI tax IDs at the species level against each phylum or taxonomic group shown on the Y-axis. The panel on the right shows the range and the respective colour code. The individual Dnd genes and their occurrence percentage are shown at the top. DndD and CxC ABC-ATPase distributions are presented separately.

In this study, we found that the genes belonging to the Dnd systems are present in 5228 taxa across both bacteria and archaea (Figure 1). Out of these, the components of the Dnd modification and related systems were found in 4287 taxa, while the DndFGH components were recovered only in 1798 taxa. The total number of taxa in which all eight Dnd genes (DndABCDE + DndFGH) are present corresponds to only a fraction of 7% (316 taxa) of all recovered taxa. In the Dnd modification operon, the two genes DndD/CxC ABC-ATPase and DndE form an ‘obligate pair’ representing its conserved core (see sections below). Among the 4287 taxa where the modification component is present, DndD/CxC and DndE are encoded in 4018 and 4006 taxa, respectively (∼93%). Careful inspection of the remaining ∼250 taxa indicated that the failure to recover DndD/CxC and DndE is likely due to the fragmentary nature or poor quality of the corresponding genome assemblies rather than genuine absence. This observation is further supported by the absolute presence of DndD/CxC and DndE in all higher-order taxa (phyla/lineages), as the ATP-dependent Dnd modification component would be nonfunctional without the ABC-ATPase and its conserved ‘DNA-scanning’ operonic partner in DndE (see sections below). As previously reported (50), the other components are underrepresented, with DndC, DndB and DndA occurring in 3010 (70%), 2227 (51%) and 1384 (32%) taxa, respectively. A part of this absence, especially of DndA, might be explained by its substitution by ‘housekeeping’ homologs of the cysteine desulfurase (IscS, NifS, SufS) (15). Indeed, IscS has been shown to be a functional substitute for DndA in *in vitro* reconstitution of the PT modification system (18). However, none of the other widely conserved PP-loop ATPases (ThiI, TilS, CysD) are close (51–54), biochemically equivalent homologs of DndC, raising the possibility that systems lacking this enzyme might rely on an alternative DNA modification or mechanism of actions.

Although the DndFGH module is present in only 1728 (33%) of all analyzed/recovered taxa (5228), it exhibits a contrasting conservation pattern (Figure 1). Unlike the modification component, where two or more genes are underrepresented, the tripartite operonic association in the form of DndFGH is invariably present in almost all taxa in which DndFGH was recovered. Out of the 1798 taxa, DndF is present in all (100%), while DndG and DndH were recovered in 1732 (96%) and 1688 taxa (93%), respectively. The absence of DndG and DndH in only a few taxa is again primarily attributable to incomplete genome assemblies (See Methods and Supplementary Data S1). This indicates a strong selection to retain all three genes of the restriction module, as they function as a large multi-domain complex where each domain plays an indispensable and specific role (see below). For the first time, we uncover the DndFGH system in numerous archaeal taxa, dispelling two earlier views: first, the restriction component is found only in bacteria and not in archaea (25); second, DndFGH does not co-occur with the so-called PbeABCD system (25) (see below for further discussion).

We then compared our appraisal of the Dnd genes with datasets from previous reports and carefully reviewed the overall presence/absence of Dnd genes, correcting instances of faulty overestimation of their distribution in the previous studies (Supplementary Data S1). For instance, while the cysteine desulfurase IscS might potentially substitute for DndA, it is a distinct gene with a clear housekeeping role in cysteine metabolism. Furthermore, IscS genes do not occur in operonic association with other Dnd genes in the genome. Thus, including it in the estimates inflated the overall phyletic distribution of the Dnd system in previous studies (50), as IscS is widespread throughout bacteria. Likewise, we observed an overestimation in the phyletic spread of DndH, resulting from the incorporation of numerous false-positive homologs that solely featured the common C-terminal P-loop ATPase domain. These homologs were devoid of any of the specific N-terminal domains characteristic of DndH (50) (see sections below explaining the domain architectures). Thus, our screen, which accounts for these problems, identified Dnd genes in 40 bacterial and 11 archaeal phyla, including 16 phyla (4 archaea, 12 bacteria) that were not previously reported. At the genus level, we have identified Dnd genes in 440 genera (401 bacterial and 39 archaeal genera) that were not reported in any previous study. In summary, we have revised the previous phyletic distributions and expanded the overall presence of the Dnd system.

### All DndE homologs are typified by two ribbon-helix-helix (MetJ-arc) DNA-binding domains

**Figure 2.**
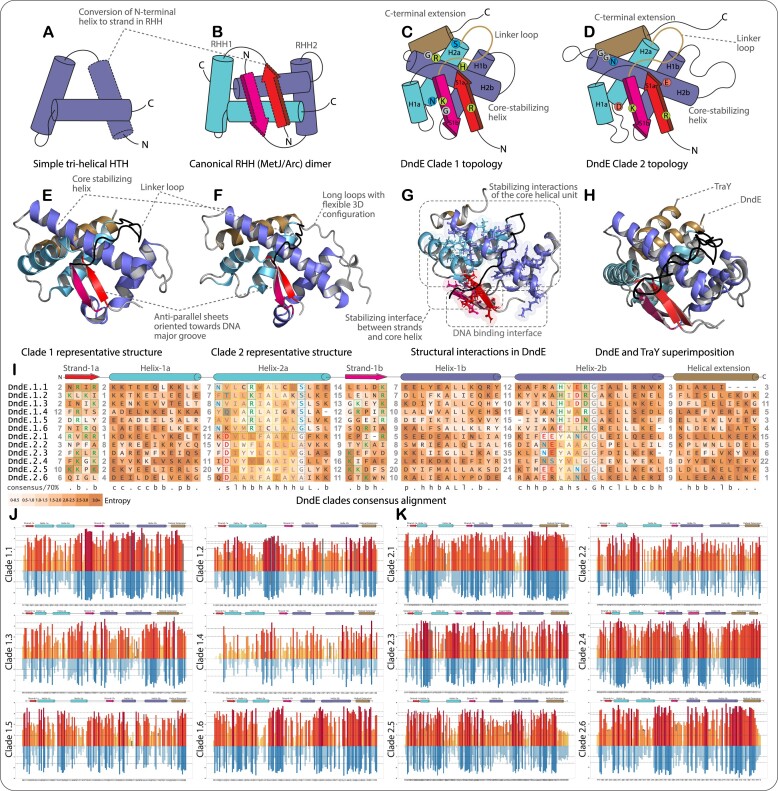
Sequence-Structure synapomorphies of DndE clades. (**A–D**) Representative structure topology diagrams showing key features: (A) a simple tri-helical HTH; (B) a canonical RHH MetJ/Arc-like dimer containing two RHH domains; (C) clade-1 DndE; (D) clade-2 DndE. Key residues on the core stabilizing helix and the antiparallel strands are depicted. (**E–H**) Representative 3D structures showing key structural synapomorphies: (E) clade-1 DndE; (F) clade-2 DndE; (G) ball and stick representation of key residues forming the DNA binding interface and interactions between the strands and the helices in DndE; (H) superimposition of DndE and F-plasmid transfer factor TraY showing identical 3D structural topology; (**I**) multiple sequence alignment showing consensus representative sequence of each DndE subclade where the background color code represents the mean column-wise Shannon entropy values of the corresponding position obtained from separate multiple alignments for each subclade. The entropy of the overall alignment reflects the high sequence heterogeneity and fast-evolving aspects of DndEs. Key residues that are moderately conserved within each subclade are color-coded based on their properties: positively charged (green); negatively charged (red); polar (light blue); hydrophobic (yellow); and small (grey). (**J**, **K**) Subclade-wise entropy plots of DndEs. Shannon entropy data, computed using the regular 20 amino acid alphabets, are depicted above the zero line in orange shades. Shannon entropy data, computed using a reduced 8-residue alphabet (based on chemical properties), are displayed below the zero line in blue shades. High entropy in both alphabets indicates potential positive selection for amino acids with diverse chemical characteristics at those positions.

While we had previously shown that the DndE family is characterized by two tandem copies of the DNA-binding ribbon-helix-helix (RHH) domains (24), recent studies in the Dnd field see DndE as a single globular *α*+*β* domain comprising two strands and five helices or as an ordinary helix-turn-helix (HTH) domain (23,55). The latter interpretations of these proteins have led to a suboptimal understanding of the structural basis for the DndE function (23,55,56). To remedy this, in the current work, we present an in-depth analysis of this family. The RHH domain is a variant of the classical tri-helical HTH domain where the first helix has transmogrified into an N-terminal β-strand, resulting in a ‘strand-helix-helix’ configuration (57) (Figure 2A, B). Consequently, single RHH domains obligately homodimerize via their β-strand through the formation of an antiparallel β-sheet. Unlike conventional HTH domains, this two-stranded sheet forms the primary DNA-binding interface via insertion into the major groove (57–59). By analyzing over eight thousand DndE sequences identified in this study, we validate that all DndEs contain two tandem copies of the RHH domain in the same polypeptide. We further confirmed this through the analysis of previously known structures and all AF2-predicted structures of DndE. To differentiate between the two units, we denote the secondary structures as ‘S1a-H1a-H2a’ and ‘S1b-H1b-H2b,’ where the subscripts ‘a’ and ‘b’ indicate the first and second RHH units, respectively, and letters ‘S’ and ‘H’ refer to strands and helices (Figure 2C–G). The two RHH units in DndE are connected by a long variable linker, between 10 and 20 residues in length (Figure 2C–G, Supplementary Data S2-S3). The DndEs are also characterized by an elongated H2b that forms the stabilizing core of the structure. Further, most DndEs possess a C-terminal helical extension beyond H2b, which potentially facilitates protein-protein interactions with its obligate ABC ATPase partner (Figure 2C–G).

While most proteins have a single RHH domain per polypeptide (e.g. Arc, MetJ, CopG) (58), structural comparisons revealed that the duplicated architecture followed by a C-terminal helical extension seen in the DndE family is also present in the F-plasmid transfer factor TraY (Figure 2H). TraY binds to the origin of transfer (oriT) to facilitate plasmid DNA-nicking by the plasmid-encoded endoDNase prior to conjugative transfer (60,61). This might be paralleled in the proposed role of DndE in DNA nicking and its preference for binding to nicked DNA (23). However, our analysis indicates that DndE does not conserve any residues suggestive of enzymatic activity and is unlikely to catalyze endonuclease activity by itself. Consistent with this, the currently proposed PT modification catalyzed by purified DndCDE appears to bypass nicking of the DNA (18,62). The presence of two tandem copies of the RHH domain in DndE enables it to function as a monomer, adopting a three-dimensional configuration and structural equivalence to dimers of the canonical RHH superfamily members with a single DNA-binding domain per polypeptide. Keeping with this, TraY also exists as a monomer in solution and binds DNA in this configuration (58,60,61). Based on these observations, we propose that one copy of DndE associates with its ABC ATPase partner and recognizes the target sequences akin to TraY binding oriT sites. Since the ABC ATPases themselves obligately dimerize via their coiled-coil inserts, the minimal functional unit can be reconstructed as containing two DndE and two ABC ATPase subunits similar to the Smc-ScpB/Kleisin complexes (63,64).

### The diversification of DndE parallels their operonic pairing with the two ABC-ATPase clades

**Figure 3.**
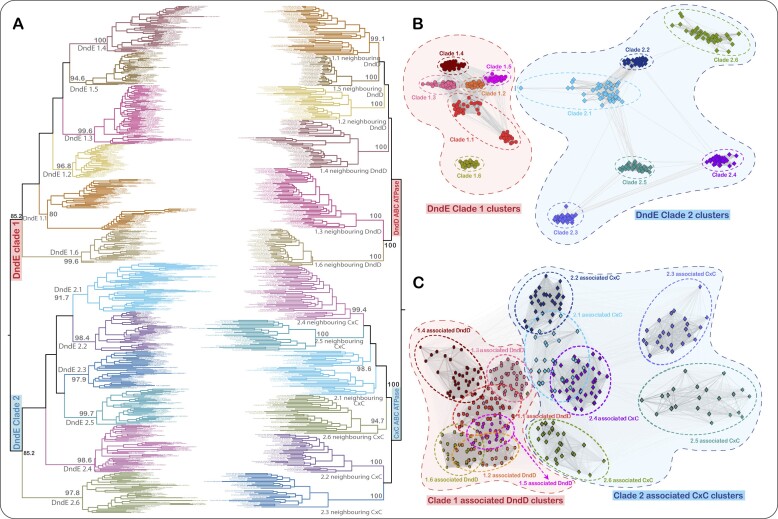
Classification and congruent evolutionary trajectories of the DndE and DndD/CxC pairs. (**A**) Maximum-likelihood tree topologies of DndE and DndD-CxC are shown as mirror trees. The tree on the left shows the grouping of DndE sequences into two major clades (Clade-1 and Clade-2) and multiple subclades. Likewise, the tree topology on the right clearly distinguishes the operonically linked DndD and CxC into two major clades and multiple subclades, corresponding to Clade-1 and Clade-2 DndEs, respectively. Percentage bootstrap values obtained using 1000 replicates are shown for each major node. (**B**, **C**) CLANS-based clustering using the pairwise ‘one-to-one’ alignment-based approach precisely reproduces the grouping of DndE and DndD/CxC, respectively, into major clades and subclades as shown in the phylogenetic tree topologies.

Based on our tracing of sequence-structure synapomorphies of all identified DndEs, we found that the DndEs can be broadly categorized into two major clades, which are perfectly congruent with their genomic association with the DndD and CxC clade ABC-ATPases. Accordingly, we term the DndEs associated with the DndD ABC-ATPases as ‘clade-1' and those paired with the CxC clade ABC-ATPases as ‘clade-2' (Figures 2C–F, 3). At the sequence level, clade-1 and clade-2 DndEs can be distinguished by multiple prominent sequence signatures (Figure 2I, Supplementary Data S2 and S3). At the structural level, both clade-1 and clade-2 DndEs are distinguished by the lengths of specific loops and helices. For instance, in clade-1, the loop connecting the helices H1a and H2a ranges between 3–5 residues, whereas in clade-2, the same loop is an average of 15 residues and displays a flexible conformation (Figures 2C–F, I, Supplementary Data S2 and S3). Similarly, the loop connecting H1b and H2b is shorter in clade-1 (average 7 residues) and much longer in clade-2 (average 14 residues). The length of the terminal extension beyond H2b, including the C-terminal helix, is relatively short in clade-1 (17 residues overall with a C-terminal helix of 10 residues) compared to clade-2 (29 residues overall and a C-terminal helix of at least 19 residues) (Figure 2C–F, I, and Supplementary Data S2 and S3). This suggests that the clade-1 DndE is much more compact and closer to the ancestral state than the clade-2 DndE with multiple long flexible regions.

The DndEs of each of the two major clades, clade-1 and clade-2, can also be further classified into subclades displaying specific synapomorphies, and each of these displays high sequence heterogeneity as observed in the Shannon-entropy analysis (47–49,65) (Figure 2I–K). BLAST-score-based clustering, followed by a thorough visual inspection of the alignments of each cluster, allowed us to categorize clades 1 and 2 into 6 subclades each, referred to as clade-1.1 to 1.6 and clade-2.1 to 2.6 (Figure 2I–K). We tested the validity of this classification by two independent analyses. First, we used a representative subset of all DndE sequences from each subclade and performed maximum-likelihood phylogenetic analysis using the IQ-TREE software (Figure 3A). Second, we performed clustering using the CLANS software with the BLOSUM62 matrix (Figure 3B-C). The tree topology completely recapitulated our original subclade classification, with clades 1.1 to 1.6 and clades 2.1 to 2.6 forming well-supported groups in the tree (Figure 3A). Similarly, the pairwise ‘one-to-one’ alignment-based approach, as implemented in the CLANS software (46), reproduced an identical clustering of DndEs as the inferred tree topology (Figure 3B, C).

We next asked if the coupled ABC ATPases also exhibit congruent evolutionary trajectories at the subclade level. We addressed this using our gene neighborhood collection to obtain the corresponding DndD and CxC clade ABC ATPases that occur in tandem in the genome with the DndEs. These were then subjected to both phylogenetic tree construction and score matrix-based clustering analysis. Both the inferred tree topology and CLANS-based approach clustered the ABC ATPases into two major clades: the DndD and CxC clades, each with distinct subclades precisely corresponding to the DndE subclades (Figure 3A–C). These observations strongly support our above proposal that the DndE and ABC-ATPases (DndD/CxC) function in a DNA-manipulating complex similar to other members of the ABC superfamily. It also suggests that these two components form the functional core of these systems, to which the other regulatory, modification and restriction components are recruited, namely DndA, DndB and DndC in the canonical Dnd systems and the HKD + SF2-helicase, DNA methylase and the SP in the CXC-ABC ATPase-based systems.

### The DNA-binding interface of the DndE family displays high sequence variability suggesting a role in target specificity

To understand the DNA-binding interface of DndE, we systematically surveyed the literature to gather information on the DNA-contacting positions and residues of all characterized RHH domains. While most of these positions are primarily located in the antiparallel sheet, a few residues from the second helix of each RHH subunit also contact DNA as in the ancestral HTH domain (59,60,66–68). The primary residues engaging the major groove from the antiparallel sheet are a conserved arginine and/or a lysine, which simultaneously establish electrostatic contacts with the phosphate backbone of the DNA and engage in cation-π interactions with the bases, exhibiting a preference for guanine and adenine, respectively (59). Furthermore, polar residues, e.g. asparagine and glutamine (such as in MetJ and Arc) from the antiparallel sheet form hydrogen bonds with the acceptor/donor groups of adenine or guanine (59,66–69). However, unlike classical RHH homodimers, where both strands are identical, the two RHH domains of DndE are divergent, allowing for a greater asymmetry in the bound target. In line with this observation, one of the characterized PT modification sites takes the form GAAC/GTTC, with the modification occurring between G–A and G–T on the respective strands.

We found striking sequence variability in the DNA binding interface of the DndE family (Figure 2I–K, Supplementary Data S2 and S3). First, there is no universally conserved residue across all DndEs in both copies of the RHH domain. However, a motif with a dyad of basic/polar residues separated by a hydrophobic residue is observed in S1a of at least six DndE subclades from both clade-1 and clade-2. The second of these polar positions tends to be an arginine in at least 85% of the exemplars with this motif, and collectively, at least one positively charged residue (mainly arginine) tends to be retained in S1a (Figure 2I, Supplementary Data S2 and S3). Thus, the first RHH domain might at least recapitulate one of the pivotal DNA interactions seen in the classical RHH domain transcription factors. S1b tends to be more divergent than S1a. However, several subclades show their own private conserved motifs with multiple charged or other hydrophilic residues that are suitably positioned to contact DNA. In most cases, at least one polar residue (usually R, K, N, Q, D, T) tends to be directed in a manner favoring major groove interactions. Additionally, both S1a and S1b feature partially conserved aromatic residues that are likely to engage in π-π interactions with the base. On the whole this is compatible with the purine-preference observed in the target sites of previously studied RHH domains and is consistent with both the *de novo* and the subsequent modification on the opposite strand occurring at sites with a guanine in currently characterized PT systems (15,18). Nevertheless, the overall observed diversity in the DNA-binding interface of the DndE family implies that different subclades recognize distinct DNA motifs (Figure 2I, Supplementary Data S2 and S3). As a corollary, this would indicate that the target motifs for PT and other modifications are likely to differ across the subclades of the systems, comparable to the target motifs seen in R-M systems (3,4,70–72).

### Duplication of the ABC ATPase-DndE pair, association with new functional partners, and subsequent re-coupling drove diversification of dnd modification and related systems

**Figure 4.**
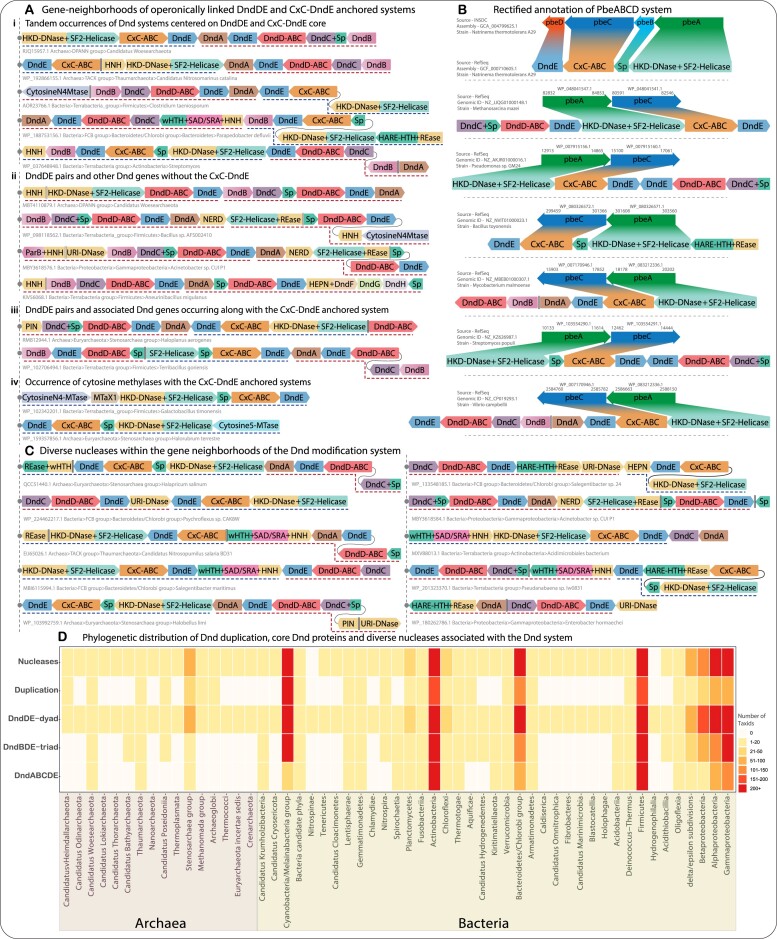
Gene-neighborhoods and phyletic distribution of operonically linked DndDE and CxC-DndE anchored systems and the rectified annotation of PbeABCD system. (**A**: i–iii) Representative gene-neighborhoods showing the tandem or immediately adjacent occurrences of (i) Dnd systems centered on DndDE and CxC-DndE core; (ii) DndDE pairs and other Dnd genes without the CxC-DndE pair; (iii) DndDE pairs and associated Dnd genes occurring along with the CxC-DndE anchored system. Gene neighborhoods are shown as box arrows, with the direction of each arrow pointing to the orientation of individual genes. The domain names and the domain architectures of each gene product are shown within box arrows and are colored accordingly. The dotted lines at the bottom mark the boundaries of the DndDE (red) and CxC-DndE (blue) anchored systems. Each domain is assigned a unique color, with nuclease domains highlighted in the same color for clear distinction and prominent visibility within the neighborhoods. SP in the gene-neighborhoods denotes the small disordered protein (A: iv) Representative gene-neighborhoods showing the occurrence of cytosine methylases with the CxC-DndE anchored systems. (**B**) Rectified annotation of the so-called pbeABCD system. Gene-neighborhoods were reconstructed using the same assembly as utilized in the earlier study and are mapped to the CxC-centered system. (**C**) Representative gene neighborhoods showing the occurrence of diverse nucleases within the gene neighborhoods of the Dnd modification system. D) The phyletic distribution of various contextual connections and occurrences of Dnd modification systems across different phyla or taxonomic groups. The Y-axis shows different categories: (i) the complete DndABCDE operon; the DndBDE triad while lacking DndA and DndC; only the DndDE dyad being present without other PT modification genes. The ‘Duplication’ category includes instances where DndD and CxC anchored systems are found sequentially within the same genome, as indicated in panel A. The ‘Nucleases’ category encompasses all occurrences of multiple distinct nucleases associated with the Dnd modification systems. As shown in Figure 1, the boxes are color-coded to represent the number of unique NCBI tax IDs at the species level within each phylum or taxonomic group.

Based on the comprehensive evaluation of the gene neighborhoods, we made two notable observations (Figure 4A, B). First, the so-called PbeABCD system, recently claimed to be an alternative for the DndFGH system, is the same as the CxC ABC ATPase-anchored system with its cognate DndE, which we had previously reported (24). Second, these are frequently located in genomes alongside the canonical Dnd systems anchored on the DndD-DndE pairs and the associated PT modification genes, resulting in a ‘mix and match’ of one or more Dnd systems with the CxC ABC ATPase-anchored systems (Figure 4A: i–iv).

In earlier work, we demonstrated that the CxC ABC ATPase-centric systems, in addition to its cognate DndE gene, include a distinct set of genes encoding: (i) an endoDNase of the HKD superfamily fused to a superfamily-2 (SF2) helicase module that resembles the restriction component of the Type-I/III-like ATP-dependent R-M systems (73,74); (ii) a rapidly evolving, mostly disordered small protein (SP) that we earlier predicted to function as a negative regulatory component or an interactor with viral triggers (Figure 4A: i–iii); (iii) some of these neighborhoods additionally code for DNA cytosine-5 and/or circularly permuted cytosine-N4 methylases (24); (vi) The systems with methylases often feature a gene coding for an MTaX1 domain potentially involved in modified DNA-discrimination that is also found in several R-M systems (24) (Figure 4A: iv). Parallel to our report of the CxC ABC ATPase-centric systems, Xiong and colleagues described a subset of these linked to the canonical Dnd genes as a new immunity system named PbeABCD (25) (Figure 4B). We herein clarify that PbeA is the aforementioned HKD endoDNase fused to a SF2 helicase module, PbeB is the rapidly evolving small protein, and PbeD is the DndE cognate. PbeC is the CxC-type ABC-ATPase that was misidentified in that study as an SMC-type ABC-ATPase (Figure 4B). It should be noted that instead of the Zn-hook found in the CxC-ABC, the SMC-type ABC-ATPases are typified by a hinge domain at the apex of the coiled-coil, and the average length of their coiled-coil region (800–1000 residues) is almost twice the length of the coiled-coil segments usually found in DndD/CxC ABC-ATPase clades (400 residues) (24).

While the canonical DndD-containing systems are more widespread than the CxC ABC ATPase-containing systems, our survey identifies those systems centered on DndD and the CxC-type ABC ATPase, each with its cognate DndE homolog, occur tandemly in the genomes of 936 bacterial taxa (spread across most bacterial clades) and 41 archaeal taxa (from across 4 major archaeal clades) (Figure 4A, C, D). Together, these constitute 22% of all taxa in which a Dnd modification system is present. Interestingly, we also found adjacent DndDE pairs occurring in the opposite orientation, with one of the pairs usually additionally containing DndB. These are found across 140 taxa from various bacterial phyla and one archaeal taxon. Notably, none of these taxa contains an adjacent system centered on a CxC-ABC ATPase. In 39 taxa distinct from the above, we found that the duplicated DndBDE/DndDE gene clusters occur along with a CxC ABC ATPase-DndE pair in between them (see Supplementary Data S1 and Figure 4A, D). These observations point to functional cooperation between distinct DndBDE/DndDE and CxC ABC-ATPase anchored systems.

The above observations suggest that, in addition to dissemination via lateral transfer across prokaryotes, these systems have undergone duplication and genomic re-association on more than one occasion: (i) an ancient duplication event involving an ancestral gene that encoded an ABC ATPase with a coiled-coil insert coupled with a DndE-like gene. This led to the formation of the core DndD and CxC systems. (ii) These systems then independently associated with distinct genes for the small protein, endonuclease and/or modification enzymes and the ParB superfamily enzyme DndB, resulting in the separate CxC- and DndD-centric systems. (iii) The DndBDE system also underwent a separate duplication event, resulting in pairs of this system oriented in opposite directions, with one copy losing the DndB component. (iv) In some instances, a CxC ABC ATPase system was inserted within the duplicated DndBDE/DndDE systems.

Further, our observations call for a reinterpretation of the possible mode of cooperative action of the canonical DndD and CxC-anchored systems. Contrary to previous reports, our analysis reveals that in many instances, the DndFGH system is encoded by the very same genomes with a DndCDEA coupled to the CxC-centric (the so-called PbeABCD) system (Supplementary Data S1 and Figure 4B). Conversely, as noted above, the CxC-anchored systems (PbeABCD) also occur independently of the DndD-centric system in numerous bacterial taxa. In these cases, they might occur with or without a co-occurring DndFGH operon in the same genome. These observations raise serious questions regarding the proposal that the so-called PbeABCD (CxC-based system) functions as a replacement restriction module for DndFGH. It was also proposed that the so-called PbeABCD module (the CxC-centric systems) operate via an enigmatic mechanism wherein no direct cleavage or degradation of the viral DNA occurs (25). However, we observed that the vast majority of these systems contain an HKD-DNase fused to the SF2 helicase (PbeA), which argues for the cleavage of DNA. Hence, we propose that nucleic acid cleavage is indeed part of the action of the systems. This could restrict the virus via two alternative processes: first, through an attack on viral DNA that has gone undetected. In this proposal, the modification component (PT or methylation), found in a large fraction of these systems could facilitate self-nonself discrimination for specifically targeting viral DNA. Alternatively, the observed restriction of the virus could proceed via cleavage of self DNA by the associated endonuclease, resulting in cell-suicide or apoptosis, thereby preventing its proliferation at the population level. In support of the latter model, we point to the small, mostly disordered protein (cognates of PbeB) that could act as a negative regulator of the system that is released by cleavage or modification by viral enzymes, unleashing the endoDNase component. Indeed, in both these processes, the ABC-ATPase-DndE pair could scan the DNA for specific target sequences. We present further evidence for the role of both DNA modifications and suicide effector actions in these systems below.

### Gene-neighborhoods centered on the ABC-ATPase-DndE core incorporate diverse nucleases

Our comprehensive collection of the gene neighborhoods centered on the ABC-ATPase-DndE core revealed the incorporation of an underappreciated diversity of nucleases alongside the Dnd modification components (Figure 4C, Supplementary Dataset S1). Interestingly, these include endoDNases on one hand, viz., HKD-endoDNase, HNH, multiple REase families (e.g. the previously defined NERD), and URI (the UvrC-Intron homing endonuclease superfamily, overlapping with Pfam: GIY-YIG) and endoRNases on the other, viz., HEPN and PIN (75–77). A subset of the endoDNases tends to primarily occur immediately downstream of the Dnd modification genes. These endoDNases are typically the aforementioned HKD fused to an SF2-helicase module, an HNH endoDNase domain fused to N-terminal winged HTH (wHTH) and SAD(SRA) domains, and a REase domain similarly fused to a HARE-HTH domain which recognizes DNA with modified bases (78) (Figure 4C). The HNH nucleases fused to SAD(SRA) domains were originally predicted to recognize epigenetic modifications in DNA, such as 5-hydroxymethylcytosine (5hmC) (10). This paradigm is illustrated by TagI, a SAD(SRA)+HNH family restriction enzyme targeting modified DNA (79). In the version found in the Dnd systems, the above three-domain HNH protein has been proposed to specifically recognize PT modification via its so-called N-terminal sulfur binding domain (SBD) (80). Rather than being a novel domain, the SBD corresponds to the wHTH domain that we identified. While the SAD(SRA) domain might augment DNA-binding by the SBD, we suspect that it might also additionally recognize other modifications, such as 5mC or 5hmC specific to the viral DNA. We propose a similar modification-discriminating role for the HARE-HTH fused to the REase domain found in several of these systems (Figure 4C).

In contrast to these endoDNases, the linked genes coding for URI, certain standalone REase domains (e.g. NERD), and the endoRNases tend to be encoded at the boundaries of the core systems and sometimes on the opposite strand. Thus, we propose two distinct roles for the Dnd-associated endonucleases: first, the endoDNases fused to additional modified DNA-binding domains or the SF2-helicase module are likely to discriminate modified DNA versus unmodified DNA and function as *bona fide* restriction enzymes. Second, the standalone endoDNases and endoRNases are likely to serve as backups that act as suicide effectors that indiscriminately target DNA or RNA upon failure of the main Dnd system due to inhibitory mechanisms deployed by the invasive elements (77,81,82). Collectively, at least one endonuclease is present in 3416 out of 4287 unique taxa (80%) in which the modification component was identified (Figure 4D, Supplementary Dataset S1). Among these 3416 taxa, only 806 possess the DndFGH module, raising the possibility that a subset of these endonucleases from the modification neighborhoods serves as the genuine alternative restriction components. Consistent with this, among the 698 taxa where the wHTH + SAD + HNH was encoded in the Dnd locus, only 197 possess the DndFGH module (Figure 4D). The simplest proposal for the action of these restriction components would be that they discriminate between host DNA modified by PT or 5-methyl/N4-methyl cytosine marks and invader DNA with no modifications or its own virus-specific modifications to cleave the latter. However, more complex modes of action, such as suicide action on self-DNA triggered by detection of the virus, are also possible, as proposed above, for systems with the HKD endoDNase fused to an SF2 helicase module.

### Apprehending the organizational and functional logic of the DndFGH system

Given the above observations suggesting that the DndFGH system is frequently decoupled from the Dnd modification system, we next sought to understand better the origin and function of this system. Our preliminary analysis also indicated that despite recent structural studies, there are several lacunae in understanding the architecture of the proteins in this system. Hence, we conducted a comprehensive sequence-structure analysis of these proteins to properly characterize their domain architectures, variability and evolutionary histories. We first consider DndG because it tends to retain a constant, relatively simple architecture in all exemplars of the system, followed by DndF and DndH, which are larger proteins with unusual architectural features or variability.

### DndG

**Figure 5.**
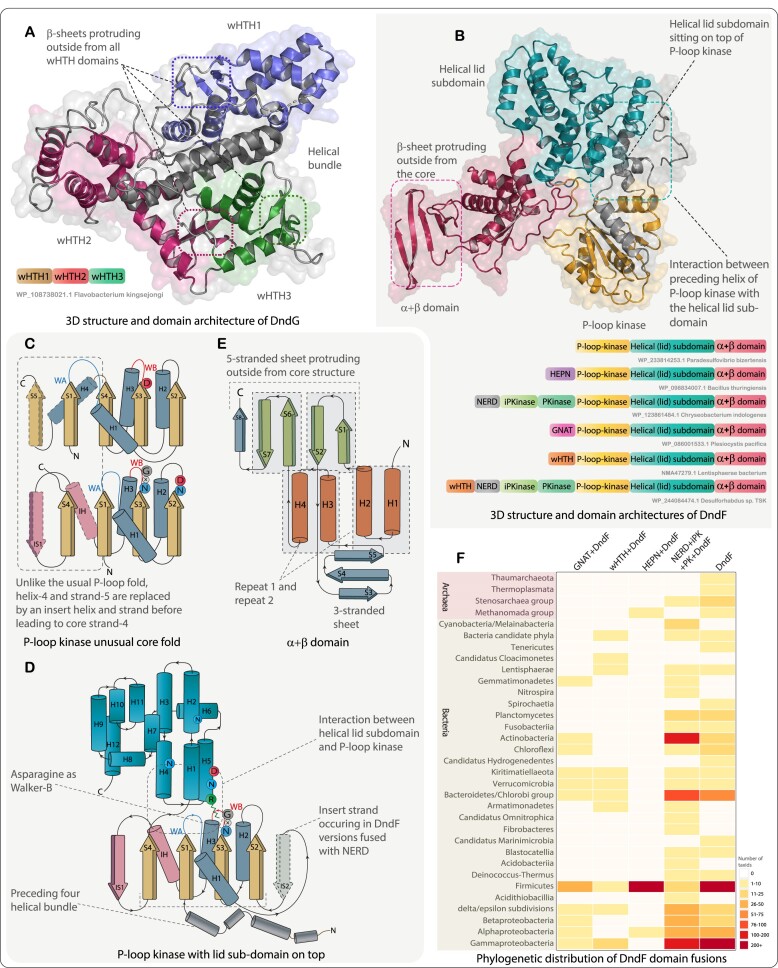
Structural features and domain architectures of DndG and DndF. (**A**, **B**) 3D structure and domain architectures of DndG and DndF, respectively. (**C–E**) structural topology diagrams showing key features of individual domains present in the DndF. (**F**) Phyletic distribution of multiple domain fusions occurring N-terminal to the P-loop kinase domain of DndF. As shown in Figure 1, the boxes are color-coded based on the number of unique NCBI tax IDs at the species level against each phylum or taxonomic group.

A recent structural study reported a C-terminal wHTH domain in DndG *Escherichia coli* B7A (residues 373–441) (16). However, systematic analysis of DndG from 1732 distinct bacterial taxa revealed that it is typically 450–500 residues long, leaving a substantial unaccounted N-terminal region. Through structure comparisons, profile-profile searches and structure prediction using AF2, we show that the C-terminal wHTH is preceded by two additional divergent N-terminal wHTH domains. Whereas the second and third wHTH domains feature a 3-stranded wing, the first domain shows a relatively rare 4-stranded wing. The second domain is also further decorated by a bihelical insert after the first core helix. Together, the wHTH domains of DndG fold into a compact 3-bladed structure (Figure 5A). Interestingly, outside of the DndFGH systems, a cognate triple wHTH module is also found in other restriction and DNA-modification systems. First, a version of this module is fused to the REase domain in the FokI restriction enzyme, where it functions as a DNA-binding domain (83,84). Second, it is also present in the SspB subunit of the variant PT restriction-modification system, sspABCDE (22,24). In that system, sspA, sspD, and sspE are equivalents of DndA, DndC, and DndB, respectively, whereas the Cdc6/Orc clade AAA+ ATPase, sspC, replaces the DndD/CXC ABC-ATPase of the Dnd system (22,24). Notably, this system does not have a DndE cognate, with its place evidently taken by sspB. Hence, as in the above two cases, DndG likely functions as a DNA-binding target-recognition and/or modification-discriminating subunit of the DndFGH system.

### DndF

We observed that DndF proteins contain a conserved two-domain core, which on occasion, in several different prokaryotes, might be extended via the fusion of biochemically diverse N-terminal domains (see below). The first of the two domains of the conserved core is a derived P-loop kinase that presents several unusual features: (i) it starts with a four-helical N-terminal extension with conserved charged residues. (ii) While the Walker A motif assumes a canonical form, the Walker B motif has an asparagine in lieu of the conserved aspartate. Interestingly, there is a conserved ND motif at the end of strand-2 of the core P-loop kinase. Such a configuration with an acidic residue at the end of strand-2 but not in the Walker B motif is seen in certain other P-loop kinases, such as the shikimate, gluconate, and dephospho-CoA kinases (85). (iii) The core strand-4 of the P-loop kinase is preceded by a helix-strand insert, with the strand replacing the ancestral strand 5 of the domain, albeit in an antiparallel configuration. (iv) The most remarkable feature of this P-loop kinase is a highly augmented lid subdomain (85) that is 200 residues in average length and comprised of 12–13 helices (Figure 5B, C and D). Some versions exhibit an additional Zn-chelating insert between the first and second helices of the lid (Supplementary Data S2-S3). Nevertheless, this subdomain retains the 3D configuration and a conserved arginine crucial for binding the base of the ATP typical of the P-loop kinases (85). This augmented lid further interacts with the above-mentioned N-terminal four-helical extension to form a large, exposed surface and is marked by multiple conserved residues. This suggests that it is favorably positioned to play an additional role in protein-protein interactions within the DndFGH complex.

The second core domain of DndF is an α+β domain that does not recover any related domains in structure similarity searches with DALI and Foldseek. However, an inspection of its structure revealed that it might have emerged via the duplication and augmentation of a relatively simple precursor: we found that the core of the domain contains two copies of a unit made up of a bihelical hairpin followed by a β-hairpin. The insertion of a 3-stranded β-meander between the two copies of the said unit expands this core, giving it a complex form with two distinct β-sheets. The two bihelical units and the β-hairpins protrude out from the P-loop kinase domain to present an extended exposed surface together with the large lid subdomain (Figure 5B, D and E). The α+β domain is fast-evolving and has no well-conserved residues indicative of a role in catalysis. However, its extensive exposed surface, positioned adjacent to part of the lid subdomain, suggests that it is favorably positioned for interactions with other similarly fast-evolving proteins, potentially from the invader. The variable N-terminal domains fused to the above core in several exemplars of DndF include: (i) the endoRNase HEPN; (ii) a REase domain of the NERD family; (iii) a S/T/Y kinase domain, typically occurring as tandem inactive and active copies; (iv) a GCN5-like acetyltransferase (GNAT) domain (annotated as DUF4338 in Pfam); (v) a wHTH; (vi) a Zn-ribbon. In some examples, multiple such domains, for instance, a wHTH, NERD, and inactive and active S/T/Y-kinase domains, are successively fused to the conserved core of DndF (Figure 5B and F, Supplementary Data S2-S3).

We interpret this architectural template of DndF in functional terms thus: while it is not certain whether the N-terminal P-loop kinase domain of DndF is catalytically active or inactive on account of the asparagine in the Walker B motif, its intact Walker A motif, together with the geometry of the Walker B, strand-2 polar residues and the lid arginine, indicate that it is definitely capable of binding a nucleotide. Several bacteriophages evade host restriction by incorporating modified nucleobases (86,87) derived from nucleotides modified by the action of phage enzymes, such as members of the thymidylate synthase superfamily, during DNA synthesis (88). Conversely, there are no endogenous signaling-nucleotide-generating enzymes encoded by any of the Dnd systems. Hence, we predict that it potentially serves as a sensor for phage-derived modified nucleotides. If it is active, it might further phosphorylate these nucleotides and prevent its use by the phage. Further, the fast-evolving α+β domain, together with the lid subdomain of the P-loop kinase, might directly interact with invader macromolecules. Therefore, we propose that a possible aspect of the activation of restriction by the DndFGH system is the sensing of invader-derived nucleotides by the DndF subunit. Under this scenario, the occasionally fused variable N-terminal domains are likely to function as co-effectors, as in other anti-invader conflict systems (81,89–91), which are brought into play if the main restriction activity were to be counteracted by inhibitory systems deployed by the selfish element. These could potentially evince their action by suicidally targeting self-molecules, including DNA (NERD), RNA (HEPN), or proteins (S/T/Y kinase or GNAT).

### DndH

**Figure 6.**
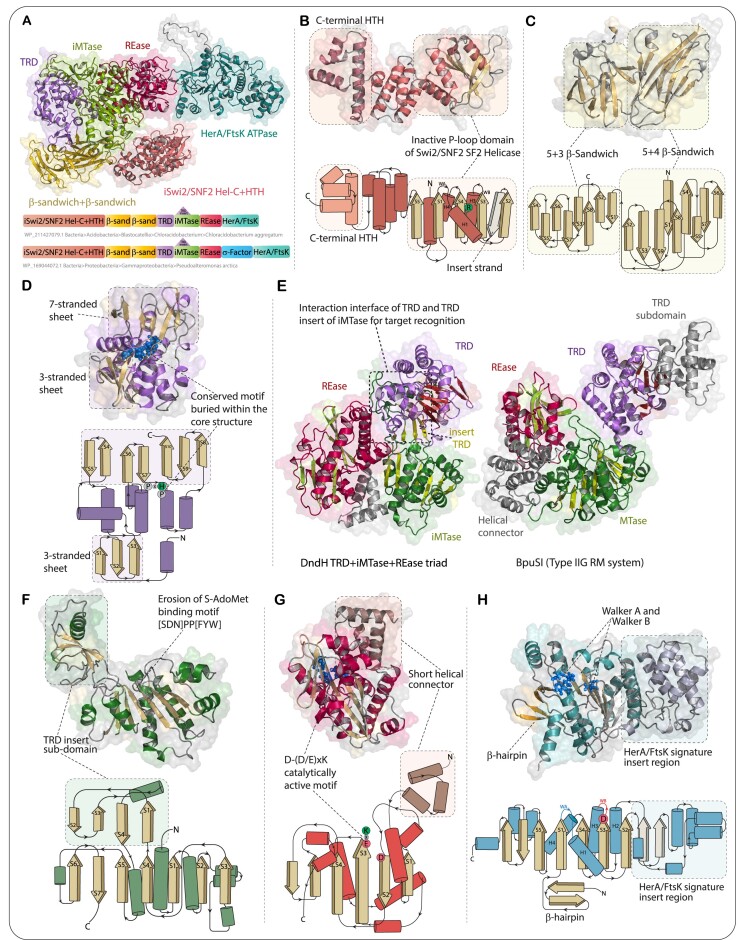
Structural features and domain architectures of DndH. (**A**) Overall 3D structure and representative domain architectures of DndH showing all individual domains. (B–D) 3D structure and structure topology diagrams showing key features of inactive C-terminal domain of SWI2/SNF2-type SF2 helicase module fused to an HTH domain (**B**), β-sandwich domains (**C**), and target recognition domain (**D**). (**E**) 3D structure of TRD + iMtase + REase triad and its comparison with equivalent domains from BpuSI (Type II-G). (F–H) 3D structure and structure topology diagrams showing key features of inactive Mtase (**F**), REase (**G**) and HerA/FtsK ATPase (**H**).

This subunit is again a large protein with a mean length of 1700 residues. Prior studies focusing on the DndFGH system had suggested that the C-terminal region of DndH contains a REase endoDNase followed by a RecA-like ATPase domain (16). However, our analysis indicated that the latter is not a member of the RecA clade; rather, it is unequivocally a HerA/FtsK-like ATPase (see below) with rather different functional implications. Moreover, the N-terminal part of 900 residues remains unaccounted for. Hence, we conducted a systematic analysis of the DndH proteins and established that they all possess a constant multidomain core of eight tandemly arranged globular domains that we outline below in order from the N- to the C-terminus (Figure 6A).

(i) Through DALI and profile-profile searches, we established that the N-terminal-most domain of DndH is a version of the second P-loop domain of the SWI2/SNF2-type SF2 helicase modules (Figure 6B). Both SF1 and SF2 helicases are comprised of an N-terminal canonical P-loop ATPase domain that binds and hydrolyzes ATP and a second C-terminal P-loop ATPase domain that lacks the Walker A and B motifs but contributes two arginine fingers that aid catalysis (92,93). Given that this domain in DndH corresponds to the second P-loop domain of the SWI2/SNF2-type SF2 modules, it lacks the characteristic ATP-binding signatures. Through sequence comparisons, we established that it also lacks the arginine fingers. However, we found that it contains a distinct, highly conserved arginine that lies precisely at the previously established nucleic-acid-binding interface of SF2 helicases. Hence, we posit that this inactive SWI2/SNF2-type P-loop domain binds DNA.

(ii) The above domain is immediately followed by a fast-evolving HTH domain augmented by an N-terminal helical bundle. This domain is predicted to extend the DNA-binding interface formed by the previous domain (Figure 6B).

(iii) These are followed by two copies of an all β-domain. DALI searches with these domains recovered carbohydrate-binding β-sandwich domains from glycosylhydrolases, e.g. the two β-sandwich domains of β-galactosidase (PDB: 1XC6). In light of their role in glycosylhydrolases, they could similarly bind the sugar in the backbone of DNA (94) or alternatively recognize proteins in the manner of β-sandwiches such as the immunoglobulin domain (95) (Figure 6C).

(iv) Beyond the above is a rapidly evolving target recognition domain (TRD) (Figure 6D) associated with the DNA adenine methyltransferase (DAM) of Type II RM systems (96,97). Consistent with this, we recovered equivalent domains from M.BpuSI (Type II-G) (98) and M.BspD6I (Type II-T) (99) as top hits (Figure 6E). In conventional R-M systems, this domain plays a key role in the recognition of their target DNA sequence motifs (3,4,70,71).

(v) The next module in the polypeptide is a DAM domain with a second TRD nested within it. A close examination of the DAM domain showed that while it retains the classic Rossmann fold morphology of a SAM-dependent methyltransferase, the characteristic sequence motif of the S-AdoMet-binding Rossmann loop between strand-1 and the following helix and the catalytic [SDN]PP[FYW] motif after strand-4 are completely eroded (10). Thus, it can be definitively seen as a catalytically inactive version. The nested TRD is inserted into the DAM domain after strand-4. A similar insert has been observed in the DAM domains of the so-called γ-group (96,100), suggesting that this inactive DAM domain of DndH has been derived from those DAMs. Additionally, structural modeling using AF2 showed that the N-terminal TRD and the inserted TRD are spatially close to each other, forming a cohesive interaction interface (Figure 6E, F).

(vi) The REase domain of DndH, which occurs after the DAM module, is similar to the eponymous domain from type-II restriction enzymes (e.g. BpuSI) and retains all key residues necessary for endoDNase activity (Figure 6E, G).

(vii) The C-terminal-most domain of DndH is a P-loop NTPase domain, which consistently recovered members of the HerA-FtsK clade of ATPase domains in sequence searches with PSI-BLAST as well as structure-similarity searches with DALI. Notably, it contains a Q as the sensor-1 residue at the end of the core strand-4 and an arginine finger just upstream of this strand. Contrary to previous claims, these observations irrefutably establish this domain as a member of the HerA/FtsK clade (101) rather than the RecA clade, which does not show any of these features (Figure 6H). Remarkably, we found that the HerA/FtsK ATPase domains at the C-termini of different DndH proteins come from several distinct clades of this class of domains, some of which are rather distant from each other (see below). The identification of this domain as HerA/FtsK suggests that it might function as a DNA translocase or pump, unlike a RecA-like ATPase, which is involved in recombination (101).

While the above multi-domain architecture of DndH is retained in most prokaryotes, we found a larger variant in certain gammaproteobacteria that features a σ-factor-related HTH domain inserted between the REase and the HerA-FtsK domains (Figure 6A).

### DndH is a member of a broader array of previously unrecognized ‘HerA-FtsK capture’ systems

**Figure 7.**
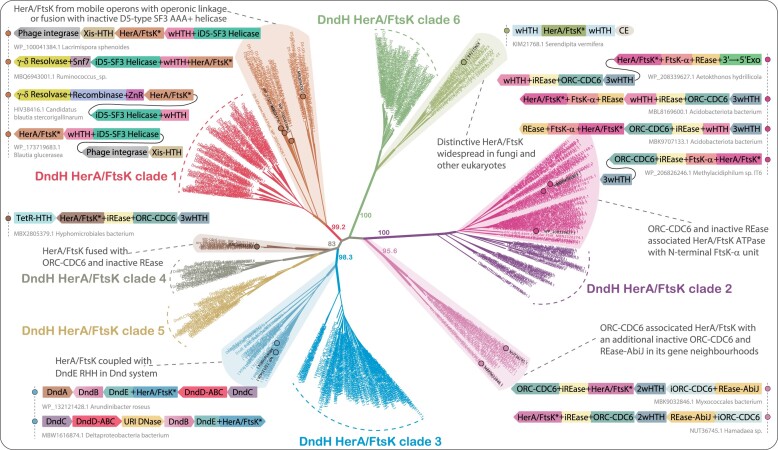
Diversity and functional radiation of HerA/FtsK capture systems. Maximum-likelihood tree topology anchored on HerA/FtsK domain of DndH informs six distinct and well-supported clades. HerA/FtsK Clades 1–6 are colored separately, and corresponding bootstrap values obtained from 1000 replicates are shown as percentages on major nodes. The ancestral counterparts of six DndH HerA/FtsK clades formed well-defined groups (highlighted as oval) and are clustered basal to the corresponding DndH HerA/FtsK clades with high confidence support. The contextual connections of the selected members of these ancestral counterparts are shown adjacent to each clade. The selected members are shown with filled circles with respective color codes of the cluster. As shown in Figure 2, gene neighborhoods are shown as box arrows, and the domain architectures of each gene product are shown within box arrows and are colored accordingly.

We next explored the implications of the striking finding that despite their conserved architecture, different DndH proteins display widely divergent HerA/FtsK domains belonging to multiple distinct clades. Phylogenetic analysis revealed that the HerA/FtsK domains from DndH proteins belong to six distinct clades, which also include homologs outside of DndFGH systems. Thus, the HerA/FtsK domains of DndH proteins are dispersed throughout a radiation of these domains rather than forming a single clade. We observe the following relationships for the HerA/FtsK domains found in different DndH proteins (Figure 7, Supplementary Dataset S1): clade-1 (red) additionally includes certain HerA-FtsK-like proteins encoded by a mobile operon, likely originating from a transposon, where they are linked or fused to a D5-type SF3 AAA+ helicase (see below); clade-2 (purple) also contains the highly conserved cellular FtsK involved in chromosome segregation (101); clade-3 (blue) further includes a group of HerA/FtsK domains that are found in a subset of Dnd PT-modification systems (24). Here, they occur adjacent to genes for a DndD-related ABC ATPase and are fused to DndE in the same polypeptide; clade-4 (grey) and clade-5 (yellow) additionally feature a class of HerA/FtsK domains coupled with an Orc/CDC6 family AAA+ ATPase (102,103) (see below); clade-6 (green) also contains a distinctive class of HerA-like ATPases found in certain eukaryotic elements (see below). This indicates that the DndH proteins have evolved through the repeated ‘capture’ of different versions of the HerA/FtsK domain from a diversity of sources ranging from the cellular DNA segregation system to disparate mobile gene neighborhoods (Figure 7). The HerA/FtsK domains from clades 1, 5, and 6 led us to a mélange of interesting mobile gene neighborhoods that display features indicative of both mobile elements and anti-selfish element defense systems.

Outside of DndH, the clade-1 HerA/FtsKs are predominantly found in mobile operons from firmicutes. We observed that the totality of HerA/FtsK domains found in these mobile neighborhoods themselves belong to two distinct branches, with all DndH-associated versions being nested within just one of the two branches. This suggests that the Clade-1 HerA/FtsKs in DndH were captured from a specific branch of these mobile operons. These operons additionally code for a D5-type SF3 helicase, a Xis-type HTH, a tyrosine (Y)-recombinase, and sometimes a γ/δ-type resolvase (Figure 7). The D5-type SF3 helicase is often fused to a C-terminal TcpK-like wHTH domain (PDB: 5VFY-B) that has been shown to bind DNA during conjugation in other systems (104). Similarly, the Xis-type HTH recognizes recombination sites in other transposons (103). Outside of these mobile operons, a closely related D5-like helicase is also found fused to Toprim primases, Primpols, phage P4-type RepBprime primases, and family A DNA polymerase with the proofreading 3′→5′ exoDNase domain (105,106). Further, the D5 SF3 helicase may also be coupled in an operon with a class II GATase domain that might function as a cell-wall peptidase during DNA transfer (107). These observations indicate that the D5-type SF3 helicase is a hallmark of multiple conjugative mobile elements. Interestingly, whereas the D5-helicase linked to the primases and polymerases is invariably active, the versions fused to the HerA/FtsK domains tend to be inactive. This suggests that while the former are functional mobile elements, the latter are potentially transposons domesticated for a role in counter-invader conflict (Supplementary Data S1).

Similarly, certain versions of clades 2 and 4 and clade-5 HerA/FtsK domains led us to a distinct class of mobile gene neighborhoods where they are coupled to a specific version of the Orc/CDC6 family of AAA+ ATPases (Figure 7). These neighborhoods usually encode the following core domains: (i) an Orc/CDC6 ATPase; (iii) an inactive REase (iREase); (ii) an active REase belonging to either of two distinct families, one of which is also found in the AbiJ antiphage systems (72,77); (iv) a HerA/FtsK; (v) a wHTH. While these domains are combined in various arrangements in single or multiple polypeptides, the iREase domain is invariably inserted between the core AAA+ ATPase and the C-terminal wHTH characteristic of the Orc/CDC6 family (103). A subset of these operons also displays a further gene for a rapidly evolving protein with 2–4 wHTH domains related to DndG/DptG (16), sspB (22) and the FokI DNA-binding domain (83,84) (see above), while others may feature a 3′→5′ exonuclease domain fused to the HerA/FtsK component (Figure 7). From the perspective of these gene neighborhoods, one observes a similar phenomenon of the ‘capture’ of HerA/FtsK domains from different sources. For instance, one set of HerA/FtsK domains in these systems are fused to a N-terminal FtsKα domain that is otherwise only found in certain DndH proteins (clade 2, above) and the classical cellular FtsK and not in other HerA/FtsKs (101). Therefore, this version was captured from the cellular versions involved in chromosome segregation. Conversely, a subset of these neighborhoods encodes another version of the HerA/FtsK domain that lacks a fusion to the FtsKα domain but has a 4-helical bundle inserted into it, evidently indicating its acquisition from a distinct source (Figure 7). Independently of the above HerA/FtsK association, related Orc/Cdc6 genes are also found in further mobile gene neighborhoods where they are coupled in operons with either genes coding for (i) a REase domain fused to the modified DNA-binding ASCH domain and a DndG-like triple wHTH protein or (ii) a second Orc/CDC6 protein with an intact Walker A but a disrupted Walker B and a KAP P-loop NTPase previously implicated in anti-phage immunity (108). The active Orc/CDC6 copy from these neighborhoods are also often fused to a STAND NTPase (e.g. NACHT) or pentapeptide repeats or a TIR domain that possesses NADase activity (109). This web of associations to domains that have been implicated previously in anti-selfish element immunity strongly supports a similar role for these systems (Supplementary Data S1).

Lastly, clade-6 also contains HerA/FtsK domains found in a group of novel genomic elements that are widespread in fungi (Ascomycota, Basidiomycota, Chytridiomycota) and other eukaryotes (*Carpediemonas*, *Kipferlia*, some plants, stramenopiles, haptophytes, *Capsaspora*) (Figure 7). These are characterized by a 4-helical insert in the HerA/FtsK P-loop NTPase domain and a peculiar C-terminal extension with a conserved motif (hxhxxRxRhsxDsGx[ST] signature), which is retained even in the cognate versions captured by DndH. AF2 modeling suggests that this extension projects out from the core and is evidently available for interaction with some other protein. Most eukaryotic members of this clade are characterized by an N-terminal extension that appears to be derived from a wHTH and 1–2 further C-terminal wHTH domains. Given that these versions show no fusions/genomic linkages to nuclease domains, they are likely to mediate their function (such as mobility) by interacting with some conserved cellular component via the above-mentioned C-terminal extension (Supplementary Data S1).

### Functional and evolutionary implications

While previous studies have postulated that DndABCDE and DndFGH together constitute a unique PT modification and restriction system, a systematic survey of those results together with our current analysis raise serious questions about their functional coupling. Instead, our results favor a scenario wherein the two, form distinct and self-contained anti-invader conflict systems that might cooperate on occasion. Indeed, multiple studies from the growing diversity of such conflict systems, including R-M, CRISPR/Cas, SMODS and the NAD^+^-targeting apparatus, point to a degree of cooperation between them, wherein the failure of one system might be backed up by another system that is otherwise capable of independent action (5,9,90,110).

#### A revised model for the action of DndABCDE and associated nucleases

**Figure 8.**
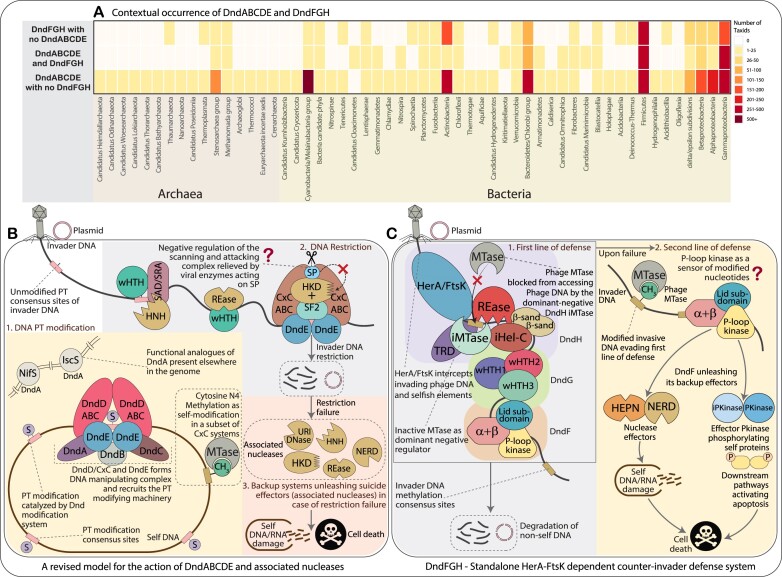
Phyletic decoupling, new functional models, and the overall summary of DndABCDE with associated nucleases and DndFGH systems. (**A**) Phyletic distribution showing different contextual occurrences and decoupling of the Dnd systems. As shown in Figure 1, the boxes are color-coded based on the number of unique NCBI tax IDs at the species level against each phylum or taxonomic group. (**B**) Schematic diagram showing the functional mechanisms of the newly proposed and hypothesized model of DndABCDE and associated nucleases incorporating both the modification and restriction components. (**C**) Schematic diagram showing the hypothesized functional mechanisms of DndFGH operating as a standalone counter-invader defense system.

While our comprehensive survey indicated a high degree of decoupling between the DndABCDE and DndFGH modules (Figure 8A, Supplementary Data S1), it recovered a strong gene-neighborhood association between the Dnd modification modules and several other endoDNases. One of these is the wHTH + SAD + HNH protein, wherein the wHTH domain corresponds to the domain previously shown to bind PT in DNA (80). Similarly, we found other endoDNases with potential modified DNA discriminant modules that could serve as restriction components that go with the modification module. Hence, we reconstruct a scenario wherein: (i) DndD/CxC ABC ATPase and DndE form a complex akin to the well-studied ABC ATPase DNA manipulation complexes seen in chromosome dynamics and DNA repair/recombination (24). The different versions of DndE co-evolving with the cognate DndD/CxC ABC ATPase could account for diversity in the modification target motifs. DndE likely scans the self-genome in conjunction with ABC ATPase-catalyzed DNA-looping to recognize such sites. In the sspABCDE systems, where the ABC ATPase is replaced by an ORC/CDC6 clade AAA+ ATPase, the scanning likely occurs through an alternative mechanism. Akin to other ORC/CDC6-type ATPases, these are predicted to form toroidal rings around DNA duplexes and translocate along them together with the triple-wHTH SspB recognition component. (ii) Once DndE and DndD/CxC ABC complex bind to a target site, it either triggers the associated PT-modification apparatus (DndA and DndC) to incorporate sulfur into the DNA backbone or potentially signals its modification status. (iii) In the restriction step, the reader modules fused to the endoDNases, such as the PT-DNA recognizing wHTH domain, discriminate self from non-self DNA and trigger invader restriction through DNA cleavage (Figure 8B).

Overlayed on this core process are several distinct regulatory and/or backup systems. First, 52% of all taxa with the Dnd modification system also code for the ParB superfamily protein DndB. While it has been proposed to be an ATP-binding transcription factor, other studies on ParB proteins in conflict suggest that they might act as antiviral effectors that degrade nucleotides or as nucleases (19,111,112). Indeed, DndB could also act similarly by guarding against failure either by targeting phage-generated modified nucleotides or suicidally by limiting nucleotides like ATP in the general pool. Further, it could also serve as a negative regulator of the modification system by limiting ATP needed for PT incorporation. Second, as we noted above, several of these systems also code for standalone RNases and DNases that could act as suicide effectors in the event of failure of the main restriction system (Figures 4A, C, 8B). Our investigations also raise a further question – whether the DndD and CXC-ABC-ATPase-centered systems exclusively facilitate PT modification. We found that PT modification genes are not found in association with the DndD-DndE core in numerous taxa. While it has been proposed that housekeeping homologs of the cysteine desulfurase (IscS, NifS, SufS) could substitute for DndA (15), there are no functionally equivalent, close PP-loop ATPases to take the place of DndC (18,51–54). Hence, it is possible that some of these systems mediate entirely different modifications. At least in the case of the CXC ABC-ATPase-centered systems, we detected a frequent coupling with cytosine methylases (24) suggesting that modified cytosines could substitute for PT as in conventional R-M systems (Figures 4A: iv, 8B).

#### DndFGH constitutes a standalone counter-invader defense system

Our comprehensive dissection of the domain architectures of the three universal components of this system allows for a revision of the possible model of its action (Figure 8C). This analysis revealed several unprecedented features of this system: (i) Taken together, the three subunits field a large number of distinct DNA binding domains, viz., a triad of wHTH domains (DndG), a C-terminal domain of the SWI2/SNF2 SF2 helicase module, a further HTH and two TRD domains (DndH). This unusual feature indicates that the system forms several distinct concomitant or successive contacts with DNA, some of which might be sequence-specific. (ii) Together, the system displays 3 enzymatic domains, namely a P-loop kinase (DndF), a REase and a HerA/FtsK class P-loop ATPase (DndH). While the P-loop kinase domain is rather derived, it possesses an intact NTP-binding site suggesting that, in the least, it can act as a sensor of nucleotides. The recognition that the HerA/FtsK ATPase domain of DndH is captured from various distinct systems featuring such ATPases is one of the most striking findings of this study. Such ATPases play a role not only in the segregation of the cellular chromosome and plasmids but also in pumping the DNA of various conjugative selfish elements and phages with internal membranes (101). Hence, given that they have been repeatedly acquired from various sources in different DndH proteins, distinct versions of this system might be able to target different plasmids and other selfish elements depending on the type of HerA/FtsK domain it carries. (iii) Despite having an active REase domain, it is notable that the DAM domain in this system is catalytically inactive. This suggests that rather than modifying the target sites, it could prevent modifications of such sites by methylases encoded in selfish elements by binding as a dominant negative. Finally, as with several other conflict systems, versions of this system display a previously unnoticed repertoire of variable backup effectors that might be activated in the event of system failure (Figure 8C).

Based on the above, we propose that the DndFGH system adopts a multi-pronged sensory approach that allows it to deploy restriction activity of the REase domain despite lacking an inbuilt modification module. Key to this are the multiple DNA-binding domains that evidently mediate specific target recognition. Notably, the triple-wHTH DndG, which is related to SspB from the sspABCDE DNA-modification systems, might explain how a subset of DndFGH systems can use PT-modified DNA as a discriminant. In addition to DNA recognition, the P-loop kinase domain is predicted to enable the sensing of modified nucleotides. Further, the specific recognition/localization to invader molecules could occur via the α+β domain in DndF and the two β-sandwich domains of DndH. Thus, we suggest that this system precisely homes in on invader DNA through a combination of macromolecular determinants and soluble signals of infection. Further specificity in terms of the location of action might be provided by the HerA/FtsK domain – given the roles of the homologous versions captured by these systems, it likely intercepts invader DNA that is segregating during cell division (plasmids) or being pumped into recipient cells (conjugative elements) or packaged into capsids (phages). Finally, earlier studies have indicated that several phages and conjugative elements encode their own DAM methylases that are delivered alongside the invading DNA and modify it to evade host restriction (113–115). The inactive DAM domain from DndH could interfere with such an evasion mechanism by acting as a dominant negative that prevents the action of the invader methylases by rendering its target sites inaccessible (Figure 8C).

#### ‘HerA/FtsK capture systems’ are a more general theme in anti-invader conflict systems

A striking result from this study is that DndH is one of several distinct systems that exhibit a similar phenomenon of incorporating HerA/FtsK domains from evolutionarily distinct sources (Figure 7). As in DndFGH systems, we propose that different versions of the HerA/FtsK domain from these systems, along with frequently associated HTH domains, allow the localization of the defense apparatus to specific invader elements even as they are engaged in the process of genome segregation, transfer, or packaging. Further, the HerA/FtsK domain might help translocate invader DNA through pumping action to bring suitable sites on the targeted genome to the restriction apparatus. Like DndH, these new systems feature active endoDNase domains that could act as their restriction component. Notably, the first of these newly recovered systems contains an inactive D5-type SF3 helicase domain that is reminiscent of the inactive SWI2/SNF2 C-terminal P-loop domain in DndH. In both these cases, these inactive derivatives of helicases could help bind replication intermediates of the invasive elements that frequently form during transfer-coupled replication. In the second of these systems, the HerA/FtsK domain is coupled to AAA+ ATPases of the Orc/CDC6 family. Several studies in the past few years have revealed that beyond their role in the replication of cellular genomes, members of the Orc/CDC6 family are the mainstay of various biological conflict systems (103). On one hand, these include ATPase domains/subunits of transposases (e.g. TN7 and Mu) and a widespread class of enzymatic effectors (the eukaryotic CR-effectors and their prokaryotic relatives) derived from them that tend to combine the CDC6/ORC1 clade NTPase domain with a C-terminal REase domain. On the other hand, this family also includes the STAND NTPases that are major players in immunity across the Tree of Life (102). We suggest that in the systems combining the ORC/Cdc6 and HerA/FtsK ATPase domains, the former might help the restriction apparatus localize to specific sites on the DNA, even as the latter intercepts the selfish element during segregation or transfer.

## Conclusions

While the Dnd systems have been the subject of intense scrutiny over the past several years, there remain several open questions regarding their mode of action and role in defense against selfish elements. Furthermore, inaccurate claims have been made regarding the constituent protein domains of components of these systems. We addressed these issues, thereby providing an improved understanding of them. Our work argues for a high degree of functional decoupling between the DndABCDE modification module and the previously implicated DndFGH systems. Instead, we present evidence that the modification module more frequently associates in the same gene neighborhood with other endoDNase domains fused to DNA-binding domains, which might serve as the actual restriction components. We also present evidence for the frequent coupling of multiple copies of Dnd systems centered on the ABC ATPase and DndE-like dyad in the same genome. Several of these systems, especially those centered on the CXC ABC ATPase, lack neighboring genes for the usual PT modification enzymes. Instead, they display one or more cytosine methylases, suggesting that a subset of these systems might facilitate modifications other than PT. Furthermore, we definitively establish that all DndEs are composed of two RHH domains, dispelling earlier structural and functional misconceptions regarding this protein. We also present evidence for the diversification of DndE and its coevolution with its DndD/CxC ABC ATPase partner. This allowed us to propose a model where it acts analogously to the Kleisin or ScpB DNA-binding component observed in ABC ATPases mediating chromosome dynamics, enabling different Dnd systems to recognize and/or modify highly divergent sites on the self-DNA.

We demonstrate that DndFGH constitutes a separate counter-invader system and identify several previously undetected domains in its subunits. This leads us to postulate that it receives multiple sensory inputs, both in the form of soluble nucleotides and invader macromolecules, to specifically deploy its REase domain despite lacking an inherent modification component. Moreover, we show for the first time that DndH contains a HerA/FtsK ATPase domain, which, in different versions of this protein, is acquired from diverse sources featuring the aforementioned domain. We term this phenomenon ‘HerA/FtsK capture’ and demonstrate that DndH is just one of several potential anti-invader systems incorporating HerA/FtsK ATPase domains from different sources.

Among the key testable hypothesis with potential for promising results is an investigation of the proposed ‘clade-specific’ DNA target sites for DndD/CxC-DndE pairs using assays to query DNA-binding specificity. Similar tests for DNA binding activity based on the domains identified here could also help test the recognition and discrimination of unmodified versus modified DNA, as well has the specificity of the domains for different modifications such a PT, 5mC or 5hmC. Regarding the DndFGH system, our results would help guide experiments to establish the specificity of the multiple DNA-binding domains in the overall complex and also test the hypotheses regarding the role of the nucleotide kinase domain in DndF and of inactive DAMs as dominant negative regulators interfering with invader methylases. The identification of the capture of HerA/FtsK domains in DndH and other systems not only opens new vistas to explore the activity of these ATPases but also suggests the use of subcellular localization studies to explore the role of invader-genome-interception during chromosome segregation, transfer or packaging. Hence, we hope that this comprehensive analysis will facilitate future wet-lab investigations not only of Dnd but also the broader spectrum of anti-invader systems discussed here.

## Supplementary Material

gkad1213_Supplemental_Files

## Data Availability

The data underlying this article are available in the article and in its online supplementary material. This data is also available in various computer-readable formats at https://zenodo.org/records/10057384.

## References

[1] Koonin E.V. , Makarova K.S., Wolf Y.I. Evolutionary genomics of defense systems in archaea and bacteria. Annu. Rev. Microbiol. 2017; 71:233–261. 28657885 10.1146/annurev-micro-090816-093830 PMC5898197

[2] Hampton H.G. , Watson B.N.J., Fineran P.C. The arms race between bacteria and their phage foes. Nature. 2020; 577:327–336. 31942051 10.1038/s41586-019-1894-8

[3] Vasu K. , Nagaraja V. Diverse functions of restriction-modification systems in addition to cellular defense. Microbiol. Mol. Biol. Rev. 2013; 77:53–72. 23471617 10.1128/MMBR.00044-12 PMC3591985

[4] Dimitriu T. , Szczelkun M.D., Westra E.R. Evolutionary ecology and interplay of prokaryotic innate and adaptive immune systems. Curr. Biol. 2020; 30:R1189–R1202. 33022264 10.1016/j.cub.2020.08.028 PMC7116224

[5] Bernheim A. , Sorek R. The pan-immune system of bacteria: antiviral defence as a community resource. Nat. Rev. Microbiol. 2020; 18:113–119. 31695182 10.1038/s41579-019-0278-2

[6] Stern A. , Sorek R. The phage-host arms race: shaping the evolution of microbes. Bioessays. 2011; 33:43–51. 20979102 10.1002/bies.201000071 PMC3274958

[7] Georjon H. , Bernheim A. The highly diverse antiphage defence systems of bacteria. Nat. Rev. Microbiol. 2023; 21:686–700. 37460672 10.1038/s41579-023-00934-x

[8] Ershova A.S. , Rusinov I.S., Spirin S.A., Karyagina A.S., Alexeevski A.V. Role of restriction-modification systems in prokaryotic evolution and ecology. Biochemistry (Mosc). 2015; 80:1373–1386. 26567582 10.1134/S0006297915100193

[9] Aravind L. , Iyer L.M., Burroughs A.M. Discovering biological conflict systems through genome analysis: evolutionary principles and biochemical novelty. Annu. Rev. Biomed Data Sci. 2022; 5:367–391. 35609893 10.1146/annurev-biodatasci-122220-101119

[10] Iyer L.M. , Zhang D., Aravind L. Adenine methylation in eukaryotes: apprehending the complex evolutionary history and functional potential of an epigenetic modification. Bioessays. 2016; 38:27–40. 26660621 10.1002/bies.201500104 PMC4738411

[11] Weigele P. , Raleigh E.A. Biosynthesis and function of modified bases in bacteria and their viruses. Chem. Rev. 2016; 116:12655–12687. 27319741 10.1021/acs.chemrev.6b00114

[12] Wang L. , Chen S., Xu T., Taghizadeh K., Wishnok J.S., Zhou X., You D., Deng Z., Dedon P.C. Phosphorothioation of DNA in bacteria by dnd genes. Nat. Chem. Biol. 2007; 3:709–710. 17934475 10.1038/nchembio.2007.39

[13] Xu T. , Liang J., Chen S., Wang L., He X., You D., Wang Z., Li A., Xu Z., Zhou X. et al. DNA phosphorothioation in Streptomyces lividans: mutational analysis of the dnd locus. BMC Microbiol. 2009; 9:41. 19232083 10.1186/1471-2180-9-41 PMC2653506

[14] Wang L. , Jiang S., Deng Z., Dedon P.C., Chen S. DNA phosphorothioate modification-a new multi-functional epigenetic system in bacteria. FEMS Microbiol. Rev. 2019; 43:109–122. 30289455 10.1093/femsre/fuy036 PMC6435447

[15] You D. , Wang L., Yao F., Zhou X., Deng Z. A novel DNA modification by sulfur: DndA is a NifS-like cysteine desulfurase capable of assembling DndC as an iron−sulfur cluster protein in streptomyces lividans. Biochemistry. 2007; 46:6126–6133. 17469805 10.1021/bi602615k

[16] Wu D. , Tang Y., Chen S., He Y., Chang X., Zheng W., Deng Z., Li Z., Wang L., Wu G. et al. The functional coupling between restriction and DNA phosphorothioate modification systems underlying the DndFGH restriction complex. Nature Catalysis. 2022; 5:1131–1144.

[17] Chen F. , Zhang Z., Lin K., Qian T., Zhang Y., You D., He X., Wang Z., Liang J., Deng Z. et al. Crystal structure of the cysteine desulfurase DndA from streptomyces lividans which is involved in DNA phosphorothioation. PLoS One. 2012; 7:e36635. 22570733 10.1371/journal.pone.0036635 PMC3343029

[18] Pu T. , Mei Z., Zhang W., Liang W.J., Zhou X., Liang J., Deng Z., Wang Z. An in vitro DNA phosphorothioate modification reaction. Mol. Microbiol. 2020; 113:452–463. 31749226 10.1111/mmi.14430

[19] Maindola P. , Raina R., Goyal P., Atmakuri K., Ojha A., Gupta S., Christie P.J., Iyer L.M., Aravind L., Arockiasamy A. Multiple enzymatic activities of ParB/Srx superfamily mediate sexual conflict among conjugative plasmids. Nat. Commun. 2014; 5:5322. 25358815 10.1038/ncomms6322 PMC4241021

[20] He W. , Huang T., Tang Y., Liu Y., Wu X., Chen S., Chan W., Wang Y., Liu X., Chen S. et al. Regulation of DNA phosphorothioate modification in Salmonella enterica by DndB. Sci. Rep. 2015; 5:12368. 26190504 10.1038/srep12368 PMC4507180

[21] Xia S. , Chen J., Liu L., Wei Y., Deng Z., Wang L., Chen S. Tight control of genomic phosphorothioate modification by the ATP-modulated autoregulation and reusability of DndB. Mol. Microbiol. 2019; 111:938–950. 30552823 10.1111/mmi.14186

[22] Xiong X. , Wu G., Wei Y., Liu L., Zhang Y., Su R., Jiang X., Li M., Gao H., Tian X. et al. SspABCD–SspE is a phosphorothioation-sensing bacterial defence system with broad anti-phage activities. Nat. Microbiol. 2020; 5:917–928. 32251370 10.1038/s41564-020-0700-6

[23] He W. , Gao H., Wu D., Jiang S., Huang W., Chen C., Deng Z., Xiong L., Wu G., Wang L. Structural and functional analysis of DndE involved in DNA phosphorothioation in the haloalkaliphilic Archaea Natronorubrum bangense JCM10635. Mbio. 2022; 13:e00716-22. 35420474 10.1128/mbio.00716-22 PMC9239217

[24] Krishnan A. , Burroughs A.M., Iyer L.M., Aravind L. Comprehensive classification of ABC ATPases and their functional radiation in nucleoprotein dynamics and biological conflict systems. Nucleic Acids Res. 2020; 48:10045–10075. 32894288 10.1093/nar/gkaa726 PMC7544218

[25] Xiong L. , Liu S., Chen S., Xiao Y., Zhu B., Gao Y., Zhang Y., Chen B., Luo J., Deng Z. et al. A new type of DNA phosphorothioation-based antiviral system in archaea. Nat. Commun. 2019; 10:1688. 30975999 10.1038/s41467-019-09390-9 PMC6459918

[26] Cao B. , Chen C., DeMott M.S., Cheng Q., Clark T.A., Xiong X., Zheng X., Butty V., Levine S.S., Yuan G. et al. Genomic mapping of phosphorothioates reveals partial modification of short consensus sequences. Nat. Commun. 2014; 5:3951. 24899568 10.1038/ncomms4951 PMC4322921

[27] Cao B. , Cheng Q., Gu C., Yao F., DeMott M.S., Zheng X., Deng Z., Dedon P.C., You D Pathological phenotypes and in vivo DNA cleavage by unrestrained activity of a phosphorothioate-based restriction system in Salmonella. Mol. Microbiol. 2014; 93:776–785. 25040300 10.1111/mmi.12692 PMC4414249

[28] Altschul S.F. , Madden T.L., Schäffer A.A., Zhang J., Zhang Z., Miller W., Lipman D.J. Gapped BLAST and PSI-BLAST: a new generation of protein database search programs. Nucleic Acids Res. 1997; 25:3389–3402. 9254694 10.1093/nar/25.17.3389 PMC146917

[29] Johnson L.S. , Eddy S.R., Portugaly E. Hidden Markov model speed heuristic and iterative HMM search procedure. BMC Bioinf. 2010; 11:431. 10.1186/1471-2105-11-431 PMC2931519 20718988

[30] Altschul S.F. , Gish W., Miller W., Myers E.W., Lipman D.J. Basic local alignment search tool. J. Mol. Biol. 1990; 215:403–410. 2231712 10.1016/S0022-2836(05)80360-2

[31] Zimmermann L. , Stephens A., Nam S.Z., Rau D., Kübler J., Lozajic M., Gabler F., Söding J., Lupas A.N., Alva V. A completely reimplemented MPI bioinformatics toolkit with a new HHpred server at its core. J. Mol. Biol. 2018; 430:2237–2243. 29258817 10.1016/j.jmb.2017.12.007

[32] Hildebrand A. , Remmert M., Biegert A., Söding J. Fast and accurate automatic structure prediction with HHpred. Proteins. 2009; 77:128–132. 19626712 10.1002/prot.22499

[33] Berman H.M. , Westbrook J., Feng Z., Gilliland G., Bhat T.N., Weissig H., Shindyalov I.N., Bourne P.E. The Protein Data Bank. Nucleic Acids Res. 2000; 28:235–242. 10592235 10.1093/nar/28.1.235 PMC102472

[34] Mistry J. , Chuguransky S., Williams L., Qureshi M., Salazar G.A., Sonnhammer E.L.L., Tosatto S.C.E., Paladin L., Raj S., Richardson L.J. et al. Pfam: the protein families database in 2021. Nucleic Acids Res. 2021; 49:D412–D419. 33125078 10.1093/nar/gkaa913 PMC7779014

[35] Remmert M. , Biegert A., Hauser A., Söding J. HHblits: lightning-fast iterative protein sequence searching by HMM-HMM alignment. Nat. Methods. 2012; 9:173–175. 10.1038/nmeth.1818 22198341

[36] Lassmann T. , Frings O., Sonnhammer E.L. Kalign2: high-performance multiple alignment of protein and nucleotide sequences allowing external features. Nucleic Acids Res. 2009; 37:858–865. 19103665 10.1093/nar/gkn1006 PMC2647288

[37] Katoh K. , Standley D.M. MAFFT multiple sequence alignment software version 7: improvements in performance and usability. Mol. Biol. Evol. 2013; 30:772–780. 23329690 10.1093/molbev/mst010 PMC3603318

[38] Holm L. , Laiho A., Törönen P., Salgado M. DALI shines a light on remote homologs: one hundred discoveries. Protein Sci. 2023; 32:e4519. 36419248 10.1002/pro.4519 PMC9793968

[39] Drozdetskiy A. , Cole C., Procter J., Barton G.J. JPred4: a protein secondary structure prediction server. Nucleic Acids Res. 2015; 43:W389–W394. 25883141 10.1093/nar/gkv332 PMC4489285

[40] Varadi M. , Anyango S., Deshpande M., Nair S., Natassia C., Yordanova G., Yuan D., Stroe O., Wood G., Laydon A. et al. AlphaFold Protein Structure Database: massively expanding the structural coverage of protein-sequence space with high-accuracy models. Nucleic Acids Res. 2022; 50:D439–D444. 34791371 10.1093/nar/gkab1061 PMC8728224

[41] Jumper J. , Evans R., Pritzel A., Green T., Figurnov M., Ronneberger O., Tunyasuvunakool K., Bates R., Žídek A., Potapenko A. et al. Highly accurate protein structure prediction with AlphaFold. Nature. 2021; 596:583–589. 34265844 10.1038/s41586-021-03819-2 PMC8371605

[42] Price M.N. , Dehal P.S., Arkin A.P. FastTree 2 – Approximately maximum-likelihood trees for large alignments. PLoS One. 2010; 5:e9490. 20224823 10.1371/journal.pone.0009490 PMC2835736

[43] Chernomor O. , von Haeseler A., Minh B.Q. Terrace aware data structure for phylogenomic inference from supermatrices. Syst. Biol. 2016; 65:997–1008. 27121966 10.1093/sysbio/syw037 PMC5066062

[44] Minh B.Q. , Schmidt H.A., Chernomor O., Schrempf D., Woodhams M.D., Haeseler A., Lanfear R. IQ-TREE 2: new models and efficient methods for phylogenetic inference in the genomic era. Mol. Biol. Evol. 2020; 37:1530–1534. 32011700 10.1093/molbev/msaa015 PMC7182206

[45] Hoang D.T. , Chernomor O., von Haeseler A., Minh B.Q., Vinh L.S. UFBoot2: improving the Ultrafast bootstrap approximation. Mol. Biol. Evol. 2018; 35:518–522. 29077904 10.1093/molbev/msx281 PMC5850222

[46] Frickey T. , Lupas A. CLANS: a Java application for visualizing protein families based on pairwise similarity. Bioinformatics. 2004; 20:3702–3704. 15284097 10.1093/bioinformatics/bth444

[47] Shannon C.E. A mathematical theory of communication. Bell Syst. Tech. J. 1948; 27:379–423.

[48] Capra J.A. , Singh M. Predicting functionally important residues from sequence conservation. Bioinformatics. 2007; 23:1875–1882. 17519246 10.1093/bioinformatics/btm270

[49] Azad R.K. , Lawrence J.G. Detecting laterally transferred genes: use of entropic clustering methods and genome position. Nucleic Acids Res. 2007; 35:4629–4639. 17591616 10.1093/nar/gkm204 PMC1950545

[50] Jian H. , Xu G., Yi Y., Hao Y., Wang Y., Xiong L., Wang S., Liu S., Meng C., Wang J. et al. The origin and impeded dissemination of the DNA phosphorothioation system in prokaryotes. Nat. Commun. 2021; 12:6382. 34737280 10.1038/s41467-021-26636-7 PMC8569181

[51] Zhou X. , He X., Liang J., Li A., Xu T., Kieser T., Helmann J.D., Deng Z. A novel DNA modification by sulphur. Mol. Microbiol. 2005; 57:1428–1438. 16102010 10.1111/j.1365-2958.2005.04764.x

[52] Pinto R. , Tang Q.X., Britton W.J., Leyh T.S., Triccas J.A. The mycobacterium tuberculosis cysD and cysNC genes form a stress-induced operon that encodes a tri-functional sulfate-activating complex. Microbiology (Reading). 2004; 150:1681–1686. 15184554 10.1099/mic.0.26894-0

[53] Mueller E.G. , Palenchar P.M., Buck C.J. The role of the cysteine residues of ThiI in the generation of 4-thiouridine in tRNA*. J. Biol. Chem. 2001; 276:33588–33595. 11443125 10.1074/jbc.M104067200

[54] Suzuki T. , Miyauchi K. Discovery and characterization of tRNAIle lysidine synthetase (TilS). FEBS Lett. 2010; 584:272–277. 19944692 10.1016/j.febslet.2009.11.085

[55] Lai C. , Wu X., Chen C., Huang T., Xiong X., Wu S., Gu M., Deng Z., Chen X., Chen S. et al. In vivo mutational characterization of DndE involved in DNA phosphorothioate modification. PLoS One. 2014; 9:e107981. 25269084 10.1371/journal.pone.0107981 PMC4182426

[56] Hu W. , Wang C., Liang J., Zhang T., Hu Z., Wang Z., Lan W., Li F., Wu H., Ding J. et al. Structural insights into DndE from Escherichia coli B7A involved in DNA phosphorothioation modification. Cell Res. 2012; 22:1203–1206. 22525332 10.1038/cr.2012.66 PMC3391021

[57] Aravind L. , Anantharaman V., Balaji S., Babu M.M., Iyer L.M. The many faces of the helix-turn-helix domain: transcription regulation and beyond. FEMS Microbiol. Rev. 2005; 29:231–262. 15808743 10.1016/j.femsre.2004.12.008

[58] Schreiter E.R. , Drennan C.L. Ribbon–helix–helix transcription factors: variations on a theme. Nat. Rev. Microbiol. 2007; 5:710–720. 17676053 10.1038/nrmicro1717

[59] Raumann B.E. , Brown B.M., Sauer R.T. Major groove DNA recognition by β-sheets: the ribbon-helix-helix family of gene regulatory proteins. Curr. Opin. Struct. Biol. 1994; 4:36–43.

[60] Nelson W.C. , Matson S.W. The F plasmid traY gene product binds DNA as a monomer or a dimer: structural and functional implications. Mol. Microbiol. 1996; 20:1179–1187. 8809770 10.1111/j.1365-2958.1996.tb02638.x

[61] Lum P.L. , Rodgers M.E., Schildbach J.F. TraY DNA recognition of its two F factor binding sites. J. Mol. Biol. 2002; 321:563–578. 12206773 10.1016/s0022-2836(02)00680-0

[62] Xiong W. , Zhao G., Yu H., He X. Interactions of Dnd proteins involved in bacterial DNA phosphorothioate modification. Front Microbiol. 2015; 6:1139. 26539172 10.3389/fmicb.2015.01139 PMC4611135

[63] Cuylen S. , Metz J., Haering C.H. Condensin structures chromosomal DNA through topological links. Nat. Struct. Mol. Biol. 2011; 18:894–901. 21765419 10.1038/nsmb.2087

[64] Wilhelm L. , Bürmann F., Minnen A., Shin H.-C., Toseland C.P., Oh B.-H., Gruber S. SMC condensin entraps chromosomal DNA by an ATP hydrolysis dependent loading mechanism in Bacillus subtilis. Elife. 2015; 4:e06659. 25951515 10.7554/eLife.06659 PMC4442127

[65] Ramazzotti M. , Degl’Innocenti D., Manao G., Ramponi G. Entropy calculator: getting the best from your multiple protein alignments. Ital. J. Biochem. 2004; 53:16–22. 15356957

[66] Lum P.L. , Schildbach J.F. Specific DNA recognition by F factor TraY involves β-sheet residues. J. Biol. Chem. 1999; 274:19644–19648. 10391902 10.1074/jbc.274.28.19644

[67] Chivers P.T. , Sauer R.T. NikR is a ribbon-helix-helix DNA-binding protein. Protein Sci. 1999; 8:2494–2500. 10595554 10.1110/ps.8.11.2494 PMC2144182

[68] Golovanov A.P. , Barillà D., Golovanova M., Hayes F., Lian L.Y. ParG, a protein required for active partition of bacterial plasmids, has a dimeric ribbon-helix-helix structure. Mol. Microbiol. 2003; 50:1141–1153. 14622405 10.1046/j.1365-2958.2003.03750.x

[69] Wintjens R. , Rooman M. Structural classification of HTH DNA-binding domains and protein-DNA interaction modes. J. Mol. Biol. 1996; 262:294–313. 8831795 10.1006/jmbi.1996.0514

[70] Roberts R.J. , Belfort M., Bestor T., Bhagwat A.S., Bickle T.A., Bitinaite J., Blumenthal R.M., Degtyarev S., Dryden D.T., Dybvig K. et al. A nomenclature for restriction enzymes, DNA methyltransferases, homing endonucleases and their genes. Nucleic Acids Res. 2003; 31:1805–1812. 12654995 10.1093/nar/gkg274 PMC152790

[71] Wilson G.G. , Murray N.E Restriction and modification systems. Annu. Rev. Genet. 1991; 25:585–627. 1812816 10.1146/annurev.ge.25.120191.003101

[72] Makarova K.S. , Wolf Y.I., Koonin E.V. Comparative genomics of defense systems in archaea and bacteria. Nucleic Acids Res. 2013; 41:4360–4377. 23470997 10.1093/nar/gkt157 PMC3632139

[73] Rao D.N. , Dryden D.T., Bheemanaik S. Type III restriction-modification enzymes: a historical perspective. Nucleic Acids Res. 2014; 42:45–55. 23863841 10.1093/nar/gkt616 PMC3874151

[74] Loenen W.A. , Dryden D.T., Raleigh E.A., Wilson G.G. Type I restriction enzymes and their relatives. Nucleic Acids Res. 2014; 42:20–44. 24068554 10.1093/nar/gkt847 PMC3874165

[75] Aravind L. , Walker D.R., Koonin E.V. Conserved domains in DNA repair proteins and evolution of repair systems. Nucleic Acids Res. 1999; 27:1223–1242. 9973609 10.1093/nar/27.5.1223 PMC148307

[76] Anantharaman V. , Aravind L. The NYN domains: novel predicted RNAses with a PIN domain-like fold. RNA Biol. 2006; 3:18–27. 17114934 10.4161/rna.3.1.2548

[77] Anantharaman V. , Makarova K.S., Burroughs A.M., Koonin E.V., Aravind L. Comprehensive analysis of the HEPN superfamily: identification of novel roles in intra-genomic conflicts, defense, pathogenesis and RNA processing. Biol. Direct. 2013; 8:15. 23768067 10.1186/1745-6150-8-15 PMC3710099

[78] Aravind L. , Iyer L.M. The HARE-HTH and associated domains: novel modules in the coordination of epigenetic DNA and protein modifications. Cell Cycle. 2012; 11:119–131. 22186017 10.4161/cc.11.1.18475 PMC3272235

[79] Kisiala M. , Copelas A., Czapinska H., Xu S.Y., Bochtler M. Crystal structure of the modification-dependent SRA-HNH endonuclease TagI. Nucleic Acids Res. 2018; 46:10489–10503. 30202937 10.1093/nar/gky781 PMC6212794

[80] Liu G. , Fu W., Zhang Z., He Y., Yu H., Wang Y., Wang X., Zhao Y.-L., Deng Z., Wu G. et al. Structural basis for the recognition of sulfur in phosphorothioated DNA. Nat. Commun. 2018; 9:4689. 30409991 10.1038/s41467-018-07093-1 PMC6224610

[81] Makarova K.S. , Anantharaman V., Aravind L., Koonin E.V. Live virus-free or die: coupling of antivirus immunity and programmed suicide or dormancy in prokaryotes. Biol. Direct. 2012; 7:40. 23151069 10.1186/1745-6150-7-40 PMC3506569

[82] Anantharaman V. , Iyer L.M., Aravind L. Ter-dependent stress response systems: novel pathways related to metal sensing, production of a nucleoside-like metabolite, and DNA-processing. Mol. Biosyst. 2012; 8:3142–3165. 23044854 10.1039/c2mb25239b PMC4104200

[83] Wah D.A. , Hirsch J.A., Dorner L.F., Schildkraut I., Aggarwal A.K. Structure of the multimodular endonuclease FokI bound to DNA. Nature. 1997; 388:97–100. 9214510 10.1038/40446

[84] Wah D.A. , Bitinaite J., Schildkraut I., Aggarwal A.K. Structure of FokI has implications for DNA cleavage. Proc. Natl. Acad. Sci. U.S.A. 1998; 95:10564–10569. 9724743 10.1073/pnas.95.18.10564 PMC27934

[85] Leipe D.D. , Koonin E.V., Aravind L. Evolution and classification of P-loop kinases and related proteins. J. Mol. Biol. 2003; 333:781–815. 14568537 10.1016/j.jmb.2003.08.040

[86] Flodman K. , Corrêa I.R., Dai N., Weigele P., Xu S.Y In vitro type II restriction of bacteriophage DNA with modified pyrimidines. Front. Microbiol. 2020; 11:604618. 33193286 10.3389/fmicb.2020.604618 PMC7653180

[87] Hutinet G. , Lee Y.-J., de Crécy-Lagard V., Weigele Peter R Hypermodified DNA in viruses of E. coli and Salmonella. EcoSal Plus. 2021; 9:eESP–0028–2019. 10.1128/ecosalplus.ESP-0028-2019 PMC11163837 34910575

[88] Iyer L.M. , Zhang D., Burroughs A.M., Aravind L. Computational identification of novel biochemical systems involved in oxidation, glycosylation and other complex modifications of bases in DNA. Nucleic Acids Res. 2013; 41:7635–7655. 23814188 10.1093/nar/gkt573 PMC3763556

[89] Iyer L.M. , Burroughs A.M., Anand S., de Souza R.F., Aravind L. Polyvalent proteins, a pervasive theme in the intergenomic biological conflicts of bacteriophages and conjugative elements. J. Bacteriol. 2017; 199:e00245-17. 28559295 10.1128/JB.00245-17 PMC5512222

[90] Burroughs A.M. , Aravind L. Identification of uncharacterized components of prokaryotic immune systems and their diverse eukaryotic reformulations. J. Bacteriol. 2020; 202:e00365-20. 32868406 10.1128/JB.00365-20 PMC7685563

[91] Kaur G. , Burroughs A.M., Iyer L.M., Aravind L. Highly regulated, diversifying NTP-dependent biological conflict systems with implications for the emergence of multicellularity. Elife. 2020; 9:e52696. 32101166 10.7554/eLife.52696 PMC7159879

[92] Fairman-Williams M.E. , Guenther U.P., Jankowsky E. SF1 and SF2 helicases: family matters. Curr. Opin. Struct. Biol. 2010; 20:313–324. 20456941 10.1016/j.sbi.2010.03.011 PMC2916977

[93] Anantharaman V. , Koonin E.V., Aravind L. Comparative genomics and evolution of proteins involved in RNA metabolism. Nucleic Acids Res. 2002; 30:1427–1464. 11917006 10.1093/nar/30.7.1427 PMC101826

[94] Boraston A.B. , Bolam D.N., Gilbert H.J., Davies G.J. Carbohydrate-binding modules: fine-tuning polysaccharide recognition. Biochem. J. 2004; 382:769–781. 15214846 10.1042/BJ20040892 PMC1133952

[95] Chidyausiku T.M. , Mendes S.R., Klima J.C., Nadal M., Eckhard U., Roel-Touris J., Houliston S., Guevara T., Haddox H.K., Moyer A. et al. De novo design of immunoglobulin-like domains. Nat. Commun. 2022; 13:5661. 36192397 10.1038/s41467-022-33004-6 PMC9530121

[96] Cheng X. , Roberts R.J. AdoMet-dependent methylation, DNA methyltransferases and base flipping. Nucleic Acids Res. 2001; 29:3784–3795. 11557810 10.1093/nar/29.18.3784 PMC55914

[97] Bujnicki J.M. Sequence permutations in the molecular evolution of DNA methyltransferases. BMC Evol. Biol. 2002; 2:3. 11914127 10.1186/1471-2148-2-3 PMC102321

[98] Shen B.W. , Xu D., Chan S.H., Zheng Y., Zhu Z., Xu S.Y., Stoddard B.L. Characterization and crystal structure of the type IIG restriction endonuclease RM.BpuSI. Nucleic Acids Res. 2011; 39:8223–8236. 21724614 10.1093/nar/gkr543 PMC3185434

[99] Kachalova G.S. , Rogulin E.A., Yunusova A.K., Artyukh R.I., Perevyazova T.A., Matvienko N.I., Zheleznaya L.A., Bartunik H.D. Structural analysis of the heterodimeric type IIS restriction endonuclease R.BspD6I acting as a complex between a monomeric site-specific nickase and a catalytic subunit. J. Mol. Biol. 2008; 384:489–502. 18835275 10.1016/j.jmb.2008.09.033

[100] Osipiuk J. , Walsh M.A., Joachimiak A. Crystal structure of MboIIA methyltransferase. Nucleic Acids Res. 2003; 31:5440–5448. 12954781 10.1093/nar/gkg713 PMC203307

[101] Iyer L.M. , Makarova K.S., Koonin E.V., Aravind L. Comparative genomics of the FtsK-HerA superfamily of pumping ATPases: implications for the origins of chromosome segregation, cell division and viral capsid packaging. Nucleic Acids Res. 2004; 32:5260–5279. 15466593 10.1093/nar/gkh828 PMC521647

[102] Iyer L.M. , Leipe D.D., Koonin E.V., Aravind L. Evolutionary history and higher order classification of AAA+ ATPases. J. Struct. Biol. 2004; 146:11–31. 15037234 10.1016/j.jsb.2003.10.010

[103] Zhang D. , Burroughs A.M., Vidal N.D., Iyer L.M., Aravind L. Transposons to toxins: the provenance, architecture and diversification of a widespread class of eukaryotic effectors. Nucleic Acids Res. 2016; 44:3513–3533. 27060143 10.1093/nar/gkw221 PMC4857004

[104] Traore D.A.K. , Wisniewski J.A., Flanigan S.F., Conroy P.J., Panjikar S., Mok Y.-F., Lao C., Griffin M.D.W., Adams V., Rood J.I. et al. Crystal structure of TcpK in complex with oriT DNA of the antibiotic resistance plasmid pCW3. Nat. Commun. 2018; 9:3732. 30213934 10.1038/s41467-018-06096-2 PMC6137059

[105] Aravind L. , Leipe D.D., Koonin E.V. Toprim—a conserved catalytic domain in type IA and II topoisomerases, DnaG-type primases, OLD family nucleases and RecR proteins. Nucleic Acids Res. 1998; 26:4205–4213. 9722641 10.1093/nar/26.18.4205 PMC147817

[106] Iyer L.M. , Abhiman S., Aravind L. A new family of polymerases related to superfamily A DNA polymerases and T7-like DNA-dependent RNA polymerases. Biol. Direct. 2008; 3:39. 18834537 10.1186/1745-6150-3-39 PMC2579912

[107] Kaur H. , Ganguli D., Bachhawat A.K. Glutathione degradation by the alternative pathway (DUG pathway) in Saccharomyces cerevisiae is initiated by (Dug2p-Dug3p)2 complex, a novel glutamine amidotransferase (GATase) enzyme acting on glutathione. J. Biol. Chem. 2012; 287:8920–8931. 22277648 10.1074/jbc.M111.327411 PMC3308760

[108] Aravind L. , Iyer L.M., Leipe D.D., Koonin E.V. A novel family of P-loop NTPases with an unusual phyletic distribution and transmembrane segments inserted within the NTPase domain. Genome Biol. 2004; 5:R30. 15128444 10.1186/gb-2004-5-5-r30 PMC416466

[109] Essuman K. , Summers D.W., Sasaki Y., Mao X., Yim A.K.Y., DiAntonio A., Milbrandt J. TIR domain proteins are an ancient Family of NAD(+)-consuming enzymes. Curr. Biol. 2018; 28:421–430. 29395922 10.1016/j.cub.2017.12.024 PMC5802418

[110] Iyer L.M. , Burroughs A.M., Anantharaman V., Aravind L. Apprehending the NAD(+)-ADPr-dependent systems in the virus world. Viruses. 2022; 14:1977. 36146784 10.3390/v14091977 PMC9503650

[111] Makarova Kira S. , Wolf Yuri I., Snir S., Koonin Eugene V. Defense islands in bacterial and archaeal genomes and prediction of novel Defense systems. J. Bacteriol. 2011; 193:6039–6056. 21908672 10.1128/JB.05535-11 PMC3194920

[112] Nicastro G.G. , Burroughs A.M., Iyer L.M., Aravind L Functionally comparable but evolutionarily distinct nucleotide-targeting effectors help identify conserved paradigms across diverse immune systems. Nucleic Acids Res. 2023; 51:11479–11503. 37889040 10.1093/nar/gkad879 PMC10681802

[113] Krüger D.H. , Bickle T.A. Bacteriophage survival: multiple mechanisms for avoiding the deoxyribonucleic acid restriction systems of their hosts. Microbiol. Rev. 1983; 47:345–360. 6314109 10.1128/mr.47.3.345-360.1983 PMC281580

[114] Miller E.S. , Kutter E., Mosig G., Arisaka F., Kunisawa T., Rüger W. Bacteriophage T4 genome. Microbiol. Mol. Biol. Rev. 2003; 67:86–156. 12626685 10.1128/MMBR.67.1.86-156.2003 PMC150520

[115] Murphy J. , Mahony J., Ainsworth S., Nauta A., van Sinderen D Bacteriophage orphan DNA methyltransferases: insights from their bacterial origin, function, and occurrence. Appl. Environ. Microbiol. 2013; 79:7547–7555. 24123737 10.1128/AEM.02229-13 PMC3837797

