# Supplementary material for: Reappraisal of the DNA phosphorothioate modification machinery: uncovering neglected functional modalities and identification of new counter-invader defense systems
Source: Nucleic Acids Res. 2024 Jan 2;52(3):1005–26. doi: 10.1093/nar/gkad1213 (PMC10853773; doi:10.1093/nar/gkad1213)
Supplement: gkad1213_Supplemental_Files [file gkad1213_supplemental_files.zip › Supplementary_Dataset_2.pdf.pdf]

Supplementary_Dataset_2_Figures_Tables_Treefile_Index

1. Supplementary additional Figures:...1
   A. DndE clade 1 additional 3D structures.
   B. DndE clade 2 additional 3D structures.
   C. DndF P-loop Kinase 3D structure.
   D. DndE clade secondary structure length plot.

2. Supplementary table listing domain names, corresponding database designation, function and additional remarks…..2-3

3. DndE sequence-structure synapomorphy table and summary………..4

4. DndF sequence-structure synapomorphy table………………………..5

5. DndH sequence-structure synapomorphy table……………………….5

6. DndE Tree Raw file Newick Format………………………………….6

7. DndD/CxC ABC Tree Raw file Newick Format……………………...7

8. HerA/FtsK Capture Systems Tree Raw file Newick format…………..8

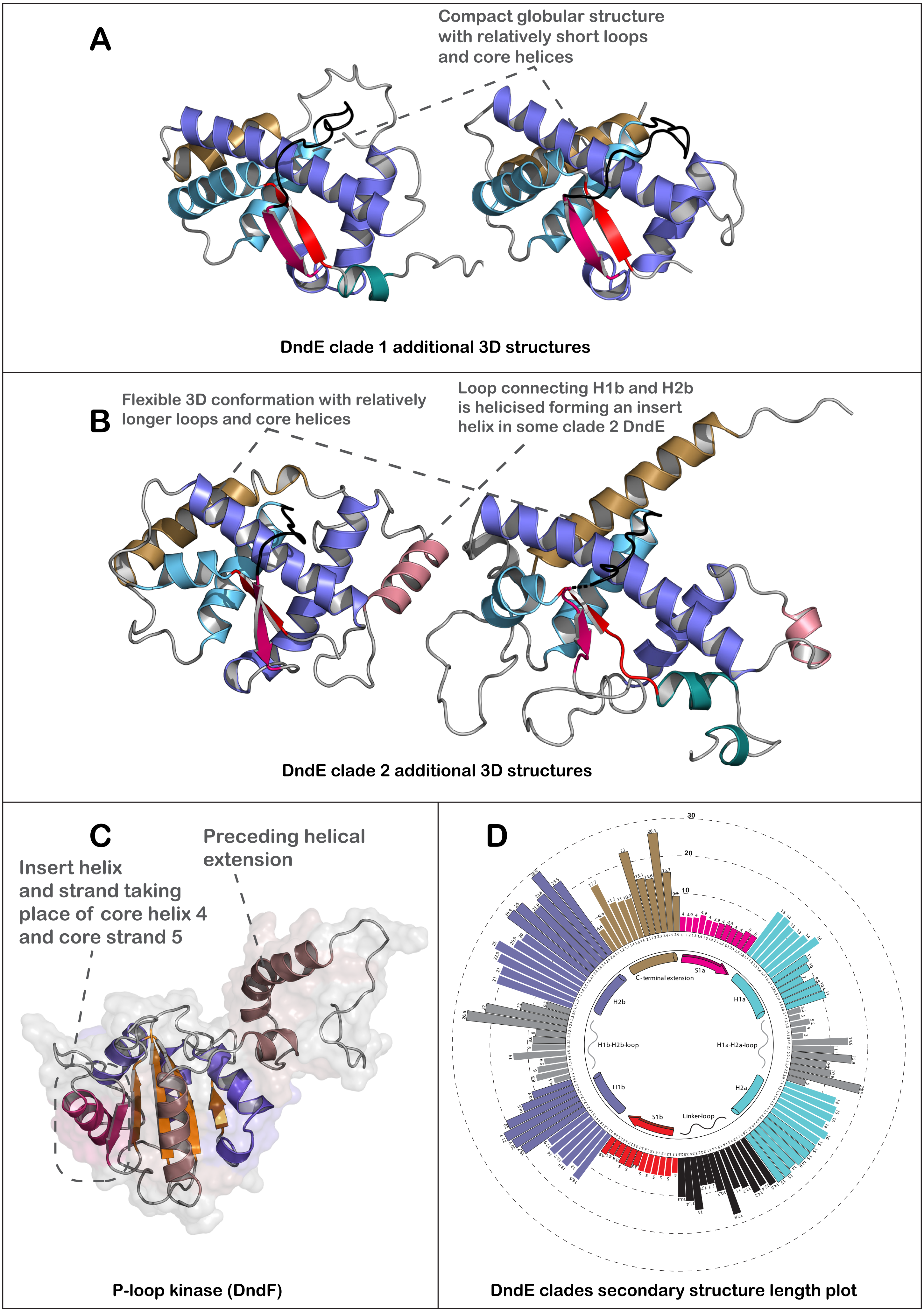
A
Compact globular structure
with relatively short loops
and core helices
DndE clade 1 additional 3D structures
B
Flexible 3D conformation with relatively
longer loops and core helices
Loop connecting H1b and H2b
is helicised forming an insert
helix in some clade 2 DndE
DndE clade 2 additional 3D structures
C
Insert helix
and strand taking
place of core helix 4
and core strand 5
Preceding helical
extension
P-loop kinase (DndF)
D
30
20
10
C - terminal extension
S1a
H1a
H2b
H1b-H2b-loop
H1a-H2a-loop
H1b
H2a
S1b
Linker-loop
DndE clades secondary structure length plot

Domain name	INTERPRO/PFAM designation	Function	Remarks	Reference
URI-DNase	GIY-YIG	Endonuclease activity.	URI (UvrC-Intron homing endonuclease superfamily) precedes the GIY-YIG motif and other numerous names in the Interpro/Pfam database. Appropriately, the term URI-DNase is used throughout the text.	**(1,2)**
REase	PD-D(D/E)XK family	Endonuclease activity.	All REase's in the gene neighborhoods and DndH domain architecture belong to the PD-D(D/E)XK clan of endonucleases. The term REase is utilised here because it precedes the term "PD-D(D/E)XK." Importantly, the motif itself is not consistently conserved within the PD-D(D/E)XK REase family. Therefore, REase is consistanttly used throughout.	**(3-5)**
Sp	Small Protein	Disordered protein with a probable negative regulatory role.	PbeB present in the Pbe system is the "Small protein" associated with Dnd systems.	**(6)**
HKD-DNase	Phospholipase-D	Endonuclease activity.	In the Pbe system, PbeA is HKD-DNase+SF2-Helicase.	**(7-9)**
DndD/CxC ABC-ATPase	-	ATPase activity.	Erroneously annotated as AAA+ ATPase in few papers. In the Pbe system, PbeC annotated as AAA+ ATPase is actually the CxC ABC-ATPase (Dnd system).<br>DndD/CxC ABC ATPase belongs to the coiled coil assemblage of ABC ATPase.	**(6,9,10)**
DndE	DndE	Encodes two RHH DNA binding domains.	Previously, in the Pbe system, PbeD is identified as a protein with unknown function. We identified it as DndE belonging to clade 2.	**(6,9)**
DndA	Cysteine Desulfurase	Transfers sulfur from cysteine to the target DNA, acting as a sulfur donor.	Core protein of the Dnd modification system.	**(11,12)**
DndB	ParB/DndB	Nuclease and/or ATPase activity.	Core protein of the Dnd modification system.	**(13)**
DndC	PAPS_reductase	Contains a 4Fe-4S Cluster, with ATP pyrophosphatase activity. Accepts the sulfur abstracted by DndA and incorporates it into the DNA backbone.	Core protein of the Dnd modification system.	**(11,12)**
wHTH+SAD/SRA+HNH	SBD (Sulfur binding domain)+SAD/SRA+HNH	PT sensing and endonuclease.	The N-terminal sulfur binding domain (SBD) is an elaborated wHTH domain, with PT sensing functionality, and the HNH acts as a sequence specific endonuclease. SAD corresponds to the SRA.	**(14,15)**

1. Aravind, L., Walker, D.R. and Koonin, E.V. (1999) Conserved domains in DNA repair proteins and evolution of repair systems. *Nucleic Acids Res*, **27**, 1223-1242.
2. Stoddard, B.L. (2005) Homing endonuclease structure and function. *Q Rev Biophys*, **38**, 49-95.
3. Steczkiewicz, K., Muszewska, A., Knizewski, L., Rychlewski, L. and Ginalski, K. (2012) Sequence, structure and functional diversity of PD-(D/E)XK phosphodiesterase superfamily. *Nucleic Acids Res*, **40**, 7016-7045.
4. Kosinski, J., Feder, M. and Bujnicki, J.M. (2005) The PD-(D/E)XK superfamily revisited: identification of new members among proteins involved in DNA metabolism and functional predictions for domains of (hitherto) unknown function. *BMC Bioinformatics*, **6**, 172.
5. Aravind, L., Makarova, K.S. and Koonin, E.V. (2000) SURVEY AND SUMMARY: holliday junction resolvases and related nucleases: identification of new families, phyletic distribution and evolutionary trajectories. *Nucleic Acids Res*, **28**, 3417-3432.
6. Krishnan, A., Burroughs, A.M., Iyer, L.M. and Aravind, L. (2020) Comprehensive classification of ABC ATPases and their functional radiation in nucleoprotein dynamics and biological conflict systems. *Nucleic Acids Res*, **48**, 10045-10075.
7. Ponting, C.P. and Kerr, I.D. (1996) A novel family of phospholipase D homologues that includes phospholipid synthases and putative endonucleases: identification of duplicated repeats and potential active site residues. *Protein Sci*, **5**, 914-922.
8. Koonin, E.V. (1996) A duplicated catalytic motif in a new superfamily of phosphohydrolases and phospholipid synthases that includes poxvirus envelope proteins. *Trends Biochem Sci*, **21**, 242-243.
9. Xiong, L., Liu, S., Chen, S., Xiao, Y., Zhu, B., Gao, Y., Zhang, Y., Chen, B., Luo, J., Deng, Z. *et al.* (2019) A new type of DNA phosphorothioation-based antiviral system in archaea. *Nature Communications*, **10**, 1688.
10. Xiong, X., Wu, G., Wei, Y., Liu, L., Zhang, Y., Su, R., Jiang, X., Li, M., Gao, H., Tian, X. *et al.* (2020) SspABCD–SspE is a phosphorothioation-sensing bacterial defence system with broad anti-phage activities. *Nature Microbiology*, **5**, 917-928.
11. You, D., Wang, L., Yao, F., Zhou, X. and Deng, Z. (2007) A Novel DNA Modification by Sulfur: DndA Is a NifS-like Cysteine Desulfurase Capable of Assembling DndC as an Iron−Sulfur Cluster Protein in Streptomyces lividans. *Biochemistry*, **46**, 6126-6133.
12. An, X., Xiong, W., Yang, Y., Li, F., Zhou, X., Wang, Z., Deng, Z. and Liang, J. (2012) A Novel Target of IscS in Escherichia coli: Participating in DNA Phosphorothioation. *PLOS ONE*, **7**, e51265.
13. Maindola, P., Raina, R., Goyal, P., Atmakuri, K., Ojha, A., Gupta, S., Christie, P.J., Iyer, L.M., Aravind, L. and Arockiasamy, A. (2014) Multiple enzymatic activities of ParB/Srx superfamily mediate sexual conflict among conjugative plasmids. *Nat Commun*, **5**, 5322.
14. Liu, G., Fu, W., Zhang, Z., He, Y., Yu, H., Wang, Y., Wang, X., Zhao, Y.-L., Deng, Z., Wu, G. *et al.* (2018) Structural basis for the recognition of sulfur in phosphorothioated DNA. *Nature Communications*, **9**, 4689.
15. Yu, H., Liu, G., Zhao, G., Hu, W., Wu, G., Deng, Z. and He, X. (2018) Identification of a conserved DNA sulfur recognition domain by characterizing the phosphorothioate-specific endonuclease SprMcrA from Streptomyces pristinaespiralis. *Mol Microbiol*, **110**, 484-497.

DndE sequence-structure synapomorphy table and summary

	N-Terminal	STRAND-1a	LOOP	HELIX-1a	LOOP	HELIX-2a	LINKER LOOP	STRAND-1b	LOOP	HELIX-1b	LOOP	INSERT-HELIX	LOOP	HELIX-2b	LOOP	C-TERMINAL EXTENSION	REMARKS
**clade_1.1**		4-5 residues, no conserved motif except for R/K in 3rd residue of strand, pointing towards binding substrate.		14 residues, begins with polar & small residues followed by highly conserved L, pointing towards helical core. +ve charged residues towards the end, with conserved K & R pointing away from core. Ends with conserved polar residue S/T.	4-5 residues, short loop with a **characteristic motif 'GxxxxN' or 'GLTPN'** in the junction between this loop and helix-2a	14-15 residues, Highly **conserved motif 'NxxCRhhhhS'**, (**NxxxR** is unique synapomorphy of entire clade 1). NxxCR is followed by hydrophobic stretch and conserved SL towards end. Two conserved C are present within these conserved stretch, some have lost one C.	14-15 residues long loop, rich in polar and small amino acid residues.	4 residues, E & hydrophobic residues M/I/L/F are conserved. E pointing towards binding substrate.	7-8 residues.	15 residues, begins with -ve charged followed by a conserved hydrophobic stretch of residues with few -ve charged residues in between.	4-5 residues short loop with no conserved residues.			22-23 residues, Begins with conserved polar & -ve charged residues, followed by hydrophobic stretch. Has a **highly conserved motif 'HxxRG'** (**HxxxG is unique synapo-morphy of entire clade 1,** where H is sometimes replaced by other reidues and **'E/DxG' is found instead.**		6 residues, short helical extension compared to clade-1 represen-tatives, with no significant conservation of residues.	Structurally and topologically more compact than clade 2 representatives. The anchoring helix-2b of the core helical bundle is significantly smaller than clade 2. Consists of well defined synapomorphic motifs **'GxxxN'** & **'NxxxR'** in helix-2a, and **'HxxxG'** in helix-2b. Presence of conserved C in **'NxxCR' unit** & **'Hx-H/E/N-RG'** motif are the uniqe features of this particular clade.
**clade_1.2**		4 residues, No conserved motif, Mainly containing +ve charged residues K & R, pointing away towards binding substrate.		14 residues, rich in polar & hydrophobic residues.	2-3 residues, No 'GxxxN' motif as seen in some other clade 1 represen-tatives.	15 residues, **conserved motif 'NxxxR' is absent**. Instead R of this motif is replaced by ***conserved K*** followed by a hydrophobic stretch. (same feature is found in clade 1.2b) The unique conserved motif is ***'KhhhhKG'.***	14-15 residues long loop, rich in small & polar residues, with no conservation.	4-5 residues, conserved unit 'ELN', E & N both pointing towards binding subs-trate.	7-8 residues, conserved TI followed by -ve residues.	12-13 residues, Begins with conserved D, followed by hydrophobic stretch with conse-rved K in between similar to clade 1.1c.	10 residues, highly conserved -ve charged stretch **'EEE/DEE'**.			21-22 residues, begins with stretches of hydrophobic & +ve charged residues, Signature motif **'HxxxG'** is represe-nted by **'Hx-D/E-RG' motif.** H & R is lost in some cases. Ends with stretches of polar & hydrophobic residues.	2-3 residues short loop.	10 residues, polar and hydrophobic residues with no conservation.	Structural features are of canonical clade 1 types, with one major difference being the complete absence of characteristic **'NxxxR' motif** in helix-1b, where R is replaced by K and maintains the same orientation as of R. -ve charged stretch **EEE** in loop connecting helix-1b & helix-2b is found. ***'KhhhhKG'*** in helix-2a is unique feature of this subclade, and **'HxxxG'** is present in helix-2b, with some representatives replacing H with **Y/L.** (H is completely lost in clade 1.2b)
**clade_1.3+FtsK**		4-5 residues, no conserved motif, polar and +ve charged residues, +ve residues H & K pointing away towards binding substrate.		14 residues, Conserved 'NK' near the beginning, followed by hydrophobic stretch and +ve residues TxK towards the end.	3-4 residues with conserved L & G.	15 residues, Highly **conserved motif 'E-NxxxR',** N & R orientation is same as that of canonical types. R is followed by hydrophobic stretch and a conserved 'SL' towards the end.	12-13 residues long loop, rich in small, polar & hydrophobic residues. Junction between this loop and strand-1b, has a highly conserved motif **'SKGK/SQGK/SDGK/SGGK',** where **GK** belonging to strand-1b is fully conserved and is a ***unique synapomorphy of this sub-clade.***	5 residues, conserved motif '**GK**EY', K & E pointing towards binding substrate.	7-8 residues with conserved hydrophobic stretch.	13-14 residues, conserved -ve residues near begin-ning followed by hydrophobic stretch and a C near the end. C (or D) is followed by a highly **conserved Q** in Ftsk fusion version of this clade.	6-7 residues with conserved yunit 'DKD' towards the end.			23-24 residues, begins with -ve residues D, followed by hydrophobic stretch with a conserved K in between, pointing towards anti-parallel strands. Signature motif **'HxxxG',** is represented by **'HxDDG' motif.**	4-5 residues, rich in polar amino acids.	16-18 residues, long helical extension, conserved D followed by stretches of hydrophobic & polar residues.	Relatively longer anchor helix-2b, and a longer C-terminal helical extension. Presence of conserved motif **'SKGK/SQGK/SDGK/SGGK',** in junction of loop and strand-1b is the major synapomorphy of this subclade. Presence of **'HxDDG' motif** in helix-2b & a *slightly longer C-terminal extension compared to other clade 1 counter-parts* are some other unique features of this subclade.
**clade_1.4+FtsK**	***Short preceding helix with polar & hydrophobic residues.***	5 residues, no conserved motif, F is conserved residue along with some +ve residues K/R, pointing away towards binding substrate.		14 residues, rich in polar, small & -ve residues with no significant conservation of amino acids.	4-5 residues with stretches of polar amino acids.	14-15 residues, **N is is replaced by Y/A/F** from conserved motif **'NxxxR'.** R is followed by hydrophobic stretch and a conserved 'SL' towards the end.	12-13 residues long loop, no conservation of amino acids.	5 residues, conserved I & +ve residues R/K, pointing towards binding substrate. Conserved motif "G(R/K)xI(R/K)".	8 residues with conserved unit 'LFG'.	14 residues, Begins with conserved -ve D, follo-wed by hydrophobic stretch with a *highly conserved W*, oriented towards helix-2a.	6 residues short loop, no conservation of amino acids.			25 residues, Begins with polar residues & conserved V, followed by characteristic **motif 'HxxxG',** contatining a highly conserved W. This motif is represented by **'HWxRG'.** ends with stretches of hydrophobic & -ve residues.	4 residues, rich in -ve charged amino acids.	12-13 residues, conserved stretches of hydropho-bic and +ve charged residues.	Anchor helix-2b is longer. Presence of ***long N-terminal loop*** and ***preceding helix*** are unique synapomorphies of this subclade. N is lost from **'NxxxR' motif** in most cases. **'HxxxG' motif** is represented by **'HWxRG'** (W is highly conserved)
**clade_1.5+FtsK**		4 residues, conserved motif 'DRLYT', R & Y pointing towards binding substrate.		14-15 residues, conserved DE followed by L & R towards the end. R is pointing away from core.		15-16 residues, **N is is replaced by hydrophobic residues Y/A/V,** from conserved motif **'NxxxR'.** R is followed by hydrophobic stretch and conserved 'SL' towards the end.	11-12 residues, rich in polar and hydrophobic residues, with conserved stretches of S & T.	5 residues, conserved motif 'GGEhR', E & R are pointing towards binding substrate.	8 residues, conserved T & F.	14 residues, begins with conserved -ve charged D/E residues, followed by hydro-phobic stretch with conserved +ve residues R/K in between. Ends with stretches of hydrophobic residues.	15 residues long loop, Conserved -ve charged stretch **'EEE/DEE'** followed by conserved **'polar stretch SNKS'.**			20 residues, Begins with conserved hydrophobic residues, followed by **'HxxxG' motif,** represented by **'HxD-N/S-G'.** Followed by stretches of hydrophobic & -ve residues. 'HxxxG' motif is *more towards the initial part of helix-2b* unlike in other clades where they are located in middle of helix-2b.		11-12 residues, rich in polar, hydrophobic & -ve charged amino acids.	Structurally similar to canonical clade 1 subtypes. Anchor helix-2b is not longer, but the *loop connecting helix-1b & helix-2b of 2nd RHH unit are longer than usual* (with a possible helical insert). This long loop along with the **polar stretch residues 'SNRS'** are some unique features of this clade.
**clade_1.6+FtsK**		4 residues, no conserved motif, polar & +ve charged resdiues, N & R pointing towards binding substrate.		16 residues, Begins with -ve residue D/E, no conservation of amino acids, mainly consists -ve charged, hydrophobic & polar residues.	*15-16 residues long loop, highly conserved NL in the beginning followed by polar and small residues with no conservation. The long loop protrudes away from the helical core, but the topology of helix of 1st RHH unit are maintained.*	15-16 residues, Begins with *conserved polar stretch* followed by characteristic **'NxxxR' motif** and a conserved C right after **'NxxxR'.** It is followed by another conserved hydrophobic stretch and conserved unit 'GLS' towards the end.	16 residues *long loop,* mainly consists of polar & hydro-phobic residues and a highly conserved R.	5 residues, no conserved motif, rich in polar, +ve charged & hydrophobic residues. T & R pointing towards binding substrate.	9 residues, conserved motif of polar & hydro-phobic residues 'SFFT/TVFT'.	14 residues, Begins with hydrophobic stretch, followed by conserved unit **'ALLKxRY',** both +ve residues K & R pointing away from the core.	8-9 residues, rich in -ve charged & hydro-phobic residues, Conserved 'DW' towards the end.			20 residues, Begins with stretches of polar & hydro-phobic residues, conserved residues S-R/K, followed by a hydrophobic stretch and the characteristic **'HxxRG' motif.** **H is lost** and **replaced by E**, and hence the motif is represented by **'ExxRG' unit.** Ends with highly conserved L.		11-12 residues, Rich in polar residues and conserved hydrophobic residues L/W.	Structurally and topologically similar to canonical clade 1 types. The *loops connecting helix-1a to helix-2a & helix-2a to strand-1b are exceptionally longer compared to other clade 1 variants.* H is completely lost and replaced by E from **'HxxRG' motif** of helix-2b, which is represented by **'ExxRG'.**
	**N-Terminal**	**STRAND-1a**	**LOOP**	**HELIX-1a**	**LOOP**	**HELIX-2a**	**LOOP**	**STRAND-1b**	**LOOP**	**HELIX-1b**	**LOOP**	**INSERT-HELIX**	**LOOP**	**HELIX-2b**	**LOOP**	**C-TERMINAL EXTENSION**	**REMARKS**
**clade_2.1**		3-4 residues, conserved motif 'RxR', Both R pointing away towards binding substrate.		10-12 residues, -ve residues with D & E being most prevalent.	12-13 residues, conserved F towards the end, long loop protruding away from helical core.	15 residues, conserved **D/E** or polar residues followed by hydrophobic stretch. conserved +ve residues near the end.	11-12 residues, conserved +ve charged stretch with R pointing away from core.	3-4 residues, conserved stretch 'P-I/H-x'.		20-21 residues, conserved F near beginning, Mainly hydrophobic and aliphatic or aromatic residues towards the later half.	10-11 residues loop, conserved L in middle.	probable short helical insert.		26-27 residues, conserved universal motif **'ExYxNGG'** of clade 2. **'EEYANGG'** singature of this subclade. EE points away from helical core.	4-5 residues short loop.	20-21 residues, rich in hydrophobic and polar residues.	Most widespread subclade within clade 2. Displays all canonical sequence and structural features of clade 2. No preceding or insert helices. Loops connecting core helices are with more open configu-ration.
**clade_2.2**		5 residues, no conserved motif, Hydrophobic residues I/F/L pointing towards binding substrate.		10-11 residues, polar, hydrophobic residues with conserved E in midpoint.	15-16 residues, long loop protruding away from helical core, rich in polar residues.	15 residues, **conserved D** followed by hydro-phobic stretch, conserved +ve residues near the end.	9-10 residues, no conservation of residues.	5-6 residues, conserved T pointing towards binding substrate, followed by hydrophobic residues.		21-22 residues, conserved motif 'DxxR' followed by hydrophobic stretch.		probable short helical insert.		25-26 residues, **'ExYxNGG'** universal motif is distorted. *E is replaced by polar residue.*		15-20 residues, rich in hydrophobic and polar residues.	Structural and sequence features are same as clade 2.1, except the **'ExYxNGG'** universal motif is different where *E is replaced by conserved polar residue N/S.*
**clade_2.3**		4 residues, conserved motif 'F-R/K-I/L-x' (similar to RxR), 1st R pointing towards binding substrate.		8-9 residues, highly conserved W/Y & F, pointing in.	10 residues, rich in polar and small residues.	15 residues, **conserved D** followed by hydro-phobic stretch with a highly conserved C in between.	8 residues, rich in polar residues.	5 residues with no significant conservation of residues except a hydrophobic F/L/M & V.	4 residues short loop.	22-23 residues, begins with conserved stretch of aromatic and polar residues, followed by hydrophobic stretch & a highly conserved E towards the end.	7-8 residues short loop.	**11-12 residues, rich in polar & hydrophobic residues.**	10 residues with conserved polar stretch residues.	21-22 residues, 'ExYxNGG' universal motif is distorted. Instead E is replaced by highly conserved N oritented towards strands. **'NxYAxxG'** is the signature motif of this subclade.	7-8 residues short loop.	14-15 residues, hydrophobic and polar residues.	Helix-1a is relatively shorter, **insert helix** & **distorted 'ExYxNGG'** motif are the synapomorphies of this clade. Sister clade to subclade 2.4 & has shorter loops in 1st RHH unit.
**clade_2.4**		4 residues, no conserved motif, rich in R & Y pointing towards binding substrate.		9-10 residues, conserved motif 'KHAx', H is highly conserved.	10-14 residues, long loop protruding away from helical core, F & R near ends are conserved.	15 residues, **conserved D** or polar residues followed by hydrophobic stretch, signature motif 'P/L/I-xxG'.	12-20 residues, long loop protruding away on top of anti-parallel sheets.	4 residues, no conserved motif, conserved residue I.		21-22 residues, begins with -ve residues, F & R towards end is conserved followed by hydrophobic stretch.		**12 residues, conserved R near the beginning & AFR motif towards the end, F & R points away from the core.**	4-5 residues, with conserved polar stretch residues.	21-22 residues, **'ExYxNGG'** universal motif is distorted. Instead *E is replaced by polar residues.* conserved motif 'EhLYE' towards the end.	5-6 residues short loop with polar stretch.	25-30 residues, elongated multi helical extension, rich in -ve & polar residues.	Helix-1a is relatively shorter, **insert helix** & **distorted 'ExYxNGG'** motif are synapomorphies of this clade. Loops connecting core helices are much longer than sister clade 2.3.
**clade_2.5**	7-8 residues, **short preceding helix** rich in polar & hydrophobic residues.	3- 4 residues, no conserved motif, rich in +ve residues R/K pointing towards binding substrate.		11-12 residues, rich in polar & hydrophobic residues with no conservation.	20-21 residues, long loop protruding away from helical core. conserved GK towards the end.	15 residues, **conserved E** followed by hydro-phobic stretch.	11-12 residues, rich in polar & hydrophobic residues.	4 residues, no conserved motif.	14-20 residues, long loop protruding away from helical core along with loop connecting core helices of 1st RHH unit. rich in +ve residues R & K,	21-22 residues, rich in polar & hydrophobic stretches of residues.		probable short helical insert with a conserved hydro-phobic & -ve residues.		28-29 residues, **'ExYxNGG'** universal motif is preserved.	8-9 residues, conserved F pointing to-wards helix-2b.	15-20 residues, conserved F pointing towards helix-2a.	**N-terminal preceding helix & significantly longer loop after strand-1b protruding away along with 1st RHH unit long loop are major synapomorphies. Universal 'ExYxNGG' motif is somewhat preserved.**
**clade_2.6**	N-terminal loop of varying length, rich in -ve & polar residues.	5 residues, conserved motif 'xIGL'.		12-13 residues, polar & hydrophobic residues with no conservation.	5 residues, short loop with conserved F.	15 residues, conserved motif **'DxxR/K',** sequence features similar to clade 2 representatives, except for ***highly conserved K/R.*** orientation of **K/R** is similar to **R of 'NxxxR' motif** from clade 1.	10-11 residues, no significant conservation.	5-6 residues, conserved T/S/N both at the beginning & end, T pointing towards binding substrate.	8-10 residues, conserved motif 'DPD' and small residues.	10-12 residues, short helix compared to clade 2, rich in hydrophobic & aliphatic residues with no conservation.	6-7 residues short loop.			20-22 residues, **conserved motif 'LxxxG',** slightly conserved E before this motif with orientation similar to E of 'ExYxNGG' motif from clade 2.		C-terminal helical extension is missing in some representatives, instead having a long loop with no coservation. Some representatives has this extension slightly shorter than those present in clade 2.	Structurally and topologically more similar to clade 1, with more compact core helices and shorter loops. Sequence features of core helix-2a and helix-2b are similar to both clade 2 and clade 1. No probable helical insert in 2nd RHH unit. Overally more closer to clade 1 based on structure and topology.

Summary

At the sequence level, clade-1 and clade-2 DndEs can be distinguished by two prominent sequence signatures precisely at the second helix of the first and second RHH domains (H2a and H2b) as described below. For instance, the clade-1 is typified by the "NhhhRhhhhhS" motif that spans the entire length of H2a: the asparagine at the beginning, arginine at the middle, and serine at the end of H2a are almost always conserved, while the "h" denotes hydrophobic residues that are mostly aliphatic. Conversely, clade-2 is typified by a conserved "D/EhhhhhhhhG/A" motif spanning the H2a, where the aspartate/glutamate (D/E) occurs at the beginning of H2a followed by a hydrophobic stretch of a fixed length containing eight residues and a mostly conserved glycine/alanine (G/A) at the end of H2a and a variation thereof. This is in contrast to clade-1 where positively charged residues in the form of arginine in the middle of H2a are absent.

The second prominent and distinguishing motif for clade-1 is an "H(D/E)xxxR" motif at the middle of long helix H2b that extends on an average over 20 residues. The histidine at the beginning of the motif is mostly conserved while the aspartate and glutamate (D/E) are also observed at the same position. Although variability is seen at the three positions (xxx) followed by H, arginine, histidine, tyrosine, and occasionally a tryptophan is found to be more common among other residues. The arginine at the end of the motif is almost always conserved and is unique for clade-1. On the contrary, clade-2 is typified by "E/NcYsN/AGG" in the middle of H2b. The "c" denotes a charged residue immediately followed by a conserved E/N and is usually glutamate and occasionally an arginine or lysine (R/K) and is less conserved relative to E/N. The "s" followed by tyrosine (Y) denotes a small residue and is an alanine in most sequences. Of the two glycines at the end of the motif, the second glycine is almost always conserved, whereas the first is replaced by other small residues.

The DNA binding interface exhibits high sequence variability. . First, there is no occurrence of any universally conserved residue across all DndEs in both S1a and S1b, nor in the helices that are involved in the backbone contacts with the DNA. For instance, S1a usually displays a variable sequence signature which can hitherto be summarized as “(b/p)h(b/p)” based on the characteristics of the sidechains, where b denotes basic, p-polar, and h-hydrophobic. These occur as R(N/T/S)hR(K/T/H) motifs across both clade-1 and clade-2. The first arginine is only partially conserved and is substituted mainly by an asparagine, preferentially followed by threonine and serine. The second arginine that occurs at the top of S1a is mostly retained and is occasionally substituted by lysine, threonine, and histidine. Nonetheless, among the subclades where the motif is observed, we find that collectively at least one positively charged residue (mainly arginine) is retained in S1a that potentially contributes to the interaction with the DNA. In a subtle variation to the above, other clades display a pattern where the asparagine that substituted the first arginine is more prevalent or completely conserved, while the second arginine is either sporadically present or mostly replaced by lysine, threonine, and serine. In these instances, the motif occurs as N(T/S/R)h(R/T/S/K) and can form hydrogen bonds with the bases. Other distinctive motifs that are restricted to a particular subclade include a conserved “NRR” and “DRLYT” that occurs in clades 1.1 and 1.5, respectively. Unlike the RHH homodimers where both the strands will be sequentially identical (MetJ, Arc, etc), the strand of the second RHH unit (S1b) in DndE displays extensive diversity. In most subclades, no clearly delineable motif occurs in S1b. On average, the S1b constitutes 5 residues and It can be summarized where at least three residues retain the polar character, while the other two are hydrophobic. While there is no strong preference for a particular residue, arginine, asparagine, lysine, threonine, glutamate, and aspartate are commonly observed in the orientation protruding toward the substrate. The majority of these charged/polar residues are also positioned outwards, favourable to interact with the DNA. Besides these, both S1a and S1b occasionally show conserved or partially conserved phenylalanine, tyrosine, and infrequently a tryptophan that are likely to form π-π interaction with the base (See Supplementary Data S3).

1. **P-loop-kinase + Helical-(lid)-subdomain + alpha+beta-domain *(canonical DndF)***

P-loop-kinase	Helical-(lid)-subdomain	alpha+beta-domain
• Belongs to the KG division of P-loop-NTPase assemblage(Only in KG division, core strand 4 and 5 are located to the left side of core strand<br>• For domain architectures **1, 3** and **4**:<br>**Topology: str-1_hx-1_str-2_hx-2_str-3_hx-3_ins-Hx_Ins-S_str4**<br>• The topology and orientation of core strand-4 and strand-5 is very peculiar compared to other P-loop-NTPases.<br>• Core helix 3 leads to an insert region with a helix and strand, before leading to core strand 4.<br>• Core helix 4 and strand 4 are degenerated and the core P-loop fold is instead formed by 4 core strands and 1 insert strand, with the insert strand forming a hairpin with strand 4.<br>• The P-loop signature itself is typified by the conserved motif **'GxxGDGK'.** First G is usually followed by N/S/H (polar residues) & DGK is fully conserved across all versions of DndF.<br>• In the case of canonical DndF and HEPN+DndF, the P-loop signature is often preceded by a conserved C.<br>• The top of strand 2 always has a conserved unit **'(N)DATE/(N)DATH/DMSE/DLSE/DGSQ/DAST'**, where D is completely conserved.<br>• The *walker B (usually D/E)*, found on top of strand 3 is substituted by an **asparagine N,** followed by a conserved G & L **(motif 'NxGxL').**<br>• Due to the presence of N instead of D/E in walker B, and the distortion and partial degeneration of the core P-loop fold, this P-loop kinase is likely to be catalytically inactive, however it can still bind to nucleotides owing to the retention of a complete functional walker A motif.	• Domain length 190-230 residues, consisting of only helical bundles and no Beta-sheets.<br>• Structurally and topologically, helical domains of all DndF are similar with 12-13 helices. Together all the helical bundles form a globular structure.<br>• In some cases, it has 3 conserved Cysteine forming a zinc chelating motif **'CxxCxxxxxC',** with polar residues in between the conserved C. location of this stretch with conserved C is usually around the 2nd helix of helical domain, and it may span across the loop and helix.<br>• Following this cysteine stretch, a highly **conserved N** is present in the middle of the next helix across all helical domains found in all DndF.<br>• Following the conserved N, a highly conserved unit **'TxR/SxR'** is present in the junction between loop and helix (usually 4th or 5th helix of helical domain). R is usually located at the beginning of the helix with its orientation away from the core helical bundle.<br>• The orientation of the helical domain on top of iP-loop kinase and conserved residues N and R pointing towards the ATP binding region of iP-loop kinase, are all synapomorphies of lid-subdomain associated with P-loop kinases.<br>• The all alpha helical domain is thus identified as a highly derived version of the lid sub-domain, which instead of occurring in the form of an insert, is present in the form of a separate domain to the C-termini of P-loop kinase.	• Domain length 165-180 residues, consisting of a helical bundle (4-5 helices) and 8-9 beta-strands arranged in two different beta-sheet orientations around the helical bundle. The 1st beta-sheet has 5 strands and the 2nd one has 3 strands, all arranged in anti-parallel fashion in the beta-sheets.<br>• Topologically it begins with 2 long helices, followed by 4-5 strands (forming parts of both beta-sheets), two more helices and ends with 3 strands always in antiparallel orientation.<br>• The core topology of the beta-rich domain is maintained across all versions of DndF. It begins with two helices, followed by 2 antiparallel strands (part of 1st beta-sheet), then 3 more antiparallel strands (forming the 2nd beta-sheet), followed by two helices and finally 3 anti-parallel strands (part of 1st beta-sheet).<br>**Topology: H1-H2-S1↑-S2↓-S3↓-S4↑-S5↓-H3-H4-S6↑-S7↓-S8↑**<br>**Arrangement: S8↑-S7↓-S6↑-S2↓-S1↑-H4-H3-H2-H1-S3↓-S4↑-S5↓**<br>• H1-H2-S1↑-S2↓ and S4↓-S5↑-H3-H4 are structurally identical repeats composed of helical and β-hairpins that likely evolved via duplication.<br>• No residues are absolutely conserved across the alpha+beta domain of all DndF.<br>• The smaller 3 stranded beta sheet is stacked near the iP-loop kinase,<br>While the larger 5 stranded beta sheet is oriented away from the core structure, is too large to fit into the DNA groove, and likely interacts with invasive phage proteins.

2. **HEPN + P-loop-kinase + Helical-(lid)-subdomain + alpha+beta-domain**

P-loop-kinase	Helical-(lid)-subdomain	alpha+beta-domain
• Same features as the canonical version of DndF.	• 3 conserved Cysteine stretch **'CxxCxxxxxC',** is not found.<br>• A highly conserved K is present in the loop following the 4th helix, with its orientation away from the helical core bundle.<br>• Rest all features are the same in all versions of DndF.	• Top of helix-2 in HEPN version also has a highly conserved W *(somewhat conserved in canonical version of DndF)*, oriented towards the helical bundle. A highly conserved D in the beginning of helix-3 is seen in HEPN, DUF4338 and NERD (somewhat conserved) versions of DndF. Another conserved D in the middle of helix-3 is seen in the wHTH versions of DndF.

3. **NERD + Protein-kinase_1 + Protein-Kinase_2 + P-loop-kinase + Helical-(lid)-subdomain + alpha+beta-domain**

Protein-kinase_1	Protein-Kinase_2	P-loop-kinase	Helical-(lid)-subdomain	alpha+beta-domain
• Domain length 290-310 residues, consisting of 9 strands and 9-10 helices. Together they form a globular domain.<br>**Topology : S1↑-S2↓-S3↑-H1-S4↓-S5↑-H2-H3-S6-S7-S8-S9-H4-H5-helical_bundle**<br>• Belongs to serine threonine protein kinase (STPkinase) family of proteins usually associated with signaling.<br>• The topology and structure are aligning with the canonical versions of STPkinase.<br>• The glycine rich loop between S1-S2, used for ATP localization, is missing.<br>• The HRD motif of the catalytic loop and DFG motif of the Mg+ binding loop in S6 and S9 respectively are distorted.<br>• No conserved D in Mg+ binding loop.<br>• Lack of all necessary catalytic motifs and loop features makes this STPkinase functionally inactive.	• Domain length 270-300 residues.<br>• Belongs to the same family of STPkinase as the previous STPkinase domain.<br>• Glycine rich loop is conserved.<br>• Catalytic loop with HxD motif and Mg+ binding loop with DFx motif are both conserved followed by the activation loop.<br>• This is a functional copy of STPkinase domain which is preceded by a non-functional STPkinase, both belonging to the same family of kinases.	• Here, there is an additional insert strand after helix-1.<br>• **Topology: str-1_hx-1_ins-str1_str-2_hx-2_str-3_hx-3_*ins-Hx_Ins-str2_str4***<br>• Arrangement of core strands are in the form of **"S4↓-s5↑-s1↑-s3(N)↑-s2↑-InS↓".**<br>• This version of iP-loop-kinase is also found in the wHTH version of DndF.	• Same features as in other versions of DndF.	• A highly conserved D in the beginning of helix-3 is seen in HEPN, DUF4338 and NERD (somewhat conserved) versions of DndF.<br>• Rest all features are the same as in other versions of DndF.

4. **DUF4338(GNAT) + P-loop-kinase + Helical-(lid)-subdomain + alpha+beta-domain**

DUF4338(GNAT)	P-loop-kinase	Helical-(lid)-subdomain	alpha+beta-domain
• DUF4338 is GNAT acetyltransferase domain containing multiple helical bundles arranged in globular structure, and two different beta-strand orientations, one of them forming antiparallel beta-sheets inside the helical bundles and the other forms a beta-hairpin on the periphery of the globular structure.	• Same features as the canonical version of DndF.	• Same features as in other versions of DndF.	• Same features as in other versions of DndF.

5. wHTH + P-loop-kinase + Helical-(lid)-subdomain + alpha+beta-domain

wHTH	P-loop-kinase	Helical-(lid)-subdomain	alpha+beta-domain
• Domain length 150-170 residues.<br>• DALI search maps it to a so-called sulfur binding domain of DNA(PT) dependent restriction endonuclease protein ScoMcrA. The SBD is a modified wHTH domain It is postulated to recognize PT-modified DNA.<br>• Here, wHTH is fused to the C-terminal of DndF. In some cases along with the NERD and Pkinase domains.<br>• In the sequence level, H&K(or R) at the beginning of the 2nd helix are conserved, both oriented outside. Both these residues have shown to be involved in forming hydrophobic interaction with sulfur atoms of PT modified DNA.	• Same features as the NERD version of DndF with an insert strand present after helix-1.	• Same features as in other versions of DndF.	• Same features as in other versions of DndF.

6. wHTH + NERD + Protein-kinase_1 + Protein-Kinase_2 + P-loop-kinase + Helical-(lid)-subdomain + alpha+beta-domain

wHTH	Protein-kinase_1	Protein-Kinase_2	P-loop-kinase	Helical-(lid)-subdomain	alpha+beta-domain
• Same features as in the other wHTH version of DndF.	• Same features as in the other NERD version of DndF	• Same features as in the other NERD version of DndF	• Same features as the NERD version of DndF with an insert strand present after helix-1.	• Same features as in other versions of DndF.	• Same features as in other versions of DndF.

DndH

5. **iSNF2/Swi2 SF2-Helicase+HTH + Beta sandwich+Beta sandwich + TRD + iMTase + REase + HerA/FtsK ATPase**

iSNF2/Swi2 SF2-Helicase+HTH	Beta sandwich+Beta sandwich	TRD	iMTase	REase	HerA/FtsK ATPase
• Domain length 270-280 residues.<br>• The core topology is (S5↑-S1↑-S4↑-S3↑-is↓-S2↑) where the insert strand stacks antiparallel to the rest of the sheet and is positioned between core S2 and S3.<br>• DALI search using this domain has recovered multiple **ATP-dependent helicases** and **DNA repair protein** with Z scores above 6.<br>• The helicase domain of the DndH precisely maps to the so-called RecA2 (catalytically inactive C-terminal ATPase domain of SF2 helicases) domain and not the RecA1 domain (catalytically active N-terminal ATPase)<br>• Consists of two different sub-domains, one of them has 3 helices and 5 strands (P-loop domain), the other has 6-7 helices packed in a bundle. The C-terminal end of the helical bundle has an HTH unit associated with DNA binding.<br>• The P-loop catalytic residues are completely distorted and inactive. Both walker A and walker B motifs are distorted with no specific sequence signature.<br>• 3rd helix present of the P-loop domain has a highly conserved stretch 'LRN'. R is pointing away, forming an arginine finger.<br>• Additional conserved residues include D & W, in the helical-subdomain, both of which are packed against the longest helix of the helical bundle.	• Domain length 205-220 residues.<br>• This domain contains 16-17 strands spread across **4 distinct beta-sheet** orientations, arranged one after another.<br>• No conservation of residues in this domain.<br>• DALI search using this domain as seed recovered multiple glycoside hydrolase domains, like **Isopullulanase, Dextranase & Beta-galactosidase** with Z scores above 7.5. However, structurally it aligns with only the non-catalytic carbohydrate-binding module (CBMs), function is to bind to a range of polysaccharides, whose primary function is to increase the catalytic efficiency of carbohydrate-active enzymes.<br>• Belongs to β-sandwich structural family among CBM structures (most common type of CBM).<br>• Structurally it adopts an immunoglobulin fold and is widely observed in cell-adhesion proteins and some carbohydrate degrading enzymes.	• Domain length 220-240 residues.<br>• No significant hits in DALI search<br>• It is characterized by two distinct three-stranded and seven-stranded antiparallel sheets where the strands are ordered as S1↑-S2↓-S3↑ and S5↓-S4↑-S6↑-S7↓-S10↑-S9↓-S8↑. These antiparallel sheets are separated by helices on either side forming a globular α+β domain.<br>• 2nd beta-hairpin leads with additional helices, the loop connecting this hairpin to the helices has a highly conserved motif **'PxHP',** all of which are packed inside the helical bundles within the core structure.<br>• However, the **'PxHP'** motif is structurally too<br>• The 1st beta-hairpin forms a **3 stranded beta sheet** in the lower periphery, oriented towards the galactosidase domain, whereas the 2nd hairpin, 3rd hairpin and 4th hairpin together forms a **6-7 stranded beta-shee**t on the top peripheral region orienting towards the methyltransferase domain.<br>• The fast-evolving nature of the domain and lack of any conserved residues dispels any possibility of an enzymatic function and it is in complete agreement with the proposed role of a TRD as observed in Type II RM-systems.	• Domain length 270-280 residues.<br>• The MTases share a common core structure of a mixed seven-stranded β-sheet (6↓ 7↑ 5↓ 4↓ 1↓ 2↓ 3↓) referred to as an **'AdoMet-dependent MTase fold'.**<br>• Structurally the core superimposes with the MTase domain of Type IIG restriction endonuclease RM.BpuSI, which also has a REase domain fusion.<br>• All conserved residues of MTase domains are found within the core strands.<br>• Conserved N is found on top of the first strand, followed by a conserved motif 'HxxF', on strand 4. The Strand 4 conserved hydrophobic stretch is the motif IV of canonical AdoMet dependent MTase.<br>• Strikingly, we observe a total erosion of both the S-AdoMet binding Rossmann loop and [SDN]PP[FYW] motif after strand-4 and thus ruling out any possibility of being a catalytically active domain.<br>• Topologically the first 4 strands are parallel within the sheet, followed by an insert away from the helical core with 4 anti-parallel strands and a helix.<br>• There is a proper insert region of around 80-90 residues that forms a separate subdomain located after the core S4. This subdomain encodes a four-stranded antiparallel β-sheet ordered as S2↓-S3↑-S4↓-S1↑ and a helix perpendicular to the plane of the sheet connecting strands S3 and S4 which then leads to core H5 of the iMTase domain. This is exactly reminiscent of TRDs occurring as an insert within the MTases belonging to the γ-group.<br>• The N-terminal TRD and the TRD occurring as an insert sub-domain are placed together posterior to the MTase domain forming an interaction interface for target recognition.	• Domain length 250-280 residues.<br>• Begins with a helical bundle of 5-6 alpha helices, followed by core fold of PD-(D/E)xK nuclease superfamily.<br>• Comprising a mixed β-sheet core of 4 strands (S1↑-S2↓-S3↑-S4↑) with a β-hairpin and two parallel strands flanked by helices on both sides.<br>• There is a helical insert between S1 and S2, two additional helices after core H2 before connecting S4, and a terminal extension after S4 comprising a beta-hairpin (S5-S6) followed by two short helices. These terminal strands stack antiparallel to S4 forming a slightly-curved six-stranded mixed β-sheet enveloped by helices on either side.<br>• The proline at the beginning of S2 is distorted, while the aspartate is absolutely conserved, present in the 2nd core strand, followed by 'ExK' in the 3rd core strand.<br>• There is a short helical connector domain that connects the REase and the iMTase domain in DndH.<br>• All top hits of this domain in DALI searches have recovered restriction endonucleases from PD-(D/E)xK nuclease superfamily with Z scores of 4 and above.	• Domain length 380-400 residues.<br>• It retains the core P-loop fold and all the characteristics that typify the HerA-FtsK superfamily.<br>• Topologically it begins with N-terminal preceding strands forming a beta-hairpin, followed by strand-1, helix-1 and strand-2 of core P-loop fold.<br>• An N-terminal extension before the core ATPase domain is observed in the form of two preceding strands forming a β-hairpin.<br>• The extension leads to the core S1 and H1 comprising the P-loop/Walker-A signature typified as "GKSGQGKT" and a variation thereof.<br>• Other key signatures include DE as the Walker-B at the top of S3, R-finger at the bottom of H3, and Q as the sensor-1 at the top of S4.<br>• Then comes the HerA/FtsK superfamily characteristic insert in the form of an ***alpha-helical bundle along with 2 additional strands,*** one on each end of the alpha-helical bundle.<br>• The core H4 leads to S5 followed by a C-terminal extension comprising H-H-S-S where the two terminal strands form a β-hairpin that is stacked to the left of core S5.<br>• These features suggest that the C-terminal domain is canonical HerA-FtsK ATPase and not a RecA as previously thought and these are observed in Dali searches as well.

DndE Tree Raw file Newick Format

(DndE.1.1_WP_235598914.1:0.3393702669,((((DndE.1.1_WP_014260552.1:0.5704881531,DndE.1.1_TAN71076.1:0.4056966592)85.4/98:0.1107849320,(((DndE.1.1_WP_006996746.1:0.4678751607,DndE.1.1_MSP11311.1:0.2919260400)98.7/100:
0.4371471650,((((DndE.1.1_MBV8388978.1:0.4871454116,DndE.1.1_MBU1801746.1:0.4451165827)63.2/98:0.1496190348,(DndE.1.1_MBN2564060.1:0.4693732725,DndE.1.1_HFD31325.1:0.3306199273)99.4/100:0.4130513144)60.7/85:0.0889004
002,(((DndE.1.1_NWJ46935.1:0.4685661352,DndE.1.1_HAT45614.1:0.3292791742)85.4/100:0.1106558994,DndE.1.1_OPX62561.1:0.5459489202)78.9/96:0.0751007872,DndE.1.1_NLT05182.1:0.6405083662)57/93:0.1001859405)92.2/92:0.247275
2112,((((((DndE.1.1_WP_218075483.1:0.4006242242,(DndE.1.1_WP_090728895.1:0.2562270191,(((((((DndE.1.1_WP_230834816.1:0.2635214449,DndE.1.1_WP_198058112.1:0.2512210750)82.3/100:0.0839986972,DndE.1.1_WP_100732329.1:0.1
631381622)98.9/100:0.4345246517,DndE.1.1_WP_183315052.1:0.3603503934)99.2/100:0.5004693051,(DndE.1.1_WP_220497152.1:0.3672925514,DndE.1.1_WP_211030026.1:0.2475793290)93.2/100:0.2267534754)0/32:0.0087765791,(DndE.1.1
_WP_222169196.1:0.2127734475,DndE.1.1_WP_196092897.1:0.2162856500)85.3/100:0.0707616296)82.9/38:0.0363139347,(DndE.1.1_WP_212614735.1:0.2738671220,DndE.1.1_WP_198866830.1:0.2426842992)66.9/41:0.0244561167)90.4/74:0.
0711039210,(DndE.1.1_WP_219233673.1:0.2068004218,(DndE.1.1_WP_222488030.1:0.2357673243,DndE.1.1_WP_226343160.1:0.3375633475)42.3/74:0.0314846267)29.6/70:0.0293409543)89/83:0.0589284542)0/60:0.0491357897)8/45:0.065382
1639,DndE.1.1_WP_237029477.1:0.2048312492)71.4/90:0.0323508988,DndE.1.1_WP_226767951.1:0.5236101552)57.8/55:0.0576710590,DndE.1.1_WP_198825600.1:0.1261879666)37.4/54:0.0503030002,DndE.1.1_WP_218312573.1:0.27874149
24)100/100:1.1876390650,((DndE.1.1_WP_211802389.1:0.7290399997,((((((((((DndE.1.2_WP_099335653.1:0.1396114254,(DndE.1.2_MBE6050176.1:0.2412181003,DndE.1.2_NOW91083.1:0.1289374012)89.3/100:0.0807737370)93/100:0.0789950
224,(DndE.1.2_WP_079492855.1:0.1440687104,DndE.1.2_WP_221701309.1:0.1782496445)84.3/99:0.0578444767)83.5/99:0.0540069802,DndE.1.2_WP_055070046.1:0.1939783951)91.6/99:0.0885791500,DndE.1.2_WP_074366245.1:0.17161889
93)86.2/99:0.0800518752,DndE.1.2_WP_216554426.1:0.3340313441)44.7/94:0.0607510941,(DndE.1.2_WP_122638758.1:0.1275979606,DndE.1.2_WP_201416388.1:0.2225772448)93.5/100:0.1202284823)97.4/100:0.1511030825,(((DndE.1.2_WP_
009244795.1:0.1047230002,DndE.1.2_WP_204613511.1:0.2469759270)89.3/100:0.0964597440,DndE.1.2_WP_087065079.1:0.3136263886)59.2/100:0.0334659277,DndE.1.2_WP_087374096.1:0.2120062721)99.9/100:0.2729538634)6.8/59:0.0452
459215,((((DndE.1.2_WP_204721309.1:0.2023352196,DndE.1.2_WP_195608303.1:0.2850009309)59.8/89:0.0480542789,DndE.1.2_WP_087414570.1:0.1585561352)99.3/100:0.2068180805,((((DndE.1.2_HJD05337.1:0.2442380043,DndE.1.2_HHU
21212.1:0.2252661003)89.3/98:0.1164131805,(DndE.1.2_MBS6835850.1:0.1236115298,DndE.1.2_MBP1546090.1:0.1846739939)99.5/100:0.2427178787)56.2/71:0.0479297804,(DndE.1.2_MBR6642126.1:0.2649317769,DndE.1.2_MBQ7833838.1:0
.1884231787)99.6/100:0.2791044781)62.3/71:0.0524679961,DndE.1.2_MBD5632087.1:0.2794102272)79.8/96:0.0473562490)81.2/91:0.0607775885,(DndE.1.2_WP_054705684.1:0.2263058721,DndE.1.2_MBQ4122639.1:0.1868278484)96/100:0.12
05836715)96.8/93:0.1210741896)86.8/98:0.0726750798,((DndE.1.2_WP_161260522.1:0.2060333932,(DndE.1.2_WP_144351260.1:0.1452995275,DndE.1.2_WP_010249533.1:0.1548705587)91.8/100:0.0842079087)90.8/100:0.0740233372,DndE.1.
2_WP_163853503.1:0.0654601552)87/100:0.0830886612)96.8/98:0.2554621806,((((((DndE.1.3_FtsK_GGH72713.1:0.2586661825,(DndE.1.3_FtsK_NTW31743.1:0.1472333186,DndE.1.3_FtsK_OFY50564.1:0.2565564520)91.5/100:0.1466772547)1
7.1/67:0.0960211241,(DndE.1.3_FtsK_WP_209319825.1:0.3464297340,(DndE.1.3_FtsK_WP_111659537.1:0.3128305753,DndE.1.3_FtsK_WP_201633183.1:0.5444077214)99.6/100:0.4339072438)79.8/98:0.1084202834)96.3/100:0.2275782922,Dnd
E.1.3_FtsK_MBL7689990.1:0.5742985227)17.5/85:0.0429008838,DndE.1.3_FtsK_WP_131850661.1:0.5212470348)95.5/100:0.3504663571,((((((((DndE.1.3_FtsK_MBK9457847.1:0.3196504746,DndE.1.3_FtsK_WP_013766328.1:0.1959814916)96.3
/100:0.1359944584,(DndE.1.3_FtsK_WP_097126506.1:0.2672143319,(DndE.1.3_FtsK_WP_111672687.1:0.2035807670,DndE.1.3_FtsK_WP_135431160.1:0.2848248627)90.7/100:0.0773134576)88.6/87:0.0861544409)71.8/25:0.0170479497,DndE.1
.3_FtsK_WP_215222604.1:0.3495334832)80.2/25:0.0391010096,((DndE.1.3_FtsK_MBW1616874.1:0.3011061311,DndE.1.3_FtsK_MBN2890288.1:0.2058198778)93.7/100:0.1160258482,DndE.1.3_FtsK_WP_109745130.1:0.4927312254)72.8/46:0.0
309125850)65.3/72:0.0443320210,(DndE.1.3_FtsK_WP_046146159.1:0.1577821323,(DndE.1.3_FtsK_WP_019150954.1:0.2548814140,(DndE.1.3_FtsK_WP_207025792.1:0.1906868739,DndE.1.3_NCP91207.1:0.1543182562)81/100:0.0364895470)
78.9/98:0.0457750982)99.2/100:0.1682808293)87.3/71:0.0629270947,(DndE.1.3_FtsK_WP_238426328.1:0.5599391224,DndE.1.3_FtsK_MBN2881439.1:0.2231283150)78.7/83:0.0531306165)82.4/85:0.0530973925,((((((((DndE.1.3_WP_180565397.
1:0.2092428262,DndE.1.3_WP_163394764.1:0.1567568968)88.4/100:0.0703351144,(DndE.1.3_WP_136468584.1:0.1648124130,DndE.1.3_MBQ50903.1:0.3305019108)87.4/99:0.0809677173)70.5/98:0.0615783194,(DndE.1.3_WP_224469999.1:0.2
789248323,(DndE.1.3_WP_195870345.1:0.1592060191,(DndE.1.3_WP_133548185.1:0.2376913989,DndE.1.3_WP_027884015.1:0.2874377443)88.9/100:0.0936403630)80.8/97:0.0444228205)80.9/97:0.0712793906)22.2/59:0.0287712357,((DndE.1
.3_MBW8334337.1:0.2997832240,DndE.1.3_RYZ91410.1:0.3177485221)94.5/95:0.1112353127,(DndE.1.3_MBT3417079.1:0.2044595548,DndE.1.3_MBQ6878930.1:0.2000000047)77/88:0.0222362417)75.6/82:0.0421598784)89.1/90:0.0470872048,
DndE.1.3_MXV38347.1:0.2294306907)90.8/93:0.0588371287,(((DndE.1.3_WP_090886045.1:0.1999029059,DndE.1.3_WP_182952643.1:0.2845740582)72.8/81:0.0814505231,DndE.1.3_WP_172335592.1:0.1754386964)97.5/100:0.1727405458,Dnd
E.1.3_WP_090497049.1:0.2607184269)68/67:0.0571249616)73.1/56:0.0122950624,((DndE.1.3_WP_073193219.1:0.2089880158,DndE.1.3_WP_139853874.1:0.3594270544)46.4/96:0.1105263768,DndE.1.3_MAC40834.1:0.4496300840)80.8/84:0.07
27993588)88.2/68:0.0267545653,((((DndE.1.3_WP_079717017.1:0.1532398233,DndE.1.3_MBN9483656.1:0.2000871414)91.5/100:0.1020102094,DndE.1.3_MBW7841606.1:0.2708645517)91.7/100:0.0764954083,(((DndE.1.3_WP_188753156.1:0.1
350840846,DndE.1.3_WP_066751076.1:0.3103645388)92.9/100:0.1328149683,DndE.1.3_WP_020597295.1:0.2085006107)98.9/100:0.1817538226,DndE.1.3_WP_002704315.1:0.2755973075)49.1/91:0.0690441501)83.7/84:0.0432604107,DndE.1.3
_WP_069843542.1:0.3431786090)71.7/63:0.0283737693)96.5/96:0.1336488739)91.9/98:0.2264184561,DndE.1.3_FtsK_WP_166918550.1:0.4265615895)82.8/99:0.2088765944)99.6/97:0.6021881434,((((((((DndE.1.4_FtsK_KQQ17380.1:0.35235621
99,(((DndE.1.4_FtsK_MCA3513892.1:0.1842996558,DndE.1.4_FtsK_MBD9526219.1:0.1457019963)86.1/100:0.0515461918,DndE.1.4_FtsK_WP_096772301.1:0.2038046636)90.6/100:0.0921131217,(DndE.1.4_FtsK_WP_106159250.1:0.169135319
3,DndE.1.4_FtsK_MBC6405082.1:0.1966475126)86.4/100:0.0946303420)96.9/100:0.1481557664)83.7/100:0.0819074886,DndE.1.4_FtsK_WP_010666007.1:0.3213025186)91.7/100:0.1412185158,(((DndE.1.4_FtsK_MCC5062977.1:0.2425571091,D
ndE.1.4_FtsK_SDG06591.1:0.1543839300)44.8/100:0.0684359593,DndE.1.4_FtsK_WP_226614268.1:0.4617617082)33.7/100:0.0936189970,DndE.1.4_FtsK_WP_218287159.1:0.2882820086)97.9/100:0.2718982929)96/100:0.2073542849,DndE.1.4
_FtsK_MBP8808960.1:0.4481879203)27.2/88:0.0934816852,DndE.1.4_FtsK_NBO92769.1:0.6232746424)36.5/95:0.1231129611,(((DndE.1.4_FtsK_MBL7648115.1:0.5038657966,((DndE.1.4_FtsK_MBP7830370.1:0.3881990543,DndE.1.4_FtsK_MSP
34067.1:0.8767495950)83.2/98:0.1099850550,DndE.1.4_FtsK_WP_165226411.1:0.3123543576)76/97:0.1190493910)86/99:0.1682404643,((DndE.1.4_FtsK_VFK30085.1:0.2538496518,DndE.1.4_FtsK_WP_148665470.1:0.1892781630)98/100:0.257
5072436,DndE.1.4_FtsK_MBL8680161.1:0.3477165770)98.9/100:0.3530926729)96.6/100:0.2376284441,((((((DndE.1.4_FtsK_HJA75297.1:0.9676120193,DndE.1.4_FtsK_MBI4937545.1:0.1108339985)88.4/93:0.1912672810,DndE.1.4_FtsK_WP_107
692183.1:0.2791037563)81.1/89:0.0794117538,((DndE.1.4_FtsK_WP_081541824.1:0.2235597814,DndE.1.4_FtsK_WP_157469888.1:0.2217167652)96.9/100:0.1561293152,DndE.1.4_FtsK_WP_213378400.1:0.4210794382)61.6/92:0.0811394507)8
3.7/93:0.1590159204,((((DndE.1.4_FtsK_AMD60415.1:0.2090350821,DndE.1.4_FtsK_MCA0275568.1:0.1816591708)76.8/100:0.0597612509,(((DndE.1.4_FtsK_AVS61880.1:0.1553345157,(DndE.1.4_FtsK_WP_080426774.1:0.2165518316,DndE.1.4
_FtsK_WP_101604532.1:0.1481430266)29.7/80:0.0473498179)79.7/82:0.0338729911,DndE.1.4_FtsK_WP_161096816.1:0.1532305158)99.4/100:0.2042164867,DndE.1.4_FtsK_WP_190276359.1:0.3001711896)48.5/77:0.0562575636)79.2/93:0.081
4051428,DndE.1.4_FtsK_NCT05142.1:0.2005947397)75/94:0.0485172058,DndE.1.4_FtsK_WP_053064070.1:0.1119340197)100/100:0.3852316176)91.3/100:0.1604250035,DndE.1.4_FtsK_WP_079854660.1:0.6376152589)1.2/60:0.0250439458,(Dn
dE.1.4_FtsK_MBL8456255.1:0.2508567053,DndE.1.4_FtsK_WP_059758885.1:0.3817853495)93.6/100:0.1816960807)98.8/100:0.3229390550)72.4/98:0.0860090379)29.2/81:0.1068120202,(DndE.1.4_FtsK_WP_198669010.1:0.4470103993,(DndE.1.
4_FtsK_MYG28480.1:0.2300008897,DndE.1.4_FtsK_VFJ55938.1:0.3275040116)91.2/100:0.1442034368)88.3/100:0.1694123640)100/100:0.8153450625,((((((((DndE.1.5_FtsK_WP_080806708.1:0.2332443607,DndE.1.5_FtsK_WP_020589864.1:0.14
44522479)82.7/96:0.0667355400,(((((DndE.1.5_FtsK_WP_229535001.1:0.1898431122,DndE.1.5_FtsK_WP_151801637.1:0.1875538654)65.4/98:0.0835931848,DndE.1.5_FtsK_WP_105446286.1:0.1315223143)94.3/100:0.0948060631,DndE.1.5_Fts
K_MBF0595633.1:0.2802954876)88/97:0.0613457691,DndE.1.5_FtsK_WP_077997876.1:0.1551318210)78.1/90:0.0626956712,(((DndE.1.5_FtsK_MBZ4194742.1:0.2010118621,DndE.1.5_FtsK_QQS54212.1:0.0714553020)55/100:0.0351430643,Dn
dE.1.5_FtsK_HAS51515.1:0.1728838031)99.7/100:0.1973095549,DndE.1.5_FtsK_HGX94088.1:0.1372332076)98.5/100:0.1602944856)75.2/86:0.0242261068)60.2/84:0.0293963973,(((DndE.1.5_FtsK_WP_090332920.1:0.0850691876,DndE.1.5_Fts
K_WP_017842564.1:0.2262383750)32.3/98:0.0553523348,DndE.1.5_FtsK_MCB5279571.1:0.6021523997)38.7/82:0.0345584450,((DndE.1.5_FtsK_WP_199263773.1:0.1641272143,DndE.1.5_FtsK_MBF0234498.1:0.1845623676)92.1/99:0.06503664
27,DndE.1.5_FtsK_MBW6485178.1:0.1315562826)52.9/92:0.0262920607)94/89:0.0886410059)93/88:0.0905829780,(DndE.1.5_FtsK_MCC6933029.1:0.1631231575,DndE.1.5_FtsK_MBF0258980.1:0.0560217651)97.6/100:0.1357909561)23.5/79:0.0
250445966,((((DndE.1.5_FtsK_WP_204345547.1:0.1838658485,DndE.1.5_FtsK_MBR6091900.1:0.2526389447)37.5/12:0.0246989681,(DndE.1.5_FtsK_MCA0231876.1:0.1489676290,DndE.1.5_FtsK_MBK8956062.1:0.1347211900)87.8/100:0.04763
73341)19/13:0.0560538204,DndE.1.5_FtsK_WP_066618672.1:0.1136211738)88.9/78:0.0610821426,(DndE.1.5_FtsK_MBD3807686.1:0.2329663402,DndE.1.5_FtsK_MBD3339596.1:0.2974612520)68.4/84:0.0674136132)98.5/99:0.1869885075)90.2/
93:0.1652478899,DndE.1.5_FtsK_MCB1192146.1:0.4049530287)94.8/98:0.2958070650,DndE.1.5_FtsK_OQX00554.1:0.9725443474)53.9/95:0.1508274194,DndE.1.5_FtsK_OGX26180.1:0.7022697823)94.6/100:0.3538844209)34.1/78:0.0626898061
)83.3/90:0.1298788196)89.1/92:0.2524829205)83/73:0.2193842083,((((((((((DndE.1.6_FtsK_WP_118327606.1:0.1777943908,DndE.1.6_FtsK_HBH21138.1:0.4590920455)97.2/99:0.1465400385,DndE.1.6_FtsK_MBP5673068.1:0.2115108258)82.7/98:
0.0512862626,(DndE.1.6_FtsK_WP_074761590.1:0.3265752967,DndE.1.6_FtsK_WP_087340755.1:0.1189362635)89.6/100:0.0786748269)85.9/98:0.0487468930,DndE.1.6_FtsK_WP_122203035.1:0.3739001096)84.6/98:0.0370259269,(((DndE.1.6_
FtsK_WP_195643387.1:0.2656979524,DndE.1.6_FtsK_MBD5296228.1:0.5211959270)60.8/95:0.0794685427,DndE.1.6_FtsK_MBO5345802.1:0.2328509815)85.3/95:0.0769060924,DndE.1.6_FtsK_MBP8934701.1:0.2531675941)20.4/69:0.03663424
47)90.5/89:0.1016502366,DndE.1.6_FtsK_MBR6103723.1:0.5227402756)96.6/99:0.1580598004,((((DndE.1.6_FtsK_WP_094162584.1:0.2145655295,(DndE.1.6_FtsK_WP_159154836.1:0.2590365195,DndE.1.6_FtsK_TXF75218.1:0.2136514661)99.
5/100:0.3301716383)98.4/100:0.2303779951,(DndE.1.6_FtsK_WP_193700739.1:0.3030087500,(DndE.1.6_FtsK_WP_224116960.1:0.1167244775,DndE.1.6_FtsK_NCA75040.1:0.2411351372)96/100:0.1271643124)37.9/99:0.0609343183)81.2/99:0.0
827114437,DndE.1.6_FtsK_MCC5946645.1:0.4217820745)73.2/97:0.0331535487,((DndE.1.6_FtsK_WP_153081150.1:0.4347967519,(DndE.1.6_FtsK_MBP5344730.1:0.4495810587,DndE.1.6_FtsK_MBO4451331.1:0.2648650134)87.3/100:0.126790
2811)89.3/100:0.1613041761,DndE.1.6_FtsK_MBQ9218150.1:0.5661584125)98.9/100:0.3095604575)90.8/50:0.0931213931)90.4/46:0.0908537060,(((DndE.1.6_FtsK_WP_089334215.1:0.6590385262,(DndE.1.6_FtsK_RYF90450.1:0.3590062157,Dn
dE.1.6_FtsK_RMG69578.1:0.2387935550)93.3/100:0.1198618776)34.3/99:0.0566781651,DndE.1.6_FtsK_WP_114069642.1:0.3988309302)68.5/98:0.0740328813,DndE.1.6_FtsK_MBL7990034.1:0.6303458955)82.7/98:0.1198494257)93.2/46:0.1968
332418,(DndE.1.6_FtsK_WP_188465132.1:0.2726056387,DndE.1.6_FtsK_MCB9191509.1:0.2683328709)0/67:0.0000029185)99.6/98:1.2874866860,(((((((((((((DndE.2.1_MBQ6138826.1:0.4458438233,DndE.2.1_MBE6511569.1:0.6714569549)19.6/8
5:0.1243396855,DndE.2.1_WP_066973323.1:0.1950616840)99.2/100:0.6024236525,((DndE.2.1_WP_142535463.1:0.3454531139,DndE.2.1_QRN84660.1:0.5664049353)91.2/100:0.4315070689,(DndE.2.1_MBR6047688.1:1.0547850952,DndE.2.1_
MBQ9573120.1:0.792556941 9)81.9/96:0.3515544237)76.8/85:0.1488758602)88.4/85:0.2611902223,DndE.2.1_MCD5397039.1:0.5283966154)69/83:0.0491467711,((((DndE.2.1_WP_206707597.1:0.1954176044,DndE.2.1_WP_096711958.1:0.445164
4314)70.8/100:0.1170021304,DndE.2.1_MCD4670738.1:0.3884067800)87.7/100:0.1316255159,DndE.2.1_WP_214166865.1:0.6227211982)89.1/98:0.1524767546,(DndE.2.1_MBI4555839.1:0.8459279209,(DndE.2.1_MBI5906863.1:1.2880542836,D
ndE.2.1_WP_009886029.1:0.5451637821)85.5/99:0.2884101101)85.1/96:0.1554851646)81.5/92:0.1060409916)95.3/98:0.2380122556,((DndE.2.1_MBI4650348.1:1.2680811589,DndE.2.1_WP_244706079.1:0.7991155662)44.7/77:0.1172462647,Dnd
E.2.1_MBD3322373.1:0.6163188405)55.2/72:0.0830370839)52.8/77:0.1953674795,(((DndE.2.1_MCA0457125.1:0.2436202744,(DndE.2.1_WP_110886491.1:0.5072100922,DndE.2.1_MBI1293683.1:0.2780462998)57.4/99:0.1636665307)80.3/99:0.1
383131993,DndE.2.1_MBI3825589.1:0.4462295191)90.8/99:0.2586875305,DndE.2.1_MCC6604720.1:0.4853916983)79.3/90:0.1231386021)28.6/88:0.0628369511,DndE.2.1_MBN1459270.1:1.5088673489)81.9/95:0.1508913222,((((DndE.2.1_WP_2
27601515.1:0.4141393837,DndE.2.1_WP_214763740.1:0.9104237999)87.4/99:0.2499513231,(((DndE.2.1_WP_152671169.1:0.8304455767,DndE.2.1_WP_156942631.1:1.0487504406)20.2/54:0.0656527275,DndE.2.1_MBK7932013.1:0.382916072
2)98.8/100:0.5811421734,DndE.2.1_MBW2977912.1:0.7222725620)50.6/87:0.0731746329)84.2/80:0.1126150904,((DndE.2.1_HHZ19159.1:0.7385033247,DndE.2.1_MBD3724023.1:1.0913158987)6.2/44:0.0667276241,(DndE.2.1_OJU18682.1:0.358
0006568,DndE.2.1_MBZ4662717.1:0.5777253615)99.9/100:0.7020212350)40.4/76:0.0676892503)88.9/95:0.2107747640,(DndE.2.1_WP_143583447.1:0.6868030676,(((((DndE.2.1_WP_059001189.1:0.5212503810,DndE.2.1_WP_207002076.1:0.501
9495594)14.2/95:0.0541888340,DndE.2.1_MBD1850959.1:0.2696330309)84.6/99:0.1065953105,(DndE.2.1_WP_106303670.1:0.1325483798,DndE.2.1_MBD2395724.1:0.4311700015)93/100:0.1290996087)25.8/84:0.0360454801,DndE.2.1_MCA660
3891.1:0.2155286410)97/99:0.2790608817,(DndE.2.1_WP_233656933.1:0.6881619849,DndE.2.1_WP_189326207.1:0.6477892716)85.9/99:0.2638056597)93.1/100:0.1889314108)83.2/100:0.0873425232)92.3/99:0.2547525162)91.7/98:0.32248640
77,((((((((DndE.2.2_WP_186394059.1:0.1342820161,DndE.2.2_MYF40691.1:0.2369306019)79.8/99:0.0727682094,DndE.2.2_MYB19207.1:0.2266946597)90.6/100:0.0883592552,DndE.2.2_SFU78155.1:0.2858931540)19.9/88:0.0191415043,(DndE.
2.2_MCB9957175.1:0.0850461109,DndE.2.2_RKU15339.1:0.3135706247)100/100:0.2523650149)98/94:0.1785575185,(DndE.2.2_WP_174163579.1:0.2222057327,DndE.2.2_WP_127281910.1:0.1429515841)100/100:0.3615340271)2.7/46:0.037011
0040,((DndE.2.2_WP_149801938.1:0.2211595196,(DndE.2.2_WP_160731419.1:0.2323693129,DndE.2.2_WP_041795176.1:0.3585674161)35/89:0.0584860601)96.6/100:0.1930211461,DndE.2.2_WP_223031698.1:0.3375055056)98/100:0.2346579
823)88.2/99:0.1315063752,((((((DndE.2.2_WP_039601359.1:0.1968004432,DndE.2.2_WP_006954029.1:0.4351262850)85.1/93:0.1040173887,(DndE.2.2_WP_166323694.1:0.5141206924,DndE.2.2_WP_144079977.1:0.1443016847)85.8/99:0.10516
87202)60.9/60:0.0894922983,DndE.2.2_WP_067084515.1:0.3248344299)7/41:0.0739883506,(DndE.2.2_WP_174482018.1:0.3263259926,DndE.2.2_WP_207250209.1:0.3558806983)31.6/85:0.1061838089)99.2/100:0.1927215284,((DndE.2.2_WP_1
93235735.1:0.2646534077,DndE.2.2_WP_237127990.1:0.1290510352)98.5/100:0.1952518376,DndE.2.2_WP_065678374.1:0.2689207168)44.6/96:0.0977517819)0/76:0.0458239815,DndE.2.2_WP_156049205.1:0.3248049081)99.9/100:0.40545887
10)91/100:0.1454850526,(((DndE.2.2_WP_052606673.1:0.3213936069,DndE.2.2_MCB1002901.1:0.1678949198)89.8/100:0.1196677406,DndE.2.2_MXZ76732.1:0.3839147605)99.6/100:0.3879052105,(DndE.2.2_MBI2830758.1:0.2536401532,DndE
.2.2_MBA4157576.1:0.3361947358)98.3/100:0.2904495952)88.5/100:0.1769978725)70.5/97:0.0754578239,(((((DndE.2.2_WP_018584454.1:0.2981948739,DndE.2.2_WP_225050998.1:0.3477755544)88.7/100:0.0853420722,DndE.2.2_GHJ45929.1:
0.1932334619)88.7/100:0.1455463926,(DndE.2.2_MCC7074554.1:0.2532595215,DndE.2.2_MXW55607.1:0.3864236446)41.4/87:0.1036310240)78.7/98:0.2427859939,DndE.2.2_MXV88013.1:0.5071634484)98.3/100:0.4159528507,DndE.2.2_WP_1
45305599.1:0.7218082751)59.9/83:0.1319203393)98.4/100:0.4898019288)89.2/94:0.3611666066,((((DndE.2.3_WP_090802715.1:0.4765141979,((((DndE.2.3_WP_073374244.1:0.3306667658,DndE.2.3_MBY6273347.1:0.2647911330)96.3/100:0.214
9181219,DndE.2.3_WP_139559691.1:0.5363234222)0/44:0.0281873721,DndE.2.3_MBC8522949.1:0.9658299794)85.4/77:0.1189605087,DndE.2.3_WP_036478246.1:0.5660493019)37.4/77:0.1115787531)90.8/75:0.1309854301,((((DndE.2.3_WP_1
66937041.1:0.3883701533,DndE.2.3_TIP92990.1:0.2323895211)94.9/100:0.1778510059,((DndE.2.3_WP_127080516.1:0.3837914767,DndE.2.3_EAZ89973.1:0.4176150314)71.1/100:0.0991308603,(DndE.2.3_HIE73037.1:0.5180972590,(DndE.2.3_
VTU38528.1:0.3794738067,DndE.2.3_MBD8566227.1:0.7583527444)96.2/100:0.3517452900)96.7/98:0.2792296380)67.6/91:0.1098034779)60/90:0.0758979876,DndE.2.3_WP_126378523.1:0.4986348399)77.8/88:0.0807367454,DndE.2.3_MBV944
9969.1:0.7701245191)42.2/72:0.0371672537)85.2/64:0.1086969736,((((((DndE.2.3_WP_007705401.1:0.6548909097,(DndE.2.3_WP_049729875.1:0.3175893815,DndE.2.3_MBS6812590.1:0.3900117342)89.4/100:0.1514972874)96.5/100:0.2604497
735,(DndE.2.3_QCC51440.1:0.2975426498,DndE.2.3_TKX87921.1:0.3749890301)100/100:0.4916774888)74.7/71:0.0583428366,(DndE.2.3_WP_188649937.1:0.3395116286,DndE.2.3_WP_095130676.1:0.3262735186)96.6/100:0.2623136067)84.6/
71:0.1054731435,(((DndE.2.3_WP_218274404.1:0.3876016870,DndE.2.3_WP_075632284.1:0.5257691076)90.1/100:0.1643911515,(DndE.2.3_WP_134643199.1:0.4632925270,DndE.2.3_WP_022730130.1:0.3536276924)98.3/100:0.2762370458)83
.2/100:0.1023258859,DndE.2.3_WP_035534525.1:0.7688278463)90.3/99:0.1710837773)84.2/80:0.1157508317,DndE.2.3_RMG31910.1:1.0134256948)86.7/72:0.1434237588,((DndE.2.3_MBT3993578.1:0.5577658191,DndE.2.3_MBT7556385.1:0.65
76404735)99.5/100:0.6476402633,((DndE.2.3_RZJ90683.1:0.6000441678,DndE.2.3_MBL6758235.1:1.2602338420)56.2/90:0.1433910800,DndE.2.3_MBE6844498.1:0.8097416349)47.3/68:0.0383194325)60.2/61:0.0536378023)83.1/76:0.135728282
0)97.9/98:0.5225826191,(((((DndE.2.5_MBO6251934.1:0.7509701478,((((DndE.2.5_MBF1060580.1:0.5593201215,DndE.2.5_WP_234155971.1:0.2393865631)89.3/100:0.1215635224,DndE.2.5_WP_041385767.1:0.2985581020)58.1/85:0.061250948
2,DndE.2.5_WP_044110554.1:0.3501019157)83.7/85:0.0660243437,DndE.2.5_WP_088161329.1:0.2668250640)93/99:0.1865826947)95.1/100:0.1881852496,(((DndE.2.5_MBT6170167.1:0.5037495910,DndE.2.5_PKP20205.1:0.4660760317)80.5/89
:0.1136090460,((DndE.2.5_HAQ71015.1:0.7952014685,DndE.2.5_CAH1001257.1:0.4189203055)80.8/95:0.0828010513,DndE.2.5_WP_109218016.1:0.4762454945)64.6/90:0.0558700503)19.6/84:0.0929379460,(DndE.2.5_WP_239776829.1:0.43511
29456,DndE.2.5_MBK7058597.1:0.2987737797)77.9/95:0.0959259225)89.3/94:0.0945861861)29.6/63:0.0648087647,DndE.2.5_QZE15893.1:0.5188222239)98.2/100:0.3003961069,((DndE.2.5_MBN2746816.1:0.5980922052,((DndE.2.5_WP_119755
246.1:0.4151648993,(DndE.2.5_WP_119654942.1:0.5605850358,DndE.2.5_WP_208309274.1:0.4176009468)99/100:0.3426008376)12.9/50:0.0649341060,DndE.2.5_MCB0522398.1:0.2408279582)92.6/99:0.1661581308)90.4/99:0.1404807672,((((D
ndE.2.5_WP_151704761.1:0.2742147312,DndE.2.5_WP_229587697.1:0.3734543994)27.3/100:0.0681403933,DndE.2.5_MBT6045225.1:0.4740189434)65.1/100:0.1179098926,DndE.2.5_WP_012516635.1:0.3565486048)99.5/100:0.4094633521,(((
DndE.2.5_WP_148372252.1:0.4739798109,(DndE.2.5_MBR5356808.1:0.4974119622,(DndE.2.5_MBR0432124.1:0.4852627814,DndE.2.5_RGY00475.1:0.2344745236)51.6/99:0.1000818003)78.9/100:0.1561073362)97.9/100:0.3134149039,((DndE.2
.5_WP_091894293.1:0.3082857350,DndE.2.5_WP_062124577.1:0.4514090820)92.7/100:0.1639968989,DndE.2.5_MBT3206777.1:0.2423762372)89.1/100:0.1624976842)27.5/82:0.0842645790,DndE.2.5_WP_193421898.1:0.4666569841)96.6/100:
0.2513526734)84.9/96:0.0855408959)83.2/95:0.0983393336)66.9/96:0.1246685745,DndE.2.5_WP_147168475.1:0.6343109389)99.7/100:0.7003187158)75.5/35:0.1778042553)63/38:0.2288695780,(((((((((DndE.2.4_MBZ4646663.1:0.3871599438,(D
ndE.2.4_WP_195966414.1:0.3036540027,DndE.2.4_MBS6820672.1:0.4931430587)91.5/100:0.1219612252)72/93:0.1074726751,DndE.2.4_MBR2247210.1:0.3711278506)96.2/100:0.1429346631,(((DndE.2.4_HAZ96918.1:0.3842962199,DndE.2.4_
WP_074932244.1:0.2568657278)99.3/100:0.2457848027,(DndE.2.4_MBP3238898.1:0.4425711295,(DndE.2.4_WP_170185947.1:0.4430766129,DndE.2.4_WP_118158237.1:0.6139054025)71.3/87:0.1173823641)83.8/79:0.0526419091)63.6/76:0.020
6545818,((DndE.2.4_WP_011016399.1:0.5744020728,DndE.2.4_MBR6122294.1:0.3428765231)89.7/97:0.1279954550,DndE.2.4_WP_204689650.1:0.4418321908)78.1/76:0.0494029168)86.9/95:0.0577004172)92.6/99:0.1054549113,(DndE.2.4_WP
_078810249.1:0.2814033360,DndE.2.4_HHV84031.1:0.4228726107)98.3/100:0.2245494607)9.1/89:0.0190504902,DndE.2.4_WP_027445293.1:0.5095519369)81/79:0.0592532257,(((DndE.2.4_WP_038705120.1:0.3409814077,DndE.2.4_WP_03481
6421.1:0.3551304852)82.1/67:0.1185056492,((DndE.2.4_WP_113712600.1:0.3301442243,DndE.2.4_WP_236337125.1:0.4062563783)100/100:0.8738400945,DndE.2.4_MBR3313092.1:0.6007077322)91.7/87:0.2202221945)0/31:0.0000029515,((Dn
dE.2.4_WP_015327755.1:0.3163665288,(DndE.2.4_WP_089865329.1:0.3507531690,(DndE.2.4_WP_195467955.1:0.4546734831,DndE.2.4_WP_079442200.1:0.5524194554)77/96:0.0780114859)82.7/96:0.0919087114)94.5/100:0.3115731980,(Dnd
E.2.4_WP_006785107.1:0.4233558504,DndE.2.4_MBN1038819.1:0.2970205975)100/100:0.8208460068)97.4/99:0.3524697230)92.3/86:0.1154574376)0/55:0.0017491273,((DndE.2.4_WP_117969894.1:0.3268164618,DndE.2.4_WP_118745901.1:0.
4469835428)96.3/100:0.2777999708,DndE.2.4_WP_215679263.1:0.4599181993)99.9/100:0.6039941802)95/72:0.1388036304,((((((DndE.2.4_MBQ3444572.1:0.3860878099,DndE.2.4_WP_117734350.1:0.2978744066)42/37:0.0512394230,(DndE.2.4
_MBS5035654.1:0.5642970390,DndE.2.4_WP_226260860.1:0.3261704916)69.7/76:0.0819430603)76/30:0.0338020151,(DndE.2.4_WP_058963327.1:0.8025231963,(DndE.2.4_WP_118589748.1:0.4849180096,DndE.2.4_HJF40857.1:0.5659509048)
41.9/93:0.0488794643)93.1/82:0.1216070583)43.8/83:0.0480617849,DndE.2.4_WP_020375125.1:0.3773488212)58.2/97:0.0666839672,DndE.2.4_WP_132017608.1:0.5751600602)85.7/87:0.0958133060,DndE.2.4_MCF2657509.1:0.4811491560)82.
1/82:0.0627544130)66.1/74:0.0225545149,(DndE.2.4_WP_235070418.1:0.5135914332,(DndE.2.4_WP_204921094.1:0.9937256299,DndE.2.4_WP_091234204.1:0.6819861088)85.4/90:0.1152252012)43.8/70:0.0133490325)98.6/99:1.0593307630)87
.1/90:0.5134939527,((((DndE.2.6_WP_141826947.1:0.7394498598,(DndE.2.6_WP_118558305.1:0.2217251214,DndE.2.6_WP_207110709.1:0.5417787118)95.3/100:0.3738869293)95.6/100:0.4137296034,((DndE.2.6_WP_152185678.1:0.549473185
3,DndE.2.6_MXX25657.1:0.4500920771)94.9/100:0.3245358462,DndE.2.6_WP_109802013.1:0.6140987835)88.7/100:0.1980766877)90.3/85:0.1921588203,((((((DndE.2.6_WP_130424768.1:0.5201351198,(DndE.2.6_MCF8118359.1:0.4245020432,
DndE.2.6_MBE0254148.1:0.3642380196)60.6/100:0.1139555447)72/100:0.1003268312,DndE.2.6_TWW08874.1:0.4599430616)87.5/100:0.1230282199,(DndE.2.6_MBK9322424.1:0.5065367147,DndE.2.6_NLK72606.1:0.7484828561)73.4/97:0.1768
774796)52.9/97:0.0412017376,DndE.2.6_MYG79463.1:0.4260245460)76.3/99:0.0950813927,((DndE.2.6_WP_172109841.1:0.2838568202,DndE.2.6_WP_122103173.1:0.5836868395)83.2/100:0.1524435607,DndE.2.6_MBM3275872.1:0.338168498
2)96.1/100:0.2715814205)62.7/96:0.1417291506,(DndE.2.6_WP_027120480.1:0.4541654004,DndE.2.6_NLG31071.1:0.8106303737)99.7/100:1.0384198540)69.3/89:0.0987424997)74.9/71:0.0990511091,((((((((DndE.2.6_MCB9980244.1:0.32950638
98,DndE.2.6_WP_182780679.1:0.9962266237)88.8/99:0.1956102268,((DndE.2.6_VDS03879.1:0.3271244039,DndE.2.6_WP_222602988.1:0.3499247080)93.5/96:0.1598250942,DndE.2.6_WP_207353167.1:0.2757370186)14.7/26:0.0194102655)90.
7/90:0.1392192138,DndE.2.6_WP_116330199.1:0.2973518796)87.9/56:0.1282972628,(((DndE.2.6_WP_194015773.1:0.0321831195,DndE.2.6_WP_124143423.1:0.6751222314)100/100:1.3571800290,DndE.2.6_QWD11446.1:0.5895410093)39/67:0.
1670292434,(DndE.2.6_WP_047858966.1:0.4134905692,DndE.2.6_NMC57799.1:0.4838840231)100/100:1.0531169170)73.8/61:0.1572403431)64.6/22:0.0797779566,(DndE.2.6_WP_143473963.1:0.6274142806,DndE.2.6_MBX3713751.1:0.558099
0503)74.3/27:0.0960864034)81/28:0.0678746354,DndE.2.6_RYZ85163.1:0.5737657640)72.3/89:0.2286943585,DndE.2.6_EAV47471.1:0.8750900642)87.1/91:0.2063449856,(DndE.2.6_MBV9857922.1:0.8417286920,(DndE.2.6_WP_123296453.1:0.
6520728060,DndE.2.6_MBU2601348.1:0.6354362580)6.7/51:0.0967550136)78.3/71:0.0852380803)74.6/61:0.1237661430)97.8/98:1.3131659480)85.2/78:0.5195580736)88.3/63:0.3693944345)79.9/45:0.1799837111)58.4/30:0.0771763657)93.5/91:0.
2853630967)98.3/100:0.3334295787,(DndE.1.1_MBR3379967.1:0.4504238914,DndE.1.1_EGC74035.1:0.3910665322)70.2/97:0.0839903272)12.6/52:0.0987747679)76.4/75:0.0594876737,(((DndE.1.1_MCA9450138.1:0.6002891650,DndE.1.1_MBA
0328751.1:0.6242244508)53.5/76:0.0693796701,((DndE.1.1_MBE9043442.1:0.9193717072,DndE.1.1_BAZ86693.1:0.2670679057)84.9/83:0.1169932411,((DndE.1.1_ABQ26654.1:0.4050626038,DndE.1.1_WP_225071831.1:0.3884880682)92.2/100:
0.2028916461,DndE.1.1_PWU19854.1:0.2482769346)99.4/100:0.3589134327)52.2/79:0.0440940510)80.3/68:0.0437639642,DndE.1.1_MAS93104.1:0.3711499877)82.5/80:0.0570576093)90.7/94:0.1066238875,(DndE.1.1_MYI07895.1:0.3899785459
,DndE.1.1_ACO32041.1:0.4160023216)85.6/99:0.0947168301)59.8/96:0.1017100868,DndE.1.1_OAD20904.1:0.3677345112);

DndD/CxC ABC Tree Raw file Newick Format

(1.1.assoc.DndD_OPX62560.1:0.6091719503,((((((((1.1.assoc.DndD_ACO32718.1:1.0551510546,1.1.assoc.DndD_MYI07896.1:0.9180439913)95/93:0.2177400150,1.1.assoc.DndD_WP_034819312.1:1.0021785344)58.1/28:0.0233654674,(((1.1.ass
oc.DndD_WP_006996745.1:0.4913319325,1.1.assoc.DndD_MSP11310.1:0.5666208617)100/100:0.6561562144,1.1.assoc.DndD_TAN71075.1:0.8528586265)89.9/81:0.1421763073,(((1.1.assoc.DndD_WP_225071830.1:0.1524523501,1.1.assoc.Dnd
D_ABQ26653.1:0.1385908210)100/100:0.3125058332,1.1.assoc.DndD_PWU19853.1:0.3979991450)100/100:0.2967111026,1.1.assoc.DndD_MAS93103.1:0.6100970698)97/100:0.1473402290)83.3/27:0.0561384770)63.8/68:0.0470489584,1.1.assoc
.DndD_WP_014260551.1:0.8756039870)92.6/97:0.0971241549,1.1.assoc.DndD_BAZ86695.1:0.9352493448)100/100:0.2673717647,((((((((1.2.assoc.DndD_MBR6642127.1:0.7569799101,1.2.assoc.DndD_MBQ4122640.1:0.4700655201)98.7/100:0.1
666832297,(1.2.assoc.DndD_WP_087414569.1:0.5014491974,(1.2.assoc.DndD_WP_195608302.1:0.5183311828,1.2.assoc.DndD_WP_204721311.1:0.4331626395)62.8/99:0.1165607002)100/100:0.3977518838)25.7/94:0.0431417162,(1.2.assoc.Dn
dD_HJD05338.1:0.8473452839,1.2.assoc.DndD_MBS6835849.1:0.5794794101)99.9/100:0.3313098384)100/100:0.3772607018,((1.2.assoc.DndD_WP_010249534.1:0.3042072107,1.2.assoc.DndD_WP_170233299.1:0.3203047982)100/100:0.23268
30329,(1.2.assoc.DndD_WP_161260521.1:0.3588607180,1.2.assoc.DndD_WP_163853504.1:0.3024433437)100/100:0.3721413810)100/100:0.2279130363)98.5/100:0.1455221554,((1.2.assoc.DndD_WP_009244796.1:0.7701178200,(1.2.assoc.Dnd
D_WP_087065078.1:0.5820919352,1.2.assoc.DndD_WP_162608173.1:0.4428141601)99.7/100:0.2883232919)94.1/100:0.1342117511,1.2.assoc.DndD_WP_204613509.1:0.5845921656)99.5/100:0.2012096360)87.4/97:0.0603023514,((((((1.2.assoc.
DndD_NOW91084.1:0.1466339027,1.2.assoc.DndD_MBE6050177.1:0.1828772753)86.8/100:0.0582249501,1.2.assoc.DndD_WP_099335652.1:0.2113217891)100/100:0.1381381164,(1.2.assoc.DndD_WP_079492854.1:0.3149019449,1.2.assoc.Dnd
D_WP_221701308.1:0.2032098569)100/100:0.1311422204)97.9/100:0.0862865754,1.2.assoc.DndD_WP_055070045.1:0.3398575664)97.6/100:0.1020770916,1.2.assoc.DndD_WP_074366246.1:0.4380661370)100/100:0.1731636865,((1.2.assoc.Dn
dD_WP_122638759.1:0.3170494335,1.2.assoc.DndD_WP_201416387.1:0.4078577670)99.6/100:0.1616037525,1.2.assoc.DndD_WP_216554428.1:0.7792937293)28.6/93:0.0716572624)100/100:0.2651989690)100/100:0.7535092249,(((((((1.3.assoc
.DndD_GGH72708.1:0.4125987997,((1.3.assoc.DndD_OFY50565.1:0.1956751967,1.3.assoc.DndD_NTW31742.1:0.2297181545)100/100:0.2065686464,((1.3.assoc.DndD_WP_127343892.1:0.1941563928,1.3.assoc.DndD_WP_111659536.1:0.21570
27532)77.8/100:0.0691073749,1.3.assoc.DndD_WP_201633186.1:0.5332868179)100/100:0.1910618504)17.7/73:0.0991244510)100/100:0.2787136940,1.3.assoc.DndD_WP_131850662.1:0.6021664627)83.2/100:0.1212908967,1.3.assoc.DndD_MB
L7689989.1:0.6304221751)99.3/100:0.2654926021,(((((((((1.3.assoc.DndD_WP_066751078.1:0.5725210015,1.3.assoc.DndD_WP_188753157.1:0.4286699968)97.1/100:0.1035505713,1.3.assoc.DndD_WP_020597294.1:0.3636789539)99.7/100:0.17
11103584,1.3.assoc.DndD_MAC40833.1:0.7034906581)66.4/100:0.1007876496,1.3.assoc.DndD_WP_002704313.1:0.5055935003)91.2/100:0.0882721121,((((((((1.3.assoc.DndD_WP_172335593.1:0.3113142474,1.3.assoc.DndD_WP_090886043.1:0
.2826368776)91.7/100:0.0784306462,1.3.assoc.DndD_WP_090497051.1:0.2907231486)98.9/100:0.1185556401,1.3.assoc.DndD_WP_182952642.1:0.4816710313)98.7/100:0.1185679676,1.3.assoc.DndD_WP_069843541.1:0.3684366135)99.5/100:
0.1487837294,1.3.assoc.DndD_RYZ91409.1:0.5030517391)30.1/73:0.0483570097,((1.3.assoc.DndD_MBN9483655.1:0.3616225875,1.3.assoc.DndD_WP_079717016.1:0.3278816202)62.3/97:0.0698599879,1.3.assoc.DndD_MBW7841605.1:0.5234
387612)57.1/74:0.0642900001)93.7/100:0.0550418115,(1.3.assoc.DndD_MBQ6878929.1:0.4907275917,1.3.assoc.DndD_MBW8334338.1:0.5788045344)98.8/100:0.1296090860)96.7/100:0.0668614801,(((((1.3.assoc.DndD_WP_073193218.1:0.3408
721805,((1.3.assoc.DndD_MBQ50902.1:0.3649417246,1.3.assoc.DndD_WP_133548184.1:0.2749457412)97.9/100:0.0746532342,1.3.assoc.DndD_WP_051191192.1:0.2771477784)86.6/100:0.0319180145)8.2/64:0.0233446400,1.3.assoc.DndD_WP
_136468585.1:0.4212854978)49.8/68:0.0362051633,(1.3.assoc.DndD_WP_195870344.1:0.2520960509,(1.3.assoc.DndD_WP_163394765.1:0.1474382591,1.3.assoc.DndD_WP_180565396.1:0.3327638705)99.9/100:0.1166215157)92.4/100:0.06216
45617)95.7/99:0.0605058027,(1.3.assoc.DndD_WP_224470000.1:0.3206608041,1.3.assoc.DndD_WP_139853873.1:0.3595411356)100/100:0.1459153973)77.4/99:0.0606834956,1.3.assoc.DndD_MXV38346.1:0.5337448670)100/100:0.3201962786)
98/100:0.0866801302)99.8/100:0.2089465977,1.3.assoc.DndD_WP_013766332.1:0.5797076624)90.4/79:0.0658607111,((1.3.assoc.DndD_WP_046146160.1:0.3252734753,1.3.assoc.DndD_WP_207025789.1:0.3891831297)99.9/100:0.1957022701,(
((1.3.assoc.DndD_WP_109745131.1:0.3864561757,1.3.assoc.DndD_WP_135431162.1:0.3723325368)99.4/100:0.1356578843,(1.3.assoc.DndD_WP_111672686.1:0.2652863943,1.3.assoc.DndD_WP_203084770.1:0.6203040824)37.8/97:0.05356394
58)91.8/98:0.0655755301,1.3.assoc.DndD_WP_215222603.1:0.4621690869)94/98:0.0579256951)93.3/98:0.0633189353)53.4/76:0.0951597314,1.3.assoc.DndD_MBW1616871.1:0.6180173829)79.9/82:0.1142087126,1.3.assoc.DndD_WP_16691854
9.1:0.7548434938)99.9/100:0.3453192805)100/100:0.6178549204,(((((((((1.6.assoc.DndD_HBH21139.1:0.2366945101,1.6.assoc.DndD_WP_118327605.1:0.2236291782)99.7/100:0.0827023451,(1.6.assoc.DndD_WP_074761591.1:0.2740126786,1.6
.assoc.DndD_WP_087340754.1:0.1648457971)98.3/96:0.0789626304)17.9/85:0.0365216630,(1.6.assoc.DndD_MBD5296227.1:0.1407091120,1.6.assoc.DndD_WP_005845249.1:0.1148003390)100/100:0.2006398773)76.5/76:0.0382512230,((1.6.as
soc.DndD_MBP5673069.1:0.2194035706,1.6.assoc.DndD_WP_259001665.1:0.2315712199)66.7/99:0.0501150771,1.6.assoc.DndD_MBP8934702.1:0.2032832610)99.3/100:0.0858486452)74.8/72:0.0270732697,1.6.assoc.DndD_MBO5345803.1:0.2
597065374)83.1/83:0.0792056581,1.6.assoc.DndD_MBR6103724.1:0.3983966631)100/98:0.2554346555,(((1.6.assoc.DndD_MBL7990033.1:0.5876663981,1.6.assoc.DndD_WP_089334216.1:0.3499739003)97.2/100:0.1016765887,(((((1.6.assoc.Dn
dD_MCB9191508.1:0.2301198141,1.6.assoc.DndD_WP_188465130.1:0.2208930131)100/100:0.1673091060,(((1.6.assoc.DndD_NCA75041.1:0.1441387445,1.6.assoc.DndD_WP_239763286.1:0.2668768498)54.4/100:0.0343536305,1.6.assoc.DndD
_WP_224116959.1:0.1031923990)100/100:0.1109175031,1.6.assoc.DndD_RYF90449.1:0.2558510201)81.3/88:0.0312841135)3/44:0.0250813541,1.6.assoc.DndD_WP_114069643.1:0.2534078813)64.2/74:0.0468936093,1.6.assoc.DndD_RMG69579
.1:0.2605608712)91.9/100:0.0629913632,1.6.assoc.DndD_MCC5946644.1:0.5700682286)18/71:0.0338764614)94.6/89:0.0704994210,((1.6.assoc.DndD_TXF74969.1:0.2297687255,1.6.assoc.DndD_WP_159154837.1:0.1722954480)100/100:0.23844
90247,1.6.assoc.DndD_WP_094162585.1:0.3211884907)100/100:0.3079883394)95.6/100:0.0938129839)99.8/78:0.1815570237,1.6.assoc.DndD_MBQ9218151.1:0.6179616352)22.8/30:0.0331731407,(1.6.assoc.DndD_MBP5344731.1:0.3205542359
,1.6.assoc.DndD_WP_153073761.1:0.2686887544)99.7/100:0.2475016893)100/100:1.6052291180)83.5/97:0.1816698506,(((((((((2.1.assoc.CxC_MBQ9573119.1:0.7267025836,(2.1.assoc.CxC_MBR6047689.1:1.1656635947,(2.1.assoc.CxC_QRN84
661.1:0.3815687382,2.1.assoc.CxC_WP_142535464.1:0.4337101481)100/100:0.4364431643)79.6/100:0.1277510444)99.9/100:0.3152473099,((2.1.assoc.CxC_WP_096711959.1:0.4675201199,(2.1.assoc.CxC_MCD4670737.1:0.8196442811,2.1.ass
oc.CxC_WP_206707598.1:0.4929448382)34.3/92:0.0563812821)97.8/99:0.1315872441,2.1.assoc.CxC_WP_214166864.1:0.8421753391)95.1/100:0.1326222443)100/100:0.3597956045,(((2.1.assoc.CxC_MBQ6138825.1:0.4285080466,2.1.assoc.Cx
C_MBE6511568.1:0.4115117921)100/100:0.7279724190,2.1.assoc.CxC_WP_009886028.1:1.0975380800)85.9/98:0.2087860137,2.1.assoc.CxC_MCD5397040.1:0.7042600933)81.5/53:0.0616926256)93.6/55:0.1095652060,(2.1.assoc.CxC_MBI4555
838.1:1.0829507978,2.1.assoc.CxC_WP_244706077.1:1.0123536876)83/47:0.1533250567)50/48:0.1714127373,2.1.assoc.CxC_MBI5906862.1:1.6620105687)98.5/99:0.2499850253,(((2.1.assoc.CxC_MBI3825590.1:0.4815824306,((2.1.assoc.CxC_
MCA0457124.1:0.2841007766,2.1.assoc.CxC_MBI1293682.1:0.2773977360)100/100:0.2234393585,2.1.assoc.CxC_MCC6604719.1:0.3766987606)98.3/100:0.1275421313)95/100:0.1308447907,2.1.assoc.CxC_WP_170130974.1:0.7784140929)99.4
/100:0.2257889508,2.1.assoc.CxC_MBN1459271.1:1.2828023658)95.9/99:0.1848198669)19.2/25:0.0437644737,(((((2.1.assoc.CxC_MBD3724022.1:1.3222525113,2.1.assoc.CxC_WP_185962559.1:0.9301039998)96.5/100:0.2478519906,(2.1.assoc.
CxC_HHZ19158.1:0.7994662727,(2.1.assoc.CxC_WP_214763741.1:1.0553803496,2.1.assoc.CxC_WP_227601516.1:0.5428973742)38.8/94:0.1073990686)97.9/100:0.1825242555)69.2/99:0.1142178700,(((2.1.assoc.CxC_MBD1850958.1:0.3170408
735,2.1.assoc.CxC_WP_059001192.1:0.3994974680)98.8/100:0.2398293149,2.1.assoc.CxC_WP_207002074.1:0.7461519514)99.9/100:0.3198997502,(2.1.assoc.CxC_WP_189326206.1:0.8002311722,2.1.assoc.CxC_WP_233656932.1:0.88637429
06)98.1/100:0.1949271713)99.2/100:0.1843405444)77.4/98:0.0998497668,(2.1.assoc.CxC_MBZ4662716.1:0.4296914562,2.1.assoc.CxC_OJU18683.1:0.5415981748)100/100:0.6785136397)98.6/100:0.1739146165,(((((((((2.4.assoc.CxC_HAZ96919.
1:0.3778183061,2.4.assoc.CxC_WP_074932242.1:0.3617772447)100/100:0.2886493399,((2.4.assoc.CxC_MBP3238899.1:0.4310777802,2.4.assoc.CxC_MBR6122295.1:0.5931869731)98.5/100:0.1444895845,2.4.assoc.CxC_WP_118158238.1:0.65
75550329)34/98:0.0614195964)89.3/100:0.0833369433,((2.4.assoc.CxC_MBR2247211.1:0.7243859580,2.4.assoc.CxC_WP_011016398.1:0.9925472236)86/94:0.1674499889,(2.4.assoc.CxC_MBS6820671.1:1.0057008863,(2.4.assoc.CxC_MBZ4646
662.1:0.4150095935,2.4.assoc.CxC_WP_195966416.1:0.6146076517)98.5/100:0.1704367724)59.2/97:0.0919247532)18.3/72:0.0647206275)92.4/99:0.0911859640,2.4.assoc.CxC_WP_170185948.1:0.7527292986)100/100:0.2328204477,((((2.4.asso
c.CxC_HHV84032.1:0.5053991482,2.4.assoc.CxC_WP_078810248.1:0.3739797502)94.5/100:0.0919170439,2.4.assoc.CxC_WP_027445292.1:0.6157148057)99.9/100:0.1723760922,(((2.4.assoc.CxC_HJF40858.1:0.4199522362,2.4.assoc.CxC_WP
_132017606.1:0.3571157826)100/100:0.2853635475,2.4.assoc.CxC_WP_091234202.1:0.8047554417)96.7/100:0.1283129552,(((((2.4.assoc.CxC_MBQ3444571.1:0.4582501100,((2.4.assoc.CxC_MBS5035653.1:0.6269234871,2.4.assoc.CxC_WP_1
17734349.1:0.5190211286)48.5/100:0.0746338888,2.4.assoc.CxC_WP_204921095.1:0.7438581332)91/99:0.0625402116)78.6/100:0.0427606989,(2.4.assoc.CxC_MCF2657510.1:0.3825449987,2.4.assoc.CxC_WP_186840529.1:0.5438345326)99.4/
100:0.1433644771)88.2/99:0.0512180135,2.4.assoc.CxC_WP_058963328.1:0.5279989993)98.8/100:0.0746081501,((2.4.assoc.CxC_WP_034816418.1:0.5413846221,2.4.assoc.CxC_WP_038705121.1:0.3490661581)100/100:0.3087766837,(2.4.ass
oc.CxC_WP_226260859.1:0.3286924046,2.4.assoc.CxC_WP_235070417.1:0.7008040743)69.4/88:0.0567471420)22.7/80:0.0283051456)99.2/100:0.1069552698,2.4.assoc.CxC_WP_020375126.1:0.6097002758)12.6/83:0.0590887439)96.8/100:0.09
14947128)99.1/100:0.1359121024,(2.4.assoc.CxC_WP_055172271.1:0.7303733719,(2.4.assoc.CxC_WP_117969892.1:0.4952952109,2.4.assoc.CxC_WP_118745900.1:0.4677476475)100/100:0.3857768472)100/100:0.6158972590)47.7/91:0.06230
08593)87.9/97:0.0912241695,(2.4.assoc.CxC_WP_181465871.1:0.3304659685,2.4.assoc.CxC_WP_236337127.1:0.3657722586)100/100:0.9612203636)98.5/100:0.2012924377,((2.4.assoc.CxC_WP_015327756.1:0.2654580231,2.4.assoc.CxC_WP
_216381428.1:0.4950867665)98.5/100:0.1338939446,(2.4.assoc.CxC_WP_079442201.1:0.4728504858,2.4.assoc.CxC_WP_195467954.1:0.4011076726)99.7/100:0.1766614551)100/100:0.2874285855)24.1/90:0.1144679713,(2.4.assoc.CxC_MBN10
38820.1:0.4391800478,2.4.assoc.CxC_WP_009607082.1:0.4802150847)100/100:0.7741392134)99.4/100:0.3019898601,((((((2.5.assoc.CxC_CAH1001256.1:0.6652069365,2.5.assoc.CxC_MBK7058596.1:0.7808331836)89.6/100:0.1472333026,2.5.a
ssoc.CxC_MBT6170168.1:0.7919124615)51.5/97:0.1016205993,(2.5.assoc.CxC_HAQ71016.1:1.1659658861,2.5.assoc.CxC_WP_239776827.1:0.8331895294)59.6/98:0.1120619806)62.4/96:0.0916273739,2.5.assoc.CxC_WP_109218015.1:0.97503
35283)88.1/99:0.1417955520,((((2.5.assoc.CxC_MBF1060579.1:1.0719911956,2.5.assoc.CxC_WP_013064822.1:0.5167713807)51.3/97:0.1593528392,2.5.assoc.CxC_WP_234155972.1:0.4454093105)95/99:0.1589441782,(2.5.assoc.CxC_WP_027
449530.1:0.6676923062,2.5.assoc.CxC_WP_088161330.1:0.4046227829)92.6/100:0.1097925656)96/100:0.1983954490,2.5.assoc.CxC_MBO6251935.1:1.2158547753)96.2/100:0.2598467186)99.9/100:0.6081020787,((((2.5.assoc.CxC_MBN274681
7.1:0.6291550202,2.5.assoc.CxC_MCB0522397.1:0.4868328898)99.5/100:0.2258787634,((2.5.assoc.CxC_WP_012516636.1:0.5284594992,(2.5.assoc.CxC_MBT6045224.1:0.6137378504,2.5.assoc.CxC_WP_168927508.1:0.5252944169)14.6/63:0.0
702021185)18.1/64:0.0693086343,2.5.assoc.CxC_WP_229587695.1:0.4706637828)100/100:0.3294589976)94.3/99:0.1438872006,(2.5.assoc.CxC_WP_119654943.1:0.6830796190,2.5.assoc.CxC_WP_147168476.1:1.0214744763)99.8/100:0.38089
82097)69.7/97:0.1945170287,((2.5.assoc.CxC_MBR0432125.1:1.0412086992,2.5.assoc.CxC_WP_148372253.1:0.5173631172)99.5/100:0.3085262796,2.5.assoc.CxC_WP_091894291.1:0.6203435244)100/100:0.6051406295)23.6/89:0.2267077796)
100/100:1.0873560170)90.5/54:0.1564568635)86/52:0.1244548059)99.4/58:0.2383551787,((((((((((2.6.assoc.CxC_MYG79462.1:0.5574024783,(2.6.assoc.CxC_MCF8118358.1:0.3877000801,2.6.assoc.CxC_WP_130424767.1:0.3996961582)98.8/100
:0.1507231317)78.3/100:0.1114982444,(2.6.assoc.CxC_WP_122103172.1:0.4070621897,2.6.assoc.CxC_WP_172109842.1:0.4243697602)100/100:0.3910519077)91.5/100:0.0821395162,2.6.assoc.CxC_MBK9322423.1:0.9983203362)87.8/100:0.09
43965431,((2.6.assoc.CxC_WP_118558304.1:0.5562326446,2.6.assoc.CxC_WP_141826948.1:0.8867097141)94.6/100:0.1929147915,2.6.assoc.CxC_NLK72607.1:0.7759761546)99.6/95:0.2684484525)77.1/89:0.0948353791,(2.6.assoc.CxC_WP_1
52185679.1:0.9530876357,2.6.assoc.CxC_WP_109802012.1:0.7593465779)76.5/93:0.1795375504)29.1/72:0.0922403900,2.6.assoc.CxC_MXX25656.1:1.1267542315)96.1/95:0.1689297334,(2.6.assoc.CxC_WP_027120481.1:0.7545292049,2.6.ass
oc.CxC_NLG31070.1:0.7122796007)100/100:1.1398703550)34.6/72:0.0753653552,((2.6.assoc.CxC_MBV9857921.1:1.2761962318,(2.6.assoc.CxC_MBU2601349.1:0.8519092860,2.6.assoc.CxC_WP_123296452.1:0.7119646361)94.5/100:0.237382
4957)97.9/100:0.2251751090,(((((((2.6.assoc.CxC_VDS03880.1:0.2383999180,2.6.assoc.CxC_WP_222602989.1:0.4095713602)96.2/100:0.0925440537,2.6.assoc.CxC_WP_207353168.1:0.2872199073)99.7/100:0.1530748820,(2.6.assoc.CxC_WP_
100173261.1:0.4594059002,(2.6.assoc.CxC_QWD11445.1:0.8481809686,2.6.assoc.CxC_WP_116330198.1:0.2758668023)91.2/57:0.0692894594)96.5/57:0.0892898454)99.6/86:0.1570665106,2.6.assoc.CxC_MBX3713752.1:0.7439430677)95.1/86:
0.1311374910,2.6.assoc.CxC_MCB9980243.1:0.4158385021)0/10:0.0978980080,2.6.assoc.CxC_WP_182780678.1:0.5698558373)98.7/100:0.1861675790,2.6.assoc.CxC_EAV47472.1:1.3498014533)93.6/100:0.1489906102)89.7/100:0.1097275405)
98.7/100:0.2107682848,2.6.assoc.CxC_WP_047858967.1:1.2199687166)99.9/100:0.3877939462,2.6.assoc.CxC_WP_194015771.1:1.6647842276)94.7/100:0.1971235213)91.9/63:0.1129076317,(((((2.2.assoc.CxC_GHJ45930.1:0.3551130638,2.2.as
soc.CxC_WP_225050999.1:0.3809282584)99.1/100:0.1587889026,2.2.assoc.CxC_WP_242796394.1:0.4559613770)99.1/100:0.1988845128,2.2.assoc.CxC_MXV88012.1:0.5659053470)99.9/100:0.3600904560,((((((((2.2.assoc.CxC_WP_186394058.
1:0.1585355384,2.2.assoc.CxC_MYB19208.1:0.3417815219)94/100:0.0636538515,(2.2.assoc.CxC_RKU15340.1:0.4985349533,2.2.assoc.CxC_MCB9957176.1:0.1118234283)98.3/100:0.0970110493)38.2/99:0.0589145191,2.2.assoc.CxC_SFU7816
5.1:0.3671460698)100/100:0.1710222507,((((2.2.assoc.CxC_WP_149801937.1:0.3507088456,2.2.assoc.CxC_WP_014415363.1:0.2796013536)97.3/100:0.1326341263,2.2.assoc.CxC_WP_160731418.1:0.3483132444)100/100:0.2984510256,2.2.ass
oc.CxC_WP_255587140.1:0.3729003661)99.9/100:0.1745433881,(2.2.assoc.CxC_WP_174163581.1:0.2180050303,2.2.assoc.CxC_WP_127281909.1:0.2433560578)100/100:0.3201116779)34.9/96:0.0682229333)93.5/99:0.1006338539,(2.2.assoc.C
xC_MBI2830759.1:0.4122487766,2.2.assoc.CxC_MBA4157577.1:0.5546772885)100/100:0.3821459052)91/50:0.0927718945,(2.2.assoc.CxC_WP_052606674.1:0.4514183025,2.2.assoc.CxC_MXZ76733.1:0.5430007907)100/100:0.5566838789)40.2
/49:0.0912466416,((((2.2.assoc.CxC_WP_174482019.1:0.2723128467,2.2.assoc.CxC_WP_207250208.1:0.3472884143)89/100:0.0647666106,2.2.assoc.CxC_WP_082679537.1:0.3354853397)99.4/100:0.1211470316,(2.2.assoc.CxC_WP_00695403
1.1:0.3648678150,2.2.assoc.CxC_WP_159453275.1:0.3398688334)99.9/100:0.1670949501)96.7/100:0.1057841284,(2.2.assoc.CxC_WP_156049207.1:0.5196808313,(2.2.assoc.CxC_WP_065678373.1:0.4712603485,(2.2.assoc.CxC_WP_19323573
6.1:0.3720867393,2.2.assoc.CxC_WP_237127991.1:0.2733454081)100/100:0.3843904038)96.5/100:0.1379737918)95.5/100:0.1145849876)100/100:0.3771012090)99.7/100:0.2731675595,2.2.assoc.CxC_WP_145305600.1:0.6922725032)89.2/100:0
.1740156417)100/100:0.7211990075,(((2.2.assoc.CxC_WP_143037134.1:0.6777329719,((2.3.assoc.CxC_MBT7556386.1:1.5359040005,(((2.3.assoc.CxC_HIE73036.1:0.7580593539,(2.3.assoc.CxC_WP_126378521.1:0.4971461882,2.3.assoc.CxC_
WP_127080515.1:0.4960939553)58.4/100:0.0941349335)92.1/100:0.1321481786,2.3.assoc.CxC_WP_166937042.1:0.5811493785)92.7/100:0.1160681237,(2.3.assoc.CxC_MBD8566226.1:0.6680439760,2.3.assoc.CxC_VTU38524.1:0.6797779292)8
8.2/100:0.1200240942)98.2/100:0.1677635102)78.3/55:0.1065207343,((((2.3.assoc.CxC_MBY6273346.1:0.4191920133,2.3.assoc.CxC_WP_073374246.1:0.4975679335)97.5/100:0.1392613683,2.3.assoc.CxC_RZJ90682.1:0.7396755162)27.8/79:0.
0540819628,2.3.assoc.CxC_WP_139559690.1:0.5797044858)99.6/100:0.1876323227,2.3.assoc.CxC_WP_036478245.1:0.6734072445)91.8/58:0.0862185245)47/55:0.0905815959)99.2/91:0.2601996267,(((2.3.assoc.CxC_MBE6844499.1:0.8334512
777,(2.3.assoc.CxC_MBS6812589.1:0.4195541381,(2.3.assoc.CxC_WP_007705399.1:0.6577765774,2.3.assoc.CxC_WP_049729874.1:0.4260162530)81.2/100:0.0899340565)98.7/100:0.2081899437)99/100:0.2093904426,(2.3.assoc.CxC_WP_095
130677.1:0.4527539583,2.3.assoc.CxC_WP_188649935.1:0.5261606017)97/100:0.1325450099)98.8/100:0.1474828498,((2.3.assoc.CxC_QCC51441.1:0.3459880213,2.3.assoc.CxC_TKX87922.1:0.4784723916)100/100:0.5317426805,((2.3.assoc.C
xC_WP_022730131.1:0.5023458592,2.3.assoc.CxC_WP_134643200.1:1.0259646615)98.1/100:0.2459247158,(2.3.assoc.CxC_WP_035534523.1:0.7200295370,2.3.assoc.CxC_WP_075632283.1:0.4496606639)87.8/100:0.1110913813)100/100:0.29
77135411)77.6/99:0.0844722655)96/100:0.1793458774)93.6/98:0.4126191581,2.3.assoc.CxC_RMG31909.1:1.5079268312)100/100:1.2589598030)85.5/95:0.1772830725)100/100:0.4426686955)40.6/97:0.0626368082,((((((_1.4.assoc.DndDAMD604
16.1:0.1943551606,_1.4.assoc.DndDMCA0275567.1:0.2152974966)97.8/100:0.0634963087,(((_1.4.assoc.DndDAVS61881.1:0.1740020941,((_1.4.assoc.DndDWP_071769100.1:0.1489092308,_1.4.assoc.DndDWP_157935657.1:0.1099538210)96.3/
100:0.0579676982,_1.4.assoc.DndDWP_161096815.1:0.0970390435)58.1/99:0.0478364105)100/100:0.1832142406,(_1.4.assoc.DndDNCT05143.1:0.1774816759,_1.4.assoc.DndDWP_047895325.1:0.1769995091)96/100:0.0575519824)89.7/100:0.0
503488971,_1.4.assoc.DndDWP_062254505.1:0.3232493169)48.2/98:0.0252868678)100/100:0.2841725880,((_1.4.assoc.DndDWP_084312306.1:0.2814397723,_1.4.assoc.DndDWP_081541825.1:0.2357576643)100/100:0.1862479173,(_1.4.assoc.
DndDMBI4937544.1:0.2964207118,_1.4.assoc.DndDWP_107692184.1:0.3721660758)93.8/100:0.0932575456)97.4/99:0.1289988773)52.6/90:0.0878104116,_1.4.assoc.DndDWP_079854659.1:0.7074155949)69.7/92:0.1499609285,(_1.4.assoc.DndD
MBL8456254.1:0.2401712748,_1.4.assoc.DndDWP_059758888.1:0.3035466048)100/100:0.6004377011)99.5/100:0.2427405453,(((((_1.4.assoc.DndDMSP34068.1:0.6760198901,_1.4.assoc.DndDWP_165226410.1:0.3342344019)96.2/100:0.151506
1238,_1.4.assoc.DndDMBP7830371.1:0.4457263619)81.5/100:0.0853336893,_1.4.assoc.DndDMBL7648116.1:0.4495197631)100/100:0.6708489222,(_1.4.assoc.DndDMBL8680162.1:0.4772147542,(_1.4.assoc.DndDWP_096461608.1:0.358846247
0,_1.4.assoc.DndDVFK30086.1:0.2980713202)99.9/100:0.2266176051)99.4/100:0.2937102933)100/100:0.5514709143,((((((((_1.4.assoc.DndDWP_106159249.1:0.1339679932,_1.4.assoc.DndDMBC6405083.1:0.2056635206)98.9/100:0.1087565394
,(_1.4.assoc.DndDMBD9526218.1:0.1061481832,_1.4.assoc.DndDMCA3513891.1:0.1286520412)100/100:0.1668629726)100/100:0.1994281604,_1.4.assoc.DndDKQQ17381.1:0.3589225908)99.1/100:0.1248370835,_1.4.assoc.DndDWP_01066600
8.1:0.5028703230)96.5/98:0.0802132505,((_1.4.assoc.DndDSDG06623.1:0.1918437432,_1.4.assoc.DndDMCC5062976.1:0.2248488907)100/100:0.2050642233,_1.4.assoc.DndDWP_226614267.1:0.3814656138)100/100:0.3075681643)11.2/89:0.05
59427039,(_1.4.assoc.DndDVFJ55931.1:0.2313354750,_1.4.assoc.DndDMYG28479.1:0.2298691464)100/100:0.5578942031)99.7/100:0.1704302327,_1.4.assoc.DndDMBP8808961.1:0.7239374247)64.2/96:0.0696155546,_1.4.assoc.DndDNBO9276
8.1:0.6536008446)90/100:0.1369555142)92.9/99:0.1840727733)100/100:0.6922129615)44.8/66:0.0980530344)92.9/100:0.1239382749,(((((((1.5.assoc.DndD_WP_105446285.1:0.0979875214,1.5.assoc.DndD_WP_229535000.1:0.1232946648)69.7/1
00:0.0158157285,1.5.assoc.DndD_WP_151801636.1:0.1161124010)99.6/100:0.0505269926,1.5.assoc.DndD_WP_020589863.1:0.1828940698)84.9/89:0.0273315733,((((1.5.assoc.DndD_WP_199263772.1:0.0550578988,1.5.assoc.DndD_MBF02344
99.1:0.2177192866)97.8/100:0.0372387534,(1.5.assoc.DndD_MBW6485177.1:0.1020791015,1.5.assoc.DndD_WP_090332918.1:0.0952163803)94.5/100:0.0243723592)100/100:0.0720762287,((1.5.assoc.DndD_MCC6933030.1:0.1076452604,1.5.as
soc.DndD_MBF0258981.1:0.1544644915)98.5/100:0.0753869733,1.5.assoc.DndD_MCB5279572.1:0.4115527780)65.1/16:0.0412463033)75.5/15:0.0284904036,(1.5.assoc.DndD_WP_017842563.1:0.1505816399,1.5.assoc.DndD_MBF0595632.1:0.1
712087261)61/93:0.0330059184)1.7/5:0.0084723730)99.6/20:0.0653422501,(1.5.assoc.DndD_MBZ4194743.1:0.0827274602,1.5.assoc.DndD_QQS54211.1:0.0535201841)100/100:0.2329030192)82.5/98:0.0481450296,(((((1.5.assoc.DndD_MBK895
6063.1:0.0745651010,1.5.assoc.DndD_MCA0231877.1:0.0593912607)95/100:0.0305956719,1.5.assoc.DndD_WP_204345546.1:0.1305106616)78.5/100:0.0236428781,1.5.assoc.DndD_WP_066618669.1:0.0935883245)94.7/100:0.0531533998,1.5.a
ssoc.DndD_MBR6091901.1:0.2320183823)97.9/100:0.0690608754,(1.5.assoc.DndD_MBD3339595.1:0.2171914043,1.5.assoc.DndD_MBD3807687.1:0.2781424920)69/100:0.0530059595)99.5/100:0.1137309975)99.8/100:0.3412045612,1.5.assoc.D
ndD_OGX26181.1:0.6722008837)100/100:0.6161317618)99.1/100:0.2157937653)93.9/98:0.1015614390,(1.1.assoc.DndD_NLT05183.1:1.0313437301,((((1.1.assoc.DndD_WP_218312572.1:0.3819768577,1.1.assoc.DndD_WP_233076199.1:0.27120
14601)95.2/100:0.1218273359,1.1.assoc.DndD_WP_226767950.1:0.3667116506)100/100:0.2782009744,((((((1.1.assoc.DndD_WP_222488031.1:0.1933122131,1.1.assoc.DndD_WP_237029476.1:0.1927668842)100/100:0.1939174180,1.1.assoc.Dn
dD_WP_219233672.1:0.3409883819)77.3/99:0.0709987916,1.1.assoc.DndD_WP_090728894.1:0.3622598617)13.1/53:0.0510696616,(((1.1.assoc.DndD_WP_261313030.1:0.0704085468,1.1.assoc.DndD_WP_227592512.1:0.0484159201)100/100:0.
2365368607,(1.1.assoc.DndD_WP_198866829.1:0.2030308472,1.1.assoc.DndD_WP_212614736.1:0.3753454806)30.4/91:0.0586775129)92.5/100:0.0736638768,1.1.assoc.DndD_WP_218075484.1:0.7829570377)76.9/53:0.1088016858)98.1/53:0.0
920531548,1.1.assoc.DndD_WP_226069790.1:0.4882181748)46/93:0.0901150047,(1.1.assoc.DndD_WP_182784004.1:0.4281078511,1.1.assoc.DndD_WP_211030024.1:0.4427075602)100/100:0.3282483873)59.2/96:0.1255262896)99/99:0.178051
9598,((1.1.assoc.DndD_WP_183315053.1:0.1358860926,1.1.assoc.DndD_WP_232341132.1:0.1124435451)26.3/39:0.0426227656,(1.1.assoc.DndD_WP_230834817.1:0.0492130647,1.1.assoc.DndD_WP_100732330.1:0.0717434838)96.7/100:0.085
8421353)100/100:0.6407005234)100/100:0.3277659615)67.2/72:0.1189485114)90.4/73:0.0987865880,1.1.assoc.DndD_NWJ46934.1:0.8610495113)58.4/97:0.0939233071,(1.1.assoc.DndD_MBN2564059.1:0.5172434912,1.1.assoc.DndD_HFD31324
.1:0.5879281658)100/100:0.3340286048)78.2/99:0.1155226762,1.1.assoc.DndD_MBV8388977.1:1.4411787298);

HerA/FtsK Capture Systems Tree Raw file Newick format

(DndH.CTD.clade.1_WP_098578207.1:0.5605939609,(DndH.CTD.clade.1_WP_126989801.1:0.3131603403,(DndH.CTD.clade.1_WP_038189768.1:0.1998477989,DndH.CTD.clade.1_EAZ83602.1:0.2041621733)99.6/100:0.1777436861)99.1/100:0.1535652994,(((DndH.CTD.clade.1_WP_16
6739478.1:0.3469348432,DndH.CTD.clade.1_WP_194561844.1:0.2516538656)99.8/100:0.2344435228,(DndH.CTD.clade.1_TCI37639.1:0.3386128020,(DndH.CTD.clade.1_NTY08282.1:0.2650632097,(DndH.CTD.clade.1_RKQ29339.1:0.0837043895,(DndH.CTD.clade.1_WP_206844000.1:
0.0657431018,DndH.CTD.clade.1_PHC41327.1:0.0845305159)90.4/100:0.0507568397)99.6/100:0.1979900079)98.8/100:0.1620919452)96/100:0.1240174797)89.5/100:0.0829808429,(((DndH.CTD.clade.1_CDE45851.1:0.6406161501,(DndH.CTD.clade.1_EIF6167388.1:0.5107328213,(Dnd
H.CTD.clade.1_WP_195244854.1:0.5374184738,(DndH.CTD.clade.1_MBS6022526.1:0.2718045372,(DndH.CTD.clade.1_WP_087387658.1:0.2808091373,(DndH.CTD.clade.1_MCD8220049.1:0.3075409652,DndH.CTD.clade.1_MBQ5562066.1:0.2696458560)93.5/100:0.1238747612)98.3/1
00:0.1483568110)9.1/53:0.0491194611)94.7/100:0.1126526231)97.8/100:0.1724256730)69.3/97:0.1177359117,((DndH.CTD.clade.1_MBQ8962525.1:0.3426226979,(DndH.CTD.clade.1_OKZ97479.1:0.2077517100,(DndH.CTD.clade.1_MBR9937630.1:0.0641762931,DndH.CTD.clade.1_MBU
9728866.1:0.0874092180)99.8/100:0.1696873361)87.4/100:0.1181172547)100/100:0.2707990944,(((DndH.CTD.clade.1_WP_180493288.1:0.3002395414,((DndH.CTD.clade.1_WP_075678616.1:0.2471658423,DndH.CTD.clade.1_WP_113886234.1:0.2073928031)37/82:0.0616181971,((Dnd
H.CTD.clade.1_WP_026527776.1:0.2281911464,(DndH.CTD.clade.1_RHT07462.1:0.2764556134,DndH.CTD.clade.1_MBQ3784038.1:0.1764302361)99.2/100:0.1261357316)99.6/100:0.1417009285,DndH.CTD.clade.1_WP_092327985.1:0.1862075399)0/36:0.0342292523)93.6/99:0.096959
9054)97.7/100:0.0918344029,(DndH.CTD.clade.1_MBQ6208118.1:0.2827633126,DndH.CTD.clade.1_MBC2726973.1:0.2144570330)91/83:0.0908929558)85.4/81:0.0607727469,DndH.CTD.clade.1_HAW57574.1:0.3619417377)99.3/100:0.1757773594)97.2/100:0.1544537518)99.1/100:0.20
06997315,((((((DndH.CTD.clade.1_WP_018086851.1:0.5420517644,DndH.CTD.clade.1_MCD5401676.1:0.3658727538)57.2/94:0.1172993479,(DndH.CTD.clade.1_MBU4270784.1:0.1546983236,DndH.CTD.clade.1_MBE3035604.1:0.2720253550)99.9/100:0.2195152865)99/100:0.16843493
17,((DndH.CTD.clade.1_MBR4258823.1:0.7155340804,(DndH.CTD.clade.1_MBC8335620.1:0.3034383470,DndH.CTD.clade.1_NMA47277.1:0.3695225808)99.7/100:0.2521897025)80.2/67:0.0753278055,(((((DndH.CTD.clade.1_MBS6841633.1:0.5290383509,DndH.CTD.clade.1_WP_17601
5021.1:0.3016392266)0/6:0.0382339655,(DndH.CTD.clade.1_MBR6588285.1:0.2613120776,DndH.CTD.clade.1_MBR4189795.1:0.4773572157)93.5/99:0.0844811537)100/100:0.2932561996,((DndH.CTD.clade.1_NCB25559.1:0.3053395841,DndH.CTD.clade.1_WP_028893213.1:0.2157746
204)100/100:0.2177886969,(DndH.CTD.clade.1_MBU4386828.1:0.1941336935,(DndH.CTD.clade.1_MBA3016877.1:0.0727173557,DndH.CTD.clade.1_MBM3862078.1:0.0812183745)98.2/100:0.0949354857)100/100:0.3832363010)94.1/100:0.0697573642)79.9/79:0.0344211329,DndH.CTD
.clade.1_MCD6304951.1:0.4299046317)88.7/90:0.0703015900,DndH.CTD.clade.1_MBT3376946.1:0.4218803715)86.7/82:0.0460745937)54.1/81:0.0763799969)99.8/100:0.2462401885,((DndH.CTD.clade.1_CAA0097914.1:0.3498711345,((DndH.CTD.clade.1_MAE21955.1:0.2988264650,(D
ndH.CTD.clade.1_RHW76733.1:0.1387799540,DndH.CTD.clade.1_WP_088208772.1:0.1283153987)77/100:0.1023421238)99.6/100:0.1706391344,(DndH.CTD.clade.1_KZY84351.1:0.2122666641,(DndH.CTD.clade.1_WP_222488027.1:0.2589060685,(DndH.CTD.clade.1_STS87979.1:0.03
07615212,DndH.CTD.clade.1_WP_102386162.1:0.0609310447)100/100:0.3558905452)91.7/100:0.0715633189)96.5/100:0.1150708997)23.2/82:0.0493339861)100/100:0.3055044257,(DndH.CTD.clade.1_WP_149489189.1:0.7813492572,((DndH.CTD.clade.1_MBU2569723.1:0.3323240994
,(((((DndH.CTD.clade.1_MBV2089322.1:0.1727653829,(DndH.CTD.clade.1_HBC3609348.1:0.1348484713,DndH.CTD.clade.1_WP_046554824.1:0.1404411842)100/100:0.2354147885)76.5/95:0.0298969054,(DndH.CTD.clade.1_WP_168669196.1:0.3258297907,DndH.CTD.clade.1_WP_20
0206119.1:0.2258257017)91.5/99:0.0632526941)97.3/99:0.0654733197,(DndH.CTD.clade.1_WP_020160012.1:0.2576155770,DndH.CTD.clade.1_RKT46609.1:0.1972910654)87.3/98:0.0563886700)93.6/99:0.0471916045,DndH.CTD.clade.1_MBC7489077.1:0.2789110977)85.9/96:0.029632
4216,(DndH.CTD.clade.1_WP_020560063.1:0.1254345422,DndH.CTD.clade.1_TXH29053.1:0.0833262453)100/100:0.1696758817)94.5/97:0.0754584618)93.1/98:0.0848561105,DndH.CTD.clade.1_WP_197710756.1:0.4055523286)97.4/100:0.1597430931)96.2/100:0.1757512499)93.6/99:0
.1464500129)99.7/100:0.2684546838,((((((((D5-Sf3-asoc.HerA.FtsK_MBR1554071.1:0.2304502783,D5-Sf3-asoc.HerA.FtsK_HAJ97822.1:0.2420566683)70.8/100:0.0863447903,D5-Sf3-asoc.HerA.FtsK_MBR3417764.1:0.6320537967)23.9/52:0.0639944918,D5-Sf3-asoc.HerA.FtsK_MBR375
9965.1:0.4775905255)81.4/100:0.0989925462,D5-Sf3-asoc.HerA.FtsK_MBR3419571.1:0.6134667231)98.2/100:0.3622440227,(D5-Sf3-asoc.HerA.FtsK_MBO5343637.1:0.5418079952,D5-Sf3-asoc.HerA.FtsK_MBQ6943001.1:0.6749179759)100/100:0.8487990432)99/100:0.5107543583,((D5
-Sf3-asoc.HerA.FtsK_MBQ8010435.1:1.1115281246,((D5-Sf3-asoc.HerA.FtsK_MBR3269145.1:0.4407042593,D5-Sf3-asoc.HerA.FtsK_MBO5320619.1:0.6668322150)23.4/96:0.0828701309,(D5-Sf3-asoc.HerA.FtsK_WP_139275585.1:0.1527434237,D5-Sf3-asoc.HerA.FtsK_MBQ6944574.1:0
.1391599388)99.6/100:0.2098800058)97.6/100:0.2437005020)91.8/100:0.2060081516,D5-Sf3-asoc.HerA.FtsK_MBO5384602.1:0.6769395767)96.9/100:0.3974111105)100/100:1.0910754610,((((((((((DndH.CTD.clade.2_WP_158617667.1:0.2468994564,DndH.CTD.clade.2_WP_146657939.1:
0.2262962042)60/97:0.1017233294,DndH.CTD.clade.2_MCB9662367.1:0.2919222720)100/100:0.8102850841,((((((DndH.CTD.clade.2_WP_157180048.1:0.1154919979,DndH.CTD.clade.2_WP_051043557.1:0.0843542456)99.8/100:0.1003296671,DndH.CTD.clade.2_WP_224298347.1:0.17
98650041)8.9/59:0.0327729442,(DndH.CTD.clade.2_WP_051740830.1:0.1275920352,(DndH.CTD.clade.2_WP_205035614.1:0.1403122572,(DndH.CTD.clade.2_WP_083449096.1:0.1612497094,DndH.CTD.clade.2_WP_203135972.1:0.1747819620)88.8/100:0.0555089673)55/99:0.0316760
119)68.2/99:0.0276148995)99.8/100:0.2364788823,(DndH.CTD.clade.2_TDC88828.1:0.2238407294,(DndH.CTD.clade.2_WP_067910818.1:0.2341286824,DndH.CTD.clade.2_WP_158689901.1:0.2482365156)99.1/100:0.1498020727)99.6/100:0.2143683645)100/100:0.5305830443,(DndH.C
TD.clade.2_WP_097644648.1:0.5967668558,DndH.CTD.clade.2_WP_157294357.1:0.6470246763)92.7/100:0.1979625835)18/66:0.0792643008,((DndH.CTD.clade.2_WP_114586949.1:0.3117442507,(DndH.CTD.clade.2_ARS89935.1:0.1451538615,DndH.CTD.clade.2_WP_178918093.1:0.
1543913010)99.7/100:0.2289428272)71.1/99:0.1373033961,(DndH.CTD.clade.2_WP_152943811.1:0.3605606441,DndH.CTD.clade.2_WP_019176260.1:0.5213135158)20.7/96:0.0665753823)100/100:0.9124679958)62.8/85:0.1703095517)98.2/87:0.2357925712,((((((((((orc-cdc6.asoc.cellul
ar.FtsK_MBO0720477.1:0.2537607818,orc-cdc6.asoc.cellular.FtsK_MBO0724456.1:0.2195574633)100/100:0.3352210451,(orc-cdc6.asoc.cellular.FtsK_MBK9707133.1:0.2685225164,orc-cdc6.asoc.cellular.FtsK_MBL8169600.1:0.1781162151)95.4/100:0.1145844788)96.2/99:0.1000028875,(
orc-cdc6.asoc.cellular.FtsK_SPF37229.1:0.3527207456,orc-cdc6.asoc.cellular.FtsK_MCA1602030.1:0.3971679578)94.5/100:0.0823677859)94.1/99:0.0512447392,(orc-cdc6.asoc.cellular.FtsK_MBI5603591.1:0.3876263004,orc-cdc6.asoc.cellular.FtsK_UCH20765.1:0.4297976735)95.6/93:0.1
059103196)38.8/84:0.0339643883,(orc-cdc6.asoc.cellular.FtsK_MBN2032073.1:0.4207628046,orc-cdc6.asoc.cellular.FtsK_WP_050434458.1:0.5110426444)89.9/93:0.0805398425)95.3/98:0.0996285376,orc-cdc6.asoc.cellular.FtsK_QGJ68620.1:0.4618982040)98.8/100:0.1471957004,(orc-cd
c6.asoc.cellular.FtsK_MCC7418778.1:0.8128816459,orc-cdc6.asoc.cellular.FtsK_MBV9504667.1:0.5366608440)71/97:0.1090743285)88.2/95:0.0745522886,(orc-cdc6.asoc.cellular.FtsK_MBN2370103.1:0.4393304016,orc-cdc6.asoc.cellular.FtsK_MCC6133430.1:0.7011349959)90.8/100:0.17
47640894)99.9/100:0.2985892560,((((((orc-cdc6.asoc.cellular.FtsK_MBX3249387.1:0.4068949931,orc-cdc6.asoc.cellular.FtsK_MCA9645156.1:0.4805265912)54.4/93:0.0815629782,orc-cdc6.asoc.cellular.FtsK_MBE7479628.1:0.4965915327)90.8/95:0.0821783055,(((orc-cdc6.asoc.cellular.Ft
sK_NUQ74056.1:0.1585844099,orc-cdc6.asoc.cellular.FtsK_WP_153823168.1:0.2413335302)77.3/100:0.0663938192,orc-cdc6.asoc.cellular.FtsK_MBI4956683.1:0.2777251314)88.5/100:0.0655271249,orc-cdc6.asoc.cellular.FtsK_KYF72901.1:0.2329631042)99.8/100:0.1861841382)82.9/94:
0.0583131828,orc-cdc6.asoc.cellular.FtsK_MBI2896578.1:0.3751228346)97.7/99:0.1653747554,orc-cdc6.asoc.cellular.FtsK_OQA38222.1:0.4471803272)100/100:0.5498746227,(((orc-cdc6.asoc.cellular.FtsK_MBS0660553.1:0.5585311659,orc-cdc6.asoc.cellular.FtsK_MBV8533635.1:0.33967
49028)24.4/93:0.0861493333,orc-cdc6.asoc.cellular.FtsK_WP_138084700.1:0.4440566973)96.7/100:0.1600224518,((((orc-cdc6.asoc.cellular.FtsK_OQX01056.1:0.1842681257,(orc-cdc6.asoc.cellular.FtsK_WP_207692172.1:0.2303826558,orc-cdc6.asoc.cellular.FtsK_MBF0226910.1:0.19454
68511)58.7/87:0.0378021089)69.2/87:0.0377920542,orc-cdc6.asoc.cellular.FtsK_OQX07616.1:0.1611286483)92.6/100:0.0614412776,((orc-cdc6.asoc.cellular.FtsK_MBF0345360.1:0.2667961939,orc-cdc6.asoc.cellular.FtsK_MBF0344850.1:0.2760139129)97.4/100:0.1463577716,orc-cdc6.as
oc.cellular.FtsK_MBF0608684.1:0.3965648023)97.3/100:0.1220385177)97.9/100:0.1279595969,orc-cdc6.asoc.cellular.FtsK_NOZ11232.1:0.2896776744)98.1/100:0.1519458762)100/100:0.4073111638)94.3/94:0.1650753094)88/76:0.0922306540,(orc-cdc6.asoc.cellular.FtsK_MBN2326274.1:
0.9786506807,((((((((orc-cdc6.asoc.cellular.FtsK_PSO70359.1:0.1111447085,orc-cdc6.asoc.cellular.FtsK_WP_006099899.1:0.1515755402)9/91:0.0358149229,orc-cdc6.asoc.cellular.FtsK_WP_071518113.1:0.2595795966)90.2/93:0.0301550130,orc-cdc6.asoc.cellular.FtsK_WP_192220496.1:
0.1063490443)95/93:0.0397113762,(orc-cdc6.asoc.cellular.FtsK_HIK28643.1:0.2251795587,(orc-cdc6.asoc.cellular.FtsK_NET87102.1:0.2376265217,orc-cdc6.asoc.cellular.FtsK_MBE9118227.1:0.1659950952)61.9/96:0.0584515858)93.7/95:0.0547485535)82.4/86:0.0249951618,((((((orc-cdc
6.asoc.cellular.FtsK_OBQ14047.1:0.0082032000,(orc-cdc6.asoc.cellular.FtsK_WP_028084559.1:0.0358400316,orc-cdc6.asoc.cellular.FtsK_WP_193890658.1:0.0322216741)86.1/98:0.0106272724)96.5/98:0.0487144837,orc-cdc6.asoc.cellular.FtsK_MBS9388808.1:0.0259859751)94/98:0.02
55444875,orc-cdc6.asoc.cellular.FtsK_WP_168491207.1:0.0281626866)75.9/98:0.0172101501,((orc-cdc6.asoc.cellular.FtsK_QSV66407.1:0.0250494944,orc-cdc6.asoc.cellular.FtsK_WP_168357748.1:0.0029006061)99.7/100:0.0465166755,orc-cdc6.asoc.cellular.FtsK_MBS3028698.1:0.0161
759941)95.1/100:0.0225006931)100/100:0.1132349288,orc-cdc6.asoc.cellular.FtsK_MBW4674934.1:0.1176695561)56/77:0.0202246137,((orc-cdc6.asoc.cellular.FtsK_WP_095723314.1:0.1368645846,orc-cdc6.asoc.cellular.FtsK_MBF2063515.1:0.0896885602)98/100:0.0862722635,orc-cdc6
.asoc.cellular.FtsK_WP_226577925.1:0.1862828673)59.3/76:0.0214896098)95/98:0.0420450451)83.6/82:0.0212884025,(((orc-cdc6.asoc.cellular.FtsK_WP_083883959.1:0.2410854334,(orc-cdc6.asoc.cellular.FtsK_WP_080805386.1:0.0748128065,orc-cdc6.asoc.cellular.FtsK_TVQ09710.1:0.
0865436283)99.7/100:0.1210856783)68.3/42:0.0364940257,(orc-cdc6.asoc.cellular.FtsK_WP_083636709.1:0.1957062802,orc-cdc6.asoc.cellular.FtsK_WP_190682060.1:0.1523062406)94.6/72:0.0607779843)86.1/57:0.0324332504,(((orc-cdc6.asoc.cellular.FtsK_KPQ35622.1:0.1439272261,
orc-cdc6.asoc.cellular.FtsK_PZO19400.1:0.1125523335)100/100:0.1533518500,orc-cdc6.asoc.cellular.FtsK_WP_045054776.1:0.1681458766)81.1/87:0.0407011795,orc-cdc6.asoc.cellular.FtsK_MCC5899704.1:0.3188572097)26.9/48:0.0123306106)95/83:0.0427544920)84.6/72:0.031382914
0,((orc-cdc6.asoc.cellular.FtsK_PIG95231.1:0.0607693868,orc-cdc6.asoc.cellular.FtsK_WP_105219461.1:0.0546442082)82.6/100:0.0220550300,orc-cdc6.asoc.cellular.FtsK_WP_104548503.1:0.0802160433)99.2/100:0.0950026342)97/75:0.0827619731,orc-cdc6.asoc.cellular.FtsK_WP_2083
39627.1:0.1979395089)100/100:0.4082810057)49.9/91:0.1060210200)69.2/72:0.0729826180,((orc-cdc6.asoc.cellular.FtsK_KPA08820.1:0.4844487025,orc-cdc6.asoc.cellular.FtsK_MBI5724653.1:0.4241306552)89.1/99:0.1336832662,orc-cdc6.asoc.cellular.FtsK_MBR1672097.1:0.511835352
4)100/100:0.4455226880)71.4/92:0.1145118016)89.9/85:0.1790190726,((DndH.CTD.clade.2_WP_026262138.1:0.1872527750,(DndH.CTD.clade.2_WP_206986794.1:0.2047077832,DndH.CTD.clade.2_WP_131527690.1:0.2879536827)90.3/100:0.1144593449)100/100:0.5052535970,((DndH
.CTD.clade.2_MBK8979716.1:0.3857834480,(DndH.CTD.clade.2_MBT4511846.1:0.4305994192,DndH.CTD.clade.2_UJR83668.1:0.4392206068)26.8/86:0.0730147746)93.9/91:0.1446809292,(DndH.CTD.clade.2_WP_027850877.1:0.1518028701,(DndH.CTD.clade.2_AMJ85185.1:0.1319093
486,((DndH.CTD.clade.2_WP_182711930.1:0.0154305381,DndH.CTD.clade.2_WP_101106963.1:0.0441819159)99.4/100:0.0632503292,DndH.CTD.clade.2_WP_126668716.1:0.0467578600)91.9/100:0.0389645403)95.5/100:0.0916015614)100/100:0.4178446217)91.4/99:0.1707437008)100
/100:0.4945047934)2.6/29:0.1117592619,(((DndH.CTD.clade.2_WP_164947261.1:0.1214713891,DndH.CTD.clade.2_WP_049179791.1:0.1660458892)99.9/100:0.2947885722,((DndH.CTD.clade.2_WP_204665033.1:0.1936350372,DndH.CTD.clade.2_WP_193774329.1:0.1538704341)99.7/1
00:0.2116150117,(DndH.CTD.clade.2_HJF36660.1:0.1803361497,DndH.CTD.clade.2_OPJ55650.1:0.1741639671)98.6/100:0.1535576792)67.9/100:0.1205009106)99.8/100:0.3198538800,(DndH.CTD.clade.2_OQY10741.1:0.5883751526,DndH.CTD.clade.2_WP_078694074.1:0.4375669317
)62/97:0.1327328452)96/100:0.2260560917)52.8/62:0.2034994909,(DndH.CTD.clade.2_MBP7950546.1:0.5157054613,DndH.CTD.clade.2_WP_107338691.1:0.5791502335)99.6/100:0.6877109148)100/100:1.8788314280,(((iorc-cdc6.and.REase-AbiJ.asoc.HerA.FtsK_TMQ17324.1:0.422712
0955,iorc-cdc6.and.REase-AbiJ.asoc.HerA.FtsK_MBK9032846.1:0.3479742223)99.3/100:0.4873597976,iorc-cdc6.and.REase-AbiJ.asoc.HerA.FtsK_RYZ03034.1:0.6869571787)100/100:1.0096551460,(((iorc-cdc6.and.REase-AbiJ.asoc.HerA.FtsK_MBL7646281.1:0.1969532172,iorc-cdc6.and
.REase-AbiJ.asoc.HerA.FtsK_MBX3180360.1:0.1979329626)100/100:0.2958690887,(iorc-cdc6.and.REase-AbiJ.asoc.HerA.FtsK_NLY00346.1:0.4136293282,iorc-cdc6.and.REase-AbiJ.asoc.HerA.FtsK_ASC73273.1:0.4593541523)87.5/100:0.0775446745)96.2/100:0.1748576044,((((((iorc-cdc
6.and.REase-AbiJ.asoc.HerA.FtsK_MBB4752195.1:0.2802173867,(iorc-cdc6.and.REase-AbiJ.asoc.HerA.FtsK_MBP2703037.1:0.2099382891,iorc-cdc6.and.REase-AbiJ.asoc.HerA.FtsK_GGK81482.1:0.2846272998)80.4/76:0.0644553113)40.6/8:0.0428934788,((((iorc-cdc6.and.REase-AbiJ.a
soc.HerA.FtsK_NUR72155.1:0.1134788214,iorc-cdc6.and.REase-AbiJ.asoc.HerA.FtsK_NUT36745.1:0.1219161443)100/100:0.2528117936,iorc-cdc6.and.REase-AbiJ.asoc.HerA.FtsK_HCT75248.1:0.2581556639)79.2/46:0.0673262032,iorc-cdc6.and.REase-AbiJ.asoc.HerA.FtsK_OLB77286.1
:0.2133189958)53.8/45:0.0446466234,iorc-cdc6.and.REase-AbiJ.asoc.HerA.FtsK_BCJ69174.1:0.2170151798)17.5/9:0.0174718829)95.2/85:0.0824631964,iorc-cdc6.and.REase-AbiJ.asoc.HerA.FtsK_SUE15246.1:0.2329790697)98.2/87:0.1274212398,((iorc-cdc6.and.REase-AbiJ.asoc.HerA.
FtsK_QHN36297.1:0.0688951399,iorc-cdc6.and.REase-AbiJ.asoc.HerA.FtsK_MCB1296808.1:0.0381139221)100/100:0.4224194314,iorc-cdc6.and.REase-AbiJ.asoc.HerA.FtsK_MBE7159683.1:0.2444071600)79.1/100:0.0961588817)91.8/91:0.1193131287,iorc-cdc6.and.REase-AbiJ.asoc.Her
A.FtsK_KXP05122.1:0.5008371759)100/100:0.2909342796,iorc-cdc6.and.REase-AbiJ.asoc.HerA.FtsK_NKJ14083.1:0.4704553453)78.2/99:0.1175527282)99.6/100:0.7772237735)95.6/100:0.7016451199)83.8/89:0.3933953797,(((((DndH.CTD.clade.6_MBQ3341209.1:0.2747106852,(DndH.C
TD.clade.6_MBN8685228.1:0.2913323555,(DndH.CTD.clade.6_WP_013687708.1:0.1951862167,DndH.CTD.clade.6_GGK34441.1:0.3506253226)96.4/100:0.0875158907)82.9/100:0.0473512093)23.4/70:0.0373622133,(DndH.CTD.clade.6_MBO4721346.1:0.1337713609,(DndH.CTD.clade.6
_MBQ7422594.1:0.2002602517,DndH.CTD.clade.6_WP_089543154.1:0.1081065535)99.2/100:0.1002075483)100/100:0.2003177427)90.4/98:0.0649271629,(DndH.CTD.clade.6_WP_126696016.1:0.3335899958,DndH.CTD.clade.6_WP_162386024.1:0.3641060053)97.7/100:0.1096728711)
83.9/91:0.0804493475,(((((DndH.CTD.clade.6_MBC8492616.1:0.4616973977,((((((DndH.CTD.clade.6_RRJ95660.1:0.3734625168,DndH.CTD.clade.6_MCC5789787.1:0.2677811389)85.3/47:0.0689671017,(((((DndH.CTD.clade.6_WP_091054375.1:0.3320984627,DndH.CTD.clade.6_MBX63
87503.1:0.1730103062)51.3/28:0.0452207733,DndH.CTD.clade.6_WP_123607077.1:0.2067547005)60.7/28:0.0234618788,(DndH.CTD.clade.6_RSS46956.1:0.2364110041,(DndH.CTD.clade.6_CNE22073.1:0.1928964786,(((DndH.CTD.clade.6_WP_091123179.1:0.2202027062,DndH.CTD.c
lade.6_WP_063910667.1:0.1221600103)0/15:0.0314596081,DndH.CTD.clade.6_MBV9382513.1:0.2318465938)38/40:0.0206131391,((DndH.CTD.clade.6_WP_176406117.1:0.0877315146,DndH.CTD.clade.6_WP_111832496.1:0.1015103988)95.2/100:0.0349553141,DndH.CTD.clade.6_WP
_189164086.1:0.1069009498)90.6/100:0.0286100089)86.6/44:0.0336033932)68.6/90:0.0284100882)75.4/100:0.0293827708)100/99:0.1042414070,(DndH.CTD.clade.6_APY87825.1:0.1434390694,DndH.CTD.clade.6_GCB46459.1:0.3269685191)51.5/89:0.0345958406)76.3/52:0.035233330
7,((DndH.CTD.clade.6_TXC95218.1:0.1741649514,DndH.CTD.clade.6_OSC73727.1:0.1454775907)63.6/99:0.0605706851,DndH.CTD.clade.6_WP_106535231.1:0.3014974084)81.9/98:0.0360428706)100/100:0.1237771250)91.1/46:0.0297972245,(((DndH.CTD.clade.6_WP_184533930.1:0.
1414718746,(DndH.CTD.clade.6_MAT24856.1:0.1172940202,DndH.CTD.clade.6_MCB9694483.1:0.1363003959)98.7/100:0.0654638302)48.6/49:0.0158052559,((DndH.CTD.clade.6_WP_145615395.1:0.1634437074,DndH.CTD.clade.6_MBE0624561.1:0.1620680822)26.4/57:0.0212828291,
DndH.CTD.clade.6_BBZ99844.1:0.1611684987)85/94:0.0319322583)94.8/100:0.0462760136,(DndH.CTD.clade.6_OGP93187.1:0.2542065997,(DndH.CTD.clade.6_WP_028489938.1:0.0897481506,DndH.CTD.clade.6_EGR0758329.1:0.1686689722)99.9/100:0.1963926691)95.3/47:0.06058
36949)89.5/44:0.0263344898)92.9/75:0.0401354782,(DndH.CTD.clade.6_WP_145039185.1:0.2598059454,(DndH.CTD.clade.6_WP_190711418.1:0.1966667295,(DndH.CTD.clade.6_MBK8463956.1:0.1935172936,(DndH.CTD.clade.6_MXZ74164.1:0.1536660496,DndH.CTD.clade.6_WP_07
3478276.1:0.1475180843)37.9/98:0.0586637724)99.2/100:0.0801886319)97.8/100:0.0735219543)46.7/70:0.0312572016)90.5/77:0.0370464322,(DndH.CTD.clade.6_MBZ2184884.1:0.3120253136,DndH.CTD.clade.6_WP_058553930.1:0.3321086330)53/71:0.0512846080)95.9/69:0.0687083
908,DndH.CTD.clade.6_WP_155300802.1:0.3524884484)99.9/100:0.1468326366)88/95:0.0531116458,(DndH.CTD.clade.6_TAK28174.1:0.4536577916,(((DndH.CTD.clade.6_NLS90948.1:0.2345298406,DndH.CTD.clade.6_MBY0276573.1:0.2016738795)76.8/98:0.0468899524,DndH.CTD.cl
ade.6_MBI5068730.1:0.2989773236)58.1/83:0.0235130150,(DndH.CTD.clade.6_NJO48468.1:0.2517242983,(DndH.CTD.clade.6_WP_129349189.1:0.2490009895,DndH.CTD.clade.6_WP_085348573.1:0.1560069272)84.4/99:0.0498148265)58.7/96:0.0296057112)100/100:0.3579342070)92.
1/93:0.0786141792)82.1/84:0.0484041973,(((DndH.CTD.clade.6_MBU6349865.1:0.2588488582,(DndH.CTD.clade.6_MCC6613392.1:0.2958929298,(DndH.CTD.clade.6_NLF00618.1:0.2189481210,DndH.CTD.clade.6_MCA9948060.1:0.1563109412)46.9/88:0.0458754163)48.7/86:0.052544
1685)97.6/99:0.0860006483,DndH.CTD.clade.6_OJV96058.1:0.2694457631)87.6/99:0.0568514474,DndH.CTD.clade.6_WP_201372618.1:0.3585210969)95.7/92:0.0770318118)23.4/65:0.0553279504,DndH.CTD.clade.6_PZS07826.1:0.5014081823)95.7/67:0.0788558468,(DndH.CTD.clade.
6_WP_173479487.1:0.2582261022,DndH.CTD.clade.6_WP_098878760.1:0.3232700244)100/100:0.3187616122)49.4/56:0.0438277370)96.3/93:0.5297439310,(((((((KIM21768.1_Fungi_Serendipita.vermifera_:0.1884432926,PVF92780.1_Fungi_Serendipita.vermifera:0.1887310867)100/100:
0.3038911003,((KZV91508.1_Fungi_Exidia.glandulosa:0.2132461626,KAH7101552.1_Fungi_Auriculariales.sp.:0.3954287843)98.1/100:0.1201088854,KAH7101569.1_Fungi_Auriculariales.sp.:0.2664782366)99.7/100:0.1878913374)34.3/98:0.0512216773,KIY48600.1_Fungi_Fistulina.hepatic
a:0.6189114911)91.7/100:0.1213144125,(KAA1120836.1_Fungi_Puccinia.graminis:0.3190006370,MBW0526223.1_Fungi_Austropuccinia.psidii:0.2820965157)100/100:0.3693439205)54.7/99:0.1134066742,(KAG8885148.1_Fungi_Tulasnella.sp.:0.3743271261,((KIP09214.1_Fungi_Phlebiopsi
s.gigantea:0.3595199770,KAF9782592.1_Fungi_Thelephora.terrestris:0.3019239978)25.6/88:0.0664207524,KAF9779993.1_Fungi_Thelephora.terrestris:0.4761981422)84.6/100:0.0774672268)100/100:0.2705687186)73.4/97:0.1684399017,((OQO23747.1_Fungi_Rachicladosporium.sp.:0.53
20168085,(TKA21706.1_Fungi_Salinomyces.thailandica:0.4047499251,KXL42170.1_Fungi_Acidomyces.sp.:0.5823606833)43.2/57:0.0567364261)62.2/57:0.0958393042,XP_033460502.1_Fungi_Dissoconium.aciculare:0.4051671532)100/100:0.4427991246)96.8/100:0.3039266413,((KAF08
52322.1_Jakob_Andalucia.godoyi:0.5551455816,XP_004364951.1_Filas_Capsaspora.owczarzaki:0.6179987349)99.8/100:0.4715438209,GIQ79440.1_Forni_Kipferlia.bialata:1.2328592299)38.4/79:0.1598904649)99.2/100:0.7290062390)100/100:1.8764445580)76.3/81:0.2171500709,(((((Dn
dH.CTD.clade.3_MBQ3250375.1:0.2258365171,DndH.CTD.clade.3_WP_081029990.1:0.2161411080)96.7/100:0.1378850752,((DndH.CTD.clade.3_WP_091957083.1:0.2140829681,((DndH.CTD.clade.3_WP_231566012.1:0.0738529886,DndH.CTD.clade.3_WP_139147270.1:0.0445816425)
94.2/100:0.0417433577,(DndH.CTD.clade.3_WP_008621055.1:0.0660293701,DndH.CTD.clade.3_MCD8290639.1:0.1011358359)98.3/100:0.0634770926)37.6/100:0.0498976158)95.1/100:0.0965145893,(DndH.CTD.clade.3_MCD7816811.1:0.2154485653,DndH.CTD.clade.3_MBP5563334.
1:0.2691165547)90.5/98:0.0678425676)95.4/98:0.1513588906)99.9/100:0.4839376079,(((((((DndH.CTD.clade.3_WP_209280800.1:0.2551656028,DndH.CTD.clade.3_MBM7629504.1:0.2124962610)81.3/95:0.0452256656,(DndH.CTD.clade.3_RCX12108.1:0.2001144598,(DndH.CTD.clade.3
_WP_218015226.1:0.2768391193,((DndH.CTD.clade.3_WP_164783225.1:0.1682926087,DndH.CTD.clade.3_WP_172251699.1:0.0553879926)39.4/83:0.0333070598,DndH.CTD.clade.3_WP_013920767.1:0.0841054931)99.7/100:0.1303902715)98.5/100:0.1067817652)88.6/96:0.046987591
4)97.6/100:0.0853453421,(((DndH.CTD.clade.3_KAB2405544.1:0.1769948685,(DndH.CTD.clade.3_WP_179124101.1:0.1572151485,(DndH.CTD.clade.3_WP_191965120.1:0.1679384099,DndH.CTD.clade.3_WP_184662913.1:0.2444953754)90.7/100:0.0777908322)99.7/100:0.1355094997)
99.9/100:0.1769495196,(DndH.CTD.clade.3_MBN2837851.1:0.5318095395,(((DndH.CTD.clade.3_TFH85692.1:0.3012242277,((DndH.CTD.clade.3_WP_230251575.1:0.3101122414,DndH.CTD.clade.3_WP_066015826.1:0.2888586365)95.6/93:0.0725947204,((DndH.CTD.clade.3_MBT6051
961.1:0.3425856534,(DndH.CTD.clade.3_AMQ88781.1:0.1892736560,DndH.CTD.clade.3_MAE92545.1:0.1297220411)32.6/92:0.0336400109)58.5/95:0.0305468525,DndH.CTD.clade.3_WP_102627100.1:0.2166683758)90.5/100:0.0501090520)54.5/84:0.0236835425)97.6/100:0.086641329
0,((((((((DndH.CTD.clade.3_WP_231899968.1:0.2140182063,((DndH.CTD.clade.3_MBX9636990.1:0.1321422197,((DndH.CTD.clade.3_XP_024572616.1:0.0407413567,(DndH.CTD.clade.3_HAL5997494.1:0.0140278105,DndH.CTD.clade.3_EDH9581117.1:0.5099140134)78.8/94:0.0129182
147)68.1/95:0.0266855504,DndH.CTD.clade.3_WP_004949442.1:0.1280077205)99.9/100:0.1252046511)100/100:0.1207076441,(DndH.CTD.clade.3_WP_068372293.1:0.0866613399,(DndH.CTD.clade.3_WP_197173445.1:0.0147192691,DndH.CTD.clade.3_WP_232735484.1:0.049430783
3)98.8/100:0.0535438480)71/100:0.0162469978)99.1/88:0.0587348535)95.3/76:0.0458463801,DndH.CTD.clade.3_MBB3047422.1:0.1707766393)87.9/64:0.0174397069,DndH.CTD.clade.3_GIU15772.1:0.0904225939)57.2/47:0.0125894496,(DndH.CTD.clade.3_WP_055022121.1:0.0929213
876,DndH.CTD.clade.3_WP_119394090.1:0.0638552567)98.6/100:0.0473221985)61.5/54:0.0327351355,DndH.CTD.clade.3_WP_222302203.1:0.1468211472)99/76:0.0827950555,DndH.CTD.clade.3_WP_091714800.1:0.1384345355)83.4/60:0.0326741857,(((DndH.CTD.clade.3_WP_04462
4140.1:0.2915850096,(DndH.CTD.clade.3_WP_206403428.1:0.0906392324,((DndH.CTD.clade.3_WP_208144343.1:0.0709428863,DndH.CTD.clade.3_WP_217513620.1:0.0662302926)39.1/98:0.0164348612,DndH.CTD.clade.3_WP_151653604.1:0.0291196925)94.3/99:0.0274316156)24.2/
66:0.0276346959)0/75:0.0000020033,(DndH.CTD.clade.3_MBE8233352.1:0.0804844053,DndH.CTD.clade.3_WP_121851609.1:0.0532162216)99.8/100:0.0752068094)91/94:0.0406462322,DndH.CTD.clade.3_WP_226421862.1:0.1437743086)85/91:0.0374779631)98.3/75:0.0721127011,((D
ndH.CTD.clade.3_WP_046889946.1:0.1831590411,(DndH.CTD.clade.3_WP_004678904.1:0.0154056170,DndH.CTD.clade.3_WP_235471579.1:0.0174650126)99.2/100:0.0705178821)98/100:0.0829377484,DndH.CTD.clade.3_WP_161417541.1:0.2227614890)97.8/95:0.0825852035)95.7/1
00:0.0806595548,DndH.CTD.clade.3_WP_233814257.1:0.4331687077)99.5/100:0.1510438016)91.7/100:0.0823752896,(DndH.CTD.clade.3_WP_231563910.1:0.3941991423,DndH.CTD.clade.3_WP_192391910.1:0.3210851335)96.8/100:0.1099195382)95.6/100:0.1281450692)90.4/94:0.09
77055254)65.4/76:0.0570657764,((DndH.CTD.clade.3_MBN2836543.1:0.4332573891,(DndH.CTD.clade.3_WP_207424294.1:0.2684986042,DndH.CTD.clade.3_WP_168882627.1:0.2301892472)97.4/100:0.1223935989)58.1/19:0.0606376862,(((DndH.CTD.clade.3_WP_231490318.1:0.2768
671441,(DndH.CTD.clade.3_WP_129715543.1:0.0797505048,DndH.CTD.clade.3_WP_162431192.1:0.0311985621)99.6/100:0.1561832220)99.9/100:0.1899942526,DndH.CTD.clade.3_WP_002704320.1:0.2188774466)86.7/7:0.0311007896,(DndH.CTD.clade.3_MBV5341494.1:0.359689549
6,(DndH.CTD.clade.3_WP_103191867.1:0.1571692396,((DndH.CTD.clade.3_WP_223052470.1:0.2047083834,DndH.CTD.clade.3_WP_198424144.1:0.0933444052)99.7/100:0.1152467963,DndH.CTD.clade.3_WP_088468727.1:0.1267977321)52.3/98:0.0528875551)99.9/100:0.1403764097)
42.2/67:0.0416882235)47.5/10:0.0536494220)96.4/90:0.0801149700)86.3/76:0.0398668360)43.9/75:0.0372806445,((DndH.CTD.clade.3_WP_055070051.1:0.2277679916,(DndH.CTD.clade.3_WP_127065281.1:0.1941833784,(DndH.CTD.clade.3_WP_122638756.1:0.1337977443,(((DndH.C
TD.clade.3_WP_231072268.1:0.1623837573,DndH.CTD.clade.3_WP_099335658.1:0.1120380617)96.6/100:0.0705260623,DndH.CTD.clade.3_NOW91076.1:0.0972377452)100/100:0.1253823866,((DndH.CTD.clade.3_WP_013726262.1:0.0608382482,(DndH.CTD.clade.3_WP_076175550.1
:0.0281546213,DndH.CTD.clade.3_MBZ1339973.1:0.0312635983)95.8/100:0.0334503930)97.9/100:0.0487480370,((DndH.CTD.clade.3_NFG76354.1:0.0641713688,DndH.CTD.clade.3_WP_069679918.1:0.0253049774)86.1/99:0.0177693109,(DndH.CTD.clade.3_WP_173713800.1:0.03628
32374,DndH.CTD.clade.3_WP_013238116.1:0.0655191915)71.3/92:0.0089315683)62.8/100:0.0286165885)70.8/100:0.0206919488)82.4/99:0.0320640620)99.4/100:0.1039447044)94.7/99:0.0634166251)53.7/70:0.0491631309,(DndH.CTD.clade.3_MBD8913298.1:0.3932486147,((DndH.CT
D.clade.3_OUP59763.1:0.2352496711,DndH.CTD.clade.3_MBP2656983.1:0.4191526568)22.4/22:0.0762207188,(((DndH.CTD.clade.3_RKJ47910.1:0.1723258605,((DndH.CTD.clade.3_MBD5540058.1:0.1757353541,DndH.CTD.clade.3_PWJ10972.1:0.1152288767)13.3/25:0.0497716239,(D
ndH.CTD.clade.3_WP_173772980.1:0.1888815997,DndH.CTD.clade.3_WP_155886450.1:0.2223570599)45.2/63:0.0463354411)67.7/90:0.0337378044)97.6/100:0.0714251237,DndH.CTD.clade.3_KUE75183.1:0.2215796876)87.7/100:0.0513475801,((DndH.CTD.clade.3_BBH26281.1:0.281
0731425,DndH.CTD.clade.3_WP_076340719.1:0.3969842346)95/100:0.0799604778,DndH.CTD.clade.3_NWO22399.1:0.3412402847)91.3/94:0.0518818154)87.6/94:0.0414400077)100/100:0.1871204386)73.4/98:0.0865731922)93.5/97:0.0692823830)91.6/79:0.0563194303,((DndH.CTD.cla
de.3_MBS5024481.1:0.2107531686,DndH.CTD.clade.3_WP_211367301.1:0.3047919357)70.2/93:0.0491074443,DndH.CTD.clade.3_PAB60530.1:0.2556406765)88.8/88:0.0564963786)74/84:0.1024374176,DndH.CTD.clade.3_NFC46410.1:0.4390357669)24.2/84:0.1122817373,DndH.CTD.cl
ade.3_WP_034328834.1:0.3831053853)98.4/100:0.3507534512)100/100:0.6897712325,((((((((((DndE.FtsK._WP_195665463.1:0.0670037342,DndE.FtsK._MBE6280335.1:0.1121138233)100/100:0.2319147452,DndE.FtsK._MBL4586840.1:0.2427085467)100/100:0.2085741358,DndE.FtsK._
WP_207025792.1:0.3828263925)20.1/90:0.0357333708,((DndE.FtsK._WP_163273966.1:0.3835638289,DndE.FtsK._WP_032572122.1:0.2678160360)96.6/96:0.1009074387,(DndE.FtsK._MBQ4408863.1:0.3349308621,DndE.FtsK._WP_144257341.1:0.3444485220)61.5/95:0.0474863803)9
9.8/100:0.1470176976)89.8/99:0.0585267129,(((DndE.FtsK._MVM40840.1:0.4804072268,(DndE.FtsK._WP_132121428.1:0.3252095880,DndE.FtsK._WP_135431160.1:0.2280889107)94.3/100:0.0953513298)98.8/100:0.1238515804,DndE.FtsK._TAG40970.1:0.1737392747)58/100:0.062937
6763,(DndE.FtsK._MBY0425657.1:0.2590306277,DndE.FtsK._WP_062588831.1:0.4934035967)92.6/99:0.0792718610)93.1/100:0.0977023720)81/99:0.0684564670,((DndE.FtsK._MBK9457847.1:0.6361584007,DndE.FtsK._WP_013766328.1:0.2924788919)99.8/100:0.2296908511,(DndE.Ft
sK._MBN2890288.1:0.3235189465,DndE.FtsK._MBW1616874.1:0.4021726615)98.4/100:0.1450646804)82.5/94:0.0589491749)4.4/62:0.0546072010,(DndE.FtsK._MBN2881439.1:0.2205853949,DndE.FtsK._WP_007096426.1:0.3659935715)99.7/100:0.1692977232)97.6/94:0.2346539647,D
ndE.FtsK._WP_051633174.1:0.8899851548)99.9/100:0.4088399548,((((DndE.FtsK._WP_131850661.1:0.5002834803,(((DndE.FtsK._WP_223255486.1:0.2058939381,(DndE.FtsK._WP_188440190.1:0.1239132985,(DndE.FtsK._MBS9783532.1:0.1989349170,DndE.FtsK._WP_198411624.1:
0.3139913842)99.9/100:0.1849023342)95.7/100:0.0942189609)88.2/100:0.0629695641,DndE.FtsK._OFY50564.1:0.3426817323)100/100:0.2194155523,DndE.FtsK._RZK26739.1:0.3816788304)78.2/97:0.0813378159)31.1/89:0.0678031923,(DndE.FtsK._MCB9310860.1:0.3130742665,Dnd
E.FtsK._MBL7689990.1:0.3557496870)100/100:0.2667797079)45.5/97:0.0838726031,DndE.FtsK._MBD3343365.1:0.5377428033)99/99:0.2336398577,(DndE.FtsK._NTW31743.1:0.4001943363,DndE.FtsK._WP_072945266.1:0.2859700284)100/100:0.6244555612)99.4/100:0.3095859879)9
6/100:0.2259893774,(DndE.FtsK._MBI3649432.1:0.5432339942,(DndE.FtsK._MCB1194022.1:0.3730997527,DndE.FtsK._NJL12890.1:0.7706574386)88.7/100:0.2817742406)100/100:0.5513237895)90.5/100:0.2498960883,(DndE.FtsK._MBK9138942.1:0.4795700417,(DndE.FtsK._MBV9658
019.1:0.2102345817,DndE.FtsK._NBP02146.1:0.5154425702)72.7/100:0.1596028518)100/100:1.3674228440)94.8/100:0.3630604060)98.3/100:0.4432336600,((((((DndH.CTD.clade.4_WP_138085268.1:0.5960083402,((DndH.CTD.clade.4_NMW21964.1:0.2938313155,(((DndH.CTD.clade.4_
MQM31000.1:0.1429754688,DndH.CTD.clade.4_MBF0158320.1:0.4370115243)58/96:0.0676843198,(DndH.CTD.clade.4_OGA48129.1:0.2155972960,DndH.CTD.clade.4_WP_090680600.1:0.2109902967)98.6/100:0.1332328450)97.3/100:0.1163275469,(DndH.CTD.clade.4_WP_179636399
.1:0.3844383040,(DndH.CTD.clade.4_WP_051497895.1:0.2901553391,DndH.CTD.clade.4_WP_200286167.1:0.3892029870)69.6/95:0.1091723145)91.4/97:0.0860379772)88.6/97:0.0954033791)98.7/100:0.1603723846,(DndH.CTD.clade.4_RTL43633.1:0.4753160491,DndH.CTD.clade.4_N
MC60188.1:0.5815677463)92.1/100:0.1224260759)11.1/94:0.0407478707)2.2/66:0.0368605787,((DndH.CTD.clade.4_MBF2760000.1:0.2670841506,(((DndH.CTD.clade.4_RLC12986.1:0.1682320535,(DndH.CTD.clade.4_MBE7489048.1:0.1522864706,DndH.CTD.clade.4_OQB75210.1:0.29
91643573)90.9/100:0.0570799029)86.4/100:0.0451562436,DndH.CTD.clade.4_MCC6454488.1:0.2141159554)49/99:0.0531853636,DndH.CTD.clade.4_HGF30716.1:0.3673958026)69.1/99:0.0963353422)88.9/100:0.0901261656,(DndH.CTD.clade.4_WP_211423609.1:0.3269340334,DndH.C
TD.clade.4_OYT73417.1:0.4302370263)99.7/100:0.2656616601)100/100:0.2432331903)33.9/77:0.0595711535,(DndH.CTD.clade.4_WP_234827951.1:0.2721402004,DndH.CTD.clade.4_WP_225028399.1:0.2584046780)100/100:0.3190673408)90.2/86:0.1487245649,((DndH.CTD.clade.4_W
P_224435281.1:0.0657753118,DndH.CTD.clade.4_WP_145095303.1:0.0531851194)70.1/73:0.0262899252,DndH.CTD.clade.4_HBJ34827.1:0.0838605489)99.9/100:0.3879951163)100/100:0.9189986649,(((((((DndH.CTD.clade.5_MBI1930032.1:0.4077221805,DndH.CTD.clade.5_MBP89511
04.1:0.8142345820)99.9/100:0.3206296754,DndH.CTD.clade.5_NLF73614.1:0.3807621403)60.8/41:0.1119993909,((DndH.CTD.clade.5_WP_011603798.1:0.1176449905,DndH.CTD.clade.5_WP_071062135.1:0.1294188610)100/100:0.4214961038,(((DndH.CTD.clade.5_WP_139573932.1:0.
2066921679,((DndH.CTD.clade.5_OLM08996.1:0.1362454544,DndH.CTD.clade.5_WP_230740323.1:0.1324181591)98.1/100:0.0874094661,(((DndH.CTD.clade.5_WP_220260123.1:0.0633916493,DndH.CTD.clade.5_WP_219417039.1:0.0911299416)99.2/100:0.0820267754,DndH.CTD.cla
de.5_WP_181778840.1:0.1808175427)36.8/99:0.0446168478,DndH.CTD.clade.5_WP_172166640.1:0.1549890150)0/23:0.0261641695)97.4/100:0.1081168717)99.3/100:0.1411648327,(DndH.CTD.clade.5_WP_111178844.1:0.3067102147,(DndH.CTD.clade.5_WP_215785315.1:0.27280222
95,((DndH.CTD.clade.5_WP_143317302.1:0.2689676032,DndH.CTD.clade.5_WP_210054905.1:0.2418287407)97.9/100:0.1293372300,(DndH.CTD.clade.5_WP_163677225.1:0.3693418555,(DndH.CTD.clade.5_WP_139073713.1:0.1399390327,DndH.CTD.clade.5_WP_151084524.1:0.2373
161284)98.2/100:0.1540176534)96.7/100:0.1011406588)58.6/99:0.0601641857)93.5/100:0.1081645585)99.3/100:0.1473301887)75.6/100:0.0607087273,DndH.CTD.clade.5_WP_056022014.1:0.2788660857)99.8/100:0.2506653049)97.7/95:0.1622157813)84.4/36:0.0977666604,DndH.CTD.c
lade.5_QDT04241.1:0.6714232188)86.6/36:0.0625365712,(DndH.CTD.clade.5_MBI4499125.1:0.4743884387,(DndH.CTD.clade.5_WP_129893628.1:0.4912610577,(DndH.CTD.clade.5_MBX5455775.1:0.3712694746,(DndH.CTD.clade.5_WP_201359961.1:0.2462990192,DndH.CTD.clade.5_
WP_201380144.1:0.2162620155)95.6/100:0.0971922062)82.8/100:0.0682039094)96/100:0.1035838791)85.1/97:0.0536460074)90.4/25:0.1026345038,DndH.CTD.clade.5_NJM05280.1:0.3388726672)46.6/25:0.1673238234,DndH.CTD.clade.5_WP_135480204.1:0.4461815587)100/100:0.73
66270222)90/98:0.3390002060,((((((((orc-cdc6.asoc_WP_013417869.1:0.0000000000,orc-cdc6.asoc_WP_201719249.1:0.0000000000):0.0000010000,orc-cdc6.asoc_WP_037234259.1:0.0423416688)0/49:0.0000010000,orc-cdc6.asoc_WP_199502315.1:0.0000010000)75.1/95:0.003172955
3,orc-cdc6.asoc_WP_127077363.1:0.0119525185)0/78:0.0000020250,orc-cdc6.asoc_WP_215029027.1:0.0181396144)99.8/100:0.2022760961,((orc-cdc6.asoc_NJM34115.1:0.0342487643,orc-cdc6.asoc_WP_088345553.1:0.0767034419)77.6/100:0.0286472768,orc-cdc6.asoc_MBX2805379
.1:0.0510887761)100/100:0.2237938215)99.8/100:0.3664832216,orc-cdc6.asoc_WP_119061881.1:0.5360316803)93.6/100:0.2278175802,orc-cdc6.asoc_WP_201717167.1:0.7641511772)100/100:0.8998102259)82.5/99:0.2016600093)49.1/80:0.2051405104)99.2/100:0.5528580119)89.9/99:
0.2821235780,((((((D5-Sf3-asoc.HerA.FtsK_WP_173719683.1:0.8214694347,D5-Sf3-asoc.HerA.FtsK_HIV38416.1:0.5848176684)88.6/100:0.1500777228,((D5-Sf3-asoc.HerA.FtsK_WP_118145807.1:0.1848405472,D5-Sf3-asoc.HerA.FtsK_WP_195389302.1:0.2623651046)100/100:0.545636
5356,D5-Sf3-asoc.HerA.FtsK_WP_235416179.1:0.5002627438)95.7/100:0.1726343677)96.9/100:0.2060025172,((D5-Sf3-asoc.HerA.FtsK_MBE5892207.1:1.0255685829,D5-Sf3-asoc.HerA.FtsK_WP_119234009.1:0.6405907977)85.8/66:0.1244100195,((D5-Sf3-asoc.HerA.FtsK_MBS642329
0.1:0.9643610503,D5-Sf3-asoc.HerA.FtsK_WP_195464968.1:0.4578132960)99.4/100:0.3254080485,D5-Sf3-asoc.HerA.FtsK_EOS72040.1:0.6020118227)91.5/78:0.1042719830)36.5/67:0.0697628647)94/95:0.2086783619,D5-Sf3-asoc.HerA.FtsK_MCD8000722.1:0.8662062407)97.4/100:0.2
970497461,(D5-Sf3-asoc.HerA.FtsK_WP_100041384.1:0.2093740428,D5-Sf3-asoc.HerA.FtsK_MBS5073964.1:0.2244051067)100/100:0.9536967062)86.6/100:0.2344599995,(D5-Sf3-asoc.HerA.FtsK_MBQ9361784.1:0.9192682214,(D5-Sf3-asoc.HerA.FtsK_WP_186802651.1:0.7588672133,
(D5-Sf3-asoc.HerA.FtsK_HJB07420.1:0.3908931669,D5-Sf3-asoc.HerA.FtsK_MCD7906236.1:0.2183129514)100/100:0.6067593898)50.1/97:0.1493600089)99.9/100:0.6015206756)99.5/100:0.6824253138)73.9/99:0.1443364989,D5-Sf3-asoc.HerA.FtsK_WP_092453426.1:1.6800835874)93/
99:0.3109795948)92.7/92:0.1151471740,(((DndH.CTD.clade.1_WP_115113380.1:0.3254510266,((DndH.CTD.clade.1_WP_199790682.1:0.2942738558,DndH.CTD.clade.1_WP_204230793.1:0.1688100866)38.8/100:0.0574946844,(DndH.CTD.clade.1_PTH62584.1:0.1943789753,DndH.CTD.
clade.1_WP_115871599.1:0.1252871989)96.1/100:0.1091094188)94.4/100:0.1371008773)91.8/100:0.1590891837,DndH.CTD.clade.1_MCA9766490.1:0.3992915900)93.6/100:0.1213339564,(((DndH.CTD.clade.1_QAS30887.1:0.3231105951,(DndH.CTD.clade.1_WP_162897925.1:0.219585
0125,(DndH.CTD.clade.1_RXE75860.1:0.0812455187,DndH.CTD.clade.1_WP_056944045.1:0.1028534999)100/100:0.1705968655)65/100:0.0762238595)56.8/99:0.1261433133,DndH.CTD.clade.1_WP_056996767.1:0.2396670650)99.5/100:0.2312034253,(DndH.CTD.clade.1_WP_0354505
54.1:0.5613507886,DndH.CTD.clade.1_WP_164507458.1:0.5052904006)97.3/100:0.1965181518)98.1/100:0.1782983641)100/100:0.3456434179)23.7/66:0.0919215466)100/100:0.4133363049)92.8/100:0.1232177331);
