# Supplementary material for: Reappraisal of the DNA phosphorothioate modification machinery: uncovering neglected functional modalities and identification of new counter-invader defense systems
Source: Nucleic Acids Res. 2024 Jan 2;52(3):1005–26. doi: 10.1093/nar/gkad1213 (PMC10853773; doi:10.1093/nar/gkad1213)
Supplement: gkad1213_Supplemental_Files [file Supplementary_Dataset_1.pdf.pdf › Supplementary_Dataset_1.pdf.pdf]

Supplementary_Dataset_1_Index

1. Phyletic distribution and domain architecture of DndA…….…………..........…...2-19
2. Phyletic distribution and domain architecture of DndB……………………..........20-61
3. Phyletic distribution and domain architecture of DndC……………………........62-104
4. Phyletic distribution and domain architecture of DndD_ABC…………...…....105-155
5. Phyletic distribution and domain architecture of CxC_ABC………………......155-185
6. Phyletic distribution, domain architecture and gene neighborhoods of DndE....186-262
7. Phyletic distribution and domain architecture of HKD-DNase+SF2-Helicase…263-291
8. Phyletic distribution, domain architecture and gene neighborhoods of DndF…..292-322
9. Phyletic distribution and domain architecture of DndG…………………….....323-351
10. Phyletic distribution and domain architecture of DndH……………………........352-382
11. DndH false positives with only HerA/FtsK…………………...….......................383-403
12. DndH true hits from previous study……………………………….................…403-404
13. PbeABCD rectified gene neighborhoods……………………………..............405-409
14. HerA/FtsK capture system domain architecture and gene neighborhoods……....410-413

*All cluster files include protein accession IDs in the first column, followed by multiple columns mentioning various pieces of information related to each protein accession ID. The information are parsed in the following order : **Accession IDs**; **annotation/domain architecture**; **Gene neighborhoods** *(only present in DndE For DndABCDE system, DndF for DndFGH system and HerA/FtsK capture system cluster files)*; **Assembly IDs**; **Protein length**; **Phylogenetic distribution**; **Strain name**; **FASTA Defline** and **Species Taxid.**

This supplementary material is publicly available in text file format in online repository zenodo in the following link :

https://zenodo.org/records/10057384

Accession	Annotation	GCA	Length	Phyletic.spread	Strain.name	Defline	Species.Taxid
MBT4835061.1	cysteine-desulfurase	GCA_018663865.1	172	Archaea>DPANN_group>Candidatus_Woesearchaeota	Candidatus_Woesearchaeota_archaeon	aminotransferase-class-V-fold-PLP-dependent-enzyme-[Candidatus-Woesearchaeota-archaeon]	2026803
RJQ15771.1	cysteine-desulfurase	GCA_003599145.1	383	Archaea>DPANN_group>Candidatus_Woesearchaeota	Candidatus_Woesearchaeota_archaeon	aminotransferase-class-V-fold-PLP-dependent-enzyme-[Candidatus-Woesearchaeota-archaeon]	2026803
MBT3813912.1	cysteine-desulfurase	GCA_018654495.1	372	Archaea>DPANN_group>Candidatus_Woesearchaeota	Candidatus_Woesearchaeota_archaeon	cysteine-desulfurase-[Candidatus-Woesearchaeota-archaeon]	2026803
MBT4110880.1	cysteine-desulfurase	GCA_018650945.1	372	Archaea>DPANN_group>Candidatus_Woesearchaeota	Candidatus_Woesearchaeota_archaeon	cysteine-desulfurase-[Candidatus-Woesearchaeota-archaeon]	2026803
MBS7256898.1	cysteine-desulfurase	GCA_018385235.1	382	Archaea>Euryarchaeota>Methanomada_group>Methanobacteria>Methanobacteriales	Methanobrevibacter_sp.	aminotransferase-class-V-fold-PLP-dependent-enzyme-[Methanobrevibacter-sp.]	66852
WP_181505017.1	cysteine-desulfurase	GCF_013761225.1	374	Archaea>Euryarchaeota>Methanomada_group>Methanococci>Methanococcales	Methanococcus_maripaludis	cysteine-desulfurase-[Methanococcus-maripaludis]	39152
WP_069449424.1	cysteine-desulfurase	GCF_001723155.1	393	Archaea>Euryarchaeota>Stenosarchaea_group>Halobacteria>Halobacteriales	Haladaptatus_sp._W1	cysteine-desulfurase-[Haladaptatus-sp.-W1]	1897478
QCC51444.1	cysteine-desulfurase	GCA_004799665.1	399	Archaea>Euryarchaeota>Stenosarchaea_group>Halobacteria>Halobacteriales	Halapricum_salinum	cysteine-desulfurase-[Halapricum-salinum]	1457250
WP_049994945.1	cysteine-desulfurase	GCF_000755225.1	399	Archaea>Euryarchaeota>Stenosarchaea_group>Halobacteria>Halobacteriales	Halapricum_salinum	cysteine-desulfurase-[Halapricum-salinum]	1457250
WP_124956056.1	cysteine-desulfurase	GCF_003862495.1	392	Archaea>Euryarchaeota>Stenosarchaea_group>Halobacteria>Halobacteriales	Halocatena_pleomorpha	cysteine-desulfurase-[Halomarina-oriensis]	1785090
WP_170094356.1	cysteine-desulfurase	GCF_012726095.1	395	Archaea>Euryarchaeota>Stenosarchaea_group>Halobacteria>Halobacteriales	Halomicrobium_mukohataei	cysteine-desulfurase-[Halomicrobium-mukohataei]	57705
WP_227261701.1	cysteine-desulfurase	GCF_020614395.1	392	Archaea>Euryarchaeota>Stenosarchaea_group>Halobacteria>Halobacteriales	Salarchaeum_japonicum	cysteine-desulfurase-[Salarchaeum-japonicum]	555573
WP_179268602.1	cysteine-desulfurase	GCF_013402875.1	394	Archaea>Euryarchaeota>Stenosarchaea_group>Halobacteria>Haloferacales	Halobaculum_salinum	cysteine-desulfurase-[Halobaculum-salinum]	2743089
WP_103992758.1	cysteine-desulfurase	GCF_004799685.1	393	Archaea>Euryarchaeota>Stenosarchaea_group>Halobacteria>Haloferacales	Halobellus_limi	cysteine-desulfurase-[Halobellus-limi]	699433
WP_185191438.1	cysteine-desulfurase	GCF_014212355.1	399	Archaea>Euryarchaeota>Stenosarchaea_group>Halobacteria>Haloferacales	Halobellus_ruber	cysteine-desulfurase-[Halobellus-ruber]	2761102
WP_089827595.1	cysteine-desulfurase	GCF_900110465.1	392	Archaea>Euryarchaeota>Stenosarchaea_group>Halobacteria>Haloferacales	Halogranum_amylolyticum	cysteine-desulfurase-[Halogranum-amylolyticum]	660520
RMB12942.1	cysteine-desulfurase	GCA_003688715.1	392	Archaea>Euryarchaeota>Stenosarchaea_group>Halobacteria>Haloferacales	Haloplanus_aerogenes	cysteine-desulfurase-[Haloplanus-aerogenes]	660522
WP_121921660.1	cysteine-desulfurase	GCF_003856835.1	392	Archaea>Euryarchaeota>Stenosarchaea_group>Halobacteria>Haloferacales	Haloplanus_aerogenes	cysteine-desulfurase-[Haloplanus-aerogenes]	660522
WP_004595343.1	cysteine-desulfurase	GCF_000337055.1	380	Archaea>Euryarchaeota>Stenosarchaea_group>Halobacteria>Haloferacales	Halorubrum_distributum_group	MULTISPECIES:-aminotransferase-class-V-fold-PLP-dependent-enzyme-[Halorubrum-distributum-group]	2703941
WP_144922265.1	cysteine-desulfurase	GCF_007671685.1	393	Archaea>Euryarchaeota>Stenosarchaea_group>Halobacteria>Haloferacales	Halorubrum_salsamenti	cysteine-desulfurase-[Halorubrum-salsamenti]	2583990
WP_123619674.1	cysteine-desulfurase	GCF_003781945.1	392	Archaea>Euryarchaeota>Stenosarchaea_group>Halobacteria>Haloferacales	Halorubrum_sp._CSM-61	cysteine-desulfurase-[Halorubrum-sp.-CSM-61]	2485838
TKX87927.1	cysteine-desulfurase	GCA_005435045.1	393	Archaea>Euryarchaeota>Stenosarchaea_group>Halobacteria>Haloferacales	Halorubrum_sp._SS5	cysteine-desulfurase-[Halorubrum-sp.-SS5]	2518118
WP_096396434.1	cysteine-desulfurase	GCF_002355655.1	392	Archaea>Euryarchaeota>Stenosarchaea_group>Halobacteria	Haloferacales	MULTISPECIES:-cysteine-desulfurase-[Haloferacales]	1644055
WP_207290817.1	cysteine-desulfurase	GCF_017352155.2	400	Archaea>Euryarchaeota>Stenosarchaea_group>Halobacteria>Natrialbales	Haloterrigena_alkaliphila	aminotransferase-class-V-fold-PLP-dependent-enzyme-[Haloterrigena-sp.-KZCA68]	2816475
WP_084158228.1	cysteine-desulfurase	GCF_000710605.1	400	Archaea>Euryarchaeota>Stenosarchaea_group>Halobacteria>Natrialbales	Haloterrigena_jeotgali	cysteine-desulfurase-[Haloterrigena-jeotgali]	413811
WP_006065546.1	cysteine-desulfurase	GCF_000337715.1	400	Archaea>Euryarchaeota>Stenosarchaea_group>Halobacteria>Natrialbales	Natronorubrum_bangense	cysteine-desulfurase-[Natronorubrum-bangense]	61858
ELY49720.1	cysteine-desulfurase	GCA_000337715.1	400	Archaea>Euryarchaeota>Stenosarchaea_group>Halobacteria>Natrialbales	Natronorubrum_bangense_JCM_10635	cysteine-desulfurase-[Natronorubrum-bangense-JCM-10635]	1227500
WP_160067771.1	cysteine-desulfurase	GCF_009834785.1	393	Archaea>Euryarchaeota>Stenosarchaea_group>Halobacteria>Natrialbales	Natronorubrum_halalkaliphilum	cysteine-desulfurase-[Natronorubrum-halalkaliphilum]	2691917
WP_141465255.1	cysteine-desulfurase	GCF_006543045.1	400	Archaea>Euryarchaeota>Stenosarchaea_group>Halobacteria>Natrialbales	Salinadaptatus_halalkaliphilus	cysteine-desulfurase-[Salinadaptatus-halalkaliphilus]	2419781
PKL63562.1	cysteine-desulfurase	GCA_002839645.1	386	Archaea>Euryarchaeota>Stenosarchaea_group>Methanomicrobia>Methanomicrobiales	Methanomicrobiales_archaeon_HGW-Methanomicrobiales-2	IscS-subfamily-cysteine-desulfurase-[Methanomicrobiales-archaeon-HGW-Methanomicrobiales-2]	2013818
PKL58360.1	cysteine-desulfurase	GCA_002839585.1	237	Archaea>Euryarchaeota>Stenosarchaea_group>Methanomicrobia>Methanomicrobiales	Methanomicrobiales_archaeon_HGW-Methanomicrobiales-5	IscS-subfamily-cysteine-desulfurase,-partial-[Methanomicrobiales-archaeon-HGW-Methanomicrobiales-5]	2013821
PKL58850.1	cysteine-desulfurase	GCA_002839585.1	149	Archaea>Euryarchaeota>Stenosarchaea_group>Methanomicrobia>Methanomicrobiales	Methanomicrobiales_archaeon_HGW-Methanomicrobiales-5	IscS-subfamily-cysteine-desulfurase,-partial-[Methanomicrobiales-archaeon-HGW-Methanomicrobiales-5]	2013821
WP_232385616.1	cysteine-desulfurase	GCF_000017625.1	381	Archaea>Euryarchaeota>Stenosarchaea_group>Methanomicrobia>Methanomicrobiales	Methanoregula_boonei	cysteine-desulfurase-[Methanoregula-boonei]	358766
OPX62562.1	cysteine-desulfurase	GCA_002067035.1	390	Archaea>Euryarchaeota>Stenosarchaea_group>Methanomicrobia>Methanomicrobiales	Methanoregula_sp._PtaB.Bin085	putative-cysteine-desulfurase-2-[Methanoregula-sp.-PtaB.Bin085]	1811680
WP_236610457.1	cysteine-desulfurase	GCF_000021965.1	379	Archaea>Euryarchaeota>Stenosarchaea_group>Methanomicrobia>Methanomicrobiales	Methanosphaerula_palustris	cysteine-desulfurase-[Methanosphaerula-palustris]	475088
ACL16702.1	cysteine-desulfurase	GCA_000021965.1	387	Archaea>Euryarchaeota>Stenosarchaea_group>Methanomicrobia>Methanomicrobiales	Methanosphaerula_palustris_E1-9c	aminotransferase-class-V-[Methanosphaerula-palustris-E1-9c]	521011
MCD4798389.1	cysteine-desulfurase	GCA_021108115.1	379	Archaea>Euryarchaeota>Stenosarchaea_group>Methanomicrobia>Methanosarcinales	Methanosarcinales_archaeon	cysteine-desulfurase-[Methanosarcinales-archaeon]	2250255
RLI44268.1	cysteine-desulfurase	GCA_003662715.1	386	Archaea>TACK_group>Candidatus_Bathyarchaeota>unclassified_Candidatus_Bathyarchaeota	Candidatus_Bathyarchaeota_archaeon	IscS-subfamily-cysteine-desulfurase-[Candidatus-Bathyarchaeota-archaeon]	2026714
AIF05629.1	cysteine-desulfurase	-	387	Archaea>TACK_group>Thaumarchaeota>environmental_samples	uncultured_marine_thaumarchaeote_KM3_186_C08	aminotransferase-class-V-(iscS,-NFS1)-[uncultured-marine-thaumarchaeote-KM3_186_C08]	1456070
AIF10948.1	cysteine-desulfurase	-	387	Archaea>TACK_group>Thaumarchaeota>environmental_samples	uncultured_marine_thaumarchaeote_KM3_47_F06	aminotransferase-class-V-(iscS,-NFS1)-[uncultured-marine-thaumarchaeote-KM3_47_F06]	1456168
WP_086907923.1	cysteine-desulfurase	GCF_002156965.1	385	Archaea>TACK_group>Thaumarchaeota>Nitrosopumilales>Nitrosopumilaceae	Candidatus_Nitrosomarinus_catalina	cysteine-desulfurase-[Candidatus-Nitrosomarinus-catalina]	1898749
ARS64879.1	cysteine-desulfurase	GCA_002156965.1	385	Archaea>TACK_group>Thaumarchaeota>Nitrosopumilales>Nitrosopumilaceae	Candidatus_Nitrosomarinus_catalina	putative-cysteine-desulfurase-2-[Candidatus-Nitrosomarinus-catalina]	1898749
EIJ65021.1	cysteine-desulfurase	GCA_000242875.3	387	Archaea>TACK_group>Thaumarchaeota>Nitrosopumilales>Nitrosopumilaceae	Candidatus_Nitrosopumilus_salaria_BD31	aminotransferase,-class-V-[Candidatus-Nitrosopumilus-salaria-BD31]	859350
WP_100222208.1	cysteine-desulfurase	GCF_000242875.2	384	Archaea>TACK_group>Thaumarchaeota>Nitrosopumilales>Nitrosopumilaceae	Candidatus_Nitrosopumilus_salaria	cysteine-desulfurase-[Candidatus-Nitrosopumilus-salaria]	1170320
PHY08229.1	cysteine-desulfurase	GCA_002737445.1	317	Archaea>TACK_group>Thaumarchaeota>Nitrosopumilales>Nitrosopumilaceae	Nitrosarchaeum_sp.	IscS-subfamily-cysteine-desulfurase,-partial-[Nitrosarchaeum-sp.]	2026886
PHY08250.1	cysteine-desulfurase	GCA_002737445.1	260	Archaea>TACK_group>Thaumarchaeota>Nitrosopumilales>Nitrosopumilaceae	Nitrosarchaeum_sp.	IscS-subfamily-cysteine-desulfurase,-partial-[Nitrosarchaeum-sp.]	2026886
VDK13105.1	cysteine-desulfurase	-	390	Archaea>TACK_group>Thaumarchaeota>Nitrosopumilales>Nitrosopumilaceae	Nitrosopumilaceae_archaeon	Cysteine-desulfurase-IscS-[Nitrosopumilaceae-archaeon]	2202732
MCE2508331.1	cysteine-desulfurase	GCA_021296165.1	379	Archaea>TACK_group>Thaumarchaeota>Nitrosopumilales>Nitrosopumilaceae	Nitrosopumilaceae_archaeon	cysteine-desulfurase-[Nitrosopumilaceae-archaeon]	2202732
WP_182133246.1	cysteine-desulfurase	GCF_014078545.1	387	Archaea>TACK_group>Thaumarchaeota>Nitrosopumilales>Nitrosopumilaceae	Nitrosopumilus_sp._b1	cysteine-desulfurase-[Nitrosopumilus-sp.-b1]	2109907
KAF6243628.1	cysteine-desulfurase	GCA_014078545.1	387	Archaea>TACK_group>Thaumarchaeota>Nitrosopumilales>Nitrosopumilaceae	Nitrosopumilus_sp._b1	IscS-subfamily-cysteine-desulfurase-[Nitrosopumilus-sp.-b1]	2109907
MBC8502142.1	cysteine-desulfurase	GCA_014384445.1	253	Archaea>TACK_group>Thaumarchaeota>Nitrosopumilales>Nitrosopumilaceae	Nitrosopumilus_sp.	cysteine-desulfurase-[Nitrosopumilus-sp.]	2024843
MBL7001902.1	cysteine-desulfurase	GCA_016784625.1	387	Archaea>TACK_group>Thaumarchaeota>Nitrosopumilales>Nitrosopumilaceae	Nitrosopumilus_sp.	cysteine-desulfurase-[Nitrosopumilus-sp.]	2024843
NRA06463.1	cysteine-desulfurase	GCA_013215285.1	387	Archaea>TACK_group>Thaumarchaeota>Nitrosopumilales>Nitrosopumilaceae	Nitrosopumilus_sp.	cysteine-desulfurase-[Nitrosopumilus-sp.]	2024843
HIO32169.1	cysteine-desulfurase	GCA_015663895.1	95	Archaea>TACK_group>Thaumarchaeota>Thaumarchaeota_incertae_sedis>Candidatus_Nitrosopelagicus	Candidatus_Nitrosopelagicus_sp.	aminotransferase-class-V-fold-PLP-dependent-enzyme-[Candidatus-Nitrosopelagicus-sp.]	2026808
MBT4326682.1	cysteine-desulfurase	GCA_018652445.1	120	Archaea>TACK_group>Thaumarchaeota>Thaumarchaeota_incertae_sedis>Candidatus_Nitrosopelagicus	Candidatus_Nitrosopelagicus_sp.	aminotransferase-class-V-fold-PLP-dependent-enzyme-[Candidatus-Nitrosopelagicus-sp.]	2026808
MBT4327996.1	cysteine-desulfurase	GCA_018652445.1	103	Archaea>TACK_group>Thaumarchaeota>Thaumarchaeota_incertae_sedis>Candidatus_Nitrosopelagicus	Candidatus_Nitrosopelagicus_sp.	aminotransferase-class-V-fold-PLP-dependent-enzyme-[Candidatus-Nitrosopelagicus-sp.]	2026808
MBT7252355.1	cysteine-desulfurase	GCA_018691945.1	106	Archaea>TACK_group>Thaumarchaeota>Thaumarchaeota_incertae_sedis>Candidatus_Nitrosopelagicus	Candidatus_Nitrosopelagicus_sp.	aminotransferase-class-V-fold-PLP-dependent-enzyme-[Candidatus-Nitrosopelagicus-sp.]	2026808
MBC8250878.1	cysteine-desulfurase	GCA_014381285.1	343	Archaea>TACK_group>Thaumarchaeota>Thaumarchaeota_incertae_sedis>Candidatus_Nitrosopelagicus	Candidatus_Nitrosopelagicus_sp.	cysteine-desulfurase-[Candidatus-Nitrosopelagicus-sp.]	2026808
MBT3761273.1	cysteine-desulfurase	GCA_018654855.1	387	Archaea>TACK_group>Thaumarchaeota>Thaumarchaeota_incertae_sedis>Candidatus_Nitrosopelagicus	Candidatus_Nitrosopelagicus_sp.	cysteine-desulfurase-[Candidatus-Nitrosopelagicus-sp.]	2026808
MBT4325603.1	cysteine-desulfurase	GCA_018652445.1	251	Archaea>TACK_group>Thaumarchaeota>Thaumarchaeota_incertae_sedis>Candidatus_Nitrosopelagicus	Candidatus_Nitrosopelagicus_sp.	cysteine-desulfurase-[Candidatus-Nitrosopelagicus-sp.]	2026808
MBT4325752.1	cysteine-desulfurase	GCA_018652445.1	329	Archaea>TACK_group>Thaumarchaeota>Thaumarchaeota_incertae_sedis>Candidatus_Nitrosopelagicus	Candidatus_Nitrosopelagicus_sp.	cysteine-desulfurase-[Candidatus-Nitrosopelagicus-sp.]	2026808
MBT4326058.1	cysteine-desulfurase	GCA_018652445.1	325	Archaea>TACK_group>Thaumarchaeota>Thaumarchaeota_incertae_sedis>Candidatus_Nitrosopelagicus	Candidatus_Nitrosopelagicus_sp.	cysteine-desulfurase-[Candidatus-Nitrosopelagicus-sp.]	2026808
MBT4328179.1	cysteine-desulfurase	GCA_018652445.1	386	Archaea>TACK_group>Thaumarchaeota>Thaumarchaeota_incertae_sedis>Candidatus_Nitrosopelagicus	Candidatus_Nitrosopelagicus_sp.	cysteine-desulfurase-[Candidatus-Nitrosopelagicus-sp.]	2026808
MBT7252297.1	cysteine-desulfurase	GCA_018691945.1	387	Archaea>TACK_group>Thaumarchaeota>Thaumarchaeota_incertae_sedis>Candidatus_Nitrosopelagicus	Candidatus_Nitrosopelagicus_sp.	cysteine-desulfurase-[Candidatus-Nitrosopelagicus-sp.]	2026808
MBT4176043.1	cysteine-desulfurase	GCA_018651685.1	87	Archaea>TACK_group>Thaumarchaeota>unclassified_Thaumarchaeota	Thaumarchaeota_archaeon	aminotransferase-class-V-fold-PLP-dependent-enzyme-[Thaumarchaeota-archaeon]	2026795
MBI1658933.1	cysteine-desulfurase	GCA_016126025.1	181	Archaea>TACK_group>Thaumarchaeota>unclassified_Thaumarchaeota	Thaumarchaeota_archaeon	aminotransferase-class-V-fold-PLP-dependent-enzyme-[Thaumarchaeota-archaeon]	2026795
MBT6646238.1	cysteine-desulfurase	GCA_018697615.1	185	Archaea>TACK_group>Thaumarchaeota>unclassified_Thaumarchaeota	Thaumarchaeota_archaeon	aminotransferase-class-V-fold-PLP-dependent-enzyme-[Thaumarchaeota-archaeon]	2026795
MBT6646714.1	cysteine-desulfurase	GCA_018697615.1	144	Archaea>TACK_group>Thaumarchaeota>unclassified_Thaumarchaeota	Thaumarchaeota_archaeon	aminotransferase-class-V-fold-PLP-dependent-enzyme-[Thaumarchaeota-archaeon]	2026795
MBT7360181.1	cysteine-desulfurase	GCA_018671875.1	389	Archaea>TACK_group>Thaumarchaeota>unclassified_Thaumarchaeota	Thaumarchaeota_archaeon	cysteine-desulfurase-[Thaumarchaeota-archaeon]	2026795
RZD40209.1	cysteine-desulfurase	GCA_004195415.1	127	Archaea>TACK_group>Thaumarchaeota>unclassified_Thaumarchaeota	Thaumarchaeota_archaeon	IscS-subfamily-cysteine-desulfurase,-partial-[Thaumarchaeota-archaeon]	2026795
RNJ74851.1	cysteine-desulfurase	GCA_003724275.1	379	Archaea>TACK_group>Thaumarchaeota>unclassified_Thaumarchaeota	Thaumarchaeota_archaeon_S13	cysteine-desulfurase-[Thaumarchaeota-archaeon-S13]	2478557
MBT4352519.1	cysteine-desulfurase	GCA_018653265.1	379	Archaea>unclassified_Archaea	archaeon	cysteine-desulfurase-[archaeon]	1906665
MBT4648400.1	cysteine-desulfurase	GCA_018657865.1	379	Archaea>unclassified_Archaea	archaeon	cysteine-desulfurase-[archaeon]	1906665
ACO34384.1	cysteine-desulfurase	GCA_000022565.1	378	Bacteria>Acidobacteria>Acidobacteriia>Acidobacteriales>Acidobacteriaceae	Acidobacterium_capsulatum_ATCC_51196	cysteine-desulfurase-[Acidobacterium-capsulatum-ATCC-51196]	240015
WP_015898230.1	cysteine-desulfurase	GCF_000022565.1	378	Bacteria>Acidobacteria>Acidobacteriia>Acidobacteriales>Acidobacteriaceae	Acidobacterium_capsulatum	cysteine-desulfurase-DndA-[Acidobacterium-capsulatum]	33075
PYP85881.1	cysteine-desulfurase	GCA_003222245.1	390	Bacteria>Acidobacteria>Blastocatellia>unclassified_Blastocatellia	Blastocatellia_bacterium_AA13	cysteine-desulfurase-NifS-[Blastocatellia-bacterium-AA13]	2169413
MYB19206.1	cysteine-desulfurase	GCA_009839125.1	400	Bacteria>Acidobacteria>Holophagae>Holophagales>unclassified_Holophagales	Holophagales_bacterium	cysteine-desulfurase-[Holophagales-bacterium]	2478486
MBM3740840.1	cysteine-desulfurase	GCA_016871015.1	389	Bacteria>Acidobacteria>unclassified_Acidobacteria	Acidobacteria_bacterium	cysteine-desulfurase-[Acidobacteria-bacterium]	1978231
MXY25142.1	cysteine-desulfurase	GCA_009838525.1	398	Bacteria>Acidobacteria>unclassified_Acidobacteria	Acidobacteria_bacterium	cysteine-desulfurase-[Acidobacteria-bacterium]	1978231
MXW44114.1	cysteine-desulfurase	GCA_009836355.1	386	Bacteria>Bacteria_incertae_sedis>Bacteria_candidate_phyla>Candidatus_Dadabacteria	Candidatus_Dadabacteria_bacterium	cysteine-desulfurase-[Candidatus-Dadabacteria-bacterium]	2080303
MBN1969155.1	cysteine-desulfurase	GCA_016931755.1	385	Bacteria>Bacteria_incertae_sedis>Bacteria_candidate_phyla>Candidatus_Delongbacteria	Candidatus_Delongbacteria_bacterium	cysteine-desulfurase-[Candidatus-Delongbacteria-bacterium]	2044594
MBN2836540.1	cysteine-desulfurase	GCA_016938275.1	371	Bacteria>Bacteria_incertae_sedis>Bacteria_candidate_phyla>Candidatus_Delongbacteria	Candidatus_Delongbacteria_bacterium	cysteine-desulfurase-[Candidatus-Delongbacteria-bacterium]	2044594
MBR6122298.1	cysteine-desulfurase	GCA_017541625.1	386	Bacteria>Bacteria_incertae_sedis>Bacteria_candidate_phyla>Candidatus_Saccharibacteria>unclassified_Candidatus_Saccharibacteria	Candidatus_Saccharibacteria_bacterium	aminotransferase-class-V-fold-PLP-dependent-enzyme-[Candidatus-Saccharibacteria-bacterium]	2026720
MBR6122370.1	cysteine-desulfurase	GCA_017541625.1	384	Bacteria>Bacteria_incertae_sedis>Bacteria_candidate_phyla>Candidatus_Saccharibacteria>unclassified_Candidatus_Saccharibacteria	Candidatus_Saccharibacteria_bacterium	aminotransferase-class-V-fold-PLP-dependent-enzyme-[Candidatus-Saccharibacteria-bacterium]	2026720
RJQ25129.1	cysteine-desulfurase	GCA_003599675.1	382	Bacteria>Bacteria_incertae_sedis>Bacteria_candidate_phyla>Patescibacteria_group>Parcubacteria_group	Candidatus_Parcubacteria_bacterium	aminotransferase-class-V-fold-PLP-dependent-enzyme-[Candidatus-Parcubacteria-bacterium]	2762014
MBU2545205.1	cysteine-desulfurase	GCA_018830125.1	380	Bacteria>Bacteria_incertae_sedis>Bacteria_candidate_phyla>Patescibacteria_group>unclassified_Patescibacteria_group	Patescibacteria_group_bacterium	cysteine-desulfurase-[Patescibacteria-group-bacterium]	2052139
MBE0567698.1	cysteine-desulfurase	GCA_014859985.1	379	Bacteria>Candidatus_Krumholzibacteriota>Candidatus_Krumholzibacteria>unclassified_Candidatus_Krumholzibacteria	Candidatus_Krumholzibacteria_bacterium	aminotransferase-class-V-fold-PLP-dependent-enzyme-[Krumholzibacteria-bacterium]	2774603
WP_118838215.1	cysteine-desulfurase	GCF_003491185.1	392	Bacteria>FCB_group>Bacteroidetes/Chlorobi_group>Bacteroidetes>Bacteroidetes_Order_II._Incertae_sedis	Salinibacter_ruber	cysteine-desulfurase-[Salinibacter-ruber]	146919
NLA94436.1	cysteine-desulfurase	GCA_012511195.1	485	Bacteria>FCB_group>Bacteroidetes/Chlorobi_group>Bacteroidetes>Bacteroidia	Bacteroidales_bacterium	aminotransferase-class-I/II-fold-pyridoxal-phosphate-dependent-enzyme-[Bacteroidales-bacterium]	2030927
MCD4666142.1	cysteine-desulfurase	GCA_021107235.1	371	Bacteria>FCB_group>Bacteroidetes/Chlorobi_group>Bacteroidetes>Bacteroidia	Bacteroidales_bacterium	cysteine-desulfurase,-partial-[Bacteroidales-bacterium]	2030927
HBG69691.1	cysteine-desulfurase	GCA_003499605.1	384	Bacteria>FCB_group>Bacteroidetes/Chlorobi_group>Bacteroidetes>Bacteroidia	Bacteroidales_bacterium	IscS-subfamily-cysteine-desulfurase-[Bacteroidales-bacterium]	2030927
MCF8254401.1	cysteine-desulfurase	GCA_021739685.1	382	Bacteria>FCB_group>Bacteroidetes/Chlorobi_group>Bacteroidetes>Bacteroidia	Bacteroidia_bacterium	cysteine-desulfurase-[Bacteroidia-bacterium]	2044936
WP_062124568.1	cysteine-desulfurase	GCF_000974365.1	382	Bacteria>FCB_group>Bacteroidetes/Chlorobi_group>Bacteroidetes>Bacteroidia	Geofilum_rubicundum	cysteine-desulfurase-[Geofilum-rubicundum]	472113
GAO29928.1	cysteine-desulfurase	GCA_000974365.1	382	Bacteria>FCB_group>Bacteroidetes/Chlorobi_group>Bacteroidetes>Bacteroidia	Geofilum_rubicundum_JCM_15548	cysteine-desulfurase-[Geofilum-rubicundum-JCM-15548]	1236989
MBI9062571.1	cysteine-desulfurase	GCA_016342595.1	377	Bacteria>FCB_group>Bacteroidetes/Chlorobi_group>Bacteroidetes>Bacteroidia	Marinilabiliaceae_bacterium	cysteine-desulfurase-[Marinilabiliaceae-bacterium]	2053574
MVZ89005.1	cysteine-desulfurase	GCA_009768845.1	394	Bacteria>FCB_group>Bacteroidetes/Chlorobi_group>Bacteroidetes>Bacteroidia	Microbacter_sp._ANSKLAB05	cysteine-desulfurase-DndA-[Microbacter-sp.-ANSKLAB05]	2684407
MCC8174477.1	cysteine-desulfurase	GCA_020860925.1	384	Bacteria>FCB_group>Bacteroidetes/Chlorobi_group>Bacteroidetes>Bacteroidia	Odoribacter_sp.	cysteine-desulfurase-[Odoribacter-sp.]	1965233
MBF1078918.1	cysteine-desulfurase	GCA_015256965.1	375	Bacteria>FCB_group>Bacteroidetes/Chlorobi_group>Bacteroidetes>Bacteroidia	Prevotellaceae_bacterium	aminotransferase-class-V-fold-PLP-dependent-enzyme-[Prevotellaceae-bacterium]	2049047
MBQ2673645.1	cysteine-desulfurase	GCA_017406665.1	398	Bacteria>FCB_group>Bacteroidetes/Chlorobi_group>Bacteroidetes>Bacteroidia	Prevotella_sp.	cysteine-desulfurase-[Prevotella-sp.]	59823
MBP7508522.1	cysteine-desulfurase	GCA_017998095.1	385	Bacteria>FCB_group>Bacteroidetes/Chlorobi_group>Bacteroidetes>Bacteroidia	Prolixibacteraceae_bacterium	aminotransferase-class-V-fold-PLP-dependent-enzyme-[Prolixibacteraceae-bacterium]	2053594
MBP7508677.1	cysteine-desulfurase	GCA_017998095.1	382	Bacteria>FCB_group>Bacteroidetes/Chlorobi_group>Bacteroidetes>Bacteroidia	Prolixibacteraceae_bacterium	aminotransferase-class-V-fold-PLP-dependent-enzyme-[Prolixibacteraceae-bacterium]	2053594
MBP7508127.1	cysteine-desulfurase	GCA_017998095.1	388	Bacteria>FCB_group>Bacteroidetes/Chlorobi_group>Bacteroidetes>Bacteroidia	Prolixibacteraceae_bacterium	cysteine-desulfurase-[Prolixibacteraceae-bacterium]	2053594
WP_028903776.1	cysteine-desulfurase	GCF_000619985.1	355	Bacteria>FCB_group>Bacteroidetes/Chlorobi_group>Bacteroidetes>Bacteroidia	unclassified_Prevotella	MULTISPECIES:-3-phosphoserine/phosphohydroxythreonine-transaminase-[unclassified-Prevotella]	2638335
WP_078669524.1	cysteine-desulfurase	GCF_900167135.1	391	Bacteria>FCB_group>Bacteroidetes/Chlorobi_group>Bacteroidetes>Chitinophagia	Chitinophaga_eiseniae	cysteine-desulfurase-[Chitinophaga-eiseniae]	634771
MBX7224973.1	cysteine-desulfurase	GCA_019694955.1	381	Bacteria>FCB_group>Bacteroidetes/Chlorobi_group>Bacteroidetes>Chitinophagia	Chitinophagales_bacterium	cysteine-desulfurase-[Chitinophagales-bacterium]	2448779
MCB9257086.1	cysteine-desulfurase	GCA_020635975.1	399	Bacteria>FCB_group>Bacteroidetes/Chlorobi_group>Bacteroidetes>Chitinophagia	Chitinophagales_bacterium	cysteine-desulfurase-[Chitinophagales-bacterium]	2448779
MBL7682035.1	cysteine-desulfurase	GCA_016786615.1	394	Bacteria>FCB_group>Bacteroidetes/Chlorobi_group>Bacteroidetes>Chitinophagia	Flavipsychrobacter_sp.	cysteine-desulfurase-[Flavipsychrobacter-sp.]	2307286
WP_090657728.1	cysteine-desulfurase	GCF_900115755.1	393	Bacteria>FCB_group>Bacteroidetes/Chlorobi_group>Bacteroidetes>Chitinophagia	Parafilimonas_terrae	cysteine-desulfurase-[Parafilimonas-terrae]	1465490
WP_026765134.1	cysteine-desulfurase	GCF_000511175.1	381	Bacteria>FCB_group>Bacteroidetes/Chlorobi_group>Bacteroidetes>Chitinophagia	Sediminibacterium_salmoneum	cysteine-desulfurase-[Sediminibacterium-salmoneum]	426421
MCE2974198.1	cysteine-desulfurase	GCA_021298755.1	385	Bacteria>FCB_group>Bacteroidetes/Chlorobi_group>Bacteroidetes>Chitinophagia	Sediminibacterium_sp.	cysteine-desulfurase-[Sediminibacterium-sp.]	1917865
WP_158531152.1	cysteine-desulfurase	GCF_003254055.1	499	Bacteria>FCB_group>Bacteroidetes/Chlorobi_group>Bacteroidetes>Cytophagia	Algoriphagus_chordae	cysteine-desulfurase-[Algoriphagus-chordae]	237019
WP_111672690.1	cysteine-desulfurase	GCF_003259255.1	391	Bacteria>FCB_group>Bacteroidetes/Chlorobi_group>Bacteroidetes>Cytophagia	Algoriphagus_litoralis	aminotransferase-class-V-fold-PLP-dependent-enzyme-[Algoriphagus-litoralis]	2202829
WP_113923495.1	cysteine-desulfurase	GCF_003316845.1	377	Bacteria>FCB_group>Bacteroidetes/Chlorobi_group>Bacteroidetes>Cytophagia	Aquiflexum_aquatile	cysteine-desulfurase-[Aquiflexum-aquatile]	2249427
WP_109745124.1	cysteine-desulfurase	GCF_003148625.1	380	Bacteria>FCB_group>Bacteroidetes/Chlorobi_group>Bacteroidetes>Cytophagia	Arcicella_aurantiaca	cysteine-desulfurase-[Arcicella-aurantiaca]	591202
WP_132121418.1	cysteine-desulfurase	GCF_004348825.1	378	Bacteria>FCB_group>Bacteroidetes/Chlorobi_group>Bacteroidetes>Cytophagia	Arundinibacter_roseus	cysteine-desulfurase-[Arundinibacter-roseus]	2070510
WP_014771600.1	cysteine-desulfurase	GCF_000265405.1	388	Bacteria>FCB_group>Bacteroidetes/Chlorobi_group>Bacteroidetes>Cytophagia	Belliella_baltica	cysteine-desulfurase-[Belliella-baltica]	232259
WP_014797642.1	cysteine-desulfurase	GCF_000265505.1	148	Bacteria>FCB_group>Bacteroidetes/Chlorobi_group>Bacteroidetes>Cytophagia	Bernardetia_litoralis	SufE-family-protein-[Bernardetia-litoralis]	999
PGH40555.1	cysteine-desulfurase	GCA_002573685.1	385	Bacteria>FCB_group>Bacteroidetes/Chlorobi_group>Bacteroidetes>Cytophagia	Candidatus_Nephrothrix_sp._EaCA	IscS-subfamily-cysteine-desulfurase-[Candidatus-Nephrothrix-sp.-EaCA]	2044729
WP_119755247.1	cysteine-desulfurase	GCF_003589925.1	383	Bacteria>FCB_group>Bacteroidetes/Chlorobi_group>Bacteroidetes>Cytophagia	Chryseolinea_soli	cysteine-desulfurase-[Chryseolinea-soli]	2321403
WP_127123865.1	cysteine-desulfurase	GCF_003990915.1	387	Bacteria>FCB_group>Bacteroidetes/Chlorobi_group>Bacteroidetes>Cytophagia	Chryseotalea_sanaruensis	cysteine-desulfurase-[Chryseotalea-sanaruensis]	2482724
MBX7090392.1	cysteine-desulfurase	GCA_019694875.1	386	Bacteria>FCB_group>Bacteroidetes/Chlorobi_group>Bacteroidetes>Cytophagia	Cyclobacteriaceae_bacterium	cysteine-desulfurase-[Cyclobacteriaceae-bacterium]	2099675
NJN26648.1	cysteine-desulfurase	GCA_012033595.1	393	Bacteria>FCB_group>Bacteroidetes/Chlorobi_group>Bacteroidetes>Cytophagia	Cyclobacteriaceae_bacterium	cysteine-desulfurase-[Cyclobacteriaceae-bacterium]	2099675
WP_166144214.1	cysteine-desulfurase	GCF_011290685.1	382	Bacteria>FCB_group>Bacteroidetes/Chlorobi_group>Bacteroidetes>Cytophagia	Cyclobacterium_plantarum	cysteine-desulfurase-[Cyclobacterium-plantarum]	2716263
MBI1231184.1	cysteine-desulfurase	GCA_016124425.1	398	Bacteria>FCB_group>Bacteroidetes/Chlorobi_group>Bacteroidetes>Cytophagia	Cytophagales_bacterium	aminotransferase-class-V-fold-PLP-dependent-enzyme-[Cytophagales-bacterium]	2053541
TAG40975.1	cysteine-desulfurase	GCA_004293645.1	384	Bacteria>FCB_group>Bacteroidetes/Chlorobi_group>Bacteroidetes>Cytophagia	Cytophagia_bacterium	cysteine-desulfurase-[Cytophagia-bacterium]	2026730

Accession	Annotation	GCA	Length	Phyletic.spread	Strain.name	Defline	Species.Taxid
MBT1712310.1	cysteine-desulfurase	GCA_018598175.1	383	Bacteria>FCB_group>Bacteroidetes/Chlorobi_group>Bacteroidetes>Cytophagia	Dawidia_cretensis	cysteine-desulfurase-[Fulvivirgaceae-bacterium-PWU5]	2782350
WP_115833036.1	cysteine-desulfurase	GCF_003383615.1	378	Bacteria>FCB_group>Bacteroidetes/Chlorobi_group>Bacteroidetes>Cytophagia	Dyadobacter_luteus	cysteine-desulfurase-[Dyadobacter-luteus]	2259619
WP_138365718.1	cysteine-desulfurase	GCF_005860805.1	380	Bacteria>FCB_group>Bacteroidetes/Chlorobi_group>Bacteroidetes>Cytophagia	Dyadobacter_luticola	cysteine-desulfurase-[Dyadobacter-luticola]	1979387
WP_225865636.1	cysteine-desulfurase	GCF_008369915.1	386	Bacteria>FCB_group>Bacteroidetes/Chlorobi_group>Bacteroidetes>Cytophagia	Dyadobacter_sp._UC_10	cysteine-desulfurase-[Dyadobacter-sp.-UC-10]	2605428
WP_215222607.1	cysteine-desulfurase	GCF_018642165.1	386	Bacteria>FCB_group>Bacteroidetes/Chlorobi_group>Bacteroidetes>Cytophagia	Echinicola_shivajiensis	aminotransferase-class-V-fold-PLP-dependent-enzyme-[Echinicola-shivajiensis]	1035916
WP_130024272.1	cysteine-desulfurase	GCF_004168285.1	377	Bacteria>FCB_group>Bacteroidetes/Chlorobi_group>Bacteroidetes>Cytophagia	Emticicia_agri	cysteine-desulfurase-[Emticicia-sp.-17J42-9]	2492393
AFK04132.1	cysteine-desulfurase	GCA_000263195.1	376	Bacteria>FCB_group>Bacteroidetes/Chlorobi_group>Bacteroidetes>Cytophagia	Emticicia_oligotrophica_DSM_17448	aminotransferase-class-V-[Emticicia-oligotrophica-DSM-17448]	929562
WP_091516378.1	cysteine-desulfurase	GCF_900112255.1	376	Bacteria>FCB_group>Bacteroidetes/Chlorobi_group>Bacteroidetes>Cytophagia	Flexibacter_flexilis	cysteine-desulfurase-[Flexibacter-flexilis]	998
WP_143401559.1	cysteine-desulfurase	GCF_007097395.1	386	Bacteria>FCB_group>Bacteroidetes/Chlorobi_group>Bacteroidetes>Cytophagia	Fulvivirga_sp._M361	cysteine-desulfurase-[Fulvivirga-sp.-M361]	2594266
WP_135431154.1	cysteine-desulfurase	GCF_004745825.1	382	Bacteria>FCB_group>Bacteroidetes/Chlorobi_group>Bacteroidetes>Cytophagia	Hymenobacter_fodinae	cysteine-desulfurase-[Hymenobacter-sp.-92R-1]	2510796
WP_206986793.1	cysteine-desulfurase	GCF_017313265.1	381	Bacteria>FCB_group>Bacteroidetes/Chlorobi_group>Bacteroidetes>Cytophagia	Hymenobacter	MULTISPECIES:-cysteine-desulfurase-[Hymenobacter]	89966
WP_208178314.1	cysteine-desulfurase	GCF_017571495.1	382	Bacteria>FCB_group>Bacteroidetes/Chlorobi_group>Bacteroidetes>Cytophagia	Hymenobacter_negativus	cysteine-desulfurase-[Hymenobacter-negativus]	2795026
WP_010854647.1	cysteine-desulfurase	GCF_000390185.1	406	Bacteria>FCB_group>Bacteroidetes/Chlorobi_group>Bacteroidetes>Cytophagia	Lunatimonas_lonarensis	cysteine-desulfurase-[Lunatimonas-lonarensis]	1232681
EON76968.1	cysteine-desulfurase	GCA_000390185.1	406	Bacteria>FCB_group>Bacteroidetes/Chlorobi_group>Bacteroidetes>Cytophagia	Lunatimonas_lonarensis	Cysteine-desulfurase-[Lunatimonas-lonarensis]	1232681
WP_002704309.1	cysteine-desulfurase	GCF_000169175.1	387	Bacteria>FCB_group>Bacteroidetes/Chlorobi_group>Bacteroidetes>Cytophagia	Microscilla_marina	cysteine-desulfurase-[Microscilla-marina]	1027
WP_004155710.1	cysteine-desulfurase	GCF_000169175.1	387	Bacteria>FCB_group>Bacteroidetes/Chlorobi_group>Bacteroidetes>Cytophagia	Microscilla_marina	cysteine-desulfurase-[Microscilla-marina]	1027
WP_187065425.1	cysteine-desulfurase	GCF_014306105.1	382	Bacteria>FCB_group>Bacteroidetes/Chlorobi_group>Bacteroidetes>Cytophagia	Pontibacter_cellulosilyticus	cysteine-desulfurase-[Pontibacter-sp.-SD6]	1720253
WP_076666410.1	cysteine-desulfurase	GCF_900156345.1	412	Bacteria>FCB_group>Bacteroidetes/Chlorobi_group>Bacteroidetes>Cytophagia	Pontibacter_indicus	cysteine-desulfurase-like-protein-[Pontibacter-indicus]	1317125
WP_076667697.1	cysteine-desulfurase	GCF_900156345.1	381	Bacteria>FCB_group>Bacteroidetes/Chlorobi_group>Bacteroidetes>Cytophagia	Pontibacter_indicus	cysteine-desulfurase-[Pontibacter-indicus]	1317125
WP_062588837.1	cysteine-desulfurase	GCF_001593005.1	383	Bacteria>FCB_group>Bacteroidetes/Chlorobi_group>Bacteroidetes>Cytophagia	Roseivirga_ehrenbergii	cysteine-desulfurase-[Roseivirga-ehrenbergii]	279360
WP_069843549.1	cysteine-desulfurase	GCF_001747095.1	384	Bacteria>FCB_group>Bacteroidetes/Chlorobi_group>Bacteroidetes>Cytophagia	Roseivirga_sp._4D4	cysteine-desulfurase-[Roseivirga-sp.-4D4]	1889784
MBO2012579.1	cysteine-desulfurase	GCA_017571495.1	382	Bacteria>FCB_group>Bacteroidetes/Chlorobi_group>Bacteroidetes>Cytophagia	Siccationidurans_soli	cysteine-desulfurase-[Siccationidurans-soli]	373890
WP_097126511.1	cysteine-desulfurase	GCF_900230225.1	410	Bacteria>FCB_group>Bacteroidetes/Chlorobi_group>Bacteroidetes>Cytophagia	Spirosoma_fluviale	cysteine-desulfurase-[Spirosoma-fluviale]	1597977
WP_020597296.1	cysteine-desulfurase	GCF_000374025.1	388	Bacteria>FCB_group>Bacteroidetes/Chlorobi_group>Bacteroidetes>Cytophagia	Spirosoma_panaciterrae	cysteine-desulfurase-[Spirosoma-panaciterrae]	496058
WP_171738715.1	cysteine-desulfurase	GCF_013127955.1	385	Bacteria>FCB_group>Bacteroidetes/Chlorobi_group>Bacteroidetes>Cytophagia	Spirosoma_taeanense	cysteine-desulfurase-[Spirosoma-taeanense]	2735870
WP_166918551.1	cysteine-desulfurase	GCF_011761635.1	398	Bacteria>FCB_group>Bacteroidetes/Chlorobi_group>Bacteroidetes>Cytophagia	Thermonema_lapsum	cysteine-desulfurase-[Thermonema-lapsum]	28195
WP_038030425.1	cysteine-desulfurase	GCF_000426825.1	398	Bacteria>FCB_group>Bacteroidetes/Chlorobi_group>Bacteroidetes>Cytophagia	Thermonema_rossianum	cysteine-desulfurase-[Thermonema-rossianum]	55505
WP_202335809.1	cysteine-desulfurase	GCF_016757735.1	382	Bacteria>FCB_group>Bacteroidetes/Chlorobi_group>Bacteroidetes>Flavobacteriia	Aequorivita	MULTISPECIES:-cysteine-desulfurase-[unclassified-Aequorivita]	153265
WP_126160986.1	cysteine-desulfurase	GCF_003957295.1	381	Bacteria>FCB_group>Bacteroidetes/Chlorobi_group>Bacteroidetes>Flavobacteriia	Arenibacter_aquaticus	cysteine-desulfurase-[Arenibacter-aquaticus]	2489054
WP_191736960.1	cysteine-desulfurase	GCF_014837145.1	369	Bacteria>FCB_group>Bacteroidetes/Chlorobi_group>Bacteroidetes>Flavobacteriia	Chryseobacterium_caseinilyticum	cysteine-desulfurase-[Chryseobacterium-caseinilyticum]	2771428
WP_066690431.1	cysteine-desulfurase	GCF_900142615.1	372	Bacteria>FCB_group>Bacteroidetes/Chlorobi_group>Bacteroidetes>Flavobacteriia	Chryseobacterium_contaminans	cysteine-desulfurase-[Chryseobacterium-contaminans]	1423959
WP_105683043.1	cysteine-desulfurase	GCF_002979665.1	371	Bacteria>FCB_group>Bacteroidetes/Chlorobi_group>Bacteroidetes>Flavobacteriia	Chryseobacterium_culicis	cysteine-desulfurase-[Chryseobacterium-culicis]	680127
WP_027385169.1	cysteine-desulfurase	GCF_000430845.1	369	Bacteria>FCB_group>Bacteroidetes/Chlorobi_group>Bacteroidetes>Flavobacteriia	Chryseobacterium_gregarium	cysteine-desulfurase-[Chryseobacterium-gregarium]	456299
WP_130868040.1	cysteine-desulfurase	GCF_900683405.1	382	Bacteria>FCB_group>Bacteroidetes/Chlorobi_group>Bacteroidetes>Flavobacteriia	Chryseobacterium_indologenes	cysteine-desulfurase-[Chryseobacterium-indologenes]	253
WP_111959906.1	cysteine-desulfurase	GCF_007991435.1	382	Bacteria>FCB_group>Bacteroidetes/Chlorobi_group>Bacteroidetes>Flavobacteriia	Chryseobacterium_lathyri	cysteine-desulfurase-[Chryseobacterium-lathyri]	395933
WP_115971822.1	cysteine-desulfurase	GCF_003385515.1	378	Bacteria>FCB_group>Bacteroidetes/Chlorobi_group>Bacteroidetes>Flavobacteriia	Chryseobacterium_pennae	cysteine-desulfurase-[Chryseobacterium-pennae]	2258962
WP_106658985.1	cysteine-desulfurase	GCF_002843175.1	380	Bacteria>FCB_group>Bacteroidetes/Chlorobi_group>Bacteroidetes>Flavobacteriia	Confluentibacter_flavum	cysteine-desulfurase-[Confluentibacter-flavum]	1909700
MBT6170171.1	cysteine-desulfurase	GCA_018700275.1	132	Bacteria>FCB_group>Bacteroidetes/Chlorobi_group>Bacteroidetes>Flavobacteriia	Flavobacteriaceae_bacterium	aminotransferase-class-V-fold-PLP-dependent-enzyme-[Flavobacteriaceae-bacterium]	1871037
MBL4662528.1	cysteine-desulfurase	GCA_016763715.1	381	Bacteria>FCB_group>Bacteroidetes/Chlorobi_group>Bacteroidetes>Flavobacteriia	Flavobacteriaceae_bacterium	cysteine-desulfurase-[Flavobacteriaceae-bacterium]	1871037
MAB47204.1	cysteine-desulfurase	GCA_002682935.1	378	Bacteria>FCB_group>Bacteroidetes/Chlorobi_group>Bacteroidetes>Flavobacteriia	Flavobacteriaceae_bacterium	IscS-subfamily-cysteine-desulfurase-[Flavobacteriaceae-bacterium]	1871037
MAD96782.1	cysteine-desulfurase	GCA_002686575.1	385	Bacteria>FCB_group>Bacteroidetes/Chlorobi_group>Bacteroidetes>Flavobacteriia	Flavobacteriaceae_bacterium	IscS-subfamily-cysteine-desulfurase-[Flavobacteriaceae-bacterium]	1871037
MBA3985237.1	cysteine-desulfurase	GCA_013820965.1	379	Bacteria>FCB_group>Bacteroidetes/Chlorobi_group>Bacteroidetes>Flavobacteriia	Flavobacteriales_bacterium	cysteine-desulfurase-[Flavobacteriales-bacterium]	2021391
MBK7482252.1	cysteine-desulfurase	GCA_016706145.1	369	Bacteria>FCB_group>Bacteroidetes/Chlorobi_group>Bacteroidetes>Flavobacteriia	Flavobacteriales_bacterium	cysteine-desulfurase-[Flavobacteriales-bacterium]	2021391
MBK8707884.1	cysteine-desulfurase	GCA_016715385.1	392	Bacteria>FCB_group>Bacteroidetes/Chlorobi_group>Bacteroidetes>Flavobacteriia	Flavobacteriales_bacterium	cysteine-desulfurase-[Flavobacteriales-bacterium]	2021391
HAQ71019.1	cysteine-desulfurase	GCA_003477125.1	341	Bacteria>FCB_group>Bacteroidetes/Chlorobi_group>Bacteroidetes>Flavobacteriia	Flavobacteriales_bacterium	hypothetical-protein-[Flavobacteriales-bacterium]	2021391
WP_072959267.1	cysteine-desulfurase	GCF_002217195.1	373	Bacteria>FCB_group>Bacteroidetes/Chlorobi_group>Bacteroidetes>Flavobacteriia	Flavobacterium_aquidurense	cysteine-desulfurase-[Flavobacterium-aquidurense]	362413
WP_097554525.1	cysteine-desulfurase	GCF_002530755.1	375	Bacteria>FCB_group>Bacteroidetes/Chlorobi_group>Bacteroidetes>Flavobacteriia	Flavobacterium_branchiophilum	cysteine-desulfurase-[Flavobacterium-branchiophilum]	55197
WP_068843552.1	cysteine-desulfurase	GCF_900142685.1	382	Bacteria>FCB_group>Bacteroidetes/Chlorobi_group>Bacteroidetes>Flavobacteriia	Flavobacterium_chilense	cysteine-desulfurase-[Flavobacterium-chilense]	946677
WP_114754045.1	cysteine-desulfurase	GCF_003350545.1	374	Bacteria>FCB_group>Bacteroidetes/Chlorobi_group>Bacteroidetes>Flavobacteriia	Flavobacterium_glaciei	cysteine-desulfurase-[Flavobacterium-glaciei]	386300
WP_035626214.1	cysteine-desulfurase	GCF_000737695.1	393	Bacteria>FCB_group>Bacteroidetes/Chlorobi_group>Bacteroidetes>Flavobacteriia	Flavobacterium_hydatis	cysteine-desulfurase-[Flavobacterium-hydatis]	991
WP_110347448.1	cysteine-desulfurase	GCF_003202405.1	393	Bacteria>FCB_group>Bacteroidetes/Chlorobi_group>Bacteroidetes>Flavobacteriia	Flavobacterium_hydrophilum	cysteine-desulfurase-[Flavobacterium-hydrophilum]	2211445
WP_014388840.1	cysteine-desulfurase	GCF_000455605.1	391	Bacteria>FCB_group>Bacteroidetes/Chlorobi_group>Bacteroidetes>Flavobacteriia	Flavobacterium_indicum	cysteine-desulfurase-[Flavobacterium-indicum]	312277
WP_113637034.1	cysteine-desulfurase	GCF_003312425.1	393	Bacteria>FCB_group>Bacteroidetes/Chlorobi_group>Bacteroidetes>Flavobacteriia	Flavobacterium_psychrolimnae	cysteine-desulfurase-[Flavobacterium-psychrolimnae]	249351
WP_134355969.1	cysteine-desulfurase	GCF_900610355.1	374	Bacteria>FCB_group>Bacteroidetes/Chlorobi_group>Bacteroidetes>Flavobacteriia	Flavobacterium_psychrophilum	cysteine-desulfurase-[Flavobacterium-psychrophilum]	96345
WP_233549818.1	cysteine-desulfurase	GCF_003634615.1	377	Bacteria>FCB_group>Bacteroidetes/Chlorobi_group>Bacteroidetes>Flavobacteriia	Flavobacterium_sp._102	cysteine-desulfurase-[Flavobacterium-sp.-102]	2135623
WP_248433246.1	cysteine-desulfurase	GCF_023195775.1	385	Bacteria>FCB_group>Bacteroidetes/Chlorobi_group>Bacteroidetes>Flavobacteriia	Flavobacterium_sp._IGB_4-14	aminotransferase-class-V-fold-PLP-dependent-enzyme-[Flavobacterium-sp.-IGB-4-14]	1871076
WP_056250046.1	cysteine-desulfurase	GCF_001422725.1	372	Bacteria>FCB_group>Bacteroidetes/Chlorobi_group>Bacteroidetes>Flavobacteriia	Flavobacterium_sp._Leaf82	cysteine-desulfurase-[Flavobacterium-sp.-Leaf82]	1736238
WP_160749866.1	cysteine-desulfurase	GCF_009826935.1	141	Bacteria>FCB_group>Bacteroidetes/Chlorobi_group>Bacteroidetes>Flavobacteriia	Flavobacterium_sp._Sd200	SufE-family-protein-[Flavobacterium-sp.-Sd200]	2692211
WP_035759213.1	cysteine-desulfurase	GCF_000425485.1	374	Bacteria>FCB_group>Bacteroidetes/Chlorobi_group>Bacteroidetes>Flavobacteriia	Flavobacterium_tegetincola	cysteine-desulfurase-[Flavobacterium-tegetincola]	150172
WP_057781616.1	cysteine-desulfurase	-	377	Bacteria>FCB_group>Bacteroidetes/Chlorobi_group>Bacteroidetes>Flavobacteriia	Formosa_algae	cysteine-desulfurase-[Formosa-algae]	225843
WP_207303286.1	cysteine-desulfurase	GCF_004310325.1	385	Bacteria>FCB_group>Bacteroidetes/Chlorobi_group>Bacteroidetes>Flavobacteriia	Hyunsoonleella_flava	cysteine-desulfurase-[Hyunsoonleella-sp.-T58]	2527939
WP_229731298.1	cysteine-desulfurase	-	360	Bacteria>FCB_group>Bacteroidetes/Chlorobi_group>Bacteroidetes>Flavobacteriia	Hyunsoonleella_pacifica	cysteine-desulfurase-[Hyunsoonleella-pacifica]	1080224
MBF8984100.1	cysteine-desulfurase	GCA_015686845.1	386	Bacteria>FCB_group>Bacteroidetes/Chlorobi_group>Bacteroidetes>Flavobacteriia	Lutibacter_sp._B2	aminotransferase-class-V-fold-PLP-dependent-enzyme-[Lutibacter-sp.-B2]	2789328
WP_196119035.1	cysteine-desulfurase	-	386	Bacteria>FCB_group>Bacteroidetes/Chlorobi_group>Bacteroidetes>Flavobacteriia	Lutibacter_sp._B2	aminotransferase-class-V-fold-PLP-dependent-enzyme-[Lutibacter-sp.-B2]	2789328
WP_053976589.1	cysteine-desulfurase	GCF_001280435.1	354	Bacteria>FCB_group>Bacteroidetes/Chlorobi_group>Bacteroidetes>Flavobacteriia	Mangrovimonas_xylaniphaga	cysteine-desulfurase-[Mangrovimonas-xylaniphaga]	1645915
WP_169932165.1	cysteine-desulfurase	GCF_012974865.1	381	Bacteria>FCB_group>Bacteroidetes/Chlorobi_group>Bacteroidetes>Flavobacteriia	Nonlabens_sp._Ci31	cysteine-desulfurase-[Nonlabens-sp.-Ci31]	2608253
WP_111659542.1	cysteine-desulfurase	GCF_003259525.1	386	Bacteria>FCB_group>Bacteroidetes/Chlorobi_group>Bacteroidetes>Flavobacteriia	Olleya_aquimaris	aminotransferase-class-V-fold-PLP-dependent-enzyme-[Olleya-aquimaris]	639310
WP_231874599.1	cysteine-desulfurase	GCF_001640115.1	353	Bacteria>FCB_group>Bacteroidetes/Chlorobi_group>Bacteroidetes>Flavobacteriia	Polaribacter_atrinae	cysteine-desulfurase-[Polaribacter-atrinae]	1333662
WP_223269093.1	cysteine-desulfurase	GCF_007997065.1	353	Bacteria>FCB_group>Bacteroidetes/Chlorobi_group>Bacteroidetes>Flavobacteriia	Polaribacter_sp._IC063	cysteine-desulfurase-[Polaribacter-sp.-IC063]	57031
WP_093026447.1	cysteine-desulfurase	GCF_900116665.1	392	Bacteria>FCB_group>Bacteroidetes/Chlorobi_group>Bacteroidetes>Flavobacteriia	Pustulibacterium_marinum	cysteine-desulfurase-[Pustulibacterium-marinum]	1224947
WP_214985603.1	cysteine-desulfurase	-	372	Bacteria>FCB_group>Bacteroidetes/Chlorobi_group>Bacteroidetes>Flavobacteriia	Tenacibaculum_dicentrarchi	aminotransferase-class-V-fold-PLP-dependent-enzyme-[Tenacibaculum-dicentrarchi]	669041
WP_101908032.1	cysteine-desulfurase	-	372	Bacteria>FCB_group>Bacteroidetes/Chlorobi_group>Bacteroidetes>Flavobacteriia	Tenacibaculum_dicentrarchi	cysteine-desulfurase-[Tenacibaculum-dicentrarchi]	669041
WP_232163698.1	cysteine-desulfurase	GCF_022380985.1	379	Bacteria>FCB_group>Bacteroidetes/Chlorobi_group>Bacteroidetes>Flavobacteriia	Tenacibaculum_dicentrarchi	cysteine-desulfurase-[Tenacibaculum-dicentrarchi]	669041
WP_239776823.1	cysteine-desulfurase	GCF_022382115.1	379	Bacteria>FCB_group>Bacteroidetes/Chlorobi_group>Bacteroidetes>Flavobacteriia	Tenacibaculum_finnmarkense	aminotransferase-class-V-fold-PLP-dependent-enzyme-[Tenacibaculum-finnmarkense]	2781243
WP_198912866.1	cysteine-desulfurase	GCF_015143465.1	379	Bacteria>FCB_group>Bacteroidetes/Chlorobi_group>Bacteroidetes>Flavobacteriia	Tenacibaculum_piscium	cysteine-desulfurase-[Tenacibaculum-piscium]	1458515
WP_233884040.1	cysteine-desulfurase	GCF_021390725.1	379	Bacteria>FCB_group>Bacteroidetes/Chlorobi_group>Bacteroidetes>Flavobacteriia	Tenacibaculum_piscium	cysteine-desulfurase-[Tenacibaculum-piscium]	1458515
WP_233898295.1	cysteine-desulfurase	GCF_021390755.1	379	Bacteria>FCB_group>Bacteroidetes/Chlorobi_group>Bacteroidetes>Flavobacteriia	Tenacibaculum_piscium	cysteine-desulfurase-[Tenacibaculum-piscium]	1458515
MCA0151712.1	cysteine-desulfurase	GCA_020166345.1	378	Bacteria>FCB_group>Bacteroidetes/Chlorobi_group>Bacteroidetes>Flavobacteriia	Winogradskyella_sp._2Y89	cysteine-desulfurase-[Winogradskyella-sp.-2Y89]	2877122
WP_224476684.1	cysteine-desulfurase	GCF_020166345.1	378	Bacteria>FCB_group>Bacteroidetes/Chlorobi_group>Bacteroidetes>Flavobacteriia	Winogradskyella_sp._2Y89	cysteine-desulfurase-[Winogradskyella-sp.-2Y89]	2877122
WP_242937761.1	cysteine-desulfurase	GCF_022760175.1	385	Bacteria>FCB_group>Bacteroidetes/Chlorobi_group>Bacteroidetes>Flavobacteriia	Zhouia_spongiae	aminotransferase-class-V-fold-PLP-dependent-enzyme-[Zhouia-spongiae]	2202721
MBK6352106.1	cysteine-desulfurase	GCA_016705435.1	393	Bacteria>FCB_group>Bacteroidetes/Chlorobi_group>Bacteroidetes>Saprospiria	Saprospiraceae_bacterium	cysteine-desulfurase-[Saprospiraceae-bacterium]	2202734
MBK6572855.1	cysteine-desulfurase	GCA_016704015.1	393	Bacteria>FCB_group>Bacteroidetes/Chlorobi_group>Bacteroidetes>Saprospiria	Saprospiraceae_bacterium	cysteine-desulfurase-[Saprospiraceae-bacterium]	2202734
MBL7823161.1	cysteine-desulfurase	GCA_016787185.1	390	Bacteria>FCB_group>Bacteroidetes/Chlorobi_group>Bacteroidetes>Saprospiria	Saprospiraceae_bacterium	cysteine-desulfurase-[Saprospiraceae-bacterium]	2202734
MBP6235338.1	cysteine-desulfurase	GCA_017986015.1	390	Bacteria>FCB_group>Bacteroidetes/Chlorobi_group>Bacteroidetes>Saprospiria	Saprospiraceae_bacterium	cysteine-desulfurase-[Saprospiraceae-bacterium]	2202734
MBV8388979.1	cysteine-desulfurase	GCA_019235595.1	388	Bacteria>FCB_group>Bacteroidetes/Chlorobi_group>Bacteroidetes>Sphingobacteriia	Mucilaginibacter_sp.	cysteine-desulfurase-[Mucilaginibacter-sp.]	1882438
WP_188753155.1	cysteine-desulfurase	GCF_014642075.1	386	Bacteria>FCB_group>Bacteroidetes/Chlorobi_group>Bacteroidetes>Sphingobacteriia	Parapedobacter_defluvii	cysteine-desulfurase-[Parapedobacter-defluvii]	2045106
WP_079717008.1	cysteine-desulfurase	GCF_900168055.1	382	Bacteria>FCB_group>Bacteroidetes/Chlorobi_group>Bacteroidetes>Sphingobacteriia	Parapedobacter_luteus	cysteine-desulfurase-[Parapedobacter-luteus]	623280
WP_079717020.1	cysteine-desulfurase	GCF_900168055.1	386	Bacteria>FCB_group>Bacteroidetes/Chlorobi_group>Bacteroidetes>Sphingobacteriia	Parapedobacter_luteus	cysteine-desulfurase-[Parapedobacter-luteus]	623280
OOK62621.1	cysteine-desulfurase	GCA_002003685.1	353	Bacteria>FCB_group>Bacteroidetes/Chlorobi_group>Bacteroidetes>Sphingobacteriia	Pedobacter_himalayensis	cysteine-desulfurase-[Pedobacter-himalayensis]	246796
RYE46053.1	cysteine-desulfurase	GCA_004144425.1	271	Bacteria>FCB_group>Bacteroidetes/Chlorobi_group>Bacteroidetes>Sphingobacteriia	Sphingobacteriales_bacterium	cysteine-desulfurase,-partial-[Sphingobacteriales-bacterium]	2044944
WP_200818754.1	cysteine-desulfurase	GCF_011046555.1	375	Bacteria>FCB_group>Bacteroidetes/Chlorobi_group>Bacteroidetes>Sphingobacteriia	Sphingobacterium_lactis	cysteine-desulfurase-[Sphingobacterium-lactis]	797291
WP_079640700.1	cysteine-desulfurase	GCF_900168125.1	389	Bacteria>FCB_group>Bacteroidetes/Chlorobi_group>Bacteroidetes>Sphingobacteriia	Sphingobacterium_nematocida	cysteine-desulfurase-[Sphingobacterium-nematocida]	1513896
GGE18221.1	cysteine-desulfurase	GCA_014644335.1	343	Bacteria>FCB_group>Bacteroidetes/Chlorobi_group>Bacteroidetes>Sphingobacteriia	Sphingobacterium_soli	cysteine-desulfurase-IscS-[Sphingobacterium-soli]	1914757
WP_182498393.1	cysteine-desulfurase	GCF_014644335.1	386	Bacteria>FCB_group>Bacteroidetes/Chlorobi_group>Bacteroidetes>Sphingobacteriia	Sphingobacterium_soli	cysteine-desulfurase-[Sphingobacterium-soli]	1914757
WP_066751073.1	cysteine-desulfurase	GCF_001586775.1	389	Bacteria>FCB_group>Bacteroidetes/Chlorobi_group>Bacteroidetes>Sphingobacteriia	Sphingobacterium_sp._CFCC_11742	cysteine-desulfurase-[Sphingobacterium-sp.-CFCC-11742]	1775560
WP_088161323.1	cysteine-desulfurase	GCF_002196555.1	379	Bacteria>FCB_group>Bacteroidetes/Chlorobi_group>Bacteroidetes>Sphingobacteriia	Sphingobacterium_sp._G1-14	cysteine-desulfurase-[Sphingobacterium-sp.-G1-14]	2003121
TCR11090.1	cysteine-desulfurase	GCA_004342495.1	378	Bacteria>FCB_group>Bacteroidetes/Chlorobi_group>Bacteroidetes>Sphingobacteriia	Sphingobacterium_sp._JUb78	cysteine-desulfurase-[Sphingobacterium-sp.-JUb78]	2485111
WP_132771222.1	cysteine-desulfurase	GCF_004342495.1	378	Bacteria>FCB_group>Bacteroidetes/Chlorobi_group>Bacteroidetes>Sphingobacteriia	Sphingobacterium_sp._JUb78	cysteine-desulfurase-[Sphingobacterium-sp.-JUb78]	2485111
MCB0536682.1	cysteine-desulfurase	GCA_020436205.1	57	Bacteria>FCB_group>Bacteroidetes/Chlorobi_group>Bacteroidetes>unclassified_Bacteroidetes	Bacteroidetes_bacterium	aminotransferase-class-V-fold-PLP-dependent-enzyme-[Bacteroidetes-bacterium]	1898104
MBK7908779.1	cysteine-desulfurase	GCA_016713765.1	393	Bacteria>FCB_group>Bacteroidetes/Chlorobi_group>Bacteroidetes>unclassified_Bacteroidetes	Bacteroidetes_bacterium	cysteine-desulfurase-[Bacteroidetes-bacterium]	1898104
MBN9483653.1	cysteine-desulfurase	GCA_017308155.1	380	Bacteria>FCB_group>Bacteroidetes/Chlorobi_group>Bacteroidetes>unclassified_Bacteroidetes	Bacteroidetes_bacterium	cysteine-desulfurase-[Bacteroidetes-bacterium]	1898104
MBS1758869.1	cysteine-desulfurase	GCA_018268055.1	382	Bacteria>FCB_group>Bacteroidetes/Chlorobi_group>Bacteroidetes>unclassified_Bacteroidetes	Bacteroidetes_bacterium	cysteine-desulfurase-[Bacteroidetes-bacterium]	1898104
TAE69642.1	cysteine-desulfurase	GCA_004293265.1	374	Bacteria>FCB_group>Bacteroidetes/Chlorobi_group>Bacteroidetes>unclassified_Bacteroidetes	Bacteroidetes_bacterium	cysteine-desulfurase-[Bacteroidetes-bacterium]	1898104
OFY07334.1	cysteine-desulfurase	GCA_001769115.1	384	Bacteria>FCB_group>Bacteroidetes/Chlorobi_group>Bacteroidetes>unclassified_Bacteroidetes	Bacteroidetes_bacterium_GWE2_42_42	IscS-subfamily-cysteine-desulfurase-[Bacteroidetes-bacterium-GWE2_42_42]	1797339
MBV5330007.1	cysteine-desulfurase	GCA_019162945.1	376	Bacteria>FCB_group>Bacteroidetes/Chlorobi_group>Chlorobi>Chlorobia	Chlorobium_sp.	cysteine-desulfurase-DndA-[Chlorobium-sp.]	1095
MBN8544907.1	cysteine-desulfurase	GCA_017302895.1	388	Bacteria>FCB_group>Bacteroidetes/Chlorobi_group>Ignavibacteriae>Ignavibacteria	Ignavibacteria_bacterium	cysteine-desulfurase-[Ignavibacteria-bacterium]	2053306
MBW7841612.1	cysteine-desulfurase	GCA_019455015.1	387	Bacteria>FCB_group>Bacteroidetes/Chlorobi_group>Ignavibacteriae>Ignavibacteria	Ignavibacterium_sp.	cysteine-desulfurase-[Ignavibacterium-sp.]	2651167
KOY84524.1	cysteine-desulfurase	GCA_001281695.1	378	Bacteria>FCB_group>Bacteroidetes/Chlorobi_group>unclassified_Bacteroidetes/Chlorobi_group	bacterium_336/3	cysteine-desulfurase-IscS-[bacterium-336/3]	1664068
HHE40066.1	cysteine-desulfurase	GCA_011372435.1	385	Bacteria>FCB_group>Candidatus_Cloacimonetes>unclassified_Candidatus_Cloacimonetes	Candidatus_Cloacimonetes_bacterium	cysteine-desulfurase-[Candidatus-Cloacimonetes-bacterium]	2030808
RLC55442.1	cysteine-desulfurase	GCA_003647865.1	386	Bacteria>FCB_group>Candidatus_Cloacimonetes>unclassified_Candidatus_Cloacimonetes	Candidatus_Cloacimonetes_bacterium	IscS-subfamily-cysteine-desulfurase-[Candidatus-Cloacimonetes-bacterium]	2030808
MBO5533291.1	cysteine-desulfurase	GCA_017626945.1	384	Bacteria>FCB_group>Fibrobacteres>Fibrobacteria>Fibrobacterales	Fibrobacter_sp.	aminotransferase-class-V-fold-PLP-dependent-enzyme-[Fibrobacter-sp.]	35828
MBO7059478.1	cysteine-desulfurase	GCA_017642335.1	384	Bacteria>FCB_group>Fibrobacteres>Fibrobacteria>Fibrobacterales	Fibrobacter_sp.	aminotransferase-class-V-fold-PLP-dependent-enzyme-[Fibrobacter-sp.]	35828
MBQ9224760.1	cysteine-desulfurase	GCA_017470355.1	384	Bacteria>FCB_group>Fibrobacteres>Fibrobacteria>Fibrobacterales	Fibrobacter_sp.	aminotransferase-class-V-fold-PLP-dependent-enzyme-[Fibrobacter-sp.]	35828
WP_014546341.1	cysteine-desulfurase	GCF_000024665.1	384	Bacteria>FCB_group>Fibrobacteres>Fibrobacteria>Fibrobacterales	Fibrobacter_succinogenes	aminotransferase-class-V-fold-PLP-dependent-enzyme-[Fibrobacter-succinogenes]	833
ADL25915.1	cysteine-desulfurase	GCA_000146505.1	384	Bacteria>FCB_group>Fibrobacteres>Fibrobacteria>Fibrobacterales	Fibrobacter_succinogenes_subsp._succinogenes_S85	cysteine-desulfurase-family-protein-[Fibrobacter-succinogenes-subsp.-succinogenes-S85]	59374
MBT4187592.1	cysteine-desulfurase	GCA_018651425.1	378	Bacteria>FCB_group>Gemmatimonadetes>Gemmatimonadetes>Gemmatimonadales	Gemmatimonadales_bacterium	aminotransferase-class-V-fold-PLP-dependent-enzyme-[Gemmatimonadales-bacterium]	2448054
MXW05562.1	cysteine-desulfurase	GCA_009835325.1	377	Bacteria>FCB_group>Gemmatimonadetes>unclassified_Gemmatimonadetes	Gemmatimonadetes_bacterium	cysteine-desulfurase-DndA-[Gemmatimonadetes-bacterium]	2026742
MBA4157584.1	cysteine-desulfurase	GCA_013820915.1	400	Bacteria>FCB_group>Gemmatimonadetes>unclassified_Gemmatimonadetes	Gemmatimonadetes_bacterium	cysteine-desulfurase-[Gemmatimonadetes-bacterium]	2026742
MXW05553.1	cysteine-desulfurase	GCA_009835325.1	383	Bacteria>FCB_group>Gemmatimonadetes>unclassified_Gemmatimonadetes	Gemmatimonadetes_bacterium	cysteine-desulfurase-[Gemmatimonadetes-bacterium]	2026742
MXX38515.1	cysteine-desulfurase	GCA_009835785.1	389	Bacteria>FCB_group>Gemmatimonadetes>unclassified_Gemmatimonadetes	Gemmatimonadetes_bacterium	cysteine-desulfurase-[Gemmatimonadetes-bacterium]	2026742
MYB66986.1	cysteine-desulfurase	GCA_009840005.1	389	Bacteria>FCB_group>Gemmatimonadetes>unclassified_Gemmatimonadetes	Gemmatimonadetes_bacterium	cysteine-desulfurase-[Gemmatimonadetes-bacterium]	2026742
MYB71148.1	cysteine-desulfurase	GCA_009840005.1	386	Bacteria>FCB_group>Gemmatimonadetes>unclassified_Gemmatimonadetes	Gemmatimonadetes_bacterium	cysteine-desulfurase-[Gemmatimonadetes-bacterium]	2026742
WP_047394003.1	cysteine-desulfurase	GCF_000799075.1	375	Bacteria>Fusobacteria>Fusobacteriia>Fusobacteriales>Fusobacteriaceae	Cetobacterium_sp._ZOR0034	aminotransferase-class-V-fold-PLP-dependent-enzyme-[Cetobacterium-sp.-ZOR0034]	1339239
MBN2837858.1	cysteine-desulfurase	GCA_016938425.1	371	Bacteria>Fusobacteria>Fusobacteriia>Fusobacteriales>Fusobacteriaceae	Fusobacteriaceae_bacterium	cysteine-desulfurase-[Fusobacteriaceae-bacterium]	2053555
WP_175613342.1	cysteine-desulfurase	GCF_903181465.1	384	Bacteria>Fusobacteria>Fusobacteriia>Fusobacteriales>Fusobacteriaceae	Fusobacterium_mortiferum	aminotransferase-class-V-fold-PLP-dependent-enzyme-[Fusobacterium-mortiferum]	850

Accession	Annotation	GCA	Length	Phyletic.spread	Strain.name	Defline	Species.Taxid
WP_239458983.1	cysteine-desulfurase	GCF_016900045.1	384	Bacteria>Fusobacteria>Fusobacteriia>Fusobacteriales>Fusobacteriaceae	Fusobacterium_mortiferum	aminotransferase-class-V-fold-PLP-dependent-enzyme-[Fusobacterium-mortiferum]	850
WP_005959416.1	cysteine-desulfurase	GCF_003019715.1	381	Bacteria>Fusobacteria>Fusobacteriia>Fusobacteriales>Fusobacteriaceae	Fusobacterium_necrophorum	aminotransferase-class-V-fold-PLP-dependent-enzyme-[Fusobacterium-necrophorum]	859
WP_008800311.1	cysteine-desulfurase	GCF_000162235.2	383	Bacteria>Fusobacteria>Fusobacteriia>Fusobacteriales>Fusobacteriaceae	Fusobacterium_nucleatum	aminotransferase-class-V-fold-PLP-dependent-enzyme-[Fusobacterium-nucleatum]	851
WP_011016395.1	cysteine-desulfurase	GCF_003019295.1	383	Bacteria>Fusobacteria>Fusobacteriia>Fusobacteriales>Fusobacteriaceae	Fusobacterium_nucleatum	aminotransferase-class-V-fold-PLP-dependent-enzyme-[Fusobacterium-nucleatum]	851
WP_170185951.1	cysteine-desulfurase	GCF_012843705.1	385	Bacteria>Fusobacteria>Fusobacteriia>Fusobacteriales>Fusobacteriaceae	Fusobacterium_sp._FSA-380-WT-3A	aminotransferase-class-V-fold-PLP-dependent-enzyme-[Fusobacterium-sp.-FSA-380-WT-3A]	2725304
MCF2640576.1	cysteine-desulfurase	GCA_021531615.1	383	Bacteria>Fusobacteria>Fusobacteriia>Fusobacteriales>Fusobacteriaceae	Fusobacterium_varium	aminotransferase-class-V-fold-PLP-dependent-enzyme-[Fusobacterium-varium]	856
MBI2058406.1	cysteine-desulfurase	GCA_016180645.1	376	Bacteria>Nitrospirae>unclassified_Nitrospirae	Nitrospirae_bacterium	aminotransferase-class-V-fold-PLP-dependent-enzyme-[Nitrospirae-bacterium]	2026887
WP_162148943.1	cysteine-desulfurase	GCF_000495815.1	386	Bacteria>Proteobacteria>Alphaproteobacteria>Caulobacterales>Caulobacteraceae	Asticcacaulis_sp._AC466	cysteine-desulfurase-[Asticcacaulis-sp.-AC466]	1282362
ESQ84196.1	cysteine-desulfurase	GCA_000495815.1	391	Bacteria>Proteobacteria>Alphaproteobacteria>Caulobacterales>Caulobacteraceae	Asticcacaulis_sp._AC466	hypothetical-protein-AEAC466_10655-[Asticcacaulis-sp.-AC466]	1282362
WP_168045876.1	cysteine-desulfurase	-	402	Bacteria>Proteobacteria>Alphaproteobacteria>Caulobacterales>Caulobacteraceae	Brevundimonas_alba	cysteine-desulfurase-[Brevundimonas-alba]	74314
WP_125255813.1	cysteine-desulfurase	GCF_003934285.1	377	Bacteria>Proteobacteria>Alphaproteobacteria>Caulobacterales>Caulobacteraceae	Brevundimonas_fluminis	cysteine-desulfurase-[Brevundimonas-fluminis]	2487274
QTC90321.1	cysteine-desulfurase	GCA_017487405.1	391	Bacteria>Proteobacteria>Alphaproteobacteria>Caulobacterales>Caulobacteraceae	Brevundimonas_goettingensis	cysteine-desulfurase-[Brevundimonas-goettingensis]	2774190
WP_207868740.1	cysteine-desulfurase	GCF_017487405.1	391	Bacteria>Proteobacteria>Alphaproteobacteria>Caulobacterales>Caulobacteraceae	Brevundimonas_goettingensis	cysteine-desulfurase-[Brevundimonas-goettingensis]	2774190
WP_221230669.1	cysteine-desulfurase	-	405	Bacteria>Proteobacteria>Alphaproteobacteria>Caulobacterales>Caulobacteraceae	Brevundimonas_variabilis	aminotransferase-class-V-fold-PLP-dependent-enzyme-[Brevundimonas-variabilis]	74312
WP_202948069.1	cysteine-desulfurase	GCF_000281955.1	423	Bacteria>Proteobacteria>Alphaproteobacteria>Caulobacterales>Caulobacteraceae	Caulobacter_sp._AP07	aminotransferase-class-V-fold-PLP-dependent-enzyme-[Caulobacter-sp.-AP07]	1144304
MBP7703726.1	cysteine-desulfurase	GCA_017999465.1	378	Bacteria>Proteobacteria>Alphaproteobacteria>Caulobacterales>Caulobacteraceae	Caulobacter_sp.	cysteine-desulfurase-[Caulobacter-sp.]	78
OHB26291.1	cysteine-desulfurase	GCA_001824475.1	379	Bacteria>Proteobacteria>Alphaproteobacteria>Caulobacterales>Caulobacteraceae	Phenylobacterium_sp._RIFCSPHIGHO2_01_FULL_69_31	cysteine-desulfurase-DndA-[Phenylobacterium-sp.-RIFCSPHIGHO2_01_FULL_69_31]	1801944
WP_188519105.1	cysteine-desulfurase	GCF_014636935.1	383	Bacteria>Proteobacteria>Alphaproteobacteria>Hyphomicrobiales>Alsobacteraceae	Alsobacter_metallidurans	cysteine-desulfurase-[Alsobacter-metallidurans]	340221
WP_237853928.1	cysteine-desulfurase	GCF_022024335.1	417	Bacteria>Proteobacteria>Alphaproteobacteria>Hyphomicrobiales>Brucellaceae	Brucella_pseudogrignonensis	cysteine-desulfurase-[Brucella-pseudogrignonensis]	419475
WP_164532785.1	cysteine-desulfurase	GCF_011058215.1	395	Bacteria>Proteobacteria>Alphaproteobacteria>Hyphomicrobiales>Devosiaceae	Devosia_aurantiaca	cysteine-desulfurase-[Devosia-aurantiaca]	2714858
WP_157290569.1	cysteine-desulfurase	-	393	Bacteria>Proteobacteria>Alphaproteobacteria>Hyphomicrobiales>Devosiaceae	Devosia_marina	cysteine-desulfurase-[Devosia-marina]	2683198
WP_088343786.1	cysteine-desulfurase	-	399	Bacteria>Proteobacteria>Alphaproteobacteria>Hyphomicrobiales>Hyphomicrobiaceae	Rhodomicrobium	MULTISPECIES:-cysteine-desulfurase-DndA-[Rhodomicrobium]	1068
WP_245296897.1	cysteine-desulfurase	GCF_002198685.1	389	Bacteria>Proteobacteria>Alphaproteobacteria>Hyphomicrobiales>Hyphomicrobiaceae	Rhodomicrobium	MULTISPECIES:-cysteine-desulfurase-DndA-[Rhodomicrobium]	1068
CAA2136845.1	cysteine-desulfurase	-	392	Bacteria>Proteobacteria>Alphaproteobacteria>Hyphomicrobiales>Methylobacteriaceae	Methylobacterium_bullatum	Cysteine-desulfurase-IscS-[Methylobacterium-bullatum]	570505
WP_058619949.1	cysteine-desulfurase	-	390	Bacteria>Proteobacteria>Alphaproteobacteria>Hyphomicrobiales>Methylobacteriaceae	Methylobacterium_indicum	aminotransferase-class-V-fold-PLP-dependent-enzyme-[Methylobacterium-indicum]	1775910
WP_127729006.1	cysteine-desulfurase	GCF_004004555.2	382	Bacteria>Proteobacteria>Alphaproteobacteria>Hyphomicrobiales>Methylobacteriaceae	Methylobacterium_oryzihabitans	aminotransferase-class-V-fold-PLP-dependent-enzyme-[Methylobacterium-sp.-TER-1]	2499852
SFU78236.1	cysteine-desulfurase	GCA_900116815.1	388	Bacteria>Proteobacteria>Alphaproteobacteria>Hyphomicrobiales>Methylobacteriaceae	Methylobacterium_sp._174MFSha1.1	cysteine-desulfurase-[Methylobacterium-sp.-174MFSha1.1]	1502749
WP_093567330.1	cysteine-desulfurase	GCF_900116815.1	388	Bacteria>Proteobacteria>Alphaproteobacteria>Hyphomicrobiales>Methylobacteriaceae	Methylobacterium_sp._174MFSha1.1	cysteine-desulfurase-[Methylobacterium-sp.-174MFSha1.1]	1502749
MBP33151.1	cysteine-desulfurase	GCA_002727035.1	382	Bacteria>Proteobacteria>Alphaproteobacteria>Hyphomicrobiales>Methylobacteriaceae	Methylobacterium_sp.	cysteine-desulfurase-DndA-[Methylobacterium-sp.]	409
KQQ13677.1	cysteine-desulfurase	GCA_001423405.1	391	Bacteria>Proteobacteria>Alphaproteobacteria>Hyphomicrobiales>Methylobacteriaceae	Methylobacterium_sp._Leaf123	cysteine-desulfurase-DndA-[Methylobacterium-sp.-Leaf123]	1736264
WP_238508667.1	cysteine-desulfurase	GCF_022214735.1	393	Bacteria>Proteobacteria>Alphaproteobacteria>Hyphomicrobiales>Methylobacteriaceae	Methylorubrum_extorquens	aminotransferase-class-V-fold-PLP-dependent-enzyme-[Methylorubrum-extorquens]	408
WP_208069172.1	cysteine-desulfurase	GCF_017565685.3	388	Bacteria>Proteobacteria>Alphaproteobacteria>Hyphomicrobiales>Nitrobacteraceae	Bradyrhizobium_sp._38S5	aminotransferase-class-V-fold-PLP-dependent-enzyme-[Bradyrhizobium-sp.-38S5]	2819348
MBR1285373.1	cysteine-desulfurase	GCA_018130125.1	376	Bacteria>Proteobacteria>Alphaproteobacteria>Hyphomicrobiales>Nitrobacteraceae	Bradyrhizobium_sp._AUGA_SZCCT0177	cysteine-desulfurase-DndA-[Bradyrhizobium-sp.-AUGA-SZCCT0177]	2807665
WP_212267796.1	cysteine-desulfurase	-	376	Bacteria>Proteobacteria>Alphaproteobacteria>Hyphomicrobiales>Nitrobacteraceae	Bradyrhizobium_sp._AUGA_SZCCT0177	cysteine-desulfurase-DndA-[Bradyrhizobium-sp.-AUGA-SZCCT0177]	2807665
WP_247356733.1	cysteine-desulfurase	GCF_023100905.1	388	Bacteria>Proteobacteria>Alphaproteobacteria>Hyphomicrobiales>Nitrobacteraceae	unclassified_Bradyrhizobium	MULTISPECIES:-aminotransferase-class-V-fold-PLP-dependent-enzyme-[unclassified-Bradyrhizobium]	2631580
WP_207621501.1	cysteine-desulfurase	GCF_011317445.1	393	Bacteria>Proteobacteria>Alphaproteobacteria>Hyphomicrobiales>Phyllobacteriaceae	Chelativorans_multitrophicus	MULTISPECIES:-cysteine-desulfurase-DndA-[Chelativorans]	449973
WP_006204723.1	cysteine-desulfurase	GCF_000233995.1	382	Bacteria>Proteobacteria>Alphaproteobacteria>Hyphomicrobiales>Phyllobacteriaceae	Mesorhizobium_amorphae	cysteine-desulfurase-[Mesorhizobium-amorphae]	71433
WP_192355953.1	cysteine-desulfurase	-	414	Bacteria>Proteobacteria>Alphaproteobacteria>Hyphomicrobiales>Phyllobacteriaceae	Mesorhizobium_mediterraneum	cysteine-desulfurase-[Mesorhizobium-mediterraneum]	43617
WP_095795929.1	cysteine-desulfurase	GCF_002294705.1	391	Bacteria>Proteobacteria>Alphaproteobacteria>Hyphomicrobiales>Phyllobacteriaceae	Mesorhizobium	MULTISPECIES:-aminotransferase-class-V-fold-PLP-dependent-enzyme-[Mesorhizobium]	68287
TIW10979.1	cysteine-desulfurase	GCA_005063115.1	385	Bacteria>Proteobacteria>Alphaproteobacteria>Hyphomicrobiales>Phyllobacteriaceae	Mesorhizobium_sp.	aminotransferase-class-V-fold-PLP-dependent-enzyme-[Mesorhizobium-sp.]	1871066
RWD39942.1	cysteine-desulfurase	GCA_004016995.1	89	Bacteria>Proteobacteria>Alphaproteobacteria>Hyphomicrobiales>Phyllobacteriaceae	Mesorhizobium_sp.	aminotransferase,-partial-[Mesorhizobium-sp.]	1871066
WP_141841106.1	cysteine-desulfurase	GCF_006658505.1	391	Bacteria>Proteobacteria>Alphaproteobacteria>Hyphomicrobiales>Phyllobacteriaceae	Mesorhizobium_sp._B2-4-15	aminotransferase-class-V-fold-PLP-dependent-enzyme-[Mesorhizobium-sp.-B2-4-15]	2589934
RWL89319.1	cysteine-desulfurase	GCA_004020305.1	388	Bacteria>Proteobacteria>Alphaproteobacteria>Hyphomicrobiales>Phyllobacteriaceae	Mesorhizobium_sp.	cysteine-desulfurase-DndA-[Mesorhizobium-sp.]	1871066
RWO22203.1	cysteine-desulfurase	GCA_004021465.1	393	Bacteria>Proteobacteria>Alphaproteobacteria>Hyphomicrobiales>Phyllobacteriaceae	Mesorhizobium_sp.	cysteine-desulfurase-DndA-[Mesorhizobium-sp.]	1871066
RWQ46778.1	cysteine-desulfurase	GCA_004021735.1	393	Bacteria>Proteobacteria>Alphaproteobacteria>Hyphomicrobiales>Phyllobacteriaceae	Mesorhizobium_sp.	cysteine-desulfurase-DndA-[Mesorhizobium-sp.]	1871066
TIV86454.1	cysteine-desulfurase	GCA_004964035.1	388	Bacteria>Proteobacteria>Alphaproteobacteria>Hyphomicrobiales>Phyllobacteriaceae	Mesorhizobium_sp.	cysteine-desulfurase-DndA-[Mesorhizobium-sp.]	1871066
RWA59261.1	cysteine-desulfurase	GCA_004016595.1	385	Bacteria>Proteobacteria>Alphaproteobacteria>Hyphomicrobiales>Phyllobacteriaceae	Mesorhizobium_sp.	cysteine-desulfurase-[Mesorhizobium-sp.]	1871066
RWD27519.1	cysteine-desulfurase	GCA_004017075.1	385	Bacteria>Proteobacteria>Alphaproteobacteria>Hyphomicrobiales>Phyllobacteriaceae	Mesorhizobium_sp.	cysteine-desulfurase-[Mesorhizobium-sp.]	1871066
RWN94630.1	cysteine-desulfurase	GCA_004021525.1	390	Bacteria>Proteobacteria>Alphaproteobacteria>Hyphomicrobiales>Phyllobacteriaceae	Mesorhizobium_sp.	cysteine-desulfurase-[Mesorhizobium-sp.]	1871066
RWP03126.1	cysteine-desulfurase	GCA_004021085.1	402	Bacteria>Proteobacteria>Alphaproteobacteria>Hyphomicrobiales>Phyllobacteriaceae	Mesorhizobium_sp.	cysteine-desulfurase-[Mesorhizobium-sp.]	1871066
RWQ12709.1	cysteine-desulfurase	GCA_004021905.1	428	Bacteria>Proteobacteria>Alphaproteobacteria>Hyphomicrobiales>Phyllobacteriaceae	Mesorhizobium_sp.	cysteine-desulfurase-[Mesorhizobium-sp.]	1871066
TIM30552.1	cysteine-desulfurase	GCA_004961485.1	385	Bacteria>Proteobacteria>Alphaproteobacteria>Hyphomicrobiales>Phyllobacteriaceae	Mesorhizobium_sp.	cysteine-desulfurase-[Mesorhizobium-sp.]	1871066
TIN32548.1	cysteine-desulfurase	GCA_004961445.1	390	Bacteria>Proteobacteria>Alphaproteobacteria>Hyphomicrobiales>Phyllobacteriaceae	Mesorhizobium_sp.	cysteine-desulfurase-[Mesorhizobium-sp.]	1871066
TIP92995.1	cysteine-desulfurase	GCA_004962305.1	390	Bacteria>Proteobacteria>Alphaproteobacteria>Hyphomicrobiales>Phyllobacteriaceae	Mesorhizobium_sp.	cysteine-desulfurase-[Mesorhizobium-sp.]	1871066
WP_119819802.1	cysteine-desulfurase	GCF_003590645.1	391	Bacteria>Proteobacteria>Alphaproteobacteria>Hyphomicrobiales>Phyllobacteriaceae	Mesorhizobium_sp._DCY119	aminotransferase-class-V-fold-PLP-dependent-enzyme-[Mesorhizobium-sp.-DCY119]	2108445
WP_201413877.1	cysteine-desulfurase	GCF_016591715.1	394	Bacteria>Proteobacteria>Alphaproteobacteria>Hyphomicrobiales>Phyllobacteriaceae	Mesorhizobium_sp._J8	cysteine-desulfurase-[Mesorhizobium-sp.-J8]	2777475
WP_051407886.1	cysteine-desulfurase	GCF_000502375.1	250	Bacteria>Proteobacteria>Alphaproteobacteria>Hyphomicrobiales>Phyllobacteriaceae	Mesorhizobium_sp._L48C026A00	aminotransferase-class-V-fold-PLP-dependent-enzyme-[Mesorhizobium-sp.-L48C026A00]	1287182
WP_083354723.1	cysteine-desulfurase	GCF_001854795.1	378	Bacteria>Proteobacteria>Alphaproteobacteria>Hyphomicrobiales>Phyllobacteriaceae	Mesorhizobium_sp._LCM_4577	aminotransferase-class-V-fold-PLP-dependent-enzyme-[Mesorhizobium-sp.-LCM-4577]	1848288
WP_128371611.1	cysteine-desulfurase	GCF_004023325.1	385	Bacteria>Proteobacteria>Alphaproteobacteria>Hyphomicrobiales>Phyllobacteriaceae	Mesorhizobium_sp._M1A.F.Ca.ET.072.01.1.1	cysteine-desulfurase-[Mesorhizobium-sp.-M1A.F.Ca.ET.072.01.1.1]	2496753
WP_127221118.1	cysteine-desulfurase	GCF_003998155.1	385	Bacteria>Proteobacteria>Alphaproteobacteria>Hyphomicrobiales>Phyllobacteriaceae	Mesorhizobium_sp._M1E.F.Ca.ET.063.01.1.1	cysteine-desulfurase-[Mesorhizobium-sp.-M1E.F.Ca.ET.063.01.1.1]	2496750
WP_189605665.1	cysteine-desulfurase	GCF_003998955.1	378	Bacteria>Proteobacteria>Alphaproteobacteria>Hyphomicrobiales>Phyllobacteriaceae	Mesorhizobium_sp._M4B.F.Ca.ET.017.02.2.1	cysteine-desulfurase-[Mesorhizobium-sp.-M4B.F.Ca.ET.017.02.2.1]	2496649
WP_149801931.1	cysteine-desulfurase	GCF_900115245.1	402	Bacteria>Proteobacteria>Alphaproteobacteria>Hyphomicrobiales>Phyllobacteriaceae	Mesorhizobium_sp._NFR06	cysteine-desulfurase-[Mesorhizobium-sp.-NFR06]	1566290
WP_027168787.1	cysteine-desulfurase	GCF_000427725.1	390	Bacteria>Proteobacteria>Alphaproteobacteria>Hyphomicrobiales>Phyllobacteriaceae	Mesorhizobium_sp._WSM3224	cysteine-desulfurase-[Mesorhizobium-sp.-WSM3224]	1040986
WP_206075248.1	cysteine-desulfurase	GCF_003863365.1	403	Bacteria>Proteobacteria>Alphaproteobacteria>Hyphomicrobiales>Phyllobacteriaceae	Mesorhizobium_tamadayense	cysteine-desulfurase-[Mesorhizobium-tamadayense]	425306
WP_235981967.1	cysteine-desulfurase	GCF_015904235.1	363	Bacteria>Proteobacteria>Alphaproteobacteria>Hyphomicrobiales>Pleomorphomonadaceae	Methylobrevis_albus	cysteine-desulfurase-[Methylobrevis-albus]	2793297
WP_027676393.1	cysteine-desulfurase	GCF_005145045.1	392	Bacteria>Proteobacteria>Alphaproteobacteria>Hyphomicrobiales>Rhizobiaceae	Agrobacterium_larrymoorei	cysteine-desulfurase-DndA-[Agrobacterium-larrymoorei]	160699
WP_059755017.1	cysteine-desulfurase	GCF_001526505.1	387	Bacteria>Proteobacteria>Alphaproteobacteria>Hyphomicrobiales>Rhizobiaceae	Agrobacterium	MULTISPECIES:-aminotransferase-class-V-fold-PLP-dependent-enzyme-[Agrobacterium]	357
WP_234908900.1	cysteine-desulfurase	GCF_013321135.1	357	Bacteria>Proteobacteria>Alphaproteobacteria>Hyphomicrobiales>Rhizobiaceae	Agrobacterium_rhizogenes	cysteine-desulfurase-[Agrobacterium-rhizogenes]	359
WP_019566299.1	cysteine-desulfurase	GCF_000381165.1	387	Bacteria>Proteobacteria>Alphaproteobacteria>Hyphomicrobiales>Rhizobiaceae	Agrobacterium_sp._10MFCol1.1	cysteine-desulfurase-[Agrobacterium-sp.-10MFCol1.1]	1150775
KVK54335.1	cysteine-desulfurase	GCA_001526505.1	387	Bacteria>Proteobacteria>Alphaproteobacteria>Hyphomicrobiales>Rhizobiaceae	Agrobacterium_sp._D14	aminotransferase-class-V-[Agrobacterium-sp.-D14]	1336743
WP_081397250.1	cysteine-desulfurase	GCF_001975795.1	399	Bacteria>Proteobacteria>Alphaproteobacteria>Hyphomicrobiales>Rhizobiaceae	Agrobacterium_tumefaciens	cysteine-desulfurase-[Agrobacterium-tumefaciens]	358
WP_052637087.1	cysteine-desulfurase	GCF_000967425.1	387	Bacteria>Proteobacteria>Alphaproteobacteria>Hyphomicrobiales>Rhizobiaceae	Pseudorhizobium_banfieldiae	cysteine-desulfurase-[Pseudorhizobium-banfieldiae]	1125847
NNU46287.1	cysteine-desulfurase	GCA_013087625.1	395	Bacteria>Proteobacteria>Alphaproteobacteria>Hyphomicrobiales>Rhizobiaceae	Rhizobium_changzhiense	cysteine-desulfurase-[Rhizobium-changzhiense]	2692317
WP_206679621.1	cysteine-desulfurase	GCF_013087625.1	389	Bacteria>Proteobacteria>Alphaproteobacteria>Hyphomicrobiales>Rhizobiaceae	Rhizobium_changzhiense	cysteine-desulfurase-[Rhizobium-changzhiense]	2692317
WP_004679895.1	cysteine-desulfurase	GCF_000194195.1	387	Bacteria>Proteobacteria>Alphaproteobacteria>Hyphomicrobiales>Rhizobiaceae	Rhizobium_etli	MULTISPECIES:-cysteine-desulfurase-[Rhizobium]	29449
WP_168337898.1	cysteine-desulfurase	GCF_012275885.1	387	Bacteria>Proteobacteria>Alphaproteobacteria>Hyphomicrobiales>Rhizobiaceae	Rhizobium_laguerreae	cysteine-desulfurase-[Rhizobium-laguerreae]	1076926
WP_130787521.1	cysteine-desulfurase	GCF_004306655.1	388	Bacteria>Proteobacteria>Alphaproteobacteria>Hyphomicrobiales>Rhizobiaceae	Rhizobium_leguminosarum	aminotransferase-class-V-fold-PLP-dependent-enzyme-[Rhizobium-leguminosarum]	384
WP_222381175.1	cysteine-desulfurase	-	393	Bacteria>Proteobacteria>Alphaproteobacteria>Hyphomicrobiales>Rhizobiaceae	Rhizobium_leguminosarum	aminotransferase-class-V-fold-PLP-dependent-enzyme-[Rhizobium-leguminosarum]	384
WP_025398073.1	cysteine-desulfurase	GCF_000517605.1	387	Bacteria>Proteobacteria>Alphaproteobacteria>Hyphomicrobiales>Rhizobiaceae	Rhizobium_leguminosarum	cysteine-desulfurase-[Rhizobium-leguminosarum]	384
WP_131702499.1	cysteine-desulfurase	GCF_019778265.1	387	Bacteria>Proteobacteria>Alphaproteobacteria>Hyphomicrobiales>Rhizobiaceae	Rhizobium_leguminosarum	cysteine-desulfurase-[Rhizobium-leguminosarum]	384
WP_235777141.1	cysteine-desulfurase	GCF_000427925.1	372	Bacteria>Proteobacteria>Alphaproteobacteria>Hyphomicrobiales>Rhizobiaceae	Rhizobium_mesoamericanum	aminotransferase-class-V-fold-PLP-dependent-enzyme-[Rhizobium-mesoamericanum]	1079800
WP_220367278.1	cysteine-desulfurase	GCF_019511305.1	382	Bacteria>Proteobacteria>Alphaproteobacteria>Hyphomicrobiales>Rhizobiaceae	Rhizobium_pusense	aminotransferase-class-V-fold-PLP-dependent-enzyme-[Rhizobium-pusense]	648995
MBP2614106.1	cysteine-desulfurase	GCA_017877395.1	387	Bacteria>Proteobacteria>Alphaproteobacteria>Hyphomicrobiales>Rhizobiaceae	Rhizobium_pusense	cysteine-desulfurase-[Rhizobium-pusense]	648995
WP_209914617.1	cysteine-desulfurase	GCF_017877395.1	387	Bacteria>Proteobacteria>Alphaproteobacteria>Hyphomicrobiales>Rhizobiaceae	Rhizobium_pusense	cysteine-desulfurase-[Rhizobium-pusense]	648995
SOD50248.1	cysteine-desulfurase	GCA_900215635.1	378	Bacteria>Proteobacteria>Alphaproteobacteria>Hyphomicrobiales>Rhizobiaceae	Rhizobium_sp._AN6A	cysteine-desulfurase-[Rhizobium-sp.-AN6A]	1841611
TKV70607.1	cysteine-desulfurase	GCA_005298145.1	381	Bacteria>Proteobacteria>Alphaproteobacteria>Hyphomicrobiales>Rhizobiaceae	Rhizobium_sp._AU243	cysteine-desulfurase-DndA-[Rhizobium-sp.-AU243]	2303425
KQM32526.1	cysteine-desulfurase	GCA_001421235.1	387	Bacteria>Proteobacteria>Alphaproteobacteria>Hyphomicrobiales>Rhizobiaceae	Rhizobium_sp._Leaf202	hypothetical-protein-ASE62_13120-[Rhizobium-sp.-Leaf202]	1735675
WP_085780254.1	cysteine-desulfurase	GCF_002117485.1	387	Bacteria>Proteobacteria>Alphaproteobacteria>Hyphomicrobiales>Rhizobiaceae	Rhizobium_sp._NXC14	cysteine-desulfurase-[Rhizobium-sp.-NXC14]	1981173
TCP80778.1	cysteine-desulfurase	GCA_004341455.1	382	Bacteria>Proteobacteria>Alphaproteobacteria>Hyphomicrobiales>Rhizobiaceae	Rhizobium_sp._PP-CC-2G-626	cysteine-desulfurase-IscS-[Rhizobium-sp.-PP-CC-2G-626]	2135646
WP_132714634.1	cysteine-desulfurase	GCF_004341455.1	382	Bacteria>Proteobacteria>Alphaproteobacteria>Hyphomicrobiales>Rhizobiaceae	Rhizobium_sp._PP-CC-2G-626	cysteine-desulfurase-[Rhizobium-sp.-PP-CC-2G-626]	2135646
WP_097588013.1	cysteine-desulfurase	GCF_002531965.1	386	Bacteria>Proteobacteria>Alphaproteobacteria>Hyphomicrobiales>Rhizobiaceae	Sinorhizobium_fredii	cysteine-desulfurase-[Sinorhizobium-fredii]	380
PDT43969.1	cysteine-desulfurase	GCA_002531965.1	386	Bacteria>Proteobacteria>Alphaproteobacteria>Hyphomicrobiales>Rhizobiaceae	Sinorhizobium_fredii	IscS-subfamily-cysteine-desulfurase-[Sinorhizobium-fredii]	380
WP_234839312.1	cysteine-desulfurase	GCF_004002725.1	392	Bacteria>Proteobacteria>Alphaproteobacteria>Hyphomicrobiales>Rhizobiaceae	Sinorhizobium_medicae	cysteine-desulfurase-[Sinorhizobium-medicae]	110321
WP_018099625.1	cysteine-desulfurase	GCF_000375585.1	399	Bacteria>Proteobacteria>Alphaproteobacteria>Hyphomicrobiales>Rhizobiaceae	Sinorhizobium_meliloti	cysteine-desulfurase-DndA-[Sinorhizobium-meliloti]	382
WP_127672679.1	cysteine-desulfurase	GCF_004004675.1	387	Bacteria>Proteobacteria>Alphaproteobacteria>Hyphomicrobiales>Rhizobiaceae	Sinorhizobium_meliloti	cysteine-desulfurase-[Sinorhizobium-meliloti]	382
WP_065785422.1	cysteine-desulfurase	GCF_001695905.1	386	Bacteria>Proteobacteria>Alphaproteobacteria>Hyphomicrobiales>Rhizobiaceae	unclassified_Ensifer	MULTISPECIES:-cysteine-desulfurase-[unclassified-Ensifer]	2633371
WP_200342397.1	cysteine-desulfurase	-	376	Bacteria>Proteobacteria>Alphaproteobacteria>Hyphomicrobiales>Rhodovibrionaceae	Rhodovibrio_sodomensis	cysteine-desulfurase-DndA-[Rhodovibrio-sodomensis]	1088
MBG6162803.1	cysteine-desulfurase	GCA_015752055.1	392	Bacteria>Proteobacteria>Alphaproteobacteria>Hyphomicrobiales>Stappiaceae	Labrenzia_sp._EL_195	cysteine-desulfurase-[Labrenzia-sp.-EL_195]	2787743
MBZ0259263.1	cysteine-desulfurase	GCA_019912605.1	391	Bacteria>Proteobacteria>Alphaproteobacteria>Hyphomicrobiales>unclassified_Hyphomicrobiales	Hyphomicrobiales_bacterium	cysteine-desulfurase-[Hyphomicrobiales-bacterium]	1909294
OYX10066.1	cysteine-desulfurase	GCA_002280205.1	80	Bacteria>Proteobacteria>Alphaproteobacteria>Hyphomicrobiales>unclassified_Hyphomicrobiales	Rhizobiales_bacterium_32-66-8	cysteine-desulfurase-DndA,-partial-[Rhizobiales-bacterium-32-66-8]	1970550
WP_206741513.1	cysteine-desulfurase	GCF_000685295.1	374	Bacteria>Proteobacteria>Alphaproteobacteria>Hyphomonadales>Hyphomonadaceae	Hyphomonas_oceanitis	cysteine-desulfurase-[Hyphomonas-oceanitis]	81033
KDA04227.1	cysteine-desulfurase	GCA_000685295.1	387	Bacteria>Proteobacteria>Alphaproteobacteria>Hyphomonadales>Hyphomonadaceae	Hyphomonas_oceanitis_SCH89	cysteine-desulfurase-[Hyphomonas-oceanitis-SCH89]	1280953
WP_114106407.1	cysteine-desulfurase	GCF_003325995.1	391	Bacteria>Proteobacteria>Alphaproteobacteria>Hyphomonadales>Hyphomonadaceae	Hyphomonas_sp._CACIAM_19H1	aminotransferase-class-V-fold-PLP-dependent-enzyme-[Hyphomonas-sp.-CACIAM-19H1]	1873716
WP_121940095.1	cysteine-desulfurase	GCF_003688555.1	389	Bacteria>Proteobacteria>Alphaproteobacteria>Kordiimonadales>Kordiimonadaceae	Eilatimonas_milleporae	cysteine-desulfurase-DndA-[Eilatimonas-milleporae]	911205
WP_208393960.1	cysteine-desulfurase	GCF_011762045.1	388	Bacteria>Proteobacteria>Alphaproteobacteria>Micropepsales>Micropepsaceae	Rhizomicrobium_electricum	aminotransferase-class-V-fold-PLP-dependent-enzyme-[Rhizomicrobium-electricum]	480070
WP_206548288.1	cysteine-desulfurase	GCF_017255295.1	393	Bacteria>Proteobacteria	Alphaproteobacteria	MULTISPECIES:-cysteine-desulfurase-[Alphaproteobacteria]	28211
AFS47939.1	cysteine-desulfurase	GCA_000299095.1	377	Bacteria>Proteobacteria>Alphaproteobacteria>Pelagibacterales>Pelagibacteraceae	alpha_proteobacterium_HIMB5	cysteine-desulfurase-DndA-[alpha-proteobacterium-HIMB5]	859653
WP_006996742.1	cysteine-desulfurase	GCF_000153525.1	378	Bacteria>Proteobacteria>Alphaproteobacteria>Pelagibacterales>Pelagibacteraceae	Candidatus_Pelagibacter_ubique	cysteine-desulfurase-DndA-[Candidatus-Pelagibacter-ubique]	198252
MBL6758241.1	cysteine-desulfurase	GCA_016779055.1	377	Bacteria>Proteobacteria>Alphaproteobacteria>Pelagibacterales>Pelagibacteraceae	Pelagibacteraceae_bacterium	cysteine-desulfurase-DndA-[Pelagibacteraceae-bacterium]	2026775
MAC40839.1	cysteine-desulfurase	GCA_002684605.1	379	Bacteria>Proteobacteria>Alphaproteobacteria>Pelagibacterales>unclassified_Pelagibacterales	Pelagibacterales_bacterium	IscS-subfamily-cysteine-desulfurase-[Pelagibacterales-bacterium]	2026776
WP_055187250.1	cysteine-desulfurase	GCF_900116725.1	390	Bacteria>Proteobacteria>Alphaproteobacteria>Rhodobacterales>Rhodobacteraceae	Aliiroseovarius_crassostreae	cysteine-desulfurase-[Aliiroseovarius-crassostreae]	154981
WP_133489744.1	cysteine-desulfurase	GCF_004360145.1	379	Bacteria>Proteobacteria>Alphaproteobacteria>Rhodobacterales>Rhodobacteraceae	Aliiroseovarius_marinus	aminotransferase-class-V-fold-PLP-dependent-enzyme-[Aliiroseovarius-marinus]	2500159
THD81589.1	cysteine-desulfurase	GCA_004799285.1	379	Bacteria>Proteobacteria>Alphaproteobacteria>Rhodobacterales>Rhodobacteraceae	Gemmobacter_aestuarii	aminotransferase-class-V-fold-PLP-dependent-enzyme-[Gemmobacter-aestuarii]	1445661
WP_211092134.1	cysteine-desulfurase	GCF_004799285.1	419	Bacteria>Proteobacteria>Alphaproteobacteria>Rhodobacterales>Rhodobacteraceae	Gemmobacter_aestuarii	aminotransferase-class-V-fold-PLP-dependent-enzyme-[Gemmobacter-aestuarii]	1445661
MCC5968799.1	cysteine-desulfurase	GCA_020832545.1	379	Bacteria>Proteobacteria>Alphaproteobacteria>Rhodobacterales>Rhodobacteraceae	Pararhodobacter_sp.	aminotransferase-class-V-fold-PLP-dependent-enzyme-[Pararhodobacter-sp.]	2127056
WP_166195108.1	cysteine-desulfurase	GCF_011290545.1	387	Bacteria>Proteobacteria>Alphaproteobacteria>Rhodobacterales>Rhodobacteraceae	Pontibrevibacter_nitratireducens	cysteine-desulfurase-[Monaibacterium-sp.-ALG8]	2758038
QIK42413.1	cysteine-desulfurase	GCA_011290545.1	387	Bacteria>Proteobacteria>Alphaproteobacteria>Rhodobacterales>Rhodobacteraceae	Pontibrevibacter_nitratireducens	cysteine-desulfurase-[Pontibrevibacter-nitratireducens]	2758038
WP_110553836.1	cysteine-desulfurase	GCF_001205715.1	390	Bacteria>Proteobacteria>Alphaproteobacteria>Rhodobacterales>Rhodobacteraceae	Pseudaestuariivita_atlantica	cysteine-desulfurase-[Pseudaestuariivita-atlantica]	1317121
WP_136637621.1	cysteine-desulfurase	GCF_004959925.1	379	Bacteria>Proteobacteria>Alphaproteobacteria>Rhodobacterales>Rhodobacteraceae	Pseudooceanicola_onchidii	aminotransferase-class-V-fold-PLP-dependent-enzyme-[Pseudooceanicola-onchidii]	2562279
MBB04748.1	cysteine-desulfurase	GCA_002709085.1	232	Bacteria>Proteobacteria>Alphaproteobacteria>Rhodobacterales>Rhodobacteraceae	Pseudooceanicola_sp.	cysteine-desulfurase-DndA-[Pseudooceanicola-sp.]	1914328
WP_050524081.1	cysteine-desulfurase	GCF_001202035.1	390	Bacteria>Proteobacteria>Alphaproteobacteria>Rhodobacterales>Rhodobacteraceae	Pseudorhodobacter_wandonensis	cysteine-desulfurase-[Pseudorhodobacter-wandonensis]	1120568
RFP86247.1	cysteine-desulfurase	GCA_003417655.1	396	Bacteria>Proteobacteria>Alphaproteobacteria>Rhodobacterales>Rhodobacteraceae	Rhodobacteraceae_bacterium_63075	cysteine-desulfurase-[Rhodobacteraceae-bacterium-63075]	2301226
PIV79052.1	cysteine-desulfurase	GCA_002783205.1	379	Bacteria>Proteobacteria>Alphaproteobacteria>Rhodobacterales>Rhodobacteraceae	Rhodobacteraceae_bacterium_CG17_big_fil_post_rev_8_21_14_2_50_63_15	cysteine-desulfurase-DndA-[Rhodobacteraceae-bacterium-CG17_big_fil_post_rev_8_21_14_2_50_63_15]	1974082
WP_078574467.1	cysteine-desulfurase	GCF_002020135.1	396	Bacteria>Proteobacteria>Alphaproteobacteria>Rhodobacterales>Rhodobacteraceae	Thioclava_marina	cysteine-desulfurase-[Thioclava-marina]	1915077
WP_038074692.1	cysteine-desulfurase	GCF_000714535.1	396	Bacteria>Proteobacteria>Alphaproteobacteria>Rhodobacterales>Rhodobacteraceae	Thioclava_pacifica	cysteine-desulfurase-[Thioclava-pacifica]	285109

Accession	Annotation	GCA	Length	Phyletic.spread	Strain.name	Defline	Species.Taxid
KEO54664.1	cysteine-desulfurase	GCA_000714535.1	336	Bacteria>Proteobacteria>Alphaproteobacteria>Rhodobacterales>Rhodobacteraceae	Thioclava_pacifica_DSM_10166	hypothetical-protein-TP2_17495-[Thioclava-pacifica-DSM-10166]	1353537
WP_226551982.1	cysteine-desulfurase	GCF_020524585.1	390	Bacteria>Proteobacteria>Alphaproteobacteria>Rhodobacterales>Roseobacteraceae	Celeribacter_naphthalenivorans	cysteine-desulfurase-[Celeribacter-naphthalenivorans]	1614694
WP_090063040.1	cysteine-desulfurase	GCF_900113955.1	387	Bacteria>Proteobacteria>Alphaproteobacteria>Rhodobacterales>Roseobacteraceae	Celeribacter_neptunius	cysteine-desulfurase-[Celeribacter-neptunius]	588602
WP_111535612.1	cysteine-desulfurase	GCF_003253995.1	376	Bacteria>Proteobacteria>Alphaproteobacteria>Rhodobacterales>Roseobacteraceae	Hwanghaeicola_aestuarii	cysteine-desulfurase-DndA-[Hwanghaeicola-aestuarii]	568105
PZX19965.1	cysteine-desulfurase	GCA_003253995.1	376	Bacteria>Proteobacteria>Alphaproteobacteria>Rhodobacterales>Roseobacteraceae	Hwanghaeicola_aestuarii	cysteine-desulfurase-[Hwanghaeicola-aestuarii]	568105
WP_092765166.1	cysteine-desulfurase	GCF_900109285.1	390	Bacteria>Proteobacteria>Alphaproteobacteria>Rhodobacterales>Roseobacteraceae	Jannaschia_helgolandensis	cysteine-desulfurase-[Jannaschia-helgolandensis]	188906
PWE34117.1	cysteine-desulfurase	GCA_003122205.1	389	Bacteria>Proteobacteria>Alphaproteobacteria>Rhodobacterales>Roseobacteraceae	Maritimibacter_sp._55A14	cysteine-desulfurase-DndA-[Maritimibacter-sp.-55A14]	2174844
WP_199221268.1	cysteine-desulfurase	GCF_003122205.1	424	Bacteria>Proteobacteria>Alphaproteobacteria>Rhodobacterales>Roseobacteraceae	Maritimibacter_sp._55A14	cysteine-desulfurase-DndA-[Maritimibacter-sp.-55A14]	2174844
WP_227363073.1	cysteine-desulfurase	GCF_020622295.1	387	Bacteria>Proteobacteria>Alphaproteobacteria>Rhodobacterales>Roseobacteraceae	Phaeobacter_sp._MR	cysteine-desulfurase-[Phaeobacter-sp.-MR]	2763265
WP_068110635.1	cysteine-desulfurase	GCF_001509585.1	379	Bacteria>Proteobacteria>Alphaproteobacteria>Rhodobacterales>Roseobacteraceae	Pseudoruegeria_marinistellae	cysteine-desulfurase-DndA-[Pseudoruegeria-marinistellae]	1739787
AVL53635.1	cysteine-desulfurase	GCA_002983865.1	387	Bacteria>Proteobacteria>Alphaproteobacteria>Rhodobacterales>Roseobacteraceae	Roseobacter_denitrificans	cysteine-desulfurase-[Roseobacter-denitrificans]	2434
WP_011567102.1	cysteine-desulfurase	GCF_000014045.1	387	Bacteria>Proteobacteria>Alphaproteobacteria>Rhodobacterales>Roseobacteraceae	Roseobacter_denitrificans	cysteine-desulfurase-[Roseobacter-denitrificans]	2434
SFF73282.1	cysteine-desulfurase	GCA_900113215.1	387	Bacteria>Proteobacteria>Alphaproteobacteria>Rhodobacterales>Roseobacteraceae	Roseobacter_denitrificans_OCh_114	cysteine-desulfurase-IscS-[Roseobacter-denitrificans-OCh-114]	375451
ABG30480.1	cysteine-desulfurase	GCA_000014045.1	387	Bacteria>Proteobacteria>Alphaproteobacteria>Rhodobacterales>Roseobacteraceae	Roseobacter_denitrificans_OCh_114	cysteine-desulfurase-[Roseobacter-denitrificans-OCh-114]	375451
NHQ74521.1	cysteine-desulfurase	GCA_011601345.1	387	Bacteria>Proteobacteria>Alphaproteobacteria>Rhodobacterales>Roseobacteraceae	Roseovarius_gahaiensis	cysteine-desulfurase-[Roseovarius-gahaiensis]	2716691
WP_167195880.1	cysteine-desulfurase	GCF_011601345.1	387	Bacteria>Proteobacteria>Alphaproteobacteria>Rhodobacterales>Roseobacteraceae	Roseovarius_gahaiensis	cysteine-desulfurase-[Roseovarius-gahaiensis]	2716691
WP_051520980.1	cysteine-desulfurase	GCF_000600335.2	375	Bacteria>Proteobacteria>Alphaproteobacteria>Rhodobacterales>Roseobacteraceae	Rubellimicrobium_mesophilum	cysteine-desulfurase-[Rubellimicrobium-mesophilum]	1123067
WP_170368872.1	cysteine-desulfurase	GCF_013030305.1	396	Bacteria>Proteobacteria>Alphaproteobacteria>Rhodobacterales>Roseobacteraceae	Ruegeria_arenilitoris	cysteine-desulfurase-[Ruegeria-arenilitoris]	1173585
WP_170394275.1	cysteine-desulfurase	-	381	Bacteria>Proteobacteria>Alphaproteobacteria>Rhodobacterales>Roseobacteraceae	Ruegeria_arenilitoris	cysteine-desulfurase-[Ruegeria-arenilitoris]	1173585
NOE18081.1	cysteine-desulfurase	GCA_013110705.1	387	Bacteria>Proteobacteria>Alphaproteobacteria>Rhodobacterales>Roseobacteraceae	Ruegeria_atlantica	aminotransferase-class-V-fold-PLP-dependent-enzyme-[Ruegeria-atlantica]	81569
WP_171329447.1	cysteine-desulfurase	GCF_013110705.1	387	Bacteria>Proteobacteria>Alphaproteobacteria>Rhodobacterales>Roseobacteraceae	Ruegeria_atlantica	aminotransferase-class-V-fold-PLP-dependent-enzyme-[Ruegeria-atlantica]	81569
WP_234136630.1	cysteine-desulfurase	GCF_021405385.1	396	Bacteria>Proteobacteria>Alphaproteobacteria>Rhodobacterales>Roseobacteraceae	Ruegeria_pomeroyi	aminotransferase-class-V-fold-PLP-dependent-enzyme-[Ruegeria-pomeroyi]	89184
WP_171172909.1	cysteine-desulfurase	GCF_013034525.1	396	Bacteria>Proteobacteria>Alphaproteobacteria>Rhodobacterales>Roseobacteraceae	Ruegeria_sp._HKCCA5929	cysteine-desulfurase-[Ruegeria-sp.-HKCCA5929]	2682988
WP_152460867.1	cysteine-desulfurase	GCF_009363615.1	387	Bacteria>Proteobacteria>Alphaproteobacteria>Rhodobacterales>Roseobacteraceae	Ruegeria_sp._THAF33	cysteine-desulfurase-[Ruegeria-sp.-THAF33]	2587853
QFT75875.1	cysteine-desulfurase	GCA_009363615.1	387	Bacteria>Proteobacteria>Alphaproteobacteria>Rhodobacterales>Roseobacteraceae	Ruegeria_sp._THAF33	Cysteine-desulfurase-[Ruegeria-sp.-THAF33]	2587853
EEW56583.1	cysteine-desulfurase	GCA_000161815.1	390	Bacteria>Proteobacteria>Alphaproteobacteria>Rhodobacterales>Roseobacteraceae	Ruegeria_sp._TrichCH4B	cysteine-desulfurase-[Ruegeria-sp.-TrichCH4B]	644076
WP_076695350.1	cysteine-desulfurase	GCF_001975705.1	389	Bacteria>Proteobacteria>Alphaproteobacteria>Rhodobacterales>Roseobacteraceae	Salipiger_abyssi	cysteine-desulfurase-[Salipiger-abyssi]	1250539
WP_028957475.1	cysteine-desulfurase	GCF_000620505.1	390	Bacteria>Proteobacteria>Alphaproteobacteria>Rhodobacterales>Roseobacteraceae	Sulfitobacter_sp._20_GPM-1509m	cysteine-desulfurase-[Sulfitobacter-sp.-20_GPM-1509m]	1380367
QUJ78345.1	cysteine-desulfurase	GCA_018200035.1	386	Bacteria>Proteobacteria>Alphaproteobacteria>Rhodobacterales>Roseobacteraceae	Sulfitobacter_sp._JK7-1	cysteine-desulfurase-[Sulfitobacter-sp.-JK7-1]	2829501
WP_212706537.1	cysteine-desulfurase	GCF_018200035.1	386	Bacteria>Proteobacteria>Alphaproteobacteria>Rhodobacterales>Roseobacteraceae	Sulfitobacter_sp._JK7-1	cysteine-desulfurase-[Sulfitobacter-sp.-JK7-1]	2829501
WP_197918473.1	cysteine-desulfurase	GCF_016019955.1	387	Bacteria>Proteobacteria>Alphaproteobacteria>Rhodobacterales>Roseobacteraceae	Thiosulfatihalobacter_marinus	cysteine-desulfurase-[Pseudoruegeria-sp.-GL-11-2]	2792481
WP_129634592.1	cysteine-desulfurase	GCF_004146235.1	386	Bacteria>Proteobacteria>Alphaproteobacteria>Rhodobacterales>Roseobacteraceae	Tropicimonas_sp._IMCC6043	cysteine-desulfurase-[Tropicimonas-sp.-IMCC6043]	2510645
PZW40406.1	cysteine-desulfurase	GCA_003253705.1	383	Bacteria>Proteobacteria>Alphaproteobacteria>Rhodospirillales>Acetobacteraceae	Humitalea_rosea	cysteine-desulfurase-[Humitalea-rosea]	990373
WP_158537302.1	cysteine-desulfurase	GCF_003253705.1	383	Bacteria>Proteobacteria>Alphaproteobacteria>Rhodospirillales>Acetobacteraceae	Humitalea_rosea	cysteine-desulfurase-[Humitalea-rosea]	990373
WP_075632287.1	cysteine-desulfurase	GCF_001938835.1	390	Bacteria>Proteobacteria>Alphaproteobacteria>Rhodospirillales>Acetobacteraceae	Novacetimonas_hansenii	cysteine-desulfurase-DndA-[Komagataeibacter-hansenii]	436
WP_207462156.1	cysteine-desulfurase	-	382	Bacteria>Proteobacteria>Alphaproteobacteria>Rhodospirillales>Azospirillaceae	Azospirillum_sp._SYSU_D00513	aminotransferase-class-V-fold-PLP-dependent-enzyme-[Azospirillum-sp.-SYSU-D00513]	2812561
WP_200483905.1	cysteine-desulfurase	-	378	Bacteria>Proteobacteria>Alphaproteobacteria>Rhodospirillales>Azospirillaceae	Azospirillum_sp._YIM_DDC1	aminotransferase-class-V-fold-PLP-dependent-enzyme-[Azospirillum-sp.-YIM-DDC1]	2802052
WP_173982211.1	cysteine-desulfurase	GCF_902729425.1	378	Bacteria>Proteobacteria>Alphaproteobacteria>Rhodospirillales>Rhodospirillaceae	Magnetospirillum_sp._SS-4	cysteine-desulfurase-DndA-[Magnetospirillum-sp.-SS-4]	2681465
WP_157879201.1	cysteine-desulfurase	-	381	Bacteria>Proteobacteria>Alphaproteobacteria>Rhodospirillales>Rhodospirillaceae	Pararhodospirillum_photometricum	cysteine-desulfurase-[Pararhodospirillum-photometricum]	1084
MAX62269.1	cysteine-desulfurase	GCA_002706125.1	395	Bacteria>Proteobacteria>Alphaproteobacteria>Rhodospirillales>Rhodospirillaceae	Rhodospirillaceae_bacterium	aminotransferase-[Rhodospirillaceae-bacterium]	1898112
MCB9957168.1	cysteine-desulfurase	GCA_020638575.1	395	Bacteria>Proteobacteria>Alphaproteobacteria>Rhodospirillales>Rhodospirillaceae	Rhodospirillaceae_bacterium	cysteine-desulfurase-[Rhodospirillaceae-bacterium]	1898112
WP_022730135.1	cysteine-desulfurase	GCF_000421265.1	387	Bacteria>Proteobacteria>Alphaproteobacteria>Rhodospirillales>Thalassospiraceae	Thalassospira_lucentensis	cysteine-desulfurase-[Thalassospira-lucentensis]	168935
HAI30482.1	cysteine-desulfurase	GCA_003449315.1	391	Bacteria>Proteobacteria>Alphaproteobacteria>Rhodospirillales>Thalassospiraceae	Thalassospira_sp.	aminotransferase-[Thalassospira-sp.]	1912094
WP_063088452.1	cysteine-desulfurase	GCF_001983425.1	391	Bacteria>Proteobacteria>Alphaproteobacteria>Rhodospirillales>Thalassospiraceae	unclassified_Thalassospira	MULTISPECIES:-cysteine-desulfurase-[unclassified-Thalassospira]	2648997
KZX87796.1	cysteine-desulfurase	GCA_001634715.1	389	Bacteria>Proteobacteria>Alphaproteobacteria>Sphingomonadales>Erythrobacteraceae	Erythrobacter_sp._HI0020	cysteine-desulfurase-DndA-[Erythrobacter-sp.-HI0020]	1822223
WP_197462321.1	cysteine-desulfurase	GCF_001634795.1	378	Bacteria>Proteobacteria>Alphaproteobacteria>Sphingomonadales>Erythrobacteraceae	Erythrobacter_sp._HI0028	cysteine-desulfurase-[Erythrobacter-sp.-HI0028]	1822227
WP_160731412.1	cysteine-desulfurase	GCF_009828095.1	383	Bacteria>Proteobacteria>Alphaproteobacteria>Sphingomonadales>Erythrobacteraceae	Pontixanthobacter_luteolus	cysteine-desulfurase-[Pontixanthobacter-luteolus]	295089
PLK22756.1	cysteine-desulfurase	GCA_002855495.1	392	Bacteria>Proteobacteria>Alphaproteobacteria>Sphingomonadales>Erythrobacteraceae	Porphyrobacter_sp._TH134	cysteine-desulfurase-DndA-[Porphyrobacter-sp.-TH134]	2067450
WP_101794622.1	cysteine-desulfurase	GCF_002855495.1	392	Bacteria>Proteobacteria>Alphaproteobacteria>Sphingomonadales>Erythrobacteraceae	Porphyrobacter_sp._TH134	cysteine-desulfurase-[Porphyrobacter-sp.-TH134]	2067450
UOR07882.1	cysteine-desulfurase	GCA_022919915.1	384	Bacteria>Proteobacteria>Alphaproteobacteria>Sphingomonadales>Erythrobacteraceae	Qipengyuania_flava	cysteine-desulfurase-[Qipengyuania-flava]	192812
WP_222828537.1	cysteine-desulfurase	GCF_019857205.1	385	Bacteria>Proteobacteria>Alphaproteobacteria>Sphingomonadales>Erythrobacteraceae	Qipengyuania_pacifica	aminotransferase-class-V-fold-PLP-dependent-enzyme-[Qipengyuania-sp.-NZ-96]	2860199
WP_170935629.1	cysteine-desulfurase	GCF_016925655.1	378	Bacteria>Proteobacteria>Alphaproteobacteria	Sphingomonadales	MULTISPECIES:-cysteine-desulfurase-[Sphingomonadales]	204457
WP_223031691.1	cysteine-desulfurase	GCF_019880555.1	395	Bacteria>Proteobacteria>Alphaproteobacteria>Sphingomonadales>Sphingomonadaceae	Hephaestia_sp._CMS5P-6	cysteine-desulfurase-[Hephaestia-sp.-CMS5P-6]	2873268
WP_054109483.1	cysteine-desulfurase	GCF_001295795.1	375	Bacteria>Proteobacteria>Alphaproteobacteria>Sphingomonadales>Sphingomonadaceae	Novosphingobium_sp._AAP83	aminotransferase-class-V-fold-PLP-dependent-enzyme-[Novosphingobium-sp.-AAP83]	1523425
KPF88413.1	cysteine-desulfurase	GCA_001295795.1	389	Bacteria>Proteobacteria>Alphaproteobacteria>Sphingomonadales>Sphingomonadaceae	Novosphingobium_sp._AAP83	cysteine-desulfurase-[Novosphingobium-sp.-AAP83]	1523425
MBK6803280.1	cysteine-desulfurase	GCA_016702815.1	377	Bacteria>Proteobacteria>Alphaproteobacteria>Sphingomonadales>Sphingomonadaceae	Novosphingobium_sp.	aminotransferase-class-V-fold-PLP-dependent-enzyme-[Novosphingobium-sp.]	1874826
WP_039391628.1	cysteine-desulfurase	GCF_000813185.1	380	Bacteria>Proteobacteria>Alphaproteobacteria>Sphingomonadales>Sphingomonadaceae	Novosphingobium_sp._MBES04	cysteine-desulfurase,-partial-[Novosphingobium-sp.-MBES04]	1206458
WP_168176244.1	cysteine-desulfurase	GCF_002556635.1	378	Bacteria>Proteobacteria>Alphaproteobacteria>Sphingomonadales>Sphingomonadaceae	Novosphingobium_sp._PC22D	cysteine-desulfurase-[Novosphingobium-sp.-PC22D]	1962403
WP_066968418.1	cysteine-desulfurase	GCF_002355275.1	391	Bacteria>Proteobacteria>Alphaproteobacteria>Sphingomonadales>Sphingomonadaceae	Rhizorhabdus_dicambivorans	aminotransferase-class-V-fold-PLP-dependent-enzyme-[Rhizorhabdus-dicambivorans]	1850238
WP_238147119.1	cysteine-desulfurase	GCF_002355275.1	363	Bacteria>Proteobacteria>Alphaproteobacteria>Sphingomonadales>Sphingomonadaceae	Rhizorhabdus_dicambivorans	cysteine-desulfurase-[Rhizorhabdus-dicambivorans]	1850238
WP_164847540.1	cysteine-desulfurase	GCF_004005485.1	376	Bacteria>Proteobacteria>Alphaproteobacteria>Sphingomonadales>Sphingomonadaceae	Sphingobium_algorifonticola	cysteine-desulfurase-[Sphingobium-algorifonticola]	2008318
WP_099185368.1	cysteine-desulfurase	GCF_002723855.1	396	Bacteria>Proteobacteria>Alphaproteobacteria>Sphingomonadales>Sphingomonadaceae	Sphingobium_fuliginis	cysteine-desulfurase-[Sphingobium-fuliginis]	336203
WP_217673802.1	cysteine-desulfurase	-	389	Bacteria>Proteobacteria>Alphaproteobacteria>Sphingomonadales>Sphingomonadaceae	Sphingobium_sp._AS12	aminotransferase-class-V-fold-PLP-dependent-enzyme-[Sphingobium-sp.-AS12]	2849495
PZU58182.1	cysteine-desulfurase	GCA_003248935.1	423	Bacteria>Proteobacteria>Alphaproteobacteria>Sphingomonadales>Sphingomonadaceae	Sphingobium_sp.	cysteine-desulfurase-[Sphingobium-sp.]	1912891
WP_137710387.1	cysteine-desulfurase	GCF_005406185.3	388	Bacteria>Proteobacteria>Alphaproteobacteria>Sphingomonadales>Sphingomonadaceae	Sphingobium_sp._RSMS	cysteine-desulfurase-[Sphingobium-sp.-RSMS]	520734
WP_093014169.1	cysteine-desulfurase	GCF_900111125.1	394	Bacteria>Proteobacteria>Alphaproteobacteria>Sphingomonadales>Sphingomonadaceae	Sphingobium_sp._YR768	cysteine-desulfurase-[Sphingobium-sp.-YR768]	1884365
WP_166748283.1	cysteine-desulfurase	GCF_011759485.1	402	Bacteria>Proteobacteria>Alphaproteobacteria>Sphingomonadales>Sphingomonadaceae	Sphingomonas_aerolata	cysteine-desulfurase-[Sphingomonas-aerolata]	185951
WP_205479532.1	cysteine-desulfurase	GCF_016924655.1	389	Bacteria>Proteobacteria>Alphaproteobacteria>Sphingomonadales>Sphingomonadaceae	Sphingomonas_arenae	MULTISPECIES:-aminotransferase-class-V-fold-PLP-dependent-enzyme-[unclassified-Sphingomonas]	2812555
TXC71324.1	cysteine-desulfurase	GCA_007995065.1	389	Bacteria>Proteobacteria>Alphaproteobacteria>Sphingomonadales>Sphingomonadaceae	Sphingomonas_ginsenosidivorax	aminotransferase-class-V-fold-PLP-dependent-enzyme-[Sphingomonas-ginsenosidivorax]	862135
WP_147082498.1	cysteine-desulfurase	GCF_007995065.1	389	Bacteria>Proteobacteria>Alphaproteobacteria>Sphingomonadales>Sphingomonadaceae	Sphingomonas_ginsenosidivorax	aminotransferase-class-V-fold-PLP-dependent-enzyme-[Sphingomonas-ginsenosidivorax]	862135
WP_157926334.1	cysteine-desulfurase	GCF_002797435.1	370	Bacteria>Proteobacteria>Alphaproteobacteria>Sphingomonadales>Sphingomonadaceae	Sphingomonas_koreensis	cysteine-desulfurase-[Sphingomonas-koreensis]	93064
WP_184160874.1	cysteine-desulfurase	GCF_014204495.1	388	Bacteria>Proteobacteria>Alphaproteobacteria>Sphingomonadales>Sphingomonadaceae	Sphingomonas_kyeonggiensis	cysteine-desulfurase-[Sphingomonas-kyeonggiensis]	1268553
WP_135984579.1	cysteine-desulfurase	GCF_011762145.1	389	Bacteria>Proteobacteria>Alphaproteobacteria>Sphingomonadales>Sphingomonadaceae	Sphingomonas_naasensis	cysteine-desulfurase-[Sphingomonas-naasensis]	1344951
WP_218139393.1	cysteine-desulfurase	GCF_900109565.1	387	Bacteria>Proteobacteria>Alphaproteobacteria>Sphingomonadales>Sphingomonadaceae	Sphingomonas_palmae	aminotransferase-class-V-fold-PLP-dependent-enzyme-[Sphingomonas-palmae]	1855283
SEL80117.1	cysteine-desulfurase	GCA_900109565.1	387	Bacteria>Proteobacteria>Alphaproteobacteria>Sphingomonadales>Sphingomonadaceae	Sphingomonas_palmae	cysteine-desulfurase-[Sphingomonas-palmae]	1855283
WP_066725645.1	cysteine-desulfurase	GCF_001598435.1	387	Bacteria>Proteobacteria>Alphaproteobacteria>Sphingomonadales>Sphingomonadaceae	Sphingomonas_pituitosa	cysteine-desulfurase-[Sphingomonas-pituitosa]	99597
WP_156681076.1	cysteine-desulfurase	GCF_009739515.1	390	Bacteria>Proteobacteria>Alphaproteobacteria>Sphingomonadales>Sphingomonadaceae	Sphingomonas_profundi	aminotransferase-class-V-fold-PLP-dependent-enzyme-[Sphingomonas-sp.-LMO-1]	2681549
OYY66575.1	cysteine-desulfurase	GCA_002278865.1	394	Bacteria>Proteobacteria>Alphaproteobacteria>Sphingomonadales>Sphingomonadaceae	Sphingomonas_sp._28-62-11	hypothetical-protein-B7Y49_02000-[Sphingomonas-sp.-28-62-11]	1970432
WP_137785128.1	cysteine-desulfurase	GCF_005502745.1	423	Bacteria>Proteobacteria>Alphaproteobacteria>Sphingomonadales>Sphingomonadaceae	Sphingomonas_sp._3P27F8	cysteine-desulfurase-[Sphingomonas-sp.-3P27F8]	2502213
THD34464.1	cysteine-desulfurase	GCA_004799495.1	388	Bacteria>Proteobacteria>Alphaproteobacteria>Sphingomonadales>Sphingomonadaceae	Sphingomonas_sp.	aminotransferase-class-V-fold-PLP-dependent-enzyme-[Sphingomonas-sp.]	28214
MBY0282501.1	cysteine-desulfurase	GCA_019749955.1	388	Bacteria>Proteobacteria>Alphaproteobacteria>Sphingomonadales>Sphingomonadaceae	Sphingomonas_sp.	cysteine-desulfurase-[Sphingomonas-sp.]	28214
MBY0303645.1	cysteine-desulfurase	GCA_019750015.1	383	Bacteria>Proteobacteria>Alphaproteobacteria>Sphingomonadales>Sphingomonadaceae	Sphingomonas_sp.	cysteine-desulfurase-[Sphingomonas-sp.]	28214
NUR36592.1	cysteine-desulfurase	GCA_013362105.1	389	Bacteria>Proteobacteria>Alphaproteobacteria>Sphingomonadales>Sphingomonadaceae	Sphingomonas_sp.	cysteine-desulfurase-[Sphingomonas-sp.]	28214
WP_231420799.1	cysteine-desulfurase	GCF_920984725.1	375	Bacteria>Proteobacteria>Alphaproteobacteria>Sphingomonadales>Sphingomonadaceae	Sphingomonas_sp._Leaf205	aminotransferase-class-V-fold-PLP-dependent-enzyme-[Sphingomonas-sp.-Leaf205]	2876551
WP_056497442.1	cysteine-desulfurase	GCF_001421635.1	380	Bacteria>Proteobacteria>Alphaproteobacteria>Sphingomonadales>Sphingomonadaceae	Sphingomonas_sp._Leaf25	aminotransferase-class-V-fold-PLP-dependent-enzyme-[Sphingomonas-sp.-Leaf25]	1735692
WP_219756603.1	cysteine-desulfurase	GCF_019429525.1	389	Bacteria>Proteobacteria>Alphaproteobacteria>Sphingomonadales>Sphingomonadaceae	Sphingomonas_sp._RHCKR7	aminotransferase-class-V-fold-PLP-dependent-enzyme-[Sphingomonas-sp.-RHCKR7]	2862497
WP_235077165.1	cysteine-desulfurase	GCF_021513175.1	390	Bacteria>Proteobacteria>Alphaproteobacteria>Sphingomonadales>Sphingomonadaceae	Sphingomonas_sp._S2-65	aminotransferase-class-V-fold-PLP-dependent-enzyme-[Sphingomonas-sp.-S2-65]	2903960
WP_009823063.1	cysteine-desulfurase	GCF_000153545.1	383	Bacteria>Proteobacteria>Alphaproteobacteria>Sphingomonadales>Sphingomonadaceae	Sphingomonas_sp._SKA58	cysteine-desulfurase-[Sphingomonas-sp.-SKA58]	314266
WP_077029274.1	cysteine-desulfurase	GCF_001984035.1	401	Bacteria>Proteobacteria>Alphaproteobacteria>Sphingomonadales>Sphingomonadaceae	Sphingopyxis_sp._QXT-31	cysteine-desulfurase-[Sphingopyxis-sp.-QXT-31]	1357916
WP_137864584.1	cysteine-desulfurase	GCF_005503355.1	386	Bacteria>Proteobacteria>Alphaproteobacteria>Sphingomonadales>Sphingomonadaceae	unclassified_Sphingomonas	MULTISPECIES:-cysteine-desulfurase-[unclassified-Sphingomonas]	196159
RYY26128.1	cysteine-desulfurase	GCA_004173185.1	388	Bacteria>Proteobacteria>Alphaproteobacteria>Sphingomonadales>unclassified_Sphingomonadales	Sphingomonadales_bacterium	aminotransferase-class-V-fold-PLP-dependent-enzyme-[Sphingomonadales-bacterium]	1978525
OUR79621.1	cysteine-desulfurase	GCA_002163135.1	395	Bacteria>Proteobacteria>Alphaproteobacteria>unclassified_Alphaproteobacteria	Alphaproteobacteria_bacterium_46_93_T64	cysteine-sulfinate-desulfinase-[Alphaproteobacteria-bacterium-46_93_T64]	1856292
MBO4521402.1	cysteine-desulfurase	GCA_017616015.1	383	Bacteria>Proteobacteria>Alphaproteobacteria>unclassified_Alphaproteobacteria	Alphaproteobacteria_bacterium	aminotransferase-class-V-fold-PLP-dependent-enzyme-[Alphaproteobacteria-bacterium]	1913988
MBQ7674107.1	cysteine-desulfurase	GCA_017450085.1	373	Bacteria>Proteobacteria>Alphaproteobacteria>unclassified_Alphaproteobacteria	Alphaproteobacteria_bacterium	aminotransferase-class-V-fold-PLP-dependent-enzyme-[Alphaproteobacteria-bacterium]	1913988
MBE6448090.1	cysteine-desulfurase	GCA_015062305.1	392	Bacteria>Proteobacteria>Alphaproteobacteria>unclassified_Alphaproteobacteria	Alphaproteobacteria_bacterium	cysteine-desulfurase-[Alphaproteobacteria-bacterium]	1913988
MBQ8464581.1	cysteine-desulfurase	GCA_017467515.1	382	Bacteria>Proteobacteria>Alphaproteobacteria>unclassified_Alphaproteobacteria	Alphaproteobacteria_bacterium	cysteine-desulfurase-[Alphaproteobacteria-bacterium]	1913988
MBQ8482198.1	cysteine-desulfurase	GCA_017471705.1	393	Bacteria>Proteobacteria>Alphaproteobacteria>unclassified_Alphaproteobacteria	Alphaproteobacteria_bacterium	cysteine-desulfurase-[Alphaproteobacteria-bacterium]	1913988
MBF0355957.1	cysteine-desulfurase	GCA_015232335.1	382	Bacteria>Proteobacteria>Alphaproteobacteria>unclassified_Alphaproteobacteria	Alphaproteobacteria_bacterium	cysteine-desulfurase-DndA-[Alphaproteobacteria-bacterium]	1913988
PCI52862.1	cysteine-desulfurase	GCA_002401695.1	387	Bacteria>Proteobacteria>Alphaproteobacteria>unclassified_Alphaproteobacteria	Alphaproteobacteria_bacterium	cysteine-desulfurase-DndA-[Alphaproteobacteria-bacterium]	1913988
PZO40727.1	cysteine-desulfurase	GCA_003241995.1	380	Bacteria>Proteobacteria>Alphaproteobacteria>unclassified_Alphaproteobacteria	Alphaproteobacteria_bacterium	hypothetical-protein-DCE92_01910-[Alphaproteobacteria-bacterium]	1913988
WP_034407618.1	cysteine-desulfurase	GCF_000761415.1	369	Bacteria>Proteobacteria>Betaproteobacteria>Burkholderiales>Comamonadaceae	Comamonas_thiooxydans	3-phosphoserine/phosphohydroxythreonine-transaminase-[Comamonas-thiooxydans]	363952
KGG85565.1	cysteine-desulfurase	GCA_000761415.1	369	Bacteria>Proteobacteria>Betaproteobacteria>Burkholderiales>Comamonadaceae	Comamonas_thiooxydans	MFS-transporter-[Comamonas-thiooxydans]	363952
MBW8298278.1	cysteine-desulfurase	GCA_019459045.1	387	Bacteria>Proteobacteria>Betaproteobacteria>Burkholderiales>Comamonadaceae	Hydrogenophaga_sp.	aminotransferase-class-V-fold-PLP-dependent-enzyme-[Hydrogenophaga-sp.]	1904254
OON62700.1	cysteine-desulfurase	GCA_002007115.1	375	Bacteria>Proteobacteria>Betaproteobacteria>Burkholderiales>Oxalobacteraceae	Massilia_sp._KIM	cysteine-desulfurase-DndA-[Massilia-sp.-KIM]	1955422
WP_078031352.1	cysteine-desulfurase	GCF_002007115.1	375	Bacteria>Proteobacteria>Betaproteobacteria>Burkholderiales>Oxalobacteraceae	Massilia_sp._KIM	cysteine-desulfurase-DndA-[Massilia-sp.-KIM]	1955422
MVW64162.1	cysteine-desulfurase	GCA_009763355.1	375	Bacteria>Proteobacteria>Betaproteobacteria>Burkholderiales>Oxalobacteraceae	Massilia_sp._NEAU-DD11	cysteine-desulfurase-DndA-[Massilia-sp.-NEAU-DD11]	2683234
WP_160410682.1	cysteine-desulfurase	GCF_009763355.1	375	Bacteria>Proteobacteria>Betaproteobacteria>Burkholderiales>Oxalobacteraceae	Massilia_sp._NEAU-DD11	cysteine-desulfurase-DndA-[Massilia-sp.-NEAU-DD11]	2683234
MBC7367895.1	cysteine-desulfurase	GCA_014376585.1	376	Bacteria>Proteobacteria>Betaproteobacteria>Burkholderiales>Oxalobacteraceae	Undibacterium_sp.	aminotransferase-class-V-fold-PLP-dependent-enzyme-[Undibacterium-sp.]	1914977
WP_007343494.1	cysteine-desulfurase	GCF_014055025.1	366	Bacteria>Proteobacteria>Betaproteobacteria>Neisseriales>Neisseriaceae	Neisseria_bacilliformis	3-phosphoserine/phosphohydroxythreonine-transaminase-[Neisseria-bacilliformis]	267212
MBX9636984.1	cysteine-desulfurase	GCA_019748355.1	374	Bacteria>Proteobacteria>Betaproteobacteria>Nitrosomonadales>Nitrosomonadaceae	Nitrosomonas_sp.	cysteine-desulfurase-DndA-[Nitrosomonas-sp.]	42353
MXS76825.1	cysteine-desulfurase	GCA_009833095.1	374	Bacteria>Proteobacteria>Betaproteobacteria>Nitrosomonadales>Nitrosomonadaceae	Nitrosomonas_sp._JL21	cysteine-desulfurase-DndA-[Nitrosomonas-sp.-JL21]	153949
WP_160827534.1	cysteine-desulfurase	GCF_009833095.1	374	Bacteria>Proteobacteria>Betaproteobacteria>Nitrosomonadales>Nitrosomonadaceae	Nitrosomonas_sp._JL21	cysteine-desulfurase-DndA-[Nitrosomonas-sp.-JL21]	153949
WP_004337192.1	cysteine-desulfurase	-	377	Bacteria>Proteobacteria>Betaproteobacteria>Rhodocyclales>Zoogloeaceae	Thauera_linaloolentis	cysteine-desulfurase-DndA-[Thauera-linaloolentis]	76112
TAK44076.1	cysteine-desulfurase	GCA_004298325.1	395	Bacteria>Proteobacteria>Betaproteobacteria>unclassified_Betaproteobacteria	Betaproteobacteria_bacterium	cysteine-desulfurase-[Betaproteobacteria-bacterium]	1891241
WP_141197004.1	cysteine-desulfurase	GCF_006517175.1	386	Bacteria>Proteobacteria>delta/epsilon_subdivisions>Deltaproteobacteria>Bradymonadales	Persicimonas_caeni	cysteine-desulfurase-[Persicimonas-caeni]	2292766
MBL6995473.1	cysteine-desulfurase	GCA_016785005.1	377	Bacteria>Proteobacteria>delta/epsilon_subdivisions>Deltaproteobacteria>Desulfobacterales	Desulfobacula_sp.	cysteine-desulfurase-DndA-[Desulfobacula-sp.]	2593537
WP_208599088.1	cysteine-desulfurase	GCF_000934745.1	378	Bacteria>Proteobacteria>delta/epsilon_subdivisions>Deltaproteobacteria>Desulfovibrionales	Desulfonatronum_thioautotrophicum	cysteine-desulfurase-DndA-[Desulfonatronum-thioautotrophicum]	617001
WP_026843201.1	cysteine-desulfurase	GCF_000426865.1	381	Bacteria>Proteobacteria>delta/epsilon_subdivisions>Deltaproteobacteria>Desulfuromonadales	Citrifermentans_bremense	cysteine-desulfurase-[Citrifermentans-bremense]	60035
WP_225071827.1	cysteine-desulfurase	GCF_020194955.2	377	Bacteria>Proteobacteria>delta/epsilon_subdivisions>Deltaproteobacteria>Desulfuromonadales	Desulfuromonas_sp._CSMB_57	cysteine-desulfurase-DndA-[Desulfuromonas-sp.-CSMB_57]	2807629
WP_145019835.1	cysteine-desulfurase	GCF_007830735.1	377	Bacteria>Proteobacteria>delta/epsilon_subdivisions>Deltaproteobacteria>Desulfuromonadales	Geobacter_argillaceus	cysteine-desulfurase-DndA-[Geobacter-argillaceus]	345631
WP_173195999.1	cysteine-desulfurase	GCF_016865365.1	382	Bacteria>Proteobacteria>delta/epsilon_subdivisions>Deltaproteobacteria>Desulfuromonadales	Geobacter_sp._SVR	cysteine-desulfurase-[Geobacter-sp.-SVR]	2495594
BCS52643.1	cysteine-desulfurase	GCA_016865365.1	382	Bacteria>Proteobacteria>delta/epsilon_subdivisions>Deltaproteobacteria>Desulfuromonadales	Geobacter_sp._SVR	cysteine-desulfurase-IscS-[Geobacter-sp.-SVR]	2495594
WP_216509722.1	cysteine-desulfurase	GCF_018919395.1	377	Bacteria>Proteobacteria>delta/epsilon_subdivisions>Deltaproteobacteria>Desulfuromonadales	Geomonas_azotofigens	cysteine-desulfurase-DndA-[Geomonas-azotofigens]	2843196
QWV98225.1	cysteine-desulfurase	GCA_018986935.1	376	Bacteria>Proteobacteria>delta/epsilon_subdivisions>Deltaproteobacteria>Desulfuromonadales	Geomonas_nitrogeniifigens	cysteine-desulfurase-DndA-[Geomonas-sp.-RG29]	2847991
WP_216790471.1	cysteine-desulfurase	GCF_018986935.1	376	Bacteria>Proteobacteria>delta/epsilon_subdivisions>Deltaproteobacteria>Desulfuromonadales	Geomonas_nitrogeniifigens	cysteine-desulfurase-DndA-[Geomonas-sp.-RG29]	2847991

Accession	Annotation	GCA	Length	Phyletic.spread	Strain.name	Defline	Species.Taxid
WP_011939338.1	cysteine-desulfurase	GCF_000016745.1	377	Bacteria>Proteobacteria>delta/epsilon_subdivisions>Deltaproteobacteria>Desulfuromonadales	Geotalea_uraniireducens	cysteine-desulfurase-DndA-[Geobacter-uraniireducens]	351604
ABQ26650.1	cysteine-desulfurase	GCA_000016745.1	377	Bacteria>Proteobacteria>delta/epsilon_subdivisions>Deltaproteobacteria>Desulfuromonadales	Geotalea_uraniireducens_Rf4	aminotransferase,-class-V-[Geotalea-uraniireducens-Rf4]	351605
WP_233189462.1	cysteine-desulfurase	GCF_002898515.1	417	Bacteria>Proteobacteria>delta/epsilon_subdivisions>Deltaproteobacteria>Desulfuromonadales	Geothermobacter_hydrogeniphilus	cysteine-desulfurase-DndA-[Geothermobacter-hydrogeniphilus]	1969733
HCX01859.1	cysteine-desulfurase	GCA_003538085.1	377	Bacteria>Proteobacteria>delta/epsilon_subdivisions>Deltaproteobacteria>Syntrophales	Syntrophaceae_bacterium	cysteine-desulfurase-DndA-[Syntrophaceae-bacterium]	2052184
MBI2388617.1	cysteine-desulfurase	GCA_016182965.1	373	Bacteria>Proteobacteria>delta/epsilon_subdivisions>Deltaproteobacteria>unclassified_Deltaproteobacteria	Deltaproteobacteria_bacterium	aminotransferase-class-V-fold-PLP-dependent-enzyme-[Deltaproteobacteria-bacterium]	2026735
NOT57126.1	cysteine-desulfurase	GCA_013140885.1	321	Bacteria>Proteobacteria>delta/epsilon_subdivisions>Deltaproteobacteria>unclassified_Deltaproteobacteria	Deltaproteobacteria_bacterium	aminotransferase-class-V-fold-PLP-dependent-enzyme-[Deltaproteobacteria-bacterium]	2026735
NQW62586.1	cysteine-desulfurase	GCA_013205435.1	170	Bacteria>Proteobacteria>delta/epsilon_subdivisions>Deltaproteobacteria>unclassified_Deltaproteobacteria	Deltaproteobacteria_bacterium	aminotransferase-class-V-fold-PLP-dependent-enzyme-[Deltaproteobacteria-bacterium]	2026735
MBT4184501.1	cysteine-desulfurase	GCA_018651485.1	261	Bacteria>Proteobacteria>delta/epsilon_subdivisions>Deltaproteobacteria>unclassified_Deltaproteobacteria	Deltaproteobacteria_bacterium	cysteine-desulfurase-[Deltaproteobacteria-bacterium]	2026735
MRR53941.1	cysteine-desulfurase	GCA_009668975.1	382	Bacteria>Proteobacteria>delta/epsilon_subdivisions>Deltaproteobacteria>unclassified_Deltaproteobacteria	Deltaproteobacteria_bacterium	cysteine-desulfurase-[Deltaproteobacteria-bacterium]	2026735
MBZ0235624.1	cysteine-desulfurase	GCA_019912685.1	392	Bacteria>Proteobacteria>delta/epsilon_subdivisions>Deltaproteobacteria>unclassified_Deltaproteobacteria	Deltaproteobacteria_bacterium	cysteine-desulfurase-DndA-[Deltaproteobacteria-bacterium]	2026735
OGP20355.1	cysteine-desulfurase	GCA_001797445.1	360	Bacteria>Proteobacteria>delta/epsilon_subdivisions>Deltaproteobacteria>unclassified_Deltaproteobacteria	Deltaproteobacteria_bacterium_GWA2_65_63	hypothetical-protein-A2X90_05260-[Deltaproteobacteria-bacterium-GWA2_65_63]	1797812
RLA92682.1	cysteine-desulfurase	GCA_003646645.1	387	Bacteria>Proteobacteria>delta/epsilon_subdivisions>Deltaproteobacteria>unclassified_Deltaproteobacteria	Deltaproteobacteria_bacterium	IscS-subfamily-cysteine-desulfurase-[Deltaproteobacteria-bacterium]	2026735
WP_234604922.1	cysteine-desulfurase	GCF_021441165.1	374	Bacteria>Proteobacteria>Gammaproteobacteria>Aeromonadales>Aeromonadaceae	Aeromonas_allosaccharophila	cysteine-desulfurase-DndA-[Aeromonas-allosaccharophila]	656
WP_202042573.1	cysteine-desulfurase	GCF_016729565.1	374	Bacteria>Proteobacteria>Gammaproteobacteria>Aeromonadales>Aeromonadaceae	Aeromonas_caviae	cysteine-desulfurase-DndA-[Aeromonas-caviae]	648
WP_081805685.1	cysteine-desulfurase	GCF_000287215.2	349	Bacteria>Proteobacteria>Gammaproteobacteria>Aeromonadales>Aeromonadaceae	Aeromonas_media	cysteine-desulfurase-[Aeromonas-media]	651
WP_159158728.1	cysteine-desulfurase	GCF_902505945.1	374	Bacteria>Proteobacteria>Gammaproteobacteria>Aeromonadales>Aeromonadaceae	Aeromonas_sp._8C	cysteine-desulfurase-DndA-[Aeromonas-sp.-8C]	2653127
WP_168965899.1	cysteine-desulfurase	-	374	Bacteria>Proteobacteria>Gammaproteobacteria>Aeromonadales>Aeromonadaceae	Aeromonas_sp._DNRA1	cysteine-desulfurase-DndA-[Aeromonas-sp.-DNRA1]	2729335
WP_216953416.1	cysteine-desulfurase	GCF_019047065.1	374	Bacteria>Proteobacteria>Gammaproteobacteria>Aeromonadales>Aeromonadaceae	Aeromonas_sp._FDAARGOS_1414	cysteine-desulfurase-DndA-[Aeromonas-sp.-FDAARGOS-1414]	2778063
WP_139473354.1	cysteine-desulfurase	GCF_006246555.1	374	Bacteria>Proteobacteria>Gammaproteobacteria>Aeromonadales>Aeromonadaceae	Aeromonas_veronii	cysteine-desulfurase-DndA-[Aeromonas-veronii]	654
WP_236317297.1	cysteine-desulfurase	GCF_021608475.1	374	Bacteria>Proteobacteria>Gammaproteobacteria>Aeromonadales>Aeromonadaceae	Aeromonas_veronii	cysteine-desulfurase-DndA-[Aeromonas-veronii]	654
VXA83220.1	cysteine-desulfurase	GCA_902505945.1	395	Bacteria>Proteobacteria>Gammaproteobacteria>Aeromonadales>Aeromonadaceae	Aeromonas_veronii	Cysteine-desulfurase-IscS-[Aeromonas-veronii]	654
WP_107851500.1	cysteine-desulfurase	GCF_003049725.1	350	Bacteria>Proteobacteria>Gammaproteobacteria>Aeromonadales>Aeromonadaceae	Oceanimonas_marisflavi	cysteine-desulfurase-[Oceanimonas-marisflavi]	2059724
WP_111977307.1	cysteine-desulfurase	GCF_003286125.1	375	Bacteria>Proteobacteria>Gammaproteobacteria>Alteromonadales>Alteromonadaceae	Algibacillus_agarilyticus	cysteine-desulfurase-DndA-[Algibacillus-agarilyticus]	2234133
NDW15801.1	cysteine-desulfurase	GCA_010500895.1	373	Bacteria>Proteobacteria>Gammaproteobacteria>Alteromonadales>Alteromonadaceae	Alteromonas_genovensis	aminotransferase-class-V-fold-PLP-dependent-enzyme-[Alteromonas-genovensis]	471225
WP_163106520.1	cysteine-desulfurase	GCF_010500895.1	373	Bacteria>Proteobacteria>Gammaproteobacteria>Alteromonadales>Alteromonadaceae	Alteromonas_genovensis	cysteine-desulfurase-[Alteromonas-genovensis]	471225
WP_012516642.1	cysteine-desulfurase	GCF_000020585.3	373	Bacteria>Proteobacteria>Gammaproteobacteria>Alteromonadales>Alteromonadaceae	Alteromonas_mediterranea	cysteine-desulfurase-[Alteromonas-mediterranea]	314275
WP_123324813.1	cysteine-desulfurase	GCF_003731635.1	365	Bacteria>Proteobacteria>Gammaproteobacteria>Alteromonadales>Alteromonadaceae	Alteromonas_oceani	cysteine-desulfurase-[Alteromonas-oceani]	2071609
WP_118490871.1	cysteine-desulfurase	GCF_003443615.1	374	Bacteria>Proteobacteria>Gammaproteobacteria>Alteromonadales>Alteromonadaceae	Alteromonas_sp._BL110	cysteine-desulfurase-DndA-[Alteromonas-sp.-BL110]	1714845
WP_061996746.1	cysteine-desulfurase	GCF_001562125.1	374	Bacteria>Proteobacteria>Gammaproteobacteria>Alteromonadales>Alteromonadaceae	Alteromonas_sp._Mac1	cysteine-desulfurase-DndA-[Alteromonas-sp.-Mac1]	1777491
MBZ2160774.1	cysteine-desulfurase	GCA_019931075.1	374	Bacteria>Proteobacteria>Gammaproteobacteria>Alteromonadales>Alteromonadaceae	Alteromonas_stellipolaris	cysteine-desulfurase-DndA-[Alteromonas-stellipolaris]	233316
WP_063457991.1	cysteine-desulfurase	GCF_001632825.1	374	Bacteria>Proteobacteria>Gammaproteobacteria>Alteromonadales>Alteromonadaceae	Alteromonas_stellipolaris	cysteine-desulfurase-DndA-[Alteromonas-stellipolaris]	233316
WP_231517568.1	cysteine-desulfurase	GCF_019931075.1	374	Bacteria>Proteobacteria>Gammaproteobacteria>Alteromonadales>Alteromonadaceae	Alteromonas_stellipolaris	cysteine-desulfurase-DndA-[Alteromonas-stellipolaris]	233316
WP_233052426.1	cysteine-desulfurase	GCF_021295315.1	374	Bacteria>Proteobacteria>Gammaproteobacteria>Alteromonadales>Alteromonadales_genera_incertae_sedis	Motilimonas_cestriensis	cysteine-desulfurase-DndA-[Motilimonas-cestriensis]	2742685
WP_126668722.1	cysteine-desulfurase	GCF_003971255.1	375	Bacteria>Proteobacteria>Gammaproteobacteria>Alteromonadales>Colwelliaceae	Colwellia_sp._Arc7-635	cysteine-desulfurase-DndA-[Colwellia-sp.-Arc7-635]	2497879
WP_108455561.1	cysteine-desulfurase	GCF_003061515.1	375	Bacteria>Proteobacteria>Gammaproteobacteria>Alteromonadales>Colwelliaceae	Colwellia_sp._Arc7-D	cysteine-desulfurase-DndA-[Colwellia-sp.-Arc7-D]	2161872
TKB46340.1	cysteine-desulfurase	GCA_005116735.1	375	Bacteria>Proteobacteria>Gammaproteobacteria>Alteromonadales>Colwelliaceae	Thalassotalea_mangrovi	cysteine-desulfurase-[Thalassotalea-mangrovi]	2572245
WP_136734916.1	cysteine-desulfurase	GCF_005116735.1	375	Bacteria>Proteobacteria>Gammaproteobacteria>Alteromonadales>Colwelliaceae	Thalassotalea_mangrovi	cysteine-desulfurase-[Thalassotalea-mangrovi]	2572245
OUS29084.1	cysteine-desulfurase	GCA_002162925.1	399	Bacteria>Proteobacteria>Gammaproteobacteria>Alteromonadales>Colwelliaceae	Thalassotalea_sp._42_200_T64	aminotransferase-[Thalassotalea-sp.-42_200_T64]	1856287
WP_006954022.1	cysteine-desulfurase	GCF_000152885.1	374	Bacteria>Proteobacteria>Gammaproteobacteria>Alteromonadales>Idiomarinaceae	Idiomarina_baltica	cysteine-desulfurase-[Idiomarina-baltica]	190892
NWO02093.1	cysteine-desulfurase	GCA_013393475.1	374	Bacteria>Proteobacteria>Gammaproteobacteria>Alteromonadales>Idiomarinaceae	Idiomarinaceae_bacterium	cysteine-desulfurase-DndA-[Idiomarinaceae-bacterium]	1898113
WP_034819306.1	cysteine-desulfurase	GCF_000320945.2	374	Bacteria>Proteobacteria>Gammaproteobacteria>Alteromonadales>Idiomarinaceae	Idiomarina_sp._28-8	cysteine-desulfurase-DndA-[Idiomarina-sp.-28-8]	1260624
EGN74182.1	cysteine-desulfurase	GCA_000218785.2	374	Bacteria>Proteobacteria>Gammaproteobacteria>Alteromonadales>Idiomarinaceae	Idiomarina_sp._A28L	cysteine-desulfurase-DndA-[Idiomarina-sp.-A28L]	1036674
WP_007421263.1	cysteine-desulfurase	GCF_000218785.1	374	Bacteria>Proteobacteria>Gammaproteobacteria>Alteromonadales>Idiomarinaceae	Idiomarina_sp._A28L	cysteine-desulfurase-DndA-[Idiomarina-sp.-A28L]	1036674
RUO81077.1	cysteine-desulfurase	GCA_003987475.1	374	Bacteria>Proteobacteria>Gammaproteobacteria>Alteromonadales>Idiomarinaceae	Idiomarina_tyrosinivorans	cysteine-desulfurase-[Idiomarina-tyrosinivorans]	1445662
WP_126841071.1	cysteine-desulfurase	GCF_003987475.1	374	Bacteria>Proteobacteria>Gammaproteobacteria>Alteromonadales>Idiomarinaceae	Idiomarina_tyrosinivorans	cysteine-desulfurase-[Idiomarina-tyrosinivorans]	1445662
WP_211676560.1	cysteine-desulfurase	GCF_018219415.1	374	Bacteria>Proteobacteria>Gammaproteobacteria>Alteromonadales>Moritellaceae	Moritella_sp._36	cysteine-desulfurase-DndA-[Moritella-sp.-36]	2746233
NQZ06737.1	cysteine-desulfurase	GCA_013216025.1	374	Bacteria>Proteobacteria>Gammaproteobacteria>Alteromonadales>Pseudoalteromonadaceae	Algicola_sp.	cysteine-desulfurase-DndA-[Algicola-sp.]	1872429
WP_138676542.1	cysteine-desulfurase	GCF_005887285.1	375	Bacteria>Proteobacteria>Gammaproteobacteria>Alteromonadales>Pseudoalteromonadaceae	Pseudoalteromonas_aurantia	cysteine-desulfurase-DndA-[Pseudoalteromonas-aurantia]	43654
WP_058155680.1	cysteine-desulfurase	GCF_002310795.1	375	Bacteria>Proteobacteria>Gammaproteobacteria>Alteromonadales>Pseudoalteromonadaceae	Pseudoalteromonas_issachenkonii	cysteine-desulfurase-DndA-[Pseudoalteromonas-issachenkonii]	152297
WP_065979252.1	cysteine-desulfurase	GCF_001704875.1	375	Bacteria>Proteobacteria>Gammaproteobacteria>Alteromonadales>Pseudoalteromonadaceae	Pseudoalteromonas_lipolytica	cysteine-desulfurase-DndA-[Pseudoalteromonas-lipolytica]	570156
WP_039610246.1	cysteine-desulfurase	GCF_000814765.1	375	Bacteria>Proteobacteria>Gammaproteobacteria>Alteromonadales>Pseudoalteromonadaceae	Pseudoalteromonas_luteoviolacea	cysteine-desulfurase-DndA-[Pseudoalteromonas-luteoviolacea]	43657
WP_010375354.1	cysteine-desulfurase	GCF_000382005.1	375	Bacteria>Proteobacteria>Gammaproteobacteria>Alteromonadales>Pseudoalteromonadaceae	Pseudoalteromonas_piscicida	cysteine-desulfurase-DndA-[Pseudoalteromonas-piscicida]	43662
WP_223229920.1	cysteine-desulfurase	GCF_003429305.1	385	Bacteria>Proteobacteria>Gammaproteobacteria>Alteromonadales>Pseudoalteromonadaceae	Pseudoalteromonas_piscicida	cysteine-desulfurase-DndA-[Pseudoalteromonas-piscicida]	43662
WP_138548066.1	cysteine-desulfurase	GCF_005887125.1	375	Bacteria>Proteobacteria>Gammaproteobacteria>Alteromonadales>Pseudoalteromonadaceae	Pseudoalteromonas_ruthenica	cysteine-desulfurase-DndA-[Pseudoalteromonas-ruthenica]	151081
WP_175078179.1	cysteine-desulfurase	GCF_013349995.1	373	Bacteria>Proteobacteria>Gammaproteobacteria>Alteromonadales>Pseudoalteromonadaceae	Pseudoalteromonas_sp._0303	cysteine-desulfurase-[Pseudoalteromonas-sp.-0303]	2743618
WP_199528832.1	cysteine-desulfurase	GCF_016464105.1	373	Bacteria>Proteobacteria>Gammaproteobacteria>Alteromonadales>Pseudoalteromonadaceae	Pseudoalteromonas_sp._bablab_jr010	cysteine-desulfurase-[Pseudoalteromonas-sp.-bablab_jr010]	2755063
WP_008465181.1	cysteine-desulfurase	GCF_000241165.1	373	Bacteria>Proteobacteria>Gammaproteobacteria>Alteromonadales>Pseudoalteromonadaceae	Pseudoalteromonas_sp._BSi20439	cysteine-desulfurase-[Pseudoalteromonas-sp.-BSi20439]	420915
PHQ89704.1	cysteine-desulfurase	GCA_002732915.1	375	Bacteria>Proteobacteria>Gammaproteobacteria>Alteromonadales>Pseudoalteromonadaceae	Pseudoalteromonas_sp.	cysteine-desulfurase-DndA-[Pseudoalteromonas-sp.]	53249
WP_237128001.1	cysteine-desulfurase	GCF_021728035.1	372	Bacteria>Proteobacteria>Gammaproteobacteria>Alteromonadales>Pseudoalteromonadaceae	Pseudoalteromonas_sp._L21	cysteine-desulfurase-[Pseudoalteromonas-sp.-L21]	1539746
WP_211055492.1	cysteine-desulfurase	GCF_018100905.1	375	Bacteria>Proteobacteria>Gammaproteobacteria>Alteromonadales>Pseudoalteromonadaceae	Pseudoalteromonas_sp._MMG010	cysteine-desulfurase-DndA-[Pseudoalteromonas-sp.-MMG010]	2822685
WP_016899089.1	cysteine-desulfurase	GCF_000263075.1	375	Bacteria>Proteobacteria>Gammaproteobacteria>Alteromonadales>Pseudoalteromonadaceae	Pseudoalteromonas_sp._PAMC_22718	cysteine-desulfurase-DndA-[Pseudoalteromonas-sp.-PAMC-22718]	1175295
WP_138540155.1	cysteine-desulfurase	GCF_005886915.1	373	Bacteria>Proteobacteria>Gammaproteobacteria>Alteromonadales>Pseudoalteromonadaceae	Pseudoalteromonas_sp._S3776	cysteine-desulfurase-[Pseudoalteromonas-sp.-S3776]	579544
WP_182780685.1	cysteine-desulfurase	GCF_014164415.1	375	Bacteria>Proteobacteria>Gammaproteobacteria>Alteromonadales>Pseudoalteromonadaceae	Pseudoalteromonas_sp._SG43-1	cysteine-desulfurase-DndA-[Pseudoalteromonas-sp.-SG43-1]	2760971
WP_182733115.1	cysteine-desulfurase	GCF_014163935.1	375	Bacteria>Proteobacteria>Gammaproteobacteria>Alteromonadales>Pseudoalteromonadaceae	Pseudoalteromonas_sp._SR41-7	cysteine-desulfurase-DndA-[Pseudoalteromonas-sp.-SR41-7]	2760947
WP_182672699.1	cysteine-desulfurase	GCF_014163785.1	375	Bacteria>Proteobacteria>Gammaproteobacteria>Alteromonadales>Pseudoalteromonadaceae	Pseudoalteromonas_sp._SR43-7	cysteine-desulfurase-DndA-[Pseudoalteromonas-sp.-SR43-7]	2760939
WP_182711923.1	cysteine-desulfurase	GCF_014163655.1	375	Bacteria>Proteobacteria>Gammaproteobacteria>Alteromonadales>Pseudoalteromonadaceae	Pseudoalteromonas_sp._SR45-1	cysteine-desulfurase-DndA-[Pseudoalteromonas-sp.-SR45-1]	2760932
WP_166377561.1	cysteine-desulfurase	GCF_011378845.1	375	Bacteria>Proteobacteria>Gammaproteobacteria>Alteromonadales>Pseudoalteromonadaceae	Pseudoalteromonas_sp._Z9A4	cysteine-desulfurase-DndA-[Pseudoalteromonas-sp.-Z9A4]	2686353
WP_011329739.1	cysteine-desulfurase	GCF_000026085.1	375	Bacteria>Proteobacteria>Gammaproteobacteria>Alteromonadales>Pseudoalteromonadaceae	Pseudoalteromonas_translucida	cysteine-desulfurase-DndA-[Pseudoalteromonas-translucida]	166935
WP_086745146.1	cysteine-desulfurase	GCF_002156545.1	374	Bacteria>Proteobacteria>Gammaproteobacteria>Alteromonadales>Pseudoalteromonadaceae	Pseudoalteromonas_ulvae	cysteine-desulfurase-DndA-[Pseudoalteromonas-ulvae]	107327
WP_101106969.1	cysteine-desulfurase	GCF_002835465.1	374	Bacteria>Proteobacteria>Gammaproteobacteria>Alteromonadales>Psychromonadaceae	Psychromonas_sp._psych-6C06	cysteine-desulfurase-DndA-[Psychromonas-sp.-psych-6C06]	2058089
WP_121851616.1	cysteine-desulfurase	GCF_004358345.1	374	Bacteria>Proteobacteria>Gammaproteobacteria>Alteromonadales>Shewanellaceae	Parashewanella_spongiae	cysteine-desulfurase-DndA-[Parashewanella-spongiae]	342950
WP_208144361.1	cysteine-desulfurase	-	374	Bacteria>Proteobacteria>Gammaproteobacteria>Alteromonadales>Shewanellaceae	Shewanella_algae	cysteine-desulfurase-DndA-[Shewanella-algae]	38313
WP_045283612.1	cysteine-desulfurase	GCF_017580335.1	351	Bacteria>Proteobacteria>Gammaproteobacteria>Alteromonadales>Shewanellaceae	Shewanella_algae	cysteine-desulfurase-[Shewanella-algae]	38313
WP_235585864.1	cysteine-desulfurase	GCF_001529365.1	418	Bacteria>Proteobacteria>Gammaproteobacteria>Alteromonadales>Shewanellaceae	Shewanella_frigidimarina	cysteine-desulfurase-DndA-[Shewanella-frigidimarina]	56812
WP_041412966.1	cysteine-desulfurase	GCF_000014705.1	373	Bacteria>Proteobacteria>Gammaproteobacteria>Alteromonadales>Shewanellaceae	Shewanella_frigidimarina	cysteine-desulfurase-[Shewanella-frigidimarina]	56812
ABI71938.1	cysteine-desulfurase	GCA_000014705.1	353	Bacteria>Proteobacteria>Gammaproteobacteria>Alteromonadales>Shewanellaceae	Shewanella_frigidimarina_NCIMB_400	aminotransferase,-class-V-[Shewanella-frigidimarina-NCIMB-400]	318167
MCC4832823.1	cysteine-desulfurase	GCA_020806285.1	372	Bacteria>Proteobacteria>Gammaproteobacteria>Alteromonadales>Shewanellaceae	Shewanella_sp._10N.7	cysteine-desulfurase-[Shewanella-sp.-10N.7]	2885093
WP_229369487.1	cysteine-desulfurase	GCF_020806285.1	372	Bacteria>Proteobacteria>Gammaproteobacteria>Alteromonadales>Shewanellaceae	Shewanella_sp._10N.7	cysteine-desulfurase-[Shewanella-sp.-10N.7]	2885093
WP_101087131.1	cysteine-desulfurase	GCF_002836315.1	373	Bacteria>Proteobacteria>Gammaproteobacteria>Alteromonadales>Shewanellaceae	Shewanella_sp._ALD9	cysteine-desulfurase-[Shewanella-sp.-ALD9]	2058330
WP_220049638.1	cysteine-desulfurase	GCF_019457475.1	374	Bacteria>Proteobacteria>Gammaproteobacteria>Alteromonadales>Shewanellaceae	Shewanella_sp._FJAT-51860	cysteine-desulfurase-DndA-[Shewanella-sp.-FJAT-51860]	2864212
WP_137227207.1	cysteine-desulfurase	GCF_005234075.1	373	Bacteria>Proteobacteria>Gammaproteobacteria>Alteromonadales>Shewanellaceae	Shewanella_sp._MEBiC00475	cysteine-desulfurase-[Shewanella-sp.-MEBiC00475]	2575361
WP_240132759.1	cysteine-desulfurase	GCF_022410515.1	351	Bacteria>Proteobacteria>Gammaproteobacteria>Alteromonadales>Shewanellaceae	Shewanella_sp._PS-2	aminotransferase-class-V-fold-PLP-dependent-enzyme-[Shewanella-sp.-PS-2]	2766780
PUA28814.1	cysteine-desulfurase	GCA_003056245.1	374	Bacteria>Proteobacteria>Gammaproteobacteria>Cellvibrionales>Cellvibrionaceae	Cellvibrio_sp._79	cysteine-desulfurase-DndA-[Cellvibrio-sp.-79]	1954207
WP_028884884.1	cysteine-desulfurase	GCF_000426805.1	374	Bacteria>Proteobacteria>Gammaproteobacteria>Cellvibrionales>Cellvibrionaceae	Teredinibacter_turnerae	aminotransferase-class-V-fold-PLP-dependent-enzyme-[Teredinibacter-turnerae]	2426
WP_097112699.1	cysteine-desulfurase	GCF_900215315.1	374	Bacteria>Proteobacteria>Gammaproteobacteria>Chromatiales>Chromatiaceae	Arsukibacterium_tuosuense	cysteine-desulfurase-DndA-[Rheinheimera-tuosuensis]	1323745
WP_046519021.1	cysteine-desulfurase	GCF_000986865.1	375	Bacteria>Proteobacteria>Gammaproteobacteria>Chromatiales>Chromatiaceae	Pararheinheimera_mesophila	cysteine-desulfurase-DndA-[Pararheinheimera-mesophila]	1547515
WP_230819861.1	cysteine-desulfurase	GCF_021021375.1	374	Bacteria>Proteobacteria>Gammaproteobacteria>Chromatiales>Chromatiaceae	Rheinheimera_aquimaris	cysteine-desulfurase-DndA-[Rheinheimera-aquimaris]	412437
WP_027671814.1	cysteine-desulfurase	GCF_000425345.1	374	Bacteria>Proteobacteria>Gammaproteobacteria>Chromatiales>Chromatiaceae	Rheinheimera_baltica	cysteine-desulfurase-DndA-[Rheinheimera-baltica]	67576
MCC5452861.1	cysteine-desulfurase	GCA_020819615.1	373	Bacteria>Proteobacteria>Gammaproteobacteria>Chromatiales>Chromatiaceae	Rheinheimera_sp._UJ51	cysteine-desulfurase-[Rheinheimera-sp.-UJ51]	2892446
WP_229587684.1	cysteine-desulfurase	GCF_020819615.1	373	Bacteria>Proteobacteria>Gammaproteobacteria>Chromatiales>Chromatiaceae	Rheinheimera_sp._UJ51	cysteine-desulfurase-[Rheinheimera-sp.-UJ51]	2892446
WP_200373298.1	cysteine-desulfurase	-	140	Bacteria>Proteobacteria>Gammaproteobacteria>Chromatiales>Chromatiaceae	Thiocystis_violacea	aminotransferase-class-V-fold-PLP-dependent-enzyme-[Thiocystis-violacea]	13725
MBK1716527.1	cysteine-desulfurase	GCA_016583575.1	146	Bacteria>Proteobacteria>Gammaproteobacteria>Chromatiales>Chromatiaceae	Thiocystis_violacea	hypothetical-protein-[Thiocystis-violacea]	13725
WP_015281553.1	cysteine-desulfurase	GCF_000327045.1	376	Bacteria>Proteobacteria>Gammaproteobacteria>Chromatiales>Chromatiaceae	Thioflavicoccus_mobilis	cysteine-desulfurase-DndA-[Thioflavicoccus-mobilis]	80679
WP_090207309.1	cysteine-desulfurase	GCF_900110965.1	384	Bacteria>Proteobacteria>Gammaproteobacteria>Chromatiales>Ectothiorhodospiraceae	Ectothiorhodospira_magna	cysteine-desulfurase-[Ectothiorhodospira-magna]	867345
WP_208326156.1	cysteine-desulfurase	-	355	Bacteria>Proteobacteria>Gammaproteobacteria>Enterobacterales>Enterobacteriaceae	Buttiauxella_sp._BIGb0552	cysteine-desulfurase-[Buttiauxella-sp.-BIGb0552]	2485120
WP_121572219.1	cysteine-desulfurase	GCF_003665615.1	353	Bacteria>Proteobacteria>Gammaproteobacteria>Enterobacterales>Enterobacteriaceae	Citrobacter_freundii	cysteine-desulfurase-[Citrobacter-freundii]	546
SQB25926.1	cysteine-desulfurase	GCA_900446925.1	120	Bacteria>Proteobacteria>Gammaproteobacteria>Enterobacterales>Enterobacteriaceae	Citrobacter_koseri	cysteine-desulfurase-[Citrobacter-koseri]	545
MBJ9245478.1	cysteine-desulfurase	GCA_016504805.1	374	Bacteria>Proteobacteria>Gammaproteobacteria>Enterobacterales>Enterobacteriaceae	Citrobacter_koseri	cysteine-desulfurase-DndA-[Citrobacter-koseri]	545
WP_040230272.1	cysteine-desulfurase	GCF_000826205.1	353	Bacteria>Proteobacteria>Gammaproteobacteria>Enterobacterales>Enterobacteriaceae	Citrobacter	MULTISPECIES:-cysteine-desulfurase-[Citrobacter]	544
WP_195296446.1	cysteine-desulfurase	GCF_015549445.1	374	Bacteria>Proteobacteria>Gammaproteobacteria>Enterobacterales>Enterobacteriaceae	Citrobacter	MULTISPECIES:-cysteine-desulfurase-DndA-[Citrobacter]	544
WP_172742361.1	cysteine-desulfurase	-	353	Bacteria>Proteobacteria>Gammaproteobacteria>Enterobacterales>Enterobacteriaceae	Citrobacter_pasteurii	cysteine-desulfurase-[Citrobacter-pasteurii]	1563222
WP_111771462.1	cysteine-desulfurase	GCF_005280815.1	374	Bacteria>Proteobacteria>Gammaproteobacteria>Enterobacterales>Enterobacteriaceae	Citrobacter_sp._TBCS-15	cysteine-desulfurase-DndA-[Citrobacter-sp.-TBCS-15]	2576407
WP_085661416.1	cysteine-desulfurase	GCF_017845305.1	353	Bacteria>Proteobacteria>Gammaproteobacteria>Enterobacterales>Enterobacteriaceae	Cronobacter_sakazakii	cysteine-desulfurase-[Cronobacter-sakazakii]	28141
WP_023619933.1	cysteine-desulfurase	GCF_025311535.1	374	Bacteria>Proteobacteria>Gammaproteobacteria>Enterobacterales>Enterobacteriaceae	Enterobacter_hormaechei	cysteine-desulfurase-DndA-[Enterobacter-hormaechei]	158836
WP_180262789.1	cysteine-desulfurase	GCF_900559085.1	374	Bacteria>Proteobacteria>Gammaproteobacteria>Enterobacterales>Enterobacteriaceae	Enterobacter_hormaechei	cysteine-desulfurase-DndA-[Enterobacter-hormaechei]	158836
MCE1383922.1	cysteine-desulfurase	GCA_021285215.1	353	Bacteria>Proteobacteria>Gammaproteobacteria>Enterobacterales>Enterobacteriaceae	Enterobacter_hormaechei	cysteine-desulfurase-[Enterobacter-hormaechei]	158836
SAE87888.1	cysteine-desulfurase	GCA_900077405.1	374	Bacteria>Proteobacteria>Gammaproteobacteria>Enterobacterales>Enterobacteriaceae	Enterobacter_hormaechei	cysteine-desulfurase-[Enterobacter-hormaechei]	158836
MBT2011241.1	cysteine-desulfurase	GCA_018597865.1	374	Bacteria>Proteobacteria>Gammaproteobacteria>Enterobacterales>Enterobacteriaceae	Enterobacter_hormaechei_subsp._xiangfangensis	cysteine-desulfurase-DndA-[Enterobacter-hormaechei-subsp.-xiangfangensis]	1296536
HAV1602348.1	cysteine-desulfurase	GCA_016430175.1	353	Bacteria>Proteobacteria>Gammaproteobacteria>Enterobacterales>Enterobacteriaceae	Enterobacter_hormaechei_subsp._xiangfangensis	cysteine-desulfurase-[Enterobacter-hormaechei-subsp.-xiangfangensis]	1296536
MBX9029825.1	cysteine-desulfurase	GCA_019731755.1	374	Bacteria>Proteobacteria>Gammaproteobacteria>Enterobacterales>Enterobacteriaceae	Enterobacter_ludwigii	cysteine-desulfurase-DndA-[Enterobacter-ludwigii]	299767
WP_221716679.1	cysteine-desulfurase	GCF_019731755.1	374	Bacteria>Proteobacteria>Gammaproteobacteria>Enterobacterales>Enterobacteriaceae	Enterobacter_ludwigii	cysteine-desulfurase-DndA-[Enterobacter-ludwigii]	299767
WP_163327004.1	cysteine-desulfurase	GCF_010590225.1	353	Bacteria>Proteobacteria>Gammaproteobacteria>Enterobacterales>Enterobacteriaceae	Enterobacter_ludwigii	cysteine-desulfurase-[Enterobacter-ludwigii]	299767
WP_248245159.1	cysteine-desulfurase	GCF_025535675.1	353	Bacteria>Proteobacteria>Gammaproteobacteria>Enterobacterales>Enterobacteriaceae	Enterobacter_roggenkampii	aminotransferase-class-V-fold-PLP-dependent-enzyme-[Enterobacter-roggenkampii]	1812935
WP_155030632.1	cysteine-desulfurase	GCF_009036245.1	374	Bacteria>Proteobacteria>Gammaproteobacteria>Enterobacterales>Enterobacteriaceae	Enterobacter_sichuanensis	cysteine-desulfurase-DndA-[Enterobacter-sichuanensis]	2071710
WP_014068705.1	cysteine-desulfurase	GCF_000224675.1	374	Bacteria>Proteobacteria>Gammaproteobacteria>Enterobacterales>Enterobacteriaceae	Enterobacter_soli	cysteine-desulfurase-DndA-[Enterobacter-soli]	885040
WP_221551581.1	cysteine-desulfurase	GCF_019718755.1	374	Bacteria>Proteobacteria>Gammaproteobacteria>Enterobacterales>Enterobacteriaceae	Enterobacter_sp._RIT637	cysteine-desulfurase-DndA-[Enterobacter-sp.-RIT637]	2870470
HBB3925495.1	cysteine-desulfurase	GCA_017891325.1	353	Bacteria>Proteobacteria>Gammaproteobacteria>Enterobacterales>Enterobacteriaceae	Escherichia_coli	cysteine-desulfurase-[Escherichia-coli]	562
WP_174562838.1	cysteine-desulfurase	GCF_013341605.1	353	Bacteria>Proteobacteria>Gammaproteobacteria>Enterobacterales>Enterobacteriaceae	Escherichia_coli	cysteine-desulfurase-[Escherichia-coli]	562
WP_217061544.1	cysteine-desulfurase	GCF_019047925.1	353	Bacteria>Proteobacteria>Gammaproteobacteria>Enterobacterales>Enterobacteriaceae	Klebsiella_aerogenes	aminotransferase-class-V-fold-PLP-dependent-enzyme-[Klebsiella-aerogenes]	548
WP_126033573.1	cysteine-desulfurase	GCF_003952125.1	374	Bacteria>Proteobacteria>Gammaproteobacteria>Enterobacterales>Enterobacteriaceae	Klebsiella_aerogenes	cysteine-desulfurase-DndA-[Klebsiella-aerogenes]	548
WP_193128989.1	cysteine-desulfurase	GCF_014902795.1	374	Bacteria>Proteobacteria>Gammaproteobacteria>Enterobacterales>Enterobacteriaceae	Klebsiella_aerogenes	cysteine-desulfurase-DndA-[Klebsiella-aerogenes]	548
HBM3157254.1	cysteine-desulfurase	GCA_021210555.1	240	Bacteria>Proteobacteria>Gammaproteobacteria>Enterobacterales>Enterobacteriaceae	Klebsiella_michiganensis	TPA:-aminotransferase-class-V-fold-PLP-dependent-enzyme,-partial-[Klebsiella-michiganensis]	1134687
WP_110221272.1	cysteine-desulfurase	GCF_013895695.1	353	Bacteria>Proteobacteria>Gammaproteobacteria>Enterobacterales>Enterobacteriaceae	Klebsiella	MULTISPECIES:-cysteine-desulfurase-[Klebsiella]	570
QGN36341.1	cysteine-desulfurase	GCA_009707385.1	374	Bacteria>Proteobacteria>Gammaproteobacteria>Enterobacterales>Enterobacteriaceae	Klebsiella_oxytoca	cysteine-desulfurase-DndA-[Klebsiella-oxytoca]	571
WP_154678881.1	cysteine-desulfurase	GCF_009707385.1	374	Bacteria>Proteobacteria>Gammaproteobacteria>Enterobacterales>Enterobacteriaceae	Klebsiella_oxytoca	cysteine-desulfurase-DndA-[Klebsiella-oxytoca]	571

Accession	Annotation	GCA	Length	Phyletic.spread	Strain.name	Defline	Species.Taxid
WP_134155554.1	cysteine-desulfurase	GCF_900497475.1	374	Bacteria>Proteobacteria>Gammaproteobacteria>Enterobacterales>Enterobacteriaceae	Klebsiella_pneumoniae	cysteine-desulfurase-DndA-[Klebsiella-pneumoniae]	573
WP_117047409.1	cysteine-desulfurase	GCF_900503965.1	353	Bacteria>Proteobacteria>Gammaproteobacteria>Enterobacterales>Enterobacteriaceae	Klebsiella_pneumoniae	cysteine-desulfurase-[Klebsiella-pneumoniae]	573
WP_101866852.1	cysteine-desulfurase	GCF_022585355.1	353	Bacteria>Proteobacteria>Gammaproteobacteria>Enterobacterales>Enterobacteriaceae	Klebsiella_quasipneumoniae	cysteine-desulfurase-[Klebsiella-quasipneumoniae]	1463165
WP_110199977.1	cysteine-desulfurase	GCF_003196975.1	374	Bacteria>Proteobacteria>Gammaproteobacteria>Enterobacterales>Enterobacteriaceae	Klebsiella_variicola	cysteine-desulfurase-DndA-[Klebsiella-variicola]	244366
WP_177334783.1	cysteine-desulfurase	GCF_902509095.1	374	Bacteria>Proteobacteria>Gammaproteobacteria>Enterobacterales>Enterobacteriaceae	Klebsiella_variicola	cysteine-desulfurase-DndA-[Klebsiella-variicola]	244366
WP_231276091.1	cysteine-desulfurase	GCF_021083665.1	353	Bacteria>Proteobacteria>Gammaproteobacteria>Enterobacterales>Enterobacteriaceae	Lelliottia_nimipressuralis	aminotransferase-class-V-fold-PLP-dependent-enzyme-[Lelliottia-nimipressuralis]	69220
WP_218574002.1	cysteine-desulfurase	GCF_009183605.1	356	Bacteria>Proteobacteria>Gammaproteobacteria>Enterobacterales>Enterobacteriaceae	Plesiomonas_shigelloides	aminotransferase-class-V-fold-PLP-dependent-enzyme-[Plesiomonas-shigelloides]	703
WP_152106761.1	cysteine-desulfurase	GCF_009183395.1	351	Bacteria>Proteobacteria>Gammaproteobacteria>Enterobacterales>Enterobacteriaceae	Plesiomonas_shigelloides	cysteine-desulfurase-[Plesiomonas-shigelloides]	703
EDQ5146974.1	cysteine-desulfurase	GCA_010401985.1	150	Bacteria>Proteobacteria>Gammaproteobacteria>Enterobacterales>Enterobacteriaceae	Salmonella_enterica	aminotransferase-class-V-fold-PLP-dependent-enzyme,-partial-[Salmonella-enterica]	28901
EAA9345543.1	cysteine-desulfurase	GCA_004226385.1	374	Bacteria>Proteobacteria>Gammaproteobacteria>Enterobacterales>Enterobacteriaceae	Salmonella_enterica	cysteine-desulfurase-DndA-[Salmonella-enterica]	28901
EAA9955124.1	cysteine-desulfurase	GCA_004229005.1	374	Bacteria>Proteobacteria>Gammaproteobacteria>Enterobacterales>Enterobacteriaceae	Salmonella_enterica	cysteine-desulfurase-DndA-[Salmonella-enterica]	28901
EAB0102596.1	cysteine-desulfurase	GCA_004229265.1	374	Bacteria>Proteobacteria>Gammaproteobacteria>Enterobacterales>Enterobacteriaceae	Salmonella_enterica	cysteine-desulfurase-DndA-[Salmonella-enterica]	28901
EAB0248291.1	cysteine-desulfurase	GCA_004228725.1	374	Bacteria>Proteobacteria>Gammaproteobacteria>Enterobacterales>Enterobacteriaceae	Salmonella_enterica	cysteine-desulfurase-DndA-[Salmonella-enterica]	28901
EAB0290135.1	cysteine-desulfurase	GCA_004230155.1	374	Bacteria>Proteobacteria>Gammaproteobacteria>Enterobacterales>Enterobacteriaceae	Salmonella_enterica	cysteine-desulfurase-DndA-[Salmonella-enterica]	28901
EAB4413791.1	cysteine-desulfurase	GCA_004247645.1	374	Bacteria>Proteobacteria>Gammaproteobacteria>Enterobacterales>Enterobacteriaceae	Salmonella_enterica	cysteine-desulfurase-DndA-[Salmonella-enterica]	28901
EAN6838701.1	cysteine-desulfurase	GCA_005472815.1	374	Bacteria>Proteobacteria>Gammaproteobacteria>Enterobacterales>Enterobacteriaceae	Salmonella_enterica	cysteine-desulfurase-DndA-[Salmonella-enterica]	28901
EAO9627568.1	cysteine-desulfurase	GCA_005533535.1	374	Bacteria>Proteobacteria>Gammaproteobacteria>Enterobacterales>Enterobacteriaceae	Salmonella_enterica	cysteine-desulfurase-DndA-[Salmonella-enterica]	28901
EAP0687436.1	cysteine-desulfurase	GCA_005538375.1	374	Bacteria>Proteobacteria>Gammaproteobacteria>Enterobacterales>Enterobacteriaceae	Salmonella_enterica	cysteine-desulfurase-DndA-[Salmonella-enterica]	28901
EAQ3358334.1	cysteine-desulfurase	GCA_005594955.1	374	Bacteria>Proteobacteria>Gammaproteobacteria>Enterobacterales>Enterobacteriaceae	Salmonella_enterica	cysteine-desulfurase-DndA-[Salmonella-enterica]	28901
EAS1741978.1	cysteine-desulfurase	GCA_005676095.1	374	Bacteria>Proteobacteria>Gammaproteobacteria>Enterobacterales>Enterobacteriaceae	Salmonella_enterica	cysteine-desulfurase-DndA-[Salmonella-enterica]	28901
EAS4117488.1	cysteine-desulfurase	GCA_005686715.1	374	Bacteria>Proteobacteria>Gammaproteobacteria>Enterobacterales>Enterobacteriaceae	Salmonella_enterica	cysteine-desulfurase-DndA-[Salmonella-enterica]	28901
EAS4728346.1	cysteine-desulfurase	GCA_005689195.1	374	Bacteria>Proteobacteria>Gammaproteobacteria>Enterobacterales>Enterobacteriaceae	Salmonella_enterica	cysteine-desulfurase-DndA-[Salmonella-enterica]	28901
EAS9544734.1	cysteine-desulfurase	GCA_005712415.1	374	Bacteria>Proteobacteria>Gammaproteobacteria>Enterobacterales>Enterobacteriaceae	Salmonella_enterica	cysteine-desulfurase-DndA-[Salmonella-enterica]	28901
EAT1573620.1	cysteine-desulfurase	GCA_005719955.1	374	Bacteria>Proteobacteria>Gammaproteobacteria>Enterobacterales>Enterobacteriaceae	Salmonella_enterica	cysteine-desulfurase-DndA-[Salmonella-enterica]	28901
EAV2276320.1	cysteine-desulfurase	GCA_005833045.1	374	Bacteria>Proteobacteria>Gammaproteobacteria>Enterobacterales>Enterobacteriaceae	Salmonella_enterica	cysteine-desulfurase-DndA-[Salmonella-enterica]	28901
EAW3258764.1	cysteine-desulfurase	GCA_005907885.1	374	Bacteria>Proteobacteria>Gammaproteobacteria>Enterobacterales>Enterobacteriaceae	Salmonella_enterica	cysteine-desulfurase-DndA-[Salmonella-enterica]	28901
EAW6002186.1	cysteine-desulfurase	GCA_005918065.1	374	Bacteria>Proteobacteria>Gammaproteobacteria>Enterobacterales>Enterobacteriaceae	Salmonella_enterica	cysteine-desulfurase-DndA-[Salmonella-enterica]	28901
EAX0430747.1	cysteine-desulfurase	GCA_005944545.1	374	Bacteria>Proteobacteria>Gammaproteobacteria>Enterobacterales>Enterobacteriaceae	Salmonella_enterica	cysteine-desulfurase-DndA-[Salmonella-enterica]	28901
EAX0547785.1	cysteine-desulfurase	GCA_005945265.1	374	Bacteria>Proteobacteria>Gammaproteobacteria>Enterobacterales>Enterobacteriaceae	Salmonella_enterica	cysteine-desulfurase-DndA-[Salmonella-enterica]	28901
EAX1037890.1	cysteine-desulfurase	GCA_005948005.1	374	Bacteria>Proteobacteria>Gammaproteobacteria>Enterobacterales>Enterobacteriaceae	Salmonella_enterica	cysteine-desulfurase-DndA-[Salmonella-enterica]	28901
EAX1104985.1	cysteine-desulfurase	GCA_005948185.1	374	Bacteria>Proteobacteria>Gammaproteobacteria>Enterobacterales>Enterobacteriaceae	Salmonella_enterica	cysteine-desulfurase-DndA-[Salmonella-enterica]	28901
EAX1133003.1	cysteine-desulfurase	GCA_005948405.1	374	Bacteria>Proteobacteria>Gammaproteobacteria>Enterobacterales>Enterobacteriaceae	Salmonella_enterica	cysteine-desulfurase-DndA-[Salmonella-enterica]	28901
EAX1153001.1	cysteine-desulfurase	GCA_005949125.1	374	Bacteria>Proteobacteria>Gammaproteobacteria>Enterobacterales>Enterobacteriaceae	Salmonella_enterica	cysteine-desulfurase-DndA-[Salmonella-enterica]	28901
EAY0142721.1	cysteine-desulfurase	GCA_005988305.1	374	Bacteria>Proteobacteria>Gammaproteobacteria>Enterobacterales>Enterobacteriaceae	Salmonella_enterica	cysteine-desulfurase-DndA-[Salmonella-enterica]	28901
EAY9333914.1	cysteine-desulfurase	GCA_006029335.1	374	Bacteria>Proteobacteria>Gammaproteobacteria>Enterobacterales>Enterobacteriaceae	Salmonella_enterica	cysteine-desulfurase-DndA-[Salmonella-enterica]	28901
EBA2396204.1	cysteine-desulfurase	GCA_006087635.1	374	Bacteria>Proteobacteria>Gammaproteobacteria>Enterobacterales>Enterobacteriaceae	Salmonella_enterica	cysteine-desulfurase-DndA-[Salmonella-enterica]	28901
EBA2832718.1	cysteine-desulfurase	GCA_006091275.1	374	Bacteria>Proteobacteria>Gammaproteobacteria>Enterobacterales>Enterobacteriaceae	Salmonella_enterica	cysteine-desulfurase-DndA-[Salmonella-enterica]	28901
EBA2865042.1	cysteine-desulfurase	GCA_006091215.1	374	Bacteria>Proteobacteria>Gammaproteobacteria>Enterobacterales>Enterobacteriaceae	Salmonella_enterica	cysteine-desulfurase-DndA-[Salmonella-enterica]	28901
EBA2870298.1	cysteine-desulfurase	GCA_006091315.1	374	Bacteria>Proteobacteria>Gammaproteobacteria>Enterobacterales>Enterobacteriaceae	Salmonella_enterica	cysteine-desulfurase-DndA-[Salmonella-enterica]	28901
EBA2926769.1	cysteine-desulfurase	GCA_006089895.1	374	Bacteria>Proteobacteria>Gammaproteobacteria>Enterobacterales>Enterobacteriaceae	Salmonella_enterica	cysteine-desulfurase-DndA-[Salmonella-enterica]	28901
EBA5752882.1	cysteine-desulfurase	GCA_006103275.1	374	Bacteria>Proteobacteria>Gammaproteobacteria>Enterobacterales>Enterobacteriaceae	Salmonella_enterica	cysteine-desulfurase-DndA-[Salmonella-enterica]	28901
EBB4168996.1	cysteine-desulfurase	GCA_006141135.1	374	Bacteria>Proteobacteria>Gammaproteobacteria>Enterobacterales>Enterobacteriaceae	Salmonella_enterica	cysteine-desulfurase-DndA-[Salmonella-enterica]	28901
EBC2382468.1	cysteine-desulfurase	GCA_006180645.1	374	Bacteria>Proteobacteria>Gammaproteobacteria>Enterobacterales>Enterobacteriaceae	Salmonella_enterica	cysteine-desulfurase-DndA-[Salmonella-enterica]	28901
EBC4298999.1	cysteine-desulfurase	GCA_006186405.1	374	Bacteria>Proteobacteria>Gammaproteobacteria>Enterobacterales>Enterobacteriaceae	Salmonella_enterica	cysteine-desulfurase-DndA-[Salmonella-enterica]	28901
EBC6584373.1	cysteine-desulfurase	GCA_006198885.1	374	Bacteria>Proteobacteria>Gammaproteobacteria>Enterobacterales>Enterobacteriaceae	Salmonella_enterica	cysteine-desulfurase-DndA-[Salmonella-enterica]	28901
EBD0574545.1	cysteine-desulfurase	GCA_006215975.1	374	Bacteria>Proteobacteria>Gammaproteobacteria>Enterobacterales>Enterobacteriaceae	Salmonella_enterica	cysteine-desulfurase-DndA-[Salmonella-enterica]	28901
EBF8990337.1	cysteine-desulfurase	GCA_006398655.1	374	Bacteria>Proteobacteria>Gammaproteobacteria>Enterobacterales>Enterobacteriaceae	Salmonella_enterica	cysteine-desulfurase-DndA-[Salmonella-enterica]	28901
EBF9289779.1	cysteine-desulfurase	GCA_006400355.1	374	Bacteria>Proteobacteria>Gammaproteobacteria>Enterobacterales>Enterobacteriaceae	Salmonella_enterica	cysteine-desulfurase-DndA-[Salmonella-enterica]	28901
EBH3200349.1	cysteine-desulfurase	GCA_006486285.1	374	Bacteria>Proteobacteria>Gammaproteobacteria>Enterobacterales>Enterobacteriaceae	Salmonella_enterica	cysteine-desulfurase-DndA-[Salmonella-enterica]	28901
EBI3283595.1	cysteine-desulfurase	GCA_006550975.1	374	Bacteria>Proteobacteria>Gammaproteobacteria>Enterobacterales>Enterobacteriaceae	Salmonella_enterica	cysteine-desulfurase-DndA-[Salmonella-enterica]	28901
EBL2556442.1	cysteine-desulfurase	GCA_006678145.1	374	Bacteria>Proteobacteria>Gammaproteobacteria>Enterobacterales>Enterobacteriaceae	Salmonella_enterica	cysteine-desulfurase-DndA-[Salmonella-enterica]	28901
EBN9070841.1	cysteine-desulfurase	GCA_006793285.1	374	Bacteria>Proteobacteria>Gammaproteobacteria>Enterobacterales>Enterobacteriaceae	Salmonella_enterica	cysteine-desulfurase-DndA-[Salmonella-enterica]	28901
EBP4718459.1	cysteine-desulfurase	GCA_006867645.1	374	Bacteria>Proteobacteria>Gammaproteobacteria>Enterobacterales>Enterobacteriaceae	Salmonella_enterica	cysteine-desulfurase-DndA-[Salmonella-enterica]	28901
EBP5433875.1	cysteine-desulfurase	GCA_006870525.1	374	Bacteria>Proteobacteria>Gammaproteobacteria>Enterobacterales>Enterobacteriaceae	Salmonella_enterica	cysteine-desulfurase-DndA-[Salmonella-enterica]	28901
EBP5571012.1	cysteine-desulfurase	GCA_006871045.1	374	Bacteria>Proteobacteria>Gammaproteobacteria>Enterobacterales>Enterobacteriaceae	Salmonella_enterica	cysteine-desulfurase-DndA-[Salmonella-enterica]	28901
EBP6085705.1	cysteine-desulfurase	GCA_006872585.1	374	Bacteria>Proteobacteria>Gammaproteobacteria>Enterobacterales>Enterobacteriaceae	Salmonella_enterica	cysteine-desulfurase-DndA-[Salmonella-enterica]	28901
EBP6147475.1	cysteine-desulfurase	GCA_006872705.1	374	Bacteria>Proteobacteria>Gammaproteobacteria>Enterobacterales>Enterobacteriaceae	Salmonella_enterica	cysteine-desulfurase-DndA-[Salmonella-enterica]	28901
EBP6316054.1	cysteine-desulfurase	GCA_006873705.1	374	Bacteria>Proteobacteria>Gammaproteobacteria>Enterobacterales>Enterobacteriaceae	Salmonella_enterica	cysteine-desulfurase-DndA-[Salmonella-enterica]	28901
ECC1569701.1	cysteine-desulfurase	GCA_007463475.1	374	Bacteria>Proteobacteria>Gammaproteobacteria>Enterobacterales>Enterobacteriaceae	Salmonella_enterica	cysteine-desulfurase-DndA-[Salmonella-enterica]	28901
ECG0564356.1	cysteine-desulfurase	GCA_007642775.1	374	Bacteria>Proteobacteria>Gammaproteobacteria>Enterobacterales>Enterobacteriaceae	Salmonella_enterica	cysteine-desulfurase-DndA-[Salmonella-enterica]	28901
ECJ8303969.1	cysteine-desulfurase	GCA_007843875.1	374	Bacteria>Proteobacteria>Gammaproteobacteria>Enterobacterales>Enterobacteriaceae	Salmonella_enterica	cysteine-desulfurase-DndA-[Salmonella-enterica]	28901
ECK0859478.1	cysteine-desulfurase	GCA_007861425.1	374	Bacteria>Proteobacteria>Gammaproteobacteria>Enterobacterales>Enterobacteriaceae	Salmonella_enterica	cysteine-desulfurase-DndA-[Salmonella-enterica]	28901
ECK5242947.1	cysteine-desulfurase	GCA_007883045.1	374	Bacteria>Proteobacteria>Gammaproteobacteria>Enterobacterales>Enterobacteriaceae	Salmonella_enterica	cysteine-desulfurase-DndA-[Salmonella-enterica]	28901
ECL1540936.1	cysteine-desulfurase	GCA_007926305.1	374	Bacteria>Proteobacteria>Gammaproteobacteria>Enterobacterales>Enterobacteriaceae	Salmonella_enterica	cysteine-desulfurase-DndA-[Salmonella-enterica]	28901
ECP3214155.1	cysteine-desulfurase	GCA_008190075.1	374	Bacteria>Proteobacteria>Gammaproteobacteria>Enterobacterales>Enterobacteriaceae	Salmonella_enterica	cysteine-desulfurase-DndA-[Salmonella-enterica]	28901
ECR0661974.1	cysteine-desulfurase	GCA_008319125.1	374	Bacteria>Proteobacteria>Gammaproteobacteria>Enterobacterales>Enterobacteriaceae	Salmonella_enterica	cysteine-desulfurase-DndA-[Salmonella-enterica]	28901
ECR5628581.1	cysteine-desulfurase	GCA_008358145.1	374	Bacteria>Proteobacteria>Gammaproteobacteria>Enterobacterales>Enterobacteriaceae	Salmonella_enterica	cysteine-desulfurase-DndA-[Salmonella-enterica]	28901
ECT9549643.1	cysteine-desulfurase	GCA_008488425.1	374	Bacteria>Proteobacteria>Gammaproteobacteria>Enterobacterales>Enterobacteriaceae	Salmonella_enterica	cysteine-desulfurase-DndA-[Salmonella-enterica]	28901
EDK9437801.1	cysteine-desulfurase	GCA_009483905.1	374	Bacteria>Proteobacteria>Gammaproteobacteria>Enterobacterales>Enterobacteriaceae	Salmonella_enterica	cysteine-desulfurase-DndA-[Salmonella-enterica]	28901
EDT0278170.1	cysteine-desulfurase	GCA_010523455.1	374	Bacteria>Proteobacteria>Gammaproteobacteria>Enterobacterales>Enterobacteriaceae	Salmonella_enterica	cysteine-desulfurase-DndA-[Salmonella-enterica]	28901
EDY3766905.1	cysteine-desulfurase	GCA_010807645.1	374	Bacteria>Proteobacteria>Gammaproteobacteria>Enterobacterales>Enterobacteriaceae	Salmonella_enterica	cysteine-desulfurase-DndA-[Salmonella-enterica]	28901
EDY4445287.1	cysteine-desulfurase	GCA_010810925.1	374	Bacteria>Proteobacteria>Gammaproteobacteria>Enterobacterales>Enterobacteriaceae	Salmonella_enterica	cysteine-desulfurase-DndA-[Salmonella-enterica]	28901
EDZ6569003.1	cysteine-desulfurase	GCA_010859125.1	374	Bacteria>Proteobacteria>Gammaproteobacteria>Enterobacterales>Enterobacteriaceae	Salmonella_enterica	cysteine-desulfurase-DndA-[Salmonella-enterica]	28901
EEF3127526.1	cysteine-desulfurase	GCA_011175615.1	374	Bacteria>Proteobacteria>Gammaproteobacteria>Enterobacterales>Enterobacteriaceae	Salmonella_enterica	cysteine-desulfurase-DndA-[Salmonella-enterica]	28901
EEG6593116.1	cysteine-desulfurase	GCA_011243875.1	374	Bacteria>Proteobacteria>Gammaproteobacteria>Enterobacterales>Enterobacteriaceae	Salmonella_enterica	cysteine-desulfurase-DndA-[Salmonella-enterica]	28901
EEW9755247.1	cysteine-desulfurase	GCA_012106055.1	374	Bacteria>Proteobacteria>Gammaproteobacteria>Enterobacterales>Enterobacteriaceae	Salmonella_enterica	cysteine-desulfurase-DndA-[Salmonella-enterica]	28901
EHQ5158493.1	cysteine-desulfurase	GCA_018945535.1	374	Bacteria>Proteobacteria>Gammaproteobacteria>Enterobacterales>Enterobacteriaceae	Salmonella_enterica	cysteine-desulfurase-DndA-[Salmonella-enterica]	28901
EHT8166082.1	cysteine-desulfurase	GCA_019256965.1	374	Bacteria>Proteobacteria>Gammaproteobacteria>Enterobacterales>Enterobacteriaceae	Salmonella_enterica	cysteine-desulfurase-DndA-[Salmonella-enterica]	28901
HAF2128882.1	cysteine-desulfurase	GCA_011226015.1	374	Bacteria>Proteobacteria>Gammaproteobacteria>Enterobacterales>Enterobacteriaceae	Salmonella_enterica	cysteine-desulfurase-DndA-[Salmonella-enterica]	28901
MEG59740.1	cysteine-desulfurase	GCA_003746145.1	374	Bacteria>Proteobacteria>Gammaproteobacteria>Enterobacterales>Enterobacteriaceae	Salmonella_enterica	cysteine-desulfurase-DndA-[Salmonella-enterica]	28901
WP_000158337.1	cysteine-desulfurase	GCF_000170195.2	374	Bacteria>Proteobacteria>Gammaproteobacteria>Enterobacterales>Enterobacteriaceae	Salmonella_enterica	cysteine-desulfurase-DndA-[Salmonella-enterica]	28901
WP_023247627.1	cysteine-desulfurase	GCF_000487635.2	374	Bacteria>Proteobacteria>Gammaproteobacteria>Enterobacterales>Enterobacteriaceae	Salmonella_enterica	cysteine-desulfurase-DndA-[Salmonella-enterica]	28901
WP_058145318.1	cysteine-desulfurase	GCF_002109255.1	374	Bacteria>Proteobacteria>Gammaproteobacteria>Enterobacterales>Enterobacteriaceae	Salmonella_enterica	cysteine-desulfurase-DndA-[Salmonella-enterica]	28901
EBQ4833793.1	cysteine-desulfurase	GCA_006912155.1	374	Bacteria>Proteobacteria>Gammaproteobacteria>Enterobacterales>Enterobacteriaceae	Salmonella_enterica_subsp._arizonae	cysteine-desulfurase-DndA-[Salmonella-enterica-subsp.-arizonae]	59203
EAA9294217.1	cysteine-desulfurase	GCA_004226185.1	374	Bacteria>Proteobacteria>Gammaproteobacteria>Enterobacterales>Enterobacteriaceae	Salmonella_enterica_subsp._enterica	cysteine-desulfurase-DndA-[Salmonella-enterica-subsp.-enterica]	59201
EBG0124146.1	cysteine-desulfurase	GCA_006404315.1	374	Bacteria>Proteobacteria>Gammaproteobacteria>Enterobacterales>Enterobacteriaceae	Salmonella_enterica_subsp._enterica	cysteine-desulfurase-DndA-[Salmonella-enterica-subsp.-enterica]	59201
EBG2839802.1	cysteine-desulfurase	GCA_006419895.1	374	Bacteria>Proteobacteria>Gammaproteobacteria>Enterobacterales>Enterobacteriaceae	Salmonella_enterica_subsp._enterica	cysteine-desulfurase-DndA-[Salmonella-enterica-subsp.-enterica]	59201
EBG5472484.1	cysteine-desulfurase	GCA_006430795.1	374	Bacteria>Proteobacteria>Gammaproteobacteria>Enterobacterales>Enterobacteriaceae	Salmonella_enterica_subsp._enterica	cysteine-desulfurase-DndA-[Salmonella-enterica-subsp.-enterica]	59201
EDR3007039.1	cysteine-desulfurase	GCA_010439755.1	374	Bacteria>Proteobacteria>Gammaproteobacteria>Enterobacterales>Enterobacteriaceae	Salmonella_enterica_subsp._enterica	cysteine-desulfurase-DndA-[Salmonella-enterica-subsp.-enterica]	59201
EGZ9673094.1	cysteine-desulfurase	GCA_016594415.1	374	Bacteria>Proteobacteria>Gammaproteobacteria>Enterobacterales>Enterobacteriaceae	Salmonella_enterica_subsp._enterica_serovar_Daytona	cysteine-desulfurase-DndA-[Salmonella-enterica-subsp.-enterica-serovar-Daytona]	1962639
EBK2662357.1	cysteine-desulfurase	GCA_006635185.1	374	Bacteria>Proteobacteria>Gammaproteobacteria>Enterobacterales>Enterobacteriaceae	Salmonella_enterica_subsp._enterica_serovar_Enteritidis	cysteine-desulfurase-DndA-[Salmonella-enterica-subsp.-enterica-serovar-Enteritidis]	149539
EBC9540807.1	cysteine-desulfurase	GCA_006211945.1	374	Bacteria>Proteobacteria>Gammaproteobacteria>Enterobacterales>Enterobacteriaceae	Salmonella_enterica_subsp._enterica_serovar_Kentucky	cysteine-desulfurase-DndA-[Salmonella-enterica-subsp.-enterica-serovar-Kentucky]	192955
EBE3986668.1	cysteine-desulfurase	GCA_006294895.1	374	Bacteria>Proteobacteria>Gammaproteobacteria>Enterobacterales>Enterobacteriaceae	Salmonella_enterica_subsp._enterica_serovar_Kentucky	cysteine-desulfurase-DndA-[Salmonella-enterica-subsp.-enterica-serovar-Kentucky]	192955
EBL3549660.1	cysteine-desulfurase	GCA_006683605.1	374	Bacteria>Proteobacteria>Gammaproteobacteria>Enterobacterales>Enterobacteriaceae	Salmonella_enterica_subsp._enterica_serovar_Kentucky	cysteine-desulfurase-DndA-[Salmonella-enterica-subsp.-enterica-serovar-Kentucky]	192955
EBL3653321.1	cysteine-desulfurase	GCA_006682345.1	374	Bacteria>Proteobacteria>Gammaproteobacteria>Enterobacterales>Enterobacteriaceae	Salmonella_enterica_subsp._enterica_serovar_Kentucky	cysteine-desulfurase-DndA-[Salmonella-enterica-subsp.-enterica-serovar-Kentucky]	192955
EBL3809778.1	cysteine-desulfurase	GCA_006684125.1	374	Bacteria>Proteobacteria>Gammaproteobacteria>Enterobacterales>Enterobacteriaceae	Salmonella_enterica_subsp._enterica_serovar_Kentucky	cysteine-desulfurase-DndA-[Salmonella-enterica-subsp.-enterica-serovar-Kentucky]	192955
EBL3982660.1	cysteine-desulfurase	GCA_006685005.1	374	Bacteria>Proteobacteria>Gammaproteobacteria>Enterobacterales>Enterobacteriaceae	Salmonella_enterica_subsp._enterica_serovar_Kentucky	cysteine-desulfurase-DndA-[Salmonella-enterica-subsp.-enterica-serovar-Kentucky]	192955
EBL4319919.1	cysteine-desulfurase	GCA_006685965.1	374	Bacteria>Proteobacteria>Gammaproteobacteria>Enterobacterales>Enterobacteriaceae	Salmonella_enterica_subsp._enterica_serovar_Kentucky	cysteine-desulfurase-DndA-[Salmonella-enterica-subsp.-enterica-serovar-Kentucky]	192955
EBL4539784.1	cysteine-desulfurase	GCA_006687125.1	374	Bacteria>Proteobacteria>Gammaproteobacteria>Enterobacterales>Enterobacteriaceae	Salmonella_enterica_subsp._enterica_serovar_Kentucky	cysteine-desulfurase-DndA-[Salmonella-enterica-subsp.-enterica-serovar-Kentucky]	192955
EBL4706650.1	cysteine-desulfurase	GCA_006688005.1	374	Bacteria>Proteobacteria>Gammaproteobacteria>Enterobacterales>Enterobacteriaceae	Salmonella_enterica_subsp._enterica_serovar_Kentucky	cysteine-desulfurase-DndA-[Salmonella-enterica-subsp.-enterica-serovar-Kentucky]	192955
EBL5191435.1	cysteine-desulfurase	GCA_006689725.1	374	Bacteria>Proteobacteria>Gammaproteobacteria>Enterobacterales>Enterobacteriaceae	Salmonella_enterica_subsp._enterica_serovar_Kentucky	cysteine-desulfurase-DndA-[Salmonella-enterica-subsp.-enterica-serovar-Kentucky]	192955
EBL6089665.1	cysteine-desulfurase	GCA_006693205.1	374	Bacteria>Proteobacteria>Gammaproteobacteria>Enterobacterales>Enterobacteriaceae	Salmonella_enterica_subsp._enterica_serovar_Kentucky	cysteine-desulfurase-DndA-[Salmonella-enterica-subsp.-enterica-serovar-Kentucky]	192955
EBM7875035.1	cysteine-desulfurase	GCA_006747645.1	374	Bacteria>Proteobacteria>Gammaproteobacteria>Enterobacterales>Enterobacteriaceae	Salmonella_enterica_subsp._enterica_serovar_Kentucky	cysteine-desulfurase-DndA-[Salmonella-enterica-subsp.-enterica-serovar-Kentucky]	192955
EBM7920145.1	cysteine-desulfurase	GCA_006747585.1	374	Bacteria>Proteobacteria>Gammaproteobacteria>Enterobacterales>Enterobacteriaceae	Salmonella_enterica_subsp._enterica_serovar_Kentucky	cysteine-desulfurase-DndA-[Salmonella-enterica-subsp.-enterica-serovar-Kentucky]	192955
EBM8846245.1	cysteine-desulfurase	GCA_006751285.1	374	Bacteria>Proteobacteria>Gammaproteobacteria>Enterobacterales>Enterobacteriaceae	Salmonella_enterica_subsp._enterica_serovar_Kentucky	cysteine-desulfurase-DndA-[Salmonella-enterica-subsp.-enterica-serovar-Kentucky]	192955
EBM9705187.1	cysteine-desulfurase	GCA_006753535.1	374	Bacteria>Proteobacteria>Gammaproteobacteria>Enterobacterales>Enterobacteriaceae	Salmonella_enterica_subsp._enterica_serovar_Kentucky	cysteine-desulfurase-DndA-[Salmonella-enterica-subsp.-enterica-serovar-Kentucky]	192955
EBO1528749.1	cysteine-desulfurase	GCA_006805025.1	374	Bacteria>Proteobacteria>Gammaproteobacteria>Enterobacterales>Enterobacteriaceae	Salmonella_enterica_subsp._enterica_serovar_Kentucky	cysteine-desulfurase-DndA-[Salmonella-enterica-subsp.-enterica-serovar-Kentucky]	192955
EBO1705381.1	cysteine-desulfurase	GCA_006806125.1	374	Bacteria>Proteobacteria>Gammaproteobacteria>Enterobacterales>Enterobacteriaceae	Salmonella_enterica_subsp._enterica_serovar_Kentucky	cysteine-desulfurase-DndA-[Salmonella-enterica-subsp.-enterica-serovar-Kentucky]	192955
EBO2779960.1	cysteine-desulfurase	GCA_006811265.1	374	Bacteria>Proteobacteria>Gammaproteobacteria>Enterobacterales>Enterobacteriaceae	Salmonella_enterica_subsp._enterica_serovar_Kentucky	cysteine-desulfurase-DndA-[Salmonella-enterica-subsp.-enterica-serovar-Kentucky]	192955
EBO3420300.1	cysteine-desulfurase	GCA_006813745.1	374	Bacteria>Proteobacteria>Gammaproteobacteria>Enterobacterales>Enterobacteriaceae	Salmonella_enterica_subsp._enterica_serovar_Kentucky	cysteine-desulfurase-DndA-[Salmonella-enterica-subsp.-enterica-serovar-Kentucky]	192955
EBO8972054.1	cysteine-desulfurase	GCA_006837705.1	374	Bacteria>Proteobacteria>Gammaproteobacteria>Enterobacterales>Enterobacteriaceae	Salmonella_enterica_subsp._enterica_serovar_Kentucky	cysteine-desulfurase-DndA-[Salmonella-enterica-subsp.-enterica-serovar-Kentucky]	192955
EBO9001753.1	cysteine-desulfurase	GCA_006837745.1	374	Bacteria>Proteobacteria>Gammaproteobacteria>Enterobacterales>Enterobacteriaceae	Salmonella_enterica_subsp._enterica_serovar_Kentucky	cysteine-desulfurase-DndA-[Salmonella-enterica-subsp.-enterica-serovar-Kentucky]	192955
EBO9072695.1	cysteine-desulfurase	GCA_006838045.1	374	Bacteria>Proteobacteria>Gammaproteobacteria>Enterobacterales>Enterobacteriaceae	Salmonella_enterica_subsp._enterica_serovar_Kentucky	cysteine-desulfurase-DndA-[Salmonella-enterica-subsp.-enterica-serovar-Kentucky]	192955
EBZ0456717.1	cysteine-desulfurase	GCA_007318215.1	374	Bacteria>Proteobacteria>Gammaproteobacteria>Enterobacterales>Enterobacteriaceae	Salmonella_enterica_subsp._enterica_serovar_Kentucky	cysteine-desulfurase-DndA-[Salmonella-enterica-subsp.-enterica-serovar-Kentucky]	192955
EBZ7419228.1	cysteine-desulfurase	GCA_007348075.1	374	Bacteria>Proteobacteria>Gammaproteobacteria>Enterobacterales>Enterobacteriaceae	Salmonella_enterica_subsp._enterica_serovar_Kentucky	cysteine-desulfurase-DndA-[Salmonella-enterica-subsp.-enterica-serovar-Kentucky]	192955
EBZ7437222.1	cysteine-desulfurase	GCA_007348055.1	374	Bacteria>Proteobacteria>Gammaproteobacteria>Enterobacterales>Enterobacteriaceae	Salmonella_enterica_subsp._enterica_serovar_Kentucky	cysteine-desulfurase-DndA-[Salmonella-enterica-subsp.-enterica-serovar-Kentucky]	192955
EBZ9321688.1	cysteine-desulfurase	GCA_007357695.1	374	Bacteria>Proteobacteria>Gammaproteobacteria>Enterobacterales>Enterobacteriaceae	Salmonella_enterica_subsp._enterica_serovar_Kentucky	cysteine-desulfurase-DndA-[Salmonella-enterica-subsp.-enterica-serovar-Kentucky]	192955
ECA5926185.1	cysteine-desulfurase	GCA_007387805.1	374	Bacteria>Proteobacteria>Gammaproteobacteria>Enterobacterales>Enterobacteriaceae	Salmonella_enterica_subsp._enterica_serovar_Kentucky	cysteine-desulfurase-DndA-[Salmonella-enterica-subsp.-enterica-serovar-Kentucky]	192955
ECA7497466.1	cysteine-desulfurase	GCA_007394755.1	374	Bacteria>Proteobacteria>Gammaproteobacteria>Enterobacterales>Enterobacteriaceae	Salmonella_enterica_subsp._enterica_serovar_Kentucky	cysteine-desulfurase-DndA-[Salmonella-enterica-subsp.-enterica-serovar-Kentucky]	192955
ECA9595336.1	cysteine-desulfurase	GCA_007404345.1	374	Bacteria>Proteobacteria>Gammaproteobacteria>Enterobacterales>Enterobacteriaceae	Salmonella_enterica_subsp._enterica_serovar_Kentucky	cysteine-desulfurase-DndA-[Salmonella-enterica-subsp.-enterica-serovar-Kentucky]	192955
ECI0796832.1	cysteine-desulfurase	GCA_007756575.1	374	Bacteria>Proteobacteria>Gammaproteobacteria>Enterobacterales>Enterobacteriaceae	Salmonella_enterica_subsp._enterica_serovar_Kentucky	cysteine-desulfurase-DndA-[Salmonella-enterica-subsp.-enterica-serovar-Kentucky]	192955
ECI0895499.1	cysteine-desulfurase	GCA_007757495.1	374	Bacteria>Proteobacteria>Gammaproteobacteria>Enterobacterales>Enterobacteriaceae	Salmonella_enterica_subsp._enterica_serovar_Kentucky	cysteine-desulfurase-DndA-[Salmonella-enterica-subsp.-enterica-serovar-Kentucky]	192955
ECI8812934.1	cysteine-desulfurase	GCA_007794175.1	374	Bacteria>Proteobacteria>Gammaproteobacteria>Enterobacterales>Enterobacteriaceae	Salmonella_enterica_subsp._enterica_serovar_Kentucky	cysteine-desulfurase-DndA-[Salmonella-enterica-subsp.-enterica-serovar-Kentucky]	192955
ECO0691592.1	cysteine-desulfurase	GCA_008114405.1	374	Bacteria>Proteobacteria>Gammaproteobacteria>Enterobacterales>Enterobacteriaceae	Salmonella_enterica_subsp._enterica_serovar_Kentucky	cysteine-desulfurase-DndA-[Salmonella-enterica-subsp.-enterica-serovar-Kentucky]	192955
ECS4448022.1	cysteine-desulfurase	GCA_008415345.1	374	Bacteria>Proteobacteria>Gammaproteobacteria>Enterobacterales>Enterobacteriaceae	Salmonella_enterica_subsp._enterica_serovar_Kentucky	cysteine-desulfurase-DndA-[Salmonella-enterica-subsp.-enterica-serovar-Kentucky]	192955

Accession	Annotation	GCA	Length	Phyletic.spread	Strain.name	Defline	Species.Taxid
ECS4718038.1	cysteine-desulfurase	GCA_008424125.1	374	Bacteria>Proteobacteria>Gammaproteobacteria>Enterobacterales>Enterobacteriaceae	Salmonella_enterica_subsp._enterica_serovar_Kentucky	cysteine-desulfurase-DndA-[Salmonella-enterica-subsp.-enterica-serovar-Kentucky]	192955
ECS6778026.1	cysteine-desulfurase	GCA_008432765.1	374	Bacteria>Proteobacteria>Gammaproteobacteria>Enterobacterales>Enterobacteriaceae	Salmonella_enterica_subsp._enterica_serovar_Kentucky	cysteine-desulfurase-DndA-[Salmonella-enterica-subsp.-enterica-serovar-Kentucky]	192955
ECT0457792.1	cysteine-desulfurase	GCA_008448565.1	374	Bacteria>Proteobacteria>Gammaproteobacteria>Enterobacterales>Enterobacteriaceae	Salmonella_enterica_subsp._enterica_serovar_Kentucky	cysteine-desulfurase-DndA-[Salmonella-enterica-subsp.-enterica-serovar-Kentucky]	192955
ECT0539252.1	cysteine-desulfurase	GCA_008449315.1	374	Bacteria>Proteobacteria>Gammaproteobacteria>Enterobacterales>Enterobacteriaceae	Salmonella_enterica_subsp._enterica_serovar_Kentucky	cysteine-desulfurase-DndA-[Salmonella-enterica-subsp.-enterica-serovar-Kentucky]	192955
ECT1522145.1	cysteine-desulfurase	GCA_008453355.1	374	Bacteria>Proteobacteria>Gammaproteobacteria>Enterobacterales>Enterobacteriaceae	Salmonella_enterica_subsp._enterica_serovar_Kentucky	cysteine-desulfurase-DndA-[Salmonella-enterica-subsp.-enterica-serovar-Kentucky]	192955
ECT2680201.1	cysteine-desulfurase	GCA_008458285.1	374	Bacteria>Proteobacteria>Gammaproteobacteria>Enterobacterales>Enterobacteriaceae	Salmonella_enterica_subsp._enterica_serovar_Kentucky	cysteine-desulfurase-DndA-[Salmonella-enterica-subsp.-enterica-serovar-Kentucky]	192955
ECT6817184.1	cysteine-desulfurase	GCA_008476745.1	374	Bacteria>Proteobacteria>Gammaproteobacteria>Enterobacterales>Enterobacteriaceae	Salmonella_enterica_subsp._enterica_serovar_Kentucky	cysteine-desulfurase-DndA-[Salmonella-enterica-subsp.-enterica-serovar-Kentucky]	192955
ECU1882915.1	cysteine-desulfurase	GCA_008497645.1	374	Bacteria>Proteobacteria>Gammaproteobacteria>Enterobacterales>Enterobacteriaceae	Salmonella_enterica_subsp._enterica_serovar_Kentucky	cysteine-desulfurase-DndA-[Salmonella-enterica-subsp.-enterica-serovar-Kentucky]	192955
ECU3329802.1	cysteine-desulfurase	GCA_008505825.1	374	Bacteria>Proteobacteria>Gammaproteobacteria>Enterobacterales>Enterobacteriaceae	Salmonella_enterica_subsp._enterica_serovar_Kentucky	cysteine-desulfurase-DndA-[Salmonella-enterica-subsp.-enterica-serovar-Kentucky]	192955
ECU4501038.1	cysteine-desulfurase	GCA_008511415.1	374	Bacteria>Proteobacteria>Gammaproteobacteria>Enterobacterales>Enterobacteriaceae	Salmonella_enterica_subsp._enterica_serovar_Kentucky	cysteine-desulfurase-DndA-[Salmonella-enterica-subsp.-enterica-serovar-Kentucky]	192955
ECU5676703.1	cysteine-desulfurase	GCA_008514735.1	374	Bacteria>Proteobacteria>Gammaproteobacteria>Enterobacterales>Enterobacteriaceae	Salmonella_enterica_subsp._enterica_serovar_Kentucky	cysteine-desulfurase-DndA-[Salmonella-enterica-subsp.-enterica-serovar-Kentucky]	192955
ECU7622353.1	cysteine-desulfurase	GCA_008523905.1	374	Bacteria>Proteobacteria>Gammaproteobacteria>Enterobacterales>Enterobacteriaceae	Salmonella_enterica_subsp._enterica_serovar_Kentucky	cysteine-desulfurase-DndA-[Salmonella-enterica-subsp.-enterica-serovar-Kentucky]	192955
ECV2383148.1	cysteine-desulfurase	GCA_008543855.1	374	Bacteria>Proteobacteria>Gammaproteobacteria>Enterobacterales>Enterobacteriaceae	Salmonella_enterica_subsp._enterica_serovar_Kentucky	cysteine-desulfurase-DndA-[Salmonella-enterica-subsp.-enterica-serovar-Kentucky]	192955
ECV4632624.1	cysteine-desulfurase	GCA_008555485.1	374	Bacteria>Proteobacteria>Gammaproteobacteria>Enterobacterales>Enterobacteriaceae	Salmonella_enterica_subsp._enterica_serovar_Kentucky	cysteine-desulfurase-DndA-[Salmonella-enterica-subsp.-enterica-serovar-Kentucky]	192955
ECW6176388.1	cysteine-desulfurase	GCA_008614315.1	374	Bacteria>Proteobacteria>Gammaproteobacteria>Enterobacterales>Enterobacteriaceae	Salmonella_enterica_subsp._enterica_serovar_Kentucky	cysteine-desulfurase-DndA-[Salmonella-enterica-subsp.-enterica-serovar-Kentucky]	192955
EDC9307179.1	cysteine-desulfurase	GCA_008992225.1	374	Bacteria>Proteobacteria>Gammaproteobacteria>Enterobacterales>Enterobacteriaceae	Salmonella_enterica_subsp._enterica_serovar_Kentucky	cysteine-desulfurase-DndA-[Salmonella-enterica-subsp.-enterica-serovar-Kentucky]	192955
EDD5540009.1	cysteine-desulfurase	GCA_009100305.1	374	Bacteria>Proteobacteria>Gammaproteobacteria>Enterobacterales>Enterobacteriaceae	Salmonella_enterica_subsp._enterica_serovar_Kentucky	cysteine-desulfurase-DndA-[Salmonella-enterica-subsp.-enterica-serovar-Kentucky]	192955
EDJ2847040.1	cysteine-desulfurase	GCA_009367195.1	374	Bacteria>Proteobacteria>Gammaproteobacteria>Enterobacterales>Enterobacteriaceae	Salmonella_enterica_subsp._enterica_serovar_Kentucky	cysteine-desulfurase-DndA-[Salmonella-enterica-subsp.-enterica-serovar-Kentucky]	192955
EDJ4199857.1	cysteine-desulfurase	GCA_009373595.1	374	Bacteria>Proteobacteria>Gammaproteobacteria>Enterobacterales>Enterobacteriaceae	Salmonella_enterica_subsp._enterica_serovar_Kentucky	cysteine-desulfurase-DndA-[Salmonella-enterica-subsp.-enterica-serovar-Kentucky]	192955
EDK7900861.1	cysteine-desulfurase	GCA_009477465.1	374	Bacteria>Proteobacteria>Gammaproteobacteria>Enterobacterales>Enterobacteriaceae	Salmonella_enterica_subsp._enterica_serovar_Kentucky	cysteine-desulfurase-DndA-[Salmonella-enterica-subsp.-enterica-serovar-Kentucky]	192955
EDL5352247.1	cysteine-desulfurase	GCA_009524095.1	374	Bacteria>Proteobacteria>Gammaproteobacteria>Enterobacterales>Enterobacteriaceae	Salmonella_enterica_subsp._enterica_serovar_Kentucky	cysteine-desulfurase-DndA-[Salmonella-enterica-subsp.-enterica-serovar-Kentucky]	192955
EDL6518946.1	cysteine-desulfurase	GCA_009528275.1	374	Bacteria>Proteobacteria>Gammaproteobacteria>Enterobacterales>Enterobacteriaceae	Salmonella_enterica_subsp._enterica_serovar_Kentucky	cysteine-desulfurase-DndA-[Salmonella-enterica-subsp.-enterica-serovar-Kentucky]	192955
EDL7627644.1	cysteine-desulfurase	GCA_009533715.1	374	Bacteria>Proteobacteria>Gammaproteobacteria>Enterobacterales>Enterobacteriaceae	Salmonella_enterica_subsp._enterica_serovar_Kentucky	cysteine-desulfurase-DndA-[Salmonella-enterica-subsp.-enterica-serovar-Kentucky]	192955
EDM1461319.1	cysteine-desulfurase	GCA_009550715.1	374	Bacteria>Proteobacteria>Gammaproteobacteria>Enterobacterales>Enterobacteriaceae	Salmonella_enterica_subsp._enterica_serovar_Kentucky	cysteine-desulfurase-DndA-[Salmonella-enterica-subsp.-enterica-serovar-Kentucky]	192955
EDM2107998.1	cysteine-desulfurase	GCA_009553715.1	374	Bacteria>Proteobacteria>Gammaproteobacteria>Enterobacterales>Enterobacteriaceae	Salmonella_enterica_subsp._enterica_serovar_Kentucky	cysteine-desulfurase-DndA-[Salmonella-enterica-subsp.-enterica-serovar-Kentucky]	192955
EDM9524857.1	cysteine-desulfurase	GCA_009584645.1	374	Bacteria>Proteobacteria>Gammaproteobacteria>Enterobacterales>Enterobacteriaceae	Salmonella_enterica_subsp._enterica_serovar_Kentucky	cysteine-desulfurase-DndA-[Salmonella-enterica-subsp.-enterica-serovar-Kentucky]	192955
EEA3561466.1	cysteine-desulfurase	GCA_010891675.1	374	Bacteria>Proteobacteria>Gammaproteobacteria>Enterobacterales>Enterobacteriaceae	Salmonella_enterica_subsp._enterica_serovar_Kentucky	cysteine-desulfurase-DndA-[Salmonella-enterica-subsp.-enterica-serovar-Kentucky]	192955
EEE4429080.1	cysteine-desulfurase	GCA_011133135.1	374	Bacteria>Proteobacteria>Gammaproteobacteria>Enterobacterales>Enterobacteriaceae	Salmonella_enterica_subsp._enterica_serovar_Kentucky	cysteine-desulfurase-DndA-[Salmonella-enterica-subsp.-enterica-serovar-Kentucky]	192955
EEH9185131.1	cysteine-desulfurase	GCA_011311895.1	374	Bacteria>Proteobacteria>Gammaproteobacteria>Enterobacterales>Enterobacteriaceae	Salmonella_enterica_subsp._enterica_serovar_Kentucky	cysteine-desulfurase-DndA-[Salmonella-enterica-subsp.-enterica-serovar-Kentucky]	192955
EEK0756063.1	cysteine-desulfurase	GCA_011453575.1	374	Bacteria>Proteobacteria>Gammaproteobacteria>Enterobacterales>Enterobacteriaceae	Salmonella_enterica_subsp._enterica_serovar_Kentucky	cysteine-desulfurase-DndA-[Salmonella-enterica-subsp.-enterica-serovar-Kentucky]	192955
EEL9418968.1	cysteine-desulfurase	GCA_011545785.1	374	Bacteria>Proteobacteria>Gammaproteobacteria>Enterobacterales>Enterobacteriaceae	Salmonella_enterica_subsp._enterica_serovar_Kentucky	cysteine-desulfurase-DndA-[Salmonella-enterica-subsp.-enterica-serovar-Kentucky]	192955
EEM4706724.1	cysteine-desulfurase	GCA_011569545.1	374	Bacteria>Proteobacteria>Gammaproteobacteria>Enterobacterales>Enterobacteriaceae	Salmonella_enterica_subsp._enterica_serovar_Kentucky	cysteine-desulfurase-DndA-[Salmonella-enterica-subsp.-enterica-serovar-Kentucky]	192955
EEM7223696.1	cysteine-desulfurase	GCA_011580205.1	374	Bacteria>Proteobacteria>Gammaproteobacteria>Enterobacterales>Enterobacteriaceae	Salmonella_enterica_subsp._enterica_serovar_Kentucky	cysteine-desulfurase-DndA-[Salmonella-enterica-subsp.-enterica-serovar-Kentucky]	192955
EFQ4540185.1	cysteine-desulfurase	GCA_013499795.1	374	Bacteria>Proteobacteria>Gammaproteobacteria>Enterobacterales>Enterobacteriaceae	Salmonella_enterica_subsp._enterica_serovar_Kentucky	cysteine-desulfurase-DndA-[Salmonella-enterica-subsp.-enterica-serovar-Kentucky]	192955
EFS2494163.1	cysteine-desulfurase	GCA_013628195.1	374	Bacteria>Proteobacteria>Gammaproteobacteria>Enterobacterales>Enterobacteriaceae	Salmonella_enterica_subsp._enterica_serovar_Kentucky	cysteine-desulfurase-DndA-[Salmonella-enterica-subsp.-enterica-serovar-Kentucky]	192955
EFS5422975.1	cysteine-desulfurase	GCA_013653695.1	374	Bacteria>Proteobacteria>Gammaproteobacteria>Enterobacterales>Enterobacteriaceae	Salmonella_enterica_subsp._enterica_serovar_Kentucky	cysteine-desulfurase-DndA-[Salmonella-enterica-subsp.-enterica-serovar-Kentucky]	192955
EFU1279002.1	cysteine-desulfurase	GCA_013735905.1	374	Bacteria>Proteobacteria>Gammaproteobacteria>Enterobacterales>Enterobacteriaceae	Salmonella_enterica_subsp._enterica_serovar_Kentucky	cysteine-desulfurase-DndA-[Salmonella-enterica-subsp.-enterica-serovar-Kentucky]	192955
EFU1700391.1	cysteine-desulfurase	GCA_013736855.1	374	Bacteria>Proteobacteria>Gammaproteobacteria>Enterobacterales>Enterobacteriaceae	Salmonella_enterica_subsp._enterica_serovar_Kentucky	cysteine-desulfurase-DndA-[Salmonella-enterica-subsp.-enterica-serovar-Kentucky]	192955
EGD3562019.1	cysteine-desulfurase	GCA_014368225.1	374	Bacteria>Proteobacteria>Gammaproteobacteria>Enterobacterales>Enterobacteriaceae	Salmonella_enterica_subsp._enterica_serovar_Kentucky	cysteine-desulfurase-DndA-[Salmonella-enterica-subsp.-enterica-serovar-Kentucky]	192955
EGI4860826.1	cysteine-desulfurase	GCA_014751225.1	374	Bacteria>Proteobacteria>Gammaproteobacteria>Enterobacterales>Enterobacteriaceae	Salmonella_enterica_subsp._enterica_serovar_Kentucky	cysteine-desulfurase-DndA-[Salmonella-enterica-subsp.-enterica-serovar-Kentucky]	192955
EGT6264013.1	cysteine-desulfurase	GCA_016117875.1	374	Bacteria>Proteobacteria>Gammaproteobacteria>Enterobacterales>Enterobacteriaceae	Salmonella_enterica_subsp._enterica_serovar_Kentucky	cysteine-desulfurase-DndA-[Salmonella-enterica-subsp.-enterica-serovar-Kentucky]	192955
EHF9078714.1	cysteine-desulfurase	GCA_017359925.1	374	Bacteria>Proteobacteria>Gammaproteobacteria>Enterobacterales>Enterobacteriaceae	Salmonella_enterica_subsp._enterica_serovar_Kentucky	cysteine-desulfurase-DndA-[Salmonella-enterica-subsp.-enterica-serovar-Kentucky]	192955
EIJ5959354.1	cysteine-desulfurase	GCA_020547245.1	374	Bacteria>Proteobacteria>Gammaproteobacteria>Enterobacterales>Enterobacteriaceae	Salmonella_enterica_subsp._enterica_serovar_Kentucky	cysteine-desulfurase-DndA-[Salmonella-enterica-subsp.-enterica-serovar-Kentucky]	192955
EEE2001769.1	cysteine-desulfurase	GCA_011120725.1	374	Bacteria>Proteobacteria>Gammaproteobacteria>Enterobacterales>Enterobacteriaceae	Salmonella_enterica_subsp._enterica_serovar_Kotte	cysteine-desulfurase-DndA-[Salmonella-enterica-subsp.-enterica-serovar-Kotte]	2048769
ECA5259055.1	cysteine-desulfurase	GCA_007385425.1	374	Bacteria>Proteobacteria>Gammaproteobacteria>Enterobacterales>Enterobacteriaceae	Salmonella_enterica_subsp._enterica_serovar_Newport	cysteine-desulfurase-DndA-[Salmonella-enterica-subsp.-enterica-serovar-Newport]	108619
ECN7187349.1	cysteine-desulfurase	GCA_008098265.1	374	Bacteria>Proteobacteria>Gammaproteobacteria>Enterobacterales>Enterobacteriaceae	Salmonella_enterica_subsp._enterica_serovar_Newport	cysteine-desulfurase-DndA-[Salmonella-enterica-subsp.-enterica-serovar-Newport]	108619
EEB8831404.1	cysteine-desulfurase	GCA_010960465.1	374	Bacteria>Proteobacteria>Gammaproteobacteria>Enterobacterales>Enterobacteriaceae	Salmonella_enterica_subsp._enterica_serovar_Newport	cysteine-desulfurase-DndA-[Salmonella-enterica-subsp.-enterica-serovar-Newport]	108619
HAU6779802.1	cysteine-desulfurase	GCA_016095455.1	374	Bacteria>Proteobacteria>Gammaproteobacteria>Enterobacterales>Enterobacteriaceae	Salmonella_enterica_subsp._enterica_serovar_Tado	cysteine-desulfurase-DndA-[Salmonella-enterica-subsp.-enterica-serovar-Tado]	2565074
HBC0145587.1	cysteine-desulfurase	GCA_018033065.1	374	Bacteria>Proteobacteria>Gammaproteobacteria>Enterobacterales>Enterobacteriaceae	Salmonella_enterica_subsp._houtenae	TPA:-cysteine-desulfurase-DndA-[Salmonella-enterica-subsp.-houtenae]	59205
ECC9154006.1	cysteine-desulfurase	GCA_007500425.1	374	Bacteria>Proteobacteria>Gammaproteobacteria>Enterobacterales>Enterobacteriaceae	Salmonella_enterica_subsp._salamae	cysteine-desulfurase-DndA-[Salmonella-enterica-subsp.-salamae]	59202
ECF6031765.1	cysteine-desulfurase	GCA_007620915.1	374	Bacteria>Proteobacteria>Gammaproteobacteria>Enterobacterales>Enterobacteriaceae	Salmonella_enterica_subsp._salamae	cysteine-desulfurase-DndA-[Salmonella-enterica-subsp.-salamae]	59202
WP_219826151.1	cysteine-desulfurase	-	355	Bacteria>Proteobacteria>Gammaproteobacteria>Enterobacterales>Enterobacteriaceae	Scandinavium_goeteborgense	aminotransferase-class-V-fold-PLP-dependent-enzyme-[Scandinavium-goeteborgense]	1851514
WP_054178967.1	cysteine-desulfurase	GCF_001297845.1	374	Bacteria>Proteobacteria>Gammaproteobacteria>Enterobacterales>Enterobacteriaceae	Trabulsiella_odontotermitis	cysteine-desulfurase-DndA-[Trabulsiella-odontotermitis]	379893
HAE78645.1	cysteine-desulfurase	GCA_003446855.1	374	Bacteria>Proteobacteria>Gammaproteobacteria>Enterobacterales>Morganellaceae	Morganella_sp._(in:_Bacteria)	cysteine-desulfurase-DndA-[Morganella-sp.-(in:-Bacteria)]	1912315
WP_116162583.1	cysteine-desulfurase	GCF_003382565.3	374	Bacteria>Proteobacteria>Gammaproteobacteria>Enterobacterales>Pectobacteriaceae	Pectobacterium_aquaticum	cysteine-desulfurase-DndA-[Pectobacterium-aquaticum]	2204145
WP_161502358.1	cysteine-desulfurase	GCF_009931195.1	374	Bacteria>Proteobacteria>Gammaproteobacteria>Enterobacterales>Pectobacteriaceae	Pectobacterium_carotovorum	cysteine-desulfurase-DndA-[Pectobacterium-carotovorum]	554
WP_161510033.1	cysteine-desulfurase	GCF_009931215.1	353	Bacteria>Proteobacteria>Gammaproteobacteria>Enterobacterales>Pectobacteriaceae	Pectobacterium_carotovorum	cysteine-desulfurase-[Pectobacterium-carotovorum]	554
QHP53816.1	cysteine-desulfurase	GCA_009931195.1	374	Bacteria>Proteobacteria>Gammaproteobacteria>Enterobacterales>Pectobacteriaceae	Pectobacterium_carotovorum_subsp._carotovorum	cysteine-desulfurase-DndA-[Pectobacterium-carotovorum-subsp.-carotovorum]	555
WP_193399476.1	cysteine-desulfurase	GCF_014946775.2	374	Bacteria>Proteobacteria>Gammaproteobacteria>Enterobacterales>Pectobacteriaceae	Pectobacterium_quasiaquaticum	cysteine-desulfurase-DndA-[Pectobacterium-quasiaquaticum]	2774015
WP_134706884.1	cysteine-desulfurase	GCF_004522185.1	374	Bacteria>Proteobacteria>Gammaproteobacteria>Enterobacterales>Yersiniaceae	Rahnella_sp._CJA17(1/100)	cysteine-desulfurase-DndA-[Rahnella-sp.-CJA17(1/100)]	2508951
TVR64216.1	cysteine-desulfurase	GCA_007695245.1	377	Bacteria>Proteobacteria>Gammaproteobacteria>Gammaproteobacteria_incertae_sedis>Candidatus_Competibacteraceae	Candidatus_Competibacteraceae_bacterium	cysteine-desulfurase-DndA-[Candidatus-Competibacteraceae-bacterium]	2053538
WP_109218019.1	cysteine-desulfurase	GCF_003121895.1	384	Bacteria>Proteobacteria>Gammaproteobacteria>Gammaproteobacteria_incertae_sedis>Ignatzschineria	Ignatzschineria_cameli	cysteine-desulfurase-[Ignatzschineria-cameli]	2182793
WP_213404188.1	cysteine-desulfurase	GCF_018415905.1	378	Bacteria>Proteobacteria>Gammaproteobacteria>Gammaproteobacteria_incertae_sedis>Wohlfahrtiimonas	Wohlfahrtiimonas_chitiniclastica	aminotransferase-class-V-fold-PLP-dependent-enzyme-[Wohlfahrtiimonas-chitiniclastica]	400946
WP_094491702.1	cysteine-desulfurase	-	378	Bacteria>Proteobacteria>Gammaproteobacteria>Gammaproteobacteria_incertae_sedis>Wohlfahrtiimonas	Wohlfahrtiimonas_chitiniclastica	cysteine-desulfurase-[Wohlfahrtiimonas-chitiniclastica]	400946
WP_094538221.1	cysteine-desulfurase	GCF_002252325.1	378	Bacteria>Proteobacteria>Gammaproteobacteria>Gammaproteobacteria_incertae_sedis>Wohlfahrtiimonas	Wohlfahrtiimonas_chitiniclastica	cysteine-desulfurase-[Wohlfahrtiimonas-chitiniclastica]	400946
OYQ69874.1	cysteine-desulfurase	GCA_002252145.1	378	Bacteria>Proteobacteria>Gammaproteobacteria>Gammaproteobacteria_incertae_sedis>Wohlfahrtiimonas	Wohlfahrtiimonas_chitiniclastica	hypothetical-protein-B9T13_06330-[Wohlfahrtiimonas-chitiniclastica]	400946
MVF23445.1	cysteine-desulfurase	GCA_009749305.1	377	Bacteria>Proteobacteria>Gammaproteobacteria>Methylococcales>Methylococcaceae	Methylocaldum_sp._BRCS4	cysteine-desulfurase-DndA-[Methylocaldum-sp.-BRCS4]	2510798
OAI06191.1	cysteine-desulfurase	GCA_001644035.1	374	Bacteria>Proteobacteria>Gammaproteobacteria>Methylococcales>Methylococcaceae	Methylomonas_methanica	cysteine-desulfurase-DndA-[Methylomonas-methanica]	421
WP_064008150.1	cysteine-desulfurase	GCF_001644035.1	374	Bacteria>Proteobacteria>Gammaproteobacteria>Methylococcales>Methylococcaceae	Methylomonas_methanica	cysteine-desulfurase-DndA-[Methylomonas-methanica]	421
MBE0471004.1	cysteine-desulfurase	GCA_014859465.1	374	Bacteria>Proteobacteria>Gammaproteobacteria>Methylococcales>Methylococcaceae	Methyloprofundus_sp.	cysteine-desulfurase-DndA-[Methyloprofundus-sp.]	2020875
MBE0470968.1	cysteine-desulfurase	GCA_014859465.1	374	Bacteria>Proteobacteria>Gammaproteobacteria>Methylococcales>Methylococcaceae	Methyloprofundus_sp.	cysteine-desulfurase-[Methyloprofundus-sp.]	2020875
WP_139559696.1	cysteine-desulfurase	GCF_006175985.1	377	Bacteria>Proteobacteria>Gammaproteobacteria>Methylococcales>Methylococcaceae	Methylotetracoccus_oryzae	cysteine-desulfurase-DndA-[Methylotetracoccus-oryzae]	1919059
WP_004956607.1	cysteine-desulfurase	GCF_000368745.1	371	Bacteria>Proteobacteria>Gammaproteobacteria>Moraxellales>Moraxellaceae	Acinetobacter_junii	cysteine-desulfurase-[Acinetobacter-junii]	40215
WP_166327585.1	cysteine-desulfurase	GCF_011578285.1	366	Bacteria>Proteobacteria>Gammaproteobacteria>Moraxellales>Moraxellaceae	Acinetobacter_lanii	cysteine-desulfurase-[Acinetobacter-lanii]	2715163
WP_154320225.1	cysteine-desulfurase	GCF_013378115.1	371	Bacteria>Proteobacteria>Gammaproteobacteria>Moraxellales>Moraxellaceae	Acinetobacter	MULTISPECIES:-cysteine-desulfurase-[Acinetobacter]	469
WP_212515132.1	cysteine-desulfurase	GCF_018139125.1	371	Bacteria>Proteobacteria>Gammaproteobacteria>Moraxellales>Moraxellaceae	Acinetobacter_soli	aminotransferase-class-V-fold-PLP-dependent-enzyme-[Acinetobacter-soli]	487316
MBY3618577.1	cysteine-desulfurase	GCA_019779985.1	377	Bacteria>Proteobacteria>Gammaproteobacteria>Moraxellales>Moraxellaceae	Acinetobacter_sp._CUI_P1	cysteine-desulfurase-[Acinetobacter-sp.-CUI-P1]	2726727
OTG78935.1	cysteine-desulfurase	GCA_002135195.1	369	Bacteria>Proteobacteria>Gammaproteobacteria>Moraxellales>Moraxellaceae	Acinetobacter_terrae	aminotransferase-[Acinetobacter-terrae]	2731247
WP_086192834.1	cysteine-desulfurase	GCF_002135195.1	369	Bacteria>Proteobacteria>Gammaproteobacteria>Moraxellales>Moraxellaceae	Acinetobacter_terrae	cysteine-desulfurase-[Acinetobacter-terrae]	2731247
WP_067059132.1	cysteine-desulfurase	GCF_001591265.1	394	Bacteria>Proteobacteria>Gammaproteobacteria>Moraxellales>Moraxellaceae	Moraxella_atlantae	cysteine-desulfurase-[Moraxella-atlantae]	34059
AKG17896.1	cysteine-desulfurase	GCA_000988745.3	375	Bacteria>Proteobacteria>Gammaproteobacteria>Moraxellales>Moraxellaceae	Moraxella_bovoculi	alanine–glyoxylate-aminotransferase-family-protein-[Moraxella-bovoculi]	386891
AKG16503.1	cysteine-desulfurase	GCA_000988745.3	388	Bacteria>Proteobacteria>Gammaproteobacteria>Moraxellales>Moraxellaceae	Moraxella_bovoculi	cysteine-desulfurase-[Moraxella-bovoculi]	386891
WP_046699611.1	cysteine-desulfurase	GCF_000988745.2	388	Bacteria>Proteobacteria>Gammaproteobacteria>Moraxellales>Moraxellaceae	Moraxella_bovoculi	cysteine-desulfurase-[Moraxella-bovoculi]	386891
WP_183618534.1	cysteine-desulfurase	GCF_904846335.1	383	Bacteria>Proteobacteria>Gammaproteobacteria>Moraxellales>Moraxellaceae	Psychrobacter_luti	cysteine-desulfurase-[Psychrobacter-luti]	198481
WP_210540727.1	cysteine-desulfurase	GCF_017942195.1	382	Bacteria>Proteobacteria>Gammaproteobacteria>Moraxellales>Moraxellaceae	Psychrobacter_sp._K31L	aminotransferase-class-V-fold-PLP-dependent-enzyme-[Psychrobacter-sp.-K31L]	2820758
WP_227511194.1	cysteine-desulfurase	GCF_001435845.1	385	Bacteria>Proteobacteria>Gammaproteobacteria>Moraxellales>Moraxellaceae	Psychrobacter_sp._P11G3	cysteine-desulfurase-[Psychrobacter-sp.-P11G3]	1699623
WP_201603089.1	cysteine-desulfurase	GCF_904846685.1	384	Bacteria>Proteobacteria>Gammaproteobacteria>Moraxellales>Moraxellaceae	Psychrobacter_submarinus	cysteine-desulfurase-[Psychrobacter-submarinus]	154108
MBI56345.1	cysteine-desulfurase	GCA_002719735.1	365	Bacteria>Proteobacteria>Gammaproteobacteria>Oceanospirillales>Alcanivoracaceae	Alcanivorax_sp.	aminotransferase-[Alcanivorax-sp.]	1872427
MBE8233346.1	cysteine-desulfurase	GCA_015163765.1	374	Bacteria>Proteobacteria>Gammaproteobacteria>Oceanospirillales>Endozoicomonadaceae	Endozoicomonadaceae_bacterium	cysteine-desulfurase-DndA-[Endozoicomonadaceae-bacterium]	2590577
WP_062270971.1	cysteine-desulfurase	GCF_001562015.1	375	Bacteria>Proteobacteria>Gammaproteobacteria>Oceanospirillales>Endozoicomonadaceae	Endozoicomonas_arenosclerae	cysteine-desulfurase-DndA-[Endozoicomonas-arenosclerae]	1633495
WP_034834765.1	cysteine-desulfurase	GCF_000722635.1	375	Bacteria>Proteobacteria>Gammaproteobacteria>Oceanospirillales>Endozoicomonadaceae	Endozoicomonas_numazuensis	cysteine-desulfurase-DndA-[Endozoicomonas-numazuensis]	1137799
WP_205743478.1	cysteine-desulfurase	GCF_009902005.1	400	Bacteria>Proteobacteria>Gammaproteobacteria>Oceanospirillales>Halomonadaceae	Halomonas_alimentaria	cysteine-desulfurase-[Halomonas-alimentaria]	147248
WP_172515309.1	cysteine-desulfurase	GCF_011764625.1	382	Bacteria>Proteobacteria>Gammaproteobacteria>Oceanospirillales>Halomonadaceae	Halomonas_meridiana	cysteine-desulfurase-[Halomonas-meridiana]	29570
KPQ26499.1	cysteine-desulfurase	GCA_001314745.1	376	Bacteria>Proteobacteria>Gammaproteobacteria>Oceanospirillales>Halomonadaceae	Halomonas_sp._HL-93	DNA-sulfur-modification-system-cysteine-sulfurtransferase-DndA-[Halomonas-sp.-HL-93]	1666906
WP_205210248.1	cysteine-desulfurase	GCF_016924145.1	375	Bacteria>Proteobacteria>Gammaproteobacteria>Oceanospirillales>Oceanospirillaceae	Amphritea_pacifica	cysteine-desulfurase-DndA-[Amphritea-sp.-ZJ14W]	2811233
WP_115467356.1	cysteine-desulfurase	GCF_003362755.1	375	Bacteria>Proteobacteria>Gammaproteobacteria>Oceanospirillales>Oceanospirillaceae	Marinomonas_piezotolerans	cysteine-desulfurase-DndA-[Marinomonas-piezotolerans]	2213058
WP_027850872.1	cysteine-desulfurase	GCF_000686905.1	373	Bacteria>Proteobacteria>Gammaproteobacteria>Oceanospirillales>Oceanospirillaceae	Marinospirillum_insulare	cysteine-desulfurase-DndA-[Marinospirillum-insulare]	217169
MBE0506981.1	cysteine-desulfurase	GCA_014859565.1	375	Bacteria>Proteobacteria>Gammaproteobacteria>Oceanospirillales>Oceanospirillaceae	Marinospirillum_sp.	cysteine-desulfurase-DndA-[Marinospirillum-sp.]	2183934
WP_210397388.1	cysteine-desulfurase	GCF_017912535.1	375	Bacteria>Proteobacteria>Gammaproteobacteria>Oceanospirillales>Oceanospirillaceae	Motiliproteus_sediminis	cysteine-desulfurase-DndA-[Motiliproteus-sediminis]	1468178
WP_151704768.1	cysteine-desulfurase	GCF_008973755.1	372	Bacteria>Proteobacteria>Gammaproteobacteria>Oceanospirillales>Oceanospirillaceae	Nitrincola_alkalilacustris	cysteine-desulfurase-[Nitrincola-alkalilacustris]	1571224
WP_036510252.1	cysteine-desulfurase	GCF_000585235.1	371	Bacteria>Proteobacteria>Gammaproteobacteria>Oceanospirillales>Oceanospirillaceae	Nitrincola_nitratireducens	cysteine-desulfurase-[Nitrincola-nitratireducens]	1229521
NRA14938.1	cysteine-desulfurase	GCA_013215505.1	373	Bacteria>Proteobacteria>Gammaproteobacteria>Oceanospirillales>Oceanospirillaceae	Oceanospirillaceae_bacterium	cysteine-desulfurase-[Oceanospirillaceae-bacterium]	1899355
CCK76816.1	cysteine-desulfurase	GCA_000967895.1	375	Bacteria>Proteobacteria>Gammaproteobacteria>Oceanospirillales>Oceanospirillaceae	Oleispira_antarctica_RB-8	Cysteine-desulfurase-[Oleispira-antarctica-RB-8]	698738
TNC82871.1	cysteine-desulfurase	GCA_006212045.1	373	Bacteria>Proteobacteria>Gammaproteobacteria>Oceanospirillales>Oleiphilaceae	Oleiphilus_sp.	cysteine-desulfurase-[Oleiphilus-sp.]	2025907
WP_226610387.1	cysteine-desulfurase	GCF_020524675.1	385	Bacteria>Proteobacteria>Gammaproteobacteria>Pseudomonadales>Marinobacteraceae	Marinobacter_nauticus	unnamed-protein-product]	2743
KXS55536.1	cysteine-desulfurase	GCA_001577575.1	385	Bacteria>Proteobacteria>Gammaproteobacteria>Pseudomonadales>Marinobacteraceae	Marinobacter_sp._T13-3	cysteine-desulfurase-[Marinobacter-sp.-T13-3]	1794906
WP_132710052.1	cysteine-desulfurase	GCF_018341805.1	435	Bacteria>Proteobacteria>Gammaproteobacteria>Pseudomonadales>Pseudomonadaceae	Pseudomonas_aeruginosa	cysteine-desulfurase-[Pseudomonas-aeruginosa]	287
WP_170954012.1	cysteine-desulfurase	GCF_002329865.1	382	Bacteria>Proteobacteria>Gammaproteobacteria>Pseudomonadales>Pseudomonadaceae	Pseudomonas_aeruginosa	cysteine-desulfurase-[Pseudomonas-aeruginosa]	287
WP_174517737.1	cysteine-desulfurase	GCF_902633025.1	382	Bacteria>Proteobacteria>Gammaproteobacteria>Pseudomonadales>Pseudomonadaceae	Pseudomonas_aeruginosa	cysteine-desulfurase-[Pseudomonas-aeruginosa]	287
WP_122320562.1	cysteine-desulfurase	GCF_019719075.1	388	Bacteria>Proteobacteria>Gammaproteobacteria>Pseudomonadales>Pseudomonadaceae	Pseudomonas_cichorii	cysteine-desulfurase-[Pseudomonas-cichorii]	36746
WP_231499298.1	cysteine-desulfurase	GCF_000732445.1	358	Bacteria>Proteobacteria>Gammaproteobacteria>Pseudomonadales>Pseudomonadaceae	Pseudomonas_oleovorans	cysteine-desulfurase-[Pseudomonas-oleovorans]	301
WP_081585368.1	cysteine-desulfurase	GCF_000313235.1	386	Bacteria>Proteobacteria>Gammaproteobacteria>Pseudomonadales>Pseudomonadaceae	Pseudomonas_sp._PAMC_26793	cysteine-desulfurase-[Pseudomonas-sp.-PAMC-26793]	1240676
WP_084296679.1	cysteine-desulfurase	GCF_900176445.1	402	Bacteria>Proteobacteria>Gammaproteobacteria>Pseudomonadales>Pseudomonadaceae	Pseudomonas_sp._URIL14HWK12:I5	cysteine-desulfurase-[Pseudomonas-sp.-URIL14HWK12:I5]	1261630
WP_027614297.1	cysteine-desulfurase	GCF_000514195.1	373	Bacteria>Proteobacteria>Gammaproteobacteria>Pseudomonadales>Pseudomonadaceae	unclassified_Pseudomonas	MULTISPECIES:-cysteine-desulfurase-[unclassified-Pseudomonas]	196821
PHS71160.1	cysteine-desulfurase	GCA_002733135.1	374	Bacteria>Proteobacteria>Gammaproteobacteria>Thiotrichales>Piscirickettsiaceae	Cycloclasticus_sp.	cysteine-desulfurase-DndA-[Cycloclasticus-sp.]	2024830
KHD08616.2	cysteine-desulfurase	GCA_000785145.2	375	Bacteria>Proteobacteria>Gammaproteobacteria>Thiotrichales>Thiotrichaceae	Candidatus_Thiomargarita_nelsonii	cysteine-desulfurase-[Candidatus-Thiomargarita-nelsonii]	1003181
MBR9897583.1	cysteine-desulfurase	GCA_018223225.1	97	Bacteria>Proteobacteria>Gammaproteobacteria>unclassified_Gammaproteobacteria	Gammaproteobacteria_bacterium	aminotransferase-class-V-fold-PLP-dependent-enzyme-[Gammaproteobacteria-bacterium]	1913989
MBM4229710.1	cysteine-desulfurase	GCA_016874565.1	379	Bacteria>Proteobacteria>Gammaproteobacteria>unclassified_Gammaproteobacteria	Gammaproteobacteria_bacterium	cysteine-desulfurase-DndA-[Gammaproteobacteria-bacterium]	1913989
RKZ53109.1	cysteine-desulfurase	GCA_003645185.1	377	Bacteria>Proteobacteria>Gammaproteobacteria>unclassified_Gammaproteobacteria	Gammaproteobacteria_bacterium	cysteine-desulfurase-DndA-[Gammaproteobacteria-bacterium]	1913989
RKZ57993.1	cysteine-desulfurase	GCA_003645245.1	377	Bacteria>Proteobacteria>Gammaproteobacteria>unclassified_Gammaproteobacteria	Gammaproteobacteria_bacterium	cysteine-desulfurase-DndA-[Gammaproteobacteria-bacterium]	1913989
HHJ14344.1	cysteine-desulfurase	GCA_011322085.1	396	Bacteria>Proteobacteria>Gammaproteobacteria>unclassified_Gammaproteobacteria	Gammaproteobacteria_bacterium	cysteine-desulfurase-[Gammaproteobacteria-bacterium]	1913989
MXW45046.1	cysteine-desulfurase	GCA_009836205.1	395	Bacteria>Proteobacteria>Gammaproteobacteria>unclassified_Gammaproteobacteria	Gammaproteobacteria_bacterium	cysteine-desulfurase-[Gammaproteobacteria-bacterium]	1913989
MYA67756.1	cysteine-desulfurase	GCA_009839005.1	390	Bacteria>Proteobacteria>Gammaproteobacteria>unclassified_Gammaproteobacteria	Gammaproteobacteria_bacterium	cysteine-desulfurase-[Gammaproteobacteria-bacterium]	1913989
MBU2571051.1	cysteine-desulfurase	GCA_018830325.1	134	Bacteria>Proteobacteria>Gammaproteobacteria>unclassified_Gammaproteobacteria	Gammaproteobacteria_bacterium	SufE-family-protein-[Gammaproteobacteria-bacterium]	1913989
EED31295.1	cysteine-desulfurase	GCA_000158155.1	392	Bacteria>Proteobacteria>Gammaproteobacteria>unclassified_Gammaproteobacteria	gamma_proteobacterium_NOR5-3	cysteine-desulfurase-DndA-[gamma-proteobacterium-NOR5-3]	566466

Accession	Annotation	GCA	Length	Phyletic.spread	Strain.name	Defline	Species.Taxid
WP_174482017.1	cysteine-desulfurase	GCF_900128525.1	387	Bacteria>Proteobacteria>Gammaproteobacteria>unclassified_Gammaproteobacteria	methanotrophic_endosymbiont_of_Bathymodiolus_puteoserpentis_(Logatchev)	aminotransferase-class-V-fold-PLP-dependent-enzyme-[methanotrophic-endosymbiont-of-Bathymodiolus-puteoserpentis-(Logatchev)]	343235
WP_233651132.1	cysteine-desulfurase	GCF_021366275.1	351	Bacteria>Proteobacteria>Gammaproteobacteria>Vibrionales>Vibrionaceae	Aliivibrio_fischeri	aminotransferase-class-V-fold-PLP-dependent-enzyme-[Aliivibrio-fischeri]	668
WP_233940345.1	cysteine-desulfurase	GCF_021394675.1	351	Bacteria>Proteobacteria>Gammaproteobacteria>Vibrionales>Vibrionaceae	Aliivibrio_fischeri	aminotransferase-class-V-fold-PLP-dependent-enzyme-[Aliivibrio-fischeri]	668
MUK28679.1	cysteine-desulfurase	GCA_009727725.1	374	Bacteria>Proteobacteria>Gammaproteobacteria>Vibrionales>Vibrionaceae	Aliivibrio_fischeri	cysteine-desulfurase-DndA-[Aliivibrio-fischeri]	668
WP_155651904.1	cysteine-desulfurase	GCF_009727725.1	360	Bacteria>Proteobacteria>Gammaproteobacteria>Vibrionales>Vibrionaceae	Aliivibrio_fischeri	cysteine-desulfurase-DndA-[Aliivibrio-fischeri]	668
WP_155659760.1	cysteine-desulfurase	GCF_009728045.1	374	Bacteria>Proteobacteria>Gammaproteobacteria>Vibrionales>Vibrionaceae	Aliivibrio_fischeri	cysteine-desulfurase-DndA-[Aliivibrio-fischeri]	668
WP_065600747.1	cysteine-desulfurase	GCF_001691065.1	374	Bacteria>Proteobacteria>Gammaproteobacteria>Vibrionales>Vibrionaceae	Aliivibrio_sp._1S128	cysteine-desulfurase-DndA-[Aliivibrio-sp.-1S128]	1840085
WP_074927716.1	cysteine-desulfurase	GCF_900115495.1	375	Bacteria>Proteobacteria>Gammaproteobacteria>Vibrionales>Vibrionaceae	Enterovibrio_norvegicus	cysteine-desulfurase-DndA-[Enterovibrio-norvegicus]	188144
KJG30977.1	cysteine-desulfurase	GCA_000950215.1	351	Bacteria>Proteobacteria>Gammaproteobacteria>Vibrionales>Vibrionaceae	Photobacterium_angustum	cysteine-desulfurase-[Photobacterium-angustum]	661
WP_045137458.1	cysteine-desulfurase	GCF_000950085.1	351	Bacteria>Proteobacteria>Gammaproteobacteria>Vibrionales>Vibrionaceae	Photobacterium_angustum	cysteine-desulfurase-[Photobacterium-angustum]	661
WP_236689464.1	cysteine-desulfurase	GCF_000950215.1	365	Bacteria>Proteobacteria>Gammaproteobacteria>Vibrionales>Vibrionaceae	Photobacterium_angustum	cysteine-desulfurase-[Photobacterium-angustum]	661
KAE8177255.1	cysteine-desulfurase	GCA_009389665.1	350	Bacteria>Proteobacteria>Gammaproteobacteria>Vibrionales>Vibrionaceae	Photobacterium_carnosum	cysteine-desulfurase-[Photobacterium-carnosum]	2023717
WP_152653472.1	cysteine-desulfurase	GCF_009389665.1	350	Bacteria>Proteobacteria>Gammaproteobacteria>Vibrionales>Vibrionaceae	Photobacterium_carnosum	cysteine-desulfurase-[Photobacterium-carnosum]	2023717
WP_065172218.1	cysteine-desulfurase	GCF_001676145.1	348	Bacteria>Proteobacteria>Gammaproteobacteria>Vibrionales>Vibrionaceae	Photobacterium_damselae	cysteine-desulfurase-[Photobacterium-damselae]	38293
WP_148109492.1	cysteine-desulfurase	GCF_006297395.2	348	Bacteria>Proteobacteria>Gammaproteobacteria>Vibrionales>Vibrionaceae	Photobacterium_damselae	cysteine-desulfurase-[Photobacterium-damselae]	38293
WP_045039848.1	cysteine-desulfurase	GCF_000950265.1	374	Bacteria>Proteobacteria>Gammaproteobacteria>Vibrionales>Vibrionaceae	Photobacterium_iliopiscarium	cysteine-desulfurase-DndA-[Photobacterium-iliopiscarium]	56192
WP_107289578.1	cysteine-desulfurase	GCF_003025915.1	374	Bacteria>Proteobacteria>Gammaproteobacteria>Vibrionales>Vibrionaceae	Photobacterium_kishitanii	cysteine-desulfurase-DndA-[Photobacterium-kishitanii]	318456
WP_087844575.1	cysteine-desulfurase	GCF_900185615.1	374	Bacteria>Proteobacteria>Gammaproteobacteria>Vibrionales>Vibrionaceae	Photobacterium_malacitanum	cysteine-desulfurase-DndA-[Photobacterium-malacitanum]	2204294
WP_065168712.1	cysteine-desulfurase	GCF_001676075.1	349	Bacteria>Proteobacteria>Gammaproteobacteria>Vibrionales>Vibrionaceae	Photobacterium	MULTISPECIES:-cysteine-desulfurase-[Photobacterium]	657
WP_077670067.1	cysteine-desulfurase	GCF_001996185.1	351	Bacteria>Proteobacteria>Gammaproteobacteria>Vibrionales>Vibrionaceae	Salinivibrio_costicola	cysteine-desulfurase-[Salinivibrio-costicola]	51367
OOF32902.1	cysteine-desulfurase	GCA_001996185.1	351	Bacteria>Proteobacteria>Gammaproteobacteria>Vibrionales>Vibrionaceae	Salinivibrio_costicola_subsp._alcaliphilus	cysteine-desulfurase-[Salinivibrio-costicola-subsp.-alcaliphilus]	272773
WP_176312931.1	cysteine-desulfurase	GCF_011090275.1	351	Bacteria>Proteobacteria>Gammaproteobacteria>Vibrionales>Vibrionaceae	Vibrio_aestuarianus	cysteine-desulfurase-[Vibrio-aestuarianus]	28171
EGR0169283.1	cysteine-desulfurase	GCA_015787435.1	374	Bacteria>Proteobacteria>Gammaproteobacteria>Vibrionales>Vibrionaceae	Vibrio_alginolyticus	cysteine-desulfurase-DndA-[Vibrio-alginolyticus]	663
EIL2909393.1	cysteine-desulfurase	GCA_020688565.1	374	Bacteria>Proteobacteria>Gammaproteobacteria>Vibrionales>Vibrionaceae	Vibrio_alginolyticus	cysteine-desulfurase-DndA-[Vibrio-alginolyticus]	663
MCA0936668.1	cysteine-desulfurase	GCA_020171485.1	351	Bacteria>Proteobacteria>Gammaproteobacteria>Vibrionales>Vibrionaceae	Vibrio_alginolyticus	cysteine-desulfurase-[Vibrio-alginolyticus]	663
WP_224915336.1	cysteine-desulfurase	GCF_020171485.1	351	Bacteria>Proteobacteria>Gammaproteobacteria>Vibrionales>Vibrionaceae	Vibrio_alginolyticus	cysteine-desulfurase-[Vibrio-alginolyticus]	663
WP_065678377.1	cysteine-desulfurase	-	351	Bacteria>Proteobacteria>Gammaproteobacteria>Vibrionales>Vibrionaceae	Vibrio_atlanticus	cysteine-desulfurase-[Vibrio-atlanticus]	693153
WP_102472175.1	cysteine-desulfurase	GCF_002873435.1	348	Bacteria>Proteobacteria>Gammaproteobacteria>Vibrionales>Vibrionaceae	Vibrio_breoganii	cysteine-desulfurase-[Vibrio-breoganii]	553239
WP_102549489.1	cysteine-desulfurase	GCF_002876375.1	349	Bacteria>Proteobacteria>Gammaproteobacteria>Vibrionales>Vibrionaceae	Vibrio_breoganii	cysteine-desulfurase-[Vibrio-breoganii]	553239
WP_077200994.1	cysteine-desulfurase	GCF_001969325.1	350	Bacteria>Proteobacteria>Gammaproteobacteria>Vibrionales>Vibrionaceae	Vibrio_campbellii	cysteine-desulfurase-[Vibrio-campbellii]	680
EGR0891594.1	cysteine-desulfurase	GCA_015794775.1	374	Bacteria>Proteobacteria>Gammaproteobacteria>Vibrionales>Vibrionaceae	Vibrio_cholerae	cysteine-desulfurase-DndA-[Vibrio-cholerae]	666
EII3091973.1	cysteine-desulfurase	GCA_020411025.1	374	Bacteria>Proteobacteria>Gammaproteobacteria>Vibrionales>Vibrionaceae	Vibrio_cholerae	cysteine-desulfurase-DndA-[Vibrio-cholerae]	666
HBC3475376.1	cysteine-desulfurase	GCA_018119245.1	374	Bacteria>Proteobacteria>Gammaproteobacteria>Vibrionales>Vibrionaceae	Vibrio_cholerae	cysteine-desulfurase-DndA-[Vibrio-cholerae]	666
KAA1199064.1	cysteine-desulfurase	GCA_008370435.1	374	Bacteria>Proteobacteria>Gammaproteobacteria>Vibrionales>Vibrionaceae	Vibrio_cholerae	cysteine-desulfurase-DndA-[Vibrio-cholerae]	666
QKU71312.1	cysteine-desulfurase	GCA_013357805.1	374	Bacteria>Proteobacteria>Gammaproteobacteria>Vibrionales>Vibrionaceae	Vibrio_cholerae	cysteine-desulfurase-DndA-[Vibrio-cholerae]	666
QKV04277.1	cysteine-desulfurase	GCA_013357605.1	374	Bacteria>Proteobacteria>Gammaproteobacteria>Vibrionales>Vibrionaceae	Vibrio_cholerae	cysteine-desulfurase-DndA-[Vibrio-cholerae]	666
RND30736.1	cysteine-desulfurase	GCA_003712005.1	374	Bacteria>Proteobacteria>Gammaproteobacteria>Vibrionales>Vibrionaceae	Vibrio_cholerae	cysteine-desulfurase-DndA-[Vibrio-cholerae]	666
WP_000214766.1	cysteine-desulfurase	GCF_000174235.1	374	Bacteria>Proteobacteria>Gammaproteobacteria>Vibrionales>Vibrionaceae	Vibrio_cholerae	cysteine-desulfurase-DndA-[Vibrio-cholerae]	666
WP_057568911.1	cysteine-desulfurase	GCF_013357605.1	374	Bacteria>Proteobacteria>Gammaproteobacteria>Vibrionales>Vibrionaceae	Vibrio_cholerae	cysteine-desulfurase-DndA-[Vibrio-cholerae]	666
WP_071180709.1	cysteine-desulfurase	GCF_001857485.1	374	Bacteria>Proteobacteria>Gammaproteobacteria>Vibrionales>Vibrionaceae	Vibrio_cholerae	cysteine-desulfurase-DndA-[Vibrio-cholerae]	666
WP_113628967.1	cysteine-desulfurase	GCF_003312945.1	374	Bacteria>Proteobacteria>Gammaproteobacteria>Vibrionales>Vibrionaceae	Vibrio_cholerae	cysteine-desulfurase-DndA-[Vibrio-cholerae]	666
WP_148503073.1	cysteine-desulfurase	GCF_008084455.1	374	Bacteria>Proteobacteria>Gammaproteobacteria>Vibrionales>Vibrionaceae	Vibrio_cholerae	cysteine-desulfurase-DndA-[Vibrio-cholerae]	666
WP_220284300.1	cysteine-desulfurase	GCF_019504425.1	374	Bacteria>Proteobacteria>Gammaproteobacteria>Vibrionales>Vibrionaceae	Vibrio_cholerae	cysteine-desulfurase-DndA-[Vibrio-cholerae]	666
EGR4135685.1	cysteine-desulfurase	GCA_015810845.1	351	Bacteria>Proteobacteria>Gammaproteobacteria>Vibrionales>Vibrionaceae	Vibrio_cholerae	cysteine-desulfurase-[Vibrio-cholerae]	666
MBJ6941745.1	cysteine-desulfurase	GCA_016456725.1	351	Bacteria>Proteobacteria>Gammaproteobacteria>Vibrionales>Vibrionaceae	Vibrio_cholerae	cysteine-desulfurase-[Vibrio-cholerae]	666
WP_179877281.1	cysteine-desulfurase	GCF_013414375.1	351	Bacteria>Proteobacteria>Gammaproteobacteria>Vibrionales>Vibrionaceae	Vibrio_cholerae	cysteine-desulfurase-[Vibrio-cholerae]	666
WP_199369976.1	cysteine-desulfurase	GCF_016456725.1	351	Bacteria>Proteobacteria>Gammaproteobacteria>Vibrionales>Vibrionaceae	Vibrio_cholerae	cysteine-desulfurase-[Vibrio-cholerae]	666
NRF14111.1	cysteine-desulfurase	GCA_013266605.1	374	Bacteria>Proteobacteria>Gammaproteobacteria>Vibrionales>Vibrionaceae	Vibrio_coralliilyticus	cysteine-desulfurase-DndA-[Vibrio-coralliilyticus]	190893
WP_172853913.1	cysteine-desulfurase	GCF_013266605.1	374	Bacteria>Proteobacteria>Gammaproteobacteria>Vibrionales>Vibrionaceae	Vibrio_coralliilyticus	cysteine-desulfurase-DndA-[Vibrio-coralliilyticus]	190893
WP_052879485.1	cysteine-desulfurase	GCF_000983095.1	374	Bacteria>Proteobacteria>Gammaproteobacteria>Vibrionales>Vibrionaceae	Vibrio_coralliirubri	cysteine-desulfurase-DndA-[Vibrio-coralliirubri]	1516159
WP_086775565.1	cysteine-desulfurase	GCF_024347375.1	374	Bacteria>Proteobacteria>Gammaproteobacteria>Vibrionales>Vibrionaceae	Vibrio_coralliirubri	cysteine-desulfurase-DndA-[Vibrio-coralliirubri]	1516159
WP_132815729.1	cysteine-desulfurase	-	374	Bacteria>Proteobacteria>Gammaproteobacteria>Vibrionales>Vibrionaceae	Vibrio_crassostreae	cysteine-desulfurase-DndA-[Vibrio-crassostreae]	246167
WP_102269954.1	cysteine-desulfurase	GCF_020806125.1	374	Bacteria>Proteobacteria>Gammaproteobacteria>Vibrionales>Vibrionaceae	Vibrio_cyclitrophicus	cysteine-desulfurase-DndA-[Vibrio-cyclitrophicus]	47951
KAA8598628.1	cysteine-desulfurase	GCA_008692145.1	375	Bacteria>Proteobacteria>Gammaproteobacteria>Vibrionales>Vibrionaceae	Vibrio_cyclitrophicus	Cysteine-desulfurase-DndA-[Vibrio-cyclitrophicus]	47951
WP_146493002.1	cysteine-desulfurase	GCF_024347435.1	348	Bacteria>Proteobacteria>Gammaproteobacteria>Vibrionales>Vibrionaceae	Vibrio_cyclitrophicus	cysteine-desulfurase-[Vibrio-cyclitrophicus]	47951
MBY7809512.1	cysteine-desulfurase	GCA_019852625.1	374	Bacteria>Proteobacteria>Gammaproteobacteria>Vibrionales>Vibrionaceae	Vibrio_fluvialis	cysteine-desulfurase-DndA-[Vibrio-fluvialis]	676
MBY8023709.1	cysteine-desulfurase	GCA_019853575.1	374	Bacteria>Proteobacteria>Gammaproteobacteria>Vibrionales>Vibrionaceae	Vibrio_fluvialis	cysteine-desulfurase-DndA-[Vibrio-fluvialis]	676
MBY8111608.1	cysteine-desulfurase	GCA_019853965.1	374	Bacteria>Proteobacteria>Gammaproteobacteria>Vibrionales>Vibrionaceae	Vibrio_fluvialis	cysteine-desulfurase-DndA-[Vibrio-fluvialis]	676
MBY8122671.1	cysteine-desulfurase	GCA_019854055.1	374	Bacteria>Proteobacteria>Gammaproteobacteria>Vibrionales>Vibrionaceae	Vibrio_fluvialis	cysteine-desulfurase-DndA-[Vibrio-fluvialis]	676
WP_072962192.1	cysteine-desulfurase	GCF_024346915.1	374	Bacteria>Proteobacteria>Gammaproteobacteria>Vibrionales>Vibrionaceae	Vibrio_gazogenes	cysteine-desulfurase-DndA-[Vibrio-gazogenes]	687
WP_023403202.1	cysteine-desulfurase	GCF_000496695.1	349	Bacteria>Proteobacteria>Gammaproteobacteria>Vibrionales>Vibrionaceae	Vibrio_halioticoli	cysteine-desulfurase-[Vibrio-halioticoli]	71388
WP_103880299.1	cysteine-desulfurase	GCF_900107935.1	351	Bacteria>Proteobacteria>Gammaproteobacteria>Vibrionales>Vibrionaceae	Vibrio_hangzhouensis	cysteine-desulfurase-[Vibrio-hangzhouensis]	462991
WP_239912671.1	cysteine-desulfurase	GCF_024531115.1	351	Bacteria>Proteobacteria>Gammaproteobacteria>Vibrionales>Vibrionaceae	Vibrio_harveyi	aminotransferase-class-V-fold-PLP-dependent-enzyme-[Vibrio-harveyi]	669
WP_053308119.1	cysteine-desulfurase	GCF_016812015.1	351	Bacteria>Proteobacteria>Gammaproteobacteria>Vibrionales>Vibrionaceae	Vibrio_harveyi_group	MULTISPECIES:-cysteine-desulfurase-[Vibrio-harveyi-group]	717610
GAM62953.1	cysteine-desulfurase	GCA_000830435.1	351	Bacteria>Proteobacteria>Gammaproteobacteria>Vibrionales>Vibrionaceae	Vibrio_ishigakensis	cysteine-desulfurase-[Vibrio-ishigakensis]	1481914
WP_117234233.1	cysteine-desulfurase	GCF_003415655.1	374	Bacteria>Proteobacteria>Gammaproteobacteria>Vibrionales>Vibrionaceae	Vibrio_maerlii	cysteine-desulfurase-DndA-[Vibrio-maerlii]	2231648
WP_081230287.1	cysteine-desulfurase	GCF_002875905.1	348	Bacteria>Proteobacteria>Gammaproteobacteria>Vibrionales>Vibrionaceae	Vibrio	MULTISPECIES:-cysteine-desulfurase-[Vibrio]	662
WP_206369015.1	cysteine-desulfurase	-	375	Bacteria>Proteobacteria>Gammaproteobacteria>Vibrionales>Vibrionaceae	Vibrio_neptunius	cysteine-desulfurase-DndA-[Vibrio-neptunius]	170651
CCO58313.1	cysteine-desulfurase	GCA_000801275.1	350	Bacteria>Proteobacteria>Gammaproteobacteria>Vibrionales>Vibrionaceae	Vibrio_nigripulchritudo	Cysteine-desulfurase,-mitosomal-[Vibrio-nigripulchritudo]	28173
WP_022551105.1	cysteine-desulfurase	GCF_000801275.2	350	Bacteria>Proteobacteria>Gammaproteobacteria>Vibrionales>Vibrionaceae	Vibrio_nigripulchritudo	cysteine-desulfurase-[Vibrio-nigripulchritudo]	28173
EGQ7686064.1	cysteine-desulfurase	GCA_015744865.1	374	Bacteria>Proteobacteria>Gammaproteobacteria>Vibrionales>Vibrionaceae	Vibrio_parahaemolyticus	cysteine-desulfurase-DndA-[Vibrio-parahaemolyticus]	670
EGR2691912.1	cysteine-desulfurase	GCA_015804235.1	374	Bacteria>Proteobacteria>Gammaproteobacteria>Vibrionales>Vibrionaceae	Vibrio_parahaemolyticus	cysteine-desulfurase-DndA-[Vibrio-parahaemolyticus]	670
MBE5147681.1	cysteine-desulfurase	GCA_014964085.1	374	Bacteria>Proteobacteria>Gammaproteobacteria>Vibrionales>Vibrionaceae	Vibrio_parahaemolyticus	cysteine-desulfurase-DndA-[Vibrio-parahaemolyticus]	670
WP_069522914.1	cysteine-desulfurase	GCF_001727925.1	374	Bacteria>Proteobacteria>Gammaproteobacteria>Vibrionales>Vibrionaceae	Vibrio_parahaemolyticus	cysteine-desulfurase-DndA-[Vibrio-parahaemolyticus]	670
WP_086484900.1	cysteine-desulfurase	GCF_905331605.1	374	Bacteria>Proteobacteria>Gammaproteobacteria>Vibrionales>Vibrionaceae	Vibrio_parahaemolyticus	cysteine-desulfurase-DndA-[Vibrio-parahaemolyticus]	670
WP_140316385.1	cysteine-desulfurase	GCF_006371275.1	374	Bacteria>Proteobacteria>Gammaproteobacteria>Vibrionales>Vibrionaceae	Vibrio_parahaemolyticus	cysteine-desulfurase-DndA-[Vibrio-parahaemolyticus]	670
EHH3645305.1	cysteine-desulfurase	GCA_017662515.1	350	Bacteria>Proteobacteria>Gammaproteobacteria>Vibrionales>Vibrionaceae	Vibrio_parahaemolyticus	cysteine-desulfurase-[Vibrio-parahaemolyticus]	670
EIT7126856.1	cysteine-desulfurase	GCA_021537615.1	351	Bacteria>Proteobacteria>Gammaproteobacteria>Vibrionales>Vibrionaceae	Vibrio_parahaemolyticus	cysteine-desulfurase-[Vibrio-parahaemolyticus]	670
HBC3543677.1	cysteine-desulfurase	GCA_018119595.1	351	Bacteria>Proteobacteria>Gammaproteobacteria>Vibrionales>Vibrionaceae	Vibrio_parahaemolyticus	cysteine-desulfurase-[Vibrio-parahaemolyticus]	670
MBE3696071.1	cysteine-desulfurase	GCA_014918685.1	351	Bacteria>Proteobacteria>Gammaproteobacteria>Vibrionales>Vibrionaceae	Vibrio_parahaemolyticus	cysteine-desulfurase-[Vibrio-parahaemolyticus]	670
MBM4946090.1	cysteine-desulfurase	GCA_016876775.1	351	Bacteria>Proteobacteria>Gammaproteobacteria>Vibrionales>Vibrionaceae	Vibrio_parahaemolyticus	cysteine-desulfurase-[Vibrio-parahaemolyticus]	670
NCN17896.1	cysteine-desulfurase	GCA_009991765.1	350	Bacteria>Proteobacteria>Gammaproteobacteria>Vibrionales>Vibrionaceae	Vibrio_parahaemolyticus	cysteine-desulfurase-[Vibrio-parahaemolyticus]	670
TBT11765.1	cysteine-desulfurase	GCA_004324425.1	351	Bacteria>Proteobacteria>Gammaproteobacteria>Vibrionales>Vibrionaceae	Vibrio_parahaemolyticus	cysteine-desulfurase-[Vibrio-parahaemolyticus]	670
TON46945.1	cysteine-desulfurase	GCA_006375215.1	351	Bacteria>Proteobacteria>Gammaproteobacteria>Vibrionales>Vibrionaceae	Vibrio_parahaemolyticus	cysteine-desulfurase-[Vibrio-parahaemolyticus]	670
WP_025586939.1	cysteine-desulfurase	GCF_000489215.1	350	Bacteria>Proteobacteria>Gammaproteobacteria>Vibrionales>Vibrionaceae	Vibrio_parahaemolyticus	cysteine-desulfurase-[Vibrio-parahaemolyticus]	670
WP_031850119.1	cysteine-desulfurase	GCF_012274985.1	351	Bacteria>Proteobacteria>Gammaproteobacteria>Vibrionales>Vibrionaceae	Vibrio_parahaemolyticus	cysteine-desulfurase-[Vibrio-parahaemolyticus]	670
WP_152919089.1	cysteine-desulfurase	GCF_009389915.1	351	Bacteria>Proteobacteria>Gammaproteobacteria>Vibrionales>Vibrionaceae	Vibrio_parahaemolyticus	cysteine-desulfurase-[Vibrio-parahaemolyticus]	670
WP_161609937.1	cysteine-desulfurase	GCF_009991765.1	350	Bacteria>Proteobacteria>Gammaproteobacteria>Vibrionales>Vibrionaceae	Vibrio_parahaemolyticus	cysteine-desulfurase-[Vibrio-parahaemolyticus]	670
WP_077336582.1	cysteine-desulfurase	GCF_024347115.1	374	Bacteria>Proteobacteria>Gammaproteobacteria>Vibrionales>Vibrionaceae	Vibrio_ruber	cysteine-desulfurase-DndA-[Vibrio-ruber]	184755
WP_170890757.1	cysteine-desulfurase	GCF_013048495.1	374	Bacteria>Proteobacteria>Gammaproteobacteria>Vibrionales>Vibrionaceae	Vibrio_sp._2-1(7)	cysteine-desulfurase-DndA-[Vibrio-sp.-2-1(7)]	2591011
AEX21537.1	cysteine-desulfurase	GCA_000241385.1	350	Bacteria>Proteobacteria>Gammaproteobacteria>Vibrionales>Vibrionaceae	Vibrio_sp._EJY3	cysteine-desulfurase-[Vibrio-sp.-EJY3]	1116375
WP_014231417.1	cysteine-desulfurase	GCF_000241385.1	350	Bacteria>Proteobacteria>Gammaproteobacteria>Vibrionales>Vibrionaceae	Vibrio_sp._EJY3	cysteine-desulfurase-[Vibrio-sp.-EJY3]	1116375
WP_136484166.1	cysteine-desulfurase	GCF_004804295.1	374	Bacteria>Proteobacteria>Gammaproteobacteria>Vibrionales>Vibrionaceae	Vibrio_sp._H11	cysteine-desulfurase-DndA-[Vibrio-sp.-H11]	2565928
WP_017074155.1	cysteine-desulfurase	GCF_000272225.2	375	Bacteria>Proteobacteria>Gammaproteobacteria>Vibrionales>Vibrionaceae	Vibrio_splendidus	cysteine-desulfurase-DndA-[Vibrio-splendidus]	29497
WP_059120163.1	cysteine-desulfurase	GCF_001506075.1	374	Bacteria>Proteobacteria>Gammaproteobacteria>Vibrionales>Vibrionaceae	Vibrio_sp._MEBiC08052	cysteine-desulfurase-DndA-[Vibrio-sp.-MEBiC08052]	1761910
WP_193841263.1	cysteine-desulfurase	GCF_015163615.1	348	Bacteria>Proteobacteria>Gammaproteobacteria>Vibrionales>Vibrionaceae	Vibrio_sp._OPT18	cysteine-desulfurase-[Vibrio-sp.-OPT18]	2778641
WP_105977280.1	cysteine-desulfurase	GCF_002995435.1	374	Bacteria>Proteobacteria>Gammaproteobacteria>Vibrionales>Vibrionaceae	Vibrio_sp._V01_P9A10T6	cysteine-desulfurase-DndA-[Vibrio-sp.-V01_P9A10T6]	2116368
WP_172380631.1	cysteine-desulfurase	GCF_902751245.1	364	Bacteria>Proteobacteria>Gammaproteobacteria>Vibrionales>Vibrionaceae	Vibrio_sp._Vb339	aminotransferase-class-V-fold-PLP-dependent-enzyme-[Vibrio-sp.-Vb339]	1192013
WP_102521373.1	cysteine-desulfurase	GCF_900233005.1	358	Bacteria>Proteobacteria>Gammaproteobacteria>Vibrionales>Vibrionaceae	Vibrio_tapetis	cysteine-desulfurase-[Vibrio-tapetis]	52443
WP_068714508.1	cysteine-desulfurase	GCF_001547935.1	374	Bacteria>Proteobacteria>Gammaproteobacteria>Vibrionales>Vibrionaceae	Vibrio_tritonius	cysteine-desulfurase-DndA-[Vibrio-tritonius]	1435069
HAS6142355.1	cysteine-desulfurase	GCA_015776655.1	350	Bacteria>Proteobacteria>Gammaproteobacteria>Vibrionales>Vibrionaceae	Vibrio_vulnificus	aminotransferase-class-V-fold-PLP-dependent-enzyme-[Vibrio-vulnificus]	672
WP_226048877.1	cysteine-desulfurase	GCF_014525125.1	249	Bacteria>Proteobacteria>Gammaproteobacteria>Vibrionales>Vibrionaceae	Vibrio_vulnificus	aminotransferase-class-V-fold-PLP-dependent-enzyme-[Vibrio-vulnificus]	672
EGQ7982862.1	cysteine-desulfurase	GCA_015960135.1	374	Bacteria>Proteobacteria>Gammaproteobacteria>Vibrionales>Vibrionaceae	Vibrio_vulnificus	cysteine-desulfurase-DndA-[Vibrio-vulnificus]	672
HAS8378256.1	cysteine-desulfurase	GCA_015821155.1	374	Bacteria>Proteobacteria>Gammaproteobacteria>Vibrionales>Vibrionaceae	Vibrio_vulnificus	cysteine-desulfurase-DndA-[Vibrio-vulnificus]	672
MCA3964059.1	cysteine-desulfurase	GCA_020252125.1	374	Bacteria>Proteobacteria>Gammaproteobacteria>Vibrionales>Vibrionaceae	Vibrio_vulnificus	cysteine-desulfurase-DndA-[Vibrio-vulnificus]	672
NTJ40905.1	cysteine-desulfurase	GCA_013319355.1	374	Bacteria>Proteobacteria>Gammaproteobacteria>Vibrionales>Vibrionaceae	Vibrio_vulnificus	cysteine-desulfurase-DndA-[Vibrio-vulnificus]	672
POB66751.1	cysteine-desulfurase	GCA_002903375.1	374	Bacteria>Proteobacteria>Gammaproteobacteria>Vibrionales>Vibrionaceae	Vibrio_vulnificus	cysteine-desulfurase-DndA-[Vibrio-vulnificus]	672
RZP63905.1	cysteine-desulfurase	GCA_004214565.1	374	Bacteria>Proteobacteria>Gammaproteobacteria>Vibrionales>Vibrionaceae	Vibrio_vulnificus	cysteine-desulfurase-DndA-[Vibrio-vulnificus]	672
WP_017790215.1	cysteine-desulfurase	GCF_000342305.2	374	Bacteria>Proteobacteria>Gammaproteobacteria>Vibrionales>Vibrionaceae	Vibrio_vulnificus	cysteine-desulfurase-DndA-[Vibrio-vulnificus]	672
WP_102887625.1	cysteine-desulfurase	GCF_002891755.1	374	Bacteria>Proteobacteria>Gammaproteobacteria>Vibrionales>Vibrionaceae	Vibrio_vulnificus	cysteine-desulfurase-DndA-[Vibrio-vulnificus]	672
WP_130249895.1	cysteine-desulfurase	GCF_020252105.1	374	Bacteria>Proteobacteria>Gammaproteobacteria>Vibrionales>Vibrionaceae	Vibrio_vulnificus	cysteine-desulfurase-DndA-[Vibrio-vulnificus]	672
WP_130309741.1	cysteine-desulfurase	GCF_004215235.1	374	Bacteria>Proteobacteria>Gammaproteobacteria>Vibrionales>Vibrionaceae	Vibrio_vulnificus	cysteine-desulfurase-DndA-[Vibrio-vulnificus]	672
WP_154142354.1	cysteine-desulfurase	GCF_009665475.1	374	Bacteria>Proteobacteria>Gammaproteobacteria>Vibrionales>Vibrionaceae	Vibrio_vulnificus	cysteine-desulfurase-DndA-[Vibrio-vulnificus]	672
WP_225483116.1	cysteine-desulfurase	GCF_020251885.1	374	Bacteria>Proteobacteria>Gammaproteobacteria>Vibrionales>Vibrionaceae	Vibrio_vulnificus	cysteine-desulfurase-DndA-[Vibrio-vulnificus]	672
WP_238822174.1	cysteine-desulfurase	GCF_022289495.1	374	Bacteria>Proteobacteria>Gammaproteobacteria>Vibrionales>Vibrionaceae	Vibrio_vulnificus	cysteine-desulfurase-DndA-[Vibrio-vulnificus]	672
EGQ9303375.1	cysteine-desulfurase	GCA_015783695.1	351	Bacteria>Proteobacteria>Gammaproteobacteria>Vibrionales>Vibrionaceae	Vibrio_vulnificus	cysteine-desulfurase-[Vibrio-vulnificus]	672
EGR7943015.1	cysteine-desulfurase	GCA_015838415.1	350	Bacteria>Proteobacteria>Gammaproteobacteria>Vibrionales>Vibrionaceae	Vibrio_vulnificus	cysteine-desulfurase-[Vibrio-vulnificus]	672
EHV9838117.1	cysteine-desulfurase	GCA_019438685.1	350	Bacteria>Proteobacteria>Gammaproteobacteria>Vibrionales>Vibrionaceae	Vibrio_vulnificus	cysteine-desulfurase-[Vibrio-vulnificus]	672
HAS8119094.1	cysteine-desulfurase	GCA_015819725.1	351	Bacteria>Proteobacteria>Gammaproteobacteria>Vibrionales>Vibrionaceae	Vibrio_vulnificus	cysteine-desulfurase-[Vibrio-vulnificus]	672
HAS8323607.1	cysteine-desulfurase	GCA_015820405.1	350	Bacteria>Proteobacteria>Gammaproteobacteria>Vibrionales>Vibrionaceae	Vibrio_vulnificus	cysteine-desulfurase-[Vibrio-vulnificus]	672
MCA3904027.1	cysteine-desulfurase	GCA_020251905.1	351	Bacteria>Proteobacteria>Gammaproteobacteria>Vibrionales>Vibrionaceae	Vibrio_vulnificus	cysteine-desulfurase-[Vibrio-vulnificus]	672
MCA3956858.1	cysteine-desulfurase	GCA_020252065.1	351	Bacteria>Proteobacteria>Gammaproteobacteria>Vibrionales>Vibrionaceae	Vibrio_vulnificus	cysteine-desulfurase-[Vibrio-vulnificus]	672
RZP66229.1	cysteine-desulfurase	GCA_004214655.1	351	Bacteria>Proteobacteria>Gammaproteobacteria>Vibrionales>Vibrionaceae	Vibrio_vulnificus	cysteine-desulfurase-[Vibrio-vulnificus]	672
RZQ28714.1	cysteine-desulfurase	GCA_004214495.1	351	Bacteria>Proteobacteria>Gammaproteobacteria>Vibrionales>Vibrionaceae	Vibrio_vulnificus	cysteine-desulfurase-[Vibrio-vulnificus]	672
RZQ77269.1	cysteine-desulfurase	GCA_004215035.1	351	Bacteria>Proteobacteria>Gammaproteobacteria>Vibrionales>Vibrionaceae	Vibrio_vulnificus	cysteine-desulfurase-[Vibrio-vulnificus]	672

Accession	Annotation	GCA	Length	Phyletic.spread	Strain.name	Defline	Species.Taxid
WP_130194895.1	cysteine-desulfurase	-	351	Bacteria>Proteobacteria>Gammaproteobacteria>Vibrionales>Vibrionaceae	Vibrio_vulnificus	cysteine-desulfurase-[Vibrio-vulnificus]	672
WP_130197603.1	cysteine-desulfurase	GCF_020252065.1	351	Bacteria>Proteobacteria>Gammaproteobacteria>Vibrionales>Vibrionaceae	Vibrio_vulnificus	cysteine-desulfurase-[Vibrio-vulnificus]	672
WP_192928191.1	cysteine-desulfurase	GCF_014525125.1	350	Bacteria>Proteobacteria>Gammaproteobacteria>Vibrionales>Vibrionaceae	Vibrio_vulnificus	cysteine-desulfurase-[Vibrio-vulnificus]	672
WP_225460608.1	cysteine-desulfurase	GCF_020251905.1	351	Bacteria>Proteobacteria>Gammaproteobacteria>Vibrionales>Vibrionaceae	Vibrio_vulnificus	cysteine-desulfurase-[Vibrio-vulnificus]	672
WP_147627462.1	cysteine-desulfurase	GCF_008000795.1	386	Bacteria>Proteobacteria>Gammaproteobacteria>Xanthomonadales>Rhodanobacteraceae	Rhodanobacter_glycinis	cysteine-desulfurase-[Rhodanobacter-glycinis]	582702
WP_232580333.1	cysteine-desulfurase	GCF_021233355.1	386	Bacteria>Proteobacteria>Gammaproteobacteria>Xanthomonadales>Xanthomonadaceae	Luteimonas_fraxinea	cysteine-desulfurase-[Luteimonas-sp.-D4P002]	2901869
WP_224096441.1	cysteine-desulfurase	GCF_020042665.1	401	Bacteria>Proteobacteria>Gammaproteobacteria>Xanthomonadales>Xanthomonadaceae	Pseudoxanthomonas_sp._X-1	cysteine-desulfurase-[Pseudoxanthomonas-sp.-X-1]	2571115
WP_205622354.1	cysteine-desulfurase	GCF_000972745.1	379	Bacteria>Proteobacteria>Gammaproteobacteria>Xanthomonadales>Xanthomonadaceae	Xanthomonas_arboricola	aminotransferase-class-V-fold-PLP-dependent-enzyme-[Xanthomonas-arboricola]	56448
MBL6991875.1	cysteine-desulfurase	GCA_016784965.1	388	Bacteria>Proteobacteria>Oligoflexia>Bacteriovoracales>Bacteriovoracaceae	Bacteriovoracaceae_bacterium	cysteine-desulfurase-[Bacteriovoracaceae-bacterium]	2081706
WP_096908805.1	cysteine-desulfurase	GCF_002442875.1	367	Bacteria>Proteobacteria>Oligoflexia>Bacteriovoracales>Halobacteriovoraceae	Halobacteriovorax_marinus	cysteine-desulfurase-[Halobacteriovorax-marinus]	97084
MCA0359069.1	cysteine-desulfurase	GCA_020161365.1	381	Bacteria>Proteobacteria>unclassified_Proteobacteria	Proteobacteria_bacterium	cysteine-desulfurase-DndA-[Proteobacteria-bacterium]	1977087
MBW4054342.1	cysteine-desulfurase	GCA_019352135.1	382	Bacteria>Proteobacteria>unclassified_Proteobacteria	Proteobacteria_bacterium	cysteine-desulfurase-[Proteobacteria-bacterium]	1977087
NBP02147.1	cysteine-desulfurase	GCA_009918765.1	377	Bacteria>Proteobacteria>unclassified_Proteobacteria	Proteobacteria_bacterium	cysteine-desulfurase-[Proteobacteria-bacterium]	1977087
MBP7830369.1	cysteine-desulfurase	GCA_018001225.1	380	Bacteria>PVC_group>Kiritimatiellaeota>Kiritimatiellae>unclassified_Kiritimatiellae	Kiritimatiellae_bacterium	aminotransferase-class-V-fold-PLP-dependent-enzyme-[Kiritimatiellae-bacterium]	2212473
MCF6175085.1	cysteine-desulfurase	GCA_021647315.1	380	Bacteria>PVC_group>Lentisphaerae>Lentisphaeria>Victivallales	Victivallaceae_bacterium	cysteine-desulfurase-DndA-[Victivallaceae-bacterium]	2793042
NLB68725.1	cysteine-desulfurase	GCA_012511845.1	382	Bacteria>PVC_group>Lentisphaerae>unclassified_Lentisphaerae	Lentisphaerae_bacterium	aminotransferase-class-V-fold-PLP-dependent-enzyme-[Lentisphaerae-bacterium]	1932692
KXK30219.1	cysteine-desulfurase	GCA_001567345.1	384	Bacteria>PVC_group>Planctomycetes>Candidatus_Brocadiia>Candidatus_Brocadiales	Candidatus_Brocadia_sinica	cysteine-desulfurase-[Candidatus-Brocadia-sinica]	795830
KAA0245644.1	cysteine-desulfurase	GCA_008363395.1	386	Bacteria>PVC_group>Planctomycetes>Candidatus_Brocadiia>Candidatus_Brocadiales	Candidatus_Brocadia_sp._AMX2	cysteine-desulfurase-[Candidatus-Brocadia-sp.-AMX2]	2293635
MBC6930713.1	cysteine-desulfurase	GCA_014338225.1	386	Bacteria>PVC_group>Planctomycetes>Candidatus_Brocadiia>Candidatus_Brocadiales	Candidatus_Brocadia_sp.	MAG:-cysteine-desulfurase-[Candidatus-Brocadia-sp.]	2172549
RIJ93306.1	cysteine-desulfurase	GCA_003577215.1	377	Bacteria>PVC_group>Planctomycetes>Candidatus_Brocadiia>Candidatus_Brocadiales	Candidatus_Brocadia_sp.	MAG:-IscS-subfamily-cysteine-desulfurase-[Candidatus-Brocadia-sp.]	2172549
TVL98787.1	cysteine-desulfurase	GCA_007618135.1	374	Bacteria>PVC_group>Planctomycetes>Candidatus_Brocadiia>Candidatus_Brocadiales	Candidatus_Brocadia_sp._WS118	cysteine-desulfurase-DndA-[Candidatus-Brocadia-sp.-WS118]	2040625
MBS1258876.1	cysteine-desulfurase	GCA_018263435.1	220	Bacteria>PVC_group>Planctomycetes>Candidatus_Brocadiia>Candidatus_Brocadiales	Candidatus_Scalindua_arabica	Cysteine-desulfurase-IscS-[Candidatus-Scalindua-arabica]	1127984
MBT6050833.1	cysteine-desulfurase	GCA_018697755.1	279	Bacteria>PVC_group>Planctomycetes>Candidatus_Brocadiia>Candidatus_Brocadiales	Candidatus_Scalindua_sp.	cysteine-desulfurase-[Candidatus-Scalindua-sp.]	2268189
MBN2564061.1	cysteine-desulfurase	GCA_016935885.1	391	Bacteria>PVC_group>Planctomycetes>Phycisphaerae>unclassified_Phycisphaerae	Phycisphaerae_bacterium	IscS-subfamily-cysteine-desulfurase-[Phycisphaerae-bacterium]	2026778
WP_165226407.1	cysteine-desulfurase	GCF_011064615.1	393	Bacteria>PVC_group>Planctomycetes>Planctomycetia>Isosphaerales	Aquisphaera_sp._JC669	cysteine-desulfurase-[Aquisphaera-sp.-JC669]	2712864
WP_145078484.1	cysteine-desulfurase	GCF_007752135.1	396	Bacteria>PVC_group>Planctomycetes>Planctomycetia>Pirellulales	Aureliella_helgolandensis	cysteine-desulfurase-[Aureliella-helgolandensis]	2527968
WP_007325804.1	cysteine-desulfurase	GCF_000195185.1	384	Bacteria>PVC_group>Planctomycetes>Planctomycetia>Pirellulales	Rhodopirellula_baltica	aminotransferase-class-V-fold-PLP-dependent-enzyme-[Rhodopirellula-baltica]	265606
EGF28179.1	cysteine-desulfurase	GCA_000195185.2	384	Bacteria>PVC_group>Planctomycetes>Planctomycetia>Pirellulales	Rhodopirellula_baltica_WH47	cysteine-desulfurase-IscS-[Rhodopirellula-baltica-WH47]	991778
MCC9654583.1	cysteine-desulfurase	GCA_020966775.1	355	Bacteria>PVC_group>Planctomycetes>Planctomycetia>Pirellulales	Rhodopirellula_sp._JC737	aminotransferase-class-V-fold-PLP-dependent-enzyme-[Rhodopirellula-sp.-JC737]	2894198
WP_230251572.1	cysteine-desulfurase	-	355	Bacteria>PVC_group>Planctomycetes>Planctomycetia>Pirellulales	Rhodopirellula_sp._JC737	aminotransferase-class-V-fold-PLP-dependent-enzyme-[Rhodopirellula-sp.-JC737]	2894198
WP_095741171.1	cysteine-desulfurase	GCF_002289565.1	384	Bacteria>PVC_group>Planctomycetes>Planctomycetia>Pirellulales	Rhodopirellula_sp._SM50	aminotransferase-class-V-fold-PLP-dependent-enzyme-[Rhodopirellula-sp.-SM50]	595453
PAY16678.1	cysteine-desulfurase	GCA_002289565.1	384	Bacteria>PVC_group>Planctomycetes>Planctomycetia>Pirellulales	Rhodopirellula_sp._SM50	cysteine-desulfurase-DndA-[Rhodopirellula-sp.-SM50]	595453
WP_238388803.1	cysteine-desulfurase	-	359	Bacteria>PVC_group>Planctomycetes>Planctomycetia>Pirellulales	Roseimaritima_ulvae	aminotransferase-class-V-fold-PLP-dependent-enzyme-[Roseimaritima-ulvae]	980254
WP_145346090.1	cysteine-desulfurase	GCF_007741475.1	387	Bacteria>PVC_group>Planctomycetes>Planctomycetia>Pirellulales	Rosistilla_ulvae	cysteine-desulfurase-[Rosistilla-ulvae]	1930277
WP_145359716.1	cysteine-desulfurase	GCF_007743815.1	387	Bacteria>PVC_group>Planctomycetes>Planctomycetia>Planctomycetales	Alienimonas_californiensis	aminotransferase-class-V-fold-PLP-dependent-enzyme-[Alienimonas-californiensis]	2527989
QDT16786.1	cysteine-desulfurase	GCA_007743815.1	387	Bacteria>PVC_group>Planctomycetes>Planctomycetia>Planctomycetales	Alienimonas_californiensis	Cysteine-desulfurase-[Alienimonas-californiensis]	2527989
WP_145305594.1	cysteine-desulfurase	GCF_007754425.1	389	Bacteria>PVC_group>Planctomycetes>Planctomycetia>Planctomycetales	Gimesia_fumaroli	cysteine-desulfurase-[Gimesia-fumaroli]	2527976
QDV48451.1	cysteine-desulfurase	GCA_007754425.1	389	Bacteria>PVC_group>Planctomycetes>Planctomycetia>Planctomycetales	Gimesia_fumaroli	Cysteine-desulfurase-[Gimesia-fumaroli]	2527976
HBJ39007.1	cysteine-desulfurase	GCA_003506095.1	377	Bacteria>PVC_group>Planctomycetes>Planctomycetia>Planctomycetales	Planctomycetaceae_bacterium	cysteine-desulfurase-DndA-[Planctomycetaceae-bacterium]	2026779
NBO92770.1	cysteine-desulfurase	GCA_009918525.1	389	Bacteria>PVC_group>Planctomycetes>Planctomycetia>unclassified_Planctomycetia	Planctomycetia_bacterium	cysteine-desulfurase-[Planctomycetia-bacterium]	2052181
MCC6781767.1	cysteine-desulfurase	GCA_020848735.1	387	Bacteria>PVC_group>Planctomycetes>unclassified_Planctomycetes	Planctomycetes_bacterium	aminotransferase-class-V-fold-PLP-dependent-enzyme-[Planctomycetes-bacterium]	2026780
MAB77743.1	cysteine-desulfurase	GCA_002683825.1	388	Bacteria>PVC_group>Planctomycetes>unclassified_Planctomycetes	Planctomycetes_bacterium	cysteine-desulfurase-DndA-[Planctomycetes-bacterium]	2026780
MBE3144917.1	cysteine-desulfurase	GCA_014894345.1	373	Bacteria>PVC_group>Planctomycetes>unclassified_Planctomycetes	Planctomycetes_bacterium	cysteine-desulfurase-[Planctomycetes-bacterium]	2026780
MBE7492975.1	cysteine-desulfurase	GCA_015075515.1	387	Bacteria>PVC_group>Planctomycetes>unclassified_Planctomycetes	Planctomycetes_bacterium	cysteine-desulfurase-[Planctomycetes-bacterium]	2026780
MBI4242776.1	cysteine-desulfurase	GCA_016206485.1	379	Bacteria>PVC_group>Planctomycetes>unclassified_Planctomycetes	Planctomycetes_bacterium	cysteine-desulfurase-[Planctomycetes-bacterium]	2026780
HAV13930.1	cysteine-desulfurase	GCA_003485085.1	374	Bacteria>PVC_group>Verrucomicrobia>Opitutae>unclassified_Opitutae	Opitutae_bacterium	cysteine-desulfurase-DndA-[Opitutae-bacterium]	2026771
MBI4028626.1	cysteine-desulfurase	GCA_016198745.1	383	Bacteria>PVC_group>Verrucomicrobia>unclassified_Verrucomicrobia	Verrucomicrobia_bacterium	aminotransferase-class-V-fold-PLP-dependent-enzyme-[Verrucomicrobia-bacterium]	2026799
MBK9138945.1	cysteine-desulfurase	GCA_016716505.1	376	Bacteria>PVC_group>Verrucomicrobia>unclassified_Verrucomicrobia	Verrucomicrobia_bacterium	aminotransferase-class-V-fold-PLP-dependent-enzyme-[Verrucomicrobia-bacterium]	2026799
MBM3861554.1	cysteine-desulfurase	GCA_016871575.1	409	Bacteria>PVC_group>Verrucomicrobia>unclassified_Verrucomicrobia	Verrucomicrobia_bacterium	aminotransferase-class-V-fold-PLP-dependent-enzyme-[Verrucomicrobia-bacterium]	2026799
MBP7826422.1	cysteine-desulfurase	GCA_018001285.1	382	Bacteria>PVC_group>Verrucomicrobia>unclassified_Verrucomicrobia	Verrucomicrobia_bacterium	aminotransferase-class-V-fold-PLP-dependent-enzyme-[Verrucomicrobia-bacterium]	2026799
MBV9658015.1	cysteine-desulfurase	GCA_019241085.1	367	Bacteria>PVC_group>Verrucomicrobia>unclassified_Verrucomicrobia	Verrucomicrobia_bacterium	aminotransferase-class-V-fold-PLP-dependent-enzyme-[Verrucomicrobia-bacterium]	2026799
MBS0659627.1	cysteine-desulfurase	GCA_018240025.1	393	Bacteria>PVC_group>Verrucomicrobia>unclassified_Verrucomicrobia	Verrucomicrobia_bacterium	cysteine-desulfurase-DndA-[Verrucomicrobia-bacterium]	2026799
PWU19849.1	cysteine-desulfurase	GCA_003176045.1	376	Bacteria>PVC_group>Verrucomicrobia>unclassified_Verrucomicrobia	Verrucomicrobia_bacterium	cysteine-desulfurase-[Verrucomicrobia-bacterium]	2026799
TLD70494.1	cysteine-desulfurase	GCA_005780165.1	387	Bacteria>PVC_group>Verrucomicrobia>Verrucomicrobiae>Verrucomicrobiales	Phragmitibacter_flavus	aminotransferase-class-V-fold-PLP-dependent-enzyme-[Phragmitibacter-flavus]	2576071
WP_166442813.1	cysteine-desulfurase	GCF_005780165.1	380	Bacteria>PVC_group>Verrucomicrobia>Verrucomicrobiae>Verrucomicrobiales	Phragmitibacter_flavus	aminotransferase-class-V-fold-PLP-dependent-enzyme-[Phragmitibacter-flavus]	2576071
MBL9115288.1	cysteine-desulfurase	GCA_016795485.1	378	Bacteria>PVC_group>Verrucomicrobia>Verrucomicrobiae>Verrucomicrobiales	Verrucomicrobiaceae_bacterium	aminotransferase-class-V-fold-PLP-dependent-enzyme-[Verrucomicrobiaceae-bacterium]	2026800
MBX7210399.1	cysteine-desulfurase	GCA_019694165.1	378	Bacteria>PVC_group>Verrucomicrobia>Verrucomicrobiae>Verrucomicrobiales	Verrucomicrobiaceae_bacterium	aminotransferase-class-V-fold-PLP-dependent-enzyme-[Verrucomicrobiaceae-bacterium]	2026800
MAS93100.1	cysteine-desulfurase	GCA_002698785.1	376	Bacteria>PVC_group>Verrucomicrobia>Verrucomicrobiae>Verrucomicrobiales	Verrucomicrobiales_bacterium	cysteine-desulfurase-DndA-[Verrucomicrobiales-bacterium]	2026801
MBL9153029.1	cysteine-desulfurase	GCA_016795505.1	377	Bacteria>PVC_group>Verrucomicrobia>Verrucomicrobiae>Verrucomicrobiales	Verrucomicrobiales_bacterium	cysteine-desulfurase-DndA-[Verrucomicrobiales-bacterium]	2026801
WP_074932241.1	cysteine-desulfurase	GCF_900113965.1	382	Bacteria>Spirochaetes>Spirochaetia>Spirochaetales>Treponemataceae	Treponema_bryantii	aminotransferase-class-V-fold-PLP-dependent-enzyme-[Treponema-bryantii]	163
WP_198442723.1	cysteine-desulfurase	GCF_016117655.1	388	Bacteria>Spirochaetes>Spirochaetia>Spirochaetales>Treponemataceae	Treponema_peruense	aminotransferase-class-V-fold-PLP-dependent-enzyme-[Treponema-sp.-RCC2812]	2787628
MBD5399456.1	cysteine-desulfurase	GCA_014803565.1	414	Bacteria>Spirochaetes>Spirochaetia>Spirochaetales>Treponemataceae	Treponema_sp.	aminotransferase-class-V-fold-PLP-dependent-enzyme-[Treponema-sp.]	166
MBD5410220.1	cysteine-desulfurase	GCA_014803695.1	414	Bacteria>Spirochaetes>Spirochaetia>Spirochaetales>Treponemataceae	Treponema_sp.	aminotransferase-class-V-fold-PLP-dependent-enzyme-[Treponema-sp.]	166
MBD5426343.1	cysteine-desulfurase	GCA_014803965.1	414	Bacteria>Spirochaetes>Spirochaetia>Spirochaetales>Treponemataceae	Treponema_sp.	aminotransferase-class-V-fold-PLP-dependent-enzyme-[Treponema-sp.]	166
MBD5447349.1	cysteine-desulfurase	GCA_014804085.1	414	Bacteria>Spirochaetes>Spirochaetia>Spirochaetales>Treponemataceae	Treponema_sp.	aminotransferase-class-V-fold-PLP-dependent-enzyme-[Treponema-sp.]	166
MBP3772093.1	cysteine-desulfurase	GCA_017940515.1	410	Bacteria>Spirochaetes>Spirochaetia>Spirochaetales>Treponemataceae	Treponema_sp.	aminotransferase-class-V-fold-PLP-dependent-enzyme-[Treponema-sp.]	166
MBQ9103461.1	cysteine-desulfurase	GCA_017461905.1	386	Bacteria>Spirochaetes>Spirochaetia>Spirochaetales>Treponemataceae	Treponema_sp.	aminotransferase-class-V-fold-PLP-dependent-enzyme-[Treponema-sp.]	166
MBQ9281149.1	cysteine-desulfurase	GCA_017469785.1	423	Bacteria>Spirochaetes>Spirochaetia>Spirochaetales>Treponemataceae	Treponema_sp.	aminotransferase-class-V-fold-PLP-dependent-enzyme-[Treponema-sp.]	166
MBR1713901.1	cysteine-desulfurase	GCA_017476625.1	396	Bacteria>Spirochaetes>Spirochaetia>Spirochaetales>Treponemataceae	Treponema_sp.	aminotransferase-class-V-fold-PLP-dependent-enzyme-[Treponema-sp.]	166
MBR4630504.1	cysteine-desulfurase	GCA_017525605.1	397	Bacteria>Spirochaetes>Spirochaetia>Spirochaetales>Treponemataceae	Treponema_sp.	aminotransferase-class-V-fold-PLP-dependent-enzyme-[Treponema-sp.]	166
MBS7266460.1	cysteine-desulfurase	GCA_018383995.1	409	Bacteria>Spirochaetes>Spirochaetia>Spirochaetales>Treponemataceae	Treponema_sp.	aminotransferase-class-V-fold-PLP-dependent-enzyme-[Treponema-sp.]	166
WP_013701821.1	cysteine-desulfurase	GCF_000195275.1	386	Bacteria>Spirochaetes>Spirochaetia>Spirochaetales>Treponemataceae	Treponema_succinifaciens	aminotransferase-class-V-fold-PLP-dependent-enzyme-[Treponema-succinifaciens]	167
MBN2532976.1	cysteine-desulfurase	GCA_016935735.1	345	Bacteria>Spirochaetes>Spirochaetia>Spirochaetales>unclassified_Spirochaetales	Spirochaetales_bacterium	aminotransferase-class-V-fold-PLP-dependent-enzyme-[Spirochaetales-bacterium]	2026792
MBT89380.1	cysteine-desulfurase	GCA_002730475.1	375	Bacteria>Spirochaetes>Spirochaetia>Spirochaetales>unclassified_Spirochaetales	Spirochaetales_bacterium	cysteine-desulfurase-DndA-[Spirochaetales-bacterium]	2026792
MXW60196.1	cysteine-desulfurase	GCA_009835385.1	377	Bacteria>Terrabacteria_group>Actinobacteria>Acidimicrobiia>Acidimicrobiales	Acidimicrobiaceae_bacterium	aminotransferase-class-V-fold-PLP-dependent-enzyme-[Acidimicrobiaceae-bacterium]	2024894
MXW74915.1	cysteine-desulfurase	GCA_009836285.1	377	Bacteria>Terrabacteria_group>Actinobacteria>Acidimicrobiia>Acidimicrobiales	Acidimicrobiaceae_bacterium	aminotransferase-class-V-fold-PLP-dependent-enzyme-[Acidimicrobiaceae-bacterium]	2024894
MYA74735.1	cysteine-desulfurase	GCA_009838925.1	377	Bacteria>Terrabacteria_group>Actinobacteria>Acidimicrobiia>Acidimicrobiales	Acidimicrobiaceae_bacterium	aminotransferase-class-V-fold-PLP-dependent-enzyme-[Acidimicrobiaceae-bacterium]	2024894
MXX42476.1	cysteine-desulfurase	GCA_009836375.1	379	Bacteria>Terrabacteria_group>Actinobacteria>Acidimicrobiia>Acidimicrobiales	Acidimicrobiales_bacterium	aminotransferase-class-V-fold-PLP-dependent-enzyme-[Acidimicrobiales-bacterium]	2201156
MYI07898.1	cysteine-desulfurase	GCA_009844415.1	379	Bacteria>Terrabacteria_group>Actinobacteria>Acidimicrobiia>Acidimicrobiales	Acidimicrobiales_bacterium	aminotransferase-class-V-fold-PLP-dependent-enzyme-[Acidimicrobiales-bacterium]	2201156
MXV88018.1	cysteine-desulfurase	GCA_009835365.1	392	Bacteria>Terrabacteria_group>Actinobacteria>Acidimicrobiia>Acidimicrobiales	Acidimicrobiales_bacterium	cysteine-desulfurase-[Acidimicrobiales-bacterium]	2201156
WP_052606672.1	cysteine-desulfurase	GCF_000949295.1	392	Bacteria>Terrabacteria_group>Actinobacteria>Acidimicrobiia>Acidimicrobiales	Acidithrix_ferrooxidans	aminotransferase-class-V-fold-PLP-dependent-enzyme-[Acidithrix-ferrooxidans]	1280514
MXW59369.1	cysteine-desulfurase	GCA_009836065.1	377	Bacteria>Terrabacteria_group>Actinobacteria>Acidimicrobiia>unclassified_Acidimicrobiia	Acidimicrobiia_bacterium	aminotransferase-class-V-fold-PLP-dependent-enzyme-[Acidimicrobiia-bacterium]	2080302
MYB08805.1	cysteine-desulfurase	GCA_009838875.1	377	Bacteria>Terrabacteria_group>Actinobacteria>Acidimicrobiia>unclassified_Acidimicrobiia	Acidimicrobiia_bacterium	aminotransferase-class-V-fold-PLP-dependent-enzyme-[Acidimicrobiia-bacterium]	2080302
MXZ76739.1	cysteine-desulfurase	GCA_009837345.1	394	Bacteria>Terrabacteria_group>Actinobacteria>Acidimicrobiia>unclassified_Acidimicrobiia	Acidimicrobiia_bacterium	cysteine-desulfurase-[Acidimicrobiia-bacterium]	2080302
MYH96489.1	cysteine-desulfurase	GCA_009843985.1	394	Bacteria>Terrabacteria_group>Actinobacteria>Acidimicrobiia>unclassified_Acidimicrobiia	Acidimicrobiia_bacterium	cysteine-desulfurase-[Acidimicrobiia-bacterium]	2080302
WP_211469983.1	cysteine-desulfurase	GCF_018283645.1	376	Bacteria>Terrabacteria_group>Actinobacteria>Actinomycetia>Catenulisporales	Actinocrinis_puniceicyclus	aminotransferase-class-V-fold-PLP-dependent-enzyme-[Actinocrinis-puniceicyclus]	977794
WP_194897795.1	cysteine-desulfurase	GCF_015356865.1	376	Bacteria>Terrabacteria_group>Actinobacteria>Actinomycetia>Catenulisporales	Catenulispora_pinisilvae	aminotransferase-class-V-fold-PLP-dependent-enzyme-[Catenulispora-pinisilvae]	2705253
WP_206474992.1	cysteine-desulfurase	GCF_017168155.1	394	Bacteria>Terrabacteria_group>Actinobacteria>Actinomycetia>Corynebacteriales	Dietzia_sp._KRD202	cysteine-desulfurase-DndA-[Dietzia-sp.-KRD202]	2729732
WP_188330213.1	cysteine-desulfurase	GCF_014622905.1	381	Bacteria>Terrabacteria_group>Actinobacteria>Actinomycetia>Corynebacteriales	Gordonia_jinghuaiqii	cysteine-desulfurase-[Gordonia-jinghuaiqii]	2758710
WP_005207127.1	cysteine-desulfurase	GCF_000248055.1	396	Bacteria>Terrabacteria_group>Actinobacteria>Actinomycetia>Corynebacteriales	Gordonia_sputi	cysteine-desulfurase-DndA-[Gordonia-sputi]	36823
MBN4927165.1	cysteine-desulfurase	GCA_017152025.1	396	Bacteria>Terrabacteria_group>Actinobacteria>Actinomycetia>Corynebacteriales	Hoyosella_rhizosphaerae	cysteine-desulfurase-DndA-[Hoyosella-rhizosphaerae]	1755582
WP_188669870.1	cysteine-desulfurase	GCF_014643175.1	396	Bacteria>Terrabacteria_group>Actinobacteria>Actinomycetia>Corynebacteriales	Hoyosella_rhizosphaerae	cysteine-desulfurase-DndA-[Hoyosella-rhizosphaerae]	1755582
GGC53499.1	cysteine-desulfurase	GCA_014643175.1	333	Bacteria>Terrabacteria_group>Actinobacteria>Actinomycetia>Corynebacteriales	Hoyosella_rhizosphaerae	putative-L-cysteine-desulfurase-[Hoyosella-rhizosphaerae]	1755582
WP_076217388.1	cysteine-desulfurase	GCF_900132695.1	399	Bacteria>Terrabacteria_group>Actinobacteria>Actinomycetia>Corynebacteriales	Mycobacteriaceae	MULTISPECIES:-cysteine-desulfurase-DndA-[Mycobacteriaceae]	1762
OBJ57439.1	cysteine-desulfurase	GCA_001673165.1	397	Bacteria>Terrabacteria_group>Actinobacteria>Actinomycetia>Corynebacteriales	Mycobacterium_asiaticum	cysteine-desulfurase-DndA-[Mycobacterium-asiaticum]	1790
OBK28876.1	cysteine-desulfurase	GCA_001673365.1	399	Bacteria>Terrabacteria_group>Actinobacteria>Actinomycetia>Corynebacteriales	Mycobacterium_asiaticum	cysteine-desulfurase-DndA-[Mycobacterium-asiaticum]	1790
WP_065143378.1	cysteine-desulfurase	-	399	Bacteria>Terrabacteria_group>Actinobacteria>Actinomycetia>Corynebacteriales	Mycobacterium_asiaticum	cysteine-desulfurase-DndA-[Mycobacterium-asiaticum]	1790
WP_065154999.1	cysteine-desulfurase	GCF_001673165.1	397	Bacteria>Terrabacteria_group>Actinobacteria>Actinomycetia>Corynebacteriales	Mycobacterium_asiaticum	cysteine-desulfurase-DndA-[Mycobacterium-asiaticum]	1790
WP_070946311.1	cysteine-desulfurase	GCF_022348025.1	395	Bacteria>Terrabacteria_group>Actinobacteria>Actinomycetia>Corynebacteriales	Mycobacterium	cysteine-desulfurase-DndA-[Mycobacterium-syngnathidarum]	1763
WP_065162944.1	cysteine-desulfurase	GCF_001673475.1	400	Bacteria>Terrabacteria_group>Actinobacteria>Actinomycetia>Corynebacteriales	Mycobacterium_gordonae	cysteine-desulfurase-DndA-[Mycobacterium-gordonae]	1778
ODQ99145.1	cysteine-desulfurase	GCA_001722345.1	397	Bacteria>Terrabacteria_group>Actinobacteria>Actinomycetia>Corynebacteriales	Mycobacterium_intermedium	cysteine-desulfurase-DndA-[Mycobacterium-intermedium]	28445
OPE50550.1	cysteine-desulfurase	GCA_002024965.1	397	Bacteria>Terrabacteria_group>Actinobacteria>Actinomycetia>Corynebacteriales	Mycobacterium_intermedium	cysteine-desulfurase-DndA-[Mycobacterium-intermedium]	28445
ORB05495.1	cysteine-desulfurase	GCA_002086275.1	397	Bacteria>Terrabacteria_group>Actinobacteria>Actinomycetia>Corynebacteriales	Mycobacterium_intermedium	cysteine-desulfurase-DndA-[Mycobacterium-intermedium]	28445
WP_069420680.1	cysteine-desulfurase	GCF_001722345.1	397	Bacteria>Terrabacteria_group>Actinobacteria>Actinomycetia>Corynebacteriales	Mycobacterium_intermedium	cysteine-desulfurase-DndA-[Mycobacterium-intermedium]	28445
PBA28937.1	cysteine-desulfurase	GCA_002292535.1	394	Bacteria>Terrabacteria_group>Actinobacteria>Actinomycetia>Corynebacteriales	Mycobacterium_intracellulare	cysteine-desulfurase-DndA-[Mycobacterium-intracellulare]	1767
WP_095763945.1	cysteine-desulfurase	GCF_002292535.1	394	Bacteria>Terrabacteria_group>Actinobacteria>Actinomycetia>Corynebacteriales	Mycobacterium_intracellulare	cysteine-desulfurase-DndA-[Mycobacterium-intracellulare]	1767
WP_065480663.1	cysteine-desulfurase	GCF_001686745.1	395	Bacteria>Terrabacteria_group>Actinobacteria>Actinomycetia>Corynebacteriales	Mycobacterium_malmoense	cysteine-desulfurase-DndA-[Mycobacterium-malmoense]	1780
WP_007170949.1	cysteine-desulfurase	GCF_000164135.1	396	Bacteria>Terrabacteria_group>Actinobacteria>Actinomycetia>Corynebacteriales	Mycobacterium	MULTISPECIES:-cysteine-desulfurase-DndA-[Mycobacterium]	1763
WP_085101888.1	cysteine-desulfurase	GCF_002102295.1	395	Bacteria>Terrabacteria_group>Actinobacteria>Actinomycetia>Corynebacteriales	Mycobacterium_paraense	cysteine-desulfurase-DndA-[Mycobacterium-paraense]	767916
EFG78013.1	cysteine-desulfurase	GCA_000164135.1	396	Bacteria>Terrabacteria_group>Actinobacteria>Actinomycetia>Corynebacteriales	Mycobacterium_parascrofulaceum_ATCC_BAA-614	cysteine-desulfurase-DndA-[Mycobacterium-parascrofulaceum-ATCC-BAA-614]	525368
OCB14188.1	cysteine-desulfurase	GCA_001686675.1	398	Bacteria>Terrabacteria_group>Actinobacteria>Actinomycetia>Corynebacteriales	Mycobacterium_porcinum	cysteine-desulfurase-DndA-[Mycobacterium-porcinum]	39693
WP_085256599.1	cysteine-desulfurase	GCF_010729105.1	399	Bacteria>Terrabacteria_group>Actinobacteria>Actinomycetia>Corynebacteriales	Mycobacterium_saskatchewanense	cysteine-desulfurase-DndA-[Mycobacterium-saskatchewanense]	220927
ORB63763.1	cysteine-desulfurase	GCA_002086755.1	405	Bacteria>Terrabacteria_group>Actinobacteria>Actinomycetia>Corynebacteriales	Mycobacterium_shinjukuense	cysteine-desulfurase-DndA-[Mycobacterium-shinjukuense]	398694
WP_083051373.1	cysteine-desulfurase	GCF_010730055.1	405	Bacteria>Terrabacteria_group>Actinobacteria>Actinomycetia>Corynebacteriales	Mycobacterium_shinjukuense	cysteine-desulfurase-DndA-[Mycobacterium-shinjukuense]	398694
BBX74264.1	cysteine-desulfurase	GCA_010730055.1	405	Bacteria>Terrabacteria_group>Actinobacteria>Actinomycetia>Corynebacteriales	Mycobacterium_shinjukuense	putative-L-cysteine-desulfurase-[Mycobacterium-shinjukuense]	398694
WP_102417213.1	cysteine-desulfurase	GCF_900240975.1	395	Bacteria>Terrabacteria_group>Actinobacteria>Actinomycetia>Corynebacteriales	Mycobacterium_sp._4858	cysteine-desulfurase-DndA-[Mycobacterium-sp.-4858]	2057185
WP_082976991.1	cysteine-desulfurase	GCF_001667185.1	395	Bacteria>Terrabacteria_group>Actinobacteria>Actinomycetia>Corynebacteriales	Mycobacterium_sp._852002-50816_SCH5313054-b	cysteine-desulfurase-DndA-[Mycobacterium-sp.-852002-50816_SCH5313054-b]	1834092
OBC03468.1	cysteine-desulfurase	GCA_001665685.1	396	Bacteria>Terrabacteria_group>Actinobacteria>Actinomycetia>Corynebacteriales	Mycobacterium_sp._852013-50091_SCH5140682	cysteine-desulfurase-DndA-[Mycobacterium-sp.-852013-50091_SCH5140682]	1834109
MBO0879189.1	cysteine-desulfurase	GCA_017354565.1	414	Bacteria>Terrabacteria_group>Actinobacteria>Actinomycetia>Corynebacteriales	Mycobacterium_sp.	cysteine-desulfurase-DndA-[Mycobacterium-sp.]	1785
WP_068128995.1	cysteine-desulfurase	GCF_001672815.1	398	Bacteria>Terrabacteria_group>Actinobacteria>Actinomycetia>Corynebacteriales	Mycobacterium_sp._E796	cysteine-desulfurase-DndA-[Mycobacterium-sp.-E796]	1834151
WP_116374763.1	cysteine-desulfurase	GCF_003402475.1	399	Bacteria>Terrabacteria_group>Actinobacteria>Actinomycetia>Corynebacteriales	Mycobacterium_sp._MFM001	cysteine-desulfurase-DndA-[Mycobacterium-sp.-MFM001]	2049453
GBE65145.1	cysteine-desulfurase	GCA_003402475.1	399	Bacteria>Terrabacteria_group>Actinobacteria>Actinomycetia>Corynebacteriales	Mycobacterium_sp._MFM001	putative-L-cysteine-desulfurase-[Mycobacterium-sp.-MFM001]	2049453
OMC01068.1	cysteine-desulfurase	GCA_001953975.1	396	Bacteria>Terrabacteria_group>Actinobacteria>Actinomycetia>Corynebacteriales	Mycobacterium_sp._NS-7484	cysteine-desulfurase-DndA-[Mycobacterium-sp.-NS-7484]	1834161
WP_076240015.1	cysteine-desulfurase	GCF_001953975.1	396	Bacteria>Terrabacteria_group>Actinobacteria>Actinomycetia>Corynebacteriales	Mycobacterium_sp._NS-7484	cysteine-desulfurase-DndA-[Mycobacterium-sp.-NS-7484]	1834161
OHT93331.1	cysteine-desulfurase	GCA_001853525.1	395	Bacteria>Terrabacteria_group>Actinobacteria>Actinomycetia>Corynebacteriales	Mycobacterium_syngnathidarum	cysteine-desulfurase-DndA-[Mycobacterium-syngnathidarum]	1908205

Accession	Annotation	GCA	Length	Phyletic.spread	Strain.name	Defline	Species.Taxid
OLT88102.1	cysteine-desulfurase	GCA_001942625.1	395	Bacteria>Terrabacteria_group>Actinobacteria>Actinomycetia>Corynebacteriales	Mycobacterium_syngnathidarum	cysteine-desulfurase-DndA-[Mycobacterium-syngnathidarum]	1908205
EIV42599.1	cysteine-desulfurase	GCA_000270585.1	399	Bacteria>Terrabacteria_group>Actinobacteria>Actinomycetia>Corynebacteriales	Mycobacteroides_abscessus_3A-0122-S	cysteine-desulfurase-DndA-[Mycobacteroides-abscessus-3A-0122-S]	1001716
EIU50872.1	cysteine-desulfurase	GCA_000270985.1	399	Bacteria>Terrabacteria_group>Actinobacteria>Actinomycetia>Corynebacteriales	Mycobacteroides_abscessus_6G-0125-R	cysteine-desulfurase-DndA-[Mycobacteroides-abscessus-6G-0125-R]	1001740
EIU48487.1	cysteine-desulfurase	GCA_000270865.1	399	Bacteria>Terrabacteria_group>Actinobacteria>Actinomycetia>Corynebacteriales	Mycobacteroides_abscessus_6G-0125-S	cysteine-desulfurase-DndA-[Mycobacteroides-abscessus-6G-0125-S]	1001741
WP_234821668.1	cysteine-desulfurase	GCF_002800315.1	356	Bacteria>Terrabacteria_group>Actinobacteria>Actinomycetia>Corynebacteriales	Mycobacteroides_abscessus	aminotransferase-class-V-fold-PLP-dependent-enzyme-[Mycobacteroides-abscessus]	36809
WP_236746516.1	cysteine-desulfurase	GCF_900137085.1	356	Bacteria>Terrabacteria_group>Actinobacteria>Actinomycetia>Corynebacteriales	Mycobacteroides_abscessus	aminotransferase-class-V-fold-PLP-dependent-enzyme-[Mycobacteroides-abscessus]	36809
PVA41051.1	cysteine-desulfurase	GCA_003071765.1	399	Bacteria>Terrabacteria_group>Actinobacteria>Actinomycetia>Corynebacteriales	Mycobacteroides_abscessus	cysteine-desulfurase-DndA-[Mycobacteroides-abscessus]	36809
PVB01786.1	cysteine-desulfurase	GCA_003072295.1	399	Bacteria>Terrabacteria_group>Actinobacteria>Actinomycetia>Corynebacteriales	Mycobacteroides_abscessus	cysteine-desulfurase-DndA-[Mycobacteroides-abscessus]	36809
WP_005109917.1	cysteine-desulfurase	GCF_000271185.1	399	Bacteria>Terrabacteria_group>Actinobacteria>Actinomycetia>Corynebacteriales	Mycobacteroides_abscessus	cysteine-desulfurase-DndA-[Mycobacteroides-abscessus]	36809
WP_100459280.1	cysteine-desulfurase	GCF_024389745.1	399	Bacteria>Terrabacteria_group>Actinobacteria>Actinomycetia>Corynebacteriales	Mycobacteroides_abscessus	cysteine-desulfurase-DndA-[Mycobacteroides-abscessus]	36809
WP_100515503.1	cysteine-desulfurase	GCF_002801525.1	398	Bacteria>Terrabacteria_group>Actinobacteria>Actinomycetia>Corynebacteriales	Mycobacteroides_abscessus	cysteine-desulfurase-DndA-[Mycobacteroides-abscessus]	36809
WP_119617425.1	cysteine-desulfurase	GCF_003582965.1	398	Bacteria>Terrabacteria_group>Actinobacteria>Actinomycetia>Corynebacteriales	Mycobacteroides_abscessus	cysteine-desulfurase-DndA-[Mycobacteroides-abscessus]	36809
WP_232690004.1	cysteine-desulfurase	GCF_000069185.1	361	Bacteria>Terrabacteria_group>Actinobacteria>Actinomycetia>Corynebacteriales	Mycobacteroides_abscessus	cysteine-desulfurase-DndA-[Mycobacteroides-abscessus]	36809
SHV98040.1	cysteine-desulfurase	GCA_900134205.1	409	Bacteria>Terrabacteria_group>Actinobacteria>Actinomycetia>Corynebacteriales	Mycobacteroides_abscessus_subsp._abscessus	Putative-L-cysteine-desulfurase-[Mycobacteroides-abscessus-subsp.-abscessus]	1185650
SHW98780.1	cysteine-desulfurase	GCA_900134175.1	409	Bacteria>Terrabacteria_group>Actinobacteria>Actinomycetia>Corynebacteriales	Mycobacteroides_abscessus_subsp._abscessus	Putative-L-cysteine-desulfurase-[Mycobacteroides-abscessus-subsp.-abscessus]	1185650
SIB24729.1	cysteine-desulfurase	GCA_900135845.1	409	Bacteria>Terrabacteria_group>Actinobacteria>Actinomycetia>Corynebacteriales	Mycobacteroides_abscessus_subsp._abscessus	Putative-L-cysteine-desulfurase-[Mycobacteroides-abscessus-subsp.-abscessus]	1185650
SIJ89839.1	cysteine-desulfurase	GCA_900140055.1	409	Bacteria>Terrabacteria_group>Actinobacteria>Actinomycetia>Corynebacteriales	Mycobacteroides_abscessus_subsp._bolletii	Putative-L-cysteine-desulfurase-[Mycobacteroides-abscessus-subsp.-bolletii]	319705
SLF81070.1	cysteine-desulfurase	GCA_900140175.1	399	Bacteria>Terrabacteria_group>Actinobacteria>Actinomycetia>Corynebacteriales	Mycobacteroides_abscessus_subsp._bolletii	Putative-L-cysteine-desulfurase-[Mycobacteroides-abscessus-subsp.-bolletii]	319705
MBN7507513.1	cysteine-desulfurase	GCA_017175855.1	399	Bacteria>Terrabacteria_group>Actinobacteria>Actinomycetia>Corynebacteriales	Mycobacteroides_abscessus_subsp._massiliense	cysteine-desulfurase-DndA-[Mycobacteroides-abscessus-subsp.-massiliense]	1962118
SKE06871.1	cysteine-desulfurase	GCA_900130575.1	409	Bacteria>Terrabacteria_group>Actinobacteria>Actinomycetia>Corynebacteriales	Mycobacteroides_abscessus_subsp._massiliense	Putative-L-cysteine-desulfurase-[Mycobacteroides-abscessus-subsp.-massiliense]	1962118
MBF9328417.1	cysteine-desulfurase	GCA_015700855.1	398	Bacteria>Terrabacteria_group>Actinobacteria>Actinomycetia>Corynebacteriales	Mycobacteroides_chelonae	cysteine-desulfurase-DndA-[Mycobacteroides-chelonae]	1774
MBF9435255.1	cysteine-desulfurase	GCA_015700815.1	399	Bacteria>Terrabacteria_group>Actinobacteria>Actinomycetia>Corynebacteriales	Mycobacteroides_chelonae	cysteine-desulfurase-DndA-[Mycobacteroides-chelonae]	1774
WP_074354977.1	cysteine-desulfurase	GCF_900134175.1	399	Bacteria>Terrabacteria_group>Actinobacteria>Actinomycetia>Corynebacteriales	Mycobacteroides	MULTISPECIES:-cysteine-desulfurase-DndA-[Mycobacteroides]	670516
WP_100479987.1	cysteine-desulfurase	GCF_015700835.1	398	Bacteria>Terrabacteria_group>Actinobacteria>Actinomycetia>Corynebacteriales	Mycobacteroides	MULTISPECIES:-cysteine-desulfurase-DndA-[Mycobacteroides]	670516
ORA45173.1	cysteine-desulfurase	GCA_002086175.1	397	Bacteria>Terrabacteria_group>Actinobacteria>Actinomycetia>Corynebacteriales	Mycolicibacterium_celeriflavum	cysteine-desulfurase-DndA-[Mycolicibacterium-celeriflavum]	1249101
WP_083005189.1	cysteine-desulfurase	GCF_010731795.1	397	Bacteria>Terrabacteria_group>Actinobacteria>Actinomycetia>Corynebacteriales	Mycolicibacterium_celeriflavum	cysteine-desulfurase-DndA-[Mycolicibacterium-celeriflavum]	1249101
BBY44171.1	cysteine-desulfurase	GCA_010731795.1	354	Bacteria>Terrabacteria_group>Actinobacteria>Actinomycetia>Corynebacteriales	Mycolicibacterium_celeriflavum	putative-L-cysteine-desulfurase-[Mycolicibacterium-celeriflavum]	1249101
WP_065063066.1	cysteine-desulfurase	GCF_001673265.1	399	Bacteria>Terrabacteria_group>Actinobacteria>Actinomycetia>Corynebacteriales	Mycolicibacterium_conceptionense	cysteine-desulfurase-DndA-[Mycolicibacterium-conceptionense]	451644
WP_234785748.1	cysteine-desulfurase	GCF_001665755.1	369	Bacteria>Terrabacteria_group>Actinobacteria>Actinomycetia>Corynebacteriales	Mycolicibacterium_elephantis	aminotransferase-class-V-fold-PLP-dependent-enzyme-[Mycolicibacterium-elephantis]	81858
NOR03465.1	cysteine-desulfurase	GCA_013138535.1	395	Bacteria>Terrabacteria_group>Actinobacteria>Actinomycetia>Corynebacteriales	Mycolicibacterium_fortuitum	cysteine-desulfurase-DndA-[Mycolicibacterium-fortuitum]	1766
WP_171508663.1	cysteine-desulfurase	GCF_013138535.1	395	Bacteria>Terrabacteria_group>Actinobacteria>Actinomycetia>Corynebacteriales	Mycolicibacterium_fortuitum	cysteine-desulfurase-DndA-[Mycolicibacterium-fortuitum]	1766
OBJ37419.1	cysteine-desulfurase	GCA_001668705.1	393	Bacteria>Terrabacteria_group>Actinobacteria>Actinomycetia>Corynebacteriales	Mycolicibacterium_mucogenicum	cysteine-desulfurase-DndA-[Mycolicibacterium-mucogenicum]	56689
WP_064985058.1	cysteine-desulfurase	GCF_001668705.1	393	Bacteria>Terrabacteria_group>Actinobacteria>Actinomycetia>Corynebacteriales	Mycolicibacterium_mucogenicum	cysteine-desulfurase-DndA-[Mycolicibacterium-mucogenicum]	56689
WP_003887892.1	cysteine-desulfurase	GCF_015333575.1	397	Bacteria>Terrabacteria_group>Actinobacteria>Actinomycetia>Corynebacteriales	Mycolicibacterium_phlei	cysteine-desulfurase-DndA-[Mycolicibacterium-phlei]	1771
MBF4191401.1	cysteine-desulfurase	GCA_015333575.1	397	Bacteria>Terrabacteria_group>Actinobacteria>Actinomycetia>Corynebacteriales	Mycolicibacterium_phlei	cysteine-desulfurase-[Mycolicibacterium-phlei]	1771
EID14666.1	cysteine-desulfurase	GCA_000257725.1	397	Bacteria>Terrabacteria_group>Actinobacteria>Actinomycetia>Corynebacteriales	Mycolicibacterium_phlei_RIVM601174	cysteine-desulfurase-[Mycolicibacterium-phlei-RIVM601174]	1150599
EJZ12215.1	cysteine-desulfurase	GCA_000295825.1	364	Bacteria>Terrabacteria_group>Actinobacteria>Actinomycetia>Corynebacteriales	Mycolicibacterium_vaccae_ATCC_25954	cysteine-desulfurase-[Mycolicibacterium-vaccae-ATCC-25954]	1194972
WP_040540736.1	cysteine-desulfurase	GCF_001552715.1	400	Bacteria>Terrabacteria_group>Actinobacteria>Actinomycetia>Corynebacteriales	Mycolicibacterium_vaccae	cysteine-desulfurase-DndA-[Mycolicibacterium-vaccae]	1810
WP_085138250.1	cysteine-desulfurase	GCF_002101775.1	405	Bacteria>Terrabacteria_group>Actinobacteria>Actinomycetia>Corynebacteriales	Mycolicibacter_nonchromogenicus	cysteine-desulfurase-DndA-[Mycolicibacter-nonchromogenicus]	1782
RRR45073.1	cysteine-desulfurase	GCA_003933725.1	399	Bacteria>Terrabacteria_group>Actinobacteria>Actinomycetia>Corynebacteriales	Mycolicibacter_terrae	cysteine-desulfurase-DndA-[Mycolicibacter-terrae]	1788
WP_125225137.1	cysteine-desulfurase	GCF_003933725.1	399	Bacteria>Terrabacteria_group>Actinobacteria>Actinomycetia>Corynebacteriales	Mycolicibacter_terrae	cysteine-desulfurase-DndA-[Mycolicibacter-terrae]	1788
WP_215918491.1	cysteine-desulfurase	GCF_018860155.1	378	Bacteria>Terrabacteria_group>Actinobacteria>Actinomycetia>Corynebacteriales	Nocardia_albiluteola	cysteine-desulfurase-DndA-[Nocardia-sp.-NEAU-G5]	2842303
WP_084823887.1	cysteine-desulfurase	GCF_001612785.1	396	Bacteria>Terrabacteria_group>Actinobacteria>Actinomycetia>Corynebacteriales	Nocardia_beijingensis	cysteine-desulfurase-DndA-[Nocardia-beijingensis]	95162
AFT98752.1	cysteine-desulfurase	GCA_000250675.3	328	Bacteria>Terrabacteria_group>Actinobacteria>Actinomycetia>Corynebacteriales	Nocardia_brasiliensis_ATCC_700358	cysteine-desulfurase-[Nocardia-brasiliensis-ATCC-700358]	1133849
WP_041562403.1	cysteine-desulfurase	GCF_000250675.2	377	Bacteria>Terrabacteria_group>Actinobacteria>Actinomycetia>Corynebacteriales	Nocardia_brasiliensis	cysteine-desulfurase-DndA-[Nocardia-brasiliensis]	37326
WP_195138097.1	cysteine-desulfurase	GCF_015478325.1	378	Bacteria>Terrabacteria_group>Actinobacteria>Actinomycetia>Corynebacteriales	Nocardia_brasiliensis	cysteine-desulfurase-DndA-[Nocardia-brasiliensis]	37326
TLG01812.1	cysteine-desulfurase	GCA_005863295.1	396	Bacteria>Terrabacteria_group>Actinobacteria>Actinomycetia>Corynebacteriales	Nocardia_cyriacigeorgica	cysteine-desulfurase-DndA-[Nocardia-cyriacigeorgica]	135487
WP_138458012.1	cysteine-desulfurase	GCF_005863295.1	396	Bacteria>Terrabacteria_group>Actinobacteria>Actinomycetia>Corynebacteriales	Nocardia_cyriacigeorgica	cysteine-desulfurase-DndA-[Nocardia-cyriacigeorgica]	135487
MBM4694349.1	cysteine-desulfurase	GCA_016878985.1	396	Bacteria>Terrabacteria_group>Actinobacteria>Actinomycetia>Corynebacteriales	Rhodococcus_equi	cysteine-desulfurase-DndA-[Rhodococcus-hoagii]	43767
NKT42201.1	cysteine-desulfurase	GCA_012394405.1	396	Bacteria>Terrabacteria_group>Actinobacteria>Actinomycetia>Corynebacteriales	Rhodococcus_equi	cysteine-desulfurase-DndA-[Rhodococcus-hoagii]	43767
ORL10309.1	cysteine-desulfurase	GCA_002094295.1	396	Bacteria>Terrabacteria_group>Actinobacteria>Actinomycetia>Corynebacteriales	Rhodococcus_equi	cysteine-desulfurase-DndA-[Rhodococcus-hoagii]	43767
WP_084956894.1	cysteine-desulfurase	GCF_002094295.1	396	Bacteria>Terrabacteria_group>Actinobacteria>Actinomycetia>Corynebacteriales	Rhodococcus_equi	cysteine-desulfurase-DndA-[Rhodococcus-hoagii]	43767
WP_238773916.1	cysteine-desulfurase	GCF_022271335.1	396	Bacteria>Terrabacteria_group>Actinobacteria>Actinomycetia>Corynebacteriales	Rhodococcus_equi	cysteine-desulfurase-DndA-[Rhodococcus-hoagii]	43767
QIP41495.1	cysteine-desulfurase	GCA_011611865.1	362	Bacteria>Terrabacteria_group>Actinobacteria>Actinomycetia>Corynebacteriales	Rhodococcus_erythropolis	cysteine-desulfurase-DndA-[Rhodococcus-erythropolis]	1833
WP_166502468.1	cysteine-desulfurase	GCF_011611865.1	397	Bacteria>Terrabacteria_group>Actinobacteria>Actinomycetia>Corynebacteriales	Rhodococcus_erythropolis_group	cysteine-desulfurase-DndA-[Rhodococcus-erythropolis]	2840174
MBY4111464.1	cysteine-desulfurase	GCA_019782405.1	390	Bacteria>Terrabacteria_group>Actinobacteria>Actinomycetia>Corynebacteriales	Rhodococcus_fascians	aminotransferase-class-V-fold-PLP-dependent-enzyme-[Rhodococcus-fascians]	1828
MBY4130975.1	cysteine-desulfurase	GCA_019782385.1	396	Bacteria>Terrabacteria_group>Actinobacteria>Actinomycetia>Corynebacteriales	Rhodococcus_fascians	cysteine-desulfurase-DndA-[Rhodococcus-fascians]	1828
WP_032393097.1	cysteine-desulfurase	GCF_000760775.1	396	Bacteria>Terrabacteria_group>Actinobacteria>Actinomycetia>Corynebacteriales	Rhodococcus_fascians	cysteine-desulfurase-DndA-[Rhodococcus-fascians]	1828
MBS3695242.1	cysteine-desulfurase	GCA_018310395.1	383	Bacteria>Terrabacteria_group>Actinobacteria>Actinomycetia>Corynebacteriales	Rhodococcus_qingshengii	cysteine-desulfurase-DndA-[Rhodococcus-qingshengii]	334542
QEM26339.1	cysteine-desulfurase	GCA_008306195.1	397	Bacteria>Terrabacteria_group>Actinobacteria>Actinomycetia>Corynebacteriales	Rhodococcus_qingshengii	cysteine-desulfurase-DndA-[Rhodococcus-qingshengii]	334542
WP_212634020.1	cysteine-desulfurase	GCF_018310395.1	383	Bacteria>Terrabacteria_group>Actinobacteria>Actinomycetia>Corynebacteriales	Rhodococcus_qingshengii	cysteine-desulfurase-DndA-[Rhodococcus-qingshengii]	334542
WP_064065957.1	cysteine-desulfurase	GCF_001894945.1	382	Bacteria>Terrabacteria_group>Actinobacteria>Actinomycetia>Corynebacteriales	Rhodococcus_ruber	cysteine-desulfurase-DndA-[Rhodococcus-ruber]	1830
WP_094713455.1	cysteine-desulfurase	GCF_002258765.1	397	Bacteria>Terrabacteria_group>Actinobacteria>Actinomycetia>Corynebacteriales	Rhodococcus_sp._06-418-1B	cysteine-desulfurase-DndA-[Rhodococcus-sp.-06-418-1B]	2022515
WP_229567704.1	cysteine-desulfurase	GCF_020885655.1	382	Bacteria>Terrabacteria_group>Actinobacteria>Actinomycetia>Corynebacteriales	Rhodococcus_sp._RDE2	cysteine-desulfurase-DndA-[Rhodococcus-sp.-RDE2]	2885078
BDB59762.1	cysteine-desulfurase	GCA_020885655.1	382	Bacteria>Terrabacteria_group>Actinobacteria>Actinomycetia>Corynebacteriales	Rhodococcus_sp._RDE2	putative-L-cysteine-desulfurase-[Rhodococcus-sp.-RDE2]	2885078
WP_094643706.1	cysteine-desulfurase	GCF_000813105.1	396	Bacteria>Terrabacteria_group>Actinobacteria>Actinomycetia>Corynebacteriales	unclassified_Rhodococcus	MULTISPECIES:-cysteine-desulfurase-DndA-[unclassified-Rhodococcus]	192944
WP_206442855.1	cysteine-desulfurase	GCF_900634765.1	388	Bacteria>Terrabacteria_group>Actinobacteria>Actinomycetia>Frankiales	Candidatus_Frankia_californiensis	aminotransferase-class-V-fold-PLP-dependent-enzyme-[Candidatus-Frankia-datiscae]	1839754
WP_165938366.1	cysteine-desulfurase	-	382	Bacteria>Terrabacteria_group>Actinobacteria>Actinomycetia>Frankiales	Frankia_sp._BMG5.11	cysteine-desulfurase-[Frankia-sp.-BMG5.11]	222540
WP_198955477.1	cysteine-desulfurase	GCF_001983215.1	365	Bacteria>Terrabacteria_group>Actinobacteria>Actinomycetia>Frankiales	Frankia_sp._CcI49	aminotransferase-class-V-fold-PLP-dependent-enzyme-[Frankia-sp.-CcI49]	1745382
WP_066070497.1	cysteine-desulfurase	GCF_001636565.1	401	Bacteria>Terrabacteria_group>Actinobacteria>Actinomycetia>Frankiales	Frankia_sp._EI5c	cysteine-desulfurase-DndA-[Frankia-sp.-EI5c]	683316
WP_091115458.1	cysteine-desulfurase	GCF_900115505.1	378	Bacteria>Terrabacteria_group>Actinobacteria>Actinomycetia>Geodermatophilales	Geodermatophilus_dictyosporus	cysteine-desulfurase-DndA-[Geodermatophilus-dictyosporus]	1523247
SFP89467.1	cysteine-desulfurase	GCA_900115505.1	378	Bacteria>Terrabacteria_group>Actinobacteria>Actinomycetia>Geodermatophilales	Geodermatophilus_dictyosporus	cysteine-desulfurase-[Geodermatophilus-dictyosporus]	1523247
WP_110005875.1	cysteine-desulfurase	GCF_003182485.1	378	Bacteria>Terrabacteria_group>Actinobacteria>Actinomycetia>Geodermatophilales	Geodermatophilus_normandii	cysteine-desulfurase-DndA-[Geodermatophilus-normandii]	1137989
WP_208983287.1	cysteine-desulfurase	GCF_900143215.1	396	Bacteria>Terrabacteria_group>Actinobacteria>Actinomycetia>Geodermatophilales	Geodermatophilus_obscurus	cysteine-desulfurase-[Geodermatophilus-obscurus]	1861
WP_097207109.1	cysteine-desulfurase	GCF_014191795.1	401	Bacteria>Terrabacteria_group>Actinobacteria>Actinomycetia>Geodermatophilales	Geodermatophilus_sabuli	cysteine-desulfurase-DndA-[Geodermatophilus-sabuli]	1564158
WP_046773079.1	cysteine-desulfurase	GCF_001005145.1	397	Bacteria>Terrabacteria_group>Actinobacteria>Actinomycetia>Jiangellales	Jiangella_alkaliphila	cysteine-desulfurase-DndA-[Jiangella-alkaliphila]	419479
MBK8078527.1	cysteine-desulfurase	GCA_016713225.1	404	Bacteria>Terrabacteria_group>Actinobacteria>Actinomycetia>Kineosporiales	Kineosporiaceae_bacterium	cysteine-desulfurase-DndA-[Kineosporiaceae-bacterium]	2487580
MCD5341433.1	cysteine-desulfurase	GCA_021129155.1	366	Bacteria>Terrabacteria_group>Actinobacteria>Actinomycetia>Micrococcales	Arthrobacter_sp._AK04	aminotransferase-class-V-fold-PLP-dependent-enzyme-[Arthrobacter-sp.-AK04]	2900048
TJY69503.1	cysteine-desulfurase	GCA_005047635.1	378	Bacteria>Terrabacteria_group>Actinobacteria>Actinomycetia>Micrococcales	Arthrobacter_sp._CAU_1506	cysteine-desulfurase-DndA-[Arthrobacter-sp.-CAU-1506]	2560052
WP_205754473.1	cysteine-desulfurase	GCF_005047635.1	395	Bacteria>Terrabacteria_group>Actinobacteria>Actinomycetia>Micrococcales	Arthrobacter_sp._CAU_1506	cysteine-desulfurase-DndA-[Arthrobacter-sp.-CAU-1506]	2560052
QYF91070.1	cysteine-desulfurase	GCA_019443425.1	376	Bacteria>Terrabacteria_group>Actinobacteria>Actinomycetia>Micrococcales	Arthrobacter_sp._PAMC25284	aminotransferase-class-V-fold-PLP-dependent-enzyme-[Arthrobacter-sp.-PAMC25284]	2861279
WP_219886707.1	cysteine-desulfurase	GCF_019443425.1	376	Bacteria>Terrabacteria_group>Actinobacteria>Actinomycetia>Micrococcales	Arthrobacter_sp._PAMC25284	aminotransferase-class-V-fold-PLP-dependent-enzyme-[Arthrobacter-sp.-PAMC25284]	2861279
MBD1591588.1	cysteine-desulfurase	GCA_014694505.1	397	Bacteria>Terrabacteria_group>Actinobacteria>Actinomycetia>Micrococcales	Arthrobacter_sp._S1_S22	aminotransferase-class-V-fold-PLP-dependent-enzyme-[Arthrobacter-sp.-S1_S22]	2720222
WP_224094978.1	cysteine-desulfurase	GCF_019977255.1	396	Bacteria>Terrabacteria_group>Actinobacteria>Actinomycetia>Micrococcales	Arthrobacter_sp._StoSoilB13	cysteine-desulfurase-DndA-[Arthrobacter-sp.-StoSoilB13]	2830993
BCW52044.1	cysteine-desulfurase	GCA_019977255.1	396	Bacteria>Terrabacteria_group>Actinobacteria>Actinomycetia>Micrococcales	Arthrobacter_sp._StoSoilB13	putative-L-cysteine-desulfurase-[Arthrobacter-sp.-StoSoilB13]	2830993
WP_082101556.1	cysteine-desulfurase	GCF_000971435.1	398	Bacteria>Terrabacteria_group>Actinobacteria>Actinomycetia>Micrococcales	Demequina_rhizosphaerae	aminotransferase-class-V-fold-PLP-dependent-enzyme-[Demequina-rhizosphaerae]	1638985
MBB4140424.1	cysteine-desulfurase	GCA_014197265.1	387	Bacteria>Terrabacteria_group>Actinobacteria>Actinomycetia>Micrococcales	Microbacterium_invictum	cysteine-desulfurase-[Microbacterium-invictum]	515415
WP_183499955.1	cysteine-desulfurase	GCF_015278225.1	387	Bacteria>Terrabacteria_group>Actinobacteria>Actinomycetia>Micrococcales	Microbacterium	MULTISPECIES:-aminotransferase-class-V-fold-PLP-dependent-enzyme-[Microbacterium]	33882
HAS32870.1	cysteine-desulfurase	GCA_003483835.1	388	Bacteria>Terrabacteria_group>Actinobacteria>Actinomycetia>Micrococcales	Microbacterium_sp.	cysteine-desulfurase-DndA-[Microbacterium-sp.]	51671
MAM53481.1	cysteine-desulfurase	GCA_002695205.1	388	Bacteria>Terrabacteria_group>Actinobacteria>Actinomycetia>Micrococcales	Microbacterium_sp.	cysteine-desulfurase-DndA-[Microbacterium-sp.]	51671
WP_242133993.1	cysteine-desulfurase	GCF_022668885.1	397	Bacteria>Terrabacteria_group>Actinobacteria>Actinomycetia>Micrococcales	Sediminivirga_luteola	cysteine-desulfurase-DndA-[Sediminivirga-luteola]	1774748
WP_184840261.1	cysteine-desulfurase	GCF_014204945.1	403	Bacteria>Terrabacteria_group>Actinobacteria>Actinomycetia>Micromonosporales	Allocatelliglobosispora_scoriae	aminotransferase-class-V-fold-PLP-dependent-enzyme-[Allocatelliglobosispora-scoriae]	643052
MBB5871899.1	cysteine-desulfurase	GCA_014204945.1	378	Bacteria>Terrabacteria_group>Actinobacteria>Actinomycetia>Micromonosporales	Allocatelliglobosispora_scoriae	cysteine-desulfurase-[Allocatelliglobosispora-scoriae]	643052
WP_212827436.1	cysteine-desulfurase	GCF_018327705.1	390	Bacteria>Terrabacteria_group>Actinobacteria>Actinomycetia>Micromonosporales	Catellatospora_sp._TT07R-123	aminotransferase-class-V-fold-PLP-dependent-enzyme-[Catellatospora-sp.-TT07R-123]	2733863
GHJ45928.1	cysteine-desulfurase	GCA_018327705.1	384	Bacteria>Terrabacteria_group>Actinobacteria>Actinomycetia>Micromonosporales	Catellatospora_sp._TT07R-123	cysteine-desulfurase-[Catellatospora-sp.-TT07R-123]	2733863
WP_242796393.1	cysteine-desulfurase	GCF_022747945.1	402	Bacteria>Terrabacteria_group>Actinobacteria>Actinomycetia>Micromonosporales	Micromonospora_sp._R77	aminotransferase-class-V-fold-PLP-dependent-enzyme-[Micromonospora-sp.-R77]	2925836
WP_229688160.1	cysteine-desulfurase	GCF_014645775.1	362	Bacteria>Terrabacteria_group>Actinobacteria>Actinomycetia>Micromonosporales	Micromonospora_yangpuensis	aminotransferase-class-V-fold-PLP-dependent-enzyme-[Micromonospora-yangpuensis]	683228
WP_090474565.1	cysteine-desulfurase	GCF_900104535.1	398	Bacteria>Terrabacteria_group>Actinobacteria>Actinomycetia>Nakamurellales	Nakamurella_panacisegetis	cysteine-desulfurase-DndA-[Nakamurella-panacisegetis]	1090615
SDO34701.1	cysteine-desulfurase	GCA_900104535.1	398	Bacteria>Terrabacteria_group>Actinobacteria>Actinomycetia>Nakamurellales	Nakamurella_panacisegetis	cysteine-desulfurase-[Nakamurella-panacisegetis]	1090615
WP_231930170.1	cysteine-desulfurase	GCF_900105065.1	360	Bacteria>Terrabacteria_group>Actinobacteria>Actinomycetia>Propionibacteriales	Friedmanniella_luteola	aminotransferase-class-V-fold-PLP-dependent-enzyme-[Friedmanniella-luteola]	546871
SDT34885.1	cysteine-desulfurase	GCA_900105065.1	381	Bacteria>Terrabacteria_group>Actinobacteria>Actinomycetia>Propionibacteriales	Friedmanniella_luteola	cysteine-desulfurase-[Friedmanniella-luteola]	546871
WP_209692272.1	cysteine-desulfurase	GCF_017876295.1	395	Bacteria>Terrabacteria_group>Actinobacteria>Actinomycetia>Propionibacteriales	Kribbella_aluminosa	cysteine-desulfurase-DndA-[Kribbella-aluminosa]	416017
QNE18710.1	cysteine-desulfurase	GCA_014217565.1	395	Bacteria>Terrabacteria_group>Actinobacteria>Actinomycetia>Propionibacteriales	Kribbella_qitaiheensis	cysteine-desulfurase-DndA-[Kribbella-qitaiheensis]	1544730
WP_185448009.1	cysteine-desulfurase	GCF_014217565.1	395	Bacteria>Terrabacteria_group>Actinobacteria>Actinomycetia>Propionibacteriales	Kribbella_qitaiheensis	cysteine-desulfurase-DndA-[Kribbella-qitaiheensis]	1544730
MCB0909929.1	cysteine-desulfurase	GCA_020438665.1	92	Bacteria>Terrabacteria_group>Actinobacteria>Actinomycetia>Propionibacteriales	Nocardioidaceae_bacterium	aminotransferase-class-V-fold-PLP-dependent-enzyme-[Nocardioidaceae-bacterium]	1871072
WP_114423340.1	cysteine-desulfurase	GCF_003333285.1	397	Bacteria>Terrabacteria_group>Actinobacteria>Actinomycetia>Propionibacteriales	Nocardioides_houyundeii	cysteine-desulfurase-DndA-[Nocardioides-houyundeii]	2045452
WP_136596311.1	cysteine-desulfurase	GCF_004919105.1	395	Bacteria>Terrabacteria_group>Actinobacteria>Actinomycetia>Propionibacteriales	Nocardioides_sp._GY_10113	cysteine-desulfurase-DndA-[Nocardioides-sp.-GY-10113]	2569761
MCC2591821.1	cysteine-desulfurase	GCA_020687685.1	397	Bacteria>Terrabacteria_group>Actinobacteria>Actinomycetia>Propionibacteriales	Tessaracoccus_sp._OS52	cysteine-desulfurase-DndA-[Tessaracoccus-sp.-OS52]	2886691
WP_227734865.1	cysteine-desulfurase	GCF_020687685.1	397	Bacteria>Terrabacteria_group>Actinobacteria>Actinomycetia>Propionibacteriales	Tessaracoccus_sp._OS52	cysteine-desulfurase-DndA-[Tessaracoccus-sp.-OS52]	2886691
RLK59600.1	cysteine-desulfurase	GCA_003663795.1	381	Bacteria>Terrabacteria_group>Actinobacteria>Actinomycetia>Pseudonocardiales	Actinokineospora_cianjurensis	cysteine-desulfurase-[Actinokineospora-cianjurensis]	585224
WP_211346405.1	cysteine-desulfurase	GCF_003663795.1	381	Bacteria>Terrabacteria_group>Actinobacteria>Actinomycetia>Pseudonocardiales	Actinokineospora_cianjurensis	cysteine-desulfurase-DndA-[Actinokineospora-cianjurensis]	585224
WP_219994298.1	cysteine-desulfurase	GCF_003182415.1	384	Bacteria>Terrabacteria_group>Actinobacteria>Actinomycetia>Pseudonocardiales	Actinokineospora_spheciospongiae	cysteine-desulfurase-DndA-[Actinokineospora-mzabensis]	909613
TDV36688.1	cysteine-desulfurase	GCA_004364325.1	381	Bacteria>Terrabacteria_group>Actinobacteria>Actinomycetia>Pseudonocardiales	Actinophytocola_oryzae	cysteine-desulfurase-[Actinophytocola-oryzae]	502181
WP_208298058.1	cysteine-desulfurase	GCF_004364325.1	381	Bacteria>Terrabacteria_group>Actinobacteria>Actinomycetia>Pseudonocardiales	Actinophytocola_oryzae	cysteine-desulfurase-DndA-[Actinophytocola-oryzae]	502181
WP_191306963.1	cysteine-desulfurase	GCF_014654205.1	383	Bacteria>Terrabacteria_group>Actinobacteria>Actinomycetia>Pseudonocardiales	Amycolatopsis_bullii	cysteine-desulfurase-DndA-[Amycolatopsis-bullii]	941987
GHF98388.1	cysteine-desulfurase	GCA_014654205.1	383	Bacteria>Terrabacteria_group>Actinobacteria>Actinomycetia>Pseudonocardiales	Amycolatopsis_bullii	putative-L-cysteine-desulfurase-[Amycolatopsis-bullii]	941987
WP_038522309.1	cysteine-desulfurase	GCF_000732925.1	383	Bacteria>Terrabacteria_group>Actinobacteria>Actinomycetia>Pseudonocardiales	Amycolatopsis_japonica	cysteine-desulfurase-DndA-[Amycolatopsis-japonica]	208439
AIG73613.1	cysteine-desulfurase	GCA_000732925.1	383	Bacteria>Terrabacteria_group>Actinobacteria>Actinomycetia>Pseudonocardiales	Amycolatopsis_japonica	Hypothetical-protein-AJAP_03435-[Amycolatopsis-japonica]	208439
WP_033288306.1	cysteine-desulfurase	GCF_000717335.1	383	Bacteria>Terrabacteria_group>Actinobacteria>Actinomycetia>Pseudonocardiales	Amycolatopsis_jejuensis	cysteine-desulfurase-DndA-[Amycolatopsis-jejuensis]	330084
WP_037364626.1	cysteine-desulfurase	GCF_000411975.1	383	Bacteria>Terrabacteria_group>Actinobacteria>Actinomycetia>Pseudonocardiales	Amycolatopsis_orientalis	cysteine-desulfurase-DndA-[Amycolatopsis-orientalis]	31958
OXM65310.1	cysteine-desulfurase	GCA_002234595.1	396	Bacteria>Terrabacteria_group>Actinobacteria>Actinomycetia>Pseudonocardiales	Amycolatopsis_vastitatis	cysteine-desulfurase-DndA-[Amycolatopsis-vastitatis]	1905142
WP_093949715.1	cysteine-desulfurase	GCF_002234595.1	396	Bacteria>Terrabacteria_group>Actinobacteria>Actinomycetia>Pseudonocardiales	Amycolatopsis_vastitatis	cysteine-desulfurase-DndA-[Amycolatopsis-vastitatis]	1905142
WP_034274441.1	cysteine-desulfurase	GCF_000504245.1	396	Bacteria>Terrabacteria_group>Actinobacteria>Actinomycetia>Pseudonocardiales	Haloechinothrix_halophila	cysteine-desulfurase-DndA-[Haloechinothrix-halophila]	1069073
WP_218596979.1	cysteine-desulfurase	GCF_019263625.1	381	Bacteria>Terrabacteria_group>Actinobacteria>Actinomycetia>Pseudonocardiales	Pseudonocardia_oceani	aminotransferase-class-V-fold-PLP-dependent-enzyme-[Pseudonocardia-oceani]	2792013
WP_226355082.1	cysteine-desulfurase	GCF_020496245.1	396	Bacteria>Terrabacteria_group>Actinobacteria>Actinomycetia>Pseudonocardiales	Pseudonocardia_sp._ICBG601	cysteine-desulfurase-DndA-[Pseudonocardia-sp.-ICBG601]	2846759

Accession	Annotation	GCA	Length	Phyletic.spread	Strain.name	Defline	Species.Taxid
WP_221456333.1	cysteine-desulfurase	-	364	Bacteria>Terrabacteria_group>Actinobacteria>Actinomycetia>Pseudonocardiales	Saccharothrix_tamanrassetensis	cysteine-desulfurase-DndA-[Saccharothrix-tamanrassetensis]	1051531
MBB5957417.1	cysteine-desulfurase	GCA_014203665.1	364	Bacteria>Terrabacteria_group>Actinobacteria>Actinomycetia>Pseudonocardiales	Saccharothrix_tamanrassetensis	cysteine-desulfurase-[Saccharothrix-tamanrassetensis]	1051531
WP_020555790.1	cysteine-desulfurase	GCF_000372745.1	377	Bacteria>Terrabacteria_group>Actinobacteria>Actinomycetia>Streptomycetales	Embleya_scabrispora	MULTISPECIES:-cysteine-desulfurase-DndA-[Streptomycetaceae]	159449
MCC9309120.1	cysteine-desulfurase	GCA_020907985.1	377	Bacteria>Terrabacteria_group>Actinobacteria>Actinomycetia>Streptomycetales	Kitasatospora_humi	cysteine-desulfurase-DndA-[Kitasatospora-sp.-RB6PN24]	2893891
WP_230052433.1	cysteine-desulfurase	-	377	Bacteria>Terrabacteria_group>Actinobacteria>Actinomycetia>Streptomycetales	Kitasatospora_humi	cysteine-desulfurase-DndA-[Kitasatospora-sp.-RB6PN24]	2893891
WP_073818552.1	cysteine-desulfurase	GCF_001905465.1	377	Bacteria>Terrabacteria_group>Actinobacteria>Actinomycetia>Streptomycetales	Kitasatospora_sp._CB01950	cysteine-desulfurase-DndA-[Kitasatospora-sp.-CB01950]	1703930
AXI78540.1	cysteine-desulfurase	GCA_003258605.2	381	Bacteria>Terrabacteria_group>Actinobacteria>Actinomycetia>Streptomycetales	Streptacidiphilus_bronchialis	cysteine-desulfurase-DndA-[Streptacidiphilus-bronchialis]	2126346
WP_111488672.1	cysteine-desulfurase	GCF_003258605.2	381	Bacteria>Terrabacteria_group>Actinobacteria>Actinomycetia>Streptomycetales	Streptacidiphilus_bronchialis	cysteine-desulfurase-DndA-[Streptacidiphilus-bronchialis]	2126346
WP_236060473.1	cysteine-desulfurase	GCF_016918855.1	359	Bacteria>Terrabacteria_group>Actinobacteria>Actinomycetia>Streptomycetales	Streptomyces_acididurans	cysteine-desulfurase-DndA-[Streptomyces-acididurans]	2784346
MPY33408.1	cysteine-desulfurase	GCA_009377175.1	384	Bacteria>Terrabacteria_group>Actinobacteria>Actinomycetia>Streptomycetales	Streptomyces_adustus	cysteine-desulfurase-DndA-[Streptomyces-adustus]	1609272
WP_238426831.1	cysteine-desulfurase	GCF_009377175.1	377	Bacteria>Terrabacteria_group>Actinobacteria>Actinomycetia>Streptomycetales	Streptomyces_adustus	cysteine-desulfurase-DndA-[Streptomyces-adustus]	1609272
WP_086562777.1	cysteine-desulfurase	-	390	Bacteria>Terrabacteria_group>Actinobacteria>Actinomycetia>Streptomycetales	Streptomyces_africanus	cysteine-desulfurase-DndA-[Streptomyces-africanus]	231024
WP_093838494.1	cysteine-desulfurase	GCF_900112355.1	378	Bacteria>Terrabacteria_group>Actinobacteria>Actinomycetia>Streptomycetales	Streptomyces_aidingensis	cysteine-desulfurase-DndA-[Streptomyces-aidingensis]	910347
RZE12947.1	cysteine-desulfurase	GCA_004195995.1	390	Bacteria>Terrabacteria_group>Actinobacteria>Actinomycetia>Streptomycetales	Streptomyces_albidoflavus	cysteine-desulfurase-DndA-[Streptomyces-albidoflavus]	1886
RZE59663.1	cysteine-desulfurase	GCA_004195845.1	390	Bacteria>Terrabacteria_group>Actinobacteria>Actinomycetia>Streptomycetales	Streptomyces_albidoflavus	cysteine-desulfurase-DndA-[Streptomyces-albidoflavus]	1886
WP_129834392.1	cysteine-desulfurase	-	390	Bacteria>Terrabacteria_group>Actinobacteria>Actinomycetia>Streptomycetales	Streptomyces_albidoflavus	cysteine-desulfurase-DndA-[Streptomyces-albidoflavus]	1886
WP_233656940.1	cysteine-desulfurase	GCF_021366495.1	384	Bacteria>Terrabacteria_group>Actinobacteria>Actinomycetia>Streptomycetales	Streptomyces_albulus	cysteine-desulfurase-DndA-[Streptomyces-albulus]	68570
KUN57075.1	cysteine-desulfurase	GCA_001514135.1	381	Bacteria>Terrabacteria_group>Actinobacteria>Actinomycetia>Streptomycetales	Streptomyces_avermitilis	cysteine-desulfurase-DndA-[Streptomyces-avermitilis]	33903
WP_010984365.1	cysteine-desulfurase	GCF_000009765.2	384	Bacteria>Terrabacteria_group>Actinobacteria>Actinomycetia>Streptomycetales	Streptomyces_avermitilis	cysteine-desulfurase-DndA-[Streptomyces-avermitilis]	33903
GDY77078.1	cysteine-desulfurase	-	153	Bacteria>Terrabacteria_group>Actinobacteria>Actinomycetia>Streptomycetales	Streptomyces_avermitilis	hypothetical-protein-SAV31267_065630-[Streptomyces-avermitilis]	33903
GDY77079.1	cysteine-desulfurase	-	249	Bacteria>Terrabacteria_group>Actinobacteria>Actinomycetia>Streptomycetales	Streptomyces_avermitilis	hypothetical-protein-SAV31267_065640-[Streptomyces-avermitilis]	33903
BAC70644.1	cysteine-desulfurase	GCA_000009765.2	384	Bacteria>Terrabacteria_group>Actinobacteria>Actinomycetia>Streptomycetales	Streptomyces_avermitilis_MA-4680_=_NBRC_14893	putative-DNA-modification-(L-cysteine-desulfurase:-pyridoxal-phosphate-dependent-[Streptomyces-avermitilis-MA-4680-=-NBRC-14893]	227882
BBJ50772.1	cysteine-desulfurase	-	384	Bacteria>Terrabacteria_group>Actinobacteria>Actinomycetia>Streptomycetales	Streptomyces_avermitilis	putative-L-cysteine-desulfurase-[Streptomyces-avermitilis]	33903
GDY62794.1	cysteine-desulfurase	-	384	Bacteria>Terrabacteria_group>Actinobacteria>Actinomycetia>Streptomycetales	Streptomyces_avermitilis	putative-L-cysteine-desulfurase-[Streptomyces-avermitilis]	33903
GDY85992.1	cysteine-desulfurase	-	384	Bacteria>Terrabacteria_group>Actinobacteria>Actinomycetia>Streptomycetales	Streptomyces_avermitilis	putative-L-cysteine-desulfurase-[Streptomyces-avermitilis]	33903
GAP46076.1	cysteine-desulfurase	GCA_001270025.1	353	Bacteria>Terrabacteria_group>Actinobacteria>Actinomycetia>Streptomycetales	Streptomyces_azureus	cysteine-desulfurase-DndA-[Streptomyces-azureus]	146537
WP_201785055.1	cysteine-desulfurase	GCF_001270025.1	379	Bacteria>Terrabacteria_group>Actinobacteria>Actinomycetia>Streptomycetales	Streptomyces_azureus	cysteine-desulfurase-DndA-[Streptomyces-azureus]	146537
RAN21678.1	cysteine-desulfurase	GCA_003263885.1	384	Bacteria>Terrabacteria_group>Actinobacteria>Actinomycetia>Streptomycetales	Streptomyces_badius	cysteine-desulfurase-DndA-[Streptomyces-badius]	1941
RAN29613.1	cysteine-desulfurase	GCA_003263925.1	384	Bacteria>Terrabacteria_group>Actinobacteria>Actinomycetia>Streptomycetales	Streptomyces_badius	cysteine-desulfurase-DndA-[Streptomyces-badius]	1941
WP_101255411.1	cysteine-desulfurase	-	390	Bacteria>Terrabacteria_group>Actinobacteria>Actinomycetia>Streptomycetales	Streptomyces_barkulensis	cysteine-desulfurase-DndA-[Streptomyces-barkulensis]	1257026
KOT38872.1	cysteine-desulfurase	GCA_001279005.1	377	Bacteria>Terrabacteria_group>Actinobacteria>Actinomycetia>Streptomycetales	Streptomyces_caelestis	cysteine-desulfurase-[Streptomyces-caelestis]	36816
WP_166027232.1	cysteine-desulfurase	-	380	Bacteria>Terrabacteria_group>Actinobacteria>Actinomycetia>Streptomycetales	Streptomyces_chilikensis	cysteine-desulfurase-DndA-[Streptomyces-chilikensis]	1194079
WP_138906430.1	cysteine-desulfurase	GCF_014650855.1	379	Bacteria>Terrabacteria_group>Actinobacteria>Actinomycetia>Streptomycetales	Streptomyces_chryseus	cysteine-desulfurase-DndA-[Streptomyces-chryseus]	68186
WP_191207876.1	cysteine-desulfurase	GCF_014779715.1	380	Bacteria>Terrabacteria_group>Actinobacteria>Actinomycetia>Streptomycetales	Streptomyces_chumphonensis	cysteine-desulfurase-DndA-[Streptomyces-chumphonensis]	1214925
WP_190186383.1	cysteine-desulfurase	GCF_014650775.1	380	Bacteria>Terrabacteria_group>Actinobacteria>Actinomycetia>Streptomycetales	Streptomyces_cirratus	cysteine-desulfurase-DndA-[Streptomyces-cirratus]	68187
KUM79257.1	cysteine-desulfurase	GCA_001513975.1	381	Bacteria>Terrabacteria_group>Actinobacteria>Actinomycetia>Streptomycetales	Streptomyces_curacoi	cysteine-desulfurase-DndA-[Streptomyces-curacoi]	146536
WP_062146839.1	cysteine-desulfurase	GCF_001513975.1	381	Bacteria>Terrabacteria_group>Actinobacteria>Actinomycetia>Streptomycetales	Streptomyces_curacoi	cysteine-desulfurase-DndA-[Streptomyces-curacoi]	146536
WP_213089403.1	cysteine-desulfurase	GCF_014647675.1	383	Bacteria>Terrabacteria_group>Actinobacteria>Actinomycetia>Streptomycetales	Streptomyces_flaveus	cysteine-desulfurase-DndA-[Streptomyces-flaveus]	66370
GGL03221.1	cysteine-desulfurase	GCA_014647675.1	380	Bacteria>Terrabacteria_group>Actinobacteria>Actinomycetia>Streptomycetales	Streptomyces_flaveus	putative-L-cysteine-desulfurase-[Streptomyces-flaveus]	66370
AWL90515.1	cysteine-desulfurase	GCA_003147545.1	384	Bacteria>Terrabacteria_group>Actinobacteria>Actinomycetia>Streptomycetales	Streptomyces_globisporus	cysteine-desulfurase-DndA-[Streptomyces-globisporus]	1908
WP_230534739.1	cysteine-desulfurase	GCF_014649595.1	384	Bacteria>Terrabacteria_group>Actinobacteria>Actinomycetia>Streptomycetales	Streptomyces_griseomycini	cysteine-desulfurase-DndA-[Streptomyces-griseomycini]	66895
PPA44459.1	cysteine-desulfurase	GCA_000932225.2	384	Bacteria>Terrabacteria_group>Actinobacteria>Actinomycetia>Streptomycetales	Streptomyces_griseus	cysteine-desulfurase-DndA-[Streptomyces-griseus]	1911
WP_234345425.1	cysteine-desulfurase	GCF_000717795.1	384	Bacteria>Terrabacteria_group>Actinobacteria>Actinomycetia>Streptomycetales	Streptomyces_griseus	cysteine-desulfurase-DndA-[Streptomyces-griseus]	1911
WP_229878417.1	cysteine-desulfurase	GCF_014649655.1	379	Bacteria>Terrabacteria_group>Actinobacteria>Actinomycetia>Streptomycetales	Streptomyces_humidus	cysteine-desulfurase-DndA-[Streptomyces-humidus]	52259
GGS15191.1	cysteine-desulfurase	GCA_014649655.1	379	Bacteria>Terrabacteria_group>Actinobacteria>Actinomycetia>Streptomycetales	Streptomyces_humidus	putative-L-cysteine-desulfurase-[Streptomyces-humidus]	52259
WP_199849948.1	cysteine-desulfurase	-	385	Bacteria>Terrabacteria_group>Actinobacteria>Actinomycetia>Streptomycetales	Streptomyces_hygroscopicus	cysteine-desulfurase-DndA-[Streptomyces-hygroscopicus]	1912
WP_209442528.1	cysteine-desulfurase	-	383	Bacteria>Terrabacteria_group>Actinobacteria>Actinomycetia>Streptomycetales	Streptomyces_iakyrus	cysteine-desulfurase-DndA-[Streptomyces-iakyrus]	68219
WP_153463766.1	cysteine-desulfurase	GCF_009569385.1	378	Bacteria>Terrabacteria_group>Actinobacteria>Actinomycetia>Streptomycetales	Streptomyces_kaniharaensis	cysteine-desulfurase-DndA-[Streptomyces-kaniharaensis]	212423
WP_079189021.1	cysteine-desulfurase	GCF_001906585.1	384	Bacteria>Terrabacteria_group>Actinobacteria>Actinomycetia>Streptomycetales	Streptomyces_kebangsaanensis	cysteine-desulfurase-DndA-[Streptomyces-kebangsaanensis]	864058
WP_148026206.1	cysteine-desulfurase	GCF_008016805.1	391	Bacteria>Terrabacteria_group>Actinobacteria>Actinomycetia>Streptomycetales	Streptomyces_lavendulae	cysteine-desulfurase-[Streptomyces-lavendulae]	1914
WP_079024898.1	cysteine-desulfurase	GCF_001047315.1	387	Bacteria>Terrabacteria_group>Actinobacteria>Actinomycetia>Streptomycetales	Streptomyces_leeuwenhoekii	cysteine-desulfurase-DndA-[Streptomyces-leeuwenhoekii]	1437453
AAZ29040.1	cysteine-desulfurase	-	380	Bacteria>Terrabacteria_group>Actinobacteria>Actinomycetia>Streptomycetales	Streptomyces_lividans_1326	putative-pyridoxal-phosphate-dependent-L-cysteine-desulfurylase-[Streptomyces-lividans-1326]	1200984
ABP49156.1	cysteine-desulfurase	-	397	Bacteria>Terrabacteria_group>Actinobacteria>Actinomycetia>Streptomycetales	Streptomyces_lividans	conserved-hypothetical-protein-[Streptomyces-lividans]	1916
WP_016327563.1	cysteine-desulfurase	GCF_000403665.1	381	Bacteria>Terrabacteria_group>Actinobacteria>Actinomycetia>Streptomycetales	Streptomyces	MULTISPECIES:-cysteine-desulfurase-DndA-[Streptomyces]	1883
WP_030834262.1	cysteine-desulfurase	GCF_000718375.1	384	Bacteria>Terrabacteria_group>Actinobacteria>Actinomycetia>Streptomycetales	Streptomyces	MULTISPECIES:-cysteine-desulfurase-DndA-[Streptomyces]	1883
WP_078955508.1	cysteine-desulfurase	GCF_003147545.1	387	Bacteria>Terrabacteria_group>Actinobacteria>Actinomycetia>Streptomycetales	Streptomyces	MULTISPECIES:-cysteine-desulfurase-DndA-[Streptomyces]	1883
WP_086809664.1	cysteine-desulfurase	GCF_002154555.1	381	Bacteria>Terrabacteria_group>Actinobacteria>Actinomycetia>Streptomycetales	Streptomyces_murinus	cysteine-desulfurase-DndA-[Streptomyces-murinus]	33900
WP_191884015.1	cysteine-desulfurase	GCF_014649815.1	380	Bacteria>Terrabacteria_group>Actinobacteria>Actinomycetia>Streptomycetales	Streptomyces_mutabilis	cysteine-desulfurase-DndA-[Streptomyces-mutabilis]	67332
WP_229832455.1	cysteine-desulfurase	GCF_014649015.1	384	Bacteria>Terrabacteria_group>Actinobacteria>Actinomycetia>Streptomycetales	Streptomyces_narbonensis	cysteine-desulfurase-DndA-[Streptomyces-narbonensis]	67333
GGV99949.1	cysteine-desulfurase	GCA_014649015.1	395	Bacteria>Terrabacteria_group>Actinobacteria>Actinomycetia>Streptomycetales	Streptomyces_narbonensis	putative-L-cysteine-desulfurase-[Streptomyces-narbonensis]	67333
WP_208838869.1	cysteine-desulfurase	GCF_014650375.1	383	Bacteria>Terrabacteria_group>Actinobacteria>Actinomycetia>Streptomycetales	Streptomyces_nitrosporeus	cysteine-desulfurase-DndA-[Streptomyces-nitrosporeus]	28894
MBC9717832.1	cysteine-desulfurase	GCA_014493765.1	380	Bacteria>Terrabacteria_group>Actinobacteria>Actinomycetia>Streptomycetales	Streptomyces_polyasparticus	cysteine-desulfurase-DndA-[Streptomyces-polyasparticus]	2767826
WP_187818319.1	cysteine-desulfurase	GCF_014493765.1	380	Bacteria>Terrabacteria_group>Actinobacteria>Actinomycetia>Streptomycetales	Streptomyces_polyasparticus	cysteine-desulfurase-DndA-[Streptomyces-polyasparticus]	2767826
WP_062712665.1	cysteine-desulfurase	GCF_001509475.1	380	Bacteria>Terrabacteria_group>Actinobacteria>Actinomycetia>Streptomycetales	Streptomyces_regalis	cysteine-desulfurase-DndA-[Streptomyces-regalis]	68262
WP_030647879.1	cysteine-desulfurase	GCF_000718675.1	384	Bacteria>Terrabacteria_group>Actinobacteria>Actinomycetia>Streptomycetales	Streptomyces_rimosus	cysteine-desulfurase-DndA-[Streptomyces-rimosus]	1927
WP_053802191.1	cysteine-desulfurase	GCF_001279345.1	380	Bacteria>Terrabacteria_group>Actinobacteria>Actinomycetia>Streptomycetales	Streptomyces_rimosus	cysteine-desulfurase-DndA-[Streptomyces-rimosus]	1927
WP_109002639.1	cysteine-desulfurase	GCF_003112535.1	384	Bacteria>Terrabacteria_group>Actinobacteria>Actinomycetia>Streptomycetales	Streptomyces_rishiriensis	cysteine-desulfurase-DndA-[Streptomyces-rishiriensis]	68264
WP_031225651.1	cysteine-desulfurase	-	381	Bacteria>Terrabacteria_group>Actinobacteria>Actinomycetia>Streptomycetales	Streptomyces_roseochromogenus	cysteine-desulfurase-DndA-[Streptomyces-roseochromogenus]	285450
WP_235481561.1	cysteine-desulfurase	GCF_021538775.1	384	Bacteria>Terrabacteria_group>Actinobacteria>Actinomycetia>Streptomycetales	Streptomyces_roseoverticillatus	cysteine-desulfurase-DndA-[Streptomyces-roseoverticillatus]	66429
WP_055699777.1	cysteine-desulfurase	GCF_001419745.1	381	Bacteria>Terrabacteria_group>Actinobacteria>Actinomycetia>Streptomycetales	Streptomyces_silaceus	cysteine-desulfurase-DndA-[Streptomyces-silaceus]	545123
WP_143626943.1	cysteine-desulfurase	GCF_007176755.1	384	Bacteria>Terrabacteria_group>Actinobacteria>Actinomycetia>Streptomycetales	Streptomyces_sp._1-11	cysteine-desulfurase-DndA-[Streptomyces-sp.-1-11]	2590549
WP_203232558.1	cysteine-desulfurase	GCF_000816025.1	383	Bacteria>Terrabacteria_group>Actinobacteria>Actinomycetia>Streptomycetales	Streptomyces_sp._769	cysteine-desulfurase-DndA-[Streptomyces-sp.-769]	1262452
WP_225051001.1	cysteine-desulfurase	GCF_020177475.1	391	Bacteria>Terrabacteria_group>Actinobacteria>Actinomycetia>Streptomycetales	Streptomyces_sp._7G	cysteine-desulfurase-[Streptomyces-sp.-7G]	2877241
MBV1949368.1	cysteine-desulfurase	GCA_019090105.1	389	Bacteria>Terrabacteria_group>Actinobacteria>Actinomycetia>Streptomycetales	Streptomyces_sp._BV129	cysteine-desulfurase-DndA-[Streptomyces-sp.-BV129]	2849671
MBV1949489.1	cysteine-desulfurase	GCA_019090105.1	389	Bacteria>Terrabacteria_group>Actinobacteria>Actinomycetia>Streptomycetales	Streptomyces_sp._BV129	cysteine-desulfurase-DndA-[Streptomyces-sp.-BV129]	2849671
WP_217464652.1	cysteine-desulfurase	GCF_019090105.1	389	Bacteria>Terrabacteria_group>Actinobacteria>Actinomycetia>Streptomycetales	Streptomyces_sp._BV129	cysteine-desulfurase-DndA-[Streptomyces-sp.-BV129]	2849671
WP_079190513.1	cysteine-desulfurase	-	390	Bacteria>Terrabacteria_group>Actinobacteria>Actinomycetia>Streptomycetales	Streptomyces_sp._CB02366	cysteine-desulfurase-DndA-[Streptomyces-sp.-CB02366]	1703935
OKJ38807.1	cysteine-desulfurase	GCA_001905905.1	381	Bacteria>Terrabacteria_group>Actinobacteria>Actinomycetia>Streptomycetales	Streptomyces_sp._CB02366	cysteine-desulfurase-[Streptomyces-sp.-CB02366]	1703935
WP_079192926.1	cysteine-desulfurase	-	390	Bacteria>Terrabacteria_group>Actinobacteria>Actinomycetia>Streptomycetales	Streptomyces_sp._CB02460	cysteine-desulfurase-DndA-[Streptomyces-sp.-CB02460]	1703941
OKJ74274.1	cysteine-desulfurase	GCA_001905705.1	381	Bacteria>Terrabacteria_group>Actinobacteria>Actinomycetia>Streptomycetales	Streptomyces_sp._CB02460	cysteine-desulfurase-[Streptomyces-sp.-CB02460]	1703941
WP_206603755.1	cysteine-desulfurase	-	383	Bacteria>Terrabacteria_group>Actinobacteria>Actinomycetia>Streptomycetales	Streptomyces_sp._CB03238	cysteine-desulfurase-DndA-[Streptomyces-sp.-CB03238]	1907777
MBD0676978.1	cysteine-desulfurase	GCA_014596515.1	381	Bacteria>Terrabacteria_group>Actinobacteria>Actinomycetia>Streptomycetales	Streptomyces_sp._CBMA156	cysteine-desulfurase-DndA-[Streptomyces-sp.-CBMA156]	1930280
WP_223245624.1	cysteine-desulfurase	GCF_014596515.1	397	Bacteria>Terrabacteria_group>Actinobacteria>Actinomycetia>Streptomycetales	Streptomyces_sp._CBMA156	cysteine-desulfurase-DndA-[Streptomyces-sp.-CBMA156]	1930280
WP_138914214.1	cysteine-desulfurase	GCF_005938145.1	391	Bacteria>Terrabacteria_group>Actinobacteria>Actinomycetia>Streptomycetales	Streptomyces_sp._DASNCL29	cysteine-desulfurase-[Streptomyces-sp.-DASNCL29]	2583819
MBJ6620037.1	cysteine-desulfurase	GCA_016432765.1	377	Bacteria>Terrabacteria_group>Actinobacteria>Actinomycetia>Streptomycetales	Streptomyces_sp._DHE17-7	cysteine-desulfurase-DndA-[Streptomyces-sp.-DHE17-7]	2759949
WP_128862882.1	cysteine-desulfurase	GCF_003353555.1	380	Bacteria>Terrabacteria_group>Actinobacteria>Actinomycetia>Streptomycetales	Streptomyces_sp._HB202	cysteine-desulfurase-DndA-[Streptomyces-sp.-HB202]	767398
OBQ50044.1	cysteine-desulfurase	GCA_001672315.1	381	Bacteria>Terrabacteria_group>Actinobacteria>Actinomycetia>Streptomycetales	Streptomyces_sp._H-KF8	cysteine-desulfurase-DndA-[Streptomyces-sp.-H-KF8]	1727216
WP_234373393.1	cysteine-desulfurase	GCF_001672315.1	384	Bacteria>Terrabacteria_group>Actinobacteria>Actinomycetia>Streptomycetales	Streptomyces_sp._H-KF8	cysteine-desulfurase-DndA-[Streptomyces-sp.-H-KF8]	1727216
MBA2808022.1	cysteine-desulfurase	GCA_004337435.2	381	Bacteria>Terrabacteria_group>Actinobacteria>Actinomycetia>Streptomycetales	Streptomyces_sp._KM273126	cysteine-desulfurase-DndA-[Streptomyces-sp.-KM273126]	2545247
WP_131558673.1	cysteine-desulfurase	GCF_004337435.2	390	Bacteria>Terrabacteria_group>Actinobacteria>Actinomycetia>Streptomycetales	Streptomyces_sp._KM273126	cysteine-desulfurase-DndA-[Streptomyces-sp.-KM273126]	2545247
WP_093866780.1	cysteine-desulfurase	GCF_900105275.1	384	Bacteria>Terrabacteria_group>Actinobacteria>Actinomycetia>Streptomycetales	Streptomyces_sp._KS_5	cysteine-desulfurase-DndA-[Streptomyces-sp.-KS_5]	1881018
SED52390.1	cysteine-desulfurase	GCA_900105275.1	381	Bacteria>Terrabacteria_group>Actinobacteria>Actinomycetia>Streptomycetales	Streptomyces_sp._KS_5	cysteine-desulfurase-[Streptomyces-sp.-KS_5]	1881018
MBK3562573.1	cysteine-desulfurase	GCA_016612625.1	383	Bacteria>Terrabacteria_group>Actinobacteria>Actinomycetia>Streptomycetales	Streptomyces_sp._MBT62	cysteine-desulfurase-DndA-[Streptomyces-sp.-MBT62]	2800410
WP_234467499.1	cysteine-desulfurase	GCF_016612625.1	376	Bacteria>Terrabacteria_group>Actinobacteria>Actinomycetia>Streptomycetales	Streptomyces_sp._MBT62	cysteine-desulfurase-DndA-[Streptomyces-sp.-MBT62]	2800410
QWB24036.1	cysteine-desulfurase	GCA_018604545.1	384	Bacteria>Terrabacteria_group>Actinobacteria>Actinomycetia>Streptomycetales	Streptomyces_sp._MG62	cysteine-desulfurase-DndA-[Streptomyces-sp.-MG62]	2838015
WP_215119892.1	cysteine-desulfurase	GCF_018604545.1	384	Bacteria>Terrabacteria_group>Actinobacteria>Actinomycetia>Streptomycetales	Streptomyces_sp._MG62	cysteine-desulfurase-DndA-[Streptomyces-sp.-MG62]	2838015
WP_077059980.1	cysteine-desulfurase	GCF_001984575.1	378	Bacteria>Terrabacteria_group>Actinobacteria>Actinomycetia>Streptomycetales	Streptomyces_sp._MP131-18	cysteine-desulfurase-DndA-[Streptomyces-sp.-MP131-18]	1857892
ONK12585.1	cysteine-desulfurase	GCA_001984575.1	378	Bacteria>Terrabacteria_group>Actinobacteria>Actinomycetia>Streptomycetales	Streptomyces_sp._MP131-18	Cysteine-desulfurase-[Streptomyces-sp.-MP131-18]	1857892
WP_240116960.1	cysteine-desulfurase	GCF_022414535.1	384	Bacteria>Terrabacteria_group>Actinobacteria>Actinomycetia>Streptomycetales	Streptomyces_sp._MUM_2J	cysteine-desulfurase-DndA-[Streptomyces-sp.-MUM-2J]	2791987
WP_208515213.1	cysteine-desulfurase	GCF_017592595.1	379	Bacteria>Terrabacteria_group>Actinobacteria>Actinomycetia>Streptomycetales	Streptomyces_sp._NEAU-YJ-81	cysteine-desulfurase-DndA-[Streptomyces-sp.-NEAU-YJ-81]	2820288
WP_232649362.1	cysteine-desulfurase	GCF_021261325.1	378	Bacteria>Terrabacteria_group>Actinobacteria>Actinomycetia>Streptomycetales	Streptomyces_sp._NR30	cysteine-desulfurase-DndA-[Streptomyces-sp.-NR30]	2886947
WP_037805983.1	cysteine-desulfurase	GCF_000720115.1	380	Bacteria>Terrabacteria_group>Actinobacteria>Actinomycetia>Streptomycetales	Streptomyces_sp._NRRL_F-2580	cysteine-desulfurase-DndA-[Streptomyces-sp.-NRRL-F-2580]	1463841
WP_031045851.1	cysteine-desulfurase	GCF_000721365.1	381	Bacteria>Terrabacteria_group>Actinobacteria>Actinomycetia>Streptomycetales	Streptomyces_sp._NRRL_F-5650	cysteine-desulfurase-DndA-[Streptomyces-sp.-NRRL-F-5650]	1463868
WP_199808700.1	cysteine-desulfurase	GCF_000721415.1	384	Bacteria>Terrabacteria_group>Actinobacteria>Actinomycetia>Streptomycetales	Streptomyces_sp._NRRL_F-5702	cysteine-desulfurase-DndA-[Streptomyces-sp.-NRRL-F-5702]	1463870
MBQ0827456.1	cysteine-desulfurase	GCA_018069565.1	390	Bacteria>Terrabacteria_group>Actinobacteria>Actinomycetia>Streptomycetales	Streptomyces_sp._RG38	cysteine-desulfurase-DndA-[Streptomyces-sp.-RG38]	2820809
WP_210871859.1	cysteine-desulfurase	GCF_018069565.1	390	Bacteria>Terrabacteria_group>Actinobacteria>Actinomycetia>Streptomycetales	Streptomyces_sp._RG38	cysteine-desulfurase-DndA-[Streptomyces-sp.-RG38]	2820809
QDN93535.1	cysteine-desulfurase	GCA_007113545.2	384	Bacteria>Terrabacteria_group>Actinobacteria>Actinomycetia>Streptomycetales	Streptomyces_sp._RLB3-6	cysteine-desulfurase-DndA-[Streptomyces-sp.-RLB3-6]	2594457
WP_143615022.1	cysteine-desulfurase	GCF_007113545.2	384	Bacteria>Terrabacteria_group>Actinobacteria>Actinomycetia>Streptomycetales	Streptomyces_sp._RLB3-6	cysteine-desulfurase-DndA-[Streptomyces-sp.-RLB3-6]	2594457
WP_125192589.1	cysteine-desulfurase	GCF_003933965.1	384	Bacteria>Terrabacteria_group>Actinobacteria>Actinomycetia>Streptomycetales	Streptomyces_sp._RP5T	cysteine-desulfurase-DndA-[Streptomyces-sp.-RP5T]	2490848
POX62400.1	cysteine-desulfurase	GCA_002920615.1	384	Bacteria>Terrabacteria_group>Actinobacteria>Actinomycetia>Streptomycetales	Streptomyces_sp._Ru62	cysteine-desulfurase-DndA-[Streptomyces-sp.-Ru62]	2080745
WP_103812575.1	cysteine-desulfurase	GCF_002920615.1	384	Bacteria>Terrabacteria_group>Actinobacteria>Actinomycetia>Streptomycetales	Streptomyces_sp._Ru62	cysteine-desulfurase-DndA-[Streptomyces-sp.-Ru62]	2080745
WP_237550167.1	cysteine-desulfurase	GCF_009865605.1	377	Bacteria>Terrabacteria_group>Actinobacteria>Actinomycetia>Streptomycetales	Streptomyces_sp._SID1034	cysteine-desulfurase-DndA-[Streptomyces-sp.-SID1034]	2690248
MYS98532.1	cysteine-desulfurase	GCA_009862865.1	381	Bacteria>Terrabacteria_group>Actinobacteria>Actinomycetia>Streptomycetales	Streptomyces_sp._SID5469	cysteine-desulfurase-DndA-[Streptomyces-sp.-SID5469]	2690296
WP_202497992.1	cysteine-desulfurase	GCF_009862865.1	377	Bacteria>Terrabacteria_group>Actinobacteria>Actinomycetia>Streptomycetales	Streptomyces_sp._SID5469	cysteine-desulfurase-DndA-[Streptomyces-sp.-SID5469]	2690296
MYS87887.1	cysteine-desulfurase	GCA_009862895.1	377	Bacteria>Terrabacteria_group>Actinobacteria>Actinomycetia>Streptomycetales	Streptomyces_sp._SID5474	cysteine-desulfurase-DndA-[Streptomyces-sp.-SID5474]	2690300
MYS52043.1	cysteine-desulfurase	GCA_009862745.1	381	Bacteria>Terrabacteria_group>Actinobacteria>Actinomycetia>Streptomycetales	Streptomyces_sp._SID6013	cysteine-desulfurase-DndA-[Streptomyces-sp.-SID6013]	2690317
WP_205526436.1	cysteine-desulfurase	GCF_009865025.1	377	Bacteria>Terrabacteria_group>Actinobacteria>Actinomycetia>Streptomycetales	Streptomyces_sp._SID8376	cysteine-desulfurase-DndA-[Streptomyces-sp.-SID8376]	2690356
WP_222126035.1	cysteine-desulfurase	GCF_006715925.1	384	Bacteria>Terrabacteria_group>Actinobacteria>Actinomycetia>Streptomycetales	Streptomyces_sp._SLBN-134	cysteine-desulfurase-DndA-[Streptomyces-sp.-SLBN-134]	2768456
WP_237404751.1	cysteine-desulfurase	GCF_016756395.1	386	Bacteria>Terrabacteria_group>Actinobacteria>Actinomycetia>Streptomycetales	Streptomyces_sp._SN-593	cysteine-desulfurase-DndA-[Streptomyces-sp.-SN-593]	659352
BBA98117.1	cysteine-desulfurase	GCA_016756395.1	383	Bacteria>Terrabacteria_group>Actinobacteria>Actinomycetia>Streptomycetales	Streptomyces_sp._SN-593	putative-cysteine-desulfurase-DndA-[Streptomyces-sp.-SN-593]	659352
PRH80007.1	cysteine-desulfurase	GCA_002982015.1	390	Bacteria>Terrabacteria_group>Actinobacteria>Actinomycetia>Streptomycetales	Streptomyces_sp._ST5x	cysteine-desulfurase-DndA-[Streptomyces-sp.-ST5x]	2100817
WP_105867838.1	cysteine-desulfurase	-	390	Bacteria>Terrabacteria_group>Actinobacteria>Actinomycetia>Streptomycetales	Streptomyces_sp._ST5x	cysteine-desulfurase-DndA-[Streptomyces-sp.-ST5x]	2100817
WP_098897939.1	cysteine-desulfurase	GCF_002551175.1	380	Bacteria>Terrabacteria_group>Actinobacteria>Actinomycetia>Streptomycetales	Streptomyces_sp._st77	cysteine-desulfurase-DndA-[Streptomyces-sp.-st77]	1828074
KAF3469784.1	cysteine-desulfurase	GCA_009852415.1	381	Bacteria>Terrabacteria_group>Actinobacteria>Actinomycetia>Streptomycetales	Streptomyces_sp._Tu_3180	cysteine-desulfurase-DndA-[Streptomyces-sp.-Tu-3180]	2682611
WP_159535780.1	cysteine-desulfurase	GCF_009852415.1	384	Bacteria>Terrabacteria_group>Actinobacteria>Actinomycetia>Streptomycetales	Streptomyces_sp._Tu_3180	cysteine-desulfurase-DndA-[Streptomyces-sp.-Tu-3180]	2682611

Accession	Annotation	GCA	Length	Phyletic.spread	Strain.name	Defline	Species.Taxid
NYV73211.1	cysteine-desulfurase	GCA_013421635.1	380	Bacteria>Terrabacteria_group>Actinobacteria>Actinomycetia>Streptomycetales	Streptomyces_sp._UH6	cysteine-desulfurase-DndA-[Streptomyces-sp.-UH6]	2748379
WP_218956490.1	cysteine-desulfurase	GCF_013421635.1	388	Bacteria>Terrabacteria_group>Actinobacteria>Actinomycetia>Streptomycetales	Streptomyces_sp._UH6	cysteine-desulfurase-DndA-[Streptomyces-sp.-UH6]	2748379
RSN63969.1	cysteine-desulfurase	GCA_003947455.1	381	Bacteria>Terrabacteria_group>Actinobacteria>Actinomycetia>Streptomycetales	Streptomyces_sp._WAC_04229	cysteine-desulfurase-DndA-[Streptomyces-sp.-WAC-04229]	2203206
WP_221763107.1	cysteine-desulfurase	GCF_003947455.1	384	Bacteria>Terrabacteria_group>Actinobacteria>Actinomycetia>Streptomycetales	Streptomyces_sp._WAC_04229	cysteine-desulfurase-DndA-[Streptomyces-sp.-WAC-04229]	2203206
WP_078903324.1	cysteine-desulfurase	GCF_000725125.1	384	Bacteria>Terrabacteria_group>Actinobacteria>Actinomycetia>Streptomycetales	Streptomyces_toyocaensis	cysteine-desulfurase-DndA-[Streptomyces-toyocaensis]	55952
WP_150185335.1	cysteine-desulfurase	GCF_008642335.1	381	Bacteria>Terrabacteria_group>Actinobacteria>Actinomycetia>Streptomycetales	Streptomyces_venezuelae	cysteine-desulfurase-DndA-[Streptomyces-venezuelae]	54571
WP_037816360.1	cysteine-desulfurase	GCF_009865125.1	380	Bacteria>Terrabacteria_group>Actinobacteria>Actinomycetia>Streptomycetales	unclassified_Streptomyces	MULTISPECIES:-cysteine-desulfurase-DndA-[unclassified-Streptomyces]	2593676
WP_088799083.1	cysteine-desulfurase	GCF_900105235.1	381	Bacteria>Terrabacteria_group>Actinobacteria>Actinomycetia>Streptomycetales	unclassified_Streptomyces	MULTISPECIES:-cysteine-desulfurase-DndA-[unclassified-Streptomyces]	2593676
WP_111582531.1	cysteine-desulfurase	GCF_003259585.1	380	Bacteria>Terrabacteria_group>Actinobacteria>Actinomycetia>Streptomycetales	unclassified_Streptomyces	MULTISPECIES:-cysteine-desulfurase-DndA-[unclassified-Streptomyces]	2593676
WP_131544677.1	cysteine-desulfurase	GCF_014281035.1	381	Bacteria>Terrabacteria_group>Actinobacteria>Actinomycetia>Streptomycetales	unclassified_Streptomyces	MULTISPECIES:-cysteine-desulfurase-DndA-[unclassified-Streptomyces]	2593676
WP_239161837.1	cysteine-desulfurase	GCF_016862995.1	402	Bacteria>Terrabacteria_group>Actinobacteria>Actinomycetia>Streptosporangiales	Acrocarpospora_phusangensis	cysteine-desulfurase-DndA-[Acrocarpospora-phusangensis]	1070424
GIH25824.1	cysteine-desulfurase	GCA_016862995.1	402	Bacteria>Terrabacteria_group>Actinobacteria>Actinomycetia>Streptosporangiales	Acrocarpospora_phusangensis	putative-L-cysteine-desulfurase-[Acrocarpospora-phusangensis]	1070424
WP_019635054.1	cysteine-desulfurase	GCF_000381885.1	381	Bacteria>Terrabacteria_group>Actinobacteria>Actinomycetia>Streptosporangiales	Actinomadura_atramentaria	cysteine-desulfurase-DndA-[Actinomadura-atramentaria]	1990
TYB49196.1	cysteine-desulfurase	GCA_008085895.1	383	Bacteria>Terrabacteria_group>Actinobacteria>Actinomycetia>Streptosporangiales	Actinomadura_chibensis	cysteine-desulfurase-DndA-[Actinomadura-chibensis]	392828
WP_067894028.1	cysteine-desulfurase	GCF_001552135.1	383	Bacteria>Terrabacteria_group>Actinobacteria>Actinomycetia>Streptosporangiales	Actinomadura_chibensis	cysteine-desulfurase-DndA-[Actinomadura-chibensis]	392828
TDD89526.1	cysteine-desulfurase	GCA_004349235.1	383	Bacteria>Terrabacteria_group>Actinobacteria>Actinomycetia>Streptosporangiales	Actinomadura_darangshiensis	cysteine-desulfurase-DndA-[Actinomadura-darangshiensis]	705336
WP_132194203.1	cysteine-desulfurase	GCF_004349235.1	383	Bacteria>Terrabacteria_group>Actinobacteria>Actinomycetia>Streptosporangiales	Actinomadura_darangshiensis	cysteine-desulfurase-DndA-[Actinomadura-darangshiensis]	705336
NDU74022.1	cysteine-desulfurase	GCA_010491635.1	383	Bacteria>Terrabacteria_group>Actinobacteria>Actinomycetia>Streptosporangiales	Actinomadura_lepetitiana	cysteine-desulfurase-DndA-[Actinomadura-lepetitiana]	2126066
NYD48455.1	cysteine-desulfurase	GCA_013409365.1	359	Bacteria>Terrabacteria_group>Actinobacteria>Actinomycetia>Streptosporangiales	Actinomadura_luteofluorescens	cysteine-desulfurase-[Actinomadura-luteofluorescens]	46163
WP_179845366.1	cysteine-desulfurase	GCF_013409365.1	383	Bacteria>Terrabacteria_group>Actinobacteria>Actinomycetia>Streptosporangiales	Actinomadura_luteofluorescens	cysteine-desulfurase-DndA-[Actinomadura-luteofluorescens]	46163
WP_121432300.1	cysteine-desulfurase	GCF_003634705.1	382	Bacteria>Terrabacteria_group>Actinobacteria>Actinomycetia>Streptosporangiales	Actinomadura_pelletieri	cysteine-desulfurase-DndA-[Actinomadura-pelletieri]	111805
MBC6456612.1	cysteine-desulfurase	GCA_014323665.1	383	Bacteria>Terrabacteria_group>Actinobacteria>Actinomycetia>Streptosporangiales	Actinomadura_sp._HBU206391	cysteine-desulfurase-DndA-[Actinomadura-sp.-HBU206391]	2731692
WP_187232368.1	cysteine-desulfurase	GCF_014323665.1	383	Bacteria>Terrabacteria_group>Actinobacteria>Actinomycetia>Streptosporangiales	Actinomadura_sp._HBU206391	cysteine-desulfurase-DndA-[Actinomadura-sp.-HBU206391]	2731692
WP_173097778.1	cysteine-desulfurase	-	383	Bacteria>Terrabacteria_group>Actinobacteria>Actinomycetia>Streptosporangiales	Actinomadura_verrucosospora	cysteine-desulfurase-DndA-[Actinomadura-verrucosospora]	46165
WP_055478297.1	cysteine-desulfurase	GCF_001417795.1	381	Bacteria>Terrabacteria_group>Actinobacteria>Actinomycetia>Streptosporangiales	Microbispora_sp._GMKU363	cysteine-desulfurase-DndA-[Microbispora-sp.-GMKU363]	718014
WP_182872765.1	cysteine-desulfurase	GCF_014161385.1	383	Bacteria>Terrabacteria_group>Actinobacteria>Actinomycetia>Streptosporangiales	Microbispora_sp._H10670	cysteine-desulfurase-DndA-[Microbispora-sp.-H10670]	2729108
RGA06202.1	cysteine-desulfurase	GCA_003260025.2	383	Bacteria>Terrabacteria_group>Actinobacteria>Actinomycetia>Streptosporangiales	Microbispora_triticiradicis	cysteine-desulfurase-DndA-[Microbispora-triticiradicis]	2200763
WP_066942356.1	cysteine-desulfurase	-	394	Bacteria>Terrabacteria_group>Actinobacteria>Actinomycetia>Streptosporangiales	Microtetraspora_fusca	cysteine-desulfurase-[Microtetraspora-fusca]	1997
QKW32074.1	cysteine-desulfurase	GCA_013364295.1	380	Bacteria>Terrabacteria_group>Actinobacteria>Actinomycetia>Streptosporangiales	Nocardiopsis_flavescens	cysteine-desulfurase-DndA-[Nocardiopsis-flavescens]	758803
WP_019607770.1	cysteine-desulfurase	GCF_000381685.1	377	Bacteria>Terrabacteria_group>Actinobacteria>Actinomycetia>Streptosporangiales	Nocardiopsis_sp._CNS-639	cysteine-desulfurase-DndA-[Nocardiopsis-sp.-CNS-639]	1169153
WP_028648084.1	cysteine-desulfurase	GCF_000515115.1	381	Bacteria>Terrabacteria_group>Actinobacteria>Actinomycetia>Streptosporangiales	Nocardiopsis_sp._CNT312	cysteine-desulfurase-DndA-[Nocardiopsis-sp.-CNT312]	1137268
WP_116248532.1	cysteine-desulfurase	GCF_003386285.1	380	Bacteria>Terrabacteria_group>Actinobacteria>Actinomycetia>Streptosporangiales	Nocardiopsis_sp._FIRDI_009	cysteine-desulfurase-DndA-[Nocardiopsis-sp.-FIRDI-009]	714197
WP_183654913.1	cysteine-desulfurase	GCF_014195505.1	383	Bacteria>Terrabacteria_group>Actinobacteria>Actinomycetia>Streptosporangiales	Nonomuraea_dietziae	cysteine-desulfurase-DndA-[Nonomuraea-dietziae]	65515
MBB3730345.1	cysteine-desulfurase	GCA_014195505.1	359	Bacteria>Terrabacteria_group>Actinobacteria>Actinomycetia>Streptosporangiales	Nonomuraea_dietziae	cysteine-desulfurase-[Nonomuraea-dietziae]	65515
WP_221341547.1	cysteine-desulfurase	-	391	Bacteria>Terrabacteria_group>Actinobacteria>Actinomycetia>Streptosporangiales	Nonomuraea_endophytica	cysteine-desulfurase-DndA-[Nonomuraea-endophytica]	714136
WP_225269637.1	cysteine-desulfurase	GCF_020215645.1	385	Bacteria>Terrabacteria_group>Actinobacteria>Actinomycetia>Streptosporangiales	Nonomuraea_gerenzanensis	cysteine-desulfurase-DndA-[Nonomuraea-gerenzanensis]	93944
NUW37329.1	cysteine-desulfurase	GCA_013363975.1	385	Bacteria>Terrabacteria_group>Actinobacteria>Actinomycetia>Streptosporangiales	Nonomuraea_montanisoli	cysteine-desulfurase-DndA-[Nonomuraea-montanisoli]	2741721
WP_235995817.1	cysteine-desulfurase	GCF_013363975.1	381	Bacteria>Terrabacteria_group>Actinobacteria>Actinomycetia>Streptosporangiales	Nonomuraea_montanisoli	cysteine-desulfurase-DndA-[Nonomuraea-montanisoli]	2741721
KAB8182368.1	cysteine-desulfurase	GCA_006334985.2	385	Bacteria>Terrabacteria_group>Actinobacteria>Actinomycetia>Streptosporangiales	Nonomuraea_phyllanthi	cysteine-desulfurase-DndA-[Nonomuraea-phyllanthi]	2219224
WP_139638315.1	cysteine-desulfurase	GCF_006334985.2	385	Bacteria>Terrabacteria_group>Actinobacteria>Actinomycetia>Streptosporangiales	Nonomuraea_phyllanthi	cysteine-desulfurase-DndA-[Nonomuraea-phyllanthi]	2219224
WP_168018178.1	cysteine-desulfurase	GCF_012034275.1	378	Bacteria>Terrabacteria_group>Actinobacteria>Actinomycetia>Streptosporangiales	Nonomuraea_sp._FMUSA5-5	cysteine-desulfurase-DndA-[Nonomuraea-sp.-FMUSA5-5]	2720023
WP_231618907.1	cysteine-desulfurase	GCF_001083785.1	391	Bacteria>Terrabacteria_group>Actinobacteria>Actinomycetia>Streptosporangiales	Nonomuraea_sp._SBT364	cysteine-desulfurase-[Nonomuraea-sp.-SBT364]	1580530
WP_132616968.1	cysteine-desulfurase	-	393	Bacteria>Terrabacteria_group>Actinobacteria>Actinomycetia>Streptosporangiales	Nonomuraea_terrae	cysteine-desulfurase-[Nonomuraea-terrae]	2530383
TMR22627.1	cysteine-desulfurase	GCA_005889735.1	376	Bacteria>Terrabacteria_group>Actinobacteria>Actinomycetia>Streptosporangiales	Nonomuraea_turkmeniaca	aminotransferase-class-V-fold-PLP-dependent-enzyme-[Nonomuraea-turkmeniaca]	103838
WP_138666002.1	cysteine-desulfurase	GCF_005889735.1	376	Bacteria>Terrabacteria_group>Actinobacteria>Actinomycetia>Streptosporangiales	Nonomuraea_turkmeniaca	aminotransferase-class-V-fold-PLP-dependent-enzyme-[Nonomuraea-turkmeniaca]	103838
WP_204069749.1	cysteine-desulfurase	GCF_016863175.1	382	Bacteria>Terrabacteria_group>Actinobacteria>Actinomycetia>Streptosporangiales	Planobispora_siamensis	cysteine-desulfurase-DndA-[Planobispora-siamensis]	936338
GIH97769.1	cysteine-desulfurase	GCA_016863175.1	382	Bacteria>Terrabacteria_group>Actinobacteria>Actinomycetia>Streptosporangiales	Planobispora_siamensis	putative-L-cysteine-desulfurase-[Planobispora-siamensis]	936338
WP_191892950.1	cysteine-desulfurase	GCF_016863215.1	382	Bacteria>Terrabacteria_group>Actinobacteria>Actinomycetia>Streptosporangiales	Planomonospora_parontospora	cysteine-desulfurase-DndA-[Planomonospora-parontospora]	58119
GGK44987.1	cysteine-desulfurase	GCA_014647935.1	382	Bacteria>Terrabacteria_group>Actinobacteria>Actinomycetia>Streptosporangiales	Planomonospora_parontospora	putative-L-cysteine-desulfurase-[Planomonospora-parontospora]	58119
GII06269.1	cysteine-desulfurase	GCA_016863215.1	382	Bacteria>Terrabacteria_group>Actinobacteria>Actinomycetia>Streptosporangiales	Planomonospora_parontospora_subsp._parontospora	putative-L-cysteine-desulfurase-[Planomonospora-parontospora-subsp.-parontospora]	97194
WP_230883972.1	cysteine-desulfurase	-	361	Bacteria>Terrabacteria_group>Actinobacteria>Actinomycetia>Streptosporangiales	Planomonospora_sp._ID82291	aminotransferase-class-V-fold-PLP-dependent-enzyme-[Planomonospora-sp.-ID82291]	2738136
WP_189165615.1	cysteine-desulfurase	GCF_014646695.1	381	Bacteria>Terrabacteria_group>Actinobacteria>Actinomycetia>Streptosporangiales	Sphaerisporangium_melleum	cysteine-desulfurase-DndA-[Sphaerisporangium-melleum]	321316
GGL03243.1	cysteine-desulfurase	GCA_014646695.1	381	Bacteria>Terrabacteria_group>Actinobacteria>Actinomycetia>Streptosporangiales	Sphaerisporangium_melleum	putative-L-cysteine-desulfurase-[Sphaerisporangium-melleum]	321316
GII69422.1	cysteine-desulfurase	GCA_016863375.1	381	Bacteria>Terrabacteria_group>Actinobacteria>Actinomycetia>Streptosporangiales	Sphaerisporangium_melleum	putative-L-cysteine-desulfurase-[Sphaerisporangium-melleum]	321316
PZG56087.1	cysteine-desulfurase	GCA_003236385.1	385	Bacteria>Terrabacteria_group>Actinobacteria>Actinomycetia>Streptosporangiales	Spongiactinospora_gelatinilytica	cysteine-desulfurase-DndA-[Spongiactinospora-gelatinilytica]	2666298
WP_233507018.1	cysteine-desulfurase	GCF_003236385.1	387	Bacteria>Terrabacteria_group>Actinobacteria>Actinomycetia>Streptosporangiales	Spongiactinospora_gelatinilytica	cysteine-desulfurase-DndA-[Spongiactinospora-gelatinilytica]	2666298
MBV9449975.1	cysteine-desulfurase	GCA_019245115.1	400	Bacteria>Terrabacteria_group>Actinobacteria>Actinomycetia>Streptosporangiales	Streptosporangiaceae_bacterium	cysteine-desulfurase-DndA-[Streptosporangiaceae-bacterium]	2339230
MBB4917452.1	cysteine-desulfurase	GCA_014203715.1	357	Bacteria>Terrabacteria_group>Actinobacteria>Actinomycetia>Streptosporangiales	Streptosporangium_saharense	cysteine-desulfurase-[Streptosporangium-saharense]	1706840
WP_012850559.1	cysteine-desulfurase	GCF_000024385.1	383	Bacteria>Terrabacteria_group>Actinobacteria>Actinomycetia>Streptosporangiales	Thermomonospora_curvata	cysteine-desulfurase-DndA-[Thermomonospora-curvata]	2020
MBU1801747.1	cysteine-desulfurase	GCA_018823685.1	389	Bacteria>Terrabacteria_group>Actinobacteria>Actinomycetia>unclassified_Actinomycetia	Actinomycetia_bacterium	cysteine-desulfurase-[Actinomycetia-bacterium]	1883427
MBR3313097.1	cysteine-desulfurase	GCA_017510745.1	68	Bacteria>Terrabacteria_group>Actinobacteria>Coriobacteriia>Coriobacteriales	Atopobiaceae_bacterium	hypothetical-protein-[Atopobiaceae-bacterium]	2485926
ERI06367.1	cysteine-desulfurase	GCA_000466405.1	386	Bacteria>Terrabacteria_group>Actinobacteria>Coriobacteriia>Coriobacteriales	Atopobium_sp._oral_taxon_810_str._F0209	cysteine-desulfurase-family-protein-[Atopobium-sp.-oral-taxon-810-str.-F0209]	1321773
WP_117734347.1	cysteine-desulfurase	GCF_003436895.1	383	Bacteria>Terrabacteria_group>Actinobacteria>Coriobacteriia>Coriobacteriales	Collinsella_sp._TF09-1AT	aminotransferase-class-V-fold-PLP-dependent-enzyme-[Collinsella-sp.-TF09-1AT]	2292334
WP_129425403.1	cysteine-desulfurase	GCF_004135645.1	385	Bacteria>Terrabacteria_group>Actinobacteria>Coriobacteriia>Coriobacteriales	Senegalimassilia_faecalis	aminotransferase-class-V-fold-PLP-dependent-enzyme-[Senegalimassilia-faecalis]	2509433
WP_165043040.1	cysteine-desulfurase	GCF_011038935.1	384	Bacteria>Terrabacteria_group>Actinobacteria>Coriobacteriia>Eggerthellales	Adlercreutzia_sp._ZJ138	aminotransferase-class-V-fold-PLP-dependent-enzyme-[Adlercreutzia-sp.-ZJ138]	2709405
WP_114551993.1	cysteine-desulfurase	GCF_003339915.1	383	Bacteria>Terrabacteria_group>Actinobacteria>Coriobacteriia>Eggerthellales	Eggerthella_lenta	aminotransferase-class-V-fold-PLP-dependent-enzyme-[Eggerthella-lenta]	84112
WP_114557010.1	cysteine-desulfurase	GCF_003340245.1	383	Bacteria>Terrabacteria_group>Actinobacteria>Coriobacteriia>Eggerthellales	Eggerthella_lenta	aminotransferase-class-V-fold-PLP-dependent-enzyme-[Eggerthella-lenta]	84112
WP_227110352.1	cysteine-desulfurase	-	383	Bacteria>Terrabacteria_group>Actinobacteria>Coriobacteriia>Eggerthellales	Eggerthella_lenta	aminotransferase-class-V-fold-PLP-dependent-enzyme-[Eggerthella-lenta]	84112
WP_161171103.1	cysteine-desulfurase	GCF_021378665.1	383	Bacteria>Terrabacteria_group>Actinobacteria>Coriobacteriia>Eggerthellales	Eggerthella	MULTISPECIES:-aminotransferase-class-V-fold-PLP-dependent-enzyme-[unclassified-Eggerthella]	84111
WP_205745444.1	cysteine-desulfurase	GCF_014640475.1	382	Bacteria>Terrabacteria_group>Actinobacteria>Nitriliruptoria>Egicoccales	Egicoccus_halophilus	cysteine-desulfurase-[Egicoccus-halophilus]	1670830
NLT05181.1	cysteine-desulfurase	GCA_012718975.1	382	Bacteria>Terrabacteria_group>Actinobacteria>Thermoleophilia>Solirubrobacterales	Solirubrobacterales_bacterium	cysteine-desulfurase-[Solirubrobacterales-bacterium]	1909295
MBX7136252.1	cysteine-desulfurase	GCA_019694835.1	406	Bacteria>Terrabacteria_group>Armatimonadetes>Fimbriimonadia>Fimbriimonadales	Fimbriimonadaceae_bacterium	cysteine-desulfurase-DndA-[Fimbriimonadaceae-bacterium]	2682144
WP_119072016.1	cysteine-desulfurase	GCF_003569045.1	388	Bacteria>Terrabacteria_group>Chloroflexi>Anaerolineae>Aggregatilineales	Aggregatilinea_lenta	cysteine-desulfurase-[Aggregatilinea-lenta]	913108
OQY34318.1	cysteine-desulfurase	GCA_002084745.1	387	Bacteria>Terrabacteria_group>Chloroflexi>Anaerolineae>Anaerolineales	Anaerolineaceae_bacterium_4572_5.2	IscS-subfamily-cysteine-desulfurase-[Anaerolineaceae-bacterium-4572_5.2]	1971725
MCA9900618.1	cysteine-desulfurase	GCA_020432515.1	392	Bacteria>Terrabacteria_group>Chloroflexi>Anaerolineae>Anaerolineales	Anaerolineales_bacterium	cysteine-desulfurase-[Anaerolineales-bacterium]	2073117
HIP71401.1	cysteine-desulfurase	GCA_015662625.1	201	Bacteria>Terrabacteria_group>Chloroflexi>Anaerolineae>unclassified_Anaerolineae	Anaerolineae_bacterium	aminotransferase-class-V-fold-PLP-dependent-enzyme-[Anaerolineae-bacterium]	2052143
MBN1877910.1	cysteine-desulfurase	GCA_016931185.1	386	Bacteria>Terrabacteria_group>Chloroflexi>Anaerolineae>unclassified_Anaerolineae	Anaerolineae_bacterium	cysteine-desulfurase-[Anaerolineae-bacterium]	2052143
MCB0164396.1	cysteine-desulfurase	GCA_020433785.1	387	Bacteria>Terrabacteria_group>Chloroflexi>Anaerolineae>unclassified_Anaerolineae	Anaerolineae_bacterium	cysteine-desulfurase-[Anaerolineae-bacterium]	2052143
MCC6604724.1	cysteine-desulfurase	GCA_020848035.1	388	Bacteria>Terrabacteria_group>Chloroflexi>Anaerolineae>unclassified_Anaerolineae	Anaerolineae_bacterium	cysteine-desulfurase-[Anaerolineae-bacterium]	2052143
WP_097643789.1	cysteine-desulfurase	GCF_002532535.1	151	Bacteria>Terrabacteria_group>Chloroflexi>Chloroflexia>Chloroflexales	Candidatus_Viridilinea_mediisalina	aminotransferase-class-V-fold-PLP-dependent-enzyme-[Candidatus-Viridilinea-mediisalina]	2024553
NJL06331.1	cysteine-desulfurase	GCA_012031225.1	389	Bacteria>Terrabacteria_group>Chloroflexi>Chloroflexia>Chloroflexales	Chloroflexaceae_bacterium	cysteine-desulfurase-[Chloroflexaceae-bacterium]	2562117
NNJ10289.1	cysteine-desulfurase	GCA_004138175.2	388	Bacteria>Terrabacteria_group>Chloroflexi>Chloroflexia>Chloroflexales	Chloroflexales_bacterium_ZM16-3	aminotransferase-class-V-fold-PLP-dependent-enzyme-[Chloroflexales-bacterium-ZM16-3]	2496870
NTW02075.1	cysteine-desulfurase	GCA_013336555.1	388	Bacteria>Terrabacteria_group>Chloroflexi>Chloroflexia>Chloroflexales	Oscillochloris_sp.	aminotransferase-class-V-fold-PLP-dependent-enzyme-[Oscillochloris-sp.]	2547424
NTW03707.1	cysteine-desulfurase	GCA_013336555.1	172	Bacteria>Terrabacteria_group>Chloroflexi>Chloroflexia>Chloroflexales	Oscillochloris_sp.	aminotransferase-class-V-fold-PLP-dependent-enzyme-[Oscillochloris-sp.]	2547424
WP_220597245.1	cysteine-desulfurase	GCF_019599295.1	388	Bacteria>Terrabacteria_group>Chloroflexi>Chloroflexia>Chloroflexales	Oscillochloris_sp._ZM17-4	aminotransferase-class-V-fold-PLP-dependent-enzyme-[Oscillochloris-sp.-ZM17-4]	2866714
NCC35356.1	cysteine-desulfurase	GCA_009929835.1	41	Bacteria>Terrabacteria_group>Chloroflexi>Chloroflexia>unclassified_Chloroflexia	Chloroflexia_bacterium	aminotransferase-class-V-fold-PLP-dependent-enzyme-[Chloroflexia-bacterium]	2448782
WP_041342072.1	cysteine-desulfurase	GCF_000830905.1	382	Bacteria>Terrabacteria_group>Chloroflexi>Dehalococcoidia>Dehalococcoidales	Dehalococcoides_mccartyi	aminotransferase-class-V-fold-PLP-dependent-enzyme-[Dehalococcoides-mccartyi]	61435
AII59478.1	cysteine-desulfurase	GCA_000830905.1	382	Bacteria>Terrabacteria_group>Chloroflexi>Dehalococcoidia>Dehalococcoidales	Dehalococcoides_mccartyi_CG4	cysteine-desulfurase-[Dehalococcoides-mccartyi-CG4]	1432060
RAL69806.1	cysteine-desulfurase	GCA_003260445.1	399	Bacteria>Terrabacteria_group>Chloroflexi>Dehalococcoidia>Dehalococcoidales	Dehalococcoides_mccartyi	Cysteine-desulfurase-[Dehalococcoides-mccartyi]	61435
RAL70564.1	cysteine-desulfurase	GCA_003260405.1	399	Bacteria>Terrabacteria_group>Chloroflexi>Dehalococcoidia>Dehalococcoidales	Dehalococcoides_mccartyi	Cysteine-desulfurase-[Dehalococcoides-mccartyi]	61435
HAT45615.1	cysteine-desulfurase	GCA_003483545.1	395	Bacteria>Terrabacteria_group>Chloroflexi>Ktedonobacteria>Ktedonobacterales	Ktedonobacter_sp.	IscS-subfamily-cysteine-desulfurase-[Ktedonobacter-sp.]	1932716
WP_069803598.1	cysteine-desulfurase	GCF_001748285.1	386	Bacteria>Terrabacteria_group>Chloroflexi>Ktedonobacteria>Thermogemmatisporales	Thermogemmatispora_onikobensis	cysteine-desulfurase-[Thermogemmatispora-onikobensis]	732234
NOK58229.1	cysteine-desulfurase	GCA_013122435.1	387	Bacteria>Terrabacteria_group>Chloroflexi>unclassified_Chloroflexi	Chloroflexi_bacterium_AL-N1	aminotransferase-class-V-fold-PLP-dependent-enzyme-[Chloroflexi-bacterium-AL-N1]	2686367
KAA3664000.1	cysteine-desulfurase	GCA_008501785.1	286	Bacteria>Terrabacteria_group>Chloroflexi>unclassified_Chloroflexi	Chloroflexi_bacterium	aminotransferase-class-V-fold-PLP-dependent-enzyme-[Chloroflexi-bacterium]	2026724
MBP7997811.1	cysteine-desulfurase	GCA_018004095.1	389	Bacteria>Terrabacteria_group>Chloroflexi>unclassified_Chloroflexi	Chloroflexi_bacterium	aminotransferase-class-V-fold-PLP-dependent-enzyme-[Chloroflexi-bacterium]	2026724
RMG70438.1	cysteine-desulfurase	GCA_003696565.1	33	Bacteria>Terrabacteria_group>Chloroflexi>unclassified_Chloroflexi	Chloroflexi_bacterium	aminotransferase-class-V-fold-PLP-dependent-enzyme,-partial-[Chloroflexi-bacterium]	2026724
MCE7947201.1	cysteine-desulfurase	GCA_021404685.1	386	Bacteria>Terrabacteria_group>Chloroflexi>unclassified_Chloroflexi	Chloroflexi_bacterium_CFX4	aminotransferase-class-V-fold-PLP-dependent-enzyme-[Chloroflexi-bacterium-CFX4]	2293709
MBK6709672.1	cysteine-desulfurase	GCA_016703445.1	389	Bacteria>Terrabacteria_group>Chloroflexi>unclassified_Chloroflexi	Chloroflexi_bacterium	cysteine-desulfurase-[Chloroflexi-bacterium]	2026724
MCA0457128.1	cysteine-desulfurase	GCA_020161935.1	388	Bacteria>Terrabacteria_group>Chloroflexi>unclassified_Chloroflexi	Chloroflexi_bacterium	cysteine-desulfurase-[Chloroflexi-bacterium]	2026724
MCC6630146.1	cysteine-desulfurase	GCA_020849725.1	426	Bacteria>Terrabacteria_group>Chloroflexi>unclassified_Chloroflexi	Chloroflexi_bacterium	cysteine-desulfurase-[Chloroflexi-bacterium]	2026724
RLC79944.1	cysteine-desulfurase	GCA_003648025.1	392	Bacteria>Terrabacteria_group>Chloroflexi>unclassified_Chloroflexi	Chloroflexi_bacterium	IscS-subfamily-cysteine-desulfurase-[Chloroflexi-bacterium]	2026724
RLC86285.1	cysteine-desulfurase	GCA_003648075.1	392	Bacteria>Terrabacteria_group>Chloroflexi>unclassified_Chloroflexi	Chloroflexi_bacterium	IscS-subfamily-cysteine-desulfurase-[Chloroflexi-bacterium]	2026724
MBY0358207.1	cysteine-desulfurase	GCA_019750335.1	396	Bacteria>Terrabacteria_group>Cyanobacteria/Melainabacteria_group>Candidatus_Melainabacteria>Candidatus_Obscuribacterales	Candidatus_Obscuribacterales_bacterium	cysteine-desulfurase-[Candidatus-Obscuribacterales-bacterium]	2364082
WP_073623016.1	cysteine-desulfurase	GCF_001904745.1	383	Bacteria>Terrabacteria_group>Cyanobacteria/Melainabacteria_group>Cyanobacteria>Nostocales	Calothrix_sp._HK-06	IscS-subfamily-cysteine-desulfurase-[Calothrix-sp.-HK-06]	1137096
WP_015197617.1	cysteine-desulfurase	GCF_000317435.1	385	Bacteria>Terrabacteria_group>Cyanobacteria/Melainabacteria_group>Cyanobacteria>Nostocales	Calothrix_sp._PCC_6303	alanine–glyoxylate-aminotransferase-family-protein-[Calothrix-sp.-PCC-6303]	1170562
WP_127080522.1	cysteine-desulfurase	GCF_003991905.1	383	Bacteria>Terrabacteria_group>Cyanobacteria/Melainabacteria_group>Cyanobacteria>Nostocales	Dulcicalothrix_desertica	IscS-subfamily-cysteine-desulfurase-[Calothrix-desertica]	32056
WP_017652020.1	cysteine-desulfurase	GCF_000332295.1	388	Bacteria>Terrabacteria_group>Cyanobacteria/Melainabacteria_group>Cyanobacteria>Nostocales	Fortiea_contorta	aminotransferase-class-V-fold-PLP-dependent-enzyme-[Fortiea-contorta]	1892405
WP_238845606.1	cysteine-desulfurase	GCF_014023275.1	356	Bacteria>Terrabacteria_group>Cyanobacteria/Melainabacteria_group>Cyanobacteria>Nostocales	Nostoc_edaphicum	cysteine-desulfurase-[Nostoc-edaphicum]	264686
AUT00534.1	cysteine-desulfurase	GCA_002896875.1	395	Bacteria>Terrabacteria_group>Cyanobacteria/Melainabacteria_group>Cyanobacteria>Nostocales	Nostoc_sp._CENA543	cysteine-lyase-[Nostoc-sp.-CENA543]	1869241
MCC5620238.1	cysteine-desulfurase	GCA_020829685.1	389	Bacteria>Terrabacteria_group>Cyanobacteria/Melainabacteria_group>Cyanobacteria>Nostocales	Nostoc_sp._CHAB_5715	cysteine-desulfurase-DndA-[Nostoc-sp.-CHAB-5715]	2780400
WP_229451341.1	cysteine-desulfurase	GCF_020829685.1	389	Bacteria>Terrabacteria_group>Cyanobacteria/Melainabacteria_group>Cyanobacteria>Nostocales	Nostoc_sp._CHAB_5715	cysteine-desulfurase-DndA-[Nostoc-sp.-CHAB-5715]	2780400
MCC5646700.1	cysteine-desulfurase	GCA_020829665.1	146	Bacteria>Terrabacteria_group>Cyanobacteria/Melainabacteria_group>Cyanobacteria>Nostocales	Nostoc_sp._CHAB_5824	SufE-family-protein-[Nostoc-sp.-CHAB-5824]	2780402
WP_229465273.1	cysteine-desulfurase	GCF_020829665.1	146	Bacteria>Terrabacteria_group>Cyanobacteria/Melainabacteria_group>Cyanobacteria>Nostocales	Nostoc_sp._CHAB_5824	SufE-family-protein-[Nostoc-sp.-CHAB-5824]	2780402
WP_236143465.1	cysteine-desulfurase	GCF_021596505.1	395	Bacteria>Terrabacteria_group>Cyanobacteria/Melainabacteria_group>Cyanobacteria>Nostocales	Nostoc_sp._CMAA1605	aminotransferase-class-V-fold-PLP-dependent-enzyme-[Nostoc-sp.-CMAA1605]	2055159
WP_228058257.1	cysteine-desulfurase	GCF_015206985.1	423	Bacteria>Terrabacteria_group>Cyanobacteria/Melainabacteria_group>Cyanobacteria>Nostocales	Nostoc_sp._LEGE_12447	cysteine-desulfurase-[Nostoc-sp.-LEGE-12447]	1828640
MBN4003505.1	cysteine-desulfurase	GCA_017115165.1	388	Bacteria>Terrabacteria_group>Cyanobacteria/Melainabacteria_group>Cyanobacteria>Nostocales	Nostoc_sp._LPT	cysteine-desulfurase-[Nostoc-sp.-LPT]	2815387
WP_163929538.1	cysteine-desulfurase	GCF_010747425.1	385	Bacteria>Terrabacteria_group>Cyanobacteria/Melainabacteria_group>Cyanobacteria>Nostocales	Nostoc_sp._UIC_10630	cysteine-desulfurase-[Nostoc-sp.-UIC-10630]	2100146
WP_190607227.1	cysteine-desulfurase	GCF_019056575.1	383	Bacteria>Terrabacteria_group>Cyanobacteria/Melainabacteria_group>Cyanobacteria>Nostocales	Richelia_sinica	alanine–glyoxylate-aminotransferase-family-protein-[Richelia-sinica]	1357545
NET00029.1	cysteine-desulfurase	GCA_010672785.1	391	Bacteria>Terrabacteria_group>Cyanobacteria/Melainabacteria_group>Cyanobacteria>Nostocales	Sphaerospermopsis_sp._SIO1G1	cysteine-desulfurase-[Sphaerospermopsis-sp.-SIO1G1]	2607814
WP_206814331.1	cysteine-desulfurase	GCF_017312365.1	140	Bacteria>Terrabacteria_group>Cyanobacteria/Melainabacteria_group>Cyanobacteria>Oscillatoriophycideae	Chroococcus_sp._FPU101	SufE-family-protein-[Chroococcus-sp.-FPU101]	1974212
WP_006101098.1	cysteine-desulfurase	GCF_000155555.1	391	Bacteria>Terrabacteria_group>Cyanobacteria/Melainabacteria_group>Cyanobacteria>Oscillatoriophycideae	Coleofasciculus_chthonoplastes	cysteine-desulfurase-[Coleofasciculus-chthonoplastes]	64178
EDX75388.1	cysteine-desulfurase	GCA_000155555.1	391	Bacteria>Terrabacteria_group>Cyanobacteria/Melainabacteria_group>Cyanobacteria>Oscillatoriophycideae	Coleofasciculus_chthonoplastes_PCC_7420	aminotransferase,-class-V-superfamily-[Coleofasciculus-chthonoplastes-PCC-7420]	118168
AFZ46557.1	cysteine-desulfurase	GCA_000317655.1	364	Bacteria>Terrabacteria_group>Cyanobacteria/Melainabacteria_group>Cyanobacteria>Oscillatoriophycideae	Cyanobacterium_stanieri_PCC_7202	aminotransferase-class-V-[Cyanobacterium-stanieri-PCC-7202]	292563
MBW4618585.1	cysteine-desulfurase	GCA_019359615.1	385	Bacteria>Terrabacteria_group>Cyanobacteria/Melainabacteria_group>Cyanobacteria>Oscillatoriophycideae	Cyanosarcina_radialis_HA8281-LM2	cysteine-desulfurase-[Cyanosarcina-radialis-HA8281-LM2]	1874554
HAC62454.1	cysteine-desulfurase	GCA_003448685.1	376	Bacteria>Terrabacteria_group>Cyanobacteria/Melainabacteria_group>Cyanobacteria>Oscillatoriophycideae	Cyanothece_sp._UBA12306	alanine–glyoxylate-aminotransferase-[Cyanothece-sp.-UBA12306]	2055757

Accession	Annotation	GCA	Length	Phyletic.spread	Strain.name	Defline	Species.Taxid
WP_166280330.1	cysteine-desulfurase	GCF_000817775.3	390	Bacteria>Terrabacteria_group>Cyanobacteria/Melainabacteria_group>Cyanobacteria>Oscillatoriophycideae	Lyngbya_confervoides	cysteine-desulfurase-[Aphanocapsa-montana]	207921
NCR54522.1	cysteine-desulfurase	GCA_009996085.1	140	Bacteria>Terrabacteria_group>Cyanobacteria/Melainabacteria_group>Cyanobacteria>Oscillatoriophycideae	Microcystis_aeruginosa_L211-07	SufE-family-protein-[Microcystis-aeruginosa-L211-07]	2685972
NCR96378.1	cysteine-desulfurase	GCA_009996025.1	140	Bacteria>Terrabacteria_group>Cyanobacteria/Melainabacteria_group>Cyanobacteria>Oscillatoriophycideae	Microcystis_aeruginosa_L311-01	SufE-family-protein-[Microcystis-aeruginosa-L311-01]	2685975
NCR69744.1	cysteine-desulfurase	GCA_009995925.1	140	Bacteria>Terrabacteria_group>Cyanobacteria/Melainabacteria_group>Cyanobacteria>Oscillatoriophycideae	Microcystis_aeruginosa_LG13-12	SufE-family-protein-[Microcystis-aeruginosa-LG13-12]	2685980
TRU36570.1	cysteine-desulfurase	GCA_007095855.1	387	Bacteria>Terrabacteria_group>Cyanobacteria/Melainabacteria_group>Cyanobacteria>Oscillatoriophycideae	Microcystis_aeruginosa_Ma_MB_F_20061100_S20D	aminotransferase-class-V-fold-PLP-dependent-enzyme-[Microcystis-aeruginosa-Ma_MB_F_20061100_S20D]	2486253
TRU33378.1	cysteine-desulfurase	GCA_007095885.1	140	Bacteria>Terrabacteria_group>Cyanobacteria/Melainabacteria_group>Cyanobacteria>Oscillatoriophycideae	Microcystis_aeruginosa_Ma_MB_F_20061100_S20	SufE-family-protein-[Microcystis-aeruginosa-Ma_MB_F_20061100_S20]	2486252
TRU21314.1	cysteine-desulfurase	GCA_007095785.1	140	Bacteria>Terrabacteria_group>Cyanobacteria/Melainabacteria_group>Cyanobacteria>Oscillatoriophycideae	Microcystis_aeruginosa_Ma_SC_T_19800800_S464	SufE-family-protein-[Microcystis-aeruginosa-Ma_SC_T_19800800_S464]	2486257
WP_002738156.1	cysteine-desulfurase	GCF_000330925.1	140	Bacteria>Terrabacteria_group>Cyanobacteria/Melainabacteria_group>Cyanobacteria>Oscillatoriophycideae	Microcystis_aeruginosa	SufE-family-protein-[Microcystis-aeruginosa]	1126
WP_002755684.1	cysteine-desulfurase	GCF_000599945.1	140	Bacteria>Terrabacteria_group>Cyanobacteria/Melainabacteria_group>Cyanobacteria>Oscillatoriophycideae	Microcystis_aeruginosa	SufE-family-protein-[Microcystis-aeruginosa]	1126
WP_002801638.1	cysteine-desulfurase	GCF_000312285.1	140	Bacteria>Terrabacteria_group>Cyanobacteria/Melainabacteria_group>Cyanobacteria>Oscillatoriophycideae	Microcystis_aeruginosa	SufE-family-protein-[Microcystis-aeruginosa]	1126
WP_110579972.1	cysteine-desulfurase	GCF_003206555.1	140	Bacteria>Terrabacteria_group>Cyanobacteria/Melainabacteria_group>Cyanobacteria>Oscillatoriophycideae	Microcystis_aeruginosa	SufE-family-protein-[Microcystis-aeruginosa]	1126
WP_151696997.1	cysteine-desulfurase	GCF_008974145.1	140	Bacteria>Terrabacteria_group>Cyanobacteria/Melainabacteria_group>Cyanobacteria>Oscillatoriophycideae	Microcystis_aeruginosa	SufE-family-protein-[Microcystis-aeruginosa]	1126
REJ56266.1	cysteine-desulfurase	GCA_003388685.1	140	Bacteria>Terrabacteria_group>Cyanobacteria/Melainabacteria_group>Cyanobacteria>Oscillatoriophycideae	Microcystis_aeruginosa_TA09	SufE-family-protein-[Microcystis-aeruginosa-TA09]	2060472
REJ42572.1	cysteine-desulfurase	GCA_003390815.1	140	Bacteria>Terrabacteria_group>Cyanobacteria/Melainabacteria_group>Cyanobacteria>Oscillatoriophycideae	Microcystis_flos-aquae_TF09	SufE-family-protein-[Microcystis-flos-aquae-TF09]	2060473
WP_002740581.1	cysteine-desulfurase	GCF_000332585.1	140	Bacteria>Terrabacteria_group>Cyanobacteria/Melainabacteria_group>Cyanobacteria>Oscillatoriophycideae	Microcystis	MULTISPECIES:-SufE-family-protein-[Microcystis]	1125
WP_002761229.1	cysteine-desulfurase	GCF_000010625.1	140	Bacteria>Terrabacteria_group>Cyanobacteria/Melainabacteria_group>Cyanobacteria>Oscillatoriophycideae	Microcystis	MULTISPECIES:-SufE-family-protein-[Microcystis]	1125
WP_002766044.1	cysteine-desulfurase	GCF_000312185.1	140	Bacteria>Terrabacteria_group>Cyanobacteria/Melainabacteria_group>Cyanobacteria>Oscillatoriophycideae	Microcystis	MULTISPECIES:-SufE-family-protein-[Microcystis]	1125
WP_045357226.1	cysteine-desulfurase	GCF_000787675.1	140	Bacteria>Terrabacteria_group>Cyanobacteria/Melainabacteria_group>Cyanobacteria>Oscillatoriophycideae	Microcystis	MULTISPECIES:-SufE-family-protein-[Microcystis]	1125
WP_046663438.1	cysteine-desulfurase	GCF_003112475.1	140	Bacteria>Terrabacteria_group>Cyanobacteria/Melainabacteria_group>Cyanobacteria>Oscillatoriophycideae	Microcystis	MULTISPECIES:-SufE-family-protein-[Microcystis]	1125
WP_069475603.1	cysteine-desulfurase	GCF_002897275.1	140	Bacteria>Terrabacteria_group>Cyanobacteria/Melainabacteria_group>Cyanobacteria>Oscillatoriophycideae	Microcystis	MULTISPECIES:-SufE-family-protein-[Microcystis]	1125
MCA2693986.1	cysteine-desulfurase	GCA_020245755.1	387	Bacteria>Terrabacteria_group>Cyanobacteria/Melainabacteria_group>Cyanobacteria>Oscillatoriophycideae	Microcystis_sp._M034S2	aminotransferase-class-V-fold-PLP-dependent-enzyme-[Microcystis-sp.-M034S2]	2771162
MCA2694229.1	cysteine-desulfurase	GCA_020245755.1	140	Bacteria>Terrabacteria_group>Cyanobacteria/Melainabacteria_group>Cyanobacteria>Oscillatoriophycideae	Microcystis_sp._M034S2	SufE-family-protein-[Microcystis-sp.-M034S2]	2771162
MCA2657081.1	cysteine-desulfurase	GCA_020245625.1	140	Bacteria>Terrabacteria_group>Cyanobacteria/Melainabacteria_group>Cyanobacteria>Oscillatoriophycideae	Microcystis_sp._M049S2	SufE-family-protein-[Microcystis-sp.-M049S2]	2771169
MCA2655795.1	cysteine-desulfurase	GCA_020245605.1	140	Bacteria>Terrabacteria_group>Cyanobacteria/Melainabacteria_group>Cyanobacteria>Oscillatoriophycideae	Microcystis_sp._M061S2	SufE-family-protein-[Microcystis-sp.-M061S2]	2771171
TRU08291.1	cysteine-desulfurase	GCA_007095705.1	140	Bacteria>Terrabacteria_group>Cyanobacteria/Melainabacteria_group>Cyanobacteria>Oscillatoriophycideae	Microcystis_sp._Msp_OC_L_20101000_S702	SufE-family-protein-[Microcystis-sp.-Msp_OC_L_20101000_S702]	2486218
TRV13632.1	cysteine-desulfurase	GCA_007096585.1	140	Bacteria>Terrabacteria_group>Cyanobacteria/Melainabacteria_group>Cyanobacteria>Oscillatoriophycideae	Microcystis_wesenbergii_Mw_MB_S_20031200_S109	SufE-family-protein-[Microcystis-wesenbergii-Mw_MB_S_20031200_S109]	2486240
WP_190358621.1	cysteine-desulfurase	GCF_014696875.1	140	Bacteria>Terrabacteria_group>Cyanobacteria/Melainabacteria_group>Cyanobacteria>Oscillatoriophycideae	Microcystis_wesenbergii	SufE-family-protein-[Microcystis-wesenbergii]	44823
REJ55268.1	cysteine-desulfurase	GCA_003386495.1	140	Bacteria>Terrabacteria_group>Cyanobacteria/Melainabacteria_group>Cyanobacteria>Oscillatoriophycideae	Microcystis_wesenbergii_TW10	SufE-family-protein-[Microcystis-wesenbergii-TW10]	2060474
NJM59250.1	cysteine-desulfurase	GCA_012032195.1	487	Bacteria>Terrabacteria_group>Cyanobacteria/Melainabacteria_group>Cyanobacteria>Oscillatoriophycideae	Oscillatoriales_cyanobacterium_RU_3_3	aminotransferase-class-I/II-fold-pyridoxal-phosphate-dependent-enzyme-[Oscillatoriales-cyanobacterium-RU_3_3]	2720441
WP_012597003.1	cysteine-desulfurase	GCF_000021805.1	375	Bacteria>Terrabacteria_group>Cyanobacteria/Melainabacteria_group>Cyanobacteria>Oscillatoriophycideae	Rippkaea_orientalis	alanine–glyoxylate-aminotransferase-family-protein-[Rippkaea-orientalis]	2546366
ACK67745.1	cysteine-desulfurase	GCA_000021805.1	375	Bacteria>Terrabacteria_group>Cyanobacteria/Melainabacteria_group>Cyanobacteria>Oscillatoriophycideae	Rippkaea_orientalis_PCC_8801	aminotransferase-class-V-[Rippkaea-orientalis-PCC-8801]	41431
OJJ18426.1	cysteine-desulfurase	GCA_001890975.1	385	Bacteria>Terrabacteria_group>Cyanobacteria/Melainabacteria_group>Cyanobacteria>Oscillatoriophycideae	Roseofilum_reptotaenium_AO1-A	IscS-subfamily-cysteine-desulfurase-[Roseofilum-reptotaenium-AO1-A]	1925591
MBP0010352.1	cysteine-desulfurase	GCA_017746785.1	385	Bacteria>Terrabacteria_group>Cyanobacteria/Melainabacteria_group>Cyanobacteria>Oscillatoriophycideae	Roseofilum_sp._Belize_Diploria	IscS-subfamily-cysteine-desulfurase-[Roseofilum-sp.-Belize-Diploria]	2821501
MBP0029688.1	cysteine-desulfurase	GCA_017746735.1	385	Bacteria>Terrabacteria_group>Cyanobacteria/Melainabacteria_group>Cyanobacteria>Oscillatoriophycideae	Roseofilum_sp._Guam	IscS-subfamily-cysteine-desulfurase-[Roseofilum-sp.-Guam]	2821502
MBP0011644.1	cysteine-desulfurase	GCA_017746835.1	385	Bacteria>Terrabacteria_group>Cyanobacteria/Melainabacteria_group>Cyanobacteria>Oscillatoriophycideae	Roseofilum_sp._SID3	IscS-subfamily-cysteine-desulfurase-[Roseofilum-sp.-SID3]	2821499
WP_144867655.1	cysteine-desulfurase	GCF_900659865.1	381	Bacteria>Terrabacteria_group>Cyanobacteria/Melainabacteria_group>Cyanobacteria>Pleurocapsales	Hyella_patelloides	alanine–glyoxylate-aminotransferase-family-protein-[Hyella-patelloides]	1982969
WP_144868125.1	cysteine-desulfurase	GCF_900659865.1	420	Bacteria>Terrabacteria_group>Cyanobacteria/Melainabacteria_group>Cyanobacteria>Pleurocapsales	Hyella_patelloides	SufS-family-cysteine-desulfurase-[Hyella-patelloides]	1982969
WP_036481564.1	cysteine-desulfurase	GCF_000756305.1	146	Bacteria>Terrabacteria_group>Cyanobacteria/Melainabacteria_group>Cyanobacteria>Pleurocapsales	Myxosarcina_sp._GI1	SufE-family-protein-[Myxosarcina-sp.-GI1]	1541065
WP_017305944.1	cysteine-desulfurase	GCF_000314005.1	383	Bacteria>Terrabacteria_group>Cyanobacteria/Melainabacteria_group>Cyanobacteria>Spirulinales	Spirulina_subsalsa	alanine–glyoxylate-aminotransferase-family-protein-[Spirulina-subsalsa]	54311
WP_010479614.1	cysteine-desulfurase	GCF_000238775.2	404	Bacteria>Terrabacteria_group>Cyanobacteria/Melainabacteria_group>Cyanobacteria>Synechococcales	Acaryochloris_sp._CCMEE_5410	IscS-subfamily-cysteine-desulfurase-[Acaryochloris-sp.-CCMEE-5410]	310037
RMH76135.1	cysteine-desulfurase	GCA_003695985.1	492	Bacteria>Terrabacteria_group>Cyanobacteria/Melainabacteria_group>Cyanobacteria>unclassified_Cyanobacteria	Cyanobacteria_bacterium_J007	aminotransferase-class-I/II-fold-pyridoxal-phosphate-dependent-enzyme-[Cyanobacteria-bacterium-J007]	2420339
MBE7709151.1	cysteine-desulfurase	GCA_015152455.1	381	Bacteria>Terrabacteria_group>Cyanobacteria/Melainabacteria_group>Cyanobacteria>unclassified_Cyanobacteria	Cyanobacteria_bacterium_SIG32	aminotransferase-class-V-fold-PLP-dependent-enzyme-[Cyanobacteria-bacterium-SIG32]	2775576
WP_191300566.1	cysteine-desulfurase	GCF_014653295.1	384	Bacteria>Terrabacteria_group>Deinococcus-Thermus>Deinococci>Deinococcales	Deinococcus_indicus	cysteine-desulfurase-[Deinococcus-indicus]	223556
GHG32622.1	cysteine-desulfurase	GCA_014653295.1	384	Bacteria>Terrabacteria_group>Deinococcus-Thermus>Deinococci>Deinococcales	Deinococcus_indicus	cysteine-desulfurase-IscS-[Deinococcus-indicus]	223556
WP_211317890.1	cysteine-desulfurase	GCF_003217515.1	390	Bacteria>Terrabacteria_group>Deinococcus-Thermus>Deinococci>Deinococcales	Deinococcus_yavapaiensis	cysteine-desulfurase-[Deinococcus-yavapaiensis]	309889
WP_188207534.1	cysteine-desulfurase	GCF_014595945.1	372	Bacteria>Terrabacteria_group>Firmicutes>Bacilli>Bacillales	Alkalibacillus_aidingensis	cysteine-desulfurase-[Alkalibacillus-aidingensis]	2747607
WP_224880493.1	cysteine-desulfurase	GCF_020171805.1	373	Bacteria>Terrabacteria_group>Firmicutes>Bacilli>Bacillales	Alkalihalobacillus_algicola	aminotransferase-class-V-fold-PLP-dependent-enzyme-[Alkalihalobacillus-algicola]	225844
WP_034625578.1	cysteine-desulfurase	GCF_000787375.1	373	Bacteria>Terrabacteria_group>Firmicutes>Bacilli>Bacillales	Alkalihalobacillus_okhensis	cysteine-desulfurase-DndA-[Alkalihalobacillus-okhensis]	333138
WP_059104435.1	cysteine-desulfurase	GCF_001484965.1	375	Bacteria>Terrabacteria_group>Firmicutes>Bacilli>Bacillales	Alkalihalobacillus_shacheensis	cysteine-desulfurase-[Alkalihalobacillus-shacheensis]	1649580
WP_227939005.1	cysteine-desulfurase	GCF_020734105.1	373	Bacteria>Terrabacteria_group>Firmicutes>Bacilli>Bacillales	Alkalihalobacillus_sp._TRPH29	aminotransferase-class-V-fold-PLP-dependent-enzyme-[Alkalihalobacillus-sp.-TRPH29]	2879466
WP_088102995.1	cysteine-desulfurase	GCF_002156385.1	374	Bacteria>Terrabacteria_group>Firmicutes>Bacilli>Bacillales	Alkalihalobacillus_urbisdiaboli	aminotransferase-class-V-fold-PLP-dependent-enzyme-[Alkalihalobacillus-urbisdiaboli]	1960589
WP_144089334.1	cysteine-desulfurase	GCF_007559425.1	375	Bacteria>Terrabacteria_group>Firmicutes>Bacilli>Bacillales	Allobacillus_salarius	aminotransferase-class-V-fold-PLP-dependent-enzyme-[Allobacillus-sp.-SKP4-8]	1955272
WP_212369038.1	cysteine-desulfurase	GCF_018138385.1	375	Bacteria>Terrabacteria_group>Firmicutes>Bacilli>Bacillales	Allobacillus_saliphilus	aminotransferase-class-V-fold-PLP-dependent-enzyme-[Allobacillus-sp.-SKP8-2]	2912308
NMA91051.1	cysteine-desulfurase	GCA_012799235.1	372	Bacteria>Terrabacteria_group>Firmicutes>Bacilli>Bacillales	Amphibacillus_sp.	cysteine-desulfurase-[Amphibacillus-sp.]	1872500
WP_043070631.1	cysteine-desulfurase	GCF_000878885.1	373	Bacteria>Terrabacteria_group>Firmicutes>Bacilli>Bacillales	Aneurinibacillus_migulanus	aminotransferase-class-V-fold-PLP-dependent-enzyme-[Aneurinibacillus-migulanus]	47500
KIV56069.1	cysteine-desulfurase	GCA_000878885.1	373	Bacteria>Terrabacteria_group>Firmicutes>Bacilli>Bacillales	Aneurinibacillus_migulanus	cysteine-desulfurase-[Aneurinibacillus-migulanus]	47500
WP_003395738.1	cysteine-desulfurase	GCF_000353425.1	382	Bacteria>Terrabacteria_group>Firmicutes>Bacilli>Bacillales	Anoxybacillus_flavithermus	cysteine-desulfurase-[Anoxybacillus-flavithermus]	33934
WP_192953794.1	cysteine-desulfurase	GCF_014890455.1	382	Bacteria>Terrabacteria_group>Firmicutes>Bacilli>Bacillales	Anoxybacillus_flavithermus	cysteine-desulfurase-[Anoxybacillus-flavithermus]	33934
AST07063.1	cysteine-desulfurase	GCA_002243705.1	382	Bacteria>Terrabacteria_group>Firmicutes>Bacilli>Bacillales	Anoxybacillus_flavithermus	cysteine-desulfurase-DndA-[Anoxybacillus-flavithermus]	33934
WP_035049619.1	cysteine-desulfurase	GCF_000753875.1	382	Bacteria>Terrabacteria_group>Firmicutes>Bacilli>Bacillales	Anoxybacillus	MULTISPECIES:-cysteine-desulfurase-[Anoxybacillus]	150247
WP_171334989.1	cysteine-desulfurase	GCF_013096955.1	382	Bacteria>Terrabacteria_group>Firmicutes>Bacilli>Bacillales	Anoxybacillus_sp._EFIL	aminotransferase-class-V-fold-PLP-dependent-enzyme-[Anoxybacillus-sp.-EFIL]	2508869
KFZ42265.1	cysteine-desulfurase	GCA_000753875.1	382	Bacteria>Terrabacteria_group>Firmicutes>Bacilli>Bacillales	Anoxybacillus_sp._KU2-6(11)	cysteine-desulfurase-[Anoxybacillus-sp.-KU2-6(11)]	1535751
MBY6274198.1	cysteine-desulfurase	GCA_019806655.1	137	Bacteria>Terrabacteria_group>Firmicutes>Bacilli>Bacillales	Bacillaceae_bacterium	cysteine-desulfurase-DndA-[Bacillaceae-bacterium]	1889774
KAB7707124.1	cysteine-desulfurase	GCA_009183655.1	372	Bacteria>Terrabacteria_group>Firmicutes>Bacilli>Bacillales	Bacillus_aerolatus	aminotransferase-class-V-fold-PLP-dependent-enzyme-[Bacillus-aerolatus]	2653354
WP_152151070.1	cysteine-desulfurase	GCF_009183655.1	372	Bacteria>Terrabacteria_group>Firmicutes>Bacilli>Bacillales	Bacillus_aerolatus	cysteine-desulfurase-[Bacillus-aerolatus]	2653354
MCC2345188.1	cysteine-desulfurase	GCA_020686345.1	373	Bacteria>Terrabacteria_group>Firmicutes>Bacilli>Bacillales	Bacillus_anthracis	cysteine-desulfurase-DndA-[Bacillus-anthracis]	1392
WP_213403031.1	cysteine-desulfurase	GCF_018390475.1	373	Bacteria>Terrabacteria_group>Firmicutes>Bacilli>Bacillales	Bacillus_cabrialesii	cysteine-desulfurase-DndA-[Bacillus-sp.-TSO2]	2487276
WP_226542328.1	cysteine-desulfurase	GCF_020524035.1	373	Bacteria>Terrabacteria_group>Firmicutes>Bacilli>Bacillales	Bacillus_carboniphilus	aminotransferase-class-V-fold-PLP-dependent-enzyme-[Bacillus-carboniphilus]	86663
WP_098529204.1	cysteine-desulfurase	GCF_002565525.1	373	Bacteria>Terrabacteria_group>Firmicutes>Bacilli>Bacillales	Bacillus_cereus	cysteine-desulfurase-[Bacillus-cereus]	1396
MBK4743765.1	cysteine-desulfurase	GCA_016632305.1	373	Bacteria>Terrabacteria_group>Firmicutes>Bacilli>Bacillales	Bacillus_cereus	cysteine-desulfurase-DndA-[Bacillus-cereus]	1396
PEQ46985.1	cysteine-desulfurase	GCA_002558315.1	373	Bacteria>Terrabacteria_group>Firmicutes>Bacilli>Bacillales	Bacillus_cereus	cysteine-desulfurase-DndA-[Bacillus-cereus]	1396
PFM97288.1	cysteine-desulfurase	GCA_002565765.1	373	Bacteria>Terrabacteria_group>Firmicutes>Bacilli>Bacillales	Bacillus_cereus	cysteine-desulfurase-DndA-[Bacillus-cereus]	1396
PFN25096.1	cysteine-desulfurase	GCA_002565815.1	373	Bacteria>Terrabacteria_group>Firmicutes>Bacilli>Bacillales	Bacillus_cereus	cysteine-desulfurase-DndA-[Bacillus-cereus]	1396
WP_000966083.1	cysteine-desulfurase	GCF_000011625.1	373	Bacteria>Terrabacteria_group>Firmicutes>Bacilli>Bacillales	Bacillus_cereus	cysteine-desulfurase-DndA-[Bacillus-cereus]	1396
WP_048551478.1	cysteine-desulfurase	GCF_001044905.1	373	Bacteria>Terrabacteria_group>Firmicutes>Bacilli>Bacillales	Bacillus_cereus	cysteine-desulfurase-DndA-[Bacillus-cereus]	1396
WP_074569142.1	cysteine-desulfurase	GCF_001913385.1	373	Bacteria>Terrabacteria_group>Firmicutes>Bacilli>Bacillales	Bacillus_cereus	cysteine-desulfurase-DndA-[Bacillus-cereus]	1396
WP_098139402.1	cysteine-desulfurase	GCF_002555205.1	373	Bacteria>Terrabacteria_group>Firmicutes>Bacilli>Bacillales	Bacillus_cereus	cysteine-desulfurase-DndA-[Bacillus-cereus]	1396
WP_098198880.1	cysteine-desulfurase	GCF_002558315.1	373	Bacteria>Terrabacteria_group>Firmicutes>Bacilli>Bacillales	Bacillus_cereus	cysteine-desulfurase-DndA-[Bacillus-cereus]	1396
WP_098448197.1	cysteine-desulfurase	GCF_002564555.1	373	Bacteria>Terrabacteria_group>Firmicutes>Bacilli>Bacillales	Bacillus_cereus	cysteine-desulfurase-DndA-[Bacillus-cereus]	1396
WP_098492095.1	cysteine-desulfurase	GCF_002565455.1	373	Bacteria>Terrabacteria_group>Firmicutes>Bacilli>Bacillales	Bacillus_cereus	cysteine-desulfurase-DndA-[Bacillus-cereus]	1396
WP_098506338.1	cysteine-desulfurase	GCF_002578485.1	373	Bacteria>Terrabacteria_group>Firmicutes>Bacilli>Bacillales	Bacillus_cereus	cysteine-desulfurase-DndA-[Bacillus-cereus]	1396
WP_098540563.1	cysteine-desulfurase	GCF_002565815.1	373	Bacteria>Terrabacteria_group>Firmicutes>Bacilli>Bacillales	Bacillus_cereus	cysteine-desulfurase-DndA-[Bacillus-cereus]	1396
WP_098850027.1	cysteine-desulfurase	GCF_002582825.1	373	Bacteria>Terrabacteria_group>Firmicutes>Bacilli>Bacillales	Bacillus_cereus	cysteine-desulfurase-DndA-[Bacillus-cereus]	1396
WP_113759474.1	cysteine-desulfurase	GCF_012395345.1	373	Bacteria>Terrabacteria_group>Firmicutes>Bacilli>Bacillales	Bacillus_cereus	cysteine-desulfurase-DndA-[Bacillus-cereus]	1396
WP_200462569.1	cysteine-desulfurase	GCF_016632305.1	373	Bacteria>Terrabacteria_group>Firmicutes>Bacilli>Bacillales	Bacillus_cereus	cysteine-desulfurase-DndA-[Bacillus-cereus]	1396
WP_227601514.1	cysteine-desulfurase	GCF_020686345.1	373	Bacteria>Terrabacteria_group>Firmicutes>Bacilli>Bacillales	Bacillus_cereus_group	cysteine-desulfurase-DndA-[Bacillus-anthracis]	86661
WP_000966080.1	cysteine-desulfurase	GCF_000161155.1	373	Bacteria>Terrabacteria_group>Firmicutes>Bacilli>Bacillales	Bacillus_cereus_group	MULTISPECIES:-cysteine-desulfurase-[Bacillus-cereus-group]	86661
WP_070184207.1	cysteine-desulfurase	GCF_001757745.1	373	Bacteria>Terrabacteria_group>Firmicutes>Bacilli>Bacillales	Bacillus_cereus_group	MULTISPECIES:-cysteine-desulfurase-[Bacillus-cereus-group]	86661
WP_098213329.1	cysteine-desulfurase	GCF_002560525.1	373	Bacteria>Terrabacteria_group>Firmicutes>Bacilli>Bacillales	Bacillus_cereus_group	MULTISPECIES:-cysteine-desulfurase-[Bacillus-cereus-group]	86661
WP_000966082.1	cysteine-desulfurase	GCF_000161655.1	373	Bacteria>Terrabacteria_group>Firmicutes>Bacilli>Bacillales	Bacillus_cereus_group	MULTISPECIES:-cysteine-desulfurase-DndA-[Bacillus-cereus-group]	86661
WP_000966084.1	cysteine-desulfurase	GCF_001692675.1	373	Bacteria>Terrabacteria_group>Firmicutes>Bacilli>Bacillales	Bacillus_cereus_group	MULTISPECIES:-cysteine-desulfurase-DndA-[Bacillus-cereus-group]	86661
EEL22915.1	cysteine-desulfurase	GCA_000161155.1	373	Bacteria>Terrabacteria_group>Firmicutes>Bacilli>Bacillales	Bacillus_cereus_Rock1-3	Aminotransferase-class-V-[Bacillus-cereus-Rock1-3]	526981
WP_248138604.1	cysteine-desulfurase	GCF_023148655.1	372	Bacteria>Terrabacteria_group>Firmicutes>Bacilli>Bacillales	Bacillus_infantis	aminotransferase-class-V-fold-PLP-dependent-enzyme-[Bacillus-infantis]	324767
WP_000966081.1	cysteine-desulfurase	GCF_019837545.1	373	Bacteria>Terrabacteria_group>Firmicutes>Bacilli>Bacillales	Bacillus	MULTISPECIES:-cysteine-desulfurase-DndA-[Bacillus]	1386
QWI75854.1	cysteine-desulfurase	GCA_018741985.1	373	Bacteria>Terrabacteria_group>Firmicutes>Bacilli>Bacillales	Bacillus_mycoides	cysteine-desulfurase-DndA-[Bacillus-mycoides]	1405
WP_215580737.1	cysteine-desulfurase	GCF_018736705.1	373	Bacteria>Terrabacteria_group>Firmicutes>Bacilli>Bacillales	Bacillus_mycoides	cysteine-desulfurase-DndA-[Bacillus-mycoides]	1405
WP_086708043.1	cysteine-desulfurase	GCF_900176985.1	373	Bacteria>Terrabacteria_group>Firmicutes>Bacilli>Bacillales	Bacillus_paranthracis	cysteine-desulfurase-DndA-[Bacillus-paranthracis]	2026186
WP_150909073.1	cysteine-desulfurase	GCF_008764035.1	373	Bacteria>Terrabacteria_group>Firmicutes>Bacilli>Bacillales	Bacillus_pumilus	cysteine-desulfurase-DndA-[Bacillus-pumilus]	1408
MBY7111616.1	cysteine-desulfurase	GCA_019837545.1	373	Bacteria>Terrabacteria_group>Firmicutes>Bacilli>Bacillales	Bacillus_sp._17RED48	cysteine-desulfurase-DndA-[Bacillus-sp.-17RED48]	2778093
WP_125653838.1	cysteine-desulfurase	-	379	Bacteria>Terrabacteria_group>Firmicutes>Bacilli>Bacillales	Bacillus_sp._2B10	cysteine-desulfurase-[Bacillus-sp.-2B10]	2483355
VXB17314.1	cysteine-desulfurase	GCA_902506085.1	372	Bacteria>Terrabacteria_group>Firmicutes>Bacilli>Bacillales	Bacillus_sp._349Y	Cysteine-desulfurase-IscS-[Bacillus-sp.-349Y]	2653176
WP_095714463.1	cysteine-desulfurase	GCF_002289525.1	373	Bacteria>Terrabacteria_group>Firmicutes>Bacilli>Bacillales	Bacillus_sp._7705b	cysteine-desulfurase-DndA-[Bacillus-sp.-7705b]	2028568
WP_098118556.1	cysteine-desulfurase	GCF_002556365.1	374	Bacteria>Terrabacteria_group>Firmicutes>Bacilli>Bacillales	Bacillus_sp._AFS002410	aminotransferase-class-V-fold-PLP-dependent-enzyme-[Bacillus-sp.-AFS002410]	2033481
WP_098353853.1	cysteine-desulfurase	GCF_002561455.1	372	Bacteria>Terrabacteria_group>Firmicutes>Bacilli>Bacillales	Bacillus_sp._AFS015802	aminotransferase-class-V-fold-PLP-dependent-enzyme-[Bacillus-sp.-AFS015802]	2033486
WP_098667783.1	cysteine-desulfurase	GCF_002571225.1	373	Bacteria>Terrabacteria_group>Firmicutes>Bacilli>Bacillales	Bacillus_sp._AFS059628	cysteine-desulfurase-DndA-[Bacillus-sp.-AFS059628]	2033508
WP_152127739.1	cysteine-desulfurase	GCF_009183805.1	373	Bacteria>Terrabacteria_group>Firmicutes>Bacilli>Bacillales	Bacillus_sp._B2-WWTP-C-10-Post-4	cysteine-desulfurase-[Bacillus-sp.-B2-WWTP-C-10-Post-4]	2653218
WP_149237993.1	cysteine-desulfurase	GCF_008368845.1	373	Bacteria>Terrabacteria_group>Firmicutes>Bacilli>Bacillales	Bacillus_sp._BB51/4	cysteine-desulfurase-DndA-[Bacillus-sp.-BB51/4]	2217819
TCN11073.1	cysteine-desulfurase	GCA_004345465.1	372	Bacteria>Terrabacteria_group>Firmicutes>Bacilli>Bacillales	Bacillus_sp._BK006	cysteine-desulfurase-[Bacillus-sp.-BK006]	2512183
WP_043934650.1	cysteine-desulfurase	GCF_000613125.1	372	Bacteria>Terrabacteria_group>Firmicutes>Bacilli>Bacillales	Bacillus_sp._EB01	aminotransferase-class-V-fold-PLP-dependent-enzyme-[Bacillus-sp.-EB01]	1347086
WP_053599595.1	cysteine-desulfurase	GCF_001278805.1	372	Bacteria>Terrabacteria_group>Firmicutes>Bacilli>Bacillales	Bacillus_sp._FJAT-18017	cysteine-desulfurase-[Bacillus-sp.-FJAT-18017]	1705566
KQL33520.1	cysteine-desulfurase	GCA_001420605.1	374	Bacteria>Terrabacteria_group>Firmicutes>Bacilli>Bacillales	Bacillus_sp._FJAT-25509	cysteine-desulfurase-[Bacillus-sp.-FJAT-25509]	1712029
WP_213122445.1	cysteine-desulfurase	GCF_018343525.1	372	Bacteria>Terrabacteria_group>Firmicutes>Bacilli>Bacillales	Bacillus_sp._FJAT-49754	aminotransferase-class-V-fold-PLP-dependent-enzyme-[Bacillus-sp.-FJAT-49754]	2833581
MBR3379966.1	cysteine-desulfurase	GCA_017509945.1	385	Bacteria>Terrabacteria_group>Firmicutes>Bacilli>Bacillales	Bacillus_sp._(in:_Bacteria)	aminotransferase-class-V-fold-PLP-dependent-enzyme-[Bacillus-sp.-(in:-Bacteria)]	1409
WP_026584152.1	cysteine-desulfurase	GCF_000518885.1	372	Bacteria>Terrabacteria_group>Firmicutes>Bacilli>Bacillales	Bacillus_sp._J33	aminotransferase-class-V-fold-PLP-dependent-enzyme-[Bacillus-sp.-J33]	935836
WP_102345894.1	cysteine-desulfurase	GCF_900240995.1	372	Bacteria>Terrabacteria_group>Firmicutes>Bacilli>Bacillales	Bacillus_sp._Marseille-P3661	aminotransferase-class-V-fold-PLP-dependent-enzyme-[Bacillus-sp.-Marseille-P3661]	1936234
OAT86001.1	cysteine-desulfurase	GCA_001655735.1	372	Bacteria>Terrabacteria_group>Firmicutes>Bacilli>Bacillales	Bacillus_sp._MKU004	cysteine-desulfurase-DndA-[Bacillus-sp.-MKU004]	1849370
RFB11802.1	cysteine-desulfurase	GCA_003399505.1	373	Bacteria>Terrabacteria_group>Firmicutes>Bacilli>Bacillales	Bacillus_sp._OE	cysteine-desulfurase-DndA-[Bacillus-sp.-OE]	2293320
WP_116349151.1	cysteine-desulfurase	GCF_003399505.1	373	Bacteria>Terrabacteria_group>Firmicutes>Bacilli>Bacillales	Bacillus_sp._OE	cysteine-desulfurase-DndA-[Bacillus-sp.-OE]	2293320
NMH73568.1	cysteine-desulfurase	GCA_012911845.1	372	Bacteria>Terrabacteria_group>Firmicutes>Bacilli>Bacillales	Bacillus_sp._RO2	cysteine-desulfurase-[Bacillus-sp.-RO2]	2723913
WP_169291164.1	cysteine-desulfurase	GCF_012911845.1	372	Bacteria>Terrabacteria_group>Firmicutes>Bacilli>Bacillales	Bacillus_sp._RO2	cysteine-desulfurase-[Bacillus-sp.-RO2]	2723913
QHT48500.1	cysteine-desulfurase	GCA_000469135.2	372	Bacteria>Terrabacteria_group>Firmicutes>Bacilli>Bacillales	Bacillus_sp._SB49	cysteine-desulfurase-[Bacillus-sp.-SB49]	1071080
WP_026577665.1	cysteine-desulfurase	GCF_000469135.2	372	Bacteria>Terrabacteria_group>Firmicutes>Bacilli>Bacillales	Bacillus_sp._SB49	cysteine-desulfurase-[Bacillus-sp.-SB49]	1071080
WP_119708123.1	cysteine-desulfurase	GCF_003586445.1	372	Bacteria>Terrabacteria_group>Firmicutes>Bacilli>Bacillales	Bacillus_sp._Y1	aminotransferase-class-V-fold-PLP-dependent-enzyme-[Bacillus-sp.-Y1]	352858
WP_069837229.1	cysteine-desulfurase	GCF_001747445.1	373	Bacteria>Terrabacteria_group>Firmicutes>Bacilli>Bacillales	Bacillus_subtilis	cysteine-desulfurase-DndA-[Bacillus-subtilis]	1423
WP_103031516.1	cysteine-desulfurase	GCF_002893805.1	373	Bacteria>Terrabacteria_group>Firmicutes>Bacilli>Bacillales	Bacillus_subtilis	cysteine-desulfurase-DndA-[Bacillus-subtilis]	1423

Accession	Annotation	GCA	Length	Phyletic.spread	Strain.name	Defline	Species.Taxid
ALQ66681.1	cysteine-desulfurase	GCA_001455345.1	373	Bacteria>Terrabacteria_group>Firmicutes>Bacilli>Bacillales	Bacillus_thuringiensis	cysteine-desulfurase-DndA-[Bacillus-thuringiensis]	1428
PFJ44434.1	cysteine-desulfurase	GCA_002566135.1	373	Bacteria>Terrabacteria_group>Firmicutes>Bacilli>Bacillales	Bacillus_thuringiensis	cysteine-desulfurase-DndA-[Bacillus-thuringiensis]	1428
PFN50594.1	cysteine-desulfurase	GCA_002565105.1	373	Bacteria>Terrabacteria_group>Firmicutes>Bacilli>Bacillales	Bacillus_thuringiensis	cysteine-desulfurase-DndA-[Bacillus-thuringiensis]	1428
PFV26125.1	cysteine-desulfurase	GCA_002570925.1	373	Bacteria>Terrabacteria_group>Firmicutes>Bacilli>Bacillales	Bacillus_thuringiensis	cysteine-desulfurase-DndA-[Bacillus-thuringiensis]	1428
WP_060629758.1	cysteine-desulfurase	GCF_001455345.1	373	Bacteria>Terrabacteria_group>Firmicutes>Bacilli>Bacillales	Bacillus_thuringiensis	cysteine-desulfurase-DndA-[Bacillus-thuringiensis]	1428
WP_086388845.1	cysteine-desulfurase	GCF_002147655.1	373	Bacteria>Terrabacteria_group>Firmicutes>Bacilli>Bacillales	Bacillus_thuringiensis	cysteine-desulfurase-DndA-[Bacillus-thuringiensis]	1428
WP_098516459.1	cysteine-desulfurase	GCF_002566135.1	373	Bacteria>Terrabacteria_group>Firmicutes>Bacilli>Bacillales	Bacillus_thuringiensis	cysteine-desulfurase-DndA-[Bacillus-thuringiensis]	1428
WP_098675169.1	cysteine-desulfurase	GCF_002576925.1	373	Bacteria>Terrabacteria_group>Firmicutes>Bacilli>Bacillales	Bacillus_thuringiensis	cysteine-desulfurase-DndA-[Bacillus-thuringiensis]	1428
WP_144476258.1	cysteine-desulfurase	GCF_007676905.1	373	Bacteria>Terrabacteria_group>Firmicutes>Bacilli>Bacillales	Bacillus_thuringiensis	cysteine-desulfurase-DndA-[Bacillus-thuringiensis]	1428
OUA58852.1	cysteine-desulfurase	GCA_002147655.1	373	Bacteria>Terrabacteria_group>Firmicutes>Bacilli>Bacillales	Bacillus_thuringiensis_serovar_aizawai	cysteine-desulfurase-DndA-[Bacillus-thuringiensis-serovar-aizawai]	1433
OUA03845.1	cysteine-desulfurase	GCA_002147505.1	373	Bacteria>Terrabacteria_group>Firmicutes>Bacilli>Bacillales	Bacillus_thuringiensis_serovar_finitimus	cysteine-desulfurase-DndA-[Bacillus-thuringiensis-serovar-finitimus]	29337
WP_098908841.1	cysteine-desulfurase	GCF_002583715.1	373	Bacteria>Terrabacteria_group>Firmicutes>Bacilli>Bacillales	Bacillus_toyonensis	cysteine-desulfurase-[Bacillus-toyonensis]	155322
WP_224993405.1	cysteine-desulfurase	GCF_020172005.1	373	Bacteria>Terrabacteria_group>Firmicutes>Bacilli>Bacillales	Bacillus_toyonensis	cysteine-desulfurase-[Bacillus-toyonensis]	155322
PHG68926.1	cysteine-desulfurase	GCA_002589375.1	373	Bacteria>Terrabacteria_group>Firmicutes>Bacilli>Bacillales	Bacillus_toyonensis	cysteine-desulfurase-DndA-[Bacillus-toyonensis]	155322
WP_098954254.1	cysteine-desulfurase	GCF_002589375.1	373	Bacteria>Terrabacteria_group>Firmicutes>Bacilli>Bacillales	Bacillus_toyonensis	cysteine-desulfurase-DndA-[Bacillus-toyonensis]	155322
EJV69666.1	cysteine-desulfurase	GCA_000293565.1	373	Bacteria>Terrabacteria_group>Firmicutes>Bacilli>Bacillales	Bacillus_wiedmannii	cysteine-desulfurase-DndA-[Bacillus-wiedmannii]	1890302
PEO17477.1	cysteine-desulfurase	GCA_002556915.1	373	Bacteria>Terrabacteria_group>Firmicutes>Bacilli>Bacillales	Bacillus_wiedmannii	cysteine-desulfurase-DndA-[Bacillus-wiedmannii]	1890302
WP_097994022.1	cysteine-desulfurase	GCF_002551545.1	373	Bacteria>Terrabacteria_group>Firmicutes>Bacilli>Bacillales	Bacillus_wiedmannii	cysteine-desulfurase-DndA-[Bacillus-wiedmannii]	1890302
WP_098048623.1	cysteine-desulfurase	GCF_002556405.1	373	Bacteria>Terrabacteria_group>Firmicutes>Bacilli>Bacillales	Bacillus_wiedmannii	cysteine-desulfurase-DndA-[Bacillus-wiedmannii]	1890302
WP_213421299.1	cysteine-desulfurase	GCF_018390575.1	372	Bacteria>Terrabacteria_group>Firmicutes>Bacilli>Bacillales	Bhargavaea_massiliensis	aminotransferase-class-V-fold-PLP-dependent-enzyme-[Bhargavaea-sp.-Marseille-Q1000]	2697500
WP_122953194.1	cysteine-desulfurase	GCF_006540025.1	375	Bacteria>Terrabacteria_group>Firmicutes>Bacilli>Bacillales	Brevibacillus_agri	cysteine-desulfurase-[Brevibacillus-agri]	51101
WP_139490101.1	cysteine-desulfurase	GCF_006170445.1	372	Bacteria>Terrabacteria_group>Firmicutes>Bacilli>Bacillales	Brevibacillus_sp._CHY01	cysteine-desulfurase-DndA-[Brevibacillus-sp.-CHY01]	1844116
HBZ83221.1	cysteine-desulfurase	GCA_003521185.1	375	Bacteria>Terrabacteria_group>Firmicutes>Bacilli>Bacillales	Brevibacillus_sp.	cysteine-desulfurase-DndA-[Brevibacillus-sp.]	1882945
WP_061568181.1	cysteine-desulfurase	GCF_001587535.1	372	Bacteria>Terrabacteria_group>Firmicutes>Bacilli>Bacillales	Caldibacillus_debilis	cysteine-desulfurase-[Caldibacillus-debilis]	301148
KYD22011.1	cysteine-desulfurase	GCA_001587535.1	372	Bacteria>Terrabacteria_group>Firmicutes>Bacilli>Bacillales	Caldibacillus_debilis	Cysteine-desulfurase-[Caldibacillus-debilis]	301148
WP_066465827.1	cysteine-desulfurase	GCF_001700325.1	372	Bacteria>Terrabacteria_group>Firmicutes>Bacilli>Bacillales	Caryophanon_latum	cysteine-desulfurase-[Caryophanon-latum]	33977
RIE01529.1	cysteine-desulfurase	GCA_003570905.1	377	Bacteria>Terrabacteria_group>Firmicutes>Bacilli>Bacillales	Cohnella_faecalis	aminotransferase-class-V-fold-PLP-dependent-enzyme-[Cohnella-faecalis]	2315694
WP_035333442.1	cysteine-desulfurase	GCF_000565285.1	372	Bacteria>Terrabacteria_group>Firmicutes>Bacilli>Bacillales	Cytobacillus_firmus	aminotransferase-class-V-fold-PLP-dependent-enzyme-[Cytobacillus-firmus]	1399
WP_058763147.1	cysteine-desulfurase	GCF_001482445.1	372	Bacteria>Terrabacteria_group>Firmicutes>Bacilli>Bacillales	Exiguobacterium_chiriqhucha	cysteine-desulfurase-[Exiguobacterium-chiriqhucha]	1385984
WP_075642723.1	cysteine-desulfurase	GCF_001939065.1	373	Bacteria>Terrabacteria_group>Firmicutes>Bacilli>Bacillales	Exiguobacterium_indicum	cysteine-desulfurase-[Exiguobacterium-indicum]	296995
WP_159174154.1	cysteine-desulfurase	GCF_902506315.1	373	Bacteria>Terrabacteria_group>Firmicutes>Bacilli>Bacillales	Exiguobacterium_sp._9Y	cysteine-desulfurase-[Exiguobacterium-sp.-9Y]	2653141
WP_034809837.1	cysteine-desulfurase	GCF_000696055.1	372	Bacteria>Terrabacteria_group>Firmicutes>Bacilli>Bacillales	Exiguobacterium_sp._AB2	cysteine-desulfurase-[Exiguobacterium-sp.-AB2]	1484479
WP_128122843.1	cysteine-desulfurase	GCF_003467445.1	372	Bacteria>Terrabacteria_group>Firmicutes>Bacilli>Bacillales	Exiguobacterium_sp._AM39-5BH	cysteine-desulfurase-[Exiguobacterium-sp.-AM39-5BH]	2292355
WP_015881064.1	cysteine-desulfurase	GCF_000023045.1	372	Bacteria>Terrabacteria_group>Firmicutes>Bacilli>Bacillales	Exiguobacterium_sp._AT1b	cysteine-desulfurase-[Exiguobacterium-sp.-AT1b]	360911
ACQ71505.1	cysteine-desulfurase	GCA_000023045.1	372	Bacteria>Terrabacteria_group>Firmicutes>Bacilli>Bacillales	Exiguobacterium_sp._AT1b	Cysteine-desulfurase-[Exiguobacterium-sp.-AT1b]	360911
WP_214875262.1	cysteine-desulfurase	GCF_018617635.1	372	Bacteria>Terrabacteria_group>Firmicutes>Bacilli>Bacillales	Exiguobacterium_sp._CH10	aminotransferase-class-V-fold-PLP-dependent-enzyme-[Exiguobacterium-sp.-CH10]	2751261
WP_211781521.1	cysteine-desulfurase	GCF_018255795.1	372	Bacteria>Terrabacteria_group>Firmicutes>Bacilli>Bacillales	Exiguobacterium_sp._PFWT01	aminotransferase-class-V-fold-PLP-dependent-enzyme-[Exiguobacterium-sp.-PFWT01]	2829816
WP_114596997.1	cysteine-desulfurase	GCF_003339585.1	373	Bacteria>Terrabacteria_group>Firmicutes>Bacilli>Bacillales	Exiguobacterium_sp._RIT594	cysteine-desulfurase-[Exiguobacterium-sp.-RIT594]	2282449
WP_214789493.1	cysteine-desulfurase	GCF_018617485.1	372	Bacteria>Terrabacteria_group>Firmicutes>Bacilli>Bacillales	Exiguobacterium_sp._s124	aminotransferase-class-V-fold-PLP-dependent-enzyme-[Exiguobacterium-sp.-s124]	2751268
WP_214811342.1	cysteine-desulfurase	GCF_018618695.1	373	Bacteria>Terrabacteria_group>Firmicutes>Bacilli>Bacillales	Exiguobacterium_sp._s127	aminotransferase-class-V-fold-PLP-dependent-enzyme-[Exiguobacterium-sp.-s127]	2751210
WP_214817549.1	cysteine-desulfurase	GCF_018617345.1	372	Bacteria>Terrabacteria_group>Firmicutes>Bacilli>Bacillales	Exiguobacterium_sp._s131	aminotransferase-class-V-fold-PLP-dependent-enzyme-[Exiguobacterium-sp.-s131]	2751278
WP_214850762.1	cysteine-desulfurase	GCF_018618775.1	373	Bacteria>Terrabacteria_group>Firmicutes>Bacilli>Bacillales	Exiguobacterium_sp._s138	aminotransferase-class-V-fold-PLP-dependent-enzyme-[Exiguobacterium-sp.-s138]	2751202
WP_214695185.1	cysteine-desulfurase	GCF_018617575.1	372	Bacteria>Terrabacteria_group>Firmicutes>Bacilli>Bacillales	Exiguobacterium_sp._s160	aminotransferase-class-V-fold-PLP-dependent-enzyme-[Exiguobacterium-sp.-s160]	2751265
WP_214782479.1	cysteine-desulfurase	GCF_018617295.1	372	Bacteria>Terrabacteria_group>Firmicutes>Bacilli>Bacillales	Exiguobacterium_sp._S3	aminotransferase-class-V-fold-PLP-dependent-enzyme-[Exiguobacterium-sp.-S3]	483245
WP_215085017.1	cysteine-desulfurase	GCF_018618985.1	373	Bacteria>Terrabacteria_group>Firmicutes>Bacilli>Bacillales	Exiguobacterium_sp._s78	aminotransferase-class-V-fold-PLP-dependent-enzyme-[Exiguobacterium-sp.-s78]	2751197
WP_131443479.1	cysteine-desulfurase	GCF_004337195.1	372	Bacteria>Terrabacteria_group>Firmicutes>Bacilli>Bacillales	Exiguobacterium_sp._SH0S7	cysteine-desulfurase-[Exiguobacterium-sp.-SH0S7]	2510951
WP_131457468.1	cysteine-desulfurase	GCF_004337045.1	372	Bacteria>Terrabacteria_group>Firmicutes>Bacilli>Bacillales	Exiguobacterium_sp._SH5S4	cysteine-desulfurase-[Exiguobacterium-sp.-SH5S4]	2510961
WP_131433387.1	cysteine-desulfurase	GCF_004337025.1	372	Bacteria>Terrabacteria_group>Firmicutes>Bacilli>Bacillales	Exiguobacterium_sp._SL-10	cysteine-desulfurase-[Exiguobacterium-sp.-SL-10]	2510962
WP_222462164.1	cysteine-desulfurase	GCF_019801215.1	373	Bacteria>Terrabacteria_group>Firmicutes>Bacilli>Bacillales	Fictibacillus_nanhaiensis	aminotransferase-class-V-fold-PLP-dependent-enzyme-[Fictibacillus-nanhaiensis]	742169
WP_099353871.1	cysteine-desulfurase	GCF_002734285.1	372	Bacteria>Terrabacteria_group>Firmicutes>Bacilli>Bacillales	Fredinandcohnia_onubensis	aminotransferase-class-V-fold-PLP-dependent-enzyme-[Fredinandcohnia-onubensis]	1571209
WP_044736684.1	cysteine-desulfurase	GCF_000948165.1	382	Bacteria>Terrabacteria_group>Firmicutes>Bacilli>Bacillales	Geobacillus	MULTISPECIES:-cysteine-desulfurase-[Geobacillus]	129337
QNU31668.1	cysteine-desulfurase	GCA_014679705.1	382	Bacteria>Terrabacteria_group>Firmicutes>Bacilli>Bacillales	Geobacillus_sp._47C-IIb	cysteine-desulfurase-[Geobacillus-sp.-47C-IIb]	1963026
WP_190300655.1	cysteine-desulfurase	GCF_014679705.1	382	Bacteria>Terrabacteria_group>Firmicutes>Bacilli>Bacillales	Geobacillus_sp._47C-IIb	cysteine-desulfurase-[Geobacillus-sp.-47C-IIb]	1963026
AGE22668.1	cysteine-desulfurase	GCA_000336445.1	382	Bacteria>Terrabacteria_group>Firmicutes>Bacilli>Bacillales	Geobacillus_sp._GHH01	cysteine-desulfurase-[Geobacillus-sp.-GHH01]	1233873
WP_015375165.1	cysteine-desulfurase	GCF_000336445.1	382	Bacteria>Terrabacteria_group>Firmicutes>Bacilli>Bacillales	Geobacillus_sp._GHH01	cysteine-desulfurase-[Geobacillus-sp.-GHH01]	1233873
WP_060476347.1	cysteine-desulfurase	GCF_001412125.1	382	Bacteria>Terrabacteria_group>Firmicutes>Bacilli>Bacillales	Geobacillus_sp._PA-3	cysteine-desulfurase-[Geobacillus-sp.-PA-3]	1699078
WP_129447535.1	cysteine-desulfurase	GCF_004123125.1	382	Bacteria>Terrabacteria_group>Firmicutes>Bacilli>Bacillales	Geobacillus_sp._PK12	cysteine-desulfurase-[Geobacillus-sp.-PK12]	2508525
PJW18180.1	cysteine-desulfurase	GCA_002809985.1	382	Bacteria>Terrabacteria_group>Firmicutes>Bacilli>Bacillales	Geobacillus_sp._WSUCF-018B	cysteine-desulfurase-DndA-[Geobacillus-sp.-WSUCF-018B]	2055939
WP_100663902.1	cysteine-desulfurase	GCF_002809985.1	382	Bacteria>Terrabacteria_group>Firmicutes>Bacilli>Bacillales	Geobacillus_sp._WSUCF-018B	cysteine-desulfurase-[Geobacillus-sp.-WSUCF-018B]	2055939
ALA71632.1	cysteine-desulfurase	GCA_001274575.1	382	Bacteria>Terrabacteria_group>Firmicutes>Bacilli>Bacillales	Geobacillus_stearothermophilus_10	cysteine-desulfurase-[Geobacillus-stearothermophilus-10]	272567
WP_121678333.1	cysteine-desulfurase	GCF_003667605.1	382	Bacteria>Terrabacteria_group>Firmicutes>Bacilli>Bacillales	Geobacillus_stearothermophilus	cysteine-desulfurase-[Geobacillus-stearothermophilus]	1422
KAF6510291.1	cysteine-desulfurase	GCA_014133975.1	382	Bacteria>Terrabacteria_group>Firmicutes>Bacilli>Bacillales	Geobacillus_stearothermophilus	Cysteine-desulfurase-[Geobacillus-stearothermophilus]	1422
KZE95795.1	cysteine-desulfurase	GCA_001620045.1	382	Bacteria>Terrabacteria_group>Firmicutes>Bacilli>Bacillales	Geobacillus_stearothermophilus	Cysteine-desulfurase-IscS-[Geobacillus-stearothermophilus]	1422
AMV11253.1	cysteine-desulfurase	GCA_001610955.1	382	Bacteria>Terrabacteria_group>Firmicutes>Bacilli>Bacillales	Geobacillus_thermoleovorans	cysteine-desulfurase-DndA-[Geobacillus-thermoleovorans]	33941
WP_069034267.1	cysteine-desulfurase	GCF_001712755.1	374	Bacteria>Terrabacteria_group>Firmicutes>Bacilli>Bacillales	Gottfriedia_luciferensis	aminotransferase-class-V-fold-PLP-dependent-enzyme-[Gottfriedia-luciferensis]	178774
ODG91507.1	cysteine-desulfurase	GCA_001712755.1	374	Bacteria>Terrabacteria_group>Firmicutes>Bacilli>Bacillales	Gottfriedia_luciferensis	cysteine-desulfurase-DndA-[Gottfriedia-luciferensis]	178774
WP_146813145.1	cysteine-desulfurase	GCF_007991335.1	373	Bacteria>Terrabacteria_group>Firmicutes>Bacilli>Bacillales	Halobacillus_faecis	cysteine-desulfurase-[Halobacillus-faecis]	360184
MYL22003.1	cysteine-desulfurase	GCA_009856285.1	373	Bacteria>Terrabacteria_group>Firmicutes>Bacilli>Bacillales	Halobacillus_litoralis	aminotransferase-class-V-fold-PLP-dependent-enzyme-[Halobacillus-litoralis]	45668
WP_160839921.1	cysteine-desulfurase	GCF_009856285.1	373	Bacteria>Terrabacteria_group>Firmicutes>Bacilli>Bacillales	Halobacillus_litoralis	cysteine-desulfurase-[Halobacillus-litoralis]	45668
WP_079478866.1	cysteine-desulfurase	GCF_900166575.1	372	Bacteria>Terrabacteria_group>Firmicutes>Bacilli>Bacillales	Halobacillus_salinus	cysteine-desulfurase-[Halobacillus-salinus]	192814
WP_089830322.1	cysteine-desulfurase	GCF_007990285.1	372	Bacteria>Terrabacteria_group>Firmicutes>Bacilli>Bacillales	Halolactibacillus_halophilus	cysteine-desulfurase-[Halolactibacillus-halophilus]	306540
WP_126344263.1	cysteine-desulfurase	GCF_900637935.1	372	Bacteria>Terrabacteria_group>Firmicutes>Bacilli>Bacillales	Kurthia_zopfii	aminotransferase-class-V-fold-PLP-dependent-enzyme-[Kurthia-zopfii]	1650
WP_100667279.1	cysteine-desulfurase	GCF_002804065.1	375	Bacteria>Terrabacteria_group>Firmicutes>Bacilli>Bacillales	Kyrpidia_spormannii	aminotransferase-class-V-fold-PLP-dependent-enzyme-[Kyrpidia-spormannii]	2055160
ATY84459.1	cysteine-desulfurase	GCA_002804065.1	375	Bacteria>Terrabacteria_group>Firmicutes>Bacilli>Bacillales	Kyrpidia_spormannii	cysteine-desulfurase-DndA-[Kyrpidia-spormannii]	2055160
WP_188614720.1	cysteine-desulfurase	GCF_014643595.1	372	Bacteria>Terrabacteria_group>Firmicutes>Bacilli>Bacillales	Lysinibacillus_alkalisoli	cysteine-desulfurase-[Lysinibacillus-alkalisoli]	1911548
WP_181574608.1	cysteine-desulfurase	GCF_900445455.1	372	Bacteria>Terrabacteria_group>Firmicutes>Bacilli>Bacillales	Lysinibacillus_capsici	aminotransferase-class-V-fold-PLP-dependent-enzyme-[Lysinibacillus-capsici]	2115968
WP_193832997.1	cysteine-desulfurase	GCF_015161405.1	372	Bacteria>Terrabacteria_group>Firmicutes>Bacilli>Bacillales	Lysinibacillus_fusiformis	aminotransferase-class-V-fold-PLP-dependent-enzyme-[Lysinibacillus-fusiformis]	28031
WP_079476231.1	cysteine-desulfurase	GCF_900166605.1	373	Bacteria>Terrabacteria_group>Firmicutes>Bacilli>Bacillales	Marinococcus_halophilus	aminotransferase-class-V-fold-PLP-dependent-enzyme-[Marinococcus-halophilus]	1371
WP_046522703.1	cysteine-desulfurase	GCF_000986785.1	372	Bacteria>Terrabacteria_group>Firmicutes>Bacilli>Bacillales	Mesobacillus_campisalis	aminotransferase-class-V-fold-PLP-dependent-enzyme-[Mesobacillus-campisalis]	1408103
WP_066227724.1	cysteine-desulfurase	GCF_001591625.1	373	Bacteria>Terrabacteria_group>Firmicutes>Bacilli>Bacillales	Metabacillus_fastidiosus	cysteine-desulfurase-DndA-[Metabacillus-fastidiosus]	1458
WP_154308355.1	cysteine-desulfurase	GCF_009674805.1	372	Bacteria>Terrabacteria_group>Firmicutes>Bacilli>Bacillales	Metabacillus_lacus	aminotransferase-class-V-fold-PLP-dependent-enzyme-[Bacillus-lacus]	1983721
WP_144568029.1	cysteine-desulfurase	GCF_007828895.1	372	Bacteria>Terrabacteria_group>Firmicutes>Bacilli>Bacillales	Neobacillus_bataviensis	cysteine-desulfurase-[Neobacillus-bataviensis]	220685
WP_235744139.1	cysteine-desulfurase	GCF_021560175.1	372	Bacteria>Terrabacteria_group>Firmicutes>Bacilli>Bacillales	Neobacillus_drentensis	aminotransferase-class-V-fold-PLP-dependent-enzyme-[Neobacillus-drentensis]	220684
QAV26940.1	cysteine-desulfurase	GCA_004116995.1	382	Bacteria>Terrabacteria_group>Firmicutes>Bacilli>Bacillales	Neobacillus_thermocopriae	cysteine-desulfurase-DndA-[Neobacillus-thermocopriae]	1215031
WP_127547365.1	cysteine-desulfurase	GCF_004001025.1	377	Bacteria>Terrabacteria_group>Firmicutes>Bacilli>Bacillales	Paenibacillus_amylolyticus	aminotransferase-class-V-fold-PLP-dependent-enzyme-[Paenibacillus-amylolyticus]	1451
WP_076332998.1	cysteine-desulfurase	GCF_001956055.1	377	Bacteria>Terrabacteria_group>Firmicutes>Bacilli>Bacillales	Paenibacillus_amylolyticus	cysteine-desulfurase-[Paenibacillus-amylolyticus]	1451
WP_133386832.1	cysteine-desulfurase	GCF_004358835.1	377	Bacteria>Terrabacteria_group>Firmicutes>Bacilli>Bacillales	Paenibacillus_amylolyticus	cysteine-desulfurase-[Paenibacillus-amylolyticus]	1451
WP_134757399.1	cysteine-desulfurase	GCF_004514475.2	378	Bacteria>Terrabacteria_group>Firmicutes>Bacilli>Bacillales	Paenibacillus_athensensis	cysteine-desulfurase-[Paenibacillus-athensensis]	1967502
WP_216515417.1	cysteine-desulfurase	GCF_018917465.1	376	Bacteria>Terrabacteria_group>Firmicutes>Bacilli>Bacillales	Paenibacillus_barcinonensis	aminotransferase-class-V-fold-PLP-dependent-enzyme-[Paenibacillus-barcinonensis]	198119
WP_095267898.1	cysteine-desulfurase	GCF_002272015.1	377	Bacteria>Terrabacteria_group>Firmicutes>Bacilli>Bacillales	Paenibacillus_campinasensis	aminotransferase-class-V-fold-PLP-dependent-enzyme-[Paenibacillus-campinasensis]	66347
WP_135556451.1	cysteine-desulfurase	GCF_004764705.1	384	Bacteria>Terrabacteria_group>Firmicutes>Bacilli>Bacillales	Paenibacillus_cymbidii	cysteine-desulfurase-[Paenibacillus-cymbidii]	1639034
WP_046228171.1	cysteine-desulfurase	GCF_000971985.1	377	Bacteria>Terrabacteria_group>Firmicutes>Bacilli>Bacillales	Paenibacillus_dauci	aminotransferase-class-V-fold-PLP-dependent-enzyme-[Paenibacillus-dauci]	1567106
WP_127484305.1	cysteine-desulfurase	GCF_004000785.1	374	Bacteria>Terrabacteria_group>Firmicutes>Bacilli>Bacillales	Paenibacillus_ehimensis	aminotransferase-class-V-fold-PLP-dependent-enzyme-[Paenibacillus-ehimensis]	79264
WP_163853502.1	cysteine-desulfurase	GCF_010894335.1	386	Bacteria>Terrabacteria_group>Firmicutes>Bacilli>Bacillales	Paenibacillus_elgii	cysteine-desulfurase-[Paenibacillus-elgii]	189691
WP_227793671.1	cysteine-desulfurase	GCF_009363075.1	374	Bacteria>Terrabacteria_group>Firmicutes>Bacilli>Bacillales	Paenibacillus_guangzhouensis	aminotransferase-class-V-fold-PLP-dependent-enzyme-[Paenibacillus-guangzhouensis]	1473112
WP_013920771.1	cysteine-desulfurase	GCF_000218915.1	377	Bacteria>Terrabacteria_group>Firmicutes>Bacilli>Bacillales	Paenibacillus_mucilaginosus	cysteine-desulfurase-[Paenibacillus-mucilaginosus]	61624
WP_076212784.1	cysteine-desulfurase	GCF_001954655.1	375	Bacteria>Terrabacteria_group>Firmicutes>Bacilli>Bacillales	Paenibacillus_odorifer	cysteine-desulfurase-[Paenibacillus-odorifer]	189426
WP_069012347.1	cysteine-desulfurase	GCF_017589695.1	376	Bacteria>Terrabacteria_group>Firmicutes>Bacilli>Bacillales	Paenibacillus_polymyxa	aminotransferase-class-V-fold-PLP-dependent-enzyme-[Paenibacillus-polymyxa]	1406
WP_111149656.1	cysteine-desulfurase	GCF_003233845.1	376	Bacteria>Terrabacteria_group>Firmicutes>Bacilli>Bacillales	Paenibacillus_sambharensis	cysteine-desulfurase-[Paenibacillus-sambharensis]	1803190
WP_137221265.1	cysteine-desulfurase	GCF_005233705.1	377	Bacteria>Terrabacteria_group>Firmicutes>Bacilli>Bacillales	Paenibacillus_sp._CFBP13512	aminotransferase-class-V-fold-PLP-dependent-enzyme-[Paenibacillus-sp.-CFBP13512]	2184007
WP_192132707.1	cysteine-desulfurase	GCF_014842675.1	377	Bacteria>Terrabacteria_group>Firmicutes>Bacilli>Bacillales	Paenibacillus_sp._CFBP_13594	aminotransferase-class-V-fold-PLP-dependent-enzyme-[Paenibacillus-sp.-CFBP-13594]	2774037
GFR37182.1	cysteine-desulfurase	GCA_015472005.1	374	Bacteria>Terrabacteria_group>Firmicutes>Bacilli>Bacillales	Paenibacillus_sp._DA-C8	cysteine-desulfurase-IscS-[Paenibacillus-sp.-DA-C8]	2749268
WP_200965463.1	cysteine-desulfurase	GCF_015472005.1	374	Bacteria>Terrabacteria_group>Firmicutes>Bacilli>Bacillales	Paenibacillus_sp._DA-C8	cysteine-desulfurase-[Paenibacillus-sp.-DA-C8]	2749268
WP_236420995.1	cysteine-desulfurase	GCF_927798175.1	377	Bacteria>Terrabacteria_group>Firmicutes>Bacilli>Bacillales	Paenibacillus_sp._JJ-223	aminotransferase-class-V-fold-PLP-dependent-enzyme-[Paenibacillus-sp.-JJ-223]	2905647
WP_056694698.1	cysteine-desulfurase	GCF_001426865.1	377	Bacteria>Terrabacteria_group>Firmicutes>Bacilli>Bacillales	Paenibacillus_sp._Root52	aminotransferase-class-V-fold-PLP-dependent-enzyme-[Paenibacillus-sp.-Root52]	1736552
WP_220030859.1	cysteine-desulfurase	GCF_019454085.1	377	Bacteria>Terrabacteria_group>Firmicutes>Bacilli>Bacillales	Paenibacillus_sp._S25	aminotransferase-class-V-fold-PLP-dependent-enzyme-[Paenibacillus-sp.-S25]	2823905
WP_216791337.1	cysteine-desulfurase	GCF_018998565.1	377	Bacteria>Terrabacteria_group>Firmicutes>Bacilli>Bacillales	Paenibacillus_sp._SM_69	aminotransferase-class-V-fold-PLP-dependent-enzyme-[Paenibacillus-sp.-SM-69]	2594886
MBU7315387.1	cysteine-desulfurase	GCA_018998565.1	377	Bacteria>Terrabacteria_group>Firmicutes>Bacilli>Bacillales	Paenibacillus_sp._SM_69	cysteine-desulfurase-[Paenibacillus-sp.-SM-69]	2594886
WP_057311493.1	cysteine-desulfurase	GCF_001428105.1	377	Bacteria>Terrabacteria_group>Firmicutes>Bacilli>Bacillales	Paenibacillus_sp._Soil766	cysteine-desulfurase-[Paenibacillus-sp.-Soil766]	1736404
WP_220350283.1	cysteine-desulfurase	GCF_019550795.1	376	Bacteria>Terrabacteria_group>Firmicutes>Bacilli>Bacillales	Paenibacillus_sp._sptzw28	aminotransferase-class-V-fold-PLP-dependent-enzyme-[Paenibacillus-sp.-sptzw28]	715179
WP_235119006.1	cysteine-desulfurase	GCF_021513275.1	372	Bacteria>Terrabacteria_group>Firmicutes>Bacilli>Bacillales	Paenibacillus_sp._YPD9-1	aminotransferase-class-V-fold-PLP-dependent-enzyme-[Paenibacillus-sp.-YPD9-1]	2908839
WP_166244009.1	cysteine-desulfurase	GCF_011421635.1	376	Bacteria>Terrabacteria_group>Firmicutes>Bacilli>Bacillales	Paenibacillus_turpanensis	cysteine-desulfurase-[Paenibacillus-turpanensis]	2689078
WP_213654333.1	cysteine-desulfurase	GCF_018403325.1	377	Bacteria>Terrabacteria_group>Firmicutes>Bacilli>Bacillales	Paenibacillus_vini	aminotransferase-class-V-fold-PLP-dependent-enzyme-[Paenibacillus-vini]	1476024
WP_154892859.1	cysteine-desulfurase	GCF_007679725.1	377	Bacteria>Terrabacteria_group>Firmicutes>Bacilli>Bacillales	Paenibacillus_xylanexedens	aminotransferase-class-V-fold-PLP-dependent-enzyme-[Paenibacillus-xylanexedens]	528191
WP_043905163.1	cysteine-desulfurase	GCF_000632515.1	382	Bacteria>Terrabacteria_group>Firmicutes>Bacilli>Bacillales	Parageobacillus_genomosp._1	cysteine-desulfurase-DndA-[Parageobacillus-genomosp.-1]	1295642
WP_204539489.1	cysteine-desulfurase	GCF_016909175.1	372	Bacteria>Terrabacteria_group>Firmicutes>Bacilli>Bacillales	Peribacillus_deserti	cysteine-desulfurase-[Peribacillus-deserti]	673318
WP_127778145.1	cysteine-desulfurase	GCF_004006475.1	372	Bacteria>Terrabacteria_group>Firmicutes>Bacilli>Bacillales	Peribacillus_frigoritolerans	cysteine-desulfurase-[[Brevibacterium]	450367
WP_144528285.1	cysteine-desulfurase	GCF_007680885.1	372	Bacteria>Terrabacteria_group>Firmicutes>Bacilli>Bacillales	Peribacillus_simplex	aminotransferase-class-V-fold-PLP-dependent-enzyme-[Peribacillus-simplex]	1478
MBX9955703.1	cysteine-desulfurase	GCA_019748875.1	372	Bacteria>Terrabacteria_group>Firmicutes>Bacilli>Bacillales	Peribacillus_simplex	cysteine-desulfurase-[Peribacillus-simplex]	1478
WP_053168290.1	cysteine-desulfurase	GCF_001270075.1	372	Bacteria>Terrabacteria_group>Firmicutes>Bacilli>Bacillales	Planococcus_glaciei	cysteine-desulfurase-[Planococcus-glaciei]	459472
WP_135501971.1	cysteine-desulfurase	GCF_014202615.1	372	Bacteria>Terrabacteria_group>Firmicutes>Bacilli>Bacillales	Planococcus_koreensis	cysteine-desulfurase-[Planomicrobium-koreense]	112331
WP_069576778.1	cysteine-desulfurase	GCF_001730005.1	372	Bacteria>Terrabacteria_group>Firmicutes>Bacilli>Bacillales	Planococcus_maritimus	cysteine-desulfurase-[Planococcus-maritimus]	192421
WP_084243798.1	cysteine-desulfurase	GCF_900176575.1	372	Bacteria>Terrabacteria_group>Firmicutes>Bacilli>Bacillales	Planomicrobium_okeanokoites	cysteine-desulfurase-[Planomicrobium-okeanokoites]	244
GGD08307.1	cysteine-desulfurase	GCA_014642405.1	372	Bacteria>Terrabacteria_group>Firmicutes>Bacilli>Bacillales	Pontibacillus_salipaludis	cysteine-desulfurase-IscS-[Pontibacillus-salipaludis]	1697394
WP_188652376.1	cysteine-desulfurase	GCF_014642405.1	372	Bacteria>Terrabacteria_group>Firmicutes>Bacilli>Bacillales	Pontibacillus_salipaludis	cysteine-desulfurase-[Pontibacillus-salipaludis]	1697394

Accession	Annotation	GCA	Length	Phyletic.spread	Strain.name	Defline	Species.Taxid
WP_154990523.1	cysteine-desulfurase	GCF_015582655.1	372	Bacteria>Terrabacteria_group>Firmicutes>Bacilli>Bacillales	Priestia	aminotransferase-class-V-fold-PLP-dependent-enzyme-[Priestia-megaterium]	2800373
MBE5103326.1	cysteine-desulfurase	GCA_014932885.1	372	Bacteria>Terrabacteria_group>Firmicutes>Bacilli>Bacillales	Priestia_aryabhattai	aminotransferase-class-V-fold-PLP-dependent-enzyme-[Priestia-aryabhattai]	412384
WP_033579262.1	cysteine-desulfurase	GCF_013372535.1	372	Bacteria>Terrabacteria_group>Firmicutes>Bacilli>Bacillales	Priestia_aryabhattai	aminotransferase-class-V-fold-PLP-dependent-enzyme-[Priestia-aryabhattai]	412384
WP_193426776.1	cysteine-desulfurase	GCF_014932885.1	372	Bacteria>Terrabacteria_group>Firmicutes>Bacilli>Bacillales	Priestia_aryabhattai	aminotransferase-class-V-fold-PLP-dependent-enzyme-[Priestia-aryabhattai]	412384
WP_075422637.1	cysteine-desulfurase	GCF_001937775.1	372	Bacteria>Terrabacteria_group>Firmicutes>Bacilli>Bacillales	Priestia_megaterium	aminotransferase-class-V-fold-PLP-dependent-enzyme-[Priestia-megaterium]	1404
WP_098383117.1	cysteine-desulfurase	GCF_002562195.1	372	Bacteria>Terrabacteria_group>Firmicutes>Bacilli>Bacillales	Priestia_megaterium	aminotransferase-class-V-fold-PLP-dependent-enzyme-[Priestia-megaterium]	1404
WP_161515511.1	cysteine-desulfurase	GCF_009935415.1	372	Bacteria>Terrabacteria_group>Firmicutes>Bacilli>Bacillales	Priestia_megaterium	aminotransferase-class-V-fold-PLP-dependent-enzyme-[Priestia-megaterium]	1404
WP_180896595.1	cysteine-desulfurase	GCF_013458535.1	372	Bacteria>Terrabacteria_group>Firmicutes>Bacilli>Bacillales	Priestia_megaterium	aminotransferase-class-V-fold-PLP-dependent-enzyme-[Priestia-megaterium]	1404
WP_205661396.1	cysteine-desulfurase	GCF_017086565.1	372	Bacteria>Terrabacteria_group>Firmicutes>Bacilli>Bacillales	Priestia_megaterium	aminotransferase-class-V-fold-PLP-dependent-enzyme-[Priestia-megaterium]	1404
WP_098791869.1	cysteine-desulfurase	GCF_002574795.1	372	Bacteria>Terrabacteria_group>Firmicutes>Bacilli>Bacillales	Priestia_megaterium	cysteine-desulfurase-DndA-[Priestia-megaterium]	1404
QLK09344.1	cysteine-desulfurase	GCA_013458535.1	372	Bacteria>Terrabacteria_group>Firmicutes>Bacilli>Bacillales	Priestia_megaterium	Cysteine-desulfurase-[Priestia-megaterium]	1404
WP_098524918.1	cysteine-desulfurase	GCF_002566345.1	372	Bacteria>Terrabacteria_group>Firmicutes>Bacilli>Bacillales	Priestia_megaterium	MULTISPECIES:-aminotransferase-class-V-fold-PLP-dependent-enzyme-[Bacillaceae]	1404
WP_160919823.1	cysteine-desulfurase	GCF_009856375.1	373	Bacteria>Terrabacteria_group>Firmicutes>Bacilli>Bacillales	Pseudalkalibacillus_hwajinpoensis	aminotransferase-class-V-fold-PLP-dependent-enzyme-[Alkalihalobacillus-hwajinpoensis]	208199
WP_226611972.1	cysteine-desulfurase	GCF_020524205.1	373	Bacteria>Terrabacteria_group>Firmicutes>Bacilli>Bacillales	Pseudalkalibacillus_hwajinpoensis	aminotransferase-class-V-fold-PLP-dependent-enzyme-[Alkalihalobacillus-hwajinpoensis]	208199
WP_148796771.1	cysteine-desulfurase	GCF_008124485.1	372	Bacteria>Terrabacteria_group>Firmicutes>Bacilli>Bacillales	Rossellomorea_marisflavi	aminotransferase-class-V-fold-PLP-dependent-enzyme-[Rossellomorea-marisflavi]	189381
WP_159129317.1	cysteine-desulfurase	GCF_009806575.1	372	Bacteria>Terrabacteria_group>Firmicutes>Bacilli>Bacillales	Rossellomorea_marisflavi	aminotransferase-class-V-fold-PLP-dependent-enzyme-[Rossellomorea-marisflavi]	189381
WP_060674948.1	cysteine-desulfurase	GCF_001305855.1	372	Bacteria>Terrabacteria_group>Firmicutes>Bacilli>Bacillales	Rossellomorea_vietnamensis	aminotransferase-class-V-fold-PLP-dependent-enzyme-[Rossellomorea-vietnamensis]	218284
NGZ74986.1	cysteine-desulfurase	GCA_011090185.1	377	Bacteria>Terrabacteria_group>Firmicutes>Bacilli>Bacillales	Saccharibacillus_alkalitolerans	aminotransferase-class-V-fold-PLP-dependent-enzyme-[Saccharibacillus-alkalitolerans]	2705290
WP_166273310.1	cysteine-desulfurase	GCF_011090185.1	377	Bacteria>Terrabacteria_group>Firmicutes>Bacilli>Bacillales	Saccharibacillus_alkalitolerans	aminotransferase-class-V-fold-PLP-dependent-enzyme-[Saccharibacillus-alkalitolerans]	2705290
WP_172251703.1	cysteine-desulfurase	GCF_013185225.1	377	Bacteria>Terrabacteria_group>Firmicutes>Bacilli>Bacillales	Saccharibacillus_deserti	aminotransferase-class-V-fold-PLP-dependent-enzyme-[Saccharibacillus-deserti]	1634444
OWA34249.1	cysteine-desulfurase	GCA_002189105.1	377	Bacteria>Terrabacteria_group>Firmicutes>Bacilli>Bacillales	Saccharibacillus_sp._O16	cysteine-desulfurase-DndA-[Saccharibacillus-sp.-O16]	1978120
WP_076558529.1	cysteine-desulfurase	GCF_900156645.1	374	Bacteria>Terrabacteria_group>Firmicutes>Bacilli>Bacillales	Salimicrobium_flavidum	cysteine-desulfurase-[Salimicrobium-flavidum]	570947
WP_069365498.1	cysteine-desulfurase	GCF_001721685.1	383	Bacteria>Terrabacteria_group>Firmicutes>Bacilli>Bacillales	Salisediminibacterium_beveridgei	cysteine-desulfurase-[Bacillus-beveridgei]	632773
AOM83526.1	cysteine-desulfurase	GCA_001721685.1	383	Bacteria>Terrabacteria_group>Firmicutes>Bacilli>Bacillales	Salisediminibacterium_beveridgei	Cysteine-desulfurase-[Bacillus-beveridgei]	632773
WP_077622441.1	cysteine-desulfurase	GCF_900156915.1	372	Bacteria>Terrabacteria_group>Firmicutes>Bacilli>Bacillales	Sediminibacillus_massiliensis	cysteine-desulfurase-[Sediminibacillus-massiliensis]	1926277
WP_039966758.1	cysteine-desulfurase	GCF_000298255.1	372	Bacteria>Terrabacteria_group>Firmicutes>Bacilli>Bacillales	Solibacillus_isronensis	aminotransferase-class-V-fold-PLP-dependent-enzyme-[Solibacillus-isronensis]	412383
WP_029055184.1	cysteine-desulfurase	GCF_000425545.1	372	Bacteria>Terrabacteria_group>Firmicutes>Bacilli>Bacillales	Sporosarcina_ureae	aminotransferase-class-V-fold-PLP-dependent-enzyme-[Sporosarcina-ureae]	1571
ARF14003.1	cysteine-desulfurase	GCA_002081995.1	372	Bacteria>Terrabacteria_group>Firmicutes>Bacilli>Bacillales	Sporosarcina_ureae	cysteine-desulfurase-DndA-[Sporosarcina-ureae]	1571
WP_102706495.1	cysteine-desulfurase	GCF_002884435.1	372	Bacteria>Terrabacteria_group>Firmicutes>Bacilli>Bacillales	Terribacillus_goriensis	cysteine-desulfurase-[Terribacillus-goriensis]	386490
WP_184662912.1	cysteine-desulfurase	GCF_014202575.1	374	Bacteria>Terrabacteria_group>Firmicutes>Bacilli>Bacillales	Texcoconibacillus_texcoconensis	aminotransferase-class-V-fold-PLP-dependent-enzyme-[Texcoconibacillus-texcoconensis]	1095777
WP_062444822.1	cysteine-desulfurase	GCF_014635125.1	372	Bacteria>Terrabacteria_group>Firmicutes>Bacilli>Bacillales	Thalassobacillus_devorans	cysteine-desulfurase-[Thalassobacillus-devorans]	279813
WP_181741769.1	cysteine-desulfurase	GCF_013868055.1	373	Bacteria>Terrabacteria_group>Firmicutes>Bacilli>Bacillales	Thermoactinomyces_mirandus	cysteine-desulfurase-[Thermoactinomyces-mirandus]	2756294
WP_198059421.1	cysteine-desulfurase	GCF_016055135.1	373	Bacteria>Terrabacteria_group>Firmicutes>Bacilli>Bacillales	Thermoactinomyces_sp._CICC_10521	cysteine-desulfurase-[Thermoactinomyces-sp.-CICC-10521]	2767426
WP_132947149.1	cysteine-desulfurase	GCF_004341205.1	382	Bacteria>Terrabacteria_group>Firmicutes>Bacilli>Bacillales	Thermolongibacillus_altinsuensis	cysteine-desulfurase-DndA-[Thermolongibacillus-altinsuensis]	575256
WP_085524481.1	cysteine-desulfurase	GCF_900178005.1	377	Bacteria>Terrabacteria_group>Firmicutes>Bacilli>Bacillales	Tuberibacillus_sp._Marseille-P3662	aminotransferase-class-V-fold-PLP-dependent-enzyme-[Tuberibacillus-sp.-Marseille-P3662]	1965358
WP_038084661.1	cysteine-desulfurase	GCF_000714935.1	384	Bacteria>Terrabacteria_group>Firmicutes>Bacilli>Bacillales	Tumebacillus_flagellatus	cysteine-desulfurase-[Tumebacillus-flagellatus]	1157490
WP_098357581.1	cysteine-desulfurase	GCF_002561435.1	373	Bacteria>Terrabacteria_group>Firmicutes>Bacilli>Bacillales	unclassified_Bacillus_(in:_Bacteria)	MULTISPECIES:-cysteine-desulfurase-DndA-[unclassified-Bacillus-(in:-Bacteria)]	185979
WP_214691171.1	cysteine-desulfurase	GCF_018617255.1	372	Bacteria>Terrabacteria_group>Firmicutes>Bacilli>Bacillales	unclassified_Exiguobacterium	MULTISPECIES:-aminotransferase-class-V-fold-PLP-dependent-enzyme-[unclassified-Exiguobacterium]	2644629
WP_214763748.1	cysteine-desulfurase	GCF_018618015.1	372	Bacteria>Terrabacteria_group>Firmicutes>Bacilli>Bacillales	unclassified_Exiguobacterium	MULTISPECIES:-aminotransferase-class-V-fold-PLP-dependent-enzyme-[unclassified-Exiguobacterium]	2644629
WP_214798991.1	cysteine-desulfurase	GCF_018618175.1	372	Bacteria>Terrabacteria_group>Firmicutes>Bacilli>Bacillales	unclassified_Exiguobacterium	MULTISPECIES:-aminotransferase-class-V-fold-PLP-dependent-enzyme-[unclassified-Exiguobacterium]	2644629
WP_214882871.1	cysteine-desulfurase	GCF_018617815.1	372	Bacteria>Terrabacteria_group>Firmicutes>Bacilli>Bacillales	unclassified_Exiguobacterium	MULTISPECIES:-aminotransferase-class-V-fold-PLP-dependent-enzyme-[unclassified-Exiguobacterium]	2644629
WP_215111921.1	cysteine-desulfurase	GCF_018617095.1	372	Bacteria>Terrabacteria_group>Firmicutes>Bacilli>Bacillales	unclassified_Exiguobacterium	MULTISPECIES:-aminotransferase-class-V-fold-PLP-dependent-enzyme-[unclassified-Exiguobacterium]	2644629
MBN2922363.1	cysteine-desulfurase	GCA_016938995.1	372	Bacteria>Terrabacteria_group>Firmicutes>Bacilli>Lactobacillales	Lactobacillus_sp.	aminotransferase-class-V-fold-PLP-dependent-enzyme-[Lactobacillus-sp.]	1591
PWM30121.1	cysteine-desulfurase	GCA_003343585.1	381	Bacteria>Terrabacteria_group>Firmicutes>Bacilli>Lactobacillales	Limosilactobacillus_fermentum	cysteine-desulfurase-[Limosilactobacillus-fermentum]	1613
MBQ9853772.1	cysteine-desulfurase	GCA_017434955.1	375	Bacteria>Terrabacteria_group>Firmicutes>Bacilli>unclassified_Bacilli	Bacilli_bacterium	aminotransferase-class-V-fold-PLP-dependent-enzyme-[Bacilli-bacterium]	1903720
MBR2247214.1	cysteine-desulfurase	GCA_017475375.1	376	Bacteria>Terrabacteria_group>Firmicutes>Bacilli>unclassified_Bacilli	Bacilli_bacterium	aminotransferase-class-V-fold-PLP-dependent-enzyme-[Bacilli-bacterium]	1903720
MBR4231125.1	cysteine-desulfurase	GCA_017544165.1	376	Bacteria>Terrabacteria_group>Firmicutes>Bacilli>unclassified_Bacilli	Bacilli_bacterium	aminotransferase-class-V-fold-PLP-dependent-enzyme-[Bacilli-bacterium]	1903720
WP_010249532.1	cysteine-desulfurase	GCF_000179595.2	402	Bacteria>Terrabacteria_group>Firmicutes>Clostridia>Eubacteriales	Acetivibrio_cellulolyticus	cysteine-desulfurase-[Acetivibrio-cellulolyticus]	35830
WP_050740329.1	cysteine-desulfurase	GCF_001263355.1	388	Bacteria>Terrabacteria_group>Firmicutes>Clostridia>Eubacteriales	Acetobacterium_bakii	aminotransferase-class-V-fold-PLP-dependent-enzyme-[Acetobacterium-bakii]	52689
WP_162624401.1	cysteine-desulfurase	GCF_003260995.1	388	Bacteria>Terrabacteria_group>Firmicutes>Clostridia>Eubacteriales	Acetobacterium_sp._KB-1	aminotransferase-class-V-fold-PLP-dependent-enzyme-[Acetobacterium-sp.-KB-1]	2184575
WP_066540133.1	cysteine-desulfurase	GCF_002201475.1	377	Bacteria>Terrabacteria_group>Firmicutes>Clostridia>Eubacteriales	Acutalibacter_muris	cysteine-desulfurase-[Acutalibacter-muris]	1796620
WP_114299091.1	cysteine-desulfurase	GCF_003337415.1	378	Bacteria>Terrabacteria_group>Firmicutes>Clostridia>Eubacteriales	Anaerobacterium_chartisolvens	cysteine-desulfurase-[Anaerobacterium-chartisolvens]	1297424
WP_091234196.1	cysteine-desulfurase	GCF_900096945.1	373	Bacteria>Terrabacteria_group>Firmicutes>Clostridia>Eubacteriales	Anaerobium_acetethylicum	aminotransferase-class-V-fold-PLP-dependent-enzyme-[Anaerobium-acetethylicum]	1619234
WP_073590728.1	cysteine-desulfurase	GCF_900143645.1	383	Bacteria>Terrabacteria_group>Firmicutes>Clostridia>Eubacteriales	Anaerocolumna_xylanovorans	aminotransferase-class-V-fold-PLP-dependent-enzyme-[Anaerocolumna-xylanovorans]	100134
WP_087241275.1	cysteine-desulfurase	GCF_002160025.1	377	Bacteria>Terrabacteria_group>Firmicutes>Clostridia>Eubacteriales	Anaeromassilibacillus_sp._An200	cysteine-desulfurase-[Anaeromassilibacillus-sp.-An200]	1965587
WP_195560291.1	cysteine-desulfurase	GCF_015558525.1	381	Bacteria>Terrabacteria_group>Firmicutes>Clostridia>Eubacteriales	Anaeromassilibacillus_sp._D41t1_190614_C2	aminotransferase-class-V-fold-PLP-dependent-enzyme-[Anaeromassilibacillus-sp.-D41t1_190614_C2]	2787078
WP_095130871.1	cysteine-desulfurase	GCF_002270055.1	387	Bacteria>Terrabacteria_group>Firmicutes>Clostridia>Eubacteriales	Anaeromicrobium_sediminis	cysteine-desulfurase-[Anaeromicrobium-sediminis]	1478221
WP_055073096.1	cysteine-desulfurase	GCF_001405635.1	382	Bacteria>Terrabacteria_group>Firmicutes>Clostridia>Eubacteriales	Anaerostipes_hadrus	aminotransferase-class-V-fold-PLP-dependent-enzyme-[Anaerostipes-hadrus]	649756
WP_137327291.1	cysteine-desulfurase	-	382	Bacteria>Terrabacteria_group>Firmicutes>Clostridia>Eubacteriales	Anaerostipes_rhamnosivorans	cysteine-desulfurase-[Anaerostipes-rhamnosivorans]	1229621
MBM6829709.1	cysteine-desulfurase	GCA_016902535.1	376	Bacteria>Terrabacteria_group>Firmicutes>Clostridia>Eubacteriales	Anaerotignum_lactatifermentans	aminotransferase-class-V-fold-PLP-dependent-enzyme-[Anaerotignum-lactatifermentans]	160404
WP_205133866.1	cysteine-desulfurase	GCF_016901915.1	376	Bacteria>Terrabacteria_group>Firmicutes>Clostridia>Eubacteriales	Anaerotignum_lactatifermentans	aminotransferase-class-V-fold-PLP-dependent-enzyme-[Anaerotignum-lactatifermentans]	160404
WP_087216841.1	cysteine-desulfurase	GCF_002159815.1	377	Bacteria>Terrabacteria_group>Firmicutes>Clostridia>Eubacteriales	Anaerotruncus_colihominis	cysteine-desulfurase-[Anaerotruncus-colihominis]	169435
WP_089282269.1	cysteine-desulfurase	GCF_900188145.1	378	Bacteria>Terrabacteria_group>Firmicutes>Clostridia>Eubacteriales	Anaerovirgula_multivorans	cysteine-desulfurase-[Anaerovirgula-multivorans]	312168
WP_173772984.1	cysteine-desulfurase	GCF_013302415.1	382	Bacteria>Terrabacteria_group>Firmicutes>Clostridia>Eubacteriales	Blautia_faecis	cysteine-desulfurase-[Blautia-faecis]	871665
WP_195979421.1	cysteine-desulfurase	GCF_015669135.1	397	Bacteria>Terrabacteria_group>Firmicutes>Clostridia>Eubacteriales	Blautia_luti	cysteine-desulfurase-[Blautia-luti]	89014
WP_158661569.1	cysteine-desulfurase	GCF_900258535.1	380	Bacteria>Terrabacteria_group>Firmicutes>Clostridia>Eubacteriales	Blautia_marasmi	aminotransferase-class-V-fold-PLP-dependent-enzyme-[Blautia-marasmi]	1917868
WP_244092346.1	cysteine-desulfurase	GCF_013304465.1	384	Bacteria>Terrabacteria_group>Firmicutes>Clostridia>Eubacteriales	Blautia_obeum	aminotransferase-class-V-fold-PLP-dependent-enzyme-[Blautia-obeum]	40520
WP_029469212.1	cysteine-desulfurase	GCF_004210255.1	397	Bacteria>Terrabacteria_group>Firmicutes>Clostridia>Eubacteriales	Blautia_producta	cysteine-desulfurase-[Blautia-producta]	33035
QBE98082.1	cysteine-desulfurase	GCA_004210255.1	397	Bacteria>Terrabacteria_group>Firmicutes>Clostridia>Eubacteriales	Blautia_producta	Cysteine-desulfurase-IscS-[Blautia-producta]	33035
MBR2258736.1	cysteine-desulfurase	GCA_017475035.1	383	Bacteria>Terrabacteria_group>Firmicutes>Clostridia>Eubacteriales	Blautia_sp.	aminotransferase-class-V-fold-PLP-dependent-enzyme-[Blautia-sp.]	1955243
MCF0132234.1	cysteine-desulfurase	GCA_021443885.1	79	Bacteria>Terrabacteria_group>Firmicutes>Clostridia>Eubacteriales	Blautia_sp.	aminotransferase-class-V-fold-PLP-dependent-enzyme,-partial-[Blautia-sp.]	1955243
WP_173702178.1	cysteine-desulfurase	GCF_013299995.1	397	Bacteria>Terrabacteria_group>Firmicutes>Clostridia>Eubacteriales	Blautia_wexlerae	cysteine-desulfurase-[Blautia-wexlerae]	418240
WP_087374099.1	cysteine-desulfurase	GCF_002160355.1	394	Bacteria>Terrabacteria_group>Firmicutes>Clostridia>Eubacteriales	Butyricicoccus_pullicaecorum	cysteine-desulfurase-[Butyricicoccus-pullicaecorum]	501571
OUP51809.1	cysteine-desulfurase	GCA_002160355.1	394	Bacteria>Terrabacteria_group>Firmicutes>Clostridia>Eubacteriales	Butyricicoccus_pullicaecorum	cysteine-desulfurase-DndA-[Butyricicoccus-pullicaecorum]	501571
MBD9030348.1	cysteine-desulfurase	GCA_014847615.1	375	Bacteria>Terrabacteria_group>Firmicutes>Clostridia>Eubacteriales	Butyrivibrio_crossotus	aminotransferase-class-V-fold-PLP-dependent-enzyme-[Butyrivibrio-crossotus]	45851
WP_074461423.1	cysteine-desulfurase	GCF_900101605.1	379	Bacteria>Terrabacteria_group>Firmicutes>Clostridia>Eubacteriales	Butyrivibrio_hungatei	aminotransferase-class-V-fold-PLP-dependent-enzyme-[Butyrivibrio-hungatei]	185008
SCX88214.1	cysteine-desulfurase	GCA_900101605.1	379	Bacteria>Terrabacteria_group>Firmicutes>Clostridia>Eubacteriales	Butyrivibrio_hungatei	cysteine-desulfurase-family-protein-[Butyrivibrio-hungatei]	185008
WP_026669775.1	cysteine-desulfurase	GCF_000424165.1	383	Bacteria>Terrabacteria_group>Firmicutes>Clostridia>Eubacteriales	Butyrivibrio_sp._AE3006	aminotransferase-class-V-fold-PLP-dependent-enzyme-[Butyrivibrio-sp.-AE3006]	1280673
MBE5838085.1	cysteine-desulfurase	GCA_015056685.1	383	Bacteria>Terrabacteria_group>Firmicutes>Clostridia>Eubacteriales	Butyrivibrio_sp.	aminotransferase-class-V-fold-PLP-dependent-enzyme-[Butyrivibrio-sp.]	28121
HAI91960.1	cysteine-desulfurase	GCA_003449225.1	381	Bacteria>Terrabacteria_group>Firmicutes>Clostridia>Eubacteriales	Butyrivibrio_sp.	cysteine-desulfurase-[Butyrivibrio-sp.]	28121
MBP3818101.1	cysteine-desulfurase	GCA_017940925.1	381	Bacteria>Terrabacteria_group>Firmicutes>Clostridia>Eubacteriales	Butyrivibrio_sp.	cysteine-desulfurase-[Butyrivibrio-sp.]	28121
WP_026504832.1	cysteine-desulfurase	GCF_000424545.1	381	Bacteria>Terrabacteria_group>Firmicutes>Clostridia>Eubacteriales	Butyrivibrio_sp._NC3005	aminotransferase-class-V-fold-PLP-dependent-enzyme-[Butyrivibrio-sp.-NC3005]	1280685
WP_103868252.1	cysteine-desulfurase	GCF_900108105.1	383	Bacteria>Terrabacteria_group>Firmicutes>Clostridia>Eubacteriales	Butyrivibrio_sp._Su6	cysteine-desulfurase-[Butyrivibrio-sp.-Su6]	1520810
WP_092045148.1	cysteine-desulfurase	GCF_900110805.1	381	Bacteria>Terrabacteria_group>Firmicutes>Clostridia>Eubacteriales	Butyrivibrio_sp._TB	aminotransferase-class-V-fold-PLP-dependent-enzyme-[Butyrivibrio-sp.-TB]	1520809
MBZ4662720.1	cysteine-desulfurase	GCA_020054765.1	392	Bacteria>Terrabacteria_group>Firmicutes>Clostridia>Eubacteriales	Caloramator_sp.	cysteine-desulfurase-[Caloramator-sp.]	1871330
HJC82450.1	cysteine-desulfurase	GCA_019120015.1	383	Bacteria>Terrabacteria_group>Firmicutes>Clostridia>Eubacteriales	Candidatus_Anaerostipes_avicola	cysteine-desulfurase-[Candidatus-Anaerostipes-avicola]	2838461
HIV38439.1	cysteine-desulfurase	GCA_019112785.1	397	Bacteria>Terrabacteria_group>Firmicutes>Clostridia>Eubacteriales	Candidatus_Blautia_stercorigallinarum	cysteine-desulfurase-[Candidatus-Blautia-stercorigallinarum]	2838501
HJB57354.1	cysteine-desulfurase	GCA_019118925.1	376	Bacteria>Terrabacteria_group>Firmicutes>Clostridia>Eubacteriales	Candidatus_Flavonifractor_intestinipullorum	cysteine-desulfurase-[Candidatus-Flavonifractor-intestinipullorum]	2838587
HJA72180.1	cysteine-desulfurase	GCA_019118365.1	376	Bacteria>Terrabacteria_group>Firmicutes>Clostridia>Eubacteriales	Candidatus_Lachnoclostridium_stercoravium	aminotransferase-class-V-fold-PLP-dependent-enzyme-[Candidatus-Lachnoclostridium-stercoravium]	2838633
MBU3840492.1	cysteine-desulfurase	GCA_018883985.1	383	Bacteria>Terrabacteria_group>Firmicutes>Clostridia>Eubacteriales	Candidatus_Ruminococcus_intestinipullorum	aminotransferase-class-V-fold-PLP-dependent-enzyme-[Candidatus-Ruminococcus-intestinipullorum]	2838750
WP_159446844.1	cysteine-desulfurase	GCF_002049255.1	380	Bacteria>Terrabacteria_group>Firmicutes>Clostridia>Eubacteriales	Carboxydocella_sp._ULO1	aminotransferase-class-V-fold-PLP-dependent-enzyme-[Carboxydocella-sp.-ULO1]	1926599
WP_183776573.1	cysteine-desulfurase	GCF_014202115.1	383	Bacteria>Terrabacteria_group>Firmicutes>Clostridia>Eubacteriales	Catenibacillus_scindens	cysteine-desulfurase-[Catenibacillus-scindens]	673271
WP_023353461.1	cysteine-desulfurase	GCF_000160035.2	382	Bacteria>Terrabacteria_group>Firmicutes>Clostridia>Eubacteriales	Catonella_morbi	aminotransferase-class-V-fold-PLP-dependent-enzyme-[Catonella-morbi]	43997
MBE6023508.1	cysteine-desulfurase	GCA_015058045.1	385	Bacteria>Terrabacteria_group>Firmicutes>Clostridia>Eubacteriales	Cellulosilyticum_sp.	cysteine-desulfurase-[Cellulosilyticum-sp.]	1926876
WP_127065286.1	cysteine-desulfurase	GCF_003990395.1	378	Bacteria>Terrabacteria_group>Firmicutes>Clostridia>Eubacteriales	Cellulosilyticum_sp._WCF-2	cysteine-desulfurase-[Cellulosilyticum-sp.-WCF-2]	2497860
RHQ22517.1	cysteine-desulfurase	GCA_003478475.1	383	Bacteria>Terrabacteria_group>Firmicutes>Clostridia>Eubacteriales	Clostridiaceae_bacterium_AF29-16BH	aminotransferase-class-V-fold-PLP-dependent-enzyme-[Clostridiaceae-bacterium-AF29-16BH]	2292179
NLD50194.1	cysteine-desulfurase	GCA_012513545.1	379	Bacteria>Terrabacteria_group>Firmicutes>Clostridia>Eubacteriales	Clostridiaceae_bacterium	aminotransferase-class-V-fold-PLP-dependent-enzyme-[Clostridiaceae-bacterium]	1898204
NLX64372.1	cysteine-desulfurase	GCA_012728095.1	379	Bacteria>Terrabacteria_group>Firmicutes>Clostridia>Eubacteriales	Clostridiaceae_bacterium	aminotransferase-class-V-fold-PLP-dependent-enzyme-[Clostridiaceae-bacterium]	1898204
NMB95827.1	cysteine-desulfurase	GCA_012798655.1	382	Bacteria>Terrabacteria_group>Firmicutes>Clostridia>Eubacteriales	Clostridiaceae_bacterium	aminotransferase-class-V-fold-PLP-dependent-enzyme-[Clostridiaceae-bacterium]	1898204
OJV65159.1	cysteine-desulfurase	GCA_001897605.1	376	Bacteria>Terrabacteria_group>Firmicutes>Clostridia>Eubacteriales	Clostridiales_bacterium_38-18	cysteine-desulfurase-DndA-[Clostridiales-bacterium-38-18]	1895746
MBD8941252.1	cysteine-desulfurase	GCA_014848415.1	393	Bacteria>Terrabacteria_group>Firmicutes>Clostridia>Eubacteriales	Clostridiales_bacterium	aminotransferase-class-V-fold-PLP-dependent-enzyme-[Clostridiales-bacterium]	1898207
MBI9010939.1	cysteine-desulfurase	GCA_016342415.1	380	Bacteria>Terrabacteria_group>Firmicutes>Clostridia>Eubacteriales	Clostridiales_bacterium	aminotransferase-class-V-fold-PLP-dependent-enzyme-[Clostridiales-bacterium]	1898207
MBS4029849.1	cysteine-desulfurase	GCA_018335855.1	384	Bacteria>Terrabacteria_group>Firmicutes>Clostridia>Eubacteriales	Clostridiales_bacterium	aminotransferase-class-V-fold-PLP-dependent-enzyme-[Clostridiales-bacterium]	1898207
MBS6250198.1	cysteine-desulfurase	GCA_018374915.1	381	Bacteria>Terrabacteria_group>Firmicutes>Clostridia>Eubacteriales	Clostridiales_bacterium	aminotransferase-class-V-fold-PLP-dependent-enzyme-[Clostridiales-bacterium]	1898207
MCC8105778.1	cysteine-desulfurase	GCA_020860345.1	375	Bacteria>Terrabacteria_group>Firmicutes>Clostridia>Eubacteriales	Clostridiales_bacterium	aminotransferase-class-V-fold-PLP-dependent-enzyme-[Clostridiales-bacterium]	1898207
NLZ35422.1	cysteine-desulfurase	GCA_012800875.1	381	Bacteria>Terrabacteria_group>Firmicutes>Clostridia>Eubacteriales	Clostridiales_bacterium	aminotransferase-class-V-fold-PLP-dependent-enzyme-[Clostridiales-bacterium]	1898207
NMP37551.1	cysteine-desulfurase	GCA_012933745.1	385	Bacteria>Terrabacteria_group>Firmicutes>Clostridia>Eubacteriales	Clostridiales_bacterium	aminotransferase-class-V-fold-PLP-dependent-enzyme-[Clostridiales-bacterium]	1898207
CVH75679.1	cysteine-desulfurase	GCA_900018345.1	376	Bacteria>Terrabacteria_group>Firmicutes>Clostridia>Eubacteriales	Clostridiales_bacterium_CHKCI006	putative-cysteine-desulfurase-[Clostridiales-bacterium-CHKCI006]	1780379
MBS6677495.1	cysteine-desulfurase	GCA_018378235.1	397	Bacteria>Terrabacteria_group>Firmicutes>Clostridia>Eubacteriales	Clostridiales_bacterium	cysteine-desulfurase-[Clostridiales-bacterium]	1898207
NLL31708.1	cysteine-desulfurase	GCA_012520205.1	392	Bacteria>Terrabacteria_group>Firmicutes>Clostridia>Eubacteriales	Clostridiales_bacterium	cysteine-desulfurase-[Clostridiales-bacterium]	1898207
QTE70309.1	cysteine-desulfurase	GCA_017569305.1	378	Bacteria>Terrabacteria_group>Firmicutes>Clostridia>Eubacteriales	Clostridiales_bacterium_FE2011	aminotransferase-class-V-fold-PLP-dependent-enzyme-[Clostridiales-bacterium-FE2011]	2806438
ERI94638.1	cysteine-desulfurase	GCA_000466585.1	397	Bacteria>Terrabacteria_group>Firmicutes>Clostridia>Eubacteriales	Clostridiales_bacterium_oral_taxon_876_str._F0540	aminotransferase,-class-V-[Clostridiales-bacterium-oral-taxon-876-str.-F0540]	1321778
WP_211144273.1	cysteine-desulfurase	GCF_016316925.1	399	Bacteria>Terrabacteria_group>Firmicutes>Clostridia>Eubacteriales	Clostridium_aciditolerans	cysteine-desulfurase-[Clostridium-aciditolerans]	339861
WP_224168029.1	cysteine-desulfurase	GCF_900461085.1	394	Bacteria>Terrabacteria_group>Firmicutes>Clostridia>Eubacteriales	Clostridium_baratii	cysteine-desulfurase-[Clostridium-baratii]	1561
WP_041896616.1	cysteine-desulfurase	GCF_000833105.2	383	Bacteria>Terrabacteria_group>Firmicutes>Clostridia>Eubacteriales	Clostridium_beijerinckii	aminotransferase-class-V-fold-PLP-dependent-enzyme-[Clostridium-beijerinckii]	1520
NOW04436.1	cysteine-desulfurase	GCA_013149485.1	396	Bacteria>Terrabacteria_group>Firmicutes>Clostridia>Eubacteriales	Clostridium_beijerinckii	cysteine-desulfurase-[Clostridium-beijerinckii]	1520
NOW91082.1	cysteine-desulfurase	GCA_013149505.1	396	Bacteria>Terrabacteria_group>Firmicutes>Clostridia>Eubacteriales	Clostridium_beijerinckii	cysteine-desulfurase-[Clostridium-beijerinckii]	1520
WP_173713797.1	cysteine-desulfurase	GCF_013294155.1	392	Bacteria>Terrabacteria_group>Firmicutes>Clostridia>Eubacteriales	Clostridium_beijerinckii	cysteine-desulfurase-[Clostridium-beijerinckii]	1520
MBN1038823.1	cysteine-desulfurase	GCA_016925905.1	385	Bacteria>Terrabacteria_group>Firmicutes>Clostridia>Eubacteriales	Clostridium_botulinum	aminotransferase-class-V-fold-PLP-dependent-enzyme-[Clostridium-botulinum]	1491
NFH70743.1	cysteine-desulfurase	GCA_011014615.1	385	Bacteria>Terrabacteria_group>Firmicutes>Clostridia>Eubacteriales	Clostridium_botulinum	aminotransferase-class-V-fold-PLP-dependent-enzyme-[Clostridium-botulinum]	1491
AVP62364.1	cysteine-desulfurase	GCA_003013655.1	369	Bacteria>Terrabacteria_group>Firmicutes>Clostridia>Eubacteriales	Clostridium_botulinum	aminotransferase-[Clostridium-botulinum]	1491
NFG76351.1	cysteine-desulfurase	GCA_011013535.1	398	Bacteria>Terrabacteria_group>Firmicutes>Clostridia>Eubacteriales	Clostridium_botulinum	cysteine-desulfurase-[Clostridium-botulinum]	1491
NFK79226.1	cysteine-desulfurase	GCA_011015635.1	392	Bacteria>Terrabacteria_group>Firmicutes>Clostridia>Eubacteriales	Clostridium_botulinum	cysteine-desulfurase-[Clostridium-botulinum]	1491
NFL33964.1	cysteine-desulfurase	GCA_011017025.1	398	Bacteria>Terrabacteria_group>Firmicutes>Clostridia>Eubacteriales	Clostridium_botulinum	cysteine-desulfurase-[Clostridium-botulinum]	1491
NFN99392.1	cysteine-desulfurase	GCA_011016295.1	398	Bacteria>Terrabacteria_group>Firmicutes>Clostridia>Eubacteriales	Clostridium_botulinum	cysteine-desulfurase-[Clostridium-botulinum]	1491

Accession	Annotation	GCA	Length	Phyletic.spread	Strain.name	Defline	Species.Taxid
NFO14260.1	cysteine-desulfurase	GCA_011016315.1	398	Bacteria>Terrabacteria_group>Firmicutes>Clostridia>Eubacteriales	Clostridium_botulinum	cysteine-desulfurase-[Clostridium-botulinum]	1491
NFT93580.1	cysteine-desulfurase	GCA_011021465.1	396	Bacteria>Terrabacteria_group>Firmicutes>Clostridia>Eubacteriales	Clostridium_botulinum	cysteine-desulfurase-[Clostridium-botulinum]	1491
WP_012450352.1	cysteine-desulfurase	GCF_000020285.1	398	Bacteria>Terrabacteria_group>Firmicutes>Clostridia>Eubacteriales	Clostridium_botulinum	cysteine-desulfurase-[Clostridium-botulinum]	1491
WP_013726261.1	cysteine-desulfurase	GCF_000204565.1	395	Bacteria>Terrabacteria_group>Firmicutes>Clostridia>Eubacteriales	Clostridium_botulinum	cysteine-desulfurase-[Clostridium-botulinum]	1491
WP_061297289.1	cysteine-desulfurase	GCF_001573635.1	398	Bacteria>Terrabacteria_group>Firmicutes>Clostridia>Eubacteriales	Clostridium_botulinum	cysteine-desulfurase-[Clostridium-botulinum]	1491
WP_076175547.1	cysteine-desulfurase	GCF_000711055.1	392	Bacteria>Terrabacteria_group>Firmicutes>Clostridia>Eubacteriales	Clostridium_botulinum	cysteine-desulfurase-[Clostridium-botulinum]	1491
AVP62354.1	cysteine-desulfurase	GCA_003013655.1	396	Bacteria>Terrabacteria_group>Firmicutes>Clostridia>Eubacteriales	Clostridium_botulinum	cysteine-desulfurase-DndA-[Clostridium-botulinum]	1491
AVP65981.1	cysteine-desulfurase	GCA_003013615.1	396	Bacteria>Terrabacteria_group>Firmicutes>Clostridia>Eubacteriales	Clostridium_botulinum	cysteine-desulfurase-DndA-[Clostridium-botulinum]	1491
WP_033127725.1	cysteine-desulfurase	GCF_018140655.1	398	Bacteria>Terrabacteria_group>Firmicutes>Clostridia>Eubacteriales	Clostridium_butyricum	cysteine-desulfurase-[Clostridium-butyricum]	1492
WP_148347235.1	cysteine-desulfurase	GCF_902362255.1	398	Bacteria>Terrabacteria_group>Firmicutes>Clostridia>Eubacteriales	Clostridium_butyricum	cysteine-desulfurase-[Clostridium-butyricum]	1492
ETI90594.1	cysteine-desulfurase	GCA_000508465.1	394	Bacteria>Terrabacteria_group>Firmicutes>Clostridia>Eubacteriales	Clostridium_butyricum_DORA_1	Cysteine-desulfurase-[Clostridium-butyricum-DORA_1]	1403941
WP_099335654.1	cysteine-desulfurase	GCF_902386485.1	391	Bacteria>Terrabacteria_group>Firmicutes>Clostridia>Eubacteriales	Clostridium_cadaveris	cysteine-desulfurase-[Clostridium-cadaveris]	1529
WP_105176700.1	cysteine-desulfurase	GCF_900290265.1	398	Bacteria>Terrabacteria_group>Firmicutes>Clostridia>Eubacteriales	Clostridium_cagae	cysteine-desulfurase-[Clostridium-cagae]	2080751
WP_079442203.1	cysteine-desulfurase	GCF_002029255.1	388	Bacteria>Terrabacteria_group>Firmicutes>Clostridia>Eubacteriales	Clostridium_chromiireducens	aminotransferase-class-V-fold-PLP-dependent-enzyme-[Clostridium-chromiireducens]	225345
WP_160359682.1	cysteine-desulfurase	GCF_009767885.1	393	Bacteria>Terrabacteria_group>Firmicutes>Clostridia>Eubacteriales	Clostridium_chromiireducens	cysteine-desulfurase-[Clostridium-chromiireducens]	225345
ENY94434.1	cysteine-desulfurase	GCA_000371405.1	377	Bacteria>Terrabacteria_group>Firmicutes>Clostridia>Eubacteriales	[Clostridium]_clostridioforme_CM201	hypothetical-protein-HMPREF1098_02232-[[Clostridium]	999410
WP_207647288.1	cysteine-desulfurase	GCF_900103025.1	386	Bacteria>Terrabacteria_group>Firmicutes>Clostridia>Eubacteriales	Clostridium_cochlearium	aminotransferase-class-V-fold-PLP-dependent-enzyme-[Clostridium-cochlearium]	1494
WP_055291307.1	cysteine-desulfurase	GCF_001405575.1	392	Bacteria>Terrabacteria_group>Firmicutes>Clostridia>Eubacteriales	Clostridium_disporicum	cysteine-desulfurase-[Clostridium-disporicum]	84024
WP_092451118.1	cysteine-desulfurase	GCF_900111235.1	374	Bacteria>Terrabacteria_group>Firmicutes>Clostridia>Eubacteriales	[Clostridium]_fimetarium	aminotransferase-class-V-fold-PLP-dependent-enzyme-[[Clostridium]	99656
WP_013238119.1	cysteine-desulfurase	GCF_000143685.1	396	Bacteria>Terrabacteria_group>Firmicutes>Clostridia>Eubacteriales	Clostridium_ljungdahlii	cysteine-desulfurase-[Clostridium-ljungdahlii]	1538
WP_055070047.1	cysteine-desulfurase	GCF_001403635.1	394	Bacteria>Terrabacteria_group>Firmicutes>Clostridia>Eubacteriales	Clostridium_massiliamazoniense	cysteine-desulfurase-[Clostridium-massiliamazoniense]	1347366
WP_039284233.1	cysteine-desulfurase	GCF_902386705.1	397	Bacteria>Terrabacteria_group>Firmicutes>Clostridia>Eubacteriales	Clostridium	MULTISPECIES:-cysteine-desulfurase-[Clostridium]	1485
WP_058295427.1	cysteine-desulfurase	GCF_001458595.1	388	Bacteria>Terrabacteria_group>Firmicutes>Clostridia>Eubacteriales	Clostridium_neonatale	cysteine-desulfurase-[Clostridium-neonatale]	137838
WP_039250926.1	cysteine-desulfurase	GCF_000768585.1	386	Bacteria>Terrabacteria_group>Firmicutes>Clostridia>Eubacteriales	Clostridium_novyi	aminotransferase-class-V-fold-PLP-dependent-enzyme-[Clostridium-novyi]	1542
KGN00095.1	cysteine-desulfurase	GCA_000768585.1	386	Bacteria>Terrabacteria_group>Firmicutes>Clostridia>Eubacteriales	Clostridium_novyi_A_str._4570	hypothetical-protein-Z969_10180-[Clostridium-novyi-A-str.-4570]	1444290
STB12647.1	cysteine-desulfurase	GCA_900461285.1	396	Bacteria>Terrabacteria_group>Firmicutes>Clostridia>Eubacteriales	Clostridium_novyi	cysteine-desulfurase-[Clostridium-novyi]	1542
WP_039227312.1	cysteine-desulfurase	GCF_000724405.2	389	Bacteria>Terrabacteria_group>Firmicutes>Clostridia>Eubacteriales	Clostridium_novyi	cysteine-desulfurase-[Clostridium-novyi]	1542
WP_039252030.1	cysteine-desulfurase	GCF_000768605.1	397	Bacteria>Terrabacteria_group>Firmicutes>Clostridia>Eubacteriales	Clostridium_novyi	cysteine-desulfurase-[Clostridium-novyi]	1542
WP_195631720.1	cysteine-desulfurase	GCF_015561155.1	384	Bacteria>Terrabacteria_group>Firmicutes>Clostridia>Eubacteriales	Clostridium_paraputrificum	aminotransferase-class-V-fold-PLP-dependent-enzyme-[Clostridium-paraputrificum]	29363
WP_099314197.1	cysteine-desulfurase	GCF_900217205.1	392	Bacteria>Terrabacteria_group>Firmicutes>Clostridia>Eubacteriales	Clostridium_paraputrificum	cysteine-desulfurase-[Clostridium-paraputrificum]	29363
WP_221365358.1	cysteine-desulfurase	GCF_019711035.1	395	Bacteria>Terrabacteria_group>Firmicutes>Clostridia>Eubacteriales	Clostridium_perfringens	aminotransferase-class-V-fold-PLP-dependent-enzyme-[Clostridium-perfringens]	1502
EHA1007705.1	cysteine-desulfurase	GCA_016610805.1	396	Bacteria>Terrabacteria_group>Firmicutes>Clostridia>Eubacteriales	Clostridium_perfringens	cysteine-desulfurase-[Clostridium-perfringens]	1502
EHK2327205.1	cysteine-desulfurase	GCA_018011795.1	394	Bacteria>Terrabacteria_group>Firmicutes>Clostridia>Eubacteriales	Clostridium_perfringens	cysteine-desulfurase-[Clostridium-perfringens]	1502
EHK2348194.1	cysteine-desulfurase	GCA_018011935.1	396	Bacteria>Terrabacteria_group>Firmicutes>Clostridia>Eubacteriales	Clostridium_perfringens	cysteine-desulfurase-[Clostridium-perfringens]	1502
EHK2406051.1	cysteine-desulfurase	GCA_018012095.1	392	Bacteria>Terrabacteria_group>Firmicutes>Clostridia>Eubacteriales	Clostridium_perfringens	cysteine-desulfurase-[Clostridium-perfringens]	1502
EHR0218641.1	cysteine-desulfurase	GCA_018992235.1	395	Bacteria>Terrabacteria_group>Firmicutes>Clostridia>Eubacteriales	Clostridium_perfringens	cysteine-desulfurase-[Clostridium-perfringens]	1502
HAT4133845.1	cysteine-desulfurase	GCA_015931425.1	393	Bacteria>Terrabacteria_group>Firmicutes>Clostridia>Eubacteriales	Clostridium_perfringens	cysteine-desulfurase-[Clostridium-perfringens]	1502
HAT4209683.1	cysteine-desulfurase	GCA_015931985.1	395	Bacteria>Terrabacteria_group>Firmicutes>Clostridia>Eubacteriales	Clostridium_perfringens	cysteine-desulfurase-[Clostridium-perfringens]	1502
WP_003471732.1	cysteine-desulfurase	GCF_000171215.1	396	Bacteria>Terrabacteria_group>Firmicutes>Clostridia>Eubacteriales	Clostridium_perfringens	cysteine-desulfurase-[Clostridium-perfringens]	1502
WP_061428142.1	cysteine-desulfurase	GCF_001579785.1	394	Bacteria>Terrabacteria_group>Firmicutes>Clostridia>Eubacteriales	Clostridium_perfringens	cysteine-desulfurase-[Clostridium-perfringens]	1502
WP_070956857.1	cysteine-desulfurase	GCF_001854085.1	396	Bacteria>Terrabacteria_group>Firmicutes>Clostridia>Eubacteriales	Clostridium_perfringens	cysteine-desulfurase-[Clostridium-perfringens]	1502
WP_110060358.1	cysteine-desulfurase	GCF_021532025.1	396	Bacteria>Terrabacteria_group>Firmicutes>Clostridia>Eubacteriales	Clostridium_perfringens	cysteine-desulfurase-[Clostridium-perfringens]	1502
WP_148335870.1	cysteine-desulfurase	GCF_902459515.1	395	Bacteria>Terrabacteria_group>Firmicutes>Clostridia>Eubacteriales	Clostridium_perfringens	cysteine-desulfurase-[Clostridium-perfringens]	1502
WP_150540974.1	cysteine-desulfurase	GCF_902459455.1	394	Bacteria>Terrabacteria_group>Firmicutes>Clostridia>Eubacteriales	Clostridium_perfringens	cysteine-desulfurase-[Clostridium-perfringens]	1502
WP_164790009.1	cysteine-desulfurase	GCF_011062525.1	394	Bacteria>Terrabacteria_group>Firmicutes>Clostridia>Eubacteriales	Clostridium_perfringens	cysteine-desulfurase-[Clostridium-perfringens]	1502
WP_164807205.1	cysteine-desulfurase	GCF_011062885.1	396	Bacteria>Terrabacteria_group>Firmicutes>Clostridia>Eubacteriales	Clostridium_perfringens	cysteine-desulfurase-[Clostridium-perfringens]	1502
WP_165473436.1	cysteine-desulfurase	GCF_011068425.1	395	Bacteria>Terrabacteria_group>Firmicutes>Clostridia>Eubacteriales	Clostridium_perfringens	cysteine-desulfurase-[Clostridium-perfringens]	1502
WP_208373302.1	cysteine-desulfurase	GCF_017590655.1	396	Bacteria>Terrabacteria_group>Firmicutes>Clostridia>Eubacteriales	Clostridium_perfringens	cysteine-desulfurase-[Clostridium-perfringens]	1502
WP_224379865.1	cysteine-desulfurase	GCF_020138715.1	396	Bacteria>Terrabacteria_group>Firmicutes>Clostridia>Eubacteriales	Clostridium_perfringens	cysteine-desulfurase-[Clostridium-perfringens]	1502
WP_224397279.1	cysteine-desulfurase	GCF_020138655.1	393	Bacteria>Terrabacteria_group>Firmicutes>Clostridia>Eubacteriales	Clostridium_perfringens	cysteine-desulfurase-[Clostridium-perfringens]	1502
WP_115641089.1	cysteine-desulfurase	GCF_900461105.1	396	Bacteria>Terrabacteria_group>Firmicutes>Clostridia>Eubacteriales	Clostridium_putrefaciens	cysteine-desulfurase-[Clostridium-putrefaciens]	99675
WP_065077350.1	cysteine-desulfurase	GCF_001675165.1	384	Bacteria>Terrabacteria_group>Firmicutes>Clostridia>Eubacteriales	Clostridium_ragsdalei	aminotransferase-class-V-fold-PLP-dependent-enzyme-[Clostridium-ragsdalei]	217158
MBE6055186.1	cysteine-desulfurase	GCA_015058425.1	393	Bacteria>Terrabacteria_group>Firmicutes>Clostridia>Eubacteriales	Clostridium_sartagoforme	cysteine-desulfurase-[Clostridium-sartagoforme]	84031
MBM6860592.1	cysteine-desulfurase	GCA_016902275.1	386	Bacteria>Terrabacteria_group>Firmicutes>Clostridia>Eubacteriales	Clostridium_saudiense	cysteine-desulfurase-[Clostridium-saudiense]	1414720
WP_195952398.1	cysteine-desulfurase	GCF_015667285.1	392	Bacteria>Terrabacteria_group>Firmicutes>Clostridia>Eubacteriales	Clostridium_saudiense	cysteine-desulfurase-[Clostridium-saudiense]	1414720
WP_195966420.1	cysteine-desulfurase	GCF_015669365.1	384	Bacteria>Terrabacteria_group>Firmicutes>Clostridia>Eubacteriales	Clostridium_sp._1001283B150210_160208_E6	aminotransferase-class-V-fold-PLP-dependent-enzyme-[Clostridium-sp.-1001283B150210_160208_E6]	2787129
WP_118570733.1	cysteine-desulfurase	GCF_003478795.1	374	Bacteria>Terrabacteria_group>Firmicutes>Clostridia>Eubacteriales	Clostridium_sp._AF21-20LB	aminotransferase-class-V-fold-PLP-dependent-enzyme-[Clostridium-sp.-AF21-20LB]	2293003
WP_117823116.1	cysteine-desulfurase	GCF_003461085.1	373	Bacteria>Terrabacteria_group>Firmicutes>Clostridia>Eubacteriales	Clostridium_sp._AF23-6LB	aminotransferase-class-V-fold-PLP-dependent-enzyme-[Clostridium-sp.-AF23-6LB]	2293005
WP_118745898.1	cysteine-desulfurase	GCF_003480745.1	389	Bacteria>Terrabacteria_group>Firmicutes>Clostridia>Eubacteriales	Clostridium_sp._AM27-31LB	aminotransferase-class-V-fold-PLP-dependent-enzyme-[Clostridium-sp.-AM27-31LB]	2293026
HHX18426.1	cysteine-desulfurase	GCA_012841175.1	382	Bacteria>Terrabacteria_group>Firmicutes>Clostridia>Eubacteriales	Clostridium_sp.	aminotransferase-class-V-fold-PLP-dependent-enzyme-[Clostridium-sp.]	1506
MBS5363944.1	cysteine-desulfurase	GCA_018367855.1	389	Bacteria>Terrabacteria_group>Firmicutes>Clostridia>Eubacteriales	Clostridium_sp.	aminotransferase-class-V-fold-PLP-dependent-enzyme-[Clostridium-sp.]	1506
MBS5674464.1	cysteine-desulfurase	GCA_018370275.1	384	Bacteria>Terrabacteria_group>Firmicutes>Clostridia>Eubacteriales	Clostridium_sp.	aminotransferase-class-V-fold-PLP-dependent-enzyme-[Clostridium-sp.]	1506
WP_176255642.1	cysteine-desulfurase	GCF_013282095.1	383	Bacteria>Terrabacteria_group>Firmicutes>Clostridia>Eubacteriales	Clostridium_sp._C5-48	aminotransferase-class-V-fold-PLP-dependent-enzyme-[Clostridium-sp.-C5-48]	2547410
OKZ81478.1	cysteine-desulfurase	GCA_001916165.1	389	Bacteria>Terrabacteria_group>Firmicutes>Clostridia>Eubacteriales	Clostridium_sp._CAG:12237_41	hypothetical-protein-BHW08_01750-[Clostridium-sp.-CAG:12237_41]	1896985
CCZ42869.1	cysteine-desulfurase	GCA_000433155.1	389	Bacteria>Terrabacteria_group>Firmicutes>Clostridia>Eubacteriales	Clostridium_sp._CAG:122	putative-uncharacterized-protein-[Clostridium-sp.-CAG:122]	1262773
CCZ05507.1	cysteine-desulfurase	GCA_000432475.1	383	Bacteria>Terrabacteria_group>Firmicutes>Clostridia>Eubacteriales	Clostridium_sp._CAG:127	putative-uncharacterized-protein-[Clostridium-sp.-CAG:127]	1262774
CDD09280.1	cysteine-desulfurase	GCA_000432975.1	379	Bacteria>Terrabacteria_group>Firmicutes>Clostridia>Eubacteriales	Clostridium_sp._CAG:349	putative-cysteine-desulfurase-family-protein-[Clostridium-sp.-CAG:349]	1262797
MCB2291338.1	cysteine-desulfurase	GCA_020443565.1	384	Bacteria>Terrabacteria_group>Firmicutes>Clostridia>Eubacteriales	Clostridium_sp._CS001	aminotransferase-class-V-fold-PLP-dependent-enzyme-[Clostridium-sp.-CS001]	2880648
WP_226260856.1	cysteine-desulfurase	GCF_020443565.1	384	Bacteria>Terrabacteria_group>Firmicutes>Clostridia>Eubacteriales	Clostridium_sp._CS001	aminotransferase-class-V-fold-PLP-dependent-enzyme-[Clostridium-sp.-CS001]	2880648
MBE6050175.1	cysteine-desulfurase	GCA_015058405.1	387	Bacteria>Terrabacteria_group>Firmicutes>Clostridia>Eubacteriales	Clostridium_sp.	cysteine-desulfurase-[Clostridium-sp.]	1506
MBS5125664.1	cysteine-desulfurase	GCA_018365735.1	393	Bacteria>Terrabacteria_group>Firmicutes>Clostridia>Eubacteriales	Clostridium_sp.	cysteine-desulfurase-[Clostridium-sp.]	1506
MBS6765677.1	cysteine-desulfurase	GCA_018379185.1	383	Bacteria>Terrabacteria_group>Firmicutes>Clostridia>Eubacteriales	Clostridium_sp.	cysteine-desulfurase-[Clostridium-sp.]	1506
WP_195467950.1	cysteine-desulfurase	GCF_015555905.1	390	Bacteria>Terrabacteria_group>Firmicutes>Clostridia>Eubacteriales	Clostridium_sp._D43t1_170807_H7	aminotransferase-class-V-fold-PLP-dependent-enzyme-[Clostridium-sp.-D43t1_170807_H7]	2787140
WP_077613035.1	cysteine-desulfurase	GCF_900155545.1	383	Bacteria>Terrabacteria_group>Firmicutes>Clostridia>Eubacteriales	Clostridium_sp._Marseille-P2415	aminotransferase-class-V-fold-PLP-dependent-enzyme-[Clostridium-sp.-Marseille-P2415]	1805471
WP_215700281.1	cysteine-desulfurase	GCF_018785315.1	376	Bacteria>Terrabacteria_group>Firmicutes>Clostridia>Eubacteriales	Clostridium_sp._MCC353	aminotransferase-class-V-fold-PLP-dependent-enzyme-[Clostridium-sp.-MCC353]	2592646
WP_074366244.1	cysteine-desulfurase	GCF_900095445.1	397	Bacteria>Terrabacteria_group>Firmicutes>Clostridia>Eubacteriales	Clostridium_sp._N3C	cysteine-desulfurase-[Clostridium-sp.-N3C]	1776758
WP_186860397.1	cysteine-desulfurase	GCF_014287825.1	395	Bacteria>Terrabacteria_group>Firmicutes>Clostridia>Eubacteriales	Clostridium_sp._NSJ-6	cysteine-desulfurase-[Clostridium-sp.-NSJ-6]	2763036
WP_077272546.1	cysteine-desulfurase	GCF_019933215.1	369	Bacteria>Terrabacteria_group>Firmicutes>Clostridia>Eubacteriales	Clostridium_sporogenes	aminotransferase-class-V-fold-PLP-dependent-enzyme-[Clostridium-sporogenes]	1509
WP_163190601.1	cysteine-desulfurase	GCF_011020555.1	369	Bacteria>Terrabacteria_group>Firmicutes>Clostridia>Eubacteriales	Clostridium_sporogenes	aminotransferase-class-V-fold-PLP-dependent-enzyme-[Clostridium-sporogenes]	1509
WP_163224233.1	cysteine-desulfurase	GCF_011019675.1	369	Bacteria>Terrabacteria_group>Firmicutes>Clostridia>Eubacteriales	Clostridium_sporogenes	aminotransferase-class-V-fold-PLP-dependent-enzyme-[Clostridium-sporogenes]	1509
WP_058007715.1	cysteine-desulfurase	GCF_001444635.1	403	Bacteria>Terrabacteria_group>Firmicutes>Clostridia>Eubacteriales	Clostridium_sporogenes	cysteine-desulfurase-[Clostridium-sporogenes]	1509
WP_058012581.1	cysteine-desulfurase	GCF_019933215.1	396	Bacteria>Terrabacteria_group>Firmicutes>Clostridia>Eubacteriales	Clostridium_sporogenes	cysteine-desulfurase-[Clostridium-sporogenes]	1509
WP_115652182.1	cysteine-desulfurase	GCF_011020555.1	396	Bacteria>Terrabacteria_group>Firmicutes>Clostridia>Eubacteriales	Clostridium_sporogenes	cysteine-desulfurase-[Clostridium-sporogenes]	1509
WP_163224225.1	cysteine-desulfurase	GCF_011019675.1	403	Bacteria>Terrabacteria_group>Firmicutes>Clostridia>Eubacteriales	Clostridium_sporogenes	cysteine-desulfurase-[Clostridium-sporogenes]	1509
WP_195344416.1	cysteine-desulfurase	GCF_015550435.1	378	Bacteria>Terrabacteria_group>Firmicutes>Clostridia>Eubacteriales	[Clostridium]_symbiosum	aminotransferase-class-V-fold-PLP-dependent-enzyme-[[Clostridium]	1512
AOR23765.1	cysteine-desulfurase	GCA_001735765.2	392	Bacteria>Terrabacteria_group>Firmicutes>Clostridia>Eubacteriales	Clostridium_taeniosporum	cysteine-desulfurase-[Clostridium-taeniosporum]	394958
WP_069679913.1	cysteine-desulfurase	GCF_001735765.2	392	Bacteria>Terrabacteria_group>Firmicutes>Clostridia>Eubacteriales	Clostridium_taeniosporum	cysteine-desulfurase-[Clostridium-taeniosporum]	394958
WP_129032076.1	cysteine-desulfurase	GCF_004115485.1	396	Bacteria>Terrabacteria_group>Firmicutes>Clostridia>Eubacteriales	Clostridium_tetani	cysteine-desulfurase-[Clostridium-tetani]	1513
WP_106024660.1	cysteine-desulfurase	GCF_002995795.1	393	Bacteria>Terrabacteria_group>Firmicutes>Clostridia>Eubacteriales	Clostridium_thermopalmarium	cysteine-desulfurase-[Clostridium-thermopalmarium]	29373
WP_071143942.1	cysteine-desulfurase	GCF_900104635.1	383	Bacteria>Terrabacteria_group>Firmicutes>Clostridia>Eubacteriales	Coprococcus_phoceensis	MULTISPECIES:-aminotransferase-class-V-fold-PLP-dependent-enzyme-[Lachnospiraceae]	1870993
WP_148808697.1	cysteine-desulfurase	GCF_008152325.1	386	Bacteria>Terrabacteria_group>Firmicutes>Clostridia>Eubacteriales	Crassaminicella_thermophila	aminotransferase-class-V-fold-PLP-dependent-enzyme-[Crassaminicella-sp.-SY095]	2599308
WP_204613513.1	cysteine-desulfurase	GCF_016908775.1	397	Bacteria>Terrabacteria_group>Firmicutes>Clostridia>Eubacteriales	Defluviitalea_raffinosedens	cysteine-desulfurase-[Defluviitalea-raffinosedens]	1450156
WP_195710845.1	cysteine-desulfurase	GCF_015594625.1	384	Bacteria>Terrabacteria_group>Firmicutes>Clostridia>Eubacteriales	Desulfallas_sp._Bu1-1	aminotransferase-class-V-fold-PLP-dependent-enzyme-[Desulfallas-sp.-Bu1-1]	2787620
WP_006717304.1	cysteine-desulfurase	GCF_000231405.2	379	Bacteria>Terrabacteria_group>Firmicutes>Clostridia>Eubacteriales	Desulfitobacterium_metallireducens	aminotransferase-class-V-fold-PLP-dependent-enzyme-[Desulfitobacterium-metallireducens]	142877
MCB8814722.1	cysteine-desulfurase	GCA_020595055.1	375	Bacteria>Terrabacteria_group>Firmicutes>Clostridia>Eubacteriales	Desulfosporosinus_sp._SRJS8	aminotransferase-class-V-fold-PLP-dependent-enzyme-[Desulfosporosinus-sp.-SRJS8]	2885154
WP_229246340.1	cysteine-desulfurase	GCF_020595055.1	375	Bacteria>Terrabacteria_group>Firmicutes>Clostridia>Eubacteriales	Desulfosporosinus_sp._SRJS8	aminotransferase-class-V-fold-PLP-dependent-enzyme-[Desulfosporosinus-sp.-SRJS8]	2885154
MBS3887415.1	cysteine-desulfurase	GCA_018333455.1	385	Bacteria>Terrabacteria_group>Firmicutes>Clostridia>Eubacteriales	Dethiobacter_sp.	aminotransferase-class-V-fold-PLP-dependent-enzyme-[Dethiobacter-sp.]	2093370
WP_028087371.1	cysteine-desulfurase	GCF_000424345.1	373	Bacteria>Terrabacteria_group>Firmicutes>Clostridia>Eubacteriales	Dorea_longicatena	aminotransferase-class-V-fold-PLP-dependent-enzyme-[Dorea-longicatena]	88431
WP_049729881.1	cysteine-desulfurase	GCF_001185345.1	293	Bacteria>Terrabacteria_group>Firmicutes>Clostridia>Eubacteriales	Dorea_sp._D27	aminotransferase-class-V-fold-PLP-dependent-enzyme-[Dorea-sp.-D27]	658665
KMZ53078.1	cysteine-desulfurase	GCA_001185345.2	293	Bacteria>Terrabacteria_group>Firmicutes>Clostridia>Eubacteriales	Dorea_sp._D27	cysteine-desulfurase-[Dorea-sp.-D27]	658665
WP_105310114.1	cysteine-desulfurase	GCF_900290215.1	369	Bacteria>Terrabacteria_group>Firmicutes>Clostridia>Eubacteriales	Dorea_sp._Marseille-P4042	aminotransferase-class-V-fold-PLP-dependent-enzyme-[Dorea-sp.-Marseille-P4042]	2080749
WP_105310110.1	cysteine-desulfurase	GCF_900290215.1	397	Bacteria>Terrabacteria_group>Firmicutes>Clostridia>Eubacteriales	Dorea_sp._Marseille-P4042	cysteine-desulfurase-[Dorea-sp.-Marseille-P4042]	2080749
WP_213038897.1	cysteine-desulfurase	GCF_018228705.1	377	Bacteria>Terrabacteria_group>Firmicutes>Clostridia>Eubacteriales	Dysosmobacter_sp._Marseille-Q4140	aminotransferase-class-V-fold-PLP-dependent-enzyme-[Dysosmobacter-sp.-Marseille-Q4140]	2830675
WP_007863440.1	cysteine-desulfurase	GCF_015548445.1	378	Bacteria>Terrabacteria_group>Firmicutes>Clostridia>Eubacteriales	Enterocloster_citroniae	aminotransferase-class-V-fold-PLP-dependent-enzyme-[Enterocloster-citroniae]	358743
MBE7715067.1	cysteine-desulfurase	GCA_015152355.1	381	Bacteria>Terrabacteria_group>Firmicutes>Clostridia>Eubacteriales	Enterocloster_clostridioformis	aminotransferase-class-V-fold-PLP-dependent-enzyme-[Enterocloster-clostridioformis]	1531
WP_235388475.1	cysteine-desulfurase	GCF_021532095.1	381	Bacteria>Terrabacteria_group>Firmicutes>Clostridia>Eubacteriales	Enterocloster_clostridioformis	aminotransferase-class-V-fold-PLP-dependent-enzyme-[Enterocloster-clostridioformis]	1531
WP_002596276.1	cysteine-desulfurase	GCF_000371405.1	377	Bacteria>Terrabacteria_group>Firmicutes>Clostridia>Eubacteriales	Enterocloster_clostridioformis	cysteine-desulfurase-[Enterocloster-clostridioformis]	1531
MBR9954042.1	cysteine-desulfurase	GCA_018223415.1	373	Bacteria>Terrabacteria_group>Firmicutes>Clostridia>Eubacteriales	Eubacteriaceae_bacterium_Marseille-Q4139	aminotransferase-class-V-fold-PLP-dependent-enzyme-[Eubacteriaceae-bacterium-Marseille-Q4139]	2830656
WP_208066143.1	cysteine-desulfurase	GCF_017566045.1	383	Bacteria>Terrabacteria_group>Firmicutes>Clostridia>Eubacteriales	Eubacterium_callanderi	aminotransferase-class-V-fold-PLP-dependent-enzyme-[Eubacterium-callanderi]	53442
WP_118518387.1	cysteine-desulfurase	GCF_902364445.2	383	Bacteria>Terrabacteria_group>Firmicutes>Clostridia>Eubacteriales	Eubacterium	MULTISPECIES:-aminotransferase-class-V-fold-PLP-dependent-enzyme-[Eubacterium]	1730
WP_004067174.1	cysteine-desulfurase	GCF_000364225.2	383	Bacteria>Terrabacteria_group>Firmicutes>Clostridia>Eubacteriales	Eubacterium_plexicaudatum	aminotransferase-class-V-fold-PLP-dependent-enzyme-[Eubacterium-plexicaudatum]	97253
WP_090164461.1	cysteine-desulfurase	GCF_900102225.1	381	Bacteria>Terrabacteria_group>Firmicutes>Clostridia>Eubacteriales	Eubacterium_pyruvativorans	aminotransferase-class-V-fold-PLP-dependent-enzyme-[Eubacterium-pyruvativorans]	155865
WP_090471945.1	cysteine-desulfurase	GCF_900115385.1	381	Bacteria>Terrabacteria_group>Firmicutes>Clostridia>Eubacteriales	Eubacterium_pyruvativorans	aminotransferase-class-V-fold-PLP-dependent-enzyme-[Eubacterium-pyruvativorans]	155865
WP_021739887.1	cysteine-desulfurase	GCF_902375155.1	383	Bacteria>Terrabacteria_group>Firmicutes>Clostridia>Eubacteriales	Eubacterium_ramulus	cysteine-desulfurase-[Eubacterium-ramulus]	39490
CBL35191.1	cysteine-desulfurase	GCA_000210635.1	381	Bacteria>Terrabacteria_group>Firmicutes>Clostridia>Eubacteriales	[Eubacterium]_siraeum_V10Sc8a	cysteine-desulfurase-family-protein-[[Eubacterium]	717961
WP_031201603.1	cysteine-desulfurase	GCF_000403845.2	386	Bacteria>Terrabacteria_group>Firmicutes>Clostridia>Eubacteriales	Eubacterium_sp._14-2	aminotransferase-class-V-fold-PLP-dependent-enzyme-[Eubacterium-sp.-14-2]	1235790
RJV86465.1	cysteine-desulfurase	GCA_003603205.1	71	Bacteria>Terrabacteria_group>Firmicutes>Clostridia>Eubacteriales	Eubacterium_sp._AF18-3	aminotransferase-class-V-fold-PLP-dependent-enzyme-[Eubacterium-sp.-AF18-3]	2302947
RJV72765.1	cysteine-desulfurase	GCA_003602875.1	366	Bacteria>Terrabacteria_group>Firmicutes>Clostridia>Eubacteriales	Eubacterium_sp._AM47-9	cysteine-desulfurase-[Eubacterium-sp.-AM47-9]	2302951
MBO5175221.1	cysteine-desulfurase	GCA_017623035.1	382	Bacteria>Terrabacteria_group>Firmicutes>Clostridia>Eubacteriales	Eubacterium_sp.	aminotransferase-class-V-fold-PLP-dependent-enzyme-[Eubacterium-sp.]	142586
MBS5620024.1	cysteine-desulfurase	GCA_018369815.1	382	Bacteria>Terrabacteria_group>Firmicutes>Clostridia>Eubacteriales	Eubacterium_sp.	aminotransferase-class-V-fold-PLP-dependent-enzyme-[Eubacterium-sp.]	142586
WP_186840527.1	cysteine-desulfurase	GCF_014287775.1	384	Bacteria>Terrabacteria_group>Firmicutes>Clostridia>Eubacteriales	Eubacterium_sp._BX4	aminotransferase-class-V-fold-PLP-dependent-enzyme-[Eubacterium-sp.-BX4]	2763045
CCY69700.1	cysteine-desulfurase	GCA_000432355.1	384	Bacteria>Terrabacteria_group>Firmicutes>Clostridia>Eubacteriales	Eubacterium_sp._CAG:161	putative-cysteine-desulfurase-family-protein-[Eubacterium-sp.-CAG:161]	1262881
CCZ04215.1	cysteine-desulfurase	GCA_000431895.1	393	Bacteria>Terrabacteria_group>Firmicutes>Clostridia>Eubacteriales	Eubacterium_sp._CAG:603	putative-cysteine-desulfurase-family-protein-[Eubacterium-sp.-CAG:603]	1262891
HAR69674.1	cysteine-desulfurase	GCA_003476125.1	394	Bacteria>Terrabacteria_group>Firmicutes>Clostridia>Eubacteriales	Eubacterium_sp.	cysteine-desulfurase-[Eubacterium-sp.]	142586
HAX60496.1	cysteine-desulfurase	GCA_003486385.1	394	Bacteria>Terrabacteria_group>Firmicutes>Clostridia>Eubacteriales	Eubacterium_sp.	cysteine-desulfurase-[Eubacterium-sp.]	142586

Accession	Annotation	GCA	Length	Phyletic.spread	Strain.name	Defline	Species.Taxid
HCS31769.1	cysteine-desulfurase	GCA_003524315.1	377	Bacteria>Terrabacteria_group>Firmicutes>Clostridia>Eubacteriales	Eubacterium_sp.	cysteine-desulfurase-[Eubacterium-sp.]	142586
MBS6668819.1	cysteine-desulfurase	GCA_018378375.1	397	Bacteria>Terrabacteria_group>Firmicutes>Clostridia>Eubacteriales	Eubacterium_sp.	cysteine-desulfurase-[Eubacterium-sp.]	142586
WP_216506829.1	cysteine-desulfurase	GCF_018918185.1	375	Bacteria>Terrabacteria_group>Firmicutes>Clostridia>Eubacteriales	Eubacterium_sp._MSJ-21	aminotransferase-class-V-fold-PLP-dependent-enzyme-[Eubacterium-sp.-MSJ-21]	2841519
WP_117969888.1	cysteine-desulfurase	GCF_003466845.1	391	Bacteria>Terrabacteria_group>Firmicutes>Clostridia>Eubacteriales	Eubacterium_ventriosum	aminotransferase-class-V-fold-PLP-dependent-enzyme-[Eubacterium-ventriosum]	39496
WP_118232025.1	cysteine-desulfurase	GCF_003470845.1	391	Bacteria>Terrabacteria_group>Firmicutes>Clostridia>Eubacteriales	Eubacterium_ventriosum	aminotransferase-class-V-fold-PLP-dependent-enzyme-[Eubacterium-ventriosum]	39496
WP_051437694.1	cysteine-desulfurase	-	381	Bacteria>Terrabacteria_group>Firmicutes>Clostridia>Eubacteriales	Eubacterium_xylanophilum	aminotransferase-class-V-fold-PLP-dependent-enzyme-[Eubacterium-xylanophilum]	39497
WP_005924597.1	cysteine-desulfurase	GCF_902373685.1	381	Bacteria>Terrabacteria_group>Firmicutes>Clostridia>Eubacteriales	Faecalibacterium_prausnitzii	aminotransferase-class-V-fold-PLP-dependent-enzyme-[Faecalibacterium-prausnitzii]	853
WP_113993128.1	cysteine-desulfurase	GCF_003324185.1	381	Bacteria>Terrabacteria_group>Firmicutes>Clostridia>Eubacteriales	Faecalibacterium_prausnitzii	aminotransferase-class-V-fold-PLP-dependent-enzyme-[Faecalibacterium-prausnitzii]	853
WP_087335638.1	cysteine-desulfurase	GCF_002159265.1	381	Bacteria>Terrabacteria_group>Firmicutes>Clostridia>Eubacteriales	Flavonifractor_sp._An82	aminotransferase-class-V-fold-PLP-dependent-enzyme-[Flavonifractor-sp.-An82]	1965660
WP_058963330.1	cysteine-desulfurase	GCF_902375855.1	381	Bacteria>Terrabacteria_group>Firmicutes>Clostridia>Eubacteriales	Fournierella_massiliensis	aminotransferase-class-V-fold-PLP-dependent-enzyme-[Fournierella-massiliensis]	1650663
MCC2198074.1	cysteine-desulfurase	GCA_020687275.1	383	Bacteria>Terrabacteria_group>Firmicutes>Clostridia>Eubacteriales	Fusicatenibacter_sp._CLA-AA-H241	aminotransferase-class-V-fold-PLP-dependent-enzyme-[Fusicatenibacter-sp.-CLA-AA-H241]	2881270
WP_227597958.1	cysteine-desulfurase	-	383	Bacteria>Terrabacteria_group>Firmicutes>Clostridia>Eubacteriales	Fusicatenibacter_sp._CLA-AA-H241	aminotransferase-class-V-fold-PLP-dependent-enzyme-[Fusicatenibacter-sp.-CLA-AA-H241]	2881270
WP_193501234.1	cysteine-desulfurase	GCF_014982805.1	394	Bacteria>Terrabacteria_group>Firmicutes>Clostridia>Eubacteriales	Gemmiger_gallinarum	cysteine-desulfurase-[Gemmiger-gallinarum]	2779354
OUN15083.1	cysteine-desulfurase	GCA_002159185.1	394	Bacteria>Terrabacteria_group>Firmicutes>Clostridia>Eubacteriales	Gemmiger_sp._An87	cysteine-desulfurase-DndA-[Gemmiger-sp.-An87]	1965662
WP_087184936.1	cysteine-desulfurase	GCF_002159185.1	394	Bacteria>Terrabacteria_group>Firmicutes>Clostridia>Eubacteriales	Gemmiger_sp._An87	cysteine-desulfurase-[Gemmiger-sp.-An87]	1965662
WP_072902602.1	cysteine-desulfurase	GCF_900142225.1	383	Bacteria>Terrabacteria_group>Firmicutes>Clostridia>Eubacteriales	Hathewaya_proteolytica	cysteine-desulfurase-[Hathewaya-proteolytica]	29365
WP_161260523.1	cysteine-desulfurase	GCF_016908025.1	392	Bacteria>Terrabacteria_group>Firmicutes>Clostridia>Eubacteriales	Heliomicrobium_gestii	cysteine-desulfurase-[Heliomicrobium-gestii]	2699
WP_154575153.1	cysteine-desulfurase	GCF_009695915.1	383	Bacteria>Terrabacteria_group>Firmicutes>Clostridia>Eubacteriales	Hornefia_butyriciproducens	aminotransferase-class-V-fold-PLP-dependent-enzyme-[Hornefia-butyriciproducens]	2652293
WP_148510072.1	cysteine-desulfurase	GCF_902363795.1	382	Bacteria>Terrabacteria_group>Firmicutes>Clostridia>Eubacteriales	Hungatella_hathewayi	aminotransferase-class-V-fold-PLP-dependent-enzyme-[Hungatella-hathewayi]	154046
WP_025529103.1	cysteine-desulfurase	GCF_003435045.1	397	Bacteria>Terrabacteria_group>Firmicutes>Clostridia>Eubacteriales	Hungatella_hathewayi	cysteine-desulfurase-[Hungatella-hathewayi]	154046
WP_087065080.1	cysteine-desulfurase	GCF_900155735.1	397	Bacteria>Terrabacteria_group>Firmicutes>Clostridia>Eubacteriales	Intestinibacillus_massiliensis	cysteine-desulfurase-[Intestinibacillus-massiliensis]	1871029
WP_227105122.1	cysteine-desulfurase	GCF_020564315.1	397	Bacteria>Terrabacteria_group>Firmicutes>Clostridia>Eubacteriales	Intestinibacillus_massiliensis	cysteine-desulfurase-[Intestinibacillus-massiliensis]	1871029
WP_204751842.1	cysteine-desulfurase	GCF_016901775.1	376	Bacteria>Terrabacteria_group>Firmicutes>Clostridia>Eubacteriales	Intestinimonas_butyriciproducens	cysteine-desulfurase-[Intestinimonas-butyriciproducens]	1297617
WP_060931485.1	cysteine-desulfurase	GCF_001552975.1	397	Bacteria>Terrabacteria_group>Firmicutes>Clostridia>Eubacteriales	Lachnoanaerobaculum_saburreum	cysteine-desulfurase-[Lachnoanaerobaculum-saburreum]	467210
WP_022749079.1	cysteine-desulfurase	GCF_000421025.1	377	Bacteria>Terrabacteria_group>Firmicutes>Clostridia>Eubacteriales	Lachnobacterium_bovis	aminotransferase-class-V-fold-PLP-dependent-enzyme-[Lachnobacterium-bovis]	140626
WP_027422479.1	cysteine-desulfurase	GCF_000622065.1	377	Bacteria>Terrabacteria_group>Firmicutes>Clostridia>Eubacteriales	Lachnobacterium_bovis	aminotransferase-class-V-fold-PLP-dependent-enzyme-[Lachnobacterium-bovis]	140626
WP_065548298.1	cysteine-desulfurase	GCF_001688665.2	381	Bacteria>Terrabacteria_group>Firmicutes>Clostridia>Eubacteriales	Lachnoclostridium_sp._YL32	aminotransferase-class-V-fold-PLP-dependent-enzyme-[Lachnoclostridium-sp.-YL32]	1834196
EGC75128.1	cysteine-desulfurase	GCA_000191805.1	383	Bacteria>Terrabacteria_group>Firmicutes>Clostridia>Eubacteriales	Lachnospiraceae_bacterium_6_1_37FAA	hypothetical-protein-HMPREF0490_01181-[Lachnospiraceae-bacterium-6_1_37FAA]	658656
EGC74034.1	cysteine-desulfurase	GCA_000191805.1	289	Bacteria>Terrabacteria_group>Firmicutes>Clostridia>Eubacteriales	Lachnospiraceae_bacterium_6_1_37FAA	hypothetical-protein-HMPREF0490_02541-[Lachnospiraceae-bacterium-6_1_37FAA]	658656
EOS47730.1	cysteine-desulfurase	GCA_000403315.2	381	Bacteria>Terrabacteria_group>Firmicutes>Clostridia>Eubacteriales	Lachnospiraceae_bacterium_A2	cysteine-desulfurase-[Lachnospiraceae-bacterium-A2]	397290
EOS24698.1	cysteine-desulfurase	GCA_000403215.2	373	Bacteria>Terrabacteria_group>Firmicutes>Clostridia>Eubacteriales	Lachnospiraceae_bacterium_A4	cysteine-desulfurase-[Lachnospiraceae-bacterium-A4]	397291
MBD5471455.1	cysteine-desulfurase	GCA_014804285.1	383	Bacteria>Terrabacteria_group>Firmicutes>Clostridia>Eubacteriales	Lachnospiraceae_bacterium	aminotransferase-class-V-fold-PLP-dependent-enzyme-[Lachnospiraceae-bacterium]	1898203
MBD5482605.1	cysteine-desulfurase	GCA_014804425.1	373	Bacteria>Terrabacteria_group>Firmicutes>Clostridia>Eubacteriales	Lachnospiraceae_bacterium	aminotransferase-class-V-fold-PLP-dependent-enzyme-[Lachnospiraceae-bacterium]	1898203
MBD5530085.1	cysteine-desulfurase	GCA_014804645.1	383	Bacteria>Terrabacteria_group>Firmicutes>Clostridia>Eubacteriales	Lachnospiraceae_bacterium	aminotransferase-class-V-fold-PLP-dependent-enzyme-[Lachnospiraceae-bacterium]	1898203
MBE5850727.1	cysteine-desulfurase	GCA_015056775.1	376	Bacteria>Terrabacteria_group>Firmicutes>Clostridia>Eubacteriales	Lachnospiraceae_bacterium	aminotransferase-class-V-fold-PLP-dependent-enzyme-[Lachnospiraceae-bacterium]	1898203
MBE5958717.1	cysteine-desulfurase	GCA_015057625.1	383	Bacteria>Terrabacteria_group>Firmicutes>Clostridia>Eubacteriales	Lachnospiraceae_bacterium	aminotransferase-class-V-fold-PLP-dependent-enzyme-[Lachnospiraceae-bacterium]	1898203
MBO5469402.1	cysteine-desulfurase	GCA_017626215.1	385	Bacteria>Terrabacteria_group>Firmicutes>Clostridia>Eubacteriales	Lachnospiraceae_bacterium	aminotransferase-class-V-fold-PLP-dependent-enzyme-[Lachnospiraceae-bacterium]	1898203
MBP3238903.1	cysteine-desulfurase	GCA_017934205.1	378	Bacteria>Terrabacteria_group>Firmicutes>Clostridia>Eubacteriales	Lachnospiraceae_bacterium	aminotransferase-class-V-fold-PLP-dependent-enzyme-[Lachnospiraceae-bacterium]	1898203
MBP3476983.1	cysteine-desulfurase	GCA_017936885.1	381	Bacteria>Terrabacteria_group>Firmicutes>Clostridia>Eubacteriales	Lachnospiraceae_bacterium	aminotransferase-class-V-fold-PLP-dependent-enzyme-[Lachnospiraceae-bacterium]	1898203
MBP3505195.1	cysteine-desulfurase	GCA_017937345.1	383	Bacteria>Terrabacteria_group>Firmicutes>Clostridia>Eubacteriales	Lachnospiraceae_bacterium	aminotransferase-class-V-fold-PLP-dependent-enzyme-[Lachnospiraceae-bacterium]	1898203
MBP8715376.1	cysteine-desulfurase	GCA_018053995.1	254	Bacteria>Terrabacteria_group>Firmicutes>Clostridia>Eubacteriales	Lachnospiraceae_bacterium	aminotransferase-class-V-fold-PLP-dependent-enzyme-[Lachnospiraceae-bacterium]	1898203
MBQ1993676.1	cysteine-desulfurase	GCA_017391645.1	384	Bacteria>Terrabacteria_group>Firmicutes>Clostridia>Eubacteriales	Lachnospiraceae_bacterium	aminotransferase-class-V-fold-PLP-dependent-enzyme-[Lachnospiraceae-bacterium]	1898203
MBQ2320513.1	cysteine-desulfurase	GCA_017425575.1	384	Bacteria>Terrabacteria_group>Firmicutes>Clostridia>Eubacteriales	Lachnospiraceae_bacterium	aminotransferase-class-V-fold-PLP-dependent-enzyme-[Lachnospiraceae-bacterium]	1898203
MBQ2800543.1	cysteine-desulfurase	GCA_017408735.1	397	Bacteria>Terrabacteria_group>Firmicutes>Clostridia>Eubacteriales	Lachnospiraceae_bacterium	aminotransferase-class-V-fold-PLP-dependent-enzyme-[Lachnospiraceae-bacterium]	1898203
MBQ3165691.1	cysteine-desulfurase	GCA_017397345.1	383	Bacteria>Terrabacteria_group>Firmicutes>Clostridia>Eubacteriales	Lachnospiraceae_bacterium	aminotransferase-class-V-fold-PLP-dependent-enzyme-[Lachnospiraceae-bacterium]	1898203
MBQ4531007.1	cysteine-desulfurase	GCA_017399445.1	413	Bacteria>Terrabacteria_group>Firmicutes>Clostridia>Eubacteriales	Lachnospiraceae_bacterium	aminotransferase-class-V-fold-PLP-dependent-enzyme-[Lachnospiraceae-bacterium]	1898203
MBQ5734818.1	cysteine-desulfurase	GCA_017383525.1	381	Bacteria>Terrabacteria_group>Firmicutes>Clostridia>Eubacteriales	Lachnospiraceae_bacterium	aminotransferase-class-V-fold-PLP-dependent-enzyme-[Lachnospiraceae-bacterium]	1898203
MBR2949463.1	cysteine-desulfurase	GCA_017500935.1	383	Bacteria>Terrabacteria_group>Firmicutes>Clostridia>Eubacteriales	Lachnospiraceae_bacterium	aminotransferase-class-V-fold-PLP-dependent-enzyme-[Lachnospiraceae-bacterium]	1898203
MBR6223845.1	cysteine-desulfurase	GCA_017540605.1	382	Bacteria>Terrabacteria_group>Firmicutes>Clostridia>Eubacteriales	Lachnospiraceae_bacterium	aminotransferase-class-V-fold-PLP-dependent-enzyme-[Lachnospiraceae-bacterium]	1898203
MBS5672760.1	cysteine-desulfurase	GCA_018370355.1	396	Bacteria>Terrabacteria_group>Firmicutes>Clostridia>Eubacteriales	Lachnospiraceae_bacterium	aminotransferase-class-V-fold-PLP-dependent-enzyme-[Lachnospiraceae-bacterium]	1898203
MBS6812595.1	cysteine-desulfurase	GCA_018379615.1	378	Bacteria>Terrabacteria_group>Firmicutes>Clostridia>Eubacteriales	Lachnospiraceae_bacterium	aminotransferase-class-V-fold-PLP-dependent-enzyme-[Lachnospiraceae-bacterium]	1898203
MBS6922323.1	cysteine-desulfurase	GCA_018380715.1	383	Bacteria>Terrabacteria_group>Firmicutes>Clostridia>Eubacteriales	Lachnospiraceae_bacterium	aminotransferase-class-V-fold-PLP-dependent-enzyme-[Lachnospiraceae-bacterium]	1898203
NBJ01288.1	cysteine-desulfurase	GCA_009911175.1	382	Bacteria>Terrabacteria_group>Firmicutes>Clostridia>Eubacteriales	Lachnospiraceae_bacterium	aminotransferase-class-V-fold-PLP-dependent-enzyme-[Lachnospiraceae-bacterium]	1898203
TGY91524.1	cysteine-desulfurase	GCA_004793545.1	386	Bacteria>Terrabacteria_group>Firmicutes>Clostridia>Eubacteriales	Lachnospiraceae_bacterium	aminotransferase-class-V-fold-PLP-dependent-enzyme-[Lachnospiraceae-bacterium]	1898203
HAP20128.1	cysteine-desulfurase	GCA_003454905.1	383	Bacteria>Terrabacteria_group>Firmicutes>Clostridia>Eubacteriales	Lachnospiraceae_bacterium	cysteine-desulfurase-DndA-[Lachnospiraceae-bacterium]	1898203
HCJ08600.1	cysteine-desulfurase	GCA_003530125.1	382	Bacteria>Terrabacteria_group>Firmicutes>Clostridia>Eubacteriales	Lachnospiraceae_bacterium	cysteine-desulfurase-DndA-[Lachnospiraceae-bacterium]	1898203
PWL93728.1	cysteine-desulfurase	GCA_003150445.1	383	Bacteria>Terrabacteria_group>Firmicutes>Clostridia>Eubacteriales	Lachnospiraceae_bacterium	cysteine-desulfurase-DndA-[Lachnospiraceae-bacterium]	1898203
GFH97090.1	cysteine-desulfurase	GCA_011959105.1	383	Bacteria>Terrabacteria_group>Firmicutes>Clostridia>Eubacteriales	Lachnospiraceae_bacterium	cysteine-desulfurase-IscS-[Lachnospiraceae-bacterium]	1898203
GFI65387.1	cysteine-desulfurase	GCA_011958345.1	376	Bacteria>Terrabacteria_group>Firmicutes>Clostridia>Eubacteriales	Lachnospiraceae_bacterium	cysteine-desulfurase-IscS-[Lachnospiraceae-bacterium]	1898203
GKH57544.1	cysteine-desulfurase	GCA_022835195.1	375	Bacteria>Terrabacteria_group>Firmicutes>Clostridia>Eubacteriales	Lachnospiraceae_bacterium	cysteine-desulfurase-[Lachnospiraceae-bacterium]	1898203
HBH98917.1	cysteine-desulfurase	GCA_003507655.1	396	Bacteria>Terrabacteria_group>Firmicutes>Clostridia>Eubacteriales	Lachnospiraceae_bacterium	cysteine-desulfurase-[Lachnospiraceae-bacterium]	1898203
HCJ09284.1	cysteine-desulfurase	GCA_003530125.1	385	Bacteria>Terrabacteria_group>Firmicutes>Clostridia>Eubacteriales	Lachnospiraceae_bacterium	cysteine-desulfurase-[Lachnospiraceae-bacterium]	1898203
MBD5135516.1	cysteine-desulfurase	GCA_014799465.1	382	Bacteria>Terrabacteria_group>Firmicutes>Clostridia>Eubacteriales	Lachnospiraceae_bacterium	cysteine-desulfurase-[Lachnospiraceae-bacterium]	1898203
MBD5476836.1	cysteine-desulfurase	GCA_014804365.1	397	Bacteria>Terrabacteria_group>Firmicutes>Clostridia>Eubacteriales	Lachnospiraceae_bacterium	cysteine-desulfurase-[Lachnospiraceae-bacterium]	1898203
MBQ3784035.1	cysteine-desulfurase	GCA_017413205.1	397	Bacteria>Terrabacteria_group>Firmicutes>Clostridia>Eubacteriales	Lachnospiraceae_bacterium	cysteine-desulfurase-[Lachnospiraceae-bacterium]	1898203
SDA76889.1	cysteine-desulfurase	GCA_900103495.1	383	Bacteria>Terrabacteria_group>Firmicutes>Clostridia>Eubacteriales	Lachnospiraceae_bacterium_G11	cysteine-desulfurase-[Lachnospiraceae-bacterium-G11]	1200747
EHO51747.1	cysteine-desulfurase	GCA_000242315.1	397	Bacteria>Terrabacteria_group>Firmicutes>Clostridia>Eubacteriales	Lachnospiraceae_bacterium_oral_taxon_082_str._F0431	aminotransferase,-class-V-[Lachnospiraceae-bacterium-oral-taxon-082-str.-F0431]	861454
PTL28734.1	cysteine-desulfurase	GCA_003043955.1	376	Bacteria>Terrabacteria_group>Firmicutes>Clostridia>Eubacteriales	Lachnospiraceae_bacterium_oral_taxon_096	cysteine-desulfurase-DndA-[Lachnospiraceae-bacterium-oral-taxon-096]	712982
SCY77701.1	cysteine-desulfurase	GCA_900102235.1	379	Bacteria>Terrabacteria_group>Firmicutes>Clostridia>Eubacteriales	Lachnospiraceae_bacterium_XBB2008	cysteine-desulfurase-[Lachnospiraceae-bacterium-XBB2008]	1520826
SFT61113.1	cysteine-desulfurase	GCA_900116395.1	383	Bacteria>Terrabacteria_group>Firmicutes>Clostridia>Eubacteriales	Lachnospiraceae_bacterium_XBD2001	cysteine-desulfurase-[Lachnospiraceae-bacterium-XBD2001]	1520820
WP_055297702.1	cysteine-desulfurase	GCF_001405795.1	383	Bacteria>Terrabacteria_group>Firmicutes>Clostridia>Eubacteriales	Lachnospiraceae	MULTISPECIES:-cysteine-desulfurase-[Lachnospiraceae]	186803
WP_118147945.1	cysteine-desulfurase	GCF_003470215.1	386	Bacteria>Terrabacteria_group>Firmicutes>Clostridia>Eubacteriales	Lachnospira_eligens	aminotransferase-class-V-fold-PLP-dependent-enzyme-[Lachnospira-eligens]	39485
OLA16392.1	cysteine-desulfurase	GCA_001916475.1	379	Bacteria>Terrabacteria_group>Firmicutes>Clostridia>Eubacteriales	Lachnospira_eligens	hypothetical-protein-BHW24_04535-[Lachnospira-eligens]	39485
WP_097006583.1	cysteine-desulfurase	GCF_900205965.1	383	Bacteria>Terrabacteria_group>Firmicutes>Clostridia>Eubacteriales	Lacrimispora_amygdalina	cysteine-desulfurase-[Lacrimispora-amygdalina]	253257
WP_100306402.1	cysteine-desulfurase	GCF_002797975.1	383	Bacteria>Terrabacteria_group>Firmicutes>Clostridia>Eubacteriales	Lacrimispora_celerecrescens	aminotransferase-class-V-fold-PLP-dependent-enzyme-[Lacrimispora-celerecrescens]	29354
MCF2657513.1	cysteine-desulfurase	GCA_021531795.1	380	Bacteria>Terrabacteria_group>Firmicutes>Clostridia>Eubacteriales	Lacrimispora_saccharolytica	aminotransferase-class-V-fold-PLP-dependent-enzyme-[Lacrimispora-saccharolytica]	84030
WP_186908955.1	cysteine-desulfurase	GCF_014287895.1	377	Bacteria>Terrabacteria_group>Firmicutes>Clostridia>Eubacteriales	Lawsonibacter_sp._NSJ-51	aminotransferase-class-V-fold-PLP-dependent-enzyme-[Lawsonibacter-sp.-NSJ-51]	2763053
WP_079492857.1	cysteine-desulfurase	GCF_900167445.1	392	Bacteria>Terrabacteria_group>Firmicutes>Clostridia>Eubacteriales	Maledivibacter_halophilus	cysteine-desulfurase-[Maledivibacter-halophilus]	36842
WP_215640146.1	cysteine-desulfurase	GCF_018783965.1	383	Bacteria>Terrabacteria_group>Firmicutes>Clostridia>Eubacteriales	Mediterraneibacter_faecis	aminotransferase-class-V-fold-PLP-dependent-enzyme-[Mediterraneibacter-faecis]	592978
WP_227182289.1	cysteine-desulfurase	GCF_020563625.1	320	Bacteria>Terrabacteria_group>Firmicutes>Clostridia>Eubacteriales	Mediterraneibacter_faecis	cysteine-desulfurase-[Mediterraneibacter-faecis]	592978
MCF2569358.1	cysteine-desulfurase	GCA_021530965.1	383	Bacteria>Terrabacteria_group>Firmicutes>Clostridia>Eubacteriales	Mediterraneibacter_glycyrrhizinilyticus	aminotransferase-class-V-fold-PLP-dependent-enzyme-[Mediterraneibacter-glycyrrhizinilyticus]	342942
WP_132283195.1	cysteine-desulfurase	-	390	Bacteria>Terrabacteria_group>Firmicutes>Clostridia>Eubacteriales	Natranaerovirga_hydrolytica	aminotransferase-class-V-fold-PLP-dependent-enzyme-[Natranaerovirga-hydrolytica]	680378
WP_179012280.1	cysteine-desulfurase	GCF_013394775.1	383	Bacteria>Terrabacteria_group>Firmicutes>Clostridia>Eubacteriales	Oribacterium_sp._oral_taxon_102	aminotransferase-class-V-fold-PLP-dependent-enzyme-[Oribacterium-sp.-oral-taxon-102]	671214
WP_016320961.1	cysteine-desulfurase	GCF_000403435.2	381	Bacteria>Terrabacteria_group>Firmicutes>Clostridia>Eubacteriales	Oscillibacter_sp._1-3	aminotransferase-class-V-fold-PLP-dependent-enzyme-[Oscillibacter-sp.-1-3]	1235797
MUU10522.1	cysteine-desulfurase	GCA_009739395.1	376	Bacteria>Terrabacteria_group>Firmicutes>Clostridia>Eubacteriales	Oscillibacter_sp.	cysteine-desulfurase-[Oscillibacter-sp.]	1945593
MBP0982396.1	cysteine-desulfurase	GCA_017829075.1	379	Bacteria>Terrabacteria_group>Firmicutes>Clostridia>Eubacteriales	Oscillospiraceae_bacterium	aminotransferase-class-V-fold-PLP-dependent-enzyme-[Oscillospiraceae-bacterium]	2485925
MBQ8012217.1	cysteine-desulfurase	GCA_017468645.1	185	Bacteria>Terrabacteria_group>Firmicutes>Clostridia>Eubacteriales	Oscillospiraceae_bacterium	aminotransferase-class-V-fold-PLP-dependent-enzyme-[Oscillospiraceae-bacterium]	2485925
MBR0451914.1	cysteine-desulfurase	GCA_017432285.1	381	Bacteria>Terrabacteria_group>Firmicutes>Clostridia>Eubacteriales	Oscillospiraceae_bacterium	aminotransferase-class-V-fold-PLP-dependent-enzyme-[Oscillospiraceae-bacterium]	2485925
BDF68163.1	cysteine-desulfurase	GCA_022846075.1	381	Bacteria>Terrabacteria_group>Firmicutes>Clostridia>Eubacteriales	Oscillospiraceae_bacterium	cysteine-desulfurase-[Oscillospiraceae-bacterium]	2485925
MBQ6207344.1	cysteine-desulfurase	GCA_017428815.1	377	Bacteria>Terrabacteria_group>Firmicutes>Clostridia>Eubacteriales	Oscillospiraceae_bacterium	cysteine-desulfurase-[Oscillospiraceae-bacterium]	2485925
MBQ9168110.1	cysteine-desulfurase	GCA_017463645.1	376	Bacteria>Terrabacteria_group>Firmicutes>Clostridia>Eubacteriales	Oscillospiraceae_bacterium	cysteine-desulfurase-[Oscillospiraceae-bacterium]	2485925
BDF69813.1	cysteine-desulfurase	GCA_022846095.1	397	Bacteria>Terrabacteria_group>Firmicutes>Clostridia>Eubacteriales	Oscillospiraceae_bacterium	IscS-subfamily-cysteine-desulfurase-[Oscillospiraceae-bacterium]	2485925
WP_084234538.1	cysteine-desulfurase	GCF_900176335.1	383	Bacteria>Terrabacteria_group>Firmicutes>Clostridia>Eubacteriales	Papillibacter_cinnamivorans	aminotransferase-class-V-fold-PLP-dependent-enzyme-[Papillibacter-cinnamivorans]	100176
WP_142535459.1	cysteine-desulfurase	GCF_006861675.1	374	Bacteria>Terrabacteria_group>Firmicutes>Clostridia>Eubacteriales	Peptacetobacter_hominis	alanine–glyoxylate-aminotransferase-family-protein-[Peptacetobacter-hominis]	2743610
KJS45990.1	cysteine-desulfurase	GCA_000961805.1	385	Bacteria>Terrabacteria_group>Firmicutes>Clostridia>Eubacteriales	Peptococcaceae_bacterium_BRH_c23	cysteine-desulfurase-[Peptococcaceae-bacterium-BRH_c23]	1629714
KJS03672.1	cysteine-desulfurase	GCA_000961585.1	376	Bacteria>Terrabacteria_group>Firmicutes>Clostridia>Eubacteriales	Peptococcaceae_bacterium_BRH_c4a	cysteine-desulfurase-[Peptococcaceae-bacterium-BRH_c4a]	1629716
KJS16035.1	cysteine-desulfurase	GCA_000961595.1	381	Bacteria>Terrabacteria_group>Firmicutes>Clostridia>Eubacteriales	Peptococcaceae_bacterium_BRH_c4b	cysteine-desulfurase-[Peptococcaceae-bacterium-BRH_c4b]	1629717
WP_211802388.1	cysteine-desulfurase	GCF_018128025.1	374	Bacteria>Terrabacteria_group>Firmicutes>Clostridia>Eubacteriales	Proteiniclasticum_sediminis	aminotransferase-class-V-fold-PLP-dependent-enzyme-[Proteiniclasticum-sp.-BAD-10]	2804028
WP_036944774.1	cysteine-desulfurase	GCF_000745845.1	385	Bacteria>Terrabacteria_group>Firmicutes>Clostridia>Eubacteriales	Pseudobacteroides_cellulosolvens	aminotransferase-class-V-fold-PLP-dependent-enzyme-[Pseudobacteroides-cellulosolvens]	35825
WP_044937802.1	cysteine-desulfurase	GCF_000701765.1	379	Bacteria>Terrabacteria_group>Firmicutes>Clostridia>Eubacteriales	Pseudobutyrivibrio_sp._LB2011	aminotransferase-class-V-fold-PLP-dependent-enzyme-[Pseudobutyrivibrio-sp.-LB2011]	1408312
WP_072918940.1	cysteine-desulfurase	GCF_900141825.1	378	Bacteria>Terrabacteria_group>Firmicutes>Clostridia>Eubacteriales	Pseudobutyrivibrio_xylanivorans	aminotransferase-class-V-fold-PLP-dependent-enzyme-[Pseudobutyrivibrio-xylanivorans]	185007
WP_193536495.1	cysteine-desulfurase	GCF_014982855.1	378	Bacteria>Terrabacteria_group>Firmicutes>Clostridia>Eubacteriales	Pseudoflavonifractor_gallinarum	cysteine-desulfurase-[Pseudoflavonifractor-gallinarum]	2779352
WP_204889867.1	cysteine-desulfurase	GCF_016902175.1	377	Bacteria>Terrabacteria_group>Firmicutes>Clostridia>Eubacteriales	Pseudoflavonifractor_phocaeensis	cysteine-desulfurase-[Pseudoflavonifractor-phocaeensis]	1870988
WP_161845270.1	cysteine-desulfurase	GCF_009935845.1	381	Bacteria>Terrabacteria_group>Firmicutes>Clostridia>Eubacteriales	Pseudoflavonifractor_sp._524-17	aminotransferase-class-V-fold-PLP-dependent-enzyme-[Pseudoflavonifractor-sp.-524-17]	2304577
WP_087390692.1	cysteine-desulfurase	GCF_002160275.1	381	Bacteria>Terrabacteria_group>Firmicutes>Clostridia>Eubacteriales	Pseudoflavonifractor_sp._An187	aminotransferase-class-V-fold-PLP-dependent-enzyme-[Pseudoflavonifractor-sp.-An187]	1965578
WP_215662665.1	cysteine-desulfurase	GCF_018784025.1	397	Bacteria>Terrabacteria_group>Firmicutes>Clostridia>Eubacteriales	Roseburia_hominis	aminotransferase-class-V-fold-PLP-dependent-enzyme-[Roseburia-hominis]	301301
MBS5515432.1	cysteine-desulfurase	GCA_018368795.1	384	Bacteria>Terrabacteria_group>Firmicutes>Clostridia>Eubacteriales	Roseburia_intestinalis	aminotransferase-class-V-fold-PLP-dependent-enzyme-[Roseburia-intestinalis]	166486
WP_006855544.1	cysteine-desulfurase	GCF_000209995.1	384	Bacteria>Terrabacteria_group>Firmicutes>Clostridia>Eubacteriales	Roseburia_intestinalis	aminotransferase-class-V-fold-PLP-dependent-enzyme-[Roseburia-intestinalis]	166486
WP_118772795.1	cysteine-desulfurase	GCF_003471045.1	384	Bacteria>Terrabacteria_group>Firmicutes>Clostridia>Eubacteriales	Roseburia_intestinalis	aminotransferase-class-V-fold-PLP-dependent-enzyme-[Roseburia-intestinalis]	166486
OLA56472.1	cysteine-desulfurase	GCA_001916935.1	384	Bacteria>Terrabacteria_group>Firmicutes>Clostridia>Eubacteriales	Roseburia_intestinalis	cysteine-desulfurase-[Roseburia-intestinalis]	166486
WP_044928373.1	cysteine-desulfurase	GCF_000174195.1	390	Bacteria>Terrabacteria_group>Firmicutes>Clostridia>Eubacteriales	Roseburia_inulinivorans	aminotransferase-class-V-fold-PLP-dependent-enzyme-[Roseburia-inulinivorans]	360807
WP_118203375.1	cysteine-desulfurase	GCF_003470885.1	381	Bacteria>Terrabacteria_group>Firmicutes>Clostridia>Eubacteriales	Roseburia_inulinivorans	aminotransferase-class-V-fold-PLP-dependent-enzyme-[Roseburia-inulinivorans]	360807
WP_021922312.1	cysteine-desulfurase	GCF_001406855.1	397	Bacteria>Terrabacteria_group>Firmicutes>Clostridia>Eubacteriales	Roseburia_inulinivorans	cysteine-desulfurase-[Roseburia-inulinivorans]	360807
WP_138346254.1	cysteine-desulfurase	GCF_015669935.1	397	Bacteria>Terrabacteria_group>Firmicutes>Clostridia>Eubacteriales	Roseburia	MULTISPECIES:-cysteine-desulfurase-[Roseburia]	841
OLA92692.1	cysteine-desulfurase	GCA_001916875.1	396	Bacteria>Terrabacteria_group>Firmicutes>Clostridia>Eubacteriales	Roseburia_sp._40_7	cysteine-desulfurase-[Roseburia-sp.-40_7]	1897015
WP_075679824.1	cysteine-desulfurase	GCF_001940165.1	384	Bacteria>Terrabacteria_group>Firmicutes>Clostridia>Eubacteriales	Roseburia_sp._831b	aminotransferase-class-V-fold-PLP-dependent-enzyme-[Roseburia-sp.-831b]	1261635
WP_118210363.1	cysteine-desulfurase	GCF_003470905.1	384	Bacteria>Terrabacteria_group>Firmicutes>Clostridia>Eubacteriales	Roseburia_sp._AM23-20	aminotransferase-class-V-fold-PLP-dependent-enzyme-[Roseburia-sp.-AM23-20]	2292066
MBD5555490.1	cysteine-desulfurase	GCA_014804775.1	376	Bacteria>Terrabacteria_group>Firmicutes>Clostridia>Eubacteriales	Roseburia_sp.	aminotransferase-class-V-fold-PLP-dependent-enzyme-[Roseburia-sp.]	2049040
MBS5096683.1	cysteine-desulfurase	GCA_018365575.1	395	Bacteria>Terrabacteria_group>Firmicutes>Clostridia>Eubacteriales	Roseburia_sp.	aminotransferase-class-V-fold-PLP-dependent-enzyme-[Roseburia-sp.]	2049040
MBS6242007.1	cysteine-desulfurase	GCA_018374815.1	395	Bacteria>Terrabacteria_group>Firmicutes>Clostridia>Eubacteriales	Roseburia_sp.	aminotransferase-class-V-fold-PLP-dependent-enzyme-[Roseburia-sp.]	2049040
MBS6820668.1	cysteine-desulfurase	GCA_018379735.1	382	Bacteria>Terrabacteria_group>Firmicutes>Clostridia>Eubacteriales	Roseburia_sp.	aminotransferase-class-V-fold-PLP-dependent-enzyme-[Roseburia-sp.]	2049040
WP_207723360.1	cysteine-desulfurase	GCF_014287635.1	393	Bacteria>Terrabacteria_group>Firmicutes>Clostridia>Eubacteriales	Roseburia_sp._BX1005	aminotransferase-class-V-fold-PLP-dependent-enzyme-[Roseburia-sp.-BX1005]	2763064
HCQ06277.1	cysteine-desulfurase	GCA_003525405.1	383	Bacteria>Terrabacteria_group>Firmicutes>Clostridia>Eubacteriales	Roseburia_sp.	cysteine-desulfurase-DndA-[Roseburia-sp.]	2049040
HCW30619.1	cysteine-desulfurase	GCA_003541855.1	227	Bacteria>Terrabacteria_group>Firmicutes>Clostridia>Eubacteriales	Roseburia_sp.	cysteine-desulfurase-[Roseburia-sp.]	2049040
MBS5230493.1	cysteine-desulfurase	GCA_018366635.1	383	Bacteria>Terrabacteria_group>Firmicutes>Clostridia>Eubacteriales	Roseburia_sp.	cysteine-desulfurase-[Roseburia-sp.]	2049040

Accession	Annotation	GCA	Length	Phyletic.spread	Strain.name	Defline	Species.Taxid
MBS6959097.1	cysteine-desulfurase	GCA_018380935.1	396	Bacteria>Terrabacteria_group>Firmicutes>Clostridia>Eubacteriales	Roseburia_sp.	cysteine-desulfurase-[Roseburia-sp.]	2049040
WP_186899187.1	cysteine-desulfurase	GCF_014287515.2	383	Bacteria>Terrabacteria_group>Firmicutes>Clostridia>Eubacteriales	Roseburia_sp._NSJ-69	aminotransferase-class-V-fold-PLP-dependent-enzyme-[Roseburia-sp.-NSJ-69]	2763062
WP_186899576.1	cysteine-desulfurase	GCF_014287515.2	384	Bacteria>Terrabacteria_group>Firmicutes>Clostridia>Eubacteriales	Roseburia_sp._NSJ-69	aminotransferase-class-V-fold-PLP-dependent-enzyme-[Roseburia-sp.-NSJ-69]	2763062
EGD45822.1	cysteine-desulfurase	GCA_000175795.2	396	Bacteria>Terrabacteria_group>Firmicutes>Clostridia>Eubacteriales	Ruminiclostridium_papyrosolvens_DSM_2782	LOW-QUALITY-PROTEIN:-Cysteine-desulfurase-[Ruminiclostridium-papyrosolvens-DSM-2782]	588581
WP_110462931.1	cysteine-desulfurase	GCF_003208175.1	373	Bacteria>Terrabacteria_group>Firmicutes>Clostridia>Eubacteriales	Ruminiclostridium_sufflavum	aminotransferase-class-V-fold-PLP-dependent-enzyme-[Ruminiclostridium-sufflavum]	396504
RHT74341.1	cysteine-desulfurase	GCA_003480625.1	376	Bacteria>Terrabacteria_group>Firmicutes>Clostridia>Eubacteriales	Ruminococcaceae_bacterium_AM28-23LB	cysteine-desulfurase-[Ruminococcaceae-bacterium-AM28-23LB]	2292269
WP_002852496.1	cysteine-desulfurase	GCF_000178155.2	382	Bacteria>Terrabacteria_group>Firmicutes>Clostridia>Eubacteriales	Ruminococcus_albus	aminotransferase-class-V-fold-PLP-dependent-enzyme-[Ruminococcus-albus]	1264
WP_037287154.1	cysteine-desulfurase	GCF_000586615.1	397	Bacteria>Terrabacteria_group>Firmicutes>Clostridia>Eubacteriales	Ruminococcus_albus	cysteine-desulfurase-[Ruminococcus-albus]	1264
MBD8913304.1	cysteine-desulfurase	GCA_014848475.1	378	Bacteria>Terrabacteria_group>Firmicutes>Clostridia>Eubacteriales	Ruminococcus_bicirculans	aminotransferase-class-V-fold-PLP-dependent-enzyme-[Ruminococcus-bicirculans]	1160721
WP_195246819.1	cysteine-desulfurase	GCF_015548975.1	378	Bacteria>Terrabacteria_group>Firmicutes>Clostridia>Eubacteriales	Ruminococcus_bicirculans	aminotransferase-class-V-fold-PLP-dependent-enzyme-[Ruminococcus-bicirculans]	1160721
WP_186489890.1	cysteine-desulfurase	GCF_014269305.1	397	Bacteria>Terrabacteria_group>Firmicutes>Clostridia>Eubacteriales	Ruminococcus_bicirculans	cysteine-desulfurase-[Ruminococcus-bicirculans]	1160721
WP_207732989.1	cysteine-desulfurase	-	387	Bacteria>Terrabacteria_group>Firmicutes>Clostridia>Eubacteriales	Ruminococcus_bromii	aminotransferase-class-V-fold-PLP-dependent-enzyme-[Ruminococcus-bromii]	40518
WP_041326546.1	cysteine-desulfurase	-	379	Bacteria>Terrabacteria_group>Firmicutes>Clostridia>Eubacteriales	Ruminococcus_champanellensis	aminotransferase-class-V-fold-PLP-dependent-enzyme-[Ruminococcus-champanellensis]	1161942
WP_109727350.1	cysteine-desulfurase	GCF_003149325.1	379	Bacteria>Terrabacteria_group>Firmicutes>Clostridia>Eubacteriales	Ruminococcus_flavefaciens	aminotransferase-class-V-fold-PLP-dependent-enzyme-[Ruminococcus-flavefaciens]	1265
WP_019680706.1	cysteine-desulfurase	GCF_000247525.1	395	Bacteria>Terrabacteria_group>Firmicutes>Clostridia>Eubacteriales	Ruminococcus_flavefaciens	cysteine-desulfurase-[Ruminococcus-flavefaciens]	1265
WP_101878791.1	cysteine-desulfurase	GCF_002865425.1	383	Bacteria>Terrabacteria_group>Firmicutes>Clostridia>Eubacteriales	[Ruminococcus]_gnavus	aminotransferase-class-V-fold-PLP-dependent-enzyme-[[Ruminococcus]	33038
WP_009244794.1	cysteine-desulfurase	GCF_902374425.1	397	Bacteria>Terrabacteria_group>Firmicutes>Clostridia>Eubacteriales	[Ruminococcus]_gnavus	cysteine-desulfurase-[[Ruminococcus]	33038
OLA68280.1	cysteine-desulfurase	GCA_001917035.1	379	Bacteria>Terrabacteria_group>Firmicutes>Clostridia>Eubacteriales	Ruminococcus_sp._37_24	cysteine-desulfurase-[Ruminococcus-sp.-37_24]	1897019
WP_117519421.1	cysteine-desulfurase	GCF_003435955.1	402	Bacteria>Terrabacteria_group>Firmicutes>Clostridia>Eubacteriales	Ruminococcus_sp._AF43-11	cysteine-desulfurase-[Ruminococcus-sp.-AF43-11]	2293181
WP_118158240.1	cysteine-desulfurase	GCF_003462045.1	382	Bacteria>Terrabacteria_group>Firmicutes>Clostridia>Eubacteriales	Ruminococcus_sp._AM31-15AC	aminotransferase-class-V-fold-PLP-dependent-enzyme-[Ruminococcus-sp.-AM31-15AC]	2293202
MBD9049687.1	cysteine-desulfurase	GCA_014848155.1	387	Bacteria>Terrabacteria_group>Firmicutes>Clostridia>Eubacteriales	Ruminococcus_sp.	aminotransferase-class-V-fold-PLP-dependent-enzyme-[Ruminococcus-sp.]	41978
MCC8195210.1	cysteine-desulfurase	GCA_020861185.1	381	Bacteria>Terrabacteria_group>Firmicutes>Clostridia>Eubacteriales	Ruminococcus_sp.	aminotransferase-class-V-fold-PLP-dependent-enzyme-[Ruminococcus-sp.]	41978
MBQ6943024.1	cysteine-desulfurase	GCA_017437285.1	395	Bacteria>Terrabacteria_group>Firmicutes>Clostridia>Eubacteriales	Ruminococcus_sp.	cysteine-desulfurase-[Ruminococcus-sp.]	41978
MBQ8514244.1	cysteine-desulfurase	GCA_017472145.1	377	Bacteria>Terrabacteria_group>Firmicutes>Clostridia>Eubacteriales	Ruminococcus_sp.	cysteine-desulfurase-[Ruminococcus-sp.]	41978
WP_195478638.1	cysteine-desulfurase	GCF_015554365.1	382	Bacteria>Terrabacteria_group>Firmicutes>Clostridia>Eubacteriales	Ruminococcus_sp._D55t1_190419_H1	cysteine-desulfurase-[Ruminococcus-sp.-D55t1_190419_H1]	2787130
WP_125128687.1	cysteine-desulfurase	GCF_003885045.1	382	Bacteria>Terrabacteria_group>Firmicutes>Clostridia>Eubacteriales	Schaedlerella_arabinosiphila	cysteine-desulfurase-[Schaedlerella-arabinosiphila]	2044587
WP_221920267.1	cysteine-desulfurase	GCF_019754295.1	383	Bacteria>Terrabacteria_group>Firmicutes>Clostridia>Eubacteriales	Sellimonas_caecigallum	aminotransferase-class-V-fold-PLP-dependent-enzyme-[Sellimonas-caecigallum]	2592333
WP_113998534.1	cysteine-desulfurase	GCF_003324125.1	381	Bacteria>Terrabacteria_group>Firmicutes>Clostridia>Eubacteriales	Subdoligranulum_sp._APC924/74	aminotransferase-class-V-fold-PLP-dependent-enzyme-[Subdoligranulum-sp.-APC924/74]	2086273
WP_020375121.1	cysteine-desulfurase	GCF_000294425.1	380	Bacteria>Terrabacteria_group>Firmicutes>Clostridia>Eubacteriales	Sulfobacillus_thermosulfidooxidans	aminotransferase-class-V-fold-PLP-dependent-enzyme-[Sulfobacillus-thermosulfidooxidans]	28034
NLU49256.1	cysteine-desulfurase	GCA_012719535.1	383	Bacteria>Terrabacteria_group>Firmicutes>Clostridia>Eubacteriales	Syntrophomonadaceae_bacterium	cysteine-desulfurase-[Syntrophomonadaceae-bacterium]	2093811
WP_118594461.1	cysteine-desulfurase	GCF_003479105.1	383	Bacteria>Terrabacteria_group>Firmicutes>Clostridia>Eubacteriales	unclassified_Blautia	MULTISPECIES:-cysteine-desulfurase-[unclassified-Blautia]	2648079
WP_117464669.1	cysteine-desulfurase	GCF_003435925.1	376	Bacteria>Terrabacteria_group>Firmicutes>Clostridia>Eubacteriales	unclassified_Clostridium	MULTISPECIES:-aminotransferase-class-V-fold-PLP-dependent-enzyme-[unclassified-Clostridium]	2614128
WP_221701310.1	cysteine-desulfurase	GCF_019734175.1	392	Bacteria>Terrabacteria_group>Firmicutes>Clostridia>Eubacteriales	unclassified_Clostridium	MULTISPECIES:-aminotransferase-class-V-fold-PLP-dependent-enzyme-[unclassified-Clostridium]	2614128
WP_118589750.1	cysteine-desulfurase	GCF_003479405.1	384	Bacteria>Terrabacteria_group>Firmicutes>Clostridia>Eubacteriales	unclassified_Eubacterium	MULTISPECIES:-aminotransferase-class-V-fold-PLP-dependent-enzyme-[unclassified-Eubacterium]	2624479
WP_118057314.1	cysteine-desulfurase	GCF_003461105.1	373	Bacteria>Terrabacteria_group>Firmicutes>Clostridia>Eubacteriales	unclassified_Roseburia	MULTISPECIES:-aminotransferase-class-V-fold-PLP-dependent-enzyme-[unclassified-Roseburia]	2637578
WP_117903515.1	cysteine-desulfurase	GCF_020559915.1	397	Bacteria>Terrabacteria_group>Firmicutes>Clostridia>Eubacteriales	unclassified_Ruminococcus	MULTISPECIES:-cysteine-desulfurase-[unclassified-Ruminococcus]	2608920
SCG94002.1	cysteine-desulfurase	GCA_900066565.1	383	Bacteria>Terrabacteria_group>Firmicutes>Clostridia>Eubacteriales	uncultured_Clostridium_sp.	Cysteine-desulfurase-[uncultured-Clostridium-sp.]	59620
SCH53762.1	cysteine-desulfurase	GCA_900066575.1	376	Bacteria>Terrabacteria_group>Firmicutes>Clostridia>Eubacteriales	uncultured_Clostridium_sp.	Cysteine-desulfurase-[uncultured-Clostridium-sp.]	59620
SCH61565.1	cysteine-desulfurase	GCA_900066525.1	383	Bacteria>Terrabacteria_group>Firmicutes>Clostridia>Eubacteriales	uncultured_Clostridium_sp.	Cysteine-desulfurase-[uncultured-Clostridium-sp.]	59620
SCJ87470.1	cysteine-desulfurase	GCA_900066245.1	387	Bacteria>Terrabacteria_group>Firmicutes>Clostridia>Eubacteriales	uncultured_Clostridium_sp.	Cysteine-desulfurase-[uncultured-Clostridium-sp.]	59620
SCJ87801.1	cysteine-desulfurase	GCA_900066105.1	393	Bacteria>Terrabacteria_group>Firmicutes>Clostridia>Eubacteriales	uncultured_Eubacterium_sp.	Probable-cysteine-desulfurase-[uncultured-Eubacterium-sp.]	165185
SCG93930.1	cysteine-desulfurase	GCA_900066395.1	381	Bacteria>Terrabacteria_group>Firmicutes>Clostridia>Eubacteriales	uncultured_Roseburia_sp.	Cysteine-desulfurase-[uncultured-Roseburia-sp.]	512314
MBB2181766.1	cysteine-desulfurase	GCA_014174405.1	397	Bacteria>Terrabacteria_group>Firmicutes>Clostridia>Eubacteriales	Variimorphobacter_saccharofermentans	cysteine-desulfurase-[Lachnospiraceae-bacterium-MD1]	2755051
WP_228351521.1	cysteine-desulfurase	GCF_014174405.1	397	Bacteria>Terrabacteria_group>Firmicutes>Clostridia>Eubacteriales	Variimorphobacter_saccharofermentans	cysteine-desulfurase-[Variimorphobacter-saccharofermentans]	2755051
WP_127015492.1	cysteine-desulfurase	GCF_003991135.1	372	Bacteria>Terrabacteria_group>Firmicutes>Clostridia>Halanaerobiales	Anoxybacter_fermentans	cysteine-desulfurase-[Anoxybacter-fermentans]	1323375
TDS26200.1	cysteine-desulfurase	GCA_004364065.1	383	Bacteria>Terrabacteria_group>Firmicutes>Clostridia>Halanaerobiales	Halanaerobium_congolense	cysteine-desulfurase-[Halanaerobium-congolense]	54121
WP_089723322.1	cysteine-desulfurase	GCF_900102975.1	383	Bacteria>Terrabacteria_group>Firmicutes>Clostridia>Halanaerobiales	Halanaerobium_congolense	cysteine-desulfurase-[Halanaerobium-congolense]	54121
WP_015327751.1	cysteine-desulfurase	GCF_000328625.1	381	Bacteria>Terrabacteria_group>Firmicutes>Clostridia>Halanaerobiales	Halobacteroides_halobius	aminotransferase-class-V-fold-PLP-dependent-enzyme-[Halobacteroides-halobius]	42422
WP_078810246.1	cysteine-desulfurase	GCF_900167185.1	381	Bacteria>Terrabacteria_group>Firmicutes>Clostridia>Halanaerobiales	Selenihalanaerobacter_shriftii	aminotransferase-class-V-fold-PLP-dependent-enzyme-[Selenihalanaerobacter-shriftii]	142842
WP_013429102.1	cysteine-desulfurase	GCF_000166775.1	389	Bacteria>Terrabacteria_group>Firmicutes>Clostridia>Thermoanaerobacterales	Caldicellulosiruptor_kronotskyensis	aminotransferase-class-V-fold-PLP-dependent-enzyme-[Caldicellulosiruptor-kronotskyensis]	413889
WP_054935968.1	cysteine-desulfurase	GCF_001373375.1	379	Bacteria>Terrabacteria_group>Firmicutes>Clostridia>Thermoanaerobacterales	Moorella	MULTISPECIES:-cysteine-desulfurase-DndA-[Moorella]	44260
HAA81077.1	cysteine-desulfurase	GCA_003444495.1	389	Bacteria>Terrabacteria_group>Firmicutes>Clostridia>Thermoanaerobacterales	Thermoanaerobacter_sp.	cysteine-desulfurase-[Thermoanaerobacter-sp.]	1755
HHV84038.1	cysteine-desulfurase	GCA_012839965.1	382	Bacteria>Terrabacteria_group>Firmicutes>Clostridia>Thermosediminibacterales	Tepidanaerobacter_syntrophicus	aminotransferase-class-V-fold-PLP-dependent-enzyme-[Tepidanaerobacter-syntrophicus]	224999
MBQ2711363.1	cysteine-desulfurase	GCA_017407405.1	384	Bacteria>Terrabacteria_group>Firmicutes>Clostridia>unclassified_Clostridia	Clostridia_bacterium	aminotransferase-class-V-fold-PLP-dependent-enzyme-[Clostridia-bacterium]	2044939
MBQ3128707.1	cysteine-desulfurase	GCA_017415025.1	377	Bacteria>Terrabacteria_group>Firmicutes>Clostridia>unclassified_Clostridia	Clostridia_bacterium	aminotransferase-class-V-fold-PLP-dependent-enzyme-[Clostridia-bacterium]	2044939
MBQ4457482.1	cysteine-desulfurase	GCA_017394825.1	373	Bacteria>Terrabacteria_group>Firmicutes>Clostridia>unclassified_Clostridia	Clostridia_bacterium	aminotransferase-class-V-fold-PLP-dependent-enzyme-[Clostridia-bacterium]	2044939
MBR1684824.1	cysteine-desulfurase	GCA_017476845.1	383	Bacteria>Terrabacteria_group>Firmicutes>Clostridia>unclassified_Clostridia	Clostridia_bacterium	aminotransferase-class-V-fold-PLP-dependent-enzyme-[Clostridia-bacterium]	2044939
MBR4710575.1	cysteine-desulfurase	GCA_017524925.1	382	Bacteria>Terrabacteria_group>Firmicutes>Clostridia>unclassified_Clostridia	Clostridia_bacterium	aminotransferase-class-V-fold-PLP-dependent-enzyme-[Clostridia-bacterium]	2044939
NCC87248.1	cysteine-desulfurase	GCA_009930155.1	383	Bacteria>Terrabacteria_group>Firmicutes>Clostridia>unclassified_Clostridia	Clostridia_bacterium	aminotransferase-class-V-fold-PLP-dependent-enzyme-[Clostridia-bacterium]	2044939
MBQ8149963.1	cysteine-desulfurase	GCA_017464615.1	376	Bacteria>Terrabacteria_group>Firmicutes>Clostridia>unclassified_Clostridia	Clostridia_bacterium	cysteine-desulfurase-[Clostridia-bacterium]	2044939
MBZ4646659.1	cysteine-desulfurase	GCA_020054655.1	382	Bacteria>Terrabacteria_group>Firmicutes>Clostridia>unclassified_Clostridia	Clostridia_bacterium	cysteine-desulfurase-[Clostridia-bacterium]	2044939
CDD71423.1	cysteine-desulfurase	GCA_000435775.1	383	Bacteria>Terrabacteria_group>Firmicutes>environmental_samples	Firmicutes_bacterium_CAG:270	putative-uncharacterized-protein-[Firmicutes-bacterium-CAG:270]	1263014
CDD63704.1	cysteine-desulfurase	GCA_000435595.1	385	Bacteria>Terrabacteria_group>Firmicutes>environmental_samples	Firmicutes_bacterium_CAG:882	putative-uncharacterized-protein-[Firmicutes-bacterium-CAG:882]	1262991
WP_183327552.1	cysteine-desulfurase	GCF_014202755.1	381	Bacteria>Terrabacteria_group>Firmicutes>Erysipelotrichia>Erysipelotrichales	Catenisphaera_adipataccumulans	aminotransferase-class-V-fold-PLP-dependent-enzyme-[Catenisphaera-adipataccumulans]	700500
WP_044907968.1	cysteine-desulfurase	GCF_000763955.1	381	Bacteria>Terrabacteria_group>Firmicutes>Erysipelotrichia>Erysipelotrichales	[Clostridium]_innocuum	aminotransferase-class-V-fold-PLP-dependent-enzyme-[[Clostridium]	1522
WP_100933613.1	cysteine-desulfurase	GCF_002814255.1	381	Bacteria>Terrabacteria_group>Firmicutes>Erysipelotrichia>Erysipelotrichales	[Clostridium]_innocuum	aminotransferase-class-V-fold-PLP-dependent-enzyme-[[Clostridium]	1522
WP_118654729.1	cysteine-desulfurase	GCF_022135235.1	373	Bacteria>Terrabacteria_group>Firmicutes>Erysipelotrichia>Erysipelotrichales	[Clostridium]_innocuum	aminotransferase-class-V-fold-PLP-dependent-enzyme-[[Clostridium]	1522
WP_242409956.1	cysteine-desulfurase	GCF_022693465.1	381	Bacteria>Terrabacteria_group>Firmicutes>Erysipelotrichia>Erysipelotrichales	[Clostridium]_innocuum	aminotransferase-class-V-fold-PLP-dependent-enzyme-[[Clostridium]	1522
KGJ51436.1	cysteine-desulfurase	GCA_000763955.1	381	Bacteria>Terrabacteria_group>Firmicutes>Erysipelotrichia>Erysipelotrichales	[Clostridium]_innocuum	cysteine-desulfurase-[[Clostridium]	1522
WP_076340715.1	cysteine-desulfurase	GCF_001945605.1	382	Bacteria>Terrabacteria_group>Firmicutes>Erysipelotrichia>Erysipelotrichales	Dubosiella_newyorkensis	cysteine-desulfurase-[Dubosiella-newyorkensis]	1862672
MBS6182773.1	cysteine-desulfurase	GCA_018374395.1	383	Bacteria>Terrabacteria_group>Firmicutes>Erysipelotrichia>Erysipelotrichales	Erysipelotrichaceae_bacterium	aminotransferase-class-V-fold-PLP-dependent-enzyme-[Erysipelotrichaceae-bacterium]	2049044
MCF0259900.1	cysteine-desulfurase	GCA_021443985.1	332	Bacteria>Terrabacteria_group>Firmicutes>Erysipelotrichia>Erysipelotrichales	Erysipelotrichaceae_bacterium	aminotransferase-class-V-fold-PLP-dependent-enzyme-[Erysipelotrichaceae-bacterium]	2049044
MCF0260213.1	cysteine-desulfurase	GCA_021443985.1	382	Bacteria>Terrabacteria_group>Firmicutes>Erysipelotrichia>Erysipelotrichales	Erysipelotrichaceae_bacterium	cysteine-desulfurase-[Erysipelotrichaceae-bacterium]	2049044
WP_183373720.1	cysteine-desulfurase	GCF_014202635.1	383	Bacteria>Terrabacteria_group>Firmicutes>Erysipelotrichia>Erysipelotrichales	Faecalicoccus_acidiformans	cysteine-desulfurase-[Faecalicoccus-acidiformans]	915173
MBD8956862.1	cysteine-desulfurase	GCA_014847315.1	396	Bacteria>Terrabacteria_group>Firmicutes>Erysipelotrichia>Erysipelotrichales	Holdemanella_biformis	cysteine-desulfurase-[Holdemanella-biformis]	1735
WP_125119176.1	cysteine-desulfurase	GCF_003925875.1	382	Bacteria>Terrabacteria_group>Firmicutes>Erysipelotrichia>Erysipelotrichales	Intestinibaculum_porci	aminotransferase-class-V-fold-PLP-dependent-enzyme-[Intestinibaculum-porci]	2487118
WP_029072274.1	cysteine-desulfurase	GCF_000621925.1	383	Bacteria>Terrabacteria_group>Firmicutes>Erysipelotrichia>Erysipelotrichales	Kandleria_vitulina	cysteine-desulfurase-[Kandleria-vitulina]	1630
MBQ8068455.1	cysteine-desulfurase	GCA_017469885.1	376	Bacteria>Terrabacteria_group>Firmicutes>Erysipelotrichia>Erysipelotrichales	Solobacterium_sp.	aminotransferase-class-V-fold-PLP-dependent-enzyme-[Solobacterium-sp.]	2060878
WP_154504225.1	cysteine-desulfurase	GCF_009696165.1	382	Bacteria>Terrabacteria_group>Firmicutes>Erysipelotrichia>Erysipelotrichales	Stecheria_intestinalis	cysteine-desulfurase-[Stecheria-intestinalis]	2606630
WP_195927051.1	cysteine-desulfurase	GCF_015667815.1	374	Bacteria>Terrabacteria_group>Firmicutes>Erysipelotrichia>Erysipelotrichales	Turicibacter_sanguinis	cysteine-desulfurase-[Turicibacter-sanguinis]	154288
WP_132017601.1	cysteine-desulfurase	GCF_004340685.1	382	Bacteria>Terrabacteria_group>Firmicutes>Firmicutes_sensu_stricto_incertae_sedis>Hydrogenispora	Hydrogenispora_ethanolica	aminotransferase-class-V-fold-PLP-dependent-enzyme-[Hydrogenispora-ethanolica]	1082276
WP_117454146.1	cysteine-desulfurase	GCF_014337235.1	382	Bacteria>Terrabacteria_group	Firmicutes	MULTISPECIES:-cysteine-desulfurase-[Firmicutes]	1239
WP_074739729.1	cysteine-desulfurase	GCF_900115425.1	381	Bacteria>Terrabacteria_group>Firmicutes>Negativicutes>Acidaminococcales	Acidaminococcus_fermentans	aminotransferase-class-V-fold-PLP-dependent-enzyme-[Acidaminococcus-fermentans]	905
WP_210684518.1	cysteine-desulfurase	GCF_017968825.1	381	Bacteria>Terrabacteria_group>Firmicutes>Negativicutes>Acidaminococcales	Phascolarctobacterium_sp._Marseille-Q4147	aminotransferase-class-V-fold-PLP-dependent-enzyme-[Phascolarctobacterium-sp.-Marseille-Q4147]	2823317
MBS5426926.1	cysteine-desulfurase	GCA_018368575.1	381	Bacteria>Terrabacteria_group>Firmicutes>Negativicutes>Acidaminococcales	Phascolarctobacterium_succinatutens	aminotransferase-class-V-fold-PLP-dependent-enzyme-[Phascolarctobacterium-succinatutens]	626940
OLA36686.1	cysteine-desulfurase	GCA_001916865.1	381	Bacteria>Terrabacteria_group>Firmicutes>Negativicutes>Acidaminococcales	Phascolarctobacterium_succinatutens	cysteine-desulfurase-[Phascolarctobacterium-succinatutens]	626940
WP_080325478.1	cysteine-desulfurase	GCF_900141865.1	379	Bacteria>Terrabacteria_group>Firmicutes>Negativicutes>Selenomonadales	Anaerovibrio_lipolyticus	aminotransferase-class-V-fold-PLP-dependent-enzyme-[Anaerovibrio-lipolyticus]	82374
WP_154405929.1	cysteine-desulfurase	GCF_009695585.1	381	Bacteria>Terrabacteria_group>Firmicutes>Negativicutes>Selenomonadales	Anaerovibrio_slackiae	aminotransferase-class-V-fold-PLP-dependent-enzyme-[Anaerovibrio-slackiae]	2652309
MBR2142529.1	cysteine-desulfurase	GCA_017475925.1	379	Bacteria>Terrabacteria_group>Firmicutes>Negativicutes>Selenomonadales	Anaerovibrio_sp.	aminotransferase-class-V-fold-PLP-dependent-enzyme-[Anaerovibrio-sp.]	1872532
WP_008539335.1	cysteine-desulfurase	GCF_010669225.1	381	Bacteria>Terrabacteria_group>Firmicutes>Negativicutes>Selenomonadales	Megamonas_funiformis	aminotransferase-class-V-fold-PLP-dependent-enzyme-[Megamonas-funiformis]	437897
WP_204921097.1	cysteine-desulfurase	GCF_016900185.1	381	Bacteria>Terrabacteria_group>Firmicutes>Negativicutes>Selenomonadales	Megamonas_rupellensis	aminotransferase-class-V-fold-PLP-dependent-enzyme-[Megamonas-rupellensis]	491921
MCB5725860.1	cysteine-desulfurase	GCA_020539255.1	381	Bacteria>Terrabacteria_group>Firmicutes>Negativicutes>Selenomonadales	Mitsuokella_jalaludinii	aminotransferase-class-V-fold-PLP-dependent-enzyme-[Mitsuokella-jalaludinii]	187979
WP_226868701.1	cysteine-desulfurase	GCF_020539255.1	381	Bacteria>Terrabacteria_group>Firmicutes>Negativicutes>Selenomonadales	Mitsuokella_jalaludinii	aminotransferase-class-V-fold-PLP-dependent-enzyme-[Mitsuokella-jalaludinii]	187979
WP_040635239.1	cysteine-desulfurase	GCF_902373875.1	381	Bacteria>Terrabacteria_group>Firmicutes>Negativicutes>Selenomonadales	Mitsuokella_multacida	aminotransferase-class-V-fold-PLP-dependent-enzyme-[Mitsuokella-multacida]	52226
WP_235269433.1	cysteine-desulfurase	GCF_021531445.1	381	Bacteria>Terrabacteria_group>Firmicutes>Negativicutes>Selenomonadales	Mitsuokella_multacida	aminotransferase-class-V-fold-PLP-dependent-enzyme-[Mitsuokella-multacida]	52226
EEX70103.1	cysteine-desulfurase	GCA_000155955.1	361	Bacteria>Terrabacteria_group>Firmicutes>Negativicutes>Selenomonadales	Mitsuokella_multacida_DSM_20544	cysteine-desulfurase-family-protein-[Mitsuokella-multacida-DSM-20544]	500635
WP_196598968.1	cysteine-desulfurase	GCF_015732285.1	384	Bacteria>Terrabacteria_group>Firmicutes>Negativicutes>Selenomonadales	Pectinatus_frisingensis	cysteine-desulfurase-[Pectinatus-frisingensis]	865
MBR1579024.1	cysteine-desulfurase	GCA_017477125.1	381	Bacteria>Terrabacteria_group>Firmicutes>Negativicutes>Selenomonadales	Selenomonadaceae_bacterium	aminotransferase-class-V-fold-PLP-dependent-enzyme-[Selenomonadaceae-bacterium]	2652289
MBR2520115.1	cysteine-desulfurase	GCA_017515635.1	381	Bacteria>Terrabacteria_group>Firmicutes>Negativicutes>Selenomonadales	Selenomonadaceae_bacterium	aminotransferase-class-V-fold-PLP-dependent-enzyme-[Selenomonadaceae-bacterium]	2652289
MBR6267407.1	cysteine-desulfurase	GCA_017539485.1	382	Bacteria>Terrabacteria_group>Firmicutes>Negativicutes>Selenomonadales	Selenomonadaceae_bacterium	aminotransferase-class-V-fold-PLP-dependent-enzyme-[Selenomonadaceae-bacterium]	2652289
WP_154620065.1	cysteine-desulfurase	GCF_009697385.1	381	Bacteria>Terrabacteria_group>Firmicutes>Negativicutes>Selenomonadales	Selenomonas_montiformis	aminotransferase-class-V-fold-PLP-dependent-enzyme-[Selenomonas-montiformis]	2652285
WP_074814340.1	cysteine-desulfurase	GCF_900111915.1	378	Bacteria>Terrabacteria_group>Firmicutes>Negativicutes>Selenomonadales	Selenomonas_ruminantium	aminotransferase-class-V-fold-PLP-dependent-enzyme-[Selenomonas-ruminantium]	971
SDG27529.1	cysteine-desulfurase	GCA_900100835.1	378	Bacteria>Terrabacteria_group>Firmicutes>Negativicutes>Selenomonadales	Selenomonas_ruminantium	cysteine-desulfurase-family-protein-[Selenomonas-ruminantium]	971
WP_009645306.1	cysteine-desulfurase	GCF_000292955.1	382	Bacteria>Terrabacteria_group>Firmicutes>Negativicutes>Selenomonadales	Selenomonas_sp._CM52	cysteine-desulfurase-[Selenomonas-sp.-CM52]	936381
EJO23459.1	cysteine-desulfurase	GCA_000286455.1	92	Bacteria>Terrabacteria_group>Firmicutes>Negativicutes>Selenomonadales	Selenomonas_sp._FOBRC6	aminotransferase,-class-V-domain-protein-[Selenomonas-sp.-FOBRC6]	936572
EJO23477.1	cysteine-desulfurase	GCA_000286455.1	180	Bacteria>Terrabacteria_group>Firmicutes>Negativicutes>Selenomonadales	Selenomonas_sp._FOBRC6	aminotransferase,-class-V-domain-protein-[Selenomonas-sp.-FOBRC6]	936572
WP_149170289.1	cysteine-desulfurase	GCF_008271295.1	378	Bacteria>Terrabacteria_group>Firmicutes>Negativicutes>Selenomonadales	Selenomonas_sp._mPRGC5	aminotransferase-class-V-fold-PLP-dependent-enzyme-[Selenomonas-sp.-mPRGC5]	2593411
WP_066844060.1	cysteine-desulfurase	GCF_001683335.1	383	Bacteria>Terrabacteria_group>Firmicutes>Negativicutes>Selenomonadales	Selenomonas_sp._oral_taxon_126	aminotransferase-class-V-fold-PLP-dependent-enzyme-[Selenomonas-sp.-oral-taxon-126]	712528
GBG56076.1	cysteine-desulfurase	GCA_005381385.1	374	Bacteria>Terrabacteria_group>Firmicutes>Negativicutes>Selenomonadales	Sporomusaceae_bacterium_FL31	putative-cysteine-desulfurase-IscS-1-[Sporomusaceae-bacterium-FL31]	2163554
WP_144351259.1	cysteine-desulfurase	GCF_007641255.1	400	Bacteria>Terrabacteria_group>Firmicutes>Negativicutes>Selenomonadales	Sporomusa_termitida	cysteine-desulfurase-[Sporomusa-termitida]	2377
MBS5035650.1	cysteine-desulfurase	GCA_018365125.1	378	Bacteria>Terrabacteria_group>Firmicutes>Negativicutes>Veillonellales	Dialister_sp.	aminotransferase-class-V-fold-PLP-dependent-enzyme-[Dialister-sp.]	1955814
CDA47868.1	cysteine-desulfurase	GCA_000434475.1	378	Bacteria>Terrabacteria_group>Firmicutes>Negativicutes>Veillonellales	Dialister_sp._CAG:486	cysteine-desulfurase-[Dialister-sp.-CAG:486]	1262870
WP_216554424.1	cysteine-desulfurase	GCF_018917285.1	393	Bacteria>Terrabacteria_group>Firmicutes>Tissierellia>Tissierellales	Tissierella_carlieri	aminotransferase-class-V-fold-PLP-dependent-enzyme-[Tissierella-carlieri]	689904
MBU5310594.1	cysteine-desulfurase	GCA_018917285.1	393	Bacteria>Terrabacteria_group>Firmicutes>Tissierellia>Tissierellales	Tissierella_carlieri	cysteine-desulfurase-[Tissierella-carlieri]	689904
MBV1756491.1	cysteine-desulfurase	GCA_019084435.1	377	Bacteria>Terrabacteria_group>Firmicutes>Tissierellia>Tissierellia_incertae_sedis	Dethiosulfatibacter_sp.	aminotransferase-class-V-fold-PLP-dependent-enzyme-[Dethiosulfatibacter-sp.]	2812851
WP_209510989.1	cysteine-desulfurase	-	384	Bacteria>Terrabacteria_group>Firmicutes>Tissierellia>Tissierellia_incertae_sedis	Sedimentibacter_acidaminivorans	aminotransferase-class-V-fold-PLP-dependent-enzyme-[Sedimentibacter-acidaminivorans]	913099
NLK65424.1	cysteine-desulfurase	GCA_012519555.1	377	Bacteria>Terrabacteria_group>Firmicutes>Tissierellia>unclassified_Tissierellia	Tissierellia_bacterium	aminotransferase-class-V-fold-PLP-dependent-enzyme-[Tissierellia-bacterium]	2049431
NLV89558.1	cysteine-desulfurase	GCA_012728125.1	381	Bacteria>Terrabacteria_group>Firmicutes>Tissierellia>unclassified_Tissierellia	Tissierellia_bacterium	aminotransferase-class-V-fold-PLP-dependent-enzyme-[Tissierellia-bacterium]	2049431
MBO8461166.1	cysteine-desulfurase	GCA_017694735.1	381	Bacteria>Terrabacteria_group>Firmicutes>unclassified_Firmicutes_sensu_stricto	Firmicutes_bacterium	aminotransferase-class-V-fold-PLP-dependent-enzyme-[Firmicutes-bacterium]	1879010
MTI69249.1	cysteine-desulfurase	GCA_009711785.1	385	Bacteria>Terrabacteria_group>Firmicutes>unclassified_Firmicutes_sensu_stricto	Firmicutes_bacterium	aminotransferase-class-V-fold-PLP-dependent-enzyme-[Firmicutes-bacterium]	1879010
MBP2656988.1	cysteine-desulfurase	GCA_017889665.1	382	Bacteria>Terrabacteria_group>Firmicutes>unclassified_Firmicutes_sensu_stricto	Firmicutes_bacterium	cysteine-desulfurase-DndA-[Firmicutes-bacterium]	1879010
NLM39740.1	cysteine-desulfurase	GCA_012521295.1	379	Bacteria>Terrabacteria_group>Firmicutes>unclassified_Firmicutes_sensu_stricto	Firmicutes_bacterium	cysteine-desulfurase-[Firmicutes-bacterium]	1879010
NLL01683.1	cysteine-desulfurase	GCA_012520055.1	370	Bacteria>Terrabacteria_group>Tenericutes>Mollicutes>unclassified_Mollicutes	Mollicutes_bacterium	cysteine-desulfurase-[Mollicutes-bacterium]	37628
MBZ4650441.1	cysteine-desulfurase	GCA_020054775.1	389	Bacteria>Thermotogae>Thermotogae>Thermotogales>Fervidobacteriaceae	Thermosipho_sp._(in:_Bacteria)	aminotransferase-class-[Thermosipho-sp.-(in:-Bacteria)]	1968895
RKJ47905.1	cysteine-desulfurase	GCA_003612585.1	383	Bacteria>unclassified_Bacteria	bacterium_1XD42-54	aminotransferase-class-V-fold-PLP-dependent-enzyme-[bacterium-1XD42-54]	2320110

Accession	Annotation	GCA	Length	Phyletic.spread	Strain.name	Defline	Species.Taxid
NBJ82822.1	cysteine-desulfurase	GCA_009917545.1	379	Bacteria>unclassified_Bacteria	bacterium_1XD42-76	aminotransferase-class-V-fold-PLP-dependent-enzyme-[bacterium-1XD42-76]	2320881
RKI76493.1	cysteine-desulfurase	GCA_003611805.1	381	Bacteria>unclassified_Bacteria	bacterium_1xD42-87	aminotransferase-class-V-fold-PLP-dependent-enzyme-[bacterium-1xD42-87]	2320113
MCB6989722.1	cysteine-desulfurase	GCA_020558425.1	401	Bacteria>unclassified_Bacteria	bacterium_210820-DFI.6.38	cysteine-desulfurase-[bacterium-210820-DFI.6.38]	2883293
MBI1293686.1	cysteine-desulfurase	GCA_016124945.1	388	Bacteria>unclassified_Bacteria	bacterium	aminotransferase-class-V-fold-PLP-dependent-enzyme-[bacterium]	1869227
MBI1294998.1	cysteine-desulfurase	GCA_016124945.1	392	Bacteria>unclassified_Bacteria	bacterium	aminotransferase-class-V-fold-PLP-dependent-enzyme-[bacterium]	1869227
MBO4516435.1	cysteine-desulfurase	GCA_017616075.1	382	Bacteria>unclassified_Bacteria	bacterium	aminotransferase-class-V-fold-PLP-dependent-enzyme-[bacterium]	1869227
NBP15849.1	cysteine-desulfurase	GCA_009918665.1	377	Bacteria>unclassified_Bacteria	bacterium	cysteine-desulfurase-[bacterium]	1869227
MBS7200429.1	cysteine-desulfurase	GCA_018382885.1	378	Bacteria>unclassified_Bacteria	butyrate-producing_bacterium	aminotransferase-class-V-fold-PLP-dependent-enzyme-[butyrate-producing-bacterium]	2811711
QSZ28062.1	cysteine-desulfurase	GCA_017357865.1	385	Bacteria>unclassified_Bacteria	thermophilic_bacterium_3443-3Ac	aminotransferase-class-V-fold-PLP-dependent-enzyme-[thermophilic-bacterium-3443-3Ac]	2755338
NBD15664.1	cysteine-desulfurase	GCA_009909085.1	126	-	-	aminotransferase-class-V-fold-PLP-dependent-enzyme]	2461416
MBE0254607.1	cysteine-desulfurase	GCA_014856545.1	372	-	-	aminotransferase-class-V-fold-PLP-dependent-enzyme-[Bacillus-sp.-SBJS01]	2596923
NLL62774.1	cysteine-desulfurase	GCA_012520505.1	381	-	-	aminotransferase-class-V-fold-PLP-dependent-enzyme-[Oscillospiraceae-bacterium]	1898205
WP_004074588.1	cysteine-desulfurase	GCF_010208005.1	381	-	-	aminotransferase-class-V-fold-PLP-dependent-enzyme-[Schaedlerella-arabinosiphila]	-
MSV24292.1	cysteine-desulfurase	GCA_009697385.1	381	-	-	aminotransferase-class-V-fold-PLP-dependent-enzyme-[Selenomonas-montiformis]	2605788
HHK96746.1	cysteine-desulfurase	-	384	-	-	aminotransferase-class-V-fold-PLP-dependent-enzyme-[Verrucomicrobia-bacterium]	-
MBR7553626.1	cysteine-desulfurase	GCA_018138385.1	375	-	-	cysteine-desulfurase-[Allobacillus-sp.-SKP8-2]	1955273
HGY50211.1	cysteine-desulfurase	-	384	-	-	cysteine-desulfurase-[Anaerolineae-bacterium]	-
MBM7629500.1	cysteine-desulfurase	-	385	-	-	cysteine-desulfurase-[Cohnella-boryungensis]	-
WP_021464718.1	cysteine-desulfurase	GCF_024655985.1	374	-	-	cysteine-desulfurase-DndA-[Aeromonas-dhakensis]	-
HGW42316.1	cysteine-desulfurase	-	392	-	-	cysteine-desulfurase-DndA-[Hyphomicrobiales-bacterium]	-
HGT45527.1	cysteine-desulfurase	-	379	-	-	cysteine-desulfurase-DndA-[Verrucomicrobia-subdivision-3-bacterium]	-
EGR5369539.1	cysteine-desulfurase	-	374	-	-	cysteine-desulfurase-DndA-[Vibrio-parahaemolyticus]	-
HHS60208.1	cysteine-desulfurase	-	390	-	-	cysteine-desulfurase-[Ignavibacteria-bacterium]	-
HHS61813.1	cysteine-desulfurase	-	358	-	-	cysteine-desulfurase-[Ignavibacteria-bacterium]	-
MBC8566214.1	cysteine-desulfurase	-	397	-	-	cysteine-desulfurase-[Lachnospiraceae-bacterium-H15]	-
HBW73401.1	cysteine-desulfurase	GCA_003514385.1	384	-	-	cysteine-desulfurase-[Oscillospiraceae-bacterium]	1898205
MBB3253893.1	cysteine-desulfurase	-	382	-	-	cysteine-desulfurase-[Rhizobium-sp.-BK007]	-
MBB5946818.1	cysteine-desulfurase	-	378	-	-	cysteine-desulfurase-[Sphingobium-sp.-B1D3D]	-
DAP91366.1	cysteine-desulfurase	-	379	Viruses>Monodnaviria>Loebvirae>Hofneiviricota>Faserviricetes	Inoviridae_sp.	TPA:-MAG-TPA:-cysteine-desulfurase-[Inoviridae-sp.]	2219103

Accession	Annotation	GCA	Length	Phyletic.spread	Strain.name	Defline	Species.Taxid
HII62662.1	DndB-ParB-nuclease	GCA_013330815.1	233	Archaea>Candidatus_Thermoplasmatota>Candidatus_Poseidoniia>Candidatus_Poseidoniales>Candidatus_Poseidoniaceae	Candidatus_Poseidoniaceae_archaeon	DGQHR-domain-containing-protein-[Candidatus-Poseidoniaceae-archaeon]	2666346
HII62667.1	DndB-ParB-nuclease	GCA_013330815.1	386	Archaea>Candidatus_Thermoplasmatota>Candidatus_Poseidoniia>Candidatus_Poseidoniales>Candidatus_Poseidoniaceae	Candidatus_Poseidoniaceae_archaeon	DGQHR-domain-containing-protein-[Candidatus-Poseidoniaceae-archaeon]	2666346
DAC17230.1	DndB-ParB-nuclease	GCA_003672705.1	386	Archaea>Candidatus_Thermoplasmatota>Candidatus_Poseidoniia>Candidatus_Poseidoniales>unclassified_Candidatus_Poseidoniales	Candidatus_Poseidoniales_archaeon	TPA:-DGQHR-domain-containing-protein-[Candidatus-Poseidoniales-archaeon]	2163009
DAC17225.1	DndB-ParB-nuclease	GCA_003672705.1	233	Archaea>Candidatus_Thermoplasmatota>Candidatus_Poseidoniia>Candidatus_Poseidoniales>unclassified_Candidatus_Poseidoniales	Candidatus_Poseidoniales_archaeon	TPA:-DGQHR-domain-containing-protein,-partial-[Candidatus-Poseidoniales-archaeon]	2163009
MBT3813916.1	DndB-ParB-nuclease	GCA_018654495.1	369	Archaea>DPANN_group>Candidatus_Woesearchaeota	Candidatus_Woesearchaeota_archaeon	DNA-sulfur-modification-protein-DndB-[Candidatus-Woesearchaeota-archaeon]	2026803
MBT4110876.1	DndB-ParB-nuclease	GCA_018650945.1	369	Archaea>DPANN_group>Candidatus_Woesearchaeota	Candidatus_Woesearchaeota_archaeon	DNA-sulfur-modification-protein-DndB-[Candidatus-Woesearchaeota-archaeon]	2026803
MBT4731422.1	DndB-ParB-nuclease	GCA_018658425.1	286	Archaea>DPANN_group>Candidatus_Woesearchaeota	Candidatus_Woesearchaeota_archaeon	DNA-sulfur-modification-protein-DndB-[Candidatus-Woesearchaeota-archaeon]	2026803
MBT7556384.1	DndB-ParB-nuclease	GCA_018674815.1	357	Archaea>DPANN_group>Candidatus_Woesearchaeota	Candidatus_Woesearchaeota_archaeon	DNA-sulfur-modification-protein-DndB-[Candidatus-Woesearchaeota-archaeon]	2026803
RJQ15774.1	DndB-ParB-nuclease	GCA_003599145.1	371	Archaea>DPANN_group>Candidatus_Woesearchaeota	Candidatus_Woesearchaeota_archaeon	DNA-sulfur-modification-protein-DndB-[Candidatus-Woesearchaeota-archaeon]	2026803
WP_214166861.1	DndB-ParB-nuclease	GCF_018495055.1	431	Archaea>Euryarchaeota>Stenosarchaea_group>Methanomicrobia>Methanomicrobiales	Methanoculleus_bourgensis	DGQHR-domain-containing-protein-[Methanoculleus-bourgensis]	83986
PKL63558.1	DndB-ParB-nuclease	GCA_002839645.1	383	Archaea>Euryarchaeota>Stenosarchaea_group>Methanomicrobia>Methanomicrobiales	Methanomicrobiales_archaeon_HGW-Methanomicrobiales-2	DNA-sulfur-modification-protein-DndB-[Methanomicrobiales-archaeon-HGW-Methanomicrobiales-2]	2013818
WP_011991368.1	DndB-ParB-nuclease	GCF_000017625.1	380	Archaea>Euryarchaeota>Stenosarchaea_group>Methanomicrobia>Methanomicrobiales	Methanoregula_boonei	DNA-sulfur-modification-protein-DndB-[Methanoregula-boonei]	358766
WP_048110974.1	DndB-ParB-nuclease	GCF_000327485.1	442	Archaea>Euryarchaeota>Stenosarchaea_group>Methanomicrobia>Methanomicrobiales	Methanoregula_formicica	DGQHR-domain-containing-protein-[Methanoregula-formicica]	882104
AGB03377.1	DndB-ParB-nuclease	GCA_000327485.1	480	Archaea>Euryarchaeota>Stenosarchaea_group>Methanomicrobia>Methanomicrobiales	Methanoregula_formicica_SMSP	DGQHR-domain-containing-protein-[Methanoregula-formicica-SMSP]	593750
OPX62558.1	DndB-ParB-nuclease	GCA_002067035.1	380	Archaea>Euryarchaeota>Stenosarchaea_group>Methanomicrobia>Methanomicrobiales	Methanoregula_sp._PtaB.Bin085	hypothetical-protein-A4E33_02307-[Methanoregula-sp.-PtaB.Bin085]	1811680
WP_012618017.1	DndB-ParB-nuclease	GCF_000021965.1	380	Archaea>Euryarchaeota>Stenosarchaea_group>Methanomicrobia>Methanomicrobiales	Methanosphaerula_palustris	DNA-sulfur-modification-protein-DndB-[Methanosphaerula-palustris]	475088
ACL16698.1	DndB-ParB-nuclease	GCA_000021965.1	380	Archaea>Euryarchaeota>Stenosarchaea_group>Methanomicrobia>Methanomicrobiales	Methanosphaerula_palustris_E1-9c	DNA-sulfur-modification-protein-DndB-[Methanosphaerula-palustris-E1-9c]	521011
NOR46498.1	DndB-ParB-nuclease	GCA_013139685.1	376	Archaea>Euryarchaeota>Stenosarchaea_group>Methanomicrobia>Methanosarcinales	Methanosarcinaceae_archaeon	DNA-sulfur-modification-protein-DndB-[Methanosarcinaceae-archaeon]	1945595
MCD4798385.1	DndB-ParB-nuclease	GCA_021108115.1	366	Archaea>Euryarchaeota>Stenosarchaea_group>Methanomicrobia>Methanosarcinales	Methanosarcinales_archaeon	DNA-sulfur-modification-protein-DndB-[Methanosarcinales-archaeon]	2250255
RLG24035.1	DndB-ParB-nuclease	GCA_003661045.1	370	Archaea>Euryarchaeota>Stenosarchaea_group>Methanomicrobia>Methanosarcinales	Methanosarcinales_archaeon	DNA-sulfur-modification-protein-DndB-[Methanosarcinales-archaeon]	2250255
WP_200895853.1	DndB-ParB-nuclease	GCF_000979335.1	374	Archaea>Euryarchaeota>Stenosarchaea_group>Methanomicrobia>Methanosarcinales	Methanosarcina_mazei	DNA-sulfur-modification-protein-DndB-[Methanosarcina-mazei]	2209
MBT0159054.1	DndB-ParB-nuclease	GCA_018475625.1	453	Archaea>TACK_group>Candidatus_Bathyarchaeota>unclassified_Candidatus_Bathyarchaeota	Candidatus_Bathyarchaeota_archaeon_A05DMB-2	DGQHR-domain-containing-protein-[Candidatus-Bathyarchaeota-archaeon-A05DMB-2]	2709783
AIF05633.1	DndB-ParB-nuclease	-	367	Archaea>TACK_group>Thaumarchaeota>environmental_samples	uncultured_marine_thaumarchaeote_KM3_186_C08	DNA-sulfur-modification-protein-DndB-[uncultured-marine-thaumarchaeote-KM3_186_C08]	1456070
ARS64883.1	DndB-ParB-nuclease	GCA_002156965.1	367	Archaea>TACK_group>Thaumarchaeota>Nitrosopumilales>Nitrosopumilaceae	Candidatus_Nitrosomarinus_catalina	DNA-sulfur-modification-protein-DndB-[Candidatus-Nitrosomarinus-catalina]	1898749
WP_086907927.1	DndB-ParB-nuclease	GCF_002156965.1	367	Archaea>TACK_group>Thaumarchaeota>Nitrosopumilales>Nitrosopumilaceae	Candidatus_Nitrosomarinus_catalina	DNA-sulfur-modification-protein-DndB-[Candidatus-Nitrosomarinus-catalina]	1898749
WP_048097632.1	DndB-ParB-nuclease	GCF_000242875.2	367	Archaea>TACK_group>Thaumarchaeota>Nitrosopumilales>Nitrosopumilaceae	Candidatus_Nitrosopumilus_salaria	DNA-sulfur-modification-protein-DndB-[Candidatus-Nitrosopumilus-salaria]	1170320
PHY08246.1	DndB-ParB-nuclease	GCA_002737445.1	361	Archaea>TACK_group>Thaumarchaeota>Nitrosopumilales>Nitrosopumilaceae	Nitrosarchaeum_sp.	DNA-sulfur-modification-protein-DndB-[Nitrosarchaeum-sp.]	2026886
PHY08230.1	DndB-ParB-nuclease	GCA_002737445.1	171	Archaea>TACK_group>Thaumarchaeota>Nitrosopumilales>Nitrosopumilaceae	Nitrosarchaeum_sp.	hypothetical-protein-CK527_07160-[Nitrosarchaeum-sp.]	2026886
MCE2508327.1	DndB-ParB-nuclease	GCA_021296165.1	376	Archaea>TACK_group>Thaumarchaeota>Nitrosopumilales>Nitrosopumilaceae	Nitrosopumilaceae_archaeon	DNA-sulfur-modification-protein-DndB-[Nitrosopumilaceae-archaeon]	2202732
KAF6243634.1	DndB-ParB-nuclease	GCA_014078545.1	348	Archaea>TACK_group>Thaumarchaeota>Nitrosopumilales>Nitrosopumilaceae	Nitrosopumilus_sp._b1	DNA-sulfur-modification-protein-DndB-[Nitrosopumilus-sp.-b1]	2109907
WP_182133300.1	DndB-ParB-nuclease	-	348	Archaea>TACK_group>Thaumarchaeota>Nitrosopumilales>Nitrosopumilaceae	Nitrosopumilus_sp._b1	DNA-sulfur-modification-protein-DndB-[Nitrosopumilus-sp.-b1]	2109907
MBC8501891.1	DndB-ParB-nuclease	GCA_014384445.1	367	Archaea>TACK_group>Thaumarchaeota>Nitrosopumilales>Nitrosopumilaceae	Nitrosopumilus_sp.	DNA-sulfur-modification-protein-DndB-[Nitrosopumilus-sp.]	2024843
MBC8502146.1	DndB-ParB-nuclease	GCA_014384445.1	343	Archaea>TACK_group>Thaumarchaeota>Nitrosopumilales>Nitrosopumilaceae	Nitrosopumilus_sp.	DNA-sulfur-modification-protein-DndB-[Nitrosopumilus-sp.]	2024843
MBL7001906.1	DndB-ParB-nuclease	GCA_016784625.1	364	Archaea>TACK_group>Thaumarchaeota>Nitrosopumilales>Nitrosopumilaceae	Nitrosopumilus_sp.	DNA-sulfur-modification-protein-DndB-[Nitrosopumilus-sp.]	2024843
NRA06467.1	DndB-ParB-nuclease	GCA_013215285.1	353	Archaea>TACK_group>Thaumarchaeota>Nitrosopumilales>Nitrosopumilaceae	Nitrosopumilus_sp.	DNA-sulfur-modification-protein-DndB-[Nitrosopumilus-sp.]	2024843
HIO32165.1	DndB-ParB-nuclease	GCA_015663895.1	76	Archaea>TACK_group>Thaumarchaeota>Thaumarchaeota_incertae_sedis>Candidatus_Nitrosopelagicus	Candidatus_Nitrosopelagicus_sp.	DNA-sulfur-modification-protein-DndB-[Candidatus-Nitrosopelagicus-sp.]	2026808
MBC8250874.1	DndB-ParB-nuclease	GCA_014381285.1	366	Archaea>TACK_group>Thaumarchaeota>Thaumarchaeota_incertae_sedis>Candidatus_Nitrosopelagicus	Candidatus_Nitrosopelagicus_sp.	DNA-sulfur-modification-protein-DndB-[Candidatus-Nitrosopelagicus-sp.]	2026808
MBT3761277.1	DndB-ParB-nuclease	GCA_018654855.1	366	Archaea>TACK_group>Thaumarchaeota>Thaumarchaeota_incertae_sedis>Candidatus_Nitrosopelagicus	Candidatus_Nitrosopelagicus_sp.	DNA-sulfur-modification-protein-DndB-[Candidatus-Nitrosopelagicus-sp.]	2026808
MBT4326513.1	DndB-ParB-nuclease	GCA_018652445.1	367	Archaea>TACK_group>Thaumarchaeota>Thaumarchaeota_incertae_sedis>Candidatus_Nitrosopelagicus	Candidatus_Nitrosopelagicus_sp.	DNA-sulfur-modification-protein-DndB-[Candidatus-Nitrosopelagicus-sp.]	2026808
MBI1658937.1	DndB-ParB-nuclease	GCA_016126025.1	350	Archaea>TACK_group>Thaumarchaeota>unclassified_Thaumarchaeota	Thaumarchaeota_archaeon	DNA-sulfur-modification-protein-DndB-[Thaumarchaeota-archaeon]	2026795
MBT4176039.1	DndB-ParB-nuclease	GCA_018651685.1	363	Archaea>TACK_group>Thaumarchaeota>unclassified_Thaumarchaeota	Thaumarchaeota_archaeon	DNA-sulfur-modification-protein-DndB-[Thaumarchaeota-archaeon]	2026795
MBT6646242.1	DndB-ParB-nuclease	GCA_018697615.1	353	Archaea>TACK_group>Thaumarchaeota>unclassified_Thaumarchaeota	Thaumarchaeota_archaeon	DNA-sulfur-modification-protein-DndB-[Thaumarchaeota-archaeon]	2026795
RZD40205.1	DndB-ParB-nuclease	GCA_004195415.1	368	Archaea>TACK_group>Thaumarchaeota>unclassified_Thaumarchaeota	Thaumarchaeota_archaeon	DNA-sulfur-modification-protein-DndB-[Thaumarchaeota-archaeon]	2026795
RZD43271.1	DndB-ParB-nuclease	GCA_004195415.1	363	Archaea>TACK_group>Thaumarchaeota>unclassified_Thaumarchaeota	Thaumarchaeota_archaeon	DNA-sulfur-modification-protein-DndB-[Thaumarchaeota-archaeon]	2026795
MBS1260673.1	DndB-ParB-nuclease	GCA_018263495.1	368	Archaea>TACK_group>Thaumarchaeota>unclassified_Thaumarchaeota	Thaumarchaeota_archaeon	hypothetical-protein-[Thaumarchaeota-archaeon]	2026795
RNJ72391.1	DndB-ParB-nuclease	GCA_003724275.1	343	Archaea>TACK_group>Thaumarchaeota>unclassified_Thaumarchaeota	Thaumarchaeota_archaeon_S13	DNA-sulfur-modification-protein-DndB-[Thaumarchaeota-archaeon-S13]	2478557
RNJ74508.1	DndB-ParB-nuclease	GCA_003724175.1	361	Archaea>TACK_group>Thaumarchaeota>unclassified_Thaumarchaeota	Thaumarchaeota_archaeon_S15	DNA-sulfur-modification-protein-DndB-[Thaumarchaeota-archaeon-S15]	2478559
MBT4648404.1	DndB-ParB-nuclease	GCA_018657865.1	61	Archaea>unclassified_Archaea	archaeon	DNA-sulfur-modification-protein-DndB-[archaeon]	1906665
MBT4352523.1	DndB-ParB-nuclease	GCA_018653265.1	359	Archaea>unclassified_Archaea	archaeon	DNA-sulfur-modification-protein-DndB-[archaeon]	1906665
ACO34108.1	DndB-ParB-nuclease	GCA_000022565.1	359	Bacteria>Acidobacteria>Acidobacteriia>Acidobacteriales>Acidobacteriaceae	Acidobacterium_capsulatum_ATCC_51196	DNA-sulfur-modification-protein-DndB-[Acidobacterium-capsulatum-ATCC-51196]	240015
ACO33955.1	DndB-ParB-nuclease	GCA_000022565.1	373	Bacteria>Acidobacteria>Acidobacteriia>Acidobacteriales>Acidobacteriaceae	Acidobacterium_capsulatum_ATCC_51196	hypothetical-protein-ACP_3191-[Acidobacterium-capsulatum-ATCC-51196]	240015
WP_015898234.1	DndB-ParB-nuclease	GCF_000022565.1	373	Bacteria>Acidobacteria>Acidobacteriia>Acidobacteriales>Acidobacteriaceae	Acidobacterium_capsulatum	DGQHR-domain-containing-protein-[Acidobacterium-capsulatum]	33075
WP_015898229.1	DndB-ParB-nuclease	GCF_000022565.1	359	Bacteria>Acidobacteria>Acidobacteriia>Acidobacteriales>Acidobacteriaceae	Acidobacterium_capsulatum	DNA-sulfur-modification-protein-DndB-[Acidobacterium-capsulatum]	33075
MBI3649438.1	DndB-ParB-nuclease	GCA_016201275.1	409	Bacteria>Acidobacteria>unclassified_Acidobacteria	Acidobacteria_bacterium	DGQHR-domain-containing-protein-[Acidobacteria-bacterium]	1978231
MXY25147.1	DndB-ParB-nuclease	GCA_009838525.1	420	Bacteria>Acidobacteria>unclassified_Acidobacteria	Acidobacteria_bacterium	DGQHR-domain-containing-protein-[Acidobacteria-bacterium]	1978231
MYI75603.1	DndB-ParB-nuclease	GCA_009845485.1	215	Bacteria>Acidobacteria>unclassified_Acidobacteria	Acidobacteria_bacterium	DGQHR-domain-containing-protein-[Acidobacteria-bacterium]	1978231
MBK7932006.1	DndB-ParB-nuclease	GCA_016713675.1	365	Bacteria>Acidobacteria>unclassified_Acidobacteria	Acidobacteria_bacterium	DNA-sulfur-modification-protein-DndB-[Acidobacteria-bacterium]	1978231
MBW4039112.1	DndB-ParB-nuclease	GCA_019351975.1	363	Bacteria>Acidobacteria>unclassified_Acidobacteria	Acidobacteria_bacterium	DNA-sulfur-modification-protein-DndB-[Acidobacteria-bacterium]	1978231
PSH04043.1	DndB-ParB-nuclease	GCA_003010405.1	377	Bacteria>Acidobacteria>unclassified_Acidobacteria	Acidobacteria_bacterium	DNA-sulfur-modification-protein-DndB-[Acidobacteria-bacterium]	1978231
WP_200674546.1	DndB-ParB-nuclease	GCF_016617615.1	350	Bacteria>Aquificae>Aquificae>Aquificales>Hydrogenothermaceae	Persephonella_atlantica	DNA-sulfur-modification-protein-DndB-[Persephonella-atlantica]	2699429
TCK02901.1	DndB-ParB-nuclease	GCA_004339575.1	350	Bacteria>Aquificae>Aquificae>Desulfurobacteriales>Desulfurobacteriaceae	Phorcysia_thermohydrogeniphila	DNA-sulfur-modification-protein-DndB-[Phorcysia-thermohydrogeniphila]	936138
WP_207891612.1	DndB-ParB-nuclease	GCF_004339575.1	350	Bacteria>Aquificae>Aquificae>Desulfurobacteriales>Desulfurobacteriaceae	Phorcysia_thermohydrogeniphila	DNA-sulfur-modification-protein-DndB-[Phorcysia-thermohydrogeniphila]	936138
MBN2836541.1	DndB-ParB-nuclease	GCA_016938275.1	368	Bacteria>Bacteria_incertae_sedis>Bacteria_candidate_phyla>Candidatus_Delongbacteria	Candidatus_Delongbacteria_bacterium	DGQHR-domain-containing-protein-[Candidatus-Delongbacteria-bacterium]	2044594
MBN1969159.1	DndB-ParB-nuclease	GCA_016931755.1	366	Bacteria>Bacteria_incertae_sedis>Bacteria_candidate_phyla>Candidatus_Delongbacteria	Candidatus_Delongbacteria_bacterium	DNA-sulfur-modification-protein-DndB-[Candidatus-Delongbacteria-bacterium]	2044594
MBN2836536.1	DndB-ParB-nuclease	GCA_016938275.1	364	Bacteria>Bacteria_incertae_sedis>Bacteria_candidate_phyla>Candidatus_Delongbacteria	Candidatus_Delongbacteria_bacterium	DNA-sulfur-modification-protein-DndB-[Candidatus-Delongbacteria-bacterium]	2044594
PJB29750.1	DndB-ParB-nuclease	GCA_002790015.1	355	Bacteria>Bacteria_incertae_sedis>Bacteria_candidate_phyla>Candidatus_Desantisbacteria	Candidatus_Desantisbacteria_bacterium_CG_4_9_14_3_um_filter_40_11	hypothetical-protein-CO110_04125-[Candidatus-Desantisbacteria-bacterium-CG_4_9_14_3_um_filter_40_11]	1974546
MBI1925911.1	DndB-ParB-nuclease	GCA_016178185.1	602	Bacteria>Bacteria_incertae_sedis>Bacteria_candidate_phyla>Candidatus_Poribacteria>unclassified_Candidatus_Poribacteria	Candidatus_Poribacteria_bacterium	DGQHR-domain-containing-protein-[Candidatus-Poribacteria-bacterium]	2026781
RKU15337.1	DndB-ParB-nuclease	GCA_003635185.1	433	Bacteria>Bacteria_incertae_sedis>Bacteria_candidate_phyla>Candidatus_Poribacteria>unclassified_Candidatus_Poribacteria	Candidatus_Poribacteria_bacterium	hypothetical-protein-C6501_06850-[Candidatus-Poribacteria-bacterium]	2026781
MBI3825587.1	DndB-ParB-nuclease	GCA_016200085.1	316	Bacteria>Bacteria_incertae_sedis>Bacteria_candidate_phyla>Candidatus_Rokubacteria>unclassified_Candidatus_Rokubacteria	Candidatus_Rokubacteria_bacterium	DGQHR-domain-containing-protein-[Candidatus-Rokubacteria-bacterium]	2053607
MBI3825593.1	DndB-ParB-nuclease	GCA_016200085.1	372	Bacteria>Bacteria_incertae_sedis>Bacteria_candidate_phyla>Candidatus_Rokubacteria>unclassified_Candidatus_Rokubacteria	Candidatus_Rokubacteria_bacterium	DNA-sulfur-modification-protein-DndB-[Candidatus-Rokubacteria-bacterium]	2053607
MBE0567699.1	DndB-ParB-nuclease	GCA_014859985.1	365	Bacteria>Candidatus_Krumholzibacteriota>Candidatus_Krumholzibacteria>unclassified_Candidatus_Krumholzibacteria	Candidatus_Krumholzibacteria_bacterium	DNA-sulfur-modification-protein-DndB-[Krumholzibacteria-bacterium]	2774603
WP_195976869.1	DndB-ParB-nuclease	GCF_015668835.1	415	Bacteria>FCB_group>Bacteroidetes/Chlorobi_group>Bacteroidetes>Bacteroidia	Alistipes_onderdonkii	DGQHR-domain-containing-protein-[Alistipes-onderdonkii]	328813
WP_229126842.1	DndB-ParB-nuclease	GCF_015561075.1	117	Bacteria>FCB_group>Bacteroidetes/Chlorobi_group>Bacteroidetes>Bacteroidia	Alistipes_onderdonkii	hypothetical-protein-[Alistipes-onderdonkii]	328813
NBJ06609.1	DndB-ParB-nuclease	GCA_009911575.1	404	Bacteria>FCB_group>Bacteroidetes/Chlorobi_group>Bacteroidetes>Bacteroidia	Alistipes_sp._Z76	DGQHR-domain-containing-protein-[Alistipes-sp.-Z76]	2304565
WP_006254211.1	DndB-ParB-nuclease	GCF_000159995.1	417	Bacteria>FCB_group>Bacteroidetes/Chlorobi_group>Bacteroidetes>Bacteroidia	Alloprevotella_tannerae	DGQHR-domain-containing-protein-[Alloprevotella-tannerae]	76122
WP_237785517.1	DndB-ParB-nuclease	-	433	Bacteria>FCB_group>Bacteroidetes/Chlorobi_group>Bacteroidetes>Bacteroidia	Alloprevotella_tannerae	DGQHR-domain-containing-protein-[Alloprevotella-tannerae]	76122
WP_195617166.1	DndB-ParB-nuclease	GCF_015557995.1	431	Bacteria>FCB_group>Bacteroidetes/Chlorobi_group>Bacteroidetes>Bacteroidia	Bacteroidaceae	MULTISPECIES:-DGQHR-domain-containing-protein-[Bacteroidaceae]	815
WP_055256800.1	DndB-ParB-nuclease	GCF_001405515.1	397	Bacteria>FCB_group>Bacteroidetes/Chlorobi_group>Bacteroidetes>Bacteroidia	Bacteroidaceae	MULTISPECIES:-DNA-phosphorothioation-associated-DGQHR-protein-1-[Bacteroidaceae]	815
OAV73661.1	DndB-ParB-nuclease	GCA_001657625.1	443	Bacteria>FCB_group>Bacteroidetes/Chlorobi_group>Bacteroidetes>Bacteroidia	Bacteroidales_bacterium_Barb6	DNA-phosphorothioation-associated-DGQHR-protein-1-[Bacteroidales-bacterium-Barb6]	1633201
MBI9055105.1	DndB-ParB-nuclease	GCA_016342685.1	392	Bacteria>FCB_group>Bacteroidetes/Chlorobi_group>Bacteroidetes>Bacteroidia	Bacteroidales_bacterium	DGQHR-domain-containing-protein-[Bacteroidales-bacterium]	2030927
MBK7625647.1	DndB-ParB-nuclease	GCA_016709865.1	435	Bacteria>FCB_group>Bacteroidetes/Chlorobi_group>Bacteroidetes>Bacteroidia	Bacteroidales_bacterium	DGQHR-domain-containing-protein-[Bacteroidales-bacterium]	2030927
MBO7224708.1	DndB-ParB-nuclease	GCA_017647065.1	415	Bacteria>FCB_group>Bacteroidetes/Chlorobi_group>Bacteroidetes>Bacteroidia	Bacteroidales_bacterium	DGQHR-domain-containing-protein-[Bacteroidales-bacterium]	2030927
MBO7276355.1	DndB-ParB-nuclease	GCA_017647785.1	428	Bacteria>FCB_group>Bacteroidetes/Chlorobi_group>Bacteroidetes>Bacteroidia	Bacteroidales_bacterium	DGQHR-domain-containing-protein-[Bacteroidales-bacterium]	2030927
MBO7278962.1	DndB-ParB-nuclease	GCA_017647795.1	436	Bacteria>FCB_group>Bacteroidetes/Chlorobi_group>Bacteroidetes>Bacteroidia	Bacteroidales_bacterium	DGQHR-domain-containing-protein-[Bacteroidales-bacterium]	2030927
MBO7494833.1	DndB-ParB-nuclease	GCA_017650065.1	417	Bacteria>FCB_group>Bacteroidetes/Chlorobi_group>Bacteroidetes>Bacteroidia	Bacteroidales_bacterium	DGQHR-domain-containing-protein-[Bacteroidales-bacterium]	2030927
MBQ0062087.1	DndB-ParB-nuclease	GCA_018065125.1	424	Bacteria>FCB_group>Bacteroidetes/Chlorobi_group>Bacteroidetes>Bacteroidia	Bacteroidales_bacterium	DGQHR-domain-containing-protein-[Bacteroidales-bacterium]	2030927
MBQ5891032.1	DndB-ParB-nuclease	GCA_017381455.1	428	Bacteria>FCB_group>Bacteroidetes/Chlorobi_group>Bacteroidetes>Bacteroidia	Bacteroidales_bacterium	DGQHR-domain-containing-protein-[Bacteroidales-bacterium]	2030927
MBQ6305725.1	DndB-ParB-nuclease	GCA_017428285.1	417	Bacteria>FCB_group>Bacteroidetes/Chlorobi_group>Bacteroidetes>Bacteroidia	Bacteroidales_bacterium	DGQHR-domain-containing-protein-[Bacteroidales-bacterium]	2030927
MBR4690158.1	DndB-ParB-nuclease	GCA_017535565.1	417	Bacteria>FCB_group>Bacteroidetes/Chlorobi_group>Bacteroidetes>Bacteroidia	Bacteroidales_bacterium	DGQHR-domain-containing-protein-[Bacteroidales-bacterium]	2030927
MCB8999921.1	DndB-ParB-nuclease	GCA_020636705.1	413	Bacteria>FCB_group>Bacteroidetes/Chlorobi_group>Bacteroidetes>Bacteroidia	Bacteroidales_bacterium	DGQHR-domain-containing-protein-[Bacteroidales-bacterium]	2030927
TGX84249.1	DndB-ParB-nuclease	GCA_004792655.1	402	Bacteria>FCB_group>Bacteroidetes/Chlorobi_group>Bacteroidetes>Bacteroidia	Bacteroidales_bacterium	DGQHR-domain-containing-protein-[Bacteroidales-bacterium]	2030927
HBG69686.1	DndB-ParB-nuclease	GCA_003499605.1	406	Bacteria>FCB_group>Bacteroidetes/Chlorobi_group>Bacteroidetes>Bacteroidia	Bacteroidales_bacterium	hypothetical-protein-[Bacteroidales-bacterium]	2030927
WP_046824815.1	DndB-ParB-nuclease	GCF_002763715.1	417	Bacteria>FCB_group>Bacteroidetes/Chlorobi_group>Bacteroidetes>Bacteroidia	Bacteroidales	MULTISPECIES:-DGQHR-domain-containing-protein-[Bacteroidales]	171549
WP_087412144.1	DndB-ParB-nuclease	GCF_015560045.1	421	Bacteria>FCB_group>Bacteroidetes/Chlorobi_group>Bacteroidetes>Bacteroidia	Bacteroides_clarus	DGQHR-domain-containing-protein-[Bacteroides-clarus]	626929
THC64555.1	DndB-ParB-nuclease	GCA_004798515.1	421	Bacteria>FCB_group>Bacteroidetes/Chlorobi_group>Bacteroidetes>Bacteroidia	Bacteroides_fragilis	DGQHR-domain-containing-protein-[Bacteroides-fragilis]	817
WP_195546717.1	DndB-ParB-nuclease	GCF_015556115.1	397	Bacteria>FCB_group>Bacteroidetes/Chlorobi_group>Bacteroidetes>Bacteroidia	Bacteroides_intestinalis	DNA-phosphorothioation-associated-DGQHR-protein-1-[Bacteroides-intestinalis]	329854
WP_211459221.1	DndB-ParB-nuclease	GCF_018291945.1	412	Bacteria>FCB_group>Bacteroidetes/Chlorobi_group>Bacteroidetes>Bacteroidia	Bacteroides_salyersiae	DGQHR-domain-containing-protein-[Bacteroides-salyersiae]	291644
EEY84705.1	DndB-ParB-nuclease	GCA_000162175.1	412	Bacteria>FCB_group>Bacteroidetes/Chlorobi_group>Bacteroidetes>Bacteroidia	Bacteroides_sp._2_1_33B	DGQHR-domain-protein-[Bacteroides-sp.-2_1_33B]	469589
WP_117578940.1	DndB-ParB-nuclease	GCF_003434395.1	427	Bacteria>FCB_group>Bacteroidetes/Chlorobi_group>Bacteroidetes>Bacteroidia	Bacteroides_sp._AM23-12	DGQHR-domain-containing-protein-[Bacteroides-sp.-AM23-12]	2292942
WP_087251535.1	DndB-ParB-nuclease	GCF_002160215.1	397	Bacteria>FCB_group>Bacteroidetes/Chlorobi_group>Bacteroidetes>Bacteroidia	Bacteroides_sp._An19	DNA-phosphorothioation-associated-DGQHR-protein-1-[Bacteroides-sp.-An19]	1965580
MBD5344274.1	DndB-ParB-nuclease	GCA_014802225.1	404	Bacteria>FCB_group>Bacteroidetes/Chlorobi_group>Bacteroidetes>Bacteroidia	Bacteroides_sp.	DGQHR-domain-containing-protein-[Bacteroides-sp.]	29523
WP_151882837.1	DndB-ParB-nuclease	GCF_009020755.1	414	Bacteria>FCB_group>Bacteroidetes/Chlorobi_group>Bacteroidetes>Bacteroidia	Bacteroides_uniformis	DGQHR-domain-containing-protein-[Bacteroides-uniformis]	820
WP_065220179.1	DndB-ParB-nuclease	GCF_902376195.1	397	Bacteria>FCB_group>Bacteroidetes/Chlorobi_group>Bacteroidetes>Bacteroidia	Butyricimonas	MULTISPECIES:-DNA-phosphorothioation-associated-DGQHR-protein-1-[Butyricimonas]	574697
MBU3808933.1	DndB-ParB-nuclease	GCA_018884025.1	421	Bacteria>FCB_group>Bacteroidetes/Chlorobi_group>Bacteroidetes>Bacteroidia	Candidatus_Phocaeicola_faecipullorum	DGQHR-domain-containing-protein-[Candidatus-Phocaeicola-faecipullorum]	2838733
HIZ05172.1	DndB-ParB-nuclease	GCA_019116145.1	354	Bacteria>FCB_group>Bacteroidetes/Chlorobi_group>Bacteroidetes>Bacteroidia	Candidatus_Phocaeicola_gallistercoris	DNA-phosphorothioation-associated-DGQHR-protein-1-[Candidatus-Phocaeicola-gallistercoris]	2838735
WP_163348040.1	DndB-ParB-nuclease	GCF_010448835.1	445	Bacteria>FCB_group>Bacteroidetes/Chlorobi_group>Bacteroidetes>Bacteroidia	Draconibacterium_halophilum	DGQHR-domain-containing-protein-[Draconibacterium-halophilum]	2706887
WP_110311907.1	DndB-ParB-nuclease	GCF_003201355.1	414	Bacteria>FCB_group>Bacteroidetes/Chlorobi_group>Bacteroidetes>Bacteroidia	Dysgonomonas_alginatilytica	DGQHR-domain-containing-protein-[Dysgonomonas-alginatilytica]	1605892
MBE6343693.1	DndB-ParB-nuclease	GCA_015061365.1	414	Bacteria>FCB_group>Bacteroidetes/Chlorobi_group>Bacteroidetes>Bacteroidia	Lentimicrobiaceae_bacterium	DGQHR-domain-containing-protein-[Lentimicrobiaceae-bacterium]	2026756
WP_136675657.1	DndB-ParB-nuclease	-	566	Bacteria>FCB_group>Bacteroidetes/Chlorobi_group>Bacteroidetes>Bacteroidia	Marinifilum_sp._JC120	hypothetical-protein-[Marinifilum-sp.-JC120]	2302941
MVZ89006.1	DndB-ParB-nuclease	GCA_009768845.1	365	Bacteria>FCB_group>Bacteroidetes/Chlorobi_group>Bacteroidetes>Bacteroidia	Microbacter_sp._ANSKLAB05	DNA-sulfur-modification-protein-DndB-[Microbacter-sp.-ANSKLAB05]	2684407
MVZ89003.1	DndB-ParB-nuclease	GCA_009768845.1	563	Bacteria>FCB_group>Bacteroidetes/Chlorobi_group>Bacteroidetes>Bacteroidia	Microbacter_sp._ANSKLAB05	hypothetical-protein-[Microbacter-sp.-ANSKLAB05]	2684407
MCC8174481.1	DndB-ParB-nuclease	GCA_020860925.1	351	Bacteria>FCB_group>Bacteroidetes/Chlorobi_group>Bacteroidetes>Bacteroidia	Odoribacter_sp.	DNA-sulfur-modification-protein-DndB-[Odoribacter-sp.]	1965233
MBO5086233.1	DndB-ParB-nuclease	GCA_017622455.1	409	Bacteria>FCB_group>Bacteroidetes/Chlorobi_group>Bacteroidetes>Bacteroidia	Paludibacteraceae_bacterium	DGQHR-domain-containing-protein-[Paludibacteraceae-bacterium]	2748031
MBP5421843.1	DndB-ParB-nuclease	GCA_017961465.1	423	Bacteria>FCB_group>Bacteroidetes/Chlorobi_group>Bacteroidetes>Bacteroidia	Paludibacteraceae_bacterium	DGQHR-domain-containing-protein-[Paludibacteraceae-bacterium]	2748031
WP_195605184.1	DndB-ParB-nuclease	GCF_015556515.1	412	Bacteria>FCB_group>Bacteroidetes/Chlorobi_group>Bacteroidetes>Bacteroidia	Parabacteroides_distasonis	DGQHR-domain-containing-protein-[Parabacteroides-distasonis]	823
WP_129734885.1	DndB-ParB-nuclease	GCF_014647375.1	410	Bacteria>FCB_group>Bacteroidetes/Chlorobi_group>Bacteroidetes>Bacteroidia	Parabacteroides	MULTISPECIES:-DGQHR-domain-containing-protein-[Parabacteroides]	375288
WP_008630087.1	DndB-ParB-nuclease	GCF_902374365.1	397	Bacteria>FCB_group>Bacteroidetes/Chlorobi_group>Bacteroidetes>Bacteroidia	Paraprevotella_xylaniphila	DNA-phosphorothioation-associated-DGQHR-protein-1-[Paraprevotella-xylaniphila]	454155
WP_204482015.1	DndB-ParB-nuclease	GCF_016901555.1	426	Bacteria>FCB_group>Bacteroidetes/Chlorobi_group>Bacteroidetes>Bacteroidia	Phocaeicola_coprocola	DGQHR-domain-containing-protein-[Phocaeicola-coprocola]	310298
WP_008450453.1	DndB-ParB-nuclease	GCF_000177355.1	394	Bacteria>FCB_group>Bacteroidetes/Chlorobi_group>Bacteroidetes>Bacteroidia	Prevotella_amnii	DNA-phosphorothioation-associated-DGQHR-protein-1-[Prevotella-amnii]	419005
WP_004335941.1	DndB-ParB-nuclease	GCF_000177315.1	402	Bacteria>FCB_group>Bacteroidetes/Chlorobi_group>Bacteroidetes>Bacteroidia	Prevotella_bivia	DNA-phosphorothioation-associated-DGQHR-protein-1-[Prevotella-bivia]	28125
MBF1399600.1	DndB-ParB-nuclease	GCA_015262435.1	428	Bacteria>FCB_group>Bacteroidetes/Chlorobi_group>Bacteroidetes>Bacteroidia	Prevotella_histicola	DGQHR-domain-containing-protein-[Prevotella-histicola]	470565
MBF1418260.1	DndB-ParB-nuclease	GCA_015262545.1	428	Bacteria>FCB_group>Bacteroidetes/Chlorobi_group>Bacteroidetes>Bacteroidia	Prevotella_histicola	DGQHR-domain-containing-protein-[Prevotella-histicola]	470565
MBF1430150.1	DndB-ParB-nuclease	GCA_015262365.1	427	Bacteria>FCB_group>Bacteroidetes/Chlorobi_group>Bacteroidetes>Bacteroidia	Prevotella_melaninogenica	DGQHR-domain-containing-protein-[Prevotella-melaninogenica]	28132
WP_024992518.1	DndB-ParB-nuclease	GCF_000613725.1	417	Bacteria>FCB_group>Bacteroidetes/Chlorobi_group>Bacteroidetes>Bacteroidia	Prevotella_pleuritidis	DGQHR-domain-containing-protein-[Prevotella-pleuritidis]	407975
WP_021825760.1	DndB-ParB-nuclease	GCF_000477535.1	417	Bacteria>FCB_group>Bacteroidetes/Chlorobi_group>Bacteroidetes>Bacteroidia	Prevotella_salivae	DGQHR-domain-containing-protein-[Prevotella-salivae]	228604

Accession	Annotation	GCA	Length	Phyletic.spread	Strain.name	Defline	Species.Taxid
MBQ2951239.1	DndB-ParB-nuclease	GCA_017410365.1	425	Bacteria>FCB_group>Bacteroidetes/Chlorobi_group>Bacteroidetes>Bacteroidia	Prevotella_sp.	DGQHR-domain-containing-protein-[Prevotella-sp.]	59823
MBR1654892.1	DndB-ParB-nuclease	GCA_017470445.1	417	Bacteria>FCB_group>Bacteroidetes/Chlorobi_group>Bacteroidetes>Bacteroidia	Prevotella_sp.	DGQHR-domain-containing-protein-[Prevotella-sp.]	59823
WP_104368834.1	DndB-ParB-nuclease	GCF_002933775.1	425	Bacteria>FCB_group>Bacteroidetes/Chlorobi_group>Bacteroidetes>Bacteroidia	Prevotella_sp._MGM1	DGQHR-domain-containing-protein-[Prevotella-sp.-MGM1]	2033405
WP_125235388.1	DndB-ParB-nuclease	GCF_003932845.1	402	Bacteria>FCB_group>Bacteroidetes/Chlorobi_group>Bacteroidetes>Bacteroidia	Prevotella_sp._OH937_COT-195	DNA-phosphorothioation-associated-DGQHR-protein-1-[Prevotella-sp.-OH937_COT-195]	2491051
WP_107620595.1	DndB-ParB-nuclease	GCF_003043945.1	392	Bacteria>FCB_group>Bacteroidetes/Chlorobi_group>Bacteroidetes>Bacteroidia	Prevotella_sp._oral_taxon_376	DNA-phosphorothioation-associated-DGQHR-protein-1-[Prevotella-sp.-oral-taxon-376]	712466
MBP7508131.1	DndB-ParB-nuclease	GCA_017998095.1	374	Bacteria>FCB_group>Bacteroidetes/Chlorobi_group>Bacteroidetes>Bacteroidia	Prolixibacteraceae_bacterium	DNA-sulfur-modification-protein-DndB-[Prolixibacteraceae-bacterium]	2053594
WP_051214524.1	DndB-ParB-nuclease	GCF_000427365.1	415	Bacteria>FCB_group>Bacteroidetes/Chlorobi_group>Bacteroidetes>Bacteroidia	Rikenella_microfusus	DGQHR-domain-containing-protein-[Rikenella-microfusus]	28139
SUE34429.1	DndB-ParB-nuclease	GCA_900455755.1	415	Bacteria>FCB_group>Bacteroidetes/Chlorobi_group>Bacteroidetes>Bacteroidia	Rikenella_microfusus	DGQHR-domain-[Rikenella-microfusus]	28139
WP_075588648.1	DndB-ParB-nuclease	GCF_001938785.1	428	Bacteria>FCB_group>Bacteroidetes/Chlorobi_group>Bacteroidetes>Bacteroidia	Tannerella_forsythia	DGQHR-domain-containing-protein-[Tannerella-forsythia]	28112
MBK6483959.1	DndB-ParB-nuclease	GCA_016704325.1	416	Bacteria>FCB_group>Bacteroidetes/Chlorobi_group>Bacteroidetes>Chitinophagia	Chitinophagaceae_bacterium	DGQHR-domain-containing-protein-[Chitinophagaceae-bacterium]	1869212
WP_078669522.1	DndB-ParB-nuclease	GCF_900167135.1	526	Bacteria>FCB_group>Bacteroidetes/Chlorobi_group>Bacteroidetes>Chitinophagia	Chitinophaga_eiseniae	DNA-phosphorothioation-associated-DGQHR-protein-1-[Chitinophaga-eiseniae]	634771
NOT92477.1	DndB-ParB-nuclease	GCA_013141115.1	417	Bacteria>FCB_group>Bacteroidetes/Chlorobi_group>Bacteroidetes>Chitinophagia	Ferruginibacter_sp.	DGQHR-domain-containing-protein-[Ferruginibacter-sp.]	1940288
NOT93840.1	DndB-ParB-nuclease	GCA_013141115.1	418	Bacteria>FCB_group>Bacteroidetes/Chlorobi_group>Bacteroidetes>Chitinophagia	Ferruginibacter_sp.	DGQHR-domain-containing-protein-[Ferruginibacter-sp.]	1940288
MBL7682033.1	DndB-ParB-nuclease	GCA_016786615.1	416	Bacteria>FCB_group>Bacteroidetes/Chlorobi_group>Bacteroidetes>Chitinophagia	Flavipsychrobacter_sp.	DGQHR-domain-containing-protein-[Flavipsychrobacter-sp.]	2307286
WP_090657726.1	DndB-ParB-nuclease	GCF_900115755.1	413	Bacteria>FCB_group>Bacteroidetes/Chlorobi_group>Bacteroidetes>Chitinophagia	Parafilimonas_terrae	DGQHR-domain-containing-protein-[Parafilimonas-terrae]	1465490
WP_111672689.1	DndB-ParB-nuclease	GCF_003259255.1	443	Bacteria>FCB_group>Bacteroidetes/Chlorobi_group>Bacteroidetes>Cytophagia	Algoriphagus_litoralis	DGQHR-domain-containing-protein-[Algoriphagus-litoralis]	2202829
WP_109745125.1	DndB-ParB-nuclease	GCF_003148625.1	425	Bacteria>FCB_group>Bacteroidetes/Chlorobi_group>Bacteroidetes>Cytophagia	Arcicella_aurantiaca	DGQHR-domain-containing-protein-[Arcicella-aurantiaca]	591202
WP_109745128.1	DndB-ParB-nuclease	GCF_003148625.1	401	Bacteria>FCB_group>Bacteroidetes/Chlorobi_group>Bacteroidetes>Cytophagia	Arcicella_aurantiaca	DGQHR-domain-containing-protein-[Arcicella-aurantiaca]	591202
WP_132121420.1	DndB-ParB-nuclease	GCF_004348825.1	404	Bacteria>FCB_group>Bacteroidetes/Chlorobi_group>Bacteroidetes>Cytophagia	Arundinibacter_roseus	DGQHR-domain-containing-protein-[Arundinibacter-roseus]	2070510
WP_132121424.1	DndB-ParB-nuclease	GCF_004348825.1	416	Bacteria>FCB_group>Bacteroidetes/Chlorobi_group>Bacteroidetes>Cytophagia	Arundinibacter_roseus	DGQHR-domain-containing-protein-[Arundinibacter-roseus]	2070510
MBY0425659.1	DndB-ParB-nuclease	GCA_019751275.1	329	Bacteria>FCB_group>Bacteroidetes/Chlorobi_group>Bacteroidetes>Cytophagia	Cytophagales_bacterium	DGQHR-domain-containing-protein-[Cytophagales-bacterium]	2053541
WP_149639896.1	DndB-ParB-nuclease	GCF_008369915.1	384	Bacteria>FCB_group>Bacteroidetes/Chlorobi_group>Bacteroidetes>Cytophagia	Dyadobacter_sp._UC_10	DGQHR-domain-containing-protein-[Dyadobacter-sp.-UC-10]	2605428
WP_215222606.1	DndB-ParB-nuclease	GCF_018642165.1	447	Bacteria>FCB_group>Bacteroidetes/Chlorobi_group>Bacteroidetes>Cytophagia	Echinicola_shivajiensis	DGQHR-domain-containing-protein-[Echinicola-shivajiensis]	1035916
MBK7649443.1	DndB-ParB-nuclease	GCA_016709845.1	418	Bacteria>FCB_group>Bacteroidetes/Chlorobi_group>Bacteroidetes>Cytophagia	Flammeovirgaceae_bacterium	DGQHR-domain-containing-protein-[Flammeovirgaceae-bacterium]	2026740
WP_091516219.1	DndB-ParB-nuclease	GCF_900112255.1	411	Bacteria>FCB_group>Bacteroidetes/Chlorobi_group>Bacteroidetes>Cytophagia	Flexibacter_flexilis	DGQHR-domain-containing-protein-[Flexibacter-flexilis]	998
WP_143401560.1	DndB-ParB-nuclease	GCF_007097395.1	405	Bacteria>FCB_group>Bacteroidetes/Chlorobi_group>Bacteroidetes>Cytophagia	Fulvivirga_sp._M361	DGQHR-domain-containing-protein-[Fulvivirga-sp.-M361]	2594266
WP_135431156.1	DndB-ParB-nuclease	GCF_004745825.1	415	Bacteria>FCB_group>Bacteroidetes/Chlorobi_group>Bacteroidetes>Cytophagia	Hymenobacter_fodinae	DGQHR-domain-containing-protein-[Hymenobacter-sp.-92R-1]	2510796
WP_206986792.1	DndB-ParB-nuclease	GCF_017313265.1	412	Bacteria>FCB_group>Bacteroidetes/Chlorobi_group>Bacteroidetes>Cytophagia	Hymenobacter	MULTISPECIES:-DGQHR-domain-containing-protein-[Hymenobacter]	89966
WP_208178315.1	DndB-ParB-nuclease	GCF_017571495.1	406	Bacteria>FCB_group>Bacteroidetes/Chlorobi_group>Bacteroidetes>Cytophagia	Hymenobacter_negativus	DGQHR-domain-containing-protein-[Hymenobacter-negativus]	2795026
WP_082685124.1	DndB-ParB-nuclease	GCF_001507645.1	430	Bacteria>FCB_group>Bacteroidetes/Chlorobi_group>Bacteroidetes>Cytophagia	Hymenobacter_sedentarius	DGQHR-domain-containing-protein-[Hymenobacter-sedentarius]	1411621
WP_068339796.1	DndB-ParB-nuclease	GCF_001596155.1	173	Bacteria>FCB_group>Bacteroidetes/Chlorobi_group>Bacteroidetes>Cytophagia	Hymenobacter_sp._PAMC_26554	hypothetical-protein-[Hymenobacter-sp.-PAMC-26554]	1484116
WP_082808820.1	DndB-ParB-nuclease	GCF_001596155.1	260	Bacteria>FCB_group>Bacteroidetes/Chlorobi_group>Bacteroidetes>Cytophagia	Hymenobacter_sp._PAMC_26554	hypothetical-protein-[Hymenobacter-sp.-PAMC-26554]	1484116
WP_035805696.1	DndB-ParB-nuclease	GCF_000390185.1	425	Bacteria>FCB_group>Bacteroidetes/Chlorobi_group>Bacteroidetes>Cytophagia	Lunatimonas_lonarensis	DGQHR-domain-containing-protein-[Lunatimonas-lonarensis]	1232681
EON76969.1	DndB-ParB-nuclease	GCA_000390185.1	429	Bacteria>FCB_group>Bacteroidetes/Chlorobi_group>Bacteroidetes>Cytophagia	Lunatimonas_lonarensis	hypothetical-protein-ADIS_2512-[Lunatimonas-lonarensis]	1232681
WP_230407009.1	DndB-ParB-nuclease	-	396	Bacteria>FCB_group>Bacteroidetes/Chlorobi_group>Bacteroidetes>Cytophagia	Pontibacter_cellulosilyticus	DGQHR-domain-containing-protein-[Pontibacter-cellulosilyticus]	1720253
WP_076666397.1	DndB-ParB-nuclease	GCF_900156345.1	407	Bacteria>FCB_group>Bacteroidetes/Chlorobi_group>Bacteroidetes>Cytophagia	Pontibacter_indicus	DNA-phosphorothioation-associated-DGQHR-protein-1-[Pontibacter-indicus]	1317125
QHT68702.1	DndB-ParB-nuclease	GCA_010119975.1	585	Bacteria>FCB_group>Bacteroidetes/Chlorobi_group>Bacteroidetes>Cytophagia	Rhodocytophaga_rosea	DGQHR-domain-containing-protein-[Rhodocytophaga-rosea]	2704465
WP_162444710.1	DndB-ParB-nuclease	GCF_010119975.1	585	Bacteria>FCB_group>Bacteroidetes/Chlorobi_group>Bacteroidetes>Cytophagia	Rhodocytophaga_rosea	DGQHR-domain-containing-protein-[Rhodocytophaga-rosea]	2704465
WP_122933603.1	DndB-ParB-nuclease	GCF_003711225.1	420	Bacteria>FCB_group>Bacteroidetes/Chlorobi_group>Bacteroidetes>Cytophagia	Runella_sp._SP2	DGQHR-domain-containing-protein-[Runella-sp.-SP2]	2268026
MBO2012580.1	DndB-ParB-nuclease	GCA_017571495.1	406	Bacteria>FCB_group>Bacteroidetes/Chlorobi_group>Bacteroidetes>Cytophagia	Siccationidurans_soli	DGQHR-domain-containing-protein-[Siccationidurans-soli]	373890
WP_097126508.1	DndB-ParB-nuclease	GCF_900230225.1	440	Bacteria>FCB_group>Bacteroidetes/Chlorobi_group>Bacteroidetes>Cytophagia	Spirosoma_fluviale	DGQHR-domain-containing-protein-[Spirosoma-fluviale]	1597977
MVM40839.1	DndB-ParB-nuclease	GCA_009754885.1	602	Bacteria>FCB_group>Bacteroidetes/Chlorobi_group>Bacteroidetes>Cytophagia	Spirosoma_telluris	DGQHR-domain-containing-protein-[Spirosoma-telluris]	2183553
WP_186738176.1	DndB-ParB-nuclease	GCF_014282275.1	286	Bacteria>FCB_group>Bacteroidetes/Chlorobi_group>Bacteroidetes>Cytophagia	Spirosoma_utsteinense	DGQHR-domain-containing-protein-[Spirosoma-utsteinense]	2585773
WP_186749959.1	DndB-ParB-nuclease	GCF_014282255.1	185	Bacteria>FCB_group>Bacteroidetes/Chlorobi_group>Bacteroidetes>Cytophagia	Spirosoma_utsteinense	hypothetical-protein-[Spirosoma-utsteinense]	2585773
WP_202335808.1	DndB-ParB-nuclease	GCF_016757735.1	410	Bacteria>FCB_group>Bacteroidetes/Chlorobi_group>Bacteroidetes>Flavobacteriia	Aequorivita	MULTISPECIES:-DGQHR-domain-containing-protein-[unclassified-Aequorivita]	153265
WP_139853876.1	DndB-ParB-nuclease	GCF_006346335.1	461	Bacteria>FCB_group>Bacteroidetes/Chlorobi_group>Bacteroidetes>Flavobacteriia	Aequorivita_sinensis	DGQHR-domain-containing-protein-[Aequorivita-sinensis]	1382458
PHR11293.1	DndB-ParB-nuclease	GCA_002733915.1	411	Bacteria>FCB_group>Bacteroidetes/Chlorobi_group>Bacteroidetes>Flavobacteriia	Aequorivita_sp.	hypothetical-protein-COA40_11805-[Aequorivita-sp.]	1872390
MBQ0737616.1	DndB-ParB-nuclease	GCA_018066725.1	446	Bacteria>FCB_group>Bacteroidetes/Chlorobi_group>Bacteroidetes>Flavobacteriia	Aquimarina_celericrescens	DGQHR-domain-containing-protein-[Aquimarina-celericrescens]	1964542
WP_126160987.1	DndB-ParB-nuclease	GCF_003957295.1	410	Bacteria>FCB_group>Bacteroidetes/Chlorobi_group>Bacteroidetes>Flavobacteriia	Arenibacter_aquaticus	DGQHR-domain-containing-protein-[Arenibacter-aquaticus]	2489054
WP_219052753.1	DndB-ParB-nuclease	GCF_019312585.1	403	Bacteria>FCB_group>Bacteroidetes/Chlorobi_group>Bacteroidetes>Flavobacteriia	Aureisphaera_sp._CAU_1614	DNA-phosphorothioation-associated-DGQHR-protein-1-[Aureisphaera-sp.-CAU-1614]	2857106
WP_089119013.1	DndB-ParB-nuclease	GCF_900197635.1	669	Bacteria>FCB_group>Bacteroidetes/Chlorobi_group>Bacteroidetes>Flavobacteriia	Cellulophaga_lytica	hypothetical-protein-[Cellulophaga-lytica]	979
WP_136521957.1	DndB-ParB-nuclease	GCF_004916905.1	414	Bacteria>FCB_group>Bacteroidetes/Chlorobi_group>Bacteroidetes>Flavobacteriia	Chryseobacterium_candidae	DGQHR-domain-containing-protein-[Chryseobacterium-candidae]	1978493
WP_123876816.1	DndB-ParB-nuclease	GCF_003815695.1	407	Bacteria>FCB_group>Bacteroidetes/Chlorobi_group>Bacteroidetes>Flavobacteriia	Chryseobacterium_carnipullorum	DGQHR-domain-containing-protein-[Chryseobacterium-carnipullorum]	1124835
WP_191736966.1	DndB-ParB-nuclease	GCF_014837145.1	418	Bacteria>FCB_group>Bacteroidetes/Chlorobi_group>Bacteroidetes>Flavobacteriia	Chryseobacterium_caseinilyticum	DGQHR-domain-containing-protein-[Chryseobacterium-caseinilyticum]	2771428
WP_066690413.1	DndB-ParB-nuclease	GCF_900142615.1	417	Bacteria>FCB_group>Bacteroidetes/Chlorobi_group>Bacteroidetes>Flavobacteriia	Chryseobacterium_contaminans	DGQHR-domain-containing-protein-[Chryseobacterium-contaminans]	1423959
WP_051349260.1	DndB-ParB-nuclease	GCF_000430845.1	419	Bacteria>FCB_group>Bacteroidetes/Chlorobi_group>Bacteroidetes>Flavobacteriia	Chryseobacterium_gregarium	DGQHR-domain-containing-protein-[Chryseobacterium-gregarium]	456299
WP_165852282.1	DndB-ParB-nuclease	GCF_003385515.1	440	Bacteria>FCB_group>Bacteroidetes/Chlorobi_group>Bacteroidetes>Flavobacteriia	Chryseobacterium_pennae	DGQHR-domain-containing-protein-[Chryseobacterium-pennae]	2258962
WP_180565398.1	DndB-ParB-nuclease	GCF_903969135.1	406	Bacteria>FCB_group>Bacteroidetes/Chlorobi_group>Bacteroidetes>Flavobacteriia	Chryseobacterium_sp._JV274	DGQHR-domain-containing-protein-[Chryseobacterium-sp.-JV274]	1932669
ACU07676.1	DndB-ParB-nuclease	GCA_000023725.1	410	Bacteria>FCB_group>Bacteroidetes/Chlorobi_group>Bacteroidetes>Flavobacteriia	Flavobacteriaceae_bacterium_3519-10	hypothetical-protein-FIC_01228-[Flavobacteriaceae-bacterium-3519-10]	531844
PIX15248.1	DndB-ParB-nuclease	GCA_002782485.1	246	Bacteria>FCB_group>Bacteroidetes/Chlorobi_group>Bacteroidetes>Flavobacteriia	Flavobacteriaceae_bacterium_CG_4_8_14_3_um_filter_31_8	hypothetical-protein-COZ74_00775,-partial-[Flavobacteriaceae-bacterium-CG_4_8_14_3_um_filter_31_8]	1973989
UPT70964.1	DndB-ParB-nuclease	GCA_023213215.1	284	Bacteria>FCB_group>Bacteroidetes/Chlorobi_group>Bacteroidetes>Flavobacteriia	Flavobacteriaceae_bacterium	DGQHR-domain-containing-protein-[Flavobacteriaceae-bacterium]	1871037
MAB47210.1	DndB-ParB-nuclease	GCA_002682935.1	411	Bacteria>FCB_group>Bacteroidetes/Chlorobi_group>Bacteroidetes>Flavobacteriia	Flavobacteriaceae_bacterium	DndB-[Flavobacteriaceae-bacterium]	1871037
HIE73038.1	DndB-ParB-nuclease	GCA_012960835.1	64	Bacteria>FCB_group>Bacteroidetes/Chlorobi_group>Bacteroidetes>Flavobacteriia	Flavobacteriales_bacterium	DGQHR-domain-containing-protein-[Flavobacteriales-bacterium]	2021391
MBA3985238.1	DndB-ParB-nuclease	GCA_013820965.1	410	Bacteria>FCB_group>Bacteroidetes/Chlorobi_group>Bacteroidetes>Flavobacteriia	Flavobacteriales_bacterium	DGQHR-domain-containing-protein-[Flavobacteriales-bacterium]	2021391
MBK7482254.1	DndB-ParB-nuclease	GCA_016706145.1	180	Bacteria>FCB_group>Bacteroidetes/Chlorobi_group>Bacteroidetes>Flavobacteriia	Flavobacteriales_bacterium	DGQHR-domain-containing-protein-[Flavobacteriales-bacterium]	2021391
MBK8707885.1	DndB-ParB-nuclease	GCA_016715385.1	415	Bacteria>FCB_group>Bacteroidetes/Chlorobi_group>Bacteroidetes>Flavobacteriia	Flavobacteriales_bacterium	DGQHR-domain-containing-protein-[Flavobacteriales-bacterium]	2021391
MBL4586842.1	DndB-ParB-nuclease	GCA_016763015.1	403	Bacteria>FCB_group>Bacteroidetes/Chlorobi_group>Bacteroidetes>Flavobacteriia	Flavobacteriales_bacterium	DGQHR-domain-containing-protein-[Flavobacteriales-bacterium]	2021391
MBS4039688.1	DndB-ParB-nuclease	GCA_018335925.1	411	Bacteria>FCB_group>Bacteroidetes/Chlorobi_group>Bacteroidetes>Flavobacteriia	Flavobacteriales_bacterium	DGQHR-domain-containing-protein-[Flavobacteriales-bacterium]	2021391
MBK6473781.1	DndB-ParB-nuclease	GCA_016704485.1	663	Bacteria>FCB_group>Bacteroidetes/Chlorobi_group>Bacteroidetes>Flavobacteriia	Flavobacteriales_bacterium	hypothetical-protein-[Flavobacteriales-bacterium]	2021391
MBK7482253.1	DndB-ParB-nuclease	GCA_016706145.1	225	Bacteria>FCB_group>Bacteroidetes/Chlorobi_group>Bacteroidetes>Flavobacteriia	Flavobacteriales_bacterium	hypothetical-protein-[Flavobacteriales-bacterium]	2021391
NCO64657.1	DndB-ParB-nuclease	GCA_009993745.1	415	Bacteria>FCB_group>Bacteroidetes/Chlorobi_group>Bacteroidetes>Flavobacteriia	Flavobacteriia_bacterium	DGQHR-domain-containing-protein-[Flavobacteriia-bacterium]	2044941
WP_089478528.1	DndB-ParB-nuclease	GCF_002222055.1	539	Bacteria>FCB_group>Bacteroidetes/Chlorobi_group>Bacteroidetes>Flavobacteriia	Flavobacterium_araucananum	AIPR-family-protein-[Flavobacterium-araucananum]	946678
WP_165571462.1	DndB-ParB-nuclease	GCF_000737695.1	428	Bacteria>FCB_group>Bacteroidetes/Chlorobi_group>Bacteroidetes>Flavobacteriia	Flavobacterium_hydatis	DGQHR-domain-containing-protein-[Flavobacterium-hydatis]	991
WP_014388838.1	DndB-ParB-nuclease	GCF_000455605.1	409	Bacteria>FCB_group>Bacteroidetes/Chlorobi_group>Bacteroidetes>Flavobacteriia	Flavobacterium_indicum	DNA-phosphorothioation-associated-DGQHR-protein-1-[Flavobacterium-indicum]	312277
WP_187656016.1	DndB-ParB-nuclease	GCF_014385475.1	446	Bacteria>FCB_group>Bacteroidetes/Chlorobi_group>Bacteroidetes>Flavobacteriia	Flavobacterium_macrobrachii	DGQHR-domain-containing-protein-[Flavobacterium-macrobrachii]	591204
WP_162845839.1	DndB-ParB-nuclease	GCF_014876705.1	451	Bacteria>FCB_group>Bacteroidetes/Chlorobi_group>Bacteroidetes>Flavobacteriia	Flavobacterium	MULTISPECIES:-DGQHR-domain-containing-protein-[unclassified-Flavobacterium]	237
WP_243230456.1	DndB-ParB-nuclease	GCF_022802975.1	443	Bacteria>FCB_group>Bacteroidetes/Chlorobi_group>Bacteroidetes>Flavobacteriia	Flavobacterium_pectinovorum	DGQHR-domain-containing-protein-[Flavobacterium-pectinovorum]	29533
WP_113637043.1	DndB-ParB-nuclease	GCF_003312425.1	422	Bacteria>FCB_group>Bacteroidetes/Chlorobi_group>Bacteroidetes>Flavobacteriia	Flavobacterium_psychrolimnae	DGQHR-domain-containing-protein-[Flavobacterium-psychrolimnae]	249351
KAF2516325.1	DndB-ParB-nuclease	GCA_004634195.1	451	Bacteria>FCB_group>Bacteroidetes/Chlorobi_group>Bacteroidetes>Flavobacteriia	Flavobacterium_salilacus_subsp._salilacus	DGQHR-domain-containing-protein-[Flavobacterium-sp.-SaA2.12]	2545633
WP_023575222.1	DndB-ParB-nuclease	GCF_000498515.1	403	Bacteria>FCB_group>Bacteroidetes/Chlorobi_group>Bacteroidetes>Flavobacteriia	Flavobacterium_saliperosum	DGQHR-domain-containing-protein-[Flavobacterium-saliperosum]	329186
ESU27662.1	DndB-ParB-nuclease	GCA_000498515.1	403	Bacteria>FCB_group>Bacteroidetes/Chlorobi_group>Bacteroidetes>Flavobacteriia	Flavobacterium_saliperosum_S13	hypothetical-protein-FSS13T_01330-[Flavobacterium-saliperosum-S13]	1341155
WP_211064954.1	DndB-ParB-nuclease	GCF_018080005.1	652	Bacteria>FCB_group>Bacteroidetes/Chlorobi_group>Bacteroidetes>Flavobacteriia	Flavobacterium_sp._CS20	hypothetical-protein-[Flavobacterium-sp.-CS20]	2775246
MBV2195056.1	DndB-ParB-nuclease	GCA_019105125.1	399	Bacteria>FCB_group>Bacteroidetes/Chlorobi_group>Bacteroidetes>Flavobacteriia	Flavobacterium_sp.	DGQHR-domain-containing-protein-[Flavobacterium-sp.]	239
MBF01201.1	DndB-ParB-nuclease	GCA_002714575.1	443	Bacteria>FCB_group>Bacteroidetes/Chlorobi_group>Bacteroidetes>Flavobacteriia	Flavobacterium_sp.	hypothetical-protein-[Flavobacterium-sp.]	239
WP_056191534.1	DndB-ParB-nuclease	GCF_001427905.1	400	Bacteria>FCB_group>Bacteroidetes/Chlorobi_group>Bacteroidetes>Flavobacteriia	Flavobacterium_sp._Root420	DGQHR-domain-containing-protein-[Flavobacterium-sp.-Root420]	1736533
WP_160749859.1	DndB-ParB-nuclease	GCF_009826935.1	673	Bacteria>FCB_group>Bacteroidetes/Chlorobi_group>Bacteroidetes>Flavobacteriia	Flavobacterium_sp._Sd200	hypothetical-protein-[Flavobacterium-sp.-Sd200]	2692211
WP_026977600.1	DndB-ParB-nuclease	GCF_000425485.1	655	Bacteria>FCB_group>Bacteroidetes/Chlorobi_group>Bacteroidetes>Flavobacteriia	Flavobacterium_tegetincola	hypothetical-protein-[Flavobacterium-tegetincola]	150172
MBL1280768.1	DndB-ParB-nuclease	GCA_002734065.2	474	Bacteria>FCB_group>Bacteroidetes/Chlorobi_group>Bacteroidetes>Flavobacteriia	Fluviicola_sp.	AIPR-family-protein-[Fluviicola-sp.]	1917219
PHR45463.1	DndB-ParB-nuclease	GCA_002734085.1	414	Bacteria>FCB_group>Bacteroidetes/Chlorobi_group>Bacteroidetes>Flavobacteriia	Fluviicola_sp.	hypothetical-protein-COA32_13560-[Fluviicola-sp.]	1917219
WP_103191865.1	DndB-ParB-nuclease	GCF_002900225.1	169	Bacteria>FCB_group>Bacteroidetes/Chlorobi_group>Bacteroidetes>Flavobacteriia	Formosa_algae	DGQHR-domain-containing-protein-[Formosa-algae]	225843
WP_130962955.1	DndB-ParB-nuclease	GCF_004310325.1	411	Bacteria>FCB_group>Bacteroidetes/Chlorobi_group>Bacteroidetes>Flavobacteriia	Hyunsoonleella_flava	DGQHR-domain-containing-protein-[Hyunsoonleella-sp.-T58]	2527939
WP_007096424.1	DndB-ParB-nuclease	GCF_000154725.1	414	Bacteria>FCB_group>Bacteroidetes/Chlorobi_group>Bacteroidetes>Flavobacteriia	Kordia_algicida	DGQHR-domain-containing-protein-[Kordia-algicida]	221066
WP_160129415.1	DndB-ParB-nuclease	GCF_009901525.1	449	Bacteria>FCB_group>Bacteroidetes/Chlorobi_group>Bacteroidetes>Flavobacteriia	Kordia_antarctica	DGQHR-domain-containing-protein-[Kordia-antarctica]	1218801
WP_036156542.1	DndB-ParB-nuclease	GCF_000744105.1	406	Bacteria>FCB_group>Bacteroidetes/Chlorobi_group>Bacteroidetes>Flavobacteriia	Maribacter_forsetii	DGQHR-domain-containing-protein-[Maribacter-forsetii]	444515
HAF77445.1	DndB-ParB-nuclease	GCA_003447055.1	411	Bacteria>FCB_group>Bacteroidetes/Chlorobi_group>Bacteroidetes>Flavobacteriia	Maribacter_sp.	hypothetical-protein-[Maribacter-sp.]	1897614
HAI44990.1	DndB-ParB-nuclease	GCA_003450275.1	411	Bacteria>FCB_group>Bacteroidetes/Chlorobi_group>Bacteroidetes>Flavobacteriia	Maribacter_sp.	hypothetical-protein-[Maribacter-sp.]	1897614
WP_027884012.1	DndB-ParB-nuclease	GCF_000423405.1	446	Bacteria>FCB_group>Bacteroidetes/Chlorobi_group>Bacteroidetes>Flavobacteriia	Mesonia_mobilis	DGQHR-domain-containing-protein-[Mesonia-mobilis]	369791
WP_159950923.1	DndB-ParB-nuclease	GCF_009832745.1	675	Bacteria>FCB_group>Bacteroidetes/Chlorobi_group>Bacteroidetes>Flavobacteriia	Polaribacter_septentrionalilitoris	hypothetical-protein-[Polaribacter-sp.-ANORD1]	2494657
WP_026776284.1	DndB-ParB-nuclease	GCF_000687935.1	445	Bacteria>FCB_group>Bacteroidetes/Chlorobi_group>Bacteroidetes>Flavobacteriia	Polaribacter_sp._Hel_I_88	DGQHR-domain-containing-protein-[Polaribacter-sp.-Hel_I_88]	1250006
WP_224463019.1	DndB-ParB-nuclease	GCF_020164535.1	675	Bacteria>FCB_group>Bacteroidetes/Chlorobi_group>Bacteroidetes>Flavobacteriia	Psychroflexus_sp._CCL10W	hypothetical-protein-[Psychroflexus-sp.-CCL10W]	2873598
WP_044630955.1	DndB-ParB-nuclease	GCF_000943565.1	450	Bacteria>FCB_group>Bacteroidetes/Chlorobi_group>Bacteroidetes>Flavobacteriia	Tamlana_sedimentorum	DGQHR-domain-containing-protein-[Tamlana-sedimentorum]	1435349
KJD36960.1	DndB-ParB-nuclease	GCA_000943565.1	450	Bacteria>FCB_group>Bacteroidetes/Chlorobi_group>Bacteroidetes>Flavobacteriia	Tamlana_sedimentorum	hypothetical-protein-PW52_00425-[Tamlana-sedimentorum]	1435349
GFD73167.1	DndB-ParB-nuclease	GCA_009811435.1	361	Bacteria>FCB_group>Bacteroidetes/Chlorobi_group>Bacteroidetes>Flavobacteriia	Tenacibaculum_sp._KUL113	hypothetical-protein-KUL113_25870-[Tenacibaculum-sp.-KUL113]	2480800
GFD73172.1	DndB-ParB-nuclease	GCA_009811435.1	386	Bacteria>FCB_group>Bacteroidetes/Chlorobi_group>Bacteroidetes>Flavobacteriia	Tenacibaculum_sp._KUL113	hypothetical-protein-KUL113_25920-[Tenacibaculum-sp.-KUL113]	2480800
WP_242937762.1	DndB-ParB-nuclease	GCF_022760175.1	411	Bacteria>FCB_group>Bacteroidetes/Chlorobi_group>Bacteroidetes>Flavobacteriia	Zhouia_spongiae	DGQHR-domain-containing-protein-[Zhouia-spongiae]	2202721
MCB9310863.1	DndB-ParB-nuclease	GCA_020636195.1	408	Bacteria>FCB_group>Bacteroidetes/Chlorobi_group>Bacteroidetes>Saprospiria	Lewinellaceae_bacterium	DGQHR-domain-containing-protein-[Lewinellaceae-bacterium]	2760807
WP_044217704.1	DndB-ParB-nuclease	GCF_000759025.1	420	Bacteria>FCB_group>Bacteroidetes/Chlorobi_group>Bacteroidetes>Saprospiria	Phaeodactylibacter_xiamenensis	DGQHR-domain-containing-protein-[Phaeodactylibacter-xiamenensis]	1524460
MBK6352111.1	DndB-ParB-nuclease	GCA_016705435.1	408	Bacteria>FCB_group>Bacteroidetes/Chlorobi_group>Bacteroidetes>Saprospiria	Saprospiraceae_bacterium	DGQHR-domain-containing-protein-[Saprospiraceae-bacterium]	2202734
MBK6572851.1	DndB-ParB-nuclease	GCA_016704015.1	368	Bacteria>FCB_group>Bacteroidetes/Chlorobi_group>Bacteroidetes>Saprospiria	Saprospiraceae_bacterium	DGQHR-domain-containing-protein-[Saprospiraceae-bacterium]	2202734
MBL7823164.1	DndB-ParB-nuclease	GCA_016787185.1	418	Bacteria>FCB_group>Bacteroidetes/Chlorobi_group>Bacteroidetes>Saprospiria	Saprospiraceae_bacterium	DGQHR-domain-containing-protein-[Saprospiraceae-bacterium]	2202734
MBP6235340.1	DndB-ParB-nuclease	GCA_017986015.1	441	Bacteria>FCB_group>Bacteroidetes/Chlorobi_group>Bacteroidetes>Saprospiria	Saprospiraceae_bacterium	DGQHR-domain-containing-protein-[Saprospiraceae-bacterium]	2202734
MCB0522400.1	DndB-ParB-nuclease	GCA_020436325.1	419	Bacteria>FCB_group>Bacteroidetes/Chlorobi_group>Bacteroidetes>Saprospiria	Saprospiraceae_bacterium	DGQHR-domain-containing-protein-[Saprospiraceae-bacterium]	2202734
MBK8405857.1	DndB-ParB-nuclease	GCA_016711565.1	179	Bacteria>FCB_group>Bacteroidetes/Chlorobi_group>Bacteroidetes>Saprospiria	Saprospiraceae_bacterium	hypothetical-protein-[Saprospiraceae-bacterium]	2202734
MBK8405858.1	DndB-ParB-nuclease	GCA_016711565.1	107	Bacteria>FCB_group>Bacteroidetes/Chlorobi_group>Bacteroidetes>Saprospiria	Saprospiraceae_bacterium	hypothetical-protein-[Saprospiraceae-bacterium]	2202734
WP_162431191.1	DndB-ParB-nuclease	GCF_010093045.1	396	Bacteria>FCB_group>Bacteroidetes/Chlorobi_group>Bacteroidetes>Sphingobacteriia	Mucilaginibacter_sp._14171R-50	DGQHR-domain-containing-protein-[Mucilaginibacter-sp.-14171R-50]	2703789
WP_090466122.1	DndB-ParB-nuclease	GCF_900103125.1	454	Bacteria>FCB_group>Bacteroidetes/Chlorobi_group>Bacteroidetes>Sphingobacteriia	Mucilaginibacter_sp._OK268	DGQHR-domain-containing-protein-[Mucilaginibacter-sp.-OK268]	1881048
WP_172335590.1	DndB-ParB-nuclease	GCF_013149275.1	354	Bacteria>FCB_group>Bacteroidetes/Chlorobi_group>Bacteroidetes>Sphingobacteriia	Mucilaginibacter_sp._SG564	DGQHR-domain-containing-protein-[Mucilaginibacter-sp.-SG564]	2587022
WP_228949118.1	DndB-ParB-nuclease	GCF_020880975.1	399	Bacteria>FCB_group>Bacteroidetes/Chlorobi_group>Bacteroidetes>Sphingobacteriia	Mucilaginibacter_sp._UR6-1	DGQHR-domain-containing-protein-[Mucilaginibacter-sp.-UR6-1]	1435643
WP_129875611.1	DndB-ParB-nuclease	GCF_004168255.1	467	Bacteria>FCB_group>Bacteroidetes/Chlorobi_group>Bacteroidetes>Sphingobacteriia	Mucilaginibacter_terrigena	DGQHR-domain-containing-protein-[Mucilaginibacter-terrigena]	2492395
WP_188753160.1	DndB-ParB-nuclease	GCF_014642075.1	455	Bacteria>FCB_group>Bacteroidetes/Chlorobi_group>Bacteroidetes>Sphingobacteriia	Parapedobacter_defluvii	DGQHR-domain-containing-protein-[Parapedobacter-defluvii]	2045106
WP_079717019.1	DndB-ParB-nuclease	GCF_900168055.1	404	Bacteria>FCB_group>Bacteroidetes/Chlorobi_group>Bacteroidetes>Sphingobacteriia	Parapedobacter_luteus	DGQHR-domain-containing-protein-[Parapedobacter-luteus]	623280
WP_079717023.1	DndB-ParB-nuclease	GCF_900168055.1	408	Bacteria>FCB_group>Bacteroidetes/Chlorobi_group>Bacteroidetes>Sphingobacteriia	Parapedobacter_luteus	DNA-phosphorothioation-associated-DGQHR-protein-1-[Parapedobacter-luteus]	623280
WP_182952648.1	DndB-ParB-nuclease	GCF_014172405.1	417	Bacteria>FCB_group>Bacteroidetes/Chlorobi_group>Bacteroidetes>Sphingobacteriia	Pedobacter_gandavensis	DGQHR-domain-containing-protein-[Pedobacter-gandavensis]	2679963
WP_138723723.1	DndB-ParB-nuclease	GCF_005925325.1	766	Bacteria>FCB_group>Bacteroidetes/Chlorobi_group>Bacteroidetes>Sphingobacteriia	Pedobacter_helvus	DGQHR-domain-containing-protein-[Pedobacter-helvus]	2563444
WP_131595917.1	DndB-ParB-nuclease	GCF_004331695.1	432	Bacteria>FCB_group>Bacteroidetes/Chlorobi_group>Bacteroidetes>Sphingobacteriia	Pedobacter_psychroterrae	DGQHR-domain-containing-protein-[Pedobacter-psychroterrae]	2530453

Accession	Annotation	GCA	Length	Phyletic.spread	Strain.name	Defline	Species.Taxid
WP_175474585.1	DndB-ParB-nuclease	GCF_900111155.1	441	Bacteria>FCB_group>Bacteroidetes/Chlorobi_group>Bacteroidetes>Sphingobacteriia	Pedobacter_rhizosphaerae	DGQHR-domain-containing-protein-[Pedobacter-rhizosphaerae]	390241
CAH0137643.1	DndB-ParB-nuclease	GCA_918698245.1	353	Bacteria>FCB_group>Bacteroidetes/Chlorobi_group>Bacteroidetes>Sphingobacteriia	Pedobacter_sp._Bi126	hypothetical-protein-SRABI126_00207-[Pedobacter-sp.-Bi126]	2822349
WP_193421891.1	DndB-ParB-nuclease	GCF_014947265.1	449	Bacteria>FCB_group>Bacteroidetes/Chlorobi_group>Bacteroidetes>Sphingobacteriia	Pedobacter_sp._MR2016-19	DGQHR-domain-containing-protein-[Pedobacter-sp.-MR2016-19]	2780089
WP_193421900.1	DndB-ParB-nuclease	GCF_014947265.1	575	Bacteria>FCB_group>Bacteroidetes/Chlorobi_group>Bacteroidetes>Sphingobacteriia	Pedobacter_sp._MR2016-19	DGQHR-domain-containing-protein-[Pedobacter-sp.-MR2016-19]	2780089
WP_090987199.1	DndB-ParB-nuclease	GCF_900111825.1	375	Bacteria>FCB_group>Bacteroidetes/Chlorobi_group>Bacteroidetes>Sphingobacteriia	Pedobacter_suwonensis	DGQHR-domain-containing-protein-[Pedobacter-suwonensis]	332999
WP_138727953.1	DndB-ParB-nuclease	GCF_005925345.1	410	Bacteria>FCB_group>Bacteroidetes/Chlorobi_group>Bacteroidetes>Sphingobacteriia	Pedobacter_ureilyticus	DGQHR-domain-containing-protein-[Pedobacter-ureilyticus]	1393051
OYZ01876.1	DndB-ParB-nuclease	GCA_002281575.1	412	Bacteria>FCB_group>Bacteroidetes/Chlorobi_group>Bacteroidetes>Sphingobacteriia	Sphingobacteriia_bacterium_28-36-52	hypothetical-protein-B7Y37_04580-[Sphingobacteriia-bacterium-28-36-52]	1970573
WP_105716847.1	DndB-ParB-nuclease	GCF_002980525.1	448	Bacteria>FCB_group>Bacteroidetes/Chlorobi_group>Bacteroidetes>Sphingobacteriia	Sphingobacterium_haloxyli	DGQHR-domain-containing-protein-[Sphingobacterium-haloxyli]	2100533
WP_103905135.1	DndB-ParB-nuclease	GCF_011046555.1	417	Bacteria>FCB_group>Bacteroidetes/Chlorobi_group>Bacteroidetes>Sphingobacteriia	Sphingobacterium_lactis	DGQHR-domain-containing-protein-[Sphingobacterium-lactis]	797291
WP_148919008.1	DndB-ParB-nuclease	GCF_011043555.1	411	Bacteria>FCB_group>Bacteroidetes/Chlorobi_group>Bacteroidetes>Sphingobacteriia	Sphingobacterium	MULTISPECIES:-DGQHR-domain-containing-protein-[Sphingobacterium]	28453
WP_079640692.1	DndB-ParB-nuclease	GCF_900168125.1	374	Bacteria>FCB_group>Bacteroidetes/Chlorobi_group>Bacteroidetes>Sphingobacteriia	Sphingobacterium_nematocida	DGQHR-domain-containing-protein-[Sphingobacterium-nematocida]	1513896
WP_182498398.1	DndB-ParB-nuclease	GCF_014644335.1	520	Bacteria>FCB_group>Bacteroidetes/Chlorobi_group>Bacteroidetes>Sphingobacteriia	Sphingobacterium_soli	DNA-phosphorothioation-associated-DGQHR-protein-1-[Sphingobacterium-soli]	1914757
WP_066751086.1	DndB-ParB-nuclease	GCF_001586775.1	409	Bacteria>FCB_group>Bacteroidetes/Chlorobi_group>Bacteroidetes>Sphingobacteriia	Sphingobacterium_sp._CFCC_11742	DGQHR-domain-containing-protein-[Sphingobacterium-sp.-CFCC-11742]	1775560
WP_230144108.1	DndB-ParB-nuclease	GCF_918697995.1	353	Bacteria>FCB_group>Bacteroidetes/Chlorobi_group>Bacteroidetes>Sphingobacteriia	unclassified_Pedobacter	MULTISPECIES:-DGQHR-domain-containing-protein-[unclassified-Pedobacter]	2628915
MBK6338773.1	DndB-ParB-nuclease	GCA_016705125.1	407	Bacteria>FCB_group>Bacteroidetes/Chlorobi_group>Bacteroidetes>unclassified_Bacteroidetes	Bacteroidetes_bacterium	DGQHR-domain-containing-protein-[Bacteroidetes-bacterium]	1898104
MBK7908783.1	DndB-ParB-nuclease	GCA_016713765.1	414	Bacteria>FCB_group>Bacteroidetes/Chlorobi_group>Bacteroidetes>unclassified_Bacteroidetes	Bacteroidetes_bacterium	DGQHR-domain-containing-protein-[Bacteroidetes-bacterium]	1898104
MBL0080068.1	DndB-ParB-nuclease	GCA_016721275.1	329	Bacteria>FCB_group>Bacteroidetes/Chlorobi_group>Bacteroidetes>unclassified_Bacteroidetes	Bacteroidetes_bacterium	DGQHR-domain-containing-protein-[Bacteroidetes-bacterium]	1898104
MBL0310166.1	DndB-ParB-nuclease	GCA_016722375.1	413	Bacteria>FCB_group>Bacteroidetes/Chlorobi_group>Bacteroidetes>unclassified_Bacteroidetes	Bacteroidetes_bacterium	DGQHR-domain-containing-protein-[Bacteroidetes-bacterium]	1898104
OFY07329.1	DndB-ParB-nuclease	GCA_001769115.1	406	Bacteria>FCB_group>Bacteroidetes/Chlorobi_group>Bacteroidetes>unclassified_Bacteroidetes	Bacteroidetes_bacterium_GWE2_42_42	hypothetical-protein-A2W94_11620-[Bacteroidetes-bacterium-GWE2_42_42]	1797339
MBV5329175.1	DndB-ParB-nuclease	GCA_019162945.1	105	Bacteria>FCB_group>Bacteroidetes/Chlorobi_group>Chlorobi>Chlorobia	Chlorobium_sp.	DGQHR-domain-containing-protein-[Chlorobium-sp.]	1095
MBV5330006.1	DndB-ParB-nuclease	GCA_019162945.1	364	Bacteria>FCB_group>Bacteroidetes/Chlorobi_group>Chlorobi>Chlorobia	Chlorobium_sp.	DNA-sulfur-modification-protein-DndB-[Chlorobium-sp.]	1095
MBV5329173.1	DndB-ParB-nuclease	GCA_019162945.1	69	Bacteria>FCB_group>Bacteroidetes/Chlorobi_group>Chlorobi>Chlorobia	Chlorobium_sp.	hypothetical-protein-[Chlorobium-sp.]	1095
MBV5329174.1	DndB-ParB-nuclease	GCA_019162945.1	105	Bacteria>FCB_group>Bacteroidetes/Chlorobi_group>Chlorobi>Chlorobia	Chlorobium_sp.	hypothetical-protein-[Chlorobium-sp.]	1095
NPA44854.1	DndB-ParB-nuclease	GCA_013153575.1	446	Bacteria>FCB_group>Bacteroidetes/Chlorobi_group>Chlorobi>unclassified_Chlorobi	Chlorobi_bacterium	DGQHR-domain-containing-protein-[Chlorobi-bacterium]	2268192
MBN8544908.1	DndB-ParB-nuclease	GCA_017302895.1	460	Bacteria>FCB_group>Bacteroidetes/Chlorobi_group>Ignavibacteriae>Ignavibacteria	Ignavibacteria_bacterium	DGQHR-domain-containing-protein-[Ignavibacteria-bacterium]	2053306
MBW7841611.1	DndB-ParB-nuclease	GCA_019455015.1	585	Bacteria>FCB_group>Bacteroidetes/Chlorobi_group>Ignavibacteriae>Ignavibacteria	Ignavibacterium_sp.	DGQHR-domain-containing-protein-[Ignavibacterium-sp.]	2651167
MBL7648114.1	DndB-ParB-nuclease	GCA_016791475.1	403	Bacteria>FCB_group>Candidatus_Hydrogenedentes>unclassified_Candidatus_Hydrogenedentes	Candidatus_Hydrogenedentes_bacterium	DGQHR-domain-containing-protein-[Candidatus-Hydrogenedentes-bacterium]	2030809
MYB66987.1	DndB-ParB-nuclease	GCA_009840005.1	418	Bacteria>FCB_group>Gemmatimonadetes>unclassified_Gemmatimonadetes	Gemmatimonadetes_bacterium	DGQHR-domain-containing-protein-[Gemmatimonadetes-bacterium]	2026742
MYB71139.1	DndB-ParB-nuclease	GCA_009840005.1	451	Bacteria>FCB_group>Gemmatimonadetes>unclassified_Gemmatimonadetes	Gemmatimonadetes_bacterium	DGQHR-domain-containing-protein-[Gemmatimonadetes-bacterium]	2026742
MBN2837854.1	DndB-ParB-nuclease	GCA_016938425.1	352	Bacteria>Fusobacteria>Fusobacteriia>Fusobacteriales>Fusobacteriaceae	Fusobacteriaceae_bacterium	DNA-sulfur-modification-protein-DndB-[Fusobacteriaceae-bacterium]	2053555
MBC8285044.1	DndB-ParB-nuclease	GCA_014382485.1	343	Bacteria>Nitrospinae/Tectomicrobia_group>Nitrospinae>unclassified_Nitrospinae	Nitrospinae_bacterium	DNA-sulfur-modification-protein-DndB-[Nitrospinae-bacterium]	2026769
WP_168058386.1	DndB-ParB-nuclease	GCF_012184425.1	414	Bacteria>Nitrospirae>Nitrospira>Candidatus_Troglogloeales>Candidatus_Manganitrophaceae	Candidatus_Manganitrophus_noduliformans	DGQHR-domain-containing-protein-[Candidatus-Manganitrophus-noduliformans]	2606439
WP_168058389.1	DndB-ParB-nuclease	GCF_012184425.1	371	Bacteria>Nitrospirae>Nitrospira>Candidatus_Troglogloeales>Candidatus_Manganitrophaceae	Candidatus_Manganitrophus_noduliformans	DNA-sulfur-modification-protein-DndB-[Candidatus-Manganitrophus-noduliformans]	2606439
MBF0555462.1	DndB-ParB-nuclease	GCA_015233485.1	427	Bacteria>Nitrospirae>unclassified_Nitrospirae	Nitrospirae_bacterium	DGQHR-domain-containing-protein-[Nitrospirae-bacterium]	2026887
MBE7562008.1	DndB-ParB-nuclease	GCA_015100135.1	376	Bacteria>Proteobacteria>Acidithiobacillia>Acidithiobacillales>Acidithiobacillaceae	Acidithiobacillus_sp._HP-6	DNA-sulfur-modification-protein-DndB-[Acidithiobacillus-sp.-HP-6]	2697655
WP_193649872.1	DndB-ParB-nuclease	GCF_015100135.1	376	Bacteria>Proteobacteria>Acidithiobacillia>Acidithiobacillales>Acidithiobacillaceae	unclassified_Acidithiobacillus	MULTISPECIES:-DNA-sulfur-modification-protein-DndB-[unclassified-Acidithiobacillus]	2614800
QTC90329.1	DndB-ParB-nuclease	GCA_017487405.1	416	Bacteria>Proteobacteria>Alphaproteobacteria>Caulobacterales>Caulobacteraceae	Brevundimonas_goettingensis	DGQHR-domain-containing-protein-[Brevundimonas-goettingensis]	2774190
WP_207868747.1	DndB-ParB-nuclease	GCF_017487405.1	416	Bacteria>Proteobacteria>Alphaproteobacteria>Caulobacterales>Caulobacteraceae	Brevundimonas_goettingensis	DGQHR-domain-containing-protein-[Brevundimonas-goettingensis]	2774190
QTC90325.1	DndB-ParB-nuclease	GCA_017487405.1	369	Bacteria>Proteobacteria>Alphaproteobacteria>Caulobacterales>Caulobacteraceae	Brevundimonas_goettingensis	DNA-sulfur-modification-protein-DndB-[Brevundimonas-goettingensis]	2774190
WP_207868744.1	DndB-ParB-nuclease	GCF_017487405.1	369	Bacteria>Proteobacteria>Alphaproteobacteria>Caulobacterales>Caulobacteraceae	Brevundimonas_goettingensis	DNA-sulfur-modification-protein-DndB-[Brevundimonas-goettingensis]	2774190
WP_183214009.1	DndB-ParB-nuclease	GCF_014199945.1	356	Bacteria>Proteobacteria>Alphaproteobacteria>Caulobacterales>Caulobacteraceae	Brevundimonas_variabilis	DNA-sulfur-modification-protein-DndB-[Brevundimonas-variabilis]	74312
WP_183214001.1	DndB-ParB-nuclease	GCF_014199945.1	606	Bacteria>Proteobacteria>Alphaproteobacteria>Caulobacterales>Caulobacteraceae	Brevundimonas_variabilis	hypothetical-protein-[Brevundimonas-variabilis]	74312
WP_007665267.1	DndB-ParB-nuclease	GCF_000281955.1	363	Bacteria>Proteobacteria>Alphaproteobacteria>Caulobacterales>Caulobacteraceae	Caulobacter_sp._AP07	DNA-sulfur-modification-protein-DndB-[Caulobacter-sp.-AP07]	1144304
OHB26292.1	DndB-ParB-nuclease	GCA_001824475.1	364	Bacteria>Proteobacteria>Alphaproteobacteria>Caulobacterales>Caulobacteraceae	Phenylobacterium_sp._RIFCSPHIGHO2_01_FULL_69_31	DNA-sulfur-modification-protein-DndB-[Phenylobacterium-sp.-RIFCSPHIGHO2_01_FULL_69_31]	1801944
OHB26274.1	DndB-ParB-nuclease	GCA_001824475.1	424	Bacteria>Proteobacteria>Alphaproteobacteria>Caulobacterales>Caulobacteraceae	Phenylobacterium_sp._RIFCSPHIGHO2_01_FULL_69_31	MAG:-DNA-phosphorothioation-associated-DGQHR-protein-1-[Phenylobacterium-sp.-RIFCSPHIGHO2_01_FULL_69_31]	1801944
MAP18045.1	DndB-ParB-nuclease	GCA_002703425.1	389	Bacteria>Proteobacteria>Alphaproteobacteria>Hyphomicrobiales>Aurantimonadaceae	Aurantimonas_sp.	hypothetical-protein-[Aurantimonas-sp.]	1872654
SDO86079.1	DndB-ParB-nuclease	GCA_900104035.1	403	Bacteria>Proteobacteria>Alphaproteobacteria>Hyphomicrobiales>Aurantimonadaceae	Aureimonas_jatrophae	DNA-phosphorothioation-associated-DGQHR-protein-1-[Aureimonas-jatrophae]	1166073
WP_082652778.1	DndB-ParB-nuclease	GCF_001463705.1	402	Bacteria>Proteobacteria>Alphaproteobacteria>Hyphomicrobiales>Aurantimonadaceae	Aureimonas_sp._AU12	DNA-phosphorothioation-associated-DGQHR-protein-1-[Aureimonas-sp.-AU12]	1638161
WP_117412162.1	DndB-ParB-nuclease	GCF_003432385.1	411	Bacteria>Proteobacteria>Alphaproteobacteria>Hyphomicrobiales>Breoghaniaceae	Breoghania_sp._L-A4	DNA-phosphorothioation-associated-DGQHR-protein-1-[Breoghania-sp.-L-A4]	2304600
WP_064322769.1	DndB-ParB-nuclease	GCF_001652485.1	426	Bacteria>Proteobacteria>Alphaproteobacteria>Hyphomicrobiales>Brucellaceae	Brucella_pseudogrignonensis	DGQHR-domain-containing-protein-[Brucella-pseudogrignonensis]	419475
WP_237853938.1	DndB-ParB-nuclease	GCF_022024335.1	419	Bacteria>Proteobacteria>Alphaproteobacteria>Hyphomicrobiales>Brucellaceae	Brucella_pseudogrignonensis	DGQHR-domain-containing-protein-[Brucella-pseudogrignonensis]	419475
WP_121539131.1	DndB-ParB-nuclease	GCF_003664555.1	426	Bacteria>Proteobacteria>Alphaproteobacteria>Hyphomicrobiales>Brucellaceae	[Ochrobactrum]_soli	DGQHR-domain-containing-protein-[[Ochrobactrum]	2448455
WP_157290577.1	DndB-ParB-nuclease	GCF_009758415.1	419	Bacteria>Proteobacteria>Alphaproteobacteria>Hyphomicrobiales>Devosiaceae	Devosia_marina	DGQHR-domain-containing-protein-[Devosia-marina]	2683198
MBL8599942.1	DndB-ParB-nuclease	GCA_016793625.1	423	Bacteria>Proteobacteria>Alphaproteobacteria>Hyphomicrobiales>Devosiaceae	Devosia_sp.	hypothetical-protein-[Devosia-sp.]	1871048
WP_088343787.1	DndB-ParB-nuclease	GCF_002198685.1	365	Bacteria>Proteobacteria>Alphaproteobacteria>Hyphomicrobiales>Hyphomicrobiaceae	Rhodomicrobium	MULTISPECIES:-DNA-sulfur-modification-protein-DndB-[Rhodomicrobium]	1068
CAA2136838.1	DndB-ParB-nuclease	-	368	Bacteria>Proteobacteria>Alphaproteobacteria>Hyphomicrobiales>Methylobacteriaceae	Methylobacterium_bullatum	hypothetical-protein-MBLL_00362-[Methylobacterium-bullatum]	570505
WP_058619947.1	DndB-ParB-nuclease	GCF_001476615.1	368	Bacteria>Proteobacteria>Alphaproteobacteria>Hyphomicrobiales>Methylobacteriaceae	Methylobacterium_indicum	DNA-sulfur-modification-protein-DndB-[Methylobacterium-indicum]	1775910
WP_010682099.1	DndB-ParB-nuclease	GCF_000364445.2	420	Bacteria>Proteobacteria>Alphaproteobacteria>Hyphomicrobiales>Methylobacteriaceae	Methylobacterium_mesophilicum	DGQHR-domain-containing-protein-[Methylobacterium-mesophilicum]	39956
WP_127729008.1	DndB-ParB-nuclease	GCF_004004555.2	369	Bacteria>Proteobacteria>Alphaproteobacteria>Hyphomicrobiales>Methylobacteriaceae	Methylobacterium_oryzihabitans	DNA-sulfur-modification-protein-DndB-[Methylobacterium-sp.-TER-1]	2499852
WP_093567321.1	DndB-ParB-nuclease	GCF_900116815.1	437	Bacteria>Proteobacteria>Alphaproteobacteria>Hyphomicrobiales>Methylobacteriaceae	Methylobacterium_sp._174MFSha1.1	DGQHR-domain-containing-protein-[Methylobacterium-sp.-174MFSha1.1]	1502749
SFU78099.1	DndB-ParB-nuclease	GCA_900116815.1	437	Bacteria>Proteobacteria>Alphaproteobacteria>Hyphomicrobiales>Methylobacteriaceae	Methylobacterium_sp._174MFSha1.1	DNA-sulfur-modification-protein-DndB-[Methylobacterium-sp.-174MFSha1.1]	1502749
MBP33150.1	DndB-ParB-nuclease	GCA_002727035.1	368	Bacteria>Proteobacteria>Alphaproteobacteria>Hyphomicrobiales>Methylobacteriaceae	Methylobacterium_sp.	DNA-sulfur-modification-protein-DndB-[Methylobacterium-sp.]	409
KQQ17379.1	DndB-ParB-nuclease	GCA_001423385.1	417	Bacteria>Proteobacteria>Alphaproteobacteria>Hyphomicrobiales>Methylobacteriaceae	Methylobacterium_sp._Leaf121	hypothetical-protein-ASF59_00090-[Methylobacterium-sp.-Leaf121]	1736262
KQQ13665.1	DndB-ParB-nuclease	GCA_001423405.1	368	Bacteria>Proteobacteria>Alphaproteobacteria>Hyphomicrobiales>Methylobacteriaceae	Methylobacterium_sp._Leaf123	DNA-sulfur-modification-protein-DndB-[Methylobacterium-sp.-Leaf123]	1736264
WP_238508663.1	DndB-ParB-nuclease	GCF_022214735.1	368	Bacteria>Proteobacteria>Alphaproteobacteria>Hyphomicrobiales>Methylobacteriaceae	Methylorubrum_extorquens	DNA-sulfur-modification-protein-DndB-[Methylorubrum-extorquens]	408
WP_208073789.1	DndB-ParB-nuclease	-	366	Bacteria>Proteobacteria>Alphaproteobacteria>Hyphomicrobiales>Nitrobacteraceae	Bradyrhizobium_sp._38S5	DNA-sulfur-modification-protein-DndB-[Bradyrhizobium-sp.-38S5]	2819348
MBR1285374.1	DndB-ParB-nuclease	GCA_018130125.1	367	Bacteria>Proteobacteria>Alphaproteobacteria>Hyphomicrobiales>Nitrobacteraceae	Bradyrhizobium_sp._AUGA_SZCCT0177	DNA-sulfur-modification-protein-DndB-[Bradyrhizobium-sp.-AUGA-SZCCT0177]	2807665
WP_212267798.1	DndB-ParB-nuclease	-	367	Bacteria>Proteobacteria>Alphaproteobacteria>Hyphomicrobiales>Nitrobacteraceae	Bradyrhizobium_sp._AUGA_SZCCT0177	DNA-sulfur-modification-protein-DndB-[Bradyrhizobium-sp.-AUGA-SZCCT0177]	2807665
WP_247356735.1	DndB-ParB-nuclease	GCF_023100905.1	418	Bacteria>Proteobacteria>Alphaproteobacteria>Hyphomicrobiales>Nitrobacteraceae	unclassified_Bradyrhizobium	MULTISPECIES:-DGQHR-domain-containing-protein-[unclassified-Bradyrhizobium]	2631580
WP_247356724.1	DndB-ParB-nuclease	GCF_023100905.1	373	Bacteria>Proteobacteria>Alphaproteobacteria>Hyphomicrobiales>Nitrobacteraceae	unclassified_Bradyrhizobium	MULTISPECIES:-DNA-sulfur-modification-protein-DndB-[unclassified-Bradyrhizobium]	2631580
WP_011579000.1	DndB-ParB-nuclease	GCF_011317445.1	370	Bacteria>Proteobacteria>Alphaproteobacteria>Hyphomicrobiales>Phyllobacteriaceae	Chelativorans	MULTISPECIES:-DNA-sulfur-modification-protein-DndB-[Chelativorans]	449972
WP_040583532.1	DndB-ParB-nuclease	GCF_000233995.1	405	Bacteria>Proteobacteria>Alphaproteobacteria>Hyphomicrobiales>Phyllobacteriaceae	Mesorhizobium_amorphae	DNA-phosphorothioation-associated-DGQHR-protein-1-[Mesorhizobium-amorphae]	71433
WP_176480232.1	DndB-ParB-nuclease	GCF_002294705.1	414	Bacteria>Proteobacteria>Alphaproteobacteria>Hyphomicrobiales>Phyllobacteriaceae	Mesorhizobium	MULTISPECIES:-DNA-phosphorothioation-associated-DGQHR-protein-1-[Mesorhizobium]	68287
WP_095795933.1	DndB-ParB-nuclease	GCF_002294705.1	369	Bacteria>Proteobacteria>Alphaproteobacteria>Hyphomicrobiales>Phyllobacteriaceae	Mesorhizobium	MULTISPECIES:-DNA-sulfur-modification-protein-DndB-[Mesorhizobium]	68287
WP_141841112.1	DndB-ParB-nuclease	-	200	Bacteria>Proteobacteria>Alphaproteobacteria>Hyphomicrobiales>Phyllobacteriaceae	Mesorhizobium_sp._B2-4-15	DGQHR-domain-containing-protein-[Mesorhizobium-sp.-B2-4-15]	2589934
WP_141841100.1	DndB-ParB-nuclease	GCF_006658505.1	415	Bacteria>Proteobacteria>Alphaproteobacteria>Hyphomicrobiales>Phyllobacteriaceae	Mesorhizobium_sp._B2-4-15	DNA-phosphorothioation-associated-DGQHR-protein-1-[Mesorhizobium-sp.-B2-4-15]	2589934
WP_141841102.1	DndB-ParB-nuclease	GCF_006658505.1	370	Bacteria>Proteobacteria>Alphaproteobacteria>Hyphomicrobiales>Phyllobacteriaceae	Mesorhizobium_sp._B2-4-15	DNA-sulfur-modification-protein-DndB-[Mesorhizobium-sp.-B2-4-15]	2589934
WP_119819822.1	DndB-ParB-nuclease	GCF_003590645.1	411	Bacteria>Proteobacteria>Alphaproteobacteria>Hyphomicrobiales>Phyllobacteriaceae	Mesorhizobium_sp._DCY119	DGQHR-domain-containing-protein-[Mesorhizobium-sp.-DCY119]	2108445
WP_119819813.1	DndB-ParB-nuclease	GCF_003590645.1	370	Bacteria>Proteobacteria>Alphaproteobacteria>Hyphomicrobiales>Phyllobacteriaceae	Mesorhizobium_sp._DCY119	DNA-sulfur-modification-protein-DndB-[Mesorhizobium-sp.-DCY119]	2108445
TIM38464.1	DndB-ParB-nuclease	GCA_004961945.1	407	Bacteria>Proteobacteria>Alphaproteobacteria>Hyphomicrobiales>Phyllobacteriaceae	Mesorhizobium_sp.	DGQHR-domain-containing-protein-[Mesorhizobium-sp.]	1871066
RWL89315.1	DndB-ParB-nuclease	GCA_004020305.1	370	Bacteria>Proteobacteria>Alphaproteobacteria>Hyphomicrobiales>Phyllobacteriaceae	Mesorhizobium_sp.	DNA-sulfur-modification-protein-DndB-[Mesorhizobium-sp.]	1871066
RWN94626.1	DndB-ParB-nuclease	GCA_004021525.1	390	Bacteria>Proteobacteria>Alphaproteobacteria>Hyphomicrobiales>Phyllobacteriaceae	Mesorhizobium_sp.	DNA-sulfur-modification-protein-DndB-[Mesorhizobium-sp.]	1871066
RWO22207.1	DndB-ParB-nuclease	GCA_004021465.1	370	Bacteria>Proteobacteria>Alphaproteobacteria>Hyphomicrobiales>Phyllobacteriaceae	Mesorhizobium_sp.	DNA-sulfur-modification-protein-DndB-[Mesorhizobium-sp.]	1871066
RWQ46782.1	DndB-ParB-nuclease	GCA_004021735.1	370	Bacteria>Proteobacteria>Alphaproteobacteria>Hyphomicrobiales>Phyllobacteriaceae	Mesorhizobium_sp.	DNA-sulfur-modification-protein-DndB-[Mesorhizobium-sp.]	1871066
TIN32544.1	DndB-ParB-nuclease	GCA_004961445.1	390	Bacteria>Proteobacteria>Alphaproteobacteria>Hyphomicrobiales>Phyllobacteriaceae	Mesorhizobium_sp.	DNA-sulfur-modification-protein-DndB-[Mesorhizobium-sp.]	1871066
TIP92999.1	DndB-ParB-nuclease	GCA_004962305.1	390	Bacteria>Proteobacteria>Alphaproteobacteria>Hyphomicrobiales>Phyllobacteriaceae	Mesorhizobium_sp.	DNA-sulfur-modification-protein-DndB-[Mesorhizobium-sp.]	1871066
WP_071071738.1	DndB-ParB-nuclease	-	360	Bacteria>Proteobacteria>Alphaproteobacteria>Hyphomicrobiales>Phyllobacteriaceae	Mesorhizobium_sp._LCM_4577	DNA-sulfur-modification-protein-DndB-[Mesorhizobium-sp.-LCM-4577]	1848288
WP_071071671.1	DndB-ParB-nuclease	GCF_001854795.1	565	Bacteria>Proteobacteria>Alphaproteobacteria>Hyphomicrobiales>Phyllobacteriaceae	Mesorhizobium_sp._LCM_4577	hypothetical-protein-[Mesorhizobium-sp.-LCM-4577]	1848288
WP_127281912.1	DndB-ParB-nuclease	GCF_003998955.1	417	Bacteria>Proteobacteria>Alphaproteobacteria>Hyphomicrobiales>Phyllobacteriaceae	Mesorhizobium_sp._M4B.F.Ca.ET.017.02.2.1	DGQHR-domain-containing-protein-[Mesorhizobium-sp.-M4B.F.Ca.ET.017.02.2.1]	2496649
WP_027168794.1	DndB-ParB-nuclease	GCF_000427725.1	424	Bacteria>Proteobacteria>Alphaproteobacteria>Hyphomicrobiales>Phyllobacteriaceae	Mesorhizobium_sp._WSM3224	DGQHR-domain-containing-protein-[Mesorhizobium-sp.-WSM3224]	1040986
WP_198021713.1	DndB-ParB-nuclease	GCF_000427725.1	347	Bacteria>Proteobacteria>Alphaproteobacteria>Hyphomicrobiales>Phyllobacteriaceae	Mesorhizobium_sp._WSM3224	DNA-sulfur-modification-protein-DndB-[Mesorhizobium-sp.-WSM3224]	1040986
WP_095517570.1	DndB-ParB-nuclease	-	415	Bacteria>Proteobacteria>Alphaproteobacteria>Hyphomicrobiales>Phyllobacteriaceae	Mesorhizobium_wenxiniae	DGQHR-domain-containing-protein-[Mesorhizobium-wenxiniae]	2014805
WP_175276880.1	DndB-ParB-nuclease	GCF_013358405.1	412	Bacteria>Proteobacteria>Alphaproteobacteria>Hyphomicrobiales>Phyllobacteriaceae	Oricola_thermophila	DNA-phosphorothioation-associated-DGQHR-protein-1-[Oricola-thermophila]	2742145
WP_084631742.1	DndB-ParB-nuclease	GCF_005145045.1	363	Bacteria>Proteobacteria>Alphaproteobacteria>Hyphomicrobiales>Rhizobiaceae	Agrobacterium_larrymoorei	DNA-sulfur-modification-protein-DndB-[Agrobacterium-larrymoorei]	160699
WP_027676401.1	DndB-ParB-nuclease	GCF_005145045.1	566	Bacteria>Proteobacteria>Alphaproteobacteria>Hyphomicrobiales>Rhizobiaceae	Agrobacterium_larrymoorei	hypothetical-protein-[Agrobacterium-larrymoorei]	160699
WP_059755023.1	DndB-ParB-nuclease	GCF_001526505.1	370	Bacteria>Proteobacteria>Alphaproteobacteria>Hyphomicrobiales>Rhizobiaceae	Agrobacterium	MULTISPECIES:-DNA-sulfur-modification-protein-DndB-[Agrobacterium]	357
WP_019566305.1	DndB-ParB-nuclease	GCF_000381165.1	415	Bacteria>Proteobacteria>Alphaproteobacteria>Hyphomicrobiales>Rhizobiaceae	Agrobacterium_sp._10MFCol1.1	DNA-phosphorothioation-associated-DGQHR-protein-1-[Agrobacterium-sp.-10MFCol1.1]	1150775
WP_019566303.1	DndB-ParB-nuclease	GCF_000381165.1	370	Bacteria>Proteobacteria>Alphaproteobacteria>Hyphomicrobiales>Rhizobiaceae	Agrobacterium_sp._10MFCol1.1	DNA-sulfur-modification-protein-DndB-[Agrobacterium-sp.-10MFCol1.1]	1150775
KVK54339.1	DndB-ParB-nuclease	GCA_001526505.1	370	Bacteria>Proteobacteria>Alphaproteobacteria>Hyphomicrobiales>Rhizobiaceae	Agrobacterium_sp._D14	DNA-sulfur-modification-protein-DndB-[Agrobacterium-sp.-D14]	1336743
WP_236775614.1	DndB-ParB-nuclease	GCF_013317565.1	375	Bacteria>Proteobacteria>Alphaproteobacteria>Hyphomicrobiales>Rhizobiaceae	Agrobacterium_tumefaciens	DGQHR-domain-containing-protein-[Agrobacterium-tumefaciens]	358
AMD60414.1	DndB-ParB-nuclease	GCA_001551895.1	411	Bacteria>Proteobacteria>Alphaproteobacteria>Hyphomicrobiales>Rhizobiaceae	Agrobacterium_tumefaciens	DNA-phosphorothioation-associated-DGQHR-protein-1-[Agrobacterium-tumefaciens]	358
WP_156584792.1	DndB-ParB-nuclease	GCF_009743995.1	406	Bacteria>Proteobacteria>Alphaproteobacteria>Hyphomicrobiales>Rhizobiaceae	Agrobacterium_vitis	DNA-phosphorothioation-associated-DGQHR-protein-1-[Agrobacterium-vitis]	373
MAM13633.1	DndB-ParB-nuclease	GCA_002695375.1	418	Bacteria>Proteobacteria>Alphaproteobacteria>Hyphomicrobiales>Rhizobiaceae	Rhizobiaceae_bacterium	hypothetical-protein-[Rhizobiaceae-bacterium]	1913961
WP_004679900.1	DndB-ParB-nuclease	GCF_000194195.1	416	Bacteria>Proteobacteria>Alphaproteobacteria>Hyphomicrobiales>Rhizobiaceae	Rhizobium_etli	MULTISPECIES:-DNA-phosphorothioation-associated-DGQHR-protein-1-[Rhizobium]	29449
WP_004679898.1	DndB-ParB-nuclease	GCF_000194195.1	370	Bacteria>Proteobacteria>Alphaproteobacteria>Hyphomicrobiales>Rhizobiaceae	Rhizobium_etli	MULTISPECIES:-DNA-sulfur-modification-protein-DndB-[Rhizobium]	29449
WP_130787517.1	DndB-ParB-nuclease	GCF_004306655.1	425	Bacteria>Proteobacteria>Alphaproteobacteria>Hyphomicrobiales>Rhizobiaceae	Rhizobium_leguminosarum	DGQHR-domain-containing-protein-[Rhizobium-leguminosarum]	384
WP_168266315.1	DndB-ParB-nuclease	-	442	Bacteria>Proteobacteria>Alphaproteobacteria>Hyphomicrobiales>Rhizobiaceae	Rhizobium_leguminosarum	DGQHR-domain-containing-protein-[Rhizobium-leguminosarum]	384
WP_025398079.1	DndB-ParB-nuclease	GCF_000517605.1	417	Bacteria>Proteobacteria>Alphaproteobacteria>Hyphomicrobiales>Rhizobiaceae	Rhizobium_leguminosarum	DNA-phosphorothioation-associated-DGQHR-protein-1-[Rhizobium-leguminosarum]	384
WP_131702494.1	DndB-ParB-nuclease	GCF_019778265.1	417	Bacteria>Proteobacteria>Alphaproteobacteria>Hyphomicrobiales>Rhizobiaceae	Rhizobium_leguminosarum	DNA-phosphorothioation-associated-DGQHR-protein-1-[Rhizobium-leguminosarum]	384
WP_025398077.1	DndB-ParB-nuclease	GCF_000517605.1	370	Bacteria>Proteobacteria>Alphaproteobacteria>Hyphomicrobiales>Rhizobiaceae	Rhizobium_leguminosarum	DNA-sulfur-modification-protein-DndB-[Rhizobium-leguminosarum]	384
WP_130787525.1	DndB-ParB-nuclease	GCF_004306655.1	370	Bacteria>Proteobacteria>Alphaproteobacteria>Hyphomicrobiales>Rhizobiaceae	Rhizobium_leguminosarum	DNA-sulfur-modification-protein-DndB-[Rhizobium-leguminosarum]	384
WP_131702495.1	DndB-ParB-nuclease	GCF_019778265.1	370	Bacteria>Proteobacteria>Alphaproteobacteria>Hyphomicrobiales>Rhizobiaceae	Rhizobium_leguminosarum	DNA-sulfur-modification-protein-DndB-[Rhizobium-leguminosarum]	384
WP_155951299.1	DndB-ParB-nuclease	GCF_000427925.1	411	Bacteria>Proteobacteria>Alphaproteobacteria>Hyphomicrobiales>Rhizobiaceae	Rhizobium_mesoamericanum	hypothetical-protein-[Rhizobium-mesoamericanum]	1079800
WP_081687995.1	DndB-ParB-nuclease	GCF_000427925.1	361	Bacteria>Proteobacteria>Alphaproteobacteria>Hyphomicrobiales>Rhizobiaceae	Rhizobium	MULTISPECIES:-DNA-sulfur-modification-protein-DndB-[Rhizobium]	379
WP_064823682.1	DndB-ParB-nuclease	GCF_001664285.1	406	Bacteria>Proteobacteria>Alphaproteobacteria>Hyphomicrobiales>Rhizobiaceae	Rhizobium_phaseoli	DNA-phosphorothioation-associated-DGQHR-protein-1-[Rhizobium-phaseoli]	396
WP_209914635.1	DndB-ParB-nuclease	GCF_017877395.1	428	Bacteria>Proteobacteria>Alphaproteobacteria>Hyphomicrobiales>Rhizobiaceae	Rhizobium_pusense	DGQHR-domain-containing-protein-[Rhizobium-pusense]	648995
MBP2614110.1	DndB-ParB-nuclease	GCA_017877395.1	370	Bacteria>Proteobacteria>Alphaproteobacteria>Hyphomicrobiales>Rhizobiaceae	Rhizobium_pusense	DNA-sulfur-modification-protein-DndB-[Rhizobium-pusense]	648995
MBP2614112.1	DndB-ParB-nuclease	GCA_017877395.1	428	Bacteria>Proteobacteria>Alphaproteobacteria>Hyphomicrobiales>Rhizobiaceae	Rhizobium_pusense	DNA-sulfur-modification-protein-DndB-[Rhizobium-pusense]	648995
WP_209914629.1	DndB-ParB-nuclease	GCF_017877395.1	370	Bacteria>Proteobacteria>Alphaproteobacteria>Hyphomicrobiales>Rhizobiaceae	Rhizobium_pusense	DNA-sulfur-modification-protein-DndB-[Rhizobium-pusense]	648995
SOD50250.1	DndB-ParB-nuclease	GCA_900215635.1	360	Bacteria>Proteobacteria>Alphaproteobacteria>Hyphomicrobiales>Rhizobiaceae	Rhizobium_sp._AN6A	DNA-sulfur-modification-protein-DndB-[Rhizobium-sp.-AN6A]	1841611
SOD50266.1	DndB-ParB-nuclease	GCA_900215635.1	417	Bacteria>Proteobacteria>Alphaproteobacteria>Hyphomicrobiales>Rhizobiaceae	Rhizobium_sp._AN6A	DNA-sulfur-modification-protein-DndB-[Rhizobium-sp.-AN6A]	1841611
TKV70611.1	DndB-ParB-nuclease	GCA_005298145.1	369	Bacteria>Proteobacteria>Alphaproteobacteria>Hyphomicrobiales>Rhizobiaceae	Rhizobium_sp._AU243	DNA-sulfur-modification-protein-DndB-[Rhizobium-sp.-AU243]	2303425
WP_085780248.1	DndB-ParB-nuclease	GCF_002117485.1	428	Bacteria>Proteobacteria>Alphaproteobacteria>Hyphomicrobiales>Rhizobiaceae	Rhizobium_sp._NXC14	DGQHR-domain-containing-protein-[Rhizobium-sp.-NXC14]	1981173
WP_085780250.1	DndB-ParB-nuclease	GCF_002117485.1	370	Bacteria>Proteobacteria>Alphaproteobacteria>Hyphomicrobiales>Rhizobiaceae	Rhizobium_sp._NXC14	DNA-sulfur-modification-protein-DndB-[Rhizobium-sp.-NXC14]	1981173
KQY41327.1	DndB-ParB-nuclease	GCA_001427205.1	409	Bacteria>Proteobacteria>Alphaproteobacteria>Hyphomicrobiales>Rhizobiaceae	Rhizobium_sp._Root491	DNA-phosphorothioation-associated-DGQHR-protein-1-[Rhizobium-sp.-Root491]	1736548

Accession	Annotation	GCA	Length	Phyletic.spread	Strain.name	Defline	Species.Taxid
WP_018099626.1	DndB-ParB-nuclease	GCF_000375585.1	370	Bacteria>Proteobacteria>Alphaproteobacteria>Hyphomicrobiales>Rhizobiaceae	Sinorhizobium_meliloti	DNA-sulfur-modification-protein-DndB-[Sinorhizobium-meliloti]	382
WP_200342396.1	DndB-ParB-nuclease	GCF_016583645.1	366	Bacteria>Proteobacteria>Alphaproteobacteria>Hyphomicrobiales>Rhodovibrionaceae	Rhodovibrio_sodomensis	DNA-sulfur-modification-protein-DndB-[Rhodovibrio-sodomensis]	1088
WP_200342398.1	DndB-ParB-nuclease	GCF_016583645.1	575	Bacteria>Proteobacteria>Alphaproteobacteria>Hyphomicrobiales>Rhodovibrionaceae	Rhodovibrio_sodomensis	hypothetical-protein-[Rhodovibrio-sodomensis]	1088
MBN9674102.1	DndB-ParB-nuclease	GCA_017309185.1	382	Bacteria>Proteobacteria>Alphaproteobacteria>Hyphomicrobiales>Stappiaceae	Roseibium_aggregatum	DGQHR-domain-containing-protein-[Labrenzia-aggregata]	187304
WP_207144393.1	DndB-ParB-nuclease	GCF_017309185.1	382	Bacteria>Proteobacteria>Alphaproteobacteria>Hyphomicrobiales>Stappiaceae	Roseibium_aggregatum	DGQHR-domain-containing-protein-[Roseibium-aggregatum]	187304
WP_224912884.1	DndB-ParB-nuclease	GCF_020177335.1	412	Bacteria>Proteobacteria>Alphaproteobacteria>Hyphomicrobiales>Stappiaceae	Stappia_indica	DNA-phosphorothioation-associated-DGQHR-protein-1-[Stappia-indica]	538381
WP_051624363.1	DndB-ParB-nuclease	GCF_000685295.1	419	Bacteria>Proteobacteria>Alphaproteobacteria>Hyphomonadales>Hyphomonadaceae	Hyphomonas_oceanitis	DNA-phosphorothioation-associated-DGQHR-protein-1-[Hyphomonas-oceanitis]	81033
WP_035534530.1	DndB-ParB-nuclease	GCF_000685295.1	362	Bacteria>Proteobacteria>Alphaproteobacteria>Hyphomonadales>Hyphomonadaceae	Hyphomonas_oceanitis	DNA-sulfur-modification-protein-DndB-[Hyphomonas-oceanitis]	81033
KDA04222.1	DndB-ParB-nuclease	GCA_000685295.1	419	Bacteria>Proteobacteria>Alphaproteobacteria>Hyphomonadales>Hyphomonadaceae	Hyphomonas_oceanitis_SCH89	hypothetical-protein-HOC_00015-[Hyphomonas-oceanitis-SCH89]	1280953
KDA04231.1	DndB-ParB-nuclease	GCA_000685295.1	362	Bacteria>Proteobacteria>Alphaproteobacteria>Hyphomonadales>Hyphomonadaceae	Hyphomonas_oceanitis_SCH89	hypothetical-protein-HOC_00060-[Hyphomonas-oceanitis-SCH89]	1280953
WP_114106411.1	DndB-ParB-nuclease	GCF_003325995.1	370	Bacteria>Proteobacteria>Alphaproteobacteria>Hyphomonadales>Hyphomonadaceae	Hyphomonas_sp._CACIAM_19H1	DNA-sulfur-modification-protein-DndB-[Hyphomonas-sp.-CACIAM-19H1]	1873716
WP_121940204.1	DndB-ParB-nuclease	GCF_003688555.1	370	Bacteria>Proteobacteria>Alphaproteobacteria>Kordiimonadales>Kordiimonadaceae	Eilatimonas_milleporae	DNA-sulfur-modification-protein-DndB-[Eilatimonas-milleporae]	911205
WP_166937040.1	DndB-ParB-nuclease	-	370	Bacteria>Proteobacteria>Alphaproteobacteria>Micropepsales>Micropepsaceae	Rhizomicrobium_electricum	DNA-sulfur-modification-protein-DndB-[Rhizomicrobium-electricum]	480070
AFS47938.1	DndB-ParB-nuclease	GCA_000299095.1	361	Bacteria>Proteobacteria>Alphaproteobacteria>Pelagibacterales>Pelagibacteraceae	alpha_proteobacterium_HIMB5	DNA-sulfur-modification-protein-DndB-[alpha-proteobacterium-HIMB5]	859653
WP_006996743.1	DndB-ParB-nuclease	GCF_000153525.1	361	Bacteria>Proteobacteria>Alphaproteobacteria>Pelagibacterales>Pelagibacteraceae	Candidatus_Pelagibacter_ubique	DNA-sulfur-modification-protein-DndB-[Candidatus-Pelagibacter-ubique]	198252
MBL6758240.1	DndB-ParB-nuclease	GCA_016779055.1	360	Bacteria>Proteobacteria>Alphaproteobacteria>Pelagibacterales>Pelagibacteraceae	Pelagibacteraceae_bacterium	DNA-sulfur-modification-protein-DndB-[Pelagibacteraceae-bacterium]	2026775
WP_055187246.1	DndB-ParB-nuclease	GCF_900116725.1	361	Bacteria>Proteobacteria>Alphaproteobacteria>Rhodobacterales>Rhodobacteraceae	Aliiroseovarius_crassostreae	DNA-sulfur-modification-protein-DndB-[Aliiroseovarius-crassostreae]	154981
WP_133489745.1	DndB-ParB-nuclease	GCF_004360145.1	364	Bacteria>Proteobacteria>Alphaproteobacteria>Rhodobacterales>Rhodobacteraceae	Aliiroseovarius_marinus	DNA-sulfur-modification-protein-DndB-[Aliiroseovarius-marinus]	2500159
MYG28481.1	DndB-ParB-nuclease	GCA_009842365.1	433	Bacteria>Proteobacteria>Alphaproteobacteria>Rhodobacterales>Rhodobacteraceae	Boseongicola_sp._SB0677_bin_26	DGQHR-domain-containing-protein-[Boseongicola-sp.-SB0677_bin_26]	2604851
THD81401.1	DndB-ParB-nuclease	GCA_004799285.1	363	Bacteria>Proteobacteria>Alphaproteobacteria>Rhodobacterales>Rhodobacteraceae	Gemmobacter_aestuarii	DNA-sulfur-modification-protein-DndB-[Gemmobacter-aestuarii]	1445661
WP_136395659.1	DndB-ParB-nuclease	GCF_004799285.1	363	Bacteria>Proteobacteria>Alphaproteobacteria>Rhodobacterales>Rhodobacteraceae	Gemmobacter_aestuarii	DNA-sulfur-modification-protein-DndB-[Gemmobacter-aestuarii]	1445661
WP_172332136.1	DndB-ParB-nuclease	GCF_013184745.1	411	Bacteria>Proteobacteria>Alphaproteobacteria>Rhodobacterales>Rhodobacteraceae	Mangrovicoccus_sp._HB161399	DNA-phosphorothioation-associated-DGQHR-protein-1-[Mangrovicoccus-sp.-HB161399]	2720392
WP_118134076.1	DndB-ParB-nuclease	GCF_003448965.1	415	Bacteria>Proteobacteria>Alphaproteobacteria>Rhodobacterales>Rhodobacteraceae	Oceanicella_sp._SM1341	DNA-phosphorothioation-associated-DGQHR-protein-1-[Oceanicella-sp.-SM1341]	1548889
MCC5968800.1	DndB-ParB-nuclease	GCA_020832545.1	363	Bacteria>Proteobacteria>Alphaproteobacteria>Rhodobacterales>Rhodobacteraceae	Pararhodobacter_sp.	DNA-sulfur-modification-protein-DndB-[Pararhodobacter-sp.]	2127056
QIK42420.1	DndB-ParB-nuclease	GCA_011290545.1	424	Bacteria>Proteobacteria>Alphaproteobacteria>Rhodobacterales>Rhodobacteraceae	Pontibrevibacter_nitratireducens	DGQHR-domain-containing-protein-[Pontibrevibacter-nitratireducens]	2758038
WP_166195129.1	DndB-ParB-nuclease	GCF_011290545.1	424	Bacteria>Proteobacteria>Alphaproteobacteria>Rhodobacterales>Rhodobacteraceae	Pontibrevibacter_nitratireducens	DNA-phosphorothioation-associated-DGQHR-protein-1-[Monaibacterium-sp.-ALG8]	2758038
WP_166195096.1	DndB-ParB-nuclease	GCF_011290545.1	368	Bacteria>Proteobacteria>Alphaproteobacteria>Rhodobacterales>Rhodobacteraceae	Pontibrevibacter_nitratireducens	DNA-sulfur-modification-protein-DndB-[Monaibacterium-sp.-ALG8]	2758038
QIK42409.1	DndB-ParB-nuclease	GCA_011290545.1	368	Bacteria>Proteobacteria>Alphaproteobacteria>Rhodobacterales>Rhodobacteraceae	Pontibrevibacter_nitratireducens	DNA-sulfur-modification-protein-DndB-[Pontibrevibacter-nitratireducens]	2758038
WP_136637576.1	DndB-ParB-nuclease	GCF_004959925.1	363	Bacteria>Proteobacteria>Alphaproteobacteria>Rhodobacterales>Rhodobacteraceae	Pseudooceanicola_onchidii	DNA-sulfur-modification-protein-DndB-[Pseudooceanicola-onchidii]	2562279
MBB04749.1	DndB-ParB-nuclease	GCA_002709085.1	370	Bacteria>Proteobacteria>Alphaproteobacteria>Rhodobacterales>Rhodobacteraceae	Pseudooceanicola_sp.	DNA-sulfur-modification-protein-DndB-[Pseudooceanicola-sp.]	1914328
WP_050524077.1	DndB-ParB-nuclease	GCF_001202035.1	361	Bacteria>Proteobacteria>Alphaproteobacteria>Rhodobacterales>Rhodobacteraceae	Pseudorhodobacter_wandonensis	DNA-sulfur-modification-protein-DndB-[Pseudorhodobacter-wandonensis]	1120568
PIV79053.1	DndB-ParB-nuclease	GCA_002783205.1	363	Bacteria>Proteobacteria>Alphaproteobacteria>Rhodobacterales>Rhodobacteraceae	Rhodobacteraceae_bacterium_CG17_big_fil_post_rev_8_21_14_2_50_63_15	DNA-sulfur-modification-protein-DndB-[Rhodobacteraceae-bacterium-CG17_big_fil_post_rev_8_21_14_2_50_63_15]	1974082
MBC6407911.1	DndB-ParB-nuclease	GCA_014323995.1	411	Bacteria>Proteobacteria>Alphaproteobacteria>Rhodobacterales>Rhodobacteraceae	Rhodobacteraceae_bacterium	DGQHR-domain-containing-protein-[Rhodobacteraceae-bacterium]	1904441
WP_226551974.1	DndB-ParB-nuclease	GCF_020524585.1	361	Bacteria>Proteobacteria>Alphaproteobacteria>Rhodobacterales>Roseobacteraceae	Celeribacter_naphthalenivorans	DNA-sulfur-modification-protein-DndB-[Celeribacter-naphthalenivorans]	1614694
WP_218151393.1	DndB-ParB-nuclease	GCF_900113955.1	424	Bacteria>Proteobacteria>Alphaproteobacteria>Rhodobacterales>Roseobacteraceae	Celeribacter_neptunius	DNA-phosphorothioation-associated-DGQHR-protein-1-[Celeribacter-neptunius]	588602
WP_090063029.1	DndB-ParB-nuclease	GCF_900113955.1	368	Bacteria>Proteobacteria>Alphaproteobacteria>Rhodobacterales>Roseobacteraceae	Celeribacter_neptunius	DNA-sulfur-modification-protein-DndB-[Celeribacter-neptunius]	588602
WP_106159251.1	DndB-ParB-nuclease	GCF_003003095.1	411	Bacteria>Proteobacteria>Alphaproteobacteria>Rhodobacterales>Roseobacteraceae	Hasllibacter_halocynthiae	DNA-phosphorothioation-associated-DGQHR-protein-1-[Hasllibacter-halocynthiae]	595589
PZX19966.1	DndB-ParB-nuclease	GCA_003253995.1	118	Bacteria>Proteobacteria>Alphaproteobacteria>Rhodobacterales>Roseobacteraceae	Hwanghaeicola_aestuarii	DNA-sulfur-modification-protein-DndB-[Hwanghaeicola-aestuarii]	568105
WP_111535613.1	DndB-ParB-nuclease	GCF_003253995.1	366	Bacteria>Proteobacteria>Alphaproteobacteria>Rhodobacterales>Roseobacteraceae	Hwanghaeicola_aestuarii	DNA-sulfur-modification-protein-DndB-[Hwanghaeicola-aestuarii]	568105
WP_092765173.1	DndB-ParB-nuclease	GCF_900109285.1	361	Bacteria>Proteobacteria>Alphaproteobacteria>Rhodobacterales>Roseobacteraceae	Jannaschia_helgolandensis	DNA-sulfur-modification-protein-DndB-[Jannaschia-helgolandensis]	188906
PWE34118.1	DndB-ParB-nuclease	GCA_003122205.1	370	Bacteria>Proteobacteria>Alphaproteobacteria>Rhodobacterales>Roseobacteraceae	Maritimibacter_sp._55A14	DNA-sulfur-modification-protein-DndB-[Maritimibacter-sp.-55A14]	2174844
WP_109423432.1	DndB-ParB-nuclease	GCF_003122205.1	370	Bacteria>Proteobacteria>Alphaproteobacteria>Rhodobacterales>Roseobacteraceae	Maritimibacter_sp._55A14	DNA-sulfur-modification-protein-DndB-[Maritimibacter-sp.-55A14]	2174844
MAE92540.1	DndB-ParB-nuclease	GCA_002687285.1	366	Bacteria>Proteobacteria>Alphaproteobacteria>Rhodobacterales>Roseobacteraceae	Pelagibaca_sp.	DNA-sulfur-modification-protein-DndB-[Pelagibaca-sp.]	1979400
MAE92544.1	DndB-ParB-nuclease	GCA_002687285.1	382	Bacteria>Proteobacteria>Alphaproteobacteria>Rhodobacterales>Roseobacteraceae	Pelagibaca_sp.	hypothetical-protein-[Pelagibaca-sp.]	1979400
WP_227363080.1	DndB-ParB-nuclease	GCF_020622295.1	409	Bacteria>Proteobacteria>Alphaproteobacteria>Rhodobacterales>Roseobacteraceae	Phaeobacter_sp._MR	DNA-phosphorothioation-associated-DGQHR-protein-1-[Phaeobacter-sp.-MR]	2763265
WP_227363070.1	DndB-ParB-nuclease	GCF_020622295.1	368	Bacteria>Proteobacteria>Alphaproteobacteria>Rhodobacterales>Roseobacteraceae	Phaeobacter_sp._MR	DNA-sulfur-modification-protein-DndB-[Phaeobacter-sp.-MR]	2763265
WP_068110636.1	DndB-ParB-nuclease	GCF_001509585.1	363	Bacteria>Proteobacteria>Alphaproteobacteria>Rhodobacterales>Roseobacteraceae	Pseudoruegeria_marinistellae	DNA-sulfur-modification-protein-DndB-[Pseudoruegeria-marinistellae]	1739787
WP_068110626.1	DndB-ParB-nuclease	GCF_001509585.1	560	Bacteria>Proteobacteria>Alphaproteobacteria>Rhodobacterales>Roseobacteraceae	Pseudoruegeria_marinistellae	hypothetical-protein-[Pseudoruegeria-marinistellae]	1739787
WP_043847329.1	DndB-ParB-nuclease	GCF_000520615.1	411	Bacteria>Proteobacteria>Alphaproteobacteria>Rhodobacterales>Roseobacteraceae	Roseivivax_marinus	DNA-phosphorothioation-associated-DGQHR-protein-1-[Roseivivax-marinus]	1379903
AVL54950.1	DndB-ParB-nuclease	GCA_002983865.1	408	Bacteria>Proteobacteria>Alphaproteobacteria>Rhodobacterales>Roseobacteraceae	Roseobacter_denitrificans	DGQHR-domain-containing-protein-[Roseobacter-denitrificans]	2434
WP_011567108.1	DndB-ParB-nuclease	GCF_000014045.1	410	Bacteria>Proteobacteria>Alphaproteobacteria>Rhodobacterales>Roseobacteraceae	Roseobacter_denitrificans	DNA-phosphorothioation-associated-DGQHR-protein-1-[Roseobacter-denitrificans]	2434
AVL53631.1	DndB-ParB-nuclease	GCA_002983865.1	368	Bacteria>Proteobacteria>Alphaproteobacteria>Rhodobacterales>Roseobacteraceae	Roseobacter_denitrificans	DNA-sulfur-modification-protein-DndB-[Roseobacter-denitrificans]	2434
WP_011567098.1	DndB-ParB-nuclease	GCF_000014045.1	368	Bacteria>Proteobacteria>Alphaproteobacteria>Rhodobacterales>Roseobacteraceae	Roseobacter_denitrificans	DNA-sulfur-modification-protein-DndB-[Roseobacter-denitrificans]	2434
ABG30476.1	DndB-ParB-nuclease	GCA_000014045.1	368	Bacteria>Proteobacteria>Alphaproteobacteria>Rhodobacterales>Roseobacteraceae	Roseobacter_denitrificans_OCh_114	conserved-hypothetical-protein-[Roseobacter-denitrificans-OCh-114]	375451
SFF73367.1	DndB-ParB-nuclease	GCA_900113215.1	410	Bacteria>Proteobacteria>Alphaproteobacteria>Rhodobacterales>Roseobacteraceae	Roseobacter_denitrificans_OCh_114	DNA-phosphorothioation-associated-DGQHR-protein-1-[Roseobacter-denitrificans-OCh-114]	375451
SFF73241.1	DndB-ParB-nuclease	GCA_900113215.1	368	Bacteria>Proteobacteria>Alphaproteobacteria>Rhodobacterales>Roseobacteraceae	Roseobacter_denitrificans_OCh_114	DNA-sulfur-modification-protein-DndB-[Roseobacter-denitrificans-OCh-114]	375451
ABG30486.1	DndB-ParB-nuclease	GCA_000014045.1	410	Bacteria>Proteobacteria>Alphaproteobacteria>Rhodobacterales>Roseobacteraceae	Roseobacter_denitrificans_OCh_114	hypothetical-protein-RD1_0808-[Roseobacter-denitrificans-OCh-114]	375451
NHQ74525.1	DndB-ParB-nuclease	GCA_011601345.1	368	Bacteria>Proteobacteria>Alphaproteobacteria>Rhodobacterales>Roseobacteraceae	Roseovarius_gahaiensis	DNA-sulfur-modification-protein-DndB-[Roseovarius-gahaiensis]	2716691
WP_167195892.1	DndB-ParB-nuclease	GCF_011601345.1	368	Bacteria>Proteobacteria>Alphaproteobacteria>Rhodobacterales>Roseobacteraceae	Roseovarius_gahaiensis	DNA-sulfur-modification-protein-DndB-[Roseovarius-gahaiensis]	2716691
NOE18077.1	DndB-ParB-nuclease	GCA_013110705.1	368	Bacteria>Proteobacteria>Alphaproteobacteria>Rhodobacterales>Roseobacteraceae	Ruegeria_atlantica	DNA-sulfur-modification-protein-DndB-[Ruegeria-atlantica]	81569
WP_171329439.1	DndB-ParB-nuclease	GCF_013110705.1	368	Bacteria>Proteobacteria>Alphaproteobacteria>Rhodobacterales>Roseobacteraceae	Ruegeria_atlantica	DNA-sulfur-modification-protein-DndB-[Ruegeria-atlantica]	81569
WP_152460875.1	DndB-ParB-nuclease	GCF_009363615.1	410	Bacteria>Proteobacteria>Alphaproteobacteria>Rhodobacterales>Roseobacteraceae	Ruegeria_sp._THAF33	DNA-phosphorothioation-associated-DGQHR-protein-1-[Ruegeria-sp.-THAF33]	2587853
WP_152460863.1	DndB-ParB-nuclease	GCF_009363615.1	368	Bacteria>Proteobacteria>Alphaproteobacteria>Rhodobacterales>Roseobacteraceae	Ruegeria_sp._THAF33	DNA-sulfur-modification-protein-DndB-[Ruegeria-sp.-THAF33]	2587853
QFT75871.1	DndB-ParB-nuclease	GCA_009363615.1	368	Bacteria>Proteobacteria>Alphaproteobacteria>Rhodobacterales>Roseobacteraceae	Ruegeria_sp._THAF33	hypothetical-protein-FIU92_22685-[Ruegeria-sp.-THAF33]	2587853
QFT75883.1	DndB-ParB-nuclease	GCA_009363615.1	410	Bacteria>Proteobacteria>Alphaproteobacteria>Rhodobacterales>Roseobacteraceae	Ruegeria_sp._THAF33	hypothetical-protein-FIU92_22745-[Ruegeria-sp.-THAF33]	2587853
EEW56580.1	DndB-ParB-nuclease	GCA_000161815.1	361	Bacteria>Proteobacteria>Alphaproteobacteria>Rhodobacterales>Roseobacteraceae	Ruegeria_sp._TrichCH4B	DNA-sulfur-modification-protein-DndB-[Ruegeria-sp.-TrichCH4B]	644076
EEW56561.1	DndB-ParB-nuclease	GCA_000161815.1	419	Bacteria>Proteobacteria>Alphaproteobacteria>Rhodobacterales>Roseobacteraceae	Ruegeria_sp._TrichCH4B	putative-dgqhr-domain-containing-protein-[Ruegeria-sp.-TrichCH4B]	644076
WP_093412625.1	DndB-ParB-nuclease	GCF_900112435.1	415	Bacteria>Proteobacteria>Alphaproteobacteria>Rhodobacterales>Roseobacteraceae	Salipiger_profundus	DNA-phosphorothioation-associated-DGQHR-protein-1-[Salipiger-profundus]	1229727
WP_093741856.1	DndB-ParB-nuclease	GCF_900102535.1	389	Bacteria>Proteobacteria>Alphaproteobacteria>Rhodobacterales>Roseobacteraceae	Sulfitobacter_delicatus	DGQHR-domain-containing-protein-[Sulfitobacter-delicatus]	218672
WP_051539629.1	DndB-ParB-nuclease	GCF_000620505.1	419	Bacteria>Proteobacteria>Alphaproteobacteria>Rhodobacterales>Roseobacteraceae	Sulfitobacter_sp._20_GPM-1509m	DNA-phosphorothioation-associated-DGQHR-protein-1-[Sulfitobacter-sp.-20_GPM-1509m]	1380367
WP_037916072.1	DndB-ParB-nuclease	GCF_000620505.1	361	Bacteria>Proteobacteria>Alphaproteobacteria>Rhodobacterales>Roseobacteraceae	Sulfitobacter_sp._20_GPM-1509m	DNA-sulfur-modification-protein-DndB-[Sulfitobacter-sp.-20_GPM-1509m]	1380367
QUJ78338.1	DndB-ParB-nuclease	GCA_018200035.1	409	Bacteria>Proteobacteria>Alphaproteobacteria>Rhodobacterales>Roseobacteraceae	Sulfitobacter_sp._JK7-1	DNA-phosphorothioation-associated-DGQHR-protein-1-[Sulfitobacter-sp.-JK7-1]	2829501
WP_212706530.1	DndB-ParB-nuclease	GCF_018200035.1	409	Bacteria>Proteobacteria>Alphaproteobacteria>Rhodobacterales>Roseobacteraceae	Sulfitobacter_sp._JK7-1	DNA-phosphorothioation-associated-DGQHR-protein-1-[Sulfitobacter-sp.-JK7-1]	2829501
WP_197918491.1	DndB-ParB-nuclease	GCF_016019955.1	409	Bacteria>Proteobacteria>Alphaproteobacteria>Rhodobacterales>Roseobacteraceae	Thiosulfatihalobacter_marinus	DNA-phosphorothioation-associated-DGQHR-protein-1-[Pseudoruegeria-sp.-GL-11-2]	2792481
WP_197918465.1	DndB-ParB-nuclease	GCF_016019955.1	368	Bacteria>Proteobacteria>Alphaproteobacteria>Rhodobacterales>Roseobacteraceae	Thiosulfatihalobacter_marinus	DNA-sulfur-modification-protein-DndB-[Pseudoruegeria-sp.-GL-11-2]	2792481
WP_075632286.1	DndB-ParB-nuclease	GCF_001938835.1	425	Bacteria>Proteobacteria>Alphaproteobacteria>Rhodospirillales>Acetobacteraceae	Novacetimonas_hansenii	DGQHR-domain-containing-protein-[Komagataeibacter-hansenii]	436
WP_075632291.1	DndB-ParB-nuclease	GCF_001938835.1	370	Bacteria>Proteobacteria>Alphaproteobacteria>Rhodospirillales>Acetobacteraceae	Novacetimonas_hansenii	DNA-sulfur-modification-protein-DndB-[Komagataeibacter-hansenii]	436
MBX9596018.1	DndB-ParB-nuclease	GCA_019748015.1	420	Bacteria>Proteobacteria>Alphaproteobacteria>Rhodospirillales>Acetobacteraceae	Roseomonas_sp.	DGQHR-domain-containing-protein-[Roseomonas-sp.]	1869190
WP_207462155.1	DndB-ParB-nuclease	GCF_017356015.1	359	Bacteria>Proteobacteria>Alphaproteobacteria>Rhodospirillales>Azospirillaceae	Azospirillum_sp._SYSU_D00513	DNA-sulfur-modification-protein-DndB-[Azospirillum-sp.-SYSU-D00513]	2812561
WP_200483906.1	DndB-ParB-nuclease	GCF_016632405.1	365	Bacteria>Proteobacteria>Alphaproteobacteria>Rhodospirillales>Azospirillaceae	Azospirillum_sp._YIM_DDC1	DNA-sulfur-modification-protein-DndB-[Azospirillum-sp.-YIM-DDC1]	2802052
MCB9968268.1	DndB-ParB-nuclease	GCA_020638495.1	410	Bacteria>Proteobacteria>Alphaproteobacteria>Rhodospirillales>Geminicoccaceae	Geminicoccaceae_bacterium	DGQHR-domain-containing-protein-[Geminicoccaceae-bacterium]	2448052
OUU31403.1	DndB-ParB-nuclease	GCA_002167635.1	365	Bacteria>Proteobacteria>Alphaproteobacteria>Rhodospirillales>Rhodospirillaceae	Candidatus_Endolissoclinum_sp._TMED37	DNA-sulfur-modification-protein-DndB-[Candidatus-Endolissoclinum-sp.-TMED37]	1986638
WP_173982210.1	DndB-ParB-nuclease	GCF_902729425.1	364	Bacteria>Proteobacteria>Alphaproteobacteria>Rhodospirillales>Rhodospirillaceae	Magnetospirillum_sp._SS-4	DNA-sulfur-modification-protein-DndB-[Magnetospirillum-sp.-SS-4]	2681465
WP_022730139.1	DndB-ParB-nuclease	GCF_000421265.1	368	Bacteria>Proteobacteria>Alphaproteobacteria>Rhodospirillales>Thalassospiraceae	Thalassospira_lucentensis	DNA-sulfur-modification-protein-DndB-[Thalassospira-lucentensis]	168935
KZX87829.1	DndB-ParB-nuclease	GCA_001634715.1	417	Bacteria>Proteobacteria>Alphaproteobacteria>Sphingomonadales>Erythrobacteraceae	Erythrobacter_sp._HI0020	hypothetical-protein-A3719_09705-[Erythrobacter-sp.-HI0020]	1822223
PLK22753.1	DndB-ParB-nuclease	GCA_002855495.1	359	Bacteria>Proteobacteria>Alphaproteobacteria>Sphingomonadales>Erythrobacteraceae	Porphyrobacter_sp._TH134	DNA-sulfur-modification-protein-DndB-[Porphyrobacter-sp.-TH134]	2067450
WP_172439028.1	DndB-ParB-nuclease	GCF_002855495.1	352	Bacteria>Proteobacteria>Alphaproteobacteria>Sphingomonadales>Erythrobacteraceae	Porphyrobacter_sp._TH134	DNA-sulfur-modification-protein-DndB-[Porphyrobacter-sp.-TH134]	2067450
KPF88412.1	DndB-ParB-nuclease	GCA_001295795.1	366	Bacteria>Proteobacteria>Alphaproteobacteria>Sphingomonadales>Sphingomonadaceae	Novosphingobium_sp._AAP83	DNA-sulfur-modification-protein-DndB-[Novosphingobium-sp.-AAP83]	1523425
WP_054109457.1	DndB-ParB-nuclease	GCF_001295795.1	366	Bacteria>Proteobacteria>Alphaproteobacteria>Sphingomonadales>Sphingomonadaceae	Novosphingobium_sp._AAP83	DNA-sulfur-modification-protein-DndB-[Novosphingobium-sp.-AAP83]	1523425
MBK6803281.1	DndB-ParB-nuclease	GCA_016702815.1	360	Bacteria>Proteobacteria>Alphaproteobacteria>Sphingomonadales>Sphingomonadaceae	Novosphingobium_sp.	DNA-sulfur-modification-protein-DndB-[Novosphingobium-sp.]	1874826
MBK6803287.1	DndB-ParB-nuclease	GCA_016702815.1	638	Bacteria>Proteobacteria>Alphaproteobacteria>Sphingomonadales>Sphingomonadaceae	Novosphingobium_sp.	hypothetical-protein-[Novosphingobium-sp.]	1874826
WP_066966589.1	DndB-ParB-nuclease	GCF_002355275.1	413	Bacteria>Proteobacteria>Alphaproteobacteria>Sphingomonadales>Sphingomonadaceae	Rhizorhabdus_dicambivorans	DGQHR-domain-containing-protein-[Rhizorhabdus-dicambivorans]	1850238
WP_066968412.1	DndB-ParB-nuclease	GCF_002355275.1	370	Bacteria>Proteobacteria>Alphaproteobacteria>Sphingomonadales>Sphingomonadaceae	Rhizorhabdus_dicambivorans	DNA-sulfur-modification-protein-DndB-[Rhizorhabdus-dicambivorans]	1850238
WP_217673801.1	DndB-ParB-nuclease	GCF_019097845.1	366	Bacteria>Proteobacteria>Alphaproteobacteria>Sphingomonadales>Sphingomonadaceae	Sphingobium_sp._AS12	DNA-sulfur-modification-protein-DndB-[Sphingobium-sp.-AS12]	2849495
WP_237219742.1	DndB-ParB-nuclease	GCF_016924655.1	328	Bacteria>Proteobacteria>Alphaproteobacteria>Sphingomonadales>Sphingomonadaceae	Sphingomonas_arenae	DNA-sulfur-modification-protein-DndB-[Sphingomonas-arenae]	2812555
TXC71320.1	DndB-ParB-nuclease	GCA_007995065.1	429	Bacteria>Proteobacteria>Alphaproteobacteria>Sphingomonadales>Sphingomonadaceae	Sphingomonas_ginsenosidivorax	DGQHR-domain-containing-protein-[Sphingomonas-ginsenosidivorax]	862135
WP_147082490.1	DndB-ParB-nuclease	GCF_007995065.1	429	Bacteria>Proteobacteria>Alphaproteobacteria>Sphingomonadales>Sphingomonadaceae	Sphingomonas_ginsenosidivorax	DGQHR-domain-containing-protein-[Sphingomonas-ginsenosidivorax]	862135
WP_147082504.1	DndB-ParB-nuclease	GCF_007995065.1	344	Bacteria>Proteobacteria>Alphaproteobacteria>Sphingomonadales>Sphingomonadaceae	Sphingomonas_ginsenosidivorax	DNA-sulfur-modification-protein-DndB-[Sphingomonas-ginsenosidivorax]	862135
WP_135984588.1	DndB-ParB-nuclease	GCF_011762145.1	350	Bacteria>Proteobacteria>Alphaproteobacteria>Sphingomonadales>Sphingomonadaceae	Sphingomonas_naasensis	DNA-sulfur-modification-protein-DndB-[Sphingomonas-naasensis]	1344951
WP_093007124.1	DndB-ParB-nuclease	GCF_900109565.1	426	Bacteria>Proteobacteria>Alphaproteobacteria>Sphingomonadales>Sphingomonadaceae	Sphingomonas_palmae	DGQHR-domain-containing-protein-[Sphingomonas-palmae]	1855283
SEL79949.1	DndB-ParB-nuclease	GCA_900109565.1	343	Bacteria>Proteobacteria>Alphaproteobacteria>Sphingomonadales>Sphingomonadaceae	Sphingomonas_palmae	DNA-sulfur-modification-protein-DndB-[Sphingomonas-palmae]	1855283
SEL80243.1	DndB-ParB-nuclease	GCA_900109565.1	426	Bacteria>Proteobacteria>Alphaproteobacteria>Sphingomonadales>Sphingomonadaceae	Sphingomonas_palmae	DNA-sulfur-modification-protein-DndB-[Sphingomonas-palmae]	1855283
WP_177171674.1	DndB-ParB-nuclease	GCF_900109565.1	343	Bacteria>Proteobacteria>Alphaproteobacteria>Sphingomonadales>Sphingomonadaceae	Sphingomonas_palmae	DNA-sulfur-modification-protein-DndB-[Sphingomonas-palmae]	1855283
WP_084253546.1	DndB-ParB-nuclease	GCF_001598435.1	416	Bacteria>Proteobacteria>Alphaproteobacteria>Sphingomonadales>Sphingomonadaceae	Sphingomonas_pituitosa	DNA-phosphorothioation-associated-DGQHR-protein-1-[Sphingomonas-pituitosa]	99597
WP_084253545.1	DndB-ParB-nuclease	GCF_001598435.1	382	Bacteria>Proteobacteria>Alphaproteobacteria>Sphingomonadales>Sphingomonadaceae	Sphingomonas_pituitosa	DNA-sulfur-modification-protein-DndB-[Sphingomonas-pituitosa]	99597
WP_156681074.1	DndB-ParB-nuclease	GCF_009739515.1	437	Bacteria>Proteobacteria>Alphaproteobacteria>Sphingomonadales>Sphingomonadaceae	Sphingomonas_profundi	DGQHR-domain-containing-protein-[Sphingomonas-sp.-LMO-1]	2681549
WP_156681079.1	DndB-ParB-nuclease	GCF_009739515.1	355	Bacteria>Proteobacteria>Alphaproteobacteria>Sphingomonadales>Sphingomonadaceae	Sphingomonas_profundi	DNA-sulfur-modification-protein-DndB-[Sphingomonas-sp.-LMO-1]	2681549
NUR36589.1	DndB-ParB-nuclease	GCA_013362105.1	410	Bacteria>Proteobacteria>Alphaproteobacteria>Sphingomonadales>Sphingomonadaceae	Sphingomonas_sp.	DGQHR-domain-containing-protein-[Sphingomonas-sp.]	28214
THD34463.1	DndB-ParB-nuclease	GCA_004799495.1	429	Bacteria>Proteobacteria>Alphaproteobacteria>Sphingomonadales>Sphingomonadaceae	Sphingomonas_sp.	DGQHR-domain-containing-protein-[Sphingomonas-sp.]	28214
WP_231420790.1	DndB-ParB-nuclease	GCF_920984725.1	624	Bacteria>Proteobacteria>Alphaproteobacteria>Sphingomonadales>Sphingomonadaceae	Sphingomonas_sp._Leaf205	hypothetical-protein-[Sphingomonas-sp.-Leaf205]	2876551
WP_056497938.1	DndB-ParB-nuclease	GCF_001421635.1	332	Bacteria>Proteobacteria>Alphaproteobacteria>Sphingomonadales>Sphingomonadaceae	Sphingomonas_sp._Leaf25	DNA-sulfur-modification-protein-DndB-[Sphingomonas-sp.-Leaf25]	1735692
WP_156351493.1	DndB-ParB-nuclease	GCF_001421635.1	561	Bacteria>Proteobacteria>Alphaproteobacteria>Sphingomonadales>Sphingomonadaceae	Sphingomonas_sp._Leaf25	hypothetical-protein-[Sphingomonas-sp.-Leaf25]	1735692
WP_219756599.1	DndB-ParB-nuclease	GCF_019429525.1	358	Bacteria>Proteobacteria>Alphaproteobacteria>Sphingomonadales>Sphingomonadaceae	Sphingomonas_sp._RHCKR7	DNA-sulfur-modification-protein-DndB-[Sphingomonas-sp.-RHCKR7]	2862497
WP_235077167.1	DndB-ParB-nuclease	GCF_021513175.1	437	Bacteria>Proteobacteria>Alphaproteobacteria>Sphingomonadales>Sphingomonadaceae	Sphingomonas_sp._S2-65	DGQHR-domain-containing-protein-[Sphingomonas-sp.-S2-65]	2903960
WP_235077159.1	DndB-ParB-nuclease	GCF_021513175.1	355	Bacteria>Proteobacteria>Alphaproteobacteria>Sphingomonadales>Sphingomonadaceae	Sphingomonas_sp._S2-65	DNA-sulfur-modification-protein-DndB-[Sphingomonas-sp.-S2-65]	2903960
WP_077029268.1	DndB-ParB-nuclease	GCF_001984035.1	415	Bacteria>Proteobacteria>Alphaproteobacteria>Sphingomonadales>Sphingomonadaceae	Sphingopyxis_sp._QXT-31	DGQHR-domain-containing-protein-[Sphingopyxis-sp.-QXT-31]	1357916
WP_137864588.1	DndB-ParB-nuclease	GCF_005503355.1	336	Bacteria>Proteobacteria>Alphaproteobacteria>Sphingomonadales>Sphingomonadaceae	unclassified_Sphingomonas	MULTISPECIES:-DNA-sulfur-modification-protein-DndB-[unclassified-Sphingomonas]	196159
MBF0355962.1	DndB-ParB-nuclease	GCA_015232335.1	446	Bacteria>Proteobacteria>Alphaproteobacteria>unclassified_Alphaproteobacteria	Alphaproteobacteria_bacterium	DGQHR-domain-containing-protein-[Alphaproteobacteria-bacterium]	1913988
MBQ2885086.1	DndB-ParB-nuclease	GCA_017409945.1	414	Bacteria>Proteobacteria>Alphaproteobacteria>unclassified_Alphaproteobacteria	Alphaproteobacteria_bacterium	DGQHR-domain-containing-protein-[Alphaproteobacteria-bacterium]	1913988
NCT05140.1	DndB-ParB-nuclease	GCA_009995245.1	419	Bacteria>Proteobacteria>Alphaproteobacteria>unclassified_Alphaproteobacteria	Alphaproteobacteria_bacterium	DGQHR-domain-containing-protein-[Alphaproteobacteria-bacterium]	1913988
MBF0355958.1	DndB-ParB-nuclease	GCA_015232335.1	365	Bacteria>Proteobacteria>Alphaproteobacteria>unclassified_Alphaproteobacteria	Alphaproteobacteria_bacterium	DNA-sulfur-modification-protein-DndB-[Alphaproteobacteria-bacterium]	1913988
MBO4521398.1	DndB-ParB-nuclease	GCA_017616015.1	358	Bacteria>Proteobacteria>Alphaproteobacteria>unclassified_Alphaproteobacteria	Alphaproteobacteria_bacterium	DNA-sulfur-modification-protein-DndB-[Alphaproteobacteria-bacterium]	1913988
MBQ8464585.1	DndB-ParB-nuclease	GCA_017467515.1	363	Bacteria>Proteobacteria>Alphaproteobacteria>unclassified_Alphaproteobacteria	Alphaproteobacteria_bacterium	DNA-sulfur-modification-protein-DndB-[Alphaproteobacteria-bacterium]	1913988
PCI52866.1	DndB-ParB-nuclease	GCA_002401695.1	372	Bacteria>Proteobacteria>Alphaproteobacteria>unclassified_Alphaproteobacteria	Alphaproteobacteria_bacterium	DNA-sulfur-modification-protein-DndB-[Alphaproteobacteria-bacterium]	1913988
WP_186407639.1	DndB-ParB-nuclease	GCF_900089955.1	432	Bacteria>Proteobacteria>Betaproteobacteria>Betaproteobacteria_incertae_sedis>Candidatus_Accumulibacter	Candidatus_Accumulibacter_aalborgensis	DGQHR-domain-containing-protein-[Candidatus-Accumulibacter-aalborgensis]	1860102

Accession	Annotation	GCA	Length	Phyletic.spread	Strain.name	Defline	Species.Taxid
WP_094797374.1	DndB-ParB-nuclease	GCF_002261265.1	418	Bacteria>Proteobacteria>Betaproteobacteria>Burkholderiales>Alcaligenaceae	Bordetella_genomosp._7	DGQHR-domain-containing-protein-[Bordetella-genomosp.-7]	1416805
WP_094797368.1	DndB-ParB-nuclease	GCF_002261265.1	373	Bacteria>Proteobacteria>Betaproteobacteria>Burkholderiales>Alcaligenaceae	Bordetella_genomosp._7	DNA-sulfur-modification-protein-DndB-[Bordetella-genomosp.-7]	1416805
AUL49004.1	DndB-ParB-nuclease	GCA_002860045.1	397	Bacteria>Proteobacteria>Betaproteobacteria>Burkholderiales>Alcaligenaceae	Bordetella_trematum	DNA-phosphorothioation-associated-DGQHR-protein-1-[Bordetella-trematum]	123899
WP_201797414.1	DndB-ParB-nuclease	GCF_002860045.1	398	Bacteria>Proteobacteria>Betaproteobacteria>Burkholderiales>Alcaligenaceae	Bordetella_trematum	DNA-phosphorothioation-associated-DGQHR-protein-1-[Bordetella-trematum]	123899
WP_175700544.1	DndB-ParB-nuclease	GCF_902829815.1	374	Bacteria>Proteobacteria>Betaproteobacteria>Burkholderiales>Burkholderiaceae	Burkholderia_ambifaria	DNA-sulfur-modification-protein-DndB-[Burkholderia-ambifaria]	152480
WP_175700548.1	DndB-ParB-nuclease	GCF_902829815.1	567	Bacteria>Proteobacteria>Betaproteobacteria>Burkholderiales>Burkholderiaceae	Burkholderia_ambifaria	hypothetical-protein-[Burkholderia-ambifaria]	152480
WP_175718793.1	DndB-ParB-nuclease	GCF_902830145.1	360	Bacteria>Proteobacteria>Betaproteobacteria>Burkholderiales>Burkholderiaceae	Burkholderia_anthina	DNA-sulfur-modification-protein-DndB-[Burkholderia-anthina]	179879
PCH58902.1	DndB-ParB-nuclease	GCA_002400655.1	363	Bacteria>Proteobacteria>Betaproteobacteria>Burkholderiales>Burkholderiaceae	Burkholderiaceae_bacterium	DNA-sulfur-modification-protein-DndB-[Burkholderiaceae-bacterium]	2030806
RQV58263.1	DndB-ParB-nuclease	GCA_003854805.1	403	Bacteria>Proteobacteria>Betaproteobacteria>Burkholderiales>Burkholderiaceae	Burkholderia_cenocepacia	DGQHR-domain-containing-protein-[Burkholderia-cenocepacia]	95486
WP_124714865.1	DndB-ParB-nuclease	GCF_003854805.1	403	Bacteria>Proteobacteria>Betaproteobacteria>Burkholderiales>Burkholderiaceae	Burkholderia_cenocepacia	DGQHR-domain-containing-protein-[Burkholderia-cenocepacia]	95486
WP_012362696.1	DndB-ParB-nuclease	GCF_000019925.1	375	Bacteria>Proteobacteria>Betaproteobacteria>Burkholderiales>Burkholderiaceae	Burkholderia_cepacia_complex	MULTISPECIES:-DNA-sulfur-modification-protein-DndB-[Burkholderia-cepacia-complex]	87882
RQT15086.1	DndB-ParB-nuclease	GCA_003854375.1	403	Bacteria>Proteobacteria>Betaproteobacteria>Burkholderiales>Burkholderiaceae	Burkholderia_contaminans	DGQHR-domain-containing-protein-[Burkholderia-contaminans]	488447
WP_082138510.1	DndB-ParB-nuclease	GCF_000987055.1	379	Bacteria>Proteobacteria>Betaproteobacteria>Burkholderiales>Burkholderiaceae	Burkholderia_contaminans	DGQHR-domain-containing-protein-[Burkholderia-contaminans]	488447
WP_124619945.1	DndB-ParB-nuclease	GCF_902499285.1	403	Bacteria>Proteobacteria>Betaproteobacteria>Burkholderiales>Burkholderiaceae	Burkholderia_contaminans	DGQHR-domain-containing-protein-[Burkholderia-contaminans]	488447
WP_046544140.1	DndB-ParB-nuclease	GCF_000987055.1	377	Bacteria>Proteobacteria>Betaproteobacteria>Burkholderiales>Burkholderiaceae	Burkholderia_contaminans	DNA-sulfur-modification-protein-DndB-[Burkholderia-contaminans]	488447
WP_186116276.1	DndB-ParB-nuclease	GCF_902831505.1	351	Bacteria>Proteobacteria>Betaproteobacteria>Burkholderiales>Burkholderiaceae	Burkholderia_gladioli	DGQHR-domain-containing-protein-[Burkholderia-gladioli]	28095
WP_217043541.1	DndB-ParB-nuclease	GCF_019041225.1	379	Bacteria>Proteobacteria>Betaproteobacteria>Burkholderiales>Burkholderiaceae	Burkholderia_gladioli	DGQHR-domain-containing-protein-[Burkholderia-gladioli]	28095
WP_186116272.1	DndB-ParB-nuclease	GCF_902831505.1	375	Bacteria>Proteobacteria>Betaproteobacteria>Burkholderiales>Burkholderiaceae	Burkholderia_gladioli	DNA-sulfur-modification-protein-DndB-[Burkholderia-gladioli]	28095
WP_186132753.1	DndB-ParB-nuclease	GCF_902832115.1	375	Bacteria>Proteobacteria>Betaproteobacteria>Burkholderiales>Burkholderiaceae	Burkholderia_gladioli	DNA-sulfur-modification-protein-DndB-[Burkholderia-gladioli]	28095
WP_186150783.1	DndB-ParB-nuclease	GCF_902832105.1	374	Bacteria>Proteobacteria>Betaproteobacteria>Burkholderiales>Burkholderiaceae	Burkholderia_gladioli	DNA-sulfur-modification-protein-DndB-[Burkholderia-gladioli]	28095
WP_217043537.1	DndB-ParB-nuclease	GCF_019041225.1	377	Bacteria>Proteobacteria>Betaproteobacteria>Burkholderiales>Burkholderiaceae	Burkholderia_gladioli	DNA-sulfur-modification-protein-DndB-[Burkholderia-gladioli]	28095
WP_171466814.1	DndB-ParB-nuclease	-	376	Bacteria>Proteobacteria>Betaproteobacteria>Burkholderiales>Burkholderiaceae	Burkholderia_glumae	DGQHR-domain-containing-protein-[Burkholderia-glumae]	337
WP_175000876.1	DndB-ParB-nuclease	GCF_902499095.1	377	Bacteria>Proteobacteria>Betaproteobacteria>Burkholderiales>Burkholderiaceae	Burkholderia_lata	DNA-sulfur-modification-protein-DndB-[Burkholderia-lata]	482957
VWC50763.1	DndB-ParB-nuclease	GCA_902499095.1	377	Bacteria>Proteobacteria>Betaproteobacteria>Burkholderiales>Burkholderiaceae	Burkholderia_lata	hypothetical-protein-BLA15816_07932-[Burkholderia-lata]	482957
WP_059642651.1	DndB-ParB-nuclease	GCF_000970935.1	377	Bacteria>Proteobacteria>Betaproteobacteria>Burkholderiales>Burkholderiaceae	Burkholderia	MULTISPECIES:-DNA-sulfur-modification-protein-DndB-[Burkholderia]	32008
WP_160640057.1	DndB-ParB-nuclease	GCF_020419375.1	377	Bacteria>Proteobacteria>Betaproteobacteria>Burkholderiales>Burkholderiaceae	Burkholderia	MULTISPECIES:-DNA-sulfur-modification-protein-DndB-[Burkholderia]	32008
WP_059875639.1	DndB-ParB-nuclease	GCF_001528425.1	403	Bacteria>Proteobacteria>Betaproteobacteria>Burkholderiales>Burkholderiaceae	Burkholderia_multivorans	DGQHR-domain-containing-protein-[Burkholderia-multivorans]	87883
KVR38311.1	DndB-ParB-nuclease	GCA_001528425.1	403	Bacteria>Proteobacteria>Betaproteobacteria>Burkholderiales>Burkholderiaceae	Burkholderia_multivorans	DNA-phosphorothioation-associated-DGQHR-protein-1-[Burkholderia-multivorans]	87883
WP_105839111.1	DndB-ParB-nuclease	GCF_002981775.1	375	Bacteria>Proteobacteria>Betaproteobacteria>Burkholderiales>Burkholderiaceae	Burkholderia_multivorans	DNA-sulfur-modification-protein-DndB-[Burkholderia-multivorans]	87883
CFD91157.1	DndB-ParB-nuclease	GCA_001327375.1	374	Bacteria>Proteobacteria>Betaproteobacteria>Burkholderiales>Burkholderiaceae	Burkholderia_pseudomallei	DNA-sulfur-modification-protein-DndB-[Burkholderia-pseudomallei]	28450
KAA8764432.1	DndB-ParB-nuclease	GCA_008693285.1	377	Bacteria>Proteobacteria>Betaproteobacteria>Burkholderiales>Burkholderiaceae	Burkholderia_pseudomallei	DNA-sulfur-modification-protein-DndB-[Burkholderia-pseudomallei]	28450
MBG1252219.1	DndB-ParB-nuclease	GCA_015714675.1	369	Bacteria>Proteobacteria>Betaproteobacteria>Burkholderiales>Burkholderiaceae	Burkholderia_pseudomallei	DNA-sulfur-modification-protein-DndB-[Burkholderia-pseudomallei]	28450
MBM5663847.1	DndB-ParB-nuclease	GCA_016881225.1	377	Bacteria>Proteobacteria>Betaproteobacteria>Burkholderiales>Burkholderiaceae	Burkholderia_pseudomallei	DNA-sulfur-modification-protein-DndB-[Burkholderia-pseudomallei]	28450
WP_052147515.1	DndB-ParB-nuclease	GCF_001446035.1	377	Bacteria>Proteobacteria>Betaproteobacteria>Burkholderiales>Burkholderiaceae	Burkholderia_pseudomallei	DNA-sulfur-modification-protein-DndB-[Burkholderia-pseudomallei]	28450
WP_196509127.1	DndB-ParB-nuclease	GCF_015714675.1	369	Bacteria>Proteobacteria>Betaproteobacteria>Burkholderiales>Burkholderiaceae	Burkholderia_pseudomallei	DNA-sulfur-modification-protein-DndB-[Burkholderia-pseudomallei]	28450
MBG1252215.1	DndB-ParB-nuclease	GCA_015714675.1	577	Bacteria>Proteobacteria>Betaproteobacteria>Burkholderiales>Burkholderiaceae	Burkholderia_pseudomallei	hypothetical-protein-[Burkholderia-pseudomallei]	28450
MBM5663838.1	DndB-ParB-nuclease	GCA_016881225.1	575	Bacteria>Proteobacteria>Betaproteobacteria>Burkholderiales>Burkholderiaceae	Burkholderia_pseudomallei	hypothetical-protein-[Burkholderia-pseudomallei]	28450
WP_128972313.1	DndB-ParB-nuclease	GCF_000770495.1	567	Bacteria>Proteobacteria>Betaproteobacteria>Burkholderiales>Burkholderiaceae	Burkholderia_pseudomallei	hypothetical-protein-[Burkholderia-pseudomallei]	28450
WP_143282701.1	DndB-ParB-nuclease	GCF_001446035.1	575	Bacteria>Proteobacteria>Betaproteobacteria>Burkholderiales>Burkholderiaceae	Burkholderia_pseudomallei	hypothetical-protein-[Burkholderia-pseudomallei]	28450
WP_196509116.1	DndB-ParB-nuclease	GCF_015714675.1	577	Bacteria>Proteobacteria>Betaproteobacteria>Burkholderiales>Burkholderiaceae	Burkholderia_pseudomallei	hypothetical-protein-[Burkholderia-pseudomallei]	28450
AIV47941.1	DndB-ParB-nuclease	GCA_000770495.1	374	Bacteria>Proteobacteria>Betaproteobacteria>Burkholderiales>Burkholderiaceae	Burkholderia_pseudomallei_TSV_48	DGQHR-domain-protein-[Burkholderia-pseudomallei-TSV-48]	1435365
AIV48338.1	DndB-ParB-nuclease	GCA_000770495.1	454	Bacteria>Proteobacteria>Betaproteobacteria>Burkholderiales>Burkholderiaceae	Burkholderia_pseudomallei_TSV_48	hypothetical-protein-X988_3080-[Burkholderia-pseudomallei-TSV-48]	1435365
CFD91140.1	DndB-ParB-nuclease	GCA_001327375.1	454	Bacteria>Proteobacteria>Betaproteobacteria>Burkholderiales>Burkholderiaceae	Burkholderia_pseudomallei	Uncharacterised-protein-[Burkholderia-pseudomallei]	28450
WP_099315881.1	DndB-ParB-nuclease	GCF_002223215.2	474	Bacteria>Proteobacteria>Betaproteobacteria>Burkholderiales>Burkholderiaceae	Burkholderia_sp._AU18528	DGQHR-domain-containing-protein-[Burkholderia-sp.-AU18528]	2015350
PHP91275.1	DndB-ParB-nuclease	GCA_002223215.2	360	Bacteria>Proteobacteria>Betaproteobacteria>Burkholderiales>Burkholderiaceae	Burkholderia_sp._AU18528	DNA-sulfur-modification-protein-DndB-[Burkholderia-sp.-AU18528]	2015350
WP_099315889.1	DndB-ParB-nuclease	GCF_002223215.2	360	Bacteria>Proteobacteria>Betaproteobacteria>Burkholderiales>Burkholderiaceae	Burkholderia_sp._AU18528	DNA-sulfur-modification-protein-DndB-[Burkholderia-sp.-AU18528]	2015350
PHP91267.1	DndB-ParB-nuclease	GCA_002223215.2	474	Bacteria>Proteobacteria>Betaproteobacteria>Burkholderiales>Burkholderiaceae	Burkholderia_sp._AU18528	hypothetical-protein-CFB52_000025-[Burkholderia-sp.-AU18528]	2015350
WP_089488104.1	DndB-ParB-nuclease	GCF_002223185.1	374	Bacteria>Proteobacteria>Betaproteobacteria>Burkholderiales>Burkholderiaceae	Burkholderia_sp._AU31280	DNA-sulfur-modification-protein-DndB-[Burkholderia-sp.-AU31280]	2015353
WP_143328863.1	DndB-ParB-nuclease	GCF_002223185.1	567	Bacteria>Proteobacteria>Betaproteobacteria>Burkholderiales>Burkholderiaceae	Burkholderia_sp._AU31280	hypothetical-protein-[Burkholderia-sp.-AU31280]	2015353
WP_176051384.1	DndB-ParB-nuclease	GCF_902833225.1	377	Bacteria>Proteobacteria>Betaproteobacteria>Burkholderiales>Burkholderiaceae	Burkholderia_sp._BCC1644	DNA-sulfur-modification-protein-DndB-[Burkholderia-sp.-BCC1644]	2676293
WP_124507759.1	DndB-ParB-nuclease	-	377	Bacteria>Proteobacteria>Betaproteobacteria>Burkholderiales>Burkholderiaceae	Burkholderia_sp._Bp9125	DNA-sulfur-modification-protein-DndB-[Burkholderia-sp.-Bp9125]	2184569
WP_124507568.1	DndB-ParB-nuclease	GCF_003853435.1	749	Bacteria>Proteobacteria>Betaproteobacteria>Burkholderiales>Burkholderiaceae	Burkholderia_sp._Bp9125	hypothetical-protein-[Burkholderia-sp.-Bp9125]	2184569
NBI47963.1	DndB-ParB-nuclease	GCA_009910715.1	377	Bacteria>Proteobacteria>Betaproteobacteria>Burkholderiales>Burkholderiaceae	Burkholderia_sp._ISTR5	DNA-sulfur-modification-protein-DndB-[Burkholderia-sp.-ISTR5]	2500161
WP_081063214.1	DndB-ParB-nuclease	GCF_001527645.1	374	Bacteria>Proteobacteria>Betaproteobacteria>Burkholderiales>Burkholderiaceae	Burkholderia_stagnalis	DNA-sulfur-modification-protein-DndB-[Burkholderia-stagnalis]	1503054
WP_081086856.1	DndB-ParB-nuclease	GCF_001533575.1	374	Bacteria>Proteobacteria>Betaproteobacteria>Burkholderiales>Burkholderiaceae	Burkholderia_stagnalis	DNA-sulfur-modification-protein-DndB-[Burkholderia-stagnalis]	1503054
WP_081060320.1	DndB-ParB-nuclease	GCF_001526805.1	382	Bacteria>Proteobacteria>Betaproteobacteria>Burkholderiales>Burkholderiaceae	Burkholderia_territorii	DGQHR-domain-containing-protein-[Burkholderia-territorii]	1503055
NBJ22092.1	DndB-ParB-nuclease	GCA_009910615.1	377	Bacteria>Proteobacteria>Betaproteobacteria>Burkholderiales>Burkholderiaceae	Burkholderia_thailandensis	DNA-sulfur-modification-protein-DndB-[Burkholderia-thailandensis]	57975
NOK40639.1	DndB-ParB-nuclease	GCA_013122325.1	374	Bacteria>Proteobacteria>Betaproteobacteria>Burkholderiales>Burkholderiaceae	Burkholderia_thailandensis	DNA-sulfur-modification-protein-DndB-[Burkholderia-thailandensis]	57975
WP_237567192.1	DndB-ParB-nuclease	GCF_009910615.1	413	Bacteria>Proteobacteria>Betaproteobacteria>Burkholderiales>Burkholderiaceae	Burkholderia_thailandensis	DNA-sulfur-modification-protein-DndB-[Burkholderia-thailandensis]	57975
NBJ22097.1	DndB-ParB-nuclease	GCA_009910615.1	541	Bacteria>Proteobacteria>Betaproteobacteria>Burkholderiales>Burkholderiaceae	Burkholderia_thailandensis	hypothetical-protein-[Burkholderia-thailandensis]	57975
WP_161654661.1	DndB-ParB-nuclease	GCF_009910615.1	541	Bacteria>Proteobacteria>Betaproteobacteria>Burkholderiales>Burkholderiaceae	Burkholderia_thailandensis	hypothetical-protein-[Burkholderia-thailandensis]	57975
KVO85082.1	DndB-ParB-nuclease	GCA_001526875.1	377	Bacteria>Proteobacteria>Betaproteobacteria>Burkholderiales>Burkholderiaceae	Burkholderia_ubonensis	DNA-sulfur-modification-protein-DndB-[Burkholderia-ubonensis]	101571
KVU54539.1	DndB-ParB-nuclease	GCA_001530285.1	360	Bacteria>Proteobacteria>Betaproteobacteria>Burkholderiales>Burkholderiaceae	Burkholderia_ubonensis	DNA-sulfur-modification-protein-DndB-[Burkholderia-ubonensis]	101571
KWE72374.1	DndB-ParB-nuclease	GCA_001532805.1	360	Bacteria>Proteobacteria>Betaproteobacteria>Burkholderiales>Burkholderiaceae	Burkholderia_ubonensis	DNA-sulfur-modification-protein-DndB-[Burkholderia-ubonensis]	101571
WP_059706179.1	DndB-ParB-nuclease	GCF_001523425.1	360	Bacteria>Proteobacteria>Betaproteobacteria>Burkholderiales>Burkholderiaceae	Burkholderia_ubonensis	DNA-sulfur-modification-protein-DndB-[Burkholderia-ubonensis]	101571
WP_060153576.1	DndB-ParB-nuclease	GCF_001530285.1	360	Bacteria>Proteobacteria>Betaproteobacteria>Burkholderiales>Burkholderiaceae	Burkholderia_ubonensis	DNA-sulfur-modification-protein-DndB-[Burkholderia-ubonensis]	101571
WP_060239631.1	DndB-ParB-nuclease	GCF_001531785.1	377	Bacteria>Proteobacteria>Betaproteobacteria>Burkholderiales>Burkholderiaceae	Burkholderia_ubonensis	DNA-sulfur-modification-protein-DndB-[Burkholderia-ubonensis]	101571
WP_060337667.1	DndB-ParB-nuclease	GCF_001532805.1	360	Bacteria>Proteobacteria>Betaproteobacteria>Burkholderiales>Burkholderiaceae	Burkholderia_ubonensis	DNA-sulfur-modification-protein-DndB-[Burkholderia-ubonensis]	101571
WP_071754716.1	DndB-ParB-nuclease	GCF_001882745.1	374	Bacteria>Proteobacteria>Betaproteobacteria>Burkholderiales>Burkholderiaceae	Burkholderia_ubonensis	DNA-sulfur-modification-protein-DndB-[Burkholderia-ubonensis]	101571
WP_232437671.1	DndB-ParB-nuclease	GCF_001526875.1	404	Bacteria>Proteobacteria>Betaproteobacteria>Burkholderiales>Burkholderiaceae	Burkholderia_ubonensis	DNA-sulfur-modification-protein-DndB-[Burkholderia-ubonensis]	101571
WP_232441673.1	DndB-ParB-nuclease	GCF_001525855.1	404	Bacteria>Proteobacteria>Betaproteobacteria>Burkholderiales>Burkholderiaceae	Burkholderia_ubonensis	DNA-sulfur-modification-protein-DndB-[Burkholderia-ubonensis]	101571
WP_232442450.1	DndB-ParB-nuclease	GCF_001527165.1	404	Bacteria>Proteobacteria>Betaproteobacteria>Burkholderiales>Burkholderiaceae	Burkholderia_ubonensis	DNA-sulfur-modification-protein-DndB-[Burkholderia-ubonensis]	101571
WP_071732995.1	DndB-ParB-nuclease	GCF_001527165.1	748	Bacteria>Proteobacteria>Betaproteobacteria>Burkholderiales>Burkholderiaceae	Burkholderia_ubonensis	hypothetical-protein-[Burkholderia-ubonensis]	101571
WP_080433201.1	DndB-ParB-nuclease	GCF_001532805.1	685	Bacteria>Proteobacteria>Betaproteobacteria>Burkholderiales>Burkholderiaceae	Burkholderia_ubonensis	hypothetical-protein-[Burkholderia-ubonensis]	101571
WP_157655045.1	DndB-ParB-nuclease	GCF_001531785.1	541	Bacteria>Proteobacteria>Betaproteobacteria>Burkholderiales>Burkholderiaceae	Burkholderia_ubonensis	hypothetical-protein-[Burkholderia-ubonensis]	101571
WP_059623949.1	DndB-ParB-nuclease	GCF_001524025.1	403	Bacteria>Proteobacteria>Betaproteobacteria>Burkholderiales>Burkholderiaceae	Burkholderia_vietnamiensis	DGQHR-domain-containing-protein-[Burkholderia-vietnamiensis]	60552
KVF26149.1	DndB-ParB-nuclease	GCA_001524025.1	403	Bacteria>Proteobacteria>Betaproteobacteria>Burkholderiales>Burkholderiaceae	Burkholderia_vietnamiensis	DNA-phosphorothioation-associated-DGQHR-protein-1-[Burkholderia-vietnamiensis]	60552
KXV06432.1	DndB-ParB-nuclease	GCA_001580565.1	351	Bacteria>Proteobacteria>Betaproteobacteria>Burkholderiales>Burkholderiaceae	Caballeronia_megalochromosomata	DNA-sulfur-modification-protein-DndB-[Caballeronia-megalochromosomata]	1399969
KXV06419.1	DndB-ParB-nuclease	GCA_001580565.1	684	Bacteria>Proteobacteria>Betaproteobacteria>Burkholderiales>Burkholderiaceae	Caballeronia_megalochromosomata	hypothetical-protein-CR51_11890-[Caballeronia-megalochromosomata]	1399969
WP_047895324.1	DndB-ParB-nuclease	GCF_001028175.1	450	Bacteria>Proteobacteria>Betaproteobacteria>Burkholderiales>Burkholderiaceae	Caballeronia_mineralivorans	DGQHR-domain-containing-protein-[Caballeronia-mineralivorans]	2010198
WP_039406208.1	DndB-ParB-nuclease	GCF_000815105.2	421	Bacteria>Proteobacteria>Betaproteobacteria>Burkholderiales>Burkholderiaceae	Pandoraea_pulmonicola	DGQHR-domain-containing-protein-[Pandoraea-pulmonicola]	93221
WP_179707531.1	DndB-ParB-nuclease	GCF_013408935.1	375	Bacteria>Proteobacteria>Betaproteobacteria>Burkholderiales>Burkholderiaceae	Paraburkholderia_bryophila	DNA-sulfur-modification-protein-DndB-[Paraburkholderia-bryophila]	420952
WP_179707537.1	DndB-ParB-nuclease	GCF_013408935.1	575	Bacteria>Proteobacteria>Betaproteobacteria>Burkholderiales>Burkholderiaceae	Paraburkholderia_bryophila	hypothetical-protein-[Paraburkholderia-bryophila]	420952
WP_134166990.1	DndB-ParB-nuclease	GCF_004365245.1	416	Bacteria>Proteobacteria>Betaproteobacteria>Burkholderiales>Burkholderiaceae	Paraburkholderia_caballeronis	DNA-phosphorothioation-associated-DGQHR-protein-1-[Paraburkholderia-caballeronis]	416943
WP_114196332.1	DndB-ParB-nuclease	GCF_003330745.1	375	Bacteria>Proteobacteria>Betaproteobacteria>Burkholderiales>Burkholderiaceae	Paraburkholderia_caledonica	DNA-sulfur-modification-protein-DndB-[Paraburkholderia-caledonica]	134536
WP_114196323.1	DndB-ParB-nuclease	GCF_003330745.1	557	Bacteria>Proteobacteria>Betaproteobacteria>Burkholderiales>Burkholderiaceae	Paraburkholderia_caledonica	hypothetical-protein-[Paraburkholderia-caledonica]	134536
WP_114196327.1	DndB-ParB-nuclease	GCF_003330745.1	656	Bacteria>Proteobacteria>Betaproteobacteria>Burkholderiales>Burkholderiaceae	Paraburkholderia_caledonica	hypothetical-protein-[Paraburkholderia-caledonica]	134536
WP_201140056.1	DndB-ParB-nuclease	GCF_905220725.1	375	Bacteria>Proteobacteria>Betaproteobacteria>Burkholderiales>Burkholderiaceae	Paraburkholderia_domus	MULTISPECIES:-DNA-sulfur-modification-protein-DndB-[Burkholderiaceae]	2793075
WP_084068893.1	DndB-ParB-nuclease	GCF_000739775.1	378	Bacteria>Proteobacteria>Betaproteobacteria>Burkholderiales>Burkholderiaceae	Paraburkholderia_heleia	DGQHR-domain-containing-protein-[Paraburkholderia-heleia]	634127
TCK42394.1	DndB-ParB-nuclease	GCA_004339005.1	384	Bacteria>Proteobacteria>Betaproteobacteria>Burkholderiales>Burkholderiaceae	Paraburkholderia_sp._BL8N3	DGQHR-domain-containing-protein-[Paraburkholderia-sp.-BL8N3]	1938808
SDQ80177.1	DndB-ParB-nuclease	GCA_900101795.1	374	Bacteria>Proteobacteria>Betaproteobacteria>Burkholderiales>Burkholderiaceae	Paraburkholderia_tuberum	DGQHR-domain-containing-protein-[Paraburkholderia-tuberum]	157910
WP_167368615.1	DndB-ParB-nuclease	GCF_902833905.1	353	Bacteria>Proteobacteria>Betaproteobacteria>Burkholderiales>Burkholderiaceae	Paraburkholderia_tuberum	DGQHR-domain-containing-protein-[Paraburkholderia-tuberum]	157910
SDQ80041.1	DndB-ParB-nuclease	GCA_900101795.1	749	Bacteria>Proteobacteria>Betaproteobacteria>Burkholderiales>Burkholderiaceae	Paraburkholderia_tuberum	DNA-sulfur-modification-associated-[Paraburkholderia-tuberum]	157910
SDQ80307.1	DndB-ParB-nuclease	GCA_900101795.1	375	Bacteria>Proteobacteria>Betaproteobacteria>Burkholderiales>Burkholderiaceae	Paraburkholderia_tuberum	DNA-sulfur-modification-protein-DndB-[Paraburkholderia-tuberum]	157910
WP_090802708.1	DndB-ParB-nuclease	GCF_902833905.1	375	Bacteria>Proteobacteria>Betaproteobacteria>Burkholderiales>Burkholderiaceae	Paraburkholderia_tuberum	DNA-sulfur-modification-protein-DndB-[Paraburkholderia-tuberum]	157910
MBT8555877.1	DndB-ParB-nuclease	GCA_018705195.1	363	Bacteria>Proteobacteria>Betaproteobacteria>Burkholderiales>Burkholderiaceae	Polynucleobacter_paneuropaeus	DNA-sulfur-modification-protein-DndB-[Polynucleobacter-paneuropaeus]	2527775
QWD40268.1	DndB-ParB-nuclease	GCA_018685155.1	363	Bacteria>Proteobacteria>Betaproteobacteria>Burkholderiales>Burkholderiaceae	Polynucleobacter_paneuropaeus	DNA-sulfur-modification-protein-DndB-[Polynucleobacter-paneuropaeus]	2527775
WP_216162824.1	DndB-ParB-nuclease	GCF_018881885.1	363	Bacteria>Proteobacteria>Betaproteobacteria>Burkholderiales>Burkholderiaceae	Polynucleobacter_sp._MWH-Creno-3A4	DNA-sulfur-modification-protein-DndB-[Polynucleobacter-sp.-MWH-Creno-3A4]	1855886
WP_080315071.1	DndB-ParB-nuclease	GCF_001327375.1	374	Bacteria>Proteobacteria>Betaproteobacteria>Burkholderiales>Burkholderiaceae	pseudomallei_group	MULTISPECIES:-DNA-sulfur-modification-protein-DndB-[pseudomallei-group]	111527
WP_154392097.1	DndB-ParB-nuclease	GCF_001327375.1	567	Bacteria>Proteobacteria>Betaproteobacteria>Burkholderiales>Burkholderiaceae	pseudomallei_group	MULTISPECIES:-hypothetical-protein-[pseudomallei-group]	111527
MBB0025845.1	DndB-ParB-nuclease	GCA_014142205.1	403	Bacteria>Proteobacteria>Betaproteobacteria>Burkholderiales>Burkholderiaceae	Ralstonia_pickettii	DGQHR-domain-containing-protein-[Ralstonia-pickettii]	329
WP_182594579.1	DndB-ParB-nuclease	GCF_019642915.1	403	Bacteria>Proteobacteria>Betaproteobacteria>Burkholderiales>Burkholderiaceae	Ralstonia_pickettii	DGQHR-domain-containing-protein-[Ralstonia-pickettii]	329
WP_167801121.1	DndB-ParB-nuclease	GCF_013306395.1	403	Bacteria>Proteobacteria>Betaproteobacteria>Burkholderiales>Burkholderiaceae	Ralstonia_solanacearum	DGQHR-domain-containing-protein-[Ralstonia-solanacearum]	305
WP_085229961.1	DndB-ParB-nuclease	GCF_902833925.1	375	Bacteria>Proteobacteria>Betaproteobacteria>Burkholderiales>Burkholderiaceae	Trinickia_caryophylli	DNA-sulfur-modification-protein-DndB-[Trinickia-caryophylli]	28094
WP_085229966.1	DndB-ParB-nuclease	GCF_902833925.1	568	Bacteria>Proteobacteria>Betaproteobacteria>Burkholderiales>Burkholderiaceae	Trinickia_caryophylli	hypothetical-protein-[Trinickia-caryophylli]	28094
WP_207002087.1	DndB-ParB-nuclease	GCF_017315705.1	377	Bacteria>Proteobacteria>Betaproteobacteria>Burkholderiales>Burkholderiaceae	Trinickia_sp._DHG64	DNA-sulfur-modification-protein-DndB-[Trinickia-sp.-DHG64]	2816356
WP_109033749.1	DndB-ParB-nuclease	GCF_003100395.1	377	Bacteria>Proteobacteria>Betaproteobacteria>Burkholderiales>Burkholderiales_genera_incertae_sedis	Aquabacterium_olei	DNA-sulfur-modification-protein-DndB-[Aquabacterium-olei]	1296669
MBE7417372.1	DndB-ParB-nuclease	GCA_015075165.1	268	Bacteria>Proteobacteria>Betaproteobacteria>Burkholderiales>Burkholderiales_genera_incertae_sedis	Ideonella_sp.	DGQHR-domain-containing-protein-[Ideonella-sp.]	1929293
MBE7417380.1	DndB-ParB-nuclease	GCA_015075165.1	374	Bacteria>Proteobacteria>Betaproteobacteria>Burkholderiales>Burkholderiales_genera_incertae_sedis	Ideonella_sp.	DNA-sulfur-modification-protein-DndB-[Ideonella-sp.]	1929293
WP_084453283.1	DndB-ParB-nuclease	GCF_001598255.1	376	Bacteria>Proteobacteria>Betaproteobacteria>Burkholderiales>Burkholderiales_genera_incertae_sedis	Mitsuaria_chitosanitabida	DNA-sulfur-modification-protein-DndB-[Mitsuaria-chitosanitabida]	65048
WP_084453284.1	DndB-ParB-nuclease	GCF_001598255.1	661	Bacteria>Proteobacteria>Betaproteobacteria>Burkholderiales>Burkholderiales_genera_incertae_sedis	Mitsuaria_chitosanitabida	hypothetical-protein-[Mitsuaria-chitosanitabida]	65048
WP_128198324.1	DndB-ParB-nuclease	GCF_004016515.1	408	Bacteria>Proteobacteria>Betaproteobacteria>Burkholderiales>Burkholderiales_genera_incertae_sedis	Rubrivivax_albus	DNA-phosphorothioation-associated-DGQHR-protein-1-[Rubrivivax-albus]	2499835
MBK1687759.1	DndB-ParB-nuclease	GCA_016583525.1	365	Bacteria>Proteobacteria>Betaproteobacteria>Burkholderiales>Burkholderiales_genera_incertae_sedis	Rubrivivax_gelatinosus	DNA-sulfur-modification-protein-DndB-[Rubrivivax-gelatinosus]	28068
WP_132645877.1	DndB-ParB-nuclease	GCF_016583525.1	365	Bacteria>Proteobacteria>Betaproteobacteria>Burkholderiales>Burkholderiales_genera_incertae_sedis	Rubrivivax_gelatinosus	DNA-sulfur-modification-protein-DndB-[Rubrivivax-gelatinosus]	28068
WP_107177699.1	DndB-ParB-nuclease	GCF_003029805.1	403	Bacteria>Proteobacteria>Betaproteobacteria>Burkholderiales>Comamonadaceae	Acidovorax_avenae	DGQHR-domain-containing-protein-[Acidovorax-avenae]	80867
AVS61879.1	DndB-ParB-nuclease	GCA_003029805.1	403	Bacteria>Proteobacteria>Betaproteobacteria>Burkholderiales>Comamonadaceae	Acidovorax_avenae_subsp._avenae	DNA-phosphorothioation-associated-DGQHR-protein-1-[Acidovorax-avenae-subsp.-avenae]	80870
WP_210570182.1	DndB-ParB-nuclease	GCF_017939685.1	403	Bacteria>Proteobacteria>Betaproteobacteria>Burkholderiales>Comamonadaceae	Acidovorax_sp._JG5	DGQHR-domain-containing-protein-[Acidovorax-sp.-JG5]	2822718
WP_176888045.1	DndB-ParB-nuclease	GCF_013376875.1	408	Bacteria>Proteobacteria>Betaproteobacteria>Burkholderiales>Comamonadaceae	Acidovorax_sp._JMULE5	DGQHR-domain-containing-protein-[Acidovorax-sp.-JMULE5]	2518343
WP_176888035.1	DndB-ParB-nuclease	GCF_013376875.1	370	Bacteria>Proteobacteria>Betaproteobacteria>Burkholderiales>Comamonadaceae	Acidovorax_sp._JMULE5	DNA-sulfur-modification-protein-DndB-[Acidovorax-sp.-JMULE5]	2518343
ABM43092.1	DndB-ParB-nuclease	GCA_000015545.1	405	Bacteria>Proteobacteria>Betaproteobacteria>Burkholderiales>Comamonadaceae	Acidovorax_sp._JS42	conserved-hypothetical-protein-[Acidovorax-sp.-JS42]	232721
MBK7509479.1	DndB-ParB-nuclease	GCA_016707245.1	123	Bacteria>Proteobacteria>Betaproteobacteria>Burkholderiales>Comamonadaceae	Comamonadaceae_bacterium	DGQHR-domain-containing-protein-[Comamonadaceae-bacterium]	1871071
HBW62212.1	DndB-ParB-nuclease	GCA_003513355.1	375	Bacteria>Proteobacteria>Betaproteobacteria>Burkholderiales>Comamonadaceae	Comamonas_kerstersii	DNA-sulfur-modification-protein-DndB-[Comamonas-kerstersii]	225992
KGG85571.1	DndB-ParB-nuclease	GCA_000761415.1	375	Bacteria>Proteobacteria>Betaproteobacteria>Burkholderiales>Comamonadaceae	Comamonas_thiooxydans	DNA-sulfur-modification-protein-DndB-[Comamonas-thiooxydans]	363952
WP_080745945.1	DndB-ParB-nuclease	GCF_000761415.1	375	Bacteria>Proteobacteria>Betaproteobacteria>Burkholderiales>Comamonadaceae	Comamonas_thiooxydans	DNA-sulfur-modification-protein-DndB-[Comamonas-thiooxydans]	363952
WP_097201854.1	DndB-ParB-nuclease	GCF_900215545.1	404	Bacteria>Proteobacteria>Betaproteobacteria>Burkholderiales>Comamonadaceae	Delftia_acidovorans	DNA-phosphorothioation-associated-DGQHR-protein-1-[Delftia-acidovorans]	80866

Accession	Annotation	GCA	Length	Phyletic.spread	Strain.name	Defline	Species.Taxid
WP_097201848.1	DndB-ParB-nuclease	GCF_900215545.1	369	Bacteria>Proteobacteria>Betaproteobacteria>Burkholderiales>Comamonadaceae	Delftia_acidovorans	DNA-sulfur-modification-protein-DndB-[Delftia-acidovorans]	80866
WP_123691680.1	DndB-ParB-nuclease	GCF_003752215.1	374	Bacteria>Proteobacteria>Betaproteobacteria>Burkholderiales>Comamonadaceae	Delftia_acidovorans	DNA-sulfur-modification-protein-DndB-[Delftia-acidovorans]	80866
MBW8298272.1	DndB-ParB-nuclease	GCA_019459045.1	425	Bacteria>Proteobacteria>Betaproteobacteria>Burkholderiales>Comamonadaceae	Hydrogenophaga_sp.	DGQHR-domain-containing-protein-[Hydrogenophaga-sp.]	1904254
MBW8298282.1	DndB-ParB-nuclease	GCA_019459045.1	370	Bacteria>Proteobacteria>Betaproteobacteria>Burkholderiales>Comamonadaceae	Hydrogenophaga_sp.	DNA-sulfur-modification-protein-DndB-[Hydrogenophaga-sp.]	1904254
WP_086121755.1	DndB-ParB-nuclease	GCF_002127215.1	378	Bacteria>Proteobacteria>Betaproteobacteria>Burkholderiales>Comamonadaceae	Hydrogenophaga_sp._IBVHS2	DNA-sulfur-modification-protein-DndB-[Hydrogenophaga-sp.-IBVHS2]	1985170
WP_083451005.1	DndB-ParB-nuclease	GCF_001270065.2	363	Bacteria>Proteobacteria>Betaproteobacteria>Burkholderiales>Comamonadaceae	Limnohabitans_planktonicus	DNA-sulfur-modification-protein-DndB-[Limnohabitans-planktonicus]	540060
WP_100146931.1	DndB-ParB-nuclease	GCF_002778315.1	365	Bacteria>Proteobacteria>Betaproteobacteria>Burkholderiales>Comamonadaceae	Limnohabitans_sp._JirII-31	DNA-sulfur-modification-protein-DndB-[Limnohabitans-sp.-JirII-31]	1977908
WP_135284309.1	DndB-ParB-nuclease	-	357	Bacteria>Proteobacteria>Betaproteobacteria>Burkholderiales>Comamonadaceae	Ramlibacter_rhizophilus	DNA-sulfur-modification-protein-DndB-[Ramlibacter-rhizophilus]	1781167
WP_206021933.1	DndB-ParB-nuclease	GCF_005876985.1	381	Bacteria>Proteobacteria>Betaproteobacteria>Burkholderiales>Comamonadaceae	Rhodoferax_bucti	DNA-sulfur-modification-protein-DndB-[Rhodoferax-bucti]	2576305
WP_076196779.1	DndB-ParB-nuclease	GCF_001955695.1	372	Bacteria>Proteobacteria>Betaproteobacteria>Burkholderiales>Comamonadaceae	Rhodoferax_koreense	DNA-sulfur-modification-protein-DndB-[Rhodoferax-koreense]	1842727
MBT9507231.1	DndB-ParB-nuclease	GCA_018780565.1	375	Bacteria>Proteobacteria>Betaproteobacteria>Burkholderiales>Comamonadaceae	Rhodoferax_sp.	DGQHR-domain-containing-protein-[Rhodoferax-sp.]	50421
MBT9507235.1	DndB-ParB-nuclease	GCA_018780565.1	374	Bacteria>Proteobacteria>Betaproteobacteria>Burkholderiales>Comamonadaceae	Rhodoferax_sp.	DNA-sulfur-modification-protein-DndB-[Rhodoferax-sp.]	50421
SEF27971.1	DndB-ParB-nuclease	GCA_900107915.1	421	Bacteria>Proteobacteria>Betaproteobacteria>Burkholderiales>Comamonadaceae	Variovorax_sp._NFACC28	DGQHR-domain-containing-protein-[Variovorax-sp.-NFACC28]	1566273
WP_162594410.1	DndB-ParB-nuclease	GCF_901827185.1	411	Bacteria>Proteobacteria>Betaproteobacteria>Burkholderiales>Comamonadaceae	Variovorax_sp._PBL-E5	DGQHR-domain-containing-protein-[Variovorax-sp.-PBL-E5]	434014
VTU38508.1	DndB-ParB-nuclease	GCA_901827185.1	411	Bacteria>Proteobacteria>Betaproteobacteria>Burkholderiales>Comamonadaceae	Variovorax_sp._PBL-E5	DNA-phosphorothioation-associated-DGQHR-protein-1-[Variovorax-sp.-PBL-E5]	434014
VTU38544.1	DndB-ParB-nuclease	GCA_901827185.1	369	Bacteria>Proteobacteria>Betaproteobacteria>Burkholderiales>Comamonadaceae	Variovorax_sp._PBL-E5	DNA-sulfur-modification-protein-DndB-[Variovorax-sp.-PBL-E5]	434014
WP_162594418.1	DndB-ParB-nuclease	GCF_901827185.1	369	Bacteria>Proteobacteria>Betaproteobacteria>Burkholderiales>Comamonadaceae	Variovorax_sp._PBL-E5	DNA-sulfur-modification-protein-DndB-[Variovorax-sp.-PBL-E5]	434014
WP_211473234.1	DndB-ParB-nuclease	GCF_018304905.1	374	Bacteria>Proteobacteria>Betaproteobacteria>Burkholderiales>Oxalobacteraceae	Collimonas_humicola	DNA-sulfur-modification-protein-DndB-[Collimonas-sp.-RXD172-2]	2825886
WP_154363895.1	DndB-ParB-nuclease	GCF_009674525.1	374	Bacteria>Proteobacteria>Betaproteobacteria>Burkholderiales>Oxalobacteraceae	Duganella_alba	MULTISPECIES:-DNA-sulfur-modification-protein-DndB-[Duganella]	2666081
MCC7684056.1	DndB-ParB-nuclease	GCA_020858135.1	371	Bacteria>Proteobacteria>Betaproteobacteria>Burkholderiales>Oxalobacteraceae	Janthinobacterium_sp._FW305-128	DGQHR-domain-containing-protein-[Janthinobacterium-sp.-FW305-128]	2775055
WP_230506427.1	DndB-ParB-nuclease	GCF_020858135.1	371	Bacteria>Proteobacteria>Betaproteobacteria>Burkholderiales>Oxalobacteraceae	Janthinobacterium_sp._FW305-128	DGQHR-domain-containing-protein-[Janthinobacterium-sp.-FW305-128]	2775055
MCC7684060.1	DndB-ParB-nuclease	GCA_020858135.1	366	Bacteria>Proteobacteria>Betaproteobacteria>Burkholderiales>Oxalobacteraceae	Janthinobacterium_sp._FW305-128	DNA-sulfur-modification-protein-DndB-[Janthinobacterium-sp.-FW305-128]	2775055
WP_230506431.1	DndB-ParB-nuclease	GCF_020858135.1	366	Bacteria>Proteobacteria>Betaproteobacteria>Burkholderiales>Oxalobacteraceae	Janthinobacterium_sp._FW305-128	DNA-sulfur-modification-protein-DndB-[Janthinobacterium-sp.-FW305-128]	2775055
WP_078031358.1	DndB-ParB-nuclease	GCF_002007115.1	365	Bacteria>Proteobacteria>Betaproteobacteria>Burkholderiales>Oxalobacteraceae	Massilia_sp._KIM	DGQHR-domain-containing-protein-[Massilia-sp.-KIM]	1955422
OON62701.1	DndB-ParB-nuclease	GCA_002007115.1	365	Bacteria>Proteobacteria>Betaproteobacteria>Burkholderiales>Oxalobacteraceae	Massilia_sp._KIM	DNA-sulfur-modification-protein-DndB-[Massilia-sp.-KIM]	1955422
WP_078031353.1	DndB-ParB-nuclease	GCF_002007115.1	365	Bacteria>Proteobacteria>Betaproteobacteria>Burkholderiales>Oxalobacteraceae	Massilia_sp._KIM	DNA-sulfur-modification-protein-DndB-[Massilia-sp.-KIM]	1955422
MVW64163.1	DndB-ParB-nuclease	GCA_009763355.1	365	Bacteria>Proteobacteria>Betaproteobacteria>Burkholderiales>Oxalobacteraceae	Massilia_sp._NEAU-DD11	DNA-sulfur-modification-protein-DndB-[Massilia-sp.-NEAU-DD11]	2683234
WP_160410683.1	DndB-ParB-nuclease	GCF_009763355.1	365	Bacteria>Proteobacteria>Betaproteobacteria>Burkholderiales>Oxalobacteraceae	Massilia_sp._NEAU-DD11	DNA-sulfur-modification-protein-DndB-[Massilia-sp.-NEAU-DD11]	2683234
MBD8566235.1	DndB-ParB-nuclease	GCA_014841525.1	407	Bacteria>Proteobacteria>Betaproteobacteria>Burkholderiales>Oxalobacteraceae	Oxalobacteraceae_sp._CFBP_8763	DGQHR-domain-containing-protein-[Oxalobacteraceae-sp.-CFBP-8763]	2775278
MBD8566233.1	DndB-ParB-nuclease	GCA_014841525.1	363	Bacteria>Proteobacteria>Betaproteobacteria>Burkholderiales>Oxalobacteraceae	Oxalobacteraceae_sp._CFBP_8763	DNA-sulfur-modification-protein-DndB-[Oxalobacteraceae-sp.-CFBP-8763]	2775278
WP_217431307.1	DndB-ParB-nuclease	GCF_009857645.1	423	Bacteria>Proteobacteria>Betaproteobacteria>Burkholderiales>Oxalobacteraceae	Rugamonas_sp._FT82W	DNA-phosphorothioation-associated-DGQHR-protein-1-[Rugamonas-sp.-FT82W]	2692167
MBI3102176.1	DndB-ParB-nuclease	GCA_016192425.1	375	Bacteria>Proteobacteria>Betaproteobacteria>Burkholderiales>unclassified_Burkholderiales	Burkholderiales_bacterium	DNA-sulfur-modification-protein-DndB-[Burkholderiales-bacterium]	1891238
MBX3650511.1	DndB-ParB-nuclease	GCA_019635015.1	375	Bacteria>Proteobacteria>Betaproteobacteria>Burkholderiales>unclassified_Burkholderiales	Burkholderiales_bacterium	DNA-sulfur-modification-protein-DndB-[Burkholderiales-bacterium]	1891238
PZO20995.1	DndB-ParB-nuclease	GCA_003241965.1	421	Bacteria>Proteobacteria>Betaproteobacteria>Burkholderiales>unclassified_Burkholderiales	Burkholderiales_bacterium	hypothetical-protein-DCF26_01570-[Burkholderiales-bacterium]	1891238
WP_081950392.1	DndB-ParB-nuclease	GCF_000758475.1	373	Bacteria>Proteobacteria>Betaproteobacteria>Neisseriales>Chromobacteriaceae	Chromobacterium_haemolyticum	DNA-sulfur-modification-protein-DndB-[Chromobacterium-haemolyticum]	394935
WP_043592541.1	DndB-ParB-nuclease	GCF_000758475.1	569	Bacteria>Proteobacteria>Betaproteobacteria>Neisseriales>Chromobacteriaceae	Chromobacterium_haemolyticum	hypothetical-protein-[Chromobacterium-haemolyticum]	394935
WP_052257894.1	DndB-ParB-nuclease	GCF_019460125.1	350	Bacteria>Proteobacteria>Betaproteobacteria>Neisseriales>Chromobacteriaceae	Chromobacterium	MULTISPECIES:-DNA-sulfur-modification-protein-DndB-[Chromobacterium]	535
WP_080770129.1	DndB-ParB-nuclease	GCF_019446955.1	653	Bacteria>Proteobacteria>Betaproteobacteria>Neisseriales>Chromobacteriaceae	Chromobacterium	MULTISPECIES:-hypothetical-protein-[Chromobacterium]	535
WP_081541823.1	DndB-ParB-nuclease	GCF_002081735.1	436	Bacteria>Proteobacteria>Betaproteobacteria>Neisseriales>Chromobacteriaceae	Chromobacterium_violaceum	DGQHR-domain-containing-protein-[Chromobacterium-violaceum]	536
WP_112692732.1	DndB-ParB-nuclease	GCF_003269145.1	374	Bacteria>Proteobacteria>Betaproteobacteria>Neisseriales>Chromobacteriaceae	Microvirgula_sp._AG722	DNA-sulfur-modification-protein-DndB-[Microvirgula-sp.-AG722]	2183901
WP_188706549.1	DndB-ParB-nuclease	GCF_014645535.1	367	Bacteria>Proteobacteria>Betaproteobacteria>Neisseriales>Chromobacteriaceae	Silvimonas_iriomotensis	DGQHR-domain-containing-protein-[Silvimonas-iriomotensis]	449662
WP_188706556.1	DndB-ParB-nuclease	GCF_014645535.1	374	Bacteria>Proteobacteria>Betaproteobacteria>Neisseriales>Chromobacteriaceae	Silvimonas_iriomotensis	DNA-sulfur-modification-protein-DndB-[Silvimonas-iriomotensis]	449662
WP_227301939.1	DndB-ParB-nuclease	GCF_020616155.1	368	Bacteria>Proteobacteria>Betaproteobacteria>Neisseriales>Chromobacteriaceae	Vogesella_sp._XCS3	DNA-sulfur-modification-protein-DndB-[Vogesella-sp.-XCS3]	2877939
RFC31744.1	DndB-ParB-nuclease	GCA_003402285.1	366	Bacteria>Proteobacteria>Betaproteobacteria>Nitrosomonadales>Gallionellaceae	Candidatus_Nitrotoga_sp._SPKER	DGQHR-domain-containing-protein-[Candidatus-Nitrotoga-sp.-SPKER]	2184312
RFC31749.1	DndB-ParB-nuclease	GCA_003402285.1	366	Bacteria>Proteobacteria>Betaproteobacteria>Nitrosomonadales>Gallionellaceae	Candidatus_Nitrotoga_sp._SPKER	DNA-sulfur-modification-protein-DndB-[Candidatus-Nitrotoga-sp.-SPKER]	2184312
TNC99129.1	DndB-ParB-nuclease	GCA_006218055.1	420	Bacteria>Proteobacteria>Betaproteobacteria>Nitrosomonadales>Gallionellaceae	Gallionellaceae_bacterium	hypothetical-protein-FD121_367-[Gallionellaceae-bacterium]	2052162
TNC99133.1	DndB-ParB-nuclease	GCA_006218055.1	363	Bacteria>Proteobacteria>Betaproteobacteria>Nitrosomonadales>Gallionellaceae	Gallionellaceae_bacterium	hypothetical-protein-FD121_371-[Gallionellaceae-bacterium]	2052162
WP_226772647.1	DndB-ParB-nuclease	GCF_020532675.1	409	Bacteria>Proteobacteria>Betaproteobacteria>Nitrosomonadales>Methylophilaceae	Methylobacillus_gramineus	unnamed-protein-product	755169
WP_226772653.1	DndB-ParB-nuclease	GCF_020532675.1	367	Bacteria>Proteobacteria>Betaproteobacteria>Nitrosomonadales>Methylophilaceae	Methylobacillus_gramineus	unnamed-protein-product	755169
MBL6791370.1	DndB-ParB-nuclease	GCA_016778485.1	366	Bacteria>Proteobacteria>Betaproteobacteria>Nitrosomonadales>Methylophilaceae	Methylophilaceae_bacterium	DNA-sulfur-modification-protein-DndB-[Methylophilaceae-bacterium]	2030816
MBX9636985.1	DndB-ParB-nuclease	GCA_019748355.1	363	Bacteria>Proteobacteria>Betaproteobacteria>Nitrosomonadales>Nitrosomonadaceae	Nitrosomonas_sp.	DNA-sulfur-modification-protein-DndB-[Nitrosomonas-sp.]	42353
MXS76826.1	DndB-ParB-nuclease	GCA_009833095.1	363	Bacteria>Proteobacteria>Betaproteobacteria>Nitrosomonadales>Nitrosomonadaceae	Nitrosomonas_sp._JL21	DNA-sulfur-modification-protein-DndB-[Nitrosomonas-sp.-JL21]	153949
WP_240143484.1	DndB-ParB-nuclease	GCF_009833095.1	338	Bacteria>Proteobacteria>Betaproteobacteria>Nitrosomonadales>Nitrosomonadaceae	Nitrosomonas_sp._JL21	DNA-sulfur-modification-protein-DndB-[Nitrosomonas-sp.-JL21]	153949
WP_107692181.1	DndB-ParB-nuclease	GCF_003050965.1	405	Bacteria>Proteobacteria>Betaproteobacteria>Nitrosomonadales>Nitrosomonadaceae	Nitrosospira_sp._Nsp2	DNA-phosphorothioation-associated-DGQHR-protein-1-[Nitrosospira-sp.-Nsp2]	136548
WP_081629071.1	DndB-ParB-nuclease	GCF_000385375.1	431	Bacteria>Proteobacteria>Betaproteobacteria>Nitrosomonadales>Sterolibacteriaceae	Methyloversatilis_discipulorum	DGQHR-domain-containing-protein-[Methyloversatilis-discipulorum]	1119528
WP_198291208.1	DndB-ParB-nuclease	GCF_000385375.1	376	Bacteria>Proteobacteria>Betaproteobacteria>Nitrosomonadales>Sterolibacteriaceae	Methyloversatilis_discipulorum	DNA-sulfur-modification-protein-DndB-[Methyloversatilis-discipulorum]	1119528
WP_009206139.1	DndB-ParB-nuclease	GCF_000297055.2	528	Bacteria>Proteobacteria>Betaproteobacteria>Nitrosomonadales>Sulfuricellaceae	Sulfuricella_denitrificans	DGQHR-domain-containing-protein-[Sulfuricella-denitrificans]	649841
WP_009206136.1	DndB-ParB-nuclease	GCF_000297055.2	362	Bacteria>Proteobacteria>Betaproteobacteria>Nitrosomonadales>Sulfuricellaceae	Sulfuricella_denitrificans	DNA-sulfur-modification-protein-DndB-[Sulfuricella-denitrificans]	649841
MBN8780910.1	DndB-ParB-nuclease	GCA_017304205.1	418	Bacteria>Proteobacteria>Betaproteobacteria>Nitrosomonadales>Thiobacillaceae	Thiobacillus_sp.	DGQHR-domain-containing-protein-[Thiobacillus-sp.]	924
MBN8780915.1	DndB-ParB-nuclease	GCA_017304205.1	374	Bacteria>Proteobacteria>Betaproteobacteria>Nitrosomonadales>Thiobacillaceae	Thiobacillus_sp.	DNA-sulfur-modification-protein-DndB-[Thiobacillus-sp.]	924
MBI4937547.1	DndB-ParB-nuclease	GCA_016213335.1	407	Bacteria>Proteobacteria>Betaproteobacteria>Nitrosomonadales>unclassified_Nitrosomonadales	Nitrosomonadales_bacterium	DGQHR-domain-containing-protein-[Nitrosomonadales-bacterium]	2026761
MBI4807743.1	DndB-ParB-nuclease	GCA_016208015.1	366	Bacteria>Proteobacteria>Betaproteobacteria>Nitrosomonadales>unclassified_Nitrosomonadales	Nitrosomonadales_bacterium	DNA-sulfur-modification-protein-DndB-[Nitrosomonadales-bacterium]	2026761
MBS4018171.1	DndB-ParB-nuclease	GCA_018335835.1	375	Bacteria>Proteobacteria>Betaproteobacteria>Rhodocyclales>Azonexaceae	Dechloromonas_sp.	DNA-sulfur-modification-protein-DndB-[Dechloromonas-sp.]	1917218
WP_004337195.1	DndB-ParB-nuclease	GCF_000310205.1	357	Bacteria>Proteobacteria>Betaproteobacteria>Rhodocyclales>Zoogloeaceae	Thauera_linaloolentis	DNA-sulfur-modification-protein-DndB-[Thauera-linaloolentis]	76112
TAK72450.1	DndB-ParB-nuclease	GCA_004298385.1	427	Bacteria>Proteobacteria>Betaproteobacteria>unclassified_Betaproteobacteria	Betaproteobacteria_bacterium	DGQHR-domain-containing-protein-[Betaproteobacteria-bacterium]	1891241
MBK6631646.1	DndB-ParB-nuclease	GCA_016703785.1	287	Bacteria>Proteobacteria>Betaproteobacteria>unclassified_Betaproteobacteria	Betaproteobacteria_bacterium	hypothetical-protein-[Betaproteobacteria-bacterium]	1891241
KAB2318672.1	DndB-ParB-nuclease	GCA_008923315.1	406	Bacteria>Proteobacteria>Betaproteobacteria>unclassified_Betaproteobacteria	Betaproteobacteria_bacterium_SCN1	DGQHR-domain-containing-protein-[Betaproteobacteria-bacterium-SCN1]	2651627
ACL06476.1	DndB-ParB-nuclease	GCA_000021905.1	364	Bacteria>Proteobacteria>delta/epsilon_subdivisions>Deltaproteobacteria>Desulfobacterales	Desulfatibacillum_aliphaticivorans	DNA-sulfur-modification-protein-DndB-[Desulfatibacillum-aliphaticivorans]	218208
WP_015949515.1	DndB-ParB-nuclease	GCF_000021905.1	364	Bacteria>Proteobacteria>delta/epsilon_subdivisions>Deltaproteobacteria>Desulfobacterales	Desulfatibacillum_aliphaticivorans	DNA-sulfur-modification-protein-DndB-[Desulfatibacillum-aliphaticivorans]	218208
WP_028324750.1	DndB-ParB-nuclease	GCF_000429925.1	361	Bacteria>Proteobacteria>delta/epsilon_subdivisions>Deltaproteobacteria>Desulfobacterales	Desulfatirhabdium_butyrativorans	DNA-sulfur-modification-protein-DndB-[Desulfatirhabdium-butyrativorans]	340467
WP_035257781.1	DndB-ParB-nuclease	GCF_000429925.1	576	Bacteria>Proteobacteria>delta/epsilon_subdivisions>Deltaproteobacteria>Desulfobacterales	Desulfatirhabdium_butyrativorans	hypothetical-protein-[Desulfatirhabdium-butyrativorans]	340467
NQT70524.1	DndB-ParB-nuclease	GCA_013203195.1	413	Bacteria>Proteobacteria>delta/epsilon_subdivisions>Deltaproteobacteria>Desulfobacterales	Desulfobacteraceae_bacterium	DGQHR-domain-containing-protein-[Desulfobacteraceae-bacterium]	2049433
NQT70520.1	DndB-ParB-nuclease	GCA_013203195.1	371	Bacteria>Proteobacteria>delta/epsilon_subdivisions>Deltaproteobacteria>Desulfobacterales	Desulfobacteraceae_bacterium	DNA-sulfur-modification-protein-DndB-[Desulfobacteraceae-bacterium]	2049433
NVM20279.1	DndB-ParB-nuclease	GCA_013375395.1	371	Bacteria>Proteobacteria>delta/epsilon_subdivisions>Deltaproteobacteria>Desulfobacterales	Desulfobacterales_bacterium	DNA-sulfur-modification-protein-DndB-[Desulfobacterales-bacterium]	2044940
MBL6995472.1	DndB-ParB-nuclease	GCA_016785005.1	367	Bacteria>Proteobacteria>delta/epsilon_subdivisions>Deltaproteobacteria>Desulfobacterales	Desulfobacula_sp.	DNA-sulfur-modification-protein-DndB-[Desulfobacula-sp.]	2593537
NTV15724.1	DndB-ParB-nuclease	GCA_013335025.1	409	Bacteria>Proteobacteria>delta/epsilon_subdivisions>Deltaproteobacteria>Desulfobacterales	Desulfobulbaceae_bacterium	DGQHR-domain-containing-protein-[Desulfobulbaceae-bacterium]	2053307
WP_084540480.1	DndB-ParB-nuclease	-	351	Bacteria>Proteobacteria>delta/epsilon_subdivisions>Deltaproteobacteria>Desulfobacterales	Desulfofustis_glycolicus	DNA-sulfur-modification-protein-DndB-[Desulfofustis-glycolicus]	51195
WP_205235778.1	DndB-ParB-nuclease	-	365	Bacteria>Proteobacteria>delta/epsilon_subdivisions>Deltaproteobacteria>Desulfobacterales	Desulfopila_inferna	DNA-sulfur-modification-protein-DndB-[Desulfopila-inferna]	468528
WP_022665363.1	DndB-ParB-nuclease	GCF_000420085.1	426	Bacteria>Proteobacteria>delta/epsilon_subdivisions>Deltaproteobacteria>Desulfobacterales	Desulfospira_joergensenii	DGQHR-domain-containing-protein-[Desulfospira-joergensenii]	53329
WP_022665361.1	DndB-ParB-nuclease	GCF_000420085.1	367	Bacteria>Proteobacteria>delta/epsilon_subdivisions>Deltaproteobacteria>Desulfobacterales	Desulfospira_joergensenii	DNA-sulfur-modification-protein-DndB-[Desulfospira-joergensenii]	53329
WP_014260554.1	DndB-ParB-nuclease	GCF_000212675.2	573	Bacteria>Proteobacteria>delta/epsilon_subdivisions>Deltaproteobacteria>Desulfovibrionales	Desulfocurvibacter_africanus	hypothetical-protein-[Desulfocurvibacter-africanus]	873
WP_084605072.1	DndB-ParB-nuclease	GCF_000934745.1	360	Bacteria>Proteobacteria>delta/epsilon_subdivisions>Deltaproteobacteria>Desulfovibrionales	Desulfonatronum_thioautotrophicum	DNA-sulfur-modification-protein-DndB-[Desulfonatronum-thioautotrophicum]	617001
WP_126378533.1	DndB-ParB-nuclease	GCF_003966735.1	368	Bacteria>Proteobacteria>delta/epsilon_subdivisions>Deltaproteobacteria>Desulfovibrionales	Desulfovibrio_ferrophilus	DNA-sulfur-modification-protein-DndB-[Desulfovibrio-ferrophilus]	241368
WP_051261066.1	DndB-ParB-nuclease	GCF_000429305.1	366	Bacteria>Proteobacteria>delta/epsilon_subdivisions>Deltaproteobacteria>Desulfovibrionales	Desulfovibrio_inopinatus	DNA-sulfur-modification-protein-DndB-[Desulfovibrio-inopinatus]	102109
WP_083909252.1	DndB-ParB-nuclease	GCF_000375485.1	366	Bacteria>Proteobacteria>delta/epsilon_subdivisions>Deltaproteobacteria>Desulfovibrionales	Desulfovibrio_oxyclinae	DNA-sulfur-modification-protein-DndB-[Desulfovibrio-oxyclinae]	63560
WP_026364795.1	DndB-ParB-nuclease	GCF_000384815.1	367	Bacteria>Proteobacteria>delta/epsilon_subdivisions>Deltaproteobacteria>Desulfovibrionales	Halodesulfovibrio_aestuarii	DNA-sulfur-modification-protein-DndB-[Halodesulfovibrio-aestuarii]	126333
MBL3582641.1	DndB-ParB-nuclease	GCA_016756815.1	360	Bacteria>Proteobacteria>delta/epsilon_subdivisions>Deltaproteobacteria>Desulfovibrionales	Oleidesulfovibrio_alaskensis	DNA-sulfur-modification-protein-DndB-[Desulfovibrio-alaskensis]	58180
WP_233814251.1	DndB-ParB-nuclease	GCF_021391355.1	384	Bacteria>Proteobacteria>delta/epsilon_subdivisions>Deltaproteobacteria>Desulfovibrionales	Paradesulfovibrio_bizertensis	DNA-sulfur-modification-protein-DndB-[Desulfovibrio-bizertensis]	376490
WP_026843198.1	DndB-ParB-nuclease	GCF_000426865.1	407	Bacteria>Proteobacteria>delta/epsilon_subdivisions>Deltaproteobacteria>Desulfuromonadales	Citrifermentans_bremense	DGQHR-domain-containing-protein-[Citrifermentans-bremense]	60035
WP_026843199.1	DndB-ParB-nuclease	GCF_000426865.1	364	Bacteria>Proteobacteria>delta/epsilon_subdivisions>Deltaproteobacteria>Desulfuromonadales	Citrifermentans_bremense	DNA-sulfur-modification-protein-DndB-[Citrifermentans-bremense]	60035
WP_225071828.1	DndB-ParB-nuclease	GCF_020194955.2	358	Bacteria>Proteobacteria>delta/epsilon_subdivisions>Deltaproteobacteria>Desulfuromonadales	Desulfuromonas_sp._CSMB_57	DNA-sulfur-modification-protein-DndB-[Desulfuromonas-sp.-CSMB_57]	2807629
WP_145019832.1	DndB-ParB-nuclease	GCF_007830735.1	358	Bacteria>Proteobacteria>delta/epsilon_subdivisions>Deltaproteobacteria>Desulfuromonadales	Geobacter_argillaceus	DNA-sulfur-modification-protein-DndB-[Geobacter-argillaceus]	345631
WP_145019815.1	DndB-ParB-nuclease	GCF_007830735.1	669	Bacteria>Proteobacteria>delta/epsilon_subdivisions>Deltaproteobacteria>Desulfuromonadales	Geobacter_argillaceus	hypothetical-protein-[Geobacter-argillaceus]	345631
WP_173196007.1	DndB-ParB-nuclease	GCF_016865365.1	364	Bacteria>Proteobacteria>delta/epsilon_subdivisions>Deltaproteobacteria>Desulfuromonadales	Geobacter_sp._SVR	DNA-sulfur-modification-protein-DndB-[Geobacter-sp.-SVR]	2495594
BCS52639.1	DndB-ParB-nuclease	GCA_016865365.1	364	Bacteria>Proteobacteria>delta/epsilon_subdivisions>Deltaproteobacteria>Desulfuromonadales	Geobacter_sp._SVR	hypothetical-protein-GSVR_09470-[Geobacter-sp.-SVR]	2495594
WP_216509723.1	DndB-ParB-nuclease	GCF_018919395.1	363	Bacteria>Proteobacteria>delta/epsilon_subdivisions>Deltaproteobacteria>Desulfuromonadales	Geomonas_azotofigens	DNA-sulfur-modification-protein-DndB-[Geomonas-azotofigens]	2843196
WP_216509728.1	DndB-ParB-nuclease	GCF_018919395.1	750	Bacteria>Proteobacteria>delta/epsilon_subdivisions>Deltaproteobacteria>Desulfuromonadales	Geomonas_azotofigens	hypothetical-protein-[Geomonas-azotofigens]	2843196
QWV98231.1	DndB-ParB-nuclease	GCA_018986935.1	326	Bacteria>Proteobacteria>delta/epsilon_subdivisions>Deltaproteobacteria>Desulfuromonadales	Geomonas_nitrogeniifigens	DGQHR-domain-containing-protein-[Geomonas-sp.-RG29]	2847991
WP_216790482.1	DndB-ParB-nuclease	GCF_018986935.1	326	Bacteria>Proteobacteria>delta/epsilon_subdivisions>Deltaproteobacteria>Desulfuromonadales	Geomonas_nitrogeniifigens	DGQHR-domain-containing-protein-[Geomonas-sp.-RG29]	2847991
QWV98226.1	DndB-ParB-nuclease	GCA_018986935.1	364	Bacteria>Proteobacteria>delta/epsilon_subdivisions>Deltaproteobacteria>Desulfuromonadales	Geomonas_nitrogeniifigens	DNA-sulfur-modification-protein-DndB-[Geomonas-sp.-RG29]	2847991
WP_216790472.1	DndB-ParB-nuclease	GCF_018986935.1	364	Bacteria>Proteobacteria>delta/epsilon_subdivisions>Deltaproteobacteria>Desulfuromonadales	Geomonas_nitrogeniifigens	DNA-sulfur-modification-protein-DndB-[Geomonas-sp.-RG29]	2847991
WP_216790485.1	DndB-ParB-nuclease	GCF_018986935.1	578	Bacteria>Proteobacteria>delta/epsilon_subdivisions>Deltaproteobacteria>Desulfuromonadales	Geomonas_nitrogeniifigens	hypothetical-protein-[Geomonas-sp.-RG29]	2847991
QWV98233.1	DndB-ParB-nuclease	GCA_018986935.1	578	Bacteria>Proteobacteria>delta/epsilon_subdivisions>Deltaproteobacteria>Desulfuromonadales	Geomonas_nitrogeniifigens	hypothetical-protein-KP005_02775-[Geomonas-sp.-RG29]	2847991
WP_041245951.1	DndB-ParB-nuclease	GCF_000016745.1	334	Bacteria>Proteobacteria>delta/epsilon_subdivisions>Deltaproteobacteria>Desulfuromonadales	Geotalea_uraniireducens	DNA-sulfur-modification-protein-DndB-[Geobacter-uraniireducens]	351604
ABQ26651.1	DndB-ParB-nuclease	GCA_000016745.1	358	Bacteria>Proteobacteria>delta/epsilon_subdivisions>Deltaproteobacteria>Desulfuromonadales	Geotalea_uraniireducens_Rf4	hypothetical-protein-Gura_2473-[Geotalea-uraniireducens-Rf4]	351605
WP_103116674.1	DndB-ParB-nuclease	GCF_002898515.1	364	Bacteria>Proteobacteria>delta/epsilon_subdivisions>Deltaproteobacteria>Desulfuromonadales	Geothermobacter_hydrogeniphilus	DNA-sulfur-modification-protein-DndB-[Geothermobacter-hydrogeniphilus]	1969733
MBP8808959.1	DndB-ParB-nuclease	GCA_018055015.1	447	Bacteria>Proteobacteria>delta/epsilon_subdivisions>Deltaproteobacteria>Myxococcales	Kofleriaceae_bacterium	DGQHR-domain-containing-protein-[Kofleriaceae-bacterium]	2212474
MBL8680153.1	DndB-ParB-nuclease	GCA_016793325.1	505	Bacteria>Proteobacteria>delta/epsilon_subdivisions>Deltaproteobacteria>Myxococcales	Myxococcales_bacterium	DGQHR-domain-containing-protein-[Myxococcales-bacterium]	2026763
MBN9164484.1	DndB-ParB-nuclease	GCA_017306215.1	567	Bacteria>Proteobacteria>delta/epsilon_subdivisions>Deltaproteobacteria>Myxococcales	Myxococcales_bacterium	hypothetical-protein-[Myxococcales-bacterium]	2026763
MCB9610590.1	DndB-ParB-nuclease	GCA_020633275.1	428	Bacteria>Proteobacteria>delta/epsilon_subdivisions>Deltaproteobacteria>Myxococcales	Polyangiaceae_bacterium	DGQHR-domain-containing-protein-[Polyangiaceae-bacterium]	2268199
HCX01854.1	DndB-ParB-nuclease	GCA_003538085.1	433	Bacteria>Proteobacteria>delta/epsilon_subdivisions>Deltaproteobacteria>Syntrophales	Syntrophaceae_bacterium	DGQHR-domain-containing-protein-[Syntrophaceae-bacterium]	2052184
HCX01858.1	DndB-ParB-nuclease	GCA_003538085.1	360	Bacteria>Proteobacteria>delta/epsilon_subdivisions>Deltaproteobacteria>Syntrophales	Syntrophaceae_bacterium	DNA-sulfur-modification-protein-DndB-[Syntrophaceae-bacterium]	2052184
MBI2388606.1	DndB-ParB-nuclease	GCA_016182965.1	362	Bacteria>Proteobacteria>delta/epsilon_subdivisions>Deltaproteobacteria>unclassified_Deltaproteobacteria	Deltaproteobacteria_bacterium	DGQHR-domain-containing-protein-[Deltaproteobacteria-bacterium]	2026735
MBW1616873.1	DndB-ParB-nuclease	GCA_019306185.1	413	Bacteria>Proteobacteria>delta/epsilon_subdivisions>Deltaproteobacteria>unclassified_Deltaproteobacteria	Deltaproteobacteria_bacterium	DGQHR-domain-containing-protein-[Deltaproteobacteria-bacterium]	2026735
MRR53946.1	DndB-ParB-nuclease	GCA_009668975.1	529	Bacteria>Proteobacteria>delta/epsilon_subdivisions>Deltaproteobacteria>unclassified_Deltaproteobacteria	Deltaproteobacteria_bacterium	DGQHR-domain-containing-protein-[Deltaproteobacteria-bacterium]	2026735
MBI2388618.1	DndB-ParB-nuclease	GCA_016182965.1	372	Bacteria>Proteobacteria>delta/epsilon_subdivisions>Deltaproteobacteria>unclassified_Deltaproteobacteria	Deltaproteobacteria_bacterium	DNA-sulfur-modification-protein-DndB-[Deltaproteobacteria-bacterium]	2026735
MBN2845540.1	DndB-ParB-nuclease	GCA_016938315.1	363	Bacteria>Proteobacteria>delta/epsilon_subdivisions>Deltaproteobacteria>unclassified_Deltaproteobacteria	Deltaproteobacteria_bacterium	DNA-sulfur-modification-protein-DndB-[Deltaproteobacteria-bacterium]	2026735
MBV5341490.1	DndB-ParB-nuclease	GCA_019162745.1	374	Bacteria>Proteobacteria>delta/epsilon_subdivisions>Deltaproteobacteria>unclassified_Deltaproteobacteria	Deltaproteobacteria_bacterium	DNA-sulfur-modification-protein-DndB-[Deltaproteobacteria-bacterium]	2026735
MBZ0235623.1	DndB-ParB-nuclease	GCA_019912685.1	370	Bacteria>Proteobacteria>delta/epsilon_subdivisions>Deltaproteobacteria>unclassified_Deltaproteobacteria	Deltaproteobacteria_bacterium	DNA-sulfur-modification-protein-DndB-[Deltaproteobacteria-bacterium]	2026735
MRR53945.1	DndB-ParB-nuclease	GCA_009668975.1	364	Bacteria>Proteobacteria>delta/epsilon_subdivisions>Deltaproteobacteria>unclassified_Deltaproteobacteria	Deltaproteobacteria_bacterium	DNA-sulfur-modification-protein-DndB-[Deltaproteobacteria-bacterium]	2026735
NQW62585.1	DndB-ParB-nuclease	GCA_013205435.1	360	Bacteria>Proteobacteria>delta/epsilon_subdivisions>Deltaproteobacteria>unclassified_Deltaproteobacteria	Deltaproteobacteria_bacterium	DNA-sulfur-modification-protein-DndB-[Deltaproteobacteria-bacterium]	2026735
MBZ0235625.1	DndB-ParB-nuclease	GCA_019912685.1	579	Bacteria>Proteobacteria>delta/epsilon_subdivisions>Deltaproteobacteria>unclassified_Deltaproteobacteria	Deltaproteobacteria_bacterium	hypothetical-protein-[Deltaproteobacteria-bacterium]	2026735
OGR02349.1	DndB-ParB-nuclease	GCA_001798725.1	375	Bacteria>Proteobacteria>delta/epsilon_subdivisions>Deltaproteobacteria>unclassified_Deltaproteobacteria	Deltaproteobacteria_bacterium_RIFOXYD12_FULL_53_23	DNA-sulfur-modification-protein-DndB-[Deltaproteobacteria-bacterium-RIFOXYD12_FULL_53_23]	1797903
CAB5161330.1	DndB-ParB-nuclease	GCA_903231505.1	365	Bacteria>Proteobacteria>delta/epsilon_subdivisions>Deltaproteobacteria>unclassified_Deltaproteobacteria	Olavius_algarvensis_associated_proteobacterium_Delta_3	DNA-sulfur-modification-protein-DndB-[Olavius-algarvensis-associated-proteobacterium-Delta-3]	426688
WP_099311374.1	DndB-ParB-nuclease	GCF_003544855.1	607	Bacteria>Proteobacteria>delta/epsilon_subdivisions>Epsilonproteobacteria>Campylobacterales	Malaciobacter_marinus	hypothetical-protein-[Malaciobacter-marinus]	505249

Accession	Annotation	GCA	Length	Phyletic.spread	Strain.name	Defline	Species.Taxid
WP_152184893.1	DndB-ParB-nuclease	GCF_009192995.1	387	Bacteria>Proteobacteria>delta/epsilon_subdivisions>Epsilonproteobacteria>Campylobacterales	Sulfurimonas_indica	DGQHR-domain-containing-protein-[Sulfurimonas-indica]	2508707
PLY14204.1	DndB-ParB-nuclease	GCA_002869525.1	491	Bacteria>Proteobacteria>delta/epsilon_subdivisions>Epsilonproteobacteria>Campylobacterales	Sulfurimonas_sp.	hypothetical-protein-C0628_04430-[Sulfurimonas-sp.]	2022749
MBL09229.1	DndB-ParB-nuclease	GCA_002721105.1	364	Bacteria>Proteobacteria>Gammaproteobacteria>Acidiferrobacterales>Acidiferrobacteraceae	Acidiferrobacteraceae_bacterium	DNA-sulfur-modification-protein-DndB-[Acidiferrobacteraceae-bacterium]	2024893
WP_234604921.1	DndB-ParB-nuclease	GCF_021441165.1	363	Bacteria>Proteobacteria>Gammaproteobacteria>Aeromonadales>Aeromonadaceae	Aeromonas_allosaccharophila	DNA-sulfur-modification-protein-DndB-[Aeromonas-allosaccharophila]	656
WP_040095322.1	DndB-ParB-nuclease	GCF_000819725.1	387	Bacteria>Proteobacteria>Gammaproteobacteria>Aeromonadales>Aeromonadaceae	Aeromonas_australiensis	DGQHR-domain-containing-protein-[Aeromonas-australiensis]	1114880
WP_040095314.1	DndB-ParB-nuclease	GCF_000819725.1	361	Bacteria>Proteobacteria>Gammaproteobacteria>Aeromonadales>Aeromonadaceae	Aeromonas_australiensis	DNA-sulfur-modification-protein-DndB-[Aeromonas-australiensis]	1114880
WP_202042574.1	DndB-ParB-nuclease	GCF_016729565.1	363	Bacteria>Proteobacteria>Gammaproteobacteria>Aeromonadales>Aeromonadaceae	Aeromonas_caviae	DNA-sulfur-modification-protein-DndB-[Aeromonas-caviae]	648
WP_202042578.1	DndB-ParB-nuclease	GCF_016729565.1	731	Bacteria>Proteobacteria>Gammaproteobacteria>Aeromonadales>Aeromonadaceae	Aeromonas_caviae	hypothetical-protein-[Aeromonas-caviae]	648
WP_082038500.1	DndB-ParB-nuclease	GCF_000820305.1	389	Bacteria>Proteobacteria>Gammaproteobacteria>Aeromonadales>Aeromonadaceae	Aeromonas_dhakensis	DGQHR-domain-containing-protein-[Aeromonas-dhakensis]	196024
WP_213343025.1	DndB-ParB-nuclease	GCF_018359865.1	418	Bacteria>Proteobacteria>Gammaproteobacteria>Aeromonadales>Aeromonadaceae	Aeromonas_dhakensis	DGQHR-domain-containing-protein-[Aeromonas-dhakensis]	196024
WP_042050418.1	DndB-ParB-nuclease	GCF_000820305.1	361	Bacteria>Proteobacteria>Gammaproteobacteria>Aeromonadales>Aeromonadaceae	Aeromonas_dhakensis	DNA-sulfur-modification-protein-DndB-[Aeromonas-dhakensis]	196024
WP_213343029.1	DndB-ParB-nuclease	GCF_018359865.1	361	Bacteria>Proteobacteria>Gammaproteobacteria>Aeromonadales>Aeromonadaceae	Aeromonas_dhakensis	DNA-sulfur-modification-protein-DndB-[Aeromonas-dhakensis]	196024
WP_201940976.1	DndB-ParB-nuclease	GCF_016730045.1	434	Bacteria>Proteobacteria>Gammaproteobacteria>Aeromonadales>Aeromonadaceae	Aeromonas_enteropelogenes	DGQHR-domain-containing-protein-[Aeromonas-enteropelogenes]	29489
WP_201940980.1	DndB-ParB-nuclease	GCF_016730045.1	360	Bacteria>Proteobacteria>Gammaproteobacteria>Aeromonadales>Aeromonadaceae	Aeromonas_enteropelogenes	DNA-sulfur-modification-protein-DndB-[Aeromonas-enteropelogenes]	29489
WP_139388653.1	DndB-ParB-nuclease	GCF_006243205.1	361	Bacteria>Proteobacteria>Gammaproteobacteria>Aeromonadales>Aeromonadaceae	Aeromonas_hydrophila	DNA-sulfur-modification-protein-DndB-[Aeromonas-hydrophila]	644
WP_017786509.1	DndB-ParB-nuclease	GCF_000354715.1	361	Bacteria>Proteobacteria>Gammaproteobacteria>Aeromonadales>Aeromonadaceae	Aeromonas	MULTISPECIES:-DNA-sulfur-modification-protein-DndB-[Aeromonas]	642
WP_021464719.1	DndB-ParB-nuclease	GCF_012843275.1	363	Bacteria>Proteobacteria>Gammaproteobacteria>Aeromonadales>Aeromonadaceae	Aeromonas	MULTISPECIES:-DNA-sulfur-modification-protein-DndB-[Aeromonas]	642
WP_041209432.1	DndB-ParB-nuclease	GCF_011045495.1	361	Bacteria>Proteobacteria>Gammaproteobacteria>Aeromonadales>Aeromonadaceae	Aeromonas	MULTISPECIES:-DNA-sulfur-modification-protein-DndB-[Aeromonas]	642
WP_042019900.1	DndB-ParB-nuclease	GCF_000820145.1	361	Bacteria>Proteobacteria>Gammaproteobacteria>Aeromonadales>Aeromonadaceae	Aeromonas	MULTISPECIES:-DNA-sulfur-modification-protein-DndB-[Aeromonas]	642
WP_042019892.1	DndB-ParB-nuclease	GCF_000820145.1	400	Bacteria>Proteobacteria>Gammaproteobacteria>Aeromonadales>Aeromonadaceae	Aeromonas_sobria	DNA-phosphorothioation-associated-DGQHR-protein-1-[Aeromonas-sobria]	646
WP_159158727.1	DndB-ParB-nuclease	GCF_902505945.1	363	Bacteria>Proteobacteria>Gammaproteobacteria>Aeromonadales>Aeromonadaceae	Aeromonas_sp._8C	DNA-sulfur-modification-protein-DndB-[Aeromonas-sp.-8C]	2653127
WP_159158723.1	DndB-ParB-nuclease	GCF_902505945.1	558	Bacteria>Proteobacteria>Gammaproteobacteria>Aeromonadales>Aeromonadaceae	Aeromonas_sp._8C	hypothetical-protein-[Aeromonas-sp.-8C]	2653127
WP_168965907.1	DndB-ParB-nuclease	GCF_012843275.1	424	Bacteria>Proteobacteria>Gammaproteobacteria>Aeromonadales>Aeromonadaceae	Aeromonas_sp._DNRA1	DGQHR-domain-containing-protein-[Aeromonas-sp.-DNRA1]	2729335
WP_216953415.1	DndB-ParB-nuclease	GCF_019047065.1	363	Bacteria>Proteobacteria>Gammaproteobacteria>Aeromonadales>Aeromonadaceae	Aeromonas_sp._FDAARGOS_1414	DNA-sulfur-modification-protein-DndB-[Aeromonas-sp.-FDAARGOS-1414]	2778063
WP_139746941.1	DndB-ParB-nuclease	GCF_006246485.1	575	Bacteria>Proteobacteria>Gammaproteobacteria>Aeromonadales>Aeromonadaceae	Aeromonas_veronii	AIPR-family-protein-[Aeromonas-veronii]	654
VXA83213.1	DndB-ParB-nuclease	GCA_902505945.1	558	Bacteria>Proteobacteria>Gammaproteobacteria>Aeromonadales>Aeromonadaceae	Aeromonas_veronii	conserved-hypothetical-protein-[Aeromonas-veronii]	654
WP_064337156.1	DndB-ParB-nuclease	GCF_001593245.1	396	Bacteria>Proteobacteria>Gammaproteobacteria>Aeromonadales>Aeromonadaceae	Aeromonas_veronii	DGQHR-domain-containing-protein-[Aeromonas-veronii]	654
WP_164537007.1	DndB-ParB-nuclease	GCF_011045495.1	415	Bacteria>Proteobacteria>Gammaproteobacteria>Aeromonadales>Aeromonadaceae	Aeromonas_veronii	DGQHR-domain-containing-protein-[Aeromonas-veronii]	654
WP_236317301.1	DndB-ParB-nuclease	GCF_021608475.1	426	Bacteria>Proteobacteria>Gammaproteobacteria>Aeromonadales>Aeromonadaceae	Aeromonas_veronii	DGQHR-domain-containing-protein-[Aeromonas-veronii]	654
VXA83219.1	DndB-ParB-nuclease	GCA_902505945.1	389	Bacteria>Proteobacteria>Gammaproteobacteria>Aeromonadales>Aeromonadaceae	Aeromonas_veronii	DNA-sulfur-modification-protein-DndB-[Aeromonas-veronii]	654
WP_005361223.1	DndB-ParB-nuclease	GCF_000298035.1	361	Bacteria>Proteobacteria>Gammaproteobacteria>Aeromonadales>Aeromonadaceae	Aeromonas_veronii	DNA-sulfur-modification-protein-DndB-[Aeromonas-veronii]	654
WP_043819646.1	DndB-ParB-nuclease	GCF_901212355.1	361	Bacteria>Proteobacteria>Gammaproteobacteria>Aeromonadales>Aeromonadaceae	Aeromonas_veronii	DNA-sulfur-modification-protein-DndB-[Aeromonas-veronii]	654
WP_139407076.1	DndB-ParB-nuclease	GCF_901212295.1	361	Bacteria>Proteobacteria>Gammaproteobacteria>Aeromonadales>Aeromonadaceae	Aeromonas_veronii	DNA-sulfur-modification-protein-DndB-[Aeromonas-veronii]	654
WP_150388949.1	DndB-ParB-nuclease	GCF_008693705.1	361	Bacteria>Proteobacteria>Gammaproteobacteria>Aeromonadales>Aeromonadaceae	Aeromonas_veronii	DNA-sulfur-modification-protein-DndB-[Aeromonas-veronii]	654
WP_181270128.1	DndB-ParB-nuclease	GCF_013620815.1	361	Bacteria>Proteobacteria>Gammaproteobacteria>Aeromonadales>Aeromonadaceae	Aeromonas_veronii	DNA-sulfur-modification-protein-DndB-[Aeromonas-veronii]	654
WP_139473350.1	DndB-ParB-nuclease	GCF_006246555.1	687	Bacteria>Proteobacteria>Gammaproteobacteria>Aeromonadales>Aeromonadaceae	Aeromonas_veronii	hypothetical-protein-[Aeromonas-veronii]	654
WP_015878667.1	DndB-ParB-nuclease	-	358	Bacteria>Proteobacteria>Gammaproteobacteria>Aeromonadales>Aeromonadaceae	Tolumonas_auensis	DNA-sulfur-modification-protein-DndB-[Tolumonas-auensis]	43948
ACQ93196.1	DndB-ParB-nuclease	GCA_000023065.1	358	Bacteria>Proteobacteria>Gammaproteobacteria>Aeromonadales>Aeromonadaceae	Tolumonas_auensis_DSM_9187	DNA-sulfur-modification-protein-DndB-[Tolumonas-auensis-DSM-9187]	595494
WP_016403375.1	DndB-ParB-nuclease	GCF_019670105.1	359	Bacteria>Proteobacteria>Gammaproteobacteria>Alteromonadales>Alteromonadaceae	Agarivorans_albus	DNA-sulfur-modification-protein-DndB-[Agarivorans-albus]	182262
WP_055731867.1	DndB-ParB-nuclease	GCF_014636215.1	359	Bacteria>Proteobacteria>Gammaproteobacteria>Alteromonadales>Alteromonadaceae	Agarivorans_gilvus	DNA-sulfur-modification-protein-DndB-[Agarivorans-gilvus]	680279
WP_111977308.1	DndB-ParB-nuclease	GCF_003286125.1	363	Bacteria>Proteobacteria>Gammaproteobacteria>Alteromonadales>Alteromonadaceae	Algibacillus_agarilyticus	DNA-sulfur-modification-protein-DndB-[Algibacillus-agarilyticus]	2234133
MAM00087.1	DndB-ParB-nuclease	GCA_002695345.1	359	Bacteria>Proteobacteria>Gammaproteobacteria>Alteromonadales>Alteromonadaceae	Alteromonadaceae_bacterium	DNA-sulfur-modification-protein-DndB-[Alteromonadaceae-bacterium]	1916082
MAM00091.1	DndB-ParB-nuclease	GCA_002695345.1	408	Bacteria>Proteobacteria>Gammaproteobacteria>Alteromonadales>Alteromonadaceae	Alteromonadaceae_bacterium	hypothetical-protein-[Alteromonadaceae-bacterium]	1916082
WP_115594566.1	DndB-ParB-nuclease	GCF_003367475.1	361	Bacteria>Proteobacteria>Gammaproteobacteria>Alteromonadales>Alteromonadaceae	Alteromonas_aestuariivivens	DNA-sulfur-modification-protein-DndB-[Alteromonas-aestuariivivens]	1938339
WP_218312570.1	DndB-ParB-nuclease	GCF_019249295.1	361	Bacteria>Proteobacteria>Gammaproteobacteria>Alteromonadales>Alteromonadaceae	Alteromonas_antoniana	DNA-sulfur-modification-protein-DndB-[Alteromonas-antoniana]	2803813
WP_052364219.1	DndB-ParB-nuclease	GCF_000730385.1	414	Bacteria>Proteobacteria>Gammaproteobacteria>Alteromonadales>Alteromonadaceae	Alteromonas_australica	DGQHR-domain-containing-protein-[Alteromonas-australica]	589873
WP_044055466.1	DndB-ParB-nuclease	GCF_000730385.1	361	Bacteria>Proteobacteria>Gammaproteobacteria>Alteromonadales>Alteromonadaceae	Alteromonas_australica	DNA-sulfur-modification-protein-DndB-[Alteromonas-australica]	589873
WP_179981943.1	DndB-ParB-nuclease	GCF_903772925.1	361	Bacteria>Proteobacteria>Gammaproteobacteria>Alteromonadales>Alteromonadaceae	Alteromonas_macleodii	DNA-sulfur-modification-protein-DndB-[Alteromonas-macleodii]	28108
WP_118490868.1	DndB-ParB-nuclease	GCF_003443615.1	363	Bacteria>Proteobacteria>Gammaproteobacteria>Alteromonadales>Alteromonadaceae	Alteromonas_sp._BL110	DNA-sulfur-modification-protein-DndB-[Alteromonas-sp.-BL110]	1714845
WP_205422071.1	DndB-ParB-nuclease	GCF_003443615.1	563	Bacteria>Proteobacteria>Gammaproteobacteria>Alteromonadales>Alteromonadaceae	Alteromonas_sp._BL110	hypothetical-protein-[Alteromonas-sp.-BL110]	1714845
WP_061996747.1	DndB-ParB-nuclease	GCF_001562125.1	363	Bacteria>Proteobacteria>Gammaproteobacteria>Alteromonadales>Alteromonadaceae	Alteromonas_sp._Mac1	DNA-sulfur-modification-protein-DndB-[Alteromonas-sp.-Mac1]	1777491
MBZ2160773.1	DndB-ParB-nuclease	GCA_019931075.1	363	Bacteria>Proteobacteria>Gammaproteobacteria>Alteromonadales>Alteromonadaceae	Alteromonas_stellipolaris	DNA-sulfur-modification-protein-DndB-[Alteromonas-stellipolaris]	233316
WP_063457992.1	DndB-ParB-nuclease	GCF_001632825.1	363	Bacteria>Proteobacteria>Gammaproteobacteria>Alteromonadales>Alteromonadaceae	Alteromonas_stellipolaris	DNA-sulfur-modification-protein-DndB-[Alteromonas-stellipolaris]	233316
WP_063457997.1	DndB-ParB-nuclease	GCF_001632825.1	648	Bacteria>Proteobacteria>Gammaproteobacteria>Alteromonadales>Alteromonadaceae	Alteromonas_stellipolaris	hypothetical-protein-[Alteromonas-stellipolaris]	233316
MBJ2138211.1	DndB-ParB-nuclease	GCA_016405925.1	361	Bacteria>Proteobacteria>Gammaproteobacteria>Alteromonadales>Alteromonadaceae	Paraglaciecola_chathamensis	DNA-sulfur-modification-protein-DndB-[Paraglaciecola-chathamensis]	368405
WP_198825603.1	DndB-ParB-nuclease	GCF_016405925.1	361	Bacteria>Proteobacteria>Gammaproteobacteria>Alteromonadales>Alteromonadaceae	Paraglaciecola_chathamensis	DNA-sulfur-modification-protein-DndB-[Paraglaciecola-chathamensis]	368405
WP_068372310.1	DndB-ParB-nuclease	GCF_001565895.1	361	Bacteria>Proteobacteria>Gammaproteobacteria>Alteromonadales>Alteromonadaceae	Paraglaciecola_hydrolytica	DNA-sulfur-modification-protein-DndB-[Paraglaciecola-hydrolytica]	1799789
WP_137165416.1	DndB-ParB-nuclease	GCF_005222225.1	408	Bacteria>Proteobacteria>Gammaproteobacteria>Alteromonadales>Alteromonadaceae	Salinimonas_lutimaris	DGQHR-domain-containing-protein-[Salinimonas-lutimaris]	914153
WP_137165421.1	DndB-ParB-nuclease	GCF_005222225.1	361	Bacteria>Proteobacteria>Gammaproteobacteria>Alteromonadales>Alteromonadaceae	Salinimonas_lutimaris	DNA-sulfur-modification-protein-DndB-[Salinimonas-lutimaris]	914153
WP_117318500.1	DndB-ParB-nuclease	GCF_003429285.1	563	Bacteria>Proteobacteria>Gammaproteobacteria>Alteromonadales>Alteromonadaceae	Salinimonas_sediminis	AIPR-family-protein-[Salinimonas-sediminis]	2303538
WP_117318490.1	DndB-ParB-nuclease	GCF_003429285.1	362	Bacteria>Proteobacteria>Gammaproteobacteria>Alteromonadales>Alteromonadaceae	Salinimonas_sediminis	DNA-sulfur-modification-protein-DndB-[Salinimonas-sediminis]	2303538
WP_233052427.1	DndB-ParB-nuclease	GCF_021295315.1	363	Bacteria>Proteobacteria>Gammaproteobacteria>Alteromonadales>Alteromonadales_genera_incertae_sedis	Motilimonas_cestriensis	DNA-sulfur-modification-protein-DndB-[Motilimonas-cestriensis]	2742685
WP_126668721.1	DndB-ParB-nuclease	GCF_003971255.1	363	Bacteria>Proteobacteria>Gammaproteobacteria>Alteromonadales>Colwelliaceae	Colwellia_sp._Arc7-635	DNA-sulfur-modification-protein-DndB-[Colwellia-sp.-Arc7-635]	2497879
WP_108455560.1	DndB-ParB-nuclease	GCF_003061515.1	363	Bacteria>Proteobacteria>Gammaproteobacteria>Alteromonadales>Colwelliaceae	Colwellia_sp._Arc7-D	DNA-sulfur-modification-protein-DndB-[Colwellia-sp.-Arc7-D]	2161872
WP_108455556.1	DndB-ParB-nuclease	GCF_003061515.1	720	Bacteria>Proteobacteria>Gammaproteobacteria>Alteromonadales>Colwelliaceae	Colwellia_sp._Arc7-D	hypothetical-protein-[Colwellia-sp.-Arc7-D]	2161872
WP_101155066.1	DndB-ParB-nuclease	GCF_002836245.1	563	Bacteria>Proteobacteria>Gammaproteobacteria>Alteromonadales>Colwelliaceae	Colwellia_sp._Bg11-28	AIPR-family-protein-[Colwellia-sp.-Bg11-28]	2058305
WP_157825877.1	DndB-ParB-nuclease	GCF_002836245.1	382	Bacteria>Proteobacteria>Gammaproteobacteria>Alteromonadales>Colwelliaceae	Colwellia_sp._Bg11-28	DGQHR-domain-containing-protein-[Colwellia-sp.-Bg11-28]	2058305
PKH87334.1	DndB-ParB-nuclease	GCA_002836245.1	364	Bacteria>Proteobacteria>Gammaproteobacteria>Alteromonadales>Colwelliaceae	Colwellia_sp._Bg11-28	DNA-sulfur-modification-protein-DndB-[Colwellia-sp.-Bg11-28]	2058305
WP_101155076.1	DndB-ParB-nuclease	GCF_002836245.1	364	Bacteria>Proteobacteria>Gammaproteobacteria>Alteromonadales>Colwelliaceae	Colwellia_sp._Bg11-28	DNA-sulfur-modification-protein-DndB-[Colwellia-sp.-Bg11-28]	2058305
PKH87330.1	DndB-ParB-nuclease	GCA_002836245.1	389	Bacteria>Proteobacteria>Gammaproteobacteria>Alteromonadales>Colwelliaceae	Colwellia_sp._Bg11-28	hypothetical-protein-CXF79_11680-[Colwellia-sp.-Bg11-28]	2058305
WP_182259190.1	DndB-ParB-nuclease	-	406	Bacteria>Proteobacteria>Gammaproteobacteria>Alteromonadales>Colwelliaceae	Colwellia_sp._BRX8-4	DGQHR-domain-containing-protein-[Colwellia-sp.-BRX8-4]	2759836
WP_182259193.1	DndB-ParB-nuclease	GCF_014077345.1	363	Bacteria>Proteobacteria>Gammaproteobacteria>Alteromonadales>Colwelliaceae	Colwellia_sp._BRX8-4	DNA-sulfur-modification-protein-DndB-[Colwellia-sp.-BRX8-4]	2759836
WP_215973835.1	DndB-ParB-nuclease	GCF_018860915.1	364	Bacteria>Proteobacteria>Gammaproteobacteria>Alteromonadales>Colwelliaceae	Colwellia_sp._C2M11	DNA-sulfur-modification-protein-DndB-[Colwellia-sp.-C2M11]	2841566
WP_118959286.1	DndB-ParB-nuclease	GCF_003545765.1	411	Bacteria>Proteobacteria>Gammaproteobacteria>Alteromonadales>Colwelliaceae	Colwellia_sp._RSH04	DGQHR-domain-containing-protein-[Colwellia-sp.-RSH04]	2305464
WP_118959282.1	DndB-ParB-nuclease	GCF_003545765.1	363	Bacteria>Proteobacteria>Gammaproteobacteria>Alteromonadales>Colwelliaceae	Colwellia_sp._RSH04	DNA-sulfur-modification-protein-DndB-[Colwellia-sp.-RSH04]	2305464
WP_057831077.1	DndB-ParB-nuclease	GCF_001440345.1	362	Bacteria>Proteobacteria>Gammaproteobacteria>Alteromonadales>Colwelliaceae	Colwellia_sp._TT2012	DNA-sulfur-modification-protein-DndB-[Colwellia-sp.-TT2012]	1720342
TRX56697.1	DndB-ParB-nuclease	GCA_007097525.1	361	Bacteria>Proteobacteria>Gammaproteobacteria>Alteromonadales>Colwelliaceae	Thalassomonas_sp._M1454	DNA-sulfur-modification-protein-DndB-[Thalassomonas-sp.-M1454]	2594477
WP_143583456.1	DndB-ParB-nuclease	GCF_007097525.1	361	Bacteria>Proteobacteria>Gammaproteobacteria>Alteromonadales>Colwelliaceae	Thalassomonas_sp._M1454	DNA-sulfur-modification-protein-DndB-[Thalassomonas-sp.-M1454]	2594477
TKB46331.1	DndB-ParB-nuclease	GCA_005116735.1	661	Bacteria>Proteobacteria>Gammaproteobacteria>Alteromonadales>Colwelliaceae	Thalassotalea_mangrovi	DGQHR-domain-containing-protein-[Thalassotalea-mangrovi]	2572245
WP_136734907.1	DndB-ParB-nuclease	GCF_005116735.1	661	Bacteria>Proteobacteria>Gammaproteobacteria>Alteromonadales>Colwelliaceae	Thalassotalea_mangrovi	DGQHR-domain-containing-protein-[Thalassotalea-mangrovi]	2572245
WP_222488029.1	DndB-ParB-nuclease	GCF_019801795.1	387	Bacteria>Proteobacteria>Gammaproteobacteria>Alteromonadales>Ferrimonadaceae	Ferrimonas_balearica	DGQHR-domain-containing-protein-[Ferrimonas-balearica]	44012
WP_222544966.1	DndB-ParB-nuclease	-	408	Bacteria>Proteobacteria>Gammaproteobacteria>Alteromonadales>Ferrimonadaceae	Ferrimonas_balearica	DGQHR-domain-containing-protein-[Ferrimonas-balearica]	44012
WP_222488033.1	DndB-ParB-nuclease	GCF_019801795.1	369	Bacteria>Proteobacteria>Gammaproteobacteria>Alteromonadales>Ferrimonadaceae	Ferrimonas_balearica	DNA-sulfur-modification-protein-DndB-[Ferrimonas-balearica]	44012
WP_222544970.1	DndB-ParB-nuclease	GCF_019802085.1	369	Bacteria>Proteobacteria>Gammaproteobacteria>Alteromonadales>Ferrimonadaceae	Ferrimonas_balearica	DNA-sulfur-modification-protein-DndB-[Ferrimonas-balearica]	44012
WP_028114351.1	DndB-ParB-nuclease	GCF_000425405.1	369	Bacteria>Proteobacteria>Gammaproteobacteria>Alteromonadales>Ferrimonadaceae	Ferrimonas_kyonanensis	DNA-sulfur-modification-protein-DndB-[Ferrimonas-kyonanensis]	364763
QGT96834.1	DndB-ParB-nuclease	GCA_009734345.1	360	Bacteria>Proteobacteria>Gammaproteobacteria>Alteromonadales>Idiomarinaceae	Idiomarina_andamanensis	DNA-sulfur-modification-protein-DndB-[Idiomarina-andamanensis]	1940690
NWO02094.1	DndB-ParB-nuclease	GCA_013393475.1	363	Bacteria>Proteobacteria>Gammaproteobacteria>Alteromonadales>Idiomarinaceae	Idiomarinaceae_bacterium	DNA-sulfur-modification-protein-DndB-[Idiomarinaceae-bacterium]	1898113
WP_034819308.1	DndB-ParB-nuclease	GCF_000320945.2	363	Bacteria>Proteobacteria>Gammaproteobacteria>Alteromonadales>Idiomarinaceae	Idiomarina_sp._28-8	DNA-sulfur-modification-protein-DndB-[Idiomarina-sp.-28-8]	1260624
EGN74188.1	DndB-ParB-nuclease	GCA_000218785.2	346	Bacteria>Proteobacteria>Gammaproteobacteria>Alteromonadales>Idiomarinaceae	Idiomarina_sp._A28L	DGQHR-domain-containing-protein-[Idiomarina-sp.-A28L]	1036674
WP_158091796.1	DndB-ParB-nuclease	GCF_000218785.1	330	Bacteria>Proteobacteria>Gammaproteobacteria>Alteromonadales>Idiomarinaceae	Idiomarina_sp._A28L	DGQHR-domain-containing-protein-[Idiomarina-sp.-A28L]	1036674
EGN74183.1	DndB-ParB-nuclease	GCA_000218785.2	363	Bacteria>Proteobacteria>Gammaproteobacteria>Alteromonadales>Idiomarinaceae	Idiomarina_sp._A28L	DNA-sulfur-modification-protein-DndB-[Idiomarina-sp.-A28L]	1036674
WP_007421264.1	DndB-ParB-nuclease	GCF_000218785.1	363	Bacteria>Proteobacteria>Gammaproteobacteria>Alteromonadales>Idiomarinaceae	Idiomarina_sp._A28L	DNA-sulfur-modification-protein-DndB-[Idiomarina-sp.-A28L]	1036674
MBT41821.1	DndB-ParB-nuclease	GCA_002729675.1	389	Bacteria>Proteobacteria>Gammaproteobacteria>Alteromonadales>Idiomarinaceae	Idiomarina_sp.	DNA-phosphorothioation-associated-DGQHR-protein-1-[Idiomarina-sp.]	1874361
WP_211676561.1	DndB-ParB-nuclease	GCF_018219415.1	364	Bacteria>Proteobacteria>Gammaproteobacteria>Alteromonadales>Moritellaceae	Moritella_sp._36	DNA-sulfur-modification-protein-DndB-[Moritella-sp.-36]	2746233
WP_211676566.1	DndB-ParB-nuclease	GCF_018219415.1	660	Bacteria>Proteobacteria>Gammaproteobacteria>Alteromonadales>Moritellaceae	Moritella_sp._36	hypothetical-protein-[Moritella-sp.-36]	2746233
WP_157824452.1	DndB-ParB-nuclease	GCF_002836355.1	407	Bacteria>Proteobacteria>Gammaproteobacteria>Alteromonadales>Moritellaceae	Moritella_sp._Urea-trap-13	DGQHR-domain-containing-protein-[Moritella-sp.-Urea-trap-13]	2058327
WP_101063146.1	DndB-ParB-nuclease	GCF_002836355.1	363	Bacteria>Proteobacteria>Gammaproteobacteria>Alteromonadales>Moritellaceae	Moritella_sp._Urea-trap-13	DNA-sulfur-modification-protein-DndB-[Moritella-sp.-Urea-trap-13]	2058327
NQZ06738.1	DndB-ParB-nuclease	GCA_013216025.1	363	Bacteria>Proteobacteria>Gammaproteobacteria>Alteromonadales>Pseudoalteromonadaceae	Algicola_sp.	DNA-sulfur-modification-protein-DndB-[Algicola-sp.]	1872429
WP_169044075.1	DndB-ParB-nuclease	-	386	Bacteria>Proteobacteria>Gammaproteobacteria>Alteromonadales>Pseudoalteromonadaceae	Pseudoalteromonas_arctica	DGQHR-domain-containing-protein-[Pseudoalteromonas-arctica]	394751
WP_169044079.1	DndB-ParB-nuclease	GCF_012933495.1	361	Bacteria>Proteobacteria>Gammaproteobacteria>Alteromonadales>Pseudoalteromonadaceae	Pseudoalteromonas_arctica	DNA-sulfur-modification-protein-DndB-[Pseudoalteromonas-arctica]	394751
WP_138674908.1	DndB-ParB-nuclease	GCF_005886875.1	392	Bacteria>Proteobacteria>Gammaproteobacteria>Alteromonadales>Pseudoalteromonadaceae	Pseudoalteromonas_aurantia	DGQHR-domain-containing-protein-[Pseudoalteromonas-aurantia]	43654
WP_138676536.1	DndB-ParB-nuclease	GCF_005887285.1	326	Bacteria>Proteobacteria>Gammaproteobacteria>Alteromonadales>Pseudoalteromonadaceae	Pseudoalteromonas_aurantia	DGQHR-domain-containing-protein-[Pseudoalteromonas-aurantia]	43654
WP_138592837.1	DndB-ParB-nuclease	GCF_005887355.1	361	Bacteria>Proteobacteria>Gammaproteobacteria>Alteromonadales>Pseudoalteromonadaceae	Pseudoalteromonas_aurantia	DNA-sulfur-modification-protein-DndB-[Pseudoalteromonas-aurantia]	43654
WP_138676541.1	DndB-ParB-nuclease	GCF_005887285.1	362	Bacteria>Proteobacteria>Gammaproteobacteria>Alteromonadales>Pseudoalteromonadaceae	Pseudoalteromonas_aurantia	DNA-sulfur-modification-protein-DndB-[Pseudoalteromonas-aurantia]	43654
WP_104644165.1	DndB-ParB-nuclease	GCF_014925295.1	361	Bacteria>Proteobacteria>Gammaproteobacteria>Alteromonadales>Pseudoalteromonadaceae	Pseudoalteromonas_carrageenovora	DNA-sulfur-modification-protein-DndB-[Pseudoalteromonas-carrageenovora]	227
WP_164504451.1	DndB-ParB-nuclease	GCF_007989585.1	376	Bacteria>Proteobacteria>Gammaproteobacteria>Alteromonadales>Pseudoalteromonadaceae	Pseudoalteromonas_espejiana	DGQHR-domain-containing-protein-[Pseudoalteromonas-espejiana]	28107
WP_089349294.1	DndB-ParB-nuclease	GCF_007989585.1	361	Bacteria>Proteobacteria>Gammaproteobacteria>Alteromonadales>Pseudoalteromonadaceae	Pseudoalteromonas_espejiana	DNA-sulfur-modification-protein-DndB-[Pseudoalteromonas-espejiana]	28107
WP_138524673.1	DndB-ParB-nuclease	GCF_005886105.2	395	Bacteria>Proteobacteria>Gammaproteobacteria>Alteromonadales>Pseudoalteromonadaceae	Pseudoalteromonas_galatheae	DGQHR-domain-containing-protein-[Pseudoalteromonas-sp.-S4498]	579562
WP_138524665.1	DndB-ParB-nuclease	GCF_005886105.2	361	Bacteria>Proteobacteria>Gammaproteobacteria>Alteromonadales>Pseudoalteromonadaceae	Pseudoalteromonas_galatheae	DNA-sulfur-modification-protein-DndB-[Pseudoalteromonas-sp.-S4498]	579562
WP_058155677.1	DndB-ParB-nuclease	GCF_002310795.1	363	Bacteria>Proteobacteria>Gammaproteobacteria>Alteromonadales>Pseudoalteromonadaceae	Pseudoalteromonas_issachenkonii	DNA-sulfur-modification-protein-DndB-[Pseudoalteromonas-issachenkonii]	152297
WP_065979251.1	DndB-ParB-nuclease	GCF_001704875.1	363	Bacteria>Proteobacteria>Gammaproteobacteria>Alteromonadales>Pseudoalteromonadaceae	Pseudoalteromonas_lipolytica	DNA-sulfur-modification-protein-DndB-[Pseudoalteromonas-lipolytica]	570156
WP_063360447.1	DndB-ParB-nuclease	GCF_001625695.1	373	Bacteria>Proteobacteria>Gammaproteobacteria>Alteromonadales>Pseudoalteromonadaceae	Pseudoalteromonas_luteoviolacea	DGQHR-domain-containing-protein-[Pseudoalteromonas-luteoviolacea]	43657
WP_039610245.1	DndB-ParB-nuclease	GCF_000814765.1	362	Bacteria>Proteobacteria>Gammaproteobacteria>Alteromonadales>Pseudoalteromonadaceae	Pseudoalteromonas_luteoviolacea	DNA-sulfur-modification-protein-DndB-[Pseudoalteromonas-luteoviolacea]	43657
WP_063360443.1	DndB-ParB-nuclease	GCF_001625695.1	361	Bacteria>Proteobacteria>Gammaproteobacteria>Alteromonadales>Pseudoalteromonadaceae	Pseudoalteromonas_luteoviolacea	DNA-sulfur-modification-protein-DndB-[Pseudoalteromonas-luteoviolacea]	43657
WP_054452429.1	DndB-ParB-nuclease	GCF_001298125.1	361	Bacteria>Proteobacteria>Gammaproteobacteria>Alteromonadales>Pseudoalteromonadaceae	Pseudoalteromonas	MULTISPECIES:-DNA-sulfur-modification-protein-DndB-[Pseudoalteromonas]	53246
WP_138566243.1	DndB-ParB-nuclease	GCF_005887275.1	411	Bacteria>Proteobacteria>Gammaproteobacteria>Alteromonadales>Pseudoalteromonadaceae	Pseudoalteromonas_phenolica	DGQHR-domain-containing-protein-[Pseudoalteromonas-phenolica]	161398
WP_138566240.1	DndB-ParB-nuclease	GCF_005887275.1	363	Bacteria>Proteobacteria>Gammaproteobacteria>Alteromonadales>Pseudoalteromonadaceae	Pseudoalteromonas_phenolica	DNA-sulfur-modification-protein-DndB-[Pseudoalteromonas-phenolica]	161398
WP_010375355.1	DndB-ParB-nuclease	GCF_000238315.3	362	Bacteria>Proteobacteria>Gammaproteobacteria>Alteromonadales>Pseudoalteromonadaceae	Pseudoalteromonas_piscicida	DNA-sulfur-modification-protein-DndB-[Pseudoalteromonas-piscicida]	43662
WP_119232774.1	DndB-ParB-nuclease	GCF_003429305.1	362	Bacteria>Proteobacteria>Gammaproteobacteria>Alteromonadales>Pseudoalteromonadaceae	Pseudoalteromonas_piscicida	DNA-sulfur-modification-protein-DndB-[Pseudoalteromonas-piscicida]	43662
WP_010380887.1	DndB-ParB-nuclease	GCF_000238295.3	392	Bacteria>Proteobacteria>Gammaproteobacteria>Alteromonadales>Pseudoalteromonadaceae	Pseudoalteromonas_rubra	DGQHR-domain-containing-protein-[Pseudoalteromonas-rubra]	43658
WP_040645631.1	DndB-ParB-nuclease	GCF_000238295.3	361	Bacteria>Proteobacteria>Gammaproteobacteria>Alteromonadales>Pseudoalteromonadaceae	Pseudoalteromonas_rubra	DNA-sulfur-modification-protein-DndB-[Pseudoalteromonas-rubra]	43658
TMO91866.1	DndB-ParB-nuclease	GCA_005886935.1	361	Bacteria>Proteobacteria>Gammaproteobacteria>Alteromonadales>Pseudoalteromonadaceae	Pseudoalteromonas_ruthenica	DNA-sulfur-modification-protein-DndB-[Pseudoalteromonas-ruthenica]	151081
TMO96666.1	DndB-ParB-nuclease	GCA_005887035.1	361	Bacteria>Proteobacteria>Gammaproteobacteria>Alteromonadales>Pseudoalteromonadaceae	Pseudoalteromonas_ruthenica	DNA-sulfur-modification-protein-DndB-[Pseudoalteromonas-ruthenica]	151081
TMP05866.1	DndB-ParB-nuclease	GCA_005887015.1	361	Bacteria>Proteobacteria>Gammaproteobacteria>Alteromonadales>Pseudoalteromonadaceae	Pseudoalteromonas_ruthenica	DNA-sulfur-modification-protein-DndB-[Pseudoalteromonas-ruthenica]	151081
TMP05970.1	DndB-ParB-nuclease	GCA_005887005.1	361	Bacteria>Proteobacteria>Gammaproteobacteria>Alteromonadales>Pseudoalteromonadaceae	Pseudoalteromonas_ruthenica	DNA-sulfur-modification-protein-DndB-[Pseudoalteromonas-ruthenica]	151081

Accession	Annotation	GCA	Length	Phyletic.spread	Strain.name	Defline	Species.Taxid
WP_045980187.1	DndB-ParB-nuclease	GCF_008808095.1	361	Bacteria>Proteobacteria>Gammaproteobacteria>Alteromonadales>Pseudoalteromonadaceae	Pseudoalteromonas_ruthenica	DNA-sulfur-modification-protein-DndB-[Pseudoalteromonas-ruthenica]	151081
WP_138509219.1	DndB-ParB-nuclease	GCF_005876865.1	361	Bacteria>Proteobacteria>Gammaproteobacteria>Alteromonadales>Pseudoalteromonadaceae	Pseudoalteromonas_ruthenica	DNA-sulfur-modification-protein-DndB-[Pseudoalteromonas-ruthenica]	151081
WP_138548067.1	DndB-ParB-nuclease	GCF_005887125.1	362	Bacteria>Proteobacteria>Gammaproteobacteria>Alteromonadales>Pseudoalteromonadaceae	Pseudoalteromonas_ruthenica	DNA-sulfur-modification-protein-DndB-[Pseudoalteromonas-ruthenica]	151081
WP_138509214.1	DndB-ParB-nuclease	GCF_005876865.1	676	Bacteria>Proteobacteria>Gammaproteobacteria>Alteromonadales>Pseudoalteromonadaceae	Pseudoalteromonas_ruthenica	hypothetical-protein-[Pseudoalteromonas-ruthenica]	151081
WP_171041814.1	DndB-ParB-nuclease	GCF_005887125.1	656	Bacteria>Proteobacteria>Gammaproteobacteria>Alteromonadales>Pseudoalteromonadaceae	Pseudoalteromonas_ruthenica	hypothetical-protein-[Pseudoalteromonas-ruthenica]	151081
QWV06568.1	DndB-ParB-nuclease	GCA_018972125.1	386	Bacteria>Proteobacteria>Gammaproteobacteria>Alteromonadales>Pseudoalteromonadaceae	Pseudoalteromonas_shioyasakiensis	DGQHR-domain-containing-protein-[Pseudoalteromonas-shioyasakiensis]	1190813
QWV06573.1	DndB-ParB-nuclease	GCA_018972125.1	361	Bacteria>Proteobacteria>Gammaproteobacteria>Alteromonadales>Pseudoalteromonadaceae	Pseudoalteromonas_shioyasakiensis	DNA-sulfur-modification-protein-DndB-[Pseudoalteromonas-shioyasakiensis]	1190813
WP_062565192.1	DndB-ParB-nuclease	GCF_001550135.1	361	Bacteria>Proteobacteria>Gammaproteobacteria>Alteromonadales>Pseudoalteromonadaceae	Pseudoalteromonas_shioyasakiensis	DNA-sulfur-modification-protein-DndB-[Pseudoalteromonas-shioyasakiensis]	1190813
WP_198522900.1	DndB-ParB-nuclease	GCF_903995135.1	563	Bacteria>Proteobacteria>Gammaproteobacteria>Alteromonadales>Pseudoalteromonadaceae	Pseudoalteromonas_sp._3J6	AIPR-family-protein-[Pseudoalteromonas-sp.-3J6]	649161
WP_008129587.1	DndB-ParB-nuclease	GCF_000241365.1	361	Bacteria>Proteobacteria>Gammaproteobacteria>Alteromonadales>Pseudoalteromonadaceae	Pseudoalteromonas_sp._BSi20480	DNA-sulfur-modification-protein-DndB-[Pseudoalteromonas-sp.-BSi20480]	386428
WP_187489652.1	DndB-ParB-nuclease	GCF_014349195.1	361	Bacteria>Proteobacteria>Gammaproteobacteria>Alteromonadales>Pseudoalteromonadaceae	Pseudoalteromonas_sp._BZK2	DNA-sulfur-modification-protein-DndB-[Pseudoalteromonas-sp.-BZK2]	1904458
HDY91004.1	DndB-ParB-nuclease	GCA_011050215.1	361	Bacteria>Proteobacteria>Gammaproteobacteria>Alteromonadales>Pseudoalteromonadaceae	Pseudoalteromonas_sp.	DNA-sulfur-modification-protein-DndB-[Pseudoalteromonas-sp.]	53249
MAY60524.1	DndB-ParB-nuclease	GCA_002706965.1	361	Bacteria>Proteobacteria>Gammaproteobacteria>Alteromonadales>Pseudoalteromonadaceae	Pseudoalteromonas_sp.	DNA-sulfur-modification-protein-DndB-[Pseudoalteromonas-sp.]	53249
PHQ89712.1	DndB-ParB-nuclease	GCA_002732915.1	363	Bacteria>Proteobacteria>Gammaproteobacteria>Alteromonadales>Pseudoalteromonadaceae	Pseudoalteromonas_sp.	DNA-sulfur-modification-protein-DndB-[Pseudoalteromonas-sp.]	53249
WP_058550467.1	DndB-ParB-nuclease	GCF_001469205.1	361	Bacteria>Proteobacteria>Gammaproteobacteria>Alteromonadales>Pseudoalteromonadaceae	Pseudoalteromonas_sp._H103	DNA-sulfur-modification-protein-DndB-[Pseudoalteromonas-sp.-H103]	1761893
WP_237127999.1	DndB-ParB-nuclease	GCF_021728035.1	401	Bacteria>Proteobacteria>Gammaproteobacteria>Alteromonadales>Pseudoalteromonadaceae	Pseudoalteromonas_sp._L21	DGQHR-domain-containing-protein-[Pseudoalteromonas-sp.-L21]	1539746
WP_166338291.1	DndB-ParB-nuclease	GCF_011319765.1	361	Bacteria>Proteobacteria>Gammaproteobacteria>Alteromonadales>Pseudoalteromonadaceae	Pseudoalteromonas_sp._MB47	DNA-sulfur-modification-protein-DndB-[Pseudoalteromonas-sp.-MB47]	2588452
WP_211055493.1	DndB-ParB-nuclease	GCF_018100905.1	363	Bacteria>Proteobacteria>Gammaproteobacteria>Alteromonadales>Pseudoalteromonadaceae	Pseudoalteromonas_sp._MMG010	DNA-sulfur-modification-protein-DndB-[Pseudoalteromonas-sp.-MMG010]	2822685
WP_211051738.1	DndB-ParB-nuclease	GCF_018100875.1	361	Bacteria>Proteobacteria>Gammaproteobacteria>Alteromonadales>Pseudoalteromonadaceae	Pseudoalteromonas_sp._MMG012	DNA-sulfur-modification-protein-DndB-[Pseudoalteromonas-sp.-MMG012]	2822686
WP_231612796.1	DndB-ParB-nuclease	GCF_015864275.1	364	Bacteria>Proteobacteria>Gammaproteobacteria>Alteromonadales>Pseudoalteromonadaceae	Pseudoalteromonas_sp._NZS11_1	DGQHR-domain-containing-protein-[Pseudoalteromonas-sp.-NZS11_1]	2792070
WP_197173438.1	DndB-ParB-nuclease	GCF_015864275.1	361	Bacteria>Proteobacteria>Gammaproteobacteria>Alteromonadales>Pseudoalteromonadaceae	Pseudoalteromonas_sp._NZS11_1	DNA-sulfur-modification-protein-DndB-[Pseudoalteromonas-sp.-NZS11_1]	2792070
WP_055022116.1	DndB-ParB-nuclease	GCF_001401795.1	361	Bacteria>Proteobacteria>Gammaproteobacteria>Alteromonadales>Pseudoalteromonadaceae	Pseudoalteromonas_sp._P1-26	DNA-sulfur-modification-protein-DndB-[Pseudoalteromonas-sp.-P1-26]	1723759
WP_054982124.1	DndB-ParB-nuclease	GCF_001400005.1	361	Bacteria>Proteobacteria>Gammaproteobacteria>Alteromonadales>Pseudoalteromonadaceae	Pseudoalteromonas_sp._P1-30	DNA-sulfur-modification-protein-DndB-[Pseudoalteromonas-sp.-P1-30]	1723760
KPV93547.1	DndB-ParB-nuclease	GCA_001400005.1	374	Bacteria>Proteobacteria>Gammaproteobacteria>Alteromonadales>Pseudoalteromonadaceae	Pseudoalteromonas_sp._P1-30	hypothetical-protein-AN395_00673-[Pseudoalteromonas-sp.-P1-30]	1723760
WP_029609902.1	DndB-ParB-nuclease	GCF_000263075.1	363	Bacteria>Proteobacteria>Gammaproteobacteria>Alteromonadales>Pseudoalteromonadaceae	Pseudoalteromonas_sp._PAMC_22718	DNA-sulfur-modification-protein-DndB-[Pseudoalteromonas-sp.-PAMC-22718]	1175295
WP_138681953.1	DndB-ParB-nuclease	GCF_005886985.1	425	Bacteria>Proteobacteria>Gammaproteobacteria>Alteromonadales>Pseudoalteromonadaceae	Pseudoalteromonas_sp._S3178	DGQHR-domain-containing-protein-[Pseudoalteromonas-sp.-S3178]	579532
WP_138681957.1	DndB-ParB-nuclease	GCF_005886985.1	361	Bacteria>Proteobacteria>Gammaproteobacteria>Alteromonadales>Pseudoalteromonadaceae	Pseudoalteromonas_sp._S3178	DNA-sulfur-modification-protein-DndB-[Pseudoalteromonas-sp.-S3178]	579532
WP_138585511.1	DndB-ParB-nuclease	GCF_005886635.1	361	Bacteria>Proteobacteria>Gammaproteobacteria>Alteromonadales>Pseudoalteromonadaceae	Pseudoalteromonas_sp._S558	DNA-sulfur-modification-protein-DndB-[Pseudoalteromonas-sp.-S558]	2066515
WP_182725028.1	DndB-ParB-nuclease	GCF_014164385.1	361	Bacteria>Proteobacteria>Gammaproteobacteria>Alteromonadales>Pseudoalteromonadaceae	Pseudoalteromonas_sp._SG43-3	DNA-sulfur-modification-protein-DndB-[Pseudoalteromonas-sp.-SG43-3]	2760970
MBB1433934.1	DndB-ParB-nuclease	GCA_014164365.1	361	Bacteria>Proteobacteria>Gammaproteobacteria>Alteromonadales>Pseudoalteromonadaceae	Pseudoalteromonas_sp._SG43-6	DNA-sulfur-modification-protein-DndB-[Pseudoalteromonas-sp.-SG43-6]	2760967
WP_182784020.1	DndB-ParB-nuclease	GCF_014164365.1	361	Bacteria>Proteobacteria>Gammaproteobacteria>Alteromonadales>Pseudoalteromonadaceae	Pseudoalteromonas_sp._SG43-6	DNA-sulfur-modification-protein-DndB-[Pseudoalteromonas-sp.-SG43-6]	2760967
WP_182733110.1	DndB-ParB-nuclease	GCF_014163935.1	347	Bacteria>Proteobacteria>Gammaproteobacteria>Alteromonadales>Pseudoalteromonadaceae	Pseudoalteromonas_sp._SR41-7	DGQHR-domain-containing-protein-[Pseudoalteromonas-sp.-SR41-7]	2760947
WP_182733114.1	DndB-ParB-nuclease	GCF_014163935.1	364	Bacteria>Proteobacteria>Gammaproteobacteria>Alteromonadales>Pseudoalteromonadaceae	Pseudoalteromonas_sp._SR41-7	DNA-sulfur-modification-protein-DndB-[Pseudoalteromonas-sp.-SR41-7]	2760947
WP_182777262.1	DndB-ParB-nuclease	GCF_014163915.1	361	Bacteria>Proteobacteria>Gammaproteobacteria>Alteromonadales>Pseudoalteromonadaceae	Pseudoalteromonas_sp._SR41-8	DNA-sulfur-modification-protein-DndB-[Pseudoalteromonas-sp.-SR41-8]	2760946
WP_182777252.1	DndB-ParB-nuclease	GCF_014163915.1	694	Bacteria>Proteobacteria>Gammaproteobacteria>Alteromonadales>Pseudoalteromonadaceae	Pseudoalteromonas_sp._SR41-8	hypothetical-protein-[Pseudoalteromonas-sp.-SR41-8]	2760946
WP_182672705.1	DndB-ParB-nuclease	GCF_014163785.1	648	Bacteria>Proteobacteria>Gammaproteobacteria>Alteromonadales>Pseudoalteromonadaceae	Pseudoalteromonas_sp._SR43-7	hypothetical-protein-[Pseudoalteromonas-sp.-SR43-7]	2760939
WP_182755828.1	DndB-ParB-nuclease	GCF_014163745.1	361	Bacteria>Proteobacteria>Gammaproteobacteria>Alteromonadales>Pseudoalteromonadaceae	Pseudoalteromonas_sp._SR44-2	DNA-sulfur-modification-protein-DndB-[Pseudoalteromonas-sp.-SR44-2]	2760937
WP_182711927.1	DndB-ParB-nuclease	GCF_014163655.1	425	Bacteria>Proteobacteria>Gammaproteobacteria>Alteromonadales>Pseudoalteromonadaceae	Pseudoalteromonas_sp._SR45-1	DGQHR-domain-containing-protein-[Pseudoalteromonas-sp.-SR45-1]	2760932
WP_054563221.1	DndB-ParB-nuclease	GCF_001306895.1	361	Bacteria>Proteobacteria>Gammaproteobacteria>Alteromonadales>Pseudoalteromonadaceae	Pseudoalteromonas_sp._UCD-33C	DNA-sulfur-modification-protein-DndB-[Pseudoalteromonas-sp.-UCD-33C]	1716175
WP_054563216.1	DndB-ParB-nuclease	GCF_001306895.1	649	Bacteria>Proteobacteria>Gammaproteobacteria>Alteromonadales>Pseudoalteromonadaceae	Pseudoalteromonas_sp._UCD-33C	hypothetical-protein-[Pseudoalteromonas-sp.-UCD-33C]	1716175
WP_166377560.1	DndB-ParB-nuclease	GCF_011378845.1	363	Bacteria>Proteobacteria>Gammaproteobacteria>Alteromonadales>Pseudoalteromonadaceae	Pseudoalteromonas_sp._Z9A4	DNA-sulfur-modification-protein-DndB-[Pseudoalteromonas-sp.-Z9A4]	2686353
WP_166377556.1	DndB-ParB-nuclease	GCF_011378845.1	557	Bacteria>Proteobacteria>Gammaproteobacteria>Alteromonadales>Pseudoalteromonadaceae	Pseudoalteromonas_sp._Z9A4	hypothetical-protein-[Pseudoalteromonas-sp.-Z9A4]	2686353
WP_011329738.1	DndB-ParB-nuclease	GCF_000026085.1	363	Bacteria>Proteobacteria>Gammaproteobacteria>Alteromonadales>Pseudoalteromonadaceae	Pseudoalteromonas_translucida	DNA-sulfur-modification-protein-DndB-[Pseudoalteromonas-translucida]	166935
WP_086745147.1	DndB-ParB-nuclease	GCF_002156545.1	363	Bacteria>Proteobacteria>Gammaproteobacteria>Alteromonadales>Pseudoalteromonadaceae	Pseudoalteromonas_ulvae	DNA-sulfur-modification-protein-DndB-[Pseudoalteromonas-ulvae]	107327
WP_086745152.1	DndB-ParB-nuclease	GCF_002156545.1	557	Bacteria>Proteobacteria>Gammaproteobacteria>Alteromonadales>Pseudoalteromonadaceae	Pseudoalteromonas_ulvae	hypothetical-protein-[Pseudoalteromonas-ulvae]	107327
MUH72712.1	DndB-ParB-nuclease	GCA_009728465.1	299	Bacteria>Proteobacteria>Gammaproteobacteria>Alteromonadales>Pseudoalteromonadaceae	Psychrosphaera_haliotis	DGQHR-domain-containing-protein-[Psychrosphaera-haliotis]	555083
WP_155696097.1	DndB-ParB-nuclease	GCF_009728465.1	299	Bacteria>Proteobacteria>Gammaproteobacteria>Alteromonadales>Pseudoalteromonadaceae	Psychrosphaera_haliotis	DGQHR-domain-containing-protein-[Psychrosphaera-haliotis]	555083
MUH72707.1	DndB-ParB-nuclease	GCA_009728465.1	361	Bacteria>Proteobacteria>Gammaproteobacteria>Alteromonadales>Pseudoalteromonadaceae	Psychrosphaera_haliotis	DNA-sulfur-modification-protein-DndB-[Psychrosphaera-haliotis]	555083
WP_155695862.1	DndB-ParB-nuclease	GCF_009728465.1	361	Bacteria>Proteobacteria>Gammaproteobacteria>Alteromonadales>Pseudoalteromonadaceae	Psychrosphaera_haliotis	DNA-sulfur-modification-protein-DndB-[Psychrosphaera-haliotis]	555083
WP_105051605.1	DndB-ParB-nuclease	GCF_014652155.1	386	Bacteria>Proteobacteria>Gammaproteobacteria>Alteromonadales>Pseudoalteromonadaceae	Psychrosphaera_saromensis	DGQHR-domain-containing-protein-[Psychrosphaera-saromensis]	716813
WP_105051609.1	DndB-ParB-nuclease	GCF_014652155.1	361	Bacteria>Proteobacteria>Gammaproteobacteria>Alteromonadales>Pseudoalteromonadaceae	Psychrosphaera_saromensis	DNA-sulfur-modification-protein-DndB-[Psychrosphaera-saromensis]	716813
WP_130167448.1	DndB-ParB-nuclease	GCF_005886125.1	568	Bacteria>Proteobacteria>Gammaproteobacteria>Alteromonadales>Pseudoalteromonadaceae	unclassified_Pseudoalteromonas	MULTISPECIES:-AIPR-family-protein-[unclassified-Pseudoalteromonas]	194690
WP_130167444.1	DndB-ParB-nuclease	GCF_005886115.1	361	Bacteria>Proteobacteria>Gammaproteobacteria>Alteromonadales>Pseudoalteromonadaceae	unclassified_Pseudoalteromonas	MULTISPECIES:-DNA-sulfur-modification-protein-DndB-[unclassified-Pseudoalteromonas]	194690
WP_182672700.1	DndB-ParB-nuclease	GCF_014163655.1	363	Bacteria>Proteobacteria>Gammaproteobacteria>Alteromonadales>Pseudoalteromonadaceae	unclassified_Pseudoalteromonas	MULTISPECIES:-DNA-sulfur-modification-protein-DndB-[unclassified-Pseudoalteromonas]	194690
WP_101106965.1	DndB-ParB-nuclease	GCF_002835465.1	349	Bacteria>Proteobacteria>Gammaproteobacteria>Alteromonadales>Psychromonadaceae	Psychromonas_sp._psych-6C06	DGQHR-domain-containing-protein-[Psychromonas-sp.-psych-6C06]	2058089
WP_238596664.1	DndB-ParB-nuclease	GCF_002835465.1	392	Bacteria>Proteobacteria>Gammaproteobacteria>Alteromonadales>Psychromonadaceae	Psychromonas_sp._psych-6C06	DNA-sulfur-modification-protein-DndB-[Psychromonas-sp.-psych-6C06]	2058089
WP_121851615.1	DndB-ParB-nuclease	GCF_004358345.1	360	Bacteria>Proteobacteria>Gammaproteobacteria>Alteromonadales>Shewanellaceae	Parashewanella_spongiae	DNA-sulfur-modification-protein-DndB-[Parashewanella-spongiae]	342950
WP_208137842.1	DndB-ParB-nuclease	GCF_017580205.1	360	Bacteria>Proteobacteria>Gammaproteobacteria>Alteromonadales>Shewanellaceae	Shewanella_algae	DNA-sulfur-modification-protein-DndB-[Shewanella-algae]	38313
WP_208144359.1	DndB-ParB-nuclease	GCF_017580505.1	364	Bacteria>Proteobacteria>Gammaproteobacteria>Alteromonadales>Shewanellaceae	Shewanella_algae	DNA-sulfur-modification-protein-DndB-[Shewanella-algae]	38313
WP_208155567.1	DndB-ParB-nuclease	GCF_017579985.1	360	Bacteria>Proteobacteria>Gammaproteobacteria>Alteromonadales>Shewanellaceae	Shewanella_algae	DNA-sulfur-modification-protein-DndB-[Shewanella-algae]	38313
WP_221059338.1	DndB-ParB-nuclease	GCF_019670665.1	360	Bacteria>Proteobacteria>Gammaproteobacteria>Alteromonadales>Shewanellaceae	Shewanella_algae	DNA-sulfur-modification-protein-DndB-[Shewanella-algae]	38313
WP_115334612.1	DndB-ParB-nuclease	GCF_900456975.1	360	Bacteria>Proteobacteria>Gammaproteobacteria>Alteromonadales>Shewanellaceae	Shewanella_baltica	DNA-sulfur-modification-protein-DndB-[Shewanella-baltica]	62322
WP_115334607.1	DndB-ParB-nuclease	GCF_900456975.1	683	Bacteria>Proteobacteria>Gammaproteobacteria>Alteromonadales>Shewanellaceae	Shewanella_baltica	hypothetical-protein-[Shewanella-baltica]	62322
WP_133180933.1	DndB-ParB-nuclease	GCF_004354305.1	360	Bacteria>Proteobacteria>Gammaproteobacteria>Alteromonadales>Shewanellaceae	Shewanella_decolorationis	DNA-sulfur-modification-protein-DndB-[Shewanella-decolorationis]	256839
WP_133180944.1	DndB-ParB-nuclease	GCF_004354305.1	648	Bacteria>Proteobacteria>Gammaproteobacteria>Alteromonadales>Shewanellaceae	Shewanella_decolorationis	hypothetical-protein-[Shewanella-decolorationis]	256839
WP_059743569.1	DndB-ParB-nuclease	GCF_001529365.1	364	Bacteria>Proteobacteria>Gammaproteobacteria>Alteromonadales>Shewanellaceae	Shewanella_frigidimarina	DNA-sulfur-modification-protein-DndB-[Shewanella-frigidimarina]	56812
WP_012155541.1	DndB-ParB-nuclease	GCF_000018285.1	369	Bacteria>Proteobacteria>Gammaproteobacteria>Alteromonadales>Shewanellaceae	Shewanella_pealeana	DNA-sulfur-modification-protein-DndB-[Shewanella-pealeana]	70864
WP_220781164.1	DndB-ParB-nuclease	GCF_019655015.1	369	Bacteria>Proteobacteria>Gammaproteobacteria>Alteromonadales>Shewanellaceae	Shewanella_sairae	DNA-sulfur-modification-protein-DndB-[Shewanella-sairae]	190310
NRB24575.1	DndB-ParB-nuclease	GCA_013214165.1	373	Bacteria>Proteobacteria>Gammaproteobacteria>Alteromonadales>Shewanellaceae	Shewanella_sp.	DGQHR-domain-containing-protein-[Shewanella-sp.]	50422
NRB24579.1	DndB-ParB-nuclease	GCA_013214165.1	369	Bacteria>Proteobacteria>Gammaproteobacteria>Alteromonadales>Shewanellaceae	Shewanella_sp.	DNA-sulfur-modification-protein-DndB-[Shewanella-sp.]	50422
WP_220049645.1	DndB-ParB-nuclease	GCF_019457475.1	424	Bacteria>Proteobacteria>Gammaproteobacteria>Alteromonadales>Shewanellaceae	Shewanella_sp._FJAT-51860	DGQHR-domain-containing-protein-[Shewanella-sp.-FJAT-51860]	2864212
WP_220049639.1	DndB-ParB-nuclease	GCF_019457475.1	364	Bacteria>Proteobacteria>Gammaproteobacteria>Alteromonadales>Shewanellaceae	Shewanella_sp._FJAT-51860	DNA-sulfur-modification-protein-DndB-[Shewanella-sp.-FJAT-51860]	2864212
WP_211030021.1	DndB-ParB-nuclease	GCF_018100775.1	360	Bacteria>Proteobacteria>Gammaproteobacteria>Alteromonadales>Shewanellaceae	Shewanella_sp._MMG014	DNA-sulfur-modification-protein-DndB-[Shewanella-sp.-MMG014]	2822691
WP_219233670.1	DndB-ParB-nuclease	GCF_019345615.1	360	Bacteria>Proteobacteria>Gammaproteobacteria>Alteromonadales>Shewanellaceae	Shewanella_sp._NKUCC01_JLK	DNA-sulfur-modification-protein-DndB-[Shewanella-sp.-NKUCC01_JLK]	2842123
WP_182743473.1	DndB-ParB-nuclease	GCF_014163715.1	409	Bacteria>Proteobacteria>Gammaproteobacteria>Alteromonadales>Shewanellaceae	Shewanella_sp._SR44-4	DGQHR-domain-containing-protein-[Shewanella-sp.-SR44-4]	2760935
WP_182743470.1	DndB-ParB-nuclease	GCF_014163715.1	369	Bacteria>Proteobacteria>Gammaproteobacteria>Alteromonadales>Shewanellaceae	Shewanella_sp._SR44-4	DNA-sulfur-modification-protein-DndB-[Shewanella-sp.-SR44-4]	2760935
WP_013051385.1	DndB-ParB-nuclease	GCF_000091325.1	369	Bacteria>Proteobacteria>Gammaproteobacteria>Alteromonadales>Shewanellaceae	Shewanella_violacea	DNA-sulfur-modification-protein-DndB-[Shewanella-violacea]	60217
WP_172589476.1	DndB-ParB-nuclease	GCF_012490605.1	360	Bacteria>Proteobacteria>Gammaproteobacteria>Alteromonadales>Shewanellaceae	Shewanella_xiamenensis	DNA-sulfur-modification-protein-DndB-[Shewanella-xiamenensis]	332186
WP_220763097.1	DndB-ParB-nuclease	GCF_019655035.1	369	Bacteria>Proteobacteria>Gammaproteobacteria>Alteromonadales>Shewanellaceae	unclassified_Shewanella	MULTISPECIES:-DNA-sulfur-modification-protein-DndB-[unclassified-Shewanella]	196818
PUA29015.1	DndB-ParB-nuclease	GCA_003056245.1	376	Bacteria>Proteobacteria>Gammaproteobacteria>Cellvibrionales>Cellvibrionaceae	Cellvibrio_sp._79	DNA-sulfur-modification-protein-DndB-[Cellvibrio-sp.-79]	1954207
PUA28819.1	DndB-ParB-nuclease	GCA_003056245.1	328	Bacteria>Proteobacteria>Gammaproteobacteria>Cellvibrionales>Cellvibrionaceae	Cellvibrio_sp._79	hypothetical-protein-B0W54_10405-[Cellvibrio-sp.-79]	1954207
WP_062061403.1	DndB-ParB-nuclease	GCF_000953825.1	366	Bacteria>Proteobacteria>Gammaproteobacteria>Cellvibrionales>Cellvibrionaceae	Cellvibrio_sp._OA-2007	DNA-sulfur-modification-protein-DndB-[Cellvibrio-sp.-OA-2007]	529823
WP_028884885.1	DndB-ParB-nuclease	GCF_000426805.1	364	Bacteria>Proteobacteria>Gammaproteobacteria>Cellvibrionales>Cellvibrionaceae	Teredinibacter_turnerae	DNA-sulfur-modification-protein-DndB-[Teredinibacter-turnerae]	2426
MAL94591.1	DndB-ParB-nuclease	GCA_002692965.1	359	Bacteria>Proteobacteria>Gammaproteobacteria>Cellvibrionales>Halieaceae	Haliea_sp.	DNA-sulfur-modification-protein-DndB-[Haliea-sp.]	1932666
RLQ20254.1	DndB-ParB-nuclease	GCA_003457605.1	365	Bacteria>Proteobacteria>Gammaproteobacteria>Cellvibrionales>Halieaceae	Seongchinamella_sediminis	DNA-sulfur-modification-protein-DndB-[Halioglobus-sediminis]	2283635
WP_117957522.1	DndB-ParB-nuclease	GCF_003457605.1	365	Bacteria>Proteobacteria>Gammaproteobacteria>Cellvibrionales>Halieaceae	Seongchinamella_sediminis	DNA-sulfur-modification-protein-DndB-[Halioglobus-sediminis]	2283635
CAA0097903.1	DndB-ParB-nuclease	GCA_902705865.1	431	Bacteria>Proteobacteria>Gammaproteobacteria>Cellvibrionales>Spongiibacteraceae	BD1-7_clade_bacterium	Uncharacterised-protein-[BD1-7-clade-bacterium]	2029982
WP_097112692.1	DndB-ParB-nuclease	GCF_900215315.1	433	Bacteria>Proteobacteria>Gammaproteobacteria>Chromatiales>Chromatiaceae	Arsukibacterium_tuosuense	DGQHR-domain-containing-protein-[Rheinheimera-tuosuensis]	1323745
WP_097112698.1	DndB-ParB-nuclease	GCF_900215315.1	363	Bacteria>Proteobacteria>Gammaproteobacteria>Chromatiales>Chromatiaceae	Arsukibacterium_tuosuense	DNA-sulfur-modification-protein-DndB-[Rheinheimera-tuosuensis]	1323745
WP_046519020.1	DndB-ParB-nuclease	GCF_000986865.1	360	Bacteria>Proteobacteria>Gammaproteobacteria>Chromatiales>Chromatiaceae	Pararheinheimera_mesophila	DNA-sulfur-modification-protein-DndB-[Pararheinheimera-mesophila]	1547515
WP_226767953.1	DndB-ParB-nuclease	GCF_020531985.1	570	Bacteria>Proteobacteria>Gammaproteobacteria>Chromatiales>Chromatiaceae	Rheinheimera_aquimaris	AIPR-family-protein-[Rheinheimera-aquimaris]	412437
WP_226767948.1	DndB-ParB-nuclease	GCF_020531985.1	361	Bacteria>Proteobacteria>Gammaproteobacteria>Chromatiales>Chromatiaceae	Rheinheimera_aquimaris	DNA-sulfur-modification-protein-DndB-[Rheinheimera-aquimaris]	412437
WP_230819862.1	DndB-ParB-nuclease	GCF_021021375.1	363	Bacteria>Proteobacteria>Gammaproteobacteria>Chromatiales>Chromatiaceae	Rheinheimera_aquimaris	DNA-sulfur-modification-protein-DndB-[Rheinheimera-aquimaris]	412437
WP_230819867.1	DndB-ParB-nuclease	GCF_021021375.1	691	Bacteria>Proteobacteria>Gammaproteobacteria>Chromatiales>Chromatiaceae	Rheinheimera_aquimaris	hypothetical-protein-[Rheinheimera-aquimaris]	412437
WP_027671813.1	DndB-ParB-nuclease	GCF_000425345.1	362	Bacteria>Proteobacteria>Gammaproteobacteria>Chromatiales>Chromatiaceae	Rheinheimera_baltica	DNA-sulfur-modification-protein-DndB-[Rheinheimera-baltica]	67576
WP_015281554.1	DndB-ParB-nuclease	GCF_000327045.1	398	Bacteria>Proteobacteria>Gammaproteobacteria>Chromatiales>Chromatiaceae	Thioflavicoccus_mobilis	DNA-sulfur-modification-protein-DndB-[Thioflavicoccus-mobilis]	80679
WP_015281561.1	DndB-ParB-nuclease	GCF_000327045.1	770	Bacteria>Proteobacteria>Gammaproteobacteria>Chromatiales>Chromatiaceae	Thioflavicoccus_mobilis	hypothetical-protein-[Thioflavicoccus-mobilis]	80679
WP_018937665.1	DndB-ParB-nuclease	GCF_000377805.1	341	Bacteria>Proteobacteria>Gammaproteobacteria>Chromatiales>Ectothiorhodospiraceae	unclassified_Thioalkalivibrio	MULTISPECIES:-DNA-sulfur-modification-protein-DndB-[unclassified-Thioalkalivibrio]	2621013
WP_159565435.1	DndB-ParB-nuclease	GCF_009800925.1	361	Bacteria>Proteobacteria>Gammaproteobacteria>Enterobacterales>Budviciaceae	Budvicia_diplopodorum	DNA-sulfur-modification-protein-DndB-[Budvicia-diplopodorum]	1119056
KFC97403.1	DndB-ParB-nuclease	GCA_000735425.1	331	Bacteria>Proteobacteria>Gammaproteobacteria>Enterobacterales>Budviciaceae	Leminorella_grimontii_ATCC_33999_=_DSM_5078	DndB-family-DNA-sulfur-modification-protein-[Leminorella-grimontii-ATCC-33999-=-DSM-5078]	1005999
WP_027274166.1	DndB-ParB-nuclease	GCF_000439085.1	361	Bacteria>Proteobacteria>Gammaproteobacteria>Enterobacterales>Budviciaceae	Leminorella_grimontii	DNA-sulfur-modification-protein-DndB-[Leminorella-grimontii]	82981
WP_108900288.1	DndB-ParB-nuclease	GCF_003096015.2	401	Bacteria>Proteobacteria>Gammaproteobacteria>Enterobacterales>Budviciaceae	Limnobaculum_parvum	DGQHR-domain-containing-protein-[Limnobaculum-parvum]	2172103
WP_108902053.1	DndB-ParB-nuclease	GCF_003096015.2	361	Bacteria>Proteobacteria>Gammaproteobacteria>Enterobacterales>Budviciaceae	Limnobaculum_parvum	DNA-sulfur-modification-protein-DndB-[Limnobaculum-parvum]	2172103
WP_039298049.1	DndB-ParB-nuclease	GCF_000758325.1	361	Bacteria>Proteobacteria>Gammaproteobacteria>Enterobacterales>Enterobacteriaceae	Cedecea_neteri	DNA-sulfur-modification-protein-DndB-[Cedecea-neteri]	158822
MBJ8848870.1	DndB-ParB-nuclease	GCA_016501405.1	382	Bacteria>Proteobacteria>Gammaproteobacteria>Enterobacterales>Enterobacteriaceae	Citrobacter_braakii	DGQHR-domain-containing-protein-[Citrobacter-braakii]	57706
EGT0678923.1	DndB-ParB-nuclease	GCA_015943465.1	361	Bacteria>Proteobacteria>Gammaproteobacteria>Enterobacterales>Enterobacteriaceae	Citrobacter_braakii	DNA-sulfur-modification-protein-DndB-[Citrobacter-braakii]	57706
MBJ8848866.1	DndB-ParB-nuclease	GCA_016501405.1	361	Bacteria>Proteobacteria>Gammaproteobacteria>Enterobacterales>Enterobacteriaceae	Citrobacter_braakii	DNA-sulfur-modification-protein-DndB-[Citrobacter-braakii]	57706
MBJ9263833.1	DndB-ParB-nuclease	GCA_016504885.1	361	Bacteria>Proteobacteria>Gammaproteobacteria>Enterobacterales>Enterobacteriaceae	Citrobacter_braakii	DNA-sulfur-modification-protein-DndB-[Citrobacter-braakii]	57706
WP_151222986.1	DndB-ParB-nuclease	GCF_019048045.1	361	Bacteria>Proteobacteria>Gammaproteobacteria>Enterobacterales>Enterobacteriaceae	Citrobacter_braakii	DNA-sulfur-modification-protein-DndB-[Citrobacter-braakii]	57706
WP_217124780.1	DndB-ParB-nuclease	GCF_019048045.1	704	Bacteria>Proteobacteria>Gammaproteobacteria>Enterobacterales>Enterobacteriaceae	Citrobacter_braakii	hypothetical-protein-[Citrobacter-braakii]	57706
WP_052250277.1	DndB-ParB-nuclease	GCF_016501405.1	382	Bacteria>Proteobacteria>Gammaproteobacteria>Enterobacterales>Enterobacteriaceae	Citrobacter_braakii	MULTISPECIES:-DGQHR-domain-containing-protein-[Enterobacteriaceae]	57706
HAT2746687.1	DndB-ParB-nuclease	GCA_015899045.1	361	Bacteria>Proteobacteria>Gammaproteobacteria>Enterobacterales>Enterobacteriaceae	Citrobacter_farmeri	DNA-sulfur-modification-protein-DndB-[Citrobacter-farmeri]	67824
WP_153880241.1	DndB-ParB-nuclease	GCF_009660335.1	387	Bacteria>Proteobacteria>Gammaproteobacteria>Enterobacterales>Enterobacteriaceae	Citrobacter_freundii	DGQHR-domain-containing-protein-[Citrobacter-freundii]	546
WP_186390423.1	DndB-ParB-nuclease	GCF_901456225.1	407	Bacteria>Proteobacteria>Gammaproteobacteria>Enterobacterales>Enterobacteriaceae	Citrobacter_freundii	DGQHR-domain-containing-protein-[Citrobacter-freundii]	546
HAT8021628.1	DndB-ParB-nuclease	GCA_015965475.1	361	Bacteria>Proteobacteria>Gammaproteobacteria>Enterobacterales>Enterobacteriaceae	Citrobacter_freundii	DNA-sulfur-modification-protein-DndB-[Citrobacter-freundii]	546
HAU5612285.1	DndB-ParB-nuclease	GCA_016075035.1	361	Bacteria>Proteobacteria>Gammaproteobacteria>Enterobacterales>Enterobacteriaceae	Citrobacter_freundii	DNA-sulfur-modification-protein-DndB-[Citrobacter-freundii]	546
WP_153880237.1	DndB-ParB-nuclease	GCF_009660335.1	361	Bacteria>Proteobacteria>Gammaproteobacteria>Enterobacterales>Enterobacteriaceae	Citrobacter_freundii	DNA-sulfur-modification-protein-DndB-[Citrobacter-freundii]	546
WP_181819927.1	DndB-ParB-nuclease	GCF_013882995.1	361	Bacteria>Proteobacteria>Gammaproteobacteria>Enterobacterales>Enterobacteriaceae	Citrobacter_freundii	DNA-sulfur-modification-protein-DndB-[Citrobacter-freundii]	546
GJK70323.1	DndB-ParB-nuclease	GCA_020889125.1	361	Bacteria>Proteobacteria>Gammaproteobacteria>Enterobacterales>Enterobacteriaceae	Citrobacter_freundii	hypothetical-protein-TUM17564_23500-[Citrobacter-freundii]	546
GJK70327.1	DndB-ParB-nuclease	GCA_020889125.1	403	Bacteria>Proteobacteria>Gammaproteobacteria>Enterobacterales>Enterobacteriaceae	Citrobacter_freundii	hypothetical-protein-TUM17564_23540-[Citrobacter-freundii]	546
HAT2285395.1	DndB-ParB-nuclease	GCA_015897485.1	361	Bacteria>Proteobacteria>Gammaproteobacteria>Enterobacterales>Enterobacteriaceae	Citrobacter_freundii	TPA:-DNA-sulfur-modification-protein-DndB-[Citrobacter-freundii]	546
SQB25929.1	DndB-ParB-nuclease	GCA_900446925.1	150	Bacteria>Proteobacteria>Gammaproteobacteria>Enterobacterales>Enterobacteriaceae	Citrobacter_koseri	DGQHR-domain-[Citrobacter-koseri]	545
MBJ9172379.1	DndB-ParB-nuclease	GCA_016504345.1	388	Bacteria>Proteobacteria>Gammaproteobacteria>Enterobacterales>Enterobacteriaceae	Citrobacter_koseri	DGQHR-domain-containing-protein-[Citrobacter-koseri]	545
MBJ9245476.1	DndB-ParB-nuclease	GCA_016504805.1	327	Bacteria>Proteobacteria>Gammaproteobacteria>Enterobacterales>Enterobacteriaceae	Citrobacter_koseri	DGQHR-domain-containing-protein-[Citrobacter-koseri]	545
WP_200074689.1	DndB-ParB-nuclease	GCF_016504345.1	388	Bacteria>Proteobacteria>Gammaproteobacteria>Enterobacterales>Enterobacteriaceae	Citrobacter_koseri	DGQHR-domain-containing-protein-[Citrobacter-koseri]	545

Accession	Annotation	GCA	Length	Phyletic.spread	Strain.name	Defline	Species.Taxid
MBJ9172375.1	DndB-ParB-nuclease	GCA_016504345.1	361	Bacteria>Proteobacteria>Gammaproteobacteria>Enterobacterales>Enterobacteriaceae	Citrobacter_koseri	DNA-sulfur-modification-protein-DndB-[Citrobacter-koseri]	545
MBJ9245479.1	DndB-ParB-nuclease	GCA_016504805.1	359	Bacteria>Proteobacteria>Gammaproteobacteria>Enterobacterales>Enterobacteriaceae	Citrobacter_koseri	DNA-sulfur-modification-protein-DndB-[Citrobacter-koseri]	545
SQB25925.1	DndB-ParB-nuclease	GCA_900446925.1	386	Bacteria>Proteobacteria>Gammaproteobacteria>Enterobacterales>Enterobacteriaceae	Citrobacter_koseri	DNA-sulfur-modification-protein-DndB-[Citrobacter-koseri]	545
WP_012134279.1	DndB-ParB-nuclease	GCF_000018045.1	361	Bacteria>Proteobacteria>Gammaproteobacteria>Enterobacterales>Enterobacteriaceae	Citrobacter_koseri	DNA-sulfur-modification-protein-DndB-[Citrobacter-koseri]	545
WP_200074685.1	DndB-ParB-nuclease	GCF_016504345.1	361	Bacteria>Proteobacteria>Gammaproteobacteria>Enterobacterales>Enterobacteriaceae	Citrobacter_koseri	DNA-sulfur-modification-protein-DndB-[Citrobacter-koseri]	545
MBJ9245484.1	DndB-ParB-nuclease	GCA_016504805.1	565	Bacteria>Proteobacteria>Gammaproteobacteria>Enterobacterales>Enterobacteriaceae	Citrobacter_koseri	hypothetical-protein-[Citrobacter-koseri]	545
SQB25919.1	DndB-ParB-nuclease	GCA_900446925.1	437	Bacteria>Proteobacteria>Gammaproteobacteria>Enterobacterales>Enterobacteriaceae	Citrobacter_koseri	Uncharacterised-protein-[Citrobacter-koseri]	545
WP_195296448.1	DndB-ParB-nuclease	GCF_015549445.1	327	Bacteria>Proteobacteria>Gammaproteobacteria>Enterobacterales>Enterobacteriaceae	Citrobacter	MULTISPECIES:-DGQHR-domain-containing-protein-[Citrobacter]	544
WP_196614802.1	DndB-ParB-nuclease	GCF_019399955.1	361	Bacteria>Proteobacteria>Gammaproteobacteria>Enterobacterales>Enterobacteriaceae	Citrobacter	MULTISPECIES:-DNA-sulfur-modification-protein-DndB-[Citrobacter]	544
WP_229057469.1	DndB-ParB-nuclease	GCF_015549445.1	334	Bacteria>Proteobacteria>Gammaproteobacteria>Enterobacterales>Enterobacteriaceae	Citrobacter	MULTISPECIES:-DNA-sulfur-modification-protein-DndB-[Citrobacter]	544
WP_195296443.1	DndB-ParB-nuclease	GCF_015549445.1	565	Bacteria>Proteobacteria>Gammaproteobacteria>Enterobacterales>Enterobacteriaceae	Citrobacter	MULTISPECIES:-hypothetical-protein-[Citrobacter]	544
WP_196614796.1	DndB-ParB-nuclease	GCF_019399955.1	704	Bacteria>Proteobacteria>Gammaproteobacteria>Enterobacterales>Enterobacteriaceae	Citrobacter	MULTISPECIES:-hypothetical-protein-[Citrobacter]	544
WP_190973163.1	DndB-ParB-nuclease	GCF_008693605.1	415	Bacteria>Proteobacteria>Gammaproteobacteria>Enterobacterales>Enterobacteriaceae	Citrobacter_portucalensis	DGQHR-domain-containing-protein-[Citrobacter-portucalensis]	1639133
WP_200920854.1	DndB-ParB-nuclease	GCF_015134935.1	386	Bacteria>Proteobacteria>Gammaproteobacteria>Enterobacterales>Enterobacteriaceae	Citrobacter_portucalensis	DGQHR-domain-containing-protein-[Citrobacter-portucalensis]	1639133
WP_200920851.1	DndB-ParB-nuclease	GCF_015134935.1	361	Bacteria>Proteobacteria>Gammaproteobacteria>Enterobacterales>Enterobacteriaceae	Citrobacter_portucalensis	DNA-sulfur-modification-protein-DndB-[Citrobacter-portucalensis]	1639133
BBV42656.1	DndB-ParB-nuclease	GCA_015134935.1	361	Bacteria>Proteobacteria>Gammaproteobacteria>Enterobacterales>Enterobacteriaceae	Citrobacter_portucalensis	hypothetical-protein-STW0522CIT26_41280-[Citrobacter-portucalensis]	1639133
BBV42661.1	DndB-ParB-nuclease	GCA_015134935.1	386	Bacteria>Proteobacteria>Gammaproteobacteria>Enterobacterales>Enterobacteriaceae	Citrobacter_portucalensis	hypothetical-protein-STW0522CIT26_41330-[Citrobacter-portucalensis]	1639133
EHG7610666.1	DndB-ParB-nuclease	GCA_017566945.1	405	Bacteria>Proteobacteria>Gammaproteobacteria>Enterobacterales>Enterobacteriaceae	Citrobacter_sedlakii	DGQHR-domain-containing-protein-[Citrobacter-sedlakii]	67826
EHG7610671.1	DndB-ParB-nuclease	GCA_017566945.1	361	Bacteria>Proteobacteria>Gammaproteobacteria>Enterobacterales>Enterobacteriaceae	Citrobacter_sedlakii	DNA-sulfur-modification-protein-DndB-[Citrobacter-sedlakii]	67826
WP_016149487.1	DndB-ParB-nuclease	GCF_000398825.1	361	Bacteria>Proteobacteria>Gammaproteobacteria>Enterobacterales>Enterobacteriaceae	Citrobacter_sp._KTE30	DNA-sulfur-modification-protein-DndB-[Citrobacter-sp.-KTE30]	1169319
WP_181547210.1	DndB-ParB-nuclease	GCF_013747755.1	388	Bacteria>Proteobacteria>Gammaproteobacteria>Enterobacterales>Enterobacteriaceae	Citrobacter_sp._RHBSTW-00524	DGQHR-domain-containing-protein-[Citrobacter-sp.-RHBSTW-00524]	2742653
WP_216404148.1	DndB-ParB-nuclease	GCF_018919545.1	153	Bacteria>Proteobacteria>Gammaproteobacteria>Enterobacterales>Enterobacteriaceae	Citrobacter_sp._S46_ASV_140	DNA-sulfur-modification-protein-DndB,-partial-[Citrobacter-sp.-S46_ASV_140]	2846983
WP_137365101.1	DndB-ParB-nuclease	GCF_005280815.1	575	Bacteria>Proteobacteria>Gammaproteobacteria>Enterobacterales>Enterobacteriaceae	Citrobacter_sp._TBCS-15	hypothetical-protein-[Citrobacter-sp.-TBCS-15]	2576407
WP_158698858.1	DndB-ParB-nuclease	GCF_002979135.1	407	Bacteria>Proteobacteria>Gammaproteobacteria>Enterobacterales>Enterobacteriaceae	Cronobacter_dublinensis	DGQHR-domain-containing-protein-[Cronobacter-dublinensis]	413497
EGT4359822.1	DndB-ParB-nuclease	GCA_016076555.1	360	Bacteria>Proteobacteria>Gammaproteobacteria>Enterobacterales>Enterobacteriaceae	Cronobacter_dublinensis	DNA-sulfur-modification-protein-DndB-[Cronobacter-dublinensis]	413497
WP_105639239.1	DndB-ParB-nuclease	GCF_002978625.1	361	Bacteria>Proteobacteria>Gammaproteobacteria>Enterobacterales>Enterobacteriaceae	Cronobacter_dublinensis	DNA-sulfur-modification-protein-DndB-[Cronobacter-dublinensis]	413497
WP_105660762.1	DndB-ParB-nuclease	GCF_002979095.1	360	Bacteria>Proteobacteria>Gammaproteobacteria>Enterobacterales>Enterobacteriaceae	Cronobacter_dublinensis	DNA-sulfur-modification-protein-DndB-[Cronobacter-dublinensis]	413497
WP_105665227.1	DndB-ParB-nuclease	GCF_002978805.1	361	Bacteria>Proteobacteria>Gammaproteobacteria>Enterobacterales>Enterobacteriaceae	Cronobacter_dublinensis	DNA-sulfur-modification-protein-DndB-[Cronobacter-dublinensis]	413497
EGT4359817.1	DndB-ParB-nuclease	GCA_016076555.1	399	Bacteria>Proteobacteria>Gammaproteobacteria>Enterobacterales>Enterobacteriaceae	Cronobacter_dublinensis	hypothetical-protein-[Cronobacter-dublinensis]	413497
EGT5662159.1	DndB-ParB-nuclease	GCA_016087115.1	361	Bacteria>Proteobacteria>Gammaproteobacteria>Enterobacterales>Enterobacteriaceae	Cronobacter_dublinensis_subsp._dublinensis	DNA-sulfur-modification-protein-DndB-[Cronobacter-dublinensis-subsp.-dublinensis]	413498
EGT5713007.1	DndB-ParB-nuclease	GCA_016086335.1	361	Bacteria>Proteobacteria>Gammaproteobacteria>Enterobacterales>Enterobacteriaceae	Cronobacter_dublinensis_subsp._dublinensis	DNA-sulfur-modification-protein-DndB-[Cronobacter-dublinensis-subsp.-dublinensis]	413498
NCH35689.1	DndB-ParB-nuclease	GCA_009938585.1	405	Bacteria>Proteobacteria>Gammaproteobacteria>Enterobacterales>Enterobacteriaceae	Cronobacter_sakazakii	DGQHR-domain-containing-protein-[Cronobacter-sakazakii]	28141
WP_161582849.1	DndB-ParB-nuclease	GCF_009938585.1	397	Bacteria>Proteobacteria>Gammaproteobacteria>Enterobacterales>Enterobacteriaceae	Cronobacter_sakazakii	DGQHR-domain-containing-protein-[Cronobacter-sakazakii]	28141
KAB1486163.1	DndB-ParB-nuclease	GCA_008830265.1	361	Bacteria>Proteobacteria>Gammaproteobacteria>Enterobacterales>Enterobacteriaceae	Cronobacter_sakazakii	DNA-sulfur-modification-protein-DndB-[Cronobacter-sakazakii]	28141
NCH35693.1	DndB-ParB-nuclease	GCA_009938585.1	361	Bacteria>Proteobacteria>Gammaproteobacteria>Enterobacterales>Enterobacteriaceae	Cronobacter_sakazakii	DNA-sulfur-modification-protein-DndB-[Cronobacter-sakazakii]	28141
WP_038859663.1	DndB-ParB-nuclease	GCF_000409405.1	361	Bacteria>Proteobacteria>Gammaproteobacteria>Enterobacterales>Enterobacteriaceae	Cronobacter_sakazakii	DNA-sulfur-modification-protein-DndB-[Cronobacter-sakazakii]	28141
WP_085017304.1	DndB-ParB-nuclease	GCF_002094725.1	361	Bacteria>Proteobacteria>Gammaproteobacteria>Enterobacterales>Enterobacteriaceae	Cronobacter_sakazakii	DNA-sulfur-modification-protein-DndB-[Cronobacter-sakazakii]	28141
WP_105602674.1	DndB-ParB-nuclease	GCF_002977475.1	361	Bacteria>Proteobacteria>Gammaproteobacteria>Enterobacterales>Enterobacteriaceae	Cronobacter_sakazakii	DNA-sulfur-modification-protein-DndB-[Cronobacter-sakazakii]	28141
WP_158683941.1	DndB-ParB-nuclease	GCF_002976555.1	384	Bacteria>Proteobacteria>Gammaproteobacteria>Enterobacterales>Enterobacteriaceae	Cronobacter_turicensis	DNA-phosphorothioation-associated-DGQHR-protein-1-[Cronobacter-turicensis]	413502
NUW57310.1	DndB-ParB-nuclease	GCA_013363985.1	360	Bacteria>Proteobacteria>Gammaproteobacteria>Enterobacterales>Enterobacteriaceae	Cronobacter_turicensis	DNA-sulfur-modification-protein-DndB-[Cronobacter-turicensis]	413502
WP_012815127.1	DndB-ParB-nuclease	GCF_002976555.1	361	Bacteria>Proteobacteria>Gammaproteobacteria>Enterobacterales>Enterobacteriaceae	Cronobacter_turicensis	DNA-sulfur-modification-protein-DndB-[Cronobacter-turicensis]	413502
WP_175572883.1	DndB-ParB-nuclease	GCF_013363985.1	360	Bacteria>Proteobacteria>Gammaproteobacteria>Enterobacterales>Enterobacteriaceae	Cronobacter_turicensis	DNA-sulfur-modification-protein-DndB-[Cronobacter-turicensis]	413502
CBA27629.1	DndB-ParB-nuclease	GCA_000027065.2	390	Bacteria>Proteobacteria>Gammaproteobacteria>Enterobacterales>Enterobacteriaceae	Cronobacter_turicensis_z3032	hypothetical-protein-CTU_05230-[Cronobacter-turicensis-z3032]	693216
CBA27636.1	DndB-ParB-nuclease	GCA_000027065.2	361	Bacteria>Proteobacteria>Gammaproteobacteria>Enterobacterales>Enterobacteriaceae	Cronobacter_turicensis_z3032	hypothetical-protein-CTU_05270-[Cronobacter-turicensis-z3032]	693216
WP_071992076.1	DndB-ParB-nuclease	GCF_000026245.1	251	Bacteria>Proteobacteria>Gammaproteobacteria>Enterobacterales	Enterobacteriaceae	DGQHR-domain-containing-protein-[Escherichia-coli]	543
WP_181547202.1	DndB-ParB-nuclease	GCF_013747755.1	363	Bacteria>Proteobacteria>Gammaproteobacteria>Enterobacterales	Enterobacteriaceae	DNA-sulfur-modification-protein-DndB-[Citrobacter-sp.-RHBSTW-00524]	543
WP_024908745.1	DndB-ParB-nuclease	GCF_000568175.1	568	Bacteria>Proteobacteria>Gammaproteobacteria>Enterobacterales>Enterobacteriaceae	Enterobacter_asburiae	AIPR-family-protein-[Enterobacter-asburiae]	61645
WP_166770723.1	DndB-ParB-nuclease	GCF_011758755.1	397	Bacteria>Proteobacteria>Gammaproteobacteria>Enterobacterales>Enterobacteriaceae	Enterobacter_asburiae	DGQHR-domain-containing-protein-[Enterobacter-asburiae]	61645
WP_024908749.1	DndB-ParB-nuclease	GCF_000568175.1	361	Bacteria>Proteobacteria>Gammaproteobacteria>Enterobacterales>Enterobacteriaceae	Enterobacter_asburiae	DNA-sulfur-modification-protein-DndB-[Enterobacter-asburiae]	61645
WP_157188548.1	DndB-ParB-nuclease	GCF_009755685.1	361	Bacteria>Proteobacteria>Gammaproteobacteria>Enterobacterales>Enterobacteriaceae	Enterobacter_asburiae	DNA-sulfur-modification-protein-DndB-[Enterobacter-asburiae]	61645
WP_072059932.1	DndB-ParB-nuclease	GCF_001023305.1	414	Bacteria>Proteobacteria>Gammaproteobacteria>Enterobacterales>Enterobacteriaceae	Enterobacter_bugandensis	DGQHR-domain-containing-protein-[Enterobacter-bugandensis]	881260
WP_047364793.1	DndB-ParB-nuclease	GCF_000210775.1	361	Bacteria>Proteobacteria>Gammaproteobacteria>Enterobacterales>Enterobacteriaceae	Enterobacter_cloacae_complex	DNA-sulfur-modification-protein-DndB-[Enterobacter-hormaechei]	354276
WP_188512215.1	DndB-ParB-nuclease	GCF_014639085.1	361	Bacteria>Proteobacteria>Gammaproteobacteria>Enterobacterales>Enterobacteriaceae	Enterobacter_cloacae_complex	DNA-sulfur-modification-protein-DndB-[Enterobacter-hormaechei]	354276
WP_063405461.1	DndB-ParB-nuclease	GCF_014982425.1	361	Bacteria>Proteobacteria>Gammaproteobacteria>Enterobacterales>Enterobacteriaceae	Enterobacter_cloacae_complex	MULTISPECIES:-DNA-sulfur-modification-protein-DndB-[Enterobacter-cloacae-complex]	354276
WP_095441788.1	DndB-ParB-nuclease	GCF_902387735.1	361	Bacteria>Proteobacteria>Gammaproteobacteria>Enterobacterales>Enterobacteriaceae	Enterobacter_cloacae_complex	MULTISPECIES:-DNA-sulfur-modification-protein-DndB-[Enterobacter-cloacae-complex]	354276
MBE4925403.1	DndB-ParB-nuclease	GCA_014982425.1	361	Bacteria>Proteobacteria>Gammaproteobacteria>Enterobacterales>Enterobacteriaceae	Enterobacter_cloacae_complex_sp._I1M	DNA-sulfur-modification-protein-DndB-[Enterobacter-cloacae-complex-sp.-I1M]	2779604
WP_192967479.1	DndB-ParB-nuclease	GCF_014893435.1	361	Bacteria>Proteobacteria>Gammaproteobacteria>Enterobacterales>Enterobacteriaceae	Enterobacter_cloacae_complex_sp._P23RS	DNA-sulfur-modification-protein-DndB-[Enterobacter-cloacae-complex-sp.-P23RS]	2779569
MBE4966136.1	DndB-ParB-nuclease	GCA_014982165.1	107	Bacteria>Proteobacteria>Gammaproteobacteria>Enterobacterales>Enterobacteriaceae	Enterobacter_cloacae_complex_sp._P24RS	DGQHR-domain-containing-protein-[Enterobacter-cloacae-complex-sp.-P24RS]	2779568
MBE4966132.1	DndB-ParB-nuclease	GCA_014982165.1	361	Bacteria>Proteobacteria>Gammaproteobacteria>Enterobacterales>Enterobacteriaceae	Enterobacter_cloacae_complex_sp._P24RS	DNA-sulfur-modification-protein-DndB-[Enterobacter-cloacae-complex-sp.-P24RS]	2779568
WP_193528004.1	DndB-ParB-nuclease	GCF_014982165.1	361	Bacteria>Proteobacteria>Gammaproteobacteria>Enterobacterales>Enterobacteriaceae	Enterobacter_cloacae_complex_sp._P24RS	DNA-sulfur-modification-protein-DndB-[Enterobacter-cloacae-complex-sp.-P24RS]	2779568
MBE4880280.1	DndB-ParB-nuclease	GCA_014930795.1	361	Bacteria>Proteobacteria>Gammaproteobacteria>Enterobacterales>Enterobacteriaceae	Enterobacter_cloacae_complex_sp._P39RS	DNA-sulfur-modification-protein-DndB-[Enterobacter-cloacae-complex-sp.-P39RS]	2779552
MBE3488289.1	DndB-ParB-nuclease	GCA_014896025.1	361	Bacteria>Proteobacteria>Gammaproteobacteria>Enterobacterales>Enterobacteriaceae	Enterobacter_cloacae_complex_sp._P8BA	DNA-sulfur-modification-protein-DndB-[Enterobacter-cloacae-complex-sp.-P8BA]	2779587
WP_202951926.1	DndB-ParB-nuclease	GCF_014982315.1	477	Bacteria>Proteobacteria>Gammaproteobacteria>Enterobacterales>Enterobacteriaceae	Enterobacter_cloacae_complex_sp._P8RS	AIPR-family-protein,-partial-[Enterobacter-cloacae-complex-sp.-P8RS]	2779586
EGS1685957.1	DndB-ParB-nuclease	GCA_015862495.1	397	Bacteria>Proteobacteria>Gammaproteobacteria>Enterobacterales>Enterobacteriaceae	Enterobacter_cloacae	DGQHR-domain-containing-protein-[Enterobacter-cloacae]	550
EGS1685961.1	DndB-ParB-nuclease	GCA_015862495.1	361	Bacteria>Proteobacteria>Gammaproteobacteria>Enterobacterales>Enterobacteriaceae	Enterobacter_cloacae	DNA-sulfur-modification-protein-DndB-[Enterobacter-cloacae]	550
WP_118283948.1	DndB-ParB-nuclease	GCF_003471865.1	361	Bacteria>Proteobacteria>Gammaproteobacteria>Enterobacterales>Enterobacteriaceae	Enterobacter_cloacae	DNA-sulfur-modification-protein-DndB-[Enterobacter-cloacae]	550
WP_222169199.1	DndB-ParB-nuclease	GCF_019796025.1	361	Bacteria>Proteobacteria>Gammaproteobacteria>Enterobacterales>Enterobacteriaceae	Enterobacter_cloacae	DNA-sulfur-modification-protein-DndB-[Enterobacter-cloacae]	550
WP_236277146.1	DndB-ParB-nuclease	GCF_021649905.1	361	Bacteria>Proteobacteria>Gammaproteobacteria>Enterobacterales>Enterobacteriaceae	Enterobacter_cloacae	DNA-sulfur-modification-protein-DndB-[Enterobacter-cloacae]	550
WP_134691035.1	DndB-ParB-nuclease	GCF_004523845.1	568	Bacteria>Proteobacteria>Gammaproteobacteria>Enterobacterales>Enterobacteriaceae	Enterobacter_hormaechei	AIPR-family-protein-[Enterobacter-hormaechei]	158836
EHN8908673.1	DndB-ParB-nuclease	GCA_018455485.1	388	Bacteria>Proteobacteria>Gammaproteobacteria>Enterobacterales>Enterobacteriaceae	Enterobacter_hormaechei	DGQHR-domain-containing-protein-[Enterobacter-hormaechei]	158836
WP_234978795.1	DndB-ParB-nuclease	GCF_021496135.1	385	Bacteria>Proteobacteria>Gammaproteobacteria>Enterobacterales>Enterobacteriaceae	Enterobacter_hormaechei	DGQHR-domain-containing-protein-[Enterobacter-hormaechei]	158836
SAE88055.1	DndB-ParB-nuclease	GCA_900077405.1	563	Bacteria>Proteobacteria>Gammaproteobacteria>Enterobacterales>Enterobacteriaceae	Enterobacter_hormaechei	DGQHR-domain-[Enterobacter-hormaechei]	158836
CBK87525.1	DndB-ParB-nuclease	GCA_000210775.1	331	Bacteria>Proteobacteria>Gammaproteobacteria>Enterobacterales>Enterobacteriaceae	Enterobacter_hormaechei	DNA-sulfur-modification-protein-DndB-[Enterobacter-hormaechei]	158836
EHN8866523.1	DndB-ParB-nuclease	GCA_018455025.1	361	Bacteria>Proteobacteria>Gammaproteobacteria>Enterobacterales>Enterobacteriaceae	Enterobacter_hormaechei	DNA-sulfur-modification-protein-DndB-[Enterobacter-hormaechei]	158836
EHN8908669.1	DndB-ParB-nuclease	GCA_018455485.1	363	Bacteria>Proteobacteria>Gammaproteobacteria>Enterobacterales>Enterobacteriaceae	Enterobacter_hormaechei	DNA-sulfur-modification-protein-DndB-[Enterobacter-hormaechei]	158836
HAS1412285.1	DndB-ParB-nuclease	GCA_015684615.1	361	Bacteria>Proteobacteria>Gammaproteobacteria>Enterobacterales>Enterobacteriaceae	Enterobacter_hormaechei	DNA-sulfur-modification-protein-DndB-[Enterobacter-hormaechei]	158836
MBG0571751.1	DndB-ParB-nuclease	GCA_015704035.1	361	Bacteria>Proteobacteria>Gammaproteobacteria>Enterobacterales>Enterobacteriaceae	Enterobacter_hormaechei	DNA-sulfur-modification-protein-DndB-[Enterobacter-hormaechei]	158836
SAE87922.1	DndB-ParB-nuclease	GCA_900077405.1	359	Bacteria>Proteobacteria>Gammaproteobacteria>Enterobacterales>Enterobacteriaceae	Enterobacter_hormaechei	DNA-sulfur-modification-protein-DndB-[Enterobacter-hormaechei]	158836
WP_063152435.1	DndB-ParB-nuclease	GCF_900075595.1	361	Bacteria>Proteobacteria>Gammaproteobacteria>Enterobacterales>Enterobacteriaceae	Enterobacter_hormaechei	DNA-sulfur-modification-protein-DndB-[Enterobacter-hormaechei]	158836
WP_090279895.1	DndB-ParB-nuclease	GCF_900102705.1	361	Bacteria>Proteobacteria>Gammaproteobacteria>Enterobacterales>Enterobacteriaceae	Enterobacter_hormaechei	DNA-sulfur-modification-protein-DndB-[Enterobacter-hormaechei]	158836
WP_126495430.1	DndB-ParB-nuclease	GCF_003965295.1	361	Bacteria>Proteobacteria>Gammaproteobacteria>Enterobacterales>Enterobacteriaceae	Enterobacter_hormaechei	DNA-sulfur-modification-protein-DndB-[Enterobacter-hormaechei]	158836
WP_134691043.1	DndB-ParB-nuclease	GCF_004523845.1	361	Bacteria>Proteobacteria>Gammaproteobacteria>Enterobacterales>Enterobacteriaceae	Enterobacter_hormaechei	DNA-sulfur-modification-protein-DndB-[Enterobacter-hormaechei]	158836
WP_150325899.1	DndB-ParB-nuclease	GCF_008692585.1	361	Bacteria>Proteobacteria>Gammaproteobacteria>Enterobacterales>Enterobacteriaceae	Enterobacter_hormaechei	DNA-sulfur-modification-protein-DndB-[Enterobacter-hormaechei]	158836
MBG0571757.1	DndB-ParB-nuclease	GCA_015704035.1	704	Bacteria>Proteobacteria>Gammaproteobacteria>Enterobacterales>Enterobacteriaceae	Enterobacter_hormaechei	hypothetical-protein-[Enterobacter-hormaechei]	158836
WP_047637790.1	DndB-ParB-nuclease	GCF_025311535.1	567	Bacteria>Proteobacteria>Gammaproteobacteria>Enterobacterales>Enterobacteriaceae	Enterobacter_hormaechei	hypothetical-protein-[Enterobacter-hormaechei]	158836
WP_063405466.1	DndB-ParB-nuclease	GCF_001631045.1	691	Bacteria>Proteobacteria>Gammaproteobacteria>Enterobacterales>Enterobacteriaceae	Enterobacter_hormaechei	hypothetical-protein-[Enterobacter-hormaechei]	158836
WP_196342898.1	DndB-ParB-nuclease	GCF_015704035.1	704	Bacteria>Proteobacteria>Gammaproteobacteria>Enterobacterales>Enterobacteriaceae	Enterobacter_hormaechei	hypothetical-protein-[Enterobacter-hormaechei]	158836
HAV1800527.1	DndB-ParB-nuclease	GCA_016431405.1	361	Bacteria>Proteobacteria>Gammaproteobacteria>Enterobacterales>Enterobacteriaceae	Enterobacter_hormaechei_subsp._steigerwaltii	DNA-sulfur-modification-protein-DndB-[Enterobacter-hormaechei-subsp.-steigerwaltii]	299766
HAV1800522.1	DndB-ParB-nuclease	GCA_016431405.1	691	Bacteria>Proteobacteria>Gammaproteobacteria>Enterobacterales>Enterobacteriaceae	Enterobacter_hormaechei_subsp._steigerwaltii	hypothetical-protein-[Enterobacter-hormaechei-subsp.-steigerwaltii]	299766
MBB1199016.1	DndB-ParB-nuclease	GCA_014156375.1	361	Bacteria>Proteobacteria>Gammaproteobacteria>Enterobacterales>Enterobacteriaceae	Enterobacteriaceae_bacterium_89	DNA-sulfur-modification-protein-DndB-[Enterobacteriaceae-bacterium-89]	2489522
EGQ5273138.1	DndB-ParB-nuclease	GCA_015684355.1	361	Bacteria>Proteobacteria>Gammaproteobacteria>Enterobacterales>Enterobacteriaceae	Enterobacter_kobei	DNA-sulfur-modification-protein-DndB-[Enterobacter-kobei]	208224
WP_071922642.1	DndB-ParB-nuclease	GCF_023153565.1	361	Bacteria>Proteobacteria>Gammaproteobacteria>Enterobacterales>Enterobacteriaceae	Enterobacter_kobei	DNA-sulfur-modification-protein-DndB-[Enterobacter-kobei]	208224
WP_047356917.1	DndB-ParB-nuclease	GCF_001022805.1	568	Bacteria>Proteobacteria>Gammaproteobacteria>Enterobacterales>Enterobacteriaceae	Enterobacter_ludwigii	AIPR-family-protein-[Enterobacter-ludwigii]	299767
MBX9029826.1	DndB-ParB-nuclease	GCA_019731755.1	359	Bacteria>Proteobacteria>Gammaproteobacteria>Enterobacterales>Enterobacteriaceae	Enterobacter_ludwigii	DNA-sulfur-modification-protein-DndB-[Enterobacter-ludwigii]	299767
WP_047357008.1	DndB-ParB-nuclease	GCF_018257815.1	361	Bacteria>Proteobacteria>Gammaproteobacteria>Enterobacterales>Enterobacteriaceae	Enterobacter_ludwigii	DNA-sulfur-modification-protein-DndB-[Enterobacter-ludwigii]	299767
WP_073397080.1	DndB-ParB-nuclease	GCF_900142835.1	361	Bacteria>Proteobacteria>Gammaproteobacteria>Enterobacterales>Enterobacteriaceae	Enterobacter_ludwigii	DNA-sulfur-modification-protein-DndB-[Enterobacter-ludwigii]	299767
WP_221716681.1	DndB-ParB-nuclease	-	359	Bacteria>Proteobacteria>Gammaproteobacteria>Enterobacterales>Enterobacteriaceae	Enterobacter_ludwigii	DNA-sulfur-modification-protein-DndB-[Enterobacter-ludwigii]	299767
MBX9029831.1	DndB-ParB-nuclease	GCA_019731755.1	563	Bacteria>Proteobacteria>Gammaproteobacteria>Enterobacterales>Enterobacteriaceae	Enterobacter_ludwigii	hypothetical-protein-[Enterobacter-ludwigii]	299767
WP_221716688.1	DndB-ParB-nuclease	GCF_019731755.1	563	Bacteria>Proteobacteria>Gammaproteobacteria>Enterobacterales>Enterobacteriaceae	Enterobacter_ludwigii	hypothetical-protein-[Enterobacter-ludwigii]	299767
WP_029484947.1	DndB-ParB-nuclease	GCF_902165275.1	404	Bacteria>Proteobacteria>Gammaproteobacteria>Enterobacterales>Enterobacteriaceae	Enterobacter	MULTISPECIES:-DNA-phosphorothioation-associated-DGQHR-protein-1-[Enterobacter]	547
WP_029484945.1	DndB-ParB-nuclease	GCF_902165275.1	361	Bacteria>Proteobacteria>Gammaproteobacteria>Enterobacterales>Enterobacteriaceae	Enterobacter	MULTISPECIES:-DNA-sulfur-modification-protein-DndB-[Enterobacter]	547
WP_047364012.1	DndB-ParB-nuclease	GCF_009905155.1	361	Bacteria>Proteobacteria>Gammaproteobacteria>Enterobacterales>Enterobacteriaceae	Enterobacter	MULTISPECIES:-DNA-sulfur-modification-protein-DndB-[Enterobacter]	547
WP_193532441.1	DndB-ParB-nuclease	GCF_019394605.1	361	Bacteria>Proteobacteria>Gammaproteobacteria>Enterobacterales>Enterobacteriaceae	Enterobacter	MULTISPECIES:-DNA-sulfur-modification-protein-DndB-[Enterobacter]	547
WP_193360916.1	DndB-ParB-nuclease	GCF_015673405.1	361	Bacteria>Proteobacteria>Gammaproteobacteria>Enterobacterales>Enterobacteriaceae	Enterobacter	MULTISPECIES:-DNA-sulfur-modification-protein-DndB-[Enterobacter-cloacae-complex]	547
WP_126545555.1	DndB-ParB-nuclease	GCF_003964905.1	361	Bacteria>Proteobacteria>Gammaproteobacteria>Enterobacterales>Enterobacteriaceae	Enterobacter_quasimori	DNA-sulfur-modification-protein-DndB-[Enterobacter-quasimori]	2838947
WP_053073520.1	DndB-ParB-nuclease	GCF_001039365.2	397	Bacteria>Proteobacteria>Gammaproteobacteria>Enterobacterales>Enterobacteriaceae	Enterobacter_quasiroggenkampii	DGQHR-domain-containing-protein-[Enterobacter-quasiroggenkampii]	2497436
WP_126306591.1	DndB-ParB-nuclease	GCF_003965005.1	387	Bacteria>Proteobacteria>Gammaproteobacteria>Enterobacterales>Enterobacteriaceae	Enterobacter_roggenkampii	DGQHR-domain-containing-protein-[Enterobacter-roggenkampii]	1812935
WP_126306587.1	DndB-ParB-nuclease	GCF_003965005.1	361	Bacteria>Proteobacteria>Gammaproteobacteria>Enterobacterales>Enterobacteriaceae	Enterobacter_roggenkampii	DNA-sulfur-modification-protein-DndB-[Enterobacter-roggenkampii]	1812935
WP_128738889.1	DndB-ParB-nuclease	-	571	Bacteria>Proteobacteria>Gammaproteobacteria>Enterobacterales>Enterobacteriaceae	Enterobacter_soli	hypothetical-protein-[Enterobacter-soli]	885040
ABP59160.1	DndB-ParB-nuclease	GCA_000016325.1	361	Bacteria>Proteobacteria>Gammaproteobacteria>Enterobacterales>Enterobacteriaceae	Enterobacter_sp._638	conserved-hypothetical-protein-[Enterobacter-sp.-638]	399742
WP_187042200.1	DndB-ParB-nuclease	GCF_014298075.1	384	Bacteria>Proteobacteria>Gammaproteobacteria>Enterobacterales>Enterobacteriaceae	Enterobacter_sp._JUb54	DNA-phosphorothioation-associated-DGQHR-protein-1-[Enterobacter-sp.-JUb54]	2724468
WP_187042197.1	DndB-ParB-nuclease	GCF_014298075.1	361	Bacteria>Proteobacteria>Gammaproteobacteria>Enterobacterales>Enterobacteriaceae	Enterobacter_sp._JUb54	DNA-sulfur-modification-protein-DndB-[Enterobacter-sp.-JUb54]	2724468
WP_149121089.1	DndB-ParB-nuclease	GCF_008271405.2	361	Bacteria>Proteobacteria>Gammaproteobacteria>Enterobacterales>Enterobacteriaceae	Enterobacter_sp._LU1	DNA-sulfur-modification-protein-DndB-[Enterobacter-sp.-LU1]	1848517
WP_221551579.1	DndB-ParB-nuclease	-	359	Bacteria>Proteobacteria>Gammaproteobacteria>Enterobacterales>Enterobacteriaceae	Enterobacter_sp._RIT637	DNA-sulfur-modification-protein-DndB-[Enterobacter-sp.-RIT637]	2870470
WP_221551573.1	DndB-ParB-nuclease	GCF_019718755.1	561	Bacteria>Proteobacteria>Gammaproteobacteria>Enterobacterales>Enterobacteriaceae	Enterobacter_sp._RIT637	hypothetical-protein-[Enterobacter-sp.-RIT637]	2870470
WP_072570392.1	DndB-ParB-nuclease	GCF_001888805.2	361	Bacteria>Proteobacteria>Gammaproteobacteria>Enterobacterales>Enterobacteriaceae	Enterobacter_sp._SA187	DNA-sulfur-modification-protein-DndB-[Enterobacter-sp.-SA187]	1914861
WP_072570394.1	DndB-ParB-nuclease	GCF_001888805.2	705	Bacteria>Proteobacteria>Gammaproteobacteria>Enterobacterales>Enterobacteriaceae	Enterobacter_sp._SA187	hypothetical-protein-[Enterobacter-sp.-SA187]	1914861
MBB3305412.1	DndB-ParB-nuclease	GCA_014193275.1	361	Bacteria>Proteobacteria>Gammaproteobacteria>Enterobacterales>Enterobacteriaceae	Enterobacter_sp._Sphag1F	DNA-sulfur-modification-protein-DndB-[Enterobacter-sp.-Sphag1F]	2587033
WP_161983474.1	DndB-ParB-nuclease	GCF_009932275.1	361	Bacteria>Proteobacteria>Gammaproteobacteria>Enterobacterales>Enterobacteriaceae	Escherichia_albertii	DNA-sulfur-modification-protein-DndB-[Escherichia-albertii]	208962
KDT12640.1	DndB-ParB-nuclease	GCA_000700605.1	331	Bacteria>Proteobacteria>Gammaproteobacteria>Enterobacterales>Enterobacteriaceae	Escherichia_coli_2-052-05_S3_C1	DGQHR-domain-protein-[Escherichia-coli-2-052-05_S3_C1]	1444119
KEL94152.1	DndB-ParB-nuclease	GCA_000714335.1	383	Bacteria>Proteobacteria>Gammaproteobacteria>Enterobacterales>Enterobacteriaceae	Escherichia_coli_5-366-08_S3_C1	DGQHR-domain-protein-[Escherichia-coli-5-366-08_S3_C1]	1444129
KEL94156.1	DndB-ParB-nuclease	GCA_000714335.1	331	Bacteria>Proteobacteria>Gammaproteobacteria>Enterobacterales>Enterobacteriaceae	Escherichia_coli_5-366-08_S3_C1	DGQHR-domain-protein-[Escherichia-coli-5-366-08_S3_C1]	1444129
CAV01834.1	DndB-ParB-nuclease	GCA_000026245.1	101	Bacteria>Proteobacteria>Gammaproteobacteria>Enterobacterales>Enterobacteriaceae	Escherichia_coli_55989	conserved-hypothetical-protein-[Escherichia-coli-55989]	585055
CAV01836.1	DndB-ParB-nuclease	GCA_000026245.1	197	Bacteria>Proteobacteria>Gammaproteobacteria>Enterobacterales>Enterobacteriaceae	Escherichia_coli_55989	conserved-hypothetical-protein-[Escherichia-coli-55989]	585055
CAV01843.1	DndB-ParB-nuclease	GCA_000026245.1	361	Bacteria>Proteobacteria>Gammaproteobacteria>Enterobacterales>Enterobacteriaceae	Escherichia_coli_55989	putative-DNA-thiolation-protein;-putative-phage-protein-[Escherichia-coli-55989]	585055

Accession	Annotation	GCA	Length	Phyletic.spread	Strain.name	Defline	Species.Taxid
EIE57773.1	DndB-ParB-nuclease	GCA_000259135.1	361	Bacteria>Proteobacteria>Gammaproteobacteria>Enterobacterales>Enterobacteriaceae	Escherichia_coli_AI27	hypothetical-protein-ECAI27_01190-[Escherichia-coli-AI27]	1089445
WP_001577775.1	DndB-ParB-nuclease	GCF_005384545.1	569	Bacteria>Proteobacteria>Gammaproteobacteria>Enterobacterales>Enterobacteriaceae	Escherichia_coli	AIPR-family-protein-[Escherichia-coli]	562
WP_231837400.1	DndB-ParB-nuclease	GCF_021167155.1	569	Bacteria>Proteobacteria>Gammaproteobacteria>Enterobacterales>Enterobacteriaceae	Escherichia_coli	AIPR-family-protein-[Escherichia-coli]	562
OSK45020.1	DndB-ParB-nuclease	GCA_002109655.1	361	Bacteria>Proteobacteria>Gammaproteobacteria>Enterobacterales>Enterobacteriaceae	Escherichia_coli_B671	DNA-sulfur-modification-protein-DndB-[Escherichia-coli-B671]	550680
EEU2439518.1	DndB-ParB-nuclease	GCA_011970185.1	383	Bacteria>Proteobacteria>Gammaproteobacteria>Enterobacterales>Enterobacteriaceae	Escherichia_coli	DGQHR-domain-containing-protein-[Escherichia-coli]	562
EEZ6063365.1	DndB-ParB-nuclease	GCA_012217305.1	268	Bacteria>Proteobacteria>Gammaproteobacteria>Enterobacterales>Enterobacteriaceae	Escherichia_coli	DGQHR-domain-containing-protein-[Escherichia-coli]	562
EFD0716369.1	DndB-ParB-nuclease	GCA_012377235.1	383	Bacteria>Proteobacteria>Gammaproteobacteria>Enterobacterales>Enterobacteriaceae	Escherichia_coli	DGQHR-domain-containing-protein-[Escherichia-coli]	562
EFE7699923.1	DndB-ParB-nuclease	GCA_012451745.1	268	Bacteria>Proteobacteria>Gammaproteobacteria>Enterobacterales>Enterobacteriaceae	Escherichia_coli	DGQHR-domain-containing-protein-[Escherichia-coli]	562
EFH3058976.1	DndB-ParB-nuclease	GCA_012594105.1	268	Bacteria>Proteobacteria>Gammaproteobacteria>Enterobacterales>Enterobacteriaceae	Escherichia_coli	DGQHR-domain-containing-protein-[Escherichia-coli]	562
EFM0245433.1	DndB-ParB-nuclease	GCA_012835375.1	268	Bacteria>Proteobacteria>Gammaproteobacteria>Enterobacterales>Enterobacteriaceae	Escherichia_coli	DGQHR-domain-containing-protein-[Escherichia-coli]	562
EFM9642119.1	DndB-ParB-nuclease	GCA_012907065.1	268	Bacteria>Proteobacteria>Gammaproteobacteria>Enterobacterales>Enterobacteriaceae	Escherichia_coli	DGQHR-domain-containing-protein-[Escherichia-coli]	562
EFN1999892.1	DndB-ParB-nuclease	GCA_012921435.1	383	Bacteria>Proteobacteria>Gammaproteobacteria>Enterobacterales>Enterobacteriaceae	Escherichia_coli	DGQHR-domain-containing-protein-[Escherichia-coli]	562
EFN4578230.1	DndB-ParB-nuclease	GCA_012990485.1	268	Bacteria>Proteobacteria>Gammaproteobacteria>Enterobacterales>Enterobacteriaceae	Escherichia_coli	DGQHR-domain-containing-protein-[Escherichia-coli]	562
EFO2898166.1	DndB-ParB-nuclease	GCA_013068925.1	268	Bacteria>Proteobacteria>Gammaproteobacteria>Enterobacterales>Enterobacteriaceae	Escherichia_coli	DGQHR-domain-containing-protein-[Escherichia-coli]	562
EFU7111978.1	DndB-ParB-nuclease	GCA_013766565.1	268	Bacteria>Proteobacteria>Gammaproteobacteria>Enterobacterales>Enterobacteriaceae	Escherichia_coli	DGQHR-domain-containing-protein-[Escherichia-coli]	562
EGM8253388.1	DndB-ParB-nuclease	GCA_015137895.1	268	Bacteria>Proteobacteria>Gammaproteobacteria>Enterobacterales>Enterobacteriaceae	Escherichia_coli	DGQHR-domain-containing-protein-[Escherichia-coli]	562
EHC4942880.1	DndB-ParB-nuclease	GCA_017057955.1	386	Bacteria>Proteobacteria>Gammaproteobacteria>Enterobacterales>Enterobacteriaceae	Escherichia_coli	DGQHR-domain-containing-protein-[Escherichia-coli]	562
EHM8362868.1	DndB-ParB-nuclease	GCA_018287815.1	386	Bacteria>Proteobacteria>Gammaproteobacteria>Enterobacterales>Enterobacteriaceae	Escherichia_coli	DGQHR-domain-containing-protein-[Escherichia-coli]	562
EHR8991537.1	DndB-ParB-nuclease	GCA_019081825.1	268	Bacteria>Proteobacteria>Gammaproteobacteria>Enterobacterales>Enterobacteriaceae	Escherichia_coli	DGQHR-domain-containing-protein-[Escherichia-coli]	562
EIL6045326.1	DndB-ParB-nuclease	GCA_020705295.1	383	Bacteria>Proteobacteria>Gammaproteobacteria>Enterobacterales>Enterobacteriaceae	Escherichia_coli	DGQHR-domain-containing-protein-[Escherichia-coli]	562
HAX5136920.1	DndB-ParB-nuclease	GCA_017018655.1	379	Bacteria>Proteobacteria>Gammaproteobacteria>Enterobacterales>Enterobacteriaceae	Escherichia_coli	DGQHR-domain-containing-protein-[Escherichia-coli]	562
HBA7721836.1	DndB-ParB-nuclease	GCA_017766625.1	382	Bacteria>Proteobacteria>Gammaproteobacteria>Enterobacterales>Enterobacteriaceae	Escherichia_coli	DGQHR-domain-containing-protein-[Escherichia-coli]	562
HBE4591071.1	DndB-ParB-nuclease	GCA_018561705.1	268	Bacteria>Proteobacteria>Gammaproteobacteria>Enterobacterales>Enterobacteriaceae	Escherichia_coli	DGQHR-domain-containing-protein-[Escherichia-coli]	562
MBB8975675.1	DndB-ParB-nuclease	GCA_012382255.2	383	Bacteria>Proteobacteria>Gammaproteobacteria>Enterobacterales>Enterobacteriaceae	Escherichia_coli	DGQHR-domain-containing-protein-[Escherichia-coli]	562
MBD3093068.1	DndB-ParB-nuclease	GCA_014712575.1	388	Bacteria>Proteobacteria>Gammaproteobacteria>Enterobacterales>Enterobacteriaceae	Escherichia_coli	DGQHR-domain-containing-protein-[Escherichia-coli]	562
MBF9078148.1	DndB-ParB-nuclease	GCA_015690245.1	268	Bacteria>Proteobacteria>Gammaproteobacteria>Enterobacterales>Enterobacteriaceae	Escherichia_coli	DGQHR-domain-containing-protein-[Escherichia-coli]	562
MBL7462792.1	DndB-ParB-nuclease	GCA_016786205.1	268	Bacteria>Proteobacteria>Gammaproteobacteria>Enterobacterales>Enterobacteriaceae	Escherichia_coli	DGQHR-domain-containing-protein-[Escherichia-coli]	562
MBZ8918110.1	DndB-ParB-nuclease	GCA_020146565.1	268	Bacteria>Proteobacteria>Gammaproteobacteria>Enterobacterales>Enterobacteriaceae	Escherichia_coli	DGQHR-domain-containing-protein-[Escherichia-coli]	562
MXI36793.1	DndB-ParB-nuclease	GCA_009824735.1	386	Bacteria>Proteobacteria>Gammaproteobacteria>Enterobacterales>Enterobacteriaceae	Escherichia_coli	DGQHR-domain-containing-protein-[Escherichia-coli]	562
QLX43079.1	DndB-ParB-nuclease	GCA_013786185.1	397	Bacteria>Proteobacteria>Gammaproteobacteria>Enterobacterales>Enterobacteriaceae	Escherichia_coli	DGQHR-domain-containing-protein-[Escherichia-coli]	562
WP_001586699.1	DndB-ParB-nuclease	GCF_005382905.1	383	Bacteria>Proteobacteria>Gammaproteobacteria>Enterobacterales>Enterobacteriaceae	Escherichia_coli	DGQHR-domain-containing-protein-[Escherichia-coli]	562
WP_047081506.1	DndB-ParB-nuclease	GCF_005394885.1	383	Bacteria>Proteobacteria>Gammaproteobacteria>Enterobacterales>Enterobacteriaceae	Escherichia_coli	DGQHR-domain-containing-protein-[Escherichia-coli]	562
WP_166488806.1	DndB-ParB-nuclease	GCF_025176285.1	268	Bacteria>Proteobacteria>Gammaproteobacteria>Enterobacterales>Enterobacteriaceae	Escherichia_coli	DGQHR-domain-containing-protein-[Escherichia-coli]	562
WP_170974616.1	DndB-ParB-nuclease	GCF_005381925.1	397	Bacteria>Proteobacteria>Gammaproteobacteria>Enterobacterales>Enterobacteriaceae	Escherichia_coli	DGQHR-domain-containing-protein-[Escherichia-coli]	562
WP_247150759.1	DndB-ParB-nuclease	GCF_022975715.1	251	Bacteria>Proteobacteria>Gammaproteobacteria>Enterobacterales>Enterobacteriaceae	Escherichia_coli	DGQHR-domain-containing-protein-[Escherichia-coli]	562
WP_250388767.1	DndB-ParB-nuclease	GCF_006237465.1	199	Bacteria>Proteobacteria>Gammaproteobacteria>Enterobacterales>Enterobacteriaceae	Escherichia_coli	DGQHR-domain-containing-protein,-partial-[Escherichia-coli]	562
WP_250388768.1	DndB-ParB-nuclease	GCF_006237465.1	151	Bacteria>Proteobacteria>Gammaproteobacteria>Enterobacterales>Enterobacteriaceae	Escherichia_coli	DGQHR-domain-containing-protein,-partial-[Escherichia-coli]	562
AVN10869.1	DndB-ParB-nuclease	GCA_002999075.1	361	Bacteria>Proteobacteria>Gammaproteobacteria>Enterobacterales>Enterobacteriaceae	Escherichia_coli	DGQHR-domain-protein-[Escherichia-coli]	562
WP_096848723.1	DndB-ParB-nuclease	GCF_022117655.1	386	Bacteria>Proteobacteria>Gammaproteobacteria>Enterobacterales>Enterobacteriaceae	Escherichia_coli	DNA-phosphorothioation-associated-DGQHR-protein-1-[Escherichia-coli]	562
WP_097178341.1	DndB-ParB-nuclease	GCF_002516295.1	386	Bacteria>Proteobacteria>Gammaproteobacteria>Enterobacterales>Enterobacteriaceae	Escherichia_coli	DNA-phosphorothioation-associated-DGQHR-protein-1-[Escherichia-coli]	562
WP_130075982.1	DndB-ParB-nuclease	GCF_019780765.1	386	Bacteria>Proteobacteria>Gammaproteobacteria>Enterobacterales>Enterobacteriaceae	Escherichia_coli	DNA-phosphorothioation-associated-DGQHR-protein-1-[Escherichia-coli]	562
AHM51228.1	DndB-ParB-nuclease	GCA_000599705.1	361	Bacteria>Proteobacteria>Gammaproteobacteria>Enterobacterales>Enterobacteriaceae	Escherichia_coli	DNA-sulfur-modification-protein-DndB-[Escherichia-coli]	562
EED0710388.1	DndB-ParB-nuclease	GCA_004183285.2	361	Bacteria>Proteobacteria>Gammaproteobacteria>Enterobacterales>Enterobacteriaceae	Escherichia_coli	DNA-sulfur-modification-protein-DndB-[Escherichia-coli]	562
EEQ4233400.1	DndB-ParB-nuclease	GCA_011816685.1	361	Bacteria>Proteobacteria>Gammaproteobacteria>Enterobacterales>Enterobacteriaceae	Escherichia_coli	DNA-sulfur-modification-protein-DndB-[Escherichia-coli]	562
EEQ7809104.1	DndB-ParB-nuclease	GCA_011830075.1	361	Bacteria>Proteobacteria>Gammaproteobacteria>Enterobacterales>Enterobacteriaceae	Escherichia_coli	DNA-sulfur-modification-protein-DndB-[Escherichia-coli]	562
EER2182200.1	DndB-ParB-nuclease	GCA_011848385.1	361	Bacteria>Proteobacteria>Gammaproteobacteria>Enterobacterales>Enterobacteriaceae	Escherichia_coli	DNA-sulfur-modification-protein-DndB-[Escherichia-coli]	562
EET0545152.1	DndB-ParB-nuclease	GCA_011921185.1	361	Bacteria>Proteobacteria>Gammaproteobacteria>Enterobacterales>Enterobacteriaceae	Escherichia_coli	DNA-sulfur-modification-protein-DndB-[Escherichia-coli]	562
EET4318490.1	DndB-ParB-nuclease	GCA_011936425.1	361	Bacteria>Proteobacteria>Gammaproteobacteria>Enterobacterales>Enterobacteriaceae	Escherichia_coli	DNA-sulfur-modification-protein-DndB-[Escherichia-coli]	562
EEU2439514.1	DndB-ParB-nuclease	GCA_011970185.1	361	Bacteria>Proteobacteria>Gammaproteobacteria>Enterobacterales>Enterobacteriaceae	Escherichia_coli	DNA-sulfur-modification-protein-DndB-[Escherichia-coli]	562
EEU5066666.1	DndB-ParB-nuclease	GCA_011980725.1	361	Bacteria>Proteobacteria>Gammaproteobacteria>Enterobacterales>Enterobacteriaceae	Escherichia_coli	DNA-sulfur-modification-protein-DndB-[Escherichia-coli]	562
EEV4636311.1	DndB-ParB-nuclease	GCA_012039475.1	361	Bacteria>Proteobacteria>Gammaproteobacteria>Enterobacterales>Enterobacteriaceae	Escherichia_coli	DNA-sulfur-modification-protein-DndB-[Escherichia-coli]	562
EEW0951710.1	DndB-ParB-nuclease	GCA_012068715.1	362	Bacteria>Proteobacteria>Gammaproteobacteria>Enterobacterales>Enterobacteriaceae	Escherichia_coli	DNA-sulfur-modification-protein-DndB-[Escherichia-coli]	562
EEY3223762.1	DndB-ParB-nuclease	GCA_012160245.1	361	Bacteria>Proteobacteria>Gammaproteobacteria>Enterobacterales>Enterobacteriaceae	Escherichia_coli	DNA-sulfur-modification-protein-DndB-[Escherichia-coli]	562
EEY4560438.1	DndB-ParB-nuclease	GCA_012166375.1	361	Bacteria>Proteobacteria>Gammaproteobacteria>Enterobacterales>Enterobacteriaceae	Escherichia_coli	DNA-sulfur-modification-protein-DndB-[Escherichia-coli]	562
EEZ2511096.1	DndB-ParB-nuclease	GCA_012193425.1	361	Bacteria>Proteobacteria>Gammaproteobacteria>Enterobacterales>Enterobacteriaceae	Escherichia_coli	DNA-sulfur-modification-protein-DndB-[Escherichia-coli]	562
EEZ3917719.1	DndB-ParB-nuclease	GCA_012204645.1	361	Bacteria>Proteobacteria>Gammaproteobacteria>Enterobacterales>Enterobacteriaceae	Escherichia_coli	DNA-sulfur-modification-protein-DndB-[Escherichia-coli]	562
EEZ6063369.1	DndB-ParB-nuclease	GCA_012217305.1	361	Bacteria>Proteobacteria>Gammaproteobacteria>Enterobacterales>Enterobacteriaceae	Escherichia_coli	DNA-sulfur-modification-protein-DndB-[Escherichia-coli]	562
EFA6126332.1	DndB-ParB-nuclease	GCA_012255245.1	362	Bacteria>Proteobacteria>Gammaproteobacteria>Enterobacterales>Enterobacteriaceae	Escherichia_coli	DNA-sulfur-modification-protein-DndB-[Escherichia-coli]	562
EFB2626163.1	DndB-ParB-nuclease	GCA_012293805.1	361	Bacteria>Proteobacteria>Gammaproteobacteria>Enterobacterales>Enterobacteriaceae	Escherichia_coli	DNA-sulfur-modification-protein-DndB-[Escherichia-coli]	562
EFB8549913.1	DndB-ParB-nuclease	GCA_012325445.1	361	Bacteria>Proteobacteria>Gammaproteobacteria>Enterobacterales>Enterobacteriaceae	Escherichia_coli	DNA-sulfur-modification-protein-DndB-[Escherichia-coli]	562
EFC5414227.1	DndB-ParB-nuclease	GCA_012351965.1	362	Bacteria>Proteobacteria>Gammaproteobacteria>Enterobacterales>Enterobacteriaceae	Escherichia_coli	DNA-sulfur-modification-protein-DndB-[Escherichia-coli]	562
EFD0716365.1	DndB-ParB-nuclease	GCA_012377235.1	361	Bacteria>Proteobacteria>Gammaproteobacteria>Enterobacterales>Enterobacteriaceae	Escherichia_coli	DNA-sulfur-modification-protein-DndB-[Escherichia-coli]	562
EFE7699336.1	DndB-ParB-nuclease	GCA_012451745.1	361	Bacteria>Proteobacteria>Gammaproteobacteria>Enterobacterales>Enterobacteriaceae	Escherichia_coli	DNA-sulfur-modification-protein-DndB-[Escherichia-coli]	562
EFF1421090.1	DndB-ParB-nuclease	GCA_012469615.1	361	Bacteria>Proteobacteria>Gammaproteobacteria>Enterobacterales>Enterobacteriaceae	Escherichia_coli	DNA-sulfur-modification-protein-DndB-[Escherichia-coli]	562
EFF9654130.1	DndB-ParB-nuclease	GCA_012528715.1	361	Bacteria>Proteobacteria>Gammaproteobacteria>Enterobacterales>Enterobacteriaceae	Escherichia_coli	DNA-sulfur-modification-protein-DndB-[Escherichia-coli]	562
EFG3337756.1	DndB-ParB-nuclease	GCA_012552645.1	362	Bacteria>Proteobacteria>Gammaproteobacteria>Enterobacterales>Enterobacteriaceae	Escherichia_coli	DNA-sulfur-modification-protein-DndB-[Escherichia-coli]	562
EFH3058980.1	DndB-ParB-nuclease	GCA_012594105.1	361	Bacteria>Proteobacteria>Gammaproteobacteria>Enterobacterales>Enterobacteriaceae	Escherichia_coli	DNA-sulfur-modification-protein-DndB-[Escherichia-coli]	562
EFH6148454.1	DndB-ParB-nuclease	GCA_012609335.1	361	Bacteria>Proteobacteria>Gammaproteobacteria>Enterobacterales>Enterobacteriaceae	Escherichia_coli	DNA-sulfur-modification-protein-DndB-[Escherichia-coli]	562
EFH6733470.1	DndB-ParB-nuclease	GCA_012611105.1	362	Bacteria>Proteobacteria>Gammaproteobacteria>Enterobacterales>Enterobacteriaceae	Escherichia_coli	DNA-sulfur-modification-protein-DndB-[Escherichia-coli]	562
EFH6854126.1	DndB-ParB-nuclease	GCA_012612425.1	362	Bacteria>Proteobacteria>Gammaproteobacteria>Enterobacterales>Enterobacteriaceae	Escherichia_coli	DNA-sulfur-modification-protein-DndB-[Escherichia-coli]	562
EFH7254308.1	DndB-ParB-nuclease	GCA_012614145.1	361	Bacteria>Proteobacteria>Gammaproteobacteria>Enterobacterales>Enterobacteriaceae	Escherichia_coli	DNA-sulfur-modification-protein-DndB-[Escherichia-coli]	562
EFH8514627.1	DndB-ParB-nuclease	GCA_012620765.1	361	Bacteria>Proteobacteria>Gammaproteobacteria>Enterobacterales>Enterobacteriaceae	Escherichia_coli	DNA-sulfur-modification-protein-DndB-[Escherichia-coli]	562
EFJ3337513.1	DndB-ParB-nuclease	GCA_012701065.1	361	Bacteria>Proteobacteria>Gammaproteobacteria>Enterobacterales>Enterobacteriaceae	Escherichia_coli	DNA-sulfur-modification-protein-DndB-[Escherichia-coli]	562
EFJ3713177.1	DndB-ParB-nuclease	GCA_012702515.1	361	Bacteria>Proteobacteria>Gammaproteobacteria>Enterobacterales>Enterobacteriaceae	Escherichia_coli	DNA-sulfur-modification-protein-DndB-[Escherichia-coli]	562
EFJ9505779.1	DndB-ParB-nuclease	GCA_012733695.1	361	Bacteria>Proteobacteria>Gammaproteobacteria>Enterobacterales>Enterobacteriaceae	Escherichia_coli	DNA-sulfur-modification-protein-DndB-[Escherichia-coli]	562
EFK7843402.1	DndB-ParB-nuclease	GCA_012773895.1	361	Bacteria>Proteobacteria>Gammaproteobacteria>Enterobacterales>Enterobacteriaceae	Escherichia_coli	DNA-sulfur-modification-protein-DndB-[Escherichia-coli]	562
EFL6831661.1	DndB-ParB-nuclease	GCA_012821675.1	361	Bacteria>Proteobacteria>Gammaproteobacteria>Enterobacterales>Enterobacteriaceae	Escherichia_coli	DNA-sulfur-modification-protein-DndB-[Escherichia-coli]	562
EFM9642115.1	DndB-ParB-nuclease	GCA_012907065.1	361	Bacteria>Proteobacteria>Gammaproteobacteria>Enterobacterales>Enterobacteriaceae	Escherichia_coli	DNA-sulfur-modification-protein-DndB-[Escherichia-coli]	562
EFN1999888.1	DndB-ParB-nuclease	GCA_012921435.1	361	Bacteria>Proteobacteria>Gammaproteobacteria>Enterobacterales>Enterobacteriaceae	Escherichia_coli	DNA-sulfur-modification-protein-DndB-[Escherichia-coli]	562
EFN3294510.1	DndB-ParB-nuclease	GCA_012969825.1	361	Bacteria>Proteobacteria>Gammaproteobacteria>Enterobacterales>Enterobacteriaceae	Escherichia_coli	DNA-sulfur-modification-protein-DndB-[Escherichia-coli]	562
EFN4578226.1	DndB-ParB-nuclease	GCA_012990485.1	361	Bacteria>Proteobacteria>Gammaproteobacteria>Enterobacterales>Enterobacteriaceae	Escherichia_coli	DNA-sulfur-modification-protein-DndB-[Escherichia-coli]	562
EFN8326890.1	DndB-ParB-nuclease	GCA_013047425.1	361	Bacteria>Proteobacteria>Gammaproteobacteria>Enterobacterales>Enterobacteriaceae	Escherichia_coli	DNA-sulfur-modification-protein-DndB-[Escherichia-coli]	562
EFN8912354.1	DndB-ParB-nuclease	GCA_013051385.1	362	Bacteria>Proteobacteria>Gammaproteobacteria>Enterobacterales>Enterobacteriaceae	Escherichia_coli	DNA-sulfur-modification-protein-DndB-[Escherichia-coli]	562
EFO1561144.1	DndB-ParB-nuclease	GCA_013063765.1	361	Bacteria>Proteobacteria>Gammaproteobacteria>Enterobacterales>Enterobacteriaceae	Escherichia_coli	DNA-sulfur-modification-protein-DndB-[Escherichia-coli]	562
EFO2280409.1	DndB-ParB-nuclease	GCA_013066625.1	361	Bacteria>Proteobacteria>Gammaproteobacteria>Enterobacterales>Enterobacteriaceae	Escherichia_coli	DNA-sulfur-modification-protein-DndB-[Escherichia-coli]	562
EFO2898170.1	DndB-ParB-nuclease	GCA_013068925.1	361	Bacteria>Proteobacteria>Gammaproteobacteria>Enterobacterales>Enterobacteriaceae	Escherichia_coli	DNA-sulfur-modification-protein-DndB-[Escherichia-coli]	562
EFO3044695.1	DndB-ParB-nuclease	GCA_013069605.1	361	Bacteria>Proteobacteria>Gammaproteobacteria>Enterobacterales>Enterobacteriaceae	Escherichia_coli	DNA-sulfur-modification-protein-DndB-[Escherichia-coli]	562
EFU7111982.1	DndB-ParB-nuclease	GCA_013766565.1	361	Bacteria>Proteobacteria>Gammaproteobacteria>Enterobacterales>Enterobacteriaceae	Escherichia_coli	DNA-sulfur-modification-protein-DndB-[Escherichia-coli]	562
EGC4097947.1	DndB-ParB-nuclease	GCA_014279245.1	361	Bacteria>Proteobacteria>Gammaproteobacteria>Enterobacterales>Enterobacteriaceae	Escherichia_coli	DNA-sulfur-modification-protein-DndB-[Escherichia-coli]	562
EGF1582132.1	DndB-ParB-nuclease	GCA_014473095.1	360	Bacteria>Proteobacteria>Gammaproteobacteria>Enterobacterales>Enterobacteriaceae	Escherichia_coli	DNA-sulfur-modification-protein-DndB-[Escherichia-coli]	562
EGF7320901.1	DndB-ParB-nuclease	GCA_014521635.1	361	Bacteria>Proteobacteria>Gammaproteobacteria>Enterobacterales>Enterobacteriaceae	Escherichia_coli	DNA-sulfur-modification-protein-DndB-[Escherichia-coli]	562
EGG1085611.1	DndB-ParB-nuclease	GCA_014546365.1	361	Bacteria>Proteobacteria>Gammaproteobacteria>Enterobacterales>Enterobacteriaceae	Escherichia_coli	DNA-sulfur-modification-protein-DndB-[Escherichia-coli]	562
EGJ2787771.1	DndB-ParB-nuclease	GCA_014814745.1	361	Bacteria>Proteobacteria>Gammaproteobacteria>Enterobacterales>Enterobacteriaceae	Escherichia_coli	DNA-sulfur-modification-protein-DndB-[Escherichia-coli]	562
EGK4222279.1	DndB-ParB-nuclease	GCA_014986865.1	361	Bacteria>Proteobacteria>Gammaproteobacteria>Enterobacterales>Enterobacteriaceae	Escherichia_coli	DNA-sulfur-modification-protein-DndB-[Escherichia-coli]	562
EGM8253392.1	DndB-ParB-nuclease	GCA_015137895.1	361	Bacteria>Proteobacteria>Gammaproteobacteria>Enterobacterales>Enterobacteriaceae	Escherichia_coli	DNA-sulfur-modification-protein-DndB-[Escherichia-coli]	562
EHC4289927.1	DndB-ParB-nuclease	GCA_017050055.1	361	Bacteria>Proteobacteria>Gammaproteobacteria>Enterobacterales>Enterobacteriaceae	Escherichia_coli	DNA-sulfur-modification-protein-DndB-[Escherichia-coli]	562
EHC4942884.1	DndB-ParB-nuclease	GCA_017057955.1	361	Bacteria>Proteobacteria>Gammaproteobacteria>Enterobacterales>Enterobacteriaceae	Escherichia_coli	DNA-sulfur-modification-protein-DndB-[Escherichia-coli]	562
EHD1139962.1	DndB-ParB-nuclease	GCA_017120305.1	362	Bacteria>Proteobacteria>Gammaproteobacteria>Enterobacterales>Enterobacteriaceae	Escherichia_coli	DNA-sulfur-modification-protein-DndB-[Escherichia-coli]	562
EHH6655538.1	DndB-ParB-nuclease	GCA_017720975.1	361	Bacteria>Proteobacteria>Gammaproteobacteria>Enterobacterales>Enterobacteriaceae	Escherichia_coli	DNA-sulfur-modification-protein-DndB-[Escherichia-coli]	562
EHM8362872.1	DndB-ParB-nuclease	GCA_018287815.1	361	Bacteria>Proteobacteria>Gammaproteobacteria>Enterobacterales>Enterobacteriaceae	Escherichia_coli	DNA-sulfur-modification-protein-DndB-[Escherichia-coli]	562
EHP1685519.1	DndB-ParB-nuclease	GCA_018725885.1	361	Bacteria>Proteobacteria>Gammaproteobacteria>Enterobacterales>Enterobacteriaceae	Escherichia_coli	DNA-sulfur-modification-protein-DndB-[Escherichia-coli]	562
EHP5157376.1	DndB-ParB-nuclease	GCA_018798725.1	361	Bacteria>Proteobacteria>Gammaproteobacteria>Enterobacterales>Enterobacteriaceae	Escherichia_coli	DNA-sulfur-modification-protein-DndB-[Escherichia-coli]	562
EHR1198353.1	DndB-ParB-nuclease	GCA_019000365.1	361	Bacteria>Proteobacteria>Gammaproteobacteria>Enterobacterales>Enterobacteriaceae	Escherichia_coli	DNA-sulfur-modification-protein-DndB-[Escherichia-coli]	562
EHR8991533.1	DndB-ParB-nuclease	GCA_019081825.1	361	Bacteria>Proteobacteria>Gammaproteobacteria>Enterobacterales>Enterobacteriaceae	Escherichia_coli	DNA-sulfur-modification-protein-DndB-[Escherichia-coli]	562
EHT7510521.1	DndB-ParB-nuclease	GCA_019253325.1	361	Bacteria>Proteobacteria>Gammaproteobacteria>Enterobacterales>Enterobacteriaceae	Escherichia_coli	DNA-sulfur-modification-protein-DndB-[Escherichia-coli]	562
EHU6114352.1	DndB-ParB-nuclease	GCA_019300275.1	361	Bacteria>Proteobacteria>Gammaproteobacteria>Enterobacterales>Enterobacteriaceae	Escherichia_coli	DNA-sulfur-modification-protein-DndB-[Escherichia-coli]	562
EHY3484739.1	DndB-ParB-nuclease	GCA_019575735.1	361	Bacteria>Proteobacteria>Gammaproteobacteria>Enterobacterales>Enterobacteriaceae	Escherichia_coli	DNA-sulfur-modification-protein-DndB-[Escherichia-coli]	562
EIQ0103648.1	DndB-ParB-nuclease	GCA_021141945.1	361	Bacteria>Proteobacteria>Gammaproteobacteria>Enterobacterales>Enterobacteriaceae	Escherichia_coli	DNA-sulfur-modification-protein-DndB-[Escherichia-coli]	562
HAN8754171.1	DndB-ParB-nuclease	GCA_014748905.1	309	Bacteria>Proteobacteria>Gammaproteobacteria>Enterobacterales>Enterobacteriaceae	Escherichia_coli	DNA-sulfur-modification-protein-DndB-[Escherichia-coli]	562
MBB8975671.1	DndB-ParB-nuclease	GCA_012382255.2	361	Bacteria>Proteobacteria>Gammaproteobacteria>Enterobacterales>Enterobacteriaceae	Escherichia_coli	DNA-sulfur-modification-protein-DndB-[Escherichia-coli]	562
MBC1014233.1	DndB-ParB-nuclease	GCA_012354025.2	361	Bacteria>Proteobacteria>Gammaproteobacteria>Enterobacterales>Enterobacteriaceae	Escherichia_coli	DNA-sulfur-modification-protein-DndB-[Escherichia-coli]	562
MBD3093072.1	DndB-ParB-nuclease	GCA_014712575.1	361	Bacteria>Proteobacteria>Gammaproteobacteria>Enterobacterales>Enterobacteriaceae	Escherichia_coli	DNA-sulfur-modification-protein-DndB-[Escherichia-coli]	562
MBF9078152.1	DndB-ParB-nuclease	GCA_015690245.1	361	Bacteria>Proteobacteria>Gammaproteobacteria>Enterobacterales>Enterobacteriaceae	Escherichia_coli	DNA-sulfur-modification-protein-DndB-[Escherichia-coli]	562
MBL7462788.1	DndB-ParB-nuclease	GCA_016786205.1	361	Bacteria>Proteobacteria>Gammaproteobacteria>Enterobacterales>Enterobacteriaceae	Escherichia_coli	DNA-sulfur-modification-protein-DndB-[Escherichia-coli]	562
MBN6377949.1	DndB-ParB-nuclease	GCA_017163475.1	361	Bacteria>Proteobacteria>Gammaproteobacteria>Enterobacterales>Enterobacteriaceae	Escherichia_coli	DNA-sulfur-modification-protein-DndB-[Escherichia-coli]	562
MBZ8918114.1	DndB-ParB-nuclease	GCA_020146565.1	361	Bacteria>Proteobacteria>Gammaproteobacteria>Enterobacterales>Enterobacteriaceae	Escherichia_coli	DNA-sulfur-modification-protein-DndB-[Escherichia-coli]	562
MWL30110.1	DndB-ParB-nuclease	GCA_009793945.1	361	Bacteria>Proteobacteria>Gammaproteobacteria>Enterobacterales>Enterobacteriaceae	Escherichia_coli	DNA-sulfur-modification-protein-DndB-[Escherichia-coli]	562
MWO98707.1	DndB-ParB-nuclease	GCA_009794705.1	361	Bacteria>Proteobacteria>Gammaproteobacteria>Enterobacterales>Enterobacteriaceae	Escherichia_coli	DNA-sulfur-modification-protein-DndB-[Escherichia-coli]	562
MWR94394.1	DndB-ParB-nuclease	GCA_009798605.1	361	Bacteria>Proteobacteria>Gammaproteobacteria>Enterobacterales>Enterobacteriaceae	Escherichia_coli	DNA-sulfur-modification-protein-DndB-[Escherichia-coli]	562
MXI36797.1	DndB-ParB-nuclease	GCA_009824735.1	361	Bacteria>Proteobacteria>Gammaproteobacteria>Enterobacterales>Enterobacteriaceae	Escherichia_coli	DNA-sulfur-modification-protein-DndB-[Escherichia-coli]	562
NIZ85215.1	DndB-ParB-nuclease	GCA_011812915.1	362	Bacteria>Proteobacteria>Gammaproteobacteria>Enterobacterales>Enterobacteriaceae	Escherichia_coli	DNA-sulfur-modification-protein-DndB-[Escherichia-coli]	562
NJA94335.1	DndB-ParB-nuclease	GCA_011881645.1	361	Bacteria>Proteobacteria>Gammaproteobacteria>Enterobacterales>Enterobacteriaceae	Escherichia_coli	DNA-sulfur-modification-protein-DndB-[Escherichia-coli]	562
QLX43083.1	DndB-ParB-nuclease	GCA_013786185.1	361	Bacteria>Proteobacteria>Gammaproteobacteria>Enterobacterales>Enterobacteriaceae	Escherichia_coli	DNA-sulfur-modification-protein-DndB-[Escherichia-coli]	562
QMH37045.1	DndB-ParB-nuclease	GCA_013833345.1	361	Bacteria>Proteobacteria>Gammaproteobacteria>Enterobacterales>Enterobacteriaceae	Escherichia_coli	DNA-sulfur-modification-protein-DndB-[Escherichia-coli]	562
QXA60628.1	DndB-ParB-nuclease	GCA_019047905.1	361	Bacteria>Proteobacteria>Gammaproteobacteria>Enterobacterales>Enterobacteriaceae	Escherichia_coli	DNA-sulfur-modification-protein-DndB-[Escherichia-coli]	562

Accession	Annotation	GCA	Length	Phyletic.spread	Strain.name	Defline	Species.Taxid
WP_000146439.1	DndB-ParB-nuclease	GCF_005383185.1	361	Bacteria>Proteobacteria>Gammaproteobacteria>Enterobacterales>Enterobacteriaceae	Escherichia_coli	DNA-sulfur-modification-protein-DndB-[Escherichia-coli]	562
WP_001577770.1	DndB-ParB-nuclease	GCF_005382905.1	361	Bacteria>Proteobacteria>Gammaproteobacteria>Enterobacterales>Enterobacteriaceae	Escherichia_coli	DNA-sulfur-modification-protein-DndB-[Escherichia-coli]	562
WP_001611274.1	DndB-ParB-nuclease	GCF_000358435.2	361	Bacteria>Proteobacteria>Gammaproteobacteria>Enterobacterales>Enterobacteriaceae	Escherichia_coli	DNA-sulfur-modification-protein-DndB-[Escherichia-coli]	562
WP_047081508.1	DndB-ParB-nuclease	GCF_005383985.1	361	Bacteria>Proteobacteria>Gammaproteobacteria>Enterobacterales>Enterobacteriaceae	Escherichia_coli	DNA-sulfur-modification-protein-DndB-[Escherichia-coli]	562
WP_097178343.1	DndB-ParB-nuclease	GCF_002516295.1	361	Bacteria>Proteobacteria>Gammaproteobacteria>Enterobacterales>Enterobacteriaceae	Escherichia_coli	DNA-sulfur-modification-protein-DndB-[Escherichia-coli]	562
WP_097746403.1	DndB-ParB-nuclease	GCF_002546565.1	362	Bacteria>Proteobacteria>Gammaproteobacteria>Enterobacterales>Enterobacteriaceae	Escherichia_coli	DNA-sulfur-modification-protein-DndB-[Escherichia-coli]	562
WP_112014321.1	DndB-ParB-nuclease	GCF_005381585.1	362	Bacteria>Proteobacteria>Gammaproteobacteria>Enterobacterales>Enterobacteriaceae	Escherichia_coli	DNA-sulfur-modification-protein-DndB-[Escherichia-coli]	562
WP_191028152.1	DndB-ParB-nuclease	GCF_014712575.1	361	Bacteria>Proteobacteria>Gammaproteobacteria>Enterobacterales>Enterobacteriaceae	Escherichia_coli	DNA-sulfur-modification-protein-DndB-[Escherichia-coli]	562
WP_213789732.1	DndB-ParB-nuclease	GCF_910594925.1	360	Bacteria>Proteobacteria>Gammaproteobacteria>Enterobacterales>Enterobacteriaceae	Escherichia_coli	DNA-sulfur-modification-protein-DndB-[Escherichia-coli]	562
WP_215263033.1	DndB-ParB-nuclease	GCF_018683545.1	361	Bacteria>Proteobacteria>Gammaproteobacteria>Enterobacterales>Enterobacteriaceae	Escherichia_coli	DNA-sulfur-modification-protein-DndB-[Escherichia-coli]	562
WP_218069839.1	DndB-ParB-nuclease	GCF_910592835.1	362	Bacteria>Proteobacteria>Gammaproteobacteria>Enterobacterales>Enterobacteriaceae	Escherichia_coli	DNA-sulfur-modification-protein-DndB-[Escherichia-coli]	562
WP_223942765.1	DndB-ParB-nuclease	GCF_910907495.1	361	Bacteria>Proteobacteria>Gammaproteobacteria>Enterobacterales>Enterobacteriaceae	Escherichia_coli	DNA-sulfur-modification-protein-DndB-[Escherichia-coli]	562
STH12459.1	DndB-ParB-nuclease	GCA_900449075.1	256	Bacteria>Proteobacteria>Gammaproteobacteria>Enterobacterales>Enterobacteriaceae	Escherichia_coli	DNA-thiolation-protein;-phage-protein-[Escherichia-coli]	562
STH12460.1	DndB-ParB-nuclease	GCA_900449075.1	112	Bacteria>Proteobacteria>Gammaproteobacteria>Enterobacterales>Enterobacteriaceae	Escherichia_coli	DNA-thiolation-protein;-phage-protein-[Escherichia-coli]	562
EKH25624.1	DndB-ParB-nuclease	GCA_000303715.2	331	Bacteria>Proteobacteria>Gammaproteobacteria>Enterobacterales>Enterobacteriaceae	Escherichia_coli_FDA506	DNA-sulfur-modification-protein-DndB-[Escherichia-coli-FDA506]	1005474
EKH25620.1	DndB-ParB-nuclease	GCA_000303715.2	197	Bacteria>Proteobacteria>Gammaproteobacteria>Enterobacterales>Enterobacteriaceae	Escherichia_coli_FDA506	hypothetical-protein-ECFDA506_0290-[Escherichia-coli-FDA506]	1005474
CAF2529509.1	DndB-ParB-nuclease	GCA_905330295.1	361	Bacteria>Proteobacteria>Gammaproteobacteria>Enterobacterales>Enterobacteriaceae	Escherichia_coli	hypothetical-protein-AI2860V1_0766-[Escherichia-coli]	562
CAF2529544.1	DndB-ParB-nuclease	GCA_905330295.1	101	Bacteria>Proteobacteria>Gammaproteobacteria>Enterobacterales>Enterobacteriaceae	Escherichia_coli	hypothetical-protein-AI2860V1_0770-[Escherichia-coli]	562
EFM9642120.1	DndB-ParB-nuclease	GCA_012907065.1	101	Bacteria>Proteobacteria>Gammaproteobacteria>Enterobacterales>Enterobacteriaceae	Escherichia_coli	hypothetical-protein-BB069_000599-[Escherichia-coli]	562
EFO2898165.1	DndB-ParB-nuclease	GCA_013068925.1	101	Bacteria>Proteobacteria>Gammaproteobacteria>Enterobacterales>Enterobacteriaceae	Escherichia_coli	hypothetical-protein-CJC78_16995-[Escherichia-coli]	562
AVN11498.1	DndB-ParB-nuclease	GCA_002999075.1	197	Bacteria>Proteobacteria>Gammaproteobacteria>Enterobacterales>Enterobacteriaceae	Escherichia_coli	hypothetical-protein-CSC11_2367-[Escherichia-coli]	562
EEZ6063364.1	DndB-ParB-nuclease	GCA_012217305.1	101	Bacteria>Proteobacteria>Gammaproteobacteria>Enterobacterales>Enterobacteriaceae	Escherichia_coli	hypothetical-protein-DA948_001986-[Escherichia-coli]	562
EFM0245434.1	DndB-ParB-nuclease	GCA_012835375.1	101	Bacteria>Proteobacteria>Gammaproteobacteria>Enterobacterales>Enterobacteriaceae	Escherichia_coli	hypothetical-protein-DD11_004273-[Escherichia-coli]	562
EFO3044699.1	DndB-ParB-nuclease	GCA_013069605.1	197	Bacteria>Proteobacteria>Gammaproteobacteria>Enterobacterales>Enterobacteriaceae	Escherichia_coli	hypothetical-protein-DQ141_25080-[Escherichia-coli]	562
EGF1582127.1	DndB-ParB-nuclease	GCA_014473095.1	696	Bacteria>Proteobacteria>Gammaproteobacteria>Enterobacterales>Enterobacteriaceae	Escherichia_coli	hypothetical-protein-[Escherichia-coli]	562
EGK4222275.1	DndB-ParB-nuclease	GCA_014986865.1	197	Bacteria>Proteobacteria>Gammaproteobacteria>Enterobacterales>Enterobacteriaceae	Escherichia_coli	hypothetical-protein-[Escherichia-coli]	562
MBC1014229.1	DndB-ParB-nuclease	GCA_012354025.2	206	Bacteria>Proteobacteria>Gammaproteobacteria>Enterobacterales>Enterobacteriaceae	Escherichia_coli	hypothetical-protein-[Escherichia-coli]	562
WP_089069124.1	DndB-ParB-nuclease	GCF_021869725.1	105	Bacteria>Proteobacteria>Gammaproteobacteria>Enterobacterales>Enterobacteriaceae	Escherichia_coli	hypothetical-protein-[Escherichia-coli]	562
WP_121856731.1	DndB-ParB-nuclease	GCF_003686745.1	197	Bacteria>Proteobacteria>Gammaproteobacteria>Enterobacterales>Enterobacteriaceae	Escherichia_coli	hypothetical-protein-[Escherichia-coli]	562
WP_218075481.1	DndB-ParB-nuclease	GCF_910594925.1	696	Bacteria>Proteobacteria>Gammaproteobacteria>Enterobacterales>Enterobacteriaceae	Escherichia_coli	hypothetical-protein-[Escherichia-coli]	562
EFE7699922.1	DndB-ParB-nuclease	GCA_012451745.1	101	Bacteria>Proteobacteria>Gammaproteobacteria>Enterobacterales>Enterobacteriaceae	Escherichia_coli	hypothetical-protein-F7M98_27195-[Escherichia-coli]	562
EFN4578231.1	DndB-ParB-nuclease	GCA_012990485.1	101	Bacteria>Proteobacteria>Gammaproteobacteria>Enterobacterales>Enterobacteriaceae	Escherichia_coli	hypothetical-protein-FQG33_15395-[Escherichia-coli]	562
EFH3058975.1	DndB-ParB-nuclease	GCA_012594105.1	101	Bacteria>Proteobacteria>Gammaproteobacteria>Enterobacterales>Enterobacteriaceae	Escherichia_coli	hypothetical-protein-GUU67_22700-[Escherichia-coli]	562
MBZ8918109.1	DndB-ParB-nuclease	GCA_020146565.1	101	Bacteria>Proteobacteria>Gammaproteobacteria>Enterobacterales>Enterobacteriaceae	Escherichia_coli	hypothetical-protein-H5T00_11255-[Escherichia-coli]	562
EFU7111977.1	DndB-ParB-nuclease	GCA_013766565.1	101	Bacteria>Proteobacteria>Gammaproteobacteria>Enterobacterales>Enterobacteriaceae	Escherichia_coli	hypothetical-protein-HTZ45_002329-[Escherichia-coli]	562
MBF9078147.1	DndB-ParB-nuclease	GCA_015690245.1	101	Bacteria>Proteobacteria>Gammaproteobacteria>Enterobacterales>Enterobacteriaceae	Escherichia_coli	hypothetical-protein-I0P45_22120-[Escherichia-coli]	562
QXA60621.1	DndB-ParB-nuclease	GCA_019047905.1	101	Bacteria>Proteobacteria>Gammaproteobacteria>Enterobacterales>Enterobacteriaceae	Escherichia_coli	hypothetical-protein-I6L18_15175-[Escherichia-coli]	562
EGM8253387.1	DndB-ParB-nuclease	GCA_015137895.1	101	Bacteria>Proteobacteria>Gammaproteobacteria>Enterobacterales>Enterobacteriaceae	Escherichia_coli	hypothetical-protein-IIN64_004006-[Escherichia-coli]	562
MBL7462793.1	DndB-ParB-nuclease	GCA_016786205.1	101	Bacteria>Proteobacteria>Gammaproteobacteria>Enterobacterales>Enterobacteriaceae	Escherichia_coli	hypothetical-protein-INQ08_05330-[Escherichia-coli]	562
EHC4289923.1	DndB-ParB-nuclease	GCA_017050055.1	197	Bacteria>Proteobacteria>Gammaproteobacteria>Enterobacterales>Enterobacteriaceae	Escherichia_coli	hypothetical-protein-JTG83_004310-[Escherichia-coli]	562
EHR8991538.1	DndB-ParB-nuclease	GCA_019081825.1	101	Bacteria>Proteobacteria>Gammaproteobacteria>Enterobacterales>Enterobacteriaceae	Escherichia_coli	hypothetical-protein-KUR90_003665-[Escherichia-coli]	562
WP_137551502.1	DndB-ParB-nuclease	GCF_005396125.1	184	Bacteria>Proteobacteria>Gammaproteobacteria>Enterobacterales>Enterobacteriaceae	Escherichia_coli	hypothetical-protein,-partial-[Escherichia-coli]	562
WP_071533805.1	DndB-ParB-nuclease	GCF_902168335.1	268	Bacteria>Proteobacteria>Gammaproteobacteria>Enterobacterales>Enterobacteriaceae	Escherichia_coli	MULTISPECIES:-DGQHR-domain-containing-protein-[Enterobacteriaceae]	562
EEZ9759811.1	DndB-ParB-nuclease	GCA_012226525.1	361	Bacteria>Proteobacteria>Gammaproteobacteria>Enterobacterales>Enterobacteriaceae	Escherichia_coli_O25	DNA-sulfur-modification-protein-DndB-[Escherichia-coli-O25]	1095709
ENF20302.1	DndB-ParB-nuclease	GCA_000358435.2	361	Bacteria>Proteobacteria>Gammaproteobacteria>Enterobacterales>Enterobacteriaceae	Escherichia_coli_P0304816.10	DGQHR-domain-protein-[Escherichia-coli-P0304816.10]	1116186
ENF20298.1	DndB-ParB-nuclease	GCA_000358435.2	197	Bacteria>Proteobacteria>Gammaproteobacteria>Enterobacterales>Enterobacteriaceae	Escherichia_coli_P0304816.10	hypothetical-protein-ECP030481610_4736-[Escherichia-coli-P0304816.10]	1116186
ENF36095.1	DndB-ParB-nuclease	GCA_000358475.1	361	Bacteria>Proteobacteria>Gammaproteobacteria>Enterobacterales>Enterobacteriaceae	Escherichia_coli_P0304816.13	DGQHR-domain-protein-[Escherichia-coli-P0304816.13]	1116189
ENF36091.1	DndB-ParB-nuclease	GCA_000358475.1	197	Bacteria>Proteobacteria>Gammaproteobacteria>Enterobacterales>Enterobacteriaceae	Escherichia_coli_P0304816.13	hypothetical-protein-ECP030481613_4730-[Escherichia-coli-P0304816.13]	1116189
ENH27951.1	DndB-ParB-nuclease	GCA_000359015.2	331	Bacteria>Proteobacteria>Gammaproteobacteria>Enterobacterales>Enterobacteriaceae	Escherichia_coli_P0304816.3	DGQHR-domain-protein-[Escherichia-coli-P0304816.3]	1116179
ENH27947.1	DndB-ParB-nuclease	GCA_000359015.2	197	Bacteria>Proteobacteria>Gammaproteobacteria>Enterobacterales>Enterobacteriaceae	Escherichia_coli_P0304816.3	hypothetical-protein-ECP03048163_4727-[Escherichia-coli-P0304816.3]	1116179
BAG80103.1	DndB-ParB-nuclease	GCA_000010385.1	361	Bacteria>Proteobacteria>Gammaproteobacteria>Enterobacterales>Enterobacteriaceae	Escherichia_coli_SE11	conserved-hypothetical-protein-[Escherichia-coli-SE11]	409438
HAG9064550.1	DndB-ParB-nuclease	GCA_012452825.1	268	Bacteria>Proteobacteria>Gammaproteobacteria>Enterobacterales>Enterobacteriaceae	Escherichia_coli	TPA:-DGQHR-domain-containing-protein-[Escherichia-coli]	562
HAI3718132.1	DndB-ParB-nuclease	GCA_012860355.1	268	Bacteria>Proteobacteria>Gammaproteobacteria>Enterobacterales>Enterobacteriaceae	Escherichia_coli	TPA:-DGQHR-domain-containing-protein-[Escherichia-coli]	562
HAM2630796.1	DndB-ParB-nuclease	GCA_014367905.1	268	Bacteria>Proteobacteria>Gammaproteobacteria>Enterobacterales>Enterobacteriaceae	Escherichia_coli	TPA:-DGQHR-domain-containing-protein-[Escherichia-coli]	562
HAM4926281.1	DndB-ParB-nuclease	GCA_014467385.1	386	Bacteria>Proteobacteria>Gammaproteobacteria>Enterobacterales>Enterobacteriaceae	Escherichia_coli	TPA:-DGQHR-domain-containing-protein-[Escherichia-coli]	562
HAN6359647.1	DndB-ParB-nuclease	GCA_014709995.1	268	Bacteria>Proteobacteria>Gammaproteobacteria>Enterobacterales>Enterobacteriaceae	Escherichia_coli	TPA:-DGQHR-domain-containing-protein-[Escherichia-coli]	562
HAN7888338.1	DndB-ParB-nuclease	GCA_014732915.1	268	Bacteria>Proteobacteria>Gammaproteobacteria>Enterobacterales>Enterobacteriaceae	Escherichia_coli	TPA:-DGQHR-domain-containing-protein-[Escherichia-coli]	562
HAW6074795.1	DndB-ParB-nuclease	GCA_016697705.1	268	Bacteria>Proteobacteria>Gammaproteobacteria>Enterobacterales>Enterobacteriaceae	Escherichia_coli	TPA:-DGQHR-domain-containing-protein-[Escherichia-coli]	562
HAW7699332.1	DndB-ParB-nuclease	GCA_016853785.1	268	Bacteria>Proteobacteria>Gammaproteobacteria>Enterobacterales>Enterobacteriaceae	Escherichia_coli	TPA:-DGQHR-domain-containing-protein-[Escherichia-coli]	562
HBH4271695.1	DndB-ParB-nuclease	GCA_019001025.1	268	Bacteria>Proteobacteria>Gammaproteobacteria>Enterobacterales>Enterobacteriaceae	Escherichia_coli	TPA:-DGQHR-domain-containing-protein-[Escherichia-coli]	562
HBL7976746.1	DndB-ParB-nuclease	GCA_021087805.1	268	Bacteria>Proteobacteria>Gammaproteobacteria>Enterobacterales>Enterobacteriaceae	Escherichia_coli	TPA:-DGQHR-domain-containing-protein-[Escherichia-coli]	562
HAG9064546.1	DndB-ParB-nuclease	GCA_012452825.1	361	Bacteria>Proteobacteria>Gammaproteobacteria>Enterobacterales>Enterobacteriaceae	Escherichia_coli	TPA:-DNA-sulfur-modification-protein-DndB-[Escherichia-coli]	562
HAH3502497.1	DndB-ParB-nuclease	GCA_012534755.1	361	Bacteria>Proteobacteria>Gammaproteobacteria>Enterobacterales>Enterobacteriaceae	Escherichia_coli	TPA:-DNA-sulfur-modification-protein-DndB-[Escherichia-coli]	562
HAI3718136.1	DndB-ParB-nuclease	GCA_012860355.1	361	Bacteria>Proteobacteria>Gammaproteobacteria>Enterobacterales>Enterobacteriaceae	Escherichia_coli	TPA:-DNA-sulfur-modification-protein-DndB-[Escherichia-coli]	562
HAJ3365511.1	DndB-ParB-nuclease	GCA_013015435.1	362	Bacteria>Proteobacteria>Gammaproteobacteria>Enterobacterales>Enterobacteriaceae	Escherichia_coli	TPA:-DNA-sulfur-modification-protein-DndB-[Escherichia-coli]	562
HAL7299092.1	DndB-ParB-nuclease	GCA_014157855.1	361	Bacteria>Proteobacteria>Gammaproteobacteria>Enterobacterales>Enterobacteriaceae	Escherichia_coli	TPA:-DNA-sulfur-modification-protein-DndB-[Escherichia-coli]	562
HAM0417112.1	DndB-ParB-nuclease	GCA_014209255.1	362	Bacteria>Proteobacteria>Gammaproteobacteria>Enterobacterales>Enterobacteriaceae	Escherichia_coli	TPA:-DNA-sulfur-modification-protein-DndB-[Escherichia-coli]	562
HAM2630800.1	DndB-ParB-nuclease	GCA_014367905.1	361	Bacteria>Proteobacteria>Gammaproteobacteria>Enterobacterales>Enterobacteriaceae	Escherichia_coli	TPA:-DNA-sulfur-modification-protein-DndB-[Escherichia-coli]	562
HAM4926285.1	DndB-ParB-nuclease	GCA_014467385.1	361	Bacteria>Proteobacteria>Gammaproteobacteria>Enterobacterales>Enterobacteriaceae	Escherichia_coli	TPA:-DNA-sulfur-modification-protein-DndB-[Escherichia-coli]	562
HAN1915192.1	DndB-ParB-nuclease	GCA_014639515.1	361	Bacteria>Proteobacteria>Gammaproteobacteria>Enterobacterales>Enterobacteriaceae	Escherichia_coli	TPA:-DNA-sulfur-modification-protein-DndB-[Escherichia-coli]	562
HAN6359643.1	DndB-ParB-nuclease	GCA_014709995.1	361	Bacteria>Proteobacteria>Gammaproteobacteria>Enterobacterales>Enterobacteriaceae	Escherichia_coli	TPA:-DNA-sulfur-modification-protein-DndB-[Escherichia-coli]	562
HAN7888334.1	DndB-ParB-nuclease	GCA_014732915.1	361	Bacteria>Proteobacteria>Gammaproteobacteria>Enterobacterales>Enterobacteriaceae	Escherichia_coli	TPA:-DNA-sulfur-modification-protein-DndB-[Escherichia-coli]	562
HAP0290523.1	DndB-ParB-nuclease	GCA_015119175.1	361	Bacteria>Proteobacteria>Gammaproteobacteria>Enterobacterales>Enterobacteriaceae	Escherichia_coli	TPA:-DNA-sulfur-modification-protein-DndB-[Escherichia-coli]	562
HAW0183548.1	DndB-ParB-nuclease	GCA_016668745.1	361	Bacteria>Proteobacteria>Gammaproteobacteria>Enterobacterales>Enterobacteriaceae	Escherichia_coli	TPA:-DNA-sulfur-modification-protein-DndB-[Escherichia-coli]	562
HAW0614602.1	DndB-ParB-nuclease	GCA_016670685.1	361	Bacteria>Proteobacteria>Gammaproteobacteria>Enterobacterales>Enterobacteriaceae	Escherichia_coli	TPA:-DNA-sulfur-modification-protein-DndB-[Escherichia-coli]	562
HAW6074799.1	DndB-ParB-nuclease	GCA_016697705.1	361	Bacteria>Proteobacteria>Gammaproteobacteria>Enterobacterales>Enterobacteriaceae	Escherichia_coli	TPA:-DNA-sulfur-modification-protein-DndB-[Escherichia-coli]	562
HAW7699328.1	DndB-ParB-nuclease	GCA_016853785.1	361	Bacteria>Proteobacteria>Gammaproteobacteria>Enterobacterales>Enterobacteriaceae	Escherichia_coli	TPA:-DNA-sulfur-modification-protein-DndB-[Escherichia-coli]	562
HAW8312422.1	DndB-ParB-nuclease	GCA_016856985.1	361	Bacteria>Proteobacteria>Gammaproteobacteria>Enterobacterales>Enterobacteriaceae	Escherichia_coli	TPA:-DNA-sulfur-modification-protein-DndB-[Escherichia-coli]	562
HAX5136930.1	DndB-ParB-nuclease	GCA_017018655.1	361	Bacteria>Proteobacteria>Gammaproteobacteria>Enterobacterales>Enterobacteriaceae	Escherichia_coli	TPA:-DNA-sulfur-modification-protein-DndB-[Escherichia-coli]	562
HAX7000543.1	DndB-ParB-nuclease	GCA_017046215.1	362	Bacteria>Proteobacteria>Gammaproteobacteria>Enterobacterales>Enterobacteriaceae	Escherichia_coli	TPA:-DNA-sulfur-modification-protein-DndB-[Escherichia-coli]	562
HAX9059299.1	DndB-ParB-nuclease	GCA_017064155.1	361	Bacteria>Proteobacteria>Gammaproteobacteria>Enterobacterales>Enterobacteriaceae	Escherichia_coli	TPA:-DNA-sulfur-modification-protein-DndB-[Escherichia-coli]	562
HAX9940824.1	DndB-ParB-nuclease	GCA_017074215.1	361	Bacteria>Proteobacteria>Gammaproteobacteria>Enterobacterales>Enterobacteriaceae	Escherichia_coli	TPA:-DNA-sulfur-modification-protein-DndB-[Escherichia-coli]	562
HAZ3906172.1	DndB-ParB-nuclease	GCA_017318465.1	362	Bacteria>Proteobacteria>Gammaproteobacteria>Enterobacterales>Enterobacteriaceae	Escherichia_coli	TPA:-DNA-sulfur-modification-protein-DndB-[Escherichia-coli]	562
HBA6987379.1	DndB-ParB-nuclease	GCA_017763325.1	361	Bacteria>Proteobacteria>Gammaproteobacteria>Enterobacterales>Enterobacteriaceae	Escherichia_coli	TPA:-DNA-sulfur-modification-protein-DndB-[Escherichia-coli]	562
HBA6989949.1	DndB-ParB-nuclease	GCA_017763325.1	361	Bacteria>Proteobacteria>Gammaproteobacteria>Enterobacterales>Enterobacteriaceae	Escherichia_coli	TPA:-DNA-sulfur-modification-protein-DndB-[Escherichia-coli]	562
HBA7189031.1	DndB-ParB-nuclease	GCA_017763505.1	362	Bacteria>Proteobacteria>Gammaproteobacteria>Enterobacterales>Enterobacteriaceae	Escherichia_coli	TPA:-DNA-sulfur-modification-protein-DndB-[Escherichia-coli]	562
HBA7306486.1	DndB-ParB-nuclease	GCA_017764385.1	361	Bacteria>Proteobacteria>Gammaproteobacteria>Enterobacterales>Enterobacteriaceae	Escherichia_coli	TPA:-DNA-sulfur-modification-protein-DndB-[Escherichia-coli]	562
HBA7721832.1	DndB-ParB-nuclease	GCA_017766625.1	361	Bacteria>Proteobacteria>Gammaproteobacteria>Enterobacterales>Enterobacteriaceae	Escherichia_coli	TPA:-DNA-sulfur-modification-protein-DndB-[Escherichia-coli]	562
HBA7918413.1	DndB-ParB-nuclease	GCA_017767425.1	361	Bacteria>Proteobacteria>Gammaproteobacteria>Enterobacterales>Enterobacteriaceae	Escherichia_coli	TPA:-DNA-sulfur-modification-protein-DndB-[Escherichia-coli]	562
HBA8591297.1	DndB-ParB-nuclease	GCA_017770505.1	361	Bacteria>Proteobacteria>Gammaproteobacteria>Enterobacterales>Enterobacteriaceae	Escherichia_coli	TPA:-DNA-sulfur-modification-protein-DndB-[Escherichia-coli]	562
HBB0225529.1	DndB-ParB-nuclease	GCA_017778625.1	361	Bacteria>Proteobacteria>Gammaproteobacteria>Enterobacterales>Enterobacteriaceae	Escherichia_coli	TPA:-DNA-sulfur-modification-protein-DndB-[Escherichia-coli]	562
HBB5045923.1	DndB-ParB-nuclease	GCA_017910475.1	361	Bacteria>Proteobacteria>Gammaproteobacteria>Enterobacterales>Enterobacteriaceae	Escherichia_coli	TPA:-DNA-sulfur-modification-protein-DndB-[Escherichia-coli]	562
HBD8387691.1	DndB-ParB-nuclease	GCA_018510145.1	361	Bacteria>Proteobacteria>Gammaproteobacteria>Enterobacterales>Enterobacteriaceae	Escherichia_coli	TPA:-DNA-sulfur-modification-protein-DndB-[Escherichia-coli]	562
HBE4591075.1	DndB-ParB-nuclease	GCA_018561705.1	361	Bacteria>Proteobacteria>Gammaproteobacteria>Enterobacterales>Enterobacteriaceae	Escherichia_coli	TPA:-DNA-sulfur-modification-protein-DndB-[Escherichia-coli]	562
HBE4605425.1	DndB-ParB-nuclease	GCA_018561605.1	362	Bacteria>Proteobacteria>Gammaproteobacteria>Enterobacterales>Enterobacteriaceae	Escherichia_coli	TPA:-DNA-sulfur-modification-protein-DndB-[Escherichia-coli]	562
HBH4271699.1	DndB-ParB-nuclease	GCA_019001025.1	361	Bacteria>Proteobacteria>Gammaproteobacteria>Enterobacterales>Enterobacteriaceae	Escherichia_coli	TPA:-DNA-sulfur-modification-protein-DndB-[Escherichia-coli]	562
HBL7976750.1	DndB-ParB-nuclease	GCA_021087805.1	361	Bacteria>Proteobacteria>Gammaproteobacteria>Enterobacterales>Enterobacteriaceae	Escherichia_coli	TPA:-DNA-sulfur-modification-protein-DndB-[Escherichia-coli]	562
HAG9064551.1	DndB-ParB-nuclease	GCA_012452825.1	101	Bacteria>Proteobacteria>Gammaproteobacteria>Enterobacterales>Enterobacteriaceae	Escherichia_coli	TPA:-hypothetical-protein-F6T32_23240-[Escherichia-coli]	562
HAH3502492.1	DndB-ParB-nuclease	GCA_012534755.1	101	Bacteria>Proteobacteria>Gammaproteobacteria>Enterobacterales>Enterobacteriaceae	Escherichia_coli	TPA:-hypothetical-protein-GFX57_24400-[Escherichia-coli]	562
HAH3502493.1	DndB-ParB-nuclease	GCA_012534755.1	197	Bacteria>Proteobacteria>Gammaproteobacteria>Enterobacterales>Enterobacteriaceae	Escherichia_coli	TPA:-hypothetical-protein-GFX57_24405-[Escherichia-coli]	562
HAI3718131.1	DndB-ParB-nuclease	GCA_012860355.1	101	Bacteria>Proteobacteria>Gammaproteobacteria>Enterobacterales>Enterobacteriaceae	Escherichia_coli	TPA:-hypothetical-protein-HJN17_001353-[Escherichia-coli]	562
HAM2630795.1	DndB-ParB-nuclease	GCA_014367905.1	101	Bacteria>Proteobacteria>Gammaproteobacteria>Enterobacterales>Enterobacteriaceae	Escherichia_coli	TPA:-hypothetical-protein-IBD52_002953-[Escherichia-coli]	562
HAN6359648.1	DndB-ParB-nuclease	GCA_014709995.1	101	Bacteria>Proteobacteria>Gammaproteobacteria>Enterobacterales>Enterobacteriaceae	Escherichia_coli	TPA:-hypothetical-protein-IF021_004857-[Escherichia-coli]	562
HAN7888339.1	DndB-ParB-nuclease	GCA_014732915.1	101	Bacteria>Proteobacteria>Gammaproteobacteria>Enterobacterales>Enterobacteriaceae	Escherichia_coli	TPA:-hypothetical-protein-IF862_002135-[Escherichia-coli]	562
HAW6074794.1	DndB-ParB-nuclease	GCA_016697705.1	101	Bacteria>Proteobacteria>Gammaproteobacteria>Enterobacterales>Enterobacteriaceae	Escherichia_coli	TPA:-hypothetical-protein-JL412_000586-[Escherichia-coli]	562
HAW7699333.1	DndB-ParB-nuclease	GCA_016853785.1	101	Bacteria>Proteobacteria>Gammaproteobacteria>Enterobacterales>Enterobacteriaceae	Escherichia_coli	TPA:-hypothetical-protein-JLJ29_002515-[Escherichia-coli]	562
HBE4591070.1	DndB-ParB-nuclease	GCA_018561705.1	101	Bacteria>Proteobacteria>Gammaproteobacteria>Enterobacterales>Enterobacteriaceae	Escherichia_coli	TPA:-hypothetical-protein-KL851_000226-[Escherichia-coli]	562
HBH4271694.1	DndB-ParB-nuclease	GCA_019001025.1	101	Bacteria>Proteobacteria>Gammaproteobacteria>Enterobacterales>Enterobacteriaceae	Escherichia_coli	TPA:-hypothetical-protein-KTD59_003734-[Escherichia-coli]	562
HBL7976745.1	DndB-ParB-nuclease	GCA_021087805.1	101	Bacteria>Proteobacteria>Gammaproteobacteria>Enterobacterales>Enterobacteriaceae	Escherichia_coli	TPA:-hypothetical-protein-LTB83_001120-[Escherichia-coli]	562
EGC96567.1	DndB-ParB-nuclease	GCA_000191665.1	331	Bacteria>Proteobacteria>Gammaproteobacteria>Enterobacterales>Enterobacteriaceae	Escherichia_fergusonii_ECD227	DNA-sulfur-modification-protein-DndB-[Escherichia-fergusonii-ECD227]	981367
EGC96571.1	DndB-ParB-nuclease	GCA_000191665.1	439	Bacteria>Proteobacteria>Gammaproteobacteria>Enterobacterales>Enterobacteriaceae	Escherichia_fergusonii_ECD227	hypothetical-protein-ECD227_2809-[Escherichia-fergusonii-ECD227]	981367
WP_001032040.1	DndB-ParB-nuclease	GCF_000191665.1	407	Bacteria>Proteobacteria>Gammaproteobacteria>Enterobacterales>Enterobacteriaceae	Escherichia	MULTISPECIES:-DGQHR-domain-containing-protein-[Escherichia]	561
WP_000179015.1	DndB-ParB-nuclease	GCF_000010385.1	361	Bacteria>Proteobacteria>Gammaproteobacteria>Enterobacterales>Enterobacteriaceae	Escherichia	MULTISPECIES:-DNA-sulfur-modification-protein-DndB-[Escherichia]	561
WP_000179016.1	DndB-ParB-nuclease	GCF_000191665.1	361	Bacteria>Proteobacteria>Gammaproteobacteria>Enterobacterales>Enterobacteriaceae	Escherichia	MULTISPECIES:-DNA-sulfur-modification-protein-DndB-[Escherichia]	561
WP_135404012.1	DndB-ParB-nuclease	GCF_004745115.1	402	Bacteria>Proteobacteria>Gammaproteobacteria>Enterobacterales>Enterobacteriaceae	Escherichia_sp._E4930	DGQHR-domain-containing-protein-[Escherichia-sp.-E4930]	2044468
WP_049294600.1	DndB-ParB-nuclease	GCF_001063355.1	360	Bacteria>Proteobacteria>Gammaproteobacteria>Enterobacterales>Enterobacteriaceae	Franconibacter_helveticus	DNA-sulfur-modification-protein-DndB-[Franconibacter-helveticus]	357240
WP_048229588.1	DndB-ParB-nuclease	GCF_001030125.1	568	Bacteria>Proteobacteria>Gammaproteobacteria>Enterobacterales>Enterobacteriaceae	Klebsiella_aerogenes	AIPR-family-protein-[Klebsiella-aerogenes]	548
MBF8484819.1	DndB-ParB-nuclease	GCA_015679435.1	361	Bacteria>Proteobacteria>Gammaproteobacteria>Enterobacterales>Enterobacteriaceae	Klebsiella_aerogenes	DNA-sulfur-modification-protein-DndB-[Klebsiella-aerogenes]	548
WP_058675773.1	DndB-ParB-nuclease	GCF_006874725.1	361	Bacteria>Proteobacteria>Gammaproteobacteria>Enterobacterales>Enterobacteriaceae	Klebsiella_aerogenes	DNA-sulfur-modification-protein-DndB-[Klebsiella-aerogenes]	548
WP_193129691.1	DndB-ParB-nuclease	GCF_014902855.1	358	Bacteria>Proteobacteria>Gammaproteobacteria>Enterobacterales>Enterobacteriaceae	Klebsiella_aerogenes	DNA-sulfur-modification-protein-DndB-[Klebsiella-aerogenes]	548
WP_196092900.1	DndB-ParB-nuclease	GCF_015679435.1	361	Bacteria>Proteobacteria>Gammaproteobacteria>Enterobacterales>Enterobacteriaceae	Klebsiella_aerogenes	DNA-sulfur-modification-protein-DndB-[Klebsiella-aerogenes]	548
WP_126033577.1	DndB-ParB-nuclease	GCF_003952125.1	575	Bacteria>Proteobacteria>Gammaproteobacteria>Enterobacterales>Enterobacteriaceae	Klebsiella_aerogenes	hypothetical-protein-[Klebsiella-aerogenes]	548
WP_193128993.1	DndB-ParB-nuclease	GCF_014902795.1	562	Bacteria>Proteobacteria>Gammaproteobacteria>Enterobacterales>Enterobacteriaceae	Klebsiella_aerogenes	hypothetical-protein-[Klebsiella-aerogenes]	548
STW09650.1	DndB-ParB-nuclease	GCA_900461515.1	387	Bacteria>Proteobacteria>Gammaproteobacteria>Enterobacterales>Enterobacteriaceae	Klebsiella_grimontii	DNA-phosphorothioation-associated-DGQHR-protein-1-[Klebsiella-grimontii]	2058152
STW09645.1	DndB-ParB-nuclease	GCA_900461515.1	361	Bacteria>Proteobacteria>Gammaproteobacteria>Enterobacterales>Enterobacteriaceae	Klebsiella_grimontii	DNA-sulfur-modification-protein-DndB-[Klebsiella-grimontii]	2058152

Accession	Annotation	GCA	Length	Phyletic.spread	Strain.name	Defline	Species.Taxid
WP_200913876.1	DndB-ParB-nuclease	GCF_015139575.1	382	Bacteria>Proteobacteria>Gammaproteobacteria>Enterobacterales>Enterobacteriaceae	Klebsiella_michiganensis	DGQHR-domain-containing-protein-[Klebsiella-michiganensis]	1134687
BBW78528.1	DndB-ParB-nuclease	GCA_015139575.1	361	Bacteria>Proteobacteria>Gammaproteobacteria>Enterobacterales>Enterobacteriaceae	Klebsiella_michiganensis	hypothetical-protein-THOKLE011_48180-[Klebsiella-michiganensis]	1134687
BBW78532.1	DndB-ParB-nuclease	GCA_015139575.1	382	Bacteria>Proteobacteria>Gammaproteobacteria>Enterobacterales>Enterobacteriaceae	Klebsiella_michiganensis	hypothetical-protein-THOKLE011_48220-[Klebsiella-michiganensis]	1134687
WP_032747693.1	DndB-ParB-nuclease	GCF_015139575.1	361	Bacteria>Proteobacteria>Gammaproteobacteria>Enterobacterales>Enterobacteriaceae	Klebsiella_michiganensis	MULTISPECIES:-DNA-sulfur-modification-protein-DndB-[Klebsiella]	1134687
HBK4730951.1	DndB-ParB-nuclease	GCA_020681625.1	405	Bacteria>Proteobacteria>Gammaproteobacteria>Enterobacterales>Enterobacteriaceae	Klebsiella_michiganensis	TPA:-DGQHR-domain-containing-protein-[Klebsiella-michiganensis]	1134687
HBK4730946.1	DndB-ParB-nuclease	GCA_020681625.1	361	Bacteria>Proteobacteria>Gammaproteobacteria>Enterobacterales>Enterobacteriaceae	Klebsiella_michiganensis	TPA:-DNA-sulfur-modification-protein-DndB-[Klebsiella-michiganensis]	1134687
HBM3157249.1	DndB-ParB-nuclease	GCA_021210555.1	575	Bacteria>Proteobacteria>Gammaproteobacteria>Enterobacterales>Enterobacteriaceae	Klebsiella_michiganensis	TPA:-hypothetical-protein-LVL83_002647-[Klebsiella-michiganensis]	1134687
WP_075666949.1	DndB-ParB-nuclease	GCF_902807145.1	403	Bacteria>Proteobacteria>Gammaproteobacteria>Enterobacterales>Enterobacteriaceae	Klebsiella	MULTISPECIES:-DGQHR-domain-containing-protein-[Klebsiella]	570
WP_047051975.1	DndB-ParB-nuclease	GCF_024917575.1	361	Bacteria>Proteobacteria>Gammaproteobacteria>Enterobacterales>Enterobacteriaceae	Klebsiella	MULTISPECIES:-DNA-sulfur-modification-protein-DndB-[Klebsiella]	570
WP_058330575.1	DndB-ParB-nuclease	GCF_004312245.1	361	Bacteria>Proteobacteria>Gammaproteobacteria>Enterobacterales>Enterobacteriaceae	Klebsiella	MULTISPECIES:-DNA-sulfur-modification-protein-DndB-[Klebsiella]	570
WP_064146465.1	DndB-ParB-nuclease	GCF_004311505.1	361	Bacteria>Proteobacteria>Gammaproteobacteria>Enterobacterales>Enterobacteriaceae	Klebsiella	MULTISPECIES:-DNA-sulfur-modification-protein-DndB-[Klebsiella]	570
WP_115240039.1	DndB-ParB-nuclease	GCF_902158635.1	361	Bacteria>Proteobacteria>Gammaproteobacteria>Enterobacterales>Enterobacteriaceae	Klebsiella	MULTISPECIES:-DNA-sulfur-modification-protein-DndB-[Klebsiella]	570
WP_135630371.1	DndB-ParB-nuclease	GCF_900776525.1	361	Bacteria>Proteobacteria>Gammaproteobacteria>Enterobacterales>Enterobacteriaceae	Klebsiella	MULTISPECIES:-DNA-sulfur-modification-protein-DndB-[Klebsiella]	570
HAT1685286.1	DndB-ParB-nuclease	GCA_015893305.1	96	Bacteria>Proteobacteria>Gammaproteobacteria>Enterobacterales>Enterobacteriaceae	Klebsiella_oxytoca	DGQHR-domain-containing-protein-[Klebsiella-oxytoca]	571
HAT1627191.1	DndB-ParB-nuclease	GCA_015893885.1	385	Bacteria>Proteobacteria>Gammaproteobacteria>Enterobacterales>Enterobacteriaceae	Klebsiella_oxytoca	DGQHR-domain-containing-protein-[Klebsiella-oxytoca]	571
HBC9240818.1	DndB-ParB-nuclease	GCA_018184775.1	389	Bacteria>Proteobacteria>Gammaproteobacteria>Enterobacterales>Enterobacteriaceae	Klebsiella_oxytoca	DGQHR-domain-containing-protein-[Klebsiella-oxytoca]	571
MBL6000772.1	DndB-ParB-nuclease	GCA_016773175.1	385	Bacteria>Proteobacteria>Gammaproteobacteria>Enterobacterales>Enterobacteriaceae	Klebsiella_oxytoca	DGQHR-domain-containing-protein-[Klebsiella-oxytoca]	571
WP_168217120.1	DndB-ParB-nuclease	GCF_004785705.1	397	Bacteria>Proteobacteria>Gammaproteobacteria>Enterobacterales>Enterobacteriaceae	Klebsiella_oxytoca	DGQHR-domain-containing-protein-[Klebsiella-oxytoca]	571
WP_202862120.1	DndB-ParB-nuclease	GCF_025548455.1	385	Bacteria>Proteobacteria>Gammaproteobacteria>Enterobacterales>Enterobacteriaceae	Klebsiella_oxytoca	DGQHR-domain-containing-protein-[Klebsiella-oxytoca]	571
HAT1627195.1	DndB-ParB-nuclease	GCA_015893885.1	361	Bacteria>Proteobacteria>Gammaproteobacteria>Enterobacterales>Enterobacteriaceae	Klebsiella_oxytoca	DNA-sulfur-modification-protein-DndB-[Klebsiella-oxytoca]	571
HAT1685290.1	DndB-ParB-nuclease	GCA_015893305.1	361	Bacteria>Proteobacteria>Gammaproteobacteria>Enterobacterales>Enterobacteriaceae	Klebsiella_oxytoca	DNA-sulfur-modification-protein-DndB-[Klebsiella-oxytoca]	571
HBC9240814.1	DndB-ParB-nuclease	GCA_018184775.1	361	Bacteria>Proteobacteria>Gammaproteobacteria>Enterobacterales>Enterobacteriaceae	Klebsiella_oxytoca	DNA-sulfur-modification-protein-DndB-[Klebsiella-oxytoca]	571
MBL6000768.1	DndB-ParB-nuclease	GCA_016773175.1	361	Bacteria>Proteobacteria>Gammaproteobacteria>Enterobacterales>Enterobacteriaceae	Klebsiella_oxytoca	DNA-sulfur-modification-protein-DndB-[Klebsiella-oxytoca]	571
QGN36340.1	DndB-ParB-nuclease	GCA_009707385.1	359	Bacteria>Proteobacteria>Gammaproteobacteria>Enterobacterales>Enterobacteriaceae	Klebsiella_oxytoca	DNA-sulfur-modification-protein-DndB-[Klebsiella-oxytoca]	571
WP_049094514.1	DndB-ParB-nuclease	GCF_001063775.1	361	Bacteria>Proteobacteria>Gammaproteobacteria>Enterobacterales>Enterobacteriaceae	Klebsiella_oxytoca	DNA-sulfur-modification-protein-DndB-[Klebsiella-oxytoca]	571
WP_154678880.1	DndB-ParB-nuclease	GCF_009707385.1	359	Bacteria>Proteobacteria>Gammaproteobacteria>Enterobacterales>Enterobacteriaceae	Klebsiella_oxytoca	DNA-sulfur-modification-protein-DndB-[Klebsiella-oxytoca]	571
WP_202862118.1	DndB-ParB-nuclease	-	361	Bacteria>Proteobacteria>Gammaproteobacteria>Enterobacterales>Enterobacteriaceae	Klebsiella_oxytoca	DNA-sulfur-modification-protein-DndB-[Klebsiella-oxytoca]	571
QGN36335.1	DndB-ParB-nuclease	GCA_009707385.1	563	Bacteria>Proteobacteria>Gammaproteobacteria>Enterobacterales>Enterobacteriaceae	Klebsiella_oxytoca	hypothetical-protein-GJ746_03035-[Klebsiella-oxytoca]	571
WP_154678876.1	DndB-ParB-nuclease	GCF_009707385.1	563	Bacteria>Proteobacteria>Gammaproteobacteria>Enterobacterales>Enterobacteriaceae	Klebsiella_oxytoca	hypothetical-protein-[Klebsiella-oxytoca]	571
HBM2923969.1	DndB-ParB-nuclease	GCA_021207155.1	378	Bacteria>Proteobacteria>Gammaproteobacteria>Enterobacterales>Enterobacteriaceae	Klebsiella_oxytoca	TPA:-DGQHR-domain-containing-protein-[Klebsiella-oxytoca]	571
HBM2923979.1	DndB-ParB-nuclease	GCA_021207155.1	361	Bacteria>Proteobacteria>Gammaproteobacteria>Enterobacterales>Enterobacteriaceae	Klebsiella_oxytoca	TPA:-DNA-sulfur-modification-protein-DndB-[Klebsiella-oxytoca]	571
WP_142471629.1	DndB-ParB-nuclease	GCF_902158695.1	403	Bacteria>Proteobacteria>Gammaproteobacteria>Enterobacterales>Enterobacteriaceae	Klebsiella_pasteurii	DNA-phosphorothioation-associated-DGQHR-protein-1-[Klebsiella-pasteurii]	2587529
WP_135630378.1	DndB-ParB-nuclease	GCF_900776525.1	389	Bacteria>Proteobacteria>Gammaproteobacteria>Enterobacterales>Enterobacteriaceae	Klebsiella_pneumoniae	DGQHR-domain-containing-protein-[Klebsiella-pneumoniae]	573
WP_172613253.1	DndB-ParB-nuclease	GCF_902166745.1	401	Bacteria>Proteobacteria>Gammaproteobacteria>Enterobacterales>Enterobacteriaceae	Klebsiella_pneumoniae	DGQHR-domain-containing-protein-[Klebsiella-pneumoniae]	573
WP_075666954.1	DndB-ParB-nuclease	GCF_008373035.2	361	Bacteria>Proteobacteria>Gammaproteobacteria>Enterobacterales>Enterobacteriaceae	Klebsiella_pneumoniae	DNA-sulfur-modification-protein-DndB-[Klebsiella-pneumoniae]	573
WP_114263368.1	DndB-ParB-nuclease	GCF_900493135.1	361	Bacteria>Proteobacteria>Gammaproteobacteria>Enterobacterales>Enterobacteriaceae	Klebsiella_pneumoniae	DNA-sulfur-modification-protein-DndB-[Klebsiella-pneumoniae]	573
WP_142675522.1	DndB-ParB-nuclease	GCF_900493135.1	688	Bacteria>Proteobacteria>Gammaproteobacteria>Enterobacterales>Enterobacteriaceae	Klebsiella_pneumoniae	hypothetical-protein-[Klebsiella-pneumoniae]	573
WP_228284130.1	DndB-ParB-nuclease	GCF_013377655.1	568	Bacteria>Proteobacteria>Gammaproteobacteria>Enterobacterales>Enterobacteriaceae	Klebsiella_quasipneumoniae	AIPR-family-protein-[Klebsiella-quasipneumoniae]	1463165
WP_205608972.1	DndB-ParB-nuclease	GCF_013377655.1	361	Bacteria>Proteobacteria>Gammaproteobacteria>Enterobacterales>Enterobacteriaceae	Klebsiella_quasipneumoniae	DNA-sulfur-modification-protein-DndB-[Klebsiella-quasipneumoniae]	1463165
MBZ6420512.1	DndB-ParB-nuclease	GCA_020093085.1	568	Bacteria>Proteobacteria>Gammaproteobacteria>Enterobacterales>Enterobacteriaceae	Klebsiella_sp.	AIPR-family-protein-[Klebsiella-sp.]	576
MBZ6420516.1	DndB-ParB-nuclease	GCA_020093085.1	361	Bacteria>Proteobacteria>Gammaproteobacteria>Enterobacterales>Enterobacteriaceae	Klebsiella_sp.	DNA-sulfur-modification-protein-DndB-[Klebsiella-sp.]	576
WP_111460401.1	DndB-ParB-nuclease	GCF_003253835.1	567	Bacteria>Proteobacteria>Gammaproteobacteria>Enterobacterales>Enterobacteriaceae	Klebsiella_variicola	AIPR-family-protein-[Klebsiella-variicola]	244366
WP_046622682.1	DndB-ParB-nuclease	GCF_021395795.1	383	Bacteria>Proteobacteria>Gammaproteobacteria>Enterobacterales>Enterobacteriaceae	Klebsiella_variicola	DGQHR-domain-containing-protein-[Klebsiella-variicola]	244366
WP_181400691.1	DndB-ParB-nuclease	GCF_023147105.1	415	Bacteria>Proteobacteria>Gammaproteobacteria>Enterobacterales>Enterobacteriaceae	Klebsiella_variicola	DGQHR-domain-containing-protein-[Klebsiella-variicola]	244366
MBC5380281.1	DndB-ParB-nuclease	GCA_014289415.1	361	Bacteria>Proteobacteria>Gammaproteobacteria>Enterobacterales>Enterobacteriaceae	Klebsiella_variicola	DNA-sulfur-modification-protein-DndB-[Klebsiella-variicola]	244366
STW25031.1	DndB-ParB-nuclease	GCA_900452005.1	361	Bacteria>Proteobacteria>Gammaproteobacteria>Enterobacterales>Enterobacteriaceae	Klebsiella_variicola	DNA-sulfur-modification-protein-DndB-[Klebsiella-variicola]	244366
WP_046622689.1	DndB-ParB-nuclease	GCF_021395795.1	361	Bacteria>Proteobacteria>Gammaproteobacteria>Enterobacterales>Enterobacteriaceae	Klebsiella_variicola	DNA-sulfur-modification-protein-DndB-[Klebsiella-variicola]	244366
WP_110184842.1	DndB-ParB-nuclease	GCF_013395165.1	361	Bacteria>Proteobacteria>Gammaproteobacteria>Enterobacterales>Enterobacteriaceae	Klebsiella_variicola	DNA-sulfur-modification-protein-DndB-[Klebsiella-variicola]	244366
WP_110217315.1	DndB-ParB-nuclease	GCF_023147105.1	361	Bacteria>Proteobacteria>Gammaproteobacteria>Enterobacterales>Enterobacteriaceae	Klebsiella_variicola	DNA-sulfur-modification-protein-DndB-[Klebsiella-variicola]	244366
WP_111460397.1	DndB-ParB-nuclease	GCF_903970905.1	361	Bacteria>Proteobacteria>Gammaproteobacteria>Enterobacterales>Enterobacteriaceae	Klebsiella_variicola	DNA-sulfur-modification-protein-DndB-[Klebsiella-variicola]	244366
WP_114500667.1	DndB-ParB-nuclease	GCF_022857325.1	361	Bacteria>Proteobacteria>Gammaproteobacteria>Enterobacterales>Enterobacteriaceae	Klebsiella_variicola	DNA-sulfur-modification-protein-DndB-[Klebsiella-variicola]	244366
WP_122463381.1	DndB-ParB-nuclease	GCF_900576775.1	361	Bacteria>Proteobacteria>Gammaproteobacteria>Enterobacterales>Enterobacteriaceae	Klebsiella_variicola	DNA-sulfur-modification-protein-DndB-[Klebsiella-variicola]	244366
WP_110199967.1	DndB-ParB-nuclease	GCF_003196975.1	575	Bacteria>Proteobacteria>Gammaproteobacteria>Enterobacterales>Enterobacteriaceae	Klebsiella_variicola	hypothetical-protein-[Klebsiella-variicola]	244366
HBQ2553114.1	DndB-ParB-nuclease	GCA_021837705.1	361	Bacteria>Proteobacteria>Gammaproteobacteria>Enterobacterales>Enterobacteriaceae	Klebsiella_variicola	TPA:-DNA-sulfur-modification-protein-DndB-[Klebsiella-variicola]	244366
TNL08473.1	DndB-ParB-nuclease	GCA_006333905.1	361	Bacteria>Proteobacteria>Gammaproteobacteria>Enterobacterales>Enterobacteriaceae	Kosakonia_cowanii	DNA-sulfur-modification-protein-DndB-[Kosakonia-cowanii]	208223
TNL08478.1	DndB-ParB-nuclease	GCA_006333905.1	195	Bacteria>Proteobacteria>Gammaproteobacteria>Enterobacterales>Enterobacteriaceae	Kosakonia_cowanii	hypothetical-protein-CYD30_16815-[Kosakonia-cowanii]	208223
WP_076768977.1	DndB-ParB-nuclease	GCF_001975225.1	361	Bacteria>Proteobacteria>Gammaproteobacteria>Enterobacterales>Enterobacteriaceae	Kosakonia	MULTISPECIES:-DNA-sulfur-modification-protein-DndB-[Kosakonia]	1330547
WP_043955296.1	DndB-ParB-nuclease	GCF_000877295.1	361	Bacteria>Proteobacteria>Gammaproteobacteria>Enterobacterales>Enterobacteriaceae	Kosakonia_radicincitans	DNA-sulfur-modification-protein-DndB-[Kosakonia-radicincitans]	283686
WP_187801138.1	DndB-ParB-nuclease	GCF_014490785.1	389	Bacteria>Proteobacteria>Gammaproteobacteria>Enterobacterales>Enterobacteriaceae	Kosakonia_sp._SMBL-WEM22	DGQHR-domain-containing-protein-[Kosakonia-sp.-SMBL-WEM22]	2725560
WP_230352729.1	DndB-ParB-nuclease	GCF_020532665.2	361	Bacteria>Proteobacteria>Gammaproteobacteria>Enterobacterales>Enterobacteriaceae	Lelliottia_sp._WAP21	DNA-sulfur-modification-protein-DndB-[Lelliottia-sp.-WAP21]	2877426
WP_049094506.1	DndB-ParB-nuclease	GCF_005518115.1	387	Bacteria>Proteobacteria>Gammaproteobacteria>Enterobacterales	Enterobacteriaceae	MULTISPECIES:-DGQHR-domain-containing-protein-[Enterobacteriaceae]	543
WP_065811018.1	DndB-ParB-nuclease	GCF_900093025.1	397	Bacteria>Proteobacteria>Gammaproteobacteria>Enterobacterales	Enterobacteriaceae	MULTISPECIES:-DGQHR-domain-containing-protein-[Enterobacteriaceae]	543
WP_162920345.1	DndB-ParB-nuclease	GCF_003590695.1	379	Bacteria>Proteobacteria>Gammaproteobacteria>Enterobacterales	Enterobacteriaceae	MULTISPECIES:-DGQHR-domain-containing-protein-[Enterobacteriaceae]	543
WP_176208046.1	DndB-ParB-nuclease	GCF_025218545.1	415	Bacteria>Proteobacteria>Gammaproteobacteria>Enterobacterales	Enterobacteriaceae	MULTISPECIES:-DGQHR-domain-containing-protein-[Enterobacteriaceae]	543
WP_024156434.1	DndB-ParB-nuclease	GCF_000416295.1	403	Bacteria>Proteobacteria>Gammaproteobacteria>Enterobacterales	Enterobacteriaceae	MULTISPECIES:-DNA-phosphorothioation-associated-DGQHR-protein-1-[Enterobacteriaceae]	543
WP_072048481.1	DndB-ParB-nuclease	GCF_005391545.1	386	Bacteria>Proteobacteria>Gammaproteobacteria>Enterobacterales	Enterobacteriaceae	MULTISPECIES:-DNA-phosphorothioation-associated-DGQHR-protein-1-[Enterobacteriaceae]	543
WP_117363024.1	DndB-ParB-nuclease	GCF_024917575.1	403	Bacteria>Proteobacteria>Gammaproteobacteria>Enterobacterales	Enterobacteriaceae	MULTISPECIES:-DNA-phosphorothioation-associated-DGQHR-protein-1-[Enterobacteriaceae]	543
WP_213839505.1	DndB-ParB-nuclease	GCF_019880265.1	404	Bacteria>Proteobacteria>Gammaproteobacteria>Enterobacterales	Enterobacteriaceae	MULTISPECIES:-DNA-phosphorothioation-associated-DGQHR-protein-1-[Enterobacteriaceae]	543
WP_000179013.1	DndB-ParB-nuclease	GCF_000167815.1	361	Bacteria>Proteobacteria>Gammaproteobacteria>Enterobacterales	Enterobacteriaceae	MULTISPECIES:-DNA-sulfur-modification-protein-DndB-[Enterobacteriaceae]	543
WP_000179014.1	DndB-ParB-nuclease	GCF_000026245.1	361	Bacteria>Proteobacteria>Gammaproteobacteria>Enterobacterales	Enterobacteriaceae	MULTISPECIES:-DNA-sulfur-modification-protein-DndB-[Enterobacteriaceae]	543
WP_021547720.1	DndB-ParB-nuclease	GCF_000503275.1	362	Bacteria>Proteobacteria>Gammaproteobacteria>Enterobacterales	Enterobacteriaceae	MULTISPECIES:-DNA-sulfur-modification-protein-DndB-[Enterobacteriaceae]	543
WP_023247626.1	DndB-ParB-nuclease	GCF_000487635.2	359	Bacteria>Proteobacteria>Gammaproteobacteria>Enterobacterales	Enterobacteriaceae	MULTISPECIES:-DNA-sulfur-modification-protein-DndB-[Enterobacteriaceae]	543
WP_024156438.1	DndB-ParB-nuclease	GCF_000416295.1	361	Bacteria>Proteobacteria>Gammaproteobacteria>Enterobacterales	Enterobacteriaceae	MULTISPECIES:-DNA-sulfur-modification-protein-DndB-[Enterobacteriaceae]	543
WP_029591344.1	DndB-ParB-nuclease	GCF_000463135.2	361	Bacteria>Proteobacteria>Gammaproteobacteria>Enterobacterales	Enterobacteriaceae	MULTISPECIES:-DNA-sulfur-modification-protein-DndB-[Enterobacteriaceae]	543
WP_039055620.1	DndB-ParB-nuclease	GCF_019880265.1	361	Bacteria>Proteobacteria>Gammaproteobacteria>Enterobacterales	Enterobacteriaceae	MULTISPECIES:-DNA-sulfur-modification-protein-DndB-[Enterobacteriaceae]	543
WP_047737533.1	DndB-ParB-nuclease	GCF_014289415.1	361	Bacteria>Proteobacteria>Gammaproteobacteria>Enterobacterales	Enterobacteriaceae	MULTISPECIES:-DNA-sulfur-modification-protein-DndB-[Enterobacteriaceae]	543
WP_076736143.1	DndB-ParB-nuclease	GCF_900774715.1	361	Bacteria>Proteobacteria>Gammaproteobacteria>Enterobacterales	Enterobacteriaceae	MULTISPECIES:-DNA-sulfur-modification-protein-DndB-[Enterobacteriaceae]	543
WP_080165625.1	DndB-ParB-nuclease	GCF_005381925.1	361	Bacteria>Proteobacteria>Gammaproteobacteria>Enterobacterales	Enterobacteriaceae	MULTISPECIES:-DNA-sulfur-modification-protein-DndB-[Enterobacteriaceae]	543
WP_109187677.1	DndB-ParB-nuclease	GCF_003590695.1	361	Bacteria>Proteobacteria>Gammaproteobacteria>Enterobacterales	Enterobacteriaceae	MULTISPECIES:-DNA-sulfur-modification-protein-DndB-[Enterobacteriaceae]	543
WP_112026128.1	DndB-ParB-nuclease	GCF_020422365.1	361	Bacteria>Proteobacteria>Gammaproteobacteria>Enterobacterales	Enterobacteriaceae	MULTISPECIES:-DNA-sulfur-modification-protein-DndB-[Enterobacteriaceae]	543
WP_172870802.1	DndB-ParB-nuclease	GCF_013266925.1	361	Bacteria>Proteobacteria>Gammaproteobacteria>Enterobacterales	Enterobacteriaceae	MULTISPECIES:-DNA-sulfur-modification-protein-DndB-[Enterobacteriaceae]	543
WP_000071509.1	DndB-ParB-nuclease	GCF_000026245.1	101	Bacteria>Proteobacteria>Gammaproteobacteria>Enterobacterales	Enterobacteriaceae	MULTISPECIES:-hypothetical-protein-[Enterobacteriaceae]	543
WP_000881801.1	DndB-ParB-nuclease	GCF_018344995.1	197	Bacteria>Proteobacteria>Gammaproteobacteria>Enterobacterales	Enterobacteriaceae	MULTISPECIES:-hypothetical-protein-[Enterobacteriaceae]	543
WP_152107861.1	DndB-ParB-nuclease	GCF_020991025.1	360	Bacteria>Proteobacteria>Gammaproteobacteria>Enterobacterales>Enterobacteriaceae	Plesiomonas_shigelloides	DNA-sulfur-modification-protein-DndB-[Plesiomonas-shigelloides]	703
WP_152136046.1	DndB-ParB-nuclease	GCF_009183385.1	360	Bacteria>Proteobacteria>Gammaproteobacteria>Enterobacterales>Enterobacteriaceae	Plesiomonas_shigelloides	DNA-sulfur-modification-protein-DndB-[Plesiomonas-shigelloides]	703
WP_237201865.1	DndB-ParB-nuclease	GCF_021698685.1	361	Bacteria>Proteobacteria>Gammaproteobacteria>Enterobacterales>Enterobacteriaceae	Raoultella_ornithinolytica	DNA-sulfur-modification-protein-DndB-[Raoultella-ornithinolytica]	54291
WP_150341503.1	DndB-ParB-nuclease	GCF_008694045.1	361	Bacteria>Proteobacteria>Gammaproteobacteria>Enterobacterales>Enterobacteriaceae	Raoultella_planticola	DNA-sulfur-modification-protein-DndB-[Raoultella-planticola]	575
WP_115193693.1	DndB-ParB-nuclease	GCF_900476485.1	567	Bacteria>Proteobacteria>Gammaproteobacteria>Enterobacterales>Enterobacteriaceae	Raoultella_terrigena	AIPR-family-protein-[Raoultella-terrigena]	577
NWK89646.1	DndB-ParB-nuclease	GCA_013393135.1	441	Bacteria>Proteobacteria>Gammaproteobacteria>Enterobacterales>Enterobacteriaceae	Raoultella_terrigena	DGQHR-domain-containing-protein-[Raoultella-terrigena]	577
WP_178996073.1	DndB-ParB-nuclease	GCF_013393135.1	406	Bacteria>Proteobacteria>Gammaproteobacteria>Enterobacterales>Enterobacteriaceae	Raoultella_terrigena	DGQHR-domain-containing-protein-[Raoultella-terrigena]	577
WP_237376790.1	DndB-ParB-nuclease	-	386	Bacteria>Proteobacteria>Gammaproteobacteria>Enterobacterales>Enterobacteriaceae	Raoultella_terrigena	DNA-phosphorothioation-associated-DGQHR-protein-1-[Raoultella-terrigena]	577
NWK89642.1	DndB-ParB-nuclease	GCA_013393135.1	361	Bacteria>Proteobacteria>Gammaproteobacteria>Enterobacterales>Enterobacteriaceae	Raoultella_terrigena	DNA-sulfur-modification-protein-DndB-[Raoultella-terrigena]	577
WP_115193689.1	DndB-ParB-nuclease	GCF_900476485.1	361	Bacteria>Proteobacteria>Gammaproteobacteria>Enterobacterales>Enterobacteriaceae	Raoultella_terrigena	DNA-sulfur-modification-protein-DndB-[Raoultella-terrigena]	577
WP_237376794.1	DndB-ParB-nuclease	-	361	Bacteria>Proteobacteria>Gammaproteobacteria>Enterobacterales>Enterobacteriaceae	Raoultella_terrigena	DNA-sulfur-modification-protein-DndB-[Raoultella-terrigena]	577
WP_079777335.1	DndB-ParB-nuclease	GCF_002037625.1	383	Bacteria>Proteobacteria>Gammaproteobacteria>Enterobacterales>Enterobacteriaceae	Salmonella_bongori	DGQHR-domain-containing-protein-[Salmonella-bongori]	54736
WP_079777327.1	DndB-ParB-nuclease	GCF_002037625.1	361	Bacteria>Proteobacteria>Gammaproteobacteria>Enterobacterales>Enterobacteriaceae	Salmonella_bongori	DNA-sulfur-modification-protein-DndB-[Salmonella-bongori]	54736
EAA9524189.1	DndB-ParB-nuclease	GCA_004227105.1	397	Bacteria>Proteobacteria>Gammaproteobacteria>Enterobacterales>Enterobacteriaceae	Salmonella_enterica	DGQHR-domain-containing-protein-[Salmonella-enterica]	28901
EAA9524196.1	DndB-ParB-nuclease	GCA_004227105.1	379	Bacteria>Proteobacteria>Gammaproteobacteria>Enterobacterales>Enterobacteriaceae	Salmonella_enterica	DGQHR-domain-containing-protein-[Salmonella-enterica]	28901
EAB4413790.1	DndB-ParB-nuclease	GCA_004247645.1	329	Bacteria>Proteobacteria>Gammaproteobacteria>Enterobacterales>Enterobacteriaceae	Salmonella_enterica	DGQHR-domain-containing-protein-[Salmonella-enterica]	28901
EAQ4037399.1	DndB-ParB-nuclease	GCA_005597995.1	395	Bacteria>Proteobacteria>Gammaproteobacteria>Enterobacterales>Enterobacteriaceae	Salmonella_enterica	DGQHR-domain-containing-protein-[Salmonella-enterica]	28901
EAQ6703721.1	DndB-ParB-nuclease	GCA_005609615.1	397	Bacteria>Proteobacteria>Gammaproteobacteria>Enterobacterales>Enterobacteriaceae	Salmonella_enterica	DGQHR-domain-containing-protein-[Salmonella-enterica]	28901
EAS1730122.1	DndB-ParB-nuclease	GCA_005676015.1	394	Bacteria>Proteobacteria>Gammaproteobacteria>Enterobacterales>Enterobacteriaceae	Salmonella_enterica	DGQHR-domain-containing-protein-[Salmonella-enterica]	28901
EAU0344595.1	DndB-ParB-nuclease	GCA_005757755.1	390	Bacteria>Proteobacteria>Gammaproteobacteria>Enterobacterales>Enterobacteriaceae	Salmonella_enterica	DGQHR-domain-containing-protein-[Salmonella-enterica]	28901
EAV3919473.1	DndB-ParB-nuclease	GCA_005841385.1	397	Bacteria>Proteobacteria>Gammaproteobacteria>Enterobacterales>Enterobacteriaceae	Salmonella_enterica	DGQHR-domain-containing-protein-[Salmonella-enterica]	28901
EAV7067906.1	DndB-ParB-nuclease	GCA_005857185.1	383	Bacteria>Proteobacteria>Gammaproteobacteria>Enterobacterales>Enterobacteriaceae	Salmonella_enterica	DGQHR-domain-containing-protein-[Salmonella-enterica]	28901
EAW0643307.1	DndB-ParB-nuclease	GCA_005896045.1	402	Bacteria>Proteobacteria>Gammaproteobacteria>Enterobacterales>Enterobacteriaceae	Salmonella_enterica	DGQHR-domain-containing-protein-[Salmonella-enterica]	28901
EAW0658841.1	DndB-ParB-nuclease	GCA_005896325.1	395	Bacteria>Proteobacteria>Gammaproteobacteria>Enterobacterales>Enterobacteriaceae	Salmonella_enterica	DGQHR-domain-containing-protein-[Salmonella-enterica]	28901
EAW0681375.1	DndB-ParB-nuclease	GCA_005896365.1	389	Bacteria>Proteobacteria>Gammaproteobacteria>Enterobacterales>Enterobacteriaceae	Salmonella_enterica	DGQHR-domain-containing-protein-[Salmonella-enterica]	28901
EAW7984085.1	DndB-ParB-nuclease	GCA_005933995.1	383	Bacteria>Proteobacteria>Gammaproteobacteria>Enterobacterales>Enterobacteriaceae	Salmonella_enterica	DGQHR-domain-containing-protein-[Salmonella-enterica]	28901
EAY5892102.1	DndB-ParB-nuclease	GCA_006014415.1	385	Bacteria>Proteobacteria>Gammaproteobacteria>Enterobacterales>Enterobacteriaceae	Salmonella_enterica	DGQHR-domain-containing-protein-[Salmonella-enterica]	28901
EBD0574544.1	DndB-ParB-nuclease	GCA_006215975.1	228	Bacteria>Proteobacteria>Gammaproteobacteria>Enterobacterales>Enterobacteriaceae	Salmonella_enterica	DGQHR-domain-containing-protein-[Salmonella-enterica]	28901
EBG1795906.1	DndB-ParB-nuclease	GCA_006411595.1	395	Bacteria>Proteobacteria>Gammaproteobacteria>Enterobacterales>Enterobacteriaceae	Salmonella_enterica	DGQHR-domain-containing-protein-[Salmonella-enterica]	28901
EBI3283594.1	DndB-ParB-nuclease	GCA_006550975.1	254	Bacteria>Proteobacteria>Gammaproteobacteria>Enterobacterales>Enterobacteriaceae	Salmonella_enterica	DGQHR-domain-containing-protein-[Salmonella-enterica]	28901
EBM3223040.1	DndB-ParB-nuclease	GCA_006725985.1	385	Bacteria>Proteobacteria>Gammaproteobacteria>Enterobacterales>Enterobacteriaceae	Salmonella_enterica	DGQHR-domain-containing-protein-[Salmonella-enterica]	28901
EBP0009265.1	DndB-ParB-nuclease	GCA_006844205.1	390	Bacteria>Proteobacteria>Gammaproteobacteria>Enterobacterales>Enterobacteriaceae	Salmonella_enterica	DGQHR-domain-containing-protein-[Salmonella-enterica]	28901
EBP7621290.1	DndB-ParB-nuclease	GCA_006881245.1	399	Bacteria>Proteobacteria>Gammaproteobacteria>Enterobacterales>Enterobacteriaceae	Salmonella_enterica	DGQHR-domain-containing-protein-[Salmonella-enterica]	28901
EDA0833653.1	DndB-ParB-nuclease	GCA_008841485.1	389	Bacteria>Proteobacteria>Gammaproteobacteria>Enterobacterales>Enterobacteriaceae	Salmonella_enterica	DGQHR-domain-containing-protein-[Salmonella-enterica]	28901
EGO1272725.1	DndB-ParB-nuclease	GCA_015250075.1	385	Bacteria>Proteobacteria>Gammaproteobacteria>Enterobacterales>Enterobacteriaceae	Salmonella_enterica	DGQHR-domain-containing-protein-[Salmonella-enterica]	28901
EHL1983409.1	DndB-ParB-nuclease	GCA_018099545.1	385	Bacteria>Proteobacteria>Gammaproteobacteria>Enterobacterales>Enterobacteriaceae	Salmonella_enterica	DGQHR-domain-containing-protein-[Salmonella-enterica]	28901
EHM6377487.1	DndB-ParB-nuclease	GCA_018276205.1	402	Bacteria>Proteobacteria>Gammaproteobacteria>Enterobacterales>Enterobacteriaceae	Salmonella_enterica	DGQHR-domain-containing-protein-[Salmonella-enterica]	28901
EHZ9859102.1	DndB-ParB-nuclease	GCA_019744335.1	390	Bacteria>Proteobacteria>Gammaproteobacteria>Enterobacterales>Enterobacteriaceae	Salmonella_enterica	DGQHR-domain-containing-protein-[Salmonella-enterica]	28901
EIE0303120.1	DndB-ParB-nuclease	GCA_020115235.1	415	Bacteria>Proteobacteria>Gammaproteobacteria>Enterobacterales>Enterobacteriaceae	Salmonella_enterica	DGQHR-domain-containing-protein-[Salmonella-enterica]	28901
EIG9525152.1	DndB-ParB-nuclease	GCA_020337775.1	379	Bacteria>Proteobacteria>Gammaproteobacteria>Enterobacterales>Enterobacteriaceae	Salmonella_enterica	DGQHR-domain-containing-protein-[Salmonella-enterica]	28901
HAF5788824.1	DndB-ParB-nuclease	GCA_011338685.1	402	Bacteria>Proteobacteria>Gammaproteobacteria>Enterobacterales>Enterobacteriaceae	Salmonella_enterica	DGQHR-domain-containing-protein-[Salmonella-enterica]	28901
MBA3146872.1	DndB-ParB-nuclease	GCA_007019015.2	379	Bacteria>Proteobacteria>Gammaproteobacteria>Enterobacterales>Enterobacteriaceae	Salmonella_enterica	DGQHR-domain-containing-protein-[Salmonella-enterica]	28901

Accession	Annotation	GCA	Length	Phyletic.spread	Strain.name	Defline	Species.Taxid
MBK0361565.1	DndB-ParB-nuclease	GCA_016518785.1	397	Bacteria>Proteobacteria>Gammaproteobacteria>Enterobacterales>Enterobacteriaceae	Salmonella_enterica	DGQHR-domain-containing-protein-[Salmonella-enterica]	28901
WP_052940714.1	DndB-ParB-nuclease	GCF_943114325.1	397	Bacteria>Proteobacteria>Gammaproteobacteria>Enterobacterales>Enterobacteriaceae	Salmonella_enterica	DGQHR-domain-containing-protein-[Salmonella-enterica]	28901
WP_053529580.1	DndB-ParB-nuclease	GCF_001276875.1	397	Bacteria>Proteobacteria>Gammaproteobacteria>Enterobacterales>Enterobacteriaceae	Salmonella_enterica	DGQHR-domain-containing-protein-[Salmonella-enterica]	28901
WP_058145319.1	DndB-ParB-nuclease	GCF_002109255.1	329	Bacteria>Proteobacteria>Gammaproteobacteria>Enterobacterales>Enterobacteriaceae	Salmonella_enterica	DGQHR-domain-containing-protein-[Salmonella-enterica]	28901
WP_079973178.1	DndB-ParB-nuclease	GCF_002046215.1	379	Bacteria>Proteobacteria>Gammaproteobacteria>Enterobacterales>Enterobacteriaceae	Salmonella_enterica	DGQHR-domain-containing-protein-[Salmonella-enterica]	28901
WP_080165622.1	DndB-ParB-nuclease	GCF_002064055.1	390	Bacteria>Proteobacteria>Gammaproteobacteria>Enterobacterales>Enterobacteriaceae	Salmonella_enterica	DGQHR-domain-containing-protein-[Salmonella-enterica]	28901
WP_136058519.1	DndB-ParB-nuclease	GCF_016030095.1	383	Bacteria>Proteobacteria>Gammaproteobacteria>Enterobacterales>Enterobacteriaceae	Salmonella_enterica	DGQHR-domain-containing-protein-[Salmonella-enterica]	28901
WP_171804769.1	DndB-ParB-nuclease	GCF_013162165.1	397	Bacteria>Proteobacteria>Gammaproteobacteria>Enterobacterales>Enterobacteriaceae	Salmonella_enterica	DGQHR-domain-containing-protein-[Salmonella-enterica]	28901
WP_183059467.1	DndB-ParB-nuclease	GCF_903930865.1	386	Bacteria>Proteobacteria>Gammaproteobacteria>Enterobacterales>Enterobacteriaceae	Salmonella_enterica	DNA-phosphorothioation-associated-DGQHR-protein-1-[Salmonella-enterica]	28901
EAA4948316.1	DndB-ParB-nuclease	GCA_004182315.1	361	Bacteria>Proteobacteria>Gammaproteobacteria>Enterobacterales>Enterobacteriaceae	Salmonella_enterica	DNA-sulfur-modification-protein-DndB-[Salmonella-enterica]	28901
EAA7241613.1	DndB-ParB-nuclease	GCA_004191175.1	361	Bacteria>Proteobacteria>Gammaproteobacteria>Enterobacterales>Enterobacteriaceae	Salmonella_enterica	DNA-sulfur-modification-protein-DndB-[Salmonella-enterica]	28901
EAA7712598.1	DndB-ParB-nuclease	GCA_004218895.1	361	Bacteria>Proteobacteria>Gammaproteobacteria>Enterobacterales>Enterobacteriaceae	Salmonella_enterica	DNA-sulfur-modification-protein-DndB-[Salmonella-enterica]	28901
EAA8284791.1	DndB-ParB-nuclease	GCA_004221485.1	361	Bacteria>Proteobacteria>Gammaproteobacteria>Enterobacterales>Enterobacteriaceae	Salmonella_enterica	DNA-sulfur-modification-protein-DndB-[Salmonella-enterica]	28901
EAA8649689.1	DndB-ParB-nuclease	GCA_004222485.1	361	Bacteria>Proteobacteria>Gammaproteobacteria>Enterobacterales>Enterobacteriaceae	Salmonella_enterica	DNA-sulfur-modification-protein-DndB-[Salmonella-enterica]	28901
EAA9524184.1	DndB-ParB-nuclease	GCA_004227105.1	361	Bacteria>Proteobacteria>Gammaproteobacteria>Enterobacterales>Enterobacteriaceae	Salmonella_enterica	DNA-sulfur-modification-protein-DndB-[Salmonella-enterica]	28901
EAB1976261.1	DndB-ParB-nuclease	GCA_004235745.1	361	Bacteria>Proteobacteria>Gammaproteobacteria>Enterobacterales>Enterobacteriaceae	Salmonella_enterica	DNA-sulfur-modification-protein-DndB-[Salmonella-enterica]	28901
EAB2881942.1	DndB-ParB-nuclease	GCA_004240805.1	361	Bacteria>Proteobacteria>Gammaproteobacteria>Enterobacterales>Enterobacteriaceae	Salmonella_enterica	DNA-sulfur-modification-protein-DndB-[Salmonella-enterica]	28901
EAB4413792.1	DndB-ParB-nuclease	GCA_004247645.1	359	Bacteria>Proteobacteria>Gammaproteobacteria>Enterobacterales>Enterobacteriaceae	Salmonella_enterica	DNA-sulfur-modification-protein-DndB-[Salmonella-enterica]	28901
EAM3348687.1	DndB-ParB-nuclease	GCA_005412355.1	361	Bacteria>Proteobacteria>Gammaproteobacteria>Enterobacterales>Enterobacteriaceae	Salmonella_enterica	DNA-sulfur-modification-protein-DndB-[Salmonella-enterica]	28901
EAM4031411.1	DndB-ParB-nuclease	GCA_005416545.1	361	Bacteria>Proteobacteria>Gammaproteobacteria>Enterobacterales>Enterobacteriaceae	Salmonella_enterica	DNA-sulfur-modification-protein-DndB-[Salmonella-enterica]	28901
EAM4691032.1	DndB-ParB-nuclease	GCA_005418355.1	361	Bacteria>Proteobacteria>Gammaproteobacteria>Enterobacterales>Enterobacteriaceae	Salmonella_enterica	DNA-sulfur-modification-protein-DndB-[Salmonella-enterica]	28901
EAM6170501.1	DndB-ParB-nuclease	GCA_005426355.1	361	Bacteria>Proteobacteria>Gammaproteobacteria>Enterobacterales>Enterobacteriaceae	Salmonella_enterica	DNA-sulfur-modification-protein-DndB-[Salmonella-enterica]	28901
EAM7300443.1	DndB-ParB-nuclease	GCA_005430615.1	361	Bacteria>Proteobacteria>Gammaproteobacteria>Enterobacterales>Enterobacteriaceae	Salmonella_enterica	DNA-sulfur-modification-protein-DndB-[Salmonella-enterica]	28901
EAM7491252.1	DndB-ParB-nuclease	GCA_005430935.1	361	Bacteria>Proteobacteria>Gammaproteobacteria>Enterobacterales>Enterobacteriaceae	Salmonella_enterica	DNA-sulfur-modification-protein-DndB-[Salmonella-enterica]	28901
EAN0729365.1	DndB-ParB-nuclease	GCA_005444665.1	361	Bacteria>Proteobacteria>Gammaproteobacteria>Enterobacterales>Enterobacteriaceae	Salmonella_enterica	DNA-sulfur-modification-protein-DndB-[Salmonella-enterica]	28901
EAN9994417.1	DndB-ParB-nuclease	GCA_005484135.1	361	Bacteria>Proteobacteria>Gammaproteobacteria>Enterobacterales>Enterobacteriaceae	Salmonella_enterica	DNA-sulfur-modification-protein-DndB-[Salmonella-enterica]	28901
EAO1643230.1	DndB-ParB-nuclease	GCA_005491125.1	361	Bacteria>Proteobacteria>Gammaproteobacteria>Enterobacterales>Enterobacteriaceae	Salmonella_enterica	DNA-sulfur-modification-protein-DndB-[Salmonella-enterica]	28901
EAO3463024.1	DndB-ParB-nuclease	GCA_005499015.1	361	Bacteria>Proteobacteria>Gammaproteobacteria>Enterobacterales>Enterobacteriaceae	Salmonella_enterica	DNA-sulfur-modification-protein-DndB-[Salmonella-enterica]	28901
EAO4598519.1	DndB-ParB-nuclease	GCA_005506635.1	361	Bacteria>Proteobacteria>Gammaproteobacteria>Enterobacterales>Enterobacteriaceae	Salmonella_enterica	DNA-sulfur-modification-protein-DndB-[Salmonella-enterica]	28901
EAO4966056.1	DndB-ParB-nuclease	GCA_005508575.1	361	Bacteria>Proteobacteria>Gammaproteobacteria>Enterobacterales>Enterobacteriaceae	Salmonella_enterica	DNA-sulfur-modification-protein-DndB-[Salmonella-enterica]	28901
EAO5562252.1	DndB-ParB-nuclease	GCA_005511335.1	361	Bacteria>Proteobacteria>Gammaproteobacteria>Enterobacterales>Enterobacteriaceae	Salmonella_enterica	DNA-sulfur-modification-protein-DndB-[Salmonella-enterica]	28901
EAO9627569.1	DndB-ParB-nuclease	GCA_005533535.1	359	Bacteria>Proteobacteria>Gammaproteobacteria>Enterobacterales>Enterobacteriaceae	Salmonella_enterica	DNA-sulfur-modification-protein-DndB-[Salmonella-enterica]	28901
EAP0376358.1	DndB-ParB-nuclease	GCA_005536955.1	361	Bacteria>Proteobacteria>Gammaproteobacteria>Enterobacterales>Enterobacteriaceae	Salmonella_enterica	DNA-sulfur-modification-protein-DndB-[Salmonella-enterica]	28901
EAP1447713.1	DndB-ParB-nuclease	GCA_005540095.1	361	Bacteria>Proteobacteria>Gammaproteobacteria>Enterobacterales>Enterobacteriaceae	Salmonella_enterica	DNA-sulfur-modification-protein-DndB-[Salmonella-enterica]	28901
EAP6139549.1	DndB-ParB-nuclease	GCA_005561835.1	361	Bacteria>Proteobacteria>Gammaproteobacteria>Enterobacterales>Enterobacteriaceae	Salmonella_enterica	DNA-sulfur-modification-protein-DndB-[Salmonella-enterica]	28901
EAP6887537.1	DndB-ParB-nuclease	GCA_005565455.1	361	Bacteria>Proteobacteria>Gammaproteobacteria>Enterobacterales>Enterobacteriaceae	Salmonella_enterica	DNA-sulfur-modification-protein-DndB-[Salmonella-enterica]	28901
EAP9726968.1	DndB-ParB-nuclease	GCA_005579375.1	361	Bacteria>Proteobacteria>Gammaproteobacteria>Enterobacterales>Enterobacteriaceae	Salmonella_enterica	DNA-sulfur-modification-protein-DndB-[Salmonella-enterica]	28901
EAQ4037403.1	DndB-ParB-nuclease	GCA_005597995.1	361	Bacteria>Proteobacteria>Gammaproteobacteria>Enterobacterales>Enterobacteriaceae	Salmonella_enterica	DNA-sulfur-modification-protein-DndB-[Salmonella-enterica]	28901
EAQ4923873.1	DndB-ParB-nuclease	GCA_005601835.1	361	Bacteria>Proteobacteria>Gammaproteobacteria>Enterobacterales>Enterobacteriaceae	Salmonella_enterica	DNA-sulfur-modification-protein-DndB-[Salmonella-enterica]	28901
EAQ6703716.1	DndB-ParB-nuclease	GCA_005609615.1	361	Bacteria>Proteobacteria>Gammaproteobacteria>Enterobacterales>Enterobacteriaceae	Salmonella_enterica	DNA-sulfur-modification-protein-DndB-[Salmonella-enterica]	28901
EAR5189484.1	DndB-ParB-nuclease	GCA_005650395.1	361	Bacteria>Proteobacteria>Gammaproteobacteria>Enterobacterales>Enterobacteriaceae	Salmonella_enterica	DNA-sulfur-modification-protein-DndB-[Salmonella-enterica]	28901
EAR9540358.1	DndB-ParB-nuclease	GCA_005670455.1	361	Bacteria>Proteobacteria>Gammaproteobacteria>Enterobacterales>Enterobacteriaceae	Salmonella_enterica	DNA-sulfur-modification-protein-DndB-[Salmonella-enterica]	28901
EAS1314890.1	DndB-ParB-nuclease	GCA_005674115.1	361	Bacteria>Proteobacteria>Gammaproteobacteria>Enterobacterales>Enterobacteriaceae	Salmonella_enterica	DNA-sulfur-modification-protein-DndB-[Salmonella-enterica]	28901
EAS1730126.1	DndB-ParB-nuclease	GCA_005676015.1	361	Bacteria>Proteobacteria>Gammaproteobacteria>Enterobacterales>Enterobacteriaceae	Salmonella_enterica	DNA-sulfur-modification-protein-DndB-[Salmonella-enterica]	28901
EAS5420496.1	DndB-ParB-nuclease	GCA_005692095.1	361	Bacteria>Proteobacteria>Gammaproteobacteria>Enterobacterales>Enterobacteriaceae	Salmonella_enterica	DNA-sulfur-modification-protein-DndB-[Salmonella-enterica]	28901
EAS5970448.1	DndB-ParB-nuclease	GCA_005695535.1	361	Bacteria>Proteobacteria>Gammaproteobacteria>Enterobacterales>Enterobacteriaceae	Salmonella_enterica	DNA-sulfur-modification-protein-DndB-[Salmonella-enterica]	28901
EAT3915312.1	DndB-ParB-nuclease	GCA_005729775.1	361	Bacteria>Proteobacteria>Gammaproteobacteria>Enterobacterales>Enterobacteriaceae	Salmonella_enterica	DNA-sulfur-modification-protein-DndB-[Salmonella-enterica]	28901
EAT3923993.1	DndB-ParB-nuclease	GCA_005729615.1	361	Bacteria>Proteobacteria>Gammaproteobacteria>Enterobacterales>Enterobacteriaceae	Salmonella_enterica	DNA-sulfur-modification-protein-DndB-[Salmonella-enterica]	28901
EAU0222766.1	DndB-ParB-nuclease	GCA_005756735.1	361	Bacteria>Proteobacteria>Gammaproteobacteria>Enterobacterales>Enterobacteriaceae	Salmonella_enterica	DNA-sulfur-modification-protein-DndB-[Salmonella-enterica]	28901
EAU0344591.1	DndB-ParB-nuclease	GCA_005757755.1	361	Bacteria>Proteobacteria>Gammaproteobacteria>Enterobacterales>Enterobacteriaceae	Salmonella_enterica	DNA-sulfur-modification-protein-DndB-[Salmonella-enterica]	28901
EAU0668412.1	DndB-ParB-nuclease	GCA_005758915.1	361	Bacteria>Proteobacteria>Gammaproteobacteria>Enterobacterales>Enterobacteriaceae	Salmonella_enterica	DNA-sulfur-modification-protein-DndB-[Salmonella-enterica]	28901
EAU4054786.1	DndB-ParB-nuclease	GCA_005775085.1	361	Bacteria>Proteobacteria>Gammaproteobacteria>Enterobacterales>Enterobacteriaceae	Salmonella_enterica	DNA-sulfur-modification-protein-DndB-[Salmonella-enterica]	28901
EAU5653681.1	DndB-ParB-nuclease	GCA_005797685.1	361	Bacteria>Proteobacteria>Gammaproteobacteria>Enterobacterales>Enterobacteriaceae	Salmonella_enterica	DNA-sulfur-modification-protein-DndB-[Salmonella-enterica]	28901
EAU6665545.1	DndB-ParB-nuclease	GCA_005807005.1	361	Bacteria>Proteobacteria>Gammaproteobacteria>Enterobacterales>Enterobacteriaceae	Salmonella_enterica	DNA-sulfur-modification-protein-DndB-[Salmonella-enterica]	28901
EAV1253571.1	DndB-ParB-nuclease	GCA_005829925.1	361	Bacteria>Proteobacteria>Gammaproteobacteria>Enterobacterales>Enterobacteriaceae	Salmonella_enterica	DNA-sulfur-modification-protein-DndB-[Salmonella-enterica]	28901
EAV1885547.1	DndB-ParB-nuclease	GCA_005832445.1	361	Bacteria>Proteobacteria>Gammaproteobacteria>Enterobacterales>Enterobacteriaceae	Salmonella_enterica	DNA-sulfur-modification-protein-DndB-[Salmonella-enterica]	28901
EAV3919477.1	DndB-ParB-nuclease	GCA_005841385.1	361	Bacteria>Proteobacteria>Gammaproteobacteria>Enterobacterales>Enterobacteriaceae	Salmonella_enterica	DNA-sulfur-modification-protein-DndB-[Salmonella-enterica]	28901
EAV7067910.1	DndB-ParB-nuclease	GCA_005857185.1	361	Bacteria>Proteobacteria>Gammaproteobacteria>Enterobacterales>Enterobacteriaceae	Salmonella_enterica	DNA-sulfur-modification-protein-DndB-[Salmonella-enterica]	28901
EAW0051565.1	DndB-ParB-nuclease	GCA_005893465.1	361	Bacteria>Proteobacteria>Gammaproteobacteria>Enterobacterales>Enterobacteriaceae	Salmonella_enterica	DNA-sulfur-modification-protein-DndB-[Salmonella-enterica]	28901
EAW0643303.1	DndB-ParB-nuclease	GCA_005896045.1	361	Bacteria>Proteobacteria>Gammaproteobacteria>Enterobacterales>Enterobacteriaceae	Salmonella_enterica	DNA-sulfur-modification-protein-DndB-[Salmonella-enterica]	28901
EAW0658837.1	DndB-ParB-nuclease	GCA_005896325.1	361	Bacteria>Proteobacteria>Gammaproteobacteria>Enterobacterales>Enterobacteriaceae	Salmonella_enterica	DNA-sulfur-modification-protein-DndB-[Salmonella-enterica]	28901
EAW0681380.1	DndB-ParB-nuclease	GCA_005896365.1	361	Bacteria>Proteobacteria>Gammaproteobacteria>Enterobacterales>Enterobacteriaceae	Salmonella_enterica	DNA-sulfur-modification-protein-DndB-[Salmonella-enterica]	28901
EAW6002187.1	DndB-ParB-nuclease	GCA_005918065.1	359	Bacteria>Proteobacteria>Gammaproteobacteria>Enterobacterales>Enterobacteriaceae	Salmonella_enterica	DNA-sulfur-modification-protein-DndB-[Salmonella-enterica]	28901
EAW7984089.1	DndB-ParB-nuclease	GCA_005933995.1	361	Bacteria>Proteobacteria>Gammaproteobacteria>Enterobacterales>Enterobacteriaceae	Salmonella_enterica	DNA-sulfur-modification-protein-DndB-[Salmonella-enterica]	28901
EAX4976606.1	DndB-ParB-nuclease	GCA_005964985.1	361	Bacteria>Proteobacteria>Gammaproteobacteria>Enterobacterales>Enterobacteriaceae	Salmonella_enterica	DNA-sulfur-modification-protein-DndB-[Salmonella-enterica]	28901
EAX8475245.1	DndB-ParB-nuclease	GCA_005980785.1	361	Bacteria>Proteobacteria>Gammaproteobacteria>Enterobacterales>Enterobacteriaceae	Salmonella_enterica	DNA-sulfur-modification-protein-DndB-[Salmonella-enterica]	28901
EAX9948343.1	DndB-ParB-nuclease	GCA_005987665.1	361	Bacteria>Proteobacteria>Gammaproteobacteria>Enterobacterales>Enterobacteriaceae	Salmonella_enterica	DNA-sulfur-modification-protein-DndB-[Salmonella-enterica]	28901
EAY5892096.1	DndB-ParB-nuclease	GCA_006014415.1	361	Bacteria>Proteobacteria>Gammaproteobacteria>Enterobacterales>Enterobacteriaceae	Salmonella_enterica	DNA-sulfur-modification-protein-DndB-[Salmonella-enterica]	28901
EAZ4759134.1	DndB-ParB-nuclease	GCA_006051715.1	361	Bacteria>Proteobacteria>Gammaproteobacteria>Enterobacterales>Enterobacteriaceae	Salmonella_enterica	DNA-sulfur-modification-protein-DndB-[Salmonella-enterica]	28901
EAZ7857500.1	DndB-ParB-nuclease	GCA_006066115.1	345	Bacteria>Proteobacteria>Gammaproteobacteria>Enterobacterales>Enterobacteriaceae	Salmonella_enterica	DNA-sulfur-modification-protein-DndB-[Salmonella-enterica]	28901
EBA4497956.1	DndB-ParB-nuclease	GCA_006097275.1	361	Bacteria>Proteobacteria>Gammaproteobacteria>Enterobacterales>Enterobacteriaceae	Salmonella_enterica	DNA-sulfur-modification-protein-DndB-[Salmonella-enterica]	28901
EBB0845090.1	DndB-ParB-nuclease	GCA_006125365.1	361	Bacteria>Proteobacteria>Gammaproteobacteria>Enterobacterales>Enterobacteriaceae	Salmonella_enterica	DNA-sulfur-modification-protein-DndB-[Salmonella-enterica]	28901
EBB5938922.1	DndB-ParB-nuclease	GCA_006151465.1	361	Bacteria>Proteobacteria>Gammaproteobacteria>Enterobacterales>Enterobacteriaceae	Salmonella_enterica	DNA-sulfur-modification-protein-DndB-[Salmonella-enterica]	28901
EBC1275293.1	DndB-ParB-nuclease	GCA_006174785.1	361	Bacteria>Proteobacteria>Gammaproteobacteria>Enterobacterales>Enterobacteriaceae	Salmonella_enterica	DNA-sulfur-modification-protein-DndB-[Salmonella-enterica]	28901
EBC1383909.1	DndB-ParB-nuclease	GCA_006175525.1	361	Bacteria>Proteobacteria>Gammaproteobacteria>Enterobacterales>Enterobacteriaceae	Salmonella_enterica	DNA-sulfur-modification-protein-DndB-[Salmonella-enterica]	28901
EBD0574546.1	DndB-ParB-nuclease	GCA_006215975.1	359	Bacteria>Proteobacteria>Gammaproteobacteria>Enterobacterales>Enterobacteriaceae	Salmonella_enterica	DNA-sulfur-modification-protein-DndB-[Salmonella-enterica]	28901
EBE0804199.1	DndB-ParB-nuclease	GCA_006279955.1	361	Bacteria>Proteobacteria>Gammaproteobacteria>Enterobacterales>Enterobacteriaceae	Salmonella_enterica	DNA-sulfur-modification-protein-DndB-[Salmonella-enterica]	28901
EBE6989298.1	DndB-ParB-nuclease	GCA_006307825.1	361	Bacteria>Proteobacteria>Gammaproteobacteria>Enterobacterales>Enterobacteriaceae	Salmonella_enterica	DNA-sulfur-modification-protein-DndB-[Salmonella-enterica]	28901
EBF1481780.1	DndB-ParB-nuclease	GCA_006326425.1	361	Bacteria>Proteobacteria>Gammaproteobacteria>Enterobacterales>Enterobacteriaceae	Salmonella_enterica	DNA-sulfur-modification-protein-DndB-[Salmonella-enterica]	28901
EBG1795902.1	DndB-ParB-nuclease	GCA_006411595.1	361	Bacteria>Proteobacteria>Gammaproteobacteria>Enterobacterales>Enterobacteriaceae	Salmonella_enterica	DNA-sulfur-modification-protein-DndB-[Salmonella-enterica]	28901
EBH0599166.1	DndB-ParB-nuclease	GCA_006476445.1	361	Bacteria>Proteobacteria>Gammaproteobacteria>Enterobacterales>Enterobacteriaceae	Salmonella_enterica	DNA-sulfur-modification-protein-DndB-[Salmonella-enterica]	28901
EBH5933277.1	DndB-ParB-nuclease	GCA_006504075.1	361	Bacteria>Proteobacteria>Gammaproteobacteria>Enterobacterales>Enterobacteriaceae	Salmonella_enterica	DNA-sulfur-modification-protein-DndB-[Salmonella-enterica]	28901
EBI3283596.1	DndB-ParB-nuclease	GCA_006550975.1	359	Bacteria>Proteobacteria>Gammaproteobacteria>Enterobacterales>Enterobacteriaceae	Salmonella_enterica	DNA-sulfur-modification-protein-DndB-[Salmonella-enterica]	28901
EBK4658632.1	DndB-ParB-nuclease	GCA_006645525.1	361	Bacteria>Proteobacteria>Gammaproteobacteria>Enterobacterales>Enterobacteriaceae	Salmonella_enterica	DNA-sulfur-modification-protein-DndB-[Salmonella-enterica]	28901
EBM3223034.1	DndB-ParB-nuclease	GCA_006725985.1	361	Bacteria>Proteobacteria>Gammaproteobacteria>Enterobacterales>Enterobacteriaceae	Salmonella_enterica	DNA-sulfur-modification-protein-DndB-[Salmonella-enterica]	28901
EBN0686389.1	DndB-ParB-nuclease	GCA_006758905.1	361	Bacteria>Proteobacteria>Gammaproteobacteria>Enterobacterales>Enterobacteriaceae	Salmonella_enterica	DNA-sulfur-modification-protein-DndB-[Salmonella-enterica]	28901
EBN3267514.1	DndB-ParB-nuclease	GCA_006768925.1	361	Bacteria>Proteobacteria>Gammaproteobacteria>Enterobacterales>Enterobacteriaceae	Salmonella_enterica	DNA-sulfur-modification-protein-DndB-[Salmonella-enterica]	28901
EBN9070842.1	DndB-ParB-nuclease	GCA_006793285.1	359	Bacteria>Proteobacteria>Gammaproteobacteria>Enterobacterales>Enterobacteriaceae	Salmonella_enterica	DNA-sulfur-modification-protein-DndB-[Salmonella-enterica]	28901
EBP0009270.1	DndB-ParB-nuclease	GCA_006844205.1	361	Bacteria>Proteobacteria>Gammaproteobacteria>Enterobacterales>Enterobacteriaceae	Salmonella_enterica	DNA-sulfur-modification-protein-DndB-[Salmonella-enterica]	28901
EBP0116615.1	DndB-ParB-nuclease	GCA_006844965.1	361	Bacteria>Proteobacteria>Gammaproteobacteria>Enterobacterales>Enterobacteriaceae	Salmonella_enterica	DNA-sulfur-modification-protein-DndB-[Salmonella-enterica]	28901
EBP7621302.1	DndB-ParB-nuclease	GCA_006881245.1	361	Bacteria>Proteobacteria>Gammaproteobacteria>Enterobacterales>Enterobacteriaceae	Salmonella_enterica	DNA-sulfur-modification-protein-DndB-[Salmonella-enterica]	28901
EBR4907779.1	DndB-ParB-nuclease	GCA_006957025.1	361	Bacteria>Proteobacteria>Gammaproteobacteria>Enterobacterales>Enterobacteriaceae	Salmonella_enterica	DNA-sulfur-modification-protein-DndB-[Salmonella-enterica]	28901
EBU3277059.1	DndB-ParB-nuclease	GCA_007098865.1	361	Bacteria>Proteobacteria>Gammaproteobacteria>Enterobacterales>Enterobacteriaceae	Salmonella_enterica	DNA-sulfur-modification-protein-DndB-[Salmonella-enterica]	28901
EBU4991830.1	DndB-ParB-nuclease	GCA_007107225.1	361	Bacteria>Proteobacteria>Gammaproteobacteria>Enterobacterales>Enterobacteriaceae	Salmonella_enterica	DNA-sulfur-modification-protein-DndB-[Salmonella-enterica]	28901
ECE1533898.1	DndB-ParB-nuclease	GCA_007552915.1	361	Bacteria>Proteobacteria>Gammaproteobacteria>Enterobacterales>Enterobacteriaceae	Salmonella_enterica	DNA-sulfur-modification-protein-DndB-[Salmonella-enterica]	28901
ECE4236715.1	DndB-ParB-nuclease	GCA_007570365.1	361	Bacteria>Proteobacteria>Gammaproteobacteria>Enterobacterales>Enterobacteriaceae	Salmonella_enterica	DNA-sulfur-modification-protein-DndB-[Salmonella-enterica]	28901
ECG0222998.1	DndB-ParB-nuclease	GCA_007641135.1	361	Bacteria>Proteobacteria>Gammaproteobacteria>Enterobacterales>Enterobacteriaceae	Salmonella_enterica	DNA-sulfur-modification-protein-DndB-[Salmonella-enterica]	28901
ECG0564357.1	DndB-ParB-nuclease	GCA_007642775.1	359	Bacteria>Proteobacteria>Gammaproteobacteria>Enterobacterales>Enterobacteriaceae	Salmonella_enterica	DNA-sulfur-modification-protein-DndB-[Salmonella-enterica]	28901
ECG8185574.1	DndB-ParB-nuclease	GCA_007692805.1	361	Bacteria>Proteobacteria>Gammaproteobacteria>Enterobacterales>Enterobacteriaceae	Salmonella_enterica	DNA-sulfur-modification-protein-DndB-[Salmonella-enterica]	28901
ECH6019811.1	DndB-ParB-nuclease	GCA_007733445.1	361	Bacteria>Proteobacteria>Gammaproteobacteria>Enterobacterales>Enterobacteriaceae	Salmonella_enterica	DNA-sulfur-modification-protein-DndB-[Salmonella-enterica]	28901
ECP2020502.1	DndB-ParB-nuclease	GCA_008186985.1	361	Bacteria>Proteobacteria>Gammaproteobacteria>Enterobacterales>Enterobacteriaceae	Salmonella_enterica	DNA-sulfur-modification-protein-DndB-[Salmonella-enterica]	28901
ECP2566441.1	DndB-ParB-nuclease	GCA_008189665.1	361	Bacteria>Proteobacteria>Gammaproteobacteria>Enterobacterales>Enterobacteriaceae	Salmonella_enterica	DNA-sulfur-modification-protein-DndB-[Salmonella-enterica]	28901
ECR0283413.1	DndB-ParB-nuclease	GCA_008316735.1	361	Bacteria>Proteobacteria>Gammaproteobacteria>Enterobacterales>Enterobacteriaceae	Salmonella_enterica	DNA-sulfur-modification-protein-DndB-[Salmonella-enterica]	28901
ECS0949467.1	DndB-ParB-nuclease	GCA_008401555.1	361	Bacteria>Proteobacteria>Gammaproteobacteria>Enterobacterales>Enterobacteriaceae	Salmonella_enterica	DNA-sulfur-modification-protein-DndB-[Salmonella-enterica]	28901
ECT9549642.1	DndB-ParB-nuclease	GCA_008488425.1	359	Bacteria>Proteobacteria>Gammaproteobacteria>Enterobacterales>Enterobacteriaceae	Salmonella_enterica	DNA-sulfur-modification-protein-DndB-[Salmonella-enterica]	28901
ECW3768046.1	DndB-ParB-nuclease	GCA_008604485.1	361	Bacteria>Proteobacteria>Gammaproteobacteria>Enterobacterales>Enterobacteriaceae	Salmonella_enterica	DNA-sulfur-modification-protein-DndB-[Salmonella-enterica]	28901
EDA0833647.1	DndB-ParB-nuclease	GCA_008841485.1	361	Bacteria>Proteobacteria>Gammaproteobacteria>Enterobacterales>Enterobacteriaceae	Salmonella_enterica	DNA-sulfur-modification-protein-DndB-[Salmonella-enterica]	28901
EDD2591201.1	DndB-ParB-nuclease	GCA_009011435.1	361	Bacteria>Proteobacteria>Gammaproteobacteria>Enterobacterales>Enterobacteriaceae	Salmonella_enterica	DNA-sulfur-modification-protein-DndB-[Salmonella-enterica]	28901
EDF9554184.1	DndB-ParB-nuclease	GCA_009151635.1	361	Bacteria>Proteobacteria>Gammaproteobacteria>Enterobacterales>Enterobacteriaceae	Salmonella_enterica	DNA-sulfur-modification-protein-DndB-[Salmonella-enterica]	28901
EDH7449021.1	DndB-ParB-nuclease	GCA_009255185.1	361	Bacteria>Proteobacteria>Gammaproteobacteria>Enterobacterales>Enterobacteriaceae	Salmonella_enterica	DNA-sulfur-modification-protein-DndB-[Salmonella-enterica]	28901
EDJ0414621.1	DndB-ParB-nuclease	GCA_009353875.1	361	Bacteria>Proteobacteria>Gammaproteobacteria>Enterobacterales>Enterobacteriaceae	Salmonella_enterica	DNA-sulfur-modification-protein-DndB-[Salmonella-enterica]	28901
EDK0389266.1	DndB-ParB-nuclease	GCA_009413715.1	361	Bacteria>Proteobacteria>Gammaproteobacteria>Enterobacterales>Enterobacteriaceae	Salmonella_enterica	DNA-sulfur-modification-protein-DndB-[Salmonella-enterica]	28901
EDM0764334.1	DndB-ParB-nuclease	GCA_009547755.1	361	Bacteria>Proteobacteria>Gammaproteobacteria>Enterobacterales>Enterobacteriaceae	Salmonella_enterica	DNA-sulfur-modification-protein-DndB-[Salmonella-enterica]	28901
EDS0719949.1	DndB-ParB-nuclease	GCA_010477065.1	361	Bacteria>Proteobacteria>Gammaproteobacteria>Enterobacterales>Enterobacteriaceae	Salmonella_enterica	DNA-sulfur-modification-protein-DndB-[Salmonella-enterica]	28901
EDS3216405.1	DndB-ParB-nuclease	GCA_010488395.1	361	Bacteria>Proteobacteria>Gammaproteobacteria>Enterobacterales>Enterobacteriaceae	Salmonella_enterica	DNA-sulfur-modification-protein-DndB-[Salmonella-enterica]	28901
EDY6736660.1	DndB-ParB-nuclease	GCA_010823125.1	361	Bacteria>Proteobacteria>Gammaproteobacteria>Enterobacterales>Enterobacteriaceae	Salmonella_enterica	DNA-sulfur-modification-protein-DndB-[Salmonella-enterica]	28901
EDZ0567979.1	DndB-ParB-nuclease	GCA_010836285.1	361	Bacteria>Proteobacteria>Gammaproteobacteria>Enterobacterales>Enterobacteriaceae	Salmonella_enterica	DNA-sulfur-modification-protein-DndB-[Salmonella-enterica]	28901
EEA1007304.1	DndB-ParB-nuclease	GCA_010878925.1	361	Bacteria>Proteobacteria>Gammaproteobacteria>Enterobacterales>Enterobacteriaceae	Salmonella_enterica	DNA-sulfur-modification-protein-DndB-[Salmonella-enterica]	28901
EEB3910495.1	DndB-ParB-nuclease	GCA_010941815.1	361	Bacteria>Proteobacteria>Gammaproteobacteria>Enterobacterales>Enterobacteriaceae	Salmonella_enterica	DNA-sulfur-modification-protein-DndB-[Salmonella-enterica]	28901
EEF8220750.1	DndB-ParB-nuclease	GCA_011196315.1	361	Bacteria>Proteobacteria>Gammaproteobacteria>Enterobacterales>Enterobacteriaceae	Salmonella_enterica	DNA-sulfur-modification-protein-DndB-[Salmonella-enterica]	28901
EEG4994863.1	DndB-ParB-nuclease	GCA_011236875.1	361	Bacteria>Proteobacteria>Gammaproteobacteria>Enterobacterales>Enterobacteriaceae	Salmonella_enterica	DNA-sulfur-modification-protein-DndB-[Salmonella-enterica]	28901
EEH7000478.1	DndB-ParB-nuclease	GCA_011299175.1	361	Bacteria>Proteobacteria>Gammaproteobacteria>Enterobacterales>Enterobacteriaceae	Salmonella_enterica	DNA-sulfur-modification-protein-DndB-[Salmonella-enterica]	28901
EEL4302736.1	DndB-ParB-nuclease	GCA_011517565.1	361	Bacteria>Proteobacteria>Gammaproteobacteria>Enterobacterales>Enterobacteriaceae	Salmonella_enterica	DNA-sulfur-modification-protein-DndB-[Salmonella-enterica]	28901
EFR8873742.1	DndB-ParB-nuclease	GCA_013585635.1	361	Bacteria>Proteobacteria>Gammaproteobacteria>Enterobacterales>Enterobacteriaceae	Salmonella_enterica	DNA-sulfur-modification-protein-DndB-[Salmonella-enterica]	28901
EGC8566112.1	DndB-ParB-nuclease	GCA_014315835.1	361	Bacteria>Proteobacteria>Gammaproteobacteria>Enterobacterales>Enterobacteriaceae	Salmonella_enterica	DNA-sulfur-modification-protein-DndB-[Salmonella-enterica]	28901

Accession	Annotation	GCA	Length	Phyletic.spread	Strain.name	Defline	Species.Taxid
EGD2522439.1	DndB-ParB-nuclease	GCA_014358505.1	361	Bacteria>Proteobacteria>Gammaproteobacteria>Enterobacterales>Enterobacteriaceae	Salmonella_enterica	DNA-sulfur-modification-protein-DndB-[Salmonella-enterica]	28901
EGH2142724.1	DndB-ParB-nuclease	GCA_014607895.1	361	Bacteria>Proteobacteria>Gammaproteobacteria>Enterobacterales>Enterobacteriaceae	Salmonella_enterica	DNA-sulfur-modification-protein-DndB-[Salmonella-enterica]	28901
EGS5922484.1	DndB-ParB-nuclease	GCA_015906155.1	361	Bacteria>Proteobacteria>Gammaproteobacteria>Enterobacterales>Enterobacteriaceae	Salmonella_enterica	DNA-sulfur-modification-protein-DndB-[Salmonella-enterica]	28901
EHI4413261.1	DndB-ParB-nuclease	GCA_017803365.1	361	Bacteria>Proteobacteria>Gammaproteobacteria>Enterobacterales>Enterobacteriaceae	Salmonella_enterica	DNA-sulfur-modification-protein-DndB-[Salmonella-enterica]	28901
EHI4488929.1	DndB-ParB-nuclease	GCA_017803615.1	361	Bacteria>Proteobacteria>Gammaproteobacteria>Enterobacterales>Enterobacteriaceae	Salmonella_enterica	DNA-sulfur-modification-protein-DndB-[Salmonella-enterica]	28901
EHJ9075388.1	DndB-ParB-nuclease	GCA_017954935.1	361	Bacteria>Proteobacteria>Gammaproteobacteria>Enterobacterales>Enterobacteriaceae	Salmonella_enterica	DNA-sulfur-modification-protein-DndB-[Salmonella-enterica]	28901
EHJ9264028.1	DndB-ParB-nuclease	GCA_017956535.1	361	Bacteria>Proteobacteria>Gammaproteobacteria>Enterobacterales>Enterobacteriaceae	Salmonella_enterica	DNA-sulfur-modification-protein-DndB-[Salmonella-enterica]	28901
EHL1983405.1	DndB-ParB-nuclease	GCA_018099545.1	361	Bacteria>Proteobacteria>Gammaproteobacteria>Enterobacterales>Enterobacteriaceae	Salmonella_enterica	DNA-sulfur-modification-protein-DndB-[Salmonella-enterica]	28901
EHM6377491.1	DndB-ParB-nuclease	GCA_018276205.1	361	Bacteria>Proteobacteria>Gammaproteobacteria>Enterobacterales>Enterobacteriaceae	Salmonella_enterica	DNA-sulfur-modification-protein-DndB-[Salmonella-enterica]	28901
EHM8751838.1	DndB-ParB-nuclease	GCA_018289695.1	361	Bacteria>Proteobacteria>Gammaproteobacteria>Enterobacterales>Enterobacteriaceae	Salmonella_enterica	DNA-sulfur-modification-protein-DndB-[Salmonella-enterica]	28901
EHO1020053.1	DndB-ParB-nuclease	GCA_018481965.1	361	Bacteria>Proteobacteria>Gammaproteobacteria>Enterobacterales>Enterobacteriaceae	Salmonella_enterica	DNA-sulfur-modification-protein-DndB-[Salmonella-enterica]	28901
EHQ5158492.1	DndB-ParB-nuclease	GCA_018945535.1	359	Bacteria>Proteobacteria>Gammaproteobacteria>Enterobacterales>Enterobacteriaceae	Salmonella_enterica	DNA-sulfur-modification-protein-DndB-[Salmonella-enterica]	28901
EHT6839805.1	DndB-ParB-nuclease	GCA_019241935.1	361	Bacteria>Proteobacteria>Gammaproteobacteria>Enterobacterales>Enterobacteriaceae	Salmonella_enterica	DNA-sulfur-modification-protein-DndB-[Salmonella-enterica]	28901
EHT7457725.1	DndB-ParB-nuclease	GCA_019253065.1	361	Bacteria>Proteobacteria>Gammaproteobacteria>Enterobacterales>Enterobacteriaceae	Salmonella_enterica	DNA-sulfur-modification-protein-DndB-[Salmonella-enterica]	28901
EHU7930404.1	DndB-ParB-nuclease	GCA_019320285.1	361	Bacteria>Proteobacteria>Gammaproteobacteria>Enterobacterales>Enterobacteriaceae	Salmonella_enterica	DNA-sulfur-modification-protein-DndB-[Salmonella-enterica]	28901
EHZ9859107.1	DndB-ParB-nuclease	GCA_019744335.1	361	Bacteria>Proteobacteria>Gammaproteobacteria>Enterobacterales>Enterobacteriaceae	Salmonella_enterica	DNA-sulfur-modification-protein-DndB-[Salmonella-enterica]	28901
EID4215722.1	DndB-ParB-nuclease	GCA_020044685.1	361	Bacteria>Proteobacteria>Gammaproteobacteria>Enterobacterales>Enterobacteriaceae	Salmonella_enterica	DNA-sulfur-modification-protein-DndB-[Salmonella-enterica]	28901
EIE0303125.1	DndB-ParB-nuclease	GCA_020115235.1	361	Bacteria>Proteobacteria>Gammaproteobacteria>Enterobacterales>Enterobacteriaceae	Salmonella_enterica	DNA-sulfur-modification-protein-DndB-[Salmonella-enterica]	28901
EIF6098105.1	DndB-ParB-nuclease	GCA_020265145.1	361	Bacteria>Proteobacteria>Gammaproteobacteria>Enterobacterales>Enterobacteriaceae	Salmonella_enterica	DNA-sulfur-modification-protein-DndB-[Salmonella-enterica]	28901
EIG9525140.1	DndB-ParB-nuclease	GCA_020337775.1	361	Bacteria>Proteobacteria>Gammaproteobacteria>Enterobacterales>Enterobacteriaceae	Salmonella_enterica	DNA-sulfur-modification-protein-DndB-[Salmonella-enterica]	28901
EIJ1959140.1	DndB-ParB-nuclease	GCA_020508645.1	361	Bacteria>Proteobacteria>Gammaproteobacteria>Enterobacterales>Enterobacteriaceae	Salmonella_enterica	DNA-sulfur-modification-protein-DndB-[Salmonella-enterica]	28901
EIK2673946.1	DndB-ParB-nuclease	GCA_020600535.1	361	Bacteria>Proteobacteria>Gammaproteobacteria>Enterobacterales>Enterobacteriaceae	Salmonella_enterica	DNA-sulfur-modification-protein-DndB-[Salmonella-enterica]	28901
EIO8151471.1	DndB-ParB-nuclease	GCA_020999715.1	361	Bacteria>Proteobacteria>Gammaproteobacteria>Enterobacterales>Enterobacteriaceae	Salmonella_enterica	DNA-sulfur-modification-protein-DndB-[Salmonella-enterica]	28901
EIQ0606588.1	DndB-ParB-nuclease	GCA_021148965.1	361	Bacteria>Proteobacteria>Gammaproteobacteria>Enterobacterales>Enterobacteriaceae	Salmonella_enterica	DNA-sulfur-modification-protein-DndB-[Salmonella-enterica]	28901
EIS5503045.1	DndB-ParB-nuclease	GCA_021370795.1	361	Bacteria>Proteobacteria>Gammaproteobacteria>Enterobacterales>Enterobacteriaceae	Salmonella_enterica	DNA-sulfur-modification-protein-DndB-[Salmonella-enterica]	28901
HAF2128881.1	DndB-ParB-nuclease	GCA_011226015.1	359	Bacteria>Proteobacteria>Gammaproteobacteria>Enterobacterales>Enterobacteriaceae	Salmonella_enterica	DNA-sulfur-modification-protein-DndB-[Salmonella-enterica]	28901
HAF5788820.1	DndB-ParB-nuclease	GCA_011338685.1	361	Bacteria>Proteobacteria>Gammaproteobacteria>Enterobacterales>Enterobacteriaceae	Salmonella_enterica	DNA-sulfur-modification-protein-DndB-[Salmonella-enterica]	28901
MBA3146881.1	DndB-ParB-nuclease	GCA_007019015.2	361	Bacteria>Proteobacteria>Gammaproteobacteria>Enterobacterales>Enterobacteriaceae	Salmonella_enterica	DNA-sulfur-modification-protein-DndB-[Salmonella-enterica]	28901
MBK0361560.1	DndB-ParB-nuclease	GCA_016518785.1	361	Bacteria>Proteobacteria>Gammaproteobacteria>Enterobacterales>Enterobacteriaceae	Salmonella_enterica	DNA-sulfur-modification-protein-DndB-[Salmonella-enterica]	28901
WP_000543904.1	DndB-ParB-nuclease	GCF_000487795.1	361	Bacteria>Proteobacteria>Gammaproteobacteria>Enterobacterales>Enterobacteriaceae	Salmonella_enterica	DNA-sulfur-modification-protein-DndB-[Salmonella-enterica]	28901
WP_023212800.1	DndB-ParB-nuclease	GCF_000486765.2	361	Bacteria>Proteobacteria>Gammaproteobacteria>Enterobacterales>Enterobacteriaceae	Salmonella_enterica	DNA-sulfur-modification-protein-DndB-[Salmonella-enterica]	28901
WP_023234371.1	DndB-ParB-nuclease	GCF_000487275.1	361	Bacteria>Proteobacteria>Gammaproteobacteria>Enterobacterales>Enterobacteriaceae	Salmonella_enterica	DNA-sulfur-modification-protein-DndB-[Salmonella-enterica]	28901
WP_023241003.1	DndB-ParB-nuclease	GCF_000487455.1	361	Bacteria>Proteobacteria>Gammaproteobacteria>Enterobacterales>Enterobacteriaceae	Salmonella_enterica	DNA-sulfur-modification-protein-DndB-[Salmonella-enterica]	28901
WP_053529583.1	DndB-ParB-nuclease	GCF_003325215.1	361	Bacteria>Proteobacteria>Gammaproteobacteria>Enterobacterales>Enterobacteriaceae	Salmonella_enterica	DNA-sulfur-modification-protein-DndB-[Salmonella-enterica]	28901
WP_058145317.1	DndB-ParB-nuclease	GCF_002109255.1	359	Bacteria>Proteobacteria>Gammaproteobacteria>Enterobacterales>Enterobacteriaceae	Salmonella_enterica	DNA-sulfur-modification-protein-DndB-[Salmonella-enterica]	28901
WP_079824007.1	DndB-ParB-nuclease	GCF_013162165.1	361	Bacteria>Proteobacteria>Gammaproteobacteria>Enterobacterales>Enterobacteriaceae	Salmonella_enterica	DNA-sulfur-modification-protein-DndB-[Salmonella-enterica]	28901
WP_080108397.1	DndB-ParB-nuclease	GCF_020520535.1	361	Bacteria>Proteobacteria>Gammaproteobacteria>Enterobacterales>Enterobacteriaceae	Salmonella_enterica	DNA-sulfur-modification-protein-DndB-[Salmonella-enterica]	28901
WP_082326722.1	DndB-ParB-nuclease	GCF_002035805.1	361	Bacteria>Proteobacteria>Gammaproteobacteria>Enterobacterales>Enterobacteriaceae	Salmonella_enterica	DNA-sulfur-modification-protein-DndB-[Salmonella-enterica]	28901
WP_136058522.1	DndB-ParB-nuclease	GCF_016030095.1	361	Bacteria>Proteobacteria>Gammaproteobacteria>Enterobacterales>Enterobacteriaceae	Salmonella_enterica	DNA-sulfur-modification-protein-DndB-[Salmonella-enterica]	28901
WP_183047446.1	DndB-ParB-nuclease	GCF_903931285.1	361	Bacteria>Proteobacteria>Gammaproteobacteria>Enterobacterales>Enterobacteriaceae	Salmonella_enterica	DNA-sulfur-modification-protein-DndB-[Salmonella-enterica]	28901
WP_183059471.1	DndB-ParB-nuclease	GCF_903930865.1	361	Bacteria>Proteobacteria>Gammaproteobacteria>Enterobacterales>Enterobacteriaceae	Salmonella_enterica	DNA-sulfur-modification-protein-DndB-[Salmonella-enterica]	28901
EBP6085710.1	DndB-ParB-nuclease	GCA_006872585.1	575	Bacteria>Proteobacteria>Gammaproteobacteria>Enterobacterales>Enterobacteriaceae	Salmonella_enterica	hypothetical-protein-ABE75_00840-[Salmonella-enterica]	28901
EBA4555411.1	DndB-ParB-nuclease	GCA_006096655.1	575	Bacteria>Proteobacteria>Gammaproteobacteria>Enterobacterales>Enterobacteriaceae	Salmonella_enterica	hypothetical-protein-AIO31_10095-[Salmonella-enterica]	28901
EAY0717629.1	DndB-ParB-nuclease	GCA_005991165.1	575	Bacteria>Proteobacteria>Gammaproteobacteria>Enterobacterales>Enterobacteriaceae	Salmonella_enterica	hypothetical-protein-B4R24_23980-[Salmonella-enterica]	28901
ECT9549637.1	DndB-ParB-nuclease	GCA_008488425.1	562	Bacteria>Proteobacteria>Gammaproteobacteria>Enterobacterales>Enterobacteriaceae	Salmonella_enterica	hypothetical-protein-CJG84_14555-[Salmonella-enterica]	28901
EAA9830762.1	DndB-ParB-nuclease	GCA_004226125.1	575	Bacteria>Proteobacteria>Gammaproteobacteria>Enterobacterales>Enterobacteriaceae	Salmonella_enterica	hypothetical-protein-D6Y25_24215-[Salmonella-enterica]	28901
EBD9702964.1	DndB-ParB-nuclease	GCA_006275155.1	575	Bacteria>Proteobacteria>Gammaproteobacteria>Enterobacterales>Enterobacteriaceae	Salmonella_enterica	hypothetical-protein-DBW21_23470-[Salmonella-enterica]	28901
EAU0751437.1	DndB-ParB-nuclease	GCA_005760355.1	575	Bacteria>Proteobacteria>Gammaproteobacteria>Enterobacterales>Enterobacteriaceae	Salmonella_enterica	hypothetical-protein-DLF99_11980-[Salmonella-enterica]	28901
EAV2276325.1	DndB-ParB-nuclease	GCA_005833045.1	575	Bacteria>Proteobacteria>Gammaproteobacteria>Enterobacterales>Enterobacteriaceae	Salmonella_enterica	hypothetical-protein-EX036_07890-[Salmonella-enterica]	28901
EAO5907480.1	DndB-ParB-nuclease	GCA_005512655.1	575	Bacteria>Proteobacteria>Gammaproteobacteria>Enterobacterales>Enterobacteriaceae	Salmonella_enterica	hypothetical-protein-FBH55_22730-[Salmonella-enterica]	28901
EBF3832249.1	DndB-ParB-nuclease	GCA_006340165.1	575	Bacteria>Proteobacteria>Gammaproteobacteria>Enterobacterales>Enterobacteriaceae	Salmonella_enterica	hypothetical-protein-FH321_19065-[Salmonella-enterica]	28901
EBG4910949.1	DndB-ParB-nuclease	GCA_006430015.1	575	Bacteria>Proteobacteria>Gammaproteobacteria>Enterobacterales>Enterobacteriaceae	Salmonella_enterica	hypothetical-protein-FI030_24125-[Salmonella-enterica]	28901
EBG7222317.1	DndB-ParB-nuclease	GCA_006458805.1	575	Bacteria>Proteobacteria>Gammaproteobacteria>Enterobacterales>Enterobacteriaceae	Salmonella_enterica	hypothetical-protein-FJI16_23250-[Salmonella-enterica]	28901
EBH3200354.1	DndB-ParB-nuclease	GCA_006486285.1	575	Bacteria>Proteobacteria>Gammaproteobacteria>Enterobacterales>Enterobacteriaceae	Salmonella_enterica	hypothetical-protein-FKH06_00710-[Salmonella-enterica]	28901
EEO8527595.1	DndB-ParB-nuclease	GCA_011693655.1	575	Bacteria>Proteobacteria>Gammaproteobacteria>Enterobacterales>Enterobacteriaceae	Salmonella_enterica	hypothetical-protein-G6D46_004724-[Salmonella-enterica]	28901
EDH7398368.1	DndB-ParB-nuclease	GCA_009255085.1	575	Bacteria>Proteobacteria>Gammaproteobacteria>Enterobacterales>Enterobacteriaceae	Salmonella_enterica	hypothetical-protein-GA162_22285-[Salmonella-enterica]	28901
EDY0215437.1	DndB-ParB-nuclease	GCA_010792795.1	575	Bacteria>Proteobacteria>Gammaproteobacteria>Enterobacterales>Enterobacteriaceae	Salmonella_enterica	hypothetical-protein-GSK36_004756-[Salmonella-enterica]	28901
EEG3003694.1	DndB-ParB-nuclease	GCA_011220445.1	575	Bacteria>Proteobacteria>Gammaproteobacteria>Enterobacterales>Enterobacteriaceae	Salmonella_enterica	hypothetical-protein-GXT81_004733-[Salmonella-enterica]	28901
EGB7953337.1	DndB-ParB-nuclease	GCA_014242295.1	548	Bacteria>Proteobacteria>Gammaproteobacteria>Enterobacterales>Enterobacteriaceae	Salmonella_enterica	hypothetical-protein-H8I18_004764,-partial-[Salmonella-enterica]	28901
EEW9755252.1	DndB-ParB-nuclease	GCA_012106055.1	575	Bacteria>Proteobacteria>Gammaproteobacteria>Enterobacterales>Enterobacteriaceae	Salmonella_enterica	hypothetical-protein-HFV75_000640-[Salmonella-enterica]	28901
EFO6415261.1	DndB-ParB-nuclease	GCA_013099915.1	575	Bacteria>Proteobacteria>Gammaproteobacteria>Enterobacterales>Enterobacteriaceae	Salmonella_enterica	hypothetical-protein-HN070_004669-[Salmonella-enterica]	28901
EHT8166087.1	DndB-ParB-nuclease	GCA_019256965.1	575	Bacteria>Proteobacteria>Gammaproteobacteria>Enterobacterales>Enterobacteriaceae	Salmonella_enterica	hypothetical-protein-KXQ91_000976-[Salmonella-enterica]	28901
EAW6002185.1	DndB-ParB-nuclease	GCA_005918065.1	98	Bacteria>Proteobacteria>Gammaproteobacteria>Enterobacterales>Enterobacteriaceae	Salmonella_enterica	hypothetical-protein-[Salmonella-enterica]	28901
EBN9070840.1	DndB-ParB-nuclease	GCA_006793285.1	87	Bacteria>Proteobacteria>Gammaproteobacteria>Enterobacterales>Enterobacteriaceae	Salmonella_enterica	hypothetical-protein-[Salmonella-enterica]	28901
ECG0564355.1	DndB-ParB-nuclease	GCA_007642775.1	78	Bacteria>Proteobacteria>Gammaproteobacteria>Enterobacterales>Enterobacteriaceae	Salmonella_enterica	hypothetical-protein-[Salmonella-enterica]	28901
EAC1850689.1	DndB-ParB-nuclease	GCA_004278635.1	575	Bacteria>Proteobacteria>Gammaproteobacteria>Enterobacterales>Enterobacteriaceae	Salmonella_enterica	hypothetical-protein-[Salmonella-enterica]	28901
EAO9627574.1	DndB-ParB-nuclease	GCA_005533535.1	562	Bacteria>Proteobacteria>Gammaproteobacteria>Enterobacterales>Enterobacteriaceae	Salmonella_enterica	hypothetical-protein-[Salmonella-enterica]	28901
EAU0668418.1	DndB-ParB-nuclease	GCA_005758915.1	653	Bacteria>Proteobacteria>Gammaproteobacteria>Enterobacterales>Enterobacteriaceae	Salmonella_enterica	hypothetical-protein-[Salmonella-enterica]	28901
EAX0971380.1	DndB-ParB-nuclease	GCA_005947645.1	373	Bacteria>Proteobacteria>Gammaproteobacteria>Enterobacterales>Enterobacteriaceae	Salmonella_enterica	hypothetical-protein-[Salmonella-enterica]	28901
EBA4497950.1	DndB-ParB-nuclease	GCA_006097275.1	706	Bacteria>Proteobacteria>Gammaproteobacteria>Enterobacterales>Enterobacteriaceae	Salmonella_enterica	hypothetical-protein-[Salmonella-enterica]	28901
EBC1275288.1	DndB-ParB-nuclease	GCA_006174785.1	696	Bacteria>Proteobacteria>Gammaproteobacteria>Enterobacterales>Enterobacteriaceae	Salmonella_enterica	hypothetical-protein-[Salmonella-enterica]	28901
EBK4658636.1	DndB-ParB-nuclease	GCA_006645525.1	404	Bacteria>Proteobacteria>Gammaproteobacteria>Enterobacterales>Enterobacteriaceae	Salmonella_enterica	hypothetical-protein-[Salmonella-enterica]	28901
EHQ5158487.1	DndB-ParB-nuclease	GCA_018945535.1	562	Bacteria>Proteobacteria>Gammaproteobacteria>Enterobacterales>Enterobacteriaceae	Salmonella_enterica	hypothetical-protein-[Salmonella-enterica]	28901
HAF2128876.1	DndB-ParB-nuclease	GCA_011226015.1	565	Bacteria>Proteobacteria>Gammaproteobacteria>Enterobacterales>Enterobacteriaceae	Salmonella_enterica	hypothetical-protein-[Salmonella-enterica]	28901
WP_001125368.1	DndB-ParB-nuclease	GCF_000170195.2	575	Bacteria>Proteobacteria>Gammaproteobacteria>Enterobacterales>Enterobacteriaceae	Salmonella_enterica	hypothetical-protein-[Salmonella-enterica]	28901
WP_023247621.1	DndB-ParB-nuclease	GCF_000487635.2	562	Bacteria>Proteobacteria>Gammaproteobacteria>Enterobacterales>Enterobacteriaceae	Salmonella_enterica	hypothetical-protein-[Salmonella-enterica]	28901
WP_058145313.1	DndB-ParB-nuclease	GCF_002109255.1	563	Bacteria>Proteobacteria>Gammaproteobacteria>Enterobacterales>Enterobacteriaceae	Salmonella_enterica	hypothetical-protein-[Salmonella-enterica]	28901
WP_131044939.1	DndB-ParB-nuclease	GCF_004310605.1	575	Bacteria>Proteobacteria>Gammaproteobacteria>Enterobacterales>Enterobacteriaceae	Salmonella_enterica	hypothetical-protein-[Salmonella-enterica]	28901
WP_183047452.1	DndB-ParB-nuclease	GCF_903931285.1	706	Bacteria>Proteobacteria>Gammaproteobacteria>Enterobacterales>Enterobacteriaceae	Salmonella_enterica	hypothetical-protein-[Salmonella-enterica]	28901
WP_241505407.1	DndB-ParB-nuclease	GCF_014121705.1	539	Bacteria>Proteobacteria>Gammaproteobacteria>Enterobacterales>Enterobacteriaceae	Salmonella_enterica	hypothetical-protein-[Salmonella-enterica]	28901
EBA2396731.1	DndB-ParB-nuclease	GCA_006087635.1	575	Bacteria>Proteobacteria>Gammaproteobacteria>Enterobacterales>Enterobacteriaceae	Salmonella_enterica	hypothetical-protein-UK64_23920-[Salmonella-enterica]	28901
EAX0463277.1	DndB-ParB-nuclease	GCA_005944685.1	575	Bacteria>Proteobacteria>Gammaproteobacteria>Enterobacterales>Enterobacteriaceae	Salmonella_enterica	hypothetical-protein-WO94_22955-[Salmonella-enterica]	28901
EBA2584001.1	DndB-ParB-nuclease	GCA_006088675.1	575	Bacteria>Proteobacteria>Gammaproteobacteria>Enterobacterales>Enterobacteriaceae	Salmonella_enterica	hypothetical-protein-WV00_23275-[Salmonella-enterica]	28901
EBP5213613.1	DndB-ParB-nuclease	GCA_006870505.1	575	Bacteria>Proteobacteria>Gammaproteobacteria>Enterobacterales>Enterobacteriaceae	Salmonella_enterica	hypothetical-protein-XP34_23500-[Salmonella-enterica]	28901
EBP4961965.1	DndB-ParB-nuclease	GCA_006868425.1	575	Bacteria>Proteobacteria>Gammaproteobacteria>Enterobacterales>Enterobacteriaceae	Salmonella_enterica	hypothetical-protein-XQ05_23815-[Salmonella-enterica]	28901
EBP5791776.1	DndB-ParB-nuclease	GCA_006871865.1	575	Bacteria>Proteobacteria>Gammaproteobacteria>Enterobacterales>Enterobacteriaceae	Salmonella_enterica	hypothetical-protein-YW68_24345-[Salmonella-enterica]	28901
SUF35273.1	DndB-ParB-nuclease	GCA_900456065.1	361	Bacteria>Proteobacteria>Gammaproteobacteria>Enterobacterales>Enterobacteriaceae	Salmonella_enterica	putative-bacteriophage-protein-[Salmonella-enterica]	28901
EBQ4833792.1	DndB-ParB-nuclease	GCA_006912155.1	359	Bacteria>Proteobacteria>Gammaproteobacteria>Enterobacterales>Enterobacteriaceae	Salmonella_enterica_subsp._arizonae	DNA-sulfur-modification-protein-DndB-[Salmonella-enterica-subsp.-arizonae]	59203
EBQ4833787.1	DndB-ParB-nuclease	GCA_006912155.1	562	Bacteria>Proteobacteria>Gammaproteobacteria>Enterobacterales>Enterobacteriaceae	Salmonella_enterica_subsp._arizonae	hypothetical-protein-[Salmonella-enterica-subsp.-arizonae]	59203
ASO61031.1	DndB-ParB-nuclease	GCA_002234855.1	361	Bacteria>Proteobacteria>Gammaproteobacteria>Enterobacterales>Enterobacteriaceae	Salmonella_enterica_subsp._arizonae_serovar_53:-:-_str._SA20100345	DNA-sulfur-modification-protein-DndB-[Salmonella-enterica-subsp.-arizonae-serovar-53:-:–str.-SA20100345]	1243606
ASO63528.1	DndB-ParB-nuclease	GCA_002234855.1	389	Bacteria>Proteobacteria>Gammaproteobacteria>Enterobacterales>Enterobacteriaceae	Salmonella_enterica_subsp._arizonae_serovar_53:-:-_str._SA20100345	hypothetical-protein-LFZ50_08885-[Salmonella-enterica-subsp.-arizonae-serovar-53:-:–str.-SA20100345]	1243606
ECI5659660.1	DndB-ParB-nuclease	GCA_007777005.1	379	Bacteria>Proteobacteria>Gammaproteobacteria>Enterobacterales>Enterobacteriaceae	Salmonella_enterica_subsp._diarizonae	DGQHR-domain-containing-protein-[Salmonella-enterica-subsp.-diarizonae]	59204
EEE1928470.1	DndB-ParB-nuclease	GCA_011120325.1	389	Bacteria>Proteobacteria>Gammaproteobacteria>Enterobacterales>Enterobacteriaceae	Salmonella_enterica_subsp._diarizonae	DGQHR-domain-containing-protein-[Salmonella-enterica-subsp.-diarizonae]	59204
SUG57652.1	DndB-ParB-nuclease	GCA_900456375.1	343	Bacteria>Proteobacteria>Gammaproteobacteria>Enterobacterales>Enterobacteriaceae	Salmonella_enterica_subsp._diarizonae	DNA-phosphorothioation-associated-DGQHR-protein-1-[Salmonella-enterica-subsp.-diarizonae]	59204
ECI5659648.1	DndB-ParB-nuclease	GCA_007777005.1	361	Bacteria>Proteobacteria>Gammaproteobacteria>Enterobacterales>Enterobacteriaceae	Salmonella_enterica_subsp._diarizonae	DNA-sulfur-modification-protein-DndB-[Salmonella-enterica-subsp.-diarizonae]	59204
EDX6248945.1	DndB-ParB-nuclease	GCA_010772755.1	361	Bacteria>Proteobacteria>Gammaproteobacteria>Enterobacterales>Enterobacteriaceae	Salmonella_enterica_subsp._diarizonae	DNA-sulfur-modification-protein-DndB-[Salmonella-enterica-subsp.-diarizonae]	59204
EEE1928475.1	DndB-ParB-nuclease	GCA_011120325.1	361	Bacteria>Proteobacteria>Gammaproteobacteria>Enterobacterales>Enterobacteriaceae	Salmonella_enterica_subsp._diarizonae	DNA-sulfur-modification-protein-DndB-[Salmonella-enterica-subsp.-diarizonae]	59204
MIE68573.1	DndB-ParB-nuclease	GCA_003869275.1	361	Bacteria>Proteobacteria>Gammaproteobacteria>Enterobacterales>Enterobacteriaceae	Salmonella_enterica_subsp._diarizonae	DNA-sulfur-modification-protein-DndB-[Salmonella-enterica-subsp.-diarizonae]	59204
EDX6248939.1	DndB-ParB-nuclease	GCA_010772755.1	706	Bacteria>Proteobacteria>Gammaproteobacteria>Enterobacterales>Enterobacteriaceae	Salmonella_enterica_subsp._diarizonae	hypothetical-protein-[Salmonella-enterica-subsp.-diarizonae]	59204
SUG57659.1	DndB-ParB-nuclease	GCA_900456375.1	361	Bacteria>Proteobacteria>Gammaproteobacteria>Enterobacterales>Enterobacteriaceae	Salmonella_enterica_subsp._diarizonae	putative-bacteriophage-protein-[Salmonella-enterica-subsp.-diarizonae]	59204
EBF8126956.1	DndB-ParB-nuclease	GCA_006393975.1	397	Bacteria>Proteobacteria>Gammaproteobacteria>Enterobacterales>Enterobacteriaceae	Salmonella_enterica_subsp._enterica	DGQHR-domain-containing-protein-[Salmonella-enterica-subsp.-enterica]	59201
EBG0124147.1	DndB-ParB-nuclease	GCA_006404315.1	329	Bacteria>Proteobacteria>Gammaproteobacteria>Enterobacterales>Enterobacteriaceae	Salmonella_enterica_subsp._enterica	DGQHR-domain-containing-protein-[Salmonella-enterica-subsp.-enterica]	59201
EBG5472483.1	DndB-ParB-nuclease	GCA_006430795.1	213	Bacteria>Proteobacteria>Gammaproteobacteria>Enterobacterales>Enterobacteriaceae	Salmonella_enterica_subsp._enterica	DGQHR-domain-containing-protein-[Salmonella-enterica-subsp.-enterica]	59201
ECR5874440.1	DndB-ParB-nuclease	GCA_008361495.1	385	Bacteria>Proteobacteria>Gammaproteobacteria>Enterobacterales>Enterobacteriaceae	Salmonella_enterica_subsp._enterica	DGQHR-domain-containing-protein-[Salmonella-enterica-subsp.-enterica]	59201
EDR3007040.1	DndB-ParB-nuclease	GCA_010439755.1	329	Bacteria>Proteobacteria>Gammaproteobacteria>Enterobacterales>Enterobacteriaceae	Salmonella_enterica_subsp._enterica	DGQHR-domain-containing-protein-[Salmonella-enterica-subsp.-enterica]	59201
EEJ2348857.1	DndB-ParB-nuclease	GCA_011411475.1	379	Bacteria>Proteobacteria>Gammaproteobacteria>Enterobacterales>Enterobacteriaceae	Salmonella_enterica_subsp._enterica	DGQHR-domain-containing-protein-[Salmonella-enterica-subsp.-enterica]	59201
EHZ3053524.1	DndB-ParB-nuclease	GCA_019691715.1	389	Bacteria>Proteobacteria>Gammaproteobacteria>Enterobacterales>Enterobacteriaceae	Salmonella_enterica_subsp._enterica	DGQHR-domain-containing-protein-[Salmonella-enterica-subsp.-enterica]	59201
VEB50957.1	DndB-ParB-nuclease	GCA_900635865.1	84	Bacteria>Proteobacteria>Gammaproteobacteria>Enterobacterales>Enterobacteriaceae	Salmonella_enterica_subsp._enterica	DNA-sulfur-modification-protein-DndB-[Salmonella-enterica-subsp.-enterica]	59201
EAA1022312.1	DndB-ParB-nuclease	GCA_004158495.1	361	Bacteria>Proteobacteria>Gammaproteobacteria>Enterobacterales>Enterobacteriaceae	Salmonella_enterica_subsp._enterica	DNA-sulfur-modification-protein-DndB-[Salmonella-enterica-subsp.-enterica]	59201
EAA1042670.1	DndB-ParB-nuclease	GCA_004159255.1	361	Bacteria>Proteobacteria>Gammaproteobacteria>Enterobacterales>Enterobacteriaceae	Salmonella_enterica_subsp._enterica	DNA-sulfur-modification-protein-DndB-[Salmonella-enterica-subsp.-enterica]	59201
EAA3135505.1	DndB-ParB-nuclease	GCA_004176515.1	361	Bacteria>Proteobacteria>Gammaproteobacteria>Enterobacterales>Enterobacteriaceae	Salmonella_enterica_subsp._enterica	DNA-sulfur-modification-protein-DndB-[Salmonella-enterica-subsp.-enterica]	59201
EAA4524710.1	DndB-ParB-nuclease	GCA_004181445.1	361	Bacteria>Proteobacteria>Gammaproteobacteria>Enterobacterales>Enterobacteriaceae	Salmonella_enterica_subsp._enterica	DNA-sulfur-modification-protein-DndB-[Salmonella-enterica-subsp.-enterica]	59201
EAA5206278.1	DndB-ParB-nuclease	GCA_004184415.1	361	Bacteria>Proteobacteria>Gammaproteobacteria>Enterobacterales>Enterobacteriaceae	Salmonella_enterica_subsp._enterica	DNA-sulfur-modification-protein-DndB-[Salmonella-enterica-subsp.-enterica]	59201
EAA6031981.1	DndB-ParB-nuclease	GCA_004187285.1	361	Bacteria>Proteobacteria>Gammaproteobacteria>Enterobacterales>Enterobacteriaceae	Salmonella_enterica_subsp._enterica	DNA-sulfur-modification-protein-DndB-[Salmonella-enterica-subsp.-enterica]	59201
EAA6710915.1	DndB-ParB-nuclease	GCA_004189995.1	361	Bacteria>Proteobacteria>Gammaproteobacteria>Enterobacterales>Enterobacteriaceae	Salmonella_enterica_subsp._enterica	DNA-sulfur-modification-protein-DndB-[Salmonella-enterica-subsp.-enterica]	59201
EAA9089421.1	DndB-ParB-nuclease	GCA_004222825.1	361	Bacteria>Proteobacteria>Gammaproteobacteria>Enterobacterales>Enterobacteriaceae	Salmonella_enterica_subsp._enterica	DNA-sulfur-modification-protein-DndB-[Salmonella-enterica-subsp.-enterica]	59201
EAA9294216.1	DndB-ParB-nuclease	GCA_004226185.1	359	Bacteria>Proteobacteria>Gammaproteobacteria>Enterobacterales>Enterobacteriaceae	Salmonella_enterica_subsp._enterica	DNA-sulfur-modification-protein-DndB-[Salmonella-enterica-subsp.-enterica]	59201
EBF8126952.1	DndB-ParB-nuclease	GCA_006393975.1	361	Bacteria>Proteobacteria>Gammaproteobacteria>Enterobacterales>Enterobacteriaceae	Salmonella_enterica_subsp._enterica	DNA-sulfur-modification-protein-DndB-[Salmonella-enterica-subsp.-enterica]	59201
EBG0124145.1	DndB-ParB-nuclease	GCA_006404315.1	359	Bacteria>Proteobacteria>Gammaproteobacteria>Enterobacterales>Enterobacteriaceae	Salmonella_enterica_subsp._enterica	DNA-sulfur-modification-protein-DndB-[Salmonella-enterica-subsp.-enterica]	59201
EBG2839801.1	DndB-ParB-nuclease	GCA_006419895.1	359	Bacteria>Proteobacteria>Gammaproteobacteria>Enterobacterales>Enterobacteriaceae	Salmonella_enterica_subsp._enterica	DNA-sulfur-modification-protein-DndB-[Salmonella-enterica-subsp.-enterica]	59201
EBG5472485.1	DndB-ParB-nuclease	GCA_006430795.1	359	Bacteria>Proteobacteria>Gammaproteobacteria>Enterobacterales>Enterobacteriaceae	Salmonella_enterica_subsp._enterica	DNA-sulfur-modification-protein-DndB-[Salmonella-enterica-subsp.-enterica]	59201
EBG8223407.1	DndB-ParB-nuclease	GCA_006463825.1	361	Bacteria>Proteobacteria>Gammaproteobacteria>Enterobacterales>Enterobacteriaceae	Salmonella_enterica_subsp._enterica	DNA-sulfur-modification-protein-DndB-[Salmonella-enterica-subsp.-enterica]	59201
ECC3322693.1	DndB-ParB-nuclease	GCA_007472615.1	361	Bacteria>Proteobacteria>Gammaproteobacteria>Enterobacterales>Enterobacteriaceae	Salmonella_enterica_subsp._enterica	DNA-sulfur-modification-protein-DndB-[Salmonella-enterica-subsp.-enterica]	59201
ECC9027371.1	DndB-ParB-nuclease	GCA_007498385.1	361	Bacteria>Proteobacteria>Gammaproteobacteria>Enterobacterales>Enterobacteriaceae	Salmonella_enterica_subsp._enterica	DNA-sulfur-modification-protein-DndB-[Salmonella-enterica-subsp.-enterica]	59201
ECE0093966.1	DndB-ParB-nuclease	GCA_007546855.1	361	Bacteria>Proteobacteria>Gammaproteobacteria>Enterobacterales>Enterobacteriaceae	Salmonella_enterica_subsp._enterica	DNA-sulfur-modification-protein-DndB-[Salmonella-enterica-subsp.-enterica]	59201

Accession	Annotation	GCA	Length	Phyletic.spread	Strain.name	Defline	Species.Taxid
ECI3716710.1	DndB-ParB-nuclease	GCA_007768905.1	361	Bacteria>Proteobacteria>Gammaproteobacteria>Enterobacterales>Enterobacteriaceae	Salmonella_enterica_subsp._enterica	DNA-sulfur-modification-protein-DndB-[Salmonella-enterica-subsp.-enterica]	59201
ECR5874436.1	DndB-ParB-nuclease	GCA_008361495.1	361	Bacteria>Proteobacteria>Gammaproteobacteria>Enterobacterales>Enterobacteriaceae	Salmonella_enterica_subsp._enterica	DNA-sulfur-modification-protein-DndB-[Salmonella-enterica-subsp.-enterica]	59201
EDR3007038.1	DndB-ParB-nuclease	GCA_010439755.1	359	Bacteria>Proteobacteria>Gammaproteobacteria>Enterobacterales>Enterobacteriaceae	Salmonella_enterica_subsp._enterica	DNA-sulfur-modification-protein-DndB-[Salmonella-enterica-subsp.-enterica]	59201
EDR7044435.1	DndB-ParB-nuclease	GCA_010456525.1	361	Bacteria>Proteobacteria>Gammaproteobacteria>Enterobacterales>Enterobacteriaceae	Salmonella_enterica_subsp._enterica	DNA-sulfur-modification-protein-DndB-[Salmonella-enterica-subsp.-enterica]	59201
EDS6425833.1	DndB-ParB-nuclease	GCA_010506355.1	361	Bacteria>Proteobacteria>Gammaproteobacteria>Enterobacterales>Enterobacteriaceae	Salmonella_enterica_subsp._enterica	DNA-sulfur-modification-protein-DndB-[Salmonella-enterica-subsp.-enterica]	59201
EDT8876177.1	DndB-ParB-nuclease	GCA_010565975.1	361	Bacteria>Proteobacteria>Gammaproteobacteria>Enterobacterales>Enterobacteriaceae	Salmonella_enterica_subsp._enterica	DNA-sulfur-modification-protein-DndB-[Salmonella-enterica-subsp.-enterica]	59201
EDV4561215.1	DndB-ParB-nuclease	GCA_010662765.1	361	Bacteria>Proteobacteria>Gammaproteobacteria>Enterobacterales>Enterobacteriaceae	Salmonella_enterica_subsp._enterica	DNA-sulfur-modification-protein-DndB-[Salmonella-enterica-subsp.-enterica]	59201
EEJ2348869.1	DndB-ParB-nuclease	GCA_011411475.1	361	Bacteria>Proteobacteria>Gammaproteobacteria>Enterobacterales>Enterobacteriaceae	Salmonella_enterica_subsp._enterica	DNA-sulfur-modification-protein-DndB-[Salmonella-enterica-subsp.-enterica]	59201
EHG8446475.1	DndB-ParB-nuclease	GCA_017573985.1	361	Bacteria>Proteobacteria>Gammaproteobacteria>Enterobacterales>Enterobacteriaceae	Salmonella_enterica_subsp._enterica	DNA-sulfur-modification-protein-DndB-[Salmonella-enterica-subsp.-enterica]	59201
EHX9260052.1	DndB-ParB-nuclease	GCA_019554875.1	361	Bacteria>Proteobacteria>Gammaproteobacteria>Enterobacterales>Enterobacteriaceae	Salmonella_enterica_subsp._enterica	DNA-sulfur-modification-protein-DndB-[Salmonella-enterica-subsp.-enterica]	59201
EAA9294211.1	DndB-ParB-nuclease	GCA_004226185.1	562	Bacteria>Proteobacteria>Gammaproteobacteria>Enterobacterales>Enterobacteriaceae	Salmonella_enterica_subsp._enterica	hypothetical-protein-[Salmonella-enterica-subsp.-enterica]	59201
EBG0124140.1	DndB-ParB-nuclease	GCA_006404315.1	563	Bacteria>Proteobacteria>Gammaproteobacteria>Enterobacterales>Enterobacteriaceae	Salmonella_enterica_subsp._enterica	hypothetical-protein-[Salmonella-enterica-subsp.-enterica]	59201
EBG2839796.1	DndB-ParB-nuclease	GCA_006419895.1	562	Bacteria>Proteobacteria>Gammaproteobacteria>Enterobacterales>Enterobacteriaceae	Salmonella_enterica_subsp._enterica	hypothetical-protein-[Salmonella-enterica-subsp.-enterica]	59201
EDR3007033.1	DndB-ParB-nuclease	GCA_010439755.1	563	Bacteria>Proteobacteria>Gammaproteobacteria>Enterobacterales>Enterobacteriaceae	Salmonella_enterica_subsp._enterica	hypothetical-protein-[Salmonella-enterica-subsp.-enterica]	59201
VEA42918.1	DndB-ParB-nuclease	GCA_900635605.1	361	Bacteria>Proteobacteria>Gammaproteobacteria>Enterobacterales>Enterobacteriaceae	Salmonella_enterica_subsp._enterica	putative-bacteriophage-protein-[Salmonella-enterica-subsp.-enterica]	59201
EHK4824003.1	DndB-ParB-nuclease	GCA_018033665.1	361	Bacteria>Proteobacteria>Gammaproteobacteria>Enterobacterales>Enterobacteriaceae	Salmonella_enterica_subsp._enterica_serovar_Abony	DNA-sulfur-modification-protein-DndB-[Salmonella-enterica-subsp.-enterica-serovar-Abony]	29482
EHK4823997.1	DndB-ParB-nuclease	GCA_018033665.1	706	Bacteria>Proteobacteria>Gammaproteobacteria>Enterobacterales>Enterobacteriaceae	Salmonella_enterica_subsp._enterica_serovar_Abony	hypothetical-protein-[Salmonella-enterica-subsp.-enterica-serovar-Abony]	29482
EGF4596950.1	DndB-ParB-nuclease	GCA_014507205.1	361	Bacteria>Proteobacteria>Gammaproteobacteria>Enterobacterales>Enterobacteriaceae	Salmonella_enterica_subsp._enterica_serovar_Agama	DNA-sulfur-modification-protein-DndB-[Salmonella-enterica-subsp.-enterica-serovar-Agama]	399581
ECB6183562.1	DndB-ParB-nuclease	GCA_007432985.1	395	Bacteria>Proteobacteria>Gammaproteobacteria>Enterobacterales>Enterobacteriaceae	Salmonella_enterica_subsp._enterica_serovar_Bareilly	DGQHR-domain-containing-protein-[Salmonella-enterica-subsp.-enterica-serovar-Bareilly]	58096
ESH75903.1	DndB-ParB-nuclease	GCA_000487455.1	361	Bacteria>Proteobacteria>Gammaproteobacteria>Enterobacterales>Enterobacteriaceae	Salmonella_enterica_subsp._enterica_serovar_Bareilly_str._CFSAN000183	hypothetical-protein-SEEB0183_04050-[Salmonella-enterica-subsp.-enterica-serovar-Bareilly-str.-CFSAN000183]	1173463
ESH75907.1	DndB-ParB-nuclease	GCA_000487455.1	393	Bacteria>Proteobacteria>Gammaproteobacteria>Enterobacterales>Enterobacteriaceae	Salmonella_enterica_subsp._enterica_serovar_Bareilly_str._CFSAN000183	hypothetical-protein-SEEB0183_04070-[Salmonella-enterica-subsp.-enterica-serovar-Bareilly-str.-CFSAN000183]	1173463
EGN4578612.1	DndB-ParB-nuclease	GCA_015201345.1	350	Bacteria>Proteobacteria>Gammaproteobacteria>Enterobacterales>Enterobacteriaceae	Salmonella_enterica_subsp._enterica_serovar_Cannstatt	DNA-sulfur-modification-protein-DndB-[Salmonella-enterica-subsp.-enterica-serovar-Cannstatt]	2564307
EBS0850553.1	DndB-ParB-nuclease	GCA_006988855.1	399	Bacteria>Proteobacteria>Gammaproteobacteria>Enterobacterales>Enterobacteriaceae	Salmonella_enterica_subsp._enterica_serovar_Carno	DGQHR-domain-containing-protein-[Salmonella-enterica-subsp.-enterica-serovar-Carno]	2024268
EBS0850541.1	DndB-ParB-nuclease	GCA_006988855.1	361	Bacteria>Proteobacteria>Gammaproteobacteria>Enterobacterales>Enterobacteriaceae	Salmonella_enterica_subsp._enterica_serovar_Carno	DNA-sulfur-modification-protein-DndB-[Salmonella-enterica-subsp.-enterica-serovar-Carno]	2024268
HAU6708552.1	DndB-ParB-nuclease	GCA_016095075.1	361	Bacteria>Proteobacteria>Gammaproteobacteria>Enterobacterales>Enterobacteriaceae	Salmonella_enterica_subsp._enterica_serovar_Casablanca	DNA-sulfur-modification-protein-DndB-[Salmonella-enterica-subsp.-enterica-serovar-Casablanca]	2564314
ADN26581.1	DndB-ParB-nuclease	-	361	Bacteria>Proteobacteria>Gammaproteobacteria>Enterobacterales>Enterobacteriaceae	Salmonella_enterica_subsp._enterica_serovar_Cerro	DNA-phosphorothioation-protein-DptB-[Salmonella-enterica-subsp.-enterica-serovar-Cerro]	340188
EBF8670341.1	DndB-ParB-nuclease	GCA_006394195.1	361	Bacteria>Proteobacteria>Gammaproteobacteria>Enterobacterales>Enterobacteriaceae	Salmonella_enterica_subsp._enterica_serovar_Cerro	DNA-sulfur-modification-protein-DndB-[Salmonella-enterica-subsp.-enterica-serovar-Cerro]	340188
EBH9152262.1	DndB-ParB-nuclease	GCA_006523015.1	361	Bacteria>Proteobacteria>Gammaproteobacteria>Enterobacterales>Enterobacteriaceae	Salmonella_enterica_subsp._enterica_serovar_Cerro	DNA-sulfur-modification-protein-DndB-[Salmonella-enterica-subsp.-enterica-serovar-Cerro]	340188
EBH9281541.1	DndB-ParB-nuclease	GCA_006520155.1	361	Bacteria>Proteobacteria>Gammaproteobacteria>Enterobacterales>Enterobacteriaceae	Salmonella_enterica_subsp._enterica_serovar_Cerro	DNA-sulfur-modification-protein-DndB-[Salmonella-enterica-subsp.-enterica-serovar-Cerro]	340188
EBH9424812.1	DndB-ParB-nuclease	GCA_006524175.1	361	Bacteria>Proteobacteria>Gammaproteobacteria>Enterobacterales>Enterobacteriaceae	Salmonella_enterica_subsp._enterica_serovar_Cerro	DNA-sulfur-modification-protein-DndB-[Salmonella-enterica-subsp.-enterica-serovar-Cerro]	340188
EBH9537652.1	DndB-ParB-nuclease	GCA_006525195.1	361	Bacteria>Proteobacteria>Gammaproteobacteria>Enterobacterales>Enterobacteriaceae	Salmonella_enterica_subsp._enterica_serovar_Cerro	DNA-sulfur-modification-protein-DndB-[Salmonella-enterica-subsp.-enterica-serovar-Cerro]	340188
EDM9772271.1	DndB-ParB-nuclease	GCA_009587045.1	361	Bacteria>Proteobacteria>Gammaproteobacteria>Enterobacterales>Enterobacteriaceae	Salmonella_enterica_subsp._enterica_serovar_Corvallis	DNA-sulfur-modification-protein-DndB-[Salmonella-enterica-subsp.-enterica-serovar-Corvallis]	593905
ECS7671244.1	DndB-ParB-nuclease	GCA_008435685.1	403	Bacteria>Proteobacteria>Gammaproteobacteria>Enterobacterales>Enterobacteriaceae	Salmonella_enterica_subsp._enterica_serovar_Cubana	DGQHR-domain-containing-protein-[Salmonella-enterica-subsp.-enterica-serovar-Cubana]	189201
EDQ3977672.1	DndB-ParB-nuclease	GCA_010396305.1	403	Bacteria>Proteobacteria>Gammaproteobacteria>Enterobacterales>Enterobacteriaceae	Salmonella_enterica_subsp._enterica_serovar_Cubana	DGQHR-domain-containing-protein-[Salmonella-enterica-subsp.-enterica-serovar-Cubana]	189201
ECS7671240.1	DndB-ParB-nuclease	GCA_008435685.1	361	Bacteria>Proteobacteria>Gammaproteobacteria>Enterobacterales>Enterobacteriaceae	Salmonella_enterica_subsp._enterica_serovar_Cubana	DNA-sulfur-modification-protein-DndB-[Salmonella-enterica-subsp.-enterica-serovar-Cubana]	189201
EDQ3977668.1	DndB-ParB-nuclease	GCA_010396305.1	361	Bacteria>Proteobacteria>Gammaproteobacteria>Enterobacterales>Enterobacteriaceae	Salmonella_enterica_subsp._enterica_serovar_Cubana	DNA-sulfur-modification-protein-DndB-[Salmonella-enterica-subsp.-enterica-serovar-Cubana]	189201
EDB6591003.1	DndB-ParB-nuclease	GCA_008915665.1	398	Bacteria>Proteobacteria>Gammaproteobacteria>Enterobacterales>Enterobacteriaceae	Salmonella_enterica_subsp._enterica_serovar_Derby	DGQHR-domain-containing-protein-[Salmonella-enterica-subsp.-enterica-serovar-Derby]	28144
EDA6306033.1	DndB-ParB-nuclease	GCA_008866505.1	361	Bacteria>Proteobacteria>Gammaproteobacteria>Enterobacterales>Enterobacteriaceae	Salmonella_enterica_subsp._enterica_serovar_Derby	DNA-sulfur-modification-protein-DndB-[Salmonella-enterica-subsp.-enterica-serovar-Derby]	28144
EDA6333781.1	DndB-ParB-nuclease	GCA_008866645.1	361	Bacteria>Proteobacteria>Gammaproteobacteria>Enterobacterales>Enterobacteriaceae	Salmonella_enterica_subsp._enterica_serovar_Derby	DNA-sulfur-modification-protein-DndB-[Salmonella-enterica-subsp.-enterica-serovar-Derby]	28144
EDB6591008.1	DndB-ParB-nuclease	GCA_008915665.1	361	Bacteria>Proteobacteria>Gammaproteobacteria>Enterobacterales>Enterobacteriaceae	Salmonella_enterica_subsp._enterica_serovar_Derby	DNA-sulfur-modification-protein-DndB-[Salmonella-enterica-subsp.-enterica-serovar-Derby]	28144
EED5673485.1	DndB-ParB-nuclease	GCA_011092605.1	361	Bacteria>Proteobacteria>Gammaproteobacteria>Enterobacterales>Enterobacteriaceae	Salmonella_enterica_subsp._enterica_serovar_Derby	DNA-sulfur-modification-protein-DndB-[Salmonella-enterica-subsp.-enterica-serovar-Derby]	28144
EEK7721885.1	DndB-ParB-nuclease	GCA_011485725.1	361	Bacteria>Proteobacteria>Gammaproteobacteria>Enterobacterales>Enterobacteriaceae	Salmonella_enterica_subsp._enterica_serovar_Derby	DNA-sulfur-modification-protein-DndB-[Salmonella-enterica-subsp.-enterica-serovar-Derby]	28144
EBK2662358.1	DndB-ParB-nuclease	GCA_006635185.1	329	Bacteria>Proteobacteria>Gammaproteobacteria>Enterobacterales>Enterobacteriaceae	Salmonella_enterica_subsp._enterica_serovar_Enteritidis	DGQHR-domain-containing-protein-[Salmonella-enterica-subsp.-enterica-serovar-Enteritidis]	149539
EBK2662356.1	DndB-ParB-nuclease	GCA_006635185.1	359	Bacteria>Proteobacteria>Gammaproteobacteria>Enterobacterales>Enterobacteriaceae	Salmonella_enterica_subsp._enterica_serovar_Enteritidis	DNA-sulfur-modification-protein-DndB-[Salmonella-enterica-subsp.-enterica-serovar-Enteritidis]	149539
EBK2662351.1	DndB-ParB-nuclease	GCA_006635185.1	563	Bacteria>Proteobacteria>Gammaproteobacteria>Enterobacterales>Enterobacteriaceae	Salmonella_enterica_subsp._enterica_serovar_Enteritidis	hypothetical-protein-[Salmonella-enterica-subsp.-enterica-serovar-Enteritidis]	149539
EBH8769757.1	DndB-ParB-nuclease	GCA_006521535.1	361	Bacteria>Proteobacteria>Gammaproteobacteria>Enterobacterales>Enterobacteriaceae	Salmonella_enterica_subsp._enterica_serovar_Freetown	DNA-sulfur-modification-protein-DndB-[Salmonella-enterica-subsp.-enterica-serovar-Freetown]	1151179
ECB3805294.1	DndB-ParB-nuclease	GCA_007422765.1	361	Bacteria>Proteobacteria>Gammaproteobacteria>Enterobacterales>Enterobacteriaceae	Salmonella_enterica_subsp._enterica_serovar_Fufu	DNA-sulfur-modification-protein-DndB-[Salmonella-enterica-subsp.-enterica-serovar-Fufu]	2564449
EDA9107296.1	DndB-ParB-nuclease	GCA_008880435.1	361	Bacteria>Proteobacteria>Gammaproteobacteria>Enterobacterales>Enterobacteriaceae	Salmonella_enterica_subsp._enterica_serovar_Gdansk	DNA-sulfur-modification-protein-DndB-[Salmonella-enterica-subsp.-enterica-serovar-Gdansk]	2564465
EDV2868615.1	DndB-ParB-nuclease	GCA_010652925.1	361	Bacteria>Proteobacteria>Gammaproteobacteria>Enterobacterales>Enterobacteriaceae	Salmonella_enterica_subsp._enterica_serovar_Haifa	DNA-sulfur-modification-protein-DndB-[Salmonella-enterica-subsp.-enterica-serovar-Haifa]	192956
EBV7267325.1	DndB-ParB-nuclease	GCA_007183715.1	423	Bacteria>Proteobacteria>Gammaproteobacteria>Enterobacterales>Enterobacteriaceae	Salmonella_enterica_subsp._enterica_serovar_Havana	DGQHR-domain-containing-protein-[Salmonella-enterica-subsp.-enterica-serovar-Havana]	179997
EBV7267330.1	DndB-ParB-nuclease	GCA_007183715.1	361	Bacteria>Proteobacteria>Gammaproteobacteria>Enterobacterales>Enterobacteriaceae	Salmonella_enterica_subsp._enterica_serovar_Havana	DNA-sulfur-modification-protein-DndB-[Salmonella-enterica-subsp.-enterica-serovar-Havana]	179997
EDU0324720.1	DndB-ParB-nuclease	GCA_010573365.1	361	Bacteria>Proteobacteria>Gammaproteobacteria>Enterobacterales>Enterobacteriaceae	Salmonella_enterica_subsp._enterica_serovar_Horsham	DNA-sulfur-modification-protein-DndB-[Salmonella-enterica-subsp.-enterica-serovar-Horsham]	2564543
EGO0257303.1	DndB-ParB-nuclease	GCA_015243815.1	361	Bacteria>Proteobacteria>Gammaproteobacteria>Enterobacterales>Enterobacteriaceae	Salmonella_enterica_subsp._enterica_serovar_Infantis	DNA-sulfur-modification-protein-DndB-[Salmonella-enterica-subsp.-enterica-serovar-Infantis]	595
ECU7221160.1	DndB-ParB-nuclease	GCA_008522465.1	575	Bacteria>Proteobacteria>Gammaproteobacteria>Enterobacterales>Enterobacteriaceae	Salmonella_enterica_subsp._enterica_serovar_Kentucky	hypothetical-protein-A9U55_23910-[Salmonella-enterica-subsp.-enterica-serovar-Kentucky]	192955
ECS4208859.1	DndB-ParB-nuclease	GCA_008414545.1	575	Bacteria>Proteobacteria>Gammaproteobacteria>Enterobacterales>Enterobacteriaceae	Salmonella_enterica_subsp._enterica_serovar_Kentucky	hypothetical-protein-A9U76_24060-[Salmonella-enterica-subsp.-enterica-serovar-Kentucky]	192955
ECT6817189.1	DndB-ParB-nuclease	GCA_008476745.1	575	Bacteria>Proteobacteria>Gammaproteobacteria>Enterobacterales>Enterobacteriaceae	Salmonella_enterica_subsp._enterica_serovar_Kentucky	hypothetical-protein-A9U91_02790-[Salmonella-enterica-subsp.-enterica-serovar-Kentucky]	192955
ECS4448027.1	DndB-ParB-nuclease	GCA_008415345.1	575	Bacteria>Proteobacteria>Gammaproteobacteria>Enterobacterales>Enterobacteriaceae	Salmonella_enterica_subsp._enterica_serovar_Kentucky	hypothetical-protein-A9V20_06070-[Salmonella-enterica-subsp.-enterica-serovar-Kentucky]	192955
EBL6181227.1	DndB-ParB-nuclease	GCA_006694785.1	575	Bacteria>Proteobacteria>Gammaproteobacteria>Enterobacterales>Enterobacteriaceae	Salmonella_enterica_subsp._enterica_serovar_Kentucky	hypothetical-protein-ADQ98_19500-[Salmonella-enterica-subsp.-enterica-serovar-Kentucky]	192955
ECS2155992.1	DndB-ParB-nuclease	GCA_008405445.1	575	Bacteria>Proteobacteria>Gammaproteobacteria>Enterobacterales>Enterobacteriaceae	Salmonella_enterica_subsp._enterica_serovar_Kentucky	hypothetical-protein-APL61_24150-[Salmonella-enterica-subsp.-enterica-serovar-Kentucky]	192955
ECI1979822.1	DndB-ParB-nuclease	GCA_007761975.1	575	Bacteria>Proteobacteria>Gammaproteobacteria>Enterobacterales>Enterobacteriaceae	Salmonella_enterica_subsp._enterica_serovar_Kentucky	hypothetical-protein-ASJ01_23660-[Salmonella-enterica-subsp.-enterica-serovar-Kentucky]	192955
EDB8255294.1	DndB-ParB-nuclease	GCA_008925665.1	575	Bacteria>Proteobacteria>Gammaproteobacteria>Enterobacterales>Enterobacteriaceae	Salmonella_enterica_subsp._enterica_serovar_Kentucky	hypothetical-protein-BCO82_24370-[Salmonella-enterica-subsp.-enterica-serovar-Kentucky]	192955
EEE3863727.1	DndB-ParB-nuclease	GCA_011130955.1	575	Bacteria>Proteobacteria>Gammaproteobacteria>Enterobacterales>Enterobacteriaceae	Salmonella_enterica_subsp._enterica_serovar_Kentucky	hypothetical-protein-C6K24_09135-[Salmonella-enterica-subsp.-enterica-serovar-Kentucky]	192955
EEQ0009598.1	DndB-ParB-nuclease	GCA_011758425.1	575	Bacteria>Proteobacteria>Gammaproteobacteria>Enterobacterales>Enterobacteriaceae	Salmonella_enterica_subsp._enterica_serovar_Kentucky	hypothetical-protein-C7S45_21480-[Salmonella-enterica-subsp.-enterica-serovar-Kentucky]	192955
EDJ4199862.1	DndB-ParB-nuclease	GCA_009373595.1	575	Bacteria>Proteobacteria>Gammaproteobacteria>Enterobacterales>Enterobacteriaceae	Salmonella_enterica_subsp._enterica_serovar_Kentucky	hypothetical-protein-CHN16_00835-[Salmonella-enterica-subsp.-enterica-serovar-Kentucky]	192955
EDL5352252.1	DndB-ParB-nuclease	GCA_009524095.1	575	Bacteria>Proteobacteria>Gammaproteobacteria>Enterobacterales>Enterobacteriaceae	Salmonella_enterica_subsp._enterica_serovar_Kentucky	hypothetical-protein-CWZ15_05635-[Salmonella-enterica-subsp.-enterica-serovar-Kentucky]	192955
ECU2240196.1	DndB-ParB-nuclease	GCA_008500565.1	575	Bacteria>Proteobacteria>Gammaproteobacteria>Enterobacterales>Enterobacteriaceae	Salmonella_enterica_subsp._enterica_serovar_Kentucky	hypothetical-protein-DS148_25090-[Salmonella-enterica-subsp.-enterica-serovar-Kentucky]	192955
ECV4632628.1	DndB-ParB-nuclease	GCA_008555485.1	575	Bacteria>Proteobacteria>Gammaproteobacteria>Enterobacterales>Enterobacteriaceae	Salmonella_enterica_subsp._enterica_serovar_Kentucky	hypothetical-protein-DXS27_10990-[Salmonella-enterica-subsp.-enterica-serovar-Kentucky]	192955
ECT2018472.1	DndB-ParB-nuclease	GCA_008455745.1	575	Bacteria>Proteobacteria>Gammaproteobacteria>Enterobacterales>Enterobacteriaceae	Salmonella_enterica_subsp._enterica_serovar_Kentucky	hypothetical-protein-DYM46_24610-[Salmonella-enterica-subsp.-enterica-serovar-Kentucky]	192955
ECU3329797.1	DndB-ParB-nuclease	GCA_008505825.1	575	Bacteria>Proteobacteria>Gammaproteobacteria>Enterobacterales>Enterobacteriaceae	Salmonella_enterica_subsp._enterica_serovar_Kentucky	hypothetical-protein-DYO76_13730-[Salmonella-enterica-subsp.-enterica-serovar-Kentucky]	192955
EBO1705386.1	DndB-ParB-nuclease	GCA_006806125.1	575	Bacteria>Proteobacteria>Gammaproteobacteria>Enterobacterales>Enterobacteriaceae	Salmonella_enterica_subsp._enterica_serovar_Kentucky	hypothetical-protein-E2J87_09035-[Salmonella-enterica-subsp.-enterica-serovar-Kentucky]	192955
EBO1754308.1	DndB-ParB-nuclease	GCA_006806245.1	575	Bacteria>Proteobacteria>Gammaproteobacteria>Enterobacterales>Enterobacteriaceae	Salmonella_enterica_subsp._enterica_serovar_Kentucky	hypothetical-protein-E2K40_23525-[Salmonella-enterica-subsp.-enterica-serovar-Kentucky]	192955
EBO2384514.1	DndB-ParB-nuclease	GCA_006809485.1	575	Bacteria>Proteobacteria>Gammaproteobacteria>Enterobacterales>Enterobacteriaceae	Salmonella_enterica_subsp._enterica_serovar_Kentucky	hypothetical-protein-E7V98_23270-[Salmonella-enterica-subsp.-enterica-serovar-Kentucky]	192955
EBM9086934.1	DndB-ParB-nuclease	GCA_006750885.1	575	Bacteria>Proteobacteria>Gammaproteobacteria>Enterobacterales>Enterobacteriaceae	Salmonella_enterica_subsp._enterica_serovar_Kentucky	hypothetical-protein-E8F33_21555-[Salmonella-enterica-subsp.-enterica-serovar-Kentucky]	192955
EBC9540812.1	DndB-ParB-nuclease	GCA_006211945.1	575	Bacteria>Proteobacteria>Gammaproteobacteria>Enterobacterales>Enterobacteriaceae	Salmonella_enterica_subsp._enterica_serovar_Kentucky	hypothetical-protein-EA481_08540-[Salmonella-enterica-subsp.-enterica-serovar-Kentucky]	192955
EBZ3382075.1	DndB-ParB-nuclease	GCA_007331095.1	575	Bacteria>Proteobacteria>Gammaproteobacteria>Enterobacterales>Enterobacteriaceae	Salmonella_enterica_subsp._enterica_serovar_Kentucky	hypothetical-protein-EAD72_23505-[Salmonella-enterica-subsp.-enterica-serovar-Kentucky]	192955
ECA3299802.1	DndB-ParB-nuclease	GCA_007375285.1	575	Bacteria>Proteobacteria>Gammaproteobacteria>Enterobacterales>Enterobacteriaceae	Salmonella_enterica_subsp._enterica_serovar_Kentucky	hypothetical-protein-EJP26_24305-[Salmonella-enterica-subsp.-enterica-serovar-Kentucky]	192955
EBC9817650.1	DndB-ParB-nuclease	GCA_006213385.1	575	Bacteria>Proteobacteria>Gammaproteobacteria>Enterobacterales>Enterobacteriaceae	Salmonella_enterica_subsp._enterica_serovar_Kentucky	hypothetical-protein-EQ497_08265-[Salmonella-enterica-subsp.-enterica-serovar-Kentucky]	192955
EBL3653316.1	DndB-ParB-nuclease	GCA_006682345.1	575	Bacteria>Proteobacteria>Gammaproteobacteria>Enterobacterales>Enterobacteriaceae	Salmonella_enterica_subsp._enterica_serovar_Kentucky	hypothetical-protein-EX441_14635-[Salmonella-enterica-subsp.-enterica-serovar-Kentucky]	192955
EBL4812220.1	DndB-ParB-nuclease	GCA_006688125.1	575	Bacteria>Proteobacteria>Gammaproteobacteria>Enterobacterales>Enterobacteriaceae	Salmonella_enterica_subsp._enterica_serovar_Kentucky	hypothetical-protein-FFW62_23950-[Salmonella-enterica-subsp.-enterica-serovar-Kentucky]	192955
TNF99121.1	DndB-ParB-nuclease	GCA_006227185.1	375	Bacteria>Proteobacteria>Gammaproteobacteria>Enterobacterales>Enterobacteriaceae	Salmonella_enterica_subsp._enterica_serovar_Kentucky	hypothetical-protein-FG050_21845,-partial-[Salmonella-enterica-subsp.-enterica-serovar-Kentucky]	192955
EEH9185136.1	DndB-ParB-nuclease	GCA_011311895.1	575	Bacteria>Proteobacteria>Gammaproteobacteria>Enterobacterales>Enterobacteriaceae	Salmonella_enterica_subsp._enterica_serovar_Kentucky	hypothetical-protein-FWX65_12935-[Salmonella-enterica-subsp.-enterica-serovar-Kentucky]	192955
EBL5311717.1	DndB-ParB-nuclease	GCA_006690085.1	575	Bacteria>Proteobacteria>Gammaproteobacteria>Enterobacterales>Enterobacteriaceae	Salmonella_enterica_subsp._enterica_serovar_Kentucky	hypothetical-protein-GN41_10895-[Salmonella-enterica-subsp.-enterica-serovar-Kentucky]	192955
EGD4038570.1	DndB-ParB-nuclease	GCA_014370525.1	575	Bacteria>Proteobacteria>Gammaproteobacteria>Enterobacterales>Enterobacteriaceae	Salmonella_enterica_subsp._enterica_serovar_Kentucky	hypothetical-protein-H2741_001546-[Salmonella-enterica-subsp.-enterica-serovar-Kentucky]	192955
EGG8235278.1	DndB-ParB-nuclease	GCA_014586675.1	575	Bacteria>Proteobacteria>Gammaproteobacteria>Enterobacterales>Enterobacteriaceae	Salmonella_enterica_subsp._enterica_serovar_Kentucky	hypothetical-protein-HH536_002169-[Salmonella-enterica-subsp.-enterica-serovar-Kentucky]	192955
EFU1700396.1	DndB-ParB-nuclease	GCA_013736855.1	575	Bacteria>Proteobacteria>Gammaproteobacteria>Enterobacterales>Enterobacteriaceae	Salmonella_enterica_subsp._enterica_serovar_Kentucky	hypothetical-protein-HP778_000102-[Salmonella-enterica-subsp.-enterica-serovar-Kentucky]	192955
EGI4860831.1	DndB-ParB-nuclease	GCA_014751225.1	575	Bacteria>Proteobacteria>Gammaproteobacteria>Enterobacterales>Enterobacteriaceae	Salmonella_enterica_subsp._enterica_serovar_Kentucky	hypothetical-protein-HRZ72_001722-[Salmonella-enterica-subsp.-enterica-serovar-Kentucky]	192955
EGR6456136.1	DndB-ParB-nuclease	GCA_015822845.1	548	Bacteria>Proteobacteria>Gammaproteobacteria>Enterobacterales>Enterobacteriaceae	Salmonella_enterica_subsp._enterica_serovar_Kentucky	hypothetical-protein-I6Q42_004758,-partial-[Salmonella-enterica-subsp.-enterica-serovar-Kentucky]	192955
EEK0756068.1	DndB-ParB-nuclease	GCA_011453575.1	575	Bacteria>Proteobacteria>Gammaproteobacteria>Enterobacterales>Enterobacteriaceae	Salmonella_enterica_subsp._enterica_serovar_Kentucky	hypothetical-protein-IL70_07200-[Salmonella-enterica-subsp.-enterica-serovar-Kentucky]	192955
EGK6826837.1	DndB-ParB-nuclease	GCA_014952605.1	575	Bacteria>Proteobacteria>Gammaproteobacteria>Enterobacterales>Enterobacteriaceae	Salmonella_enterica_subsp._enterica_serovar_Kentucky	hypothetical-protein-IO665_002659-[Salmonella-enterica-subsp.-enterica-serovar-Kentucky]	192955
EHO8033140.1	DndB-ParB-nuclease	GCA_018582625.1	575	Bacteria>Proteobacteria>Gammaproteobacteria>Enterobacterales>Enterobacteriaceae	Salmonella_enterica_subsp._enterica_serovar_Kentucky	hypothetical-protein-KJA11_004336-[Salmonella-enterica-subsp.-enterica-serovar-Kentucky]	192955
EBL5191440.1	DndB-ParB-nuclease	GCA_006689725.1	575	Bacteria>Proteobacteria>Gammaproteobacteria>Enterobacterales>Enterobacteriaceae	Salmonella_enterica_subsp._enterica_serovar_Kentucky	hypothetical-protein-KX23_06300-[Salmonella-enterica-subsp.-enterica-serovar-Kentucky]	192955
EHT2592568.1	DndB-ParB-nuclease	GCA_019199355.1	575	Bacteria>Proteobacteria>Gammaproteobacteria>Enterobacterales>Enterobacteriaceae	Salmonella_enterica_subsp._enterica_serovar_Kentucky	hypothetical-protein-KXB25_004500-[Salmonella-enterica-subsp.-enterica-serovar-Kentucky]	192955
EIJ5959349.1	DndB-ParB-nuclease	GCA_020547245.1	575	Bacteria>Proteobacteria>Gammaproteobacteria>Enterobacterales>Enterobacteriaceae	Salmonella_enterica_subsp._enterica_serovar_Kentucky	hypothetical-protein-LJI98_003970-[Salmonella-enterica-subsp.-enterica-serovar-Kentucky]	192955
EIL5300226.1	DndB-ParB-nuclease	GCA_020701515.1	575	Bacteria>Proteobacteria>Gammaproteobacteria>Enterobacterales>Enterobacteriaceae	Salmonella_enterica_subsp._enterica_serovar_Kentucky	hypothetical-protein-LLT78_001020-[Salmonella-enterica-subsp.-enterica-serovar-Kentucky]	192955
EEL9418973.1	DndB-ParB-nuclease	GCA_011545785.1	575	Bacteria>Proteobacteria>Gammaproteobacteria>Enterobacterales>Enterobacteriaceae	Salmonella_enterica_subsp._enterica_serovar_Kentucky	hypothetical-protein-S691_07715-[Salmonella-enterica-subsp.-enterica-serovar-Kentucky]	192955
ECJ5209099.1	DndB-ParB-nuclease	GCA_007823445.1	480	Bacteria>Proteobacteria>Gammaproteobacteria>Enterobacterales>Enterobacteriaceae	Salmonella_enterica_subsp._enterica_serovar_Kentucky	hypothetical-protein-[Salmonella-enterica-subsp.-enterica-serovar-Kentucky]	192955
EIC9384099.1	DndB-ParB-nuclease	GCA_020000505.1	415	Bacteria>Proteobacteria>Gammaproteobacteria>Enterobacterales>Enterobacteriaceae	Salmonella_enterica_subsp._enterica_serovar_Kentucky	hypothetical-protein-[Salmonella-enterica-subsp.-enterica-serovar-Kentucky]	192955
ECD2671689.1	DndB-ParB-nuclease	GCA_007515885.1	361	Bacteria>Proteobacteria>Gammaproteobacteria>Enterobacterales>Enterobacteriaceae	Salmonella_enterica_subsp._enterica_serovar_Korlebu	DNA-sulfur-modification-protein-DndB-[Salmonella-enterica-subsp.-enterica-serovar-Korlebu]	2564642
EEE2001775.1	DndB-ParB-nuclease	GCA_011120725.1	575	Bacteria>Proteobacteria>Gammaproteobacteria>Enterobacterales>Enterobacteriaceae	Salmonella_enterica_subsp._enterica_serovar_Kotte	hypothetical-protein-[Salmonella-enterica-subsp.-enterica-serovar-Kotte]	2048769
EBX5570122.1	DndB-ParB-nuclease	GCA_007259425.1	361	Bacteria>Proteobacteria>Gammaproteobacteria>Enterobacterales>Enterobacteriaceae	Salmonella_enterica_subsp._enterica_serovar_Kuessel	DNA-sulfur-modification-protein-DndB-[Salmonella-enterica-subsp.-enterica-serovar-Kuessel]	2564653
EDQ3448686.1	DndB-ParB-nuclease	GCA_010394745.1	402	Bacteria>Proteobacteria>Gammaproteobacteria>Enterobacterales>Enterobacteriaceae	Salmonella_enterica_subsp._enterica_serovar_Larochelle	DGQHR-domain-containing-protein-[Salmonella-enterica-subsp.-enterica-serovar-Larochelle]	2306633
EDQ3448682.1	DndB-ParB-nuclease	GCA_010394745.1	361	Bacteria>Proteobacteria>Gammaproteobacteria>Enterobacterales>Enterobacteriaceae	Salmonella_enterica_subsp._enterica_serovar_Larochelle	DNA-sulfur-modification-protein-DndB-[Salmonella-enterica-subsp.-enterica-serovar-Larochelle]	2306633
EBX2840923.1	DndB-ParB-nuclease	GCA_007246535.1	361	Bacteria>Proteobacteria>Gammaproteobacteria>Enterobacterales>Enterobacteriaceae	Salmonella_enterica_subsp._enterica_serovar_Leeuwarden	DNA-sulfur-modification-protein-DndB-[Salmonella-enterica-subsp.-enterica-serovar-Leeuwarden]	2564678
EBU9874996.1	DndB-ParB-nuclease	GCA_007151785.1	361	Bacteria>Proteobacteria>Gammaproteobacteria>Enterobacterales>Enterobacteriaceae	Salmonella_enterica_subsp._enterica_serovar_Livingstone	DNA-sulfur-modification-protein-DndB-[Salmonella-enterica-subsp.-enterica-serovar-Livingstone]	260680
ECA5305116.1	DndB-ParB-nuclease	GCA_007384565.1	361	Bacteria>Proteobacteria>Gammaproteobacteria>Enterobacterales>Enterobacteriaceae	Salmonella_enterica_subsp._enterica_serovar_Miami	DNA-sulfur-modification-protein-DndB-[Salmonella-enterica-subsp.-enterica-serovar-Miami]	286780
EIF2896256.1	DndB-ParB-nuclease	GCA_020239425.1	415	Bacteria>Proteobacteria>Gammaproteobacteria>Enterobacterales>Enterobacteriaceae	Salmonella_enterica_subsp._enterica_serovar_Mishmarhaemek	DGQHR-domain-containing-protein-[Salmonella-enterica-subsp.-enterica-serovar-Mishmarhaemek]	1629955
EIF2896261.1	DndB-ParB-nuclease	GCA_020239425.1	361	Bacteria>Proteobacteria>Gammaproteobacteria>Enterobacterales>Enterobacteriaceae	Salmonella_enterica_subsp._enterica_serovar_Mishmarhaemek	DNA-sulfur-modification-protein-DndB-[Salmonella-enterica-subsp.-enterica-serovar-Mishmarhaemek]	1629955
EBS3175636.1	DndB-ParB-nuclease	GCA_006998125.1	382	Bacteria>Proteobacteria>Gammaproteobacteria>Enterobacterales>Enterobacteriaceae	Salmonella_enterica_subsp._enterica_serovar_Newport	DGQHR-domain-containing-protein-[Salmonella-enterica-subsp.-enterica-serovar-Newport]	108619
EBY7427504.1	DndB-ParB-nuclease	GCA_007304735.1	379	Bacteria>Proteobacteria>Gammaproteobacteria>Enterobacterales>Enterobacteriaceae	Salmonella_enterica_subsp._enterica_serovar_Newport	DGQHR-domain-containing-protein-[Salmonella-enterica-subsp.-enterica-serovar-Newport]	108619
ECA5259056.1	DndB-ParB-nuclease	GCA_007385425.1	329	Bacteria>Proteobacteria>Gammaproteobacteria>Enterobacterales>Enterobacteriaceae	Salmonella_enterica_subsp._enterica_serovar_Newport	DGQHR-domain-containing-protein-[Salmonella-enterica-subsp.-enterica-serovar-Newport]	108619
ECN7187350.1	DndB-ParB-nuclease	GCA_008098265.1	329	Bacteria>Proteobacteria>Gammaproteobacteria>Enterobacterales>Enterobacteriaceae	Salmonella_enterica_subsp._enterica_serovar_Newport	DGQHR-domain-containing-protein-[Salmonella-enterica-subsp.-enterica-serovar-Newport]	108619
EEB8831403.1	DndB-ParB-nuclease	GCA_010960465.1	250	Bacteria>Proteobacteria>Gammaproteobacteria>Enterobacterales>Enterobacteriaceae	Salmonella_enterica_subsp._enterica_serovar_Newport	DGQHR-domain-containing-protein-[Salmonella-enterica-subsp.-enterica-serovar-Newport]	108619
EBS3175640.1	DndB-ParB-nuclease	GCA_006998125.1	361	Bacteria>Proteobacteria>Gammaproteobacteria>Enterobacterales>Enterobacteriaceae	Salmonella_enterica_subsp._enterica_serovar_Newport	DNA-sulfur-modification-protein-DndB-[Salmonella-enterica-subsp.-enterica-serovar-Newport]	108619
EBY7427516.1	DndB-ParB-nuclease	GCA_007304735.1	361	Bacteria>Proteobacteria>Gammaproteobacteria>Enterobacterales>Enterobacteriaceae	Salmonella_enterica_subsp._enterica_serovar_Newport	DNA-sulfur-modification-protein-DndB-[Salmonella-enterica-subsp.-enterica-serovar-Newport]	108619
ECA5259054.1	DndB-ParB-nuclease	GCA_007385425.1	359	Bacteria>Proteobacteria>Gammaproteobacteria>Enterobacterales>Enterobacteriaceae	Salmonella_enterica_subsp._enterica_serovar_Newport	DNA-sulfur-modification-protein-DndB-[Salmonella-enterica-subsp.-enterica-serovar-Newport]	108619
ECN7187348.1	DndB-ParB-nuclease	GCA_008098265.1	359	Bacteria>Proteobacteria>Gammaproteobacteria>Enterobacterales>Enterobacteriaceae	Salmonella_enterica_subsp._enterica_serovar_Newport	DNA-sulfur-modification-protein-DndB-[Salmonella-enterica-subsp.-enterica-serovar-Newport]	108619
EEB8831405.1	DndB-ParB-nuclease	GCA_010960465.1	359	Bacteria>Proteobacteria>Gammaproteobacteria>Enterobacterales>Enterobacteriaceae	Salmonella_enterica_subsp._enterica_serovar_Newport	DNA-sulfur-modification-protein-DndB-[Salmonella-enterica-subsp.-enterica-serovar-Newport]	108619
ECN7187343.1	DndB-ParB-nuclease	GCA_008098265.1	563	Bacteria>Proteobacteria>Gammaproteobacteria>Enterobacterales>Enterobacteriaceae	Salmonella_enterica_subsp._enterica_serovar_Newport	hypothetical-protein-ZJ17_09310-[Salmonella-enterica-subsp.-enterica-serovar-Newport]	108619
ECA7213746.1	DndB-ParB-nuclease	GCA_007390865.1	397	Bacteria>Proteobacteria>Gammaproteobacteria>Enterobacterales>Enterobacteriaceae	Salmonella_enterica_subsp._enterica_serovar_Ohio	DGQHR-domain-containing-protein-[Salmonella-enterica-subsp.-enterica-serovar-Ohio]	117541
ECA7213751.1	DndB-ParB-nuclease	GCA_007390865.1	361	Bacteria>Proteobacteria>Gammaproteobacteria>Enterobacterales>Enterobacteriaceae	Salmonella_enterica_subsp._enterica_serovar_Ohio	DNA-sulfur-modification-protein-DndB-[Salmonella-enterica-subsp.-enterica-serovar-Ohio]	117541
EDT6212355.1	DndB-ParB-nuclease	GCA_010553225.1	361	Bacteria>Proteobacteria>Gammaproteobacteria>Enterobacterales>Enterobacteriaceae	Salmonella_enterica_subsp._enterica_serovar_Ohio	DNA-sulfur-modification-protein-DndB-[Salmonella-enterica-subsp.-enterica-serovar-Ohio]	117541
HAE0856674.1	DndB-ParB-nuclease	GCA_010265245.1	397	Bacteria>Proteobacteria>Gammaproteobacteria>Enterobacterales>Enterobacteriaceae	Salmonella_enterica_subsp._enterica_serovar_Ohio	TPA_asm:-DGQHR-domain-containing-protein-[Salmonella-enterica-subsp.-enterica-serovar-Ohio]	117541
HAE0856669.1	DndB-ParB-nuclease	GCA_010265245.1	361	Bacteria>Proteobacteria>Gammaproteobacteria>Enterobacterales>Enterobacteriaceae	Salmonella_enterica_subsp._enterica_serovar_Ohio	TPA_asm:-DNA-sulfur-modification-protein-DndB-[Salmonella-enterica-subsp.-enterica-serovar-Ohio]	117541
EBV4202221.1	DndB-ParB-nuclease	GCA_007169755.1	395	Bacteria>Proteobacteria>Gammaproteobacteria>Enterobacterales>Enterobacteriaceae	Salmonella_enterica_subsp._enterica_serovar_Oslo	DGQHR-domain-containing-protein-[Salmonella-enterica-subsp.-enterica-serovar-Oslo]	1005394
EDQ6567822.1	DndB-ParB-nuclease	GCA_010407685.1	395	Bacteria>Proteobacteria>Gammaproteobacteria>Enterobacterales>Enterobacteriaceae	Salmonella_enterica_subsp._enterica_serovar_Oslo	DGQHR-domain-containing-protein-[Salmonella-enterica-subsp.-enterica-serovar-Oslo]	1005394

Accession	Annotation	GCA	Length	Phyletic.spread	Strain.name	Defline	Species.Taxid
EIC3317861.1	DndB-ParB-nuclease	GCA_019948415.1	385	Bacteria>Proteobacteria>Gammaproteobacteria>Enterobacterales>Enterobacteriaceae	Salmonella_enterica_subsp._enterica_serovar_Oslo	DGQHR-domain-containing-protein-[Salmonella-enterica-subsp.-enterica-serovar-Oslo]	1005394
EDQ6567818.1	DndB-ParB-nuclease	GCA_010407685.1	361	Bacteria>Proteobacteria>Gammaproteobacteria>Enterobacterales>Enterobacteriaceae	Salmonella_enterica_subsp._enterica_serovar_Oslo	DNA-sulfur-modification-protein-DndB-[Salmonella-enterica-subsp.-enterica-serovar-Oslo]	1005394
EIC3317857.1	DndB-ParB-nuclease	GCA_019948415.1	361	Bacteria>Proteobacteria>Gammaproteobacteria>Enterobacterales>Enterobacteriaceae	Salmonella_enterica_subsp._enterica_serovar_Oslo	DNA-sulfur-modification-protein-DndB-[Salmonella-enterica-subsp.-enterica-serovar-Oslo]	1005394
ECF0509062.1	DndB-ParB-nuclease	GCA_007597475.1	361	Bacteria>Proteobacteria>Gammaproteobacteria>Enterobacterales>Enterobacteriaceae	Salmonella_enterica_subsp._enterica_serovar_Panama	DNA-sulfur-modification-protein-DndB-[Salmonella-enterica-subsp.-enterica-serovar-Panama]	29472
HAT0112381.1	DndB-ParB-nuclease	GCA_015881405.1	361	Bacteria>Proteobacteria>Gammaproteobacteria>Enterobacterales>Enterobacteriaceae	Salmonella_enterica_subsp._enterica_serovar_Saintpaul	TPA:-DNA-sulfur-modification-protein-DndB-[Salmonella-enterica-subsp.-enterica-serovar-Saintpaul]	90105
EHK9152797.1	DndB-ParB-nuclease	GCA_018081325.1	386	Bacteria>Proteobacteria>Gammaproteobacteria>Enterobacterales>Enterobacteriaceae	Salmonella_enterica_subsp._enterica_serovar_Stanley	DGQHR-domain-containing-protein-[Salmonella-enterica-subsp.-enterica-serovar-Stanley]	192953
EHK9152793.1	DndB-ParB-nuclease	GCA_018081325.1	362	Bacteria>Proteobacteria>Gammaproteobacteria>Enterobacterales>Enterobacteriaceae	Salmonella_enterica_subsp._enterica_serovar_Stanley	DNA-sulfur-modification-protein-DndB-[Salmonella-enterica-subsp.-enterica-serovar-Stanley]	192953
HAU6779797.1	DndB-ParB-nuclease	GCA_016095455.1	575	Bacteria>Proteobacteria>Gammaproteobacteria>Enterobacterales>Enterobacteriaceae	Salmonella_enterica_subsp._enterica_serovar_Tado	hypothetical-protein-[Salmonella-enterica-subsp.-enterica-serovar-Tado]	2565074
KSU45721.1	DndB-ParB-nuclease	GCA_001456375.1	361	Bacteria>Proteobacteria>Gammaproteobacteria>Enterobacterales>Enterobacteriaceae	Salmonella_enterica_subsp._enterica_serovar_Veneziana	DNA-sulfur-modification-protein-DndB-[Salmonella-enterica-subsp.-enterica]	2565153
EBY6258138.1	DndB-ParB-nuclease	GCA_007300615.1	361	Bacteria>Proteobacteria>Gammaproteobacteria>Enterobacterales>Enterobacteriaceae	Salmonella_enterica_subsp._enterica_serovar_Warnow	DNA-sulfur-modification-protein-DndB-[Salmonella-enterica-subsp.-enterica-serovar-Warnow]	2565179
EBX8652481.1	DndB-ParB-nuclease	GCA_007269975.1	361	Bacteria>Proteobacteria>Gammaproteobacteria>Enterobacterales>Enterobacteriaceae	Salmonella_enterica_subsp._enterica_serovar_Westhampton	DNA-sulfur-modification-protein-DndB-[Salmonella-enterica-subsp.-enterica-serovar-Westhampton]	356621
ECB7304915.1	DndB-ParB-nuclease	GCA_007438025.1	361	Bacteria>Proteobacteria>Gammaproteobacteria>Enterobacterales>Enterobacteriaceae	Salmonella_enterica_subsp._enterica_serovar_Yovokome	DNA-sulfur-modification-protein-DndB-[Salmonella-enterica-subsp.-enterica-serovar-Yovokome]	1243599
ECC1504578.1	DndB-ParB-nuclease	GCA_007462915.1	397	Bacteria>Proteobacteria>Gammaproteobacteria>Enterobacterales>Enterobacteriaceae	Salmonella_enterica_subsp._houtenae	DGQHR-domain-containing-protein-[Salmonella-enterica-subsp.-houtenae]	59205
ECC1504585.1	DndB-ParB-nuclease	GCA_007462915.1	379	Bacteria>Proteobacteria>Gammaproteobacteria>Enterobacterales>Enterobacteriaceae	Salmonella_enterica_subsp._houtenae	DGQHR-domain-containing-protein-[Salmonella-enterica-subsp.-houtenae]	59205
EGJ3413375.1	DndB-ParB-nuclease	GCA_014820065.1	397	Bacteria>Proteobacteria>Gammaproteobacteria>Enterobacterales>Enterobacteriaceae	Salmonella_enterica_subsp._houtenae	DGQHR-domain-containing-protein-[Salmonella-enterica-subsp.-houtenae]	59205
EAA5286901.1	DndB-ParB-nuclease	GCA_004182655.1	361	Bacteria>Proteobacteria>Gammaproteobacteria>Enterobacterales>Enterobacteriaceae	Salmonella_enterica_subsp._houtenae	DNA-sulfur-modification-protein-DndB-[Salmonella-enterica-subsp.-houtenae]	59205
ECC1504573.1	DndB-ParB-nuclease	GCA_007462915.1	361	Bacteria>Proteobacteria>Gammaproteobacteria>Enterobacterales>Enterobacteriaceae	Salmonella_enterica_subsp._houtenae	DNA-sulfur-modification-protein-DndB-[Salmonella-enterica-subsp.-houtenae]	59205
EGJ3413380.1	DndB-ParB-nuclease	GCA_014820065.1	361	Bacteria>Proteobacteria>Gammaproteobacteria>Enterobacterales>Enterobacteriaceae	Salmonella_enterica_subsp._houtenae	DNA-sulfur-modification-protein-DndB-[Salmonella-enterica-subsp.-houtenae]	59205
HBC0145593.1	DndB-ParB-nuclease	GCA_018033065.1	562	Bacteria>Proteobacteria>Gammaproteobacteria>Enterobacterales>Enterobacteriaceae	Salmonella_enterica_subsp._houtenae	hypothetical-protein-[Salmonella-enterica-subsp.-houtenae]	59205
HBC0145588.1	DndB-ParB-nuclease	GCA_018033065.1	359	Bacteria>Proteobacteria>Gammaproteobacteria>Enterobacterales>Enterobacteriaceae	Salmonella_enterica_subsp._houtenae	TPA:-DNA-sulfur-modification-protein-DndB-[Salmonella-enterica-subsp.-houtenae]	59205
ECG8513411.1	DndB-ParB-nuclease	GCA_007695765.1	405	Bacteria>Proteobacteria>Gammaproteobacteria>Enterobacterales>Enterobacteriaceae	Salmonella_enterica_subsp._salamae	DGQHR-domain-containing-protein-[Salmonella-enterica-subsp.-salamae]	59202
EEN7281760.1	DndB-ParB-nuclease	GCA_011634405.1	389	Bacteria>Proteobacteria>Gammaproteobacteria>Enterobacterales>Enterobacteriaceae	Salmonella_enterica_subsp._salamae	DGQHR-domain-containing-protein-[Salmonella-enterica-subsp.-salamae]	59202
ECC9154007.1	DndB-ParB-nuclease	GCA_007500425.1	359	Bacteria>Proteobacteria>Gammaproteobacteria>Enterobacterales>Enterobacteriaceae	Salmonella_enterica_subsp._salamae	DNA-sulfur-modification-protein-DndB-[Salmonella-enterica-subsp.-salamae]	59202
ECG8513416.1	DndB-ParB-nuclease	GCA_007695765.1	361	Bacteria>Proteobacteria>Gammaproteobacteria>Enterobacterales>Enterobacteriaceae	Salmonella_enterica_subsp._salamae	DNA-sulfur-modification-protein-DndB-[Salmonella-enterica-subsp.-salamae]	59202
ECG8630373.1	DndB-ParB-nuclease	GCA_007695825.1	361	Bacteria>Proteobacteria>Gammaproteobacteria>Enterobacterales>Enterobacteriaceae	Salmonella_enterica_subsp._salamae	DNA-sulfur-modification-protein-DndB-[Salmonella-enterica-subsp.-salamae]	59202
ECI4644830.1	DndB-ParB-nuclease	GCA_007775225.1	361	Bacteria>Proteobacteria>Gammaproteobacteria>Enterobacterales>Enterobacteriaceae	Salmonella_enterica_subsp._salamae	DNA-sulfur-modification-protein-DndB-[Salmonella-enterica-subsp.-salamae]	59202
ECJ5917127.1	DndB-ParB-nuclease	GCA_007826285.1	361	Bacteria>Proteobacteria>Gammaproteobacteria>Enterobacterales>Enterobacteriaceae	Salmonella_enterica_subsp._salamae	DNA-sulfur-modification-protein-DndB-[Salmonella-enterica-subsp.-salamae]	59202
EEN7281765.1	DndB-ParB-nuclease	GCA_011634405.1	361	Bacteria>Proteobacteria>Gammaproteobacteria>Enterobacterales>Enterobacteriaceae	Salmonella_enterica_subsp._salamae	DNA-sulfur-modification-protein-DndB-[Salmonella-enterica-subsp.-salamae]	59202
ECC9154005.1	DndB-ParB-nuclease	GCA_007500425.1	329	Bacteria>Proteobacteria>Gammaproteobacteria>Enterobacterales>Enterobacteriaceae	Salmonella_enterica_subsp._salamae	hypothetical-protein-[Salmonella-enterica-subsp.-salamae]	59202
ECC9154012.1	DndB-ParB-nuclease	GCA_007500425.1	562	Bacteria>Proteobacteria>Gammaproteobacteria>Enterobacterales>Enterobacteriaceae	Salmonella_enterica_subsp._salamae	hypothetical-protein-[Salmonella-enterica-subsp.-salamae]	59202
ECF6031760.1	DndB-ParB-nuclease	GCA_007620915.1	575	Bacteria>Proteobacteria>Gammaproteobacteria>Enterobacterales>Enterobacteriaceae	Salmonella_enterica_subsp._salamae	hypothetical-protein-[Salmonella-enterica-subsp.-salamae]	59202
HAF5505244.1	DndB-ParB-nuclease	GCA_011304775.1	361	Bacteria>Proteobacteria>Gammaproteobacteria>Enterobacterales>Enterobacteriaceae	Salmonella_enterica	TPA:-DNA-sulfur-modification-protein-DndB-[Salmonella-enterica]	28901
HAK2173732.1	DndB-ParB-nuclease	GCA_013536055.1	361	Bacteria>Proteobacteria>Gammaproteobacteria>Enterobacterales>Enterobacteriaceae	Salmonella_enterica	TPA:-DNA-sulfur-modification-protein-DndB-[Salmonella-enterica]	28901
HAK8390318.1	DndB-ParB-nuclease	GCA_013645515.1	361	Bacteria>Proteobacteria>Gammaproteobacteria>Enterobacterales>Enterobacteriaceae	Salmonella_enterica	TPA:-DNA-sulfur-modification-protein-DndB-[Salmonella-enterica]	28901
HAV1236091.1	DndB-ParB-nuclease	GCA_016344635.1	361	Bacteria>Proteobacteria>Gammaproteobacteria>Enterobacterales>Enterobacteriaceae	Salmonella_enterica	TPA:-DNA-sulfur-modification-protein-DndB-[Salmonella-enterica]	28901
WP_071786425.1	DndB-ParB-nuclease	GCF_000487455.1	385	Bacteria>Proteobacteria>Gammaproteobacteria>Enterobacterales>Enterobacteriaceae	Salmonella	MULTISPECIES:-DGQHR-domain-containing-protein-[Salmonella]	590
WP_001520283.1	DndB-ParB-nuclease	GCF_000170215.1	361	Bacteria>Proteobacteria>Gammaproteobacteria>Enterobacterales>Enterobacteriaceae	Salmonella	MULTISPECIES:-DNA-sulfur-modification-protein-DndB-[Salmonella]	590
WP_175067181.1	DndB-ParB-nuclease	GCF_013350485.1	361	Bacteria>Proteobacteria>Gammaproteobacteria>Enterobacterales>Enterobacteriaceae	Salmonella_sp._SG193	DNA-sulfur-modification-protein-DndB-[Salmonella-sp.-SG193]	2555402
WP_054178972.1	DndB-ParB-nuclease	GCF_001297845.1	577	Bacteria>Proteobacteria>Gammaproteobacteria>Enterobacterales>Enterobacteriaceae	Trabulsiella_odontotermitis	hypothetical-protein-[Trabulsiella-odontotermitis]	379893
WP_193042681.1	DndB-ParB-nuclease	GCF_014896025.1	361	Bacteria>Proteobacteria>Gammaproteobacteria>Enterobacterales>Enterobacteriaceae	unclassified_Enterobacter_cloacae_complex	MULTISPECIES:-DNA-sulfur-modification-protein-DndB-[unclassified-Enterobacter-cloacae-complex]	2757714
WP_159515434.1	DndB-ParB-nuclease	GCF_009811955.1	402	Bacteria>Proteobacteria>Gammaproteobacteria>Enterobacterales>Enterobacteriaceae	unclassified_Enterobacter	MULTISPECIES:-DGQHR-domain-containing-protein-[unclassified-Enterobacter]	2608935
WP_159515437.1	DndB-ParB-nuclease	GCF_009811955.1	361	Bacteria>Proteobacteria>Gammaproteobacteria>Enterobacterales>Enterobacteriaceae	unclassified_Enterobacter	MULTISPECIES:-DNA-sulfur-modification-protein-DndB-[unclassified-Enterobacter]	2608935
WP_105269615.1	DndB-ParB-nuclease	GCF_013368815.2	404	Bacteria>Proteobacteria>Gammaproteobacteria>Enterobacterales>Enterobacteriaceae	unclassified_Escherichia	MULTISPECIES:-DNA-phosphorothioation-associated-DGQHR-protein-1-[unclassified-Escherichia]	2608889
WP_105269598.1	DndB-ParB-nuclease	GCF_013368815.2	361	Bacteria>Proteobacteria>Gammaproteobacteria>Enterobacterales>Enterobacteriaceae	unclassified_Escherichia	MULTISPECIES:-DNA-sulfur-modification-protein-DndB-[unclassified-Escherichia]	2608889
WP_120817102.1	DndB-ParB-nuclease	GCF_900706795.1	361	Bacteria>Proteobacteria>Gammaproteobacteria>Enterobacterales>Enterobacteriaceae	Yokenella_regensburgei	DNA-sulfur-modification-protein-DndB-[Yokenella-regensburgei]	158877
KMV72650.1	DndB-ParB-nuclease	GCA_001078075.1	361	Bacteria>Proteobacteria>Gammaproteobacteria>Enterobacterales>Erwiniaceae	bacteria_symbiont_BFo1_of_Frankliniella_occidentalis	DNA-sulfur-modification-protein-DndB-[bacteria-symbiont-BFo1-of-Frankliniella-occidentalis]	1628855
KYP86733.1	DndB-ParB-nuclease	GCA_001602545.1	361	Bacteria>Proteobacteria>Gammaproteobacteria>Enterobacterales>Erwiniaceae	bacteria_symbiont_BFo1_of_Frankliniella_occidentalis	DNA-sulfur-modification-protein-DndB-[bacteria-symbiont-BFo1-of-Frankliniella-occidentalis]	1628855
CBX79209.1	DndB-ParB-nuclease	-	361	Bacteria>Proteobacteria>Gammaproteobacteria>Enterobacterales>Erwiniaceae	Erwinia_amylovora_ATCC_BAA-2158	hypothetical-protein-EAIL5_0389-[Erwinia-amylovora-ATCC-BAA-2158]	889211
WP_004155179.1	DndB-ParB-nuclease	GCF_000091565.1	361	Bacteria>Proteobacteria>Gammaproteobacteria>Enterobacterales>Erwiniaceae	Erwinia_amylovora	DNA-sulfur-modification-protein-DndB-[Erwinia-amylovora]	552
WP_206403434.1	DndB-ParB-nuclease	GCF_017168115.1	361	Bacteria>Proteobacteria>Gammaproteobacteria>Enterobacterales>Erwiniaceae	Erwinia_billingiae	DNA-sulfur-modification-protein-DndB-[Erwinia-billingiae]	182337
WP_072277439.1	DndB-ParB-nuclease	GCF_001571305.1	415	Bacteria>Proteobacteria>Gammaproteobacteria>Enterobacterales>Erwiniaceae	Erwinia_persicina	DGQHR-domain-containing-protein-[Erwinia-persicina]	55211
WP_062744413.1	DndB-ParB-nuclease	GCF_001571305.1	361	Bacteria>Proteobacteria>Gammaproteobacteria>Enterobacterales>Erwiniaceae	Erwinia_persicina	DNA-sulfur-modification-protein-DndB-[Erwinia-persicina]	55211
WP_141174850.1	DndB-ParB-nuclease	GCF_006517625.1	387	Bacteria>Proteobacteria>Gammaproteobacteria>Enterobacterales>Erwiniaceae	Mixta_tenebrionis	DGQHR-domain-containing-protein-[Mixta-tenebrionis]	2562439
WP_141174853.1	DndB-ParB-nuclease	GCF_006517625.1	361	Bacteria>Proteobacteria>Gammaproteobacteria>Enterobacterales>Erwiniaceae	Mixta_tenebrionis	DNA-sulfur-modification-protein-DndB-[Mixta-tenebrionis]	2562439
WP_045140820.1	DndB-ParB-nuclease	GCF_000952095.1	567	Bacteria>Proteobacteria>Gammaproteobacteria>Enterobacterales>Erwiniaceae	Pantoea_agglomerans	AIPR-family-protein-[Pantoea-agglomerans]	549
WP_207254244.1	DndB-ParB-nuclease	GCF_017348995.1	381	Bacteria>Proteobacteria>Gammaproteobacteria>Enterobacterales>Erwiniaceae	Pantoea_agglomerans	DGQHR-domain-containing-protein-[Pantoea-agglomerans]	549
WP_045140816.1	DndB-ParB-nuclease	GCF_004136415.1	361	Bacteria>Proteobacteria>Gammaproteobacteria>Enterobacterales>Erwiniaceae	Pantoea_agglomerans	DNA-sulfur-modification-protein-DndB-[Pantoea-agglomerans]	549
WP_187492258.1	DndB-ParB-nuclease	GCF_014354065.1	361	Bacteria>Proteobacteria>Gammaproteobacteria>Enterobacterales>Erwiniaceae	Pantoea_agglomerans	DNA-sulfur-modification-protein-DndB-[Pantoea-agglomerans]	549
WP_145031600.1	DndB-ParB-nuclease	-	388	Bacteria>Proteobacteria>Gammaproteobacteria>Enterobacterales>Erwiniaceae	Pantoea_ananatis	DGQHR-domain-containing-protein-[Pantoea-ananatis]	553
WP_210446414.1	DndB-ParB-nuclease	GCF_017920255.1	397	Bacteria>Proteobacteria>Gammaproteobacteria>Enterobacterales>Erwiniaceae	Pantoea_ananatis	DGQHR-domain-containing-protein-[Pantoea-ananatis]	553
WP_210522864.1	DndB-ParB-nuclease	GCF_017920475.1	389	Bacteria>Proteobacteria>Gammaproteobacteria>Enterobacterales>Erwiniaceae	Pantoea_ananatis	DGQHR-domain-containing-protein-[Pantoea-ananatis]	553
WP_089517527.1	DndB-ParB-nuclease	GCF_002224585.2	404	Bacteria>Proteobacteria>Gammaproteobacteria>Enterobacterales>Erwiniaceae	Pantoea_ananatis	DNA-phosphorothioation-associated-DGQHR-protein-1-[Pantoea-ananatis]	553
WP_089517523.1	DndB-ParB-nuclease	GCF_002224585.2	361	Bacteria>Proteobacteria>Gammaproteobacteria>Enterobacterales>Erwiniaceae	Pantoea_ananatis	DNA-sulfur-modification-protein-DndB-[Pantoea-ananatis]	553
WP_105080456.1	DndB-ParB-nuclease	GCF_017920435.1	361	Bacteria>Proteobacteria>Gammaproteobacteria>Enterobacterales>Erwiniaceae	Pantoea_ananatis	DNA-sulfur-modification-protein-DndB-[Pantoea-ananatis]	553
WP_145031418.1	DndB-ParB-nuclease	GCF_007665685.1	361	Bacteria>Proteobacteria>Gammaproteobacteria>Enterobacterales>Erwiniaceae	Pantoea_ananatis	DNA-sulfur-modification-protein-DndB-[Pantoea-ananatis]	553
WP_210446410.1	DndB-ParB-nuclease	GCF_017920255.1	361	Bacteria>Proteobacteria>Gammaproteobacteria>Enterobacterales>Erwiniaceae	Pantoea_ananatis	DNA-sulfur-modification-protein-DndB-[Pantoea-ananatis]	553
WP_105080445.1	DndB-ParB-nuclease	GCF_017920435.1	704	Bacteria>Proteobacteria>Gammaproteobacteria>Enterobacterales>Erwiniaceae	Pantoea_ananatis	hypothetical-protein-[Pantoea-ananatis]	553
WP_128084107.1	DndB-ParB-nuclease	GCF_002095375.1	567	Bacteria>Proteobacteria>Gammaproteobacteria>Enterobacterales>Erwiniaceae	Pantoea_deleyi	AIPR-family-protein-[Pantoea-deleyi]	470932
WP_128084099.1	DndB-ParB-nuclease	GCF_022647325.1	361	Bacteria>Proteobacteria>Gammaproteobacteria>Enterobacterales>Erwiniaceae	Pantoea_deleyi	DNA-sulfur-modification-protein-DndB-[Pantoea-deleyi]	470932
MBZ6390429.1	DndB-ParB-nuclease	GCA_020092975.1	403	Bacteria>Proteobacteria>Gammaproteobacteria>Enterobacterales>Erwiniaceae	Pantoea_dispersa	DNA-phosphorothioation-associated-DGQHR-protein-1-[Pantoea-dispersa]	59814
WP_211725745.1	DndB-ParB-nuclease	GCF_018257625.1	403	Bacteria>Proteobacteria>Gammaproteobacteria>Enterobacterales>Erwiniaceae	Pantoea_dispersa	DNA-phosphorothioation-associated-DGQHR-protein-1-[Pantoea-dispersa]	59814
MBZ6390425.1	DndB-ParB-nuclease	GCA_020092975.1	361	Bacteria>Proteobacteria>Gammaproteobacteria>Enterobacterales>Erwiniaceae	Pantoea_dispersa	DNA-sulfur-modification-protein-DndB-[Pantoea-dispersa]	59814
WP_211725741.1	DndB-ParB-nuclease	GCF_018257625.1	361	Bacteria>Proteobacteria>Gammaproteobacteria>Enterobacterales>Erwiniaceae	Pantoea_dispersa	DNA-sulfur-modification-protein-DndB-[Pantoea-dispersa]	59814
WP_084935446.1	DndB-ParB-nuclease	GCF_002095475.1	231	Bacteria>Proteobacteria>Gammaproteobacteria>Enterobacterales>Erwiniaceae	Pantoea_rwandensis	DGQHR-domain-containing-protein-[Pantoea-rwandensis]	1076550
WP_084935442.1	DndB-ParB-nuclease	GCF_002095475.1	361	Bacteria>Proteobacteria>Gammaproteobacteria>Enterobacterales>Erwiniaceae	Pantoea_rwandensis	DNA-sulfur-modification-protein-DndB-[Pantoea-rwandensis]	1076550
WP_167429867.1	DndB-ParB-nuclease	GCF_011800685.1	361	Bacteria>Proteobacteria>Gammaproteobacteria>Enterobacterales>Erwiniaceae	Pantoea_sp._EKM10T	DNA-sulfur-modification-protein-DndB-[Pantoea-sp.-EKM10T]	2708058
WP_167429857.1	DndB-ParB-nuclease	GCF_011800685.1	685	Bacteria>Proteobacteria>Gammaproteobacteria>Enterobacterales>Erwiniaceae	Pantoea_sp._EKM10T	hypothetical-protein-[Pantoea-sp.-EKM10T]	2708058
MXP49291.1	DndB-ParB-nuclease	GCA_009830035.1	204	Bacteria>Proteobacteria>Gammaproteobacteria>Enterobacterales>Erwiniaceae	Pantoea_sp._Eser	hypothetical-protein-[Pantoea-sp.-Eser]	2576758
MXP52006.1	DndB-ParB-nuclease	GCA_009829935.1	361	Bacteria>Proteobacteria>Gammaproteobacteria>Enterobacterales>Erwiniaceae	Pantoea_sp._Seng	DNA-sulfur-modification-protein-DndB-[Pantoea-sp.-Seng]	2576761
WP_160832644.1	DndB-ParB-nuclease	GCF_009829935.1	361	Bacteria>Proteobacteria>Gammaproteobacteria>Enterobacterales>Erwiniaceae	Pantoea_sp._Seng	DNA-sulfur-modification-protein-DndB-[Pantoea-sp.-Seng]	2576761
WP_088516824.1	DndB-ParB-nuclease	GCF_002207995.1	361	Bacteria>Proteobacteria>Gammaproteobacteria>Enterobacterales>Erwiniaceae	Pantoea_sp._VS1	DNA-sulfur-modification-protein-DndB-[Pantoea-sp.-VS1]	2003658
WP_179897475.1	DndB-ParB-nuclease	GCF_013415305.1	361	Bacteria>Proteobacteria>Gammaproteobacteria>Enterobacterales>Erwiniaceae	Pantoea_sp._WMus005	DNA-sulfur-modification-protein-DndB-[Pantoea-sp.-WMus005]	2750734
TPE16810.1	DndB-ParB-nuclease	GCA_006385265.1	361	Bacteria>Proteobacteria>Gammaproteobacteria>Enterobacterales>Erwiniaceae	Pantoea_vagans	DNA-sulfur-modification-protein-DndB-[Pantoea-vagans]	470934
WP_140028814.1	DndB-ParB-nuclease	GCF_006385265.1	361	Bacteria>Proteobacteria>Gammaproteobacteria>Enterobacterales>Erwiniaceae	Pantoea_vagans	DNA-sulfur-modification-protein-DndB-[Pantoea-vagans]	470934
WP_109728483.1	DndB-ParB-nuclease	GCF_003113475.2	388	Bacteria>Proteobacteria>Gammaproteobacteria>Enterobacterales>Hafniaceae	Edwardsiella_tarda	DGQHR-domain-containing-protein-[Edwardsiella-tarda]	636
WP_109728487.1	DndB-ParB-nuclease	GCF_003113475.2	361	Bacteria>Proteobacteria>Gammaproteobacteria>Enterobacterales>Hafniaceae	Edwardsiella_tarda	DNA-sulfur-modification-protein-DndB-[Edwardsiella-tarda]	636
WP_123567794.1	DndB-ParB-nuclease	GCF_003730795.1	405	Bacteria>Proteobacteria>Gammaproteobacteria>Enterobacterales>Morganellaceae	Morganella_morganii	DGQHR-domain-containing-protein-[Morganella-morganii]	582
MBC6659179.1	DndB-ParB-nuclease	GCA_014333575.1	361	Bacteria>Proteobacteria>Gammaproteobacteria>Enterobacterales>Morganellaceae	Morganella_morganii	DNA-sulfur-modification-protein-DndB-[Morganella-morganii]	582
WP_123567803.1	DndB-ParB-nuclease	GCF_003730795.1	361	Bacteria>Proteobacteria>Gammaproteobacteria>Enterobacterales>Morganellaceae	Morganella_morganii	DNA-sulfur-modification-protein-DndB-[Morganella-morganii]	582
WP_214168472.1	DndB-ParB-nuclease	GCF_018802565.1	361	Bacteria>Proteobacteria>Gammaproteobacteria>Enterobacterales>Morganellaceae	Morganella_morganii	DNA-sulfur-modification-protein-DndB-[Morganella-morganii]	582
WP_214191433.1	DndB-ParB-nuclease	GCF_018802525.1	361	Bacteria>Proteobacteria>Gammaproteobacteria>Enterobacterales>Morganellaceae	Morganella_morganii	DNA-sulfur-modification-protein-DndB-[Morganella-morganii]	582
WP_222839889.1	DndB-ParB-nuclease	-	366	Bacteria>Proteobacteria>Gammaproteobacteria>Enterobacterales>Morganellaceae	Morganella_morganii	DNA-sulfur-modification-protein-DndB-[Morganella-morganii]	582
HBC7441569.1	DndB-ParB-nuclease	GCA_018165895.1	361	Bacteria>Proteobacteria>Gammaproteobacteria>Enterobacterales>Morganellaceae	Morganella_morganii	TPA:-DNA-sulfur-modification-protein-DndB-[Morganella-morganii]	582
HAE78646.1	DndB-ParB-nuclease	GCA_003446855.1	359	Bacteria>Proteobacteria>Gammaproteobacteria>Enterobacterales>Morganellaceae	Morganella_sp._(in:_Bacteria)	DNA-sulfur-modification-protein-DndB-[Morganella-sp.-(in:-Bacteria)]	1912315
HAE78643.1	DndB-ParB-nuclease	GCA_003446855.1	327	Bacteria>Proteobacteria>Gammaproteobacteria>Enterobacterales>Morganellaceae	Morganella_sp._(in:_Bacteria)	hypothetical-protein-[Morganella-sp.-(in:-Bacteria)]	1912315
HAE78651.1	DndB-ParB-nuclease	GCA_003446855.1	563	Bacteria>Proteobacteria>Gammaproteobacteria>Enterobacterales>Morganellaceae	Morganella_sp._(in:_Bacteria)	hypothetical-protein-[Morganella-sp.-(in:-Bacteria)]	1912315
NDK99878.1	DndB-ParB-nuclease	GCA_010119675.1	361	Bacteria>Proteobacteria>Gammaproteobacteria>Enterobacterales>Morganellaceae	Photorhabdus_bodei	DNA-sulfur-modification-protein-DndB-[Photorhabdus-bodei]	2029681
WP_162120125.1	DndB-ParB-nuclease	GCF_010119675.1	361	Bacteria>Proteobacteria>Gammaproteobacteria>Enterobacterales>Morganellaceae	Photorhabdus_bodei	DNA-sulfur-modification-protein-DndB-[Photorhabdus-bodei]	2029681
WP_036811616.1	DndB-ParB-nuclease	GCF_000767775.1	361	Bacteria>Proteobacteria>Gammaproteobacteria>Enterobacterales>Morganellaceae	Photorhabdus_luminescens	DNA-sulfur-modification-protein-DndB-[Photorhabdus-luminescens]	29488
WP_036843964.1	DndB-ParB-nuclease	GCF_000517265.1	361	Bacteria>Proteobacteria>Gammaproteobacteria>Enterobacterales>Morganellaceae	Photorhabdus	MULTISPECIES:-DNA-sulfur-modification-protein-DndB-[Photorhabdus]	29487
WP_214089794.1	DndB-ParB-nuclease	GCF_023108895.1	361	Bacteria>Proteobacteria>Gammaproteobacteria>Enterobacterales>Morganellaceae	Photorhabdus_noenieputensis	DNA-sulfur-modification-protein-DndB-[Photorhabdus-noenieputensis]	1208607
WP_133813768.1	DndB-ParB-nuclease	GCF_011189505.1	397	Bacteria>Proteobacteria>Gammaproteobacteria>Enterobacterales>Morganellaceae	Photorhabdus_tasmaniensis	DGQHR-domain-containing-protein-[Photorhabdus-tasmaniensis]	1004159
WP_133813764.1	DndB-ParB-nuclease	GCF_011189505.1	361	Bacteria>Proteobacteria>Gammaproteobacteria>Enterobacterales>Morganellaceae	Photorhabdus_tasmaniensis	DNA-sulfur-modification-protein-DndB-[Photorhabdus-tasmaniensis]	1004159
WP_161710620.1	DndB-ParB-nuclease	GCF_009915995.1	361	Bacteria>Proteobacteria>Gammaproteobacteria>Enterobacterales>Morganellaceae	Proteus	DNA-sulfur-modification-protein-DndB-[Proteus-sp.-G2618]	583
WP_049196863.1	DndB-ParB-nuclease	GCF_021373395.1	388	Bacteria>Proteobacteria>Gammaproteobacteria>Enterobacterales>Morganellaceae	Proteus_mirabilis	DGQHR-domain-containing-protein-[Proteus-mirabilis]	584
MBG6027592.1	DndB-ParB-nuclease	GCA_015740195.1	361	Bacteria>Proteobacteria>Gammaproteobacteria>Enterobacterales>Morganellaceae	Proteus_mirabilis	DNA-sulfur-modification-protein-DndB-[Proteus-mirabilis]	584
WP_017827637.1	DndB-ParB-nuclease	GCF_000372565.1	361	Bacteria>Proteobacteria>Gammaproteobacteria>Enterobacterales>Morganellaceae	Proteus_mirabilis	DNA-sulfur-modification-protein-DndB-[Proteus-mirabilis]	584
WP_049196871.1	DndB-ParB-nuclease	GCF_021373395.1	361	Bacteria>Proteobacteria>Gammaproteobacteria>Enterobacterales>Morganellaceae	Proteus_mirabilis	DNA-sulfur-modification-protein-DndB-[Proteus-mirabilis]	584
NBN69808.1	DndB-ParB-nuclease	GCA_009915995.1	361	Bacteria>Proteobacteria>Gammaproteobacteria>Enterobacterales>Morganellaceae	Proteus_sp._G2618	DNA-sulfur-modification-protein-DndB-[Proteus-sp.-G2618]	2698841
MTC36441.1	DndB-ParB-nuclease	GCA_009706385.1	388	Bacteria>Proteobacteria>Gammaproteobacteria>Enterobacterales>Morganellaceae	Providencia_alcalifaciens	DGQHR-domain-containing-protein-[Providencia-alcalifaciens]	126385
MTC36437.1	DndB-ParB-nuclease	GCA_009706385.1	361	Bacteria>Proteobacteria>Gammaproteobacteria>Enterobacterales>Morganellaceae	Providencia_alcalifaciens	DNA-sulfur-modification-protein-DndB-[Providencia-alcalifaciens]	126385
WP_154633515.1	DndB-ParB-nuclease	GCF_009706385.1	361	Bacteria>Proteobacteria>Gammaproteobacteria>Enterobacterales>Morganellaceae	Providencia_alcalifaciens	DNA-sulfur-modification-protein-DndB-[Providencia-alcalifaciens]	126385
WP_163767677.1	DndB-ParB-nuclease	GCF_014652175.1	567	Bacteria>Proteobacteria>Gammaproteobacteria>Enterobacterales>Morganellaceae	Providencia	MULTISPECIES:-AIPR-family-protein-[Providencia]	586
WP_154633519.1	DndB-ParB-nuclease	GCF_015571575.1	388	Bacteria>Proteobacteria>Gammaproteobacteria>Enterobacterales>Morganellaceae	Providencia	MULTISPECIES:-DGQHR-domain-containing-protein-[Providencia]	586
WP_154625381.1	DndB-ParB-nuclease	GCF_014652175.1	360	Bacteria>Proteobacteria>Gammaproteobacteria>Enterobacterales>Morganellaceae	Providencia	MULTISPECIES:-DNA-sulfur-modification-protein-DndB-[Providencia]	586
MBG5899959.1	DndB-ParB-nuclease	GCA_015739335.1	361	Bacteria>Proteobacteria>Gammaproteobacteria>Enterobacterales>Morganellaceae	Providencia_rettgeri	DNA-sulfur-modification-protein-DndB-[Providencia-rettgeri]	587
WP_166687415.1	DndB-ParB-nuclease	GCF_015571575.1	361	Bacteria>Proteobacteria>Gammaproteobacteria>Enterobacterales>Morganellaceae	Providencia_rettgeri	DNA-sulfur-modification-protein-DndB-[Providencia-rettgeri]	587
WP_196723543.1	DndB-ParB-nuclease	GCF_015739335.1	361	Bacteria>Proteobacteria>Gammaproteobacteria>Enterobacterales>Morganellaceae	Providencia_rettgeri	DNA-sulfur-modification-protein-DndB-[Providencia-rettgeri]	587
WP_112877462.1	DndB-ParB-nuclease	GCF_003287585.1	361	Bacteria>Proteobacteria>Gammaproteobacteria>Enterobacterales>Morganellaceae	unclassified_Photorhabdus	MULTISPECIES:-DNA-sulfur-modification-protein-DndB-[unclassified-Photorhabdus]	2620880
WP_115826129.1	DndB-ParB-nuclease	GCF_003386665.1	362	Bacteria>Proteobacteria>Gammaproteobacteria>Enterobacterales>Morganellaceae	Xenorhabdus_cabanillasii	DNA-sulfur-modification-protein-DndB-[Xenorhabdus-cabanillasii]	351673
WP_047961984.1	DndB-ParB-nuclease	GCF_001037465.1	567	Bacteria>Proteobacteria>Gammaproteobacteria>Enterobacterales>Morganellaceae	Xenorhabdus_khoisanae	AIPR-family-protein-[Xenorhabdus-khoisanae]	880157
WP_047961980.1	DndB-ParB-nuclease	GCF_001037465.1	362	Bacteria>Proteobacteria>Gammaproteobacteria>Enterobacterales>Morganellaceae	Xenorhabdus_khoisanae	DNA-sulfur-modification-protein-DndB-[Xenorhabdus-khoisanae]	880157
WP_237388241.1	DndB-ParB-nuclease	GCF_021730485.1	484	Bacteria>Proteobacteria>Gammaproteobacteria>Enterobacterales>Morganellaceae	Xenorhabdus_sp._Sc-CR9	AIPR-family-protein-[Xenorhabdus-sp.-Sc-CR9]	2584468
WP_237388245.1	DndB-ParB-nuclease	GCF_021730485.1	362	Bacteria>Proteobacteria>Gammaproteobacteria>Enterobacterales>Morganellaceae	Xenorhabdus_sp._Sc-CR9	DNA-sulfur-modification-protein-DndB-[Xenorhabdus-sp.-Sc-CR9]	2584468
WP_187652431.1	DndB-ParB-nuclease	GCF_014467265.1	567	Bacteria>Proteobacteria>Gammaproteobacteria>Enterobacterales>Morganellaceae	Xenorhabdus_sp._TS4	AIPR-family-protein-[Xenorhabdus-sp.-TS4]	1873483

Accession	Annotation	GCA	Length	Phyletic.spread	Strain.name	Defline	Species.Taxid
WP_187652435.1	DndB-ParB-nuclease	GCF_014467265.1	362	Bacteria>Proteobacteria>Gammaproteobacteria>Enterobacterales>Morganellaceae	Xenorhabdus_sp._TS4	DNA-sulfur-modification-protein-DndB-[Xenorhabdus-sp.-TS4]	1873483
WP_094107501.1	DndB-ParB-nuclease	GCF_014712575.1	388	Bacteria>Proteobacteria>Gammaproteobacteria	Enterobacterales	MULTISPECIES:-DGQHR-domain-containing-protein-[Enterobacterales]	91347
WP_021552210.1	DndB-ParB-nuclease	GCF_005384545.1	361	Bacteria>Proteobacteria>Gammaproteobacteria	Enterobacterales	MULTISPECIES:-DNA-sulfur-modification-protein-DndB-[Enterobacterales]	91347
WP_079973167.1	DndB-ParB-nuclease	GCF_013348865.1	361	Bacteria>Proteobacteria>Gammaproteobacteria	Enterobacterales	MULTISPECIES:-DNA-sulfur-modification-protein-DndB-[Enterobacterales]	91347
WP_114683290.1	DndB-ParB-nuclease	GCF_946226485.1	361	Bacteria>Proteobacteria>Gammaproteobacteria	Enterobacterales	MULTISPECIES:-DNA-sulfur-modification-protein-DndB-[Enterobacterales]	91347
WP_109053332.1	DndB-ParB-nuclease	GCF_003115815.1	361	Bacteria>Proteobacteria>Gammaproteobacteria>Enterobacterales>Pectobacteriaceae	Brenneria_roseae	DNA-sulfur-modification-protein-DndB-[Brenneria-roseae]	1509241
WP_169050486.1	DndB-ParB-nuclease	GCF_012932875.1	407	Bacteria>Proteobacteria>Gammaproteobacteria>Enterobacterales>Pectobacteriaceae	Brenneria_salicis	DGQHR-domain-containing-protein-[Brenneria-salicis]	55214
WP_113868843.1	DndB-ParB-nuclease	GCF_012932875.1	361	Bacteria>Proteobacteria>Gammaproteobacteria>Enterobacterales>Pectobacteriaceae	Brenneria_salicis	DNA-sulfur-modification-protein-DndB-[Brenneria-salicis]	55214
NPD01331.1	DndB-ParB-nuclease	GCA_013155275.1	403	Bacteria>Proteobacteria>Gammaproteobacteria>Enterobacterales>Pectobacteriaceae	Brenneria_sp._hezel4-2-4	DGQHR-domain-containing-protein-[Brenneria-sp.-hezel4-2-4]	2737661
WP_172290308.1	DndB-ParB-nuclease	GCF_013155275.1	403	Bacteria>Proteobacteria>Gammaproteobacteria>Enterobacterales>Pectobacteriaceae	Brenneria_sp._hezel4-2-4	DNA-phosphorothioation-associated-DGQHR-protein-1-[Brenneria-sp.-hezel4-2-4]	2737661
NPD01335.1	DndB-ParB-nuclease	GCA_013155275.1	361	Bacteria>Proteobacteria>Gammaproteobacteria>Enterobacterales>Pectobacteriaceae	Brenneria_sp._hezel4-2-4	DNA-sulfur-modification-protein-DndB-[Brenneria-sp.-hezel4-2-4]	2737661
ADM97032.1	DndB-ParB-nuclease	GCA_000147055.1	361	Bacteria>Proteobacteria>Gammaproteobacteria>Enterobacterales>Pectobacteriaceae	Dickeya_dadantii_3937	hypothetical-protein-Dda3937_01728-[Dickeya-dadantii-3937]	198628
WP_013316508.1	DndB-ParB-nuclease	GCF_000147055.1	361	Bacteria>Proteobacteria>Gammaproteobacteria>Enterobacterales>Pectobacteriaceae	Dickeya_dadantii	DNA-sulfur-modification-protein-DndB-[Dickeya-dadantii]	204038
WP_216282513.1	DndB-ParB-nuclease	GCF_018904205.1	361	Bacteria>Proteobacteria>Gammaproteobacteria>Enterobacterales>Pectobacteriaceae	Dickeya_dadantii	DNA-sulfur-modification-protein-DndB-[Dickeya-dadantii]	204038
WP_072035288.1	DndB-ParB-nuclease	GCF_000803195.1	421	Bacteria>Proteobacteria>Gammaproteobacteria>Enterobacterales>Pectobacteriaceae	Dickeya_fangzhongdai	DGQHR-domain-containing-protein-[Dickeya-fangzhongdai]	1778540
WP_039693439.1	DndB-ParB-nuclease	GCF_014893095.1	361	Bacteria>Proteobacteria>Gammaproteobacteria>Enterobacterales>Pectobacteriaceae	Dickeya	MULTISPECIES:-DNA-sulfur-modification-protein-DndB-[Dickeya]	204037
WP_210182298.1	DndB-ParB-nuclease	GCF_017897605.1	384	Bacteria>Proteobacteria>Gammaproteobacteria>Enterobacterales>Pectobacteriaceae	Dickeya_parazeae	DGQHR-domain-containing-protein-[Dickeya-zeae]	2893572
WP_210182293.1	DndB-ParB-nuclease	GCF_017897605.1	361	Bacteria>Proteobacteria>Gammaproteobacteria>Enterobacterales>Pectobacteriaceae	Dickeya_parazeae	DNA-sulfur-modification-protein-DndB-[Dickeya-zeae]	2893572
MBP2836922.1	DndB-ParB-nuclease	GCA_017897605.1	384	Bacteria>Proteobacteria>Gammaproteobacteria>Enterobacterales>Pectobacteriaceae	Dickeya_zeae	DGQHR-domain-containing-protein-[Dickeya-zeae]	204042
MBP2836917.1	DndB-ParB-nuclease	GCA_017897605.1	361	Bacteria>Proteobacteria>Gammaproteobacteria>Enterobacterales>Pectobacteriaceae	Dickeya_zeae	DNA-sulfur-modification-protein-DndB-[Dickeya-zeae]	204042
WP_094107504.1	DndB-ParB-nuclease	GCF_002111555.1	361	Bacteria>Proteobacteria>Gammaproteobacteria>Enterobacterales>Pectobacteriaceae	Lonsdalea_populi	DNA-sulfur-modification-protein-DndB-[Lonsdalea-populi]	1172565
WP_112094749.1	DndB-ParB-nuclease	GCF_003269515.1	361	Bacteria>Proteobacteria>Gammaproteobacteria>Enterobacterales>Pectobacteriaceae	Lonsdalea_populi	DNA-sulfur-modification-protein-DndB-[Lonsdalea-populi]	1172565
WP_167469132.1	DndB-ParB-nuclease	GCF_003382565.3	330	Bacteria>Proteobacteria>Gammaproteobacteria>Enterobacterales>Pectobacteriaceae	Pectobacterium_aquaticum	DGQHR-domain-containing-protein-[Pectobacterium-aquaticum]	2204145
WP_116162585.1	DndB-ParB-nuclease	GCF_003382565.3	359	Bacteria>Proteobacteria>Gammaproteobacteria>Enterobacterales>Pectobacteriaceae	Pectobacterium_aquaticum	DNA-sulfur-modification-protein-DndB-[Pectobacterium-aquaticum]	2204145
WP_116162595.1	DndB-ParB-nuclease	GCF_003382565.3	563	Bacteria>Proteobacteria>Gammaproteobacteria>Enterobacterales>Pectobacteriaceae	Pectobacterium_aquaticum	hypothetical-protein-[Pectobacterium-aquaticum]	2204145
MBA5226616.1	DndB-ParB-nuclease	GCA_013913585.1	403	Bacteria>Proteobacteria>Gammaproteobacteria>Enterobacterales>Pectobacteriaceae	Pectobacterium_aroidearum	DGQHR-domain-containing-protein-[Pectobacterium-aroidearum]	1201031
WP_181838194.1	DndB-ParB-nuclease	GCF_024343035.1	403	Bacteria>Proteobacteria>Gammaproteobacteria>Enterobacterales>Pectobacteriaceae	Pectobacterium_aroidearum	DNA-phosphorothioation-associated-DGQHR-protein-1-[Pectobacterium-aroidearum]	1201031
MBA5226621.1	DndB-ParB-nuclease	GCA_013913585.1	361	Bacteria>Proteobacteria>Gammaproteobacteria>Enterobacterales>Pectobacteriaceae	Pectobacterium_aroidearum	DNA-sulfur-modification-protein-DndB-[Pectobacterium-aroidearum]	1201031
WP_052238903.1	DndB-ParB-nuclease	-	149	Bacteria>Proteobacteria>Gammaproteobacteria>Enterobacterales>Pectobacteriaceae	Pectobacterium_brasiliense	DGQHR-domain-containing-protein-[Pectobacterium-brasiliense]	180957
WP_039524347.1	DndB-ParB-nuclease	GCF_000808385.1	361	Bacteria>Proteobacteria>Gammaproteobacteria>Enterobacterales>Pectobacteriaceae	Pectobacterium_brasiliense	DNA-sulfur-modification-protein-DndB-[Pectobacterium-brasiliense]	180957
WP_039524348.1	DndB-ParB-nuclease	GCF_000808385.1	705	Bacteria>Proteobacteria>Gammaproteobacteria>Enterobacterales>Pectobacteriaceae	Pectobacterium_brasiliense	hypothetical-protein-[Pectobacterium-brasiliense]	180957
WP_080764411.1	DndB-ParB-nuclease	-	234	Bacteria>Proteobacteria>Gammaproteobacteria>Enterobacterales>Pectobacteriaceae	Pectobacterium_brasiliense	hypothetical-protein-[Pectobacterium-brasiliense]	180957
WP_161502357.1	DndB-ParB-nuclease	GCF_009931195.1	359	Bacteria>Proteobacteria>Gammaproteobacteria>Enterobacterales>Pectobacteriaceae	Pectobacterium_carotovorum	DNA-sulfur-modification-protein-DndB-[Pectobacterium-carotovorum]	554
WP_161502353.1	DndB-ParB-nuclease	GCF_009931195.1	179	Bacteria>Proteobacteria>Gammaproteobacteria>Enterobacterales>Pectobacteriaceae	Pectobacterium_carotovorum	hypothetical-protein-[Pectobacterium-carotovorum]	554
WP_161502354.1	DndB-ParB-nuclease	GCF_009931195.1	305	Bacteria>Proteobacteria>Gammaproteobacteria>Enterobacterales>Pectobacteriaceae	Pectobacterium_carotovorum	hypothetical-protein-[Pectobacterium-carotovorum]	554
QHP53815.1	DndB-ParB-nuclease	GCA_009931195.1	359	Bacteria>Proteobacteria>Gammaproteobacteria>Enterobacterales>Pectobacteriaceae	Pectobacterium_carotovorum_subsp._carotovorum	DNA-sulfur-modification-protein-DndB-[Pectobacterium-carotovorum-subsp.-carotovorum]	555
QHP53810.1	DndB-ParB-nuclease	GCA_009931195.1	179	Bacteria>Proteobacteria>Gammaproteobacteria>Enterobacterales>Pectobacteriaceae	Pectobacterium_carotovorum_subsp._carotovorum	hypothetical-protein-EH203_08215-[Pectobacterium-carotovorum-subsp.-carotovorum]	555
QHP53811.1	DndB-ParB-nuclease	GCA_009931195.1	305	Bacteria>Proteobacteria>Gammaproteobacteria>Enterobacterales>Pectobacteriaceae	Pectobacterium_carotovorum_subsp._carotovorum	hypothetical-protein-EH203_08220-[Pectobacterium-carotovorum-subsp.-carotovorum]	555
WP_130587489.1	DndB-ParB-nuclease	GCF_922022005.1	361	Bacteria>Proteobacteria>Gammaproteobacteria>Enterobacterales>Pectobacteriaceae	Pectobacterium	MULTISPECIES:-DNA-sulfur-modification-protein-DndB-[Pectobacterium]	122277
WP_161528659.1	DndB-ParB-nuclease	GCF_009931295.1	361	Bacteria>Proteobacteria>Gammaproteobacteria>Enterobacterales>Pectobacteriaceae	Pectobacterium	MULTISPECIES:-DNA-sulfur-modification-protein-DndB-[Pectobacterium]	122277
WP_180742539.1	DndB-ParB-nuclease	GCF_024343035.1	361	Bacteria>Proteobacteria>Gammaproteobacteria>Enterobacterales>Pectobacteriaceae	Pectobacterium	MULTISPECIES:-DNA-sulfur-modification-protein-DndB-[Pectobacterium]	122277
WP_103161855.1	DndB-ParB-nuclease	GCF_002904105.1	390	Bacteria>Proteobacteria>Gammaproteobacteria>Enterobacterales>Pectobacteriaceae	Pectobacterium_odoriferum	DGQHR-domain-containing-protein-[Pectobacterium-odoriferum]	78398
WP_103161859.1	DndB-ParB-nuclease	GCF_002904105.1	361	Bacteria>Proteobacteria>Gammaproteobacteria>Enterobacterales>Pectobacteriaceae	Pectobacterium_odoriferum	DNA-sulfur-modification-protein-DndB-[Pectobacterium-odoriferum]	78398
WP_103808655.1	DndB-ParB-nuclease	GCF_003628595.1	388	Bacteria>Proteobacteria>Gammaproteobacteria>Enterobacterales>Pectobacteriaceae	Pectobacterium_parmentieri	DGQHR-domain-containing-protein-[Pectobacterium-parmentieri]	1905730
WP_103808659.1	DndB-ParB-nuclease	GCF_003628595.1	361	Bacteria>Proteobacteria>Gammaproteobacteria>Enterobacterales>Pectobacteriaceae	Pectobacterium_parmentieri	DNA-sulfur-modification-protein-DndB-[Pectobacterium-parmentieri]	1905730
WP_226308762.1	DndB-ParB-nuclease	GCF_020406755.1	406	Bacteria>Proteobacteria>Gammaproteobacteria>Enterobacterales>Pectobacteriaceae	Pectobacterium_polaris	unnamed-protein-product]	2042057
WP_226373467.1	DndB-ParB-nuclease	GCF_014946805.1	352	Bacteria>Proteobacteria>Gammaproteobacteria>Enterobacterales>Pectobacteriaceae	Pectobacterium_quasiaquaticum	DNA-sulfur-modification-protein-DndB-[Pectobacterium-quasiaquaticum]	2774015
MBA0167868.1	DndB-ParB-nuclease	GCA_013449375.1	361	Bacteria>Proteobacteria>Gammaproteobacteria>Enterobacterales>Pectobacteriaceae	Pectobacterium_sp._CFBP8739	DNA-sulfur-modification-protein-DndB-[Pectobacterium-sp.-CFBP8739]	2748908
MBA0167862.1	DndB-ParB-nuclease	GCA_013449375.1	688	Bacteria>Proteobacteria>Gammaproteobacteria>Enterobacterales>Pectobacteriaceae	Pectobacterium_sp._CFBP8739	hypothetical-protein-[Pectobacterium-sp.-CFBP8739]	2748908
WP_180742533.1	DndB-ParB-nuclease	-	688	Bacteria>Proteobacteria>Gammaproteobacteria>Enterobacterales>Pectobacteriaceae	Pectobacterium_sp._CFBP8739	hypothetical-protein-[Pectobacterium-sp.-CFBP8739]	2748908
QQK71687.1	DndB-ParB-nuclease	GCA_016495705.1	382	Bacteria>Proteobacteria>Gammaproteobacteria>Enterobacterales>Pectobacteriaceae	Pectobacterium_versatile	DGQHR-domain-containing-protein-[Pectobacterium-versatile]	2488639
WP_161569011.1	DndB-ParB-nuclease	GCF_004296725.1	386	Bacteria>Proteobacteria>Gammaproteobacteria>Enterobacterales>Pectobacteriaceae	Pectobacterium_versatile	DGQHR-domain-containing-protein-[Pectobacterium-versatile]	2488639
QQK71691.1	DndB-ParB-nuclease	GCA_016495705.1	362	Bacteria>Proteobacteria>Gammaproteobacteria>Enterobacterales>Pectobacteriaceae	Pectobacterium_versatile	DNA-sulfur-modification-protein-DndB-[Pectobacterium-versatile]	2488639
WP_116586721.1	DndB-ParB-nuclease	GCF_922021695.1	361	Bacteria>Proteobacteria>Gammaproteobacteria>Enterobacterales>Pectobacteriaceae	Pectobacterium_versatile	DNA-sulfur-modification-protein-DndB-[Pectobacterium-versatile]	2488639
WP_233977243.1	DndB-ParB-nuclease	GCF_922016765.1	361	Bacteria>Proteobacteria>Gammaproteobacteria>Enterobacterales>Pectobacteriaceae	Pectobacterium_versatile	DNA-sulfur-modification-protein-DndB-[Pectobacterium-versatile]	2488639
WP_233962266.1	DndB-ParB-nuclease	GCF_922022005.1	688	Bacteria>Proteobacteria>Gammaproteobacteria>Enterobacterales>Pectobacteriaceae	Pectobacterium_versatile	hypothetical-protein-[Pectobacterium-versatile]	2488639
VXD08499.1	DndB-ParB-nuclease	GCA_902506615.1	361	Bacteria>Proteobacteria>Gammaproteobacteria>Enterobacterales>unclassified_Enterobacterales	Enterobacterales_bacterium_8AC	DNA-sulfur-modification-protein-DndB-[Enterobacterales-bacterium-8AC]	2653184
WP_101814947.1	DndB-ParB-nuclease	GCF_002858675.1	402	Bacteria>Proteobacteria>Gammaproteobacteria>Enterobacterales>Yersiniaceae	Chimaeribacter_californicus	DGQHR-domain-containing-protein-[Chimaeribacter-californicus]	2060067
WP_101815029.1	DndB-ParB-nuclease	GCF_002858675.1	361	Bacteria>Proteobacteria>Gammaproteobacteria>Enterobacterales>Yersiniaceae	Chimaeribacter_californicus	DNA-sulfur-modification-protein-DndB-[Chimaeribacter-californicus]	2060067
WP_095846987.1	DndB-ParB-nuclease	GCF_002291425.1	361	Bacteria>Proteobacteria>Gammaproteobacteria>Enterobacterales>Yersiniaceae	Gibbsiella_quercinecans	DNA-sulfur-modification-protein-DndB-[Gibbsiella-quercinecans]	929813
WP_131928980.1	DndB-ParB-nuclease	GCF_002291425.1	688	Bacteria>Proteobacteria>Gammaproteobacteria>Enterobacterales>Yersiniaceae	Gibbsiella_quercinecans	hypothetical-protein-[Gibbsiella-quercinecans]	929813
MBU9857675.1	DndB-ParB-nuclease	GCA_019049675.1	361	Bacteria>Proteobacteria>Gammaproteobacteria>Enterobacterales>Yersiniaceae	Rahnella_bonaserana	DNA-sulfur-modification-protein-DndB-[Rahnella-sp.-H11b]	2816248
WP_217174744.1	DndB-ParB-nuclease	GCF_019049675.1	361	Bacteria>Proteobacteria>Gammaproteobacteria>Enterobacterales>Yersiniaceae	Rahnella_bonaserana	DNA-sulfur-modification-protein-DndB-[Rahnella-sp.-H11b]	2816248
WP_134706883.1	DndB-ParB-nuclease	GCF_004522185.1	359	Bacteria>Proteobacteria>Gammaproteobacteria>Enterobacterales>Yersiniaceae	Rahnella_sp._CJA17(1/100)	DNA-sulfur-modification-protein-DndB-[Rahnella-sp.-CJA17(1/100)]	2508951
WP_045047178.1	DndB-ParB-nuclease	GCF_000951135.1	361	Bacteria>Proteobacteria>Gammaproteobacteria>Enterobacterales>Yersiniaceae	Rouxiella_chamberiensis	DNA-sulfur-modification-protein-DndB-[Rouxiella-chamberiensis]	1513468
MBC3228936.1	DndB-ParB-nuclease	GCA_014268125.1	361	Bacteria>Proteobacteria>Gammaproteobacteria>Enterobacterales>Yersiniaceae	Serratia_fonticola	DNA-sulfur-modification-protein-DndB-[Serratia-fonticola]	47917
WP_186529595.1	DndB-ParB-nuclease	GCF_014268125.1	361	Bacteria>Proteobacteria>Gammaproteobacteria>Enterobacterales>Yersiniaceae	Serratia_fonticola	DNA-sulfur-modification-protein-DndB-[Serratia-fonticola]	47917
WP_142816037.1	DndB-ParB-nuclease	GCF_006970665.1	361	Bacteria>Proteobacteria>Gammaproteobacteria>Enterobacterales>Yersiniaceae	Serratia_liquefaciens	DNA-sulfur-modification-protein-DndB-[Serratia-liquefaciens]	614
MBH2916109.1	DndB-ParB-nuclease	GCA_016006595.1	415	Bacteria>Proteobacteria>Gammaproteobacteria>Enterobacterales>Yersiniaceae	Serratia_marcescens	DGQHR-domain-containing-protein-[Serratia-marcescens]	615
MBH3051754.1	DndB-ParB-nuclease	GCA_016007085.1	404	Bacteria>Proteobacteria>Gammaproteobacteria>Enterobacterales>Yersiniaceae	Serratia_marcescens	DGQHR-domain-containing-protein-[Serratia-marcescens]	615
MBH3276228.1	DndB-ParB-nuclease	GCA_016008575.1	392	Bacteria>Proteobacteria>Gammaproteobacteria>Enterobacterales>Yersiniaceae	Serratia_marcescens	DGQHR-domain-containing-protein-[Serratia-marcescens]	615
MBH2916104.1	DndB-ParB-nuclease	GCA_016006595.1	361	Bacteria>Proteobacteria>Gammaproteobacteria>Enterobacterales>Yersiniaceae	Serratia_marcescens	DNA-sulfur-modification-protein-DndB-[Serratia-marcescens]	615
MBH3015159.1	DndB-ParB-nuclease	GCA_016006945.1	361	Bacteria>Proteobacteria>Gammaproteobacteria>Enterobacterales>Yersiniaceae	Serratia_marcescens	DNA-sulfur-modification-protein-DndB-[Serratia-marcescens]	615
MBH3051750.1	DndB-ParB-nuclease	GCA_016007085.1	361	Bacteria>Proteobacteria>Gammaproteobacteria>Enterobacterales>Yersiniaceae	Serratia_marcescens	DNA-sulfur-modification-protein-DndB-[Serratia-marcescens]	615
MBH3276223.1	DndB-ParB-nuclease	GCA_016008575.1	361	Bacteria>Proteobacteria>Gammaproteobacteria>Enterobacterales>Yersiniaceae	Serratia_marcescens	DNA-sulfur-modification-protein-DndB-[Serratia-marcescens]	615
WP_060443717.1	DndB-ParB-nuclease	GCF_001537405.1	361	Bacteria>Proteobacteria>Gammaproteobacteria>Enterobacterales>Yersiniaceae	Serratia_marcescens	DNA-sulfur-modification-protein-DndB-[Serratia-marcescens]	615
WP_235363439.1	DndB-ParB-nuclease	GCF_021533385.1	361	Bacteria>Proteobacteria>Gammaproteobacteria>Enterobacterales>Yersiniaceae	Serratia_marcescens	DNA-sulfur-modification-protein-DndB-[Serratia-marcescens]	615
WP_080435347.1	DndB-ParB-nuclease	GCF_001537405.1	705	Bacteria>Proteobacteria>Gammaproteobacteria>Enterobacterales>Yersiniaceae	Serratia_marcescens	hypothetical-protein-[Serratia-marcescens]	615
WP_127960402.1	DndB-ParB-nuclease	GCF_004011885.1	363	Bacteria>Proteobacteria>Gammaproteobacteria>Enterobacterales>Yersiniaceae	Serratia_microhaemolytica	DNA-sulfur-modification-protein-DndB-[Serratia-microhaemolytica]	2675110
OQV34756.1	DndB-ParB-nuclease	GCA_002082115.1	361	Bacteria>Proteobacteria>Gammaproteobacteria>Enterobacterales>Yersiniaceae	Serratia_nematodiphila	DNA-sulfur-modification-protein-DndB-[Serratia-nematodiphila]	458197
OQV34760.1	DndB-ParB-nuclease	GCA_002082115.1	696	Bacteria>Proteobacteria>Gammaproteobacteria>Enterobacterales>Yersiniaceae	Serratia_nematodiphila	hypothetical-protein-BV901_12540-[Serratia-nematodiphila]	458197
OQV34766.1	DndB-ParB-nuclease	GCA_002082115.1	379	Bacteria>Proteobacteria>Gammaproteobacteria>Enterobacterales>Yersiniaceae	Serratia_nematodiphila	hypothetical-protein-BV901_12575-[Serratia-nematodiphila]	458197
MBL0872187.1	DndB-ParB-nuclease	GCA_016742975.1	361	Bacteria>Proteobacteria>Gammaproteobacteria>Enterobacterales>Yersiniaceae	Serratia_nevei	DNA-sulfur-modification-protein-DndB-[Serratia-nevei]	2703794
WP_201965229.1	DndB-ParB-nuclease	GCF_016742975.1	361	Bacteria>Proteobacteria>Gammaproteobacteria>Enterobacterales>Yersiniaceae	Serratia_nevei	DNA-sulfur-modification-protein-DndB-[Serratia-nevei]	2703794
WP_004949445.1	DndB-ParB-nuclease	GCF_000176835.2	399	Bacteria>Proteobacteria>Gammaproteobacteria>Enterobacterales>Yersiniaceae	Serratia_plymuthica	DNA-phosphorothioation-associated-DGQHR-protein-1-[Serratia-plymuthica]	82996
WP_004949453.1	DndB-ParB-nuclease	GCF_000176835.2	361	Bacteria>Proteobacteria>Gammaproteobacteria>Enterobacterales>Yersiniaceae	Serratia_plymuthica	DNA-sulfur-modification-protein-DndB-[Serratia-plymuthica]	82996
WP_015670714.1	DndB-ParB-nuclease	GCF_000330865.1	361	Bacteria>Proteobacteria>Gammaproteobacteria>Enterobacterales>Yersiniaceae	Serratia_sp._FGI94	DNA-sulfur-modification-protein-DndB-[Serratia-sp.-FGI94]	671990
WP_223498814.1	DndB-ParB-nuclease	GCF_019968745.1	388	Bacteria>Proteobacteria>Gammaproteobacteria>Enterobacterales>Yersiniaceae	Serratia_sp._JSRIV001	DGQHR-domain-containing-protein-[Serratia-sp.-JSRIV001]	2831893
WP_223498818.1	DndB-ParB-nuclease	GCF_019968745.1	361	Bacteria>Proteobacteria>Gammaproteobacteria>Enterobacterales>Yersiniaceae	Serratia_sp._JSRIV001	DNA-sulfur-modification-protein-DndB-[Serratia-sp.-JSRIV001]	2831893
WP_187043724.1	DndB-ParB-nuclease	GCF_014298095.1	407	Bacteria>Proteobacteria>Gammaproteobacteria>Enterobacterales>Yersiniaceae	Serratia_sp._JUb9	DGQHR-domain-containing-protein-[Serratia-sp.-JUb9]	2724469
WP_187043720.1	DndB-ParB-nuclease	GCF_014298095.1	361	Bacteria>Proteobacteria>Gammaproteobacteria>Enterobacterales>Yersiniaceae	Serratia_sp._JUb9	DNA-sulfur-modification-protein-DndB-[Serratia-sp.-JUb9]	2724469
WP_105229600.1	DndB-ParB-nuclease	GCF_002966855.1	387	Bacteria>Proteobacteria>Gammaproteobacteria>Enterobacterales>Yersiniaceae	Serratia_sp._MYb239	DGQHR-domain-containing-protein-[Serratia-sp.-MYb239]	2033438
WP_105229603.1	DndB-ParB-nuclease	GCF_002966855.1	361	Bacteria>Proteobacteria>Gammaproteobacteria>Enterobacterales>Yersiniaceae	Serratia_sp._MYb239	DNA-sulfur-modification-protein-DndB-[Serratia-sp.-MYb239]	2033438
MBL0880122.1	DndB-ParB-nuclease	GCA_016743015.1	415	Bacteria>Proteobacteria>Gammaproteobacteria>Enterobacterales>Yersiniaceae	Serratia_ureilytica	DGQHR-domain-containing-protein-[Serratia-ureilytica]	300181
WP_201884556.1	DndB-ParB-nuclease	GCF_016743015.1	415	Bacteria>Proteobacteria>Gammaproteobacteria>Enterobacterales>Yersiniaceae	Serratia_ureilytica	DGQHR-domain-containing-protein-[Serratia-ureilytica]	300181
MBH2895917.1	DndB-ParB-nuclease	GCA_016006465.1	361	Bacteria>Proteobacteria>Gammaproteobacteria>Enterobacterales>Yersiniaceae	Serratia_ureilytica	DNA-sulfur-modification-protein-DndB-[Serratia-ureilytica]	300181
MBL0880127.1	DndB-ParB-nuclease	GCA_016743015.1	361	Bacteria>Proteobacteria>Gammaproteobacteria>Enterobacterales>Yersiniaceae	Serratia_ureilytica	DNA-sulfur-modification-protein-DndB-[Serratia-ureilytica]	300181
WP_176589341.1	DndB-ParB-nuclease	GCF_013375155.1	361	Bacteria>Proteobacteria>Gammaproteobacteria>Enterobacterales>Yersiniaceae	Serratia_ureilytica	DNA-sulfur-modification-protein-DndB-[Serratia-ureilytica]	300181
WP_197761225.1	DndB-ParB-nuclease	GCF_016743015.1	361	Bacteria>Proteobacteria>Gammaproteobacteria>Enterobacterales>Yersiniaceae	Serratia_ureilytica	DNA-sulfur-modification-protein-DndB-[Serratia-ureilytica]	300181
WP_074006606.1	DndB-ParB-nuclease	GCF_001140765.1	384	Bacteria>Proteobacteria>Gammaproteobacteria>Enterobacterales>Yersiniaceae	Yersinia_intermedia	DGQHR-domain-containing-protein-[Yersinia-intermedia]	631
WP_050085539.1	DndB-ParB-nuclease	GCF_001140765.1	361	Bacteria>Proteobacteria>Gammaproteobacteria>Enterobacterales>Yersiniaceae	Yersinia_intermedia	DNA-sulfur-modification-protein-DndB-[Yersinia-intermedia]	631
WP_050536366.1	DndB-ParB-nuclease	GCF_001220845.1	690	Bacteria>Proteobacteria>Gammaproteobacteria>Enterobacterales>Yersiniaceae	Yersinia_mollaretii	hypothetical-protein-[Yersinia-mollaretii]	33060
WP_192945361.1	DndB-ParB-nuclease	GCF_002073315.2	384	Bacteria>Proteobacteria>Gammaproteobacteria>Enterobacterales>Yersiniaceae	Yersinia	MULTISPECIES:-DGQHR-domain-containing-protein-[Yersinia]	629
WP_050103238.1	DndB-ParB-nuclease	GCF_902168505.1	361	Bacteria>Proteobacteria>Gammaproteobacteria>Enterobacterales>Yersiniaceae	Yersinia	MULTISPECIES:-DNA-sulfur-modification-protein-DndB-[Yersinia]	629
WP_050536361.1	DndB-ParB-nuclease	GCF_902170545.1	361	Bacteria>Proteobacteria>Gammaproteobacteria>Enterobacterales>Yersiniaceae	Yersinia	MULTISPECIES:-DNA-sulfur-modification-protein-DndB-[Yersinia]	629
WP_057648604.1	DndB-ParB-nuclease	GCF_902388035.1	361	Bacteria>Proteobacteria>Gammaproteobacteria>Enterobacterales>Yersiniaceae	Yersinia	MULTISPECIES:-DNA-sulfur-modification-protein-DndB-[Yersinia]	629
WP_145539364.1	DndB-ParB-nuclease	GCF_902170405.1	361	Bacteria>Proteobacteria>Gammaproteobacteria>Enterobacterales>Yersiniaceae	Yersinia	MULTISPECIES:-DNA-sulfur-modification-protein-DndB-[Yersinia]	629
WP_052470854.1	DndB-ParB-nuclease	GCF_000834255.1	398	Bacteria>Proteobacteria>Gammaproteobacteria>Enterobacterales>Yersiniaceae	Yersinia_ruckeri	DGQHR-domain-containing-protein-[Yersinia-ruckeri]	29486
WP_145497530.1	DndB-ParB-nuclease	GCF_902173145.1	388	Bacteria>Proteobacteria>Gammaproteobacteria>Enterobacterales>Yersiniaceae	Yersinia_ruckeri	DGQHR-domain-containing-protein-[Yersinia-ruckeri]	29486
WP_042527209.1	DndB-ParB-nuclease	GCF_902173145.1	361	Bacteria>Proteobacteria>Gammaproteobacteria>Enterobacterales>Yersiniaceae	Yersinia_ruckeri	DNA-sulfur-modification-protein-DndB-[Yersinia-ruckeri]	29486
WP_186372046.1	DndB-ParB-nuclease	GCF_902170405.1	384	Bacteria>Proteobacteria>Gammaproteobacteria>Enterobacterales>Yersiniaceae	Yersinia_vastinensis	DGQHR-domain-containing-protein-[Yersinia-frederiksenii]	2890318
TVR64213.1	DndB-ParB-nuclease	GCA_007695245.1	370	Bacteria>Proteobacteria>Gammaproteobacteria>Gammaproteobacteria_incertae_sedis>Candidatus_Competibacteraceae	Candidatus_Competibacteraceae_bacterium	DNA-sulfur-modification-protein-DndB-[Candidatus-Competibacteraceae-bacterium]	2053538
TVR64210.1	DndB-ParB-nuclease	GCA_007695245.1	767	Bacteria>Proteobacteria>Gammaproteobacteria>Gammaproteobacteria_incertae_sedis>Candidatus_Competibacteraceae	Candidatus_Competibacteraceae_bacterium	hypothetical-protein-EA420_05695-[Candidatus-Competibacteraceae-bacterium]	2053538
WP_183410046.1	DndB-ParB-nuclease	GCF_014191715.1	393	Bacteria>Proteobacteria>Gammaproteobacteria>Gammaproteobacteria_incertae_sedis>Litorivivens	Litorivivens_lipolytica	DGQHR-domain-containing-protein-[Litorivivens-lipolytica]	1524264
MBB3047424.1	DndB-ParB-nuclease	GCA_014191715.1	393	Bacteria>Proteobacteria>Gammaproteobacteria>Gammaproteobacteria_incertae_sedis>Litorivivens	Litorivivens_lipolytica	DNA-phosphorothioation-associated-DGQHR-protein-1-[Litorivivens-lipolytica]	1524264
MBB3047425.1	DndB-ParB-nuclease	GCA_014191715.1	360	Bacteria>Proteobacteria>Gammaproteobacteria>Gammaproteobacteria_incertae_sedis>Litorivivens	Litorivivens_lipolytica	DNA-sulfur-modification-protein-DndB-[Litorivivens-lipolytica]	1524264
WP_183410047.1	DndB-ParB-nuclease	GCF_014191715.1	360	Bacteria>Proteobacteria>Gammaproteobacteria>Gammaproteobacteria_incertae_sedis>Litorivivens	Litorivivens_lipolytica	DNA-sulfur-modification-protein-DndB-[Litorivivens-lipolytica]	1524264
WP_083218547.1	DndB-ParB-nuclease	GCF_001708965.1	361	Bacteria>Proteobacteria>Gammaproteobacteria>Gammaproteobacteria_incertae_sedis>sulfur-oxidizing_symbionts	Candidatus_Thiodiazotropha_endoloripes	DNA-sulfur-modification-protein-DndB-[Candidatus-Thiodiazotropha-endoloripes]	1818881
WP_127030696.1	DndB-ParB-nuclease	GCF_003994235.2	371	Bacteria>Proteobacteria>Gammaproteobacteria>Methylococcales>Methylococcaceae	Candidatus_Methylobacter_oryzae	DNA-sulfur-modification-protein-DndB-[Candidatus-Methylobacter-oryzae]	2497749
TAN71122.1	DndB-ParB-nuclease	GCA_004299305.1	367	Bacteria>Proteobacteria>Gammaproteobacteria>Methylococcales>Methylococcaceae	Methylobacter_sp.	DNA-sulfur-modification-protein-DndB-[Methylobacter-sp.]	2051955
MVF23447.1	DndB-ParB-nuclease	GCA_009749305.1	360	Bacteria>Proteobacteria>Gammaproteobacteria>Methylococcales>Methylococcaceae	Methylocaldum_sp._BRCS4	DNA-sulfur-modification-protein-DndB-[Methylocaldum-sp.-BRCS4]	2510798
TXL03710.1	DndB-ParB-nuclease	GCA_008030215.1	361	Bacteria>Proteobacteria>Gammaproteobacteria>Methylococcales>Methylococcaceae	Methylococcaceae_bacterium_CS1	DNA-sulfur-modification-protein-DndB-[Methylococcaceae-bacterium-CS1]	1916066
TXL07191.1	DndB-ParB-nuclease	GCA_008030155.1	361	Bacteria>Proteobacteria>Gammaproteobacteria>Methylococcales>Methylococcaceae	Methylococcaceae_bacterium_CS2	DNA-sulfur-modification-protein-DndB-[Methylococcaceae-bacterium-CS2]	1916067
TXK94276.1	DndB-ParB-nuclease	GCA_008030135.1	361	Bacteria>Proteobacteria>Gammaproteobacteria>Methylococcales>Methylococcaceae	Methylococcaceae_bacterium_CS4	DNA-sulfur-modification-protein-DndB-[Methylococcaceae-bacterium-CS4]	1916069
TXL13078.1	DndB-ParB-nuclease	GCA_008030225.1	361	Bacteria>Proteobacteria>Gammaproteobacteria>Methylococcales>Methylococcaceae	Methylococcaceae_bacterium_HT4	DNA-sulfur-modification-protein-DndB-[Methylococcaceae-bacterium-HT4]	1916064

Accession	Annotation	GCA	Length	Phyletic.spread	Strain.name	Defline	Species.Taxid
WP_192394977.1	DndB-ParB-nuclease	-	361	Bacteria>Proteobacteria>Gammaproteobacteria>Methylococcales>Methylococcaceae	Methylomonas_fluvii	DNA-sulfur-modification-protein-DndB-[Methylomonas-sp.-EbB]	1854564
WP_064008154.1	DndB-ParB-nuclease	GCF_001644035.1	368	Bacteria>Proteobacteria>Gammaproteobacteria>Methylococcales>Methylococcaceae	Methylomonas_methanica	DGQHR-domain-containing-protein-[Methylomonas-methanica]	421
OAI06192.1	DndB-ParB-nuclease	GCA_001644035.1	363	Bacteria>Proteobacteria>Gammaproteobacteria>Methylococcales>Methylococcaceae	Methylomonas_methanica	DNA-sulfur-modification-protein-DndB-[Methylomonas-methanica]	421
WP_064008151.1	DndB-ParB-nuclease	GCF_001644035.1	363	Bacteria>Proteobacteria>Gammaproteobacteria>Methylococcales>Methylococcaceae	Methylomonas_methanica	DNA-sulfur-modification-protein-DndB-[Methylomonas-methanica]	421
OAI06195.1	DndB-ParB-nuclease	GCA_001644035.1	368	Bacteria>Proteobacteria>Gammaproteobacteria>Methylococcales>Methylococcaceae	Methylomonas_methanica	hypothetical-protein-A1332_01430-[Methylomonas-methanica]	421
MBE0470999.1	DndB-ParB-nuclease	GCA_014859465.1	325	Bacteria>Proteobacteria>Gammaproteobacteria>Methylococcales>Methylococcaceae	Methyloprofundus_sp.	DGQHR-domain-containing-protein-[Methyloprofundus-sp.]	2020875
MBE0471003.1	DndB-ParB-nuclease	GCA_014859465.1	361	Bacteria>Proteobacteria>Gammaproteobacteria>Methylococcales>Methylococcaceae	Methyloprofundus_sp.	DNA-sulfur-modification-protein-DndB-[Methyloprofundus-sp.]	2020875
MBE0470996.1	DndB-ParB-nuclease	GCA_014859465.1	566	Bacteria>Proteobacteria>Gammaproteobacteria>Methylococcales>Methylococcaceae	Methyloprofundus_sp.	hypothetical-protein-[Methyloprofundus-sp.]	2020875
WP_139559695.1	DndB-ParB-nuclease	GCF_006175985.1	361	Bacteria>Proteobacteria>Gammaproteobacteria>Methylococcales>Methylococcaceae	Methylotetracoccus_oryzae	DNA-sulfur-modification-protein-DndB-[Methylotetracoccus-oryzae]	1919059
WP_004736023.1	DndB-ParB-nuclease	GCF_001007705.1	414	Bacteria>Proteobacteria>Gammaproteobacteria>Moraxellales>Moraxellaceae	Acinetobacter_baumannii	DGQHR-domain-containing-protein-[Acinetobacter-baumannii]	470
WP_237812695.1	DndB-ParB-nuclease	GCF_022014125.1	355	Bacteria>Proteobacteria>Gammaproteobacteria>Moraxellales>Moraxellaceae	Acinetobacter_baumannii	DGQHR-domain-containing-protein-[Acinetobacter-baumannii]	470
OIG01335.1	DndB-ParB-nuclease	GCA_001864735.1	371	Bacteria>Proteobacteria>Gammaproteobacteria>Moraxellales>Moraxellaceae	Acinetobacter_baumannii	DNA-sulfur-modification-protein-DndB-[Acinetobacter-baumannii]	470
WP_004736028.1	DndB-ParB-nuclease	GCF_001007705.1	369	Bacteria>Proteobacteria>Gammaproteobacteria>Moraxellales>Moraxellaceae	Acinetobacter_baumannii	DNA-sulfur-modification-protein-DndB-[Acinetobacter-baumannii]	470
WP_065725928.1	DndB-ParB-nuclease	GCF_001693205.1	369	Bacteria>Proteobacteria>Gammaproteobacteria>Moraxellales>Moraxellaceae	Acinetobacter_baumannii	DNA-sulfur-modification-protein-DndB-[Acinetobacter-baumannii]	470
WP_162278790.1	DndB-ParB-nuclease	GCF_902705405.1	369	Bacteria>Proteobacteria>Gammaproteobacteria>Moraxellales>Moraxellaceae	Acinetobacter_baumannii	DNA-sulfur-modification-protein-DndB-[Acinetobacter-baumannii]	470
WP_169538759.1	DndB-ParB-nuclease	GCF_012974585.1	372	Bacteria>Proteobacteria>Gammaproteobacteria>Moraxellales>Moraxellaceae	Acinetobacter_baumannii	DNA-sulfur-modification-protein-DndB-[Acinetobacter-baumannii]	470
WP_237812691.1	DndB-ParB-nuclease	GCF_022014125.1	371	Bacteria>Proteobacteria>Gammaproteobacteria>Moraxellales>Moraxellaceae	Acinetobacter_baumannii	DNA-sulfur-modification-protein-DndB-[Acinetobacter-baumannii]	470
WP_199958127.1	DndB-ParB-nuclease	GCF_016502565.1	369	Bacteria>Proteobacteria>Gammaproteobacteria>Moraxellales>Moraxellaceae	Acinetobacter_bereziniae	DNA-sulfur-modification-protein-DndB-[Acinetobacter-bereziniae]	106648
WP_004991911.1	DndB-ParB-nuclease	GCF_001485045.1	411	Bacteria>Proteobacteria>Gammaproteobacteria>Moraxellales>Moraxellaceae	Acinetobacter	DGQHR-domain-containing-protein-[Acinetobacter-ursingii]	469
WP_004991905.1	DndB-ParB-nuclease	GCF_001485045.1	369	Bacteria>Proteobacteria>Gammaproteobacteria>Moraxellales>Moraxellaceae	Acinetobacter	DNA-sulfur-modification-protein-DndB-[Acinetobacter-ursingii]	469
WP_054580574.1	DndB-ParB-nuclease	GCF_001307195.1	355	Bacteria>Proteobacteria>Gammaproteobacteria>Moraxellales>Moraxellaceae	Acinetobacter_equi	DGQHR-domain-containing-protein-[Acinetobacter-equi]	1324350
WP_054580570.1	DndB-ParB-nuclease	GCF_001307195.1	373	Bacteria>Proteobacteria>Gammaproteobacteria>Moraxellales>Moraxellaceae	Acinetobacter_equi	DNA-sulfur-modification-protein-DndB-[Acinetobacter-equi]	1324350
WP_004718771.1	DndB-ParB-nuclease	GCF_000414055.1	409	Bacteria>Proteobacteria>Gammaproteobacteria>Moraxellales>Moraxellaceae	Acinetobacter_guillouiae	DNA-phosphorothioation-associated-DGQHR-protein-1-[Acinetobacter-guillouiae]	106649
WP_042140937.1	DndB-ParB-nuclease	GCF_000829655.1	371	Bacteria>Proteobacteria>Gammaproteobacteria>Moraxellales>Moraxellaceae	Acinetobacter_guillouiae	DNA-sulfur-modification-protein-DndB-[Acinetobacter-guillouiae]	106649
WP_161417526.1	DndB-ParB-nuclease	GCF_009899885.1	371	Bacteria>Proteobacteria>Gammaproteobacteria>Moraxellales>Moraxellaceae	Acinetobacter_haemolyticus	DNA-sulfur-modification-protein-DndB-[Acinetobacter-haemolyticus]	29430
WP_168390284.1	DndB-ParB-nuclease	GCF_012370935.1	331	Bacteria>Proteobacteria>Gammaproteobacteria>Moraxellales>Moraxellaceae	Acinetobacter_indicus	DGQHR-domain-containing-protein-[Acinetobacter-indicus]	756892
WP_168390288.1	DndB-ParB-nuclease	GCF_012370935.1	372	Bacteria>Proteobacteria>Gammaproteobacteria>Moraxellales>Moraxellaceae	Acinetobacter_indicus	DNA-sulfur-modification-protein-DndB-[Acinetobacter-indicus]	756892
WP_199955420.1	DndB-ParB-nuclease	GCF_016502415.1	429	Bacteria>Proteobacteria>Gammaproteobacteria>Moraxellales>Moraxellaceae	Acinetobacter_junii	DGQHR-domain-containing-protein-[Acinetobacter-junii]	40215
WP_199955425.1	DndB-ParB-nuclease	GCF_016502415.1	371	Bacteria>Proteobacteria>Gammaproteobacteria>Moraxellales>Moraxellaceae	Acinetobacter_junii	DNA-sulfur-modification-protein-DndB-[Acinetobacter-junii]	40215
WP_151862525.1	DndB-ParB-nuclease	GCF_009013375.1	424	Bacteria>Proteobacteria>Gammaproteobacteria>Moraxellales>Moraxellaceae	Acinetobacter	MULTISPECIES:-DGQHR-domain-containing-protein-[Acinetobacter]	469
WP_092819309.1	DndB-ParB-nuclease	GCF_900096895.1	372	Bacteria>Proteobacteria>Gammaproteobacteria>Moraxellales>Moraxellaceae	Acinetobacter	MULTISPECIES:-DNA-sulfur-modification-protein-DndB-[Acinetobacter]	469
WP_207693812.1	DndB-ParB-nuclease	GCF_017377435.1	425	Bacteria>Proteobacteria>Gammaproteobacteria>Moraxellales>Moraxellaceae	Acinetobacter_nosocomialis	DGQHR-domain-containing-protein-[Acinetobacter-nosocomialis]	106654
WP_207693808.1	DndB-ParB-nuclease	GCF_017377435.1	369	Bacteria>Proteobacteria>Gammaproteobacteria>Moraxellales>Moraxellaceae	Acinetobacter_nosocomialis	DNA-sulfur-modification-protein-DndB-[Acinetobacter-nosocomialis]	106654
WP_212266789.1	DndB-ParB-nuclease	GCF_018139515.1	372	Bacteria>Proteobacteria>Gammaproteobacteria>Moraxellales>Moraxellaceae	Acinetobacter_nosocomialis	DNA-sulfur-modification-protein-DndB-[Acinetobacter-nosocomialis]	106654
WP_228129083.1	DndB-ParB-nuclease	GCF_000368005.1	363	Bacteria>Proteobacteria>Gammaproteobacteria>Moraxellales>Moraxellaceae	Acinetobacter_parvus	DNA-sulfur-modification-protein-DndB-[Acinetobacter-parvus]	134533
WP_130072404.1	DndB-ParB-nuclease	GCF_004152775.1	435	Bacteria>Proteobacteria>Gammaproteobacteria>Moraxellales>Moraxellaceae	Acinetobacter_piscicola	DGQHR-domain-containing-protein-[Acinetobacter-piscicola]	2006115
WP_130072399.1	DndB-ParB-nuclease	GCF_004152775.1	373	Bacteria>Proteobacteria>Gammaproteobacteria>Moraxellales>Moraxellaceae	Acinetobacter_piscicola	DNA-sulfur-modification-protein-DndB-[Acinetobacter-piscicola]	2006115
WP_238514401.1	DndB-ParB-nuclease	GCF_022213985.1	417	Bacteria>Proteobacteria>Gammaproteobacteria>Moraxellales>Moraxellaceae	Acinetobacter_pittii	DNA-phosphorothioation-associated-DGQHR-protein-1-[Acinetobacter-pittii]	48296
WP_238514379.1	DndB-ParB-nuclease	GCF_022213985.1	371	Bacteria>Proteobacteria>Gammaproteobacteria>Moraxellales>Moraxellaceae	Acinetobacter_pittii	DNA-sulfur-modification-protein-DndB-[Acinetobacter-pittii]	48296
WP_087618684.1	DndB-ParB-nuclease	GCF_002174125.1	331	Bacteria>Proteobacteria>Gammaproteobacteria>Moraxellales>Moraxellaceae	Acinetobacter_populi	DGQHR-domain-containing-protein-[Acinetobacter-populi]	1582270
WP_087618680.1	DndB-ParB-nuclease	-	374	Bacteria>Proteobacteria>Gammaproteobacteria>Moraxellales>Moraxellaceae	Acinetobacter_populi	DNA-sulfur-modification-protein-DndB-[Acinetobacter-populi]	1582270
WP_074382652.1	DndB-ParB-nuclease	GCF_900088145.1	373	Bacteria>Proteobacteria>Gammaproteobacteria>Moraxellales>Moraxellaceae	Acinetobacter_pseudolwoffii	DNA-sulfur-modification-protein-DndB-[Acinetobacter-pseudolwoffii]	2053287
WP_235471587.1	DndB-ParB-nuclease	GCF_021538705.1	363	Bacteria>Proteobacteria>Gammaproteobacteria>Moraxellales>Moraxellaceae	Acinetobacter_soli	DNA-sulfur-modification-protein-DndB-[Acinetobacter-soli]	487316
MBY3618573.1	DndB-ParB-nuclease	GCA_019779985.1	365	Bacteria>Proteobacteria>Gammaproteobacteria>Moraxellales>Moraxellaceae	Acinetobacter_sp._CUI_P1	DNA-sulfur-modification-protein-DndB-[Acinetobacter-sp.-CUI-P1]	2726727
WP_104852685.1	DndB-ParB-nuclease	GCF_002946595.1	424	Bacteria>Proteobacteria>Gammaproteobacteria>Moraxellales>Moraxellaceae	Acinetobacter_sp._SWBY1	DNA-phosphorothioation-associated-DGQHR-protein-1-[Acinetobacter-sp.-SWBY1]	2079596
WP_104852680.1	DndB-ParB-nuclease	GCF_002946595.1	373	Bacteria>Proteobacteria>Gammaproteobacteria>Moraxellales>Moraxellaceae	Acinetobacter_sp._SWBY1	DNA-sulfur-modification-protein-DndB-[Acinetobacter-sp.-SWBY1]	2079596
WP_180190159.1	DndB-ParB-nuclease	GCF_013420765.1	373	Bacteria>Proteobacteria>Gammaproteobacteria>Moraxellales>Moraxellaceae	Acinetobacter_sp._YH01009	DNA-sulfur-modification-protein-DndB-[Acinetobacter-sp.-YH01009]	2601025
WP_034171424.1	DndB-ParB-nuclease	GCF_000804305.1	417	Bacteria>Proteobacteria>Gammaproteobacteria>Moraxellales>Moraxellaceae	Acinetobacter_sp._YZS-X1-1	DGQHR-domain-containing-protein-[Acinetobacter-sp.-YZS-X1-1]	1501691
WP_034171429.1	DndB-ParB-nuclease	GCF_000804305.1	371	Bacteria>Proteobacteria>Gammaproteobacteria>Moraxellales>Moraxellaceae	Acinetobacter_sp._YZS-X1-1	DNA-sulfur-modification-protein-DndB-[Acinetobacter-sp.-YZS-X1-1]	1501691
WP_171541369.1	DndB-ParB-nuclease	GCF_013004255.1	424	Bacteria>Proteobacteria>Gammaproteobacteria>Moraxellales>Moraxellaceae	Acinetobacter_terrae	DGQHR-domain-containing-protein-[Acinetobacter-terrae]	2731247
WP_171541373.1	DndB-ParB-nuclease	GCF_013004255.1	372	Bacteria>Proteobacteria>Gammaproteobacteria>Moraxellales>Moraxellaceae	Acinetobacter_terrae	DNA-sulfur-modification-protein-DndB-[Acinetobacter-terrae]	2731247
WP_151862524.1	DndB-ParB-nuclease	GCF_009013375.1	372	Bacteria>Proteobacteria>Gammaproteobacteria>Moraxellales>Moraxellaceae	Acinetobacter_ursingii	DNA-sulfur-modification-protein-DndB-[Acinetobacter-ursingii]	108980
WP_061517724.1	DndB-ParB-nuclease	GCF_001584665.1	393	Bacteria>Proteobacteria>Gammaproteobacteria>Moraxellales>Moraxellaceae	Acinetobacter_venetianus	DNA-phosphorothioation-associated-DGQHR-protein-1-[Acinetobacter-venetianus]	52133
WP_061525700.1	DndB-ParB-nuclease	GCF_001584655.1	373	Bacteria>Proteobacteria>Gammaproteobacteria>Moraxellales>Moraxellaceae	Acinetobacter_venetianus	DNA-sulfur-modification-protein-DndB-[Acinetobacter-venetianus]	52133
WP_236714458.1	DndB-ParB-nuclease	GCF_001584665.1	363	Bacteria>Proteobacteria>Gammaproteobacteria>Moraxellales>Moraxellaceae	Acinetobacter_venetianus	DNA-sulfur-modification-protein-DndB-[Acinetobacter-venetianus]	52133
KXZ62321.1	DndB-ParB-nuclease	GCA_001584665.1	363	Bacteria>Proteobacteria>Gammaproteobacteria>Moraxellales>Moraxellaceae	Acinetobacter_venetianus	hypothetical-protein-AVENLUH7437_03440-[Acinetobacter-venetianus]	52133
KXZ62326.1	DndB-ParB-nuclease	GCA_001584665.1	393	Bacteria>Proteobacteria>Gammaproteobacteria>Moraxellales>Moraxellaceae	Acinetobacter_venetianus	hypothetical-protein-AVENLUH7437_03445-[Acinetobacter-venetianus]	52133
WP_107866212.1	DndB-ParB-nuclease	GCF_003051055.1	403	Bacteria>Proteobacteria>Gammaproteobacteria>Moraxellales>Moraxellaceae	Agitococcus_lubricus	DGQHR-domain-containing-protein-[Agitococcus-lubricus]	1077255
WP_107866216.1	DndB-ParB-nuclease	GCF_003051055.1	360	Bacteria>Proteobacteria>Gammaproteobacteria>Moraxellales>Moraxellaceae	Agitococcus_lubricus	DNA-sulfur-modification-protein-DndB-[Agitococcus-lubricus]	1077255
WP_218947525.1	DndB-ParB-nuclease	GCF_013418535.1	435	Bacteria>Proteobacteria>Gammaproteobacteria>Moraxellales>Moraxellaceae	unclassified_Acinetobacter	MULTISPECIES:-DGQHR-domain-containing-protein-[unclassified-Acinetobacter]	196816
WP_200481970.1	DndB-ParB-nuclease	GCF_016624945.1	395	Bacteria>Proteobacteria>Gammaproteobacteria>Moraxellales>Moraxellaceae	unclassified_Acinetobacter	MULTISPECIES:-DNA-phosphorothioation-associated-DGQHR-protein-1-[unclassified-Acinetobacter]	196816
WP_180052915.1	DndB-ParB-nuclease	GCF_013418535.1	373	Bacteria>Proteobacteria>Gammaproteobacteria>Moraxellales>Moraxellaceae	unclassified_Acinetobacter	MULTISPECIES:-DNA-sulfur-modification-protein-DndB-[unclassified-Acinetobacter]	196816
WP_200481965.1	DndB-ParB-nuclease	GCF_016624945.1	371	Bacteria>Proteobacteria>Gammaproteobacteria>Moraxellales>Moraxellaceae	unclassified_Acinetobacter	MULTISPECIES:-DNA-sulfur-modification-protein-DndB-[unclassified-Acinetobacter]	196816
MBL7252029.1	DndB-ParB-nuclease	GCA_016785095.1	361	Bacteria>Proteobacteria>Gammaproteobacteria>Oceanospirillales>Alcanivoracaceae	Alcanivorax_marinus	DNA-sulfur-modification-protein-DndB-[Alcanivorax-marinus]	1177169
MCC4307087.1	DndB-ParB-nuclease	GCA_020782135.1	359	Bacteria>Proteobacteria>Gammaproteobacteria>Oceanospirillales>Alcanivoracaceae	Alcanivorax_marinus	DNA-sulfur-modification-protein-DndB-[Alcanivorax-marinus]	1177169
WP_203105465.1	DndB-ParB-nuclease	GCF_016785095.1	361	Bacteria>Proteobacteria>Gammaproteobacteria>Oceanospirillales>Alcanivoracaceae	Alcanivorax_marinus	DNA-sulfur-modification-protein-DndB-[Alcanivorax-marinus]	1177169
WP_228232226.1	DndB-ParB-nuclease	GCF_020782135.1	359	Bacteria>Proteobacteria>Gammaproteobacteria>Oceanospirillales>Alcanivoracaceae	Alcanivorax_marinus	DNA-sulfur-modification-protein-DndB-[Alcanivorax-marinus]	1177169
MBL7252031.1	DndB-ParB-nuclease	GCA_016785095.1	690	Bacteria>Proteobacteria>Gammaproteobacteria>Oceanospirillales>Alcanivoracaceae	Alcanivorax_marinus	hypothetical-protein-[Alcanivorax-marinus]	1177169
WP_203105467.1	DndB-ParB-nuclease	GCF_016785095.1	690	Bacteria>Proteobacteria>Gammaproteobacteria>Oceanospirillales>Alcanivoracaceae	Alcanivorax_marinus	hypothetical-protein-[Alcanivorax-marinus]	1177169
MCC1498177.1	DndB-ParB-nuclease	GCA_020667895.1	359	Bacteria>Proteobacteria>Gammaproteobacteria>Oceanospirillales>Alcanivoracaceae	Alcanivorax_sp._1008	DNA-sulfur-modification-protein-DndB-[Alcanivorax-sp.-1008]	2816853
WP_229149565.1	DndB-ParB-nuclease	GCF_020667895.1	359	Bacteria>Proteobacteria>Gammaproteobacteria>Oceanospirillales>Alcanivoracaceae	Alcanivorax_sp._1008	DNA-sulfur-modification-protein-DndB-[Alcanivorax-sp.-1008]	2816853
WP_229149568.1	DndB-ParB-nuclease	GCF_020667895.1	688	Bacteria>Proteobacteria>Gammaproteobacteria>Oceanospirillales>Alcanivoracaceae	Alcanivorax_sp._1008	hypothetical-protein-[Alcanivorax-sp.-1008]	2816853
MCC1498179.1	DndB-ParB-nuclease	GCA_020667895.1	688	Bacteria>Proteobacteria>Gammaproteobacteria>Oceanospirillales>Alcanivoracaceae	Alcanivorax_sp._1008	hypothetical-protein-J1C54_15260-[Alcanivorax-sp.-1008]	2816853
MBE8233347.1	DndB-ParB-nuclease	GCA_015163765.1	368	Bacteria>Proteobacteria>Gammaproteobacteria>Oceanospirillales>Endozoicomonadaceae	Endozoicomonadaceae_bacterium	DNA-sulfur-modification-protein-DndB-[Endozoicomonadaceae-bacterium]	2590577
WP_079254102.1	DndB-ParB-nuclease	GCF_001562015.1	373	Bacteria>Proteobacteria>Gammaproteobacteria>Oceanospirillales>Endozoicomonadaceae	Endozoicomonas_arenosclerae	DNA-sulfur-modification-protein-DndB-[Endozoicomonas-arenosclerae]	1633495
WP_066015820.1	DndB-ParB-nuclease	GCF_001647025.1	365	Bacteria>Proteobacteria>Gammaproteobacteria>Oceanospirillales>Endozoicomonadaceae	Endozoicomonas_atrinae	DNA-sulfur-modification-protein-DndB-[Endozoicomonas-atrinae]	1333660
WP_034834764.1	DndB-ParB-nuclease	GCF_000722635.1	349	Bacteria>Proteobacteria>Gammaproteobacteria>Oceanospirillales>Endozoicomonadaceae	Endozoicomonas_numazuensis	DNA-sulfur-modification-protein-DndB-[Endozoicomonas-numazuensis]	1137799
WP_011400707.1	DndB-ParB-nuclease	GCF_000012985.1	366	Bacteria>Proteobacteria>Gammaproteobacteria>Oceanospirillales>Hahellaceae	Hahella_chejuensis	DNA-sulfur-modification-protein-DndB-[Hahella-chejuensis]	158327
WP_219790987.1	DndB-ParB-nuclease	GCF_019430905.1	358	Bacteria>Proteobacteria>Gammaproteobacteria>Oceanospirillales>Halomonadaceae	Halomonas_antri	DNA-sulfur-modification-protein-DndB-[Halomonas-sp.-Y3S6]	2846777
MCD1652053.1	DndB-ParB-nuclease	GCA_021021635.1	371	Bacteria>Proteobacteria>Gammaproteobacteria>Oceanospirillales>Halomonadaceae	Halomonas_axialensis	DNA-sulfur-modification-protein-DndB-[Halomonas-axialensis]	115555
TFH85696.1	DndB-ParB-nuclease	GCA_004551485.1	370	Bacteria>Proteobacteria>Gammaproteobacteria>Oceanospirillales>Halomonadaceae	Halomonas_azerbaijanica	DNA-sulfur-modification-protein-DndB-[Halomonas-azerbaijanica]	2599398
WP_217873842.1	DndB-ParB-nuclease	-	369	Bacteria>Proteobacteria>Gammaproteobacteria>Oceanospirillales>Halomonadaceae	Halomonas_desiderata	DNA-sulfur-modification-protein-DndB-[Halomonas-desiderata]	52021
WP_086510477.1	DndB-ParB-nuclease	GCF_002151265.1	650	Bacteria>Proteobacteria>Gammaproteobacteria>Oceanospirillales>Halomonadaceae	Halomonas_desiderata	hypothetical-protein-[Halomonas-desiderata]	52021
WP_183315055.1	DndB-ParB-nuclease	GCF_014192285.1	369	Bacteria>Proteobacteria>Gammaproteobacteria>Oceanospirillales>Halomonadaceae	Halomonas_fontilapidosi	DNA-sulfur-modification-protein-DndB-[Halomonas-fontilapidosi]	616675
WP_102627105.1	DndB-ParB-nuclease	GCF_003202165.1	359	Bacteria>Proteobacteria>Gammaproteobacteria>Oceanospirillales>Halomonadaceae	Halomonas_heilongjiangensis	DNA-sulfur-modification-protein-DndB-[Halomonas-heilongjiangensis]	1387883
WP_084123806.1	DndB-ParB-nuclease	GCF_000800185.1	371	Bacteria>Proteobacteria>Gammaproteobacteria>Oceanospirillales>Halomonadaceae	Halomonas_hydrothermalis	DNA-sulfur-modification-protein-DndB-[Halomonas-hydrothermalis]	115561
WP_084123808.1	DndB-ParB-nuclease	GCF_000800185.1	733	Bacteria>Proteobacteria>Gammaproteobacteria>Oceanospirillales>Halomonadaceae	Halomonas_hydrothermalis	hypothetical-protein-[Halomonas-hydrothermalis]	115561
WP_230834830.1	DndB-ParB-nuclease	GCF_021021635.1	342	Bacteria>Proteobacteria>Gammaproteobacteria>Oceanospirillales>Halomonadaceae	Halomonas	MULTISPECIES:-DNA-sulfur-modification-protein-DndB-[Halomonas]	2745
MBH8581093.1	DndB-ParB-nuclease	GCA_016056335.1	402	Bacteria>Proteobacteria>Gammaproteobacteria>Oceanospirillales>Halomonadaceae	Halomonas_pacifica	DGQHR-domain-containing-protein-[Halomonas-pacifica]	77098
WP_198058110.1	DndB-ParB-nuclease	GCF_016056335.1	402	Bacteria>Proteobacteria>Gammaproteobacteria>Oceanospirillales>Halomonadaceae	Halomonas_pacifica	DNA-phosphorothioation-associated-DGQHR-protein-1-[Halomonas-pacifica]	77098
MBH8581098.1	DndB-ParB-nuclease	GCA_016056335.1	367	Bacteria>Proteobacteria>Gammaproteobacteria>Oceanospirillales>Halomonadaceae	Halomonas_pacifica	DNA-sulfur-modification-protein-DndB-[Halomonas-pacifica]	77098
WP_198058115.1	DndB-ParB-nuclease	GCF_016056335.1	367	Bacteria>Proteobacteria>Gammaproteobacteria>Oceanospirillales>Halomonadaceae	Halomonas_pacifica	DNA-sulfur-modification-protein-DndB-[Halomonas-pacifica]	77098
UEQ03893.1	DndB-ParB-nuclease	GCA_020865425.1	382	Bacteria>Proteobacteria>Gammaproteobacteria>Oceanospirillales>Halomonadaceae	Halomonas_profundus	DGQHR-domain-containing-protein-[Halomonas-profundus]	310345
UEQ03889.1	DndB-ParB-nuclease	GCA_020865425.1	358	Bacteria>Proteobacteria>Gammaproteobacteria>Oceanospirillales>Halomonadaceae	Halomonas_profundus	DNA-sulfur-modification-protein-DndB-[Halomonas-profundus]	310345
WP_232710991.1	DndB-ParB-nuclease	GCF_002810305.1	588	Bacteria>Proteobacteria>Gammaproteobacteria>Oceanospirillales>Halomonadaceae	Halomonas_sp._141	hypothetical-protein-[Halomonas-sp.-141]	2056666
TKJ09415.1	DndB-ParB-nuclease	GCA_005218165.1	358	Bacteria>Proteobacteria>Gammaproteobacteria>Oceanospirillales>Halomonadaceae	Halomonas_sp._15WGF	DNA-sulfur-modification-protein-DndB-[Halomonas-sp.-15WGF]	2570357
WP_192539717.1	DndB-ParB-nuclease	GCF_014861465.1	358	Bacteria>Proteobacteria>Gammaproteobacteria>Oceanospirillales>Halomonadaceae	Halomonas_sp._FME20	DNA-sulfur-modification-protein-DndB-[Halomonas-sp.-FME20]	2487703
KPQ26500.1	DndB-ParB-nuclease	GCA_001314745.1	366	Bacteria>Proteobacteria>Gammaproteobacteria>Oceanospirillales>Halomonadaceae	Halomonas_sp._HL-93	DNA-sulfur-modification-system-ATPase-DndB-[Halomonas-sp.-HL-93]	1666906
WP_206477969.1	DndB-ParB-nuclease	GCF_017168345.1	358	Bacteria>Proteobacteria>Gammaproteobacteria>Oceanospirillales>Halomonadaceae	Halomonas_sp._KRD171	DNA-sulfur-modification-protein-DndB-[Halomonas-sp.-KRD171]	2729726
WP_039868890.1	DndB-ParB-nuclease	GCF_000219565.1	358	Bacteria>Proteobacteria>Gammaproteobacteria>Oceanospirillales>Halomonadaceae	Halomonas_sp._TD01	DNA-sulfur-modification-protein-DndB-[Halomonas-sp.-TD01]	999141
WP_035581740.1	DndB-ParB-nuclease	GCF_000744395.1	358	Bacteria>Proteobacteria>Gammaproteobacteria>Oceanospirillales>Halomonadaceae	Halomonas_sp._TG39a	DNA-sulfur-modification-protein-DndB-[Halomonas-sp.-TG39a]	1415755
WP_086900327.1	DndB-ParB-nuclease	GCF_002157205.1	358	Bacteria>Proteobacteria>Gammaproteobacteria>Oceanospirillales>Halomonadaceae	Kushneria_marisflavi	DNA-sulfur-modification-protein-DndB-[Kushneria-marisflavi]	157779
WP_137095109.1	DndB-ParB-nuclease	GCF_024158645.1	358	Bacteria>Proteobacteria>Gammaproteobacteria>Oceanospirillales>Halomonadaceae	unclassified_Halomonas	DNA-sulfur-modification-protein-DndB-[Halomonas-sp.-15WGF]	2609666
WP_197030423.1	DndB-ParB-nuclease	GCF_000568385.1	369	Bacteria>Proteobacteria>Gammaproteobacteria>Oceanospirillales>Halomonadaceae	unclassified_Halomonas	DNA-sulfur-modification-protein-DndB-[Halomonas-sp.-ML-15]	2609666
WP_205210247.1	DndB-ParB-nuclease	GCF_016924145.1	364	Bacteria>Proteobacteria>Gammaproteobacteria>Oceanospirillales>Oceanospirillaceae	Amphritea_pacifica	DNA-sulfur-modification-protein-DndB-[Amphritea-sp.-ZJ14W]	2811233
WP_007018021.1	DndB-ParB-nuclease	GCF_000153565.1	365	Bacteria>Proteobacteria>Gammaproteobacteria>Oceanospirillales>Oceanospirillaceae	Bermanella_marisrubri	DNA-sulfur-modification-protein-DndB-[Bermanella-marisrubri]	207949
WP_133504235.1	DndB-ParB-nuclease	GCF_004362145.1	415	Bacteria>Proteobacteria>Gammaproteobacteria>Oceanospirillales>Oceanospirillaceae	Marinomonas_balearica	DGQHR-domain-containing-protein-[Marinomonas-balearica]	491947
WP_208111435.1	DndB-ParB-nuclease	-	366	Bacteria>Proteobacteria>Gammaproteobacteria>Oceanospirillales>Oceanospirillaceae	Marinomonas_balearica	DNA-sulfur-modification-protein-DndB-[Marinomonas-balearica]	491947
WP_115467357.1	DndB-ParB-nuclease	GCF_003362755.1	363	Bacteria>Proteobacteria>Gammaproteobacteria>Oceanospirillales>Oceanospirillaceae	Marinomonas_piezotolerans	DNA-sulfur-modification-protein-DndB-[Marinomonas-piezotolerans]	2213058
WP_115467362.1	DndB-ParB-nuclease	GCF_003362755.1	564	Bacteria>Proteobacteria>Gammaproteobacteria>Oceanospirillales>Oceanospirillaceae	Marinomonas_piezotolerans	hypothetical-protein-[Marinomonas-piezotolerans]	2213058
WP_067341413.1	DndB-ParB-nuclease	GCF_900089745.1	387	Bacteria>Proteobacteria>Gammaproteobacteria>Oceanospirillales>Oceanospirillaceae	Marinomonas_spartinae	DGQHR-domain-containing-protein-[Marinomonas-spartinae]	1792290
WP_067341401.1	DndB-ParB-nuclease	GCF_900089745.1	364	Bacteria>Proteobacteria>Gammaproteobacteria>Oceanospirillales>Oceanospirillaceae	Marinomonas_spartinae	DNA-sulfur-modification-protein-DndB-[Marinomonas-spartinae]	1792290
WP_027850873.1	DndB-ParB-nuclease	GCF_000686905.1	362	Bacteria>Proteobacteria>Gammaproteobacteria>Oceanospirillales>Oceanospirillaceae	Marinospirillum_insulare	DNA-sulfur-modification-protein-DndB-[Marinospirillum-insulare]	217169
MBE0506982.1	DndB-ParB-nuclease	GCA_014859565.1	362	Bacteria>Proteobacteria>Gammaproteobacteria>Oceanospirillales>Oceanospirillaceae	Marinospirillum_sp.	DNA-sulfur-modification-protein-DndB-[Marinospirillum-sp.]	2183934
MBE0506987.1	DndB-ParB-nuclease	GCA_014859565.1	646	Bacteria>Proteobacteria>Gammaproteobacteria>Oceanospirillales>Oceanospirillaceae	Marinospirillum_sp.	hypothetical-protein-[Marinospirillum-sp.]	2183934
WP_210397393.1	DndB-ParB-nuclease	GCF_017912535.1	426	Bacteria>Proteobacteria>Gammaproteobacteria>Oceanospirillales>Oceanospirillaceae	Motiliproteus_sediminis	DGQHR-domain-containing-protein-[Motiliproteus-sediminis]	1468178
WP_210397389.1	DndB-ParB-nuclease	GCF_017912535.1	365	Bacteria>Proteobacteria>Gammaproteobacteria>Oceanospirillales>Oceanospirillaceae	Motiliproteus_sediminis	DNA-sulfur-modification-protein-DndB-[Motiliproteus-sediminis]	1468178
WP_201348557.1	DndB-ParB-nuclease	GCF_016592555.1	386	Bacteria>Proteobacteria>Gammaproteobacteria>Oceanospirillales>Oceanospirillaceae	Neptunomonas_japonica	DGQHR-domain-containing-protein-[Neptunomonas-japonica]	417574
WP_201348561.1	DndB-ParB-nuclease	GCF_016592555.1	361	Bacteria>Proteobacteria>Gammaproteobacteria>Oceanospirillales>Oceanospirillaceae	Neptunomonas_japonica	DNA-sulfur-modification-protein-DndB-[Neptunomonas-japonica]	417574
WP_090728892.1	DndB-ParB-nuclease	GCF_016082375.1	360	Bacteria>Proteobacteria>Gammaproteobacteria>Oceanospirillales>Oceanospirillaceae	Neptunomonas_qingdaonensis	DNA-sulfur-modification-protein-DndB-[Neptunomonas-qingdaonensis]	1045558
MAX86745.1	DndB-ParB-nuclease	GCA_002706225.1	366	Bacteria>Proteobacteria>Gammaproteobacteria>Oceanospirillales>Oceanospirillaceae	Oceanospirillaceae_bacterium	DNA-sulfur-modification-protein-DndB-[Oceanospirillaceae-bacterium]	1899355
CCK76817.1	DndB-ParB-nuclease	GCA_000967895.1	366	Bacteria>Proteobacteria>Gammaproteobacteria>Oceanospirillales>Oceanospirillaceae	Oleispira_antarctica_RB-8	conserved-hypothetical-protein-[Oleispira-antarctica-RB-8]	698738
MBL4798496.1	DndB-ParB-nuclease	GCA_016764675.1	365	Bacteria>Proteobacteria>Gammaproteobacteria>Oceanospirillales>Oceanospirillaceae	Oleispira_sp.	DNA-sulfur-modification-protein-DndB-[Oleispira-sp.]	2024846
KZY75026.1	DndB-ParB-nuclease	GCA_001635765.1	259	Bacteria>Proteobacteria>Gammaproteobacteria>Oceanospirillales>Oleiphilaceae	Oleiphilus_sp._HI0068	DNA-sulfur-modification-protein-DndB,-partial-[Oleiphilus-sp.-HI0068]	1822244
WP_068739906.1	DndB-ParB-nuclease	GCF_001635235.1	397	Bacteria>Proteobacteria>Gammaproteobacteria>Oceanospirillales>Oleiphilaceae	unclassified_Oleiphilus	MULTISPECIES:-DGQHR-domain-containing-protein-[unclassified-Oleiphilus]	2631174

Accession	Annotation	GCA	Length	Phyletic.spread	Strain.name	Defline	Species.Taxid
WP_068739830.1	DndB-ParB-nuclease	GCF_001635235.1	362	Bacteria>Proteobacteria>Gammaproteobacteria>Oceanospirillales>Oleiphilaceae	unclassified_Oleiphilus	MULTISPECIES:-DNA-sulfur-modification-protein-DndB-[unclassified-Oleiphilus]	2631174
WP_108124332.1	DndB-ParB-nuclease	GCF_003367315.1	359	Bacteria>Proteobacteria>Gammaproteobacteria>Oceanospirillales>Saccharospirillaceae	Saccharospirillum_mangrovi	DNA-sulfur-modification-protein-DndB-[Saccharospirillum-mangrovi]	2161747
WP_119394084.1	DndB-ParB-nuclease	GCF_003577475.1	360	Bacteria>Proteobacteria>Gammaproteobacteria>Oceanospirillales>Saccharospirillaceae	Salinibius_halmophilus	DNA-sulfur-modification-protein-DndB-[Salinibius-halmophilus]	1853216
WP_216355085.1	DndB-ParB-nuclease	-	194	Bacteria>Proteobacteria>Gammaproteobacteria>Orbales>Orbaceae	Gilliamella_apicola	DGQHR-domain-containing-protein-[Gilliamella-apicola]	1196095
WP_039127239.1	DndB-ParB-nuclease	GCF_003202655.1	360	Bacteria>Proteobacteria>Gammaproteobacteria>Orbales>Orbaceae	Gilliamella_apicola	DNA-sulfur-modification-protein-DndB-[Gilliamella-apicola]	1196095
WP_086336134.1	DndB-ParB-nuclease	GCF_002142285.1	360	Bacteria>Proteobacteria>Gammaproteobacteria>Orbales>Orbaceae	Gilliamella_apicola	DNA-sulfur-modification-protein-DndB-[Gilliamella-apicola]	1196095
WP_216355084.1	DndB-ParB-nuclease	-	110	Bacteria>Proteobacteria>Gammaproteobacteria>Orbales>Orbaceae	Gilliamella_apicola	hypothetical-protein-[Gilliamella-apicola]	1196095
KES19505.1	DndB-ParB-nuclease	GCA_000725165.1	360	Bacteria>Proteobacteria>Gammaproteobacteria>Orbales>Orbaceae	Gilliamella_apicola_SCGC_AB-598-B02	hypothetical-protein-GASC598B02_015910-[Gilliamella-apicola-SCGC-AB-598-B02]	1445513
WP_110453920.1	DndB-ParB-nuclease	GCF_003202875.1	360	Bacteria>Proteobacteria>Gammaproteobacteria>Orbales>Orbaceae	Gilliamella_apis	DNA-sulfur-modification-protein-DndB-[Gilliamella-apis]	1970738
WP_198227280.1	DndB-ParB-nuclease	GCF_016101785.1	360	Bacteria>Proteobacteria>Gammaproteobacteria>Orbales>Orbaceae	unclassified_Gilliamella	MULTISPECIES:-DNA-sulfur-modification-protein-DndB-[unclassified-Gilliamella]	2685620
WP_198236353.1	DndB-ParB-nuclease	GCF_016101825.1	360	Bacteria>Proteobacteria>Gammaproteobacteria>Orbales>Orbaceae	unclassified_Gilliamella	MULTISPECIES:-DNA-sulfur-modification-protein-DndB-[unclassified-Gilliamella]	2685620
SHK65334.1	DndB-ParB-nuclease	GCA_900142385.1	700	Bacteria>Proteobacteria>Gammaproteobacteria>Pseudomonadales>Marinobacteraceae	Marinobacter_antarcticus	DNA-sulfur-modification-associated-[Marinobacter-antarcticus]	564117
SHK65278.1	DndB-ParB-nuclease	GCA_900142385.1	360	Bacteria>Proteobacteria>Gammaproteobacteria>Pseudomonadales>Marinobacteraceae	Marinobacter_antarcticus	DNA-sulfur-modification-protein-DndB-[Marinobacter-antarcticus]	564117
WP_072798377.1	DndB-ParB-nuclease	GCF_900142385.1	360	Bacteria>Proteobacteria>Gammaproteobacteria>Pseudomonadales>Marinobacteraceae	Marinobacter_antarcticus	DNA-sulfur-modification-protein-DndB-[Marinobacter-antarcticus]	564117
WP_072798380.1	DndB-ParB-nuclease	GCF_900142385.1	700	Bacteria>Proteobacteria>Gammaproteobacteria>Pseudomonadales>Marinobacteraceae	Marinobacter_antarcticus	hypothetical-protein-[Marinobacter-antarcticus]	564117
WP_061333480.1	DndB-ParB-nuclease	GCF_001574445.1	424	Bacteria>Proteobacteria>Gammaproteobacteria>Pseudomonadales>Marinobacteraceae	Marinobacter_excellens	DGQHR-domain-containing-protein-[Marinobacter-excellens]	218670
WP_099616162.1	DndB-ParB-nuclease	GCF_002744735.1	396	Bacteria>Proteobacteria>Gammaproteobacteria>Pseudomonadales>Marinobacteraceae	Marinobacter_guineae	DGQHR-domain-containing-protein-[Marinobacter-guineae]	432303
PHQ26083.1	DndB-ParB-nuclease	GCA_002744735.1	363	Bacteria>Proteobacteria>Gammaproteobacteria>Pseudomonadales>Marinobacteraceae	Marinobacter_guineae	DNA-sulfur-modification-protein-DndB-[Marinobacter-guineae]	432303
WP_228152733.1	DndB-ParB-nuclease	GCF_002744735.1	359	Bacteria>Proteobacteria>Gammaproteobacteria>Pseudomonadales>Marinobacteraceae	Marinobacter_guineae	DNA-sulfur-modification-protein-DndB-[Marinobacter-guineae]	432303
PHQ26079.1	DndB-ParB-nuclease	GCA_002744735.1	396	Bacteria>Proteobacteria>Gammaproteobacteria>Pseudomonadales>Marinobacteraceae	Marinobacter_guineae	hypothetical-protein-CLH62_00240-[Marinobacter-guineae]	432303
TBW50367.1	DndB-ParB-nuclease	GCA_004327985.1	393	Bacteria>Proteobacteria>Gammaproteobacteria>Pseudomonadales>Marinobacteraceae	Marinobacter_halodurans	DGQHR-domain-containing-protein-[Marinobacter-halodurans]	2528979
WP_165495797.1	DndB-ParB-nuclease	GCF_004327985.1	382	Bacteria>Proteobacteria>Gammaproteobacteria>Pseudomonadales>Marinobacteraceae	Marinobacter_halodurans	DGQHR-domain-containing-protein-[Marinobacter-halodurans]	2528979
TBW50371.1	DndB-ParB-nuclease	GCA_004327985.1	363	Bacteria>Proteobacteria>Gammaproteobacteria>Pseudomonadales>Marinobacteraceae	Marinobacter_halodurans	DNA-sulfur-modification-protein-DndB-[Marinobacter-halodurans]	2528979
WP_131483366.1	DndB-ParB-nuclease	-	363	Bacteria>Proteobacteria>Gammaproteobacteria>Pseudomonadales>Marinobacteraceae	Marinobacter_halodurans	DNA-sulfur-modification-protein-DndB-[Marinobacter-halodurans]	2528979
WP_036134109.1	DndB-ParB-nuclease	GCF_000708045.1	359	Bacteria>Proteobacteria>Gammaproteobacteria>Pseudomonadales>Marinobacteraceae	Marinobacter	MULTISPECIES:-DNA-sulfur-modification-protein-DndB-[Marinobacter]	2742
WP_226610395.1	DndB-ParB-nuclease	GCF_020524675.1	585	Bacteria>Proteobacteria>Gammaproteobacteria>Pseudomonadales>Marinobacteraceae	Marinobacter_nauticus	unnamed-protein-product]	2743
MBS8230287.1	DndB-ParB-nuclease	GCA_018424725.1	358	Bacteria>Proteobacteria>Gammaproteobacteria>Pseudomonadales>Marinobacteraceae	Marinobacter_salarius	DNA-sulfur-modification-protein-DndB-[Marinobacter-salarius]	1420917
WP_213479618.1	DndB-ParB-nuclease	GCF_018424725.1	358	Bacteria>Proteobacteria>Gammaproteobacteria>Pseudomonadales>Marinobacteraceae	Marinobacter_salarius	DNA-sulfur-modification-protein-DndB-[Marinobacter-salarius]	1420917
MBS8230285.1	DndB-ParB-nuclease	GCA_018424725.1	705	Bacteria>Proteobacteria>Gammaproteobacteria>Pseudomonadales>Marinobacteraceae	Marinobacter_salarius	hypothetical-protein-[Marinobacter-salarius]	1420917
WP_213479616.1	DndB-ParB-nuclease	GCF_018424725.1	705	Bacteria>Proteobacteria>Gammaproteobacteria>Pseudomonadales>Marinobacteraceae	Marinobacter_salarius	hypothetical-protein-[Marinobacter-salarius]	1420917
WP_075196079.1	DndB-ParB-nuclease	-	363	Bacteria>Proteobacteria>Gammaproteobacteria>Pseudomonadales>Marinobacteraceae	Marinobacter_sp._C18	DNA-sulfur-modification-protein-DndB-[Marinobacter-sp.-C18]	1772288
WP_022992092.1	DndB-ParB-nuclease	GCF_000475355.1	359	Bacteria>Proteobacteria>Gammaproteobacteria>Pseudomonadales>Marinobacteraceae	Marinobacter_sp._C1S70	DNA-sulfur-modification-protein-DndB-[Marinobacter-sp.-C1S70]	1396859
HDZ39879.1	DndB-ParB-nuclease	GCA_011050535.1	409	Bacteria>Proteobacteria>Gammaproteobacteria>Pseudomonadales>Marinobacteraceae	Marinobacter_sp.	DGQHR-domain-containing-protein-[Marinobacter-sp.]	50741
MBO6813108.1	DndB-ParB-nuclease	GCA_017643545.1	360	Bacteria>Proteobacteria>Gammaproteobacteria>Pseudomonadales>Marinobacteraceae	Marinobacter_sp.	DNA-sulfur-modification-protein-DndB-[Marinobacter-sp.]	50741
WP_081698862.1	DndB-ParB-nuclease	GCF_000475315.1	368	Bacteria>Proteobacteria>Gammaproteobacteria>Pseudomonadales>Marinobacteraceae	Marinobacter_sp._EN3	DGQHR-domain-containing-protein-[Marinobacter-sp.-EN3]	1397533
WP_023011228.1	DndB-ParB-nuclease	GCF_000475315.1	373	Bacteria>Proteobacteria>Gammaproteobacteria>Pseudomonadales>Marinobacteraceae	Marinobacter_sp._EN3	DNA-sulfur-modification-protein-DndB-[Marinobacter-sp.-EN3]	1397533
MBW7469717.1	DndB-ParB-nuclease	GCA_019443085.1	363	Bacteria>Proteobacteria>Gammaproteobacteria>Pseudomonadales>Marinobacteraceae	Marinobacter_sp._F4218	DNA-sulfur-modification-protein-DndB-[Marinobacter-sp.-F4218]	2862868
WP_219865551.1	DndB-ParB-nuclease	GCF_019443085.1	363	Bacteria>Proteobacteria>Gammaproteobacteria>Pseudomonadales>Marinobacteraceae	Marinobacter_sp._F4218	DNA-sulfur-modification-protein-DndB-[Marinobacter-sp.-F4218]	2862868
MBO6813106.1	DndB-ParB-nuclease	GCA_017643545.1	706	Bacteria>Proteobacteria>Gammaproteobacteria>Pseudomonadales>Marinobacteraceae	Marinobacter_sp.	hypothetical-protein-[Marinobacter-sp.]	50741
WP_058091265.1	DndB-ParB-nuclease	GCF_001447155.2	409	Bacteria>Proteobacteria>Gammaproteobacteria>Pseudomonadales>Marinobacteraceae	Marinobacter_sp._LQ44	DGQHR-domain-containing-protein-[Marinobacter-sp.-LQ44]	1749259
WP_058091229.1	DndB-ParB-nuclease	GCF_001447155.2	359	Bacteria>Proteobacteria>Gammaproteobacteria>Pseudomonadales>Marinobacteraceae	Marinobacter_sp._LQ44	DNA-sulfur-modification-protein-DndB-[Marinobacter-sp.-LQ44]	1749259
WP_198866827.1	DndB-ParB-nuclease	GCF_016406165.1	361	Bacteria>Proteobacteria>Gammaproteobacteria>Pseudomonadales>Pseudomonadaceae	Azotobacter_chroococcum	DNA-sulfur-modification-protein-DndB-[Azotobacter-chroococcum]	353
EIU3620488.1	DndB-ParB-nuclease	GCA_021644545.1	415	Bacteria>Proteobacteria>Gammaproteobacteria>Pseudomonadales>Pseudomonadaceae	Pseudomonas_aeruginosa	DGQHR-domain-containing-protein-[Pseudomonas-aeruginosa]	287
KAB0740550.1	DndB-ParB-nuclease	GCA_008802595.1	415	Bacteria>Proteobacteria>Gammaproteobacteria>Pseudomonadales>Pseudomonadaceae	Pseudomonas_aeruginosa	DGQHR-domain-containing-protein-[Pseudomonas-aeruginosa]	287
MBI7468809.1	DndB-ParB-nuclease	GCA_016339065.1	415	Bacteria>Proteobacteria>Gammaproteobacteria>Pseudomonadales>Pseudomonadaceae	Pseudomonas_aeruginosa	DGQHR-domain-containing-protein-[Pseudomonas-aeruginosa]	287
MCC4281564.1	DndB-ParB-nuclease	GCA_020781855.1	357	Bacteria>Proteobacteria>Gammaproteobacteria>Pseudomonadales>Pseudomonadaceae	Pseudomonas_aeruginosa	DGQHR-domain-containing-protein-[Pseudomonas-aeruginosa]	287
WP_023081372.1	DndB-ParB-nuclease	GCF_900707475.1	357	Bacteria>Proteobacteria>Gammaproteobacteria>Pseudomonadales>Pseudomonadaceae	Pseudomonas_aeruginosa	DGQHR-domain-containing-protein-[Pseudomonas-aeruginosa]	287
WP_079383963.1	DndB-ParB-nuclease	GCF_016745155.1	363	Bacteria>Proteobacteria>Gammaproteobacteria>Pseudomonadales>Pseudomonadaceae	Pseudomonas_aeruginosa	DGQHR-domain-containing-protein-[Pseudomonas-aeruginosa]	287
WP_124213811.1	DndB-ParB-nuclease	GCF_024714025.1	353	Bacteria>Proteobacteria>Gammaproteobacteria>Pseudomonadales>Pseudomonadaceae	Pseudomonas_aeruginosa	DGQHR-domain-containing-protein-[Pseudomonas-aeruginosa]	287
WP_151163737.1	DndB-ParB-nuclease	GCF_008802595.1	415	Bacteria>Proteobacteria>Gammaproteobacteria>Pseudomonadales>Pseudomonadaceae	Pseudomonas_aeruginosa	DGQHR-domain-containing-protein-[Pseudomonas-aeruginosa]	287
WP_161806845.1	DndB-ParB-nuclease	GCF_001280755.1	415	Bacteria>Proteobacteria>Gammaproteobacteria>Pseudomonadales>Pseudomonadaceae	Pseudomonas_aeruginosa	DGQHR-domain-containing-protein-[Pseudomonas-aeruginosa]	287
WP_196187729.1	DndB-ParB-nuclease	GCF_015680105.1	357	Bacteria>Proteobacteria>Gammaproteobacteria>Pseudomonadales>Pseudomonadaceae	Pseudomonas_aeruginosa	DGQHR-domain-containing-protein-[Pseudomonas-aeruginosa]	287
WP_228203225.1	DndB-ParB-nuclease	GCF_020781855.1	357	Bacteria>Proteobacteria>Gammaproteobacteria>Pseudomonadales>Pseudomonadaceae	Pseudomonas_aeruginosa	DGQHR-domain-containing-protein-[Pseudomonas-aeruginosa]	287
EIU3620485.1	DndB-ParB-nuclease	GCA_021644545.1	359	Bacteria>Proteobacteria>Gammaproteobacteria>Pseudomonadales>Pseudomonadaceae	Pseudomonas_aeruginosa	DNA-sulfur-modification-protein-DndB-[Pseudomonas-aeruginosa]	287
KAB0740546.1	DndB-ParB-nuclease	GCA_008802595.1	366	Bacteria>Proteobacteria>Gammaproteobacteria>Pseudomonadales>Pseudomonadaceae	Pseudomonas_aeruginosa	DNA-sulfur-modification-protein-DndB-[Pseudomonas-aeruginosa]	287
MBI7468812.1	DndB-ParB-nuclease	GCA_016339065.1	359	Bacteria>Proteobacteria>Gammaproteobacteria>Pseudomonadales>Pseudomonadaceae	Pseudomonas_aeruginosa	DNA-sulfur-modification-protein-DndB-[Pseudomonas-aeruginosa]	287
MCC4281558.1	DndB-ParB-nuclease	GCA_020781855.1	366	Bacteria>Proteobacteria>Gammaproteobacteria>Pseudomonadales>Pseudomonadaceae	Pseudomonas_aeruginosa	DNA-sulfur-modification-protein-DndB-[Pseudomonas-aeruginosa]	287
RFJ35024.1	DndB-ParB-nuclease	GCA_003411585.1	366	Bacteria>Proteobacteria>Gammaproteobacteria>Pseudomonadales>Pseudomonadaceae	Pseudomonas_aeruginosa	DNA-sulfur-modification-protein-DndB-[Pseudomonas-aeruginosa]	287
RPU10900.1	DndB-ParB-nuclease	GCA_003858155.1	366	Bacteria>Proteobacteria>Gammaproteobacteria>Pseudomonadales>Pseudomonadaceae	Pseudomonas_aeruginosa	DNA-sulfur-modification-protein-DndB-[Pseudomonas-aeruginosa]	287
RPU44301.1	DndB-ParB-nuclease	GCA_003837225.1	366	Bacteria>Proteobacteria>Gammaproteobacteria>Pseudomonadales>Pseudomonadaceae	Pseudomonas_aeruginosa	DNA-sulfur-modification-protein-DndB-[Pseudomonas-aeruginosa]	287
RUC61431.1	DndB-ParB-nuclease	GCA_003974145.1	366	Bacteria>Proteobacteria>Gammaproteobacteria>Pseudomonadales>Pseudomonadaceae	Pseudomonas_aeruginosa	DNA-sulfur-modification-protein-DndB-[Pseudomonas-aeruginosa]	287
WP_023081377.1	DndB-ParB-nuclease	GCF_900707475.1	366	Bacteria>Proteobacteria>Gammaproteobacteria>Pseudomonadales>Pseudomonadaceae	Pseudomonas_aeruginosa	DNA-sulfur-modification-protein-DndB-[Pseudomonas-aeruginosa]	287
WP_033939754.1	DndB-ParB-nuclease	GCF_000791325.1	360	Bacteria>Proteobacteria>Gammaproteobacteria>Pseudomonadales>Pseudomonadaceae	Pseudomonas_aeruginosa	DNA-sulfur-modification-protein-DndB-[Pseudomonas-aeruginosa]	287
WP_044264552.1	DndB-ParB-nuclease	GCF_900708185.1	361	Bacteria>Proteobacteria>Gammaproteobacteria>Pseudomonadales>Pseudomonadaceae	Pseudomonas_aeruginosa	DNA-sulfur-modification-protein-DndB-[Pseudomonas-aeruginosa]	287
WP_052155983.1	DndB-ParB-nuclease	GCF_000793665.1	361	Bacteria>Proteobacteria>Gammaproteobacteria>Pseudomonadales>Pseudomonadaceae	Pseudomonas_aeruginosa	DNA-sulfur-modification-protein-DndB-[Pseudomonas-aeruginosa]	287
WP_052156300.1	DndB-ParB-nuclease	GCF_013285125.1	361	Bacteria>Proteobacteria>Gammaproteobacteria>Pseudomonadales>Pseudomonadaceae	Pseudomonas_aeruginosa	DNA-sulfur-modification-protein-DndB-[Pseudomonas-aeruginosa]	287
WP_071535852.1	DndB-ParB-nuclease	GCF_020781855.1	366	Bacteria>Proteobacteria>Gammaproteobacteria>Pseudomonadales>Pseudomonadaceae	Pseudomonas_aeruginosa	DNA-sulfur-modification-protein-DndB-[Pseudomonas-aeruginosa]	287
WP_071540053.1	DndB-ParB-nuclease	GCF_000271365.1	366	Bacteria>Proteobacteria>Gammaproteobacteria>Pseudomonadales>Pseudomonadaceae	Pseudomonas_aeruginosa	DNA-sulfur-modification-protein-DndB-[Pseudomonas-aeruginosa]	287
WP_071550077.1	DndB-ParB-nuclease	GCF_013178785.1	366	Bacteria>Proteobacteria>Gammaproteobacteria>Pseudomonadales>Pseudomonadaceae	Pseudomonas_aeruginosa	DNA-sulfur-modification-protein-DndB-[Pseudomonas-aeruginosa]	287
WP_079383028.1	DndB-ParB-nuclease	GCF_021061525.1	360	Bacteria>Proteobacteria>Gammaproteobacteria>Pseudomonadales>Pseudomonadaceae	Pseudomonas_aeruginosa	DNA-sulfur-modification-protein-DndB-[Pseudomonas-aeruginosa]	287
WP_079387923.1	DndB-ParB-nuclease	GCF_900708705.1	366	Bacteria>Proteobacteria>Gammaproteobacteria>Pseudomonadales>Pseudomonadaceae	Pseudomonas_aeruginosa	DNA-sulfur-modification-protein-DndB-[Pseudomonas-aeruginosa]	287
WP_121211367.1	DndB-ParB-nuclease	GCF_025697585.1	366	Bacteria>Proteobacteria>Gammaproteobacteria>Pseudomonadales>Pseudomonadaceae	Pseudomonas_aeruginosa	DNA-sulfur-modification-protein-DndB-[Pseudomonas-aeruginosa]	287
WP_124169766.1	DndB-ParB-nuclease	GCF_020640395.1	360	Bacteria>Proteobacteria>Gammaproteobacteria>Pseudomonadales>Pseudomonadaceae	Pseudomonas_aeruginosa	DNA-sulfur-modification-protein-DndB-[Pseudomonas-aeruginosa]	287
WP_124213816.1	DndB-ParB-nuclease	GCF_022552935.1	366	Bacteria>Proteobacteria>Gammaproteobacteria>Pseudomonadales>Pseudomonadaceae	Pseudomonas_aeruginosa	DNA-sulfur-modification-protein-DndB-[Pseudomonas-aeruginosa]	287
WP_196187732.1	DndB-ParB-nuclease	GCF_015680105.1	366	Bacteria>Proteobacteria>Gammaproteobacteria>Pseudomonadales>Pseudomonadaceae	Pseudomonas_aeruginosa	DNA-sulfur-modification-protein-DndB-[Pseudomonas-aeruginosa]	287
WP_200582117.1	DndB-ParB-nuclease	GCF_016622185.1	366	Bacteria>Proteobacteria>Gammaproteobacteria>Pseudomonadales>Pseudomonadaceae	Pseudomonas_aeruginosa	DNA-sulfur-modification-protein-DndB-[Pseudomonas-aeruginosa]	287
WP_228203219.1	DndB-ParB-nuclease	GCF_020781855.1	366	Bacteria>Proteobacteria>Gammaproteobacteria>Pseudomonadales>Pseudomonadaceae	Pseudomonas_aeruginosa	DNA-sulfur-modification-protein-DndB-[Pseudomonas-aeruginosa]	287
WP_235577375.1	DndB-ParB-nuclease	GCF_001280755.1	365	Bacteria>Proteobacteria>Gammaproteobacteria>Pseudomonadales>Pseudomonadaceae	Pseudomonas_aeruginosa	DNA-sulfur-modification-protein-DndB-[Pseudomonas-aeruginosa]	287
WP_237754639.1	DndB-ParB-nuclease	GCF_018966705.1	366	Bacteria>Proteobacteria>Gammaproteobacteria>Pseudomonadales>Pseudomonadaceae	Pseudomonas_aeruginosa	DNA-sulfur-modification-protein-DndB-[Pseudomonas-aeruginosa]	287
RUC61437.1	DndB-ParB-nuclease	GCA_003974145.1	652	Bacteria>Proteobacteria>Gammaproteobacteria>Pseudomonadales>Pseudomonadaceae	Pseudomonas_aeruginosa	hypothetical-protein-IPC1396_22550-[Pseudomonas-aeruginosa]	287
MCC4281563.1	DndB-ParB-nuclease	GCA_020781855.1	730	Bacteria>Proteobacteria>Gammaproteobacteria>Pseudomonadales>Pseudomonadaceae	Pseudomonas_aeruginosa	hypothetical-protein-LL325_27550-[Pseudomonas-aeruginosa]	287
WP_023081373.1	DndB-ParB-nuclease	GCF_900707475.1	583	Bacteria>Proteobacteria>Gammaproteobacteria>Pseudomonadales>Pseudomonadaceae	Pseudomonas_aeruginosa	hypothetical-protein-[Pseudomonas-aeruginosa]	287
WP_023121803.1	DndB-ParB-nuclease	GCF_000271365.1	587	Bacteria>Proteobacteria>Gammaproteobacteria>Pseudomonadales>Pseudomonadaceae	Pseudomonas_aeruginosa	hypothetical-protein-[Pseudomonas-aeruginosa]	287
WP_034077319.1	DndB-ParB-nuclease	GCF_021061525.1	632	Bacteria>Proteobacteria>Gammaproteobacteria>Pseudomonadales>Pseudomonadaceae	Pseudomonas_aeruginosa	hypothetical-protein-[Pseudomonas-aeruginosa]	287
WP_071535855.1	DndB-ParB-nuclease	GCF_020781855.1	577	Bacteria>Proteobacteria>Gammaproteobacteria>Pseudomonadales>Pseudomonadaceae	Pseudomonas_aeruginosa	hypothetical-protein-[Pseudomonas-aeruginosa]	287
WP_079389079.1	DndB-ParB-nuclease	GCF_000793665.1	691	Bacteria>Proteobacteria>Gammaproteobacteria>Pseudomonadales>Pseudomonadaceae	Pseudomonas_aeruginosa	hypothetical-protein-[Pseudomonas-aeruginosa]	287
WP_079389431.1	DndB-ParB-nuclease	GCF_000794335.1	691	Bacteria>Proteobacteria>Gammaproteobacteria>Pseudomonadales>Pseudomonadaceae	Pseudomonas_aeruginosa	hypothetical-protein-[Pseudomonas-aeruginosa]	287
WP_121211357.1	DndB-ParB-nuclease	GCF_025697585.1	652	Bacteria>Proteobacteria>Gammaproteobacteria>Pseudomonadales>Pseudomonadaceae	Pseudomonas_aeruginosa	hypothetical-protein-[Pseudomonas-aeruginosa]	287
WP_124169768.1	DndB-ParB-nuclease	GCF_020640395.1	665	Bacteria>Proteobacteria>Gammaproteobacteria>Pseudomonadales>Pseudomonadaceae	Pseudomonas_aeruginosa	hypothetical-protein-[Pseudomonas-aeruginosa]	287
WP_124213812.1	DndB-ParB-nuclease	GCF_024714025.1	583	Bacteria>Proteobacteria>Gammaproteobacteria>Pseudomonadales>Pseudomonadaceae	Pseudomonas_aeruginosa	hypothetical-protein-[Pseudomonas-aeruginosa]	287
WP_141563017.1	DndB-ParB-nuclease	GCF_013285125.1	708	Bacteria>Proteobacteria>Gammaproteobacteria>Pseudomonadales>Pseudomonadaceae	Pseudomonas_aeruginosa	hypothetical-protein-[Pseudomonas-aeruginosa]	287
WP_160328377.1	DndB-ParB-nuclease	GCF_013178785.1	730	Bacteria>Proteobacteria>Gammaproteobacteria>Pseudomonadales>Pseudomonadaceae	Pseudomonas_aeruginosa	hypothetical-protein-[Pseudomonas-aeruginosa]	287
WP_196187730.1	DndB-ParB-nuclease	GCF_015680105.1	730	Bacteria>Proteobacteria>Gammaproteobacteria>Pseudomonadales>Pseudomonadaceae	Pseudomonas_aeruginosa	hypothetical-protein-[Pseudomonas-aeruginosa]	287
WP_228203224.1	DndB-ParB-nuclease	GCF_020781855.1	730	Bacteria>Proteobacteria>Gammaproteobacteria>Pseudomonadales>Pseudomonadaceae	Pseudomonas_aeruginosa	hypothetical-protein-[Pseudomonas-aeruginosa]	287
HBN8433577.1	DndB-ParB-nuclease	GCA_021587605.1	359	Bacteria>Proteobacteria>Gammaproteobacteria>Pseudomonadales>Pseudomonadaceae	Pseudomonas_aeruginosa	TPA:-DNA-sulfur-modification-protein-DndB-[Pseudomonas-aeruginosa]	287
HBO1348756.1	DndB-ParB-nuclease	GCA_021597085.1	359	Bacteria>Proteobacteria>Gammaproteobacteria>Pseudomonadales>Pseudomonadaceae	Pseudomonas_aeruginosa	TPA:-DNA-sulfur-modification-protein-DndB-[Pseudomonas-aeruginosa]	287
HBO1348754.1	DndB-ParB-nuclease	GCA_021597085.1	681	Bacteria>Proteobacteria>Gammaproteobacteria>Pseudomonadales>Pseudomonadaceae	Pseudomonas_aeruginosa	TPA:-hypothetical-protein-L4F11_002907-[Pseudomonas-aeruginosa]	287
WP_223886802.1	DndB-ParB-nuclease	GCF_003701185.1	306	Bacteria>Proteobacteria>Gammaproteobacteria>Pseudomonadales>Pseudomonadaceae	Pseudomonas_amygdali	DGQHR-domain-containing-protein-[Pseudomonas-amygdali]	47877
WP_122389926.1	DndB-ParB-nuclease	GCF_003701185.1	708	Bacteria>Proteobacteria>Gammaproteobacteria>Pseudomonadales>Pseudomonadaceae	Pseudomonas_amygdali	hypothetical-protein-[Pseudomonas-amygdali]	47877
WP_061434216.1	DndB-ParB-nuclease	GCF_001579805.1	366	Bacteria>Proteobacteria>Gammaproteobacteria>Pseudomonadales>Pseudomonadaceae	Pseudomonas_azotoformans	DNA-sulfur-modification-protein-DndB-[Pseudomonas-azotoformans]	47878
WP_177429534.1	DndB-ParB-nuclease	GCF_021389915.1	403	Bacteria>Proteobacteria>Gammaproteobacteria>Pseudomonadales>Pseudomonadaceae	Pseudomonas_brassicacearum	DGQHR-domain-containing-protein-[Pseudomonas-brassicacearum]	930166
UCR86792.1	DndB-ParB-nuclease	GCA_020424105.1	360	Bacteria>Proteobacteria>Gammaproteobacteria>Pseudomonadales>Pseudomonadaceae	Pseudomonas_chlororaphis	DNA-sulfur-modification-protein-DndB-[Pseudomonas-chlororaphis]	587753
WP_226305321.1	DndB-ParB-nuclease	GCF_020424105.1	360	Bacteria>Proteobacteria>Gammaproteobacteria>Pseudomonadales>Pseudomonadaceae	Pseudomonas_chlororaphis	DNA-sulfur-modification-protein-DndB-[Pseudomonas-chlororaphis]	587753
UCR86790.1	DndB-ParB-nuclease	GCA_020424105.1	666	Bacteria>Proteobacteria>Gammaproteobacteria>Pseudomonadales>Pseudomonadaceae	Pseudomonas_chlororaphis	hypothetical-protein-K9V45_12035-[Pseudomonas-chlororaphis]	587753
WP_226305319.1	DndB-ParB-nuclease	GCF_020424105.1	666	Bacteria>Proteobacteria>Gammaproteobacteria>Pseudomonadales>Pseudomonadaceae	Pseudomonas_chlororaphis	hypothetical-protein-[Pseudomonas-chlororaphis]	587753
WP_122320567.1	DndB-ParB-nuclease	-	402	Bacteria>Proteobacteria>Gammaproteobacteria>Pseudomonadales>Pseudomonadaceae	Pseudomonas_cichorii	DGQHR-domain-containing-protein-[Pseudomonas-cichorii]	36746
WP_183143745.1	DndB-ParB-nuclease	GCF_003701435.1	419	Bacteria>Proteobacteria>Gammaproteobacteria>Pseudomonadales>Pseudomonadaceae	Pseudomonas_cichorii	DGQHR-domain-containing-protein-[Pseudomonas-cichorii]	36746
WP_200998827.1	DndB-ParB-nuclease	GCF_015471265.1	414	Bacteria>Proteobacteria>Gammaproteobacteria>Pseudomonadales>Pseudomonadaceae	Pseudomonas_cichorii	DGQHR-domain-containing-protein-[Pseudomonas-cichorii]	36746
WP_221616943.1	DndB-ParB-nuclease	GCF_019719075.1	419	Bacteria>Proteobacteria>Gammaproteobacteria>Pseudomonadales>Pseudomonadaceae	Pseudomonas_cichorii	DGQHR-domain-containing-protein-[Pseudomonas-cichorii]	36746
WP_221033360.1	DndB-ParB-nuclease	-	358	Bacteria>Proteobacteria>Gammaproteobacteria>Pseudomonadales>Pseudomonadaceae	Pseudomonas_cichorii	DNA-sulfur-modification-protein-DndB-[Pseudomonas-cichorii]	36746
WP_221581759.1	DndB-ParB-nuclease	GCF_019718815.1	360	Bacteria>Proteobacteria>Gammaproteobacteria>Pseudomonadales>Pseudomonadaceae	Pseudomonas_cichorii	DNA-sulfur-modification-protein-DndB-[Pseudomonas-cichorii]	36746
WP_221616951.1	DndB-ParB-nuclease	-	358	Bacteria>Proteobacteria>Gammaproteobacteria>Pseudomonadales>Pseudomonadaceae	Pseudomonas_cichorii	DNA-sulfur-modification-protein-DndB-[Pseudomonas-cichorii]	36746
WP_236249291.1	DndB-ParB-nuclease	GCF_015471265.1	379	Bacteria>Proteobacteria>Gammaproteobacteria>Pseudomonadales>Pseudomonadaceae	Pseudomonas_cichorii	DNA-sulfur-modification-protein-DndB-[Pseudomonas-cichorii]	36746
WP_221581761.1	DndB-ParB-nuclease	GCF_019718815.1	708	Bacteria>Proteobacteria>Gammaproteobacteria>Pseudomonadales>Pseudomonadaceae	Pseudomonas_cichorii	hypothetical-protein-[Pseudomonas-cichorii]	36746
WP_096101238.1	DndB-ParB-nuclease	GCF_002318645.1	379	Bacteria>Proteobacteria>Gammaproteobacteria>Pseudomonadales>Pseudomonadaceae	Pseudomonas_congelans	DGQHR-domain-containing-protein-[Pseudomonas-congelans]	200452
WP_096101244.1	DndB-ParB-nuclease	GCF_002318645.1	394	Bacteria>Proteobacteria>Gammaproteobacteria>Pseudomonadales>Pseudomonadaceae	Pseudomonas_congelans	DGQHR-domain-containing-protein-[Pseudomonas-congelans]	200452
WP_096101248.1	DndB-ParB-nuclease	GCF_002318645.1	360	Bacteria>Proteobacteria>Gammaproteobacteria>Pseudomonadales>Pseudomonadaceae	Pseudomonas_congelans	DNA-sulfur-modification-protein-DndB-[Pseudomonas-congelans]	200452
WP_096101250.1	DndB-ParB-nuclease	GCF_002318645.1	708	Bacteria>Proteobacteria>Gammaproteobacteria>Pseudomonadales>Pseudomonadaceae	Pseudomonas_congelans	hypothetical-protein-[Pseudomonas-congelans]	200452
WP_191867893.1	DndB-ParB-nuclease	GCF_011765175.1	362	Bacteria>Proteobacteria>Gammaproteobacteria>Pseudomonadales>Pseudomonadaceae	Pseudomonas_coronafaciens	DNA-sulfur-modification-protein-DndB-[Pseudomonas-coronafaciens]	53409
WP_226439086.1	DndB-ParB-nuclease	GCF_020515645.1	403	Bacteria>Proteobacteria>Gammaproteobacteria>Pseudomonadales>Pseudomonadaceae	Pseudomonas	DGQHR-domain-containing-protein-[Pseudomonas-sp.-MWU16_30316]	286
WP_150596556.1	DndB-ParB-nuclease	GCF_902497985.1	360	Bacteria>Proteobacteria>Gammaproteobacteria>Pseudomonadales>Pseudomonadaceae	Pseudomonas	DNA-sulfur-modification-protein-DndB-[Pseudomonas-fluorescens]	286
WP_226439091.1	DndB-ParB-nuclease	GCF_020515645.1	360	Bacteria>Proteobacteria>Gammaproteobacteria>Pseudomonadales>Pseudomonadaceae	Pseudomonas	DNA-sulfur-modification-protein-DndB-[Pseudomonas-sp.-MWU16_30316]	286
WP_100264298.1	DndB-ParB-nuclease	GCF_021389915.1	362	Bacteria>Proteobacteria>Gammaproteobacteria>Pseudomonadales>Pseudomonadaceae	Pseudomonas	DNA-sulfur-modification-protein-DndB-[Pseudomonas-sp.-WCS365]	286
WP_213624584.1	DndB-ParB-nuclease	GCF_023701885.1	360	Bacteria>Proteobacteria>Gammaproteobacteria>Pseudomonadales>Pseudomonadaceae	Pseudomonas	DNA-sulfur-modification-protein-DndB-[Pseudomonas-syringae]	286
WP_143481162.1	DndB-ParB-nuclease	GCF_900167635.1	366	Bacteria>Proteobacteria>Gammaproteobacteria>Pseudomonadales>Pseudomonadaceae	Pseudomonas_extremaustralis	DNA-sulfur-modification-protein-DndB-[Pseudomonas-extremaustralis]	359110

Accession	Annotation	GCA	Length	Phyletic.spread	Strain.name	Defline	Species.Taxid
WP_078833124.1	DndB-ParB-nuclease	GCF_900167635.1	647	Bacteria>Proteobacteria>Gammaproteobacteria>Pseudomonadales>Pseudomonadaceae	Pseudomonas_extremaustralis	hypothetical-protein-[Pseudomonas-extremaustralis]	359110
WP_143481163.1	DndB-ParB-nuclease	GCF_900167635.1	578	Bacteria>Proteobacteria>Gammaproteobacteria>Pseudomonadales>Pseudomonadaceae	Pseudomonas_extremaustralis	hypothetical-protein-[Pseudomonas-extremaustralis]	359110
WP_058545059.1	DndB-ParB-nuclease	GCF_001468775.2	482	Bacteria>Proteobacteria>Gammaproteobacteria>Pseudomonadales>Pseudomonadaceae	Pseudomonas_fluorescens	DGQHR-domain-containing-protein-[Pseudomonas-fluorescens]	294
WP_123452820.1	DndB-ParB-nuclease	-	399	Bacteria>Proteobacteria>Gammaproteobacteria>Pseudomonadales>Pseudomonadaceae	Pseudomonas_fluorescens	DGQHR-domain-containing-protein-[Pseudomonas-fluorescens]	294
WP_191627069.1	DndB-ParB-nuclease	GCF_902497935.1	403	Bacteria>Proteobacteria>Gammaproteobacteria>Pseudomonadales>Pseudomonadaceae	Pseudomonas_fluorescens	DGQHR-domain-containing-protein-[Pseudomonas-fluorescens]	294
RON79959.1	DndB-ParB-nuclease	GCA_003732425.1	399	Bacteria>Proteobacteria>Gammaproteobacteria>Pseudomonadales>Pseudomonadaceae	Pseudomonas_fluorescens	DNA-phosphorothioation-associated-DGQHR-protein-1-[Pseudomonas-fluorescens]	294
RON79123.1	DndB-ParB-nuclease	GCA_003732425.1	362	Bacteria>Proteobacteria>Gammaproteobacteria>Pseudomonadales>Pseudomonadaceae	Pseudomonas_fluorescens	DNA-sulfur-modification-protein-DndB-[Pseudomonas-fluorescens]	294
WP_011332373.1	DndB-ParB-nuclease	GCF_000012445.1	375	Bacteria>Proteobacteria>Gammaproteobacteria>Pseudomonadales>Pseudomonadaceae	Pseudomonas_fluorescens	DNA-sulfur-modification-protein-DndB-[Pseudomonas-fluorescens]	294
WP_111539363.1	DndB-ParB-nuclease	-	369	Bacteria>Proteobacteria>Gammaproteobacteria>Pseudomonadales>Pseudomonadaceae	Pseudomonas_fluorescens	DNA-sulfur-modification-protein-DndB-[Pseudomonas-fluorescens]	294
WP_123451520.1	DndB-ParB-nuclease	GCF_003732425.1	362	Bacteria>Proteobacteria>Gammaproteobacteria>Pseudomonadales>Pseudomonadaceae	Pseudomonas_fluorescens	DNA-sulfur-modification-protein-DndB-[Pseudomonas-fluorescens]	294
WP_123594475.1	DndB-ParB-nuclease	GCF_003732405.1	372	Bacteria>Proteobacteria>Gammaproteobacteria>Pseudomonadales>Pseudomonadaceae	Pseudomonas_fluorescens	DNA-sulfur-modification-protein-DndB-[Pseudomonas-fluorescens]	294
WP_154974245.1	DndB-ParB-nuclease	GCF_902497935.1	360	Bacteria>Proteobacteria>Gammaproteobacteria>Pseudomonadales>Pseudomonadaceae	Pseudomonas_fluorescens	DNA-sulfur-modification-protein-DndB-[Pseudomonas-fluorescens]	294
WP_239065512.1	DndB-ParB-nuclease	GCF_010448615.1	379	Bacteria>Proteobacteria>Gammaproteobacteria>Pseudomonadales>Pseudomonadaceae	Pseudomonas_fluorescens	DNA-sulfur-modification-protein-DndB-[Pseudomonas-fluorescens]	294
WP_168224077.1	DndB-ParB-nuclease	GCF_012273645.1	385	Bacteria>Proteobacteria>Gammaproteobacteria>Pseudomonadales>Pseudomonadaceae	Pseudomonas_fluorescens_group	MULTISPECIES:-DGQHR-domain-containing-protein-[Pseudomonas-fluorescens-group]	136843
WP_168224084.1	DndB-ParB-nuclease	GCF_012273645.1	360	Bacteria>Proteobacteria>Gammaproteobacteria>Pseudomonadales>Pseudomonadaceae	Pseudomonas_fluorescens_group	MULTISPECIES:-DNA-sulfur-modification-protein-DndB-[Pseudomonas-fluorescens-group]	136843
WP_123594479.1	DndB-ParB-nuclease	GCF_003732405.1	647	Bacteria>Proteobacteria>Gammaproteobacteria>Pseudomonadales>Pseudomonadaceae	Pseudomonas_fluorescens	hypothetical-protein-[Pseudomonas-fluorescens]	294
WP_150596558.1	DndB-ParB-nuclease	GCF_902497985.1	765	Bacteria>Proteobacteria>Gammaproteobacteria>Pseudomonadales>Pseudomonadaceae	Pseudomonas_fluorescens	hypothetical-protein-[Pseudomonas-fluorescens]	294
WP_154974244.1	DndB-ParB-nuclease	GCF_902497935.1	708	Bacteria>Proteobacteria>Gammaproteobacteria>Pseudomonadales>Pseudomonadaceae	Pseudomonas_fluorescens	hypothetical-protein-[Pseudomonas-fluorescens]	294
WP_155742053.1	DndB-ParB-nuclease	GCF_001468775.2	665	Bacteria>Proteobacteria>Gammaproteobacteria>Pseudomonadales>Pseudomonadaceae	Pseudomonas_fluorescens	hypothetical-protein-[Pseudomonas-fluorescens]	294
WP_163975313.1	DndB-ParB-nuclease	GCF_010448615.1	665	Bacteria>Proteobacteria>Gammaproteobacteria>Pseudomonadales>Pseudomonadaceae	Pseudomonas_fluorescens	hypothetical-protein-[Pseudomonas-fluorescens]	294
ABA72481.1	DndB-ParB-nuclease	GCA_000012445.1	375	Bacteria>Proteobacteria>Gammaproteobacteria>Pseudomonadales>Pseudomonadaceae	Pseudomonas_fluorescens_Pf0-1	putative-bacteriophage-protein-[Pseudomonas-fluorescens-Pf0-1]	205922
WP_169845419.1	DndB-ParB-nuclease	GCF_012986685.1	418	Bacteria>Proteobacteria>Gammaproteobacteria>Pseudomonadales>Pseudomonadaceae	Pseudomonas_fragi	DGQHR-domain-containing-protein-[Pseudomonas-fragi]	296
WP_169845424.1	DndB-ParB-nuclease	GCF_012986685.1	361	Bacteria>Proteobacteria>Gammaproteobacteria>Pseudomonadales>Pseudomonadaceae	Pseudomonas_fragi	DNA-sulfur-modification-protein-DndB-[Pseudomonas-fragi]	296
WP_157969664.1	DndB-ParB-nuclease	GCF_003231275.1	418	Bacteria>Proteobacteria>Gammaproteobacteria>Pseudomonadales>Pseudomonadaceae	Pseudomonas_huaxiensis	DGQHR-domain-containing-protein-[Pseudomonas-huaxiensis]	2213017
WP_110968937.1	DndB-ParB-nuclease	GCF_003231275.1	361	Bacteria>Proteobacteria>Gammaproteobacteria>Pseudomonadales>Pseudomonadaceae	Pseudomonas_huaxiensis	DNA-sulfur-modification-protein-DndB-[Pseudomonas-huaxiensis]	2213017
WP_213624585.1	DndB-ParB-nuclease	GCF_023701885.1	682	Bacteria>Proteobacteria>Gammaproteobacteria>Pseudomonadales>Pseudomonadaceae	Pseudomonas	hypothetical-protein-[Pseudomonas-syringae]	286
WP_110718461.1	DndB-ParB-nuclease	GCF_003205375.1	482	Bacteria>Proteobacteria>Gammaproteobacteria>Pseudomonadales>Pseudomonadaceae	Pseudomonas_jessenii	DGQHR-domain-containing-protein-[Pseudomonas-jessenii]	77298
WP_110718454.1	DndB-ParB-nuclease	GCF_003205375.1	360	Bacteria>Proteobacteria>Gammaproteobacteria>Pseudomonadales>Pseudomonadaceae	Pseudomonas_jessenii	DNA-sulfur-modification-protein-DndB-[Pseudomonas-jessenii]	77298
WP_110718452.1	DndB-ParB-nuclease	GCF_003205375.1	665	Bacteria>Proteobacteria>Gammaproteobacteria>Pseudomonadales>Pseudomonadaceae	Pseudomonas_jessenii	hypothetical-protein-[Pseudomonas-jessenii]	77298
NNA53897.1	DndB-ParB-nuclease	GCA_012986545.1	495	Bacteria>Proteobacteria>Gammaproteobacteria>Pseudomonadales>Pseudomonadaceae	Pseudomonas_lactis	DGQHR-domain-containing-protein-[Pseudomonas-lactis]	1615674
WP_169913706.1	DndB-ParB-nuclease	GCF_012986545.1	495	Bacteria>Proteobacteria>Gammaproteobacteria>Pseudomonadales>Pseudomonadaceae	Pseudomonas_lactis	DGQHR-domain-containing-protein-[Pseudomonas-lactis]	1615674
NNA53902.1	DndB-ParB-nuclease	GCA_012986545.1	360	Bacteria>Proteobacteria>Gammaproteobacteria>Pseudomonadales>Pseudomonadaceae	Pseudomonas_lactis	DNA-sulfur-modification-protein-DndB-[Pseudomonas-lactis]	1615674
WP_169913714.1	DndB-ParB-nuclease	GCF_012986545.1	360	Bacteria>Proteobacteria>Gammaproteobacteria>Pseudomonadales>Pseudomonadaceae	Pseudomonas_lactis	DNA-sulfur-modification-protein-DndB-[Pseudomonas-lactis]	1615674
WP_055134720.1	DndB-ParB-nuclease	GCF_001412185.1	360	Bacteria>Proteobacteria>Gammaproteobacteria>Pseudomonadales>Pseudomonadaceae	Pseudomonas_mediterranea	DNA-sulfur-modification-protein-DndB-[Pseudomonas-mediterranea]	183795
WP_159266621.1	DndB-ParB-nuclease	GCF_009831395.1	363	Bacteria>Proteobacteria>Gammaproteobacteria>Pseudomonadales>Pseudomonadaceae	Pseudomonas_monteilii	DGQHR-domain-containing-protein-[Pseudomonas-monteilii]	76759
WP_233454311.1	DndB-ParB-nuclease	GCF_009831395.1	369	Bacteria>Proteobacteria>Gammaproteobacteria>Pseudomonadales>Pseudomonadaceae	Pseudomonas_monteilii	DNA-sulfur-modification-protein-DndB-[Pseudomonas-monteilii]	76759
WP_150760988.1	DndB-ParB-nuclease	GCF_902497805.1	405	Bacteria>Proteobacteria>Gammaproteobacteria>Pseudomonadales>Pseudomonadaceae	Pseudomonas	MULTISPECIES:-DNA-phosphorothioation-associated-DGQHR-protein-1-[Pseudomonas]	286
WP_057416152.1	DndB-ParB-nuclease	GCF_902498175.1	366	Bacteria>Proteobacteria>Gammaproteobacteria>Pseudomonadales>Pseudomonadaceae	Pseudomonas	MULTISPECIES:-DNA-sulfur-modification-protein-DndB-[Pseudomonas]	286
WP_058545066.1	DndB-ParB-nuclease	GCF_001468775.2	360	Bacteria>Proteobacteria>Gammaproteobacteria>Pseudomonadales>Pseudomonadaceae	Pseudomonas	MULTISPECIES:-DNA-sulfur-modification-protein-DndB-[Pseudomonas]	286
WP_108538370.1	DndB-ParB-nuclease	GCF_902497935.1	360	Bacteria>Proteobacteria>Gammaproteobacteria>Pseudomonadales>Pseudomonadaceae	Pseudomonas	MULTISPECIES:-DNA-sulfur-modification-protein-DndB-[Pseudomonas]	286
WP_150347177.1	DndB-ParB-nuclease	GCF_008693965.1	363	Bacteria>Proteobacteria>Gammaproteobacteria>Pseudomonadales>Pseudomonadaceae	Pseudomonas	MULTISPECIES:-DNA-sulfur-modification-protein-DndB-[Pseudomonas]	286
WP_150760986.1	DndB-ParB-nuclease	GCF_902497805.1	361	Bacteria>Proteobacteria>Gammaproteobacteria>Pseudomonadales>Pseudomonadaceae	Pseudomonas	MULTISPECIES:-DNA-sulfur-modification-protein-DndB-[Pseudomonas]	286
WP_150347182.1	DndB-ParB-nuclease	GCF_008693965.1	731	Bacteria>Proteobacteria>Gammaproteobacteria>Pseudomonadales>Pseudomonadaceae	Pseudomonas	MULTISPECIES:-hypothetical-protein-[Pseudomonas]	286
WP_130137889.1	DndB-ParB-nuclease	GCF_004210175.1	495	Bacteria>Proteobacteria>Gammaproteobacteria>Pseudomonadales>Pseudomonadaceae	Pseudomonas_orientalis	DGQHR-domain-containing-protein-[Pseudomonas-orientalis]	76758
WP_130137894.1	DndB-ParB-nuclease	GCF_004210175.1	360	Bacteria>Proteobacteria>Gammaproteobacteria>Pseudomonadales>Pseudomonadaceae	Pseudomonas_orientalis	DNA-sulfur-modification-protein-DndB-[Pseudomonas-orientalis]	76758
WP_130137895.1	DndB-ParB-nuclease	GCF_004210175.1	682	Bacteria>Proteobacteria>Gammaproteobacteria>Pseudomonadales>Pseudomonadaceae	Pseudomonas_orientalis	hypothetical-protein-[Pseudomonas-orientalis]	76758
MXS19115.1	DndB-ParB-nuclease	GCA_009832405.1	357	Bacteria>Proteobacteria>Gammaproteobacteria>Pseudomonadales>Pseudomonadaceae	Pseudomonas_oryzihabitans	DGQHR-domain-containing-protein-[Pseudomonas-oryzihabitans]	47885
WP_160922913.1	DndB-ParB-nuclease	GCF_009832405.1	357	Bacteria>Proteobacteria>Gammaproteobacteria>Pseudomonadales>Pseudomonadaceae	Pseudomonas_oryzihabitans	DGQHR-domain-containing-protein-[Pseudomonas-oryzihabitans]	47885
MXS19104.1	DndB-ParB-nuclease	GCA_009832405.1	366	Bacteria>Proteobacteria>Gammaproteobacteria>Pseudomonadales>Pseudomonadaceae	Pseudomonas_oryzihabitans	DNA-sulfur-modification-protein-DndB-[Pseudomonas-oryzihabitans]	47885
WP_160922903.1	DndB-ParB-nuclease	GCF_009832405.1	366	Bacteria>Proteobacteria>Gammaproteobacteria>Pseudomonadales>Pseudomonadaceae	Pseudomonas_oryzihabitans	DNA-sulfur-modification-protein-DndB-[Pseudomonas-oryzihabitans]	47885
WP_090262893.1	DndB-ParB-nuclease	GCF_900099785.1	363	Bacteria>Proteobacteria>Gammaproteobacteria>Pseudomonadales>Pseudomonadaceae	Pseudomonas_panipatensis	DNA-sulfur-modification-protein-DndB-[Pseudomonas-panipatensis]	428992
WP_121731794.1	DndB-ParB-nuclease	GCF_003671865.1	405	Bacteria>Proteobacteria>Gammaproteobacteria>Pseudomonadales>Pseudomonadaceae	Pseudomonas_prosekii	DNA-phosphorothioation-associated-DGQHR-protein-1-[Pseudomonas-prosekii]	1148509
RLU09079.1	DndB-ParB-nuclease	GCA_003671895.1	361	Bacteria>Proteobacteria>Gammaproteobacteria>Pseudomonadales>Pseudomonadaceae	Pseudomonas_prosekii	DNA-sulfur-modification-protein-DndB-[Pseudomonas-prosekii]	1148509
WP_121731795.1	DndB-ParB-nuclease	GCF_003671865.1	361	Bacteria>Proteobacteria>Gammaproteobacteria>Pseudomonadales>Pseudomonadaceae	Pseudomonas_prosekii	DNA-sulfur-modification-protein-DndB-[Pseudomonas-prosekii]	1148509
RLU09080.1	DndB-ParB-nuclease	GCA_003671895.1	405	Bacteria>Proteobacteria>Gammaproteobacteria>Pseudomonadales>Pseudomonadaceae	Pseudomonas_prosekii	hypothetical-protein-CS078_13070-[Pseudomonas-prosekii]	1148509
WP_108544315.1	DndB-ParB-nuclease	GCF_003057655.1	360	Bacteria>Proteobacteria>Gammaproteobacteria>Pseudomonadales>Pseudomonadaceae	Pseudomonas_protegens	DNA-sulfur-modification-protein-DndB-[Pseudomonas-protegens]	380021
WP_236058866.1	DndB-ParB-nuclease	GCF_017948435.1	369	Bacteria>Proteobacteria>Gammaproteobacteria>Pseudomonadales>Pseudomonadaceae	Pseudomonas_protegens	DNA-sulfur-modification-protein-DndB-[Pseudomonas-protegens]	380021
WP_181321572.1	DndB-ParB-nuclease	GCF_003057655.1	666	Bacteria>Proteobacteria>Gammaproteobacteria>Pseudomonadales>Pseudomonadaceae	Pseudomonas_protegens	hypothetical-protein-[Pseudomonas-protegens]	380021
WP_074584347.1	DndB-ParB-nuclease	GCF_900102665.1	374	Bacteria>Proteobacteria>Gammaproteobacteria>Pseudomonadales>Pseudomonadaceae	Pseudomonas_psychrotolerans	DNA-sulfur-modification-protein-DndB-[Pseudomonas-psychrotolerans]	237610
WP_081323343.1	DndB-ParB-nuclease	GCF_001477295.1	374	Bacteria>Proteobacteria>Gammaproteobacteria>Pseudomonadales>Pseudomonadaceae	Pseudomonas_psychrotolerans	DNA-sulfur-modification-protein-DndB-[Pseudomonas-psychrotolerans]	237610
WP_153010406.1	DndB-ParB-nuclease	GCF_001477295.1	578	Bacteria>Proteobacteria>Gammaproteobacteria>Pseudomonadales>Pseudomonadaceae	Pseudomonas_psychrotolerans	hypothetical-protein-[Pseudomonas-psychrotolerans]	237610
QVL17258.1	DndB-ParB-nuclease	GCA_018398475.1	360	Bacteria>Proteobacteria>Gammaproteobacteria>Pseudomonadales>Pseudomonadaceae	Pseudomonas_qingdaonensis	DNA-sulfur-modification-protein-DndB-[Pseudomonas-qingdaonensis]	2056231
WP_213605976.1	DndB-ParB-nuclease	GCF_018398475.1	360	Bacteria>Proteobacteria>Gammaproteobacteria>Pseudomonadales>Pseudomonadaceae	Pseudomonas_qingdaonensis	DNA-sulfur-modification-protein-DndB-[Pseudomonas-qingdaonensis]	2056231
QVL17256.1	DndB-ParB-nuclease	GCA_018398475.1	666	Bacteria>Proteobacteria>Gammaproteobacteria>Pseudomonadales>Pseudomonadaceae	Pseudomonas_qingdaonensis	hypothetical-protein-KH389_17820-[Pseudomonas-qingdaonensis]	2056231
WP_213605974.1	DndB-ParB-nuclease	GCF_018398475.1	666	Bacteria>Proteobacteria>Gammaproteobacteria>Pseudomonadales>Pseudomonadaceae	Pseudomonas_qingdaonensis	hypothetical-protein-[Pseudomonas-qingdaonensis]	2056231
WP_207886928.1	DndB-ParB-nuclease	GCF_017491605.1	360	Bacteria>Proteobacteria>Gammaproteobacteria>Pseudomonadales>Pseudomonadaceae	Pseudomonas_sp._30_B	DNA-sulfur-modification-protein-DndB-[Pseudomonas-sp.-30_B]	2813575
WP_234562668.1	DndB-ParB-nuclease	GCF_021462485.1	366	Bacteria>Proteobacteria>Gammaproteobacteria>Pseudomonadales>Pseudomonadaceae	Pseudomonas_sp._AA27	DNA-sulfur-modification-protein-DndB-[Pseudomonas-sp.-AA27]	2908652
WP_234562663.1	DndB-ParB-nuclease	GCF_021462485.1	587	Bacteria>Proteobacteria>Gammaproteobacteria>Pseudomonadales>Pseudomonadaceae	Pseudomonas_sp._AA27	hypothetical-protein-[Pseudomonas-sp.-AA27]	2908652
KTC39031.1	DndB-ParB-nuclease	GCA_001467175.1	327	Bacteria>Proteobacteria>Gammaproteobacteria>Pseudomonadales>Pseudomonadaceae	Pseudomonas_sp._ABAC61	hypothetical-protein-AO265_14855-[Pseudomonas-sp.-ABAC61]	1230461
WP_223497056.1	DndB-ParB-nuclease	GCF_019947495.1	368	Bacteria>Proteobacteria>Gammaproteobacteria>Pseudomonadales>Pseudomonadaceae	Pseudomonas_sp._A-RE-26	DGQHR-domain-containing-protein-[Pseudomonas-sp.-A-RE-26]	2832402
WP_223497048.1	DndB-ParB-nuclease	GCF_019947495.1	365	Bacteria>Proteobacteria>Gammaproteobacteria>Pseudomonadales>Pseudomonadaceae	Pseudomonas_sp._A-RE-26	DNA-sulfur-modification-protein-DndB-[Pseudomonas-sp.-A-RE-26]	2832402
WP_223497061.1	DndB-ParB-nuclease	GCF_019947495.1	651	Bacteria>Proteobacteria>Gammaproteobacteria>Pseudomonadales>Pseudomonadaceae	Pseudomonas_sp._A-RE-26	hypothetical-protein-[Pseudomonas-sp.-A-RE-26]	2832402
WP_212614737.1	DndB-ParB-nuclease	GCF_018135665.1	362	Bacteria>Proteobacteria>Gammaproteobacteria>Pseudomonadales>Pseudomonadaceae	Pseudomonas_sp._B2021	DNA-sulfur-modification-protein-DndB-[Pseudomonas-sp.-B2021]	2546445
WP_128576160.1	DndB-ParB-nuclease	-	373	Bacteria>Proteobacteria>Gammaproteobacteria>Pseudomonadales>Pseudomonadaceae	Pseudomonas_sp._BAY1663	DNA-sulfur-modification-protein-DndB-[Pseudomonas-sp.-BAY1663]	1439940
WP_128576148.1	DndB-ParB-nuclease	GCF_000582595.1	657	Bacteria>Proteobacteria>Gammaproteobacteria>Pseudomonadales>Pseudomonadaceae	Pseudomonas_sp._BAY1663	hypothetical-protein-[Pseudomonas-sp.-BAY1663]	1439940
WP_223508649.1	DndB-ParB-nuclease	GCF_019947295.1	360	Bacteria>Proteobacteria>Gammaproteobacteria>Pseudomonadales>Pseudomonadaceae	Pseudomonas_sp._BF-RE-29	DNA-sulfur-modification-protein-DndB-[Pseudomonas-sp.-BF-RE-29]	2832378
WP_223508596.1	DndB-ParB-nuclease	GCF_019947295.1	691	Bacteria>Proteobacteria>Gammaproteobacteria>Pseudomonadales>Pseudomonadaceae	Pseudomonas_sp._BF-RE-29	hypothetical-protein-[Pseudomonas-sp.-BF-RE-29]	2832378
WP_177040236.1	DndB-ParB-nuclease	GCF_013385805.1	362	Bacteria>Proteobacteria>Gammaproteobacteria>Pseudomonadales>Pseudomonadaceae	Pseudomonas_sp._C6002	DNA-sulfur-modification-protein-DndB-[Pseudomonas-sp.-C6002]	2738814
WP_223505745.1	DndB-ParB-nuclease	GCF_019947275.1	365	Bacteria>Proteobacteria>Gammaproteobacteria>Pseudomonadales>Pseudomonadaceae	Pseudomonas_sp._GL-RE-29	DNA-sulfur-modification-protein-DndB-[Pseudomonas-sp.-GL-RE-29]	2832375
WP_223505743.1	DndB-ParB-nuclease	GCF_019947275.1	691	Bacteria>Proteobacteria>Gammaproteobacteria>Pseudomonadales>Pseudomonadaceae	Pseudomonas_sp._GL-RE-29	hypothetical-protein-[Pseudomonas-sp.-GL-RE-29]	2832375
EJM00840.1	DndB-ParB-nuclease	GCA_000282155.1	360	Bacteria>Proteobacteria>Gammaproteobacteria>Pseudomonadales>Pseudomonadaceae	Pseudomonas_sp._GM16	DNA-sulfur-modification-protein-DndB-[Pseudomonas-sp.-GM16]	1144322
EJM45670.1	DndB-ParB-nuclease	GCA_000282235.1	360	Bacteria>Proteobacteria>Gammaproteobacteria>Pseudomonadales>Pseudomonadaceae	Pseudomonas_sp._GM24	DNA-sulfur-modification-protein-DndB-[Pseudomonas-sp.-GM24]	1144326
WP_108574285.1	DndB-ParB-nuclease	GCF_003058245.1	405	Bacteria>Proteobacteria>Gammaproteobacteria>Pseudomonadales>Pseudomonadaceae	Pseudomonas_sp._GV047	DNA-phosphorothioation-associated-DGQHR-protein-1-[Pseudomonas-sp.-GV047]	2135751
WP_108574284.1	DndB-ParB-nuclease	GCF_003058245.1	361	Bacteria>Proteobacteria>Gammaproteobacteria>Pseudomonadales>Pseudomonadaceae	Pseudomonas_sp._GV047	DNA-sulfur-modification-protein-DndB-[Pseudomonas-sp.-GV047]	2135751
PTT12144.1	DndB-ParB-nuclease	GCA_003060935.1	360	Bacteria>Proteobacteria>Gammaproteobacteria>Pseudomonadales>Pseudomonadaceae	Pseudomonas_sp._HMWF034	DNA-sulfur-modification-protein-DndB-[Pseudomonas-sp.-HMWF034]	2056867
WP_088422655.1	DndB-ParB-nuclease	GCF_002204795.1	361	Bacteria>Proteobacteria>Gammaproteobacteria>Pseudomonadales>Pseudomonadaceae	Pseudomonas_sp._K2I15	DNA-sulfur-modification-protein-DndB-[Pseudomonas-sp.-K2I15]	2013577
WP_159894346.1	DndB-ParB-nuclease	GCF_009861525.1	359	Bacteria>Proteobacteria>Gammaproteobacteria>Pseudomonadales>Pseudomonadaceae	Pseudomonas_sp._LD120	DNA-sulfur-modification-protein-DndB-[Pseudomonas-sp.-LD120]	485751
WP_159894342.1	DndB-ParB-nuclease	GCF_009861525.1	665	Bacteria>Proteobacteria>Gammaproteobacteria>Pseudomonadales>Pseudomonadaceae	Pseudomonas_sp._LD120	hypothetical-protein-[Pseudomonas-sp.-LD120]	485751
WP_056785538.1	DndB-ParB-nuclease	GCF_001425545.1	360	Bacteria>Proteobacteria>Gammaproteobacteria>Pseudomonadales>Pseudomonadaceae	Pseudomonas_sp._Leaf434	DNA-sulfur-modification-protein-DndB-[Pseudomonas-sp.-Leaf434]	1736376
WP_104910336.1	DndB-ParB-nuclease	GCF_002949995.1	359	Bacteria>Proteobacteria>Gammaproteobacteria>Pseudomonadales>Pseudomonadaceae	Pseudomonas_sp._LG1D9	DNA-sulfur-modification-protein-DndB-[Pseudomonas-sp.-LG1D9]	2083054
WP_187679976.1	DndB-ParB-nuclease	GCF_014472415.1	362	Bacteria>Proteobacteria>Gammaproteobacteria>Pseudomonadales>Pseudomonadaceae	Pseudomonas_sp._N40(2020)	DNA-sulfur-modification-protein-DndB-[Pseudomonas-sp.-N40(2020)]	2767798
WP_092254268.1	DndB-ParB-nuclease	GCF_900104425.1	398	Bacteria>Proteobacteria>Gammaproteobacteria>Pseudomonadales>Pseudomonadaceae	Pseudomonas_sp._NFACC13-1	DGQHR-domain-containing-protein-[Pseudomonas-sp.-NFACC13-1]	1566245
WP_092254250.1	DndB-ParB-nuclease	GCF_900104425.1	360	Bacteria>Proteobacteria>Gammaproteobacteria>Pseudomonadales>Pseudomonadaceae	Pseudomonas_sp._NFACC13-1	DNA-sulfur-modification-protein-DndB-[Pseudomonas-sp.-NFACC13-1]	1566245
WP_226033868.1	DndB-ParB-nuclease	GCF_020405165.1	382	Bacteria>Proteobacteria>Gammaproteobacteria>Pseudomonadales>Pseudomonadaceae	Pseudomonas_sp._PS1(2021)	DGQHR-domain-containing-protein-[Pseudomonas-sp.-PS1(2021)]	2866282
WP_226033872.1	DndB-ParB-nuclease	GCF_020405165.1	360	Bacteria>Proteobacteria>Gammaproteobacteria>Pseudomonadales>Pseudomonadaceae	Pseudomonas_sp._PS1(2021)	DNA-sulfur-modification-protein-DndB-[Pseudomonas-sp.-PS1(2021)]	2866282
WP_226033874.1	DndB-ParB-nuclease	GCF_020405165.1	631	Bacteria>Proteobacteria>Gammaproteobacteria>Pseudomonadales>Pseudomonadaceae	Pseudomonas_sp._PS1(2021)	hypothetical-protein-[Pseudomonas-sp.-PS1(2021)]	2866282
WP_145165856.1	DndB-ParB-nuclease	GCF_007713445.1	385	Bacteria>Proteobacteria>Gammaproteobacteria>Pseudomonadales>Pseudomonadaceae	Pseudomonas_sp._RGB	DGQHR-domain-containing-protein-[Pseudomonas-sp.-RGB]	2598474
WP_145165863.1	DndB-ParB-nuclease	GCF_007713445.1	360	Bacteria>Proteobacteria>Gammaproteobacteria>Pseudomonadales>Pseudomonadaceae	Pseudomonas_sp._RGB	DNA-sulfur-modification-protein-DndB-[Pseudomonas-sp.-RGB]	2598474
RAU42143.1	DndB-ParB-nuclease	GCA_003057735.2	359	Bacteria>Proteobacteria>Gammaproteobacteria>Pseudomonadales>Pseudomonadaceae	Pseudomonas_sp._RIT_411	DNA-sulfur-modification-protein-DndB-[Pseudomonas-sp.-RIT-411]	2202160
WP_108581435.1	DndB-ParB-nuclease	-	359	Bacteria>Proteobacteria>Gammaproteobacteria>Pseudomonadales>Pseudomonadaceae	Pseudomonas_sp._RIT_411	DNA-sulfur-modification-protein-DndB-[Pseudomonas-sp.-RIT-411]	2202160
QBQ10269.1	DndB-ParB-nuclease	GCA_004379315.1	362	Bacteria>Proteobacteria>Gammaproteobacteria>Pseudomonadales>Pseudomonadaceae	Pseudomonas_sp._SXM-1	DNA-sulfur-modification-protein-DndB-[Pseudomonas-sp.-SXM-1]	2169583
WP_133076164.1	DndB-ParB-nuclease	GCF_004379315.1	362	Bacteria>Proteobacteria>Gammaproteobacteria>Pseudomonadales>Pseudomonadaceae	Pseudomonas_sp._SXM-1	DNA-sulfur-modification-protein-DndB-[Pseudomonas-sp.-SXM-1]	2169583
PJH86288.1	DndB-ParB-nuclease	GCA_002796855.1	399	Bacteria>Proteobacteria>Gammaproteobacteria>Pseudomonadales>Pseudomonadaceae	Pseudomonas_sp._WCS365	DNA-phosphorothioation-associated-DGQHR-protein-1-[Pseudomonas-sp.-WCS365]	2054929
PJH86268.1	DndB-ParB-nuclease	GCA_002796855.1	362	Bacteria>Proteobacteria>Gammaproteobacteria>Pseudomonadales>Pseudomonadaceae	Pseudomonas_sp._WCS365	DNA-sulfur-modification-protein-DndB-[Pseudomonas-sp.-WCS365]	2054929
AUZ58462.1	DndB-ParB-nuclease	GCA_002953115.1	333	Bacteria>Proteobacteria>Gammaproteobacteria>Pseudomonadales>Pseudomonadaceae	Pseudomonas_sp._XWY-1	DNA-sulfur-modification-protein-DndB-[Pseudomonas-sp.-XWY-1]	2069256
WP_104961170.1	DndB-ParB-nuclease	GCF_002953115.1	359	Bacteria>Proteobacteria>Gammaproteobacteria>Pseudomonadales>Pseudomonadaceae	Pseudomonas_sp._XWY-1	DNA-sulfur-modification-protein-DndB-[Pseudomonas-sp.-XWY-1]	2069256
AUZ58464.1	DndB-ParB-nuclease	GCA_002953115.1	665	Bacteria>Proteobacteria>Gammaproteobacteria>Pseudomonadales>Pseudomonadaceae	Pseudomonas_sp._XWY-1	hypothetical-protein-PRJ_1858-[Pseudomonas-sp.-XWY-1]	2069256
WP_104961172.1	DndB-ParB-nuclease	GCF_002953115.1	665	Bacteria>Proteobacteria>Gammaproteobacteria>Pseudomonadales>Pseudomonadaceae	Pseudomonas_sp._XWY-1	hypothetical-protein-[Pseudomonas-sp.-XWY-1]	2069256
WP_225569944.1	DndB-ParB-nuclease	GCF_020295325.1	499	Bacteria>Proteobacteria>Gammaproteobacteria>Pseudomonadales>Pseudomonadaceae	Pseudomonas_sp._Y24-6	DGQHR-domain-containing-protein-[Pseudomonas-sp.-Y24-6]	2750013
WP_225569949.1	DndB-ParB-nuclease	GCF_020295325.1	360	Bacteria>Proteobacteria>Gammaproteobacteria>Pseudomonadales>Pseudomonadaceae	Pseudomonas_sp._Y24-6	DNA-sulfur-modification-protein-DndB-[Pseudomonas-sp.-Y24-6]	2750013
WP_225569951.1	DndB-ParB-nuclease	GCF_020295325.1	666	Bacteria>Proteobacteria>Gammaproteobacteria>Pseudomonadales>Pseudomonadaceae	Pseudomonas_sp._Y24-6	hypothetical-protein-[Pseudomonas-sp.-Y24-6]	2750013
MBS7463061.1	DndB-ParB-nuclease	GCA_018383485.1	354	Bacteria>Proteobacteria>Gammaproteobacteria>Pseudomonadales>Pseudomonadaceae	Pseudomonas_syringae	DGQHR-domain-containing-protein-[Pseudomonas-syringae]	317
WP_074955467.1	DndB-ParB-nuclease	GCF_900113535.1	398	Bacteria>Proteobacteria>Gammaproteobacteria>Pseudomonadales>Pseudomonadaceae	Pseudomonas_syringae	DGQHR-domain-containing-protein-[Pseudomonas-syringae]	317
WP_213624845.1	DndB-ParB-nuclease	-	354	Bacteria>Proteobacteria>Gammaproteobacteria>Pseudomonadales>Pseudomonadaceae	Pseudomonas_syringae	DGQHR-domain-containing-protein-[Pseudomonas-syringae]	317
MBS7463069.1	DndB-ParB-nuclease	GCA_018383485.1	360	Bacteria>Proteobacteria>Gammaproteobacteria>Pseudomonadales>Pseudomonadaceae	Pseudomonas_syringae	DNA-sulfur-modification-protein-DndB-[Pseudomonas-syringae]	317
PBP51608.1	DndB-ParB-nuclease	GCA_002318665.1	360	Bacteria>Proteobacteria>Gammaproteobacteria>Pseudomonadales>Pseudomonadaceae	Pseudomonas_syringae	DNA-sulfur-modification-protein-DndB-[Pseudomonas-syringae]	317
WP_058823904.1	DndB-ParB-nuclease	GCF_001466965.1	361	Bacteria>Proteobacteria>Gammaproteobacteria>Pseudomonadales>Pseudomonadaceae	Pseudomonas_syringae	DNA-sulfur-modification-protein-DndB-[Pseudomonas-syringae]	317
WP_074955488.1	DndB-ParB-nuclease	GCF_900113535.1	360	Bacteria>Proteobacteria>Gammaproteobacteria>Pseudomonadales>Pseudomonadaceae	Pseudomonas_syringae	DNA-sulfur-modification-protein-DndB-[Pseudomonas-syringae]	317
WP_096103676.1	DndB-ParB-nuclease	GCF_002318665.1	360	Bacteria>Proteobacteria>Gammaproteobacteria>Pseudomonadales>Pseudomonadaceae	Pseudomonas_syringae	DNA-sulfur-modification-protein-DndB-[Pseudomonas-syringae]	317
WP_198703567.1	DndB-ParB-nuclease	GCF_016308215.1	362	Bacteria>Proteobacteria>Gammaproteobacteria>Pseudomonadales>Pseudomonadaceae	Pseudomonas_syringae	DNA-sulfur-modification-protein-DndB-[Pseudomonas-syringae]	317
WP_029242070.1	DndB-ParB-nuclease	GCF_000452485.1	360	Bacteria>Proteobacteria>Gammaproteobacteria>Pseudomonadales>Pseudomonadaceae	Pseudomonas_syringae_group	MULTISPECIES:-DNA-sulfur-modification-protein-DndB-[Pseudomonas-syringae-group]	136849
MBS7463070.1	DndB-ParB-nuclease	GCA_018383485.1	682	Bacteria>Proteobacteria>Gammaproteobacteria>Pseudomonadales>Pseudomonadaceae	Pseudomonas_syringae	hypothetical-protein-[Pseudomonas-syringae]	317
WP_074955490.1	DndB-ParB-nuclease	GCF_900113535.1	682	Bacteria>Proteobacteria>Gammaproteobacteria>Pseudomonadales>Pseudomonadaceae	Pseudomonas_syringae	hypothetical-protein-[Pseudomonas-syringae]	317
WP_058431209.1	DndB-ParB-nuclease	GCF_900580495.1	379	Bacteria>Proteobacteria>Gammaproteobacteria>Pseudomonadales>Pseudomonadaceae	Pseudomonas_viridiflava	DGQHR-domain-containing-protein-[Pseudomonas-viridiflava]	33069
WP_122555860.1	DndB-ParB-nuclease	GCF_900580945.1	482	Bacteria>Proteobacteria>Gammaproteobacteria>Pseudomonadales>Pseudomonadaceae	Pseudomonas_viridiflava	DGQHR-domain-containing-protein-[Pseudomonas-viridiflava]	33069

Accession	Annotation	GCA	Length	Phyletic.spread	Strain.name	Defline	Species.Taxid
WP_122557486.1	DndB-ParB-nuclease	GCF_900576665.1	394	Bacteria>Proteobacteria>Gammaproteobacteria>Pseudomonadales>Pseudomonadaceae	Pseudomonas_viridiflava	DGQHR-domain-containing-protein-[Pseudomonas-viridiflava]	33069
WP_122557492.1	DndB-ParB-nuclease	GCF_900576665.1	379	Bacteria>Proteobacteria>Gammaproteobacteria>Pseudomonadales>Pseudomonadaceae	Pseudomonas_viridiflava	DGQHR-domain-containing-protein-[Pseudomonas-viridiflava]	33069
WP_122679005.1	DndB-ParB-nuclease	GCF_900598765.1	363	Bacteria>Proteobacteria>Gammaproteobacteria>Pseudomonadales>Pseudomonadaceae	Pseudomonas_viridiflava	DGQHR-domain-containing-protein-[Pseudomonas-viridiflava]	33069
WP_205870349.1	DndB-ParB-nuclease	GCF_900580495.1	346	Bacteria>Proteobacteria>Gammaproteobacteria>Pseudomonadales>Pseudomonadaceae	Pseudomonas_viridiflava	DGQHR-domain-containing-protein-[Pseudomonas-viridiflava]	33069
WP_235160754.1	DndB-ParB-nuclease	GCF_000452485.1	403	Bacteria>Proteobacteria>Gammaproteobacteria>Pseudomonadales>Pseudomonadaceae	Pseudomonas_viridiflava	DGQHR-domain-containing-protein-[Pseudomonas-viridiflava]	33069
WP_122764102.1	DndB-ParB-nuclease	GCF_900589385.1	405	Bacteria>Proteobacteria>Gammaproteobacteria>Pseudomonadales>Pseudomonadaceae	Pseudomonas_viridiflava	DNA-phosphorothioation-associated-DGQHR-protein-1-[Pseudomonas-viridiflava]	33069
WP_058431215.1	DndB-ParB-nuclease	GCF_900580495.1	360	Bacteria>Proteobacteria>Gammaproteobacteria>Pseudomonadales>Pseudomonadaceae	Pseudomonas_viridiflava	DNA-sulfur-modification-protein-DndB-[Pseudomonas-viridiflava]	33069
WP_122426637.1	DndB-ParB-nuclease	GCF_900580655.1	360	Bacteria>Proteobacteria>Gammaproteobacteria>Pseudomonadales>Pseudomonadaceae	Pseudomonas_viridiflava	DNA-sulfur-modification-protein-DndB-[Pseudomonas-viridiflava]	33069
WP_122557482.1	DndB-ParB-nuclease	GCF_900576665.1	360	Bacteria>Proteobacteria>Gammaproteobacteria>Pseudomonadales>Pseudomonadaceae	Pseudomonas_viridiflava	DNA-sulfur-modification-protein-DndB-[Pseudomonas-viridiflava]	33069
WP_122605672.1	DndB-ParB-nuclease	GCF_900580885.1	359	Bacteria>Proteobacteria>Gammaproteobacteria>Pseudomonadales>Pseudomonadaceae	Pseudomonas_viridiflava	DNA-sulfur-modification-protein-DndB-[Pseudomonas-viridiflava]	33069
WP_122679009.1	DndB-ParB-nuclease	GCF_900602085.1	369	Bacteria>Proteobacteria>Gammaproteobacteria>Pseudomonadales>Pseudomonadaceae	Pseudomonas_viridiflava	DNA-sulfur-modification-protein-DndB-[Pseudomonas-viridiflava]	33069
WP_122764101.1	DndB-ParB-nuclease	-	361	Bacteria>Proteobacteria>Gammaproteobacteria>Pseudomonadales>Pseudomonadaceae	Pseudomonas_viridiflava	DNA-sulfur-modification-protein-DndB-[Pseudomonas-viridiflava]	33069
WP_198695797.1	DndB-ParB-nuclease	GCF_016308365.1	361	Bacteria>Proteobacteria>Gammaproteobacteria>Pseudomonadales>Pseudomonadaceae	Pseudomonas_viridiflava	DNA-sulfur-modification-protein-DndB-[Pseudomonas-viridiflava]	33069
WP_029242072.1	DndB-ParB-nuclease	GCF_000452485.1	708	Bacteria>Proteobacteria>Gammaproteobacteria>Pseudomonadales>Pseudomonadaceae	Pseudomonas_viridiflava	hypothetical-protein-[Pseudomonas-viridiflava]	33069
WP_122555865.1	DndB-ParB-nuclease	GCF_900580945.1	665	Bacteria>Proteobacteria>Gammaproteobacteria>Pseudomonadales>Pseudomonadaceae	Pseudomonas_viridiflava	hypothetical-protein-[Pseudomonas-viridiflava]	33069
WP_122557481.1	DndB-ParB-nuclease	GCF_900576665.1	708	Bacteria>Proteobacteria>Gammaproteobacteria>Pseudomonadales>Pseudomonadaceae	Pseudomonas_viridiflava	hypothetical-protein-[Pseudomonas-viridiflava]	33069
WP_198695795.1	DndB-ParB-nuclease	GCF_016308365.1	666	Bacteria>Proteobacteria>Gammaproteobacteria>Pseudomonadales>Pseudomonadaceae	Pseudomonas_viridiflava	hypothetical-protein-[Pseudomonas-viridiflava]	33069
WP_186623146.1	DndB-ParB-nuclease	GCF_014268485.2	398	Bacteria>Proteobacteria>Gammaproteobacteria>Pseudomonadales>Pseudomonadaceae	Pseudomonas_zeae	DGQHR-domain-containing-protein-[Pseudomonas-zeae]	2745510
WP_186622424.1	DndB-ParB-nuclease	GCF_014268485.2	360	Bacteria>Proteobacteria>Gammaproteobacteria>Pseudomonadales>Pseudomonadaceae	Pseudomonas_zeae	DNA-sulfur-modification-protein-DndB-[Pseudomonas-zeae]	2745510
WP_131187635.1	DndB-ParB-nuclease	GCF_004327305.1	360	Bacteria>Proteobacteria>Gammaproteobacteria>Pseudomonadales>Pseudomonadaceae	Stutzerimonas_kirkiae	DNA-sulfur-modification-protein-DndB-[Pseudomonas-kirkiae]	2211392
WP_131187637.1	DndB-ParB-nuclease	GCF_004327305.1	706	Bacteria>Proteobacteria>Gammaproteobacteria>Pseudomonadales>Pseudomonadaceae	Stutzerimonas_kirkiae	hypothetical-protein-[Pseudomonas-kirkiae]	2211392
NNJ37370.1	DndB-ParB-nuclease	GCA_013036985.1	359	Bacteria>Proteobacteria>Gammaproteobacteria>Pseudomonadales>Pseudomonadaceae	Stutzerimonas_stutzeri	DNA-sulfur-modification-protein-DndB-[Pseudomonas-stutzeri]	316
WP_080558867.1	DndB-ParB-nuclease	GCF_000195105.1	359	Bacteria>Proteobacteria>Gammaproteobacteria>Pseudomonadales>Pseudomonadaceae	Stutzerimonas_stutzeri	DNA-sulfur-modification-protein-DndB-[Pseudomonas-stutzeri]	316
WP_080902101.1	DndB-ParB-nuclease	GCF_000935215.1	363	Bacteria>Proteobacteria>Gammaproteobacteria>Pseudomonadales>Pseudomonadaceae	Stutzerimonas_stutzeri	DNA-sulfur-modification-protein-DndB-[Pseudomonas-stutzeri]	316
WP_170801270.1	DndB-ParB-nuclease	GCF_013036985.1	359	Bacteria>Proteobacteria>Gammaproteobacteria>Pseudomonadales>Pseudomonadaceae	Stutzerimonas_stutzeri	DNA-sulfur-modification-protein-DndB-[Pseudomonas-stutzeri]	316
WP_181077337.1	DndB-ParB-nuclease	GCF_013522915.1	363	Bacteria>Proteobacteria>Gammaproteobacteria>Pseudomonadales>Pseudomonadaceae	Stutzerimonas_stutzeri	DNA-sulfur-modification-protein-DndB-[Pseudomonas-stutzeri]	316
WP_212619819.1	DndB-ParB-nuclease	GCF_018138085.1	363	Bacteria>Proteobacteria>Gammaproteobacteria>Pseudomonadales>Pseudomonadaceae	Stutzerimonas_stutzeri	DNA-sulfur-modification-protein-DndB-[Pseudomonas-stutzeri]	316
WP_226687149.1	DndB-ParB-nuclease	GCF_020524725.1	359	Bacteria>Proteobacteria>Gammaproteobacteria>Pseudomonadales>Pseudomonadaceae	Stutzerimonas_stutzeri	DNA-sulfur-modification-protein-DndB-[Pseudomonas-stutzeri]	316
WP_014596865.1	DndB-ParB-nuclease	GCF_000195105.1	681	Bacteria>Proteobacteria>Gammaproteobacteria>Pseudomonadales>Pseudomonadaceae	Stutzerimonas_stutzeri	hypothetical-protein-[Pseudomonas-stutzeri]	316
WP_044315060.1	DndB-ParB-nuclease	GCF_000935215.1	657	Bacteria>Proteobacteria>Gammaproteobacteria>Pseudomonadales>Pseudomonadaceae	Stutzerimonas_stutzeri	hypothetical-protein-[Pseudomonas-stutzeri]	316
WP_181077348.1	DndB-ParB-nuclease	GCF_013522915.1	485	Bacteria>Proteobacteria>Gammaproteobacteria>Pseudomonadales>Pseudomonadaceae	Stutzerimonas_stutzeri	hypothetical-protein-[Pseudomonas-stutzeri]	316
WP_226687147.1	DndB-ParB-nuclease	GCF_020524725.1	680	Bacteria>Proteobacteria>Gammaproteobacteria>Pseudomonadales>Pseudomonadaceae	Stutzerimonas_stutzeri	hypothetical-protein-[Pseudomonas-stutzeri]	316
WP_103324383.1	DndB-ParB-nuclease	GCF_017350515.1	363	Bacteria>Proteobacteria>Gammaproteobacteria>Pseudomonadales>Pseudomonadaceae	unclassified_Pseudomonas	MULTISPECIES:-DGQHR-domain-containing-protein-[unclassified-Pseudomonas]	196821
WP_124411229.1	DndB-ParB-nuclease	-	400	Bacteria>Proteobacteria>Gammaproteobacteria>Pseudomonadales>Pseudomonadaceae	unclassified_Pseudomonas	MULTISPECIES:-DGQHR-domain-containing-protein-[unclassified-Pseudomonas]	196821
WP_007915173.1	DndB-ParB-nuclease	GCF_000282235.1	360	Bacteria>Proteobacteria>Gammaproteobacteria>Pseudomonadales>Pseudomonadaceae	unclassified_Pseudomonas	MULTISPECIES:-DNA-sulfur-modification-protein-DndB-[unclassified-Pseudomonas]	196821
WP_064594905.1	DndB-ParB-nuclease	GCF_946901175.1	359	Bacteria>Proteobacteria>Gammaproteobacteria>Pseudomonadales>Pseudomonadaceae	unclassified_Pseudomonas	MULTISPECIES:-DNA-sulfur-modification-protein-DndB-[unclassified-Pseudomonas]	196821
WP_103324387.1	DndB-ParB-nuclease	GCF_017350515.1	366	Bacteria>Proteobacteria>Gammaproteobacteria>Pseudomonadales>Pseudomonadaceae	unclassified_Pseudomonas	MULTISPECIES:-DNA-sulfur-modification-protein-DndB-[unclassified-Pseudomonas]	196821
WP_124410806.1	DndB-ParB-nuclease	GCF_003852235.1	360	Bacteria>Proteobacteria>Gammaproteobacteria>Pseudomonadales>Pseudomonadaceae	unclassified_Pseudomonas	MULTISPECIES:-DNA-sulfur-modification-protein-DndB-[unclassified-Pseudomonas]	196821
MBL4680888.1	DndB-ParB-nuclease	GCA_016763855.1	682	Bacteria>Proteobacteria>Gammaproteobacteria>Pseudomonadales>unclassified_Pseudomonadales	Pseudomonadales_bacterium	hypothetical-protein-[Pseudomonadales-bacterium]	1891229
OHC30138.1	DndB-ParB-nuclease	GCA_001828745.1	360	Bacteria>Proteobacteria>Gammaproteobacteria>Pseudomonadales>unclassified_Pseudomonadales	Pseudomonadales_bacterium_RIFCSPHIGHO2_02_FULL_60_43	DNA-sulfur-modification-protein-DndB-[Pseudomonadales-bacterium-RIFCSPHIGHO2_02_FULL_60_43]	1802005
OHC30134.1	DndB-ParB-nuclease	GCA_001828745.1	399	Bacteria>Proteobacteria>Gammaproteobacteria>Pseudomonadales>unclassified_Pseudomonadales	Pseudomonadales_bacterium_RIFCSPHIGHO2_02_FULL_60_43	hypothetical-protein-A3J71_01720-[Pseudomonadales-bacterium-RIFCSPHIGHO2_02_FULL_60_43]	1802005
PHS71159.1	DndB-ParB-nuclease	GCA_002733135.1	360	Bacteria>Proteobacteria>Gammaproteobacteria>Thiotrichales>Piscirickettsiaceae	Cycloclasticus_sp.	DNA-sulfur-modification-protein-DndB-[Cycloclasticus-sp.]	2024830
PHS73531.1	DndB-ParB-nuclease	GCA_002733135.1	241	Bacteria>Proteobacteria>Gammaproteobacteria>Thiotrichales>Piscirickettsiaceae	Cycloclasticus_sp.	hypothetical-protein-COB22_02225-[Cycloclasticus-sp.]	2024830
MCB2426047.1	DndB-ParB-nuclease	GCA_020447225.1	411	Bacteria>Proteobacteria>Gammaproteobacteria>Thiotrichales>Piscirickettsiaceae	Methylophaga_pinxianii	DGQHR-domain-containing-protein-[Methylophaga-pinxianii]	2881052
MCB2426042.1	DndB-ParB-nuclease	GCA_020447225.1	356	Bacteria>Proteobacteria>Gammaproteobacteria>Thiotrichales>Piscirickettsiaceae	Methylophaga_pinxianii	DNA-sulfur-modification-protein-DndB-[Methylophaga-pinxianii]	2881052
WP_226069787.1	DndB-ParB-nuclease	GCF_020447175.2	411	Bacteria>Proteobacteria>Gammaproteobacteria>Thiotrichales>Piscirickettsiaceae	Methylophaga_pinxianii	MULTISPECIES:-DGQHR-domain-containing-protein-[unclassified-Methylophaga]	2881052
WP_226069792.1	DndB-ParB-nuclease	GCF_020447175.2	356	Bacteria>Proteobacteria>Gammaproteobacteria>Thiotrichales>Piscirickettsiaceae	Methylophaga_pinxianii	MULTISPECIES:-DNA-sulfur-modification-protein-DndB-[unclassified-Methylophaga]	2881052
WP_091714792.1	DndB-ParB-nuclease	GCF_900114205.1	343	Bacteria>Proteobacteria>Gammaproteobacteria>Thiotrichales>Piscirickettsiaceae	Methylophaga_sulfidovorans	DGQHR-domain-containing-protein-[Methylophaga-sulfidovorans]	45496
WP_091714780.1	DndB-ParB-nuclease	GCF_900114205.1	356	Bacteria>Proteobacteria>Gammaproteobacteria>Thiotrichales>Piscirickettsiaceae	Methylophaga_sulfidovorans	DNA-sulfur-modification-protein-DndB-[Methylophaga-sulfidovorans]	45496
WP_165395081.1	DndB-ParB-nuclease	GCF_004293625.1	424	Bacteria>Proteobacteria>Gammaproteobacteria>Thiotrichales>Piscirickettsiaceae	Thiomicrorhabdus_indica	DGQHR-domain-containing-protein-[Thiomicrorhabdus-indica]	2267253
WP_130537860.1	DndB-ParB-nuclease	GCF_004293625.1	358	Bacteria>Proteobacteria>Gammaproteobacteria>Thiotrichales>Piscirickettsiaceae	Thiomicrorhabdus_indica	DNA-sulfur-modification-protein-DndB-[Thiomicrorhabdus-indica]	2267253
MBJ6610746.1	DndB-ParB-nuclease	GCA_016292235.1	366	Bacteria>Proteobacteria>Gammaproteobacteria>Thiotrichales>Thiotrichaceae	Candidatus_Thiothrix_moscowensis	DNA-sulfur-modification-protein-DndB-[Candidatus-Thiothrix-moscowensis]	2799668
RVU83647.1	DndB-ParB-nuclease	GCA_004010795.1	362	Bacteria>Proteobacteria>Gammaproteobacteria>Thiotrichales>Thiotrichaceae	Leucothrix_sargassi	DNA-sulfur-modification-protein-DndB-[Leucothrix-sargassi]	2500160
RVU83642.1	DndB-ParB-nuclease	GCA_004010795.1	538	Bacteria>Proteobacteria>Gammaproteobacteria>Thiotrichales>Thiotrichaceae	Leucothrix_sargassi	hypothetical-protein-EOL70_15050-[Leucothrix-sargassi]	2500160
GFO73386.1	DndB-ParB-nuclease	GCA_019721995.1	361	Bacteria>Proteobacteria>Gammaproteobacteria>unclassified_Gammaproteobacteria	Bathymodiolus_japonicus_methanotrophic_gill_symbiont	DNA-sulfur-modification-protein-DndB-[Bathymodiolus-japonicus-methanotrophic-gill-symbiont]	113269
WP_221898134.1	DndB-ParB-nuclease	GCF_019721995.1	361	Bacteria>Proteobacteria>Gammaproteobacteria>unclassified_Gammaproteobacteria	Bathymodiolus_japonicus_methanotrophic_gill_symbiont	DNA-sulfur-modification-protein-DndB-[Bathymodiolus-japonicus-methanotrophic-gill-symbiont]	113269
HFD31326.1	DndB-ParB-nuclease	GCA_011322795.1	435	Bacteria>Proteobacteria>Gammaproteobacteria>unclassified_Gammaproteobacteria	Gammaproteobacteria_bacterium	DGQHR-domain-containing-protein-[Gammaproteobacteria-bacterium]	1913989
MCB1753245.1	DndB-ParB-nuclease	GCA_020445405.1	424	Bacteria>Proteobacteria>Gammaproteobacteria>unclassified_Gammaproteobacteria	Gammaproteobacteria_bacterium	DGQHR-domain-containing-protein-[Gammaproteobacteria-bacterium]	1913989
MYG96060.1	DndB-ParB-nuclease	GCA_009842725.1	443	Bacteria>Proteobacteria>Gammaproteobacteria>unclassified_Gammaproteobacteria	Gammaproteobacteria_bacterium	DGQHR-domain-containing-protein-[Gammaproteobacteria-bacterium]	1913989
NPU90389.1	DndB-ParB-nuclease	GCA_013178375.1	394	Bacteria>Proteobacteria>Gammaproteobacteria>unclassified_Gammaproteobacteria	Gammaproteobacteria_bacterium	DGQHR-domain-containing-protein-[Gammaproteobacteria-bacterium]	1913989
HFD31322.1	DndB-ParB-nuclease	GCA_011322795.1	371	Bacteria>Proteobacteria>Gammaproteobacteria>unclassified_Gammaproteobacteria	Gammaproteobacteria_bacterium	DNA-sulfur-modification-protein-DndB-[Gammaproteobacteria-bacterium]	1913989
MAY03702.1	DndB-ParB-nuclease	GCA_002710315.1	359	Bacteria>Proteobacteria>Gammaproteobacteria>unclassified_Gammaproteobacteria	Gammaproteobacteria_bacterium	DNA-sulfur-modification-protein-DndB-[Gammaproteobacteria-bacterium]	1913989
MBM4229711.1	DndB-ParB-nuclease	GCA_016874565.1	362	Bacteria>Proteobacteria>Gammaproteobacteria>unclassified_Gammaproteobacteria	Gammaproteobacteria_bacterium	DNA-sulfur-modification-protein-DndB-[Gammaproteobacteria-bacterium]	1913989
MBU1215699.1	DndB-ParB-nuclease	GCA_018819205.1	365	Bacteria>Proteobacteria>Gammaproteobacteria>unclassified_Gammaproteobacteria	Gammaproteobacteria_bacterium	DNA-sulfur-modification-protein-DndB-[Gammaproteobacteria-bacterium]	1913989
MCF6257082.1	DndB-ParB-nuclease	GCA_021647705.1	361	Bacteria>Proteobacteria>Gammaproteobacteria>unclassified_Gammaproteobacteria	Gammaproteobacteria_bacterium	DNA-sulfur-modification-protein-DndB-[Gammaproteobacteria-bacterium]	1913989
MXZ45327.1	DndB-ParB-nuclease	GCA_009837465.1	362	Bacteria>Proteobacteria>Gammaproteobacteria>unclassified_Gammaproteobacteria	Gammaproteobacteria_bacterium	DNA-sulfur-modification-protein-DndB-[Gammaproteobacteria-bacterium]	1913989
MYE25508.1	DndB-ParB-nuclease	GCA_009839405.1	363	Bacteria>Proteobacteria>Gammaproteobacteria>unclassified_Gammaproteobacteria	Gammaproteobacteria_bacterium	DNA-sulfur-modification-protein-DndB-[Gammaproteobacteria-bacterium]	1913989
MYF51351.1	DndB-ParB-nuclease	GCA_009842155.1	363	Bacteria>Proteobacteria>Gammaproteobacteria>unclassified_Gammaproteobacteria	Gammaproteobacteria_bacterium	DNA-sulfur-modification-protein-DndB-[Gammaproteobacteria-bacterium]	1913989
NOY72393.1	DndB-ParB-nuclease	GCA_013151745.1	361	Bacteria>Proteobacteria>Gammaproteobacteria>unclassified_Gammaproteobacteria	Gammaproteobacteria_bacterium	DNA-sulfur-modification-protein-DndB-[Gammaproteobacteria-bacterium]	1913989
NPU90393.1	DndB-ParB-nuclease	GCA_013178375.1	365	Bacteria>Proteobacteria>Gammaproteobacteria>unclassified_Gammaproteobacteria	Gammaproteobacteria_bacterium	DNA-sulfur-modification-protein-DndB-[Gammaproteobacteria-bacterium]	1913989
RLA53319.1	DndB-ParB-nuclease	GCA_003646365.1	620	Bacteria>Proteobacteria>Gammaproteobacteria>unclassified_Gammaproteobacteria	Gammaproteobacteria_bacterium	hypothetical-protein-DRR42_05145-[Gammaproteobacteria-bacterium]	1913989
MAY03704.1	DndB-ParB-nuclease	GCA_002710315.1	684	Bacteria>Proteobacteria>Gammaproteobacteria>unclassified_Gammaproteobacteria	Gammaproteobacteria_bacterium	hypothetical-protein-[Gammaproteobacteria-bacterium]	1913989
EED31296.1	DndB-ParB-nuclease	GCA_000158155.1	364	Bacteria>Proteobacteria>Gammaproteobacteria>unclassified_Gammaproteobacteria	gamma_proteobacterium_NOR5-3	DNA-sulfur-modification-protein-DndB-[gamma-proteobacterium-NOR5-3]	566466
RBW65348.1	DndB-ParB-nuclease	GCA_003318175.1	359	Bacteria>Proteobacteria>Gammaproteobacteria>Vibrionales>unclassified_Vibrionales	Vibrionales_bacterium_C3R12	DNA-sulfur-modification-protein-DndB-[Vibrionales-bacterium-C3R12]	2267228
RBW65352.1	DndB-ParB-nuclease	GCA_003318175.1	412	Bacteria>Proteobacteria>Gammaproteobacteria>Vibrionales>unclassified_Vibrionales	Vibrionales_bacterium_C3R12	hypothetical-protein-DS893_09650-[Vibrionales-bacterium-C3R12]	2267228
WP_151653622.1	DndB-ParB-nuclease	GCF_008933155.1	359	Bacteria>Proteobacteria>Gammaproteobacteria>Vibrionales>Vibrionaceae	Aliivibrio_finisterrensis	DNA-sulfur-modification-protein-DndB-[Aliivibrio-finisterrensis]	511998
WP_065640572.1	DndB-ParB-nuclease	GCF_001690915.1	410	Bacteria>Proteobacteria>Gammaproteobacteria>Vibrionales>Vibrionaceae	Aliivibrio_fischeri	DGQHR-domain-containing-protein-[Aliivibrio-fischeri]	668
WP_155694225.1	DndB-ParB-nuclease	GCF_009727835.1	412	Bacteria>Proteobacteria>Gammaproteobacteria>Vibrionales>Vibrionaceae	Aliivibrio_fischeri	DGQHR-domain-containing-protein-[Aliivibrio-fischeri]	668
WP_086028465.1	DndB-ParB-nuclease	GCF_000020845.1	375	Bacteria>Proteobacteria>Gammaproteobacteria>Vibrionales>Vibrionaceae	Aliivibrio_fischeri	DNA-phosphorothioation-associated-DGQHR-protein-1-[Aliivibrio-fischeri]	668
MUK28680.1	DndB-ParB-nuclease	GCA_009727725.1	361	Bacteria>Proteobacteria>Gammaproteobacteria>Vibrionales>Vibrionaceae	Aliivibrio_fischeri	DNA-sulfur-modification-protein-DndB-[Aliivibrio-fischeri]	668
WP_012535250.1	DndB-ParB-nuclease	GCF_000020845.1	359	Bacteria>Proteobacteria>Gammaproteobacteria>Vibrionales>Vibrionaceae	Aliivibrio_fischeri	DNA-sulfur-modification-protein-DndB-[Aliivibrio-fischeri]	668
WP_063646198.1	DndB-ParB-nuclease	GCF_001640325.1	359	Bacteria>Proteobacteria>Gammaproteobacteria>Vibrionales>Vibrionaceae	Aliivibrio_fischeri	DNA-sulfur-modification-protein-DndB-[Aliivibrio-fischeri]	668
WP_063664055.1	DndB-ParB-nuclease	GCF_001640415.1	359	Bacteria>Proteobacteria>Gammaproteobacteria>Vibrionales>Vibrionaceae	Aliivibrio_fischeri	DNA-sulfur-modification-protein-DndB-[Aliivibrio-fischeri]	668
WP_065594764.1	DndB-ParB-nuclease	GCF_001691095.1	359	Bacteria>Proteobacteria>Gammaproteobacteria>Vibrionales>Vibrionaceae	Aliivibrio_fischeri	DNA-sulfur-modification-protein-DndB-[Aliivibrio-fischeri]	668
WP_065640568.1	DndB-ParB-nuclease	GCF_001690915.1	359	Bacteria>Proteobacteria>Gammaproteobacteria>Vibrionales>Vibrionaceae	Aliivibrio_fischeri	DNA-sulfur-modification-protein-DndB-[Aliivibrio-fischeri]	668
WP_069582520.1	DndB-ParB-nuclease	GCF_001730145.1	359	Bacteria>Proteobacteria>Gammaproteobacteria>Vibrionales>Vibrionaceae	Aliivibrio_fischeri	DNA-sulfur-modification-protein-DndB-[Aliivibrio-fischeri]	668
WP_146865014.1	DndB-ParB-nuclease	GCF_007989845.1	359	Bacteria>Proteobacteria>Gammaproteobacteria>Vibrionales>Vibrionaceae	Aliivibrio_fischeri	DNA-sulfur-modification-protein-DndB-[Aliivibrio-fischeri]	668
WP_155651850.1	DndB-ParB-nuclease	GCF_009727725.1	361	Bacteria>Proteobacteria>Gammaproteobacteria>Vibrionales>Vibrionaceae	Aliivibrio_fischeri	DNA-sulfur-modification-protein-DndB-[Aliivibrio-fischeri]	668
WP_155659759.1	DndB-ParB-nuclease	GCF_009728045.1	364	Bacteria>Proteobacteria>Gammaproteobacteria>Vibrionales>Vibrionaceae	Aliivibrio_fischeri	DNA-sulfur-modification-protein-DndB-[Aliivibrio-fischeri]	668
WP_155694217.1	DndB-ParB-nuclease	GCF_009727835.1	359	Bacteria>Proteobacteria>Gammaproteobacteria>Vibrionales>Vibrionaceae	Aliivibrio_fischeri	DNA-sulfur-modification-protein-DndB-[Aliivibrio-fischeri]	668
WP_233941555.1	DndB-ParB-nuclease	GCF_021394875.1	694	Bacteria>Proteobacteria>Gammaproteobacteria>Vibrionales>Vibrionaceae	Aliivibrio_fischeri	hypothetical-protein-[Aliivibrio-fischeri]	668
ACH64120.1	DndB-ParB-nuclease	GCA_000020845.1	359	Bacteria>Proteobacteria>Gammaproteobacteria>Vibrionales>Vibrionaceae	Aliivibrio_fischeri_MJ11	DNA-sulfur-modification-protein-DndB-[Aliivibrio-fischeri-MJ11]	388396
ACH63886.1	DndB-ParB-nuclease	GCA_000020845.1	372	Bacteria>Proteobacteria>Gammaproteobacteria>Vibrionales>Vibrionaceae	Aliivibrio_fischeri_MJ11	putative-DGQHR-domain-protein-[Aliivibrio-fischeri-MJ11]	388396
WP_065600746.1	DndB-ParB-nuclease	GCF_001691065.1	364	Bacteria>Proteobacteria>Gammaproteobacteria>Vibrionales>Vibrionaceae	Aliivibrio_sp._1S128	DNA-sulfur-modification-protein-DndB-[Aliivibrio-sp.-1S128]	1840085
WP_192957947.1	DndB-ParB-nuclease	-	426	Bacteria>Proteobacteria>Gammaproteobacteria>Vibrionales>Vibrionaceae	Aliivibrio_wodanis	DGQHR-domain-containing-protein-[Aliivibrio-wodanis]	80852
WP_192957943.1	DndB-ParB-nuclease	-	359	Bacteria>Proteobacteria>Gammaproteobacteria>Vibrionales>Vibrionaceae	Aliivibrio_wodanis	DNA-sulfur-modification-protein-DndB-[Aliivibrio-wodanis]	80852
WP_102386159.1	DndB-ParB-nuclease	GCF_002873035.1	388	Bacteria>Proteobacteria>Gammaproteobacteria>Vibrionales>Vibrionaceae	Enterovibrio_norvegicus	DGQHR-domain-containing-protein-[Enterovibrio-norvegicus]	188144
WP_074927703.1	DndB-ParB-nuclease	GCF_900115495.1	361	Bacteria>Proteobacteria>Gammaproteobacteria>Vibrionales>Vibrionaceae	Enterovibrio_norvegicus	DNA-sulfur-modification-protein-DndB-[Enterovibrio-norvegicus]	188144
WP_102386155.1	DndB-ParB-nuclease	GCF_002873035.1	359	Bacteria>Proteobacteria>Gammaproteobacteria>Vibrionales>Vibrionaceae	Enterovibrio_norvegicus	DNA-sulfur-modification-protein-DndB-[Enterovibrio-norvegicus]	188144
WP_083417934.1	DndB-ParB-nuclease	GCF_900115495.1	556	Bacteria>Proteobacteria>Gammaproteobacteria>Vibrionales>Vibrionaceae	Enterovibrio_norvegicus	hypothetical-protein-[Enterovibrio-norvegicus]	188144
EEY72700.1	DndB-ParB-nuclease	GCA_000176515.1	358	Bacteria>Proteobacteria>Gammaproteobacteria>Vibrionales>Vibrionaceae	Grimontia_hollisae_CIP_101886	hypothetical-protein-VHA_001805-[Grimontia-hollisae-CIP-101886]	675812
WP_005503791.1	DndB-ParB-nuclease	GCF_000176515.1	358	Bacteria>Proteobacteria>Gammaproteobacteria>Vibrionales>Vibrionaceae	Grimontia_hollisae	DNA-sulfur-modification-protein-DndB-[Grimontia-hollisae]	673
WP_045149210.1	DndB-ParB-nuclease	GCF_000950005.1	568	Bacteria>Proteobacteria>Gammaproteobacteria>Vibrionales>Vibrionaceae	Photobacterium_angustum	AIPR-family-protein-[Photobacterium-angustum]	661
WP_045149213.1	DndB-ParB-nuclease	GCF_000950005.1	369	Bacteria>Proteobacteria>Gammaproteobacteria>Vibrionales>Vibrionaceae	Photobacterium_angustum	DNA-sulfur-modification-protein-DndB-[Photobacterium-angustum]	661
WP_230626982.1	DndB-ParB-nuclease	GCF_000950015.1	297	Bacteria>Proteobacteria>Gammaproteobacteria>Vibrionales>Vibrionaceae	Photobacterium_angustum	DNA-sulfur-modification-protein-DndB-[Photobacterium-angustum]	661
WP_230626983.1	DndB-ParB-nuclease	GCF_000950015.1	54	Bacteria>Proteobacteria>Gammaproteobacteria>Vibrionales>Vibrionaceae	Photobacterium_angustum	hypothetical-protein-[Photobacterium-angustum]	661
WP_045127915.1	DndB-ParB-nuclease	GCF_000950015.1	695	Bacteria>Proteobacteria>Gammaproteobacteria>Vibrionales>Vibrionaceae	Photobacterium_angustum	hypothetical-protein-[Photobacterium-angustum]	661
WP_232577265.1	DndB-ParB-nuclease	GCF_021504665.1	369	Bacteria>Proteobacteria>Gammaproteobacteria>Vibrionales>Vibrionaceae	Photobacterium_carnosum	DNA-sulfur-modification-protein-DndB-[Photobacterium-carnosum]	2023717
WP_237029478.1	DndB-ParB-nuclease	GCF_021768385.1	412	Bacteria>Proteobacteria>Gammaproteobacteria>Vibrionales>Vibrionaceae	Photobacterium_damselae	DGQHR-domain-containing-protein-[Photobacterium-damselae]	38293
WP_176285880.1	DndB-ParB-nuclease	GCF_013371545.1	370	Bacteria>Proteobacteria>Gammaproteobacteria>Vibrionales>Vibrionaceae	Photobacterium_damselae	DNA-sulfur-modification-protein-DndB-[Photobacterium-damselae]	38293
WP_237029474.1	DndB-ParB-nuclease	GCF_021768385.1	370	Bacteria>Proteobacteria>Gammaproteobacteria>Vibrionales>Vibrionaceae	Photobacterium_damselae	DNA-sulfur-modification-protein-DndB-[Photobacterium-damselae]	38293
WP_044624138.1	DndB-ParB-nuclease	GCF_000940995.1	407	Bacteria>Proteobacteria>Gammaproteobacteria>Vibrionales>Vibrionaceae	Photobacterium_gaetbulicola	DGQHR-domain-containing-protein-[Photobacterium-gaetbulicola]	1295392
WP_044624134.1	DndB-ParB-nuclease	GCF_000940995.1	359	Bacteria>Proteobacteria>Gammaproteobacteria>Vibrionales>Vibrionaceae	Photobacterium_gaetbulicola	DNA-sulfur-modification-protein-DndB-[Photobacterium-gaetbulicola]	1295392
WP_107275196.1	DndB-ParB-nuclease	GCF_003025455.1	426	Bacteria>Proteobacteria>Gammaproteobacteria>Vibrionales>Vibrionaceae	Photobacterium_iliopiscarium	DGQHR-domain-containing-protein-[Photobacterium-iliopiscarium]	56192
WP_107275199.1	DndB-ParB-nuclease	GCF_003025455.1	364	Bacteria>Proteobacteria>Gammaproteobacteria>Vibrionales>Vibrionaceae	Photobacterium_iliopiscarium	DNA-sulfur-modification-protein-DndB-[Photobacterium-iliopiscarium]	56192
WP_036789716.1	DndB-ParB-nuclease	GCF_000613045.3	369	Bacteria>Proteobacteria>Gammaproteobacteria>Vibrionales>Vibrionaceae	Photobacterium_kishitanii	DNA-sulfur-modification-protein-DndB-[Photobacterium-kishitanii]	318456
WP_107289579.1	DndB-ParB-nuclease	GCF_003025915.1	364	Bacteria>Proteobacteria>Gammaproteobacteria>Vibrionales>Vibrionaceae	Photobacterium_kishitanii	DNA-sulfur-modification-protein-DndB-[Photobacterium-kishitanii]	318456
WP_201797899.1	DndB-ParB-nuclease	GCF_003026055.1	376	Bacteria>Proteobacteria>Gammaproteobacteria>Vibrionales>Vibrionaceae	Photobacterium_leiognathi	DGQHR-domain-containing-protein-[Photobacterium-leiognathi]	553611
WP_107185525.1	DndB-ParB-nuclease	GCF_003026055.1	369	Bacteria>Proteobacteria>Gammaproteobacteria>Vibrionales>Vibrionaceae	Photobacterium_leiognathi	DNA-sulfur-modification-protein-DndB-[Photobacterium-leiognathi]	553611
WP_087844576.1	DndB-ParB-nuclease	GCF_900185615.1	364	Bacteria>Proteobacteria>Gammaproteobacteria>Vibrionales>Vibrionaceae	Photobacterium_malacitanum	DNA-sulfur-modification-protein-DndB-[Photobacterium-malacitanum]	2204294
WP_107273130.1	DndB-ParB-nuclease	GCF_003026535.1	359	Bacteria>Proteobacteria>Gammaproteobacteria>Vibrionales>Vibrionaceae	Photobacterium_phosphoreum	DNA-sulfur-modification-protein-DndB-[Photobacterium-phosphoreum]	659
WP_232610744.1	DndB-ParB-nuclease	GCF_021236585.1	369	Bacteria>Proteobacteria>Gammaproteobacteria>Vibrionales>Vibrionaceae	Photobacterium_phosphoreum	DNA-sulfur-modification-protein-DndB-[Photobacterium-phosphoreum]	659
WP_075762466.1	DndB-ParB-nuclease	GCF_001939735.1	376	Bacteria>Proteobacteria>Gammaproteobacteria>Vibrionales>Vibrionaceae	Photobacterium_proteolyticum	DGQHR-domain-containing-protein-[Photobacterium-proteolyticum]	1903952
WP_075762462.1	DndB-ParB-nuclease	GCF_001939735.1	369	Bacteria>Proteobacteria>Gammaproteobacteria>Vibrionales>Vibrionaceae	Photobacterium_proteolyticum	DNA-sulfur-modification-protein-DndB-[Photobacterium-proteolyticum]	1903952
WP_077661891.1	DndB-ParB-nuclease	GCF_001996225.1	568	Bacteria>Proteobacteria>Gammaproteobacteria>Vibrionales>Vibrionaceae	Salinivibrio_proteolyticus	AIPR-family-protein-[Salinivibrio-proteolyticus]	334715

Accession	Annotation	GCA	Length	Phyletic.spread	Strain.name	Defline	Species.Taxid
OOF31911.1	DndB-ParB-nuclease	GCA_001996225.1	358	Bacteria>Proteobacteria>Gammaproteobacteria>Vibrionales>Vibrionaceae	Salinivibrio_proteolyticus	DNA-sulfur-modification-protein-DndB-[Salinivibrio-proteolyticus]	334715
WP_077661895.1	DndB-ParB-nuclease	GCF_001996225.1	358	Bacteria>Proteobacteria>Gammaproteobacteria>Vibrionales>Vibrionaceae	Salinivibrio_proteolyticus	DNA-sulfur-modification-protein-DndB-[Salinivibrio-proteolyticus]	334715
OOF31907.1	DndB-ParB-nuclease	GCA_001996225.1	568	Bacteria>Proteobacteria>Gammaproteobacteria>Vibrionales>Vibrionaceae	Salinivibrio_proteolyticus	hypothetical-protein-BZJ20_01825-[Salinivibrio-proteolyticus]	334715
WP_086939616.1	DndB-ParB-nuclease	GCF_002157795.1	369	Bacteria>Proteobacteria>Gammaproteobacteria>Vibrionales>Vibrionaceae	Thaumasiovibrio_occultus	DNA-sulfur-modification-protein-DndB-[Thaumasiovibrio-occultus]	1891184
WP_102479236.1	DndB-ParB-nuclease	GCF_002874195.1	359	Bacteria>Proteobacteria>Gammaproteobacteria>Vibrionales>Vibrionaceae	unclassified_Vibrio	MULTISPECIES:-DNA-sulfur-modification-protein-DndB-[unclassified-Vibrio]	2614977
WP_213917105.1	DndB-ParB-nuclease	GCF_018458675.1	568	Bacteria>Proteobacteria>Gammaproteobacteria>Vibrionales>Vibrionaceae	Vibrio_alginolyticus	AIPR-family-protein-[Vibrio-alginolyticus]	663
HBC3524044.1	DndB-ParB-nuclease	GCA_018119365.1	374	Bacteria>Proteobacteria>Gammaproteobacteria>Vibrionales>Vibrionaceae	Vibrio_alginolyticus	DGQHR-domain-containing-protein-[Vibrio-alginolyticus]	663
EGR0146571.1	DndB-ParB-nuclease	GCA_015787635.1	359	Bacteria>Proteobacteria>Gammaproteobacteria>Vibrionales>Vibrionaceae	Vibrio_alginolyticus	DNA-sulfur-modification-protein-DndB-[Vibrio-alginolyticus]	663
EGR0169284.1	DndB-ParB-nuclease	GCA_015787435.1	364	Bacteria>Proteobacteria>Gammaproteobacteria>Vibrionales>Vibrionaceae	Vibrio_alginolyticus	DNA-sulfur-modification-protein-DndB-[Vibrio-alginolyticus]	663
EGR0721917.1	DndB-ParB-nuclease	GCA_015789455.1	359	Bacteria>Proteobacteria>Gammaproteobacteria>Vibrionales>Vibrionaceae	Vibrio_alginolyticus	DNA-sulfur-modification-protein-DndB-[Vibrio-alginolyticus]	663
EGR0801714.1	DndB-ParB-nuclease	GCA_015790525.1	359	Bacteria>Proteobacteria>Gammaproteobacteria>Vibrionales>Vibrionaceae	Vibrio_alginolyticus	DNA-sulfur-modification-protein-DndB-[Vibrio-alginolyticus]	663
EIL2909394.1	DndB-ParB-nuclease	GCA_020688565.1	364	Bacteria>Proteobacteria>Gammaproteobacteria>Vibrionales>Vibrionaceae	Vibrio_alginolyticus	DNA-sulfur-modification-protein-DndB-[Vibrio-alginolyticus]	663
EIP0119384.1	DndB-ParB-nuclease	GCA_021014255.1	359	Bacteria>Proteobacteria>Gammaproteobacteria>Vibrionales>Vibrionaceae	Vibrio_alginolyticus	DNA-sulfur-modification-protein-DndB-[Vibrio-alginolyticus]	663
HBC3524040.1	DndB-ParB-nuclease	GCA_018119365.1	359	Bacteria>Proteobacteria>Gammaproteobacteria>Vibrionales>Vibrionaceae	Vibrio_alginolyticus	DNA-sulfur-modification-protein-DndB-[Vibrio-alginolyticus]	663
MBS9955703.1	DndB-ParB-nuclease	GCA_018458025.1	359	Bacteria>Proteobacteria>Gammaproteobacteria>Vibrionales>Vibrionaceae	Vibrio_alginolyticus	DNA-sulfur-modification-protein-DndB-[Vibrio-alginolyticus]	663
UFN70403.1	DndB-ParB-nuclease	GCA_020991225.1	359	Bacteria>Proteobacteria>Gammaproteobacteria>Vibrionales>Vibrionaceae	Vibrio_alginolyticus	DNA-sulfur-modification-protein-DndB-[Vibrio-alginolyticus]	663
WP_054576714.1	DndB-ParB-nuclease	GCF_022343165.1	359	Bacteria>Proteobacteria>Gammaproteobacteria>Vibrionales>Vibrionaceae	Vibrio_alginolyticus	DNA-sulfur-modification-protein-DndB-[Vibrio-alginolyticus]	663
WP_213893704.1	DndB-ParB-nuclease	GCF_018458025.1	359	Bacteria>Proteobacteria>Gammaproteobacteria>Vibrionales>Vibrionaceae	Vibrio_alginolyticus	DNA-sulfur-modification-protein-DndB-[Vibrio-alginolyticus]	663
WP_225455701.1	DndB-ParB-nuclease	GCF_020223855.1	359	Bacteria>Proteobacteria>Gammaproteobacteria>Vibrionales>Vibrionaceae	Vibrio_alginolyticus	DNA-sulfur-modification-protein-DndB-[Vibrio-alginolyticus]	663
BCB43912.1	DndB-ParB-nuclease	GCA_012151155.1	359	Bacteria>Proteobacteria>Gammaproteobacteria>Vibrionales>Vibrionaceae	Vibrio_alginolyticus	hypothetical-protein-Vag1382_30390-[Vibrio-alginolyticus]	663
WP_194663165.1	DndB-ParB-nuclease	GCF_015343015.1	570	Bacteria>Proteobacteria>Gammaproteobacteria>Vibrionales>Vibrionaceae	Vibrio_anguillarum	AIPR-family-protein-[Vibrio-anguillarum]	55601
WP_088720975.1	DndB-ParB-nuclease	GCF_002211985.1	359	Bacteria>Proteobacteria>Gammaproteobacteria>Vibrionales>Vibrionaceae	Vibrio_anguillarum	DNA-sulfur-modification-protein-DndB-[Vibrio-anguillarum]	55601
WP_102382459.1	DndB-ParB-nuclease	GCF_002877085.1	360	Bacteria>Proteobacteria>Gammaproteobacteria>Vibrionales>Vibrionaceae	Vibrio_breoganii	DNA-sulfur-modification-protein-DndB-[Vibrio-breoganii]	553239
WP_102499009.1	DndB-ParB-nuclease	GCF_002877285.1	360	Bacteria>Proteobacteria>Gammaproteobacteria>Vibrionales>Vibrionaceae	Vibrio_breoganii	DNA-sulfur-modification-protein-DndB-[Vibrio-breoganii]	553239
WP_102501900.1	DndB-ParB-nuclease	GCF_002877305.1	360	Bacteria>Proteobacteria>Gammaproteobacteria>Vibrionales>Vibrionaceae	Vibrio_breoganii	DNA-sulfur-modification-protein-DndB-[Vibrio-breoganii]	553239
WP_102480103.1	DndB-ParB-nuclease	GCF_002875195.1	687	Bacteria>Proteobacteria>Gammaproteobacteria>Vibrionales>Vibrionaceae	Vibrio_breoganii	hypothetical-protein-[Vibrio-breoganii]	553239
WP_045370511.1	DndB-ParB-nuclease	GCF_000818315.1	568	Bacteria>Proteobacteria>Gammaproteobacteria>Vibrionales>Vibrionaceae	Vibrio_campbellii	AIPR-family-protein-[Vibrio-campbellii]	680
PQJ47546.1	DndB-ParB-nuclease	GCA_002954485.1	359	Bacteria>Proteobacteria>Gammaproteobacteria>Vibrionales>Vibrionaceae	Vibrio_campbellii	DNA-sulfur-modification-protein-DndB-[Vibrio-campbellii]	680
WP_050910231.1	DndB-ParB-nuclease	GCF_001185265.1	359	Bacteria>Proteobacteria>Gammaproteobacteria>Vibrionales>Vibrionaceae	Vibrio_campbellii	DNA-sulfur-modification-protein-DndB-[Vibrio-campbellii]	680
WP_081234397.1	DndB-ParB-nuclease	GCF_002078065.1	359	Bacteria>Proteobacteria>Gammaproteobacteria>Vibrionales>Vibrionaceae	Vibrio_campbellii	DNA-sulfur-modification-protein-DndB-[Vibrio-campbellii]	680
PQJ47544.1	DndB-ParB-nuclease	GCA_002954485.1	379	Bacteria>Proteobacteria>Gammaproteobacteria>Vibrionales>Vibrionaceae	Vibrio_campbellii	hypothetical-protein-BTN99_06315-[Vibrio-campbellii]	680
WP_137408654.1	DndB-ParB-nuclease	GCF_024347355.1	386	Bacteria>Proteobacteria>Gammaproteobacteria>Vibrionales>Vibrionaceae	Vibrio_chagasii	DGQHR-domain-containing-protein-[Vibrio-chagasii]	170679
WP_137408658.1	DndB-ParB-nuclease	GCF_024347355.1	359	Bacteria>Proteobacteria>Gammaproteobacteria>Vibrionales>Vibrionaceae	Vibrio_chagasii	DNA-sulfur-modification-protein-DndB-[Vibrio-chagasii]	170679
EKG72082.1	DndB-ParB-nuclease	GCA_000302965.1	359	Bacteria>Proteobacteria>Gammaproteobacteria>Vibrionales>Vibrionaceae	Vibrio_cholerae_CP1037(10)	DGQHR-domain-protein-[Vibrio-cholerae-CP1037(10)]	991992
EKG72083.1	DndB-ParB-nuclease	GCA_000302965.1	404	Bacteria>Proteobacteria>Gammaproteobacteria>Vibrionales>Vibrionaceae	Vibrio_cholerae_CP1037(10)	DGQHR-domain-protein-[Vibrio-cholerae-CP1037(10)]	991992
EGR0493525.1	DndB-ParB-nuclease	GCA_015788535.1	407	Bacteria>Proteobacteria>Gammaproteobacteria>Vibrionales>Vibrionaceae	Vibrio_cholerae	DGQHR-domain-containing-protein-[Vibrio-cholerae]	666
EGR1963853.1	DndB-ParB-nuclease	GCA_015801135.1	376	Bacteria>Proteobacteria>Gammaproteobacteria>Vibrionales>Vibrionaceae	Vibrio_cholerae	DGQHR-domain-containing-protein-[Vibrio-cholerae]	666
EGR2240179.1	DndB-ParB-nuclease	GCA_015803195.1	402	Bacteria>Proteobacteria>Gammaproteobacteria>Vibrionales>Vibrionaceae	Vibrio_cholerae	DGQHR-domain-containing-protein-[Vibrio-cholerae]	666
EGR2448902.1	DndB-ParB-nuclease	GCA_015802055.1	388	Bacteria>Proteobacteria>Gammaproteobacteria>Vibrionales>Vibrionaceae	Vibrio_cholerae	DGQHR-domain-containing-protein-[Vibrio-cholerae]	666
EGR5122227.1	DndB-ParB-nuclease	GCA_015815185.1	387	Bacteria>Proteobacteria>Gammaproteobacteria>Vibrionales>Vibrionaceae	Vibrio_cholerae	DGQHR-domain-containing-protein-[Vibrio-cholerae]	666
EHB5528753.1	DndB-ParB-nuclease	GCA_016982955.1	415	Bacteria>Proteobacteria>Gammaproteobacteria>Vibrionales>Vibrionaceae	Vibrio_cholerae	DGQHR-domain-containing-protein-[Vibrio-cholerae]	666
EII3091978.1	DndB-ParB-nuclease	GCA_020411025.1	427	Bacteria>Proteobacteria>Gammaproteobacteria>Vibrionales>Vibrionaceae	Vibrio_cholerae	DGQHR-domain-containing-protein-[Vibrio-cholerae]	666
HBC3377930.1	DndB-ParB-nuclease	GCA_018118685.1	376	Bacteria>Proteobacteria>Gammaproteobacteria>Vibrionales>Vibrionaceae	Vibrio_cholerae	DGQHR-domain-containing-protein-[Vibrio-cholerae]	666
MVB47363.1	DndB-ParB-nuclease	GCA_009746685.1	415	Bacteria>Proteobacteria>Gammaproteobacteria>Vibrionales>Vibrionaceae	Vibrio_cholerae	DGQHR-domain-containing-protein-[Vibrio-cholerae]	666
WP_000066187.1	DndB-ParB-nuclease	GCF_000788715.2	404	Bacteria>Proteobacteria>Gammaproteobacteria>Vibrionales>Vibrionaceae	Vibrio_cholerae	DGQHR-domain-containing-protein-[Vibrio-cholerae]	666
WP_000632620.1	DndB-ParB-nuclease	GCF_019092835.1	415	Bacteria>Proteobacteria>Gammaproteobacteria>Vibrionales>Vibrionaceae	Vibrio_cholerae	DGQHR-domain-containing-protein-[Vibrio-cholerae]	666
WP_001896510.1	DndB-ParB-nuclease	GCF_000168935.2	402	Bacteria>Proteobacteria>Gammaproteobacteria>Vibrionales>Vibrionaceae	Vibrio_cholerae	DGQHR-domain-containing-protein-[Vibrio-cholerae]	666
WP_032471453.1	DndB-ParB-nuclease	GCF_000736765.1	402	Bacteria>Proteobacteria>Gammaproteobacteria>Vibrionales>Vibrionaceae	Vibrio_cholerae	DGQHR-domain-containing-protein-[Vibrio-cholerae]	666
WP_095476952.1	DndB-ParB-nuclease	GCF_002284125.1	374	Bacteria>Proteobacteria>Gammaproteobacteria>Vibrionales>Vibrionaceae	Vibrio_cholerae	DGQHR-domain-containing-protein-[Vibrio-cholerae]	666
WP_119467560.1	DndB-ParB-nuclease	GCF_003585405.1	393	Bacteria>Proteobacteria>Gammaproteobacteria>Vibrionales>Vibrionaceae	Vibrio_cholerae	DGQHR-domain-containing-protein-[Vibrio-cholerae]	666
WP_187373357.1	DndB-ParB-nuclease	GCF_008083915.1	403	Bacteria>Proteobacteria>Gammaproteobacteria>Vibrionales>Vibrionaceae	Vibrio_cholerae	DGQHR-domain-containing-protein-[Vibrio-cholerae]	666
WP_199370457.1	DndB-ParB-nuclease	GCF_016456815.1	376	Bacteria>Proteobacteria>Gammaproteobacteria>Vibrionales>Vibrionaceae	Vibrio_cholerae	DGQHR-domain-containing-protein-[Vibrio-cholerae]	666
WP_230606207.1	DndB-ParB-nuclease	GCF_021014705.1	374	Bacteria>Proteobacteria>Gammaproteobacteria>Vibrionales>Vibrionaceae	Vibrio_cholerae	DGQHR-domain-containing-protein-[Vibrio-cholerae]	666
EGQ8410296.1	DndB-ParB-nuclease	GCA_015775875.1	359	Bacteria>Proteobacteria>Gammaproteobacteria>Vibrionales>Vibrionaceae	Vibrio_cholerae	DNA-sulfur-modification-protein-DndB-[Vibrio-cholerae]	666
EGQ9729705.1	DndB-ParB-nuclease	GCA_015785955.1	359	Bacteria>Proteobacteria>Gammaproteobacteria>Vibrionales>Vibrionaceae	Vibrio_cholerae	DNA-sulfur-modification-protein-DndB-[Vibrio-cholerae]	666
EGR0493521.1	DndB-ParB-nuclease	GCA_015788535.1	359	Bacteria>Proteobacteria>Gammaproteobacteria>Vibrionales>Vibrionaceae	Vibrio_cholerae	DNA-sulfur-modification-protein-DndB-[Vibrio-cholerae]	666
EGR0891595.1	DndB-ParB-nuclease	GCA_015794775.1	364	Bacteria>Proteobacteria>Gammaproteobacteria>Vibrionales>Vibrionaceae	Vibrio_cholerae	DNA-sulfur-modification-protein-DndB-[Vibrio-cholerae]	666
EGR1963849.1	DndB-ParB-nuclease	GCA_015801135.1	359	Bacteria>Proteobacteria>Gammaproteobacteria>Vibrionales>Vibrionaceae	Vibrio_cholerae	DNA-sulfur-modification-protein-DndB-[Vibrio-cholerae]	666
EGR2240175.1	DndB-ParB-nuclease	GCA_015803195.1	359	Bacteria>Proteobacteria>Gammaproteobacteria>Vibrionales>Vibrionaceae	Vibrio_cholerae	DNA-sulfur-modification-protein-DndB-[Vibrio-cholerae]	666
EGR2448906.1	DndB-ParB-nuclease	GCA_015802055.1	359	Bacteria>Proteobacteria>Gammaproteobacteria>Vibrionales>Vibrionaceae	Vibrio_cholerae	DNA-sulfur-modification-protein-DndB-[Vibrio-cholerae]	666
EGR5122223.1	DndB-ParB-nuclease	GCA_015815185.1	359	Bacteria>Proteobacteria>Gammaproteobacteria>Vibrionales>Vibrionaceae	Vibrio_cholerae	DNA-sulfur-modification-protein-DndB-[Vibrio-cholerae]	666
EHB5528749.1	DndB-ParB-nuclease	GCA_016982955.1	359	Bacteria>Proteobacteria>Gammaproteobacteria>Vibrionales>Vibrionaceae	Vibrio_cholerae	DNA-sulfur-modification-protein-DndB-[Vibrio-cholerae]	666
EII3091974.1	DndB-ParB-nuclease	GCA_020411025.1	364	Bacteria>Proteobacteria>Gammaproteobacteria>Vibrionales>Vibrionaceae	Vibrio_cholerae	DNA-sulfur-modification-protein-DndB-[Vibrio-cholerae]	666
HAS3168881.1	DndB-ParB-nuclease	GCA_015746565.1	359	Bacteria>Proteobacteria>Gammaproteobacteria>Vibrionales>Vibrionaceae	Vibrio_cholerae	DNA-sulfur-modification-protein-DndB-[Vibrio-cholerae]	666
HAS3591800.1	DndB-ParB-nuclease	GCA_015749205.1	359	Bacteria>Proteobacteria>Gammaproteobacteria>Vibrionales>Vibrionaceae	Vibrio_cholerae	DNA-sulfur-modification-protein-DndB-[Vibrio-cholerae]	666
KAA1199063.1	DndB-ParB-nuclease	GCA_008370435.1	364	Bacteria>Proteobacteria>Gammaproteobacteria>Vibrionales>Vibrionaceae	Vibrio_cholerae	DNA-sulfur-modification-protein-DndB-[Vibrio-cholerae]	666
MVB19476.1	DndB-ParB-nuclease	GCA_009746845.1	359	Bacteria>Proteobacteria>Gammaproteobacteria>Vibrionales>Vibrionaceae	Vibrio_cholerae	DNA-sulfur-modification-protein-DndB-[Vibrio-cholerae]	666
MVB47367.1	DndB-ParB-nuclease	GCA_009746685.1	359	Bacteria>Proteobacteria>Gammaproteobacteria>Vibrionales>Vibrionaceae	Vibrio_cholerae	DNA-sulfur-modification-protein-DndB-[Vibrio-cholerae]	666
ORP16208.1	DndB-ParB-nuclease	GCA_002097735.1	359	Bacteria>Proteobacteria>Gammaproteobacteria>Vibrionales>Vibrionaceae	Vibrio_cholerae	DNA-sulfur-modification-protein-DndB-[Vibrio-cholerae]	666
QKU71313.1	DndB-ParB-nuclease	GCA_013357805.1	364	Bacteria>Proteobacteria>Gammaproteobacteria>Vibrionales>Vibrionaceae	Vibrio_cholerae	DNA-sulfur-modification-protein-DndB-[Vibrio-cholerae]	666
QKV04278.1	DndB-ParB-nuclease	GCA_013357605.1	364	Bacteria>Proteobacteria>Gammaproteobacteria>Vibrionales>Vibrionaceae	Vibrio_cholerae	DNA-sulfur-modification-protein-DndB-[Vibrio-cholerae]	666
RND30737.1	DndB-ParB-nuclease	GCA_003712005.1	364	Bacteria>Proteobacteria>Gammaproteobacteria>Vibrionales>Vibrionaceae	Vibrio_cholerae	DNA-sulfur-modification-protein-DndB-[Vibrio-cholerae]	666
WP_000141154.1	DndB-ParB-nuclease	GCF_000174235.1	364	Bacteria>Proteobacteria>Gammaproteobacteria>Vibrionales>Vibrionaceae	Vibrio_cholerae	DNA-sulfur-modification-protein-DndB-[Vibrio-cholerae]	666
WP_000551843.1	DndB-ParB-nuclease	GCF_000788715.2	359	Bacteria>Proteobacteria>Gammaproteobacteria>Vibrionales>Vibrionaceae	Vibrio_cholerae	DNA-sulfur-modification-protein-DndB-[Vibrio-cholerae]	666
WP_057568912.1	DndB-ParB-nuclease	GCF_013357605.1	364	Bacteria>Proteobacteria>Gammaproteobacteria>Vibrionales>Vibrionaceae	Vibrio_cholerae	DNA-sulfur-modification-protein-DndB-[Vibrio-cholerae]	666
WP_149145369.1	DndB-ParB-nuclease	GCF_008269365.1	359	Bacteria>Proteobacteria>Gammaproteobacteria>Vibrionales>Vibrionaceae	Vibrio_cholerae	DNA-sulfur-modification-protein-DndB-[Vibrio-cholerae]	666
WP_175248210.1	DndB-ParB-nuclease	GCF_013357685.1	359	Bacteria>Proteobacteria>Gammaproteobacteria>Vibrionales>Vibrionaceae	Vibrio_cholerae	DNA-sulfur-modification-protein-DndB-[Vibrio-cholerae]	666
WP_199369008.1	DndB-ParB-nuclease	GCF_016457125.1	359	Bacteria>Proteobacteria>Gammaproteobacteria>Vibrionales>Vibrionaceae	Vibrio_cholerae	DNA-sulfur-modification-protein-DndB-[Vibrio-cholerae]	666
WP_230606208.1	DndB-ParB-nuclease	GCF_021014625.1	359	Bacteria>Proteobacteria>Gammaproteobacteria>Vibrionales>Vibrionaceae	Vibrio_cholerae	DNA-sulfur-modification-protein-DndB-[Vibrio-cholerae]	666
EGR0891599.1	DndB-ParB-nuclease	GCA_015794775.1	391	Bacteria>Proteobacteria>Gammaproteobacteria>Vibrionales>Vibrionaceae	Vibrio_cholerae	hypothetical-protein-[Vibrio-cholerae]	666
WP_080389580.1	DndB-ParB-nuclease	GCF_001402535.1	722	Bacteria>Proteobacteria>Gammaproteobacteria>Vibrionales>Vibrionaceae	Vibrio_cholerae	hypothetical-protein-[Vibrio-cholerae]	666
WP_142625638.1	DndB-ParB-nuclease	GCF_006802865.1	660	Bacteria>Proteobacteria>Gammaproteobacteria>Vibrionales>Vibrionaceae	Vibrio_cholerae	hypothetical-protein-[Vibrio-cholerae]	666
WP_205351946.1	DndB-ParB-nuclease	GCF_002284235.1	494	Bacteria>Proteobacteria>Gammaproteobacteria>Vibrionales>Vibrionaceae	Vibrio_cholerae	hypothetical-protein-[Vibrio-cholerae]	666
WP_217633251.1	DndB-ParB-nuclease	GCF_001857485.1	557	Bacteria>Proteobacteria>Gammaproteobacteria>Vibrionales>Vibrionaceae	Vibrio_cholerae	hypothetical-protein-[Vibrio-cholerae]	666
HBC3377926.1	DndB-ParB-nuclease	GCA_018118685.1	359	Bacteria>Proteobacteria>Gammaproteobacteria>Vibrionales>Vibrionaceae	Vibrio_cholerae	TPA:-DNA-sulfur-modification-protein-DndB-[Vibrio-cholerae]	666
HBC3475375.1	DndB-ParB-nuclease	GCA_018119245.1	364	Bacteria>Proteobacteria>Gammaproteobacteria>Vibrionales>Vibrionaceae	Vibrio_cholerae	TPA:-DNA-sulfur-modification-protein-DndB-[Vibrio-cholerae]	666
WP_065819647.1	DndB-ParB-nuclease	GCF_009763805.1	359	Bacteria>Proteobacteria>Gammaproteobacteria>Vibrionales>Vibrionaceae	Vibrio_cidicii	DNA-sulfur-modification-protein-DndB-[Vibrio-cidicii]	1763883
NRF14112.1	DndB-ParB-nuclease	GCA_013266605.1	364	Bacteria>Proteobacteria>Gammaproteobacteria>Vibrionales>Vibrionaceae	Vibrio_coralliilyticus	DNA-sulfur-modification-protein-DndB-[Vibrio-coralliilyticus]	190893
WP_172853914.1	DndB-ParB-nuclease	GCF_013266605.1	364	Bacteria>Proteobacteria>Gammaproteobacteria>Vibrionales>Vibrionaceae	Vibrio_coralliilyticus	DNA-sulfur-modification-protein-DndB-[Vibrio-coralliilyticus]	190893
NRF14116.1	DndB-ParB-nuclease	GCA_013266605.1	723	Bacteria>Proteobacteria>Gammaproteobacteria>Vibrionales>Vibrionaceae	Vibrio_coralliilyticus	hypothetical-protein-[Vibrio-coralliilyticus]	190893
WP_172853918.1	DndB-ParB-nuclease	GCF_013266605.1	723	Bacteria>Proteobacteria>Gammaproteobacteria>Vibrionales>Vibrionaceae	Vibrio_coralliilyticus	hypothetical-protein-[Vibrio-coralliilyticus]	190893
WP_050052920.1	DndB-ParB-nuclease	GCF_001048595.1	568	Bacteria>Proteobacteria>Gammaproteobacteria>Vibrionales>Vibrionaceae	Vibrio_coralliirubri	AIPR-family-protein-[Vibrio-coralliirubri]	1516159
WP_048666157.1	DndB-ParB-nuclease	GCF_024347415.1	387	Bacteria>Proteobacteria>Gammaproteobacteria>Vibrionales>Vibrionaceae	Vibrio_crassostreae	DGQHR-domain-containing-protein-[Vibrio-crassostreae]	246167
WP_171349757.1	DndB-ParB-nuclease	GCF_013114165.1	376	Bacteria>Proteobacteria>Gammaproteobacteria>Vibrionales>Vibrionaceae	Vibrio_crassostreae	DGQHR-domain-containing-protein-[Vibrio-crassostreae]	246167
WP_213865437.1	DndB-ParB-nuclease	-	411	Bacteria>Proteobacteria>Gammaproteobacteria>Vibrionales>Vibrionaceae	Vibrio_crassostreae	DGQHR-domain-containing-protein-[Vibrio-crassostreae]	246167
WP_123422800.1	DndB-ParB-nuclease	GCF_003751155.1	359	Bacteria>Proteobacteria>Gammaproteobacteria>Vibrionales>Vibrionaceae	Vibrio_crassostreae	DNA-sulfur-modification-protein-DndB-[Vibrio-crassostreae]	246167
WP_132815727.1	DndB-ParB-nuclease	GCF_017917535.1	364	Bacteria>Proteobacteria>Gammaproteobacteria>Vibrionales>Vibrionaceae	Vibrio_crassostreae	DNA-sulfur-modification-protein-DndB-[Vibrio-crassostreae]	246167
WP_132968426.1	DndB-ParB-nuclease	GCF_004342815.1	359	Bacteria>Proteobacteria>Gammaproteobacteria>Vibrionales>Vibrionaceae	Vibrio_crassostreae	DNA-sulfur-modification-protein-DndB-[Vibrio-crassostreae]	246167
WP_213865439.1	DndB-ParB-nuclease	GCF_018440535.1	359	Bacteria>Proteobacteria>Gammaproteobacteria>Vibrionales>Vibrionaceae	Vibrio_crassostreae	DNA-sulfur-modification-protein-DndB-[Vibrio-crassostreae]	246167
WP_210444872.1	DndB-ParB-nuclease	GCF_017917535.1	739	Bacteria>Proteobacteria>Gammaproteobacteria>Vibrionales>Vibrionaceae	Vibrio_crassostreae	hypothetical-protein-[Vibrio-crassostreae]	246167
WP_102465668.1	DndB-ParB-nuclease	GCF_002873515.1	565	Bacteria>Proteobacteria>Gammaproteobacteria>Vibrionales>Vibrionaceae	Vibrio_cyclitrophicus	AIPR-family-protein-[Vibrio-cyclitrophicus]	47951
WP_065107147.1	DndB-ParB-nuclease	GCF_001675875.1	394	Bacteria>Proteobacteria>Gammaproteobacteria>Vibrionales>Vibrionaceae	Vibrio_cyclitrophicus	DGQHR-domain-containing-protein-[Vibrio-cyclitrophicus]	47951
WP_171342060.1	DndB-ParB-nuclease	GCF_013113845.1	392	Bacteria>Proteobacteria>Gammaproteobacteria>Vibrionales>Vibrionaceae	Vibrio_cyclitrophicus	DGQHR-domain-containing-protein-[Vibrio-cyclitrophicus]	47951
KAA8598629.1	DndB-ParB-nuclease	GCA_008692145.1	362	Bacteria>Proteobacteria>Gammaproteobacteria>Vibrionales>Vibrionaceae	Vibrio_cyclitrophicus	DNA-sulfur-modification-protein-DndB-[Vibrio-cyclitrophicus]	47951
WP_016794128.1	DndB-ParB-nuclease	GCF_000256325.2	359	Bacteria>Proteobacteria>Gammaproteobacteria>Vibrionales>Vibrionaceae	Vibrio_cyclitrophicus	DNA-sulfur-modification-protein-DndB-[Vibrio-cyclitrophicus]	47951
WP_102269953.1	DndB-ParB-nuclease	GCF_020806125.1	364	Bacteria>Proteobacteria>Gammaproteobacteria>Vibrionales>Vibrionaceae	Vibrio_cyclitrophicus	DNA-sulfur-modification-protein-DndB-[Vibrio-cyclitrophicus]	47951
WP_102305505.1	DndB-ParB-nuclease	GCF_023206055.1	359	Bacteria>Proteobacteria>Gammaproteobacteria>Vibrionales>Vibrionaceae	Vibrio_cyclitrophicus	DNA-sulfur-modification-protein-DndB-[Vibrio-cyclitrophicus]	47951
WP_102418246.1	DndB-ParB-nuclease	GCF_002874945.1	359	Bacteria>Proteobacteria>Gammaproteobacteria>Vibrionales>Vibrionaceae	Vibrio_cyclitrophicus	DNA-sulfur-modification-protein-DndB-[Vibrio-cyclitrophicus]	47951
WP_102465674.1	DndB-ParB-nuclease	GCF_002873515.1	359	Bacteria>Proteobacteria>Gammaproteobacteria>Vibrionales>Vibrionaceae	Vibrio_cyclitrophicus	DNA-sulfur-modification-protein-DndB-[Vibrio-cyclitrophicus]	47951
WP_102269949.1	DndB-ParB-nuclease	GCF_020806125.1	730	Bacteria>Proteobacteria>Gammaproteobacteria>Vibrionales>Vibrionaceae	Vibrio_cyclitrophicus	hypothetical-protein-[Vibrio-cyclitrophicus]	47951
WP_132968429.1	DndB-ParB-nuclease	GCF_020806315.1	403	Bacteria>Proteobacteria>Gammaproteobacteria>Vibrionales>Vibrionaceae	Vibrio	DGQHR-domain-containing-protein-[Vibrio-crassostreae]	662
WP_104974352.1	DndB-ParB-nuclease	GCF_002953355.1	359	Bacteria>Proteobacteria>Gammaproteobacteria>Vibrionales>Vibrionaceae	Vibrio_diabolicus	DNA-sulfur-modification-protein-DndB-[Vibrio-diabolicus]	50719
WP_102965365.1	DndB-ParB-nuclease	GCF_002892945.1	359	Bacteria>Proteobacteria>Gammaproteobacteria>Vibrionales>Vibrionaceae	Vibrio_diazotrophicus	DNA-sulfur-modification-protein-DndB-[Vibrio-diazotrophicus]	685
WP_032471449.1	DndB-ParB-nuclease	GCF_000736765.1	359	Bacteria>Proteobacteria>Gammaproteobacteria>Vibrionales>Vibrionaceae	Vibrio	DNA-sulfur-modification-protein-DndB-[Vibrio-cholerae]	662
WP_104046517.1	DndB-ParB-nuclease	GCF_023277445.1	359	Bacteria>Proteobacteria>Gammaproteobacteria>Vibrionales>Vibrionaceae	Vibrio	DNA-sulfur-modification-protein-DndB-[Vibrio-jasicida]	662
WP_170890755.1	DndB-ParB-nuclease	GCF_905331605.1	364	Bacteria>Proteobacteria>Gammaproteobacteria>Vibrionales>Vibrionaceae	Vibrio	DNA-sulfur-modification-protein-DndB-[Vibrio-sp.-2-1(7)]	662
WP_102488382.1	DndB-ParB-nuclease	GCF_023161685.1	359	Bacteria>Proteobacteria>Gammaproteobacteria>Vibrionales>Vibrionaceae	Vibrio	DNA-sulfur-modification-protein-DndB-[Vibrio-splendidus]	662
WP_152468238.1	DndB-ParB-nuclease	GCF_009363355.1	359	Bacteria>Proteobacteria>Gammaproteobacteria>Vibrionales>Vibrionaceae	Vibrio	DNA-sulfur-modification-protein-DndB-[Vibrio-sp.-THAF190c]	662
WP_021713935.1	DndB-ParB-nuclease	GCF_000467185.1	559	Bacteria>Proteobacteria>Gammaproteobacteria>Vibrionales>Vibrionaceae	Vibrio_ezurae	AIPR-family-protein-[Vibrio-ezurae]	252583
WP_021713939.1	DndB-ParB-nuclease	GCF_000467185.1	359	Bacteria>Proteobacteria>Gammaproteobacteria>Vibrionales>Vibrionaceae	Vibrio_ezurae	DNA-sulfur-modification-protein-DndB-[Vibrio-ezurae]	252583
MBY7766407.1	DndB-ParB-nuclease	GCA_019852375.1	412	Bacteria>Proteobacteria>Gammaproteobacteria>Vibrionales>Vibrionaceae	Vibrio_fluvialis	DGQHR-domain-containing-protein-[Vibrio-fluvialis]	676
MBY8023704.1	DndB-ParB-nuclease	GCA_019853575.1	427	Bacteria>Proteobacteria>Gammaproteobacteria>Vibrionales>Vibrionaceae	Vibrio_fluvialis	DGQHR-domain-containing-protein-[Vibrio-fluvialis]	676
MBY8048816.1	DndB-ParB-nuclease	GCA_019853625.1	415	Bacteria>Proteobacteria>Gammaproteobacteria>Vibrionales>Vibrionaceae	Vibrio_fluvialis	DGQHR-domain-containing-protein-[Vibrio-fluvialis]	676
MBY8141814.1	DndB-ParB-nuclease	GCA_019854145.1	387	Bacteria>Proteobacteria>Gammaproteobacteria>Vibrionales>Vibrionaceae	Vibrio_fluvialis	DGQHR-domain-containing-protein-[Vibrio-fluvialis]	676
WP_171933412.1	DndB-ParB-nuclease	GCF_013181325.1	374	Bacteria>Proteobacteria>Gammaproteobacteria>Vibrionales>Vibrionaceae	Vibrio_fluvialis	DGQHR-domain-containing-protein-[Vibrio-fluvialis]	676

Accession	Annotation	GCA	Length	Phyletic.spread	Strain.name	Defline	Species.Taxid
WP_222729277.1	DndB-ParB-nuclease	GCF_021394795.1	412	Bacteria>Proteobacteria>Gammaproteobacteria>Vibrionales>Vibrionaceae	Vibrio_fluvialis	DGQHR-domain-containing-protein-[Vibrio-fluvialis]	676
MBY7766411.1	DndB-ParB-nuclease	GCA_019852375.1	359	Bacteria>Proteobacteria>Gammaproteobacteria>Vibrionales>Vibrionaceae	Vibrio_fluvialis	DNA-sulfur-modification-protein-DndB-[Vibrio-fluvialis]	676
MBY7809513.1	DndB-ParB-nuclease	GCA_019852625.1	364	Bacteria>Proteobacteria>Gammaproteobacteria>Vibrionales>Vibrionaceae	Vibrio_fluvialis	DNA-sulfur-modification-protein-DndB-[Vibrio-fluvialis]	676
MBY8023708.1	DndB-ParB-nuclease	GCA_019853575.1	364	Bacteria>Proteobacteria>Gammaproteobacteria>Vibrionales>Vibrionaceae	Vibrio_fluvialis	DNA-sulfur-modification-protein-DndB-[Vibrio-fluvialis]	676
MBY8048820.1	DndB-ParB-nuclease	GCA_019853625.1	359	Bacteria>Proteobacteria>Gammaproteobacteria>Vibrionales>Vibrionaceae	Vibrio_fluvialis	DNA-sulfur-modification-protein-DndB-[Vibrio-fluvialis]	676
MBY8111607.1	DndB-ParB-nuclease	GCA_019853965.1	364	Bacteria>Proteobacteria>Gammaproteobacteria>Vibrionales>Vibrionaceae	Vibrio_fluvialis	DNA-sulfur-modification-protein-DndB-[Vibrio-fluvialis]	676
MBY8122670.1	DndB-ParB-nuclease	GCA_019854055.1	364	Bacteria>Proteobacteria>Gammaproteobacteria>Vibrionales>Vibrionaceae	Vibrio_fluvialis	DNA-sulfur-modification-protein-DndB-[Vibrio-fluvialis]	676
MBY8141810.1	DndB-ParB-nuclease	GCA_019854145.1	359	Bacteria>Proteobacteria>Gammaproteobacteria>Vibrionales>Vibrionaceae	Vibrio_fluvialis	DNA-sulfur-modification-protein-DndB-[Vibrio-fluvialis]	676
MBY8111602.1	DndB-ParB-nuclease	GCA_019853965.1	660	Bacteria>Proteobacteria>Gammaproteobacteria>Vibrionales>Vibrionaceae	Vibrio_fluvialis	hypothetical-protein-[Vibrio-fluvialis]	676
MBY8122666.1	DndB-ParB-nuclease	GCA_019854055.1	687	Bacteria>Proteobacteria>Gammaproteobacteria>Vibrionales>Vibrionaceae	Vibrio_fluvialis	hypothetical-protein-[Vibrio-fluvialis]	676
WP_072962179.1	DndB-ParB-nuclease	GCF_024346915.1	425	Bacteria>Proteobacteria>Gammaproteobacteria>Vibrionales>Vibrionaceae	Vibrio_gazogenes	DGQHR-domain-containing-protein-[Vibrio-gazogenes]	687
WP_072962189.1	DndB-ParB-nuclease	GCF_024346915.1	359	Bacteria>Proteobacteria>Gammaproteobacteria>Vibrionales>Vibrionaceae	Vibrio_gazogenes	DNA-sulfur-modification-protein-DndB-[Vibrio-gazogenes]	687
WP_222302176.1	DndB-ParB-nuclease	GCF_019800855.1	369	Bacteria>Proteobacteria>Gammaproteobacteria>Vibrionales>Vibrionaceae	Vibrio_hangzhouensis	DNA-sulfur-modification-protein-DndB-[Vibrio-hangzhouensis]	462991
WP_222302192.1	DndB-ParB-nuclease	GCF_019800855.1	555	Bacteria>Proteobacteria>Gammaproteobacteria>Vibrionales>Vibrionaceae	Vibrio_hangzhouensis	hypothetical-protein-[Vibrio-hangzhouensis]	462991
WP_050548758.1	DndB-ParB-nuclease	GCF_000259935.1	325	Bacteria>Proteobacteria>Gammaproteobacteria>Vibrionales>Vibrionaceae	Vibrio_harveyi	DGQHR-domain-containing-protein-[Vibrio-harveyi]	669
WP_005446514.1	DndB-ParB-nuclease	GCF_000259935.1	359	Bacteria>Proteobacteria>Gammaproteobacteria>Vibrionales>Vibrionaceae	Vibrio_harveyi	DNA-sulfur-modification-protein-DndB-[Vibrio-harveyi]	669
WP_025541728.1	DndB-ParB-nuclease	GCF_000489435.1	359	Bacteria>Proteobacteria>Gammaproteobacteria>Vibrionales>Vibrionaceae	Vibrio_harveyi_group	MULTISPECIES:-DNA-sulfur-modification-protein-DndB-[Vibrio-harveyi-group]	717610
WP_053320519.1	DndB-ParB-nuclease	GCF_024531375.1	359	Bacteria>Proteobacteria>Gammaproteobacteria>Vibrionales>Vibrionaceae	Vibrio_harveyi_group	MULTISPECIES:-DNA-sulfur-modification-protein-DndB-[Vibrio-harveyi-group]	717610
WP_050548757.1	DndB-ParB-nuclease	GCF_000259935.1	69	Bacteria>Proteobacteria>Gammaproteobacteria>Vibrionales>Vibrionaceae	Vibrio_harveyi	hypothetical-protein-[Vibrio-harveyi]	669
GAM62832.1	DndB-ParB-nuclease	GCA_000830435.1	101	Bacteria>Proteobacteria>Gammaproteobacteria>Vibrionales>Vibrionaceae	Vibrio_ishigakensis	DNA-sulfur-modification-protein-dndB-[Vibrio-ishigakensis]	1481914
GAM62833.1	DndB-ParB-nuclease	GCA_000830435.1	244	Bacteria>Proteobacteria>Gammaproteobacteria>Vibrionales>Vibrionaceae	Vibrio_ishigakensis	DNA-sulfur-modification-protein-dndB-[Vibrio-ishigakensis]	1481914
GAM62952.1	DndB-ParB-nuclease	GCA_000830435.1	362	Bacteria>Proteobacteria>Gammaproteobacteria>Vibrionales>Vibrionaceae	Vibrio_ishigakensis	DNA-sulfur-modification-protein-dndB-[Vibrio-ishigakensis]	1481914
WP_117234234.1	DndB-ParB-nuclease	GCF_003415655.1	364	Bacteria>Proteobacteria>Gammaproteobacteria>Vibrionales>Vibrionaceae	Vibrio_maerlii	DNA-sulfur-modification-protein-DndB-[Vibrio-maerlii]	2231648
GAL18578.1	DndB-ParB-nuclease	GCA_000755385.1	40	Bacteria>Proteobacteria>Gammaproteobacteria>Vibrionales>Vibrionaceae	Vibrio_maritimus	DNA-sulfur-modification-protein-DndB-[Vibrio-maritimus]	990268
GAL18579.1	DndB-ParB-nuclease	GCA_000755385.1	302	Bacteria>Proteobacteria>Gammaproteobacteria>Vibrionales>Vibrionaceae	Vibrio_maritimus	DNA-sulfur-modification-protein-DndB-[Vibrio-maritimus]	990268
WP_171392385.1	DndB-ParB-nuclease	GCF_013114285.1	359	Bacteria>Proteobacteria>Gammaproteobacteria>Vibrionales>Vibrionaceae	Vibrio_mediterranei	DNA-sulfur-modification-protein-DndB-[Vibrio-mediterranei]	689
WP_162232776.1	DndB-ParB-nuclease	GCF_001402495.1	403	Bacteria>Proteobacteria>Gammaproteobacteria>Vibrionales>Vibrionaceae	Vibrio_metoecus	DGQHR-domain-containing-protein-[Vibrio-metoecus]	1481663
WP_055033594.1	DndB-ParB-nuclease	GCF_001402495.1	359	Bacteria>Proteobacteria>Gammaproteobacteria>Vibrionales>Vibrionaceae	Vibrio_metoecus	DNA-sulfur-modification-protein-DndB-[Vibrio-metoecus]	1481663
WP_217513625.1	DndB-ParB-nuclease	GCF_019092155.1	359	Bacteria>Proteobacteria>Gammaproteobacteria>Vibrionales>Vibrionaceae	Vibrio_metschnikovii	DNA-sulfur-modification-protein-DndB-[Vibrio-metschnikovii]	28172
WP_217525508.1	DndB-ParB-nuclease	GCF_019092555.1	359	Bacteria>Proteobacteria>Gammaproteobacteria>Vibrionales>Vibrionaceae	Vibrio_metschnikovii	DNA-sulfur-modification-protein-DndB-[Vibrio-metschnikovii]	28172
WP_065819644.1	DndB-ParB-nuclease	GCF_009763805.1	387	Bacteria>Proteobacteria>Gammaproteobacteria>Vibrionales>Vibrionaceae	Vibrio	MULTISPECIES:-DGQHR-domain-containing-protein-[Vibrio]	662
WP_017065615.1	DndB-ParB-nuclease	GCF_000272065.2	359	Bacteria>Proteobacteria>Gammaproteobacteria>Vibrionales>Vibrionaceae	Vibrio	MULTISPECIES:-DNA-sulfur-modification-protein-DndB-[Vibrio]	662
WP_017104390.1	DndB-ParB-nuclease	GCF_000272365.2	359	Bacteria>Proteobacteria>Gammaproteobacteria>Vibrionales>Vibrionaceae	Vibrio	MULTISPECIES:-DNA-sulfur-modification-protein-DndB-[Vibrio]	662
WP_022591085.1	DndB-ParB-nuclease	GCF_000182465.1	359	Bacteria>Proteobacteria>Gammaproteobacteria>Vibrionales>Vibrionaceae	Vibrio	MULTISPECIES:-DNA-sulfur-modification-protein-DndB-[Vibrio]	662
WP_025552312.1	DndB-ParB-nuclease	GCF_000489195.1	359	Bacteria>Proteobacteria>Gammaproteobacteria>Vibrionales>Vibrionaceae	Vibrio	MULTISPECIES:-DNA-sulfur-modification-protein-DndB-[Vibrio]	662
WP_045455115.1	DndB-ParB-nuclease	GCF_000818355.1	359	Bacteria>Proteobacteria>Gammaproteobacteria>Vibrionales>Vibrionaceae	Vibrio	MULTISPECIES:-DNA-sulfur-modification-protein-DndB-[Vibrio]	662
WP_048666154.1	DndB-ParB-nuclease	GCF_024347415.1	359	Bacteria>Proteobacteria>Gammaproteobacteria>Vibrionales>Vibrionaceae	Vibrio	MULTISPECIES:-DNA-sulfur-modification-protein-DndB-[Vibrio]	662
WP_050052916.1	DndB-ParB-nuclease	GCF_001048595.1	359	Bacteria>Proteobacteria>Gammaproteobacteria>Vibrionales>Vibrionaceae	Vibrio	MULTISPECIES:-DNA-sulfur-modification-protein-DndB-[Vibrio]	662
WP_072609042.1	DndB-ParB-nuclease	GCF_013181325.1	359	Bacteria>Proteobacteria>Gammaproteobacteria>Vibrionales>Vibrionaceae	Vibrio	MULTISPECIES:-DNA-sulfur-modification-protein-DndB-[Vibrio]	662
WP_094147719.1	DndB-ParB-nuclease	GCF_002241255.1	359	Bacteria>Proteobacteria>Gammaproteobacteria>Vibrionales>Vibrionaceae	Vibrio	MULTISPECIES:-DNA-sulfur-modification-protein-DndB-[Vibrio]	662
WP_113628966.1	DndB-ParB-nuclease	GCF_009665475.1	364	Bacteria>Proteobacteria>Gammaproteobacteria>Vibrionales>Vibrionaceae	Vibrio	MULTISPECIES:-DNA-sulfur-modification-protein-DndB-[Vibrio]	662
WP_119467557.1	DndB-ParB-nuclease	GCF_003585405.1	359	Bacteria>Proteobacteria>Gammaproteobacteria>Vibrionales>Vibrionaceae	Vibrio	MULTISPECIES:-DNA-sulfur-modification-protein-DndB-[Vibrio]	662
WP_130249896.1	DndB-ParB-nuclease	GCF_020252105.1	364	Bacteria>Proteobacteria>Gammaproteobacteria>Vibrionales>Vibrionaceae	Vibrio	MULTISPECIES:-DNA-sulfur-modification-protein-DndB-[Vibrio]	662
WP_202933119.1	DndB-ParB-nuclease	GCF_014905705.1	412	Bacteria>Proteobacteria>Gammaproteobacteria>Vibrionales>Vibrionaceae	Vibrio_navarrensis	DGQHR-domain-containing-protein-[Vibrio-navarrensis]	29495
WP_193167171.1	DndB-ParB-nuclease	GCF_014905775.1	359	Bacteria>Proteobacteria>Gammaproteobacteria>Vibrionales>Vibrionaceae	Vibrio_navarrensis	DNA-sulfur-modification-protein-DndB-[Vibrio-navarrensis]	29495
WP_202933299.1	DndB-ParB-nuclease	GCF_014905705.1	359	Bacteria>Proteobacteria>Gammaproteobacteria>Vibrionales>Vibrionaceae	Vibrio_navarrensis	DNA-sulfur-modification-protein-DndB-[Vibrio-navarrensis]	29495
WP_206369014.1	DndB-ParB-nuclease	GCF_022603415.1	362	Bacteria>Proteobacteria>Gammaproteobacteria>Vibrionales>Vibrionaceae	Vibrio_neptunius	DNA-sulfur-modification-protein-DndB-[Vibrio-neptunius]	170651
WP_206369009.1	DndB-ParB-nuclease	GCF_022603415.1	560	Bacteria>Proteobacteria>Gammaproteobacteria>Vibrionales>Vibrionaceae	Vibrio_neptunius	hypothetical-protein-[Vibrio-neptunius]	170651
WP_022602079.1	DndB-ParB-nuclease	GCF_900005135.1	359	Bacteria>Proteobacteria>Gammaproteobacteria>Vibrionales>Vibrionaceae	Vibrio_nigripulchritudo	DNA-sulfur-modification-protein-DndB-[Vibrio-nigripulchritudo]	28173
WP_077313475.1	DndB-ParB-nuclease	GCF_024346995.1	358	Bacteria>Proteobacteria>Gammaproteobacteria>Vibrionales>Vibrionaceae	Vibrio_palustris	DNA-sulfur-modification-protein-DndB-[Vibrio-palustris]	1918946
WP_151148863.1	DndB-ParB-nuclease	GCF_009938205.1	382	Bacteria>Proteobacteria>Gammaproteobacteria>Vibrionales>Vibrionaceae	Vibrio_panuliri	DGQHR-domain-containing-protein-[Vibrio-panuliri]	1381081
WP_075713419.1	DndB-ParB-nuclease	GCF_009938205.1	359	Bacteria>Proteobacteria>Gammaproteobacteria>Vibrionales>Vibrionaceae	Vibrio_panuliri	DNA-sulfur-modification-protein-DndB-[Vibrio-panuliri]	1381081
EGQ8902400.1	DndB-ParB-nuclease	GCA_015781875.1	390	Bacteria>Proteobacteria>Gammaproteobacteria>Vibrionales>Vibrionaceae	Vibrio_parahaemolyticus	DGQHR-domain-containing-protein-[Vibrio-parahaemolyticus]	670
EGQ8955959.1	DndB-ParB-nuclease	GCA_015781615.1	374	Bacteria>Proteobacteria>Gammaproteobacteria>Vibrionales>Vibrionaceae	Vibrio_parahaemolyticus	DGQHR-domain-containing-protein-[Vibrio-parahaemolyticus]	670
EGR2182580.1	DndB-ParB-nuclease	GCA_015803155.1	386	Bacteria>Proteobacteria>Gammaproteobacteria>Vibrionales>Vibrionaceae	Vibrio_parahaemolyticus	DGQHR-domain-containing-protein-[Vibrio-parahaemolyticus]	670
EHH1037077.1	DndB-ParB-nuclease	GCA_017603215.1	387	Bacteria>Proteobacteria>Gammaproteobacteria>Vibrionales>Vibrionaceae	Vibrio_parahaemolyticus	DGQHR-domain-containing-protein-[Vibrio-parahaemolyticus]	670
EHJ9984790.1	DndB-ParB-nuclease	GCA_017968045.1	408	Bacteria>Proteobacteria>Gammaproteobacteria>Vibrionales>Vibrionaceae	Vibrio_parahaemolyticus	DGQHR-domain-containing-protein-[Vibrio-parahaemolyticus]	670
WP_053320516.1	DndB-ParB-nuclease	GCF_024746295.1	387	Bacteria>Proteobacteria>Gammaproteobacteria>Vibrionales>Vibrionaceae	Vibrio_parahaemolyticus	DGQHR-domain-containing-protein-[Vibrio-parahaemolyticus]	670
WP_053320918.1	DndB-ParB-nuclease	GCF_001267555.1	409	Bacteria>Proteobacteria>Gammaproteobacteria>Vibrionales>Vibrionaceae	Vibrio_parahaemolyticus	DGQHR-domain-containing-protein-[Vibrio-parahaemolyticus]	670
WP_140078362.1	DndB-ParB-nuclease	GCF_024744395.1	412	Bacteria>Proteobacteria>Gammaproteobacteria>Vibrionales>Vibrionaceae	Vibrio_parahaemolyticus	DGQHR-domain-containing-protein-[Vibrio-parahaemolyticus]	670
WP_140214735.1	DndB-ParB-nuclease	GCF_006368565.1	425	Bacteria>Proteobacteria>Gammaproteobacteria>Vibrionales>Vibrionaceae	Vibrio_parahaemolyticus	DGQHR-domain-containing-protein-[Vibrio-parahaemolyticus]	670
WP_140266332.1	DndB-ParB-nuclease	GCF_006372635.1	386	Bacteria>Proteobacteria>Gammaproteobacteria>Vibrionales>Vibrionaceae	Vibrio_parahaemolyticus	DGQHR-domain-containing-protein-[Vibrio-parahaemolyticus]	670
WP_140289249.1	DndB-ParB-nuclease	GCF_006373635.1	386	Bacteria>Proteobacteria>Gammaproteobacteria>Vibrionales>Vibrionaceae	Vibrio_parahaemolyticus	DGQHR-domain-containing-protein-[Vibrio-parahaemolyticus]	670
WP_069201733.1	DndB-ParB-nuclease	GCF_001433415.1	401	Bacteria>Proteobacteria>Gammaproteobacteria>Vibrionales>Vibrionaceae	Vibrio_parahaemolyticus	DNA-phosphorothioation-associated-DGQHR-protein-1-[Vibrio-parahaemolyticus]	670
EGQ7686065.1	DndB-ParB-nuclease	GCA_015744865.1	364	Bacteria>Proteobacteria>Gammaproteobacteria>Vibrionales>Vibrionaceae	Vibrio_parahaemolyticus	DNA-sulfur-modification-protein-DndB-[Vibrio-parahaemolyticus]	670
EGQ7768661.1	DndB-ParB-nuclease	GCA_015745765.1	359	Bacteria>Proteobacteria>Gammaproteobacteria>Vibrionales>Vibrionaceae	Vibrio_parahaemolyticus	DNA-sulfur-modification-protein-DndB-[Vibrio-parahaemolyticus]	670
EGQ7839264.1	DndB-ParB-nuclease	GCA_015746045.1	359	Bacteria>Proteobacteria>Gammaproteobacteria>Vibrionales>Vibrionaceae	Vibrio_parahaemolyticus	DNA-sulfur-modification-protein-DndB-[Vibrio-parahaemolyticus]	670
EGQ8902404.1	DndB-ParB-nuclease	GCA_015781875.1	359	Bacteria>Proteobacteria>Gammaproteobacteria>Vibrionales>Vibrionaceae	Vibrio_parahaemolyticus	DNA-sulfur-modification-protein-DndB-[Vibrio-parahaemolyticus]	670
EGQ8955955.1	DndB-ParB-nuclease	GCA_015781615.1	359	Bacteria>Proteobacteria>Gammaproteobacteria>Vibrionales>Vibrionaceae	Vibrio_parahaemolyticus	DNA-sulfur-modification-protein-DndB-[Vibrio-parahaemolyticus]	670
EGR1479647.1	DndB-ParB-nuclease	GCA_015799375.1	359	Bacteria>Proteobacteria>Gammaproteobacteria>Vibrionales>Vibrionaceae	Vibrio_parahaemolyticus	DNA-sulfur-modification-protein-DndB-[Vibrio-parahaemolyticus]	670
EGR2182584.1	DndB-ParB-nuclease	GCA_015803155.1	359	Bacteria>Proteobacteria>Gammaproteobacteria>Vibrionales>Vibrionaceae	Vibrio_parahaemolyticus	DNA-sulfur-modification-protein-DndB-[Vibrio-parahaemolyticus]	670
EGR2691911.1	DndB-ParB-nuclease	GCA_015804235.1	364	Bacteria>Proteobacteria>Gammaproteobacteria>Vibrionales>Vibrionaceae	Vibrio_parahaemolyticus	DNA-sulfur-modification-protein-DndB-[Vibrio-parahaemolyticus]	670
EGR3110185.1	DndB-ParB-nuclease	GCA_015806675.1	359	Bacteria>Proteobacteria>Gammaproteobacteria>Vibrionales>Vibrionaceae	Vibrio_parahaemolyticus	DNA-sulfur-modification-protein-DndB-[Vibrio-parahaemolyticus]	670
EGR3264933.1	DndB-ParB-nuclease	GCA_015806875.1	359	Bacteria>Proteobacteria>Gammaproteobacteria>Vibrionales>Vibrionaceae	Vibrio_parahaemolyticus	DNA-sulfur-modification-protein-DndB-[Vibrio-parahaemolyticus]	670
EHH1037073.1	DndB-ParB-nuclease	GCA_017603215.1	359	Bacteria>Proteobacteria>Gammaproteobacteria>Vibrionales>Vibrionaceae	Vibrio_parahaemolyticus	DNA-sulfur-modification-protein-DndB-[Vibrio-parahaemolyticus]	670
EHJ9984794.1	DndB-ParB-nuclease	GCA_017968045.1	359	Bacteria>Proteobacteria>Gammaproteobacteria>Vibrionales>Vibrionaceae	Vibrio_parahaemolyticus	DNA-sulfur-modification-protein-DndB-[Vibrio-parahaemolyticus]	670
EHR6002935.1	DndB-ParB-nuclease	GCA_019062905.1	359	Bacteria>Proteobacteria>Gammaproteobacteria>Vibrionales>Vibrionaceae	Vibrio_parahaemolyticus	DNA-sulfur-modification-protein-DndB-[Vibrio-parahaemolyticus]	670
HAS7007405.1	DndB-ParB-nuclease	GCA_015782475.1	359	Bacteria>Proteobacteria>Gammaproteobacteria>Vibrionales>Vibrionaceae	Vibrio_parahaemolyticus	DNA-sulfur-modification-protein-DndB-[Vibrio-parahaemolyticus]	670
MBE3823317.1	DndB-ParB-nuclease	GCA_014918955.1	359	Bacteria>Proteobacteria>Gammaproteobacteria>Vibrionales>Vibrionaceae	Vibrio_parahaemolyticus	DNA-sulfur-modification-protein-DndB-[Vibrio-parahaemolyticus]	670
MBE5147680.1	DndB-ParB-nuclease	GCA_014964085.1	364	Bacteria>Proteobacteria>Gammaproteobacteria>Vibrionales>Vibrionaceae	Vibrio_parahaemolyticus	DNA-sulfur-modification-protein-DndB-[Vibrio-parahaemolyticus]	670
WP_021453164.1	DndB-ParB-nuclease	GCF_000454245.1	359	Bacteria>Proteobacteria>Gammaproteobacteria>Vibrionales>Vibrionaceae	Vibrio_parahaemolyticus	DNA-sulfur-modification-protein-DndB-[Vibrio-parahaemolyticus]	670
WP_053320922.1	DndB-ParB-nuclease	GCF_001267555.1	359	Bacteria>Proteobacteria>Gammaproteobacteria>Vibrionales>Vibrionaceae	Vibrio_parahaemolyticus	DNA-sulfur-modification-protein-DndB-[Vibrio-parahaemolyticus]	670
WP_069522915.1	DndB-ParB-nuclease	GCF_001727925.1	364	Bacteria>Proteobacteria>Gammaproteobacteria>Vibrionales>Vibrionaceae	Vibrio_parahaemolyticus	DNA-sulfur-modification-protein-DndB-[Vibrio-parahaemolyticus]	670
WP_086484901.1	DndB-ParB-nuclease	GCF_002150465.1	364	Bacteria>Proteobacteria>Gammaproteobacteria>Vibrionales>Vibrionaceae	Vibrio_parahaemolyticus	DNA-sulfur-modification-protein-DndB-[Vibrio-parahaemolyticus]	670
WP_140258798.1	DndB-ParB-nuclease	GCF_006372235.1	359	Bacteria>Proteobacteria>Gammaproteobacteria>Vibrionales>Vibrionaceae	Vibrio_parahaemolyticus	DNA-sulfur-modification-protein-DndB-[Vibrio-parahaemolyticus]	670
WP_140289256.1	DndB-ParB-nuclease	GCF_006373635.1	359	Bacteria>Proteobacteria>Gammaproteobacteria>Vibrionales>Vibrionaceae	Vibrio_parahaemolyticus	DNA-sulfur-modification-protein-DndB-[Vibrio-parahaemolyticus]	670
EGR3110181.1	DndB-ParB-nuclease	GCA_015806675.1	387	Bacteria>Proteobacteria>Gammaproteobacteria>Vibrionales>Vibrionaceae	Vibrio_parahaemolyticus	hypothetical-protein-[Vibrio-parahaemolyticus]	670
EGR3264938.1	DndB-ParB-nuclease	GCA_015806875.1	695	Bacteria>Proteobacteria>Gammaproteobacteria>Vibrionales>Vibrionaceae	Vibrio_parahaemolyticus	hypothetical-protein-[Vibrio-parahaemolyticus]	670
EHR6002930.1	DndB-ParB-nuclease	GCA_019062905.1	695	Bacteria>Proteobacteria>Gammaproteobacteria>Vibrionales>Vibrionaceae	Vibrio_parahaemolyticus	hypothetical-protein-[Vibrio-parahaemolyticus]	670
MBE5147675.1	DndB-ParB-nuclease	GCA_014964085.1	237	Bacteria>Proteobacteria>Gammaproteobacteria>Vibrionales>Vibrionaceae	Vibrio_parahaemolyticus	hypothetical-protein-[Vibrio-parahaemolyticus]	670
WP_069522920.1	DndB-ParB-nuclease	GCF_001727925.1	660	Bacteria>Proteobacteria>Gammaproteobacteria>Vibrionales>Vibrionaceae	Vibrio_parahaemolyticus	hypothetical-protein-[Vibrio-parahaemolyticus]	670
HAS6739050.1	DndB-ParB-nuclease	GCA_015779935.1	359	Bacteria>Proteobacteria>Gammaproteobacteria>Vibrionales>Vibrionaceae	Vibrio_parahaemolyticus	TPA:-DNA-sulfur-modification-protein-DndB-[Vibrio-parahaemolyticus]	670
HBC3450716.1	DndB-ParB-nuclease	GCA_018119085.1	359	Bacteria>Proteobacteria>Gammaproteobacteria>Vibrionales>Vibrionaceae	Vibrio_parahaemolyticus	TPA:-DNA-sulfur-modification-protein-DndB-[Vibrio-parahaemolyticus]	670
HBC3588065.1	DndB-ParB-nuclease	GCA_018122395.1	359	Bacteria>Proteobacteria>Gammaproteobacteria>Vibrionales>Vibrionaceae	Vibrio_parahaemolyticus	TPA:-DNA-sulfur-modification-protein-DndB-[Vibrio-parahaemolyticus]	670
HBN6302088.1	DndB-ParB-nuclease	GCA_021553655.1	359	Bacteria>Proteobacteria>Gammaproteobacteria>Vibrionales>Vibrionaceae	Vibrio_parahaemolyticus	TPA:-DNA-sulfur-modification-protein-DndB-[Vibrio-parahaemolyticus]	670
WP_171361137.1	DndB-ParB-nuclease	GCF_013114615.1	359	Bacteria>Proteobacteria>Gammaproteobacteria>Vibrionales>Vibrionaceae	Vibrio_pectenicida	DNA-sulfur-modification-protein-DndB-[Vibrio-pectenicida]	62763
WP_138955735.1	DndB-ParB-nuclease	GCF_006125005.1	359	Bacteria>Proteobacteria>Gammaproteobacteria>Vibrionales>Vibrionaceae	Vibrio_rotiferianus	DNA-sulfur-modification-protein-DndB-[Vibrio-rotiferianus]	190895
WP_077336581.1	DndB-ParB-nuclease	GCF_024347115.1	359	Bacteria>Proteobacteria>Gammaproteobacteria>Vibrionales>Vibrionaceae	Vibrio_ruber	DNA-sulfur-modification-protein-DndB-[Vibrio-ruber]	184755
WP_077336576.1	DndB-ParB-nuclease	GCF_024347115.1	565	Bacteria>Proteobacteria>Gammaproteobacteria>Vibrionales>Vibrionaceae	Vibrio_ruber	hypothetical-protein-[Vibrio-ruber]	184755
WP_102425883.1	DndB-ParB-nuclease	GCF_002876325.1	359	Bacteria>Proteobacteria>Gammaproteobacteria>Vibrionales>Vibrionaceae	Vibrio_sp._10N.261.52.A1	DNA-sulfur-modification-protein-DndB-[Vibrio-sp.-10N.261.52.A1]	1880849
WP_170890747.1	DndB-ParB-nuclease	GCF_013048495.1	724	Bacteria>Proteobacteria>Gammaproteobacteria>Vibrionales>Vibrionaceae	Vibrio_sp._2-1(7)	hypothetical-protein-[Vibrio-sp.-2-1(7)]	2591011
WP_171762084.1	DndB-ParB-nuclease	GCF_013114585.1	393	Bacteria>Proteobacteria>Gammaproteobacteria>Vibrionales>Vibrionaceae	Vibrio_sp._99K-1	DGQHR-domain-containing-protein-[Vibrio-sp.-99K-1]	2607603
WP_171762080.1	DndB-ParB-nuclease	GCF_013114585.1	359	Bacteria>Proteobacteria>Gammaproteobacteria>Vibrionales>Vibrionaceae	Vibrio_sp._99K-1	DNA-sulfur-modification-protein-DndB-[Vibrio-sp.-99K-1]	2607603
WP_194435408.1	DndB-ParB-nuclease	GCF_015278125.1	359	Bacteria>Proteobacteria>Gammaproteobacteria>Vibrionales>Vibrionaceae	Vibrio_sp._A621	DNA-sulfur-modification-protein-DndB-[Vibrio-sp.-A621]	2783614
WP_074374006.1	DndB-ParB-nuclease	GCF_024347135.1	408	Bacteria>Proteobacteria>Gammaproteobacteria>Vibrionales>Vibrionaceae	Vibrio_spartinae	DGQHR-domain-containing-protein-[Vibrio-spartinae]	1918945
WP_182288255.1	DndB-ParB-nuclease	GCF_014083925.1	359	Bacteria>Proteobacteria>Gammaproteobacteria>Vibrionales>Vibrionaceae	Vibrio_spartinae	DNA-sulfur-modification-protein-DndB-[Vibrio-spartinae]	1918945
WP_182008420.1	DndB-ParB-nuclease	GCF_903986855.1	369	Bacteria>Proteobacteria>Gammaproteobacteria>Vibrionales>Vibrionaceae	Vibrio_sp._B1FLJ16	DNA-sulfur-modification-protein-DndB-[Vibrio-sp.-B1FLJ16]	2751178
WP_226421864.1	DndB-ParB-nuclease	GCF_020515785.1	412	Bacteria>Proteobacteria>Gammaproteobacteria>Vibrionales>Vibrionaceae	Vibrio_sp._E14	DGQHR-domain-containing-protein-[Vibrio-sp.-E14]	2849869
WP_226421868.1	DndB-ParB-nuclease	GCF_020515785.1	358	Bacteria>Proteobacteria>Gammaproteobacteria>Vibrionales>Vibrionaceae	Vibrio_sp._E14	DNA-sulfur-modification-protein-DndB-[Vibrio-sp.-E14]	2849869
WP_136484167.1	DndB-ParB-nuclease	GCF_004804295.1	364	Bacteria>Proteobacteria>Gammaproteobacteria>Vibrionales>Vibrionaceae	Vibrio_sp._H11	DNA-sulfur-modification-protein-DndB-[Vibrio-sp.-H11]	2565928
WP_050712511.1	DndB-ParB-nuclease	GCF_001262155.1	387	Bacteria>Proteobacteria>Gammaproteobacteria>Vibrionales>Vibrionaceae	Vibrio_sp._J2-26	DGQHR-domain-containing-protein-[Vibrio-sp.-J2-26]	1507978
WP_050712507.1	DndB-ParB-nuclease	GCF_001262155.1	359	Bacteria>Proteobacteria>Gammaproteobacteria>Vibrionales>Vibrionaceae	Vibrio_sp._J2-26	DNA-sulfur-modification-protein-DndB-[Vibrio-sp.-J2-26]	1507978
WP_162092652.1	DndB-ParB-nuclease	GCF_010092875.1	359	Bacteria>Proteobacteria>Gammaproteobacteria>Vibrionales>Vibrionaceae	Vibrio_sp._LB10LO1	DNA-sulfur-modification-protein-DndB-[Vibrio-sp.-LB10LO1]	2711207
MCC4859884.1	DndB-ParB-nuclease	GCA_020806315.1	403	Bacteria>Proteobacteria>Gammaproteobacteria>Vibrionales>Vibrionaceae	Vibrio_splendidus	DGQHR-domain-containing-protein-[Vibrio-splendidus]	29497
WP_102488385.1	DndB-ParB-nuclease	GCF_002875305.1	386	Bacteria>Proteobacteria>Gammaproteobacteria>Vibrionales>Vibrionaceae	Vibrio_splendidus	DGQHR-domain-containing-protein-[Vibrio-splendidus]	29497
WP_108189767.1	DndB-ParB-nuclease	GCF_003050385.1	425	Bacteria>Proteobacteria>Gammaproteobacteria>Vibrionales>Vibrionaceae	Vibrio_splendidus	DGQHR-domain-containing-protein-[Vibrio-splendidus]	29497
MCC4859880.1	DndB-ParB-nuclease	GCA_020806315.1	359	Bacteria>Proteobacteria>Gammaproteobacteria>Vibrionales>Vibrionaceae	Vibrio_splendidus	DNA-sulfur-modification-protein-DndB-[Vibrio-splendidus]	29497
WP_017074154.1	DndB-ParB-nuclease	GCF_000272225.2	362	Bacteria>Proteobacteria>Gammaproteobacteria>Vibrionales>Vibrionaceae	Vibrio_splendidus	DNA-sulfur-modification-protein-DndB-[Vibrio-splendidus]	29497
WP_108189763.1	DndB-ParB-nuclease	GCF_003050385.1	359	Bacteria>Proteobacteria>Gammaproteobacteria>Vibrionales>Vibrionaceae	Vibrio_splendidus	DNA-sulfur-modification-protein-DndB-[Vibrio-splendidus]	29497
WP_215919698.1	DndB-ParB-nuclease	GCF_018860405.1	673	Bacteria>Proteobacteria>Gammaproteobacteria>Vibrionales>Vibrionaceae	Vibrio_splendidus	hypothetical-protein-[Vibrio-splendidus]	29497
WP_059120162.1	DndB-ParB-nuclease	GCF_001506075.1	359	Bacteria>Proteobacteria>Gammaproteobacteria>Vibrionales>Vibrionaceae	Vibrio_sp._MEBiC08052	DNA-sulfur-modification-protein-DndB-[Vibrio-sp.-MEBiC08052]	1761910
WP_059120157.1	DndB-ParB-nuclease	GCF_001506075.1	554	Bacteria>Proteobacteria>Gammaproteobacteria>Vibrionales>Vibrionaceae	Vibrio_sp._MEBiC08052	hypothetical-protein-[Vibrio-sp.-MEBiC08052]	1761910
WP_193841245.1	DndB-ParB-nuclease	GCF_015163615.1	643	Bacteria>Proteobacteria>Gammaproteobacteria>Vibrionales>Vibrionaceae	Vibrio_sp._OPT18	hypothetical-protein-[Vibrio-sp.-OPT18]	2778641
WP_182796904.1	DndB-ParB-nuclease	GCF_014164465.1	359	Bacteria>Proteobacteria>Gammaproteobacteria>Vibrionales>Vibrionaceae	Vibrio_sp._SG41-7	DNA-sulfur-modification-protein-DndB-[Vibrio-sp.-SG41-7]	2760973
WP_105977285.1	DndB-ParB-nuclease	GCF_002995435.1	428	Bacteria>Proteobacteria>Gammaproteobacteria>Vibrionales>Vibrionaceae	Vibrio_sp._V01_P9A10T6	DGQHR-domain-containing-protein-[Vibrio-sp.-V01_P9A10T6]	2116368

Accession	Annotation	GCA	Length	Phyletic.spread	Strain.name	Defline	Species.Taxid
WP_105977281.1	DndB-ParB-nuclease	GCF_002995435.1	364	Bacteria>Proteobacteria>Gammaproteobacteria>Vibrionales>Vibrionaceae	Vibrio_sp._V01_P9A10T6	DNA-sulfur-modification-protein-DndB-[Vibrio-sp.-V01_P9A10T6]	2116368
WP_254918032.1	DndB-ParB-nuclease	GCF_002241165.1	360	Bacteria>Proteobacteria>Gammaproteobacteria>Vibrionales>Vibrionaceae	Vibrio_sp._V07_P2A8T137	DNA-sulfur-modification-protein-DndB,-partial-[Vibrio-sp.-V07_P2A8T137]	1938662
WP_166538045.1	DndB-ParB-nuclease	GCF_009905095.1	353	Bacteria>Proteobacteria>Gammaproteobacteria>Vibrionales>Vibrionaceae	Vibrio_sp._V22_P2S10T140	DNA-sulfur-modification-protein-DndB,-partial-[Vibrio-sp.-V22_P2S10T140]	1938674
WP_068714510.1	DndB-ParB-nuclease	GCF_001547935.1	365	Bacteria>Proteobacteria>Gammaproteobacteria>Vibrionales>Vibrionaceae	Vibrio_tritonius	DNA-sulfur-modification-protein-DndB-[Vibrio-tritonius]	1435069
WP_068714520.1	DndB-ParB-nuclease	GCF_001547935.1	557	Bacteria>Proteobacteria>Gammaproteobacteria>Vibrionales>Vibrionaceae	Vibrio_tritonius	hypothetical-protein-[Vibrio-tritonius]	1435069
WP_124937919.1	DndB-ParB-nuclease	GCF_003856525.1	358	Bacteria>Proteobacteria>Gammaproteobacteria>Vibrionales>Vibrionaceae	Vibrio_viridaestus	DNA-sulfur-modification-protein-DndB-[Vibrio-viridaestus]	2487322
WP_130196831.1	DndB-ParB-nuclease	GCF_004215035.1	570	Bacteria>Proteobacteria>Gammaproteobacteria>Vibrionales>Vibrionaceae	Vibrio_vulnificus	AIPR-family-protein-[Vibrio-vulnificus]	672
WP_130349860.1	DndB-ParB-nuclease	GCF_004215105.1	562	Bacteria>Proteobacteria>Gammaproteobacteria>Vibrionales>Vibrionaceae	Vibrio_vulnificus	AIPR-family-protein-[Vibrio-vulnificus]	672
EGR7976418.1	DndB-ParB-nuclease	GCA_015838355.1	425	Bacteria>Proteobacteria>Gammaproteobacteria>Vibrionales>Vibrionaceae	Vibrio_vulnificus	DGQHR-domain-containing-protein-[Vibrio-vulnificus]	672
EHK2771662.1	DndB-ParB-nuclease	GCA_018015775.1	386	Bacteria>Proteobacteria>Gammaproteobacteria>Vibrionales>Vibrionaceae	Vibrio_vulnificus	DGQHR-domain-containing-protein-[Vibrio-vulnificus]	672
EHU4931615.1	DndB-ParB-nuclease	GCA_019290835.1	393	Bacteria>Proteobacteria>Gammaproteobacteria>Vibrionales>Vibrionaceae	Vibrio_vulnificus	DGQHR-domain-containing-protein-[Vibrio-vulnificus]	672
HAS6174911.1	DndB-ParB-nuclease	GCA_015776995.1	411	Bacteria>Proteobacteria>Gammaproteobacteria>Vibrionales>Vibrionaceae	Vibrio_vulnificus	DGQHR-domain-containing-protein-[Vibrio-vulnificus]	672
HAS6220676.1	DndB-ParB-nuclease	GCA_015777135.1	388	Bacteria>Proteobacteria>Gammaproteobacteria>Vibrionales>Vibrionaceae	Vibrio_vulnificus	DGQHR-domain-containing-protein-[Vibrio-vulnificus]	672
HAS8195580.1	DndB-ParB-nuclease	GCA_015819905.1	407	Bacteria>Proteobacteria>Gammaproteobacteria>Vibrionales>Vibrionaceae	Vibrio_vulnificus	DGQHR-domain-containing-protein-[Vibrio-vulnificus]	672
HAS8342869.1	DndB-ParB-nuclease	GCA_015820575.1	393	Bacteria>Proteobacteria>Gammaproteobacteria>Vibrionales>Vibrionaceae	Vibrio_vulnificus	DGQHR-domain-containing-protein-[Vibrio-vulnificus]	672
HAS8378261.1	DndB-ParB-nuclease	GCA_015821155.1	427	Bacteria>Proteobacteria>Gammaproteobacteria>Vibrionales>Vibrionaceae	Vibrio_vulnificus	DGQHR-domain-containing-protein-[Vibrio-vulnificus]	672
WP_052687805.1	DndB-ParB-nuclease	GCF_020169615.1	392	Bacteria>Proteobacteria>Gammaproteobacteria>Vibrionales>Vibrionaceae	Vibrio_vulnificus	DGQHR-domain-containing-protein-[Vibrio-vulnificus]	672
WP_072609046.1	DndB-ParB-nuclease	GCF_001890545.1	396	Bacteria>Proteobacteria>Gammaproteobacteria>Vibrionales>Vibrionaceae	Vibrio_vulnificus	DGQHR-domain-containing-protein-[Vibrio-vulnificus]	672
WP_130240364.1	DndB-ParB-nuclease	GCF_024745375.1	405	Bacteria>Proteobacteria>Gammaproteobacteria>Vibrionales>Vibrionaceae	Vibrio_vulnificus	DGQHR-domain-containing-protein-[Vibrio-vulnificus]	672
WP_130241238.1	DndB-ParB-nuclease	GCF_021023015.1	387	Bacteria>Proteobacteria>Gammaproteobacteria>Vibrionales>Vibrionaceae	Vibrio_vulnificus	DGQHR-domain-containing-protein-[Vibrio-vulnificus]	672
WP_154186258.1	DndB-ParB-nuclease	GCF_009665455.1	386	Bacteria>Proteobacteria>Gammaproteobacteria>Vibrionales>Vibrionaceae	Vibrio_vulnificus	DGQHR-domain-containing-protein-[Vibrio-vulnificus]	672
WP_206627811.1	DndB-ParB-nuclease	GCF_017290195.1	396	Bacteria>Proteobacteria>Gammaproteobacteria>Vibrionales>Vibrionaceae	Vibrio_vulnificus	DGQHR-domain-containing-protein-[Vibrio-vulnificus]	672
AXX59371.1	DndB-ParB-nuclease	GCA_003522555.1	359	Bacteria>Proteobacteria>Gammaproteobacteria>Vibrionales>Vibrionaceae	Vibrio_vulnificus	DNA-sulfur-modification-protein-DndB-[Vibrio-vulnificus]	672
EGQ7757801.1	DndB-ParB-nuclease	GCA_015745685.1	359	Bacteria>Proteobacteria>Gammaproteobacteria>Vibrionales>Vibrionaceae	Vibrio_vulnificus	DNA-sulfur-modification-protein-DndB-[Vibrio-vulnificus]	672
EGQ7936424.1	DndB-ParB-nuclease	GCA_015960105.1	359	Bacteria>Proteobacteria>Gammaproteobacteria>Vibrionales>Vibrionaceae	Vibrio_vulnificus	DNA-sulfur-modification-protein-DndB-[Vibrio-vulnificus]	672
EGQ7982861.1	DndB-ParB-nuclease	GCA_015960135.1	364	Bacteria>Proteobacteria>Gammaproteobacteria>Vibrionales>Vibrionaceae	Vibrio_vulnificus	DNA-sulfur-modification-protein-DndB-[Vibrio-vulnificus]	672
EGR0086540.1	DndB-ParB-nuclease	GCA_015787575.1	359	Bacteria>Proteobacteria>Gammaproteobacteria>Vibrionales>Vibrionaceae	Vibrio_vulnificus	DNA-sulfur-modification-protein-DndB-[Vibrio-vulnificus]	672
EGR7976414.1	DndB-ParB-nuclease	GCA_015838355.1	359	Bacteria>Proteobacteria>Gammaproteobacteria>Vibrionales>Vibrionaceae	Vibrio_vulnificus	DNA-sulfur-modification-protein-DndB-[Vibrio-vulnificus]	672
EHD2233392.1	DndB-ParB-nuclease	GCA_017126245.1	359	Bacteria>Proteobacteria>Gammaproteobacteria>Vibrionales>Vibrionaceae	Vibrio_vulnificus	DNA-sulfur-modification-protein-DndB-[Vibrio-vulnificus]	672
EHK2771666.1	DndB-ParB-nuclease	GCA_018015775.1	359	Bacteria>Proteobacteria>Gammaproteobacteria>Vibrionales>Vibrionaceae	Vibrio_vulnificus	DNA-sulfur-modification-protein-DndB-[Vibrio-vulnificus]	672
EHK9000471.1	DndB-ParB-nuclease	GCA_018080885.1	359	Bacteria>Proteobacteria>Gammaproteobacteria>Vibrionales>Vibrionaceae	Vibrio_vulnificus	DNA-sulfur-modification-protein-DndB-[Vibrio-vulnificus]	672
EHU4931611.1	DndB-ParB-nuclease	GCA_019290835.1	359	Bacteria>Proteobacteria>Gammaproteobacteria>Vibrionales>Vibrionaceae	Vibrio_vulnificus	DNA-sulfur-modification-protein-DndB-[Vibrio-vulnificus]	672
HAS6174915.1	DndB-ParB-nuclease	GCA_015776995.1	359	Bacteria>Proteobacteria>Gammaproteobacteria>Vibrionales>Vibrionaceae	Vibrio_vulnificus	DNA-sulfur-modification-protein-DndB-[Vibrio-vulnificus]	672
HAS6220672.1	DndB-ParB-nuclease	GCA_015777135.1	359	Bacteria>Proteobacteria>Gammaproteobacteria>Vibrionales>Vibrionaceae	Vibrio_vulnificus	DNA-sulfur-modification-protein-DndB-[Vibrio-vulnificus]	672
HAS8195576.1	DndB-ParB-nuclease	GCA_015819905.1	359	Bacteria>Proteobacteria>Gammaproteobacteria>Vibrionales>Vibrionaceae	Vibrio_vulnificus	DNA-sulfur-modification-protein-DndB-[Vibrio-vulnificus]	672
HAS8342865.1	DndB-ParB-nuclease	GCA_015820575.1	359	Bacteria>Proteobacteria>Gammaproteobacteria>Vibrionales>Vibrionaceae	Vibrio_vulnificus	DNA-sulfur-modification-protein-DndB-[Vibrio-vulnificus]	672
HAS8378257.1	DndB-ParB-nuclease	GCA_015821155.1	364	Bacteria>Proteobacteria>Gammaproteobacteria>Vibrionales>Vibrionaceae	Vibrio_vulnificus	DNA-sulfur-modification-protein-DndB-[Vibrio-vulnificus]	672
HAS8614604.1	DndB-ParB-nuclease	GCA_015821865.1	359	Bacteria>Proteobacteria>Gammaproteobacteria>Vibrionales>Vibrionaceae	Vibrio_vulnificus	DNA-sulfur-modification-protein-DndB-[Vibrio-vulnificus]	672
MCA3964058.1	DndB-ParB-nuclease	GCA_020252125.1	364	Bacteria>Proteobacteria>Gammaproteobacteria>Vibrionales>Vibrionaceae	Vibrio_vulnificus	DNA-sulfur-modification-protein-DndB-[Vibrio-vulnificus]	672
NTJ40906.1	DndB-ParB-nuclease	GCA_013319355.1	364	Bacteria>Proteobacteria>Gammaproteobacteria>Vibrionales>Vibrionaceae	Vibrio_vulnificus	DNA-sulfur-modification-protein-DndB-[Vibrio-vulnificus]	672
POB66752.1	DndB-ParB-nuclease	GCA_002903375.1	364	Bacteria>Proteobacteria>Gammaproteobacteria>Vibrionales>Vibrionaceae	Vibrio_vulnificus	DNA-sulfur-modification-protein-DndB-[Vibrio-vulnificus]	672
RZP63906.1	DndB-ParB-nuclease	GCA_004214565.1	364	Bacteria>Proteobacteria>Gammaproteobacteria>Vibrionales>Vibrionaceae	Vibrio_vulnificus	DNA-sulfur-modification-protein-DndB-[Vibrio-vulnificus]	672
WP_017421950.1	DndB-ParB-nuclease	GCF_000303175.1	359	Bacteria>Proteobacteria>Gammaproteobacteria>Vibrionales>Vibrionaceae	Vibrio_vulnificus	DNA-sulfur-modification-protein-DndB-[Vibrio-vulnificus]	672
WP_017790214.1	DndB-ParB-nuclease	GCF_000342305.2	364	Bacteria>Proteobacteria>Gammaproteobacteria>Vibrionales>Vibrionaceae	Vibrio_vulnificus	DNA-sulfur-modification-protein-DndB-[Vibrio-vulnificus]	672
WP_039446571.1	DndB-ParB-nuclease	GCF_002215135.1	359	Bacteria>Proteobacteria>Gammaproteobacteria>Vibrionales>Vibrionaceae	Vibrio_vulnificus	DNA-sulfur-modification-protein-DndB-[Vibrio-vulnificus]	672
WP_045611599.1	DndB-ParB-nuclease	GCF_020169615.1	359	Bacteria>Proteobacteria>Gammaproteobacteria>Vibrionales>Vibrionaceae	Vibrio_vulnificus	DNA-sulfur-modification-protein-DndB-[Vibrio-vulnificus]	672
WP_072616054.1	DndB-ParB-nuclease	GCF_001890565.1	359	Bacteria>Proteobacteria>Gammaproteobacteria>Vibrionales>Vibrionaceae	Vibrio_vulnificus	DNA-sulfur-modification-protein-DndB-[Vibrio-vulnificus]	672
WP_101958813.1	DndB-ParB-nuclease	GCF_002863725.1	359	Bacteria>Proteobacteria>Gammaproteobacteria>Vibrionales>Vibrionaceae	Vibrio_vulnificus	DNA-sulfur-modification-protein-DndB-[Vibrio-vulnificus]	672
WP_102887624.1	DndB-ParB-nuclease	GCF_002891755.1	364	Bacteria>Proteobacteria>Gammaproteobacteria>Vibrionales>Vibrionaceae	Vibrio_vulnificus	DNA-sulfur-modification-protein-DndB-[Vibrio-vulnificus]	672
WP_103201251.1	DndB-ParB-nuclease	GCF_002903785.1	359	Bacteria>Proteobacteria>Gammaproteobacteria>Vibrionales>Vibrionaceae	Vibrio_vulnificus	DNA-sulfur-modification-protein-DndB-[Vibrio-vulnificus]	672
WP_118893508.1	DndB-ParB-nuclease	GCF_003522555.1	359	Bacteria>Proteobacteria>Gammaproteobacteria>Vibrionales>Vibrionaceae	Vibrio_vulnificus	DNA-sulfur-modification-protein-DndB-[Vibrio-vulnificus]	672
WP_130241242.1	DndB-ParB-nuclease	GCF_020251945.1	359	Bacteria>Proteobacteria>Gammaproteobacteria>Vibrionales>Vibrionaceae	Vibrio_vulnificus	DNA-sulfur-modification-protein-DndB-[Vibrio-vulnificus]	672
WP_130349864.1	DndB-ParB-nuclease	GCF_004215105.1	359	Bacteria>Proteobacteria>Gammaproteobacteria>Vibrionales>Vibrionaceae	Vibrio_vulnificus	DNA-sulfur-modification-protein-DndB-[Vibrio-vulnificus]	672
WP_154186250.1	DndB-ParB-nuclease	GCF_009665455.1	359	Bacteria>Proteobacteria>Gammaproteobacteria>Vibrionales>Vibrionaceae	Vibrio_vulnificus	DNA-sulfur-modification-protein-DndB-[Vibrio-vulnificus]	672
WP_224640685.1	DndB-ParB-nuclease	GCF_020169625.1	359	Bacteria>Proteobacteria>Gammaproteobacteria>Vibrionales>Vibrionaceae	Vibrio_vulnificus	DNA-sulfur-modification-protein-DndB-[Vibrio-vulnificus]	672
WP_039559844.1	DndB-ParB-nuclease	GCF_000743095.1	130	Bacteria>Proteobacteria>Gammaproteobacteria>Vibrionales>Vibrionaceae	Vibrio_vulnificus	hypothetical-protein,-partial-[Vibrio-vulnificus]	672
EGQ7982857.1	DndB-ParB-nuclease	GCA_015960135.1	721	Bacteria>Proteobacteria>Gammaproteobacteria>Vibrionales>Vibrionaceae	Vibrio_vulnificus	hypothetical-protein-[Vibrio-vulnificus]	672
MCA3964053.1	DndB-ParB-nuclease	GCA_020252125.1	660	Bacteria>Proteobacteria>Gammaproteobacteria>Vibrionales>Vibrionaceae	Vibrio_vulnificus	hypothetical-protein-[Vibrio-vulnificus]	672
WP_195721975.1	DndB-ParB-nuclease	GCF_009665475.1	660	Bacteria>Proteobacteria>Gammaproteobacteria>Vibrionales>Vibrionaceae	Vibrio_vulnificus	hypothetical-protein-[Vibrio-vulnificus]	672
WP_225483112.1	DndB-ParB-nuclease	GCF_020251885.1	722	Bacteria>Proteobacteria>Gammaproteobacteria>Vibrionales>Vibrionaceae	Vibrio_vulnificus	hypothetical-protein-[Vibrio-vulnificus]	672
WP_228749002.1	DndB-ParB-nuclease	GCF_020252125.1	669	Bacteria>Proteobacteria>Gammaproteobacteria>Vibrionales>Vibrionaceae	Vibrio_vulnificus	hypothetical-protein-[Vibrio-vulnificus]	672
HAS6089998.1	DndB-ParB-nuclease	GCA_015776485.1	359	Bacteria>Proteobacteria>Gammaproteobacteria>Vibrionales>Vibrionaceae	Vibrio_vulnificus	TPA:-DNA-sulfur-modification-protein-DndB-[Vibrio-vulnificus]	672
WP_147627458.1	DndB-ParB-nuclease	GCF_008000795.1	373	Bacteria>Proteobacteria>Gammaproteobacteria>Xanthomonadales>Rhodanobacteraceae	Rhodanobacter_glycinis	DNA-sulfur-modification-protein-DndB-[Rhodanobacter-glycinis]	582702
WP_232580336.1	DndB-ParB-nuclease	GCF_021233355.1	423	Bacteria>Proteobacteria>Gammaproteobacteria>Xanthomonadales>Xanthomonadaceae	Luteimonas_fraxinea	DGQHR-domain-containing-protein-[Luteimonas-sp.-D4P002]	2901869
WP_232580329.1	DndB-ParB-nuclease	GCF_021233355.1	372	Bacteria>Proteobacteria>Gammaproteobacteria>Xanthomonadales>Xanthomonadaceae	Luteimonas_fraxinea	DNA-sulfur-modification-protein-DndB-[Luteimonas-sp.-D4P002]	2901869
WP_138652046.1	DndB-ParB-nuclease	GCF_020042665.1	421	Bacteria>Proteobacteria>Gammaproteobacteria>Xanthomonadales>Xanthomonadaceae	Pseudoxanthomonas_sp._X-1	DGQHR-domain-containing-protein-[Pseudoxanthomonas-sp.-X-1]	2571115
WP_138652066.1	DndB-ParB-nuclease	GCF_020042665.1	359	Bacteria>Proteobacteria>Gammaproteobacteria>Xanthomonadales>Xanthomonadaceae	Pseudoxanthomonas_sp._X-1	DNA-sulfur-modification-protein-DndB-[Pseudoxanthomonas-sp.-X-1]	2571115
WP_220075462.1	DndB-ParB-nuclease	-	396	Bacteria>Proteobacteria>Gammaproteobacteria>Xanthomonadales>Xanthomonadaceae	Stenotrophomonas_maltophilia	DGQHR-domain-containing-protein-[Stenotrophomonas-maltophilia]	40324
WP_193601154.1	DndB-ParB-nuclease	GCF_006385505.1	413	Bacteria>Proteobacteria>Gammaproteobacteria>Xanthomonadales>Xanthomonadaceae	Stenotrophomonas_maltophilia	DNA-phosphorothioation-associated-DGQHR-protein-1-[Stenotrophomonas-maltophilia]	40324
WP_188252566.1	DndB-ParB-nuclease	GCF_014581855.1	650	Bacteria>Proteobacteria>Gammaproteobacteria>Xanthomonadales>Xanthomonadaceae	Stenotrophomonas_maltophilia	hypothetical-protein-[Stenotrophomonas-maltophilia]	40324
WP_206621788.1	DndB-ParB-nuclease	GCF_017287815.1	650	Bacteria>Proteobacteria>Gammaproteobacteria>Xanthomonadales>Xanthomonadaceae	Stenotrophomonas_maltophilia	hypothetical-protein-[Stenotrophomonas-maltophilia]	40324
WP_229290972.1	DndB-ParB-nuclease	GCF_020883215.1	650	Bacteria>Proteobacteria>Gammaproteobacteria>Xanthomonadales>Xanthomonadaceae	Stenotrophomonas_sp._SI-NJAU-1	hypothetical-protein-[Stenotrophomonas-sp.-SI-NJAU-1]	2886359
WP_046344839.1	DndB-ParB-nuclease	GCF_000972745.1	379	Bacteria>Proteobacteria>Gammaproteobacteria>Xanthomonadales>Xanthomonadaceae	Xanthomonas_arboricola	DNA-sulfur-modification-protein-DndB-[Xanthomonas-arboricola]	56448
WP_046344833.1	DndB-ParB-nuclease	GCF_000972745.1	649	Bacteria>Proteobacteria>Gammaproteobacteria>Xanthomonadales>Xanthomonadaceae	Xanthomonas_arboricola	hypothetical-protein-[Xanthomonas-arboricola]	56448
WP_167766505.1	DndB-ParB-nuclease	GCF_011927445.1	650	Bacteria>Proteobacteria>Gammaproteobacteria>Xanthomonadales>Xanthomonadaceae	Xanthomonas_arboricola	hypothetical-protein-[Xanthomonas-arboricola]	56448
WP_228442810.1	DndB-ParB-nuclease	GCF_020813075.1	413	Bacteria>Proteobacteria>Gammaproteobacteria>Xanthomonadales>Xanthomonadaceae	Xanthomonas_campestris	DGQHR-domain-containing-protein-[Xanthomonas-campestris]	339
WP_218541290.1	DndB-ParB-nuclease	GCF_019201425.1	650	Bacteria>Proteobacteria>Gammaproteobacteria>Xanthomonadales>Xanthomonadaceae	Xanthomonas_campestris	hypothetical-protein-[Xanthomonas-campestris]	339
MCC5062986.1	DndB-ParB-nuclease	GCA_020813075.1	413	Bacteria>Proteobacteria>Gammaproteobacteria>Xanthomonadales>Xanthomonadaceae	Xanthomonas_campestris_pv._raphani	DGQHR-domain-containing-protein-[Xanthomonas-campestris-pv.-raphani]	359385
WP_222103545.1	DndB-ParB-nuclease	GCF_007559225.1	409	Bacteria>Proteobacteria>Gammaproteobacteria>Xanthomonadales>Xanthomonadaceae	Xanthomonas_citri	DNA-phosphorothioation-associated-DGQHR-protein-1-[Xanthomonas-citri]	346
TSK03043.1	DndB-ParB-nuclease	GCA_007559225.1	408	Bacteria>Proteobacteria>Gammaproteobacteria>Xanthomonadales>Xanthomonadaceae	Xanthomonas_citri_pv._glycines	DGQHR-domain-containing-protein-[Xanthomonas-citri-pv.-glycines]	473421
WP_228323400.1	DndB-ParB-nuclease	GCF_020783675.1	413	Bacteria>Proteobacteria>Gammaproteobacteria>Xanthomonadales>Xanthomonadaceae	Xanthomonas_dyei	DGQHR-domain-containing-protein-[Xanthomonas-dyei]	743699
WP_078517602.1	DndB-ParB-nuclease	GCF_002019375.1	416	Bacteria>Proteobacteria>Gammaproteobacteria>Xanthomonadales>Xanthomonadaceae	Xanthomonas	MULTISPECIES:-DGQHR-domain-containing-protein-[Xanthomonas]	338
MBL6991879.1	DndB-ParB-nuclease	GCA_016784965.1	364	Bacteria>Proteobacteria>Oligoflexia>Bacteriovoracales>Bacteriovoracaceae	Bacteriovoracaceae_bacterium	DNA-sulfur-modification-protein-DndB-[Bacteriovoracaceae-bacterium]	2081706
WP_096908809.1	DndB-ParB-nuclease	GCF_002442875.1	358	Bacteria>Proteobacteria>Oligoflexia>Bacteriovoracales>Halobacteriovoraceae	Halobacteriovorax_marinus	DNA-sulfur-modification-protein-DndB-[Halobacteriovorax-marinus]	97084
MBL1147149.1	DndB-ParB-nuclease	GCA_016743515.1	453	Bacteria>Proteobacteria>unclassified_Proteobacteria	Proteobacteria_bacterium	DGQHR-domain-containing-protein-[Proteobacteria-bacterium]	1977087
MCA0359068.1	DndB-ParB-nuclease	GCA_020161365.1	414	Bacteria>Proteobacteria>unclassified_Proteobacteria	Proteobacteria_bacterium	DNA-phosphorothioation-associated-DGQHR-protein-1-[Proteobacteria-bacterium]	1977087
MBU4186208.1	DndB-ParB-nuclease	GCA_018898135.1	364	Bacteria>Proteobacteria>unclassified_Proteobacteria	Proteobacteria_bacterium	DNA-sulfur-modification-protein-DndB-[Proteobacteria-bacterium]	1977087
MCA0359070.1	DndB-ParB-nuclease	GCA_020161365.1	363	Bacteria>Proteobacteria>unclassified_Proteobacteria	Proteobacteria_bacterium	DNA-sulfur-modification-protein-DndB-[Proteobacteria-bacterium]	1977087
MBQ8570607.1	DndB-ParB-nuclease	GCA_017481725.1	354	Bacteria>PVC_group>Kiritimatiellaeota>Kiritimatiellae>unclassified_Kiritimatiellae	Kiritimatiellae_bacterium	DNA-sulfur-modification-protein-DndB-[Kiritimatiellae-bacterium]	2212473
MCF6175086.1	DndB-ParB-nuclease	GCA_021647315.1	361	Bacteria>PVC_group>Lentisphaerae>Lentisphaeria>Victivallales	Victivallaceae_bacterium	DNA-sulfur-modification-protein-DndB-[Victivallaceae-bacterium]	2793042
KAA0245643.1	DndB-ParB-nuclease	GCA_008363395.1	457	Bacteria>PVC_group>Planctomycetes>Candidatus_Brocadiia>Candidatus_Brocadiales	Candidatus_Brocadia_sp._AMX2	DGQHR-domain-containing-protein-[Candidatus-Brocadia-sp.-AMX2]	2293635
MBC6930714.1	DndB-ParB-nuclease	GCA_014338225.1	457	Bacteria>PVC_group>Planctomycetes>Candidatus_Brocadiia>Candidatus_Brocadiales	Candidatus_Brocadia_sp.	MAG:-DGQHR-domain-containing-protein-[Candidatus-Brocadia-sp.]	2172549
RIJ93307.1	DndB-ParB-nuclease	GCA_003577215.1	457	Bacteria>PVC_group>Planctomycetes>Candidatus_Brocadiia>Candidatus_Brocadiales	Candidatus_Brocadia_sp.	MAG:-hypothetical-protein-DB853_01410-[Candidatus-Brocadia-sp.]	2172549
TVL98795.1	DndB-ParB-nuclease	GCA_007618135.1	363	Bacteria>PVC_group>Planctomycetes>Candidatus_Brocadiia>Candidatus_Brocadiales	Candidatus_Brocadia_sp._WS118	DNA-sulfur-modification-protein-DndB-[Candidatus-Brocadia-sp.-WS118]	2040625
TVL98794.1	DndB-ParB-nuclease	GCA_007618135.1	229	Bacteria>PVC_group>Planctomycetes>Candidatus_Brocadiia>Candidatus_Brocadiales	Candidatus_Brocadia_sp._WS118	hypothetical-protein-CV087_20435-[Candidatus-Brocadia-sp.-WS118]	2040625
MBS1258872.1	DndB-ParB-nuclease	GCA_018263435.1	364	Bacteria>PVC_group>Planctomycetes>Candidatus_Brocadiia>Candidatus_Brocadiales	Candidatus_Scalindua_arabica	hypothetical-protein-[Candidatus-Scalindua-arabica]	1127984
MBT6051956.1	DndB-ParB-nuclease	GCA_018697755.1	363	Bacteria>PVC_group>Planctomycetes>Candidatus_Brocadiia>Candidatus_Brocadiales	Candidatus_Scalindua_sp.	DNA-sulfur-modification-protein-DndB-[Candidatus-Scalindua-sp.]	2268189
MAX27768.1	DndB-ParB-nuclease	GCA_002704825.1	326	Bacteria>PVC_group>Planctomycetes>Phycisphaerae>Phycisphaerales	Phycisphaeraceae_bacterium	hypothetical-protein-[Phycisphaeraceae-bacterium]	2026777
MBN2564057.1	DndB-ParB-nuclease	GCA_016935885.1	369	Bacteria>PVC_group>Planctomycetes>Phycisphaerae>unclassified_Phycisphaerae	Phycisphaerae_bacterium	DNA-sulfur-modification-protein-DndB-[Phycisphaerae-bacterium]	2026778
WP_145078521.1	DndB-ParB-nuclease	GCF_007752135.1	430	Bacteria>PVC_group>Planctomycetes>Planctomycetia>Pirellulales	Aureliella_helgolandensis	DGQHR-domain-containing-protein-[Aureliella-helgolandensis]	2527968
WP_145078487.1	DndB-ParB-nuclease	GCF_007752135.1	365	Bacteria>PVC_group>Planctomycetes>Planctomycetia>Pirellulales	Aureliella_helgolandensis	DNA-sulfur-modification-protein-DndB-[Aureliella-helgolandensis]	2527968
WP_007325803.1	DndB-ParB-nuclease	GCF_000195185.1	364	Bacteria>PVC_group>Planctomycetes>Planctomycetia>Pirellulales	Rhodopirellula_baltica	DNA-sulfur-modification-protein-DndB-[Rhodopirellula-baltica]	265606
EGF28178.1	DndB-ParB-nuclease	GCA_000195185.2	364	Bacteria>PVC_group>Planctomycetes>Planctomycetia>Pirellulales	Rhodopirellula_baltica_WH47	DNA-sulfur-modification-protein-DndB-[Rhodopirellula-baltica-WH47]	991778
MCC9654582.1	DndB-ParB-nuclease	GCA_020966775.1	367	Bacteria>PVC_group>Planctomycetes>Planctomycetia>Pirellulales	Rhodopirellula_sp._JC737	DNA-sulfur-modification-protein-DndB-[Rhodopirellula-sp.-JC737]	2894198
WP_230251571.1	DndB-ParB-nuclease	GCF_020966775.1	367	Bacteria>PVC_group>Planctomycetes>Planctomycetia>Pirellulales	Rhodopirellula_sp._JC737	DNA-sulfur-modification-protein-DndB-[Rhodopirellula-sp.-JC737]	2894198
PAY16679.1	DndB-ParB-nuclease	GCA_002289565.1	364	Bacteria>PVC_group>Planctomycetes>Planctomycetia>Pirellulales	Rhodopirellula_sp._SM50	DNA-sulfur-modification-protein-DndB-[Rhodopirellula-sp.-SM50]	595453
WP_095741172.1	DndB-ParB-nuclease	GCF_002289565.1	364	Bacteria>PVC_group>Planctomycetes>Planctomycetia>Pirellulales	Rhodopirellula_sp._SM50	DNA-sulfur-modification-protein-DndB-[Rhodopirellula-sp.-SM50]	595453
WP_068138744.1	DndB-ParB-nuclease	GCF_008065135.1	367	Bacteria>PVC_group>Planctomycetes>Planctomycetia>Pirellulales	Roseimaritima_ulvae	DNA-sulfur-modification-protein-DndB-[Roseimaritima-ulvae]	980254
WP_145346088.1	DndB-ParB-nuclease	GCF_007741475.1	368	Bacteria>PVC_group>Planctomycetes>Planctomycetia>Pirellulales	Rosistilla_ulvae	DNA-sulfur-modification-protein-DndB-[Rosistilla-ulvae]	1930277
WP_146520993.1	DndB-ParB-nuclease	GCF_007860045.1	344	Bacteria>PVC_group>Planctomycetes>Planctomycetia>Pirellulales	Stieleria_varia	DNA-sulfur-modification-protein-DndB-[Stieleria-varia]	2528005
WP_207622006.1	DndB-ParB-nuclease	GCF_007743815.1	387	Bacteria>PVC_group>Planctomycetes>Planctomycetia>Planctomycetales	Alienimonas_californiensis	DNA-sulfur-modification-protein-DndB-[Alienimonas-californiensis]	2527989
QDT16787.1	DndB-ParB-nuclease	GCA_007743815.1	389	Bacteria>PVC_group>Planctomycetes>Planctomycetia>Planctomycetales	Alienimonas_californiensis	hypothetical-protein-CA12_28940-[Alienimonas-californiensis]	2527989
WP_145305603.1	DndB-ParB-nuclease	GCF_007754425.1	420	Bacteria>PVC_group>Planctomycetes>Planctomycetia>Planctomycetales	Gimesia_fumaroli	DGQHR-domain-containing-protein-[Gimesia-fumaroli]	2527976
QDV48460.1	DndB-ParB-nuclease	GCA_007754425.1	420	Bacteria>PVC_group>Planctomycetes>Planctomycetia>Planctomycetales	Gimesia_fumaroli	hypothetical-protein-Enr17x_04720-[Gimesia-fumaroli]	2527976
HBJ39009.1	DndB-ParB-nuclease	GCA_003506095.1	366	Bacteria>PVC_group>Planctomycetes>Planctomycetia>Planctomycetales	Planctomycetaceae_bacterium	DNA-sulfur-modification-protein-DndB-[Planctomycetaceae-bacterium]	2026779
MBI2480686.1	DndB-ParB-nuclease	GCA_016188635.1	321	Bacteria>PVC_group>Planctomycetes>Planctomycetia>unclassified_Planctomycetia	Planctomycetia_bacterium	DNA-sulfur-modification-protein-DndB-[Planctomycetia-bacterium]	2052181
MBI3865717.1	DndB-ParB-nuclease	GCA_016200145.1	362	Bacteria>PVC_group>Planctomycetes>Planctomycetia>unclassified_Planctomycetia	Planctomycetia_bacterium	DNA-sulfur-modification-protein-DndB-[Planctomycetia-bacterium]	2052181
MBK8099840.1	DndB-ParB-nuclease	GCA_016713175.1	408	Bacteria>PVC_group>Planctomycetes>unclassified_Planctomycetes	Planctomycetes_bacterium	DGQHR-domain-containing-protein-[Planctomycetes-bacterium]	2026780
MAB77744.1	DndB-ParB-nuclease	GCA_002683825.1	377	Bacteria>PVC_group>Planctomycetes>unclassified_Planctomycetes	Planctomycetes_bacterium	DNA-sulfur-modification-protein-DndB-[Planctomycetes-bacterium]	2026780
MBE3144921.1	DndB-ParB-nuclease	GCA_014894345.1	367	Bacteria>PVC_group>Planctomycetes>unclassified_Planctomycetes	Planctomycetes_bacterium	DNA-sulfur-modification-protein-DndB-[Planctomycetes-bacterium]	2026780
MBI2808694.1	DndB-ParB-nuclease	GCA_016191165.1	420	Bacteria>PVC_group>Planctomycetes>unclassified_Planctomycetes	Planctomycetes_bacterium	DNA-sulfur-modification-protein-DndB-[Planctomycetes-bacterium]	2026780
MCC6781768.1	DndB-ParB-nuclease	GCA_020848735.1	374	Bacteria>PVC_group>Planctomycetes>unclassified_Planctomycetes	Planctomycetes_bacterium	DNA-sulfur-modification-protein-DndB-[Planctomycetes-bacterium]	2026780
MCC6781766.1	DndB-ParB-nuclease	GCA_020848735.1	683	Bacteria>PVC_group>Planctomycetes>unclassified_Planctomycetes	Planctomycetes_bacterium	hypothetical-protein-IT457_02900-[Planctomycetes-bacterium]	2026780
MBC8523696.1	DndB-ParB-nuclease	GCA_014384575.1	223	Bacteria>PVC_group>unclassified_PVC_group	PVC_group_bacterium	DGQHR-domain-containing-protein-[PVC-group-bacterium]	2056642
NJL62451.1	DndB-ParB-nuclease	GCA_012031725.1	532	Bacteria>PVC_group>Verrucomicrobia>Methylacidiphilae>Methylacidiphilales	Methylacidiphilales_bacterium	DGQHR-domain-containing-protein-[Methylacidiphilales-bacterium]	2720631

Accession	Annotation	GCA	Length	Phyletic.spread	Strain.name	Defline	Species.Taxid
HAV13931.1	DndB-ParB-nuclease	GCA_003485085.1	375	Bacteria>PVC_group>Verrucomicrobia>Opitutae>unclassified_Opitutae	Opitutae_bacterium	DNA-sulfur-modification-protein-DndB-[Opitutae-bacterium]	2026771
KAB2654360.1	DndB-ParB-nuclease	GCA_008933045.1	411	Bacteria>PVC_group>Verrucomicrobia>unclassified_Verrucomicrobia	Verrucomicrobia_bacterium	DGQHR-domain-containing-protein-[Verrucomicrobia-bacterium]	2026799
MBS0659624.1	DndB-ParB-nuclease	GCA_018240025.1	380	Bacteria>PVC_group>Verrucomicrobia>unclassified_Verrucomicrobia	Verrucomicrobia_bacterium	DGQHR-domain-containing-protein-[Verrucomicrobia-bacterium]	2026799
MBV9658017.1	DndB-ParB-nuclease	GCA_019241085.1	386	Bacteria>PVC_group>Verrucomicrobia>unclassified_Verrucomicrobia	Verrucomicrobia_bacterium	DGQHR-domain-containing-protein-[Verrucomicrobia-bacterium]	2026799
PWU19850.1	DndB-ParB-nuclease	GCA_003176045.1	363	Bacteria>PVC_group>Verrucomicrobia>unclassified_Verrucomicrobia	Verrucomicrobia_bacterium	DNA-sulfur-modification-protein-DndB-[Verrucomicrobia-bacterium]	2026799
WP_226894716.1	DndB-ParB-nuclease	GCF_014904755.1	595	Bacteria>PVC_group>Verrucomicrobia>Verrucomicrobiae>Verrucomicrobiales	Luteolibacter_sp._NBU1238	hypothetical-protein-[Luteolibacter-sp.-NBU1238]	2776705
TLD70493.1	DndB-ParB-nuclease	GCA_005780165.1	366	Bacteria>PVC_group>Verrucomicrobia>Verrucomicrobiae>Verrucomicrobiales	Phragmitibacter_flavus	DGQHR-domain-containing-protein-[Phragmitibacter-flavus]	2576071
WP_138086549.1	DndB-ParB-nuclease	GCF_005780165.1	366	Bacteria>PVC_group>Verrucomicrobia>Verrucomicrobiae>Verrucomicrobiales	Phragmitibacter_flavus	DGQHR-domain-containing-protein-[Phragmitibacter-flavus]	2576071
MBL9115291.1	DndB-ParB-nuclease	GCA_016795485.1	421	Bacteria>PVC_group>Verrucomicrobia>Verrucomicrobiae>Verrucomicrobiales	Verrucomicrobiaceae_bacterium	DGQHR-domain-containing-protein-[Verrucomicrobiaceae-bacterium]	2026800
MAS93101.1	DndB-ParB-nuclease	GCA_002698785.1	366	Bacteria>PVC_group>Verrucomicrobia>Verrucomicrobiae>Verrucomicrobiales	Verrucomicrobiales_bacterium	DNA-sulfur-modification-protein-DndB-[Verrucomicrobiales-bacterium]	2026801
MBL9153027.1	DndB-ParB-nuclease	GCA_016795505.1	358	Bacteria>PVC_group>Verrucomicrobia>Verrucomicrobiae>Verrucomicrobiales	Verrucomicrobiales_bacterium	DNA-sulfur-modification-protein-DndB-[Verrucomicrobiales-bacterium]	2026801
MAS93105.1	DndB-ParB-nuclease	GCA_002698785.1	388	Bacteria>PVC_group>Verrucomicrobia>Verrucomicrobiae>Verrucomicrobiales	Verrucomicrobiales_bacterium	hypothetical-protein-[Verrucomicrobiales-bacterium]	2026801
MBL9153022.1	DndB-ParB-nuclease	GCA_016795505.1	157	Bacteria>PVC_group>Verrucomicrobia>Verrucomicrobiae>Verrucomicrobiales	Verrucomicrobiales_bacterium	hypothetical-protein-[Verrucomicrobiales-bacterium]	2026801
MBN2532977.1	DndB-ParB-nuclease	GCA_016935735.1	364	Bacteria>Spirochaetes>Spirochaetia>Spirochaetales>unclassified_Spirochaetales	Spirochaetales_bacterium	DNA-sulfur-modification-protein-DndB-[Spirochaetales-bacterium]	2026792
MBT89379.1	DndB-ParB-nuclease	GCA_002730475.1	364	Bacteria>Spirochaetes>Spirochaetia>Spirochaetales>unclassified_Spirochaetales	Spirochaetales_bacterium	DNA-sulfur-modification-protein-DndB-[Spirochaetales-bacterium]	2026792
MXX42484.1	DndB-ParB-nuclease	GCA_009836375.1	716	Bacteria>Terrabacteria_group>Actinobacteria>Acidimicrobiia>Acidimicrobiales	Acidimicrobiales_bacterium	hypothetical-protein-[Acidimicrobiales-bacterium]	2201156
MYI07906.1	DndB-ParB-nuclease	GCA_009844415.1	716	Bacteria>Terrabacteria_group>Actinobacteria>Acidimicrobiia>Acidimicrobiales	Acidimicrobiales_bacterium	hypothetical-protein-[Acidimicrobiales-bacterium]	2201156
WP_211469982.1	DndB-ParB-nuclease	GCF_018283645.1	365	Bacteria>Terrabacteria_group>Actinobacteria>Actinomycetia>Catenulisporales	Actinocrinis_puniceicyclus	DNA-sulfur-modification-protein-DndB-[Actinocrinis-puniceicyclus]	977794
WP_228555600.1	DndB-ParB-nuclease	GCF_015356865.1	342	Bacteria>Terrabacteria_group>Actinobacteria>Actinomycetia>Catenulisporales	Catenulispora_pinisilvae	DNA-sulfur-modification-protein-DndB-[Catenulispora-pinisilvae]	2705253
OLE29078.1	DndB-ParB-nuclease	GCA_001920315.1	342	Bacteria>Terrabacteria_group>Actinobacteria>Actinomycetia>Catenulisporales	Catenulispora_sp._13_1_20CM_3_70_7	DNA-sulfur-modification-protein-DndB-[Catenulispora-sp.-13_1_20CM_3_70_7]	1805055
OLE29077.1	DndB-ParB-nuclease	GCA_001920315.1	553	Bacteria>Terrabacteria_group>Actinobacteria>Actinomycetia>Catenulisporales	Catenulispora_sp._13_1_20CM_3_70_7	hypothetical-protein-AUG49_00570-[Catenulispora-sp.-13_1_20CM_3_70_7]	1805055
WP_206475048.1	DndB-ParB-nuclease	-	365	Bacteria>Terrabacteria_group>Actinobacteria>Actinomycetia>Corynebacteriales	Dietzia_sp._KRD202	DNA-sulfur-modification-protein-DndB-[Dietzia-sp.-KRD202]	2729732
WP_241261673.1	DndB-ParB-nuclease	GCF_017168155.1	366	Bacteria>Terrabacteria_group>Actinobacteria>Actinomycetia>Corynebacteriales	Dietzia_sp._KRD202	DNA-sulfur-modification-protein-DndB-[Dietzia-sp.-KRD202]	2729732
WP_188330217.1	DndB-ParB-nuclease	GCF_014622905.1	423	Bacteria>Terrabacteria_group>Actinobacteria>Actinomycetia>Corynebacteriales	Gordonia_jinghuaiqii	DGQHR-domain-containing-protein-[Gordonia-jinghuaiqii]	2758710
WP_005207128.1	DndB-ParB-nuclease	GCF_000248055.1	380	Bacteria>Terrabacteria_group>Actinobacteria>Actinomycetia>Corynebacteriales	Gordonia_sputi	DNA-sulfur-modification-protein-DndB-[Gordonia-sputi]	36823
WP_039856128.1	DndB-ParB-nuclease	GCF_000248055.1	429	Bacteria>Terrabacteria_group>Actinobacteria>Actinomycetia>Corynebacteriales	Gordonia_sputi	hypothetical-protein-[Gordonia-sputi]	36823
MBN4927164.1	DndB-ParB-nuclease	GCA_017152025.1	379	Bacteria>Terrabacteria_group>Actinobacteria>Actinomycetia>Corynebacteriales	Hoyosella_rhizosphaerae	DNA-sulfur-modification-protein-DndB-[Hoyosella-rhizosphaerae]	1755582
WP_229675833.1	DndB-ParB-nuclease	GCF_014643175.1	341	Bacteria>Terrabacteria_group>Actinobacteria>Actinomycetia>Corynebacteriales	Hoyosella_rhizosphaerae	DNA-sulfur-modification-protein-DndB-[Hoyosella-rhizosphaerae]	1755582
GGC53507.1	DndB-ParB-nuclease	GCA_014643175.1	379	Bacteria>Terrabacteria_group>Actinobacteria>Actinomycetia>Corynebacteriales	Hoyosella_rhizosphaerae	hypothetical-protein-GCM10011410_02330-[Hoyosella-rhizosphaerae]	1755582
GGC53547.1	DndB-ParB-nuclease	GCA_014643175.1	753	Bacteria>Terrabacteria_group>Actinobacteria>Actinomycetia>Corynebacteriales	Hoyosella_rhizosphaerae	hypothetical-protein-GCM10011410_02390-[Hoyosella-rhizosphaerae]	1755582
MBN4927158.1	DndB-ParB-nuclease	GCA_017152025.1	722	Bacteria>Terrabacteria_group>Actinobacteria>Actinomycetia>Corynebacteriales	Hoyosella_rhizosphaerae	hypothetical-protein-[Hoyosella-rhizosphaerae]	1755582
WP_206050433.1	DndB-ParB-nuclease	GCF_014643175.1	722	Bacteria>Terrabacteria_group>Actinobacteria>Actinomycetia>Corynebacteriales	Hoyosella_rhizosphaerae	hypothetical-protein-[Hoyosella-rhizosphaerae]	1755582
WP_076217387.1	DndB-ParB-nuclease	GCF_900132695.1	382	Bacteria>Terrabacteria_group>Actinobacteria>Actinomycetia>Corynebacteriales	Mycobacteriaceae	DNA-sulfur-modification-protein-DndB-[Mycobacteroides-abscessus]	1762
WP_081290253.1	DndB-ParB-nuclease	GCF_001673365.1	381	Bacteria>Terrabacteria_group>Actinobacteria>Actinomycetia>Corynebacteriales	Mycobacteriaceae	MULTISPECIES:-DNA-sulfur-modification-protein-DndB-[Mycobacteriaceae]	1762
WP_076217382.1	DndB-ParB-nuclease	GCF_900132695.1	744	Bacteria>Terrabacteria_group>Actinobacteria>Actinomycetia>Corynebacteriales	Mycobacteriaceae	MULTISPECIES:-hypothetical-protein-[Mycobacteriaceae]	1762
OBJ57440.1	DndB-ParB-nuclease	GCA_001673165.1	382	Bacteria>Terrabacteria_group>Actinobacteria>Actinomycetia>Corynebacteriales	Mycobacterium_asiaticum	DNA-sulfur-modification-protein-DndB-[Mycobacterium-asiaticum]	1790
OBK28912.1	DndB-ParB-nuclease	GCA_001673365.1	330	Bacteria>Terrabacteria_group>Actinobacteria>Actinomycetia>Corynebacteriales	Mycobacterium_asiaticum	DNA-sulfur-modification-protein-DndB-[Mycobacterium-asiaticum]	1790
WP_065155000.1	DndB-ParB-nuclease	-	382	Bacteria>Terrabacteria_group>Actinobacteria>Actinomycetia>Corynebacteriales	Mycobacterium_asiaticum	DNA-sulfur-modification-protein-DndB-[Mycobacterium-asiaticum]	1790
WP_070946310.1	DndB-ParB-nuclease	GCF_022348025.1	382	Bacteria>Terrabacteria_group>Actinobacteria>Actinomycetia>Corynebacteriales	Mycobacterium	DNA-sulfur-modification-protein-DndB-[Mycobacterium-syngnathidarum]	1763
WP_172834738.1	DndB-ParB-nuclease	-	359	Bacteria>Terrabacteria_group>Actinobacteria>Actinomycetia>Corynebacteriales	Mycobacterium_gordonae	DNA-sulfur-modification-protein-DndB-[Mycobacterium-gordonae]	1778
WP_156772785.1	DndB-ParB-nuclease	GCF_001673475.1	573	Bacteria>Terrabacteria_group>Actinobacteria>Actinomycetia>Corynebacteriales	Mycobacterium_gordonae	hypothetical-protein-[Mycobacterium-gordonae]	1778
WP_070946305.1	DndB-ParB-nuclease	GCF_022348025.1	743	Bacteria>Terrabacteria_group>Actinobacteria>Actinomycetia>Corynebacteriales	Mycobacterium	hypothetical-protein-[Mycobacterium-syngnathidarum]	1763
ODQ99146.1	DndB-ParB-nuclease	GCA_001722345.1	382	Bacteria>Terrabacteria_group>Actinobacteria>Actinomycetia>Corynebacteriales	Mycobacterium_intermedium	DNA-sulfur-modification-protein-DndB-[Mycobacterium-intermedium]	28445
OPE50549.1	DndB-ParB-nuclease	GCA_002024965.1	382	Bacteria>Terrabacteria_group>Actinobacteria>Actinomycetia>Corynebacteriales	Mycobacterium_intermedium	DNA-sulfur-modification-protein-DndB-[Mycobacterium-intermedium]	28445
ORB05494.1	DndB-ParB-nuclease	GCA_002086275.1	382	Bacteria>Terrabacteria_group>Actinobacteria>Actinomycetia>Corynebacteriales	Mycobacterium_intermedium	DNA-sulfur-modification-protein-DndB-[Mycobacterium-intermedium]	28445
WP_232007362.1	DndB-ParB-nuclease	GCF_001722345.1	342	Bacteria>Terrabacteria_group>Actinobacteria>Actinomycetia>Corynebacteriales	Mycobacterium_intermedium	DNA-sulfur-modification-protein-DndB-[Mycobacterium-intermedium]	28445
PBA28938.1	DndB-ParB-nuclease	GCA_002292535.1	382	Bacteria>Terrabacteria_group>Actinobacteria>Actinomycetia>Corynebacteriales	Mycobacterium_intracellulare	DNA-sulfur-modification-protein-DndB-[Mycobacterium-intracellulare]	1767
WP_179293553.1	DndB-ParB-nuclease	-	384	Bacteria>Terrabacteria_group>Actinobacteria>Actinomycetia>Corynebacteriales	Mycobacterium_intracellulare	DNA-sulfur-modification-protein-DndB-[Mycobacterium-intracellulare]	1767
WP_083213348.1	DndB-ParB-nuclease	GCF_001686745.1	382	Bacteria>Terrabacteria_group>Actinobacteria>Actinomycetia>Corynebacteriales	Mycobacterium_malmoense	DNA-sulfur-modification-protein-DndB-[Mycobacterium-malmoense]	1780
WP_065480632.1	DndB-ParB-nuclease	GCF_001686745.1	741	Bacteria>Terrabacteria_group>Actinobacteria>Actinomycetia>Corynebacteriales	Mycobacterium_malmoense	hypothetical-protein-[Mycobacterium-malmoense]	1780
WP_007170950.1	DndB-ParB-nuclease	GCF_000164135.1	383	Bacteria>Terrabacteria_group>Actinobacteria>Actinomycetia>Corynebacteriales	Mycobacterium	MULTISPECIES:-DNA-sulfur-modification-protein-DndB-[Mycobacterium]	1763
WP_170929194.1	DndB-ParB-nuclease	-	357	Bacteria>Terrabacteria_group>Actinobacteria>Actinomycetia>Corynebacteriales	Mycobacterium_paraense	DNA-sulfur-modification-protein-DndB-[Mycobacterium-paraense]	767916
WP_139830798.1	DndB-ParB-nuclease	GCF_002102295.1	576	Bacteria>Terrabacteria_group>Actinobacteria>Actinomycetia>Corynebacteriales	Mycobacterium_paraense	hypothetical-protein-[Mycobacterium-paraense]	767916
EFG78014.1	DndB-ParB-nuclease	GCA_000164135.1	383	Bacteria>Terrabacteria_group>Actinobacteria>Actinomycetia>Corynebacteriales	Mycobacterium_parascrofulaceum_ATCC_BAA-614	DNA-sulfur-modification-protein-DndB-[Mycobacterium-parascrofulaceum-ATCC-BAA-614]	525368
OCB14089.1	DndB-ParB-nuclease	GCA_001686675.1	379	Bacteria>Terrabacteria_group>Actinobacteria>Actinomycetia>Corynebacteriales	Mycobacterium_porcinum	DNA-sulfur-modification-protein-DndB-[Mycobacterium-porcinum]	39693
WP_232069330.1	DndB-ParB-nuclease	GCF_010729105.1	340	Bacteria>Terrabacteria_group>Actinobacteria>Actinomycetia>Corynebacteriales	Mycobacterium_saskatchewanense	DNA-sulfur-modification-protein-DndB-[Mycobacterium-saskatchewanense]	220927
ORB63764.1	DndB-ParB-nuclease	GCA_002086755.1	384	Bacteria>Terrabacteria_group>Actinobacteria>Actinomycetia>Corynebacteriales	Mycobacterium_shinjukuense	DNA-sulfur-modification-protein-DndB-[Mycobacterium-shinjukuense]	398694
WP_232065330.1	DndB-ParB-nuclease	GCF_010730055.1	384	Bacteria>Terrabacteria_group>Actinobacteria>Actinomycetia>Corynebacteriales	Mycobacterium_shinjukuense	DNA-sulfur-modification-protein-DndB-[Mycobacterium-shinjukuense]	398694
BBX74263.1	DndB-ParB-nuclease	GCA_010730055.1	384	Bacteria>Terrabacteria_group>Actinobacteria>Actinomycetia>Corynebacteriales	Mycobacterium_shinjukuense	hypothetical-protein-MSHI_21690-[Mycobacterium-shinjukuense]	398694
WP_102417214.1	DndB-ParB-nuclease	GCF_900240975.1	382	Bacteria>Terrabacteria_group>Actinobacteria>Actinomycetia>Corynebacteriales	Mycobacterium_sp._4858	DNA-sulfur-modification-protein-DndB-[Mycobacterium-sp.-4858]	2057185
WP_082976986.1	DndB-ParB-nuclease	GCF_001667185.1	382	Bacteria>Terrabacteria_group>Actinobacteria>Actinomycetia>Corynebacteriales	Mycobacterium_sp._852002-50816_SCH5313054-b	DNA-sulfur-modification-protein-DndB-[Mycobacterium-sp.-852002-50816_SCH5313054-b]	1834092
WP_066959871.1	DndB-ParB-nuclease	GCF_001667185.1	743	Bacteria>Terrabacteria_group>Actinobacteria>Actinomycetia>Corynebacteriales	Mycobacterium_sp._852002-50816_SCH5313054-b	hypothetical-protein-[Mycobacterium-sp.-852002-50816_SCH5313054-b]	1834092
OBC03469.1	DndB-ParB-nuclease	GCA_001665685.1	382	Bacteria>Terrabacteria_group>Actinobacteria>Actinomycetia>Corynebacteriales	Mycobacterium_sp._852013-50091_SCH5140682	DNA-sulfur-modification-protein-DndB-[Mycobacterium-sp.-852013-50091_SCH5140682]	1834109
OBC03473.1	DndB-ParB-nuclease	GCA_001665685.1	575	Bacteria>Terrabacteria_group>Actinobacteria>Actinomycetia>Corynebacteriales	Mycobacterium_sp._852013-50091_SCH5140682	hypothetical-protein-A5784_14800-[Mycobacterium-sp.-852013-50091_SCH5140682]	1834109
MBO0879188.1	DndB-ParB-nuclease	GCA_017354565.1	380	Bacteria>Terrabacteria_group>Actinobacteria>Actinomycetia>Corynebacteriales	Mycobacterium_sp.	DNA-sulfur-modification-protein-DndB-[Mycobacterium-sp.]	1785
WP_231985130.1	DndB-ParB-nuclease	-	341	Bacteria>Terrabacteria_group>Actinobacteria>Actinomycetia>Corynebacteriales	Mycobacterium_sp._E796	DNA-sulfur-modification-protein-DndB-[Mycobacterium-sp.-E796]	1834151
WP_068129007.1	DndB-ParB-nuclease	GCF_001672815.1	531	Bacteria>Terrabacteria_group>Actinobacteria>Actinomycetia>Corynebacteriales	Mycobacterium_sp._E796	hypothetical-protein-[Mycobacterium-sp.-E796]	1834151
WP_238159064.1	DndB-ParB-nuclease	-	342	Bacteria>Terrabacteria_group>Actinobacteria>Actinomycetia>Corynebacteriales	Mycobacterium_sp._MFM001	DNA-sulfur-modification-protein-DndB-[Mycobacterium-sp.-MFM001]	2049453
GBE65146.1	DndB-ParB-nuclease	GCA_003402475.1	381	Bacteria>Terrabacteria_group>Actinobacteria>Actinomycetia>Corynebacteriales	Mycobacterium_sp._MFM001	hypothetical-protein-MFM001_16080-[Mycobacterium-sp.-MFM001]	2049453
OMC01069.1	DndB-ParB-nuclease	GCA_001953975.1	382	Bacteria>Terrabacteria_group>Actinobacteria>Actinomycetia>Corynebacteriales	Mycobacterium_sp._NS-7484	DNA-sulfur-modification-protein-DndB-[Mycobacterium-sp.-NS-7484]	1834161
WP_076240016.1	DndB-ParB-nuclease	GCF_001953975.1	382	Bacteria>Terrabacteria_group>Actinobacteria>Actinomycetia>Corynebacteriales	Mycobacterium_sp._NS-7484	DNA-sulfur-modification-protein-DndB-[Mycobacterium-sp.-NS-7484]	1834161
OHT93330.1	DndB-ParB-nuclease	GCA_001853525.1	382	Bacteria>Terrabacteria_group>Actinobacteria>Actinomycetia>Corynebacteriales	Mycobacterium_syngnathidarum	DNA-sulfur-modification-protein-DndB-[Mycobacterium-syngnathidarum]	1908205
OLT88101.1	DndB-ParB-nuclease	GCA_001942625.1	382	Bacteria>Terrabacteria_group>Actinobacteria>Actinomycetia>Corynebacteriales	Mycobacterium_syngnathidarum	DNA-sulfur-modification-protein-DndB-[Mycobacterium-syngnathidarum]	1908205
OLT88097.1	DndB-ParB-nuclease	GCA_001942625.1	743	Bacteria>Terrabacteria_group>Actinobacteria>Actinomycetia>Corynebacteriales	Mycobacterium_syngnathidarum	hypothetical-protein-BKG60_27560-[Mycobacterium-syngnathidarum]	1908205
OHT93326.1	DndB-ParB-nuclease	GCA_001853525.1	743	Bacteria>Terrabacteria_group>Actinobacteria>Actinomycetia>Corynebacteriales	Mycobacterium_syngnathidarum	hypothetical-protein-BKG61_22365-[Mycobacterium-syngnathidarum]	1908205
EIV42605.1	DndB-ParB-nuclease	GCA_000270585.1	744	Bacteria>Terrabacteria_group>Actinobacteria>Actinomycetia>Corynebacteriales	Mycobacteroides_abscessus_3A-0122-S	hypothetical-protein-MA3A0122S_0655-[Mycobacteroides-abscessus-3A-0122-S]	1001716
EIV42600.1	DndB-ParB-nuclease	GCA_000270585.1	330	Bacteria>Terrabacteria_group>Actinobacteria>Actinomycetia>Corynebacteriales	Mycobacteroides_abscessus_3A-0122-S	putative-ATPase-[Mycobacteroides-abscessus-3A-0122-S]	1001716
EIU50878.1	DndB-ParB-nuclease	GCA_000270985.1	744	Bacteria>Terrabacteria_group>Actinobacteria>Actinomycetia>Corynebacteriales	Mycobacteroides_abscessus_6G-0125-R	hypothetical-protein-MA6G0125R_0154-[Mycobacteroides-abscessus-6G-0125-R]	1001740
EIU50873.1	DndB-ParB-nuclease	GCA_000270985.1	330	Bacteria>Terrabacteria_group>Actinobacteria>Actinomycetia>Corynebacteriales	Mycobacteroides_abscessus_6G-0125-R	putative-ATPase-[Mycobacteroides-abscessus-6G-0125-R]	1001740
EIU48493.1	DndB-ParB-nuclease	GCA_000270865.1	760	Bacteria>Terrabacteria_group>Actinobacteria>Actinomycetia>Corynebacteriales	Mycobacteroides_abscessus_6G-0125-S	hypothetical-protein-MA6G0125S_1124-[Mycobacteroides-abscessus-6G-0125-S]	1001741
EIU48488.1	DndB-ParB-nuclease	GCA_000270865.1	341	Bacteria>Terrabacteria_group>Actinobacteria>Actinomycetia>Corynebacteriales	Mycobacteroides_abscessus_6G-0125-S	putative-ATPase-[Mycobacteroides-abscessus-6G-0125-S]	1001741
PVB01792.1	DndB-ParB-nuclease	GCA_003072295.1	421	Bacteria>Terrabacteria_group>Actinobacteria>Actinomycetia>Corynebacteriales	Mycobacteroides_abscessus	DGQHR-domain-containing-protein-[Mycobacteroides-abscessus]	36809
WP_100459286.1	DndB-ParB-nuclease	GCF_024389745.1	421	Bacteria>Terrabacteria_group>Actinobacteria>Actinomycetia>Corynebacteriales	Mycobacteroides_abscessus	DGQHR-domain-containing-protein-[Mycobacteroides-abscessus]	36809
PVA41050.1	DndB-ParB-nuclease	GCA_003071765.1	374	Bacteria>Terrabacteria_group>Actinobacteria>Actinomycetia>Corynebacteriales	Mycobacteroides_abscessus	DNA-sulfur-modification-protein-DndB-[Mycobacteroides-abscessus]	36809
PVB02276.1	DndB-ParB-nuclease	GCA_003072295.1	376	Bacteria>Terrabacteria_group>Actinobacteria>Actinomycetia>Corynebacteriales	Mycobacteroides_abscessus	DNA-sulfur-modification-protein-DndB-[Mycobacteroides-abscessus]	36809
WP_005134924.1	DndB-ParB-nuclease	GCF_000069185.1	380	Bacteria>Terrabacteria_group>Actinobacteria>Actinomycetia>Corynebacteriales	Mycobacteroides_abscessus	DNA-sulfur-modification-protein-DndB-[Mycobacteroides-abscessus]	36809
WP_074354978.1	DndB-ParB-nuclease	GCF_900134175.1	381	Bacteria>Terrabacteria_group>Actinobacteria>Actinomycetia>Corynebacteriales	Mycobacteroides_abscessus	DNA-sulfur-modification-protein-DndB-[Mycobacteroides-abscessus]	36809
WP_100479986.1	DndB-ParB-nuclease	GCF_002801525.1	382	Bacteria>Terrabacteria_group>Actinobacteria>Actinomycetia>Corynebacteriales	Mycobacteroides_abscessus	DNA-sulfur-modification-protein-DndB-[Mycobacteroides-abscessus]	36809
WP_234803502.1	DndB-ParB-nuclease	GCF_900140055.1	341	Bacteria>Terrabacteria_group>Actinobacteria>Actinomycetia>Corynebacteriales	Mycobacteroides_abscessus	DNA-sulfur-modification-protein-DndB-[Mycobacteroides-abscessus]	36809
WP_234821667.1	DndB-ParB-nuclease	GCF_002800315.1	381	Bacteria>Terrabacteria_group>Actinobacteria>Actinomycetia>Corynebacteriales	Mycobacteroides_abscessus	DNA-sulfur-modification-protein-DndB-[Mycobacteroides-abscessus]	36809
WP_235661570.1	DndB-ParB-nuclease	GCF_024389745.1	382	Bacteria>Terrabacteria_group>Actinobacteria>Actinomycetia>Corynebacteriales	Mycobacteroides_abscessus	DNA-sulfur-modification-protein-DndB-[Mycobacteroides-abscessus]	36809
WP_005109927.1	DndB-ParB-nuclease	GCF_000069185.1	744	Bacteria>Terrabacteria_group>Actinobacteria>Actinomycetia>Corynebacteriales	Mycobacteroides_abscessus	hypothetical-protein-[Mycobacteroides-abscessus]	36809
WP_100515508.1	DndB-ParB-nuclease	GCF_002801525.1	533	Bacteria>Terrabacteria_group>Actinobacteria>Actinomycetia>Corynebacteriales	Mycobacteroides_abscessus	hypothetical-protein-[Mycobacteroides-abscessus]	36809
SHV98227.1	DndB-ParB-nuclease	GCA_900134205.1	421	Bacteria>Terrabacteria_group>Actinobacteria>Actinomycetia>Corynebacteriales	Mycobacteroides_abscessus_subsp._abscessus	DGQHR-domain-[Mycobacteroides-abscessus-subsp.-abscessus]	1185650
SHW98526.1	DndB-ParB-nuclease	GCA_900134175.1	421	Bacteria>Terrabacteria_group>Actinobacteria>Actinomycetia>Corynebacteriales	Mycobacteroides_abscessus_subsp._abscessus	DGQHR-domain-[Mycobacteroides-abscessus-subsp.-abscessus]	1185650
SIB24935.1	DndB-ParB-nuclease	GCA_900135845.1	421	Bacteria>Terrabacteria_group>Actinobacteria>Actinomycetia>Corynebacteriales	Mycobacteroides_abscessus_subsp._abscessus	DGQHR-domain-[Mycobacteroides-abscessus-subsp.-abscessus]	1185650
SHV98072.1	DndB-ParB-nuclease	GCA_900134205.1	381	Bacteria>Terrabacteria_group>Actinobacteria>Actinomycetia>Corynebacteriales	Mycobacteroides_abscessus_subsp._abscessus	DNA-sulfur-modification-protein-DndB-[Mycobacteroides-abscessus-subsp.-abscessus]	1185650
SHW98742.1	DndB-ParB-nuclease	GCA_900134175.1	381	Bacteria>Terrabacteria_group>Actinobacteria>Actinomycetia>Corynebacteriales	Mycobacteroides_abscessus_subsp._abscessus	DNA-sulfur-modification-protein-DndB-[Mycobacteroides-abscessus-subsp.-abscessus]	1185650
SIB24757.1	DndB-ParB-nuclease	GCA_900135845.1	381	Bacteria>Terrabacteria_group>Actinobacteria>Actinomycetia>Corynebacteriales	Mycobacteroides_abscessus_subsp._abscessus	DNA-sulfur-modification-protein-DndB-[Mycobacteroides-abscessus-subsp.-abscessus]	1185650
SIJ89828.1	DndB-ParB-nuclease	GCA_900140055.1	380	Bacteria>Terrabacteria_group>Actinobacteria>Actinomycetia>Corynebacteriales	Mycobacteroides_abscessus_subsp._bolletii	DNA-sulfur-modification-protein-DndB-[Mycobacteroides-abscessus-subsp.-bolletii]	319705
SLF81078.1	DndB-ParB-nuclease	GCA_900140175.1	380	Bacteria>Terrabacteria_group>Actinobacteria>Actinomycetia>Corynebacteriales	Mycobacteroides_abscessus_subsp._bolletii	DNA-sulfur-modification-protein-DndB-[Mycobacteroides-abscessus-subsp.-bolletii]	319705
MBN7507520.1	DndB-ParB-nuclease	GCA_017175855.1	410	Bacteria>Terrabacteria_group>Actinobacteria>Actinomycetia>Corynebacteriales	Mycobacteroides_abscessus_subsp._massiliense	DGQHR-domain-containing-protein-[Mycobacteroides-abscessus-subsp.-massiliense]	1962118
SKE06315.1	DndB-ParB-nuclease	GCA_900130575.1	421	Bacteria>Terrabacteria_group>Actinobacteria>Actinomycetia>Corynebacteriales	Mycobacteroides_abscessus_subsp._massiliense	DGQHR-domain-[Mycobacteroides-abscessus-subsp.-massiliense]	1962118
MBN7507514.1	DndB-ParB-nuclease	GCA_017175855.1	376	Bacteria>Terrabacteria_group>Actinobacteria>Actinomycetia>Corynebacteriales	Mycobacteroides_abscessus_subsp._massiliense	DNA-sulfur-modification-protein-DndB-[Mycobacteroides-abscessus-subsp.-massiliense]	1962118
SKE06759.1	DndB-ParB-nuclease	GCA_900130575.1	381	Bacteria>Terrabacteria_group>Actinobacteria>Actinomycetia>Corynebacteriales	Mycobacteroides_abscessus_subsp._massiliense	DNA-sulfur-modification-protein-DndB-[Mycobacteroides-abscessus-subsp.-massiliense]	1962118
MBF9435262.1	DndB-ParB-nuclease	GCA_015700815.1	410	Bacteria>Terrabacteria_group>Actinobacteria>Actinomycetia>Corynebacteriales	Mycobacteroides_chelonae	DGQHR-domain-containing-protein-[Mycobacteroides-chelonae]	1774
MBF9328416.1	DndB-ParB-nuclease	GCA_015700855.1	357	Bacteria>Terrabacteria_group>Actinobacteria>Actinomycetia>Corynebacteriales	Mycobacteroides_chelonae	DNA-sulfur-modification-protein-DndB-[Mycobacteroides-chelonae]	1774
MBF9435256.1	DndB-ParB-nuclease	GCA_015700815.1	376	Bacteria>Terrabacteria_group>Actinobacteria>Actinomycetia>Corynebacteriales	Mycobacteroides_chelonae	DNA-sulfur-modification-protein-DndB-[Mycobacteroides-chelonae]	1774
WP_235886914.1	DndB-ParB-nuclease	GCF_015700835.1	340	Bacteria>Terrabacteria_group>Actinobacteria>Actinomycetia>Corynebacteriales	Mycobacteroides_chelonae	DNA-sulfur-modification-protein-DndB-[Mycobacteroides-chelonae]	1774
WP_074354984.1	DndB-ParB-nuclease	GCF_900134175.1	410	Bacteria>Terrabacteria_group>Actinobacteria>Actinomycetia>Corynebacteriales	Mycobacteroides	MULTISPECIES:-DGQHR-domain-containing-protein-[Mycobacteroides]	670516
WP_234824572.1	DndB-ParB-nuclease	GCF_900130575.1	339	Bacteria>Terrabacteria_group>Actinobacteria>Actinomycetia>Corynebacteriales	Mycobacteroides	MULTISPECIES:-DNA-sulfur-modification-protein-DndB-[Mycobacteroides]	670516
ORA45172.1	DndB-ParB-nuclease	GCA_002086175.1	383	Bacteria>Terrabacteria_group>Actinobacteria>Actinomycetia>Corynebacteriales	Mycolicibacterium_celeriflavum	DNA-sulfur-modification-protein-DndB-[Mycolicibacterium-celeriflavum]	1249101
WP_234806227.1	DndB-ParB-nuclease	GCF_010731795.1	342	Bacteria>Terrabacteria_group>Actinobacteria>Actinomycetia>Corynebacteriales	Mycolicibacterium_celeriflavum	DNA-sulfur-modification-protein-DndB-[Mycolicibacterium-celeriflavum]	1249101
BBY44172.1	DndB-ParB-nuclease	GCA_010731795.1	383	Bacteria>Terrabacteria_group>Actinobacteria>Actinomycetia>Corynebacteriales	Mycolicibacterium_celeriflavum	hypothetical-protein-MCEL_24670-[Mycolicibacterium-celeriflavum]	1249101
WP_109783709.1	DndB-ParB-nuclease	GCF_001673265.1	382	Bacteria>Terrabacteria_group>Actinobacteria>Actinomycetia>Corynebacteriales	Mycolicibacterium_conceptionense	DNA-sulfur-modification-protein-DndB-[Mycolicibacterium-conceptionense]	451644
WP_065063057.1	DndB-ParB-nuclease	GCF_001673265.1	744	Bacteria>Terrabacteria_group>Actinobacteria>Actinomycetia>Corynebacteriales	Mycolicibacterium_conceptionense	hypothetical-protein-[Mycolicibacterium-conceptionense]	451644
WP_064889480.1	DndB-ParB-nuclease	GCF_001665755.1	413	Bacteria>Terrabacteria_group>Actinobacteria>Actinomycetia>Corynebacteriales	Mycolicibacterium_elephantis	DGQHR-domain-containing-protein-[Mycolicibacterium-elephantis]	81858
WP_064889482.1	DndB-ParB-nuclease	GCF_001665755.1	439	Bacteria>Terrabacteria_group>Actinobacteria>Actinomycetia>Corynebacteriales	Mycolicibacterium_elephantis	DGQHR-domain-containing-protein-[Mycolicibacterium-elephantis]	81858
WP_082944227.1	DndB-ParB-nuclease	GCF_001665755.1	529	Bacteria>Terrabacteria_group>Actinobacteria>Actinomycetia>Corynebacteriales	Mycolicibacterium_elephantis	DGQHR-domain-containing-protein-[Mycolicibacterium-elephantis]	81858
WP_234785747.1	DndB-ParB-nuclease	GCF_001665755.1	342	Bacteria>Terrabacteria_group>Actinobacteria>Actinomycetia>Corynebacteriales	Mycolicibacterium_elephantis	DNA-sulfur-modification-protein-DndB-[Mycolicibacterium-elephantis]	81858
NOR03464.1	DndB-ParB-nuclease	GCA_013138535.1	382	Bacteria>Terrabacteria_group>Actinobacteria>Actinomycetia>Corynebacteriales	Mycolicibacterium_fortuitum	DNA-sulfur-modification-protein-DndB-[Mycolicibacterium-fortuitum]	1766
WP_171508662.1	DndB-ParB-nuclease	GCF_013138535.1	382	Bacteria>Terrabacteria_group>Actinobacteria>Actinomycetia>Corynebacteriales	Mycolicibacterium_fortuitum	DNA-sulfur-modification-protein-DndB-[Mycolicibacterium-fortuitum]	1766
NOR03458.1	DndB-ParB-nuclease	GCA_013138535.1	743	Bacteria>Terrabacteria_group>Actinobacteria>Actinomycetia>Corynebacteriales	Mycolicibacterium_fortuitum	hypothetical-protein-[Mycolicibacterium-fortuitum]	1766
WP_171508656.1	DndB-ParB-nuclease	GCF_013138535.1	743	Bacteria>Terrabacteria_group>Actinobacteria>Actinomycetia>Corynebacteriales	Mycolicibacterium_fortuitum	hypothetical-protein-[Mycolicibacterium-fortuitum]	1766
OBJ37451.1	DndB-ParB-nuclease	GCA_001668705.1	331	Bacteria>Terrabacteria_group>Actinobacteria>Actinomycetia>Corynebacteriales	Mycolicibacterium_mucogenicum	DNA-sulfur-modification-protein-DndB-[Mycolicibacterium-mucogenicum]	56689

Accession	Annotation	GCA	Length	Phyletic.spread	Strain.name	Defline	Species.Taxid
WP_234791653.1	DndB-ParB-nuclease	GCF_001668705.1	383	Bacteria>Terrabacteria_group>Actinobacteria>Actinomycetia>Corynebacteriales	Mycolicibacterium_mucogenicum	DNA-sulfur-modification-protein-DndB-[Mycolicibacterium-mucogenicum]	56689
WP_040633802.1	DndB-ParB-nuclease	GCF_015333575.1	439	Bacteria>Terrabacteria_group>Actinobacteria>Actinomycetia>Corynebacteriales	Mycolicibacterium_phlei	DGQHR-domain-containing-protein-[Mycolicibacterium-phlei]	1771
WP_081491126.1	DndB-ParB-nuclease	GCF_015333575.1	424	Bacteria>Terrabacteria_group>Actinobacteria>Actinomycetia>Corynebacteriales	Mycolicibacterium_phlei	DGQHR-domain-containing-protein-[Mycolicibacterium-phlei]	1771
WP_237721084.1	DndB-ParB-nuclease	GCF_000257725.1	342	Bacteria>Terrabacteria_group>Actinobacteria>Actinomycetia>Corynebacteriales	Mycolicibacterium_phlei	DNA-sulfur-modification-protein-DndB-[Mycolicibacterium-phlei]	1771
MBF4191400.1	DndB-ParB-nuclease	GCA_015333575.1	382	Bacteria>Terrabacteria_group>Actinobacteria>Actinomycetia>Corynebacteriales	Mycolicibacterium_phlei	hypothetical-protein-[Mycolicibacterium-phlei]	1771
MBF4191402.1	DndB-ParB-nuclease	GCA_015333575.1	367	Bacteria>Terrabacteria_group>Actinobacteria>Actinomycetia>Corynebacteriales	Mycolicibacterium_phlei	hypothetical-protein-[Mycolicibacterium-phlei]	1771
MBF4191404.1	DndB-ParB-nuclease	GCA_015333575.1	267	Bacteria>Terrabacteria_group>Actinobacteria>Actinomycetia>Corynebacteriales	Mycolicibacterium_phlei	hypothetical-protein-[Mycolicibacterium-phlei]	1771
EID14665.1	DndB-ParB-nuclease	GCA_000257725.1	381	Bacteria>Terrabacteria_group>Actinobacteria>Actinomycetia>Corynebacteriales	Mycolicibacterium_phlei_RIVM601174	hypothetical-protein-MPHLEI_10034-[Mycolicibacterium-phlei-RIVM601174]	1150599
EID14667.1	DndB-ParB-nuclease	GCA_000257725.1	367	Bacteria>Terrabacteria_group>Actinobacteria>Actinomycetia>Corynebacteriales	Mycolicibacterium_phlei_RIVM601174	hypothetical-protein-MPHLEI_10044-[Mycolicibacterium-phlei-RIVM601174]	1150599
EID14668.1	DndB-ParB-nuclease	GCA_000257725.1	267	Bacteria>Terrabacteria_group>Actinobacteria>Actinomycetia>Corynebacteriales	Mycolicibacterium_phlei_RIVM601174	hypothetical-protein-MPHLEI_10049-[Mycolicibacterium-phlei-RIVM601174]	1150599
EJZ12216.1	DndB-ParB-nuclease	GCA_000295825.1	307	Bacteria>Terrabacteria_group>Actinobacteria>Actinomycetia>Corynebacteriales	Mycolicibacterium_vaccae_ATCC_25954	hypothetical-protein-MVAC_03131-[Mycolicibacterium-vaccae-ATCC-25954]	1194972
EJZ12223.1	DndB-ParB-nuclease	GCA_000295825.1	576	Bacteria>Terrabacteria_group>Actinobacteria>Actinomycetia>Corynebacteriales	Mycolicibacterium_vaccae_ATCC_25954	hypothetical-protein-MVAC_03166-[Mycolicibacterium-vaccae-ATCC-25954]	1194972
WP_081529015.1	DndB-ParB-nuclease	GCF_001552715.1	381	Bacteria>Terrabacteria_group>Actinobacteria>Actinomycetia>Corynebacteriales	Mycolicibacterium_vaccae	DNA-sulfur-modification-protein-DndB-[Mycolicibacterium-vaccae]	1810
WP_157895601.1	DndB-ParB-nuclease	GCF_001655245.1	577	Bacteria>Terrabacteria_group>Actinobacteria>Actinomycetia>Corynebacteriales	Mycolicibacterium_vaccae	hypothetical-protein-[Mycolicibacterium-vaccae]	1810
WP_234806454.1	DndB-ParB-nuclease	GCF_002101775.1	340	Bacteria>Terrabacteria_group>Actinobacteria>Actinomycetia>Corynebacteriales	Mycolicibacter_nonchromogenicus	DNA-sulfur-modification-protein-DndB-[Mycolicibacter-nonchromogenicus]	1782
RRR45072.1	DndB-ParB-nuclease	GCA_003933725.1	381	Bacteria>Terrabacteria_group>Actinobacteria>Actinomycetia>Corynebacteriales	Mycolicibacter_terrae	DNA-sulfur-modification-protein-DndB-[Mycolicibacter-terrae]	1788
WP_215918492.1	DndB-ParB-nuclease	-	376	Bacteria>Terrabacteria_group>Actinobacteria>Actinomycetia>Corynebacteriales	Nocardia_albiluteola	DNA-sulfur-modification-protein-DndB-[Nocardia-sp.-NEAU-G5]	2842303
WP_067811376.1	DndB-ParB-nuclease	GCF_001612785.1	379	Bacteria>Terrabacteria_group>Actinobacteria>Actinomycetia>Corynebacteriales	Nocardia_beijingensis	DNA-sulfur-modification-protein-DndB-[Nocardia-beijingensis]	95162
WP_067811368.1	DndB-ParB-nuclease	GCF_001612785.1	733	Bacteria>Terrabacteria_group>Actinobacteria>Actinomycetia>Corynebacteriales	Nocardia_beijingensis	hypothetical-protein-[Nocardia-beijingensis]	95162
AFT98753.1	DndB-ParB-nuclease	GCA_000250675.3	349	Bacteria>Terrabacteria_group>Actinobacteria>Actinomycetia>Corynebacteriales	Nocardia_brasiliensis_ATCC_700358	hypothetical-protein-O3I_003955-[Nocardia-brasiliensis-ATCC-700358]	1133849
WP_051066476.1	DndB-ParB-nuclease	GCF_000250675.2	375	Bacteria>Terrabacteria_group>Actinobacteria>Actinomycetia>Corynebacteriales	Nocardia_brasiliensis	DNA-sulfur-modification-protein-DndB-[Nocardia-brasiliensis]	37326
WP_228833487.1	DndB-ParB-nuclease	GCF_015478325.1	339	Bacteria>Terrabacteria_group>Actinobacteria>Actinomycetia>Corynebacteriales	Nocardia_brasiliensis	DNA-sulfur-modification-protein-DndB-[Nocardia-brasiliensis]	37326
TLG01813.1	DndB-ParB-nuclease	GCA_005863295.1	379	Bacteria>Terrabacteria_group>Actinobacteria>Actinomycetia>Corynebacteriales	Nocardia_cyriacigeorgica	DNA-sulfur-modification-protein-DndB-[Nocardia-cyriacigeorgica]	135487
WP_138458013.1	DndB-ParB-nuclease	GCF_005863295.1	379	Bacteria>Terrabacteria_group>Actinobacteria>Actinomycetia>Corynebacteriales	Nocardia_cyriacigeorgica	DNA-sulfur-modification-protein-DndB-[Nocardia-cyriacigeorgica]	135487
MBM4694350.1	DndB-ParB-nuclease	GCA_016878985.1	374	Bacteria>Terrabacteria_group>Actinobacteria>Actinomycetia>Corynebacteriales	Rhodococcus_equi	DNA-sulfur-modification-protein-DndB-[Rhodococcus-hoagii]	43767
NKT42202.1	DndB-ParB-nuclease	GCA_012394405.1	374	Bacteria>Terrabacteria_group>Actinobacteria>Actinomycetia>Corynebacteriales	Rhodococcus_equi	DNA-sulfur-modification-protein-DndB-[Rhodococcus-hoagii]	43767
ORL10308.1	DndB-ParB-nuclease	GCA_002094295.1	374	Bacteria>Terrabacteria_group>Actinobacteria>Actinomycetia>Corynebacteriales	Rhodococcus_equi	DNA-sulfur-modification-protein-DndB-[Rhodococcus-hoagii]	43767
WP_237168071.1	DndB-ParB-nuclease	GCF_002094295.1	341	Bacteria>Terrabacteria_group>Actinobacteria>Actinomycetia>Corynebacteriales	Rhodococcus_equi	DNA-sulfur-modification-protein-DndB-[Rhodococcus-hoagii]	43767
WP_238773973.1	DndB-ParB-nuclease	GCF_022271335.1	341	Bacteria>Terrabacteria_group>Actinobacteria>Actinomycetia>Corynebacteriales	Rhodococcus_equi	DNA-sulfur-modification-protein-DndB-[Rhodococcus-hoagii]	43767
MBM4694361.1	DndB-ParB-nuclease	GCA_016878985.1	566	Bacteria>Terrabacteria_group>Actinobacteria>Actinomycetia>Corynebacteriales	Rhodococcus_equi	hypothetical-protein-[Rhodococcus-hoagii]	43767
WP_238773932.1	DndB-ParB-nuclease	GCF_022271335.1	566	Bacteria>Terrabacteria_group>Actinobacteria>Actinomycetia>Corynebacteriales	Rhodococcus_equi	hypothetical-protein-[Rhodococcus-hoagii]	43767
QIP41494.1	DndB-ParB-nuclease	GCA_011611865.1	375	Bacteria>Terrabacteria_group>Actinobacteria>Actinomycetia>Corynebacteriales	Rhodococcus_erythropolis	DNA-sulfur-modification-protein-DndB-[Rhodococcus-erythropolis]	1833
WP_166502467.1	DndB-ParB-nuclease	GCF_011611865.1	375	Bacteria>Terrabacteria_group>Actinobacteria>Actinomycetia>Corynebacteriales	Rhodococcus_erythropolis	DNA-sulfur-modification-protein-DndB-[Rhodococcus-erythropolis]	1833
WP_214646752.1	DndB-ParB-nuclease	GCF_008306195.1	697	Bacteria>Terrabacteria_group>Actinobacteria>Actinomycetia>Corynebacteriales	Rhodococcus_erythropolis_group	MULTISPECIES:-hypothetical-protein-[Rhodococcus-erythropolis-group]	2840174
MBY4111463.1	DndB-ParB-nuclease	GCA_019782405.1	375	Bacteria>Terrabacteria_group>Actinobacteria>Actinomycetia>Corynebacteriales	Rhodococcus_fascians	DNA-sulfur-modification-protein-DndB-[Rhodococcus-fascians]	1828
MBY4130976.1	DndB-ParB-nuclease	GCA_019782385.1	374	Bacteria>Terrabacteria_group>Actinobacteria>Actinomycetia>Corynebacteriales	Rhodococcus_fascians	DNA-sulfur-modification-protein-DndB-[Rhodococcus-fascians]	1828
WP_230598461.1	DndB-ParB-nuclease	GCF_000760775.1	341	Bacteria>Terrabacteria_group>Actinobacteria>Actinomycetia>Corynebacteriales	Rhodococcus_fascians	DNA-sulfur-modification-protein-DndB-[Rhodococcus-fascians]	1828
MBY4111466.1	DndB-ParB-nuclease	GCA_019782405.1	563	Bacteria>Terrabacteria_group>Actinobacteria>Actinomycetia>Corynebacteriales	Rhodococcus_fascians	hypothetical-protein-[Rhodococcus-fascians]	1828
MBS3695243.1	DndB-ParB-nuclease	GCA_018310395.1	366	Bacteria>Terrabacteria_group>Actinobacteria>Actinomycetia>Corynebacteriales	Rhodococcus_qingshengii	DNA-sulfur-modification-protein-DndB-[Rhodococcus-qingshengii]	334542
QEM26340.1	DndB-ParB-nuclease	GCA_008306195.1	393	Bacteria>Terrabacteria_group>Actinobacteria>Actinomycetia>Corynebacteriales	Rhodococcus_qingshengii	DNA-sulfur-modification-protein-DndB-[Rhodococcus-qingshengii]	334542
WP_212634075.1	DndB-ParB-nuclease	GCF_018310395.1	366	Bacteria>Terrabacteria_group>Actinobacteria>Actinomycetia>Corynebacteriales	Rhodococcus_qingshengii	DNA-sulfur-modification-protein-DndB-[Rhodococcus-qingshengii]	334542
WP_234552454.1	DndB-ParB-nuclease	GCF_008306195.1	341	Bacteria>Terrabacteria_group>Actinobacteria>Actinomycetia>Corynebacteriales	Rhodococcus_qingshengii	DNA-sulfur-modification-protein-DndB-[Rhodococcus-qingshengii]	334542
QEM31080.1	DndB-ParB-nuclease	GCA_008306195.1	755	Bacteria>Terrabacteria_group>Actinobacteria>Actinomycetia>Corynebacteriales	Rhodococcus_qingshengii	hypothetical-protein-D6M20_05890-[Rhodococcus-qingshengii]	334542
WP_234552424.1	DndB-ParB-nuclease	GCF_021457505.1	771	Bacteria>Terrabacteria_group>Actinobacteria>Actinomycetia>Corynebacteriales	Rhodococcus_qingshengii	hypothetical-protein-[Rhodococcus-qingshengii]	334542
WP_072786298.1	DndB-ParB-nuclease	GCF_001894945.1	375	Bacteria>Terrabacteria_group>Actinobacteria>Actinomycetia>Corynebacteriales	Rhodococcus_ruber	DNA-sulfur-modification-protein-DndB-[Rhodococcus-ruber]	1830
WP_064065952.1	DndB-ParB-nuclease	GCF_001894945.1	568	Bacteria>Terrabacteria_group>Actinobacteria>Actinomycetia>Corynebacteriales	Rhodococcus_ruber	hypothetical-protein-[Rhodococcus-ruber]	1830
WP_094713514.1	DndB-ParB-nuclease	GCF_002258765.1	375	Bacteria>Terrabacteria_group>Actinobacteria>Actinomycetia>Corynebacteriales	Rhodococcus_sp._06-418-1B	DNA-sulfur-modification-protein-DndB-[Rhodococcus-sp.-06-418-1B]	2022515
WP_143546977.1	DndB-ParB-nuclease	GCF_002258765.1	755	Bacteria>Terrabacteria_group>Actinobacteria>Actinomycetia>Corynebacteriales	Rhodococcus_sp._06-418-1B	hypothetical-protein-[Rhodococcus-sp.-06-418-1B]	2022515
WP_237669127.1	DndB-ParB-nuclease	GCF_000813105.1	341	Bacteria>Terrabacteria_group>Actinobacteria>Actinomycetia>Corynebacteriales	Rhodococcus_sp._BS-15	DNA-sulfur-modification-protein-DndB-[Rhodococcus-sp.-BS-15]	1304954
WP_229567705.1	DndB-ParB-nuclease	GCF_020885655.1	375	Bacteria>Terrabacteria_group>Actinobacteria>Actinomycetia>Corynebacteriales	Rhodococcus_sp._RDE2	DNA-sulfur-modification-protein-DndB-[Rhodococcus-sp.-RDE2]	2885078
BDB59763.1	DndB-ParB-nuclease	GCA_020885655.1	360	Bacteria>Terrabacteria_group>Actinobacteria>Actinomycetia>Corynebacteriales	Rhodococcus_sp._RDE2	hypothetical-protein-RDE2_15570-[Rhodococcus-sp.-RDE2]	2885078
BDB59769.1	DndB-ParB-nuclease	GCA_020885655.1	745	Bacteria>Terrabacteria_group>Actinobacteria>Actinomycetia>Corynebacteriales	Rhodococcus_sp._RDE2	hypothetical-protein-RDE2_15630-[Rhodococcus-sp.-RDE2]	2885078
WP_229567713.1	DndB-ParB-nuclease	GCF_020885655.1	685	Bacteria>Terrabacteria_group>Actinobacteria>Actinomycetia>Corynebacteriales	Rhodococcus_sp._RDE2	hypothetical-protein-[Rhodococcus-sp.-RDE2]	2885078
WP_094643707.1	DndB-ParB-nuclease	GCF_002258975.1	375	Bacteria>Terrabacteria_group>Actinobacteria>Actinomycetia>Corynebacteriales	unclassified_Rhodococcus	MULTISPECIES:-DNA-sulfur-modification-protein-DndB-[unclassified-Rhodococcus]	192944
WP_128645940.1	DndB-ParB-nuclease	GCF_000813105.1	736	Bacteria>Terrabacteria_group>Actinobacteria>Actinomycetia>Corynebacteriales	unclassified_Rhodococcus	MULTISPECIES:-hypothetical-protein-[unclassified-Rhodococcus]	192944
WP_206442854.1	DndB-ParB-nuclease	-	401	Bacteria>Terrabacteria_group>Actinobacteria>Actinomycetia>Frankiales	Candidatus_Frankia_datiscae	DNA-sulfur-modification-protein-DndB-[Candidatus-Frankia-datiscae]	2716812
WP_066070325.1	DndB-ParB-nuclease	-	425	Bacteria>Terrabacteria_group>Actinobacteria>Actinomycetia>Frankiales	Frankia_sp._EI5c	DGQHR-domain-containing-protein-[Frankia-sp.-EI5c]	683316
WP_083986929.1	DndB-ParB-nuclease	GCF_001636565.1	384	Bacteria>Terrabacteria_group>Actinobacteria>Actinomycetia>Frankiales	Frankia_sp._EI5c	DNA-sulfur-modification-protein-DndB-[Frankia-sp.-EI5c]	683316
SFP89448.1	DndB-ParB-nuclease	GCA_900115505.1	410	Bacteria>Terrabacteria_group>Actinobacteria>Actinomycetia>Geodermatophilales	Geodermatophilus_dictyosporus	DNA-sulfur-modification-protein-DndB-[Geodermatophilus-dictyosporus]	1523247
WP_169064610.1	DndB-ParB-nuclease	GCF_900115505.1	369	Bacteria>Terrabacteria_group>Actinobacteria>Actinomycetia>Geodermatophilales	Geodermatophilus_dictyosporus	DNA-sulfur-modification-protein-DndB-[Geodermatophilus-dictyosporus]	1523247
WP_170149191.1	DndB-ParB-nuclease	GCF_003182485.1	428	Bacteria>Terrabacteria_group>Actinobacteria>Actinomycetia>Geodermatophilales	Geodermatophilus_normandii	DGQHR-domain-containing-protein-[Geodermatophilus-normandii]	1137989
WP_170149192.1	DndB-ParB-nuclease	GCF_003182485.1	427	Bacteria>Terrabacteria_group>Actinobacteria>Actinomycetia>Geodermatophilales	Geodermatophilus_normandii	DGQHR-domain-containing-protein-[Geodermatophilus-normandii]	1137989
PWW23477.1	DndB-ParB-nuclease	GCA_003182485.1	417	Bacteria>Terrabacteria_group>Actinobacteria>Actinomycetia>Geodermatophilales	Geodermatophilus_normandii	DNA-sulfur-modification-protein-DndB-[Geodermatophilus-normandii]	1137989
PWW23479.1	DndB-ParB-nuclease	GCA_003182485.1	438	Bacteria>Terrabacteria_group>Actinobacteria>Actinomycetia>Geodermatophilales	Geodermatophilus_normandii	DNA-sulfur-modification-protein-DndB-[Geodermatophilus-normandii]	1137989
WP_211307957.1	DndB-ParB-nuclease	GCF_003182485.1	408	Bacteria>Terrabacteria_group>Actinobacteria>Actinomycetia>Geodermatophilales	Geodermatophilus_normandii	DNA-sulfur-modification-protein-DndB-[Geodermatophilus-normandii]	1137989
WP_097207110.1	DndB-ParB-nuclease	GCF_014191795.1	379	Bacteria>Terrabacteria_group>Actinobacteria>Actinomycetia>Geodermatophilales	Geodermatophilus_sabuli	DNA-sulfur-modification-protein-DndB-[Geodermatophilus-sabuli]	1564158
WP_197683604.1	DndB-ParB-nuclease	GCF_001005145.1	383	Bacteria>Terrabacteria_group>Actinobacteria>Actinomycetia>Jiangellales	Jiangella_alkaliphila	DNA-sulfur-modification-protein-DndB-[Jiangella-alkaliphila]	419479
MBK8078526.1	DndB-ParB-nuclease	GCA_016713225.1	363	Bacteria>Terrabacteria_group>Actinobacteria>Actinomycetia>Kineosporiales	Kineosporiaceae_bacterium	DNA-sulfur-modification-protein-DndB-[Kineosporiaceae-bacterium]	2487580
MCD5341434.1	DndB-ParB-nuclease	GCA_021129155.1	384	Bacteria>Terrabacteria_group>Actinobacteria>Actinomycetia>Micrococcales	Arthrobacter_sp._AK04	DNA-sulfur-modification-protein-DndB-[Arthrobacter-sp.-AK04]	2900048
TJY69473.1	DndB-ParB-nuclease	GCA_005047635.1	384	Bacteria>Terrabacteria_group>Actinobacteria>Actinomycetia>Micrococcales	Arthrobacter_sp._CAU_1506	DNA-sulfur-modification-protein-DndB-[Arthrobacter-sp.-CAU-1506]	2560052
WP_136786208.1	DndB-ParB-nuclease	GCF_005047635.1	384	Bacteria>Terrabacteria_group>Actinobacteria>Actinomycetia>Micrococcales	Arthrobacter_sp._CAU_1506	DNA-sulfur-modification-protein-DndB-[Arthrobacter-sp.-CAU-1506]	2560052
QYF91071.1	DndB-ParB-nuclease	GCA_019443425.1	166	Bacteria>Terrabacteria_group>Actinobacteria>Actinomycetia>Micrococcales	Arthrobacter_sp._PAMC25284	DGQHR-domain-containing-protein-[Arthrobacter-sp.-PAMC25284]	2861279
QYF91072.1	DndB-ParB-nuclease	GCA_019443425.1	214	Bacteria>Terrabacteria_group>Actinobacteria>Actinomycetia>Micrococcales	Arthrobacter_sp._PAMC25284	DGQHR-domain-containing-protein-[Arthrobacter-sp.-PAMC25284]	2861279
WP_219886708.1	DndB-ParB-nuclease	GCF_019443425.1	166	Bacteria>Terrabacteria_group>Actinobacteria>Actinomycetia>Micrococcales	Arthrobacter_sp._PAMC25284	DGQHR-domain-containing-protein-[Arthrobacter-sp.-PAMC25284]	2861279
WP_219886709.1	DndB-ParB-nuclease	GCF_019443425.1	214	Bacteria>Terrabacteria_group>Actinobacteria>Actinomycetia>Micrococcales	Arthrobacter_sp._PAMC25284	DGQHR-domain-containing-protein-[Arthrobacter-sp.-PAMC25284]	2861279
MBD1591589.1	DndB-ParB-nuclease	GCA_014694505.1	384	Bacteria>Terrabacteria_group>Actinobacteria>Actinomycetia>Micrococcales	Arthrobacter_sp._S1_S22	DNA-sulfur-modification-protein-DndB-[Arthrobacter-sp.-S1_S22]	2720222
MBD1591587.1	DndB-ParB-nuclease	GCA_014694505.1	536	Bacteria>Terrabacteria_group>Actinobacteria>Actinomycetia>Micrococcales	Arthrobacter_sp._S1_S22	hypothetical-protein-[Arthrobacter-sp.-S1_S22]	2720222
WP_224094976.1	DndB-ParB-nuclease	GCF_019977255.1	381	Bacteria>Terrabacteria_group>Actinobacteria>Actinomycetia>Micrococcales	Arthrobacter_sp._StoSoilB13	DNA-sulfur-modification-protein-DndB-[Arthrobacter-sp.-StoSoilB13]	2830993
BCW52043.1	DndB-ParB-nuclease	GCA_019977255.1	381	Bacteria>Terrabacteria_group>Actinobacteria>Actinomycetia>Micrococcales	Arthrobacter_sp._StoSoilB13	hypothetical-protein-StoSoilB13_43850-[Arthrobacter-sp.-StoSoilB13]	2830993
WP_134374322.1	DndB-ParB-nuclease	GCF_004403415.1	434	Bacteria>Terrabacteria_group>Actinobacteria>Actinomycetia>Micrococcales	Cryobacterium_sp._TMT2-16	hypothetical-protein-[Cryobacterium-sp.-TMT2-16]	1259247
WP_062526636.1	DndB-ParB-nuclease	GCF_000971435.1	374	Bacteria>Terrabacteria_group>Actinobacteria>Actinomycetia>Micrococcales	Demequina_rhizosphaerae	DNA-sulfur-modification-protein-DndB-[Demequina-rhizosphaerae]	1638985
WP_156165364.1	DndB-ParB-nuclease	GCF_000971435.1	561	Bacteria>Terrabacteria_group>Actinobacteria>Actinomycetia>Micrococcales	Demequina_rhizosphaerae	hypothetical-protein-[Demequina-rhizosphaerae]	1638985
MBB4140423.1	DndB-ParB-nuclease	GCA_014197265.1	378	Bacteria>Terrabacteria_group>Actinobacteria>Actinomycetia>Micrococcales	Microbacterium_invictum	DNA-sulfur-modification-protein-DndB-[Microbacterium-invictum]	515415
WP_183499954.1	DndB-ParB-nuclease	GCF_015278285.1	378	Bacteria>Terrabacteria_group>Actinobacteria>Actinomycetia>Micrococcales	Microbacterium_invictum	DNA-sulfur-modification-protein-DndB-[Microbacterium-invictum]	515415
HAS32869.1	DndB-ParB-nuclease	GCA_003483835.1	377	Bacteria>Terrabacteria_group>Actinobacteria>Actinomycetia>Micrococcales	Microbacterium_sp.	DNA-sulfur-modification-protein-DndB-[Microbacterium-sp.]	51671
MAM53482.1	DndB-ParB-nuclease	GCA_002695205.1	377	Bacteria>Terrabacteria_group>Actinobacteria>Actinomycetia>Micrococcales	Microbacterium_sp.	DNA-sulfur-modification-protein-DndB-[Microbacterium-sp.]	51671
WP_242133992.1	DndB-ParB-nuclease	GCF_022668885.1	384	Bacteria>Terrabacteria_group>Actinobacteria>Actinomycetia>Micrococcales	Sediminivirga_luteola	DNA-sulfur-modification-protein-DndB-[Sediminivirga-luteola]	1774748
MBB5871900.1	DndB-ParB-nuclease	GCA_014204945.1	388	Bacteria>Terrabacteria_group>Actinobacteria>Actinomycetia>Micromonosporales	Allocatelliglobosispora_scoriae	DNA-sulfur-modification-protein-DndB-[Allocatelliglobosispora-scoriae]	643052
WP_184841264.1	DndB-ParB-nuclease	-	363	Bacteria>Terrabacteria_group>Actinobacteria>Actinomycetia>Micromonosporales	Allocatelliglobosispora_scoriae	DNA-sulfur-modification-protein-DndB-[Allocatelliglobosispora-scoriae]	643052
MBB5871897.1	DndB-ParB-nuclease	GCA_014204945.1	564	Bacteria>Terrabacteria_group>Actinobacteria>Actinomycetia>Micromonosporales	Allocatelliglobosispora_scoriae	hypothetical-protein-[Allocatelliglobosispora-scoriae]	643052
WP_184840257.1	DndB-ParB-nuclease	GCF_014204945.1	564	Bacteria>Terrabacteria_group>Actinobacteria>Actinomycetia>Micromonosporales	Allocatelliglobosispora_scoriae	hypothetical-protein-[Allocatelliglobosispora-scoriae]	643052
WP_091434189.1	DndB-ParB-nuclease	GCF_014645775.1	379	Bacteria>Terrabacteria_group>Actinobacteria>Actinomycetia>Micromonosporales	Micromonospora_yangpuensis	DNA-sulfur-modification-protein-DndB-[Micromonospora-yangpuensis]	683228
WP_139135572.1	DndB-ParB-nuclease	GCF_014645775.1	579	Bacteria>Terrabacteria_group>Actinobacteria>Actinomycetia>Micromonosporales	Micromonospora_yangpuensis	hypothetical-protein-[Micromonospora-yangpuensis]	683228
SDO34674.1	DndB-ParB-nuclease	GCA_900104535.1	385	Bacteria>Terrabacteria_group>Actinobacteria>Actinomycetia>Nakamurellales	Nakamurella_panacisegetis	DNA-sulfur-modification-protein-DndB-[Nakamurella-panacisegetis]	1090615
SDO35026.1	DndB-ParB-nuclease	GCA_900104535.1	438	Bacteria>Terrabacteria_group>Actinobacteria>Actinomycetia>Nakamurellales	Nakamurella_panacisegetis	DNA-sulfur-modification-protein-DndB-[Nakamurella-panacisegetis]	1090615
SDO35060.1	DndB-ParB-nuclease	GCA_900104535.1	461	Bacteria>Terrabacteria_group>Actinobacteria>Actinomycetia>Nakamurellales	Nakamurella_panacisegetis	DNA-sulfur-modification-protein-DndB-[Nakamurella-panacisegetis]	1090615
WP_090481183.1	DndB-ParB-nuclease	GCF_900104535.1	378	Bacteria>Terrabacteria_group>Actinobacteria>Actinomycetia>Nakamurellales	Nakamurella_panacisegetis	DNA-sulfur-modification-protein-DndB-[Nakamurella-panacisegetis]	1090615
SDT34934.1	DndB-ParB-nuclease	GCA_900105065.1	749	Bacteria>Terrabacteria_group>Actinobacteria>Actinomycetia>Propionibacteriales	Friedmanniella_luteola	DNA-sulfur-modification-associated-[Friedmanniella-luteola]	546871
SDT34869.1	DndB-ParB-nuclease	GCA_900105065.1	369	Bacteria>Terrabacteria_group>Actinobacteria>Actinomycetia>Propionibacteriales	Friedmanniella_luteola	DNA-sulfur-modification-protein-DndB-[Friedmanniella-luteola]	546871
WP_197677102.1	DndB-ParB-nuclease	GCF_900105065.1	377	Bacteria>Terrabacteria_group>Actinobacteria>Actinomycetia>Propionibacteriales	Friedmanniella_luteola	DNA-sulfur-modification-protein-DndB-[Friedmanniella-luteola]	546871
WP_197677103.1	DndB-ParB-nuclease	GCF_900105065.1	747	Bacteria>Terrabacteria_group>Actinobacteria>Actinomycetia>Propionibacteriales	Friedmanniella_luteola	hypothetical-protein-[Friedmanniella-luteola]	546871
WP_209692271.1	DndB-ParB-nuclease	GCF_017876295.1	406	Bacteria>Terrabacteria_group>Actinobacteria>Actinomycetia>Propionibacteriales	Kribbella_aluminosa	DNA-sulfur-modification-protein-DndB-[Kribbella-aluminosa]	416017
QNE18709.1	DndB-ParB-nuclease	GCA_014217565.1	365	Bacteria>Terrabacteria_group>Actinobacteria>Actinomycetia>Propionibacteriales	Kribbella_qitaiheensis	DNA-sulfur-modification-protein-DndB-[Kribbella-qitaiheensis]	1544730
WP_185448008.1	DndB-ParB-nuclease	GCF_014217565.1	365	Bacteria>Terrabacteria_group>Actinobacteria>Actinomycetia>Propionibacteriales	Kribbella_qitaiheensis	DNA-sulfur-modification-protein-DndB-[Kribbella-qitaiheensis]	1544730
WP_185447998.1	DndB-ParB-nuclease	GCF_014217565.1	337	Bacteria>Terrabacteria_group>Actinobacteria>Actinomycetia>Propionibacteriales	Kribbella_qitaiheensis	hypothetical-protein-[Kribbella-qitaiheensis]	1544730
MCB0909928.1	DndB-ParB-nuclease	GCA_020438665.1	385	Bacteria>Terrabacteria_group>Actinobacteria>Actinomycetia>Propionibacteriales	Nocardioidaceae_bacterium	DNA-sulfur-modification-protein-DndB-[Nocardioidaceae-bacterium]	1871072
WP_114423083.1	DndB-ParB-nuclease	GCF_003333285.1	384	Bacteria>Terrabacteria_group>Actinobacteria>Actinomycetia>Propionibacteriales	Nocardioides_houyundeii	DNA-sulfur-modification-protein-DndB-[Nocardioides-houyundeii]	2045452
WP_162794435.1	DndB-ParB-nuclease	GCF_003333285.1	509	Bacteria>Terrabacteria_group>Actinobacteria>Actinomycetia>Propionibacteriales	Nocardioides_houyundeii	hypothetical-protein-[Nocardioides-houyundeii]	2045452
WP_206056813.1	DndB-ParB-nuclease	GCF_004919105.1	374	Bacteria>Terrabacteria_group>Actinobacteria>Actinomycetia>Propionibacteriales	Nocardioides_sp._GY_10113	DNA-sulfur-modification-protein-DndB-[Nocardioides-sp.-GY-10113]	2569761
MCC2591822.1	DndB-ParB-nuclease	GCA_020687685.1	386	Bacteria>Terrabacteria_group>Actinobacteria>Actinomycetia>Propionibacteriales	Tessaracoccus_sp._OS52	DNA-sulfur-modification-protein-DndB-[Tessaracoccus-sp.-OS52]	2886691
WP_227734866.1	DndB-ParB-nuclease	-	386	Bacteria>Terrabacteria_group>Actinobacteria>Actinomycetia>Propionibacteriales	Tessaracoccus_sp._OS52	DNA-sulfur-modification-protein-DndB-[Tessaracoccus-sp.-OS52]	2886691
RLK59599.1	DndB-ParB-nuclease	GCA_003663795.1	383	Bacteria>Terrabacteria_group>Actinobacteria>Actinomycetia>Pseudonocardiales	Actinokineospora_cianjurensis	DNA-sulfur-modification-protein-DndB-[Actinokineospora-cianjurensis]	585224
WP_211346404.1	DndB-ParB-nuclease	GCF_003663795.1	387	Bacteria>Terrabacteria_group>Actinobacteria>Actinomycetia>Pseudonocardiales	Actinokineospora_cianjurensis	DNA-sulfur-modification-protein-DndB-[Actinokineospora-cianjurensis]	585224
WP_110079050.1	DndB-ParB-nuclease	GCF_003182415.1	385	Bacteria>Terrabacteria_group>Actinobacteria>Actinomycetia>Pseudonocardiales	Actinokineospora_spheciospongiae	DNA-sulfur-modification-protein-DndB-[Actinokineospora-mzabensis]	909613
WP_110079047.1	DndB-ParB-nuclease	GCF_003182415.1	743	Bacteria>Terrabacteria_group>Actinobacteria>Actinomycetia>Pseudonocardiales	Actinokineospora_spheciospongiae	hypothetical-protein-[Actinokineospora-mzabensis]	909613
WP_133909215.1	DndB-ParB-nuclease	GCF_004364325.1	385	Bacteria>Terrabacteria_group>Actinobacteria>Actinomycetia>Pseudonocardiales	Actinophytocola_oryzae	DNA-sulfur-modification-protein-DndB-[Actinophytocola-oryzae]	502181
WP_191306964.1	DndB-ParB-nuclease	GCF_014654205.1	384	Bacteria>Terrabacteria_group>Actinobacteria>Actinomycetia>Pseudonocardiales	Amycolatopsis_bullii	DNA-sulfur-modification-protein-DndB-[Amycolatopsis-bullii]	941987
GHF98397.1	DndB-ParB-nuclease	GCA_014654205.1	384	Bacteria>Terrabacteria_group>Actinobacteria>Actinomycetia>Pseudonocardiales	Amycolatopsis_bullii	hypothetical-protein-GCM10017567_11370-[Amycolatopsis-bullii]	941987
WP_084098561.1	DndB-ParB-nuclease	GCF_000732925.1	384	Bacteria>Terrabacteria_group>Actinobacteria>Actinomycetia>Pseudonocardiales	Amycolatopsis_japonica	DNA-sulfur-modification-protein-DndB-[Amycolatopsis-japonica]	208439
AIG73614.1	DndB-ParB-nuclease	GCA_000732925.1	384	Bacteria>Terrabacteria_group>Actinobacteria>Actinomycetia>Pseudonocardiales	Amycolatopsis_japonica	Hypothetical-protein-AJAP_03440-[Amycolatopsis-japonica]	208439
WP_148311444.1	DndB-ParB-nuclease	GCF_000732925.1	566	Bacteria>Terrabacteria_group>Actinobacteria>Actinomycetia>Pseudonocardiales	Amycolatopsis_japonica	hypothetical-protein-[Amycolatopsis-japonica]	208439
WP_156096169.1	DndB-ParB-nuclease	GCF_000717335.1	383	Bacteria>Terrabacteria_group>Actinobacteria>Actinomycetia>Pseudonocardiales	Amycolatopsis_jejuensis	DNA-sulfur-modification-protein-DndB-[Amycolatopsis-jejuensis]	330084
WP_084144692.1	DndB-ParB-nuclease	GCF_000717335.1	726	Bacteria>Terrabacteria_group>Actinobacteria>Actinomycetia>Pseudonocardiales	Amycolatopsis_jejuensis	hypothetical-protein-[Amycolatopsis-jejuensis]	330084
WP_106965009.1	DndB-ParB-nuclease	GCF_000411975.1	383	Bacteria>Terrabacteria_group>Actinobacteria>Actinomycetia>Pseudonocardiales	Amycolatopsis_orientalis	DNA-sulfur-modification-protein-DndB-[Amycolatopsis-orientalis]	31958
WP_037364638.1	DndB-ParB-nuclease	GCF_000411975.1	726	Bacteria>Terrabacteria_group>Actinobacteria>Actinomycetia>Pseudonocardiales	Amycolatopsis_orientalis	hypothetical-protein-[Amycolatopsis-orientalis]	31958

Accession	Annotation	GCA	Length	Phyletic.spread	Strain.name	Defline	Species.Taxid
OXM65311.1	DndB-ParB-nuclease	GCA_002234595.1	379	Bacteria>Terrabacteria_group>Actinobacteria>Actinomycetia>Pseudonocardiales	Amycolatopsis_vastitatis	DNA-sulfur-modification-protein-DndB-[Amycolatopsis-vastitatis]	1905142
WP_093949716.1	DndB-ParB-nuclease	GCF_002234595.1	379	Bacteria>Terrabacteria_group>Actinobacteria>Actinomycetia>Pseudonocardiales	Amycolatopsis_vastitatis	DNA-sulfur-modification-protein-DndB-[Amycolatopsis-vastitatis]	1905142
WP_084037447.1	DndB-ParB-nuclease	GCF_000504245.1	383	Bacteria>Terrabacteria_group>Actinobacteria>Actinomycetia>Pseudonocardiales	Haloechinothrix_halophila	DNA-sulfur-modification-protein-DndB-[Haloechinothrix-halophila]	1069073
WP_218589625.1	DndB-ParB-nuclease	GCF_019263625.1	427	Bacteria>Terrabacteria_group>Actinobacteria>Actinomycetia>Pseudonocardiales	Pseudonocardia_oceani	DGQHR-domain-containing-protein-[Pseudonocardia-oceani]	2792013
WP_218589620.1	DndB-ParB-nuclease	GCF_019263585.2	407	Bacteria>Terrabacteria_group>Actinobacteria>Actinomycetia>Pseudonocardiales	Pseudonocardia_oceani	DNA-sulfur-modification-protein-DndB-[Pseudonocardia-oceani]	2792013
WP_225922793.1	DndB-ParB-nuclease	GCF_019263625.1	404	Bacteria>Terrabacteria_group>Actinobacteria>Actinomycetia>Pseudonocardiales	Pseudonocardia_oceani	DNA-sulfur-modification-protein-DndB-[Pseudonocardia-oceani]	2792013
WP_226355083.1	DndB-ParB-nuclease	GCF_020496245.1	376	Bacteria>Terrabacteria_group>Actinobacteria>Actinomycetia>Pseudonocardiales	Pseudonocardia_sp._ICBG601	DNA-sulfur-modification-protein-DndB-[Pseudonocardia-sp.-ICBG601]	2846759
WP_226355088.1	DndB-ParB-nuclease	GCF_020496245.1	719	Bacteria>Terrabacteria_group>Actinobacteria>Actinomycetia>Pseudonocardiales	Pseudonocardia_sp._ICBG601	hypothetical-protein-[Pseudonocardia-sp.-ICBG601]	2846759
MBB5957416.1	DndB-ParB-nuclease	GCA_014203665.1	375	Bacteria>Terrabacteria_group>Actinobacteria>Actinomycetia>Pseudonocardiales	Saccharothrix_tamanrassetensis	DNA-sulfur-modification-protein-DndB-[Saccharothrix-tamanrassetensis]	1051531
WP_184692501.1	DndB-ParB-nuclease	-	375	Bacteria>Terrabacteria_group>Actinobacteria>Actinomycetia>Pseudonocardiales	Saccharothrix_tamanrassetensis	DNA-sulfur-modification-protein-DndB-[Saccharothrix-tamanrassetensis]	1051531
WP_020555789.1	DndB-ParB-nuclease	GCF_000372745.1	377	Bacteria>Terrabacteria_group>Actinobacteria>Actinomycetia>Streptomycetales	Embleya_scabrispora	DNA-sulfur-modification-protein-DndB-[Embleya-scabrispora]	159449
WP_026219300.1	DndB-ParB-nuclease	GCF_000372745.1	534	Bacteria>Terrabacteria_group>Actinobacteria>Actinomycetia>Streptomycetales	Embleya_scabrispora	MULTISPECIES:-DGQHR-domain-containing-protein-[Streptomycetaceae]	159449
MCC9309127.1	DndB-ParB-nuclease	GCA_020907985.1	534	Bacteria>Terrabacteria_group>Actinobacteria>Actinomycetia>Streptomycetales	Kitasatospora_humi	DGQHR-domain-containing-protein-[Kitasatospora-sp.-RB6PN24]	2893891
WP_230052440.1	DndB-ParB-nuclease	GCF_020907985.1	534	Bacteria>Terrabacteria_group>Actinobacteria>Actinomycetia>Streptomycetales	Kitasatospora_humi	DGQHR-domain-containing-protein-[Kitasatospora-sp.-RB6PN24]	2893891
MCC9309121.1	DndB-ParB-nuclease	GCA_020907985.1	377	Bacteria>Terrabacteria_group>Actinobacteria>Actinomycetia>Streptomycetales	Kitasatospora_humi	DNA-sulfur-modification-protein-DndB-[Kitasatospora-sp.-RB6PN24]	2893891
WP_230052434.1	DndB-ParB-nuclease	-	377	Bacteria>Terrabacteria_group>Actinobacteria>Actinomycetia>Streptomycetales	Kitasatospora_humi	DNA-sulfur-modification-protein-DndB-[Kitasatospora-sp.-RB6PN24]	2893891
WP_073818554.1	DndB-ParB-nuclease	GCF_001905465.1	377	Bacteria>Terrabacteria_group>Actinobacteria>Actinomycetia>Streptomycetales	Kitasatospora_sp._CB01950	DNA-sulfur-modification-protein-DndB-[Kitasatospora-sp.-CB01950]	1703930
AXI78539.1	DndB-ParB-nuclease	GCA_003258605.2	376	Bacteria>Terrabacteria_group>Actinobacteria>Actinomycetia>Streptomycetales	Streptacidiphilus_bronchialis	DNA-sulfur-modification-protein-DndB-[Streptacidiphilus-bronchialis]	2126346
WP_111488674.1	DndB-ParB-nuclease	GCF_003258605.2	376	Bacteria>Terrabacteria_group>Actinobacteria>Actinomycetia>Streptomycetales	Streptacidiphilus_bronchialis	DNA-sulfur-modification-protein-DndB-[Streptacidiphilus-bronchialis]	2126346
AXI78528.1	DndB-ParB-nuclease	GCA_003258605.2	725	Bacteria>Terrabacteria_group>Actinobacteria>Actinomycetia>Streptomycetales	Streptacidiphilus_bronchialis	hypothetical-protein-C7M71_014915-[Streptacidiphilus-bronchialis]	2126346
WP_229758734.1	DndB-ParB-nuclease	GCF_003258605.2	694	Bacteria>Terrabacteria_group>Actinobacteria>Actinomycetia>Streptomycetales	Streptacidiphilus_bronchialis	hypothetical-protein-[Streptacidiphilus-bronchialis]	2126346
WP_236060474.1	DndB-ParB-nuclease	GCF_016918855.1	339	Bacteria>Terrabacteria_group>Actinobacteria>Actinomycetia>Streptomycetales	Streptomyces_acididurans	DNA-sulfur-modification-protein-DndB-[Streptomyces-acididurans]	2784346
WP_205357131.1	DndB-ParB-nuclease	GCF_016918855.1	593	Bacteria>Terrabacteria_group>Actinobacteria>Actinomycetia>Streptomycetales	Streptomyces_acididurans	hypothetical-protein-[Streptomyces-acididurans]	2784346
MPY33409.1	DndB-ParB-nuclease	GCA_009377175.1	379	Bacteria>Terrabacteria_group>Actinobacteria>Actinomycetia>Streptomycetales	Streptomyces_adustus	DNA-sulfur-modification-protein-DndB-[Streptomyces-adustus]	1609272
WP_238426833.1	DndB-ParB-nuclease	GCF_009377175.1	339	Bacteria>Terrabacteria_group>Actinobacteria>Actinomycetia>Streptomycetales	Streptomyces_adustus	DNA-sulfur-modification-protein-DndB-[Streptomyces-adustus]	1609272
MPY33413.1	DndB-ParB-nuclease	GCA_009377175.1	727	Bacteria>Terrabacteria_group>Actinobacteria>Actinomycetia>Streptomycetales	Streptomyces_adustus	hypothetical-protein-[Streptomyces-adustus]	1609272
WP_238426821.1	DndB-ParB-nuclease	GCF_009377175.1	724	Bacteria>Terrabacteria_group>Actinobacteria>Actinomycetia>Streptomycetales	Streptomyces_adustus	hypothetical-protein-[Streptomyces-adustus]	1609272
WP_086562781.1	DndB-ParB-nuclease	-	377	Bacteria>Terrabacteria_group>Actinobacteria>Actinomycetia>Streptomycetales	Streptomyces_africanus	DNA-sulfur-modification-protein-DndB-[Streptomyces-africanus]	231024
WP_093838495.1	DndB-ParB-nuclease	GCF_900112355.1	377	Bacteria>Terrabacteria_group>Actinobacteria>Actinomycetia>Streptomycetales	Streptomyces_aidingensis	DNA-sulfur-modification-protein-DndB-[Streptomyces-aidingensis]	910347
WP_093838501.1	DndB-ParB-nuclease	-	750	Bacteria>Terrabacteria_group>Actinobacteria>Actinomycetia>Streptomycetales	Streptomyces_aidingensis	hypothetical-protein-[Streptomyces-aidingensis]	910347
RZE12948.1	DndB-ParB-nuclease	GCA_004195995.1	379	Bacteria>Terrabacteria_group>Actinobacteria>Actinomycetia>Streptomycetales	Streptomyces_albidoflavus	DNA-sulfur-modification-protein-DndB-[Streptomyces-albidoflavus]	1886
RZE59664.1	DndB-ParB-nuclease	GCA_004195845.1	379	Bacteria>Terrabacteria_group>Actinobacteria>Actinomycetia>Streptomycetales	Streptomyces_albidoflavus	DNA-sulfur-modification-protein-DndB-[Streptomyces-albidoflavus]	1886
WP_129834394.1	DndB-ParB-nuclease	GCF_004195845.1	379	Bacteria>Terrabacteria_group>Actinobacteria>Actinomycetia>Streptomycetales	Streptomyces_albidoflavus	DNA-sulfur-modification-protein-DndB-[Streptomyces-albidoflavus]	1886
WP_233656939.1	DndB-ParB-nuclease	GCF_021366495.1	379	Bacteria>Terrabacteria_group>Actinobacteria>Actinomycetia>Streptomycetales	Streptomyces_albulus	DNA-sulfur-modification-protein-DndB-[Streptomyces-albulus]	68570
WP_233656935.1	DndB-ParB-nuclease	GCF_021366495.1	568	Bacteria>Terrabacteria_group>Actinobacteria>Actinomycetia>Streptomycetales	Streptomyces_albulus	hypothetical-protein-[Streptomyces-albulus]	68570
KUN56169.1	DndB-ParB-nuclease	GCA_001514135.1	379	Bacteria>Terrabacteria_group>Actinobacteria>Actinomycetia>Streptomycetales	Streptomyces_avermitilis	DNA-sulfur-modification-protein-DndB-[Streptomyces-avermitilis]	33903
WP_010984364.1	DndB-ParB-nuclease	GCF_000009765.2	379	Bacteria>Terrabacteria_group>Actinobacteria>Actinomycetia>Streptomycetales	Streptomyces_avermitilis	DNA-sulfur-modification-protein-DndB-[Streptomyces-avermitilis]	33903
KUN56159.1	DndB-ParB-nuclease	GCA_001514135.1	371	Bacteria>Terrabacteria_group>Actinobacteria>Actinomycetia>Streptomycetales	Streptomyces_avermitilis	hypothetical-protein-AQJ43_00635-[Streptomyces-avermitilis]	33903
GDY62784.1	DndB-ParB-nuclease	-	567	Bacteria>Terrabacteria_group>Actinobacteria>Actinomycetia>Streptomycetales	Streptomyces_avermitilis	hypothetical-protein-SAV14893_021770-[Streptomyces-avermitilis]	33903
GDY62793.1	DndB-ParB-nuclease	-	379	Bacteria>Terrabacteria_group>Actinobacteria>Actinomycetia>Streptomycetales	Streptomyces_avermitilis	hypothetical-protein-SAV14893_021860-[Streptomyces-avermitilis]	33903
GDY77080.1	DndB-ParB-nuclease	-	379	Bacteria>Terrabacteria_group>Actinobacteria>Actinomycetia>Streptomycetales	Streptomyces_avermitilis	hypothetical-protein-SAV31267_065650-[Streptomyces-avermitilis]	33903
GDY77089.1	DndB-ParB-nuclease	-	567	Bacteria>Terrabacteria_group>Actinobacteria>Actinomycetia>Streptomycetales	Streptomyces_avermitilis	hypothetical-protein-SAV31267_065740-[Streptomyces-avermitilis]	33903
GDY85993.1	DndB-ParB-nuclease	-	379	Bacteria>Terrabacteria_group>Actinobacteria>Actinomycetia>Streptomycetales	Streptomyces_avermitilis	hypothetical-protein-SAVCW2_51920-[Streptomyces-avermitilis]	33903
GDY86003.1	DndB-ParB-nuclease	-	567	Bacteria>Terrabacteria_group>Actinobacteria>Actinomycetia>Streptomycetales	Streptomyces_avermitilis	hypothetical-protein-SAVCW2_52020-[Streptomyces-avermitilis]	33903
BBJ50762.1	DndB-ParB-nuclease	-	567	Bacteria>Terrabacteria_group>Actinobacteria>Actinomycetia>Streptomycetales	Streptomyces_avermitilis	hypothetical-protein-SAVMC3_33910-[Streptomyces-avermitilis]	33903
BBJ50771.1	DndB-ParB-nuclease	-	338	Bacteria>Terrabacteria_group>Actinobacteria>Actinomycetia>Streptomycetales	Streptomyces_avermitilis	hypothetical-protein-SAVMC3_34000-[Streptomyces-avermitilis]	33903
BAC70636.1	DndB-ParB-nuclease	GCA_000009765.2	602	Bacteria>Terrabacteria_group>Actinobacteria>Actinomycetia>Streptomycetales	Streptomyces_avermitilis_MA-4680_=_NBRC_14893	hypothetical-protein-SAVERM_2925-[Streptomyces-avermitilis-MA-4680-=-NBRC-14893]	227882
BAC70643.2	DndB-ParB-nuclease	GCA_000009765.2	339	Bacteria>Terrabacteria_group>Actinobacteria>Actinomycetia>Streptomycetales	Streptomyces_avermitilis_MA-4680_=_NBRC_14893	putative-DNA-modification-(ATPase)-[Streptomyces-avermitilis-MA-4680-=-NBRC-14893]	227882
GAP46077.1	DndB-ParB-nuclease	GCA_001270025.1	376	Bacteria>Terrabacteria_group>Actinobacteria>Actinomycetia>Streptomycetales	Streptomyces_azureus	DNA-sulfur-modification-protein-DndB-[Streptomyces-azureus]	146537
WP_099054519.1	DndB-ParB-nuclease	GCF_001270025.1	376	Bacteria>Terrabacteria_group>Actinobacteria>Actinomycetia>Streptomycetales	Streptomyces_azureus	DNA-sulfur-modification-protein-DndB-[Streptomyces-azureus]	146537
WP_236711551.1	DndB-ParB-nuclease	GCF_001270025.1	716	Bacteria>Terrabacteria_group>Actinobacteria>Actinomycetia>Streptomycetales	Streptomyces_azureus	hypothetical-protein-[Streptomyces-azureus]	146537
GAP46082.1	DndB-ParB-nuclease	GCA_001270025.1	743	Bacteria>Terrabacteria_group>Actinobacteria>Actinomycetia>Streptomycetales	Streptomyces_azureus	uncharacterized-protein-SAZU_0815-[Streptomyces-azureus]	146537
RAN19086.1	DndB-ParB-nuclease	GCA_003263885.1	379	Bacteria>Terrabacteria_group>Actinobacteria>Actinomycetia>Streptomycetales	Streptomyces_badius	DNA-sulfur-modification-protein-DndB-[Streptomyces-badius]	1941
RAN26996.1	DndB-ParB-nuclease	GCA_003263925.1	379	Bacteria>Terrabacteria_group>Actinobacteria>Actinomycetia>Streptomycetales	Streptomyces_badius	DNA-sulfur-modification-protein-DndB-[Streptomyces-badius]	1941
WP_101255410.1	DndB-ParB-nuclease	GCF_002811705.1	379	Bacteria>Terrabacteria_group>Actinobacteria>Actinomycetia>Streptomycetales	Streptomyces_barkulensis	DNA-sulfur-modification-protein-DndB-[Streptomyces-barkulensis]	1257026
KOT38821.1	DndB-ParB-nuclease	GCA_001279005.1	339	Bacteria>Terrabacteria_group>Actinobacteria>Actinomycetia>Streptomycetales	Streptomyces_caelestis	DNA-sulfur-modification-protein-DndB-[Streptomyces-caelestis]	36816
WP_166027208.1	DndB-ParB-nuclease	GCF_001642695.1	376	Bacteria>Terrabacteria_group>Actinobacteria>Actinomycetia>Streptomycetales	Streptomyces_chilikensis	DNA-sulfur-modification-protein-DndB-[Streptomyces-chilikensis]	1194079
WP_138906429.1	DndB-ParB-nuclease	GCF_014650855.1	385	Bacteria>Terrabacteria_group>Actinobacteria>Actinomycetia>Streptomycetales	Streptomyces_chryseus	DNA-sulfur-modification-protein-DndB-[Streptomyces-chryseus]	68186
GGW95437.1	DndB-ParB-nuclease	GCA_014650855.1	695	Bacteria>Terrabacteria_group>Actinobacteria>Actinomycetia>Streptomycetales	Streptomyces_chryseus	hypothetical-protein-GCM10010353_08230-[Streptomyces-chryseus]	68186
WP_229867691.1	DndB-ParB-nuclease	GCF_014650855.1	714	Bacteria>Terrabacteria_group>Actinobacteria>Actinomycetia>Streptomycetales	Streptomyces_chryseus	hypothetical-protein,-partial-[Streptomyces-chryseus]	68186
WP_224754418.1	DndB-ParB-nuclease	-	339	Bacteria>Terrabacteria_group>Actinobacteria>Actinomycetia>Streptomycetales	Streptomyces_chumphonensis	DNA-sulfur-modification-protein-DndB-[Streptomyces-chumphonensis]	1214925
WP_191208258.1	DndB-ParB-nuclease	GCF_014779715.1	752	Bacteria>Terrabacteria_group>Actinobacteria>Actinomycetia>Streptomycetales	Streptomyces_chumphonensis	hypothetical-protein-[Streptomyces-chumphonensis]	1214925
WP_190186382.1	DndB-ParB-nuclease	GCF_014650775.1	376	Bacteria>Terrabacteria_group>Actinobacteria>Actinomycetia>Streptomycetales	Streptomyces_cirratus	DNA-sulfur-modification-protein-DndB-[Streptomyces-cirratus]	68187
WP_229873930.1	DndB-ParB-nuclease	GCF_014650775.1	692	Bacteria>Terrabacteria_group>Actinobacteria>Actinomycetia>Streptomycetales	Streptomyces_cirratus	hypothetical-protein-[Streptomyces-cirratus]	68187
KUM79256.1	DndB-ParB-nuclease	GCA_001513975.1	379	Bacteria>Terrabacteria_group>Actinobacteria>Actinomycetia>Streptomycetales	Streptomyces_curacoi	DNA-sulfur-modification-protein-DndB-[Streptomyces-curacoi]	146536
WP_062146838.1	DndB-ParB-nuclease	GCF_001513975.1	379	Bacteria>Terrabacteria_group>Actinobacteria>Actinomycetia>Streptomycetales	Streptomyces_curacoi	DNA-sulfur-modification-protein-DndB-[Streptomyces-curacoi]	146536
WP_189326211.1	DndB-ParB-nuclease	GCF_014647675.1	379	Bacteria>Terrabacteria_group>Actinobacteria>Actinomycetia>Streptomycetales	Streptomyces_flaveus	DNA-sulfur-modification-protein-DndB-[Streptomyces-flaveus]	66370
GGL03218.1	DndB-ParB-nuclease	GCA_014647675.1	397	Bacteria>Terrabacteria_group>Actinobacteria>Actinomycetia>Streptomycetales	Streptomyces_flaveus	hypothetical-protein-GCM10010094_75040-[Streptomyces-flaveus]	66370
AWL87906.1	DndB-ParB-nuclease	GCA_003147545.1	379	Bacteria>Terrabacteria_group>Actinobacteria>Actinomycetia>Streptomycetales	Streptomyces_globisporus	DNA-sulfur-modification-protein-DndB-[Streptomyces-globisporus]	1908
WP_193473726.1	DndB-ParB-nuclease	GCF_014649595.1	380	Bacteria>Terrabacteria_group>Actinobacteria>Actinomycetia>Streptomycetales	Streptomyces_griseomycini	DNA-sulfur-modification-protein-DndB-[Streptomyces-griseomycini]	66895
WP_193473731.1	DndB-ParB-nuclease	GCF_014649595.1	742	Bacteria>Terrabacteria_group>Actinobacteria>Actinomycetia>Streptomycetales	Streptomyces_griseomycini	hypothetical-protein-[Streptomyces-griseomycini]	66895
PPA41773.1	DndB-ParB-nuclease	GCA_000932225.2	379	Bacteria>Terrabacteria_group>Actinobacteria>Actinomycetia>Streptomycetales	Streptomyces_griseus	DNA-sulfur-modification-protein-DndB-[Streptomyces-griseus]	1911
WP_032774037.1	DndB-ParB-nuclease	GCF_000717795.1	379	Bacteria>Terrabacteria_group>Actinobacteria>Actinomycetia>Streptomycetales	Streptomyces_griseus	DNA-sulfur-modification-protein-DndB-[Streptomyces-griseus]	1911
WP_229878418.1	DndB-ParB-nuclease	GCF_014649655.1	376	Bacteria>Terrabacteria_group>Actinobacteria>Actinomycetia>Streptomycetales	Streptomyces_humidus	DNA-sulfur-modification-protein-DndB-[Streptomyces-humidus]	52259
GGS15197.1	DndB-ParB-nuclease	GCA_014649655.1	408	Bacteria>Terrabacteria_group>Actinobacteria>Actinomycetia>Streptomycetales	Streptomyces_humidus	hypothetical-protein-GCM10010269_63050-[Streptomyces-humidus]	52259
GGS15233.1	DndB-ParB-nuclease	GCA_014649655.1	740	Bacteria>Terrabacteria_group>Actinobacteria>Actinomycetia>Streptomycetales	Streptomyces_humidus	hypothetical-protein-GCM10010269_63100-[Streptomyces-humidus]	52259
WP_190152736.1	DndB-ParB-nuclease	GCF_014649655.1	740	Bacteria>Terrabacteria_group>Actinobacteria>Actinomycetia>Streptomycetales	Streptomyces_humidus	hypothetical-protein-[Streptomyces-humidus]	52259
WP_079106484.1	DndB-ParB-nuclease	GCF_001553435.1	384	Bacteria>Terrabacteria_group>Actinobacteria>Actinomycetia>Streptomycetales	Streptomyces_hygroscopicus	DNA-sulfur-modification-protein-DndB-[Streptomyces-hygroscopicus]	1912
WP_060951189.1	DndB-ParB-nuclease	-	750	Bacteria>Terrabacteria_group>Actinobacteria>Actinomycetia>Streptomycetales	Streptomyces_hygroscopicus	hypothetical-protein-[Streptomyces-hygroscopicus]	1912
WP_033306244.1	DndB-ParB-nuclease	GCF_000717055.1	376	Bacteria>Terrabacteria_group>Actinobacteria>Actinomycetia>Streptomycetales	Streptomyces_iakyrus	DNA-sulfur-modification-protein-DndB-[Streptomyces-iakyrus]	68219
WP_157852658.1	DndB-ParB-nuclease	GCF_000717055.1	593	Bacteria>Terrabacteria_group>Actinobacteria>Actinomycetia>Streptomycetales	Streptomyces_iakyrus	hypothetical-protein-[Streptomyces-iakyrus]	68219
MQS14739.1	DndB-ParB-nuclease	GCA_009569385.1	369	Bacteria>Terrabacteria_group>Actinobacteria>Actinomycetia>Streptomycetales	Streptomyces_kaniharaensis	DNA-sulfur-modification-protein-DndB-[Streptomyces-kaniharaensis]	212423
WP_230210794.1	DndB-ParB-nuclease	GCF_009569385.1	339	Bacteria>Terrabacteria_group>Actinobacteria>Actinomycetia>Streptomycetales	Streptomyces_kaniharaensis	DNA-sulfur-modification-protein-DndB-[Streptomyces-kaniharaensis]	212423
MQS14746.1	DndB-ParB-nuclease	GCA_009569385.1	749	Bacteria>Terrabacteria_group>Actinobacteria>Actinomycetia>Streptomycetales	Streptomyces_kaniharaensis	hypothetical-protein-[Streptomyces-kaniharaensis]	212423
WP_230210796.1	DndB-ParB-nuclease	GCF_009569385.1	718	Bacteria>Terrabacteria_group>Actinobacteria>Actinomycetia>Streptomycetales	Streptomyces_kaniharaensis	hypothetical-protein-[Streptomyces-kaniharaensis]	212423
WP_073947943.1	DndB-ParB-nuclease	GCF_001906585.1	379	Bacteria>Terrabacteria_group>Actinobacteria>Actinomycetia>Streptomycetales	Streptomyces_kebangsaanensis	DNA-sulfur-modification-protein-DndB-[Streptomyces-kebangsaanensis]	864058
WP_073947938.1	DndB-ParB-nuclease	GCF_001906585.1	743	Bacteria>Terrabacteria_group>Actinobacteria>Actinomycetia>Streptomycetales	Streptomyces_kebangsaanensis	hypothetical-protein-[Streptomyces-kebangsaanensis]	864058
WP_148026201.1	DndB-ParB-nuclease	GCF_008016805.1	435	Bacteria>Terrabacteria_group>Actinobacteria>Actinomycetia>Streptomycetales	Streptomyces_lavendulae	DGQHR-domain-containing-protein-[Streptomyces-lavendulae]	1914
WP_234342087.1	DndB-ParB-nuclease	GCF_001047315.1	379	Bacteria>Terrabacteria_group>Actinobacteria>Actinomycetia>Streptomycetales	Streptomyces_leeuwenhoekii	DNA-sulfur-modification-protein-DndB-[Streptomyces-leeuwenhoekii]	1437453
AAZ29041.1	DndB-ParB-nuclease	-	339	Bacteria>Terrabacteria_group>Actinobacteria>Actinomycetia>Streptomycetales	Streptomyces_lividans_1326	putative-ATPase-[Streptomyces-lividans-1326]	1200984
ABP49155.1	DndB-ParB-nuclease	-	312	Bacteria>Terrabacteria_group>Actinobacteria>Actinomycetia>Streptomycetales	Streptomyces_lividans	conserved-hypothetical-protein-[Streptomyces-lividans]	1916
ABP49148.1	DndB-ParB-nuclease	-	692	Bacteria>Terrabacteria_group>Actinobacteria>Actinomycetia>Streptomycetales	Streptomyces_lividans	hypothetical-protein-SLG72-[Streptomyces-lividans]	1916
WP_003972933.1	DndB-ParB-nuclease	GCF_000403665.1	376	Bacteria>Terrabacteria_group>Actinobacteria>Actinomycetia>Streptomycetales	Streptomyces	MULTISPECIES:-DNA-sulfur-modification-protein-DndB-[Streptomyces]	1883
WP_037805139.1	DndB-ParB-nuclease	GCF_000718375.1	379	Bacteria>Terrabacteria_group>Actinobacteria>Actinomycetia>Streptomycetales	Streptomyces	MULTISPECIES:-DNA-sulfur-modification-protein-DndB-[Streptomyces]	1883
WP_044369502.1	DndB-ParB-nuclease	GCF_003147545.1	379	Bacteria>Terrabacteria_group>Actinobacteria>Actinomycetia>Streptomycetales	Streptomyces	MULTISPECIES:-DNA-sulfur-modification-protein-DndB-[Streptomyces]	1883
WP_137865241.1	DndB-ParB-nuclease	GCF_000009765.2	567	Bacteria>Terrabacteria_group>Actinobacteria>Actinomycetia>Streptomycetales	Streptomyces	MULTISPECIES:-hypothetical-protein-[Streptomyces]	1883
WP_086809676.1	DndB-ParB-nuclease	GCF_002154555.1	376	Bacteria>Terrabacteria_group>Actinobacteria>Actinomycetia>Streptomycetales	Streptomyces_murinus	DNA-sulfur-modification-protein-DndB-[Streptomyces-murinus]	33900
WP_086809650.1	DndB-ParB-nuclease	-	726	Bacteria>Terrabacteria_group>Actinobacteria>Actinomycetia>Streptomycetales	Streptomyces_murinus	hypothetical-protein-[Streptomyces-murinus]	33900
WP_229830112.1	DndB-ParB-nuclease	GCF_014649815.1	376	Bacteria>Terrabacteria_group>Actinobacteria>Actinomycetia>Streptomycetales	Streptomyces_mutabilis	DNA-sulfur-modification-protein-DndB-[Streptomyces-mutabilis]	67332
WP_191884020.1	DndB-ParB-nuclease	GCF_014649815.1	593	Bacteria>Terrabacteria_group>Actinobacteria>Actinomycetia>Streptomycetales	Streptomyces_mutabilis	hypothetical-protein-[Streptomyces-mutabilis]	67332
WP_229832421.1	DndB-ParB-nuclease	GCF_014649015.1	379	Bacteria>Terrabacteria_group>Actinobacteria>Actinomycetia>Streptomycetales	Streptomyces_narbonensis	DNA-sulfur-modification-protein-DndB-[Streptomyces-narbonensis]	67333
GGV99952.1	DndB-ParB-nuclease	GCA_014649015.1	421	Bacteria>Terrabacteria_group>Actinobacteria>Actinomycetia>Streptomycetales	Streptomyces_narbonensis	hypothetical-protein-GCM10010230_26920-[Streptomyces-narbonensis]	67333
WP_229888462.1	DndB-ParB-nuclease	GCF_014650375.1	379	Bacteria>Terrabacteria_group>Actinobacteria>Actinomycetia>Streptomycetales	Streptomyces_nitrosporeus	DNA-sulfur-modification-protein-DndB-[Streptomyces-nitrosporeus]	28894
WP_150488508.1	DndB-ParB-nuclease	GCF_014650375.1	571	Bacteria>Terrabacteria_group>Actinobacteria>Actinomycetia>Streptomycetales	Streptomyces_nitrosporeus	hypothetical-protein-[Streptomyces-nitrosporeus]	28894
MBC9717833.1	DndB-ParB-nuclease	GCA_014493765.1	361	Bacteria>Terrabacteria_group>Actinobacteria>Actinomycetia>Streptomycetales	Streptomyces_polyasparticus	DNA-sulfur-modification-protein-DndB-[Streptomyces-polyasparticus]	2767826
WP_236071866.1	DndB-ParB-nuclease	GCF_014493765.1	393	Bacteria>Terrabacteria_group>Actinobacteria>Actinomycetia>Streptomycetales	Streptomyces_polyasparticus	DNA-sulfur-modification-protein-DndB-[Streptomyces-polyasparticus]	2767826
WP_062712667.1	DndB-ParB-nuclease	GCF_001509475.1	376	Bacteria>Terrabacteria_group>Actinobacteria>Actinomycetia>Streptomycetales	Streptomyces_regalis	DNA-sulfur-modification-protein-DndB-[Streptomyces-regalis]	68262
WP_062712675.1	DndB-ParB-nuclease	GCF_001509475.1	723	Bacteria>Terrabacteria_group>Actinobacteria>Actinomycetia>Streptomycetales	Streptomyces_regalis	hypothetical-protein-[Streptomyces-regalis]	68262
WP_159053446.1	DndB-ParB-nuclease	GCF_001509475.1	563	Bacteria>Terrabacteria_group>Actinobacteria>Actinomycetia>Streptomycetales	Streptomyces_regalis	hypothetical-protein-[Streptomyces-regalis]	68262
WP_033042010.1	DndB-ParB-nuclease	GCF_000718675.1	377	Bacteria>Terrabacteria_group>Actinobacteria>Actinomycetia>Streptomycetales	Streptomyces_rimosus	DNA-sulfur-modification-protein-DndB-[Streptomyces-rimosus]	1927
WP_053802161.1	DndB-ParB-nuclease	GCF_001279345.1	376	Bacteria>Terrabacteria_group>Actinobacteria>Actinomycetia>Streptomycetales	Streptomyces_rimosus	DNA-sulfur-modification-protein-DndB-[Streptomyces-rimosus]	1927
WP_030647893.1	DndB-ParB-nuclease	GCF_000718675.1	570	Bacteria>Terrabacteria_group>Actinobacteria>Actinomycetia>Streptomycetales	Streptomyces_rimosus	hypothetical-protein-[Streptomyces-rimosus]	1927
WP_159393311.1	DndB-ParB-nuclease	GCF_001279345.1	776	Bacteria>Terrabacteria_group>Actinobacteria>Actinomycetia>Streptomycetales	Streptomyces_rimosus	hypothetical-protein-[Streptomyces-rimosus]	1927
WP_218029887.1	DndB-ParB-nuclease	GCF_003112535.1	379	Bacteria>Terrabacteria_group>Actinobacteria>Actinomycetia>Streptomycetales	Streptomyces_rishiriensis	DNA-sulfur-modification-protein-DndB-[Streptomyces-rishiriensis]	68264
WP_031225652.1	DndB-ParB-nuclease	GCF_000497445.1	376	Bacteria>Terrabacteria_group>Actinobacteria>Actinomycetia>Streptomycetales	Streptomyces_roseochromogenus	DNA-sulfur-modification-protein-DndB-[Streptomyces-roseochromogenus]	285450
WP_235481562.1	DndB-ParB-nuclease	GCF_021538775.1	379	Bacteria>Terrabacteria_group>Actinobacteria>Actinomycetia>Streptomycetales	Streptomyces_roseoverticillatus	DNA-sulfur-modification-protein-DndB-[Streptomyces-roseoverticillatus]	66429
WP_055699756.1	DndB-ParB-nuclease	GCF_001419745.1	376	Bacteria>Terrabacteria_group>Actinobacteria>Actinomycetia>Streptomycetales	Streptomyces_silaceus	DNA-sulfur-modification-protein-DndB-[Streptomyces-silaceus]	545123
WP_055699749.1	DndB-ParB-nuclease	GCF_001419745.1	726	Bacteria>Terrabacteria_group>Actinobacteria>Actinomycetia>Streptomycetales	Streptomyces_silaceus	hypothetical-protein-[Streptomyces-silaceus]	545123
WP_216856565.1	DndB-ParB-nuclease	GCF_007176755.1	379	Bacteria>Terrabacteria_group>Actinobacteria>Actinomycetia>Streptomycetales	Streptomyces_sp._1-11	DNA-sulfur-modification-protein-DndB-[Streptomyces-sp.-1-11]	2590549
WP_143626934.1	DndB-ParB-nuclease	GCF_007176755.1	742	Bacteria>Terrabacteria_group>Actinobacteria>Actinomycetia>Streptomycetales	Streptomyces_sp._1-11	hypothetical-protein-[Streptomyces-sp.-1-11]	2590549
WP_039633860.1	DndB-ParB-nuclease	GCF_000816025.1	376	Bacteria>Terrabacteria_group>Actinobacteria>Actinomycetia>Streptomycetales	Streptomyces_sp._769	DNA-sulfur-modification-protein-DndB-[Streptomyces-sp.-769]	1262452
WP_052286786.1	DndB-ParB-nuclease	GCF_000816025.1	570	Bacteria>Terrabacteria_group>Actinobacteria>Actinomycetia>Streptomycetales	Streptomyces_sp._769	hypothetical-protein-[Streptomyces-sp.-769]	1262452
WP_225051005.1	DndB-ParB-nuclease	GCF_020177475.1	435	Bacteria>Terrabacteria_group>Actinobacteria>Actinomycetia>Streptomycetales	Streptomyces_sp._7G	DGQHR-domain-containing-protein-[Streptomyces-sp.-7G]	2877241
MBV1949367.1	DndB-ParB-nuclease	GCA_019090105.1	379	Bacteria>Terrabacteria_group>Actinobacteria>Actinomycetia>Streptomycetales	Streptomyces_sp._BV129	DNA-sulfur-modification-protein-DndB-[Streptomyces-sp.-BV129]	2849671
MBV1949488.1	DndB-ParB-nuclease	GCA_019090105.1	379	Bacteria>Terrabacteria_group>Actinobacteria>Actinomycetia>Streptomycetales	Streptomyces_sp._BV129	DNA-sulfur-modification-protein-DndB-[Streptomyces-sp.-BV129]	2849671

Accession	Annotation	GCA	Length	Phyletic.spread	Strain.name	Defline	Species.Taxid
WP_217464567.1	DndB-ParB-nuclease	-	379	Bacteria>Terrabacteria_group>Actinobacteria>Actinomycetia>Streptomycetales	Streptomyces_sp._BV129	DNA-sulfur-modification-protein-DndB-[Streptomyces-sp.-BV129]	2849671
OKJ38679.1	DndB-ParB-nuclease	GCA_001905905.1	387	Bacteria>Terrabacteria_group>Actinobacteria>Actinomycetia>Streptomycetales	Streptomyces_sp._CB02366	DNA-sulfur-modification-protein-DndB-[Streptomyces-sp.-CB02366]	1703935
WP_073768643.1	DndB-ParB-nuclease	-	387	Bacteria>Terrabacteria_group>Actinobacteria>Actinomycetia>Streptomycetales	Streptomyces_sp._CB02366	DNA-sulfur-modification-protein-DndB-[Streptomyces-sp.-CB02366]	1703935
OKJ73809.1	DndB-ParB-nuclease	GCA_001905705.1	379	Bacteria>Terrabacteria_group>Actinobacteria>Actinomycetia>Streptomycetales	Streptomyces_sp._CB02460	DNA-sulfur-modification-protein-DndB-[Streptomyces-sp.-CB02460]	1703941
WP_107426833.1	DndB-ParB-nuclease	-	377	Bacteria>Terrabacteria_group>Actinobacteria>Actinomycetia>Streptomycetales	Streptomyces_sp._CB02460	DNA-sulfur-modification-protein-DndB-[Streptomyces-sp.-CB02460]	1703941
WP_084907751.1	DndB-ParB-nuclease	GCF_002099185.1	376	Bacteria>Terrabacteria_group>Actinobacteria>Actinomycetia>Streptomycetales	Streptomyces_sp._CB03238	DNA-sulfur-modification-protein-DndB-[Streptomyces-sp.-CB03238]	1907777
WP_084907765.1	DndB-ParB-nuclease	GCF_002099185.1	567	Bacteria>Terrabacteria_group>Actinobacteria>Actinomycetia>Streptomycetales	Streptomyces_sp._CB03238	hypothetical-protein-[Streptomyces-sp.-CB03238]	1907777
MBD0676979.1	DndB-ParB-nuclease	GCA_014596515.1	379	Bacteria>Terrabacteria_group>Actinobacteria>Actinomycetia>Streptomycetales	Streptomyces_sp._CBMA156	DNA-sulfur-modification-protein-DndB-[Streptomyces-sp.-CBMA156]	1930280
WP_223245625.1	DndB-ParB-nuclease	GCF_014596515.1	380	Bacteria>Terrabacteria_group>Actinobacteria>Actinomycetia>Streptomycetales	Streptomyces_sp._CBMA156	DNA-sulfur-modification-protein-DndB-[Streptomyces-sp.-CBMA156]	1930280
WP_138914219.1	DndB-ParB-nuclease	-	435	Bacteria>Terrabacteria_group>Actinobacteria>Actinomycetia>Streptomycetales	Streptomyces_sp._DASNCL29	DGQHR-domain-containing-protein-[Streptomyces-sp.-DASNCL29]	2583819
WP_252884062.1	DndB-ParB-nuclease	GCF_003353555.1	379	Bacteria>Terrabacteria_group>Actinobacteria>Actinomycetia>Streptomycetales	Streptomyces_sp._HB202	DNA-sulfur-modification-protein-DndB-[Streptomyces-sp.-HB202]	767398
OBQ49258.1	DndB-ParB-nuclease	GCA_001672315.1	356	Bacteria>Terrabacteria_group>Actinobacteria>Actinomycetia>Streptomycetales	Streptomyces_sp._H-KF8	DNA-sulfur-modification-protein-DndB-[Streptomyces-sp.-H-KF8]	1727216
WP_234373392.1	DndB-ParB-nuclease	GCF_001672315.1	379	Bacteria>Terrabacteria_group>Actinobacteria>Actinomycetia>Streptomycetales	Streptomyces_sp._H-KF8	DNA-sulfur-modification-protein-DndB-[Streptomyces-sp.-H-KF8]	1727216
MBA2808021.1	DndB-ParB-nuclease	GCA_004337435.2	377	Bacteria>Terrabacteria_group>Actinobacteria>Actinomycetia>Streptomycetales	Streptomyces_sp._KM273126	DNA-sulfur-modification-protein-DndB-[Streptomyces-sp.-KM273126]	2545247
SED52204.1	DndB-ParB-nuclease	GCA_900105275.1	726	Bacteria>Terrabacteria_group>Actinobacteria>Actinomycetia>Streptomycetales	Streptomyces_sp._KS_5	DNA-sulfur-modification-associated-[Streptomyces-sp.-KS_5]	1881018
SED52360.1	DndB-ParB-nuclease	GCA_900105275.1	379	Bacteria>Terrabacteria_group>Actinobacteria>Actinomycetia>Streptomycetales	Streptomyces_sp._KS_5	DNA-sulfur-modification-protein-DndB-[Streptomyces-sp.-KS_5]	1881018
WP_093866779.1	DndB-ParB-nuclease	GCF_900105275.1	379	Bacteria>Terrabacteria_group>Actinobacteria>Actinomycetia>Streptomycetales	Streptomyces_sp._KS_5	DNA-sulfur-modification-protein-DndB-[Streptomyces-sp.-KS_5]	1881018
WP_093866776.1	DndB-ParB-nuclease	-	726	Bacteria>Terrabacteria_group>Actinobacteria>Actinomycetia>Streptomycetales	Streptomyces_sp._KS_5	hypothetical-protein-[Streptomyces-sp.-KS_5]	1881018
MBK3562572.1	DndB-ParB-nuclease	GCA_016612625.1	411	Bacteria>Terrabacteria_group>Actinobacteria>Actinomycetia>Streptomycetales	Streptomyces_sp._MBT62	DNA-sulfur-modification-protein-DndB-[Streptomyces-sp.-MBT62]	2800410
WP_234467498.1	DndB-ParB-nuclease	GCF_016612625.1	339	Bacteria>Terrabacteria_group>Actinobacteria>Actinomycetia>Streptomycetales	Streptomyces_sp._MBT62	DNA-sulfur-modification-protein-DndB-[Streptomyces-sp.-MBT62]	2800410
QWB28284.1	DndB-ParB-nuclease	GCA_018604545.1	377	Bacteria>Terrabacteria_group>Actinobacteria>Actinomycetia>Streptomycetales	Streptomyces_sp._MG62	DNA-sulfur-modification-protein-DndB-[Streptomyces-sp.-MG62]	2838015
WP_215124309.1	DndB-ParB-nuclease	-	377	Bacteria>Terrabacteria_group>Actinobacteria>Actinomycetia>Streptomycetales	Streptomyces_sp._MG62	DNA-sulfur-modification-protein-DndB-[Streptomyces-sp.-MG62]	2838015
ONK12584.1	DndB-ParB-nuclease	GCA_001984575.1	368	Bacteria>Terrabacteria_group>Actinobacteria>Actinomycetia>Streptomycetales	Streptomyces_sp._MP131-18	DNA-sulfur-modification-protein-DndB-[Streptomyces-sp.-MP131-18]	1857892
WP_077059979.1	DndB-ParB-nuclease	GCF_001984575.1	368	Bacteria>Terrabacteria_group>Actinobacteria>Actinomycetia>Streptomycetales	Streptomyces_sp._MP131-18	DNA-sulfur-modification-protein-DndB-[Streptomyces-sp.-MP131-18]	1857892
WP_240116961.1	DndB-ParB-nuclease	GCF_022414535.1	379	Bacteria>Terrabacteria_group>Actinobacteria>Actinomycetia>Streptomycetales	Streptomyces_sp._MUM_2J	DNA-sulfur-modification-protein-DndB-[Streptomyces-sp.-MUM-2J]	2791987
WP_208515212.1	DndB-ParB-nuclease	GCF_017592595.1	385	Bacteria>Terrabacteria_group>Actinobacteria>Actinomycetia>Streptomycetales	Streptomyces_sp._NEAU-YJ-81	DNA-sulfur-modification-protein-DndB-[Streptomyces-sp.-NEAU-YJ-81]	2820288
WP_208515206.1	DndB-ParB-nuclease	GCF_017592595.1	749	Bacteria>Terrabacteria_group>Actinobacteria>Actinomycetia>Streptomycetales	Streptomyces_sp._NEAU-YJ-81	hypothetical-protein-[Streptomyces-sp.-NEAU-YJ-81]	2820288
WP_232649361.1	DndB-ParB-nuclease	GCF_021261325.1	376	Bacteria>Terrabacteria_group>Actinobacteria>Actinomycetia>Streptomycetales	Streptomyces_sp._NR30	DNA-sulfur-modification-protein-DndB-[Streptomyces-sp.-NR30]	2886947
WP_232649356.1	DndB-ParB-nuclease	GCF_021261325.1	742	Bacteria>Terrabacteria_group>Actinobacteria>Actinomycetia>Streptomycetales	Streptomyces_sp._NR30	hypothetical-protein-[Streptomyces-sp.-NR30]	2886947
WP_232649365.1	DndB-ParB-nuclease	GCF_021261325.1	572	Bacteria>Terrabacteria_group>Actinobacteria>Actinomycetia>Streptomycetales	Streptomyces_sp._NR30	hypothetical-protein-[Streptomyces-sp.-NR30]	2886947
WP_030711227.1	DndB-ParB-nuclease	GCF_000720115.1	376	Bacteria>Terrabacteria_group>Actinobacteria>Actinomycetia>Streptomycetales	Streptomyces_sp._NRRL_F-2580	DNA-sulfur-modification-protein-DndB-[Streptomyces-sp.-NRRL-F-2580]	1463841
WP_030711239.1	DndB-ParB-nuclease	GCF_000720115.1	724	Bacteria>Terrabacteria_group>Actinobacteria>Actinomycetia>Streptomycetales	Streptomyces_sp._NRRL_F-2580	hypothetical-protein-[Streptomyces-sp.-NRRL-F-2580]	1463841
WP_037836665.1	DndB-ParB-nuclease	GCF_000721365.1	376	Bacteria>Terrabacteria_group>Actinobacteria>Actinomycetia>Streptomycetales	Streptomyces_sp._NRRL_F-5650	DNA-sulfur-modification-protein-DndB-[Streptomyces-sp.-NRRL-F-5650]	1463868
WP_051852053.1	DndB-ParB-nuclease	GCF_000721365.1	715	Bacteria>Terrabacteria_group>Actinobacteria>Actinomycetia>Streptomycetales	Streptomyces_sp._NRRL_F-5650	hypothetical-protein-[Streptomyces-sp.-NRRL-F-5650]	1463868
WP_078874309.1	DndB-ParB-nuclease	GCF_000721415.1	379	Bacteria>Terrabacteria_group>Actinobacteria>Actinomycetia>Streptomycetales	Streptomyces_sp._NRRL_F-5702	DNA-sulfur-modification-protein-DndB-[Streptomyces-sp.-NRRL-F-5702]	1463870
WP_031053288.1	DndB-ParB-nuclease	GCF_000721415.1	743	Bacteria>Terrabacteria_group>Actinobacteria>Actinomycetia>Streptomycetales	Streptomyces_sp._NRRL_F-5702	hypothetical-protein-[Streptomyces-sp.-NRRL-F-5702]	1463870
MBQ0827457.1	DndB-ParB-nuclease	GCA_018069565.1	356	Bacteria>Terrabacteria_group>Actinobacteria>Actinomycetia>Streptomycetales	Streptomyces_sp._RG38	DNA-sulfur-modification-protein-DndB-[Streptomyces-sp.-RG38]	2820809
WP_210872738.1	DndB-ParB-nuclease	-	356	Bacteria>Terrabacteria_group>Actinobacteria>Actinomycetia>Streptomycetales	Streptomyces_sp._RG38	DNA-sulfur-modification-protein-DndB-[Streptomyces-sp.-RG38]	2820809
QDN93536.1	DndB-ParB-nuclease	GCA_007113545.2	379	Bacteria>Terrabacteria_group>Actinobacteria>Actinomycetia>Streptomycetales	Streptomyces_sp._RLB3-6	DNA-sulfur-modification-protein-DndB-[Streptomyces-sp.-RLB3-6]	2594457
WP_143615023.1	DndB-ParB-nuclease	GCF_007113545.2	379	Bacteria>Terrabacteria_group>Actinobacteria>Actinomycetia>Streptomycetales	Streptomyces_sp._RLB3-6	DNA-sulfur-modification-protein-DndB-[Streptomyces-sp.-RLB3-6]	2594457
WP_221740749.1	DndB-ParB-nuclease	GCF_003933965.1	379	Bacteria>Terrabacteria_group>Actinobacteria>Actinomycetia>Streptomycetales	Streptomyces_sp._RP5T	DNA-sulfur-modification-protein-DndB-[Streptomyces-sp.-RP5T]	2490848
POX62401.1	DndB-ParB-nuclease	GCA_002920615.1	379	Bacteria>Terrabacteria_group>Actinobacteria>Actinomycetia>Streptomycetales	Streptomyces_sp._Ru62	DNA-sulfur-modification-protein-DndB-[Streptomyces-sp.-Ru62]	2080745
WP_103812576.1	DndB-ParB-nuclease	GCF_002920615.1	379	Bacteria>Terrabacteria_group>Actinobacteria>Actinomycetia>Streptomycetales	Streptomyces_sp._Ru62	DNA-sulfur-modification-protein-DndB-[Streptomyces-sp.-Ru62]	2080745
POX62405.1	DndB-ParB-nuclease	GCA_002920615.1	742	Bacteria>Terrabacteria_group>Actinobacteria>Actinomycetia>Streptomycetales	Streptomyces_sp._Ru62	hypothetical-protein-C3492_16880-[Streptomyces-sp.-Ru62]	2080745
WP_103812580.1	DndB-ParB-nuclease	GCF_002920615.1	742	Bacteria>Terrabacteria_group>Actinobacteria>Actinomycetia>Streptomycetales	Streptomyces_sp._Ru62	hypothetical-protein-[Streptomyces-sp.-Ru62]	2080745
WP_146075694.1	DndB-ParB-nuclease	GCF_002920615.1	572	Bacteria>Terrabacteria_group>Actinobacteria>Actinomycetia>Streptomycetales	Streptomyces_sp._Ru62	hypothetical-protein-[Streptomyces-sp.-Ru62]	2080745
QCW80578.1	DndB-ParB-nuclease	GCA_004521945.2	374	Bacteria>Terrabacteria_group>Actinobacteria>Actinomycetia>Streptomycetales	Streptomyces_sp._S6	DNA-sulfur-modification-protein-DndB-[Streptomyces-sp.-S6]	2506427
QCW78227.1	DndB-ParB-nuclease	GCA_004521945.2	571	Bacteria>Terrabacteria_group>Actinobacteria>Actinomycetia>Streptomycetales	Streptomyces_sp._S6	hypothetical-protein-EQG64_15005-[Streptomyces-sp.-S6]	2506427
WP_237550166.1	DndB-ParB-nuclease	GCF_009865605.1	339	Bacteria>Terrabacteria_group>Actinobacteria>Actinomycetia>Streptomycetales	Streptomyces_sp._SID1034	DNA-sulfur-modification-protein-DndB-[Streptomyces-sp.-SID1034]	2690248
MYS98531.1	DndB-ParB-nuclease	GCA_009862865.1	379	Bacteria>Terrabacteria_group>Actinobacteria>Actinomycetia>Streptomycetales	Streptomyces_sp._SID5469	DNA-sulfur-modification-protein-DndB-[Streptomyces-sp.-SID5469]	2690296
WP_237529334.1	DndB-ParB-nuclease	GCF_009862865.1	339	Bacteria>Terrabacteria_group>Actinobacteria>Actinomycetia>Streptomycetales	Streptomyces_sp._SID5469	DNA-sulfur-modification-protein-DndB-[Streptomyces-sp.-SID5469]	2690296
MYS98522.1	DndB-ParB-nuclease	GCA_009862865.1	567	Bacteria>Terrabacteria_group>Actinobacteria>Actinomycetia>Streptomycetales	Streptomyces_sp._SID5469	hypothetical-protein-[Streptomyces-sp.-SID5469]	2690296
MYS87881.1	DndB-ParB-nuclease	GCA_009862895.1	534	Bacteria>Terrabacteria_group>Actinobacteria>Actinomycetia>Streptomycetales	Streptomyces_sp._SID5474	DGQHR-domain-containing-protein-[Streptomyces-sp.-SID5474]	2690300
MYS87886.1	DndB-ParB-nuclease	GCA_009862895.1	364	Bacteria>Terrabacteria_group>Actinobacteria>Actinomycetia>Streptomycetales	Streptomyces_sp._SID5474	DNA-sulfur-modification-protein-DndB-[Streptomyces-sp.-SID5474]	2690300
MYS52042.1	DndB-ParB-nuclease	GCA_009862745.1	379	Bacteria>Terrabacteria_group>Actinobacteria>Actinomycetia>Streptomycetales	Streptomyces_sp._SID6013	DNA-sulfur-modification-protein-DndB-[Streptomyces-sp.-SID6013]	2690317
WP_237500777.1	DndB-ParB-nuclease	GCF_009865085.1	721	Bacteria>Terrabacteria_group>Actinobacteria>Actinomycetia>Streptomycetales	Streptomyces_sp._SID8370	hypothetical-protein-[Streptomyces-sp.-SID8370]	2690351
WP_142168569.1	DndB-ParB-nuclease	GCF_006715925.1	379	Bacteria>Terrabacteria_group>Actinobacteria>Actinomycetia>Streptomycetales	Streptomyces_sp._SLBN-134	DNA-sulfur-modification-protein-DndB-[Streptomyces-sp.-SLBN-134]	2768456
WP_237404752.1	DndB-ParB-nuclease	GCF_016756395.1	379	Bacteria>Terrabacteria_group>Actinobacteria>Actinomycetia>Streptomycetales	Streptomyces_sp._SN-593	DNA-sulfur-modification-protein-DndB-[Streptomyces-sp.-SN-593]	659352
BBA98118.1	DndB-ParB-nuclease	GCA_016756395.1	379	Bacteria>Terrabacteria_group>Actinobacteria>Actinomycetia>Streptomycetales	Streptomyces_sp._SN-593	putative-DNA-sulfur-modification-protein-DndB-[Streptomyces-sp.-SN-593]	659352
PRH80050.1	DndB-ParB-nuclease	GCA_002982015.1	377	Bacteria>Terrabacteria_group>Actinobacteria>Actinomycetia>Streptomycetales	Streptomyces_sp._ST5x	DNA-sulfur-modification-protein-DndB-[Streptomyces-sp.-ST5x]	2100817
WP_105867882.1	DndB-ParB-nuclease	-	377	Bacteria>Terrabacteria_group>Actinobacteria>Actinomycetia>Streptomycetales	Streptomyces_sp._ST5x	DNA-sulfur-modification-protein-DndB-[Streptomyces-sp.-ST5x]	2100817
WP_179886749.1	DndB-ParB-nuclease	-	389	Bacteria>Terrabacteria_group>Actinobacteria>Actinomycetia>Streptomycetales	Streptomyces_sp._st77	DNA-sulfur-modification-protein-DndB-[Streptomyces-sp.-st77]	1828074
WP_098897879.1	DndB-ParB-nuclease	GCF_002551175.1	723	Bacteria>Terrabacteria_group>Actinobacteria>Actinomycetia>Streptomycetales	Streptomyces_sp._st77	hypothetical-protein-[Streptomyces-sp.-st77]	1828074
KAF3467487.1	DndB-ParB-nuclease	GCA_009852415.1	379	Bacteria>Terrabacteria_group>Actinobacteria>Actinomycetia>Streptomycetales	Streptomyces_sp._Tu_3180	DNA-sulfur-modification-protein-DndB-[Streptomyces-sp.-Tu-3180]	2682611
WP_159535781.1	DndB-ParB-nuclease	GCF_009852415.1	379	Bacteria>Terrabacteria_group>Actinobacteria>Actinomycetia>Streptomycetales	Streptomyces_sp._Tu_3180	DNA-sulfur-modification-protein-DndB-[Streptomyces-sp.-Tu-3180]	2682611
NYV73210.1	DndB-ParB-nuclease	GCA_013421635.1	369	Bacteria>Terrabacteria_group>Actinobacteria>Actinomycetia>Streptomycetales	Streptomyces_sp._UH6	DNA-sulfur-modification-protein-DndB-[Streptomyces-sp.-UH6]	2748379
RSN64054.1	DndB-ParB-nuclease	GCA_003947455.1	377	Bacteria>Terrabacteria_group>Actinobacteria>Actinomycetia>Streptomycetales	Streptomyces_sp._WAC_04229	DNA-sulfur-modification-protein-DndB-[Streptomyces-sp.-WAC-04229]	2203206
WP_125490234.1	DndB-ParB-nuclease	-	377	Bacteria>Terrabacteria_group>Actinobacteria>Actinomycetia>Streptomycetales	Streptomyces_sp._WAC_04229	DNA-sulfur-modification-protein-DndB-[Streptomyces-sp.-WAC-04229]	2203206
RSN63973.1	DndB-ParB-nuclease	GCA_003947455.1	568	Bacteria>Terrabacteria_group>Actinobacteria>Actinomycetia>Streptomycetales	Streptomyces_sp._WAC_04229	hypothetical-protein-DMH12_04130-[Streptomyces-sp.-WAC-04229]	2203206
WP_125490157.1	DndB-ParB-nuclease	GCF_003947455.1	568	Bacteria>Terrabacteria_group>Actinobacteria>Actinomycetia>Streptomycetales	Streptomyces_sp._WAC_04229	hypothetical-protein-[Streptomyces-sp.-WAC-04229]	2203206
WP_016327561.1	DndB-ParB-nuclease	GCF_000403665.1	743	Bacteria>Terrabacteria_group>Actinobacteria>Actinomycetia>Streptomycetales	Streptomyces_sp._WM6391	hypothetical-protein-[Streptomyces-sp.-WM6391]	1415559
WP_037935911.1	DndB-ParB-nuclease	GCF_000725125.1	379	Bacteria>Terrabacteria_group>Actinobacteria>Actinomycetia>Streptomycetales	Streptomyces_toyocaensis	DNA-sulfur-modification-protein-DndB-[Streptomyces-toyocaensis]	55952
WP_150185334.1	DndB-ParB-nuclease	GCF_008642335.1	376	Bacteria>Terrabacteria_group>Actinobacteria>Actinomycetia>Streptomycetales	Streptomyces_venezuelae	DNA-sulfur-modification-protein-DndB-[Streptomyces-venezuelae]	54571
WP_223832902.1	DndB-ParB-nuclease	GCF_008642335.1	695	Bacteria>Terrabacteria_group>Actinobacteria>Actinomycetia>Streptomycetales	Streptomyces_venezuelae	hypothetical-protein-[Streptomyces-venezuelae]	54571
WP_018468041.1	DndB-ParB-nuclease	GCF_009865125.1	337	Bacteria>Terrabacteria_group>Actinobacteria>Actinomycetia>Streptomycetales	unclassified_Streptomyces	MULTISPECIES:-DNA-sulfur-modification-protein-DndB-[unclassified-Streptomyces]	2593676
WP_088802218.1	DndB-ParB-nuclease	GCF_900102245.1	376	Bacteria>Terrabacteria_group>Actinobacteria>Actinomycetia>Streptomycetales	unclassified_Streptomyces	MULTISPECIES:-DNA-sulfur-modification-protein-DndB-[unclassified-Streptomyces]	2593676
WP_237520726.1	DndB-ParB-nuclease	GCF_009862035.1	339	Bacteria>Terrabacteria_group>Actinobacteria>Actinomycetia>Streptomycetales	unclassified_Streptomyces	MULTISPECIES:-DNA-sulfur-modification-protein-DndB-[unclassified-Streptomyces]	2593676
WP_019524114.1	DndB-ParB-nuclease	GCF_000373465.1	742	Bacteria>Terrabacteria_group>Actinobacteria>Actinomycetia>Streptomycetales	unclassified_Streptomyces	MULTISPECIES:-hypothetical-protein-[unclassified-Streptomyces]	2593676
WP_088799076.1	DndB-ParB-nuclease	-	726	Bacteria>Terrabacteria_group>Actinobacteria>Actinomycetia>Streptomycetales	unclassified_Streptomyces	MULTISPECIES:-hypothetical-protein-[unclassified-Streptomyces]	2593676
WP_111582530.1	DndB-ParB-nuclease	GCF_009862035.1	752	Bacteria>Terrabacteria_group>Actinobacteria>Actinomycetia>Streptomycetales	unclassified_Streptomyces	MULTISPECIES:-hypothetical-protein-[unclassified-Streptomyces]	2593676
WP_131544670.1	DndB-ParB-nuclease	GCF_014281035.1	726	Bacteria>Terrabacteria_group>Actinobacteria>Actinomycetia>Streptomycetales	unclassified_Streptomyces	MULTISPECIES:-hypothetical-protein-[unclassified-Streptomyces]	2593676
WP_158688208.1	DndB-ParB-nuclease	GCF_000373465.1	564	Bacteria>Terrabacteria_group>Actinobacteria>Actinomycetia>Streptomycetales	unclassified_Streptomyces	MULTISPECIES:-hypothetical-protein-[unclassified-Streptomyces]	2593676
WP_239161844.1	DndB-ParB-nuclease	GCF_016862995.1	340	Bacteria>Terrabacteria_group>Actinobacteria>Actinomycetia>Streptosporangiales	Acrocarpospora_phusangensis	DNA-sulfur-modification-protein-DndB-[Acrocarpospora-phusangensis]	1070424
WP_033324950.1	DndB-ParB-nuclease	GCF_000381885.1	376	Bacteria>Terrabacteria_group>Actinobacteria>Actinomycetia>Streptosporangiales	Actinomadura_atramentaria	DNA-sulfur-modification-protein-DndB-[Actinomadura-atramentaria]	1990
WP_019635061.1	DndB-ParB-nuclease	GCF_000381885.1	720	Bacteria>Terrabacteria_group>Actinobacteria>Actinomycetia>Streptosporangiales	Actinomadura_atramentaria	hypothetical-protein-[Actinomadura-atramentaria]	1990
TYB49197.1	DndB-ParB-nuclease	GCA_008085895.1	389	Bacteria>Terrabacteria_group>Actinobacteria>Actinomycetia>Streptosporangiales	Actinomadura_chibensis	DNA-sulfur-modification-protein-DndB-[Actinomadura-chibensis]	392828
WP_148344159.1	DndB-ParB-nuclease	-	389	Bacteria>Terrabacteria_group>Actinobacteria>Actinomycetia>Streptosporangiales	Actinomadura_chibensis	DNA-sulfur-modification-protein-DndB-[Actinomadura-chibensis]	392828
TDD89527.1	DndB-ParB-nuclease	GCA_004349235.1	376	Bacteria>Terrabacteria_group>Actinobacteria>Actinomycetia>Streptosporangiales	Actinomadura_darangshiensis	DNA-sulfur-modification-protein-DndB-[Actinomadura-darangshiensis]	705336
WP_207938669.1	DndB-ParB-nuclease	-	372	Bacteria>Terrabacteria_group>Actinobacteria>Actinomycetia>Streptosporangiales	Actinomadura_darangshiensis	DNA-sulfur-modification-protein-DndB-[Actinomadura-darangshiensis]	705336
NDU74021.1	DndB-ParB-nuclease	GCA_010491635.1	376	Bacteria>Terrabacteria_group>Actinobacteria>Actinomycetia>Streptosporangiales	Actinomadura_lepetitiana	DNA-sulfur-modification-protein-DndB-[Actinomadura-lepetitiana]	2126066
NYD48456.1	DndB-ParB-nuclease	GCA_013409365.1	340	Bacteria>Terrabacteria_group>Actinobacteria>Actinomycetia>Streptosporangiales	Actinomadura_luteofluorescens	DNA-sulfur-modification-protein-DndB-[Actinomadura-luteofluorescens]	46163
WP_179849543.1	DndB-ParB-nuclease	-	372	Bacteria>Terrabacteria_group>Actinobacteria>Actinomycetia>Streptosporangiales	Actinomadura_luteofluorescens	DNA-sulfur-modification-protein-DndB-[Actinomadura-luteofluorescens]	46163
WP_211342855.1	DndB-ParB-nuclease	-	356	Bacteria>Terrabacteria_group>Actinobacteria>Actinomycetia>Streptosporangiales	Actinomadura_pelletieri	DNA-sulfur-modification-protein-DndB-[Actinomadura-pelletieri]	111805
WP_121432292.1	DndB-ParB-nuclease	GCF_003634705.1	722	Bacteria>Terrabacteria_group>Actinobacteria>Actinomycetia>Streptosporangiales	Actinomadura_pelletieri	hypothetical-protein-[Actinomadura-pelletieri]	111805
MBC6456613.1	DndB-ParB-nuclease	GCA_014323665.1	374	Bacteria>Terrabacteria_group>Actinobacteria>Actinomycetia>Streptosporangiales	Actinomadura_sp._HBU206391	DNA-sulfur-modification-protein-DndB-[Actinomadura-sp.-HBU206391]	2731692
WP_222720225.1	DndB-ParB-nuclease	GCF_014323665.1	383	Bacteria>Terrabacteria_group>Actinobacteria>Actinomycetia>Streptosporangiales	Actinomadura_sp._HBU206391	DNA-sulfur-modification-protein-DndB-[Actinomadura-sp.-HBU206391]	2731692
WP_173101458.1	DndB-ParB-nuclease	-	372	Bacteria>Terrabacteria_group>Actinobacteria>Actinomycetia>Streptosporangiales	Actinomadura_verrucosospora	DNA-sulfur-modification-protein-DndB-[Actinomadura-verrucosospora]	46165
WP_235557961.1	DndB-ParB-nuclease	GCF_001417795.1	379	Bacteria>Terrabacteria_group>Actinobacteria>Actinomycetia>Streptosporangiales	Microbispora_sp._GMKU363	DNA-sulfur-modification-protein-DndB-[Microbispora-sp.-GMKU363]	718014
WP_182872764.1	DndB-ParB-nuclease	GCF_014161385.1	372	Bacteria>Terrabacteria_group>Actinobacteria>Actinomycetia>Streptosporangiales	Microbispora_sp._H10670	DNA-sulfur-modification-protein-DndB-[Microbispora-sp.-H10670]	2729108
RGA06201.1	DndB-ParB-nuclease	GCA_003260025.2	372	Bacteria>Terrabacteria_group>Actinobacteria>Actinomycetia>Streptosporangiales	Microbispora_triticiradicis	DNA-sulfur-modification-protein-DndB-[Microbispora-triticiradicis]	2200763
QKW32075.1	DndB-ParB-nuclease	GCA_013364295.1	376	Bacteria>Terrabacteria_group>Actinobacteria>Actinomycetia>Streptosporangiales	Nocardiopsis_flavescens	DNA-sulfur-modification-protein-DndB-[Nocardiopsis-flavescens]	758803
WP_026338082.1	DndB-ParB-nuclease	GCF_000381685.1	380	Bacteria>Terrabacteria_group>Actinobacteria>Actinomycetia>Streptosporangiales	Nocardiopsis_sp._CNS-639	DNA-sulfur-modification-protein-DndB-[Nocardiopsis-sp.-CNS-639]	1169153
WP_231391392.1	DndB-ParB-nuclease	GCF_000381685.1	726	Bacteria>Terrabacteria_group>Actinobacteria>Actinomycetia>Streptosporangiales	Nocardiopsis_sp._CNS-639	hypothetical-protein-[Nocardiopsis-sp.-CNS-639]	1169153
WP_028648085.1	DndB-ParB-nuclease	GCF_000515115.1	376	Bacteria>Terrabacteria_group>Actinobacteria>Actinomycetia>Streptosporangiales	Nocardiopsis_sp._CNT312	DNA-sulfur-modification-protein-DndB-[Nocardiopsis-sp.-CNT312]	1137268
WP_036554450.1	DndB-ParB-nuclease	GCF_000515115.1	727	Bacteria>Terrabacteria_group>Actinobacteria>Actinomycetia>Streptosporangiales	Nocardiopsis_sp._CNT312	hypothetical-protein-[Nocardiopsis-sp.-CNT312]	1137268
WP_232832342.1	DndB-ParB-nuclease	GCF_003386285.1	376	Bacteria>Terrabacteria_group>Actinobacteria>Actinomycetia>Streptosporangiales	Nocardiopsis_sp._FIRDI_009	DNA-sulfur-modification-protein-DndB-[Nocardiopsis-sp.-FIRDI-009]	714197
MBB3730344.1	DndB-ParB-nuclease	GCA_014195505.1	376	Bacteria>Terrabacteria_group>Actinobacteria>Actinomycetia>Streptosporangiales	Nonomuraea_dietziae	DNA-sulfur-modification-protein-DndB-[Nonomuraea-dietziae]	65515
WP_246453207.1	DndB-ParB-nuclease	GCF_014195505.1	340	Bacteria>Terrabacteria_group>Actinobacteria>Actinomycetia>Streptosporangiales	Nonomuraea_dietziae	DNA-sulfur-modification-protein-DndB-[Nonomuraea-dietziae]	65515
WP_221341568.1	DndB-ParB-nuclease	-	372	Bacteria>Terrabacteria_group>Actinobacteria>Actinomycetia>Streptosporangiales	Nonomuraea_endophytica	DNA-sulfur-modification-protein-DndB-[Nonomuraea-endophytica]	714136
WP_225269636.1	DndB-ParB-nuclease	GCF_020215645.1	377	Bacteria>Terrabacteria_group>Actinobacteria>Actinomycetia>Streptosporangiales	Nonomuraea_gerenzanensis	DNA-sulfur-modification-protein-DndB-[Nonomuraea-gerenzanensis]	93944
WP_225269639.1	DndB-ParB-nuclease	GCF_020215645.1	746	Bacteria>Terrabacteria_group>Actinobacteria>Actinomycetia>Streptosporangiales	Nonomuraea_gerenzanensis	hypothetical-protein-[Nonomuraea-gerenzanensis]	93944
NUW37328.1	DndB-ParB-nuclease	GCA_013363975.1	377	Bacteria>Terrabacteria_group>Actinobacteria>Actinomycetia>Streptosporangiales	Nonomuraea_montanisoli	DNA-sulfur-modification-protein-DndB-[Nonomuraea-montanisoli]	2741721
WP_235995816.1	DndB-ParB-nuclease	GCF_013363975.1	340	Bacteria>Terrabacteria_group>Actinobacteria>Actinomycetia>Streptosporangiales	Nonomuraea_montanisoli	DNA-sulfur-modification-protein-DndB-[Nonomuraea-montanisoli]	2741721
NUW37323.1	DndB-ParB-nuclease	GCA_013363975.1	745	Bacteria>Terrabacteria_group>Actinobacteria>Actinomycetia>Streptosporangiales	Nonomuraea_montanisoli	hypothetical-protein-[Nonomuraea-montanisoli]	2741721
WP_175594902.1	DndB-ParB-nuclease	GCF_013363975.1	745	Bacteria>Terrabacteria_group>Actinobacteria>Actinomycetia>Streptosporangiales	Nonomuraea_montanisoli	hypothetical-protein-[Nonomuraea-montanisoli]	2741721
KAB8182369.1	DndB-ParB-nuclease	GCA_006334985.2	377	Bacteria>Terrabacteria_group>Actinobacteria>Actinomycetia>Streptosporangiales	Nonomuraea_phyllanthi	DNA-sulfur-modification-protein-DndB-[Nonomuraea-phyllanthi]	2219224
WP_139638316.1	DndB-ParB-nuclease	GCF_006334985.2	377	Bacteria>Terrabacteria_group>Actinobacteria>Actinomycetia>Streptosporangiales	Nonomuraea_phyllanthi	DNA-sulfur-modification-protein-DndB-[Nonomuraea-phyllanthi]	2219224
KAB8182373.1	DndB-ParB-nuclease	GCA_006334985.2	572	Bacteria>Terrabacteria_group>Actinobacteria>Actinomycetia>Streptosporangiales	Nonomuraea_phyllanthi	hypothetical-protein-FH608_050300-[Nonomuraea-phyllanthi]	2219224
WP_139638320.1	DndB-ParB-nuclease	GCF_006334985.2	572	Bacteria>Terrabacteria_group>Actinobacteria>Actinomycetia>Streptosporangiales	Nonomuraea_phyllanthi	hypothetical-protein-[Nonomuraea-phyllanthi]	2219224
WP_168018177.1	DndB-ParB-nuclease	GCF_012034275.1	382	Bacteria>Terrabacteria_group>Actinobacteria>Actinomycetia>Streptosporangiales	Nonomuraea_sp._FMUSA5-5	DNA-sulfur-modification-protein-DndB-[Nonomuraea-sp.-FMUSA5-5]	2720023
TMR22655.1	DndB-ParB-nuclease	GCA_005889735.1	364	Bacteria>Terrabacteria_group>Actinobacteria>Actinomycetia>Streptosporangiales	Nonomuraea_turkmeniaca	DNA-sulfur-modification-protein-DndB-[Nonomuraea-turkmeniaca]	103838
WP_138666030.1	DndB-ParB-nuclease	-	364	Bacteria>Terrabacteria_group>Actinobacteria>Actinomycetia>Streptosporangiales	Nonomuraea_turkmeniaca	DNA-sulfur-modification-protein-DndB-[Nonomuraea-turkmeniaca]	103838
TMR22630.1	DndB-ParB-nuclease	GCA_005889735.1	562	Bacteria>Terrabacteria_group>Actinobacteria>Actinomycetia>Streptosporangiales	Nonomuraea_turkmeniaca	hypothetical-protein-ETD86_10885-[Nonomuraea-turkmeniaca]	103838
WP_138666005.1	DndB-ParB-nuclease	GCF_005889735.1	562	Bacteria>Terrabacteria_group>Actinobacteria>Actinomycetia>Streptosporangiales	Nonomuraea_turkmeniaca	hypothetical-protein-[Nonomuraea-turkmeniaca]	103838
WP_239128430.1	DndB-ParB-nuclease	GCF_016863175.1	377	Bacteria>Terrabacteria_group>Actinobacteria>Actinomycetia>Streptosporangiales	Planobispora_siamensis	DNA-sulfur-modification-protein-DndB-[Planobispora-siamensis]	936338

Accession	Annotation	GCA	Length	Phyletic.spread	Strain.name	Defline	Species.Taxid
GHH97770.1	DndB-ParB-nuclease	GCA_016863175.1	340	Bacteria>Terrabacteria_group>Actinobacteria>Actinomycetia>Streptosporangiales	Planobispora_siamensis	hypothetical-protein-Psi01_84000-[Planobispora-siamensis]	936338
WP_239319206.1	DndB-ParB-nuclease	GCF_016863215.1	376	Bacteria>Terrabacteria_group>Actinobacteria>Actinomycetia>Streptosporangiales	Planomonospora_parontospora	DNA-sulfur-modification-protein-DndB-[Planomonospora-parontospora]	58119
GGK44997.1	DndB-ParB-nuclease	GCA_014647935.1	379	Bacteria>Terrabacteria_group>Actinobacteria>Actinomycetia>Streptosporangiales	Planomonospora_parontospora	hypothetical-protein-GCM10010126_00680-[Planomonospora-parontospora]	58119
GGK45065.1	DndB-ParB-nuclease	GCA_014647935.1	746	Bacteria>Terrabacteria_group>Actinobacteria>Actinomycetia>Streptosporangiales	Planomonospora_parontospora	hypothetical-protein-GCM10010126_00720-[Planomonospora-parontospora]	58119
WP_239319220.1	DndB-ParB-nuclease	GCF_016863215.1	719	Bacteria>Terrabacteria_group>Actinobacteria>Actinomycetia>Streptosporangiales	Planomonospora_parontospora	hypothetical-protein-[Planomonospora-parontospora]	58119
GII06270.1	DndB-ParB-nuclease	GCA_016863215.1	322	Bacteria>Terrabacteria_group>Actinobacteria>Actinomycetia>Streptosporangiales	Planomonospora_parontospora_subsp._parontospora	hypothetical-protein-Ppa06_00680-[Planomonospora-parontospora-subsp.-parontospora]	97194
GII06274.1	DndB-ParB-nuclease	GCA_016863215.1	746	Bacteria>Terrabacteria_group>Actinobacteria>Actinomycetia>Streptosporangiales	Planomonospora_parontospora_subsp._parontospora	hypothetical-protein-Ppa06_00720-[Planomonospora-parontospora-subsp.-parontospora]	97194
WP_230883971.1	DndB-ParB-nuclease	-	340	Bacteria>Terrabacteria_group>Actinobacteria>Actinomycetia>Streptosporangiales	Planomonospora_sp._ID82291	DNA-sulfur-modification-protein-DndB-[Planomonospora-sp.-ID82291]	2738136
WP_189165616.1	DndB-ParB-nuclease	GCF_014646695.1	376	Bacteria>Terrabacteria_group>Actinobacteria>Actinomycetia>Streptosporangiales	Sphaerisporangium_melleum	DNA-sulfur-modification-protein-DndB-[Sphaerisporangium-melleum]	321316
GGL03255.1	DndB-ParB-nuclease	GCA_014646695.1	376	Bacteria>Terrabacteria_group>Actinobacteria>Actinomycetia>Streptosporangiales	Sphaerisporangium_melleum	hypothetical-protein-GCM10007964_51680-[Sphaerisporangium-melleum]	321316
GII69421.1	DndB-ParB-nuclease	GCA_016863375.1	376	Bacteria>Terrabacteria_group>Actinobacteria>Actinomycetia>Streptosporangiales	Sphaerisporangium_melleum	hypothetical-protein-Sme01_18970-[Sphaerisporangium-melleum]	321316
PZG56086.1	DndB-ParB-nuclease	GCA_003236385.1	372	Bacteria>Terrabacteria_group>Actinobacteria>Actinomycetia>Streptosporangiales	Spongiactinospora_gelatinilytica	DNA-sulfur-modification-protein-DndB-[Spongiactinospora-gelatinilytica]	2666298
WP_233507017.1	DndB-ParB-nuclease	GCF_003236385.1	380	Bacteria>Terrabacteria_group>Actinobacteria>Actinomycetia>Streptosporangiales	Spongiactinospora_gelatinilytica	DNA-sulfur-modification-protein-DndB-[Spongiactinospora-gelatinilytica]	2666298
MBV9449981.1	DndB-ParB-nuclease	GCA_019245115.1	448	Bacteria>Terrabacteria_group>Actinobacteria>Actinomycetia>Streptosporangiales	Streptosporangiaceae_bacterium	DGQHR-domain-containing-protein-[Streptosporangiaceae-bacterium]	2339230
MBV9449974.1	DndB-ParB-nuclease	GCA_019245115.1	371	Bacteria>Terrabacteria_group>Actinobacteria>Actinomycetia>Streptosporangiales	Streptosporangiaceae_bacterium	DNA-sulfur-modification-protein-DndB-[Streptosporangiaceae-bacterium]	2339230
MBB4917451.1	DndB-ParB-nuclease	GCA_014203715.1	376	Bacteria>Terrabacteria_group>Actinobacteria>Actinomycetia>Streptosporangiales	Streptosporangium_saharense	DNA-sulfur-modification-protein-DndB-[Streptosporangium-saharense]	1706840
WP_184717664.1	DndB-ParB-nuclease	GCF_014203715.1	376	Bacteria>Terrabacteria_group>Actinobacteria>Actinomycetia>Streptosporangiales	Streptosporangium_saharense	DNA-sulfur-modification-protein-DndB-[Streptosporangium-saharense]	1706840
WP_184717682.1	DndB-ParB-nuclease	GCF_014203715.1	572	Bacteria>Terrabacteria_group>Actinobacteria>Actinomycetia>Streptosporangiales	Streptosporangium_saharense	hypothetical-protein-[Streptosporangium-saharense]	1706840
WP_169313066.1	DndB-ParB-nuclease	-	367	Bacteria>Terrabacteria_group>Actinobacteria>Actinomycetia>Streptosporangiales	Thermomonospora_curvata	DNA-sulfur-modification-protein-DndB-[Thermomonospora-curvata]	2020
WP_012850557.1	DndB-ParB-nuclease	-	720	Bacteria>Terrabacteria_group>Actinobacteria>Actinomycetia>Streptosporangiales	Thermomonospora_curvata	hypothetical-protein-[Thermomonospora-curvata]	2020
WP_165403845.1	DndB-ParB-nuclease	GCF_014640475.1	655	Bacteria>Terrabacteria_group>Actinobacteria>Nitriliruptoria>Egicoccales	Egicoccus_halophilus	hypothetical-protein-[Egicoccus-halophilus]	1670830
NLT05185.1	DndB-ParB-nuclease	GCA_012718975.1	373	Bacteria>Terrabacteria_group>Actinobacteria>Thermoleophilia>Solirubrobacterales	Solirubrobacterales_bacterium	DNA-sulfur-modification-protein-DndB-[Solirubrobacterales-bacterium]	1909295
MBX7136251.1	DndB-ParB-nuclease	GCA_019694835.1	360	Bacteria>Terrabacteria_group>Armatimonadetes>Fimbriimonadia>Fimbriimonadales	Fimbriimonadaceae_bacterium	DNA-sulfur-modification-protein-DndB-[Fimbriimonadaceae-bacterium]	2682144
WP_119072018.1	DndB-ParB-nuclease	GCF_003569045.1	334	Bacteria>Terrabacteria_group>Chloroflexi>Anaerolineae>Aggregatilineales	Aggregatilinea_lenta	DGQHR-domain-containing-protein-[Aggregatilinea-lenta]	913108
WP_119072012.1	DndB-ParB-nuclease	GCF_003569045.1	363	Bacteria>Terrabacteria_group>Chloroflexi>Anaerolineae>Aggregatilineales	Aggregatilinea_lenta	DNA-sulfur-modification-protein-DndB-[Aggregatilinea-lenta]	913108
NMC85959.1	DndB-ParB-nuclease	GCA_012797695.1	239	Bacteria>Terrabacteria_group>Chloroflexi>Anaerolineae>Anaerolineales	Anaerolineaceae_bacterium	DGQHR-domain-containing-protein-[Anaerolineaceae-bacterium]	2024896
MCA9900616.1	DndB-ParB-nuclease	GCA_020432515.1	423	Bacteria>Terrabacteria_group>Chloroflexi>Anaerolineae>Anaerolineales	Anaerolineales_bacterium	DGQHR-domain-containing-protein-[Anaerolineales-bacterium]	2073117
MCC6604722.1	DndB-ParB-nuclease	GCA_020848035.1	392	Bacteria>Terrabacteria_group>Chloroflexi>Anaerolineae>unclassified_Anaerolineae	Anaerolineae_bacterium	DGQHR-domain-containing-protein-[Anaerolineae-bacterium]	2052143
WP_205704058.1	DndB-ParB-nuclease	-	363	Bacteria>Terrabacteria_group>Chloroflexi>Chloroflexia>Chloroflexales	Candidatus_Chloroploca_sp._Khr17	DGQHR-domain-containing-protein-[Candidatus-Chloroploca-sp.-Khr17]	2496869
WP_129677576.1	DndB-ParB-nuclease	GCF_004138165.1	367	Bacteria>Terrabacteria_group>Chloroflexi>Chloroflexia>Chloroflexales	Candidatus_Chloroploca_sp._Khr17	DNA-sulfur-modification-protein-DndB-[Candidatus-Chloroploca-sp.-Khr17]	2496869
RRR72240.1	DndB-ParB-nuclease	GCA_003934145.1	367	Bacteria>Terrabacteria_group>Chloroflexi>Chloroflexia>Chloroflexales	Candidatus_Viridilinea_halotolerans	DNA-sulfur-modification-protein-DndB-[Candidatus-Viridilinea-halotolerans]	2491704
WP_097643793.1	DndB-ParB-nuclease	GCF_002532535.1	212	Bacteria>Terrabacteria_group>Chloroflexi>Chloroflexia>Chloroflexales	Candidatus_Viridilinea_mediisalina	DNA-sulfur-modification-protein-DndB-[Candidatus-Viridilinea-mediisalina]	2024553
WP_165774632.1	DndB-ParB-nuclease	GCF_002532535.1	356	Bacteria>Terrabacteria_group>Chloroflexi>Chloroflexia>Chloroflexales	Candidatus_Viridilinea_mediisalina	DNA-sulfur-modification-protein-DndB-[Candidatus-Viridilinea-mediisalina]	2024553
NJL06327.1	DndB-ParB-nuclease	GCA_012031225.1	368	Bacteria>Terrabacteria_group>Chloroflexi>Chloroflexia>Chloroflexales	Chloroflexaceae_bacterium	DNA-sulfur-modification-protein-DndB-[Chloroflexaceae-bacterium]	2562117
NNJ10294.1	DndB-ParB-nuclease	GCA_004138175.2	373	Bacteria>Terrabacteria_group>Chloroflexi>Chloroflexia>Chloroflexales	Chloroflexales_bacterium_ZM16-3	DNA-sulfur-modification-protein-DndB-[Chloroflexales-bacterium-ZM16-3]	2496870
NTW03706.1	DndB-ParB-nuclease	GCA_013336555.1	263	Bacteria>Terrabacteria_group>Chloroflexi>Chloroflexia>Chloroflexales	Oscillochloris_sp.	DGQHR-domain-containing-protein-[Oscillochloris-sp.]	2547424
WP_220597244.1	DndB-ParB-nuclease	GCF_019599295.1	361	Bacteria>Terrabacteria_group>Chloroflexi>Chloroflexia>Chloroflexales	Oscillochloris_sp._ZM17-4	DNA-sulfur-modification-protein-DndB-[Oscillochloris-sp.-ZM17-4]	2866714
RJQ36123.1	DndB-ParB-nuclease	GCA_003599745.1	365	Bacteria>Terrabacteria_group>Chloroflexi>Dehalococcoidia>unclassified_Dehalococcoidia	Dehalococcoidia_bacterium	DNA-sulfur-modification-protein-DndB-[Dehalococcoidia-bacterium]	2026734
NOK58225.1	DndB-ParB-nuclease	GCA_013122435.1	375	Bacteria>Terrabacteria_group>Chloroflexi>unclassified_Chloroflexi	Chloroflexi_bacterium_AL-N1	DNA-sulfur-modification-protein-DndB-[Chloroflexi-bacterium-AL-N1]	2686367
MCE7947205.1	DndB-ParB-nuclease	GCA_021404685.1	363	Bacteria>Terrabacteria_group>Chloroflexi>unclassified_Chloroflexi	Chloroflexi_bacterium_CFX4	DNA-sulfur-modification-protein-DndB-[Chloroflexi-bacterium-CFX4]	2293709
MBI2830757.1	DndB-ParB-nuclease	GCA_016190925.1	421	Bacteria>Terrabacteria_group>Chloroflexi>unclassified_Chloroflexi	Chloroflexi_bacterium	DGQHR-domain-containing-protein-[Chloroflexi-bacterium]	2026724
MYA59521.1	DndB-ParB-nuclease	GCA_009840665.1	242	Bacteria>Terrabacteria_group>Chloroflexi>unclassified_Chloroflexi	Chloroflexi_bacterium	DGQHR-domain-containing-protein-[Chloroflexi-bacterium]	2026724
NWJ46936.1	DndB-ParB-nuclease	GCA_013390565.1	355	Bacteria>Terrabacteria_group>Chloroflexi>unclassified_Chloroflexi	Chloroflexi_bacterium	DGQHR-domain-containing-protein-[Chloroflexi-bacterium]	2026724
NWJ95387.1	DndB-ParB-nuclease	GCA_013390945.1	317	Bacteria>Terrabacteria_group>Chloroflexi>unclassified_Chloroflexi	Chloroflexi_bacterium	DGQHR-domain-containing-protein-[Chloroflexi-bacterium]	2026724
HDQ71120.1	DndB-ParB-nuclease	GCA_011046405.1	371	Bacteria>Terrabacteria_group>Chloroflexi>unclassified_Chloroflexi	Chloroflexi_bacterium	DNA-sulfur-modification-protein-DndB-[Chloroflexi-bacterium]	2026724
HDQ71127.1	DndB-ParB-nuclease	GCA_011046405.1	363	Bacteria>Terrabacteria_group>Chloroflexi>unclassified_Chloroflexi	Chloroflexi_bacterium	DNA-sulfur-modification-protein-DndB-[Chloroflexi-bacterium]	2026724
MCA0457132.1	DndB-ParB-nuclease	GCA_020161935.1	363	Bacteria>Terrabacteria_group>Chloroflexi>unclassified_Chloroflexi	Chloroflexi_bacterium	DNA-sulfur-modification-protein-DndB-[Chloroflexi-bacterium]	2026724
NWJ46932.1	DndB-ParB-nuclease	GCA_013390565.1	365	Bacteria>Terrabacteria_group>Chloroflexi>unclassified_Chloroflexi	Chloroflexi_bacterium	DNA-sulfur-modification-protein-DndB-[Chloroflexi-bacterium]	2026724
RLC79940.1	DndB-ParB-nuclease	GCA_003648025.1	370	Bacteria>Terrabacteria_group>Chloroflexi>unclassified_Chloroflexi	Chloroflexi_bacterium	DNA-sulfur-modification-protein-DndB-[Chloroflexi-bacterium]	2026724
RLC86281.1	DndB-ParB-nuclease	GCA_003648075.1	370	Bacteria>Terrabacteria_group>Chloroflexi>unclassified_Chloroflexi	Chloroflexi_bacterium	DNA-sulfur-modification-protein-DndB-[Chloroflexi-bacterium]	2026724
RMG70434.1	DndB-ParB-nuclease	GCA_003696565.1	368	Bacteria>Terrabacteria_group>Chloroflexi>unclassified_Chloroflexi	Chloroflexi_bacterium	DNA-sulfur-modification-protein-DndB-[Chloroflexi-bacterium]	2026724
MBY0358216.1	DndB-ParB-nuclease	GCA_019750335.1	416	Bacteria>Terrabacteria_group>Cyanobacteria/Melainabacteria_group>Candidatus_Melainabacteria>Candidatus_Obscuribacterales	Candidatus_Obscuribacterales_bacterium	DGQHR-domain-containing-protein-[Candidatus-Obscuribacterales-bacterium]	2364082
KJH73125.1	DndB-ParB-nuclease	GCA_000952155.1	577	Bacteria>Terrabacteria_group>Cyanobacteria/Melainabacteria_group>Cyanobacteria>Chroococcidiopsidales	Aliterella_atlantica_CENA595	hypothetical-protein-UH38_03440-[Aliterella-atlantica-CENA595]	1618023
WP_045053223.1	DndB-ParB-nuclease	GCF_000952155.1	577	Bacteria>Terrabacteria_group>Cyanobacteria/Melainabacteria_group>Cyanobacteria>Chroococcidiopsidales	Aliterella_atlantica	hypothetical-protein-[Aliterella-atlantica]	1827278
MBE9017875.1	DndB-ParB-nuclease	GCA_015206905.1	523	Bacteria>Terrabacteria_group>Cyanobacteria/Melainabacteria_group>Cyanobacteria>Chroococcidiopsidales	Chroococcidiopsidales_cyanobacterium_LEGE_13417	DGQHR-domain-containing-protein-[Chroococcidiopsidales-cyanobacterium-LEGE-13417]	1828829
MBE9017876.1	DndB-ParB-nuclease	GCA_015206905.1	542	Bacteria>Terrabacteria_group>Cyanobacteria/Melainabacteria_group>Cyanobacteria>Chroococcidiopsidales	Chroococcidiopsidales_cyanobacterium_LEGE_13417	hypothetical-protein-[Chroococcidiopsidales-cyanobacterium-LEGE-13417]	1828829
WP_106166477.1	DndB-ParB-nuclease	GCF_003003835.1	524	Bacteria>Terrabacteria_group>Cyanobacteria/Melainabacteria_group>Cyanobacteria>Chroococcidiopsidales	Chroococcidiopsis_cubana	DGQHR-domain-containing-protein-[Chroococcidiopsis-cubana]	171392
WP_206751815.1	DndB-ParB-nuclease	GCF_003991895.1	348	Bacteria>Terrabacteria_group>Cyanobacteria/Melainabacteria_group>Cyanobacteria>Chroococcidiopsidales	Chroococcidiopsis_cubana	DNA-sulfur-modification-protein-DndB-[Chroococcidiopsis-cubana]	171392
WP_106546997.1	DndB-ParB-nuclease	GCF_003015105.1	523	Bacteria>Terrabacteria_group>Cyanobacteria/Melainabacteria_group>Cyanobacteria>Chroococcidiopsidales	Chroococcidiopsis_sp._CCALA_051	DGQHR-domain-containing-protein-[Chroococcidiopsis-sp.-CCALA-051]	869949
PSM47328.1	DndB-ParB-nuclease	GCA_003015105.1	523	Bacteria>Terrabacteria_group>Cyanobacteria/Melainabacteria_group>Cyanobacteria>Chroococcidiopsidales	Chroococcidiopsis_sp._CCALA_051	hypothetical-protein-C7Y66_20305-[Chroococcidiopsis-sp.-CCALA-051]	869949
WP_192160748.1	DndB-ParB-nuclease	GCF_014696895.1	524	Bacteria>Terrabacteria_group>Cyanobacteria/Melainabacteria_group>Cyanobacteria>Chroococcidiopsidales	Chroococcidiopsis_sp._FACHB-1243	DGQHR-domain-containing-protein-[Chroococcidiopsis-sp.-FACHB-1243]	2692781
WP_192160749.1	DndB-ParB-nuclease	GCF_014696895.1	394	Bacteria>Terrabacteria_group>Cyanobacteria/Melainabacteria_group>Cyanobacteria>Chroococcidiopsidales	Chroococcidiopsis_sp._FACHB-1243	DNA-sulfur-modification-protein-DndB-[Chroococcidiopsis-sp.-FACHB-1243]	2692781
WP_104545194.1	DndB-ParB-nuclease	-	522	Bacteria>Terrabacteria_group>Cyanobacteria/Melainabacteria_group>Cyanobacteria>Chroococcidiopsidales	Chroococcidiopsis_sp._TS-821	DGQHR-domain-containing-protein-[Chroococcidiopsis-sp.-TS-821]	1378066
WP_015154698.1	DndB-ParB-nuclease	GCF_000317125.1	523	Bacteria>Terrabacteria_group>Cyanobacteria/Melainabacteria_group>Cyanobacteria>Chroococcidiopsidales	Chroococcidiopsis_thermalis	DGQHR-domain-containing-protein-[Chroococcidiopsis-thermalis]	54299
WP_219336150.1	DndB-ParB-nuclease	GCF_000317125.1	669	Bacteria>Terrabacteria_group>Cyanobacteria/Melainabacteria_group>Cyanobacteria>Chroococcidiopsidales	Chroococcidiopsis_thermalis	DNA-sulfur-modification-protein-DndB-[Chroococcidiopsis-thermalis]	54299
WP_190427678.1	DndB-ParB-nuclease	GCF_014695585.1	532	Bacteria>Terrabacteria_group>Cyanobacteria/Melainabacteria_group	Cyanobacteria	DGQHR-domain-containing-protein-[Microcoleus-sp.-FACHB-831]	1117
WP_190427692.1	DndB-ParB-nuclease	-	387	Bacteria>Terrabacteria_group>Cyanobacteria/Melainabacteria_group	Cyanobacteria	MULTISPECIES:-DGQHR-domain-containing-protein-[Cyanobacteria]	1117
WP_208345814.1	DndB-ParB-nuclease	GCF_017591665.1	532	Bacteria>Terrabacteria_group>Cyanobacteria/Melainabacteria_group>Cyanobacteria>Nostocales	Aetokthonos_hydrillicola	DGQHR-domain-containing-protein-[Aetokthonos-hydrillicola]	1550245
WP_194006182.1	DndB-ParB-nuclease	GCF_015206995.1	532	Bacteria>Terrabacteria_group>Cyanobacteria/Melainabacteria_group>Cyanobacteria>Nostocales	aff._Roholtiella_sp._LEGE_12411	DGQHR-domain-containing-protein-[aff.-Roholtiella-sp.-LEGE-12411]	1828822
WP_198127572.1	DndB-ParB-nuclease	GCF_016056305.1	532	Bacteria>Terrabacteria_group>Cyanobacteria/Melainabacteria_group>Cyanobacteria>Nostocales	Amazonocrinis_nigriterrae	DGQHR-domain-containing-protein-[Amazonocrinis-nigriterrae]	2840443
WP_198127571.1	DndB-ParB-nuclease	GCF_016056305.1	389	Bacteria>Terrabacteria_group>Cyanobacteria/Melainabacteria_group>Cyanobacteria>Nostocales	Amazonocrinis_nigriterrae	DNA-sulfur-modification-protein-DndB-[Amazonocrinis-nigriterrae]	2840443
WP_190474877.1	DndB-ParB-nuclease	GCF_014697625.1	532	Bacteria>Terrabacteria_group>Cyanobacteria/Melainabacteria_group>Cyanobacteria>Nostocales	Anabaena_azotica	DGQHR-domain-containing-protein-[Anabaena-azotica]	197653
WP_190474879.1	DndB-ParB-nuclease	GCF_014697625.1	396	Bacteria>Terrabacteria_group>Cyanobacteria/Melainabacteria_group>Cyanobacteria>Nostocales	Anabaena_azotica	DNA-sulfur-modification-protein-DndB-[Anabaena-azotica]	197653
MBD2502533.1	DndB-ParB-nuclease	GCA_014697625.1	532	Bacteria>Terrabacteria_group>Cyanobacteria/Melainabacteria_group>Cyanobacteria>Nostocales	Anabaena_azotica_FACHB-119	DGQHR-domain-containing-protein-[Anabaena-azotica-FACHB-119]	947527
MBD2502534.1	DndB-ParB-nuclease	GCA_014697625.1	396	Bacteria>Terrabacteria_group>Cyanobacteria/Melainabacteria_group>Cyanobacteria>Nostocales	Anabaena_azotica_FACHB-119	DNA-sulfur-modification-protein-DndB-[Anabaena-azotica-FACHB-119]	947527
WP_190906909.1	DndB-ParB-nuclease	GCF_014698735.1	533	Bacteria>Terrabacteria_group>Cyanobacteria/Melainabacteria_group>Cyanobacteria>Nostocales	Anabaena_catenula	DGQHR-domain-containing-protein-[Anabaena-catenula]	1296320
WP_190906908.1	DndB-ParB-nuclease	-	400	Bacteria>Terrabacteria_group>Cyanobacteria/Melainabacteria_group>Cyanobacteria>Nostocales	Anabaena_catenula	DNA-sulfur-modification-protein-DndB-[Anabaena-catenula]	1296320
MBD2692525.1	DndB-ParB-nuclease	GCA_014698735.1	533	Bacteria>Terrabacteria_group>Cyanobacteria/Melainabacteria_group>Cyanobacteria>Nostocales	Anabaena_catenula_FACHB-362	DGQHR-domain-containing-protein-[Anabaena-catenula-FACHB-362]	2692877
MBD2692524.1	DndB-ParB-nuclease	GCA_014698735.1	400	Bacteria>Terrabacteria_group>Cyanobacteria/Melainabacteria_group>Cyanobacteria>Nostocales	Anabaena_catenula_FACHB-362	DNA-sulfur-modification-protein-DndB-[Anabaena-catenula-FACHB-362]	2692877
MBD2285238.1	DndB-ParB-nuclease	GCA_014696775.1	532	Bacteria>Terrabacteria_group>Cyanobacteria/Melainabacteria_group>Cyanobacteria>Nostocales	Anabaena_cylindrica_FACHB-170	DGQHR-domain-containing-protein-[Anabaena-cylindrica-FACHB-170]	2692878
MBD2173232.1	DndB-ParB-nuclease	GCA_014696465.1	532	Bacteria>Terrabacteria_group>Cyanobacteria/Melainabacteria_group>Cyanobacteria>Nostocales	Anabaena_cylindrica_FACHB-318	DGQHR-domain-containing-protein-[Anabaena-cylindrica-FACHB-318]	2692880
WP_015216814.1	DndB-ParB-nuclease	GCF_000317695.1	389	Bacteria>Terrabacteria_group>Cyanobacteria/Melainabacteria_group>Cyanobacteria>Nostocales	Anabaena	DGQHR-domain-containing-protein-[Anabaena-cylindrica]	1163
WP_015216816.1	DndB-ParB-nuclease	GCF_000317695.1	532	Bacteria>Terrabacteria_group>Cyanobacteria/Melainabacteria_group>Cyanobacteria>Nostocales	Anabaena	DGQHR-domain-containing-protein-[Anabaena-cylindrica]	1163
WP_190711471.1	DndB-ParB-nuclease	GCF_014698305.1	533	Bacteria>Terrabacteria_group>Cyanobacteria/Melainabacteria_group>Cyanobacteria>Nostocales	Anabaena_lutea	DGQHR-domain-containing-protein-[Anabaena-lutea]	212350
WP_190711470.1	DndB-ParB-nuclease	GCF_014698305.1	401	Bacteria>Terrabacteria_group>Cyanobacteria/Melainabacteria_group>Cyanobacteria>Nostocales	Anabaena_lutea	DNA-sulfur-modification-protein-DndB-[Anabaena-lutea]	212350
MBD2566660.1	DndB-ParB-nuclease	GCA_014698305.1	533	Bacteria>Terrabacteria_group>Cyanobacteria/Melainabacteria_group>Cyanobacteria>Nostocales	Anabaena_lutea_FACHB-196	DGQHR-domain-containing-protein-[Anabaena-lutea-FACHB-196]	2692881
MBD2566659.1	DndB-ParB-nuclease	GCA_014698305.1	401	Bacteria>Terrabacteria_group>Cyanobacteria/Melainabacteria_group>Cyanobacteria>Nostocales	Anabaena_lutea_FACHB-196	DNA-sulfur-modification-protein-DndB-[Anabaena-lutea-FACHB-196]	2692881
WP_190702558.1	DndB-ParB-nuclease	GCF_014697125.1	532	Bacteria>Terrabacteria_group>Cyanobacteria/Melainabacteria_group>Cyanobacteria>Nostocales	Anabaena_minutissima	DGQHR-domain-containing-protein-[Anabaena-minutissima]	2303152
WP_066425724.1	DndB-ParB-nuclease	GCF_001597745.1	532	Bacteria>Terrabacteria_group>Cyanobacteria/Melainabacteria_group>Cyanobacteria>Nostocales	Anabaena_sp._4-3	DGQHR-domain-containing-protein-[Anabaena-sp.-4-3]	1811979
MCE2718162.1	DndB-ParB-nuclease	GCA_021300315.1	533	Bacteria>Terrabacteria_group>Cyanobacteria/Melainabacteria_group>Cyanobacteria>Nostocales	Anabaena_sp._49628_E55	DGQHR-domain-containing-protein-[Anabaena-sp.-49628_E55]	2904988
MCE2718161.1	DndB-ParB-nuclease	GCA_021300315.1	343	Bacteria>Terrabacteria_group>Cyanobacteria/Melainabacteria_group>Cyanobacteria>Nostocales	Anabaena_sp._49628_E55	DNA-sulfur-modification-protein-DndB-[Anabaena-sp.-49628_E55]	2904988
AFW94735.1	DndB-ParB-nuclease	GCA_000312705.1	532	Bacteria>Terrabacteria_group>Cyanobacteria/Melainabacteria_group>Cyanobacteria>Nostocales	Anabaena_sp._90	DGQHR-domain-containing-protein-[Anabaena-sp.-90]	46234
WP_015079896.1	DndB-ParB-nuclease	GCF_000312705.1	532	Bacteria>Terrabacteria_group>Cyanobacteria/Melainabacteria_group>Cyanobacteria>Nostocales	Anabaena_sp._90	DGQHR-domain-containing-protein-[Anabaena-sp.-90]	46234
OBQ07588.1	DndB-ParB-nuclease	GCA_001672255.1	391	Bacteria>Terrabacteria_group>Cyanobacteria/Melainabacteria_group>Cyanobacteria>Nostocales	Anabaena_sp._AL09	hypothetical-protein-AN490_11545-[Anabaena-sp.-AL09]	1710891
OBQ07589.1	DndB-ParB-nuclease	GCA_001672255.1	533	Bacteria>Terrabacteria_group>Cyanobacteria/Melainabacteria_group>Cyanobacteria>Nostocales	Anabaena_sp._AL09	hypothetical-protein-AN490_11550-[Anabaena-sp.-AL09]	1710891
OBQ21648.1	DndB-ParB-nuclease	GCA_001672085.1	532	Bacteria>Terrabacteria_group>Cyanobacteria/Melainabacteria_group>Cyanobacteria>Nostocales	Anabaena_sp._AL93	hypothetical-protein-AN486_03850-[Anabaena-sp.-AL93]	1678133
WP_066374765.1	DndB-ParB-nuclease	GCF_001597855.1	532	Bacteria>Terrabacteria_group>Cyanobacteria/Melainabacteria_group>Cyanobacteria>Nostocales	Anabaena_sp._CA_=_ATCC_33047	DGQHR-domain-containing-protein-[Anabaena-sp.-CA-=-ATCC-33047]	52271
WP_084386859.1	DndB-ParB-nuclease	GCF_001597855.1	396	Bacteria>Terrabacteria_group>Cyanobacteria/Melainabacteria_group>Cyanobacteria>Nostocales	Anabaena_sp._CA_=_ATCC_33047	DNA-sulfur-modification-protein-DndB-[Anabaena-sp.-CA-=-ATCC-33047]	52271
OBQ40416.1	DndB-ParB-nuclease	GCA_001672075.1	661	Bacteria>Terrabacteria_group>Cyanobacteria/Melainabacteria_group>Cyanobacteria>Nostocales	Anabaena_sp._CRKS33	hypothetical-protein-AN487_01735-[Anabaena-sp.-CRKS33]	1710888
OBQ40417.1	DndB-ParB-nuclease	GCA_001672075.1	532	Bacteria>Terrabacteria_group>Cyanobacteria/Melainabacteria_group>Cyanobacteria>Nostocales	Anabaena_sp._CRKS33	hypothetical-protein-AN487_01740-[Anabaena-sp.-CRKS33]	1710888
WP_190368618.1	DndB-ParB-nuclease	GCF_014696225.1	530	Bacteria>Terrabacteria_group>Cyanobacteria/Melainabacteria_group>Cyanobacteria>Nostocales	Anabaena_sp._FACHB-1237	DGQHR-domain-containing-protein-[Anabaena-sp.-FACHB-1237]	2692769
WP_190396643.1	DndB-ParB-nuclease	GCF_014696335.1	532	Bacteria>Terrabacteria_group>Cyanobacteria/Melainabacteria_group>Cyanobacteria>Nostocales	Anabaena_sp._FACHB-1250	DGQHR-domain-containing-protein-[Anabaena-sp.-FACHB-1250]	2692770
WP_190396642.1	DndB-ParB-nuclease	GCF_014696335.1	661	Bacteria>Terrabacteria_group>Cyanobacteria/Melainabacteria_group>Cyanobacteria>Nostocales	Anabaena_sp._FACHB-1250	hypothetical-protein-[Anabaena-sp.-FACHB-1250]	2692770
WP_190371209.1	DndB-ParB-nuclease	GCF_014696755.1	534	Bacteria>Terrabacteria_group>Cyanobacteria/Melainabacteria_group>Cyanobacteria>Nostocales	Anabaena_sp._FACHB-1391	DGQHR-domain-containing-protein-[Anabaena-sp.-FACHB-1391]	2692771
MBD2264983.1	DndB-ParB-nuclease	GCA_014696765.1	532	Bacteria>Terrabacteria_group>Cyanobacteria/Melainabacteria_group>Cyanobacteria>Nostocales	Anabaena_sp._FACHB-709	DGQHR-domain-containing-protein-[Anabaena-sp.-FACHB-709]	1086822
MBD2477440.1	DndB-ParB-nuclease	GCA_014697485.1	532	Bacteria>Terrabacteria_group>Cyanobacteria/Melainabacteria_group>Cyanobacteria>Nostocales	Anabaena_sp._FACHB-83	DGQHR-domain-containing-protein-[Anabaena-sp.-FACHB-83]	2692772
WP_190556379.1	DndB-ParB-nuclease	GCF_014696825.1	532	Bacteria>Terrabacteria_group>Cyanobacteria/Melainabacteria_group>Cyanobacteria>Nostocales	Anabaena_sphaerica	DGQHR-domain-containing-protein-[Anabaena-sphaerica]	212446
MBD2292192.1	DndB-ParB-nuclease	GCA_014696825.1	532	Bacteria>Terrabacteria_group>Cyanobacteria/Melainabacteria_group>Cyanobacteria>Nostocales	Anabaena_sphaerica_FACHB-251	DGQHR-domain-containing-protein-[Anabaena-sphaerica-FACHB-251]	2692883
OBQ08857.1	DndB-ParB-nuclease	GCA_001672225.1	401	Bacteria>Terrabacteria_group>Cyanobacteria/Melainabacteria_group>Cyanobacteria>Nostocales	Anabaena_sp._LE011-02	hypothetical-protein-AN482_12400-[Anabaena-sp.-LE011-02]	1710892
OBQ08858.1	DndB-ParB-nuclease	GCA_001672225.1	533	Bacteria>Terrabacteria_group>Cyanobacteria/Melainabacteria_group>Cyanobacteria>Nostocales	Anabaena_sp._LE011-02	hypothetical-protein-AN482_12405-[Anabaena-sp.-LE011-02]	1710892
OBQ41785.1	DndB-ParB-nuclease	GCA_001672195.1	222	Bacteria>Terrabacteria_group>Cyanobacteria/Melainabacteria_group>Cyanobacteria>Nostocales	Anabaena_sp._MDT14b	hypothetical-protein-AN485_01080,-partial-[Anabaena-sp.-MDT14b]	1710886
WP_016953349.1	DndB-ParB-nuclease	GCF_000332135.1	532	Bacteria>Terrabacteria_group>Cyanobacteria/Melainabacteria_group>Cyanobacteria>Nostocales	Anabaena_sp._PCC_7108	DGQHR-domain-containing-protein-[Anabaena-sp.-PCC-7108]	163908
WP_168502833.1	DndB-ParB-nuclease	GCF_009712035.1	533	Bacteria>Terrabacteria_group>Cyanobacteria/Melainabacteria_group>Cyanobacteria>Nostocales	Anabaena_sp._UHCC_0187	DGQHR-domain-containing-protein-[Anabaena-sp.-UHCC-0187]	2590018
WP_168502834.1	DndB-ParB-nuclease	GCF_009712035.1	396	Bacteria>Terrabacteria_group>Cyanobacteria/Melainabacteria_group>Cyanobacteria>Nostocales	Anabaena_sp._UHCC_0187	DNA-sulfur-modification-protein-DndB-[Anabaena-sp.-UHCC-0187]	2590018
ALB42518.1	DndB-ParB-nuclease	GCA_001277295.1	401	Bacteria>Terrabacteria_group>Cyanobacteria/Melainabacteria_group>Cyanobacteria>Nostocales	Anabaena_sp._WA102	DGQHR-domain-containing-protein-[Anabaena-sp.-WA102]	1647413
ALB42519.1	DndB-ParB-nuclease	GCA_001277295.1	533	Bacteria>Terrabacteria_group>Cyanobacteria/Melainabacteria_group>Cyanobacteria>Nostocales	Anabaena_sp._WA102	DGQHR-domain-containing-protein-[Anabaena-sp.-WA102]	1647413
WP_053540434.1	DndB-ParB-nuclease	GCF_001277295.1	401	Bacteria>Terrabacteria_group>Cyanobacteria/Melainabacteria_group>Cyanobacteria>Nostocales	Anabaena_sp._WA102	DNA-sulfur-modification-protein-DndB-[Anabaena-sp.-WA102]	1647413
OBQ21148.1	DndB-ParB-nuclease	GCA_001672155.1	532	Bacteria>Terrabacteria_group>Cyanobacteria/Melainabacteria_group>Cyanobacteria>Nostocales	Anabaena_sp._WA113	hypothetical-protein-AN488_11065-[Anabaena-sp.-WA113]	1710889
WP_190405670.1	DndB-ParB-nuclease	GCF_014697105.1	532	Bacteria>Terrabacteria_group>Cyanobacteria/Melainabacteria_group>Cyanobacteria>Nostocales	Anabaena_subtropica	DGQHR-domain-containing-protein-[Anabaena-subtropica]	425380
WP_190405671.1	DndB-ParB-nuclease	GCF_014697105.1	396	Bacteria>Terrabacteria_group>Cyanobacteria/Melainabacteria_group>Cyanobacteria>Nostocales	Anabaena_subtropica	DNA-sulfur-modification-protein-DndB-[Anabaena-subtropica]	425380
MBD2343188.1	DndB-ParB-nuclease	GCA_014697105.1	532	Bacteria>Terrabacteria_group>Cyanobacteria/Melainabacteria_group>Cyanobacteria>Nostocales	Anabaena_subtropica_FACHB-260	DGQHR-domain-containing-protein-[Anabaena-subtropica-FACHB-260]	2692884
MBD2343189.1	DndB-ParB-nuclease	GCA_014697105.1	396	Bacteria>Terrabacteria_group>Cyanobacteria/Melainabacteria_group>Cyanobacteria>Nostocales	Anabaena_subtropica_FACHB-260	DNA-sulfur-modification-protein-DndB-[Anabaena-subtropica-FACHB-260]	2692884
WP_096577133.1	DndB-ParB-nuclease	GCF_002367975.1	532	Bacteria>Terrabacteria_group>Cyanobacteria/Melainabacteria_group>Cyanobacteria>Nostocales	Anabaenopsis_circularis	DGQHR-domain-containing-protein-[Anabaenopsis-circularis]	109266
WP_096577129.1	DndB-ParB-nuclease	GCF_002367975.1	665	Bacteria>Terrabacteria_group>Cyanobacteria/Melainabacteria_group>Cyanobacteria>Nostocales	Anabaenopsis_circularis	hypothetical-protein-[Anabaenopsis-circularis]	109266
QOV23073.1	DndB-ParB-nuclease	GCA_015245355.1	526	Bacteria>Terrabacteria_group>Cyanobacteria/Melainabacteria_group>Cyanobacteria>Nostocales	Anabaenopsis_elenkinii_CCIBt3563	DGQHR-domain-containing-protein-[Anabaenopsis-elenkinii-CCIBt3563]	2779889

Accession	Annotation	GCA	Length	Phyletic.spread	Strain.name	Defline	Species.Taxid
WP_200988677.1	DndB-ParB-nuclease	GCF_015245355.1	526	Bacteria>Terrabacteria_group>Cyanobacteria/Melainabacteria_group>Cyanobacteria>Nostocales	Anabaenopsis_elenkinii	DGQHR-domain-containing-protein-[Anabaenopsis-elenkinii]	156213
KHG41074.1	DndB-ParB-nuclease	GCA_000789435.1	533	Bacteria>Terrabacteria_group>Cyanobacteria/Melainabacteria_group>Cyanobacteria>Nostocales	Aphanizomenon_flos-aquae_2012/KM1/D3	DGQHR-domain-containing-protein-[Aphanizomenon-flos-aquae-2012/KM1/D3]	1532906
MBD1218426.1	DndB-ParB-nuclease	GCA_014654815.1	532	Bacteria>Terrabacteria_group>Cyanobacteria/Melainabacteria_group>Cyanobacteria>Nostocales	Aphanizomenon_flos-aquae_Clear-A1	DGQHR-domain-containing-protein-[Aphanizomenon-flos-aquae-Clear-A1]	2766972
MBO1060910.1	DndB-ParB-nuclease	GCA_017355665.1	532	Bacteria>Terrabacteria_group>Cyanobacteria/Melainabacteria_group>Cyanobacteria>Nostocales	Aphanizomenon_flos-aquae_CP01	DGQHR-domain-containing-protein-[Aphanizomenon-flos-aquae-CP01]	2717881
QSV66184.1	DndB-ParB-nuclease	GCA_017346855.1	533	Bacteria>Terrabacteria_group>Cyanobacteria/Melainabacteria_group>Cyanobacteria>Nostocales	Aphanizomenon_flos-aquae_DEX188	DGQHR-domain-containing-protein-[Aphanizomenon-flos-aquae-DEX188]	2717882
QSV66185.1	DndB-ParB-nuclease	GCA_017346855.1	401	Bacteria>Terrabacteria_group>Cyanobacteria/Melainabacteria_group>Cyanobacteria>Nostocales	Aphanizomenon_flos-aquae_DEX188	hypothetical-protein-HEQ12_03945-[Aphanizomenon-flos-aquae-DEX188]	2717882
WP_027404586.1	DndB-ParB-nuclease	GCF_024584745.1	532	Bacteria>Terrabacteria_group>Cyanobacteria/Melainabacteria_group>Cyanobacteria>Nostocales	Aphanizomenon_flos-aquae	DGQHR-domain-containing-protein-[Aphanizomenon-flos-aquae]	1176
WP_190383618.1	DndB-ParB-nuclease	GCF_014696815.1	532	Bacteria>Terrabacteria_group>Cyanobacteria/Melainabacteria_group>Cyanobacteria>Nostocales	Aphanizomenon_flos-aquae	DGQHR-domain-containing-protein-[Aphanizomenon-flos-aquae]	1176
WP_190383617.1	DndB-ParB-nuclease	GCF_014696815.1	661	Bacteria>Terrabacteria_group>Cyanobacteria/Melainabacteria_group>Cyanobacteria>Nostocales	Aphanizomenon_flos-aquae	hypothetical-protein-[Aphanizomenon-flos-aquae]	1176
QSV73953.1	DndB-ParB-nuclease	GCA_017346875.1	533	Bacteria>Terrabacteria_group>Cyanobacteria/Melainabacteria_group>Cyanobacteria>Nostocales	Aphanizomenon_flos-aquae_KM1D3_PB	DGQHR-domain-containing-protein-[Aphanizomenon-flos-aquae-KM1D3_PB]	2723857
QSV74539.1	DndB-ParB-nuclease	GCA_017346875.1	401	Bacteria>Terrabacteria_group>Cyanobacteria/Melainabacteria_group>Cyanobacteria>Nostocales	Aphanizomenon_flos-aquae_KM1D3_PB	DGQHR-domain-containing-protein-[Aphanizomenon-flos-aquae-KM1D3_PB]	2723857
MBO1044685.1	DndB-ParB-nuclease	GCA_001593825.2	532	Bacteria>Terrabacteria_group>Cyanobacteria/Melainabacteria_group>Cyanobacteria>Nostocales	Aphanizomenon_flos-aquae_UKL13-PB	DGQHR-domain-containing-protein-[Aphanizomenon-flos-aquae-UKL13-PB]	1803587
OBQ44766.1	DndB-ParB-nuclease	GCA_001672105.1	532	Bacteria>Terrabacteria_group>Cyanobacteria/Melainabacteria_group>Cyanobacteria>Nostocales	Aphanizomenon_flos-aquae_WA102	hypothetical-protein-AN484_05395-[Aphanizomenon-flos-aquae-WA102]	1710896
WP_168466407.1	DndB-ParB-nuclease	GCF_009712065.1	532	Bacteria>Terrabacteria_group>Cyanobacteria/Melainabacteria_group>Cyanobacteria>Nostocales	Aphanizomenon_sp._UHCC_0183	DGQHR-domain-containing-protein-[Aphanizomenon-sp.-UHCC-0183]	2590028
MBH8555648.1	DndB-ParB-nuclease	GCA_016056295.1	533	Bacteria>Terrabacteria_group>Cyanobacteria/Melainabacteria_group>Cyanobacteria>Nostocales	Atlanticothrix_silvestris_CENA357	DGQHR-domain-containing-protein-[Atlanticothrix-silvestris-CENA357]	1725252
MBH8555649.1	DndB-ParB-nuclease	GCA_016056295.1	319	Bacteria>Terrabacteria_group>Cyanobacteria/Melainabacteria_group>Cyanobacteria>Nostocales	Atlanticothrix_silvestris_CENA357	hypothetical-protein-[Atlanticothrix-silvestris-CENA357]	1725252
MBH8555650.1	DndB-ParB-nuclease	GCA_016056295.1	232	Bacteria>Terrabacteria_group>Cyanobacteria/Melainabacteria_group>Cyanobacteria>Nostocales	Atlanticothrix_silvestris_CENA357	hypothetical-protein-[Atlanticothrix-silvestris-CENA357]	1725252
WP_214441847.1	DndB-ParB-nuclease	GCF_016056295.1	533	Bacteria>Terrabacteria_group>Cyanobacteria/Melainabacteria_group>Cyanobacteria>Nostocales	Atlanticothrix_silvestris	DGQHR-domain-containing-protein-[Atlanticothrix-silvestris]	2840444
MBD2487716.1	DndB-ParB-nuclease	GCA_014698045.1	533	Bacteria>Terrabacteria_group>Cyanobacteria/Melainabacteria_group>Cyanobacteria>Nostocales	Aulosira_sp._FACHB-615	DGQHR-domain-containing-protein-[Aulosira-sp.-FACHB-615]	2692777
WP_190871385.1	DndB-ParB-nuclease	GCF_014698045.1	533	Bacteria>Terrabacteria_group>Cyanobacteria/Melainabacteria_group>Cyanobacteria>Nostocales	Aulosira_sp._FACHB-615	DGQHR-domain-containing-protein-[Aulosira-sp.-FACHB-615]	2692777
MBD2487717.1	DndB-ParB-nuclease	GCA_014698045.1	389	Bacteria>Terrabacteria_group>Cyanobacteria/Melainabacteria_group>Cyanobacteria>Nostocales	Aulosira_sp._FACHB-615	DNA-sulfur-modification-protein-DndB-[Aulosira-sp.-FACHB-615]	2692777
WP_190871386.1	DndB-ParB-nuclease	GCF_014698045.1	389	Bacteria>Terrabacteria_group>Cyanobacteria/Melainabacteria_group>Cyanobacteria>Nostocales	Aulosira_sp._FACHB-615	DNA-sulfur-modification-protein-DndB-[Aulosira-sp.-FACHB-615]	2692777
MBW4591773.1	DndB-ParB-nuclease	GCA_019359695.1	533	Bacteria>Terrabacteria_group>Cyanobacteria/Melainabacteria_group>Cyanobacteria>Nostocales	Brasilonema_angustatum_HA4187-MV1	DGQHR-domain-containing-protein-[Brasilonema-angustatum-HA4187-MV1]	1049008
MBW4591772.1	DndB-ParB-nuclease	GCA_019359695.1	556	Bacteria>Terrabacteria_group>Cyanobacteria/Melainabacteria_group>Cyanobacteria>Nostocales	Brasilonema_angustatum_HA4187-MV1	DNA-sulfur-modification-protein-DndB-[Brasilonema-angustatum-HA4187-MV1]	1049008
WP_169156721.1	DndB-ParB-nuclease	GCF_012912135.1	532	Bacteria>Terrabacteria_group>Cyanobacteria/Melainabacteria_group>Cyanobacteria>Nostocales	Brasilonema_bromeliae	DGQHR-domain-containing-protein-[Brasilonema-bromeliae]	383615
WP_169156722.1	DndB-ParB-nuclease	GCF_012912135.1	391	Bacteria>Terrabacteria_group>Cyanobacteria/Melainabacteria_group>Cyanobacteria>Nostocales	Brasilonema_bromeliae	DNA-sulfur-modification-protein-DndB-[Brasilonema-bromeliae]	383615
NMG21484.1	DndB-ParB-nuclease	GCA_012912135.1	532	Bacteria>Terrabacteria_group>Cyanobacteria/Melainabacteria_group>Cyanobacteria>Nostocales	Brasilonema_bromeliae_SPC951	hypothetical-protein-[Brasilonema-bromeliae-SPC951]	385972
NMG21485.1	DndB-ParB-nuclease	GCA_012912135.1	391	Bacteria>Terrabacteria_group>Cyanobacteria/Melainabacteria_group>Cyanobacteria>Nostocales	Brasilonema_bromeliae_SPC951	hypothetical-protein-[Brasilonema-bromeliae-SPC951]	385972
WP_169266174.1	DndB-ParB-nuclease	GCF_012912125.1	532	Bacteria>Terrabacteria_group>Cyanobacteria/Melainabacteria_group>Cyanobacteria>Nostocales	Brasilonema_octagenarum	DGQHR-domain-containing-protein-[Brasilonema-octagenarum]	417105
MBW4629250.1	DndB-ParB-nuclease	GCA_019359625.1	532	Bacteria>Terrabacteria_group>Cyanobacteria/Melainabacteria_group>Cyanobacteria>Nostocales	Brasilonema_octagenarum_HA4186-MV1	DGQHR-domain-containing-protein-[Brasilonema-octagenarum-HA4186-MV1]	1049025
QDL18095.1	DndB-ParB-nuclease	GCA_006968915.1	532	Bacteria>Terrabacteria_group>Cyanobacteria/Melainabacteria_group>Cyanobacteria>Nostocales	Brasilonema_octagenarum_UFV-E1	hypothetical-protein-DP113_31020-[Brasilonema-octagenarum-UFV-E1]	417106
NMF64591.1	DndB-ParB-nuclease	GCA_012912125.1	532	Bacteria>Terrabacteria_group>Cyanobacteria/Melainabacteria_group>Cyanobacteria>Nostocales	Brasilonema_octagenarum_UFV-OR1	hypothetical-protein-[Brasilonema-octagenarum-UFV-OR1]	417115
QDL11715.1	DndB-ParB-nuclease	GCA_006968745.1	532	Bacteria>Terrabacteria_group>Cyanobacteria/Melainabacteria_group>Cyanobacteria>Nostocales	Brasilonema_sennae_CENA114	hypothetical-protein-DP114_30880-[Brasilonema-sennae-CENA114]	415709
WP_171977910.1	DndB-ParB-nuclease	GCF_006968745.1	532	Bacteria>Terrabacteria_group>Cyanobacteria/Melainabacteria_group>Cyanobacteria>Nostocales	Brasilonema_sennae	DGQHR-domain-containing-protein-[Brasilonema-sennae]	1397703
WP_210612110.1	DndB-ParB-nuclease	GCF_017982655.1	532	Bacteria>Terrabacteria_group>Cyanobacteria/Melainabacteria_group>Cyanobacteria>Nostocales	Brasilonema_sp._CT11	DGQHR-domain-containing-protein-[Brasilonema-sp.-CT11]	2748322
WP_169218966.1	DndB-ParB-nuclease	GCF_012912105.1	532	Bacteria>Terrabacteria_group>Cyanobacteria/Melainabacteria_group>Cyanobacteria>Nostocales	Brasilonema_sp._UFV-L1	DGQHR-domain-containing-protein-[Brasilonema-sp.-UFV-L1]	2234130
MBD2225563.1	DndB-ParB-nuclease	GCA_014696435.1	532	Bacteria>Terrabacteria_group>Cyanobacteria/Melainabacteria_group>Cyanobacteria>Nostocales	Calothrix_anomala_FACHB-343	DGQHR-domain-containing-protein-[Calothrix-anomala-FACHB-343]	2692894
WP_096645140.1	DndB-ParB-nuclease	GCF_002367995.1	532	Bacteria>Terrabacteria_group>Cyanobacteria/Melainabacteria_group>Cyanobacteria>Nostocales	Calothrix_brevissima	DGQHR-domain-containing-protein-[Calothrix-brevissima]	938406
BAY63999.1	DndB-ParB-nuclease	GCA_002367995.1	532	Bacteria>Terrabacteria_group>Cyanobacteria/Melainabacteria_group>Cyanobacteria>Nostocales	Calothrix_brevissima_NIES-22	hypothetical-protein-NIES22_40890-[Calothrix-brevissima-NIES-22]	1973478
TWH42727.1	DndB-ParB-nuclease	GCA_007830875.1	531	Bacteria>Terrabacteria_group>Cyanobacteria/Melainabacteria_group>Cyanobacteria>Nostocales	Calothrix_desertica_PCC_7102	DGQHR-domain-containing-protein-[Calothrix-desertica-PCC-7102]	232991
TWH42722.1	DndB-ParB-nuclease	GCA_007830875.1	446	Bacteria>Terrabacteria_group>Cyanobacteria/Melainabacteria_group>Cyanobacteria>Nostocales	Calothrix_desertica_PCC_7102	DNA-sulfur-modification-protein-DndB-[Calothrix-desertica-PCC-7102]	232991
RUT04714.1	DndB-ParB-nuclease	GCA_003991905.1	446	Bacteria>Terrabacteria_group>Cyanobacteria/Melainabacteria_group>Cyanobacteria>Nostocales	Calothrix_desertica_PCC_7102	hypothetical-protein-DSM106972_042830-[Calothrix-desertica-PCC-7102]	232991
RUT04719.1	DndB-ParB-nuclease	GCA_003991905.1	531	Bacteria>Terrabacteria_group>Cyanobacteria/Melainabacteria_group>Cyanobacteria>Nostocales	Calothrix_desertica_PCC_7102	hypothetical-protein-DSM106972_042880-[Calothrix-desertica-PCC-7102]	232991
PAX56185.1	DndB-ParB-nuclease	GCA_002289455.1	532	Bacteria>Terrabacteria_group>Cyanobacteria/Melainabacteria_group>Cyanobacteria>Nostocales	Calothrix_elsteri_CCALA_953	hypothetical-protein-CK510_10330-[Calothrix-elsteri-CCALA-953]	987040
WP_095721621.1	DndB-ParB-nuclease	GCF_002289455.1	532	Bacteria>Terrabacteria_group>Cyanobacteria/Melainabacteria_group>Cyanobacteria>Nostocales	Calothrix_elsteri	DGQHR-domain-containing-protein-[Calothrix-elsteri]	1247191
WP_190619543.1	DndB-ParB-nuclease	GCF_014696315.1	532	Bacteria>Terrabacteria_group>Cyanobacteria/Melainabacteria_group>Cyanobacteria>Nostocales	Calothrix_membranacea	DGQHR-domain-containing-protein-[Calothrix-membranacea]	212352
WP_190619542.1	DndB-ParB-nuclease	GCF_014696315.1	396	Bacteria>Terrabacteria_group>Cyanobacteria/Melainabacteria_group>Cyanobacteria>Nostocales	Calothrix_membranacea	DNA-sulfur-modification-protein-DndB-[Calothrix-membranacea]	212352
MBD2165263.1	DndB-ParB-nuclease	GCA_014696315.1	532	Bacteria>Terrabacteria_group>Cyanobacteria/Melainabacteria_group>Cyanobacteria>Nostocales	Calothrix_membranacea_FACHB-236	DGQHR-domain-containing-protein-[Calothrix-membranacea-FACHB-236]	2692895
MBD2165262.1	DndB-ParB-nuclease	GCA_014696315.1	396	Bacteria>Terrabacteria_group>Cyanobacteria/Melainabacteria_group>Cyanobacteria>Nostocales	Calothrix_membranacea_FACHB-236	DNA-sulfur-modification-protein-DndB-[Calothrix-membranacea-FACHB-236]	2692895
WP_190543205.1	DndB-ParB-nuclease	GCF_014696435.1	532	Bacteria>Terrabacteria_group>Cyanobacteria/Melainabacteria_group>Cyanobacteria>Nostocales	Calothrix	MULTISPECIES:-DGQHR-domain-containing-protein-[Calothrix]	1186
WP_096656562.1	DndB-ParB-nuclease	GCF_002368095.1	524	Bacteria>Terrabacteria_group>Cyanobacteria/Melainabacteria_group>Cyanobacteria>Nostocales	Calothrix_parasitica	DGQHR-domain-containing-protein-[Calothrix-parasitica]	1973486
BAY83614.1	DndB-ParB-nuclease	GCA_002368095.1	524	Bacteria>Terrabacteria_group>Cyanobacteria/Melainabacteria_group>Cyanobacteria>Nostocales	Calothrix_parasitica_NIES-267	hypothetical-protein-NIES267_31030-[Calothrix-parasitica-NIES-267]	1973488
MBD2197012.1	DndB-ParB-nuclease	GCA_014696555.1	532	Bacteria>Terrabacteria_group>Cyanobacteria/Melainabacteria_group>Cyanobacteria>Nostocales	Calothrix_parietina_FACHB-288	DGQHR-domain-containing-protein-[Calothrix-parietina-FACHB-288]	2692896
WP_088240539.1	DndB-ParB-nuclease	GCF_900185595.1	532	Bacteria>Terrabacteria_group>Cyanobacteria/Melainabacteria_group>Cyanobacteria>Nostocales	Calothrix_rhizosoleniae	DGQHR-domain-containing-protein-[Calothrix-rhizosoleniae]	888997
WP_088240540.1	DndB-ParB-nuclease	GCF_900185595.1	397	Bacteria>Terrabacteria_group>Cyanobacteria/Melainabacteria_group>Cyanobacteria>Nostocales	Calothrix_rhizosoleniae	DNA-sulfur-modification-protein-DndB-[Calothrix-rhizosoleniae]	888997
AKG22163.1	DndB-ParB-nuclease	GCA_000734895.2	391	Bacteria>Terrabacteria_group>Cyanobacteria/Melainabacteria_group>Cyanobacteria>Nostocales	Calothrix_sp._336/3	DGQHR-domain-containing-protein-[Calothrix-sp.-336/3]	1337936
AKG22164.1	DndB-ParB-nuclease	GCA_000734895.2	532	Bacteria>Terrabacteria_group>Cyanobacteria/Melainabacteria_group>Cyanobacteria>Nostocales	Calothrix_sp._336/3	DGQHR-domain-containing-protein-[Calothrix-sp.-336/3]	1337936
WP_035153866.1	DndB-ParB-nuclease	GCF_000734895.2	532	Bacteria>Terrabacteria_group>Cyanobacteria/Melainabacteria_group>Cyanobacteria>Nostocales	Calothrix_sp._336/3	DGQHR-domain-containing-protein-[Calothrix-sp.-336/3]	1337936
WP_035153865.1	DndB-ParB-nuclease	GCF_000734895.2	391	Bacteria>Terrabacteria_group>Cyanobacteria/Melainabacteria_group>Cyanobacteria>Nostocales	Calothrix_sp._336/3	DNA-sulfur-modification-protein-DndB-[Calothrix-sp.-336/3]	1337936
MBF2066569.1	DndB-ParB-nuclease	GCA_015272395.1	531	Bacteria>Terrabacteria_group>Cyanobacteria/Melainabacteria_group>Cyanobacteria>Nostocales	Calothrix_sp._C42_A2020_038	DGQHR-domain-containing-protein-[Calothrix-sp.-C42_A2020_038]	2774333
NJR16401.1	DndB-ParB-nuclease	GCA_012034595.1	532	Bacteria>Terrabacteria_group>Cyanobacteria/Melainabacteria_group>Cyanobacteria>Nostocales	Calothrix_sp._CSU_2_0	DGQHR-domain-containing-protein-[Calothrix-sp.-CSU_2_0]	2720413
MBD2220068.1	DndB-ParB-nuclease	GCA_014696565.1	532	Bacteria>Terrabacteria_group>Cyanobacteria/Melainabacteria_group>Cyanobacteria>Nostocales	Calothrix_sp._FACHB-1219	DGQHR-domain-containing-protein-[Calothrix-sp.-FACHB-1219]	2692778
MBD2220067.1	DndB-ParB-nuclease	GCA_014696565.1	396	Bacteria>Terrabacteria_group>Cyanobacteria/Melainabacteria_group>Cyanobacteria>Nostocales	Calothrix_sp._FACHB-1219	DNA-sulfur-modification-protein-DndB-[Calothrix-sp.-FACHB-1219]	2692778
MBD2339204.1	DndB-ParB-nuclease	GCA_014697055.1	532	Bacteria>Terrabacteria_group>Cyanobacteria/Melainabacteria_group>Cyanobacteria>Nostocales	Calothrix_sp._FACHB-156	DGQHR-domain-containing-protein-[Calothrix-sp.-FACHB-156]	2692779
WP_190584326.1	DndB-ParB-nuclease	GCF_014697055.1	532	Bacteria>Terrabacteria_group>Cyanobacteria/Melainabacteria_group>Cyanobacteria>Nostocales	Calothrix_sp._FACHB-156	DGQHR-domain-containing-protein-[Calothrix-sp.-FACHB-156]	2692779
MBD2339205.1	DndB-ParB-nuclease	GCA_014697055.1	390	Bacteria>Terrabacteria_group>Cyanobacteria/Melainabacteria_group>Cyanobacteria>Nostocales	Calothrix_sp._FACHB-156	DNA-sulfur-modification-protein-DndB-[Calothrix-sp.-FACHB-156]	2692779
WP_190584327.1	DndB-ParB-nuclease	GCF_014697055.1	390	Bacteria>Terrabacteria_group>Cyanobacteria/Melainabacteria_group>Cyanobacteria>Nostocales	Calothrix_sp._FACHB-156	DNA-sulfur-modification-protein-DndB-[Calothrix-sp.-FACHB-156]	2692779
MBD2205295.1	DndB-ParB-nuclease	GCA_014696495.1	532	Bacteria>Terrabacteria_group>Cyanobacteria/Melainabacteria_group>Cyanobacteria>Nostocales	Calothrix_sp._FACHB-168	DGQHR-domain-containing-protein-[Calothrix-sp.-FACHB-168]	2692780
MBD2205294.1	DndB-ParB-nuclease	GCA_014696495.1	396	Bacteria>Terrabacteria_group>Cyanobacteria/Melainabacteria_group>Cyanobacteria>Nostocales	Calothrix_sp._FACHB-168	DNA-sulfur-modification-protein-DndB-[Calothrix-sp.-FACHB-168]	2692780
MBW4602001.1	DndB-ParB-nuclease	GCA_019359675.1	446	Bacteria>Terrabacteria_group>Cyanobacteria/Melainabacteria_group>Cyanobacteria>Nostocales	Calothrix_sp._FI2-JRJ7	DGQHR-domain-containing-protein-[Calothrix-sp.-FI2-JRJ7]	2839656
MBW4602007.1	DndB-ParB-nuclease	GCA_019359675.1	531	Bacteria>Terrabacteria_group>Cyanobacteria/Melainabacteria_group>Cyanobacteria>Nostocales	Calothrix_sp._FI2-JRJ7	DGQHR-domain-containing-protein-[Calothrix-sp.-FI2-JRJ7]	2839656
WP_073618850.1	DndB-ParB-nuclease	GCF_001904745.1	447	Bacteria>Terrabacteria_group>Cyanobacteria/Melainabacteria_group>Cyanobacteria>Nostocales	Calothrix_sp._HK-06	DGQHR-domain-containing-protein-[Calothrix-sp.-HK-06]	1137096
WP_143175925.1	DndB-ParB-nuclease	GCF_001904745.1	531	Bacteria>Terrabacteria_group>Cyanobacteria/Melainabacteria_group>Cyanobacteria>Nostocales	Calothrix_sp._HK-06	DGQHR-domain-containing-protein-[Calothrix-sp.-HK-06]	1137096
WP_096588265.1	DndB-ParB-nuclease	GCF_002368175.1	532	Bacteria>Terrabacteria_group>Cyanobacteria/Melainabacteria_group>Cyanobacteria>Nostocales	Calothrix_sp._NIES-2098	DGQHR-domain-containing-protein-[Calothrix-sp.-NIES-2098]	1954171
BAY08725.1	DndB-ParB-nuclease	GCA_002368175.1	532	Bacteria>Terrabacteria_group>Cyanobacteria/Melainabacteria_group>Cyanobacteria>Nostocales	Calothrix_sp._NIES-2098	hypothetical-protein-NIES2098_18850-[Calothrix-sp.-NIES-2098]	1954171
WP_096597682.1	DndB-ParB-nuclease	GCF_002368195.1	532	Bacteria>Terrabacteria_group>Cyanobacteria/Melainabacteria_group>Cyanobacteria>Nostocales	Calothrix_sp._NIES-2100	DGQHR-domain-containing-protein-[Calothrix-sp.-NIES-2100]	1954172
BAY20831.1	DndB-ParB-nuclease	GCA_002368195.1	532	Bacteria>Terrabacteria_group>Cyanobacteria/Melainabacteria_group>Cyanobacteria>Nostocales	Calothrix_sp._NIES-2100	hypothetical-protein-NIES2100_05750-[Calothrix-sp.-NIES-2100]	1954172
WP_231943065.1	DndB-ParB-nuclease	GCF_002368395.1	515	Bacteria>Terrabacteria_group>Cyanobacteria/Melainabacteria_group>Cyanobacteria>Nostocales	Calothrix_sp._NIES-3974	DGQHR-domain-containing-protein-[Calothrix-sp.-NIES-3974]	2005462
BAZ09872.1	DndB-ParB-nuclease	GCA_002368455.1	593	Bacteria>Terrabacteria_group>Cyanobacteria/Melainabacteria_group>Cyanobacteria>Nostocales	Calothrix_sp._NIES-4071	hypothetical-protein-NIES4071_16860-[Calothrix-sp.-NIES-4071]	2005469
BAZ41311.1	DndB-ParB-nuclease	GCA_004296455.1	530	Bacteria>Terrabacteria_group>Cyanobacteria/Melainabacteria_group>Cyanobacteria>Nostocales	Calothrix_sp._NIES-4101	hypothetical-protein-NIES4101_72750-[Calothrix-sp.-NIES-4101]	2005461
BAZ56019.1	DndB-ParB-nuclease	GCA_002368415.1	593	Bacteria>Terrabacteria_group>Cyanobacteria/Melainabacteria_group>Cyanobacteria>Nostocales	Calothrix_sp._NIES-4105	hypothetical-protein-NIES4105_16810-[Calothrix-sp.-NIES-4105]	2005463
WP_015200485.1	DndB-ParB-nuclease	GCF_000317435.1	536	Bacteria>Terrabacteria_group>Cyanobacteria/Melainabacteria_group>Cyanobacteria>Nostocales	Calothrix_sp._PCC_6303	DGQHR-domain-containing-protein-[Calothrix-sp.-PCC-6303]	1170562
AFZ03874.1	DndB-ParB-nuclease	GCA_000317435.1	536	Bacteria>Terrabacteria_group>Cyanobacteria/Melainabacteria_group>Cyanobacteria>Nostocales	Calothrix_sp._PCC_6303	DGQHR-domain-protein-[Calothrix-sp.-PCC-6303]	1170562
WP_019489235.1	DndB-ParB-nuclease	GCF_000331305.1	531	Bacteria>Terrabacteria_group>Cyanobacteria/Melainabacteria_group>Cyanobacteria>Nostocales	Calothrix_sp._PCC_7103	DGQHR-domain-containing-protein-[Calothrix-sp.-PCC-7103]	32057
WP_019489240.1	DndB-ParB-nuclease	GCF_000331305.1	303	Bacteria>Terrabacteria_group>Cyanobacteria/Melainabacteria_group>Cyanobacteria>Nostocales	Calothrix_sp._PCC_7103	DGQHR-domain-containing-protein-[Calothrix-sp.-PCC-7103]	32057
WP_019489234.1	DndB-ParB-nuclease	GCF_000331305.1	387	Bacteria>Terrabacteria_group>Cyanobacteria/Melainabacteria_group>Cyanobacteria>Nostocales	Calothrix_sp._PCC_7103	DNA-sulfur-modification-protein-DndB-[Calothrix-sp.-PCC-7103]	32057
WP_015130699.1	DndB-ParB-nuclease	GCF_000316575.1	532	Bacteria>Terrabacteria_group>Cyanobacteria/Melainabacteria_group>Cyanobacteria>Nostocales	Calothrix_sp._PCC_7507	DGQHR-domain-containing-protein-[Calothrix-sp.-PCC-7507]	99598
AFY34900.1	DndB-ParB-nuclease	GCA_000316575.1	396	Bacteria>Terrabacteria_group>Cyanobacteria/Melainabacteria_group>Cyanobacteria>Nostocales	Calothrix_sp._PCC_7507	DGQHR-domain-protein-[Calothrix-sp.-PCC-7507]	99598
AFY34901.1	DndB-ParB-nuclease	GCA_000316575.1	532	Bacteria>Terrabacteria_group>Cyanobacteria/Melainabacteria_group>Cyanobacteria>Nostocales	Calothrix_sp._PCC_7507	DGQHR-domain-protein-[Calothrix-sp.-PCC-7507]	99598
WP_015130698.1	DndB-ParB-nuclease	GCF_000316575.1	396	Bacteria>Terrabacteria_group>Cyanobacteria/Melainabacteria_group>Cyanobacteria>Nostocales	Calothrix_sp._PCC_7507	DNA-sulfur-modification-protein-DndB-[Calothrix-sp.-PCC-7507]	99598
WP_224175158.1	DndB-ParB-nuclease	GCF_019977735.1	531	Bacteria>Terrabacteria_group>Cyanobacteria/Melainabacteria_group>Cyanobacteria>Nostocales	Calothrix_sp._PCC_7716	DGQHR-domain-containing-protein-[Calothrix-sp.-PCC-7716]	2853227
WP_224175164.1	DndB-ParB-nuclease	GCF_019977735.1	447	Bacteria>Terrabacteria_group>Cyanobacteria/Melainabacteria_group>Cyanobacteria>Nostocales	Calothrix_sp._PCC_7716	DGQHR-domain-containing-protein-[Calothrix-sp.-PCC-7716]	2853227
BDA72811.1	DndB-ParB-nuclease	GCA_019977735.1	593	Bacteria>Terrabacteria_group>Cyanobacteria/Melainabacteria_group>Cyanobacteria>Nostocales	Calothrix_sp._PCC_7716	hypothetical-protein-CAL7716_069770-[Calothrix-sp.-PCC-7716]	2853227
MBF2005940.1	DndB-ParB-nuclease	GCA_015272425.1	532	Bacteria>Terrabacteria_group>Cyanobacteria/Melainabacteria_group>Cyanobacteria>Nostocales	Chlorogloeopsis_fritschii_C42_A2020_084	DGQHR-domain-containing-protein-[Chlorogloeopsis-fritschii-C42_A2020_084]	2774336
WP_016872838.1	DndB-ParB-nuclease	GCF_000317265.1	532	Bacteria>Terrabacteria_group>Cyanobacteria/Melainabacteria_group>Cyanobacteria>Nostocales	Chlorogloeopsis_fritschii	DGQHR-domain-containing-protein-[Chlorogloeopsis-fritschii]	1124
CEJ43511.1	DndB-ParB-nuclease	GCA_001458455.1	532	Bacteria>Terrabacteria_group>Cyanobacteria/Melainabacteria_group>Cyanobacteria>Nostocales	Chrysosporum_ovalisporum	Uncharacterized-protein-apha_00869-[Chrysosporum-ovalisporum]	75695
WP_104387761.1	DndB-ParB-nuclease	GCF_002934005.1	531	Bacteria>Terrabacteria_group>Cyanobacteria/Melainabacteria_group>Cyanobacteria>Nostocales	Cuspidothrix_issatschenkoi	DGQHR-domain-containing-protein-[Cuspidothrix-issatschenkoi]	230752
WP_193935811.1	DndB-ParB-nuclease	GCF_015207795.1	531	Bacteria>Terrabacteria_group>Cyanobacteria/Melainabacteria_group>Cyanobacteria>Nostocales	Cuspidothrix_issatschenkoi	DGQHR-domain-containing-protein-[Cuspidothrix-issatschenkoi]	230752
MBW4670348.1	DndB-ParB-nuclease	GCA_019359595.1	531	Bacteria>Terrabacteria_group>Cyanobacteria/Melainabacteria_group>Cyanobacteria>Nostocales	Cyanomargarita_calcarea_GSE-NOS-MK-12-04C	DGQHR-domain-containing-protein-[Cyanomargarita-calcarea-GSE-NOS-MK-12-04C]	2839659
MBW4670347.1	DndB-ParB-nuclease	GCA_019359595.1	396	Bacteria>Terrabacteria_group>Cyanobacteria/Melainabacteria_group>Cyanobacteria>Nostocales	Cyanomargarita_calcarea_GSE-NOS-MK-12-04C	DNA-sulfur-modification-protein-DndB-[Cyanomargarita-calcarea-GSE-NOS-MK-12-04C]	2839659
WP_096545327.1	DndB-ParB-nuclease	GCF_014489415.1	519	Bacteria>Terrabacteria_group>Cyanobacteria/Melainabacteria_group>Cyanobacteria>Nostocales	Cylindrospermopsis_curvispora	MULTISPECIES:-DGQHR-domain-containing-protein-[Aphanizomenonaceae]	747548
MBA4455665.1	DndB-ParB-nuclease	GCA_013867425.1	517	Bacteria>Terrabacteria_group>Cyanobacteria/Melainabacteria_group>Cyanobacteria>Nostocales	Cylindrospermopsis_raciborskii_CS-506_B	DGQHR-domain-containing-protein-[Cylindrospermopsis-raciborskii-CS-506_B]	2585139
WP_006275786.1	DndB-ParB-nuclease	GCF_000175835.1	517	Bacteria>Terrabacteria_group>Cyanobacteria/Melainabacteria_group>Cyanobacteria>Nostocales	Cylindrospermopsis_raciborskii	DGQHR-domain-containing-protein-[Cylindrospermopsis-raciborskii]	77022
WP_061545029.1	DndB-ParB-nuclease	GCF_001586755.1	517	Bacteria>Terrabacteria_group>Cyanobacteria/Melainabacteria_group>Cyanobacteria>Nostocales	Cylindrospermopsis_raciborskii	DGQHR-domain-containing-protein-[Cylindrospermopsis-raciborskii]	77022
WP_071241852.1	DndB-ParB-nuclease	GCF_001858115.1	517	Bacteria>Terrabacteria_group>Cyanobacteria/Melainabacteria_group>Cyanobacteria>Nostocales	Cylindrospermopsis_raciborskii	DGQHR-domain-containing-protein-[Cylindrospermopsis-raciborskii]	77022
WP_071250240.1	DndB-ParB-nuclease	GCF_001858125.1	517	Bacteria>Terrabacteria_group>Cyanobacteria/Melainabacteria_group>Cyanobacteria>Nostocales	Cylindrospermopsis_raciborskii	DGQHR-domain-containing-protein-[Cylindrospermopsis-raciborskii]	77022
WP_085728472.1	DndB-ParB-nuclease	GCF_002114155.1	517	Bacteria>Terrabacteria_group>Cyanobacteria/Melainabacteria_group>Cyanobacteria>Nostocales	Cylindrospermopsis_raciborskii	DGQHR-domain-containing-protein-[Cylindrospermopsis-raciborskii]	77022
WP_115538317.1	DndB-ParB-nuclease	GCF_003367075.2	517	Bacteria>Terrabacteria_group>Cyanobacteria/Melainabacteria_group>Cyanobacteria>Nostocales	Cylindrospermopsis_raciborskii	DGQHR-domain-containing-protein-[Cylindrospermopsis-raciborskii]	77022
MBG0743223.1	DndB-ParB-nuclease	GCA_015708265.1	517	Bacteria>Terrabacteria_group>Cyanobacteria/Melainabacteria_group>Cyanobacteria>Nostocales	Cylindrospermopsis_raciborskii_KL1	DGQHR-domain-containing-protein-[Cylindrospermopsis-raciborskii-KL1]	2787621
WP_057177280.1	DndB-ParB-nuclease	GCF_001432185.1	517	Bacteria>Terrabacteria_group>Cyanobacteria/Melainabacteria_group>Cyanobacteria>Nostocales	Cylindrospermopsis_sp._CR12	DGQHR-domain-containing-protein-[Cylindrospermopsis-sp.-CR12]	1747196
MBD2387764.1	DndB-ParB-nuclease	GCA_014697195.1	532	Bacteria>Terrabacteria_group>Cyanobacteria/Melainabacteria_group>Cyanobacteria>Nostocales	Cylindrospermum_sp._FACHB-282	DGQHR-domain-containing-protein-[Cylindrospermum-sp.-FACHB-282]	2692794
WP_190461572.1	DndB-ParB-nuclease	GCF_014697195.1	532	Bacteria>Terrabacteria_group>Cyanobacteria/Melainabacteria_group>Cyanobacteria>Nostocales	Cylindrospermum_sp._FACHB-282	DGQHR-domain-containing-protein-[Cylindrospermum-sp.-FACHB-282]	2692794
BAZ29469.1	DndB-ParB-nuclease	GCA_003994795.1	408	Bacteria>Terrabacteria_group>Cyanobacteria/Melainabacteria_group>Cyanobacteria>Nostocales	Cylindrospermum_sp._NIES-4074	hypothetical-protein-NIES4074_19160-[Cylindrospermum-sp.-NIES-4074]	2005457
BAZ29470.1	DndB-ParB-nuclease	GCA_003994795.1	533	Bacteria>Terrabacteria_group>Cyanobacteria/Melainabacteria_group>Cyanobacteria>Nostocales	Cylindrospermum_sp._NIES-4074	hypothetical-protein-NIES4074_19170-[Cylindrospermum-sp.-NIES-4074]	2005457
WP_015206898.1	DndB-ParB-nuclease	GCF_000317535.1	533	Bacteria>Terrabacteria_group>Cyanobacteria/Melainabacteria_group>Cyanobacteria>Nostocales	Cylindrospermum_stagnale	DGQHR-domain-containing-protein-[Cylindrospermum-stagnale]	142864
WP_015206897.1	DndB-ParB-nuclease	GCF_000317535.1	396	Bacteria>Terrabacteria_group>Cyanobacteria/Melainabacteria_group>Cyanobacteria>Nostocales	Cylindrospermum_stagnale	DNA-sulfur-modification-protein-DndB-[Cylindrospermum-stagnale]	142864
AFZ23641.1	DndB-ParB-nuclease	GCA_000317535.1	396	Bacteria>Terrabacteria_group>Cyanobacteria/Melainabacteria_group>Cyanobacteria>Nostocales	Cylindrospermum_stagnale_PCC_7417	DGQHR-domain-containing-protein-[Cylindrospermum-stagnale-PCC-7417]	56107
AFZ23642.1	DndB-ParB-nuclease	GCA_000317535.1	533	Bacteria>Terrabacteria_group>Cyanobacteria/Melainabacteria_group>Cyanobacteria>Nostocales	Cylindrospermum_stagnale_PCC_7417	DGQHR-domain-containing-protein-[Cylindrospermum-stagnale-PCC-7417]	56107
MBH8572198.1	DndB-ParB-nuclease	GCA_016056275.1	532	Bacteria>Terrabacteria_group>Cyanobacteria/Melainabacteria_group>Cyanobacteria>Nostocales	Dendronalium_phyllosphericum_CENA369	DGQHR-domain-containing-protein-[Dendronalium-phyllosphericum-CENA369]	1725256
MBH8572199.1	DndB-ParB-nuclease	GCA_016056275.1	396	Bacteria>Terrabacteria_group>Cyanobacteria/Melainabacteria_group>Cyanobacteria>Nostocales	Dendronalium_phyllosphericum_CENA369	DNA-sulfur-modification-protein-DndB-[Dendronalium-phyllosphericum-CENA369]	1725256
WP_214431027.1	DndB-ParB-nuclease	GCF_016056275.1	532	Bacteria>Terrabacteria_group>Cyanobacteria/Melainabacteria_group>Cyanobacteria>Nostocales	Dendronalium_phyllosphericum	DGQHR-domain-containing-protein-[Dendronalium-phyllosphericum]	2840445
WP_214431028.1	DndB-ParB-nuclease	GCF_016056275.1	396	Bacteria>Terrabacteria_group>Cyanobacteria/Melainabacteria_group>Cyanobacteria>Nostocales	Dendronalium_phyllosphericum	DGQHR-domain-containing-protein-[Dendronalium-phyllosphericum]	2840445
MBW4675535.1	DndB-ParB-nuclease	GCA_019359545.1	532	Bacteria>Terrabacteria_group>Cyanobacteria/Melainabacteria_group>Cyanobacteria>Nostocales	Desmonostoc_geniculatum_HA4340-LM1	DGQHR-domain-containing-protein-[Desmonostoc-geniculatum-HA4340-LM1]	1874567
MBX9253357.1	DndB-ParB-nuclease	GCA_019734875.1	532	Bacteria>Terrabacteria_group>Cyanobacteria/Melainabacteria_group>Cyanobacteria>Nostocales	Desmonostoc_muscorum_CCALA_125	DGQHR-domain-containing-protein-[Desmonostoc-muscorum-CCALA-125]	2756293
WP_190961939.1	DndB-ParB-nuclease	GCF_014698035.1	532	Bacteria>Terrabacteria_group>Cyanobacteria/Melainabacteria_group>Cyanobacteria>Nostocales	Desmonostoc_muscorum	DGQHR-domain-containing-protein-[Desmonostoc-muscorum]	1179

Accession	Annotation	GCA	Length	Phyletic.spread	Strain.name	Defline	Species.Taxid
WP_193918348.1	DndB-ParB-nuclease	GCF_015207005.2	533	Bacteria>Terrabacteria_group>Cyanobacteria/Melainabacteria_group>Cyanobacteria>Nostocales	Desmonostoc_muscorum	DGQHR-domain-containing-protein-[Desmonostoc-muscorum]	1179
WP_221705036.1	DndB-ParB-nuclease	GCF_019734875.1	532	Bacteria>Terrabacteria_group>Cyanobacteria/Melainabacteria_group>Cyanobacteria>Nostocales	Desmonostoc_muscorum	DGQHR-domain-containing-protein-[Desmonostoc-muscorum]	1179
WP_228043161.1	DndB-ParB-nuclease	GCF_015207005.2	396	Bacteria>Terrabacteria_group>Cyanobacteria/Melainabacteria_group>Cyanobacteria>Nostocales	Desmonostoc_muscorum	DNA-sulfur-modification-protein-DndB-[Desmonostoc-muscorum]	1179
MBD2508123.1	DndB-ParB-nuclease	GCA_014698035.1	532	Bacteria>Terrabacteria_group>Cyanobacteria/Melainabacteria_group>Cyanobacteria>Nostocales	Desmonostoc_muscorum_FACHB-395	DGQHR-domain-containing-protein-[Desmonostoc-muscorum-FACHB-395]	2692907
MBW4616831.1	DndB-ParB-nuclease	GCA_019359515.1	532	Bacteria>Terrabacteria_group>Cyanobacteria/Melainabacteria_group>Cyanobacteria>Nostocales	Desmonostoc_vinosum_HA7617-LM4	DGQHR-domain-containing-protein-[Desmonostoc-vinosum-HA7617-LM4]	1382956
MBW4616833.1	DndB-ParB-nuclease	GCA_019359515.1	664	Bacteria>Terrabacteria_group>Cyanobacteria/Melainabacteria_group>Cyanobacteria>Nostocales	Desmonostoc_vinosum_HA7617-LM4	hypothetical-protein-[Desmonostoc-vinosum-HA7617-LM4]	1382956
MBD1214622.1	DndB-ParB-nuclease	GCA_014654715.1	532	Bacteria>Terrabacteria_group>Cyanobacteria/Melainabacteria_group>Cyanobacteria>Nostocales	Dolichospermum_circinale_Clear-D4	DGQHR-domain-containing-protein-[Dolichospermum-circinale-Clear-D4]	2766971
MBD1214623.1	DndB-ParB-nuclease	GCA_014654715.1	661	Bacteria>Terrabacteria_group>Cyanobacteria/Melainabacteria_group>Cyanobacteria>Nostocales	Dolichospermum_circinale_Clear-D4	hypothetical-protein-[Dolichospermum-circinale-Clear-D4]	2766971
WP_028085158.1	DndB-ParB-nuclease	GCF_000426925.1	533	Bacteria>Terrabacteria_group>Cyanobacteria/Melainabacteria_group>Cyanobacteria>Nostocales	Dolichospermum_circinale	DGQHR-domain-containing-protein-[Dolichospermum-circinale]	109265
WP_028091677.1	DndB-ParB-nuclease	GCF_000426905.1	533	Bacteria>Terrabacteria_group>Cyanobacteria/Melainabacteria_group>Cyanobacteria>Nostocales	Dolichospermum_circinale	DGQHR-domain-containing-protein-[Dolichospermum-circinale]	109265
WP_028085157.1	DndB-ParB-nuclease	GCF_000426905.1	401	Bacteria>Terrabacteria_group>Cyanobacteria/Melainabacteria_group>Cyanobacteria>Nostocales	Dolichospermum_circinale	DNA-sulfur-modification-protein-DndB-[Dolichospermum-circinale]	109265
WP_096668305.1	DndB-ParB-nuclease	GCF_002368115.1	532	Bacteria>Terrabacteria_group>Cyanobacteria/Melainabacteria_group>Cyanobacteria>Nostocales	Dolichospermum_compactum	DGQHR-domain-containing-protein-[Dolichospermum-compactum]	136073
BAZ86697.1	DndB-ParB-nuclease	GCA_002368115.1	532	Bacteria>Terrabacteria_group>Cyanobacteria/Melainabacteria_group>Cyanobacteria>Nostocales	Dolichospermum_compactum_NIES-806	hypothetical-protein-NIES806_29130-[Dolichospermum-compactum-NIES-806]	1973481
WP_168635573.1	DndB-ParB-nuclease	GCF_009712125.1	533	Bacteria>Terrabacteria_group>Cyanobacteria/Melainabacteria_group>Cyanobacteria>Nostocales	Dolichospermum_flos-aquae	DGQHR-domain-containing-protein-[Dolichospermum-flos-aquae]	1166
WP_168696058.1	DndB-ParB-nuclease	GCF_012516395.1	532	Bacteria>Terrabacteria_group>Cyanobacteria/Melainabacteria_group>Cyanobacteria>Nostocales	Dolichospermum_flos-aquae	DGQHR-domain-containing-protein-[Dolichospermum-flos-aquae]	1166
WP_168635572.1	DndB-ParB-nuclease	GCF_009712125.1	396	Bacteria>Terrabacteria_group>Cyanobacteria/Melainabacteria_group>Cyanobacteria>Nostocales	Dolichospermum_flos-aquae	DNA-sulfur-modification-protein-DndB-[Dolichospermum-flos-aquae]	1166
WP_193848895.1	DndB-ParB-nuclease	GCF_015207785.1	403	Bacteria>Terrabacteria_group>Cyanobacteria/Melainabacteria_group>Cyanobacteria>Nostocales	Dolichospermum_flos-aquae	DNA-sulfur-modification-protein-DndB-[Dolichospermum-flos-aquae]	1166
MBE9219464.1	DndB-ParB-nuclease	GCA_015207785.1	533	Bacteria>Terrabacteria_group>Cyanobacteria/Melainabacteria_group>Cyanobacteria>Nostocales	Dolichospermum_flos-aquae_LEGE_04289	DGQHR-domain-containing-protein-[Dolichospermum-flos-aquae-LEGE-04289]	1828708
MBE9219465.1	DndB-ParB-nuclease	GCA_015207785.1	403	Bacteria>Terrabacteria_group>Cyanobacteria/Melainabacteria_group>Cyanobacteria>Nostocales	Dolichospermum_flos-aquae_LEGE_04289	DNA-sulfur-modification-protein-DndB-[Dolichospermum-flos-aquae-LEGE-04289]	1828708
WP_137907217.1	DndB-ParB-nuclease	GCF_005402965.1	533	Bacteria>Terrabacteria_group>Cyanobacteria/Melainabacteria_group>Cyanobacteria>Nostocales	Dolichospermum	MULTISPECIES:-DGQHR-domain-containing-protein-[Dolichospermum]	748770
WP_168358180.1	DndB-ParB-nuclease	GCF_009712075.1	534	Bacteria>Terrabacteria_group>Cyanobacteria/Melainabacteria_group>Cyanobacteria>Nostocales	Dolichospermum_planctonicum	DGQHR-domain-containing-protein-[Dolichospermum-planctonicum]	136072
WP_137907216.1	DndB-ParB-nuclease	GCF_005402965.1	401	Bacteria>Terrabacteria_group>Cyanobacteria/Melainabacteria_group>Cyanobacteria>Nostocales	Dolichospermum_planctonicum	DNA-sulfur-modification-protein-DndB-[Dolichospermum-planctonicum]	136072
GCL41495.1	DndB-ParB-nuclease	GCA_005402965.1	401	Bacteria>Terrabacteria_group>Cyanobacteria/Melainabacteria_group>Cyanobacteria>Nostocales	Dolichospermum_planctonicum	hypothetical-protein-NIES80_11900-[Dolichospermum-planctonicum]	136072
GCL41496.1	DndB-ParB-nuclease	GCA_005402965.1	533	Bacteria>Terrabacteria_group>Cyanobacteria/Melainabacteria_group>Cyanobacteria>Nostocales	Dolichospermum_planctonicum	hypothetical-protein-NIES80_11910-[Dolichospermum-planctonicum]	136072
MBS9383817.1	DndB-ParB-nuclease	GCA_018447705.1	533	Bacteria>Terrabacteria_group>Cyanobacteria/Melainabacteria_group>Cyanobacteria>Nostocales	Dolichospermum_sp._BR01	DGQHR-domain-containing-protein-[Dolichospermum-sp.-BR01]	2717868
MBS9383816.1	DndB-ParB-nuclease	GCA_018447705.1	391	Bacteria>Terrabacteria_group>Cyanobacteria/Melainabacteria_group>Cyanobacteria>Nostocales	Dolichospermum_sp._BR01	DNA-sulfur-modification-protein-DndB-[Dolichospermum-sp.-BR01]	2717868
MBS3038897.1	DndB-ParB-nuclease	GCA_018295975.1	533	Bacteria>Terrabacteria_group>Cyanobacteria/Melainabacteria_group>Cyanobacteria>Nostocales	Dolichospermum_sp._DET50	DGQHR-domain-containing-protein-[Dolichospermum-sp.-DET50]	2704414
MBS3038898.1	DndB-ParB-nuclease	GCA_018295975.1	391	Bacteria>Terrabacteria_group>Cyanobacteria/Melainabacteria_group>Cyanobacteria>Nostocales	Dolichospermum_sp._DET50	DNA-sulfur-modification-protein-DndB-[Dolichospermum-sp.-DET50]	2704414
MBS3028494.1	DndB-ParB-nuclease	GCA_018295985.1	533	Bacteria>Terrabacteria_group>Cyanobacteria/Melainabacteria_group>Cyanobacteria>Nostocales	Dolichospermum_sp._DET66	DGQHR-domain-containing-protein-[Dolichospermum-sp.-DET66]	2704415
MBS3028495.1	DndB-ParB-nuclease	GCA_018295985.1	391	Bacteria>Terrabacteria_group>Cyanobacteria/Melainabacteria_group>Cyanobacteria>Nostocales	Dolichospermum_sp._DET66	DNA-sulfur-modification-protein-DndB-[Dolichospermum-sp.-DET66]	2704415
MBS3033694.1	DndB-ParB-nuclease	GCA_018295965.1	533	Bacteria>Terrabacteria_group>Cyanobacteria/Melainabacteria_group>Cyanobacteria>Nostocales	Dolichospermum_sp._DET67	DGQHR-domain-containing-protein-[Dolichospermum-sp.-DET67]	2704416
MBS3033695.1	DndB-ParB-nuclease	GCA_018295965.1	391	Bacteria>Terrabacteria_group>Cyanobacteria/Melainabacteria_group>Cyanobacteria>Nostocales	Dolichospermum_sp._DET67	DNA-sulfur-modification-protein-DndB-[Dolichospermum-sp.-DET67]	2704416
QSX66155.1	DndB-ParB-nuclease	GCA_017355425.1	533	Bacteria>Terrabacteria_group>Cyanobacteria/Melainabacteria_group>Cyanobacteria>Nostocales	Dolichospermum_sp._DET69	DGQHR-domain-containing-protein-[Dolichospermum-sp.-DET69]	2529119
QSX66156.1	DndB-ParB-nuclease	GCA_017355425.1	391	Bacteria>Terrabacteria_group>Cyanobacteria/Melainabacteria_group>Cyanobacteria>Nostocales	Dolichospermum_sp._DET69	DNA-sulfur-modification-protein-DndB-[Dolichospermum-sp.-DET69]	2529119
MBO1051188.1	DndB-ParB-nuclease	GCA_017355625.1	532	Bacteria>Terrabacteria_group>Cyanobacteria/Melainabacteria_group>Cyanobacteria>Nostocales	Dolichospermum_sp._DET73	DGQHR-domain-containing-protein-[Dolichospermum-sp.-DET73]	2717879
MBO1047880.1	DndB-ParB-nuclease	GCA_017355645.1	391	Bacteria>Terrabacteria_group>Cyanobacteria/Melainabacteria_group>Cyanobacteria>Nostocales	Dolichospermum_sp._DEX182a	DGQHR-domain-containing-protein-[Dolichospermum-sp.-DEX182a]	2717869
MBO1047881.1	DndB-ParB-nuclease	GCA_017355645.1	533	Bacteria>Terrabacteria_group>Cyanobacteria/Melainabacteria_group>Cyanobacteria>Nostocales	Dolichospermum_sp._DEX182a	DGQHR-domain-containing-protein-[Dolichospermum-sp.-DEX182a]	2717869
MBO1069364.1	DndB-ParB-nuclease	GCA_017355655.1	532	Bacteria>Terrabacteria_group>Cyanobacteria/Melainabacteria_group>Cyanobacteria>Nostocales	Dolichospermum_sp._DEX189	DGQHR-domain-containing-protein-[Dolichospermum-sp.-DEX189]	2717870
MBJ7297758.1	DndB-ParB-nuclease	GCA_016462165.1	533	Bacteria>Terrabacteria_group>Cyanobacteria/Melainabacteria_group>Cyanobacteria>Nostocales	Dolichospermum_sp.	DGQHR-domain-containing-protein-[Dolichospermum-sp.]	2720786
QSV64816.1	DndB-ParB-nuclease	GCA_017346835.1	532	Bacteria>Terrabacteria_group>Cyanobacteria/Melainabacteria_group>Cyanobacteria>Nostocales	Dolichospermum_sp._DL01	DGQHR-domain-containing-protein-[Dolichospermum-sp.-DL01]	2717871
MBJ7297757.1	DndB-ParB-nuclease	GCA_016462165.1	401	Bacteria>Terrabacteria_group>Cyanobacteria/Melainabacteria_group>Cyanobacteria>Nostocales	Dolichospermum_sp.	DNA-sulfur-modification-protein-DndB-[Dolichospermum-sp.]	2720786
WP_190393665.1	DndB-ParB-nuclease	GCF_014697585.1	532	Bacteria>Terrabacteria_group>Cyanobacteria/Melainabacteria_group>Cyanobacteria>Nostocales	Dolichospermum_sp._FACHB-1091	DGQHR-domain-containing-protein-[Dolichospermum-sp.-FACHB-1091]	2692798
WP_193890831.1	DndB-ParB-nuclease	GCF_015207875.1	532	Bacteria>Terrabacteria_group>Cyanobacteria/Melainabacteria_group>Cyanobacteria>Nostocales	Dolichospermum_sp._LEGE_00240	DGQHR-domain-containing-protein-[Dolichospermum-sp.-LEGE-00240]	1828603
MBE9257155.1	DndB-ParB-nuclease	GCA_015207865.1	533	Bacteria>Terrabacteria_group>Cyanobacteria/Melainabacteria_group>Cyanobacteria>Nostocales	Dolichospermum_sp._LEGE_00246	DGQHR-domain-containing-protein-[Dolichospermum-sp.-LEGE-00246]	1828605
WP_193962525.1	DndB-ParB-nuclease	GCF_015207865.1	533	Bacteria>Terrabacteria_group>Cyanobacteria/Melainabacteria_group>Cyanobacteria>Nostocales	Dolichospermum_sp._LEGE_00246	DGQHR-domain-containing-protein-[Dolichospermum-sp.-LEGE-00246]	1828605
MBE9257156.1	DndB-ParB-nuclease	GCA_015207865.1	401	Bacteria>Terrabacteria_group>Cyanobacteria/Melainabacteria_group>Cyanobacteria>Nostocales	Dolichospermum_sp._LEGE_00246	DNA-sulfur-modification-protein-DndB-[Dolichospermum-sp.-LEGE-00246]	1828605
WP_193962526.1	DndB-ParB-nuclease	GCF_015207865.1	401	Bacteria>Terrabacteria_group>Cyanobacteria/Melainabacteria_group>Cyanobacteria>Nostocales	Dolichospermum_sp._LEGE_00246	DNA-sulfur-modification-protein-DndB-[Dolichospermum-sp.-LEGE-00246]	1828605
MBS9393285.1	DndB-ParB-nuclease	GCA_018447755.1	533	Bacteria>Terrabacteria_group>Cyanobacteria/Melainabacteria_group>Cyanobacteria>Nostocales	Dolichospermum_sp._OL01	DGQHR-domain-containing-protein-[Dolichospermum-sp.-OL01]	2717874
MBS9393286.1	DndB-ParB-nuclease	GCA_018447755.1	391	Bacteria>Terrabacteria_group>Cyanobacteria/Melainabacteria_group>Cyanobacteria>Nostocales	Dolichospermum_sp._OL01	DNA-sulfur-modification-protein-DndB-[Dolichospermum-sp.-OL01]	2717874
WP_168642238.1	DndB-ParB-nuclease	GCF_009711935.1	533	Bacteria>Terrabacteria_group>Cyanobacteria/Melainabacteria_group>Cyanobacteria>Nostocales	Dolichospermum_sp._UHCC_0259	DGQHR-domain-containing-protein-[Dolichospermum-sp.-UHCC-0259]	2590010
WP_168642237.1	DndB-ParB-nuclease	GCF_009711935.1	396	Bacteria>Terrabacteria_group>Cyanobacteria/Melainabacteria_group>Cyanobacteria>Nostocales	Dolichospermum_sp._UHCC_0259	DNA-sulfur-modification-protein-DndB-[Dolichospermum-sp.-UHCC-0259]	2590010
WP_168633478.1	DndB-ParB-nuclease	GCF_009711985.1	532	Bacteria>Terrabacteria_group>Cyanobacteria/Melainabacteria_group>Cyanobacteria>Nostocales	Dolichospermum_sp._UHCC_0260	DGQHR-domain-containing-protein-[Dolichospermum-sp.-UHCC-0260]	2590025
WP_148760506.1	DndB-ParB-nuclease	GCF_008121535.1	532	Bacteria>Terrabacteria_group>Cyanobacteria/Melainabacteria_group>Cyanobacteria>Nostocales	Dolichospermum_sp._UHCC_0315A	DGQHR-domain-containing-protein-[Dolichospermum-sp.-UHCC-0315A]	1914871
QEI40020.1	DndB-ParB-nuclease	GCA_008121535.1	532	Bacteria>Terrabacteria_group>Cyanobacteria/Melainabacteria_group>Cyanobacteria>Nostocales	Dolichospermum_sp._UHCC_0315A	hypothetical-protein-BMF77_00577-[Dolichospermum-sp.-UHCC-0315A]	1914871
WP_168652251.1	DndB-ParB-nuclease	GCF_009711925.1	532	Bacteria>Terrabacteria_group>Cyanobacteria/Melainabacteria_group>Cyanobacteria>Nostocales	Dolichospermum_sp._UHCC_0352	DGQHR-domain-containing-protein-[Dolichospermum-sp.-UHCC-0352]	2590011
QSV55038.1	DndB-ParB-nuclease	GCA_017346795.1	533	Bacteria>Terrabacteria_group>Cyanobacteria/Melainabacteria_group>Cyanobacteria>Nostocales	Dolichospermum_sp._UKL201	DGQHR-domain-containing-protein-[Dolichospermum-sp.-UKL201]	2717876
QSV55039.1	DndB-ParB-nuclease	GCA_017346795.1	391	Bacteria>Terrabacteria_group>Cyanobacteria/Melainabacteria_group>Cyanobacteria>Nostocales	Dolichospermum_sp._UKL201	DGQHR-domain-containing-protein-[Dolichospermum-sp.-UKL201]	2717876
MBS9388623.1	DndB-ParB-nuclease	GCA_018447775.1	533	Bacteria>Terrabacteria_group>Cyanobacteria/Melainabacteria_group>Cyanobacteria>Nostocales	Dolichospermum_sp._WA123	DGQHR-domain-containing-protein-[Dolichospermum-sp.-WA123]	2717878
MBS9388622.1	DndB-ParB-nuclease	GCA_018447775.1	401	Bacteria>Terrabacteria_group>Cyanobacteria/Melainabacteria_group>Cyanobacteria>Nostocales	Dolichospermum_sp._WA123	DNA-sulfur-modification-protein-DndB-[Dolichospermum-sp.-WA123]	2717878
WP_127082687.1	DndB-ParB-nuclease	GCF_003991905.1	446	Bacteria>Terrabacteria_group>Cyanobacteria/Melainabacteria_group>Cyanobacteria>Nostocales	Dulcicalothrix_desertica	DGQHR-domain-containing-protein-[Calothrix-desertica]	32056
WP_127082692.1	DndB-ParB-nuclease	GCF_003991905.1	531	Bacteria>Terrabacteria_group>Cyanobacteria/Melainabacteria_group>Cyanobacteria>Nostocales	Dulcicalothrix_desertica	DGQHR-domain-containing-protein-[Calothrix-desertica]	32056
WP_073556525.1	DndB-ParB-nuclease	GCF_001904645.1	532	Bacteria>Terrabacteria_group>Cyanobacteria/Melainabacteria_group>Cyanobacteria>Nostocales	Fischerella_major	DGQHR-domain-containing-protein-[Fischerella-major]	210993
OKH12209.1	DndB-ParB-nuclease	GCA_001904645.1	532	Bacteria>Terrabacteria_group>Cyanobacteria/Melainabacteria_group>Cyanobacteria>Nostocales	Fischerella_major_NIES-592	hypothetical-protein-NIES592_18525-[Fischerella-major-NIES-592]	210994
WP_016868054.1	DndB-ParB-nuclease	GCF_000317205.1	532	Bacteria>Terrabacteria_group>Cyanobacteria/Melainabacteria_group>Cyanobacteria>Nostocales	Fischerella	MULTISPECIES:-DGQHR-domain-containing-protein-[Fischerella]	1190
PLZ85818.1	DndB-ParB-nuclease	GCA_002870665.1	532	Bacteria>Terrabacteria_group>Cyanobacteria/Melainabacteria_group>Cyanobacteria>Nostocales	Fischerella_muscicola_CCMEE_5323	DGQHR-domain-containing-protein-[Fischerella-muscicola-CCMEE-5323]	2019572
WP_016859405.1	DndB-ParB-nuclease	GCF_000317245.1	532	Bacteria>Terrabacteria_group>Cyanobacteria/Melainabacteria_group>Cyanobacteria>Nostocales	Fischerella_muscicola	DGQHR-domain-containing-protein-[Fischerella-muscicola]	92938
NWF61948.1	DndB-ParB-nuclease	GCA_013388695.1	532	Bacteria>Terrabacteria_group>Cyanobacteria/Melainabacteria_group>Cyanobacteria>Nostocales	Fischerella_sp.	DGQHR-domain-containing-protein-[Fischerella-sp.]	1191
MBD2429720.1	DndB-ParB-nuclease	GCA_014697535.1	532	Bacteria>Terrabacteria_group>Cyanobacteria/Melainabacteria_group>Cyanobacteria>Nostocales	Fischerella_sp._FACHB-380	DGQHR-domain-containing-protein-[Fischerella-sp.-FACHB-380]	2692799
WP_062244432.1	DndB-ParB-nuclease	GCF_001548455.1	532	Bacteria>Terrabacteria_group>Cyanobacteria/Melainabacteria_group>Cyanobacteria>Nostocales	Fischerella_sp._NIES-3754	DGQHR-domain-containing-protein-[Fischerella-sp.-NIES-3754]	1752063
WP_096678462.1	DndB-ParB-nuclease	GCF_002368315.1	533	Bacteria>Terrabacteria_group>Cyanobacteria/Melainabacteria_group>Cyanobacteria>Nostocales	Fischerella_sp._NIES-4106	DGQHR-domain-containing-protein-[Fischerella-sp.-NIES-4106]	2005456
BAZ70453.1	DndB-ParB-nuclease	GCA_002368315.1	533	Bacteria>Terrabacteria_group>Cyanobacteria/Melainabacteria_group>Cyanobacteria>Nostocales	Fischerella_sp._NIES-4106	hypothetical-protein-NIES4106_52470-[Fischerella-sp.-NIES-4106]	2005456
WP_017310455.1	DndB-ParB-nuclease	GCF_000315585.1	533	Bacteria>Terrabacteria_group>Cyanobacteria/Melainabacteria_group>Cyanobacteria>Nostocales	Fischerella_sp._PCC_9339	DGQHR-domain-containing-protein-[Fischerella-sp.-PCC-9339]	1174528
WP_026722162.1	DndB-ParB-nuclease	GCF_000447295.1	388	Bacteria>Terrabacteria_group>Cyanobacteria/Melainabacteria_group>Cyanobacteria>Nostocales	Fischerella_sp._PCC_9431	DGQHR-domain-containing-protein-[Fischerella-sp.-PCC-9431]	1173023
WP_026722163.1	DndB-ParB-nuclease	GCF_000447295.1	532	Bacteria>Terrabacteria_group>Cyanobacteria/Melainabacteria_group>Cyanobacteria>Nostocales	Fischerella_sp._PCC_9431	DGQHR-domain-containing-protein-[Fischerella-sp.-PCC-9431]	1173023
WP_026731959.1	DndB-ParB-nuclease	GCF_000517105.1	532	Bacteria>Terrabacteria_group>Cyanobacteria/Melainabacteria_group>Cyanobacteria>Nostocales	Fischerella_sp._PCC_9605	DGQHR-domain-containing-protein-[Fischerella-sp.-PCC-9605]	1173024
PMB29432.1	DndB-ParB-nuclease	GCA_002870705.1	533	Bacteria>Terrabacteria_group>Cyanobacteria/Melainabacteria_group>Cyanobacteria>Nostocales	Fischerella_thermalis_BR2B	DGQHR-domain-containing-protein-[Fischerella-thermalis-BR2B]	2019668
PLZ85436.1	DndB-ParB-nuclease	GCA_002870795.1	533	Bacteria>Terrabacteria_group>Cyanobacteria/Melainabacteria_group>Cyanobacteria>Nostocales	Fischerella_thermalis_CCMEE_5194	hypothetical-protein-CI593_21030-[Fischerella-thermalis-CCMEE-5194]	2026246
PLZ87942.1	DndB-ParB-nuclease	GCA_002870825.1	516	Bacteria>Terrabacteria_group>Cyanobacteria/Melainabacteria_group>Cyanobacteria>Nostocales	Fischerella_thermalis_CCMEE_5196	hypothetical-protein-CI594_20820,-partial-[Fischerella-thermalis-CCMEE-5196]	2026245
PMB47196.1	DndB-ParB-nuclease	GCA_002870785.1	533	Bacteria>Terrabacteria_group>Cyanobacteria/Melainabacteria_group>Cyanobacteria>Nostocales	Fischerella_thermalis_CCMEE_5201	hypothetical-protein-CEN39_24735-[Fischerella-thermalis-CCMEE-5201]	2019672
PMB40669.1	DndB-ParB-nuclease	GCA_002870745.1	533	Bacteria>Terrabacteria_group>Cyanobacteria/Melainabacteria_group>Cyanobacteria>Nostocales	Fischerella_thermalis_CCMEE_5205	hypothetical-protein-CEN40_21875-[Fischerella-thermalis-CCMEE-5205]	2019671
PLZ99956.1	DndB-ParB-nuclease	GCA_002870565.1	532	Bacteria>Terrabacteria_group>Cyanobacteria/Melainabacteria_group>Cyanobacteria>Nostocales	Fischerella_thermalis_CCMEE_5268	hypothetical-protein-CEN50_05385-[Fischerella-thermalis-CCMEE-5268]	2019662
PMB11459.1	DndB-ParB-nuclease	GCA_002870585.1	533	Bacteria>Terrabacteria_group>Cyanobacteria/Melainabacteria_group>Cyanobacteria>Nostocales	Fischerella_thermalis_CCMEE_5273	hypothetical-protein-CEN49_01520-[Fischerella-thermalis-CCMEE-5273]	2019663
PMB18875.1	DndB-ParB-nuclease	GCA_002870675.1	533	Bacteria>Terrabacteria_group>Cyanobacteria/Melainabacteria_group>Cyanobacteria>Nostocales	Fischerella_thermalis_CCMEE_5318	hypothetical-protein-CEN46_20215-[Fischerella-thermalis-CCMEE-5318]	2019666
WP_009455568.1	DndB-ParB-nuclease	GCF_000231365.1	533	Bacteria>Terrabacteria_group>Cyanobacteria/Melainabacteria_group>Cyanobacteria>Nostocales	Fischerella_thermalis	DGQHR-domain-containing-protein-[Fischerella-thermalis]	372787
WP_102146389.1	DndB-ParB-nuclease	GCF_002870525.1	533	Bacteria>Terrabacteria_group>Cyanobacteria/Melainabacteria_group>Cyanobacteria>Nostocales	Fischerella_thermalis	DGQHR-domain-containing-protein-[Fischerella-thermalis]	372787
WP_102147404.1	DndB-ParB-nuclease	GCF_003346965.1	533	Bacteria>Terrabacteria_group>Cyanobacteria/Melainabacteria_group>Cyanobacteria>Nostocales	Fischerella_thermalis	DGQHR-domain-containing-protein-[Fischerella-thermalis]	372787
WP_102171772.1	DndB-ParB-nuclease	GCF_002870565.1	532	Bacteria>Terrabacteria_group>Cyanobacteria/Melainabacteria_group>Cyanobacteria>Nostocales	Fischerella_thermalis	DGQHR-domain-containing-protein-[Fischerella-thermalis]	372787
WP_102178129.1	DndB-ParB-nuclease	GCF_002870635.1	533	Bacteria>Terrabacteria_group>Cyanobacteria/Melainabacteria_group>Cyanobacteria>Nostocales	Fischerella_thermalis	DGQHR-domain-containing-protein-[Fischerella-thermalis]	372787
WP_102182911.1	DndB-ParB-nuclease	GCF_002870675.1	533	Bacteria>Terrabacteria_group>Cyanobacteria/Melainabacteria_group>Cyanobacteria>Nostocales	Fischerella_thermalis	DGQHR-domain-containing-protein-[Fischerella-thermalis]	372787
WP_102206589.1	DndB-ParB-nuclease	GCF_002870725.1	532	Bacteria>Terrabacteria_group>Cyanobacteria/Melainabacteria_group>Cyanobacteria>Nostocales	Fischerella_thermalis	DGQHR-domain-containing-protein-[Fischerella-thermalis]	372787
EHC18149.1	DndB-ParB-nuclease	GCA_000231365.2	533	Bacteria>Terrabacteria_group>Cyanobacteria/Melainabacteria_group>Cyanobacteria>Nostocales	Fischerella_thermalis_JSC-11	DGQHR-domain-protein-[Fischerella-thermalis-JSC-11]	741277
MBF2070898.1	DndB-ParB-nuclease	GCA_015272315.1	533	Bacteria>Terrabacteria_group>Cyanobacteria/Melainabacteria_group>Cyanobacteria>Nostocales	Fischerella_thermalis_M48_A2018_028	DGQHR-domain-containing-protein-[Fischerella-thermalis-M48_A2018_028]	2774337
MBF1989650.1	DndB-ParB-nuclease	GCA_015272275.1	533	Bacteria>Terrabacteria_group>Cyanobacteria/Melainabacteria_group>Cyanobacteria>Nostocales	Fischerella_thermalis_M58_A2018_009	DGQHR-domain-containing-protein-[Fischerella-thermalis-M58_A2018_009]	2774338
MBF2058838.1	DndB-ParB-nuclease	GCA_015272255.1	533	Bacteria>Terrabacteria_group>Cyanobacteria/Melainabacteria_group>Cyanobacteria>Nostocales	Fischerella_thermalis_M66_A2018_004	DGQHR-domain-containing-protein-[Fischerella-thermalis-M66_A2018_004]	2774339
PLZ09415.1	DndB-ParB-nuclease	GCA_002870545.1	533	Bacteria>Terrabacteria_group>Cyanobacteria/Melainabacteria_group>Cyanobacteria>Nostocales	Fischerella_thermalis_WC114	hypothetical-protein-CBP17_14450-[Fischerella-thermalis-WC114]	1521403
PLZ08892.1	DndB-ParB-nuclease	GCA_002870265.1	533	Bacteria>Terrabacteria_group>Cyanobacteria/Melainabacteria_group>Cyanobacteria>Nostocales	Fischerella_thermalis_WC119	hypothetical-protein-CBP18_12965-[Fischerella-thermalis-WC119]	1891707
PLZ53068.1	DndB-ParB-nuclease	GCA_002870475.1	533	Bacteria>Terrabacteria_group>Cyanobacteria/Melainabacteria_group>Cyanobacteria>Nostocales	Fischerella_thermalis_WC439	hypothetical-protein-CBP24_17420-[Fischerella-thermalis-WC439]	1521418
PLZ35425.1	DndB-ParB-nuclease	GCA_002870365.1	533	Bacteria>Terrabacteria_group>Cyanobacteria/Melainabacteria_group>Cyanobacteria>Nostocales	Fischerella_thermalis_WC558	hypothetical-protein-CBP10_03420-[Fischerella-thermalis-WC558]	1521425
WP_017652011.1	DndB-ParB-nuclease	GCF_000332295.1	533	Bacteria>Terrabacteria_group>Cyanobacteria/Melainabacteria_group>Cyanobacteria>Nostocales	Fortiea_contorta	DGQHR-domain-containing-protein-[Fortiea-contorta]	1892405
MBE9004658.1	DndB-ParB-nuclease	GCA_015206815.1	532	Bacteria>Terrabacteria_group>Cyanobacteria/Melainabacteria_group>Cyanobacteria>Nostocales	Fortiea_sp._LEGE_XX443	DGQHR-domain-containing-protein-[Fortiea-sp.-LEGE-XX443]	1828611
WP_193882524.1	DndB-ParB-nuclease	GCF_015206815.1	532	Bacteria>Terrabacteria_group>Cyanobacteria/Melainabacteria_group>Cyanobacteria>Nostocales	Fortiea_sp._LEGE_XX443	DGQHR-domain-containing-protein-[Fortiea-sp.-LEGE-XX443]	1828611
MBE9004660.1	DndB-ParB-nuclease	GCA_015206815.1	665	Bacteria>Terrabacteria_group>Cyanobacteria/Melainabacteria_group>Cyanobacteria>Nostocales	Fortiea_sp._LEGE_XX443	hypothetical-protein-[Fortiea-sp.-LEGE-XX443]	1828611
WP_193882530.1	DndB-ParB-nuclease	GCF_015206815.1	665	Bacteria>Terrabacteria_group>Cyanobacteria/Melainabacteria_group>Cyanobacteria>Nostocales	Fortiea_sp._LEGE_XX443	hypothetical-protein-[Fortiea-sp.-LEGE-XX443]	1828611
BAY90012.1	DndB-ParB-nuclease	GCA_002368275.1	81	Bacteria>Terrabacteria_group>Cyanobacteria/Melainabacteria_group>Cyanobacteria>Nostocales	Fremyella_diplosiphon_NIES-3275	hypothetical-protein-NIES3275_20220-[Fremyella-diplosiphon-NIES-3275]	1973485
BAY90014.1	DndB-ParB-nuclease	GCA_002368275.1	433	Bacteria>Terrabacteria_group>Cyanobacteria/Melainabacteria_group>Cyanobacteria>Nostocales	Fremyella_diplosiphon_NIES-3275	hypothetical-protein-NIES3275_20240-[Fremyella-diplosiphon-NIES-3275]	1973485
MBW4642826.1	DndB-ParB-nuclease	GCA_019359495.1	532	Bacteria>Terrabacteria_group>Cyanobacteria/Melainabacteria_group>Cyanobacteria>Nostocales	Goleter_apudmare_HA4340-LM2	DGQHR-domain-containing-protein-[Goleter-apudmare-HA4340-LM2]	1382955
RAM50683.1	DndB-ParB-nuclease	GCA_003261315.1	532	Bacteria>Terrabacteria_group>Cyanobacteria/Melainabacteria_group>Cyanobacteria>Nostocales	Hapalosiphonaceae_cyanobacterium_JJU2	hypothetical-protein-C6Y22_15315-[Hapalosiphonaceae-cyanobacterium-JJU2]	2109628
WP_053454959.1	DndB-ParB-nuclease	GCF_001275395.1	532	Bacteria>Terrabacteria_group>Cyanobacteria/Melainabacteria_group>Cyanobacteria>Nostocales	Hapalosiphon_sp._MRB220	DGQHR-domain-containing-protein-[Hapalosiphon-sp.-MRB220]	1704290
KOP27933.1	DndB-ParB-nuclease	GCA_001275395.1	532	Bacteria>Terrabacteria_group>Cyanobacteria/Melainabacteria_group>Cyanobacteria>Nostocales	Hapalosiphon_sp._MRB220	hypothetical-protein-AMR41_02295-[Hapalosiphon-sp.-MRB220]	1704290
WP_039752462.1	DndB-ParB-nuclease	GCF_000817785.2	528	Bacteria>Terrabacteria_group>Cyanobacteria/Melainabacteria_group>Cyanobacteria>Nostocales	Hassallia_byssoidea	DGQHR-domain-containing-protein-[Hassallia-byssoidea]	482630
NEU77104.1	DndB-ParB-nuclease	GCA_000817785.2	528	Bacteria>Terrabacteria_group>Cyanobacteria/Melainabacteria_group>Cyanobacteria>Nostocales	Hassallia_byssoidea_VB512170	DGQHR-domain-containing-protein-[Hassallia-byssoidea-VB512170]	1304833
MBW4609798.1	DndB-ParB-nuclease	GCA_019359425.1	529	Bacteria>Terrabacteria_group>Cyanobacteria/Melainabacteria_group>Cyanobacteria>Nostocales	Hassallia_sp._WJT32-NPBG1	DGQHR-domain-containing-protein-[Hassallia-sp.-WJT32-NPBG1]	2835088
MBD2774650.1	DndB-ParB-nuclease	GCA_014698965.1	531	Bacteria>Terrabacteria_group>Cyanobacteria/Melainabacteria_group>Cyanobacteria>Nostocales	Iningainema_tapete_BLCC-T55	DGQHR-domain-containing-protein-[Iningainema-tapete-BLCC-T55]	2748662
WP_190831995.1	DndB-ParB-nuclease	GCF_014698965.1	531	Bacteria>Terrabacteria_group>Cyanobacteria/Melainabacteria_group>Cyanobacteria>Nostocales	Iningainema_tapete	DGQHR-domain-containing-protein-[Iningainema-tapete]	2806730
WP_202895653.1	DndB-ParB-nuclease	GCF_014698965.1	490	Bacteria>Terrabacteria_group>Cyanobacteria/Melainabacteria_group>Cyanobacteria>Nostocales	Iningainema_tapete	hypothetical-protein-[Iningainema-tapete]	2806730
MBW4631064.1	DndB-ParB-nuclease	GCA_019359415.1	532	Bacteria>Terrabacteria_group>Cyanobacteria/Melainabacteria_group>Cyanobacteria>Nostocales	Iphinoe_sp._HA4291-MV1	DGQHR-domain-containing-protein-[Iphinoe-sp.-HA4291-MV1]	1494767
MBW4684400.1	DndB-ParB-nuclease	GCA_019359395.1	532	Bacteria>Terrabacteria_group>Cyanobacteria/Melainabacteria_group>Cyanobacteria>Nostocales	Komarekiella_atlantica_HA4396-MV6	DGQHR-domain-containing-protein-[Komarekiella-atlantica-HA4396-MV6]	2024215
MBD6617450.1	DndB-ParB-nuclease	GCA_014831675.1	530	Bacteria>Terrabacteria_group>Cyanobacteria/Melainabacteria_group>Cyanobacteria>Nostocales	Komarekiella_sp._'clone_1'	DGQHR-domain-containing-protein-[Komarekiella-sp.-'clone-1']	2593771
WP_191758667.1	DndB-ParB-nuclease	GCF_014831675.1	530	Bacteria>Terrabacteria_group>Cyanobacteria/Melainabacteria_group>Cyanobacteria>Nostocales	Komarekiella_sp._'clone_1'	DGQHR-domain-containing-protein-[Komarekiella-sp.-'clone-1']	2593771
WP_017315876.1	DndB-ParB-nuclease	GCF_000315565.1	533	Bacteria>Terrabacteria_group>Cyanobacteria/Melainabacteria_group>Cyanobacteria>Nostocales	Mastigocladopsis_repens	DGQHR-domain-containing-protein-[Mastigocladopsis-repens]	221287
WP_044449302.1	DndB-ParB-nuclease	GCF_001990805.2	532	Bacteria>Terrabacteria_group>Cyanobacteria/Melainabacteria_group>Cyanobacteria>Nostocales	Mastigocladus_laminosus	DGQHR-domain-containing-protein-[Mastigocladus-laminosus]	83541
TFI53829.1	DndB-ParB-nuclease	GCA_001990805.2	532	Bacteria>Terrabacteria_group>Cyanobacteria/Melainabacteria_group>Cyanobacteria>Nostocales	Mastigocladus_laminosus_UU774	DGQHR-domain-containing-protein-[Mastigocladus-laminosus-UU774]	1594576
WP_027845912.1	DndB-ParB-nuclease	GCF_001456025.1	533	Bacteria>Terrabacteria_group>Cyanobacteria/Melainabacteria_group>Cyanobacteria>Nostocales	Mastigocoleus_testarum	DGQHR-domain-containing-protein-[Mastigocoleus-testarum]	996925
MBW4561798.1	DndB-ParB-nuclease	GCA_019359325.1	532	Bacteria>Terrabacteria_group>Cyanobacteria/Melainabacteria_group>Cyanobacteria>Nostocales	Mojavia_pulchra_JT2-VF2	DGQHR-domain-containing-protein-[Mojavia-pulchra-JT2-VF2]	287848
WP_029636025.1	DndB-ParB-nuclease	GCF_014698435.1	528	Bacteria>Terrabacteria_group>Cyanobacteria/Melainabacteria_group>Cyanobacteria	Nostocales	MULTISPECIES:-DGQHR-domain-containing-protein-[Nostocales]	1161

Accession	Annotation	GCA	Length	Phyletic.spread	Strain.name	Defline	Species.Taxid
WP_039202166.1	DndB-ParB-nuclease	GCF_001277295.1	533	Bacteria>Terrabacteria_group>Cyanobacteria/Melainabacteria_group>Cyanobacteria	Nostocales	MULTISPECIES:-DGQHR-domain-containing-protein-[Nostocales]	1161
WP_029636023.1	DndB-ParB-nuclease	GCF_014698435.1	395	Bacteria>Terrabacteria_group>Cyanobacteria/Melainabacteria_group>Cyanobacteria	Nostocales	MULTISPECIES:-DNA-sulfur-modification-protein-DndB-[Nostocales]	1161
WP_096577884.1	DndB-ParB-nuclease	GCF_002367975.1	544	Bacteria>Terrabacteria_group>Cyanobacteria/Melainabacteria_group>Cyanobacteria	Nostocales	MULTISPECIES:-hypothetical-protein-[Nostocales]	1161
WP_239730005.1	DndB-ParB-nuclease	GCF_022376295.1	532	Bacteria>Terrabacteria_group>Cyanobacteria/Melainabacteria_group>Cyanobacteria>Nostocales	Nodularia_sphaerocarpa	DGQHR-domain-containing-protein-[Nodularia-sphaerocarpa]	137816
WP_239730006.1	DndB-ParB-nuclease	GCF_022376295.1	401	Bacteria>Terrabacteria_group>Cyanobacteria/Melainabacteria_group>Cyanobacteria>Nostocales	Nodularia_sphaerocarpa	DGQHR-domain-containing-protein-[Nodularia-sphaerocarpa]	137816
TVP62544.1	DndB-ParB-nuclease	GCA_007692755.1	532	Bacteria>Terrabacteria_group>Cyanobacteria/Melainabacteria_group>Cyanobacteria>Nostocales	Nodularia_sp._(in:_Bacteria)	DGQHR-domain-containing-protein-[Nodularia-sp.-(in:-Bacteria)]	2047085
TVP62545.1	DndB-ParB-nuclease	GCA_007692755.1	394	Bacteria>Terrabacteria_group>Cyanobacteria/Melainabacteria_group>Cyanobacteria>Nostocales	Nodularia_sp._(in:_Bacteria)	DGQHR-domain-containing-protein-[Nodularia-sp.-(in:-Bacteria)]	2047085
MBE9200250.1	DndB-ParB-nuclease	GCA_015207755.1	532	Bacteria>Terrabacteria_group>Cyanobacteria/Melainabacteria_group>Cyanobacteria>Nostocales	Nodularia_sp._LEGE_06071	DGQHR-domain-containing-protein-[Nodularia-sp.-LEGE-06071]	2777965
WP_217899883.1	DndB-ParB-nuclease	-	377	Bacteria>Terrabacteria_group>Cyanobacteria/Melainabacteria_group>Cyanobacteria>Nostocales	Nodularia_sp._NIES-3585	DGQHR-domain-containing-protein-[Nodularia-sp.-NIES-3585]	1973477
WP_089093062.1	DndB-ParB-nuclease	GCF_002218065.1	371	Bacteria>Terrabacteria_group>Cyanobacteria/Melainabacteria_group>Cyanobacteria>Nostocales	Nodularia_sp._NIES-3585	DNA-sulfur-modification-protein-DndB-[Nodularia-sp.-NIES-3585]	1973477
EAW46159.1	DndB-ParB-nuclease	GCA_000169135.1	532	Bacteria>Terrabacteria_group>Cyanobacteria/Melainabacteria_group>Cyanobacteria>Nostocales	Nodularia_spumigena_CCY9414	hypothetical-protein-N9414_19672-[Nodularia-spumigena-CCY9414]	313624
AHJ26580.1	DndB-ParB-nuclease	GCA_000340565.3	532	Bacteria>Terrabacteria_group>Cyanobacteria/Melainabacteria_group>Cyanobacteria>Nostocales	Nodularia_spumigena_CCY9414	hypothetical-protein-NSP_2240-[Nodularia-spumigena-CCY9414]	313624
KZL49701.1	DndB-ParB-nuclease	GCA_001623485.1	532	Bacteria>Terrabacteria_group>Cyanobacteria/Melainabacteria_group>Cyanobacteria>Nostocales	Nodularia_spumigena_CENA596	hypothetical-protein-A2T98_11435-[Nodularia-spumigena-CENA596]	1819295
WP_006195422.1	DndB-ParB-nuclease	GCF_000169135.1	532	Bacteria>Terrabacteria_group>Cyanobacteria/Melainabacteria_group>Cyanobacteria>Nostocales	Nodularia_spumigena	DGQHR-domain-containing-protein-[Nodularia-spumigena]	70799
WP_063872877.1	DndB-ParB-nuclease	GCF_001623485.1	532	Bacteria>Terrabacteria_group>Cyanobacteria/Melainabacteria_group>Cyanobacteria>Nostocales	Nodularia_spumigena	DGQHR-domain-containing-protein-[Nodularia-spumigena]	70799
MBA3921225.1	DndB-ParB-nuclease	GCA_013821625.1	139	Bacteria>Terrabacteria_group>Cyanobacteria/Melainabacteria_group>Cyanobacteria>Nostocales	Nostocaceae_cyanobacterium	hypothetical-protein-[Nostocaceae-cyanobacterium]	2723028
WP_010999042.1	DndB-ParB-nuclease	GCF_000009705.1	532	Bacteria>Terrabacteria_group>Cyanobacteria/Melainabacteria_group>Cyanobacteria>Nostocales	Nostocaceae	MULTISPECIES:-DGQHR-domain-containing-protein-[Nostocaceae]	1162
WP_011318993.1	DndB-ParB-nuclease	GCF_000204075.1	532	Bacteria>Terrabacteria_group>Cyanobacteria/Melainabacteria_group>Cyanobacteria>Nostocales	Nostocaceae	MULTISPECIES:-DGQHR-domain-containing-protein-[Nostocaceae]	1162
WP_190591281.1	DndB-ParB-nuclease	GCF_014697495.1	532	Bacteria>Terrabacteria_group>Cyanobacteria/Melainabacteria_group>Cyanobacteria>Nostocales	Nostocaceae	MULTISPECIES:-DGQHR-domain-containing-protein-[Nostocaceae]	1162
WP_011318991.1	DndB-ParB-nuclease	GCF_000204075.1	664	Bacteria>Terrabacteria_group>Cyanobacteria/Melainabacteria_group>Cyanobacteria>Nostocales	Nostocaceae	MULTISPECIES:-hypothetical-protein-[Nostocaceae]	1162
TAE57047.1	DndB-ParB-nuclease	GCA_004292695.1	531	Bacteria>Terrabacteria_group>Cyanobacteria/Melainabacteria_group>Cyanobacteria>Nostocales	Nostocales_cyanobacterium	DGQHR-domain-containing-protein-[Nostocales-cyanobacterium]	2447902
TAF05706.1	DndB-ParB-nuclease	GCA_004293145.1	532	Bacteria>Terrabacteria_group>Cyanobacteria/Melainabacteria_group>Cyanobacteria>Nostocales	Nostocales_cyanobacterium	DGQHR-domain-containing-protein-[Nostocales-cyanobacterium]	2447902
ARV61685.1	DndB-ParB-nuclease	GCA_002163975.1	333	Bacteria>Terrabacteria_group>Cyanobacteria/Melainabacteria_group>Cyanobacteria>Nostocales	Nostocales_cyanobacterium_HT-58-2	hypothetical-protein-BZZ01_26425-[Nostocales-cyanobacterium-HT-58-2]	1940762
ARV61687.1	DndB-ParB-nuclease	GCA_002163975.1	532	Bacteria>Terrabacteria_group>Cyanobacteria/Melainabacteria_group>Cyanobacteria>Nostocales	Nostocales_cyanobacterium_HT-58-2	hypothetical-protein-BZZ01_26435-[Nostocales-cyanobacterium-HT-58-2]	1940762
MBE8968157.1	DndB-ParB-nuclease	GCA_015206945.1	532	Bacteria>Terrabacteria_group>Cyanobacteria/Melainabacteria_group>Cyanobacteria>Nostocales	Nostocales_cyanobacterium_LEGE_12452	DGQHR-domain-containing-protein-[Nostocales-cyanobacterium-LEGE-12452]	2777975
MBE8968155.1	DndB-ParB-nuclease	GCA_015206945.1	665	Bacteria>Terrabacteria_group>Cyanobacteria/Melainabacteria_group>Cyanobacteria>Nostocales	Nostocales_cyanobacterium_LEGE_12452	hypothetical-protein-[Nostocales-cyanobacterium-LEGE-12452]	2777975
WP_073641339.1	DndB-ParB-nuclease	GCF_014697355.1	533	Bacteria>Terrabacteria_group>Cyanobacteria/Melainabacteria_group>Cyanobacteria>Nostocales	Nostoc_calcicola	DGQHR-domain-containing-protein-[Nostoc-calcicola]	212354
WP_073641340.1	DndB-ParB-nuclease	GCF_014697355.1	396	Bacteria>Terrabacteria_group>Cyanobacteria/Melainabacteria_group>Cyanobacteria>Nostocales	Nostoc_calcicola	DNA-sulfur-modification-protein-DndB-[Nostoc-calcicola]	212354
MBD2411573.1	DndB-ParB-nuclease	GCA_014697355.1	533	Bacteria>Terrabacteria_group>Cyanobacteria/Melainabacteria_group>Cyanobacteria>Nostocales	Nostoc_calcicola_FACHB-3891	DGQHR-domain-containing-protein-[Nostoc-calcicola-FACHB-3891]	2692903
MBD2411572.1	DndB-ParB-nuclease	GCA_014697355.1	396	Bacteria>Terrabacteria_group>Cyanobacteria/Melainabacteria_group>Cyanobacteria>Nostocales	Nostoc_calcicola_FACHB-3891	DNA-sulfur-modification-protein-DndB-[Nostoc-calcicola-FACHB-3891]	2692903
OKH34971.1	DndB-ParB-nuclease	GCA_001904715.1	533	Bacteria>Terrabacteria_group>Cyanobacteria/Melainabacteria_group>Cyanobacteria>Nostocales	Nostoc_calcicola_FACHB-389	hypothetical-protein-FACHB389_13570-[Nostoc-calcicola-FACHB-389]	1357508
OKH34972.1	DndB-ParB-nuclease	GCA_001904715.1	396	Bacteria>Terrabacteria_group>Cyanobacteria/Melainabacteria_group>Cyanobacteria>Nostocales	Nostoc_calcicola_FACHB-389	hypothetical-protein-FACHB389_13575-[Nostoc-calcicola-FACHB-389]	1357508
BAY31099.1	DndB-ParB-nuclease	GCA_002368155.1	532	Bacteria>Terrabacteria_group>Cyanobacteria/Melainabacteria_group>Cyanobacteria>Nostocales	Nostoc_carneum_NIES-2107	hypothetical-protein-NIES2107_29530-[Nostoc-carneum-NIES-2107]	1973483
MBG1262552.1	DndB-ParB-nuclease	GCA_015714765.1	532	Bacteria>Terrabacteria_group>Cyanobacteria/Melainabacteria_group>Cyanobacteria>Nostocales	Nostoc_commune_BAE	DGQHR-domain-containing-protein-[Nostoc-commune-BAE]	2650610
WP_109011371.1	DndB-ParB-nuclease	GCF_003113895.1	531	Bacteria>Terrabacteria_group>Cyanobacteria/Melainabacteria_group>Cyanobacteria>Nostocales	Nostoc_commune	DGQHR-domain-containing-protein-[Nostoc-commune]	1178
WP_196526942.1	DndB-ParB-nuclease	GCF_015714765.1	532	Bacteria>Terrabacteria_group>Cyanobacteria/Melainabacteria_group>Cyanobacteria>Nostocales	Nostoc_commune	DGQHR-domain-containing-protein-[Nostoc-commune]	1178
WP_109011370.1	DndB-ParB-nuclease	GCF_003113895.1	401	Bacteria>Terrabacteria_group>Cyanobacteria/Melainabacteria_group>Cyanobacteria>Nostocales	Nostoc_commune	DNA-sulfur-modification-protein-DndB-[Nostoc-commune]	1178
BBD67538.1	DndB-ParB-nuclease	GCA_003990685.1	531	Bacteria>Terrabacteria_group>Cyanobacteria/Melainabacteria_group>Cyanobacteria>Nostocales	Nostoc_commune_HK-02	hypothetical-protein-NIES4070_39290-[Nostoc-commune-HK-02]	1137087
BBD67539.1	DndB-ParB-nuclease	GCA_003990685.1	401	Bacteria>Terrabacteria_group>Cyanobacteria/Melainabacteria_group>Cyanobacteria>Nostocales	Nostoc_commune_HK-02	hypothetical-protein-NIES4070_39300-[Nostoc-commune-HK-02]	1137087
GBG21479.1	DndB-ParB-nuclease	GCA_003113895.1	401	Bacteria>Terrabacteria_group>Cyanobacteria/Melainabacteria_group>Cyanobacteria>Nostocales	Nostoc_commune_NIES-4072	hypothetical-protein-NIES4072_51650-[Nostoc-commune-NIES-4072]	2005467
GBG21480.1	DndB-ParB-nuclease	GCA_003113895.1	531	Bacteria>Terrabacteria_group>Cyanobacteria/Melainabacteria_group>Cyanobacteria>Nostocales	Nostoc_commune_NIES-4072	hypothetical-protein-NIES4072_51660-[Nostoc-commune-NIES-4072]	2005467
WP_103126460.1	DndB-ParB-nuclease	GCF_002897135.1	532	Bacteria>Terrabacteria_group>Cyanobacteria/Melainabacteria_group>Cyanobacteria>Nostocales	Nostoc_cycadae	DGQHR-domain-containing-protein-[Nostoc-cycadae]	246795
GBE94859.1	DndB-ParB-nuclease	GCA_002897135.1	532	Bacteria>Terrabacteria_group>Cyanobacteria/Melainabacteria_group>Cyanobacteria>Nostocales	Nostoc_cycadae_WK-1	DGQHR-domain-containing-protein-[Nostoc-cycadae-WK-1]	1861711
MBW4426671.1	DndB-ParB-nuclease	GCA_019359245.1	532	Bacteria>Terrabacteria_group>Cyanobacteria/Melainabacteria_group>Cyanobacteria>Nostocales	Nostoc_desertorum_CM1-VF14	DGQHR-domain-containing-protein-[Nostoc-desertorum-CM1-VF14]	287862
QMS90720.1	DndB-ParB-nuclease	GCA_014023275.1	533	Bacteria>Terrabacteria_group>Cyanobacteria/Melainabacteria_group>Cyanobacteria>Nostocales	Nostoc_edaphicum_CCNP1411	DGQHR-domain-containing-protein-[Nostoc-edaphicum-CCNP1411]	1472755
QMS90721.1	DndB-ParB-nuclease	GCA_014023275.1	401	Bacteria>Terrabacteria_group>Cyanobacteria/Melainabacteria_group>Cyanobacteria>Nostocales	Nostoc_edaphicum_CCNP1411	DGQHR-domain-containing-protein-[Nostoc-edaphicum-CCNP1411]	1472755
WP_181928474.1	DndB-ParB-nuclease	GCF_014023275.1	533	Bacteria>Terrabacteria_group>Cyanobacteria/Melainabacteria_group>Cyanobacteria>Nostocales	Nostoc_edaphicum	DGQHR-domain-containing-protein-[Nostoc-edaphicum]	264686
WP_181928475.1	DndB-ParB-nuclease	GCF_014023275.1	401	Bacteria>Terrabacteria_group>Cyanobacteria/Melainabacteria_group>Cyanobacteria>Nostocales	Nostoc_edaphicum	DNA-sulfur-modification-protein-DndB-[Nostoc-edaphicum]	264686
MCC5598640.1	DndB-ParB-nuclease	GCA_020829495.1	532	Bacteria>Terrabacteria_group>Cyanobacteria/Melainabacteria_group>Cyanobacteria>Nostocales	Nostoc_favosum_CHAB5714	DGQHR-domain-containing-protein-[Nostoc-sp.-CHAB-5714]	2780399
WP_229483466.1	DndB-ParB-nuclease	GCF_020829495.1	532	Bacteria>Terrabacteria_group>Cyanobacteria/Melainabacteria_group>Cyanobacteria>Nostocales	Nostoc_favosum	DGQHR-domain-containing-protein-[Nostoc-favosum]	2907819
AUB40444.1	DndB-ParB-nuclease	GCA_002813575.1	532	Bacteria>Terrabacteria_group>Cyanobacteria/Melainabacteria_group>Cyanobacteria>Nostocales	Nostoc_flagelliforme_CCNUN1	DndB-like-protein-[Nostoc-flagelliforme-CCNUN1]	2038116
WP_100901157.1	DndB-ParB-nuclease	GCF_002813575.1	532	Bacteria>Terrabacteria_group>Cyanobacteria/Melainabacteria_group>Cyanobacteria>Nostocales	Nostoc_flagelliforme	DGQHR-domain-containing-protein-[Nostoc-flagelliforme]	1306274
MBD2529183.1	DndB-ParB-nuclease	GCA_014698065.1	69	Bacteria>Terrabacteria_group>Cyanobacteria/Melainabacteria_group>Cyanobacteria>Nostocales	Nostoc_flagelliforme_FACHB-838	hypothetical-protein-[Nostoc-flagelliforme-FACHB-838]	2692904
MBD2649193.1	DndB-ParB-nuclease	GCA_014698505.1	532	Bacteria>Terrabacteria_group>Cyanobacteria/Melainabacteria_group>Cyanobacteria>Nostocales	Nostoc_foliaceum_FACHB-393	DGQHR-domain-containing-protein-[Nostoc-foliaceum-FACHB-393]	2692915
WP_096537786.1	DndB-ParB-nuclease	GCF_002368035.1	532	Bacteria>Terrabacteria_group>Cyanobacteria/Melainabacteria_group>Cyanobacteria>Nostocales	Nostoc_linckia	DGQHR-domain-containing-protein-[Nostoc-linckia]	92942
WP_099071150.1	DndB-ParB-nuclease	GCF_002607965.1	532	Bacteria>Terrabacteria_group>Cyanobacteria/Melainabacteria_group>Cyanobacteria>Nostocales	Nostoc_linckia	DGQHR-domain-containing-protein-[Nostoc-linckia]	92942
WP_190657930.1	DndB-ParB-nuclease	GCF_014696615.1	532	Bacteria>Terrabacteria_group>Cyanobacteria/Melainabacteria_group>Cyanobacteria>Nostocales	Nostoc_linckia	DGQHR-domain-containing-protein-[Nostoc-linckia]	92942
WP_096537785.1	DndB-ParB-nuclease	GCF_002368035.1	396	Bacteria>Terrabacteria_group>Cyanobacteria/Melainabacteria_group>Cyanobacteria>Nostocales	Nostoc_linckia	DNA-sulfur-modification-protein-DndB-[Nostoc-linckia]	92942
WP_190657928.1	DndB-ParB-nuclease	GCF_014696615.1	389	Bacteria>Terrabacteria_group>Cyanobacteria/Melainabacteria_group>Cyanobacteria>Nostocales	Nostoc_linckia	DNA-sulfur-modification-protein-DndB-[Nostoc-linckia]	92942
MBD2212365.1	DndB-ParB-nuclease	GCA_014696615.1	532	Bacteria>Terrabacteria_group>Cyanobacteria/Melainabacteria_group>Cyanobacteria>Nostocales	Nostoc_linckia_FACHB-104	DGQHR-domain-containing-protein-[Nostoc-linckia-FACHB-104]	2692905
MBD2212364.1	DndB-ParB-nuclease	GCA_014696615.1	389	Bacteria>Terrabacteria_group>Cyanobacteria/Melainabacteria_group>Cyanobacteria>Nostocales	Nostoc_linckia_FACHB-104	DNA-sulfur-modification-protein-DndB-[Nostoc-linckia-FACHB-104]	2692905
MBD2563001.1	DndB-ParB-nuclease	GCA_014698525.1	532	Bacteria>Terrabacteria_group>Cyanobacteria/Melainabacteria_group>Cyanobacteria>Nostocales	Nostoc_linckia_FACHB-391	DGQHR-domain-containing-protein-[Nostoc-linckia-FACHB-391]	2692906
BAY77300.1	DndB-ParB-nuclease	GCA_002368035.1	396	Bacteria>Terrabacteria_group>Cyanobacteria/Melainabacteria_group>Cyanobacteria>Nostocales	Nostoc_linckia_NIES-25	hypothetical-protein-NIES25_37610-[Nostoc-linckia-NIES-25]	1091006
BAY77301.1	DndB-ParB-nuclease	GCA_002368035.1	532	Bacteria>Terrabacteria_group>Cyanobacteria/Melainabacteria_group>Cyanobacteria>Nostocales	Nostoc_linckia_NIES-25	hypothetical-protein-NIES25_37620-[Nostoc-linckia-NIES-25]	1091006
PHJ56143.1	DndB-ParB-nuclease	GCA_002607965.1	532	Bacteria>Terrabacteria_group>Cyanobacteria/Melainabacteria_group>Cyanobacteria>Nostocales	Nostoc_linckia_z1	DGQHR-domain-containing-protein-[Nostoc-linckia-z1]	1612423
PHJ66186.1	DndB-ParB-nuclease	GCA_002607955.1	532	Bacteria>Terrabacteria_group>Cyanobacteria/Melainabacteria_group>Cyanobacteria>Nostocales	Nostoc_linckia_z2	DGQHR-domain-containing-protein-[Nostoc-linckia-z2]	1628741
PHJ63406.1	DndB-ParB-nuclease	GCA_002608015.1	532	Bacteria>Terrabacteria_group>Cyanobacteria/Melainabacteria_group>Cyanobacteria>Nostocales	Nostoc_linckia_z3	DGQHR-domain-containing-protein-[Nostoc-linckia-z3]	1628742
PHJ77884.1	DndB-ParB-nuclease	GCA_002608075.1	532	Bacteria>Terrabacteria_group>Cyanobacteria/Melainabacteria_group>Cyanobacteria>Nostocales	Nostoc_linckia_z4	DGQHR-domain-containing-protein-[Nostoc-linckia-z4]	1628743
RCJ28464.1	DndB-ParB-nuclease	GCA_003326215.1	532	Bacteria>Terrabacteria_group>Cyanobacteria/Melainabacteria_group>Cyanobacteria>Nostocales	Nostoc_minutum_NIES-26	hypothetical-protein-A6770_23665-[Nostoc-minutum-NIES-26]	1844469
MCC5664173.1	DndB-ParB-nuclease	GCA_020829645.1	532	Bacteria>Terrabacteria_group>Cyanobacteria/Melainabacteria_group>Cyanobacteria>Nostocales	Nostoc_mirabile_CHAB5784	DGQHR-domain-containing-protein-[Nostoc-sp.-CHAB-5784]	2780401
WP_229494265.1	DndB-ParB-nuclease	GCF_020829645.1	532	Bacteria>Terrabacteria_group>Cyanobacteria/Melainabacteria_group>Cyanobacteria>Nostocales	Nostoc_mirabile	DGQHR-domain-containing-protein-[Nostoc-mirabile]	2907820
WP_190896436.1	DndB-ParB-nuclease	GCF_014698505.1	532	Bacteria>Terrabacteria_group>Cyanobacteria/Melainabacteria_group>Cyanobacteria>Nostocales	Nostoc	MULTISPECIES:-DGQHR-domain-containing-protein-[Nostoc]	1177
WP_190954514.1	DndB-ParB-nuclease	GCF_014698635.1	532	Bacteria>Terrabacteria_group>Cyanobacteria/Melainabacteria_group>Cyanobacteria>Nostocales	Nostoc	MULTISPECIES:-DGQHR-domain-containing-protein-[Nostoc]	1177
MBD2733775.1	DndB-ParB-nuclease	GCA_014698835.1	532	Bacteria>Terrabacteria_group>Cyanobacteria/Melainabacteria_group>Cyanobacteria>Nostocales	Nostoc_paludosum_FACHB-159	DGQHR-domain-containing-protein-[Nostoc-paludosum-FACHB-159]	2692908
WP_190566095.1	DndB-ParB-nuclease	GCF_014696625.1	532	Bacteria>Terrabacteria_group>Cyanobacteria/Melainabacteria_group>Cyanobacteria>Nostocales	Nostoc_parmelioides	DGQHR-domain-containing-protein-[Nostoc-parmelioides]	1521621
MBD2250741.1	DndB-ParB-nuclease	GCA_014696625.1	532	Bacteria>Terrabacteria_group>Cyanobacteria/Melainabacteria_group>Cyanobacteria>Nostocales	Nostoc_parmelioides_FACHB-3921	DGQHR-domain-containing-protein-[Nostoc-parmelioides-FACHB-3921]	2692909
WP_144427458.1	DndB-ParB-nuclease	GCF_001298445.1	348	Bacteria>Terrabacteria_group>Cyanobacteria/Melainabacteria_group>Cyanobacteria>Nostocales	Nostoc_piscinale	DNA-sulfur-modification-protein-DndB-[Nostoc-piscinale]	224012
WP_012410818.1	DndB-ParB-nuclease	GCF_000020025.1	532	Bacteria>Terrabacteria_group>Cyanobacteria/Melainabacteria_group>Cyanobacteria>Nostocales	Nostoc_punctiforme	DGQHR-domain-containing-protein-[Nostoc-punctiforme]	272131
WP_167315654.1	DndB-ParB-nuclease	GCF_000020025.1	333	Bacteria>Terrabacteria_group>Cyanobacteria/Melainabacteria_group>Cyanobacteria>Nostocales	Nostoc_punctiforme	DGQHR-domain-containing-protein-[Nostoc-punctiforme]	272131
WP_190950974.1	DndB-ParB-nuclease	GCF_014698495.1	532	Bacteria>Terrabacteria_group>Cyanobacteria/Melainabacteria_group>Cyanobacteria>Nostocales	Nostoc_punctiforme	DGQHR-domain-containing-protein-[Nostoc-punctiforme]	272131
MBD2613786.1	DndB-ParB-nuclease	GCA_014698495.1	532	Bacteria>Terrabacteria_group>Cyanobacteria/Melainabacteria_group>Cyanobacteria>Nostocales	Nostoc_punctiforme_FACHB-252	DGQHR-domain-containing-protein-[Nostoc-punctiforme-FACHB-252]	1357509
RCJ35713.1	DndB-ParB-nuclease	GCA_003326195.1	532	Bacteria>Terrabacteria_group>Cyanobacteria/Melainabacteria_group>Cyanobacteria>Nostocales	Nostoc_punctiforme_NIES-2108	hypothetical-protein-A6769_18905-[Nostoc-punctiforme-NIES-2108]	1356359
WP_086758235.1	DndB-ParB-nuclease	GCF_002154725.1	532	Bacteria>Terrabacteria_group>Cyanobacteria/Melainabacteria_group>Cyanobacteria>Nostocales	Nostoc_sp._106C	DGQHR-domain-containing-protein-[Nostoc-sp.-106C]	1932667
OUL33915.1	DndB-ParB-nuclease	GCA_002154725.1	532	Bacteria>Terrabacteria_group>Cyanobacteria/Melainabacteria_group>Cyanobacteria>Nostocales	Nostoc_sp._106C	hypothetical-protein-BV375_05895-[Nostoc-sp.-106C]	1932667
MBC1237749.1	DndB-ParB-nuclease	GCA_014222165.1	532	Bacteria>Terrabacteria_group>Cyanobacteria/Melainabacteria_group>Cyanobacteria>Nostocales	Nostoc_sp._2RC	DGQHR-domain-containing-protein-[Nostoc-sp.-2RC]	2485484
WP_185565287.1	DndB-ParB-nuclease	GCF_014222165.1	532	Bacteria>Terrabacteria_group>Cyanobacteria/Melainabacteria_group>Cyanobacteria>Nostocales	Nostoc_sp._2RC	DGQHR-domain-containing-protein-[Nostoc-sp.-2RC]	2485484
RCJ24980.1	DndB-ParB-nuclease	GCA_003326205.1	396	Bacteria>Terrabacteria_group>Cyanobacteria/Melainabacteria_group>Cyanobacteria>Nostocales	Nostoc_sp._ATCC_43529	hypothetical-protein-A6S26_17420-[Nostoc-sp.-ATCC-43529]	1840705
RCJ24981.1	DndB-ParB-nuclease	GCA_003326205.1	533	Bacteria>Terrabacteria_group>Cyanobacteria/Melainabacteria_group>Cyanobacteria>Nostocales	Nostoc_sp._ATCC_43529	hypothetical-protein-A6S26_17425-[Nostoc-sp.-ATCC-43529]	1840705
QHG18286.1	DndB-ParB-nuclease	GCA_009873495.1	532	Bacteria>Terrabacteria_group>Cyanobacteria/Melainabacteria_group>Cyanobacteria>Nostocales	Nostoc_sp._ATCC_53789	DGQHR-domain-containing-protein-[Nostoc-sp.-ATCC-53789]	76335
WP_114082719.1	DndB-ParB-nuclease	GCF_009873495.1	532	Bacteria>Terrabacteria_group>Cyanobacteria/Melainabacteria_group>Cyanobacteria>Nostocales	Nostoc_sp._ATCC_53789	DGQHR-domain-containing-protein-[Nostoc-sp.-ATCC-53789]	76335
RCJ26700.1	DndB-ParB-nuclease	GCA_003326245.1	532	Bacteria>Terrabacteria_group>Cyanobacteria/Melainabacteria_group>Cyanobacteria>Nostocales	Nostoc_sp._ATCC_53789	hypothetical-protein-A6V25_19385-[Nostoc-sp.-ATCC-53789]	76335
NDJ22560.1	DndB-ParB-nuclease	GCA_010091925.1	533	Bacteria>Terrabacteria_group>Cyanobacteria/Melainabacteria_group>Cyanobacteria>Nostocales	Nostoc_sp._B(2019)	DGQHR-domain-containing-protein-[Nostoc-sp.-B(2019)]	2690446
WP_162396449.1	DndB-ParB-nuclease	GCF_010091925.1	533	Bacteria>Terrabacteria_group>Cyanobacteria/Melainabacteria_group>Cyanobacteria>Nostocales	Nostoc_sp._B(2019)	DGQHR-domain-containing-protein-[Nostoc-sp.-B(2019)]	2690446
WP_162396450.1	DndB-ParB-nuclease	GCF_010091925.1	396	Bacteria>Terrabacteria_group>Cyanobacteria/Melainabacteria_group>Cyanobacteria>Nostocales	Nostoc_sp._B(2019)	DNA-sulfur-modification-protein-DndB-[Nostoc-sp.-B(2019)]	2690446
NDJ22561.1	DndB-ParB-nuclease	GCA_010091925.1	396	Bacteria>Terrabacteria_group>Cyanobacteria/Melainabacteria_group>Cyanobacteria>Nostocales	Nostoc_sp._B(2019)	hypothetical-protein-[Nostoc-sp.-B(2019)]	2690446
QLE42304.1	DndB-ParB-nuclease	GCA_013393905.1	532	Bacteria>Terrabacteria_group>Cyanobacteria/Melainabacteria_group>Cyanobacteria>Nostocales	Nostoc_sp._C052	DGQHR-domain-containing-protein-[Nostoc-sp.-C052]	2576902
WP_179066735.1	DndB-ParB-nuclease	GCF_013393905.1	532	Bacteria>Terrabacteria_group>Cyanobacteria/Melainabacteria_group>Cyanobacteria>Nostocales	Nostoc_sp._C052	DGQHR-domain-containing-protein-[Nostoc-sp.-C052]	2576902
QLE51040.1	DndB-ParB-nuclease	GCA_013393925.1	532	Bacteria>Terrabacteria_group>Cyanobacteria/Melainabacteria_group>Cyanobacteria>Nostocales	Nostoc_sp._C057	DGQHR-domain-containing-protein-[Nostoc-sp.-C057]	2576903
WP_179074459.1	DndB-ParB-nuclease	GCF_013393925.1	532	Bacteria>Terrabacteria_group>Cyanobacteria/Melainabacteria_group>Cyanobacteria>Nostocales	Nostoc_sp._C057	DGQHR-domain-containing-protein-[Nostoc-sp.-C057]	2576903
MBD0387459.1	DndB-ParB-nuclease	GCA_014534555.1	391	Bacteria>Terrabacteria_group>Cyanobacteria/Melainabacteria_group>Cyanobacteria>Nostocales	Nostoc_sp._C3-bin3	DNA-sulfur-modification-protein-DndB-[Nostoc-sp.-C3-bin3]	2769069
WP_103136924.1	DndB-ParB-nuclease	GCF_002896875.1	532	Bacteria>Terrabacteria_group>Cyanobacteria/Melainabacteria_group>Cyanobacteria>Nostocales	Nostoc_sp._CENA543	DGQHR-domain-containing-protein-[Nostoc-sp.-CENA543]	1869241
AUT00531.1	DndB-ParB-nuclease	GCA_002896875.1	532	Bacteria>Terrabacteria_group>Cyanobacteria/Melainabacteria_group>Cyanobacteria>Nostocales	Nostoc_sp._CENA543	hypothetical-protein-CLI64_09090-[Nostoc-sp.-CENA543]	1869241
WP_229453936.1	DndB-ParB-nuclease	GCF_020829685.1	531	Bacteria>Terrabacteria_group>Cyanobacteria/Melainabacteria_group>Cyanobacteria>Nostocales	Nostoc_sp._CHAB_5715	DGQHR-domain-containing-protein-[Nostoc-sp.-CHAB-5715]	2780400
MCC5620242.1	DndB-ParB-nuclease	GCA_020829685.1	369	Bacteria>Terrabacteria_group>Cyanobacteria/Melainabacteria_group>Cyanobacteria>Nostocales	Nostoc_sp._CHAB_5715	DNA-sulfur-modification-protein-DndB-[Nostoc-sp.-CHAB-5715]	2780400
WP_229451345.1	DndB-ParB-nuclease	GCF_020829685.1	369	Bacteria>Terrabacteria_group>Cyanobacteria/Melainabacteria_group>Cyanobacteria>Nostocales	Nostoc_sp._CHAB_5715	DNA-sulfur-modification-protein-DndB-[Nostoc-sp.-CHAB-5715]	2780400
WP_229453935.1	DndB-ParB-nuclease	GCF_020829685.1	553	Bacteria>Terrabacteria_group>Cyanobacteria/Melainabacteria_group>Cyanobacteria>Nostocales	Nostoc_sp._CHAB_5715	DNA-sulfur-modification-protein-DndB-[Nostoc-sp.-CHAB-5715]	2780400
MCC5646777.1	DndB-ParB-nuclease	GCA_020829665.1	532	Bacteria>Terrabacteria_group>Cyanobacteria/Melainabacteria_group>Cyanobacteria>Nostocales	Nostoc_sp._CHAB_5824	DGQHR-domain-containing-protein-[Nostoc-sp.-CHAB-5824]	2780402
WP_229465346.1	DndB-ParB-nuclease	GCF_020829665.1	532	Bacteria>Terrabacteria_group>Cyanobacteria/Melainabacteria_group>Cyanobacteria>Nostocales	Nostoc_sp._CHAB_5824	DGQHR-domain-containing-protein-[Nostoc-sp.-CHAB-5824]	2780402
MCC5605808.1	DndB-ParB-nuclease	GCA_020829485.1	532	Bacteria>Terrabacteria_group>Cyanobacteria/Melainabacteria_group>Cyanobacteria>Nostocales	Nostoc_sp._CHAB_5834	DGQHR-domain-containing-protein-[Nostoc-sp.-CHAB-5834]	2780403
WP_229468242.1	DndB-ParB-nuclease	-	532	Bacteria>Terrabacteria_group>Cyanobacteria/Melainabacteria_group>Cyanobacteria>Nostocales	Nostoc_sp._CHAB_5834	DGQHR-domain-containing-protein-[Nostoc-sp.-CHAB-5834]	2780403
MCC5605810.1	DndB-ParB-nuclease	GCA_020829485.1	665	Bacteria>Terrabacteria_group>Cyanobacteria/Melainabacteria_group>Cyanobacteria>Nostocales	Nostoc_sp._CHAB_5834	hypothetical-protein-LC612_03115-[Nostoc-sp.-CHAB-5834]	2780403
WP_229468243.1	DndB-ParB-nuclease	-	665	Bacteria>Terrabacteria_group>Cyanobacteria/Melainabacteria_group>Cyanobacteria>Nostocales	Nostoc_sp._CHAB_5834	hypothetical-protein-[Nostoc-sp.-CHAB-5834]	2780403
MCC5615560.1	DndB-ParB-nuclease	GCA_020829445.1	532	Bacteria>Terrabacteria_group>Cyanobacteria/Melainabacteria_group>Cyanobacteria>Nostocales	Nostoc_sp._CHAB_5836	DGQHR-domain-containing-protein-[Nostoc-sp.-CHAB-5836]	2780404
WP_229547540.1	DndB-ParB-nuclease	GCF_020829445.1	532	Bacteria>Terrabacteria_group>Cyanobacteria/Melainabacteria_group>Cyanobacteria>Nostocales	Nostoc_sp._CHAB_5836	DGQHR-domain-containing-protein-[Nostoc-sp.-CHAB-5836]	2780404
MCC5640693.1	DndB-ParB-nuclease	GCA_020829595.1	532	Bacteria>Terrabacteria_group>Cyanobacteria/Melainabacteria_group>Cyanobacteria>Nostocales	Nostoc_sp._CHAB_5844	DGQHR-domain-containing-protein-[Nostoc-sp.-CHAB-5844]	2780405
WP_229544890.1	DndB-ParB-nuclease	GCF_020829595.1	532	Bacteria>Terrabacteria_group>Cyanobacteria/Melainabacteria_group>Cyanobacteria>Nostocales	Nostoc_sp._CHAB_5844	DGQHR-domain-containing-protein-[Nostoc-sp.-CHAB-5844]	2780405
MCC5640692.1	DndB-ParB-nuclease	GCA_020829595.1	554	Bacteria>Terrabacteria_group>Cyanobacteria/Melainabacteria_group>Cyanobacteria>Nostocales	Nostoc_sp._CHAB_5844	DNA-sulfur-modification-protein-DndB-[Nostoc-sp.-CHAB-5844]	2780405
WP_229544889.1	DndB-ParB-nuclease	GCF_020829595.1	554	Bacteria>Terrabacteria_group>Cyanobacteria/Melainabacteria_group>Cyanobacteria>Nostocales	Nostoc_sp._CHAB_5844	DNA-sulfur-modification-protein-DndB-[Nostoc-sp.-CHAB-5844]	2780405
WP_236143462.1	DndB-ParB-nuclease	GCF_021596505.1	532	Bacteria>Terrabacteria_group>Cyanobacteria/Melainabacteria_group>Cyanobacteria>Nostocales	Nostoc_sp._CMAA1605	DGQHR-domain-containing-protein-[Nostoc-sp.-CMAA1605]	2055159
WP_236143463.1	DndB-ParB-nuclease	GCF_021596505.1	348	Bacteria>Terrabacteria_group>Cyanobacteria/Melainabacteria_group>Cyanobacteria>Nostocales	Nostoc_sp._CMAA1605	DNA-sulfur-modification-protein-DndB-[Nostoc-sp.-CMAA1605]	2055159
WP_190684946.1	DndB-ParB-nuclease	GCF_014697425.1	532	Bacteria>Terrabacteria_group>Cyanobacteria/Melainabacteria_group>Cyanobacteria>Nostocales	Nostoc_sp._FACHB-110	DGQHR-domain-containing-protein-[Nostoc-sp.-FACHB-110]	2692834
MBD2524976.1	DndB-ParB-nuclease	GCA_014698055.1	532	Bacteria>Terrabacteria_group>Cyanobacteria/Melainabacteria_group>Cyanobacteria>Nostocales	Nostoc_sp._FACHB-133	DGQHR-domain-containing-protein-[Nostoc-sp.-FACHB-133]	2692835
WP_190936016.1	DndB-ParB-nuclease	GCF_014698055.1	532	Bacteria>Terrabacteria_group>Cyanobacteria/Melainabacteria_group>Cyanobacteria>Nostocales	Nostoc_sp._FACHB-133	DGQHR-domain-containing-protein-[Nostoc-sp.-FACHB-133]	2692835
MBD2466782.1	DndB-ParB-nuclease	GCA_014697455.1	532	Bacteria>Terrabacteria_group>Cyanobacteria/Melainabacteria_group>Cyanobacteria>Nostocales	Nostoc_sp._FACHB-145	DGQHR-domain-containing-protein-[Nostoc-sp.-FACHB-145]	2692836
MBD2466781.1	DndB-ParB-nuclease	GCA_014697455.1	310	Bacteria>Terrabacteria_group>Cyanobacteria/Melainabacteria_group>Cyanobacteria>Nostocales	Nostoc_sp._FACHB-145	DNA-sulfur-modification-protein-DndB-[Nostoc-sp.-FACHB-145]	2692836

Accession	Annotation	GCA	Length	Phyletic.spread	Strain.name	Defline	Species.Taxid
MBD2445668.1	DndB-ParB-nuclease	GCA_014697475.1	532	Bacteria>Terrabacteria_group>Cyanobacteria/Melainabacteria_group>Cyanobacteria>Nostocales	Nostoc_sp._FACHB-152	DGQHR-domain-containing-protein-[Nostoc-sp.-FACHB-152]	2692837
MBD2445667.1	DndB-ParB-nuclease	GCA_014697475.1	310	Bacteria>Terrabacteria_group>Cyanobacteria/Melainabacteria_group>Cyanobacteria>Nostocales	Nostoc_sp._FACHB-152	DNA-sulfur-modification-protein-DndB-[Nostoc-sp.-FACHB-152]	2692837
MBD2300109.1	DndB-ParB-nuclease	GCA_014696905.1	532	Bacteria>Terrabacteria_group>Cyanobacteria/Melainabacteria_group>Cyanobacteria>Nostocales	Nostoc_sp._FACHB-190	DGQHR-domain-containing-protein-[Nostoc-sp.-FACHB-190]	2692838
WP_190672195.1	DndB-ParB-nuclease	GCF_014696905.1	532	Bacteria>Terrabacteria_group>Cyanobacteria/Melainabacteria_group>Cyanobacteria>Nostocales	Nostoc_sp._FACHB-190	DGQHR-domain-containing-protein-[Nostoc-sp.-FACHB-190]	2692838
MBD2300108.1	DndB-ParB-nuclease	GCA_014696905.1	396	Bacteria>Terrabacteria_group>Cyanobacteria/Melainabacteria_group>Cyanobacteria>Nostocales	Nostoc_sp._FACHB-190	DNA-sulfur-modification-protein-DndB-[Nostoc-sp.-FACHB-190]	2692838
WP_190672192.1	DndB-ParB-nuclease	GCF_014696905.1	396	Bacteria>Terrabacteria_group>Cyanobacteria/Melainabacteria_group>Cyanobacteria>Nostocales	Nostoc_sp._FACHB-190	DNA-sulfur-modification-protein-DndB-[Nostoc-sp.-FACHB-190]	2692838
MBD2493708.1	DndB-ParB-nuclease	GCA_014697615.1	532	Bacteria>Terrabacteria_group>Cyanobacteria/Melainabacteria_group>Cyanobacteria>Nostocales	Nostoc_sp._FACHB-280	DGQHR-domain-containing-protein-[Nostoc-sp.-FACHB-280]	2692839
WP_190978888.1	DndB-ParB-nuclease	GCF_014697615.1	532	Bacteria>Terrabacteria_group>Cyanobacteria/Melainabacteria_group>Cyanobacteria>Nostocales	Nostoc_sp._FACHB-280	DGQHR-domain-containing-protein-[Nostoc-sp.-FACHB-280]	2692839
MBD2493707.1	DndB-ParB-nuclease	GCA_014697615.1	396	Bacteria>Terrabacteria_group>Cyanobacteria/Melainabacteria_group>Cyanobacteria>Nostocales	Nostoc_sp._FACHB-280	DNA-sulfur-modification-protein-DndB-[Nostoc-sp.-FACHB-280]	2692839
WP_190978887.1	DndB-ParB-nuclease	GCF_014697615.1	396	Bacteria>Terrabacteria_group>Cyanobacteria/Melainabacteria_group>Cyanobacteria>Nostocales	Nostoc_sp._FACHB-280	DNA-sulfur-modification-protein-DndB-[Nostoc-sp.-FACHB-280]	2692839
MBD2677727.1	DndB-ParB-nuclease	GCA_014698635.1	532	Bacteria>Terrabacteria_group>Cyanobacteria/Melainabacteria_group>Cyanobacteria>Nostocales	Nostoc_sp._FACHB-857	DGQHR-domain-containing-protein-[Nostoc-sp.-FACHB-857]	2692840
MBD2455584.1	DndB-ParB-nuclease	GCA_014697495.1	532	Bacteria>Terrabacteria_group>Cyanobacteria/Melainabacteria_group>Cyanobacteria>Nostocales	Nostoc_sp._FACHB-87	DGQHR-domain-containing-protein-[Nostoc-sp.-FACHB-87]	2692841
MBD2243243.1	DndB-ParB-nuclease	GCA_014696655.1	532	Bacteria>Terrabacteria_group>Cyanobacteria/Melainabacteria_group>Cyanobacteria>Nostocales	Nostoc_sp._FACHB-888	DGQHR-domain-containing-protein-[Nostoc-sp.-FACHB-888]	2692842
WP_190728428.1	DndB-ParB-nuclease	GCF_014696655.1	532	Bacteria>Terrabacteria_group>Cyanobacteria/Melainabacteria_group>Cyanobacteria>Nostocales	Nostoc_sp._FACHB-888	DGQHR-domain-containing-protein-[Nostoc-sp.-FACHB-888]	2692842
MBD2725479.1	DndB-ParB-nuclease	GCA_014698795.1	532	Bacteria>Terrabacteria_group>Cyanobacteria/Melainabacteria_group>Cyanobacteria>Nostocales	Nostoc_sp._FACHB-892	DGQHR-domain-containing-protein-[Nostoc-sp.-FACHB-892]	2692843
WP_190910231.1	DndB-ParB-nuclease	GCF_014698795.1	532	Bacteria>Terrabacteria_group>Cyanobacteria/Melainabacteria_group>Cyanobacteria>Nostocales	Nostoc_sp._FACHB-892	DGQHR-domain-containing-protein-[Nostoc-sp.-FACHB-892]	2692843
MBD2725481.1	DndB-ParB-nuclease	GCA_014698795.1	577	Bacteria>Terrabacteria_group>Cyanobacteria/Melainabacteria_group>Cyanobacteria>Nostocales	Nostoc_sp._FACHB-892	hypothetical-protein-[Nostoc-sp.-FACHB-892]	2692843
WP_190910233.1	DndB-ParB-nuclease	GCF_014698795.1	577	Bacteria>Terrabacteria_group>Cyanobacteria/Melainabacteria_group>Cyanobacteria>Nostocales	Nostoc_sp._FACHB-892	hypothetical-protein-[Nostoc-sp.-FACHB-892]	2692843
MBD2519010.1	DndB-ParB-nuclease	GCA_014698115.1	532	Bacteria>Terrabacteria_group>Cyanobacteria/Melainabacteria_group>Cyanobacteria>Nostocales	Nostoc_sp._FACHB-973	DGQHR-domain-containing-protein-[Nostoc-sp.-FACHB-973]	2692844
WP_190882965.1	DndB-ParB-nuclease	GCF_014698115.1	532	Bacteria>Terrabacteria_group>Cyanobacteria/Melainabacteria_group>Cyanobacteria>Nostocales	Nostoc_sp._FACHB-973	DGQHR-domain-containing-protein-[Nostoc-sp.-FACHB-973]	2692844
MBL1198049.1	DndB-ParB-nuclease	GCA_016743225.1	532	Bacteria>Terrabacteria_group>Cyanobacteria/Melainabacteria_group>Cyanobacteria>Nostocales	Nostoc_sp._GBBB01	DGQHR-domain-containing-protein-[Nostoc-sp.-GBBB01]	2604138
QFS45413.1	DndB-ParB-nuclease	GCA_009372195.1	532	Bacteria>Terrabacteria_group>Cyanobacteria/Melainabacteria_group>Cyanobacteria>Nostocales	Nostoc_sphaeroides_CCNUC1	DGQHR-domain-containing-protein-[Nostoc-sphaeroides-CCNUC1]	2653204
WP_118169239.1	DndB-ParB-nuclease	GCF_003443655.1	532	Bacteria>Terrabacteria_group>Cyanobacteria/Melainabacteria_group>Cyanobacteria>Nostocales	Nostoc_sphaeroides	DGQHR-domain-containing-protein-[Nostoc-sphaeroides]	446679
WP_152589055.1	DndB-ParB-nuclease	GCF_009372195.1	532	Bacteria>Terrabacteria_group>Cyanobacteria/Melainabacteria_group>Cyanobacteria>Nostocales	Nostoc_sphaeroides	DGQHR-domain-containing-protein-[Nostoc-sphaeroides]	446679
MBC6433132.1	DndB-ParB-nuclease	GCA_014324315.1	532	Bacteria>Terrabacteria_group>Cyanobacteria/Melainabacteria_group>Cyanobacteria>Nostocales	Nostoc_sp._HG1	DGQHR-domain-containing-protein-[Nostoc-sp.-HG1]	2593659
BBD60336.1	DndB-ParB-nuclease	GCA_003990705.1	532	Bacteria>Terrabacteria_group>Cyanobacteria/Melainabacteria_group>Cyanobacteria>Nostocales	Nostoc_sp._HK-01	hypothetical-protein-NIES2109_31330-[Nostoc-sp.-HK-01]	196308
MBN3878032.1	DndB-ParB-nuclease	GCA_017115065.1	532	Bacteria>Terrabacteria_group>Cyanobacteria/Melainabacteria_group>Cyanobacteria>Nostocales	Nostoc_sp._JL23	DGQHR-domain-containing-protein-[Nostoc-sp.-JL23]	2815394
MBN3878034.1	DndB-ParB-nuclease	GCA_017115065.1	576	Bacteria>Terrabacteria_group>Cyanobacteria/Melainabacteria_group>Cyanobacteria>Nostocales	Nostoc_sp._JL23	hypothetical-protein-[Nostoc-sp.-JL23]	2815394
MBN3890980.1	DndB-ParB-nuclease	GCA_017114965.1	533	Bacteria>Terrabacteria_group>Cyanobacteria/Melainabacteria_group>Cyanobacteria>Nostocales	Nostoc_sp._JL31	DGQHR-domain-containing-protein-[Nostoc-sp.-JL31]	2815395
MBN3890979.1	DndB-ParB-nuclease	GCA_017114965.1	385	Bacteria>Terrabacteria_group>Cyanobacteria/Melainabacteria_group>Cyanobacteria>Nostocales	Nostoc_sp._JL31	hypothetical-protein-[Nostoc-sp.-JL31]	2815395
MBN3887330.1	DndB-ParB-nuclease	GCA_017114945.1	531	Bacteria>Terrabacteria_group>Cyanobacteria/Melainabacteria_group>Cyanobacteria>Nostocales	Nostoc_sp._JL34	DGQHR-domain-containing-protein-[Nostoc-sp.-JL34]	2815397
MBN3887332.1	DndB-ParB-nuclease	GCA_017114945.1	401	Bacteria>Terrabacteria_group>Cyanobacteria/Melainabacteria_group>Cyanobacteria>Nostocales	Nostoc_sp._JL34	DGQHR-domain-containing-protein-[Nostoc-sp.-JL34]	2815397
WP_069068984.1	DndB-ParB-nuclease	GCF_001712795.1	532	Bacteria>Terrabacteria_group>Cyanobacteria/Melainabacteria_group>Cyanobacteria>Nostocales	Nostoc_sp._KVJ20	DGQHR-domain-containing-protein-[Nostoc-sp.-KVJ20]	457944
ODG98960.1	DndB-ParB-nuclease	GCA_001712795.1	532	Bacteria>Terrabacteria_group>Cyanobacteria/Melainabacteria_group>Cyanobacteria>Nostocales	Nostoc_sp._KVJ20	hypothetical-protein-A4S05_06360-[Nostoc-sp.-KVJ20]	457944
WP_193889805.1	DndB-ParB-nuclease	GCF_015207705.1	532	Bacteria>Terrabacteria_group>Cyanobacteria/Melainabacteria_group>Cyanobacteria>Nostocales	Nostoc_sp._LEGE_06077	DGQHR-domain-containing-protein-[Nostoc-sp.-LEGE-06077]	915325
WP_193889803.1	DndB-ParB-nuclease	GCF_015207705.1	665	Bacteria>Terrabacteria_group>Cyanobacteria/Melainabacteria_group>Cyanobacteria>Nostocales	Nostoc_sp._LEGE_06077	hypothetical-protein-[Nostoc-sp.-LEGE-06077]	915325
MBE9000391.1	DndB-ParB-nuclease	GCA_015206985.1	532	Bacteria>Terrabacteria_group>Cyanobacteria/Melainabacteria_group>Cyanobacteria>Nostocales	Nostoc_sp._LEGE_12447	DGQHR-domain-containing-protein-[Nostoc-sp.-LEGE-12447]	1828640
WP_194143183.1	DndB-ParB-nuclease	GCF_015206985.1	532	Bacteria>Terrabacteria_group>Cyanobacteria/Melainabacteria_group>Cyanobacteria>Nostocales	Nostoc_sp._LEGE_12447	DGQHR-domain-containing-protein-[Nostoc-sp.-LEGE-12447]	1828640
MBE8989711.1	DndB-ParB-nuclease	GCA_015206895.1	532	Bacteria>Terrabacteria_group>Cyanobacteria/Melainabacteria_group>Cyanobacteria>Nostocales	Nostoc_sp._LEGE_12450	DGQHR-domain-containing-protein-[Nostoc-sp.-LEGE-12450]	1828643
WP_193948409.1	DndB-ParB-nuclease	GCF_015206895.1	532	Bacteria>Terrabacteria_group>Cyanobacteria/Melainabacteria_group>Cyanobacteria>Nostocales	Nostoc_sp._LEGE_12450	DGQHR-domain-containing-protein-[Nostoc-sp.-LEGE-12450]	1828643
MBE8989713.1	DndB-ParB-nuclease	GCA_015206895.1	665	Bacteria>Terrabacteria_group>Cyanobacteria/Melainabacteria_group>Cyanobacteria>Nostocales	Nostoc_sp._LEGE_12450	hypothetical-protein-[Nostoc-sp.-LEGE-12450]	1828643
WP_193948411.1	DndB-ParB-nuclease	GCF_015206895.1	665	Bacteria>Terrabacteria_group>Cyanobacteria/Melainabacteria_group>Cyanobacteria>Nostocales	Nostoc_sp._LEGE_12450	hypothetical-protein-[Nostoc-sp.-LEGE-12450]	1828643
WP_104906130.1	DndB-ParB-nuclease	GCF_002949795.1	532	Bacteria>Terrabacteria_group>Cyanobacteria/Melainabacteria_group>Cyanobacteria>Nostocales	Nostoc_sp._'Lobaria_pulmonaria_(5183)_cyanobiont'	DGQHR-domain-containing-protein-[Nostoc-sp.-'Lobaria-pulmonaria-(5183)-cyanobiont']	1618022
AVH70541.1	DndB-ParB-nuclease	GCA_002949795.1	532	Bacteria>Terrabacteria_group>Cyanobacteria/Melainabacteria_group>Cyanobacteria>Nostocales	Nostoc_sp._'Lobaria_pulmonaria_(5183)_cyanobiont'	DNA-sulfur-modification-protein-DndB-[Nostoc-sp.-'Lobaria-pulmonaria-(5183)-cyanobiont']	1618022
MBN4003519.1	DndB-ParB-nuclease	GCA_017115165.1	532	Bacteria>Terrabacteria_group>Cyanobacteria/Melainabacteria_group>Cyanobacteria>Nostocales	Nostoc_sp._LPT	DGQHR-domain-containing-protein-[Nostoc-sp.-LPT]	2815387
OCQ97794.1	DndB-ParB-nuclease	GCA_001698435.1	396	Bacteria>Terrabacteria_group>Cyanobacteria/Melainabacteria_group>Cyanobacteria>Nostocales	Nostoc_sp._MBR_210	hypothetical-protein-BCD64_06765-[Nostoc-sp.-MBR-210]	1881015
OCQ97795.1	DndB-ParB-nuclease	GCA_001698435.1	532	Bacteria>Terrabacteria_group>Cyanobacteria/Melainabacteria_group>Cyanobacteria>Nostocales	Nostoc_sp._MBR_210	hypothetical-protein-BCD64_06770-[Nostoc-sp.-MBR-210]	1881015
WP_193197690.1	DndB-ParB-nuclease	GCF_014904695.1	532	Bacteria>Terrabacteria_group>Cyanobacteria/Melainabacteria_group>Cyanobacteria>Nostocales	Nostoc_sp._MG11	DGQHR-domain-containing-protein-[Nostoc-sp.-MG11]	2721166
WP_224094536.1	DndB-ParB-nuclease	GCF_019976755.1	532	Bacteria>Terrabacteria_group>Cyanobacteria/Melainabacteria_group>Cyanobacteria>Nostocales	Nostoc_sp._MS1	DGQHR-domain-containing-protein-[Nostoc-sp.-MS1]	2764711
BCL36399.1	DndB-ParB-nuclease	GCA_019976755.1	532	Bacteria>Terrabacteria_group>Cyanobacteria/Melainabacteria_group>Cyanobacteria>Nostocales	Nostoc_sp._MS1	hypothetical-protein-NSMS1_28460-[Nostoc-sp.-MS1]	2764711
WP_096680742.1	DndB-ParB-nuclease	GCF_002368215.1	532	Bacteria>Terrabacteria_group>Cyanobacteria/Melainabacteria_group>Cyanobacteria>Nostocales	Nostoc_sp._NIES-2111	DGQHR-domain-containing-protein-[Nostoc-sp.-NIES-2111]	1973475
BAY38174.1	DndB-ParB-nuclease	GCA_002368215.1	532	Bacteria>Terrabacteria_group>Cyanobacteria/Melainabacteria_group>Cyanobacteria>Nostocales	Nostoc_sp._NIES-2111	hypothetical-protein-NIES2111_25190-[Nostoc-sp.-NIES-2111]	1973475
WP_067769689.1	DndB-ParB-nuclease	GCF_001548375.1	388	Bacteria>Terrabacteria_group>Cyanobacteria/Melainabacteria_group>Cyanobacteria>Nostocales	Nostoc_sp._NIES-3756	DGQHR-domain-containing-protein-[Nostoc-sp.-NIES-3756]	1751286
WP_067769693.1	DndB-ParB-nuclease	GCF_001548375.1	533	Bacteria>Terrabacteria_group>Cyanobacteria/Melainabacteria_group>Cyanobacteria>Nostocales	Nostoc_sp._NIES-3756	DGQHR-domain-containing-protein-[Nostoc-sp.-NIES-3756]	1751286
BAT54085.1	DndB-ParB-nuclease	GCA_001548375.1	388	Bacteria>Terrabacteria_group>Cyanobacteria/Melainabacteria_group>Cyanobacteria>Nostocales	Nostoc_sp._NIES-3756	hypothetical-protein-NOS3756_30500-[Nostoc-sp.-NIES-3756]	1751286
BAT54087.1	DndB-ParB-nuclease	GCA_001548375.1	533	Bacteria>Terrabacteria_group>Cyanobacteria/Melainabacteria_group>Cyanobacteria>Nostocales	Nostoc_sp._NIES-3756	unknown-protein-[Nostoc-sp.-NIES-3756]	1751286
WP_096555927.1	DndB-ParB-nuclease	GCF_002368335.1	532	Bacteria>Terrabacteria_group>Cyanobacteria/Melainabacteria_group>Cyanobacteria>Nostocales	Nostoc_sp._NIES-4103	DGQHR-domain-containing-protein-[Nostoc-sp.-NIES-4103]	2005458
BAZ50837.1	DndB-ParB-nuclease	GCA_002368335.1	394	Bacteria>Terrabacteria_group>Cyanobacteria/Melainabacteria_group>Cyanobacteria>Nostocales	Nostoc_sp._NIES-4103	DGQHR-domain-protein-[Nostoc-sp.-NIES-4103]	2005458
WP_096555925.1	DndB-ParB-nuclease	GCF_002368335.1	394	Bacteria>Terrabacteria_group>Cyanobacteria/Melainabacteria_group>Cyanobacteria>Nostocales	Nostoc_sp._NIES-4103	DNA-sulfur-modification-protein-DndB-[Nostoc-sp.-NIES-4103]	2005458
BAZ50838.1	DndB-ParB-nuclease	GCA_002368335.1	532	Bacteria>Terrabacteria_group>Cyanobacteria/Melainabacteria_group>Cyanobacteria>Nostocales	Nostoc_sp._NIES-4103	hypothetical-protein-NIES4103_34560-[Nostoc-sp.-NIES-4103]	2005458
MBN3908414.1	DndB-ParB-nuclease	GCA_017115145.1	532	Bacteria>Terrabacteria_group>Cyanobacteria/Melainabacteria_group>Cyanobacteria>Nostocales	Nostoc_sp._NMS1	DGQHR-domain-containing-protein-[Nostoc-sp.-NMS1]	2815388
MBN3908412.1	DndB-ParB-nuclease	GCA_017115145.1	665	Bacteria>Terrabacteria_group>Cyanobacteria/Melainabacteria_group>Cyanobacteria>Nostocales	Nostoc_sp._NMS1	hypothetical-protein-[Nostoc-sp.-NMS1]	2815388
MBN3993676.1	DndB-ParB-nuclease	GCA_017115105.1	532	Bacteria>Terrabacteria_group>Cyanobacteria/Melainabacteria_group>Cyanobacteria>Nostocales	Nostoc_sp._NMS2	DGQHR-domain-containing-protein-[Nostoc-sp.-NMS2]	2815389
MBN3993674.1	DndB-ParB-nuclease	GCA_017115105.1	665	Bacteria>Terrabacteria_group>Cyanobacteria/Melainabacteria_group>Cyanobacteria>Nostocales	Nostoc_sp._NMS2	hypothetical-protein-[Nostoc-sp.-NMS2]	2815389
MBN3927232.1	DndB-ParB-nuclease	GCA_017115095.1	532	Bacteria>Terrabacteria_group>Cyanobacteria/Melainabacteria_group>Cyanobacteria>Nostocales	Nostoc_sp._NMS4	DGQHR-domain-containing-protein-[Nostoc-sp.-NMS4]	2815390
MBN3949438.1	DndB-ParB-nuclease	GCA_017115085.1	532	Bacteria>Terrabacteria_group>Cyanobacteria/Melainabacteria_group>Cyanobacteria>Nostocales	Nostoc_sp._NMS7	DGQHR-domain-containing-protein-[Nostoc-sp.-NMS7]	2815391
MBN3960699.1	DndB-ParB-nuclease	GCA_017115045.1	533	Bacteria>Terrabacteria_group>Cyanobacteria/Melainabacteria_group>Cyanobacteria>Nostocales	Nostoc_sp._NMS8	DGQHR-domain-containing-protein-[Nostoc-sp.-NMS8]	2815392
MBN3960700.1	DndB-ParB-nuclease	GCA_017115045.1	396	Bacteria>Terrabacteria_group>Cyanobacteria/Melainabacteria_group>Cyanobacteria>Nostocales	Nostoc_sp._NMS8	hypothetical-protein-[Nostoc-sp.-NMS8]	2815392
MBN3940321.1	DndB-ParB-nuclease	GCA_017114995.1	532	Bacteria>Terrabacteria_group>Cyanobacteria/Melainabacteria_group>Cyanobacteria>Nostocales	Nostoc_sp._NMS9	DGQHR-domain-containing-protein-[Nostoc-sp.-NMS9]	2815393
MBN3940320.1	DndB-ParB-nuclease	GCA_017114995.1	248	Bacteria>Terrabacteria_group>Cyanobacteria/Melainabacteria_group>Cyanobacteria>Nostocales	Nostoc_sp._NMS9	hypothetical-protein-[Nostoc-sp.-NMS9]	2815393
MBN3897751.1	DndB-ParB-nuclease	GCA_017115025.1	533	Bacteria>Terrabacteria_group>Cyanobacteria/Melainabacteria_group>Cyanobacteria>Nostocales	Nostoc_sp._NOS(2021)	DGQHR-domain-containing-protein-[Nostoc-sp.-NOS(2021)]	2815407
MBN3897750.1	DndB-ParB-nuclease	GCA_017115025.1	381	Bacteria>Terrabacteria_group>Cyanobacteria/Melainabacteria_group>Cyanobacteria>Nostocales	Nostoc_sp._NOS(2021)	hypothetical-protein-[Nostoc-sp.-NOS(2021)]	2815407
MBG1242992.1	DndB-ParB-nuclease	GCA_015714705.1	532	Bacteria>Terrabacteria_group>Cyanobacteria/Melainabacteria_group>Cyanobacteria>Nostocales	Nostoc_sp._NZL	DGQHR-domain-containing-protein-[Nostoc-sp.-NZL]	2650612
WP_196511870.1	DndB-ParB-nuclease	GCF_015714705.1	532	Bacteria>Terrabacteria_group>Cyanobacteria/Melainabacteria_group>Cyanobacteria>Nostocales	Nostoc_sp._NZL	DGQHR-domain-containing-protein-[Nostoc-sp.-NZL]	2650612
WP_190965951.1	DndB-ParB-nuclease	GCF_014698475.1	533	Bacteria>Terrabacteria_group>Cyanobacteria/Melainabacteria_group>Cyanobacteria>Nostocales	Nostoc_spongiaeforme	DGQHR-domain-containing-protein-[Nostoc-spongiaeforme]	502487
WP_190965950.1	DndB-ParB-nuclease	GCF_014698475.1	389	Bacteria>Terrabacteria_group>Cyanobacteria/Melainabacteria_group>Cyanobacteria>Nostocales	Nostoc_spongiaeforme	DNA-sulfur-modification-protein-DndB-[Nostoc-spongiaeforme]	502487
MBD2592986.1	DndB-ParB-nuclease	GCA_014698475.1	533	Bacteria>Terrabacteria_group>Cyanobacteria/Melainabacteria_group>Cyanobacteria>Nostocales	Nostoc_spongiaeforme_FACHB-130	DGQHR-domain-containing-protein-[Nostoc-spongiaeforme-FACHB-130]	1357510
MBD2592985.1	DndB-ParB-nuclease	GCA_014698475.1	389	Bacteria>Terrabacteria_group>Cyanobacteria/Melainabacteria_group>Cyanobacteria>Nostocales	Nostoc_spongiaeforme_FACHB-130	DNA-sulfur-modification-protein-DndB-[Nostoc-spongiaeforme-FACHB-130]	1357510
WP_138505153.1	DndB-ParB-nuclease	GCF_005869855.1	530	Bacteria>Terrabacteria_group>Cyanobacteria/Melainabacteria_group>Cyanobacteria>Nostocales	Nostoc_sp._PA-18-2419	DGQHR-domain-containing-protein-[Nostoc-sp.-PA-18-2419]	2575443
WP_015115987.1	DndB-ParB-nuclease	GCF_000316625.1	532	Bacteria>Terrabacteria_group>Cyanobacteria/Melainabacteria_group>Cyanobacteria>Nostocales	Nostoc_sp._PCC_7107	DGQHR-domain-containing-protein-[Nostoc-sp.-PCC-7107]	317936
AFY45806.1	DndB-ParB-nuclease	GCA_000316625.1	532	Bacteria>Terrabacteria_group>Cyanobacteria/Melainabacteria_group>Cyanobacteria>Nostocales	Nostoc_sp._PCC_7107	DGQHR-domain-protein-[Nostoc-sp.-PCC-7107]	317936
MBD2274293.1	DndB-ParB-nuclease	GCA_014696735.1	532	Bacteria>Terrabacteria_group>Cyanobacteria/Melainabacteria_group>Cyanobacteria>Nostocales	Nostoc_sp._PCC_7120_=_FACHB-418	DGQHR-domain-containing-protein-[Nostoc-sp.-PCC-7120-=-FACHB-418]	103690
AFY48441.1	DndB-ParB-nuclease	GCA_000316645.1	532	Bacteria>Terrabacteria_group>Cyanobacteria/Melainabacteria_group>Cyanobacteria>Nostocales	Nostoc_sp._PCC_7524	DGQHR-domain-containing-protein-[Nostoc-sp.-PCC-7524]	28072
WP_015138879.1	DndB-ParB-nuclease	GCF_000316645.1	532	Bacteria>Terrabacteria_group>Cyanobacteria/Melainabacteria_group>Cyanobacteria>Nostocales	Nostoc_sp._PCC_7524	DGQHR-domain-containing-protein-[Nostoc-sp.-PCC-7524]	28072
PHM07327.1	DndB-ParB-nuclease	GCA_002631755.1	532	Bacteria>Terrabacteria_group>Cyanobacteria/Melainabacteria_group>Cyanobacteria>Nostocales	Nostoc_sp._'Peltigera_malacea_cyanobiont'_DB3992	hypothetical-protein-CK516_27770-[Nostoc-sp.-'Peltigera-malacea-cyanobiont'-DB3992]	1206980
WP_094353035.1	DndB-ParB-nuclease	GCF_002246015.1	532	Bacteria>Terrabacteria_group>Cyanobacteria/Melainabacteria_group>Cyanobacteria>Nostocales	Nostoc_sp._'Peltigera_membranacea_cyanobiont'_210A	DGQHR-domain-containing-protein-[Nostoc-sp.-'Peltigera-membranacea-cyanobiont'-210A]	2014529
OYD90136.1	DndB-ParB-nuclease	GCA_002246015.1	532	Bacteria>Terrabacteria_group>Cyanobacteria/Melainabacteria_group>Cyanobacteria>Nostocales	Nostoc_sp._'Peltigera_membranacea_cyanobiont'_210A	hypothetical-protein-CDG76_32885-[Nostoc-sp.-'Peltigera-membranacea-cyanobiont'-210A]	2014529
WP_094331647.1	DndB-ParB-nuclease	GCF_002245975.1	532	Bacteria>Terrabacteria_group>Cyanobacteria/Melainabacteria_group>Cyanobacteria>Nostocales	Nostoc_sp._'Peltigera_membranacea_cyanobiont'_213	DGQHR-domain-containing-protein-[Nostoc-sp.-'Peltigera-membranacea-cyanobiont'-213]	2014530
OYD88594.1	DndB-ParB-nuclease	GCA_002245975.1	532	Bacteria>Terrabacteria_group>Cyanobacteria/Melainabacteria_group>Cyanobacteria>Nostocales	Nostoc_sp._'Peltigera_membranacea_cyanobiont'_213	hypothetical-protein-CDG77_22805-[Nostoc-sp.-'Peltigera-membranacea-cyanobiont'-213]	2014530
WP_094345026.1	DndB-ParB-nuclease	GCF_002245985.1	533	Bacteria>Terrabacteria_group>Cyanobacteria/Melainabacteria_group>Cyanobacteria>Nostocales	Nostoc_sp._'Peltigera_membranacea_cyanobiont'_232	DGQHR-domain-containing-protein-[Nostoc-sp.-'Peltigera-membranacea-cyanobiont'-232]	2014531
WP_094345027.1	DndB-ParB-nuclease	GCF_002245985.1	401	Bacteria>Terrabacteria_group>Cyanobacteria/Melainabacteria_group>Cyanobacteria>Nostocales	Nostoc_sp._'Peltigera_membranacea_cyanobiont'_232	DNA-sulfur-modification-protein-DndB-[Nostoc-sp.-'Peltigera-membranacea-cyanobiont'-232]	2014531
OYE01750.1	DndB-ParB-nuclease	GCA_002245985.1	533	Bacteria>Terrabacteria_group>Cyanobacteria/Melainabacteria_group>Cyanobacteria>Nostocales	Nostoc_sp._'Peltigera_membranacea_cyanobiont'_232	hypothetical-protein-CDG79_27880-[Nostoc-sp.-'Peltigera-membranacea-cyanobiont'-232]	2014531
OYE01751.1	DndB-ParB-nuclease	GCA_002245985.1	401	Bacteria>Terrabacteria_group>Cyanobacteria/Melainabacteria_group>Cyanobacteria>Nostocales	Nostoc_sp._'Peltigera_membranacea_cyanobiont'_232	hypothetical-protein-CDG79_27885-[Nostoc-sp.-'Peltigera-membranacea-cyanobiont'-232]	2014531
WP_104898961.1	DndB-ParB-nuclease	GCF_002949735.1	532	Bacteria>Terrabacteria_group>Cyanobacteria/Melainabacteria_group>Cyanobacteria>Nostocales	Nostoc_sp._'Peltigera_membranacea_cyanobiont'_N6	DGQHR-domain-containing-protein-[Nostoc-sp.-'Peltigera-membranacea-cyanobiont'-N6]	1261031
AVH62932.1	DndB-ParB-nuclease	GCA_002949735.1	576	Bacteria>Terrabacteria_group>Cyanobacteria/Melainabacteria_group>Cyanobacteria>Nostocales	Nostoc_sp._'Peltigera_membranacea_cyanobiont'_N6	DNA-sulfur-modification-associated-protein-[Nostoc-sp.-'Peltigera-membranacea-cyanobiont'-N6]	1261031
AVH62930.1	DndB-ParB-nuclease	GCA_002949735.1	532	Bacteria>Terrabacteria_group>Cyanobacteria/Melainabacteria_group>Cyanobacteria>Nostocales	Nostoc_sp._'Peltigera_membranacea_cyanobiont'_N6	DNA-sulfur-modification-protein-DndB-[Nostoc-sp.-'Peltigera-membranacea-cyanobiont'-N6]	1261031
WP_104898962.1	DndB-ParB-nuclease	GCF_002949735.1	576	Bacteria>Terrabacteria_group>Cyanobacteria/Melainabacteria_group>Cyanobacteria>Nostocales	Nostoc_sp._'Peltigera_membranacea_cyanobiont'_N6	hypothetical-protein-[Nostoc-sp.-'Peltigera-membranacea-cyanobiont'-N6]	1261031
WP_086835802.1	DndB-ParB-nuclease	GCF_002155185.1	532	Bacteria>Terrabacteria_group>Cyanobacteria/Melainabacteria_group>Cyanobacteria>Nostocales	Nostoc_sp._RF31YmG	DGQHR-domain-containing-protein-[Nostoc-sp.-RF31YmG]	1932668
OUL22934.1	DndB-ParB-nuclease	GCA_002155185.1	532	Bacteria>Terrabacteria_group>Cyanobacteria/Melainabacteria_group>Cyanobacteria>Nostocales	Nostoc_sp._RF31YmG	hypothetical-protein-BV378_23800-[Nostoc-sp.-RF31YmG]	1932668
MBI1240667.1	DndB-ParB-nuclease	-	532	Bacteria>Terrabacteria_group>Cyanobacteria/Melainabacteria_group>Cyanobacteria>Nostocales	Nostoc_sp._RI_552	DGQHR-domain-containing-protein-[Nostoc-sp.-RI_552]	2654185
WP_086690316.1	DndB-ParB-nuclease	GCF_002154695.1	532	Bacteria>Terrabacteria_group>Cyanobacteria/Melainabacteria_group>Cyanobacteria>Nostocales	Nostoc_sp._T09	DGQHR-domain-containing-protein-[Nostoc-sp.-T09]	1932621
WP_086690319.1	DndB-ParB-nuclease	GCF_002154695.1	663	Bacteria>Terrabacteria_group>Cyanobacteria/Melainabacteria_group>Cyanobacteria>Nostocales	Nostoc_sp._T09	hypothetical-protein-[Nostoc-sp.-T09]	1932621
QKQ77305.1	DndB-ParB-nuclease	GCA_013343235.1	532	Bacteria>Terrabacteria_group>Cyanobacteria/Melainabacteria_group>Cyanobacteria>Nostocales	Nostoc_sp._TCL240-02	DGQHR-domain-containing-protein-[Nostoc-sp.-TCL240-02]	2572090
WP_174712957.1	DndB-ParB-nuclease	GCF_013343235.1	532	Bacteria>Terrabacteria_group>Cyanobacteria/Melainabacteria_group>Cyanobacteria>Nostocales	Nostoc_sp._TCL240-02	DGQHR-domain-containing-protein-[Nostoc-sp.-TCL240-02]	2572090
QLE55595.1	DndB-ParB-nuclease	GCA_013393945.1	532	Bacteria>Terrabacteria_group>Cyanobacteria/Melainabacteria_group>Cyanobacteria>Nostocales	Nostoc_sp._TCL26-01	DGQHR-domain-containing-protein-[Nostoc-sp.-TCL26-01]	2576904
WP_179047760.1	DndB-ParB-nuclease	GCF_013393945.1	532	Bacteria>Terrabacteria_group>Cyanobacteria/Melainabacteria_group>Cyanobacteria>Nostocales	Nostoc_sp._TCL26-01	DGQHR-domain-containing-protein-[Nostoc-sp.-TCL26-01]	2576904
MBU7581828.1	DndB-ParB-nuclease	GCA_018993265.1	532	Bacteria>Terrabacteria_group>Cyanobacteria/Melainabacteria_group>Cyanobacteria>Nostocales	Nostoc_sp._TH1S01	DGQHR-domain-containing-protein-[Nostoc-sp.-TH1S01]	442893
MBU7586913.1	DndB-ParB-nuclease	GCA_018993265.1	138	Bacteria>Terrabacteria_group>Cyanobacteria/Melainabacteria_group>Cyanobacteria>Nostocales	Nostoc_sp._TH1S01	hypothetical-protein-[Nostoc-sp.-TH1S01]	442893
HBW32264.1	DndB-ParB-nuclease	GCA_003514525.1	532	Bacteria>Terrabacteria_group>Cyanobacteria/Melainabacteria_group>Cyanobacteria>Nostocales	Nostoc_sp._UBA8866	DGQHR-domain-containing-protein-[Nostoc-sp.-UBA8866]	2055761
QSJ17856.1	DndB-ParB-nuclease	GCA_017164015.1	532	Bacteria>Terrabacteria_group>Cyanobacteria/Melainabacteria_group>Cyanobacteria>Nostocales	Nostoc_sp._UHCC_0702	DGQHR-domain-containing-protein-[Nostoc-sp.-UHCC-0702]	2814655
WP_206266006.1	DndB-ParB-nuclease	GCF_017164015.1	532	Bacteria>Terrabacteria_group>Cyanobacteria/Melainabacteria_group>Cyanobacteria>Nostocales	Nostoc_sp._UHCC_0702	DGQHR-domain-containing-protein-[Nostoc-sp.-UHCC-0702]	2814655
QSJ17857.1	DndB-ParB-nuclease	GCA_017164015.1	394	Bacteria>Terrabacteria_group>Cyanobacteria/Melainabacteria_group>Cyanobacteria>Nostocales	Nostoc_sp._UHCC_0702	DNA-sulfur-modification-protein-DndB-[Nostoc-sp.-UHCC-0702]	2814655
WP_206266007.1	DndB-ParB-nuclease	GCF_017164015.1	394	Bacteria>Terrabacteria_group>Cyanobacteria/Melainabacteria_group>Cyanobacteria>Nostocales	Nostoc_sp._UHCC_0702	DNA-sulfur-modification-protein-DndB-[Nostoc-sp.-UHCC-0702]	2814655
WP_237988787.1	DndB-ParB-nuclease	GCF_022063185.1	533	Bacteria>Terrabacteria_group>Cyanobacteria/Melainabacteria_group>Cyanobacteria>Nostocales	Nostoc_sp._UHCC_0870	DGQHR-domain-containing-protein-[Nostoc-sp.-UHCC-0870]	2914041
WP_163929524.1	DndB-ParB-nuclease	GCF_010747425.1	532	Bacteria>Terrabacteria_group>Cyanobacteria/Melainabacteria_group>Cyanobacteria>Nostocales	Nostoc_sp._UIC_10630	DGQHR-domain-containing-protein-[Nostoc-sp.-UIC-10630]	2100146
WP_163929526.1	DndB-ParB-nuclease	GCF_010747425.1	577	Bacteria>Terrabacteria_group>Cyanobacteria/Melainabacteria_group>Cyanobacteria>Nostocales	Nostoc_sp._UIC_10630	hypothetical-protein-[Nostoc-sp.-UIC-10630]	2100146
MBG1266307.1	DndB-ParB-nuclease	GCA_015714725.1	533	Bacteria>Terrabacteria_group>Cyanobacteria/Melainabacteria_group>Cyanobacteria>Nostocales	Nostoc_sp._WHI	DGQHR-domain-containing-protein-[Nostoc-sp.-WHI]	2650611
WP_196515739.1	DndB-ParB-nuclease	GCF_015714725.1	533	Bacteria>Terrabacteria_group>Cyanobacteria/Melainabacteria_group>Cyanobacteria>Nostocales	Nostoc_sp._WHI	DGQHR-domain-containing-protein-[Nostoc-sp.-WHI]	2650611
WP_196515740.1	DndB-ParB-nuclease	GCF_015714725.1	396	Bacteria>Terrabacteria_group>Cyanobacteria/Melainabacteria_group>Cyanobacteria>Nostocales	Nostoc_sp._WHI	DNA-sulfur-modification-protein-DndB-[Nostoc-sp.-WHI]	2650611
MBG1266308.1	DndB-ParB-nuclease	GCA_015714725.1	381	Bacteria>Terrabacteria_group>Cyanobacteria/Melainabacteria_group>Cyanobacteria>Nostocales	Nostoc_sp._WHI	hypothetical-protein-[Nostoc-sp.-WHI]	2650611
MCC5660089.1	DndB-ParB-nuclease	GCA_020829625.1	532	Bacteria>Terrabacteria_group>Cyanobacteria/Melainabacteria_group>Cyanobacteria>Nostocales	Nostoc_sp._XA010	DGQHR-domain-containing-protein-[Nostoc-sp.-XA010]	2780407
WP_229556042.1	DndB-ParB-nuclease	GCF_020829625.1	532	Bacteria>Terrabacteria_group>Cyanobacteria/Melainabacteria_group>Cyanobacteria>Nostocales	Nostoc_sp._XA010	DGQHR-domain-containing-protein-[Nostoc-sp.-XA010]	2780407
MCC5651747.1	DndB-ParB-nuclease	GCA_020829585.1	532	Bacteria>Terrabacteria_group>Cyanobacteria/Melainabacteria_group>Cyanobacteria>Nostocales	Nostoc_sp._XA013	DGQHR-domain-containing-protein-[Nostoc-sp.-XA013]	2780408
WP_229560842.1	DndB-ParB-nuclease	-	532	Bacteria>Terrabacteria_group>Cyanobacteria/Melainabacteria_group>Cyanobacteria>Nostocales	Nostoc_sp._XA013	DGQHR-domain-containing-protein-[Nostoc-sp.-XA013]	2780408
MBW4430239.1	DndB-ParB-nuclease	GCA_019359115.1	533	Bacteria>Terrabacteria_group>Cyanobacteria/Melainabacteria_group>Cyanobacteria>Nostocales	Pelatocladus_maniniholoensis_HA4357-MV3	DGQHR-domain-containing-protein-[Pelatocladus-maniniholoensis-HA4357-MV3]	1117104

Accession	Annotation	GCA	Length	Phyletic.spread	Strain.name	Defline	Species.Taxid
BAZ89881.1	DndB-ParB-nuclease	GCA_002368135.1	519	Bacteria>Terrabacteria_group>Cyanobacteria/Melainabacteria_group>Cyanobacteria>Nostocales	Raphidiopsis_curvata_NIES-932	hypothetical-protein-NIES932_13650-[Raphidiopsis-curvata-NIES-932]	1973482
WP_190607209.1	DndB-ParB-nuclease	GCF_019056575.1	533	Bacteria>Terrabacteria_group>Cyanobacteria/Melainabacteria_group>Cyanobacteria>Nostocales	Richelia_sinica	DGQHR-domain-containing-protein-[Richelia-sinica]	1357545
WP_190607207.1	DndB-ParB-nuclease	GCF_019056575.1	396	Bacteria>Terrabacteria_group>Cyanobacteria/Melainabacteria_group>Cyanobacteria>Nostocales	Richelia_sinica	DNA-sulfur-modification-protein-DndB-[Richelia-sinica]	1357545
NJR22006.1	DndB-ParB-nuclease	GCA_012034575.1	223	Bacteria>Terrabacteria_group>Cyanobacteria/Melainabacteria_group>Cyanobacteria>Nostocales	Richelia_sp._CSU_2_1	DGQHR-domain-containing-protein-[Richelia-sp.-CSU_2_1]	2720414
NJR22008.1	DndB-ParB-nuclease	GCA_012034575.1	391	Bacteria>Terrabacteria_group>Cyanobacteria/Melainabacteria_group>Cyanobacteria>Nostocales	Richelia_sp._CSU_2_1	DGQHR-domain-containing-protein-[Richelia-sp.-CSU_2_1]	2720414
NJR21326.1	DndB-ParB-nuclease	GCA_012034575.1	183	Bacteria>Terrabacteria_group>Cyanobacteria/Melainabacteria_group>Cyanobacteria>Nostocales	Richelia_sp._CSU_2_1	hypothetical-protein-[Richelia-sp.-CSU_2_1]	2720414
NJN08420.1	DndB-ParB-nuclease	GCA_012032385.1	526	Bacteria>Terrabacteria_group>Cyanobacteria/Melainabacteria_group>Cyanobacteria>Nostocales	Richelia_sp._RM1_1_1	DGQHR-domain-containing-protein-[Richelia-sp.-RM1_1_1]	2720426
NJO63571.1	DndB-ParB-nuclease	GCA_012033295.1	526	Bacteria>Terrabacteria_group>Cyanobacteria/Melainabacteria_group>Cyanobacteria>Nostocales	Richelia_sp._RM2_1_2	DGQHR-domain-containing-protein-[Richelia-sp.-RM2_1_2]	2720436
NJO27003.1	DndB-ParB-nuclease	GCA_012033205.1	526	Bacteria>Terrabacteria_group>Cyanobacteria/Melainabacteria_group>Cyanobacteria>Nostocales	Richelia_sp._SL_2_1	DGQHR-domain-containing-protein-[Richelia-sp.-SL_2_1]	2720451
NJM17981.1	DndB-ParB-nuclease	GCA_012031715.1	526	Bacteria>Terrabacteria_group>Cyanobacteria/Melainabacteria_group>Cyanobacteria>Nostocales	Richelia_sp._SM1_7_0	DGQHR-domain-containing-protein-[Richelia-sp.-SM1_7_0]	2720461
WP_223972884.1	DndB-ParB-nuclease	GCF_019973055.1	531	Bacteria>Terrabacteria_group>Cyanobacteria/Melainabacteria_group>Cyanobacteria>Nostocales	Rivularia_sp._IAM_M-261	DGQHR-domain-containing-protein-[Rivularia-sp.-IAM-M-261]	448371
WP_223972894.1	DndB-ParB-nuclease	GCF_019973055.1	447	Bacteria>Terrabacteria_group>Cyanobacteria/Melainabacteria_group>Cyanobacteria>Nostocales	Rivularia_sp._IAM_M-261	DGQHR-domain-containing-protein-[Rivularia-sp.-IAM-M-261]	448371
GJD16139.1	DndB-ParB-nuclease	GCA_019973055.1	593	Bacteria>Terrabacteria_group>Cyanobacteria/Melainabacteria_group>Cyanobacteria>Nostocales	Rivularia_sp._IAM_M-261	hypothetical-protein-RIVM261_010950-[Rivularia-sp.-IAM-M-261]	448371
GJD16144.1	DndB-ParB-nuclease	GCA_019973055.1	447	Bacteria>Terrabacteria_group>Cyanobacteria/Melainabacteria_group>Cyanobacteria>Nostocales	Rivularia_sp._IAM_M-261	hypothetical-protein-RIVM261_011000-[Rivularia-sp.-IAM-M-261]	448371
MBV6622204.1	DndB-ParB-nuclease	GCA_019192545.1	525	Bacteria>Terrabacteria_group>Cyanobacteria/Melainabacteria_group>Cyanobacteria>Nostocales	Rivularia_sp._(in:_Bacteria)	DGQHR-domain-containing-protein-[Rivularia-sp.-(in:-Bacteria)]	2047365
AFY54911.1	DndB-ParB-nuclease	GCA_000316665.1	525	Bacteria>Terrabacteria_group>Cyanobacteria/Melainabacteria_group>Cyanobacteria>Nostocales	Rivularia_sp._PCC_7116	DGQHR-domain-containing-protein-[Rivularia-sp.-PCC-7116]	373994
WP_015118485.1	DndB-ParB-nuclease	GCF_000316665.1	525	Bacteria>Terrabacteria_group>Cyanobacteria/Melainabacteria_group>Cyanobacteria>Nostocales	Rivularia_sp._PCC_7116	DGQHR-domain-containing-protein-[Rivularia-sp.-PCC-7116]	373994
MBF2015209.1	DndB-ParB-nuclease	GCA_015272215.1	526	Bacteria>Terrabacteria_group>Cyanobacteria/Melainabacteria_group>Cyanobacteria>Nostocales	Rivularia_sp._T60_A2020_040	DGQHR-domain-containing-protein-[Rivularia-sp.-T60_A2020_040]	2774335
WP_026134357.1	DndB-ParB-nuclease	GCF_000346485.2	532	Bacteria>Terrabacteria_group>Cyanobacteria/Melainabacteria_group>Cyanobacteria>Nostocales	Scytonema_hofmannii	DGQHR-domain-containing-protein-[Scytonema-hofmannii]	34078
MBW4501190.1	DndB-ParB-nuclease	GCA_019359075.1	532	Bacteria>Terrabacteria_group>Cyanobacteria/Melainabacteria_group>Cyanobacteria>Nostocales	Scytonema_hyalinum_WJT4-NPBG1	DGQHR-domain-containing-protein-[Scytonema-hyalinum-WJT4-NPBG1]	1939789
MBW4501191.1	DndB-ParB-nuclease	GCA_019359075.1	396	Bacteria>Terrabacteria_group>Cyanobacteria/Melainabacteria_group>Cyanobacteria>Nostocales	Scytonema_hyalinum_WJT4-NPBG1	DNA-sulfur-modification-protein-DndB-[Scytonema-hyalinum-WJT4-NPBG1]	1939789
WP_073628423.1	DndB-ParB-nuclease	GCF_002368235.1	532	Bacteria>Terrabacteria_group>Cyanobacteria/Melainabacteria_group>Cyanobacteria>Nostocales	Scytonema_sp._HK-05	DGQHR-domain-containing-protein-[Scytonema-sp.-HK-05]	1137095
WP_073628427.1	DndB-ParB-nuclease	GCF_002368235.1	665	Bacteria>Terrabacteria_group>Cyanobacteria/Melainabacteria_group>Cyanobacteria>Nostocales	Scytonema_sp._HK-05	hypothetical-protein-[Scytonema-sp.-HK-05]	1137095
WP_096561489.1	DndB-ParB-nuclease	GCF_002368435.1	532	Bacteria>Terrabacteria_group>Cyanobacteria/Melainabacteria_group>Cyanobacteria>Nostocales	Scytonema_sp._NIES-4073	DGQHR-domain-containing-protein-[Scytonema-sp.-NIES-4073]	2005464
WP_155744418.1	DndB-ParB-nuclease	GCF_009725235.1	532	Bacteria>Terrabacteria_group>Cyanobacteria/Melainabacteria_group>Cyanobacteria>Nostocales	Scytonema_sp._UIC_10036	DGQHR-domain-containing-protein-[Scytonema-sp.-UIC-10036]	2304196
WP_048866999.1	DndB-ParB-nuclease	GCF_000828085.3	532	Bacteria>Terrabacteria_group>Cyanobacteria/Melainabacteria_group>Cyanobacteria>Nostocales	Scytonema_tolypothrichoides	DGQHR-domain-containing-protein-[Scytonema-tolypothrichoides]	1233230
KAB8334997.1	DndB-ParB-nuclease	GCA_000828085.3	532	Bacteria>Terrabacteria_group>Cyanobacteria/Melainabacteria_group>Cyanobacteria>Nostocales	Scytonema_tolypothrichoides_VB-61278	DGQHR-domain-containing-protein-[Scytonema-tolypothrichoides-VB-61278]	1233231
MBW4507930.1	DndB-ParB-nuclease	GCA_019359035.1	530	Bacteria>Terrabacteria_group>Cyanobacteria/Melainabacteria_group>Cyanobacteria>Nostocales	Scytonematopsis_contorta_HA4267-MV1	DGQHR-domain-containing-protein-[Scytonematopsis-contorta-HA4267-MV1]	1049028
MBW4507928.1	DndB-ParB-nuclease	GCA_019359035.1	233	Bacteria>Terrabacteria_group>Cyanobacteria/Melainabacteria_group>Cyanobacteria>Nostocales	Scytonematopsis_contorta_HA4267-MV1	DNA-sulfur-modification-protein-DndB-[Scytonematopsis-contorta-HA4267-MV1]	1049028
MBW4507929.1	DndB-ParB-nuclease	GCA_019359035.1	169	Bacteria>Terrabacteria_group>Cyanobacteria/Melainabacteria_group>Cyanobacteria>Nostocales	Scytonematopsis_contorta_HA4267-MV1	DNA-sulfur-modification-protein-DndB-[Scytonematopsis-contorta-HA4267-MV1]	1049028
WP_193942464.1	DndB-ParB-nuclease	GCF_015207835.1	531	Bacteria>Terrabacteria_group>Cyanobacteria/Melainabacteria_group>Cyanobacteria>Nostocales	Sphaerospermopsis_aphanizomenoides	DGQHR-domain-containing-protein-[Sphaerospermopsis-aphanizomenoides]	459663
WP_200324503.1	DndB-ParB-nuclease	GCF_001586785.1	531	Bacteria>Terrabacteria_group>Cyanobacteria/Melainabacteria_group>Cyanobacteria>Nostocales	Sphaerospermopsis_aphanizomenoides	DGQHR-domain-containing-protein-[Sphaerospermopsis-aphanizomenoides]	459663
WP_096567845.1	DndB-ParB-nuclease	GCF_002368075.1	391	Bacteria>Terrabacteria_group>Cyanobacteria/Melainabacteria_group>Cyanobacteria>Nostocales	Sphaerospermopsis_kisseleviana	DGQHR-domain-containing-protein-[Sphaerospermopsis-kisseleviana]	289435
WP_096567849.1	DndB-ParB-nuclease	GCF_002368075.1	531	Bacteria>Terrabacteria_group>Cyanobacteria/Melainabacteria_group>Cyanobacteria>Nostocales	Sphaerospermopsis_kisseleviana	DGQHR-domain-containing-protein-[Sphaerospermopsis-kisseleviana]	289435
BAZ80548.1	DndB-ParB-nuclease	GCA_002368075.1	391	Bacteria>Terrabacteria_group>Cyanobacteria/Melainabacteria_group>Cyanobacteria>Nostocales	Sphaerospermopsis_kisseleviana_NIES-73	hypothetical-protein-NIES73_18090-[Sphaerospermopsis-kisseleviana-NIES-73]	1973480
BAZ80550.1	DndB-ParB-nuclease	GCA_002368075.1	531	Bacteria>Terrabacteria_group>Cyanobacteria/Melainabacteria_group>Cyanobacteria>Nostocales	Sphaerospermopsis_kisseleviana_NIES-73	hypothetical-protein-NIES73_18110-[Sphaerospermopsis-kisseleviana-NIES-73]	1973480
WP_137669004.1	DndB-ParB-nuclease	GCF_005402885.1	531	Bacteria>Terrabacteria_group>Cyanobacteria/Melainabacteria_group>Cyanobacteria>Nostocales	Sphaerospermopsis_reniformis	DGQHR-domain-containing-protein-[Sphaerospermopsis-reniformis]	531300
GCL39425.1	DndB-ParB-nuclease	GCA_005402885.1	531	Bacteria>Terrabacteria_group>Cyanobacteria/Melainabacteria_group>Cyanobacteria>Nostocales	Sphaerospermopsis_reniformis	hypothetical-protein-SR1949_45510-[Sphaerospermopsis-reniformis]	531300
MBD2134777.1	DndB-ParB-nuclease	GCA_014696195.1	532	Bacteria>Terrabacteria_group>Cyanobacteria/Melainabacteria_group>Cyanobacteria>Nostocales	Sphaerospermopsis_sp._FACHB-1094	DGQHR-domain-containing-protein-[Sphaerospermopsis-sp.-FACHB-1094]	2692861
WP_190650749.1	DndB-ParB-nuclease	GCF_014696195.1	532	Bacteria>Terrabacteria_group>Cyanobacteria/Melainabacteria_group>Cyanobacteria>Nostocales	Sphaerospermopsis_sp._FACHB-1094	DGQHR-domain-containing-protein-[Sphaerospermopsis-sp.-FACHB-1094]	2692861
MBD2134778.1	DndB-ParB-nuclease	GCA_014696195.1	400	Bacteria>Terrabacteria_group>Cyanobacteria/Melainabacteria_group>Cyanobacteria>Nostocales	Sphaerospermopsis_sp._FACHB-1094	DNA-sulfur-modification-protein-DndB-[Sphaerospermopsis-sp.-FACHB-1094]	2692861
WP_199291239.1	DndB-ParB-nuclease	GCF_014696195.1	403	Bacteria>Terrabacteria_group>Cyanobacteria/Melainabacteria_group>Cyanobacteria>Nostocales	Sphaerospermopsis_sp._FACHB-1094	DNA-sulfur-modification-protein-DndB-[Sphaerospermopsis-sp.-FACHB-1094]	2692861
WP_190348155.1	DndB-ParB-nuclease	GCF_014696235.1	531	Bacteria>Terrabacteria_group>Cyanobacteria/Melainabacteria_group>Cyanobacteria>Nostocales	Sphaerospermopsis_sp._FACHB-1194	DGQHR-domain-containing-protein-[Sphaerospermopsis-sp.-FACHB-1194]	2692862
WP_187041724.1	DndB-ParB-nuclease	GCF_014299995.1	531	Bacteria>Terrabacteria_group>Cyanobacteria/Melainabacteria_group>Cyanobacteria>Nostocales	Sphaerospermopsis_sp._LEGE_00249	DGQHR-domain-containing-protein-[Sphaerospermopsis-sp.-LEGE-00249]	1380707
WP_194054063.1	DndB-ParB-nuclease	GCF_015207205.1	531	Bacteria>Terrabacteria_group>Cyanobacteria/Melainabacteria_group>Cyanobacteria>Nostocales	Sphaerospermopsis_sp._LEGE_08334	DGQHR-domain-containing-protein-[Sphaerospermopsis-sp.-LEGE-08334]	1828651
NET02082.1	DndB-ParB-nuclease	GCA_010672785.1	533	Bacteria>Terrabacteria_group>Cyanobacteria/Melainabacteria_group>Cyanobacteria>Nostocales	Sphaerospermopsis_sp._SIO1G1	DGQHR-domain-containing-protein-[Sphaerospermopsis-sp.-SIO1G1]	2607814
WP_220608354.1	DndB-ParB-nuclease	GCF_019598945.1	531	Bacteria>Terrabacteria_group>Cyanobacteria/Melainabacteria_group>Cyanobacteria>Nostocales	Sphaerospermopsis_torques-reginae	DGQHR-domain-containing-protein-[Sphaerospermopsis-torques-reginae]	984207
QYX30111.1	DndB-ParB-nuclease	GCA_019598945.1	531	Bacteria>Terrabacteria_group>Cyanobacteria/Melainabacteria_group>Cyanobacteria>Nostocales	Sphaerospermopsis_torques-reginae_ITEP-024	DGQHR-domain-containing-protein-[Sphaerospermopsis-torques-reginae-ITEP-024]	984208
MBW4449971.1	DndB-ParB-nuclease	GCA_019359015.1	524	Bacteria>Terrabacteria_group>Cyanobacteria/Melainabacteria_group>Cyanobacteria>Nostocales	Spirirestis_rafaelensis_WJT71-NPBG6	DGQHR-domain-containing-protein-[Spirirestis-rafaelensis-WJT71-NPBG6]	2835094
MBR8839595.1	DndB-ParB-nuclease	GCA_016903675.1	532	Bacteria>Terrabacteria_group>Cyanobacteria/Melainabacteria_group>Cyanobacteria>Nostocales	Stigonema_ocellatum_SAG_48.90_=_DSM_106950	DGQHR-domain-containing-protein-[Stigonema-ocellatum-SAG-48.90-=-DSM-106950]	221294
MBR8839596.1	DndB-ParB-nuclease	GCA_016903675.1	396	Bacteria>Terrabacteria_group>Cyanobacteria/Melainabacteria_group>Cyanobacteria>Nostocales	Stigonema_ocellatum_SAG_48.90_=_DSM_106950	DNA-sulfur-modification-protein-DndB-[Stigonema-ocellatum-SAG-48.90-=-DSM-106950]	221294
WP_038086276.1	DndB-ParB-nuclease	GCF_000760695.4	403	Bacteria>Terrabacteria_group>Cyanobacteria/Melainabacteria_group>Cyanobacteria>Nostocales	Tolypothrix_bouteillei	DGQHR-domain-containing-protein-[Tolypothrix-bouteillei]	1246981
WP_038086301.1	DndB-ParB-nuclease	GCF_000760695.4	532	Bacteria>Terrabacteria_group>Cyanobacteria/Melainabacteria_group>Cyanobacteria>Nostocales	Tolypothrix_bouteillei	DGQHR-domain-containing-protein-[Tolypothrix-bouteillei]	1246981
MBW4480206.1	DndB-ParB-nuclease	GCA_019358985.1	528	Bacteria>Terrabacteria_group>Cyanobacteria/Melainabacteria_group>Cyanobacteria>Nostocales	Tolypothrix_brevis_GSE-NOS-MK-07-07A	DGQHR-domain-containing-protein-[Tolypothrix-brevis-GSE-NOS-MK-07-07A]	2835095
WP_041035371.1	DndB-ParB-nuclease	-	532	Bacteria>Terrabacteria_group>Cyanobacteria/Melainabacteria_group>Cyanobacteria>Nostocales	Tolypothrix_campylonemoides	DGQHR-domain-containing-protein-[Tolypothrix-campylonemoides]	1136105
KAB8316855.1	DndB-ParB-nuclease	GCA_000828075.3	532	Bacteria>Terrabacteria_group>Cyanobacteria/Melainabacteria_group>Cyanobacteria>Nostocales	Tolypothrix_campylonemoides_VB511288	DGQHR-domain-containing-protein-[Tolypothrix-campylonemoides-VB511288]	1245935
MBW4570384.1	DndB-ParB-nuclease	GCA_019358935.1	525	Bacteria>Terrabacteria_group>Cyanobacteria/Melainabacteria_group>Cyanobacteria>Nostocales	Tolypothrix_carrinoi_HA7290-LM1	DGQHR-domain-containing-protein-[Tolypothrix-carrinoi-HA7290-LM1]	1874569
MBD2356270.1	DndB-ParB-nuclease	GCA_014697075.1	532	Bacteria>Terrabacteria_group>Cyanobacteria/Melainabacteria_group>Cyanobacteria>Nostocales	Tolypothrix_sp._FACHB-123	DGQHR-domain-containing-protein-[Tolypothrix-sp.-FACHB-123]	2692868
WP_190747329.1	DndB-ParB-nuclease	GCF_014697075.1	532	Bacteria>Terrabacteria_group>Cyanobacteria/Melainabacteria_group>Cyanobacteria>Nostocales	Tolypothrix_sp._FACHB-123	DGQHR-domain-containing-protein-[Tolypothrix-sp.-FACHB-123]	2692868
MBD2356269.1	DndB-ParB-nuclease	GCA_014697075.1	400	Bacteria>Terrabacteria_group>Cyanobacteria/Melainabacteria_group>Cyanobacteria>Nostocales	Tolypothrix_sp._FACHB-123	DNA-sulfur-modification-protein-DndB-[Tolypothrix-sp.-FACHB-123]	2692868
WP_190747328.1	DndB-ParB-nuclease	GCF_014697075.1	400	Bacteria>Terrabacteria_group>Cyanobacteria/Melainabacteria_group>Cyanobacteria>Nostocales	Tolypothrix_sp._FACHB-123	DNA-sulfur-modification-protein-DndB-[Tolypothrix-sp.-FACHB-123]	2692868
WP_089129163.1	DndB-ParB-nuclease	GCF_002218085.1	529	Bacteria>Terrabacteria_group>Cyanobacteria/Melainabacteria_group>Cyanobacteria>Nostocales	Tolypothrix_sp._NIES-4075	DGQHR-domain-containing-protein-[Tolypothrix-sp.-NIES-4075]	2005459
WP_089129164.1	DndB-ParB-nuclease	GCF_002218085.1	395	Bacteria>Terrabacteria_group>Cyanobacteria/Melainabacteria_group>Cyanobacteria>Nostocales	Tolypothrix_sp._NIES-4075	DNA-sulfur-modification-protein-DndB-[Tolypothrix-sp.-NIES-4075]	2005459
EKE98718.1	DndB-ParB-nuclease	GCA_000300115.1	433	Bacteria>Terrabacteria_group>Cyanobacteria/Melainabacteria_group>Cyanobacteria>Nostocales	Tolypothrix_sp._PCC_7601	DGQHR-domain-protein-[Tolypothrix-sp.-PCC-7601]	1188
EKE98794.1	DndB-ParB-nuclease	GCA_000300115.1	81	Bacteria>Terrabacteria_group>Cyanobacteria/Melainabacteria_group>Cyanobacteria>Nostocales	Tolypothrix_sp._PCC_7601	hypothetical-protein-FDUTEX481_03793-[Tolypothrix-sp.-PCC-7601]	1188
QIR41096.1	DndB-ParB-nuclease	GCA_011769525.1	532	Bacteria>Terrabacteria_group>Cyanobacteria/Melainabacteria_group>Cyanobacteria>Nostocales	Tolypothrix_sp._PCC_7910	DGQHR-domain-containing-protein-[Tolypothrix-sp.-PCC-7910]	2099387
WP_167727191.1	DndB-ParB-nuclease	GCF_011769525.1	532	Bacteria>Terrabacteria_group>Cyanobacteria/Melainabacteria_group>Cyanobacteria>Nostocales	Tolypothrix_sp._PCC_7910	DGQHR-domain-containing-protein-[Tolypothrix-sp.-PCC-7910]	2099387
WP_096578794.1	DndB-ParB-nuclease	GCF_002368295.1	532	Bacteria>Terrabacteria_group>Cyanobacteria/Melainabacteria_group>Cyanobacteria>Nostocales	Tolypothrix_tenuis	MULTISPECIES:-DGQHR-domain-containing-protein-[Nostocales]	457083
WP_096578798.1	DndB-ParB-nuclease	GCF_002368295.1	333	Bacteria>Terrabacteria_group>Cyanobacteria/Melainabacteria_group>Cyanobacteria>Nostocales	Tolypothrix_tenuis	MULTISPECIES:-DGQHR-domain-containing-protein-[Nostocales]	457083
MBW4554518.1	DndB-ParB-nuclease	GCA_019358915.1	532	Bacteria>Terrabacteria_group>Cyanobacteria/Melainabacteria_group>Cyanobacteria>Nostocales	Trichormus_sp._ATA11-4-KO1	DGQHR-domain-containing-protein-[Trichormus-sp.-ATA11-4-KO1]	2835091
MBW4554519.1	DndB-ParB-nuclease	GCA_019358915.1	414	Bacteria>Terrabacteria_group>Cyanobacteria/Melainabacteria_group>Cyanobacteria>Nostocales	Trichormus_sp._ATA11-4-KO1	DNA-sulfur-modification-protein-DndB-[Trichormus-sp.-ATA11-4-KO1]	2835091
WP_071190034.1	DndB-ParB-nuclease	GCF_001858025.1	532	Bacteria>Terrabacteria_group>Cyanobacteria/Melainabacteria_group>Cyanobacteria>Nostocales	Trichormus_sp._NMC-1	DGQHR-domain-containing-protein-[Trichormus-sp.-NMC-1]	1853259
WP_127053024.1	DndB-ParB-nuclease	GCF_014698355.1	532	Bacteria>Terrabacteria_group>Cyanobacteria/Melainabacteria_group>Cyanobacteria>Nostocales	Trichormus_variabilis	DGQHR-domain-containing-protein-[Trichormus-variabilis]	264691
WP_185478871.1	DndB-ParB-nuclease	GCF_014222125.1	515	Bacteria>Terrabacteria_group>Cyanobacteria/Melainabacteria_group>Cyanobacteria>Nostocales	Trichormus_variabilis	hypothetical-protein,-partial-[Trichormus-variabilis]	264691
BAY70522.1	DndB-ParB-nuclease	GCA_002368015.1	532	Bacteria>Terrabacteria_group>Cyanobacteria/Melainabacteria_group>Cyanobacteria>Nostocales	Trichormus_variabilis_NIES-23	hypothetical-protein-NIES23_33270-[Trichormus-variabilis-NIES-23]	1973479
WP_168494441.1	DndB-ParB-nuclease	GCF_009711975.1	532	Bacteria>Terrabacteria_group>Cyanobacteria/Melainabacteria_group>Cyanobacteria>Nostocales	unclassified_Anabaena	MULTISPECIES:-DGQHR-domain-containing-protein-[unclassified-Anabaena]	2619674
WP_096685521.1	DndB-ParB-nuclease	GCF_002368455.1	447	Bacteria>Terrabacteria_group>Cyanobacteria/Melainabacteria_group>Cyanobacteria>Nostocales	unclassified_Calothrix	MULTISPECIES:-DGQHR-domain-containing-protein-[unclassified-Calothrix]	2619626
WP_096693963.1	DndB-ParB-nuclease	GCF_002368455.1	531	Bacteria>Terrabacteria_group>Cyanobacteria/Melainabacteria_group>Cyanobacteria>Nostocales	unclassified_Calothrix	MULTISPECIES:-DGQHR-domain-containing-protein-[unclassified-Calothrix]	2619626
WP_190633347.1	DndB-ParB-nuclease	GCF_014696495.1	532	Bacteria>Terrabacteria_group>Cyanobacteria/Melainabacteria_group>Cyanobacteria>Nostocales	unclassified_Calothrix	MULTISPECIES:-DGQHR-domain-containing-protein-[unclassified-Calothrix]	2619626
WP_190633346.1	DndB-ParB-nuclease	GCF_014696495.1	396	Bacteria>Terrabacteria_group>Cyanobacteria/Melainabacteria_group>Cyanobacteria>Nostocales	unclassified_Calothrix	MULTISPECIES:-DNA-sulfur-modification-protein-DndB-[unclassified-Calothrix]	2619626
WP_168653173.1	DndB-ParB-nuclease	GCF_009711965.1	532	Bacteria>Terrabacteria_group>Cyanobacteria/Melainabacteria_group>Cyanobacteria>Nostocales	unclassified_Dolichospermum	MULTISPECIES:-DGQHR-domain-containing-protein-[unclassified-Dolichospermum]	2622029
WP_194000216.1	DndB-ParB-nuclease	GCF_020702635.1	532	Bacteria>Terrabacteria_group>Cyanobacteria/Melainabacteria_group>Cyanobacteria>Nostocales	unclassified_Nodularia	DGQHR-domain-containing-protein-[Nodularia-sp.-LEGE-06071]	2656917
WP_185582260.1	DndB-ParB-nuclease	GCF_014222255.1	532	Bacteria>Terrabacteria_group>Cyanobacteria/Melainabacteria_group>Cyanobacteria>Nostocales	unclassified_Nostoc	MULTISPECIES:-DGQHR-domain-containing-protein-[unclassified-Nostoc]	2593658
WP_190694213.1	DndB-ParB-nuclease	GCF_014697475.1	532	Bacteria>Terrabacteria_group>Cyanobacteria/Melainabacteria_group>Cyanobacteria>Nostocales	unclassified_Nostoc	MULTISPECIES:-DGQHR-domain-containing-protein-[unclassified-Nostoc]	2593658
WP_131119847.1	DndB-ParB-nuclease	GCF_004323185.1	533	Bacteria>Terrabacteria_group>Cyanobacteria/Melainabacteria_group>Cyanobacteria>Nostocales	Westiellopsis_prolifica	DGQHR-domain-containing-protein-[Westiellopsis-prolifica]	221298
TBR57831.1	DndB-ParB-nuclease	GCA_004323185.1	533	Bacteria>Terrabacteria_group>Cyanobacteria/Melainabacteria_group>Cyanobacteria>Nostocales	Westiellopsis_prolifica_IICB1	hypothetical-protein-B4U84_17845-[Westiellopsis-prolifica-IICB1]	1970206
WP_015181012.1	DndB-ParB-nuclease	GCF_000317515.1	532	Bacteria>Terrabacteria_group>Cyanobacteria/Melainabacteria_group>Cyanobacteria>Oscillatoriophycideae	Allocoleopsis_franciscana	DGQHR-domain-containing-protein-[Allocoleopsis-franciscana-PCC-7113]	2886352
WP_015181011.1	DndB-ParB-nuclease	GCF_000317515.1	365	Bacteria>Terrabacteria_group>Cyanobacteria/Melainabacteria_group>Cyanobacteria>Oscillatoriophycideae	Allocoleopsis_franciscana	DNA-sulfur-modification-protein-DndB-[Allocoleopsis-franciscana-PCC-7113]	2886352
WP_106458015.1	DndB-ParB-nuclease	GCF_003007785.1	417	Bacteria>Terrabacteria_group>Cyanobacteria/Melainabacteria_group>Cyanobacteria>Oscillatoriophycideae	Aphanothece_hegewaldii	DNA-sulfur-modification-protein-DndB-[Aphanothece-hegewaldii]	1521625
WP_124976748.1	DndB-ParB-nuclease	GCF_003864295.1	534	Bacteria>Terrabacteria_group>Cyanobacteria/Melainabacteria_group>Cyanobacteria>Oscillatoriophycideae	Aphanothece_sacrum	DGQHR-domain-containing-protein-[Aphanothece-sacrum]	1122
WP_124976752.1	DndB-ParB-nuclease	GCF_003864295.1	177	Bacteria>Terrabacteria_group>Cyanobacteria/Melainabacteria_group>Cyanobacteria>Oscillatoriophycideae	Aphanothece_sacrum	DGQHR-domain-containing-protein-[Aphanothece-sacrum]	1122
MBW4574446.1	DndB-ParB-nuclease	GCA_019359765.1	533	Bacteria>Terrabacteria_group>Cyanobacteria/Melainabacteria_group>Cyanobacteria>Oscillatoriophycideae	Aphanothece_sp._CMT-3BRIN-NPC111	DGQHR-domain-containing-protein-[Aphanothece-sp.-CMT-3BRIN-NPC111]	2839655
MBW4574448.1	DndB-ParB-nuclease	GCA_019359765.1	350	Bacteria>Terrabacteria_group>Cyanobacteria/Melainabacteria_group>Cyanobacteria>Oscillatoriophycideae	Aphanothece_sp._CMT-3BRIN-NPC111	DNA-sulfur-modification-protein-DndB-[Aphanothece-sp.-CMT-3BRIN-NPC111]	2839655
MBW4574449.1	DndB-ParB-nuclease	GCA_019359765.1	663	Bacteria>Terrabacteria_group>Cyanobacteria/Melainabacteria_group>Cyanobacteria>Oscillatoriophycideae	Aphanothece_sp._CMT-3BRIN-NPC111	hypothetical-protein-[Aphanothece-sp.-CMT-3BRIN-NPC111]	2839655
EKD09860.1	DndB-ParB-nuclease	GCA_000307915.1	292	Bacteria>Terrabacteria_group>Cyanobacteria/Melainabacteria_group>Cyanobacteria>Oscillatoriophycideae	Arthrospira_platensis_C1	hypothetical-protein-SPLC1_S130970-[Arthrospira-platensis-C1]	459495
EKD09861.1	DndB-ParB-nuclease	GCA_000307915.1	204	Bacteria>Terrabacteria_group>Cyanobacteria/Melainabacteria_group>Cyanobacteria>Oscillatoriophycideae	Arthrospira_platensis_C1	hypothetical-protein-SPLC1_S130980-[Arthrospira-platensis-C1]	459495
EKD09862.1	DndB-ParB-nuclease	GCA_000307915.1	393	Bacteria>Terrabacteria_group>Cyanobacteria/Melainabacteria_group>Cyanobacteria>Oscillatoriophycideae	Arthrospira_platensis_C1	hypothetical-protein-SPLC1_S130990-[Arthrospira-platensis-C1]	459495
GCE93034.1	DndB-ParB-nuclease	GCA_009176225.1	526	Bacteria>Terrabacteria_group>Cyanobacteria/Melainabacteria_group>Cyanobacteria>Oscillatoriophycideae	Arthrospira_platensis_NIES-46	hypothetical-protein-NIES46_10830-[Arthrospira-platensis-NIES-46]	1236695
GCE93035.1	DndB-ParB-nuclease	GCA_009176225.1	393	Bacteria>Terrabacteria_group>Cyanobacteria/Melainabacteria_group>Cyanobacteria>Oscillatoriophycideae	Arthrospira_platensis_NIES-46	hypothetical-protein-NIES46_10840-[Arthrospira-platensis-NIES-46]	1236695
AMW31427.1	DndB-ParB-nuclease	GCA_001611905.1	526	Bacteria>Terrabacteria_group>Cyanobacteria/Melainabacteria_group>Cyanobacteria>Oscillatoriophycideae	Arthrospira_platensis_YZ	hypothetical-protein-AP285_29450-[Arthrospira-platensis-YZ]	1738638
AMW31428.1	DndB-ParB-nuclease	GCA_001611905.1	393	Bacteria>Terrabacteria_group>Cyanobacteria/Melainabacteria_group>Cyanobacteria>Oscillatoriophycideae	Arthrospira_platensis_YZ	hypothetical-protein-AP285_29455-[Arthrospira-platensis-YZ]	1738638
TVU53046.1	DndB-ParB-nuclease	GCA_007732545.1	393	Bacteria>Terrabacteria_group>Cyanobacteria/Melainabacteria_group>Cyanobacteria>Oscillatoriophycideae	Arthrospira_sp._PLM2.Bin9	DGQHR-domain-containing-protein-[Arthrospira-sp.-PLM2.Bin9]	2599626
TVU53047.1	DndB-ParB-nuclease	GCA_007732545.1	526	Bacteria>Terrabacteria_group>Cyanobacteria/Melainabacteria_group>Cyanobacteria>Oscillatoriophycideae	Arthrospira_sp._PLM2.Bin9	DGQHR-domain-containing-protein-[Arthrospira-sp.-PLM2.Bin9]	2599626
WP_106367341.1	DndB-ParB-nuclease	GCF_003003885.1	397	Bacteria>Terrabacteria_group>Cyanobacteria/Melainabacteria_group>Cyanobacteria>Oscillatoriophycideae	Chlorogloea_sp._CCALA_695	DNA-sulfur-modification-protein-DndB-[Chlorogloea-sp.-CCALA-695]	2107693
PSB34998.1	DndB-ParB-nuclease	GCA_003003885.1	397	Bacteria>Terrabacteria_group>Cyanobacteria/Melainabacteria_group>Cyanobacteria>Oscillatoriophycideae	Chlorogloea_sp._CCALA_695	hypothetical-protein-C7B70_01705-[Chlorogloea-sp.-CCALA-695]	2107693
PIG93201.1	DndB-ParB-nuclease	GCA_002749975.1	522	Bacteria>Terrabacteria_group>Cyanobacteria/Melainabacteria_group>Cyanobacteria>Oscillatoriophycideae	Chroococcales_cyanobacterium_IPPAS_B-1203	hypothetical-protein-CSQ79_13480-[Chroococcales-cyanobacterium-IPPAS-B-1203]	2049454
WP_206814321.1	DndB-ParB-nuclease	GCF_017312365.1	389	Bacteria>Terrabacteria_group>Cyanobacteria/Melainabacteria_group>Cyanobacteria>Oscillatoriophycideae	Chroococcus_sp._FPU101	DNA-sulfur-modification-protein-DndB-[Chroococcus-sp.-FPU101]	1974212
OKH24276.1	DndB-ParB-nuclease	GCA_001904655.1	394	Bacteria>Terrabacteria_group>Cyanobacteria/Melainabacteria_group>Cyanobacteria>Oscillatoriophycideae	Chroogloeocystis_siderophila_5.2_s.c.1	hypothetical-protein-NIES1031_15910-[Chroogloeocystis-siderophila-5.2-s.c.1]	247279
OKH24277.1	DndB-ParB-nuclease	GCA_001904655.1	522	Bacteria>Terrabacteria_group>Cyanobacteria/Melainabacteria_group>Cyanobacteria>Oscillatoriophycideae	Chroogloeocystis_siderophila_5.2_s.c.1	hypothetical-protein-NIES1031_15920-[Chroogloeocystis-siderophila-5.2-s.c.1]	247279
WP_073550506.1	DndB-ParB-nuclease	GCF_001904655.1	522	Bacteria>Terrabacteria_group>Cyanobacteria/Melainabacteria_group>Cyanobacteria>Oscillatoriophycideae	Chroogloeocystis_siderophila	DGQHR-domain-containing-protein-[Chroogloeocystis-siderophila]	329163
WP_073550505.1	DndB-ParB-nuclease	GCF_001904655.1	394	Bacteria>Terrabacteria_group>Cyanobacteria/Melainabacteria_group>Cyanobacteria>Oscillatoriophycideae	Chroogloeocystis_siderophila	DNA-sulfur-modification-protein-DndB-[Chroogloeocystis-siderophila]	329163
WP_006101196.1	DndB-ParB-nuclease	GCF_000155555.1	532	Bacteria>Terrabacteria_group>Cyanobacteria/Melainabacteria_group>Cyanobacteria>Oscillatoriophycideae	Coleofasciculus_chthonoplastes	DGQHR-domain-containing-protein-[Coleofasciculus-chthonoplastes]	64178
WP_006101039.1	DndB-ParB-nuclease	GCF_000155555.1	354	Bacteria>Terrabacteria_group>Cyanobacteria/Melainabacteria_group>Cyanobacteria>Oscillatoriophycideae	Coleofasciculus_chthonoplastes	DNA-sulfur-modification-protein-DndB-[Coleofasciculus-chthonoplastes]	64178
WP_044207305.1	DndB-ParB-nuclease	GCF_000155555.1	391	Bacteria>Terrabacteria_group>Cyanobacteria/Melainabacteria_group>Cyanobacteria>Oscillatoriophycideae	Coleofasciculus_chthonoplastes	DNA-sulfur-modification-protein-DndB-[Coleofasciculus-chthonoplastes]	64178
MBD2744200.1	DndB-ParB-nuclease	GCA_014698845.1	533	Bacteria>Terrabacteria_group>Cyanobacteria/Melainabacteria_group>Cyanobacteria>Oscillatoriophycideae	Coleofasciculus_sp._FACHB-1120	DGQHR-domain-containing-protein-[Coleofasciculus-sp.-FACHB-1120]	2692783
WP_190821760.1	DndB-ParB-nuclease	GCF_014698845.1	533	Bacteria>Terrabacteria_group>Cyanobacteria/Melainabacteria_group>Cyanobacteria>Oscillatoriophycideae	Coleofasciculus_sp._FACHB-1120	DGQHR-domain-containing-protein-[Coleofasciculus-sp.-FACHB-1120]	2692783
MBD2744201.1	DndB-ParB-nuclease	GCA_014698845.1	354	Bacteria>Terrabacteria_group>Cyanobacteria/Melainabacteria_group>Cyanobacteria>Oscillatoriophycideae	Coleofasciculus_sp._FACHB-1120	DNA-sulfur-modification-protein-DndB-[Coleofasciculus-sp.-FACHB-1120]	2692783
WP_190821762.1	DndB-ParB-nuclease	GCF_014698845.1	354	Bacteria>Terrabacteria_group>Cyanobacteria/Melainabacteria_group>Cyanobacteria>Oscillatoriophycideae	Coleofasciculus_sp._FACHB-1120	DNA-sulfur-modification-protein-DndB-[Coleofasciculus-sp.-FACHB-1120]	2692783
MBD2744202.1	DndB-ParB-nuclease	GCA_014698845.1	669	Bacteria>Terrabacteria_group>Cyanobacteria/Melainabacteria_group>Cyanobacteria>Oscillatoriophycideae	Coleofasciculus_sp._FACHB-1120	hypothetical-protein-[Coleofasciculus-sp.-FACHB-1120]	2692783
WP_190821764.1	DndB-ParB-nuclease	GCF_014698845.1	669	Bacteria>Terrabacteria_group>Cyanobacteria/Melainabacteria_group>Cyanobacteria>Oscillatoriophycideae	Coleofasciculus_sp._FACHB-1120	hypothetical-protein-[Coleofasciculus-sp.-FACHB-1120]	2692783
MBD1899385.1	DndB-ParB-nuclease	GCA_014695485.1	533	Bacteria>Terrabacteria_group>Cyanobacteria/Melainabacteria_group>Cyanobacteria>Oscillatoriophycideae	Coleofasciculus_sp._FACHB-125	DGQHR-domain-containing-protein-[Coleofasciculus-sp.-FACHB-125]	2692784
WP_190411008.1	DndB-ParB-nuclease	GCF_014695485.1	533	Bacteria>Terrabacteria_group>Cyanobacteria/Melainabacteria_group>Cyanobacteria>Oscillatoriophycideae	Coleofasciculus_sp._FACHB-125	DGQHR-domain-containing-protein-[Coleofasciculus-sp.-FACHB-125]	2692784
MBD1899383.1	DndB-ParB-nuclease	GCA_014695485.1	380	Bacteria>Terrabacteria_group>Cyanobacteria/Melainabacteria_group>Cyanobacteria>Oscillatoriophycideae	Coleofasciculus_sp._FACHB-125	DNA-sulfur-modification-protein-DndB-[Coleofasciculus-sp.-FACHB-125]	2692784
MBD1899384.1	DndB-ParB-nuclease	GCA_014695485.1	362	Bacteria>Terrabacteria_group>Cyanobacteria/Melainabacteria_group>Cyanobacteria>Oscillatoriophycideae	Coleofasciculus_sp._FACHB-125	DNA-sulfur-modification-protein-DndB-[Coleofasciculus-sp.-FACHB-125]	2692784
WP_190411006.1	DndB-ParB-nuclease	-	380	Bacteria>Terrabacteria_group>Cyanobacteria/Melainabacteria_group>Cyanobacteria>Oscillatoriophycideae	Coleofasciculus_sp._FACHB-125	DNA-sulfur-modification-protein-DndB-[Coleofasciculus-sp.-FACHB-125]	2692784

Accession	Annotation	GCA	Length	Phyletic.spread	Strain.name	Defline	Species.Taxid
WP_190411007.1	DndB-ParB-nuclease	GCF_014695485.1	362	Bacteria>Terrabacteria_group>Cyanobacteria/Melainabacteria_group>Cyanobacteria>Oscillatoriophycideae	Coleofasciculus_sp._FACHB-125	DNA-sulfur-modification-protein-DndB-[Coleofasciculus-sp.-FACHB-125]	2692784
MBD1897629.1	DndB-ParB-nuclease	GCA_014695435.1	533	Bacteria>Terrabacteria_group>Cyanobacteria/Melainabacteria_group>Cyanobacteria>Oscillatoriophycideae	Coleofasciculus_sp._FACHB-129	DGQHR-domain-containing-protein-[Coleofasciculus-sp.-FACHB-129]	2692785
WP_190678472.1	DndB-ParB-nuclease	GCF_014695435.1	533	Bacteria>Terrabacteria_group>Cyanobacteria/Melainabacteria_group>Cyanobacteria>Oscillatoriophycideae	Coleofasciculus_sp._FACHB-129	DGQHR-domain-containing-protein-[Coleofasciculus-sp.-FACHB-129]	2692785
MBD1897630.1	DndB-ParB-nuclease	GCA_014695435.1	362	Bacteria>Terrabacteria_group>Cyanobacteria/Melainabacteria_group>Cyanobacteria>Oscillatoriophycideae	Coleofasciculus_sp._FACHB-129	DNA-sulfur-modification-protein-DndB-[Coleofasciculus-sp.-FACHB-129]	2692785
WP_190678473.1	DndB-ParB-nuclease	GCF_014695435.1	362	Bacteria>Terrabacteria_group>Cyanobacteria/Melainabacteria_group>Cyanobacteria>Oscillatoriophycideae	Coleofasciculus_sp._FACHB-129	DNA-sulfur-modification-protein-DndB-[Coleofasciculus-sp.-FACHB-129]	2692785
MBD1897631.1	DndB-ParB-nuclease	GCA_014695435.1	576	Bacteria>Terrabacteria_group>Cyanobacteria/Melainabacteria_group>Cyanobacteria>Oscillatoriophycideae	Coleofasciculus_sp._FACHB-129	hypothetical-protein-[Coleofasciculus-sp.-FACHB-129]	2692785
WP_190678474.1	DndB-ParB-nuclease	GCF_014695435.1	576	Bacteria>Terrabacteria_group>Cyanobacteria/Melainabacteria_group>Cyanobacteria>Oscillatoriophycideae	Coleofasciculus_sp._FACHB-129	hypothetical-protein-[Coleofasciculus-sp.-FACHB-129]	2692785
MBD1838185.1	DndB-ParB-nuclease	GCA_014695285.1	533	Bacteria>Terrabacteria_group>Cyanobacteria/Melainabacteria_group>Cyanobacteria>Oscillatoriophycideae	Coleofasciculus_sp._FACHB-501	DGQHR-domain-containing-protein-[Coleofasciculus-sp.-FACHB-501]	2692786
MBD1838183.1	DndB-ParB-nuclease	GCA_014695285.1	391	Bacteria>Terrabacteria_group>Cyanobacteria/Melainabacteria_group>Cyanobacteria>Oscillatoriophycideae	Coleofasciculus_sp._FACHB-501	DNA-sulfur-modification-protein-DndB-[Coleofasciculus-sp.-FACHB-501]	2692786
MBD1838184.1	DndB-ParB-nuclease	GCA_014695285.1	362	Bacteria>Terrabacteria_group>Cyanobacteria/Melainabacteria_group>Cyanobacteria>Oscillatoriophycideae	Coleofasciculus_sp._FACHB-501	DNA-sulfur-modification-protein-DndB-[Coleofasciculus-sp.-FACHB-501]	2692786
MBD2085496.1	DndB-ParB-nuclease	GCA_014695825.1	533	Bacteria>Terrabacteria_group>Cyanobacteria/Melainabacteria_group>Cyanobacteria>Oscillatoriophycideae	Coleofasciculus_sp._FACHB-542	DGQHR-domain-containing-protein-[Coleofasciculus-sp.-FACHB-542]	2692787
MBD2085494.1	DndB-ParB-nuclease	GCA_014695825.1	391	Bacteria>Terrabacteria_group>Cyanobacteria/Melainabacteria_group>Cyanobacteria>Oscillatoriophycideae	Coleofasciculus_sp._FACHB-542	DNA-sulfur-modification-protein-DndB-[Coleofasciculus-sp.-FACHB-542]	2692787
MBD2085495.1	DndB-ParB-nuclease	GCA_014695825.1	362	Bacteria>Terrabacteria_group>Cyanobacteria/Melainabacteria_group>Cyanobacteria>Oscillatoriophycideae	Coleofasciculus_sp._FACHB-542	DNA-sulfur-modification-protein-DndB-[Coleofasciculus-sp.-FACHB-542]	2692787
WP_190534466.1	DndB-ParB-nuclease	GCF_014695825.1	391	Bacteria>Terrabacteria_group>Cyanobacteria/Melainabacteria_group>Cyanobacteria>Oscillatoriophycideae	Coleofasciculus_sp._FACHB-542	DNA-sulfur-modification-protein-DndB-[Coleofasciculus-sp.-FACHB-542]	2692787
WP_190534469.1	DndB-ParB-nuclease	GCF_014695825.1	362	Bacteria>Terrabacteria_group>Cyanobacteria/Melainabacteria_group>Cyanobacteria>Oscillatoriophycideae	Coleofasciculus_sp._FACHB-542	DNA-sulfur-modification-protein-DndB-[Coleofasciculus-sp.-FACHB-542]	2692787
WP_190430827.1	DndB-ParB-nuclease	GCF_014695635.1	477	Bacteria>Terrabacteria_group>Cyanobacteria/Melainabacteria_group>Cyanobacteria>Oscillatoriophycideae	Coleofasciculus_sp._FACHB-712	DGQHR-domain-containing-protein,-partial-[Coleofasciculus-sp.-FACHB-712]	2692789
MBD1945600.1	DndB-ParB-nuclease	GCA_014695635.1	150	Bacteria>Terrabacteria_group>Cyanobacteria/Melainabacteria_group>Cyanobacteria>Oscillatoriophycideae	Coleofasciculus_sp._FACHB-712	DNA-sulfur-modification-protein-DndB-[Coleofasciculus-sp.-FACHB-712]	2692789
MBD2540899.1	DndB-ParB-nuclease	GCA_014698185.1	533	Bacteria>Terrabacteria_group>Cyanobacteria/Melainabacteria_group>Cyanobacteria>Oscillatoriophycideae	Coleofasciculus_sp._FACHB-SPT36	DGQHR-domain-containing-protein-[Coleofasciculus-sp.-FACHB-SPT36]	2692790
MBD2540900.1	DndB-ParB-nuclease	GCA_014698185.1	367	Bacteria>Terrabacteria_group>Cyanobacteria/Melainabacteria_group>Cyanobacteria>Oscillatoriophycideae	Coleofasciculus_sp._FACHB-SPT36	DNA-sulfur-modification-protein-DndB-[Coleofasciculus-sp.-FACHB-SPT36]	2692790
MBD2540901.1	DndB-ParB-nuclease	GCA_014698185.1	418	Bacteria>Terrabacteria_group>Cyanobacteria/Melainabacteria_group>Cyanobacteria>Oscillatoriophycideae	Coleofasciculus_sp._FACHB-SPT36	DNA-sulfur-modification-protein-DndB-[Coleofasciculus-sp.-FACHB-SPT36]	2692790
WP_190986473.1	DndB-ParB-nuclease	GCF_014698185.1	367	Bacteria>Terrabacteria_group>Cyanobacteria/Melainabacteria_group>Cyanobacteria>Oscillatoriophycideae	Coleofasciculus_sp._FACHB-SPT36	DNA-sulfur-modification-protein-DndB-[Coleofasciculus-sp.-FACHB-SPT36]	2692790
WP_190986477.1	DndB-ParB-nuclease	-	418	Bacteria>Terrabacteria_group>Cyanobacteria/Melainabacteria_group>Cyanobacteria>Oscillatoriophycideae	Coleofasciculus_sp._FACHB-SPT36	DNA-sulfur-modification-protein-DndB-[Coleofasciculus-sp.-FACHB-SPT36]	2692790
MBD1889271.1	DndB-ParB-nuclease	GCA_014695475.1	533	Bacteria>Terrabacteria_group>Cyanobacteria/Melainabacteria_group>Cyanobacteria>Oscillatoriophycideae	Coleofasciculus_sp._FACHB-SPT9	DGQHR-domain-containing-protein-[Coleofasciculus-sp.-FACHB-SPT9]	2692791
WP_190684050.1	DndB-ParB-nuclease	GCF_014695475.1	533	Bacteria>Terrabacteria_group>Cyanobacteria/Melainabacteria_group>Cyanobacteria>Oscillatoriophycideae	Coleofasciculus_sp._FACHB-SPT9	DGQHR-domain-containing-protein-[Coleofasciculus-sp.-FACHB-SPT9]	2692791
MBD1889270.1	DndB-ParB-nuclease	GCA_014695475.1	362	Bacteria>Terrabacteria_group>Cyanobacteria/Melainabacteria_group>Cyanobacteria>Oscillatoriophycideae	Coleofasciculus_sp._FACHB-SPT9	DNA-sulfur-modification-protein-DndB-[Coleofasciculus-sp.-FACHB-SPT9]	2692791
WP_190684047.1	DndB-ParB-nuclease	GCF_014695475.1	362	Bacteria>Terrabacteria_group>Cyanobacteria/Melainabacteria_group>Cyanobacteria>Oscillatoriophycideae	Coleofasciculus_sp._FACHB-SPT9	DNA-sulfur-modification-protein-DndB-[Coleofasciculus-sp.-FACHB-SPT9]	2692791
MBD1881861.1	DndB-ParB-nuclease	GCA_014695375.1	533	Bacteria>Terrabacteria_group>Cyanobacteria/Melainabacteria_group>Cyanobacteria>Oscillatoriophycideae	Coleofasciculus_sp._FACHB-T130	DGQHR-domain-containing-protein-[Coleofasciculus-sp.-FACHB-T130]	2692792
WP_190529803.1	DndB-ParB-nuclease	GCF_014695375.1	533	Bacteria>Terrabacteria_group>Cyanobacteria/Melainabacteria_group>Cyanobacteria>Oscillatoriophycideae	Coleofasciculus_sp._FACHB-T130	DGQHR-domain-containing-protein-[Coleofasciculus-sp.-FACHB-T130]	2692792
MBD1881862.1	DndB-ParB-nuclease	GCA_014695375.1	362	Bacteria>Terrabacteria_group>Cyanobacteria/Melainabacteria_group>Cyanobacteria>Oscillatoriophycideae	Coleofasciculus_sp._FACHB-T130	DNA-sulfur-modification-protein-DndB-[Coleofasciculus-sp.-FACHB-T130]	2692792
WP_190529805.1	DndB-ParB-nuclease	GCF_014695375.1	362	Bacteria>Terrabacteria_group>Cyanobacteria/Melainabacteria_group>Cyanobacteria>Oscillatoriophycideae	Coleofasciculus_sp._FACHB-T130	DNA-sulfur-modification-protein-DndB-[Coleofasciculus-sp.-FACHB-T130]	2692792
MBD1881863.1	DndB-ParB-nuclease	GCA_014695375.1	573	Bacteria>Terrabacteria_group>Cyanobacteria/Melainabacteria_group>Cyanobacteria>Oscillatoriophycideae	Coleofasciculus_sp._FACHB-T130	hypothetical-protein-[Coleofasciculus-sp.-FACHB-T130]	2692792
WP_190529807.1	DndB-ParB-nuclease	GCF_014695375.1	573	Bacteria>Terrabacteria_group>Cyanobacteria/Melainabacteria_group>Cyanobacteria>Oscillatoriophycideae	Coleofasciculus_sp._FACHB-T130	hypothetical-protein-[Coleofasciculus-sp.-FACHB-T130]	2692792
MBE9127657.1	DndB-ParB-nuclease	GCA_015207445.1	532	Bacteria>Terrabacteria_group>Cyanobacteria/Melainabacteria_group>Cyanobacteria>Oscillatoriophycideae	Coleofasciculus_sp._LEGE_07081	DGQHR-domain-containing-protein-[Coleofasciculus-sp.-LEGE-07081]	2777967
MBE9127656.1	DndB-ParB-nuclease	GCA_015207445.1	365	Bacteria>Terrabacteria_group>Cyanobacteria/Melainabacteria_group>Cyanobacteria>Oscillatoriophycideae	Coleofasciculus_sp._LEGE_07081	DNA-sulfur-modification-protein-DndB-[Coleofasciculus-sp.-LEGE-07081]	2777967
WP_015202529.1	DndB-ParB-nuclease	GCF_000317495.1	529	Bacteria>Terrabacteria_group>Cyanobacteria/Melainabacteria_group>Cyanobacteria>Oscillatoriophycideae	Crinalium_epipsammum	DGQHR-domain-containing-protein-[Crinalium-epipsammum]	241425
WP_015202528.1	DndB-ParB-nuclease	GCF_000317495.1	345	Bacteria>Terrabacteria_group>Cyanobacteria/Melainabacteria_group>Cyanobacteria>Oscillatoriophycideae	Crinalium_epipsammum	DNA-sulfur-modification-protein-DndB-[Crinalium-epipsammum]	241425
AFZ12408.1	DndB-ParB-nuclease	GCA_000317495.1	529	Bacteria>Terrabacteria_group>Cyanobacteria/Melainabacteria_group>Cyanobacteria>Oscillatoriophycideae	Crinalium_epipsammum_PCC_9333	DGQHR-domain-protein-[Crinalium-epipsammum-PCC-9333]	1173022
EAZ93061.1	DndB-ParB-nuclease	GCA_000169335.1	537	Bacteria>Terrabacteria_group>Cyanobacteria/Melainabacteria_group>Cyanobacteria>Oscillatoriophycideae	Crocosphaera_chwakensis_CCY0110	hypothetical-protein-CY0110_03294-[Crocosphaera-chwakensis-CCY0110]	391612
EAZ93062.1	DndB-ParB-nuclease	GCA_000169335.1	266	Bacteria>Terrabacteria_group>Cyanobacteria/Melainabacteria_group>Cyanobacteria>Oscillatoriophycideae	Crocosphaera_chwakensis_CCY0110	hypothetical-protein-CY0110_03299-[Crocosphaera-chwakensis-CCY0110]	391612
EAZ93064.1	DndB-ParB-nuclease	GCA_000169335.1	401	Bacteria>Terrabacteria_group>Cyanobacteria/Melainabacteria_group>Cyanobacteria>Oscillatoriophycideae	Crocosphaera_chwakensis_CCY0110	hypothetical-protein-CY0110_03309-[Crocosphaera-chwakensis-CCY0110]	391612
WP_008273731.1	DndB-ParB-nuclease	GCF_000169335.1	537	Bacteria>Terrabacteria_group>Cyanobacteria/Melainabacteria_group>Cyanobacteria>Oscillatoriophycideae	Crocosphaera_chwakensis	DGQHR-domain-containing-protein-[Crocosphaera-chwakensis]	2546361
WP_008273734.1	DndB-ParB-nuclease	GCF_000169335.1	401	Bacteria>Terrabacteria_group>Cyanobacteria/Melainabacteria_group>Cyanobacteria>Oscillatoriophycideae	Crocosphaera_chwakensis	DNA-sulfur-modification-protein-DndB-[Crocosphaera-chwakensis]	2546361
WP_232285684.1	DndB-ParB-nuclease	GCF_000169335.1	267	Bacteria>Terrabacteria_group>Cyanobacteria/Melainabacteria_group>Cyanobacteria>Oscillatoriophycideae	Crocosphaera_chwakensis	DNA-sulfur-modification-protein-DndB-[Crocosphaera-chwakensis]	2546361
WP_007312080.1	DndB-ParB-nuclease	GCF_000235665.1	531	Bacteria>Terrabacteria_group>Cyanobacteria/Melainabacteria_group>Cyanobacteria>Oscillatoriophycideae	Crocosphaera_watsonii	DGQHR-domain-containing-protein-[Crocosphaera-watsonii]	263511
WP_231599079.1	DndB-ParB-nuclease	GCF_000235665.1	292	Bacteria>Terrabacteria_group>Cyanobacteria/Melainabacteria_group>Cyanobacteria>Oscillatoriophycideae	Crocosphaera_watsonii	DNA-sulfur-modification-protein-DndB-[Crocosphaera-watsonii]	263511
PSB49695.1	DndB-ParB-nuclease	GCA_003004015.1	394	Bacteria>Terrabacteria_group>Cyanobacteria/Melainabacteria_group>Cyanobacteria>Oscillatoriophycideae	Cyanosarcina_cf._burmensis_CCALA_770	hypothetical-protein-C7B80_01305-[Cyanosarcina-cf.-burmensis-CCALA-770]	2107697
PSB49696.1	DndB-ParB-nuclease	GCA_003004015.1	524	Bacteria>Terrabacteria_group>Cyanobacteria/Melainabacteria_group>Cyanobacteria>Oscillatoriophycideae	Cyanosarcina_cf._burmensis_CCALA_770	hypothetical-protein-C7B80_01310-[Cyanosarcina-cf.-burmensis-CCALA-770]	2107697
HAC63415.1	DndB-ParB-nuclease	GCA_003448685.1	482	Bacteria>Terrabacteria_group>Cyanobacteria/Melainabacteria_group>Cyanobacteria>Oscillatoriophycideae	Cyanothece_sp._UBA12306	hypothetical-protein-[Cyanothece-sp.-UBA12306]	2055757
PNW37489.1	DndB-ParB-nuclease	GCA_002900215.1	215	Bacteria>Terrabacteria_group>Cyanobacteria/Melainabacteria_group>Cyanobacteria>Oscillatoriophycideae	Euhalothece_sp._KZN_001	hypothetical-protein-BEN50_16105,-partial-[Euhalothece-sp.-KZN-001]	1884791
WP_071518862.1	DndB-ParB-nuclease	GCF_001870905.1	516	Bacteria>Terrabacteria_group>Cyanobacteria/Melainabacteria_group>Cyanobacteria>Oscillatoriophycideae	Geitlerinema_sp._PCC_9228	DGQHR-domain-containing-protein-[Geitlerinema-sp.-PCC-9228]	111611
TVQ44085.1	DndB-ParB-nuclease	GCA_007693955.1	383	Bacteria>Terrabacteria_group>Cyanobacteria/Melainabacteria_group>Cyanobacteria>Oscillatoriophycideae	Gloeocapsa_sp._DLM2.Bin57	DGQHR-domain-containing-protein-[Gloeocapsa-sp.-DLM2.Bin57]	2480214
WP_015188358.1	DndB-ParB-nuclease	GCF_000317555.1	522	Bacteria>Terrabacteria_group>Cyanobacteria/Melainabacteria_group>Cyanobacteria>Oscillatoriophycideae	Gloeocapsa_sp._PCC_7428	DGQHR-domain-containing-protein-[Gloeocapsa-sp.-PCC-7428]	1173026
AFZ30483.1	DndB-ParB-nuclease	GCA_000317555.1	395	Bacteria>Terrabacteria_group>Cyanobacteria/Melainabacteria_group>Cyanobacteria>Oscillatoriophycideae	Gloeocapsa_sp._PCC_7428	DGQHR-domain-protein-[Gloeocapsa-sp.-PCC-7428]	1173026
AFZ30484.1	DndB-ParB-nuclease	GCA_000317555.1	522	Bacteria>Terrabacteria_group>Cyanobacteria/Melainabacteria_group>Cyanobacteria>Oscillatoriophycideae	Gloeocapsa_sp._PCC_7428	DGQHR-domain-protein-[Gloeocapsa-sp.-PCC-7428]	1173026
WP_015188357.1	DndB-ParB-nuclease	GCF_000317555.1	395	Bacteria>Terrabacteria_group>Cyanobacteria/Melainabacteria_group>Cyanobacteria>Oscillatoriophycideae	Gloeocapsa_sp._PCC_7428	DNA-sulfur-modification-protein-DndB-[Gloeocapsa-sp.-PCC-7428]	1173026
WP_193932010.1	DndB-ParB-nuclease	GCF_015207655.1	523	Bacteria>Terrabacteria_group>Cyanobacteria/Melainabacteria_group>Cyanobacteria>Oscillatoriophycideae	Gloeocapsopsis_crepidinum	DGQHR-domain-containing-protein-[Gloeocapsopsis-crepidinum]	693223
WP_193932011.1	DndB-ParB-nuclease	GCF_015207655.1	396	Bacteria>Terrabacteria_group>Cyanobacteria/Melainabacteria_group>Cyanobacteria>Oscillatoriophycideae	Gloeocapsopsis_crepidinum	DNA-sulfur-modification-protein-DndB-[Gloeocapsopsis-crepidinum]	693223
MBE9190841.1	DndB-ParB-nuclease	GCA_015207655.1	523	Bacteria>Terrabacteria_group>Cyanobacteria/Melainabacteria_group>Cyanobacteria>Oscillatoriophycideae	Gloeocapsopsis_crepidinum_LEGE_06123	DGQHR-domain-containing-protein-[Gloeocapsopsis-crepidinum-LEGE-06123]	588587
MBE9190842.1	DndB-ParB-nuclease	GCA_015207655.1	396	Bacteria>Terrabacteria_group>Cyanobacteria/Melainabacteria_group>Cyanobacteria>Oscillatoriophycideae	Gloeocapsopsis_crepidinum_LEGE_06123	DNA-sulfur-modification-protein-DndB-[Gloeocapsopsis-crepidinum-LEGE-06123]	588587
MUL38628.1	DndB-ParB-nuclease	GCA_009728615.1	522	Bacteria>Terrabacteria_group>Cyanobacteria/Melainabacteria_group>Cyanobacteria>Oscillatoriophycideae	Gloeocapsopsis_dulcis_AAB1_=_1H9	hypothetical-protein-[Gloeocapsopsis-dulcis-AAB1-=-1H9]	1433147
WP_105218976.1	DndB-ParB-nuclease	GCF_009728615.1	522	Bacteria>Terrabacteria_group>Cyanobacteria/Melainabacteria_group>Cyanobacteria>Oscillatoriophycideae	Gloeocapsopsis_dulcis	DGQHR-domain-containing-protein-[Gloeocapsopsis-dulcis]	2859516
WP_015956775.1	DndB-ParB-nuclease	GCF_000021825.1	527	Bacteria>Terrabacteria_group>Cyanobacteria/Melainabacteria_group>Cyanobacteria>Oscillatoriophycideae	Gloeothece_citriformis	DGQHR-domain-containing-protein-[Gloeothece-citriformis]	2546356
WP_015956776.1	DndB-ParB-nuclease	GCF_000021825.1	249	Bacteria>Terrabacteria_group>Cyanobacteria/Melainabacteria_group>Cyanobacteria>Oscillatoriophycideae	Gloeothece_citriformis	DNA-sulfur-modification-protein-DndB-[Gloeothece-citriformis]	2546356
WP_015956778.1	DndB-ParB-nuclease	GCF_000021825.1	391	Bacteria>Terrabacteria_group>Cyanobacteria/Melainabacteria_group>Cyanobacteria>Oscillatoriophycideae	Gloeothece_citriformis	DNA-sulfur-modification-protein-DndB-[Gloeothece-citriformis]	2546356
WP_013324693.1	DndB-ParB-nuclease	GCF_000147335.1	527	Bacteria>Terrabacteria_group>Cyanobacteria/Melainabacteria_group>Cyanobacteria>Oscillatoriophycideae	Gloeothece_verrucosa	DGQHR-domain-containing-protein-[Gloeothece-verrucosa]	2546359
WP_013324694.1	DndB-ParB-nuclease	-	266	Bacteria>Terrabacteria_group>Cyanobacteria/Melainabacteria_group>Cyanobacteria>Oscillatoriophycideae	Gloeothece_verrucosa	DNA-sulfur-modification-protein-DndB-[Gloeothece-verrucosa]	2546359
WP_013324696.1	DndB-ParB-nuclease	GCF_000147335.1	393	Bacteria>Terrabacteria_group>Cyanobacteria/Melainabacteria_group>Cyanobacteria>Oscillatoriophycideae	Gloeothece_verrucosa	DNA-sulfur-modification-protein-DndB-[Gloeothece-verrucosa]	2546359
WP_015227019.1	DndB-ParB-nuclease	GCF_000317635.1	272	Bacteria>Terrabacteria_group>Cyanobacteria/Melainabacteria_group>Cyanobacteria>Oscillatoriophycideae	Halothece_sp._PCC_7418	DNA-sulfur-modification-protein-DndB-[Halothece-sp.-PCC-7418]	65093
MBC6474592.1	DndB-ParB-nuclease	GCA_014323895.1	140	Bacteria>Terrabacteria_group>Cyanobacteria/Melainabacteria_group>Cyanobacteria>Oscillatoriophycideae	Hormoscilla_sp._GM102CHS1	DGQHR-domain-containing-protein-[Hormoscilla-sp.-GM102CHS1]	2653676
MBC6481181.1	DndB-ParB-nuclease	GCA_014323675.1	534	Bacteria>Terrabacteria_group>Cyanobacteria/Melainabacteria_group>Cyanobacteria>Oscillatoriophycideae	Hormoscilla_sp._GM7CHS1pb	DGQHR-domain-containing-protein-[Hormoscilla-sp.-GM7CHS1pb]	2653677
MBC6481180.1	DndB-ParB-nuclease	GCA_014323675.1	394	Bacteria>Terrabacteria_group>Cyanobacteria/Melainabacteria_group>Cyanobacteria>Oscillatoriophycideae	Hormoscilla_sp._GM7CHS1pb	DNA-sulfur-modification-protein-DndB-[Hormoscilla-sp.-GM7CHS1pb]	2653677
MBC6422703.1	DndB-ParB-nuclease	GCA_014324065.1	136	Bacteria>Terrabacteria_group>Cyanobacteria/Melainabacteria_group>Cyanobacteria>Oscillatoriophycideae	Hormoscilla_sp._SP12CHS1	DGQHR-domain-containing-protein-[Hormoscilla-sp.-SP12CHS1]	2653674
WP_007353671.1	DndB-ParB-nuclease	GCF_000180455.1	531	Bacteria>Terrabacteria_group>Cyanobacteria/Melainabacteria_group>Cyanobacteria>Oscillatoriophycideae	Kamptonema	MULTISPECIES:-DGQHR-domain-containing-protein-[Kamptonema]	1501433
WP_007353672.1	DndB-ParB-nuclease	GCF_000180455.1	349	Bacteria>Terrabacteria_group>Cyanobacteria/Melainabacteria_group>Cyanobacteria>Oscillatoriophycideae	Kamptonema	MULTISPECIES:-DNA-sulfur-modification-protein-DndB-[Kamptonema]	1501433
WP_007353673.1	DndB-ParB-nuclease	GCF_000180455.1	388	Bacteria>Terrabacteria_group>Cyanobacteria/Melainabacteria_group>Cyanobacteria>Oscillatoriophycideae	Kamptonema	MULTISPECIES:-DNA-sulfur-modification-protein-DndB-[Kamptonema]	1501433
CBN54446.1	DndB-ParB-nuclease	GCA_000180455.1	531	Bacteria>Terrabacteria_group>Cyanobacteria/Melainabacteria_group>Cyanobacteria>Oscillatoriophycideae	Kamptonema_sp._PCC_6506	conserved-hypothetical-protein-[Kamptonema-sp.-PCC-6506]	272129
CBN54448.1	DndB-ParB-nuclease	GCA_000180455.1	388	Bacteria>Terrabacteria_group>Cyanobacteria/Melainabacteria_group>Cyanobacteria>Oscillatoriophycideae	Kamptonema_sp._PCC_6506	conserved-hypothetical-protein-[Kamptonema-sp.-PCC-6506]	272129
CBN54447.1	DndB-ParB-nuclease	GCA_000180455.1	349	Bacteria>Terrabacteria_group>Cyanobacteria/Melainabacteria_group>Cyanobacteria>Oscillatoriophycideae	Kamptonema_sp._PCC_6506	DNA-sulfur-modification-protein-DndB-[Kamptonema-sp.-PCC-6506]	272129
NET86747.1	DndB-ParB-nuclease	GCA_010672865.1	534	Bacteria>Terrabacteria_group>Cyanobacteria/Melainabacteria_group>Cyanobacteria>Oscillatoriophycideae	Kamptonema_sp._SIO1D9	DGQHR-domain-containing-protein-[Kamptonema-sp.-SIO1D9]	2607808
WP_008051689.1	DndB-ParB-nuclease	GCF_000176895.2	526	Bacteria>Terrabacteria_group>Cyanobacteria/Melainabacteria_group>Cyanobacteria>Oscillatoriophycideae	Limnospira_indica	DGQHR-domain-containing-protein-[Limnospira-indica]	147322
WP_008051688.1	DndB-ParB-nuclease	GCF_000176895.2	393	Bacteria>Terrabacteria_group>Cyanobacteria/Melainabacteria_group>Cyanobacteria>Oscillatoriophycideae	Limnospira_indica	DNA-sulfur-modification-protein-DndB-[Limnospira-indica]	147322
WP_006670170.1	DndB-ParB-nuclease	GCF_000173555.1	526	Bacteria>Terrabacteria_group>Cyanobacteria/Melainabacteria_group>Cyanobacteria>Oscillatoriophycideae	Limnospira_maxima	MULTISPECIES:-DGQHR-domain-containing-protein-[Microcoleaceae]	129910
WP_006670169.1	DndB-ParB-nuclease	GCF_000173555.1	393	Bacteria>Terrabacteria_group>Cyanobacteria/Melainabacteria_group>Cyanobacteria>Oscillatoriophycideae	Limnospira_maxima	MULTISPECIES:-DNA-sulfur-modification-protein-DndB-[Microcoleaceae]	129910
WP_023069167.1	DndB-ParB-nuclease	GCF_000478195.2	387	Bacteria>Terrabacteria_group>Cyanobacteria/Melainabacteria_group>Cyanobacteria>Oscillatoriophycideae	Lyngbya_aestuarii	DGQHR-domain-containing-protein,-partial-[Lyngbya-aestuarii]	118322
MBW4692621.1	DndB-ParB-nuclease	GCA_019359375.1	358	Bacteria>Terrabacteria_group>Cyanobacteria/Melainabacteria_group>Cyanobacteria>Oscillatoriophycideae	Lyngbya_sp._HA4199-MV5	DNA-sulfur-modification-protein-DndB-[Lyngbya-sp.-HA4199-MV5]	2835090
MBW4692623.1	DndB-ParB-nuclease	GCA_019359375.1	585	Bacteria>Terrabacteria_group>Cyanobacteria/Melainabacteria_group>Cyanobacteria>Oscillatoriophycideae	Lyngbya_sp._HA4199-MV5	hypothetical-protein-[Lyngbya-sp.-HA4199-MV5]	2835090
WP_009786704.1	DndB-ParB-nuclease	GCF_000169095.1	524	Bacteria>Terrabacteria_group>Cyanobacteria/Melainabacteria_group>Cyanobacteria>Oscillatoriophycideae	Lyngbya_sp._PCC_8106	DGQHR-domain-containing-protein-[Lyngbya-sp.-PCC-8106]	313612
HCV30568.1	DndB-ParB-nuclease	GCA_003542015.1	379	Bacteria>Terrabacteria_group>Cyanobacteria/Melainabacteria_group>Cyanobacteria>Oscillatoriophycideae	Microcoleaceae_bacterium_UBA9251	DNA-sulfur-modification-protein-DndB-[Microcoleaceae-bacterium-UBA9251]	2055794
HCV30567.1	DndB-ParB-nuclease	GCA_003542015.1	534	Bacteria>Terrabacteria_group>Cyanobacteria/Melainabacteria_group>Cyanobacteria>Oscillatoriophycideae	Microcoleaceae_bacterium_UBA9251	hypothetical-protein-[Microcoleaceae-bacterium-UBA9251]	2055794
WP_172191411.1	DndB-ParB-nuclease	GCF_013179805.1	530	Bacteria>Terrabacteria_group>Cyanobacteria/Melainabacteria_group>Cyanobacteria>Oscillatoriophycideae	Microcoleus_asticus	DGQHR-domain-containing-protein-[Microcoleus-asticus]	2815231
WP_172191409.1	DndB-ParB-nuclease	GCF_013179805.1	370	Bacteria>Terrabacteria_group>Cyanobacteria/Melainabacteria_group>Cyanobacteria>Oscillatoriophycideae	Microcoleus_asticus	DNA-sulfur-modification-protein-DndB-[Microcoleus-asticus]	2815231
MBD2125671.1	DndB-ParB-nuclease	GCA_014696155.1	532	Bacteria>Terrabacteria_group>Cyanobacteria/Melainabacteria_group>Cyanobacteria>Oscillatoriophycideae	Microcoleus_sp._FACHB-1	DGQHR-domain-containing-protein-[Microcoleus-sp.-FACHB-1]	2692820
WP_190776509.1	DndB-ParB-nuclease	GCF_014696155.1	532	Bacteria>Terrabacteria_group>Cyanobacteria/Melainabacteria_group>Cyanobacteria>Oscillatoriophycideae	Microcoleus_sp._FACHB-1	DGQHR-domain-containing-protein-[Microcoleus-sp.-FACHB-1]	2692820
MBD2125670.1	DndB-ParB-nuclease	GCA_014696155.1	382	Bacteria>Terrabacteria_group>Cyanobacteria/Melainabacteria_group>Cyanobacteria>Oscillatoriophycideae	Microcoleus_sp._FACHB-1	DNA-sulfur-modification-protein-DndB-[Microcoleus-sp.-FACHB-1]	2692820
WP_190776508.1	DndB-ParB-nuclease	GCF_014696155.1	382	Bacteria>Terrabacteria_group>Cyanobacteria/Melainabacteria_group>Cyanobacteria>Oscillatoriophycideae	Microcoleus_sp._FACHB-1	DNA-sulfur-modification-protein-DndB-[Microcoleus-sp.-FACHB-1]	2692820
MBD2012752.1	DndB-ParB-nuclease	GCA_014695895.1	532	Bacteria>Terrabacteria_group>Cyanobacteria/Melainabacteria_group>Cyanobacteria>Oscillatoriophycideae	Microcoleus_sp._FACHB-53	DGQHR-domain-containing-protein-[Microcoleus-sp.-FACHB-53]	2692823
WP_190493966.1	DndB-ParB-nuclease	GCF_014695895.1	532	Bacteria>Terrabacteria_group>Cyanobacteria/Melainabacteria_group>Cyanobacteria>Oscillatoriophycideae	Microcoleus_sp._FACHB-53	DGQHR-domain-containing-protein-[Microcoleus-sp.-FACHB-53]	2692823
MBD2012753.1	DndB-ParB-nuclease	GCA_014695895.1	374	Bacteria>Terrabacteria_group>Cyanobacteria/Melainabacteria_group>Cyanobacteria>Oscillatoriophycideae	Microcoleus_sp._FACHB-53	DNA-sulfur-modification-protein-DndB-[Microcoleus-sp.-FACHB-53]	2692823
WP_190493967.1	DndB-ParB-nuclease	GCF_014695895.1	374	Bacteria>Terrabacteria_group>Cyanobacteria/Melainabacteria_group>Cyanobacteria>Oscillatoriophycideae	Microcoleus_sp._FACHB-53	DNA-sulfur-modification-protein-DndB-[Microcoleus-sp.-FACHB-53]	2692823
MBD1828882.1	DndB-ParB-nuclease	GCA_014695295.1	530	Bacteria>Terrabacteria_group>Cyanobacteria/Melainabacteria_group>Cyanobacteria>Oscillatoriophycideae	Microcoleus_sp._FACHB-61	DGQHR-domain-containing-protein-[Microcoleus-sp.-FACHB-61]	2692824
MBD1828881.1	DndB-ParB-nuclease	GCA_014695295.1	370	Bacteria>Terrabacteria_group>Cyanobacteria/Melainabacteria_group>Cyanobacteria>Oscillatoriophycideae	Microcoleus_sp._FACHB-61	DNA-sulfur-modification-protein-DndB-[Microcoleus-sp.-FACHB-61]	2692824
MBD2043093.1	DndB-ParB-nuclease	GCA_014695725.1	534	Bacteria>Terrabacteria_group>Cyanobacteria/Melainabacteria_group>Cyanobacteria>Oscillatoriophycideae	Microcoleus_sp._FACHB-672	DGQHR-domain-containing-protein-[Microcoleus-sp.-FACHB-672]	2692825
WP_190672096.1	DndB-ParB-nuclease	GCF_014695725.1	534	Bacteria>Terrabacteria_group>Cyanobacteria/Melainabacteria_group>Cyanobacteria>Oscillatoriophycideae	Microcoleus_sp._FACHB-672	DGQHR-domain-containing-protein-[Microcoleus-sp.-FACHB-672]	2692825
MBD2043094.1	DndB-ParB-nuclease	GCA_014695725.1	350	Bacteria>Terrabacteria_group>Cyanobacteria/Melainabacteria_group>Cyanobacteria>Oscillatoriophycideae	Microcoleus_sp._FACHB-672	DNA-sulfur-modification-protein-DndB-[Microcoleus-sp.-FACHB-672]	2692825
MBD2043096.1	DndB-ParB-nuclease	GCA_014695725.1	417	Bacteria>Terrabacteria_group>Cyanobacteria/Melainabacteria_group>Cyanobacteria>Oscillatoriophycideae	Microcoleus_sp._FACHB-672	DNA-sulfur-modification-protein-DndB-[Microcoleus-sp.-FACHB-672]	2692825
WP_190672098.1	DndB-ParB-nuclease	GCF_014695725.1	350	Bacteria>Terrabacteria_group>Cyanobacteria/Melainabacteria_group>Cyanobacteria>Oscillatoriophycideae	Microcoleus_sp._FACHB-672	DNA-sulfur-modification-protein-DndB-[Microcoleus-sp.-FACHB-672]	2692825
WP_190672105.1	DndB-ParB-nuclease	GCF_014695725.1	417	Bacteria>Terrabacteria_group>Cyanobacteria/Melainabacteria_group>Cyanobacteria>Oscillatoriophycideae	Microcoleus_sp._FACHB-672	DNA-sulfur-modification-protein-DndB-[Microcoleus-sp.-FACHB-672]	2692825
MBD1936332.1	DndB-ParB-nuclease	GCA_014695715.1	534	Bacteria>Terrabacteria_group>Cyanobacteria/Melainabacteria_group>Cyanobacteria>Oscillatoriophycideae	Microcoleus_sp._FACHB-68	DGQHR-domain-containing-protein-[Microcoleus-sp.-FACHB-68]	2692826
WP_190757373.1	DndB-ParB-nuclease	GCF_014695715.1	534	Bacteria>Terrabacteria_group>Cyanobacteria/Melainabacteria_group>Cyanobacteria>Oscillatoriophycideae	Microcoleus_sp._FACHB-68	DGQHR-domain-containing-protein-[Microcoleus-sp.-FACHB-68]	2692826
MBD1936330.1	DndB-ParB-nuclease	GCA_014695715.1	351	Bacteria>Terrabacteria_group>Cyanobacteria/Melainabacteria_group>Cyanobacteria>Oscillatoriophycideae	Microcoleus_sp._FACHB-68	DNA-sulfur-modification-protein-DndB-[Microcoleus-sp.-FACHB-68]	2692826
WP_190757371.1	DndB-ParB-nuclease	GCF_014695715.1	351	Bacteria>Terrabacteria_group>Cyanobacteria/Melainabacteria_group>Cyanobacteria>Oscillatoriophycideae	Microcoleus_sp._FACHB-68	DNA-sulfur-modification-protein-DndB-[Microcoleus-sp.-FACHB-68]	2692826
MBD1936329.1	DndB-ParB-nuclease	GCA_014695715.1	578	Bacteria>Terrabacteria_group>Cyanobacteria/Melainabacteria_group>Cyanobacteria>Oscillatoriophycideae	Microcoleus_sp._FACHB-68	hypothetical-protein-[Microcoleus-sp.-FACHB-68]	2692826
WP_190757370.1	DndB-ParB-nuclease	GCF_014695715.1	578	Bacteria>Terrabacteria_group>Cyanobacteria/Melainabacteria_group>Cyanobacteria>Oscillatoriophycideae	Microcoleus_sp._FACHB-68	hypothetical-protein-[Microcoleus-sp.-FACHB-68]	2692826
MBD1922218.1	DndB-ParB-nuclease	GCA_014695585.1	387	Bacteria>Terrabacteria_group>Cyanobacteria/Melainabacteria_group>Cyanobacteria>Oscillatoriophycideae	Microcoleus_sp._FACHB-831	DGQHR-domain-containing-protein-[Microcoleus-sp.-FACHB-831]	2692827
MBD1922220.1	DndB-ParB-nuclease	GCA_014695585.1	532	Bacteria>Terrabacteria_group>Cyanobacteria/Melainabacteria_group>Cyanobacteria>Oscillatoriophycideae	Microcoleus_sp._FACHB-831	DGQHR-domain-containing-protein-[Microcoleus-sp.-FACHB-831]	2692827
MBD1919660.1	DndB-ParB-nuclease	GCA_014695585.1	385	Bacteria>Terrabacteria_group>Cyanobacteria/Melainabacteria_group>Cyanobacteria>Oscillatoriophycideae	Microcoleus_sp._FACHB-831	DNA-sulfur-modification-protein-DndB-[Microcoleus-sp.-FACHB-831]	2692827
WP_190484360.1	DndB-ParB-nuclease	GCF_014695585.1	385	Bacteria>Terrabacteria_group>Cyanobacteria/Melainabacteria_group>Cyanobacteria>Oscillatoriophycideae	Microcoleus_sp._FACHB-831	DNA-sulfur-modification-protein-DndB-[Microcoleus-sp.-FACHB-831]	2692827
MBD1885157.1	DndB-ParB-nuclease	GCA_014695525.1	530	Bacteria>Terrabacteria_group>Cyanobacteria/Melainabacteria_group>Cyanobacteria>Oscillatoriophycideae	Microcoleus_sp._FACHB-84	DGQHR-domain-containing-protein-[Microcoleus-sp.-FACHB-84]	2692828
MBD1885156.1	DndB-ParB-nuclease	GCA_014695525.1	378	Bacteria>Terrabacteria_group>Cyanobacteria/Melainabacteria_group>Cyanobacteria>Oscillatoriophycideae	Microcoleus_sp._FACHB-84	DNA-sulfur-modification-protein-DndB-[Microcoleus-sp.-FACHB-84]	2692828
MBD1809248.1	DndB-ParB-nuclease	GCA_014849525.1	355	Bacteria>Terrabacteria_group>Cyanobacteria/Melainabacteria_group>Cyanobacteria>Oscillatoriophycideae	Microcoleus_sp._FACHB-SPT15	DNA-sulfur-modification-protein-DndB-[Microcoleus-sp.-FACHB-SPT15]	2692830
WP_192221989.1	DndB-ParB-nuclease	GCF_014849525.1	355	Bacteria>Terrabacteria_group>Cyanobacteria/Melainabacteria_group>Cyanobacteria>Oscillatoriophycideae	Microcoleus_sp._FACHB-SPT15	DNA-sulfur-modification-protein-DndB-[Microcoleus-sp.-FACHB-SPT15]	2692830
MBD1809246.1	DndB-ParB-nuclease	GCA_014849525.1	670	Bacteria>Terrabacteria_group>Cyanobacteria/Melainabacteria_group>Cyanobacteria>Oscillatoriophycideae	Microcoleus_sp._FACHB-SPT15	hypothetical-protein-[Microcoleus-sp.-FACHB-SPT15]	2692830
WP_192221987.1	DndB-ParB-nuclease	GCF_014849525.1	670	Bacteria>Terrabacteria_group>Cyanobacteria/Melainabacteria_group>Cyanobacteria>Oscillatoriophycideae	Microcoleus_sp._FACHB-SPT15	hypothetical-protein-[Microcoleus-sp.-FACHB-SPT15]	2692830
MBE9186975.1	DndB-ParB-nuclease	GCA_015207455.1	530	Bacteria>Terrabacteria_group>Cyanobacteria/Melainabacteria_group>Cyanobacteria>Oscillatoriophycideae	Microcoleus_sp._LEGE_07076	DGQHR-domain-containing-protein-[Microcoleus-sp.-LEGE-07076]	915322
MCC3424607.1	DndB-ParB-nuclease	GCA_020738945.1	530	Bacteria>Terrabacteria_group>Cyanobacteria/Melainabacteria_group>Cyanobacteria>Oscillatoriophycideae	Microcoleus_sp._PH2017_01_SCD_O_A	DGQHR-domain-containing-protein-[Microcoleus-sp.-PH2017_01_SCD_O_A]	2798812

Accession	Annotation	GCA	Length	Phyletic.spread	Strain.name	Defline	Species.Taxid
MCC3424608.1	DndB-ParB-nuclease	GCA_020738945.1	392	Bacteria>Terrabacteria_group>Cyanobacteria/Melainabacteria_group>Cyanobacteria>Oscillatoriophycideae	Microcoleus_sp._PH2017_01_SCD_O_A	DNA-sulfur-modification-protein-DndB-[Microcoleus-sp.-PH2017_01_SCD_O_A]	2798812
MCC3411999.1	DndB-ParB-nuclease	GCA_020738905.1	530	Bacteria>Terrabacteria_group>Cyanobacteria/Melainabacteria_group>Cyanobacteria>Oscillatoriophycideae	Microcoleus_sp._PH2017_02_FOX_O_A	DGQHR-domain-containing-protein-[Microcoleus-sp.-PH2017_02_FOX_O_A]	2798813
MCC3411998.1	DndB-ParB-nuclease	GCA_020738905.1	392	Bacteria>Terrabacteria_group>Cyanobacteria/Melainabacteria_group>Cyanobacteria>Oscillatoriophycideae	Microcoleus_sp._PH2017_02_FOX_O_A	DNA-sulfur-modification-protein-DndB-[Microcoleus-sp.-PH2017_02_FOX_O_A]	2798813
MCC3430558.1	DndB-ParB-nuclease	GCA_020738835.1	530	Bacteria>Terrabacteria_group>Cyanobacteria/Melainabacteria_group>Cyanobacteria>Oscillatoriophycideae	Microcoleus_sp._PH2017_04_SCI_O_A	DGQHR-domain-containing-protein-[Microcoleus-sp.-PH2017_04_SCI_O_A]	2798815
MCC3430559.1	DndB-ParB-nuclease	GCA_020738835.1	392	Bacteria>Terrabacteria_group>Cyanobacteria/Melainabacteria_group>Cyanobacteria>Oscillatoriophycideae	Microcoleus_sp._PH2017_04_SCI_O_A	DNA-sulfur-modification-protein-DndB-[Microcoleus-sp.-PH2017_04_SCI_O_A]	2798815
MCC3438980.1	DndB-ParB-nuclease	GCA_020738785.1	530	Bacteria>Terrabacteria_group>Cyanobacteria/Melainabacteria_group>Cyanobacteria>Oscillatoriophycideae	Microcoleus_sp._PH2017_05_CCC_O_A	DGQHR-domain-containing-protein-[Microcoleus-sp.-PH2017_05_CCC_O_A]	2798816
MCC3438981.1	DndB-ParB-nuclease	GCA_020738785.1	366	Bacteria>Terrabacteria_group>Cyanobacteria/Melainabacteria_group>Cyanobacteria>Oscillatoriophycideae	Microcoleus_sp._PH2017_05_CCC_O_A	DNA-sulfur-modification-protein-DndB-[Microcoleus-sp.-PH2017_05_CCC_O_A]	2798816
MCC3418689.1	DndB-ParB-nuclease	GCA_020738685.1	530	Bacteria>Terrabacteria_group>Cyanobacteria/Melainabacteria_group>Cyanobacteria>Oscillatoriophycideae	Microcoleus_sp._PH2017_07_MST_O_A	DGQHR-domain-containing-protein-[Microcoleus-sp.-PH2017_07_MST_O_A]	2798818
MCC3418690.1	DndB-ParB-nuclease	GCA_020738685.1	362	Bacteria>Terrabacteria_group>Cyanobacteria/Melainabacteria_group>Cyanobacteria>Oscillatoriophycideae	Microcoleus_sp._PH2017_07_MST_O_A	DNA-sulfur-modification-protein-DndB-[Microcoleus-sp.-PH2017_07_MST_O_A]	2798818
MCC3418691.1	DndB-ParB-nuclease	GCA_020738685.1	392	Bacteria>Terrabacteria_group>Cyanobacteria/Melainabacteria_group>Cyanobacteria>Oscillatoriophycideae	Microcoleus_sp._PH2017_07_MST_O_A	DNA-sulfur-modification-protein-DndB-[Microcoleus-sp.-PH2017_07_MST_O_A]	2798818
MCC3454903.1	DndB-ParB-nuclease	GCA_020738665.1	530	Bacteria>Terrabacteria_group>Cyanobacteria/Melainabacteria_group>Cyanobacteria>Oscillatoriophycideae	Microcoleus_sp._PH2017_08_TRC_O_A	DGQHR-domain-containing-protein-[Microcoleus-sp.-PH2017_08_TRC_O_A]	2798819
MCC3454902.1	DndB-ParB-nuclease	GCA_020738665.1	392	Bacteria>Terrabacteria_group>Cyanobacteria/Melainabacteria_group>Cyanobacteria>Oscillatoriophycideae	Microcoleus_sp._PH2017_08_TRC_O_A	DNA-sulfur-modification-protein-DndB-[Microcoleus-sp.-PH2017_08_TRC_O_A]	2798819
MCC3448142.1	DndB-ParB-nuclease	GCA_020738625.1	530	Bacteria>Terrabacteria_group>Cyanobacteria/Melainabacteria_group>Cyanobacteria>Oscillatoriophycideae	Microcoleus_sp._PH2017_09_SFU_O_A	DGQHR-domain-containing-protein-[Microcoleus-sp.-PH2017_09_SFU_O_A]	2798820
MCC3448141.1	DndB-ParB-nuclease	GCA_020738625.1	365	Bacteria>Terrabacteria_group>Cyanobacteria/Melainabacteria_group>Cyanobacteria>Oscillatoriophycideae	Microcoleus_sp._PH2017_09_SFU_O_A	DNA-sulfur-modification-protein-DndB-[Microcoleus-sp.-PH2017_09_SFU_O_A]	2798820
MCC3404951.1	DndB-ParB-nuclease	GCA_020738635.1	530	Bacteria>Terrabacteria_group>Cyanobacteria/Melainabacteria_group>Cyanobacteria>Oscillatoriophycideae	Microcoleus_sp._PH2017_10_PVI_O_A	DGQHR-domain-containing-protein-[Microcoleus-sp.-PH2017_10_PVI_O_A]	2798821
MCC3404952.1	DndB-ParB-nuclease	GCA_020738635.1	380	Bacteria>Terrabacteria_group>Cyanobacteria/Melainabacteria_group>Cyanobacteria>Oscillatoriophycideae	Microcoleus_sp._PH2017_10_PVI_O_A	DNA-sulfur-modification-protein-DndB-[Microcoleus-sp.-PH2017_10_PVI_O_A]	2798821
MCC3473247.1	DndB-ParB-nuclease	GCA_020738505.1	530	Bacteria>Terrabacteria_group>Cyanobacteria/Melainabacteria_group>Cyanobacteria>Oscillatoriophycideae	Microcoleus_sp._PH2017_13_LAR_U_A	DGQHR-domain-containing-protein-[Microcoleus-sp.-PH2017_13_LAR_U_A]	2798824
MCC3473248.1	DndB-ParB-nuclease	GCA_020738505.1	392	Bacteria>Terrabacteria_group>Cyanobacteria/Melainabacteria_group>Cyanobacteria>Oscillatoriophycideae	Microcoleus_sp._PH2017_13_LAR_U_A	DNA-sulfur-modification-protein-DndB-[Microcoleus-sp.-PH2017_13_LAR_U_A]	2798824
MCC3541813.1	DndB-ParB-nuclease	GCA_020738275.1	530	Bacteria>Terrabacteria_group>Cyanobacteria/Melainabacteria_group>Cyanobacteria>Oscillatoriophycideae	Microcoleus_sp._PH2017_22_RUC_O_B	DGQHR-domain-containing-protein-[Microcoleus-sp.-PH2017_22_RUC_O_B]	2798833
MCC3541812.1	DndB-ParB-nuclease	GCA_020738275.1	380	Bacteria>Terrabacteria_group>Cyanobacteria/Melainabacteria_group>Cyanobacteria>Oscillatoriophycideae	Microcoleus_sp._PH2017_22_RUC_O_B	DNA-sulfur-modification-protein-DndB-[Microcoleus-sp.-PH2017_22_RUC_O_B]	2798833
MCC3585697.1	DndB-ParB-nuclease	GCA_020737965.1	530	Bacteria>Terrabacteria_group>Cyanobacteria/Melainabacteria_group>Cyanobacteria>Oscillatoriophycideae	Microcoleus_sp._PH2017_30_WIL_O_A	DGQHR-domain-containing-protein-[Microcoleus-sp.-PH2017_30_WIL_O_A]	2798840
MCC3585698.1	DndB-ParB-nuclease	GCA_020737965.1	392	Bacteria>Terrabacteria_group>Cyanobacteria/Melainabacteria_group>Cyanobacteria>Oscillatoriophycideae	Microcoleus_sp._PH2017_30_WIL_O_A	DNA-sulfur-modification-protein-DndB-[Microcoleus-sp.-PH2017_30_WIL_O_A]	2798840
MCC3570161.1	DndB-ParB-nuclease	GCA_020737885.1	530	Bacteria>Terrabacteria_group>Cyanobacteria/Melainabacteria_group>Cyanobacteria>Oscillatoriophycideae	Microcoleus_sp._PH2017_34_RAT_O_A	DGQHR-domain-containing-protein-[Microcoleus-sp.-PH2017_34_RAT_O_A]	2798844
MCC3570160.1	DndB-ParB-nuclease	GCA_020737885.1	365	Bacteria>Terrabacteria_group>Cyanobacteria/Melainabacteria_group>Cyanobacteria>Oscillatoriophycideae	Microcoleus_sp._PH2017_34_RAT_O_A	DNA-sulfur-modification-protein-DndB-[Microcoleus-sp.-PH2017_34_RAT_O_A]	2798844
NJK66771.1	DndB-ParB-nuclease	GCA_012030835.1	529	Bacteria>Terrabacteria_group>Cyanobacteria/Melainabacteria_group>Cyanobacteria>Oscillatoriophycideae	Microcoleus_sp._SU_5_3	DGQHR-domain-containing-protein-[Microcoleus-sp.-SU_5_3]	2720482
NJK68553.1	DndB-ParB-nuclease	GCA_012030835.1	376	Bacteria>Terrabacteria_group>Cyanobacteria/Melainabacteria_group>Cyanobacteria>Oscillatoriophycideae	Microcoleus_sp._SU_5_3	DNA-sulfur-modification-protein-DndB-[Microcoleus-sp.-SU_5_3]	2720482
MBD0308700.1	DndB-ParB-nuclease	GCA_014534015.1	156	Bacteria>Terrabacteria_group>Cyanobacteria/Melainabacteria_group>Cyanobacteria>Oscillatoriophycideae	Microcoleus_sp._T1-bin1	hypothetical-protein-[Microcoleus-sp.-T1-bin1]	2769071
WP_006632977.1	DndB-ParB-nuclease	GCF_022701275.1	530	Bacteria>Terrabacteria_group>Cyanobacteria/Melainabacteria_group>Cyanobacteria>Oscillatoriophycideae	Microcoleus_vaginatus	DGQHR-domain-containing-protein-[Microcoleus-vaginatus]	119532
WP_242723686.1	DndB-ParB-nuclease	GCF_022701255.1	530	Bacteria>Terrabacteria_group>Cyanobacteria/Melainabacteria_group>Cyanobacteria>Oscillatoriophycideae	Microcoleus_vaginatus	DGQHR-domain-containing-protein-[Microcoleus-vaginatus]	119532
WP_006632974.1	DndB-ParB-nuclease	GCF_022701275.1	392	Bacteria>Terrabacteria_group>Cyanobacteria/Melainabacteria_group>Cyanobacteria>Oscillatoriophycideae	Microcoleus_vaginatus	DNA-sulfur-modification-protein-DndB-[Microcoleus-vaginatus]	119532
WP_006632976.1	DndB-ParB-nuclease	GCF_022701275.1	373	Bacteria>Terrabacteria_group>Cyanobacteria/Melainabacteria_group>Cyanobacteria>Oscillatoriophycideae	Microcoleus_vaginatus	DNA-sulfur-modification-protein-DndB-[Microcoleus-vaginatus]	119532
WP_242723684.1	DndB-ParB-nuclease	GCF_022701255.1	370	Bacteria>Terrabacteria_group>Cyanobacteria/Melainabacteria_group>Cyanobacteria>Oscillatoriophycideae	Microcoleus_vaginatus	DNA-sulfur-modification-protein-DndB-[Microcoleus-vaginatus]	119532
EGK88192.1	DndB-ParB-nuclease	GCA_000214075.2	392	Bacteria>Terrabacteria_group>Cyanobacteria/Melainabacteria_group>Cyanobacteria>Oscillatoriophycideae	Microcoleus_vaginatus_FGP-2	DGQHR-domain-protein-[Microcoleus-vaginatus-FGP-2]	756067
EGK88195.1	DndB-ParB-nuclease	GCA_000214075.2	530	Bacteria>Terrabacteria_group>Cyanobacteria/Melainabacteria_group>Cyanobacteria>Oscillatoriophycideae	Microcoleus_vaginatus_FGP-2	DGQHR-domain-protein-[Microcoleus-vaginatus-FGP-2]	756067
EGK88194.1	DndB-ParB-nuclease	GCA_000214075.2	373	Bacteria>Terrabacteria_group>Cyanobacteria/Melainabacteria_group>Cyanobacteria>Oscillatoriophycideae	Microcoleus_vaginatus_FGP-2	DNA-sulfur-modification-protein-DndB-[Microcoleus-vaginatus-FGP-2]	756067
MBW4679699.1	DndB-ParB-nuclease	GCA_019359355.1	534	Bacteria>Terrabacteria_group>Cyanobacteria/Melainabacteria_group>Cyanobacteria>Oscillatoriophycideae	Microcoleus_vaginatus_WJT46-NPBG5	DGQHR-domain-containing-protein-[Microcoleus-vaginatus-WJT46-NPBG5]	2839660
MBW4679700.1	DndB-ParB-nuclease	GCA_019359355.1	350	Bacteria>Terrabacteria_group>Cyanobacteria/Melainabacteria_group>Cyanobacteria>Oscillatoriophycideae	Microcoleus_vaginatus_WJT46-NPBG5	DNA-sulfur-modification-protein-DndB-[Microcoleus-vaginatus-WJT46-NPBG5]	2839660
MCA2584640.1	DndB-ParB-nuclease	GCA_020245085.1	517	Bacteria>Terrabacteria_group>Cyanobacteria/Melainabacteria_group>Cyanobacteria>Oscillatoriophycideae	Microcystis_sp._M34BS1	hypothetical-protein-[Microcystis-sp.-M34BS1]	2771187
WP_226590119.1	DndB-ParB-nuclease	GCF_020521235.1	533	Bacteria>Terrabacteria_group>Cyanobacteria/Melainabacteria_group>Cyanobacteria>Oscillatoriophycideae	Microseira_wollei	DGQHR-domain-containing-protein-[Microseira-wollei]	467598
WP_226590117.1	DndB-ParB-nuclease	GCF_020521235.1	367	Bacteria>Terrabacteria_group>Cyanobacteria/Melainabacteria_group>Cyanobacteria>Oscillatoriophycideae	Microseira_wollei	DNA-sulfur-modification-protein-DndB-[Microseira-wollei]	467598
WP_075900188.1	DndB-ParB-nuclease	GCF_001942495.1	533	Bacteria>Terrabacteria_group>Cyanobacteria/Melainabacteria_group>Cyanobacteria>Oscillatoriophycideae	Moorena_bouillonii	DGQHR-domain-containing-protein-[Moorea-bouillonii]	207920
OLT60176.1	DndB-ParB-nuclease	GCA_001942495.1	533	Bacteria>Terrabacteria_group>Cyanobacteria/Melainabacteria_group>Cyanobacteria>Oscillatoriophycideae	Moorena_bouillonii_PNG	hypothetical-protein-BJP37_15245-[Moorena-bouillonii-PNG]	568701
EGJ29463.1	DndB-ParB-nuclease	GCA_000211815.1	533	Bacteria>Terrabacteria_group>Cyanobacteria/Melainabacteria_group>Cyanobacteria>Oscillatoriophycideae	Moorena_producens_3L	DGQHR-domain-protein-[Moorena-producens-3L]	489825
OLT67932.1	DndB-ParB-nuclease	GCA_001942475.1	533	Bacteria>Terrabacteria_group>Cyanobacteria/Melainabacteria_group>Cyanobacteria>Oscillatoriophycideae	Moorena_producens_3L	hypothetical-protein-BI334_25490-[Moorena-producens-3L]	489825
WP_008189444.1	DndB-ParB-nuclease	GCF_000211815.1	533	Bacteria>Terrabacteria_group>Cyanobacteria/Melainabacteria_group>Cyanobacteria>Oscillatoriophycideae	Moorena_producens	DGQHR-domain-containing-protein-[Moorea-producens]	1155739
WP_070393352.1	DndB-ParB-nuclease	GCF_001767235.1	533	Bacteria>Terrabacteria_group>Cyanobacteria/Melainabacteria_group>Cyanobacteria>Oscillatoriophycideae	Moorena_producens	DGQHR-domain-containing-protein-[Moorea-producens]	1155739
WP_071104691.1	DndB-ParB-nuclease	GCF_001854205.1	533	Bacteria>Terrabacteria_group>Cyanobacteria/Melainabacteria_group>Cyanobacteria>Oscillatoriophycideae	Moorena_producens	DGQHR-domain-containing-protein-[Moorea-producens]	1155739
AOY81349.1	DndB-ParB-nuclease	GCA_001854205.1	533	Bacteria>Terrabacteria_group>Cyanobacteria/Melainabacteria_group>Cyanobacteria>Oscillatoriophycideae	Moorena_producens_JHB	hypothetical-protein-BJP36_16995-[Moorena-producens-JHB]	1454205
AOX00901.1	DndB-ParB-nuclease	GCA_001767235.1	533	Bacteria>Terrabacteria_group>Cyanobacteria/Melainabacteria_group>Cyanobacteria>Oscillatoriophycideae	Moorena_producens_PAL-8-15-08-1	hypothetical-protein-BJP34_16910-[Moorena-producens-PAL-8-15-08-1]	1458985
NET80947.1	DndB-ParB-nuclease	GCA_010672755.1	533	Bacteria>Terrabacteria_group>Cyanobacteria/Melainabacteria_group>Cyanobacteria>Oscillatoriophycideae	Moorena_sp._SIO1F2	DGQHR-domain-containing-protein-[Moorea-sp.-SIO1F2]	2607819
NET63034.1	DndB-ParB-nuclease	GCA_010672795.1	533	Bacteria>Terrabacteria_group>Cyanobacteria/Melainabacteria_group>Cyanobacteria>Oscillatoriophycideae	Moorena_sp._SIO1G6	DGQHR-domain-containing-protein-[Moorena-sp.-SIO1G6]	2607840
NEQ82060.1	DndB-ParB-nuclease	GCA_010672285.1	533	Bacteria>Terrabacteria_group>Cyanobacteria/Melainabacteria_group>Cyanobacteria>Oscillatoriophycideae	Moorena_sp._SIO2I5	DGQHR-domain-containing-protein-[Moorena-sp.-SIO2I5]	2607825
NEQ89164.1	DndB-ParB-nuclease	GCA_010672285.1	61	Bacteria>Terrabacteria_group>Cyanobacteria/Melainabacteria_group>Cyanobacteria>Oscillatoriophycideae	Moorena_sp._SIO2I5	hypothetical-protein-[Moorena-sp.-SIO2I5]	2607825
NER91759.1	DndB-ParB-nuclease	GCA_010672265.1	533	Bacteria>Terrabacteria_group>Cyanobacteria/Melainabacteria_group>Cyanobacteria>Oscillatoriophycideae	Moorena_sp._SIO3A2	DGQHR-domain-containing-protein-[Moorea-sp.-SIO3A2]	2607841
NEP68332.1	DndB-ParB-nuclease	GCA_010672205.1	533	Bacteria>Terrabacteria_group>Cyanobacteria/Melainabacteria_group>Cyanobacteria>Oscillatoriophycideae	Moorena_sp._SIO3A5	DGQHR-domain-containing-protein-[Moorena-sp.-SIO3A5]	2607822
NEP32084.1	DndB-ParB-nuclease	GCA_010692525.1	533	Bacteria>Terrabacteria_group>Cyanobacteria/Melainabacteria_group>Cyanobacteria>Oscillatoriophycideae	Moorena_sp._SIO3B2	DGQHR-domain-containing-protein-[Moorena-sp.-SIO3B2]	2607827
NEP48666.1	DndB-ParB-nuclease	GCA_010692465.1	533	Bacteria>Terrabacteria_group>Cyanobacteria/Melainabacteria_group>Cyanobacteria>Oscillatoriophycideae	Moorena_sp._SIO3C2	DGQHR-domain-containing-protein-[Moorea-sp.-SIO3C2]	2607842
NEO11344.1	DndB-ParB-nuclease	GCA_010692445.1	533	Bacteria>Terrabacteria_group>Cyanobacteria/Melainabacteria_group>Cyanobacteria>Oscillatoriophycideae	Moorena_sp._SIO3E8	DGQHR-domain-containing-protein-[Moorea-sp.-SIO3E8]	2607830
NEP99180.1	DndB-ParB-nuclease	GCA_010672105.1	533	Bacteria>Terrabacteria_group>Cyanobacteria/Melainabacteria_group>Cyanobacteria>Oscillatoriophycideae	Moorena_sp._SIO3F7	DGQHR-domain-containing-protein-[Moorena-sp.-SIO3F7]	2607839
NEO89124.1	DndB-ParB-nuclease	GCA_010692495.1	533	Bacteria>Terrabacteria_group>Cyanobacteria/Melainabacteria_group>Cyanobacteria>Oscillatoriophycideae	Moorena_sp._SIO3G5	DGQHR-domain-containing-protein-[Moorena-sp.-SIO3G5]	2607837
NEO71932.1	DndB-ParB-nuclease	GCA_010692395.1	533	Bacteria>Terrabacteria_group>Cyanobacteria/Melainabacteria_group>Cyanobacteria>Oscillatoriophycideae	Moorena_sp._SIO3H5	DGQHR-domain-containing-protein-[Moorena-sp.-SIO3H5]	2607834
NEP20767.1	DndB-ParB-nuclease	GCA_010672095.1	533	Bacteria>Terrabacteria_group>Cyanobacteria/Melainabacteria_group>Cyanobacteria>Oscillatoriophycideae	Moorena_sp._SIO3I6	DGQHR-domain-containing-protein-[Moorea-sp.-SIO3I6]	2607831
NEN96031.1	DndB-ParB-nuclease	GCA_010692375.1	187	Bacteria>Terrabacteria_group>Cyanobacteria/Melainabacteria_group>Cyanobacteria>Oscillatoriophycideae	Moorena_sp._SIO3I7	DGQHR-domain-containing-protein-[Moorena-sp.-SIO3I7]	2607832
NEN96030.1	DndB-ParB-nuclease	GCA_010692375.1	72	Bacteria>Terrabacteria_group>Cyanobacteria/Melainabacteria_group>Cyanobacteria>Oscillatoriophycideae	Moorena_sp._SIO3I7	hypothetical-protein-[Moorena-sp.-SIO3I7]	2607832
NEO07601.1	DndB-ParB-nuclease	GCA_010692325.1	533	Bacteria>Terrabacteria_group>Cyanobacteria/Melainabacteria_group>Cyanobacteria>Oscillatoriophycideae	Moorena_sp._SIO3I8	DGQHR-domain-containing-protein-[Moorea-sp.-SIO3I8]	2607833
NEQ57113.1	DndB-ParB-nuclease	GCA_010672065.1	533	Bacteria>Terrabacteria_group>Cyanobacteria/Melainabacteria_group>Cyanobacteria>Oscillatoriophycideae	Moorena_sp._SIO4A1	DGQHR-domain-containing-protein-[Moorena-sp.-SIO4A1]	2607835
NEO43253.1	DndB-ParB-nuclease	GCA_010672045.1	153	Bacteria>Terrabacteria_group>Cyanobacteria/Melainabacteria_group>Cyanobacteria>Oscillatoriophycideae	Moorena_sp._SIO4A3	hypothetical-protein-[Moorena-sp.-SIO4A3]	2607836
NEO46572.1	DndB-ParB-nuclease	GCA_010672045.1	153	Bacteria>Terrabacteria_group>Cyanobacteria/Melainabacteria_group>Cyanobacteria>Oscillatoriophycideae	Moorena_sp._SIO4A3	hypothetical-protein-[Moorena-sp.-SIO4A3]	2607836
NEO19113.1	DndB-ParB-nuclease	GCA_010672005.1	533	Bacteria>Terrabacteria_group>Cyanobacteria/Melainabacteria_group>Cyanobacteria>Oscillatoriophycideae	Moorena_sp._SIO4A5	DGQHR-domain-containing-protein-[Moorea-sp.-SIO4A5]	2607838
NEO74802.1	DndB-ParB-nuclease	GCA_010692305.1	533	Bacteria>Terrabacteria_group>Cyanobacteria/Melainabacteria_group>Cyanobacteria>Oscillatoriophycideae	Moorena_sp._SIO4G3	DGQHR-domain-containing-protein-[Moorena-sp.-SIO4G3]	2607821
NEO40700.1	DndB-ParB-nuclease	GCA_010671925.1	533	Bacteria>Terrabacteria_group>Cyanobacteria/Melainabacteria_group>Cyanobacteria>Oscillatoriophycideae	Moorena_sp._SIOASIH	DGQHR-domain-containing-protein-[Moorea-sp.-SIOASIH]	2607817
WP_124143741.1	DndB-ParB-nuclease	GCF_003838195.1	533	Bacteria>Terrabacteria_group>Cyanobacteria/Melainabacteria_group>Cyanobacteria>Oscillatoriophycideae	Okeania_hirsuta	DGQHR-domain-containing-protein-[Okeania-hirsuta]	1458930
WP_237397288.1	DndB-ParB-nuclease	GCF_014132355.1	488	Bacteria>Terrabacteria_group>Cyanobacteria/Melainabacteria_group>Cyanobacteria>Oscillatoriophycideae	Okeania_sp._KiyG1	DGQHR-domain-containing-protein-[Okeania-sp.-KiyG1]	2720165
GGA58919.1	DndB-ParB-nuclease	GCA_014132355.1	488	Bacteria>Terrabacteria_group>Cyanobacteria/Melainabacteria_group>Cyanobacteria>Oscillatoriophycideae	Okeania_sp._KiyG1	hypothetical-protein-CYANOKiyG1_80260-[Okeania-sp.-KiyG1]	2720165
NET25977.1	DndB-ParB-nuclease	GCA_010672585.1	533	Bacteria>Terrabacteria_group>Cyanobacteria/Melainabacteria_group>Cyanobacteria>Oscillatoriophycideae	Okeania_sp._SIO1I7	DGQHR-domain-containing-protein-[Okeania-sp.-SIO1I7]	2607772
NET44943.1	DndB-ParB-nuclease	GCA_010672525.1	533	Bacteria>Terrabacteria_group>Cyanobacteria/Melainabacteria_group>Cyanobacteria>Oscillatoriophycideae	Okeania_sp._SIO2B3	DGQHR-domain-containing-protein-[Okeania-sp.-SIO2B3]	2607784
NEP86515.1	DndB-ParB-nuclease	GCA_010692625.1	533	Bacteria>Terrabacteria_group>Cyanobacteria/Melainabacteria_group>Cyanobacteria>Oscillatoriophycideae	Okeania_sp._SIO2C2	DGQHR-domain-containing-protein-[Okeania-sp.-SIO2C2]	2607787
NEQ76285.1	DndB-ParB-nuclease	GCA_010672455.1	533	Bacteria>Terrabacteria_group>Cyanobacteria/Melainabacteria_group>Cyanobacteria>Oscillatoriophycideae	Okeania_sp._SIO2C9	DGQHR-domain-containing-protein-[Okeania-sp.-SIO2C9]	2607791
NES04339.1	DndB-ParB-nuclease	GCA_010672385.1	533	Bacteria>Terrabacteria_group>Cyanobacteria/Melainabacteria_group>Cyanobacteria>Oscillatoriophycideae	Okeania_sp._SIO2F4	DGQHR-domain-containing-protein-[Okeania-sp.-SIO2F4]	2607790
NEP43476.1	DndB-ParB-nuclease	GCA_010692585.1	364	Bacteria>Terrabacteria_group>Cyanobacteria/Melainabacteria_group>Cyanobacteria>Oscillatoriophycideae	Okeania_sp._SIO2H7	DGQHR-domain-containing-protein-[Okeania-sp.-SIO2H7]	2607802
NEO53784.1	DndB-ParB-nuclease	GCA_010692555.1	533	Bacteria>Terrabacteria_group>Cyanobacteria/Melainabacteria_group>Cyanobacteria>Oscillatoriophycideae	Okeania_sp._SIO3B5	DGQHR-domain-containing-protein-[Okeania-sp.-SIO3B5]	2607811
NEQ40292.1	DndB-ParB-nuclease	GCA_010672085.1	533	Bacteria>Terrabacteria_group>Cyanobacteria/Melainabacteria_group>Cyanobacteria>Oscillatoriophycideae	Okeania_sp._SIO3I5	DGQHR-domain-containing-protein-[Okeania-sp.-SIO3I5]	2607805
WP_044198010.1	DndB-ParB-nuclease	GCF_000317105.1	532	Bacteria>Terrabacteria_group>Cyanobacteria/Melainabacteria_group>Cyanobacteria>Oscillatoriophycideae	Oscillatoria_acuminata	DGQHR-domain-containing-protein-[Oscillatoria-acuminata]	118323
WP_015151420.1	DndB-ParB-nuclease	GCF_000317105.1	356	Bacteria>Terrabacteria_group>Cyanobacteria/Melainabacteria_group>Cyanobacteria>Oscillatoriophycideae	Oscillatoria_acuminata	DNA-sulfur-modification-protein-DndB-[Oscillatoria-acuminata]	118323
WP_015151421.1	DndB-ParB-nuclease	GCF_000317105.1	391	Bacteria>Terrabacteria_group>Cyanobacteria/Melainabacteria_group>Cyanobacteria>Oscillatoriophycideae	Oscillatoria_acuminata	DNA-sulfur-modification-protein-DndB-[Oscillatoria-acuminata]	118323
AFY84807.1	DndB-ParB-nuclease	GCA_000317105.1	553	Bacteria>Terrabacteria_group>Cyanobacteria/Melainabacteria_group>Cyanobacteria>Oscillatoriophycideae	Oscillatoria_acuminata_PCC_6304	DGQHR-domain-containing-protein-[Oscillatoria-acuminata-PCC-6304]	56110
AFY84809.1	DndB-ParB-nuclease	GCA_000317105.1	391	Bacteria>Terrabacteria_group>Cyanobacteria/Melainabacteria_group>Cyanobacteria>Oscillatoriophycideae	Oscillatoria_acuminata_PCC_6304	DGQHR-domain-containing-protein-[Oscillatoria-acuminata-PCC-6304]	56110
AFY84808.1	DndB-ParB-nuclease	GCA_000317105.1	356	Bacteria>Terrabacteria_group>Cyanobacteria/Melainabacteria_group>Cyanobacteria>Oscillatoriophycideae	Oscillatoria_acuminata_PCC_6304	DNA-sulfur-modification-protein-DndB-[Oscillatoria-acuminata-PCC-6304]	56110
HIK26858.1	DndB-ParB-nuclease	GCA_015295835.1	534	Bacteria>Terrabacteria_group>Cyanobacteria/Melainabacteria_group>Cyanobacteria>Oscillatoriophycideae	Oscillatoriaceae_cyanobacterium_M7585_C2015_266	DGQHR-domain-containing-protein-[Oscillatoriaceae-cyanobacterium-M7585_C2015_266]	2774341
OIP67429.1	DndB-ParB-nuclease	GCA_001873365.1	519	Bacteria>Terrabacteria_group>Cyanobacteria/Melainabacteria_group>Cyanobacteria>Oscillatoriophycideae	Oscillatoriales_cyanobacterium_CG2_30_40_61	hypothetical-protein-AUK43_19655-[Oscillatoriales-cyanobacterium-CG2_30_40_61]	1805291
OIP67430.1	DndB-ParB-nuclease	GCA_001873365.1	392	Bacteria>Terrabacteria_group>Cyanobacteria/Melainabacteria_group>Cyanobacteria>Oscillatoriophycideae	Oscillatoriales_cyanobacterium_CG2_30_40_61	hypothetical-protein-AUK43_19660-[Oscillatoriales-cyanobacterium-CG2_30_40_61]	1805291
TAD82211.1	DndB-ParB-nuclease	GCA_004292525.1	530	Bacteria>Terrabacteria_group>Cyanobacteria/Melainabacteria_group>Cyanobacteria>Oscillatoriophycideae	Oscillatoriales_cyanobacterium	DGQHR-domain-containing-protein-[Oscillatoriales-cyanobacterium]	1151
TAE10926.1	DndB-ParB-nuclease	GCA_004292775.1	530	Bacteria>Terrabacteria_group>Cyanobacteria/Melainabacteria_group>Cyanobacteria>Oscillatoriophycideae	Oscillatoriales_cyanobacterium	DGQHR-domain-containing-protein-[Oscillatoriales-cyanobacterium]	1151
TAE10928.1	DndB-ParB-nuclease	GCA_004292775.1	392	Bacteria>Terrabacteria_group>Cyanobacteria/Melainabacteria_group>Cyanobacteria>Oscillatoriophycideae	Oscillatoriales_cyanobacterium	DGQHR-domain-containing-protein-[Oscillatoriales-cyanobacterium]	1151
TAE38820.1	DndB-ParB-nuclease	GCA_004292895.1	530	Bacteria>Terrabacteria_group>Cyanobacteria/Melainabacteria_group>Cyanobacteria>Oscillatoriophycideae	Oscillatoriales_cyanobacterium	DGQHR-domain-containing-protein-[Oscillatoriales-cyanobacterium]	1151
TAE65063.1	DndB-ParB-nuclease	GCA_004292955.1	530	Bacteria>Terrabacteria_group>Cyanobacteria/Melainabacteria_group>Cyanobacteria>Oscillatoriophycideae	Oscillatoriales_cyanobacterium	DGQHR-domain-containing-protein-[Oscillatoriales-cyanobacterium]	1151
TAE65064.1	DndB-ParB-nuclease	GCA_004292955.1	392	Bacteria>Terrabacteria_group>Cyanobacteria/Melainabacteria_group>Cyanobacteria>Oscillatoriophycideae	Oscillatoriales_cyanobacterium	DGQHR-domain-containing-protein-[Oscillatoriales-cyanobacterium]	1151
TAE81536.1	DndB-ParB-nuclease	GCA_004293075.1	530	Bacteria>Terrabacteria_group>Cyanobacteria/Melainabacteria_group>Cyanobacteria>Oscillatoriophycideae	Oscillatoriales_cyanobacterium	DGQHR-domain-containing-protein-[Oscillatoriales-cyanobacterium]	1151
TAG66801.1	DndB-ParB-nuclease	GCA_004294345.1	530	Bacteria>Terrabacteria_group>Cyanobacteria/Melainabacteria_group>Cyanobacteria>Oscillatoriophycideae	Oscillatoriales_cyanobacterium	DGQHR-domain-containing-protein-[Oscillatoriales-cyanobacterium]	1151
TAG66802.1	DndB-ParB-nuclease	GCA_004294345.1	392	Bacteria>Terrabacteria_group>Cyanobacteria/Melainabacteria_group>Cyanobacteria>Oscillatoriophycideae	Oscillatoriales_cyanobacterium	DGQHR-domain-containing-protein-[Oscillatoriales-cyanobacterium]	1151
TAG94357.1	DndB-ParB-nuclease	GCA_004294385.1	530	Bacteria>Terrabacteria_group>Cyanobacteria/Melainabacteria_group>Cyanobacteria>Oscillatoriophycideae	Oscillatoriales_cyanobacterium	DGQHR-domain-containing-protein-[Oscillatoriales-cyanobacterium]	1151
TAG94404.1	DndB-ParB-nuclease	GCA_004294385.1	390	Bacteria>Terrabacteria_group>Cyanobacteria/Melainabacteria_group>Cyanobacteria>Oscillatoriophycideae	Oscillatoriales_cyanobacterium	DGQHR-domain-containing-protein-[Oscillatoriales-cyanobacterium]	1151
TAE10927.1	DndB-ParB-nuclease	GCA_004292775.1	362	Bacteria>Terrabacteria_group>Cyanobacteria/Melainabacteria_group>Cyanobacteria>Oscillatoriophycideae	Oscillatoriales_cyanobacterium	DNA-sulfur-modification-protein-DndB-[Oscillatoriales-cyanobacterium]	1151
TAE56631.1	DndB-ParB-nuclease	GCA_004292945.1	362	Bacteria>Terrabacteria_group>Cyanobacteria/Melainabacteria_group>Cyanobacteria>Oscillatoriophycideae	Oscillatoriales_cyanobacterium	DNA-sulfur-modification-protein-DndB-[Oscillatoriales-cyanobacterium]	1151
TAE81535.1	DndB-ParB-nuclease	GCA_004293075.1	380	Bacteria>Terrabacteria_group>Cyanobacteria/Melainabacteria_group>Cyanobacteria>Oscillatoriophycideae	Oscillatoriales_cyanobacterium	DNA-sulfur-modification-protein-DndB-[Oscillatoriales-cyanobacterium]	1151
TAD82214.1	DndB-ParB-nuclease	GCA_004292525.1	362	Bacteria>Terrabacteria_group>Cyanobacteria/Melainabacteria_group>Cyanobacteria>Oscillatoriophycideae	Oscillatoriales_cyanobacterium	hypothetical-protein-EA000_18285,-partial-[Oscillatoriales-cyanobacterium]	1151
TAF99612.1	DndB-ParB-nuclease	GCA_004294025.1	48	Bacteria>Terrabacteria_group>Cyanobacteria/Melainabacteria_group>Cyanobacteria>Oscillatoriophycideae	Oscillatoriales_cyanobacterium	hypothetical-protein-EAZ45_17385,-partial-[Oscillatoriales-cyanobacterium]	1151
MBE9040626.1	DndB-ParB-nuclease	GCA_015207075.1	512	Bacteria>Terrabacteria_group>Cyanobacteria/Melainabacteria_group>Cyanobacteria>Oscillatoriophycideae	Oscillatoriales_cyanobacterium_LEGE_11467	DGQHR-domain-containing-protein-[Oscillatoriales-cyanobacterium-LEGE-11467]	1828826
TAE22565.1	DndB-ParB-nuclease	GCA_004292845.1	530	Bacteria>Terrabacteria_group>Cyanobacteria/Melainabacteria_group>Cyanobacteria>Oscillatoriophycideae	Oscillatoriales_cyanobacterium	MAG:-DGQHR-domain-containing-protein-[Oscillatoriales-cyanobacterium]	1151
TAE22567.1	DndB-ParB-nuclease	GCA_004292845.1	392	Bacteria>Terrabacteria_group>Cyanobacteria/Melainabacteria_group>Cyanobacteria>Oscillatoriophycideae	Oscillatoriales_cyanobacterium	MAG:-DGQHR-domain-containing-protein-[Oscillatoriales-cyanobacterium]	1151
TAE38819.1	DndB-ParB-nuclease	GCA_004292895.1	392	Bacteria>Terrabacteria_group>Cyanobacteria/Melainabacteria_group>Cyanobacteria>Oscillatoriophycideae	Oscillatoriales_cyanobacterium	MAG:-DGQHR-domain-containing-protein-[Oscillatoriales-cyanobacterium]	1151
TAE56630.1	DndB-ParB-nuclease	GCA_004292945.1	530	Bacteria>Terrabacteria_group>Cyanobacteria/Melainabacteria_group>Cyanobacteria>Oscillatoriophycideae	Oscillatoriales_cyanobacterium	MAG:-DGQHR-domain-containing-protein-[Oscillatoriales-cyanobacterium]	1151
TAE56632.1	DndB-ParB-nuclease	GCA_004292945.1	392	Bacteria>Terrabacteria_group>Cyanobacteria/Melainabacteria_group>Cyanobacteria>Oscillatoriophycideae	Oscillatoriales_cyanobacterium	MAG:-DGQHR-domain-containing-protein-[Oscillatoriales-cyanobacterium]	1151
TAE22566.1	DndB-ParB-nuclease	GCA_004292845.1	362	Bacteria>Terrabacteria_group>Cyanobacteria/Melainabacteria_group>Cyanobacteria>Oscillatoriophycideae	Oscillatoriales_cyanobacterium	MAG:-DNA-sulfur-modification-protein-DndB-[Oscillatoriales-cyanobacterium]	1151
NJO44455.1	DndB-ParB-nuclease	GCA_012033335.1	394	Bacteria>Terrabacteria_group>Cyanobacteria/Melainabacteria_group>Cyanobacteria>Oscillatoriophycideae	Oscillatoriales_cyanobacterium_RM2_1_1	DGQHR-domain-containing-protein-[Oscillatoriales-cyanobacterium-RM2_1_1]	2720435
NJO44453.1	DndB-ParB-nuclease	GCA_012033335.1	354	Bacteria>Terrabacteria_group>Cyanobacteria/Melainabacteria_group>Cyanobacteria>Oscillatoriophycideae	Oscillatoriales_cyanobacterium_RM2_1_1	DNA-sulfur-modification-protein-DndB-[Oscillatoriales-cyanobacterium-RM2_1_1]	2720435
NJM62610.1	DndB-ParB-nuclease	GCA_012032195.1	193	Bacteria>Terrabacteria_group>Cyanobacteria/Melainabacteria_group>Cyanobacteria>Oscillatoriophycideae	Oscillatoriales_cyanobacterium_RU_3_3	DGQHR-domain-containing-protein-[Oscillatoriales-cyanobacterium-RU_3_3]	2720441
NJK38819.1	DndB-ParB-nuclease	GCA_012030745.1	394	Bacteria>Terrabacteria_group>Cyanobacteria/Melainabacteria_group>Cyanobacteria>Oscillatoriophycideae	Oscillatoriales_cyanobacterium_SM2_3_0	DGQHR-domain-containing-protein-[Oscillatoriales-cyanobacterium-SM2_3_0]	2720468
NJK38816.1	DndB-ParB-nuclease	GCA_012030745.1	354	Bacteria>Terrabacteria_group>Cyanobacteria/Melainabacteria_group>Cyanobacteria>Oscillatoriophycideae	Oscillatoriales_cyanobacterium_SM2_3_0	DNA-sulfur-modification-protein-DndB-[Oscillatoriales-cyanobacterium-SM2_3_0]	2720468
OCR02692.1	DndB-ParB-nuclease	GCA_001698425.1	344	Bacteria>Terrabacteria_group>Cyanobacteria/Melainabacteria_group>Cyanobacteria>Oscillatoriophycideae	Oscillatoriales_cyanobacterium_USR001	DNA-sulfur-modification-protein-DndB-[Oscillatoriales-cyanobacterium-USR001]	1880991
OCR02665.1	DndB-ParB-nuclease	GCA_001698425.1	388	Bacteria>Terrabacteria_group>Cyanobacteria/Melainabacteria_group>Cyanobacteria>Oscillatoriophycideae	Oscillatoriales_cyanobacterium_USR001	hypothetical-protein-BCD67_15885-[Oscillatoriales-cyanobacterium-USR001]	1880991
OCR02666.1	DndB-ParB-nuclease	GCA_001698425.1	531	Bacteria>Terrabacteria_group>Cyanobacteria/Melainabacteria_group>Cyanobacteria>Oscillatoriophycideae	Oscillatoriales_cyanobacterium_USR001	hypothetical-protein-BCD67_15900-[Oscillatoriales-cyanobacterium-USR001]	1880991
WP_015179049.1	DndB-ParB-nuclease	GCF_000317475.1	530	Bacteria>Terrabacteria_group>Cyanobacteria/Melainabacteria_group>Cyanobacteria>Oscillatoriophycideae	Oscillatoria_nigro-viridis	DGQHR-domain-containing-protein-[Oscillatoria-nigro-viridis]	482564
WP_015179046.1	DndB-ParB-nuclease	GCF_000317475.1	391	Bacteria>Terrabacteria_group>Cyanobacteria/Melainabacteria_group>Cyanobacteria>Oscillatoriophycideae	Oscillatoria_nigro-viridis	DNA-sulfur-modification-protein-DndB-[Oscillatoria-nigro-viridis]	482564
WP_015179047.1	DndB-ParB-nuclease	GCF_000317475.1	379	Bacteria>Terrabacteria_group>Cyanobacteria/Melainabacteria_group>Cyanobacteria>Oscillatoriophycideae	Oscillatoria_nigro-viridis	DNA-sulfur-modification-protein-DndB-[Oscillatoria-nigro-viridis]	482564

Accession	Annotation	GCA	Length	Phyletic.spread	Strain.name	Defline	Species.Taxid
MBW4491811.1	DndB-ParB-nuclease	GCA_019359235.1	533	Bacteria>Terrabacteria_group>Cyanobacteria/Melainabacteria_group>Cyanobacteria>Oscillatoriophycideae	Oscillatoria_princeps_RMCB-10	DGQHR-domain-containing-protein-[Oscillatoria-princeps-RMCB-10]	2839661
MBW4491812.1	DndB-ParB-nuclease	GCA_019359235.1	390	Bacteria>Terrabacteria_group>Cyanobacteria/Melainabacteria_group>Cyanobacteria>Oscillatoriophycideae	Oscillatoria_princeps_RMCB-10	DNA-sulfur-modification-protein-DndB-[Oscillatoria-princeps-RMCB-10]	2839661
WP_224409258.1	DndB-ParB-nuclease	GCF_020144665.1	534	Bacteria>Terrabacteria_group>Cyanobacteria/Melainabacteria_group>Cyanobacteria>Oscillatoriophycideae	Oscillatoria_salina	DGQHR-domain-containing-protein-[Oscillatoria-salina]	331517
WP_224409259.1	DndB-ParB-nuclease	GCF_020144665.1	391	Bacteria>Terrabacteria_group>Cyanobacteria/Melainabacteria_group>Cyanobacteria>Oscillatoriophycideae	Oscillatoria_salina	DNA-sulfur-modification-protein-DndB-[Oscillatoria-salina]	331517
MBZ8179284.1	DndB-ParB-nuclease	GCA_020144665.1	534	Bacteria>Terrabacteria_group>Cyanobacteria/Melainabacteria_group>Cyanobacteria>Oscillatoriophycideae	Oscillatoria_salina_IIICB1	DGQHR-domain-containing-protein-[Oscillatoria-salina-IIICB1]	2708302
MBZ8179285.1	DndB-ParB-nuclease	GCA_020144665.1	391	Bacteria>Terrabacteria_group>Cyanobacteria/Melainabacteria_group>Cyanobacteria>Oscillatoriophycideae	Oscillatoria_salina_IIICB1	DGQHR-domain-containing-protein-[Oscillatoria-salina-IIICB1]	2708302
WP_190717652.1	DndB-ParB-nuclease	GCF_014698145.1	358	Bacteria>Terrabacteria_group>Cyanobacteria/Melainabacteria_group>Cyanobacteria>Oscillatoriophycideae	Oscillatoria_sp._FACHB-1406	DNA-sulfur-modification-protein-DndB-[Oscillatoria-sp.-FACHB-1406]	2692846
WP_017721076.1	DndB-ParB-nuclease	GCF_000332335.1	533	Bacteria>Terrabacteria_group>Cyanobacteria/Melainabacteria_group>Cyanobacteria>Oscillatoriophycideae	Oscillatoria_sp._PCC_10802	DGQHR-domain-containing-protein-[Oscillatoria-sp.-PCC-10802]	1173028
WP_017721077.1	DndB-ParB-nuclease	GCF_000332335.1	390	Bacteria>Terrabacteria_group>Cyanobacteria/Melainabacteria_group>Cyanobacteria>Oscillatoriophycideae	Oscillatoria_sp._PCC_10802	DNA-sulfur-modification-protein-DndB-[Oscillatoria-sp.-PCC-10802]	1173028
WP_168572068.1	DndB-ParB-nuclease	GCF_012295525.1	532	Bacteria>Terrabacteria_group>Cyanobacteria/Melainabacteria_group>Cyanobacteria>Oscillatoriophycideae	Oxynema_aestuarii	DGQHR-domain-containing-protein-[Oxynema-aestuarii]	2874213
WP_168571559.1	DndB-ParB-nuclease	GCF_012295525.1	360	Bacteria>Terrabacteria_group>Cyanobacteria/Melainabacteria_group>Cyanobacteria>Oscillatoriophycideae	Oxynema_aestuarii	DNA-sulfur-modification-protein-DndB-[Oxynema-aestuarii]	2874213
WP_200669863.1	DndB-ParB-nuclease	GCF_016632315.1	532	Bacteria>Terrabacteria_group>Cyanobacteria/Melainabacteria_group>Cyanobacteria>Oscillatoriophycideae	Oxynema_sp._CENA135	DGQHR-domain-containing-protein-[Oxynema-sp.-CENA135]	984206
WP_200669864.1	DndB-ParB-nuclease	GCF_016632315.1	360	Bacteria>Terrabacteria_group>Cyanobacteria/Melainabacteria_group>Cyanobacteria>Oscillatoriophycideae	Oxynema_sp._CENA135	DNA-sulfur-modification-protein-DndB-[Oxynema-sp.-CENA135]	984206
WP_073594395.1	DndB-ParB-nuclease	GCF_001904725.1	532	Bacteria>Terrabacteria_group>Cyanobacteria/Melainabacteria_group>Cyanobacteria>Oscillatoriophycideae	Phormidium_ambiguum	DGQHR-domain-containing-protein-[Phormidium-ambiguum]	71191
WP_073594397.1	DndB-ParB-nuclease	GCF_001904725.1	348	Bacteria>Terrabacteria_group>Cyanobacteria/Melainabacteria_group>Cyanobacteria>Oscillatoriophycideae	Phormidium_ambiguum	DNA-sulfur-modification-protein-DndB-[Phormidium-ambiguum]	71191
WP_207089075.1	DndB-ParB-nuclease	GCF_017313335.1	532	Bacteria>Terrabacteria_group>Cyanobacteria/Melainabacteria_group>Cyanobacteria>Oscillatoriophycideae	Phormidium_pseudopriestleyi	DGQHR-domain-containing-protein-[Phormidium-pseudopriestleyi]	1759527
WP_207089076.1	DndB-ParB-nuclease	-	357	Bacteria>Terrabacteria_group>Cyanobacteria/Melainabacteria_group>Cyanobacteria>Oscillatoriophycideae	Phormidium_pseudopriestleyi	DNA-sulfur-modification-protein-DndB-[Phormidium-pseudopriestleyi]	1759527
WP_207089077.1	DndB-ParB-nuclease	GCF_017313335.1	391	Bacteria>Terrabacteria_group>Cyanobacteria/Melainabacteria_group>Cyanobacteria>Oscillatoriophycideae	Phormidium_pseudopriestleyi	DNA-sulfur-modification-protein-DndB-[Phormidium-pseudopriestleyi]	1759527
MBO0350601.1	DndB-ParB-nuclease	GCA_017313335.1	532	Bacteria>Terrabacteria_group>Cyanobacteria/Melainabacteria_group>Cyanobacteria>Oscillatoriophycideae	Phormidium_pseudopriestleyi_FRX01	DGQHR-domain-containing-protein-[Phormidium-pseudopriestleyi-FRX01]	1759528
MBO0350602.1	DndB-ParB-nuclease	GCA_017313335.1	357	Bacteria>Terrabacteria_group>Cyanobacteria/Melainabacteria_group>Cyanobacteria>Oscillatoriophycideae	Phormidium_pseudopriestleyi_FRX01	DNA-sulfur-modification-protein-DndB-[Phormidium-pseudopriestleyi-FRX01]	1759528
MBO0350603.1	DndB-ParB-nuclease	GCA_017313335.1	391	Bacteria>Terrabacteria_group>Cyanobacteria/Melainabacteria_group>Cyanobacteria>Oscillatoriophycideae	Phormidium_pseudopriestleyi_FRX01	DNA-sulfur-modification-protein-DndB-[Phormidium-pseudopriestleyi-FRX01]	1759528
WP_193981072.1	DndB-ParB-nuclease	GCF_015207735.1	532	Bacteria>Terrabacteria_group>Cyanobacteria/Melainabacteria_group>Cyanobacteria>Oscillatoriophycideae	Phormidium_sp._LEGE_05292	DGQHR-domain-containing-protein-[Phormidium-sp.-LEGE-05292]	2777964
WP_193981074.1	DndB-ParB-nuclease	GCF_015207735.1	347	Bacteria>Terrabacteria_group>Cyanobacteria/Melainabacteria_group>Cyanobacteria>Oscillatoriophycideae	Phormidium_sp._LEGE_05292	DNA-sulfur-modification-protein-DndB-[Phormidium-sp.-LEGE-05292]	2777964
MBD2583817.1	DndB-ParB-nuclease	GCA_014698535.1	533	Bacteria>Terrabacteria_group>Cyanobacteria/Melainabacteria_group>Cyanobacteria>Oscillatoriophycideae	Planktothricoides_sp._FACHB-1261	DGQHR-domain-containing-protein-[Planktothricoides-sp.-FACHB-1261]	2692852
MBD2583816.1	DndB-ParB-nuclease	GCA_014698535.1	371	Bacteria>Terrabacteria_group>Cyanobacteria/Melainabacteria_group>Cyanobacteria>Oscillatoriophycideae	Planktothricoides_sp._FACHB-1261	DNA-sulfur-modification-protein-DndB-[Planktothricoides-sp.-FACHB-1261]	2692852
MBD2583818.1	DndB-ParB-nuclease	GCA_014698535.1	729	Bacteria>Terrabacteria_group>Cyanobacteria/Melainabacteria_group>Cyanobacteria>Oscillatoriophycideae	Planktothricoides_sp._FACHB-1261	hypothetical-protein-[Planktothricoides-sp.-FACHB-1261]	2692852
MBD2546185.1	DndB-ParB-nuclease	GCA_014698175.1	533	Bacteria>Terrabacteria_group>Cyanobacteria/Melainabacteria_group>Cyanobacteria>Oscillatoriophycideae	Planktothricoides_sp._FACHB-1370	DGQHR-domain-containing-protein-[Planktothricoides-sp.-FACHB-1370]	2692853
MBD2546186.1	DndB-ParB-nuclease	GCA_014698175.1	371	Bacteria>Terrabacteria_group>Cyanobacteria/Melainabacteria_group>Cyanobacteria>Oscillatoriophycideae	Planktothricoides_sp._FACHB-1370	DNA-sulfur-modification-protein-DndB-[Planktothricoides-sp.-FACHB-1370]	2692853
MBD2546184.1	DndB-ParB-nuclease	GCA_014698175.1	729	Bacteria>Terrabacteria_group>Cyanobacteria/Melainabacteria_group>Cyanobacteria>Oscillatoriophycideae	Planktothricoides_sp._FACHB-1370	hypothetical-protein-[Planktothricoides-sp.-FACHB-1370]	2692853
KOR35186.1	DndB-ParB-nuclease	GCA_001276715.1	729	Bacteria>Terrabacteria_group>Cyanobacteria/Melainabacteria_group>Cyanobacteria>Oscillatoriophycideae	Planktothricoides_sp._SR001	hypothetical-protein-AM228_19930-[Planktothricoides-sp.-SR001]	1705388
KOR35187.1	DndB-ParB-nuclease	GCA_001276715.1	533	Bacteria>Terrabacteria_group>Cyanobacteria/Melainabacteria_group>Cyanobacteria>Oscillatoriophycideae	Planktothricoides_sp._SR001	hypothetical-protein-AM228_19935-[Planktothricoides-sp.-SR001]	1705388
KOR35188.1	DndB-ParB-nuclease	GCA_001276715.1	371	Bacteria>Terrabacteria_group>Cyanobacteria/Melainabacteria_group>Cyanobacteria>Oscillatoriophycideae	Planktothricoides_sp._SR001	hypothetical-protein-AM228_19940-[Planktothricoides-sp.-SR001]	1705388
GDZ96327.1	DndB-ParB-nuclease	GCA_005402765.2	520	Bacteria>Terrabacteria_group>Cyanobacteria/Melainabacteria_group>Cyanobacteria>Oscillatoriophycideae	Planktothrix_agardhii_CCAP_1459/11A	hypothetical-protein-PA905_48700-[Planktothrix-agardhii-CCAP-1459/11A]	282420
CUM58832.1	DndB-ParB-nuclease	-	520	Bacteria>Terrabacteria_group>Cyanobacteria/Melainabacteria_group>Cyanobacteria>Oscillatoriophycideae	Planktothrix_agardhii	conserved-protein-of-unknown-function-[Planktothrix-agardhii]	1160
WP_027249946.1	DndB-ParB-nuclease	GCF_000464665.1	520	Bacteria>Terrabacteria_group>Cyanobacteria/Melainabacteria_group>Cyanobacteria>Oscillatoriophycideae	Planktothrix_agardhii	DGQHR-domain-containing-protein-[Planktothrix-agardhii]	1160
WP_027255627.1	DndB-ParB-nuclease	GCF_000464825.1	520	Bacteria>Terrabacteria_group>Cyanobacteria/Melainabacteria_group>Cyanobacteria>Oscillatoriophycideae	Planktothrix_agardhii	DGQHR-domain-containing-protein-[Planktothrix-agardhii]	1160
WP_042154688.1	DndB-ParB-nuclease	GCF_000710505.1	520	Bacteria>Terrabacteria_group>Cyanobacteria/Melainabacteria_group>Cyanobacteria>Oscillatoriophycideae	Planktothrix_agardhii	DGQHR-domain-containing-protein-[Planktothrix-agardhii]	1160
WP_141296476.1	DndB-ParB-nuclease	GCF_005402765.1	520	Bacteria>Terrabacteria_group>Cyanobacteria/Melainabacteria_group>Cyanobacteria>Oscillatoriophycideae	Planktothrix_agardhii	DGQHR-domain-containing-protein-[Planktothrix-agardhii]	1160
WP_214560473.1	DndB-ParB-nuclease	GCF_903969095.1	520	Bacteria>Terrabacteria_group>Cyanobacteria/Melainabacteria_group>Cyanobacteria>Oscillatoriophycideae	Planktothrix_agardhii	DGQHR-domain-containing-protein-[Planktothrix-agardhii]	1160
WP_227350757.1	DndB-ParB-nuclease	GCF_020594555.1	520	Bacteria>Terrabacteria_group>Cyanobacteria/Melainabacteria_group>Cyanobacteria>Oscillatoriophycideae	Planktothrix_agardhii	DGQHR-domain-containing-protein-[Planktothrix-agardhii]	1160
WP_227381495.1	DndB-ParB-nuclease	GCF_020594575.1	519	Bacteria>Terrabacteria_group>Cyanobacteria/Melainabacteria_group>Cyanobacteria>Oscillatoriophycideae	Planktothrix_agardhii	DGQHR-domain-containing-protein-[Planktothrix-agardhii]	1160
WP_227397625.1	DndB-ParB-nuclease	GCF_020594695.1	520	Bacteria>Terrabacteria_group>Cyanobacteria/Melainabacteria_group>Cyanobacteria>Oscillatoriophycideae	Planktothrix_agardhii	DGQHR-domain-containing-protein-[Planktothrix-agardhii]	1160
WP_027249945.1	DndB-ParB-nuclease	GCF_903969095.1	391	Bacteria>Terrabacteria_group>Cyanobacteria/Melainabacteria_group>Cyanobacteria>Oscillatoriophycideae	Planktothrix_agardhii	DNA-sulfur-modification-protein-DndB-[Planktothrix-agardhii]	1160
WP_042154690.1	DndB-ParB-nuclease	GCF_000710505.1	392	Bacteria>Terrabacteria_group>Cyanobacteria/Melainabacteria_group>Cyanobacteria>Oscillatoriophycideae	Planktothrix_agardhii	DNA-sulfur-modification-protein-DndB-[Planktothrix-agardhii]	1160
MBG0746692.1	DndB-ParB-nuclease	GCA_015708255.1	122	Bacteria>Terrabacteria_group>Cyanobacteria/Melainabacteria_group>Cyanobacteria>Oscillatoriophycideae	Planktothrix_agardhii_KL2	hypothetical-protein-[Planktothrix-agardhii-KL2]	2789715
WP_193867884.1	DndB-ParB-nuclease	GCF_015207485.1	519	Bacteria>Terrabacteria_group>Cyanobacteria/Melainabacteria_group>Cyanobacteria>Oscillatoriophycideae	Planktothrix_mougeotii	DGQHR-domain-containing-protein-[Planktothrix-mougeotii]	54306
WP_193867883.1	DndB-ParB-nuclease	GCF_015207485.1	392	Bacteria>Terrabacteria_group>Cyanobacteria/Melainabacteria_group>Cyanobacteria>Oscillatoriophycideae	Planktothrix_mougeotii	DNA-sulfur-modification-protein-DndB-[Planktothrix-mougeotii]	54306
WP_026795254.1	DndB-ParB-nuclease	GCF_000464725.1	519	Bacteria>Terrabacteria_group>Cyanobacteria/Melainabacteria_group>Cyanobacteria>Oscillatoriophycideae	Planktothrix	MULTISPECIES:-DGQHR-domain-containing-protein-[Planktothrix]	54304
WP_026796652.1	DndB-ParB-nuclease	GCF_000464765.1	519	Bacteria>Terrabacteria_group>Cyanobacteria/Melainabacteria_group>Cyanobacteria>Oscillatoriophycideae	Planktothrix	MULTISPECIES:-DGQHR-domain-containing-protein-[Planktothrix]	54304
WP_036832905.1	DndB-ParB-nuclease	GCF_000464725.1	407	Bacteria>Terrabacteria_group>Cyanobacteria/Melainabacteria_group>Cyanobacteria>Oscillatoriophycideae	Planktothrix	MULTISPECIES:-DNA-sulfur-modification-protein-DndB-[Planktothrix]	54304
WP_083621156.1	DndB-ParB-nuclease	GCF_900009265.2	519	Bacteria>Terrabacteria_group>Cyanobacteria/Melainabacteria_group>Cyanobacteria>Oscillatoriophycideae	Planktothrix_paucivesiculata	DGQHR-domain-containing-protein-[Planktothrix-paucivesiculata]	1678308
WP_083621158.1	DndB-ParB-nuclease	GCF_900009265.2	408	Bacteria>Terrabacteria_group>Cyanobacteria/Melainabacteria_group>Cyanobacteria>Oscillatoriophycideae	Planktothrix_paucivesiculata	DNA-sulfur-modification-protein-DndB-[Planktothrix-paucivesiculata]	1678308
WP_026787999.1	DndB-ParB-nuclease	GCF_000464785.1	520	Bacteria>Terrabacteria_group>Cyanobacteria/Melainabacteria_group>Cyanobacteria>Oscillatoriophycideae	Planktothrix_rubescens	DGQHR-domain-containing-protein-[Planktothrix-rubescens]	59512
WP_083617402.1	DndB-ParB-nuclease	GCF_900010725.2	524	Bacteria>Terrabacteria_group>Cyanobacteria/Melainabacteria_group>Cyanobacteria>Oscillatoriophycideae	Planktothrix_serta	DGQHR-domain-containing-protein-[Planktothrix-serta]	1678310
WP_190517563.1	DndB-ParB-nuclease	GCF_014697575.1	519	Bacteria>Terrabacteria_group>Cyanobacteria/Melainabacteria_group>Cyanobacteria>Oscillatoriophycideae	Planktothrix_sp._FACHB-1365	DGQHR-domain-containing-protein-[Planktothrix-sp.-FACHB-1365]	2692855
WP_199314121.1	DndB-ParB-nuclease	-	393	Bacteria>Terrabacteria_group>Cyanobacteria/Melainabacteria_group>Cyanobacteria>Oscillatoriophycideae	Planktothrix_sp._FACHB-1365	DNA-sulfur-modification-protein-DndB-[Planktothrix-sp.-FACHB-1365]	2692855
MBD2179699.1	DndB-ParB-nuclease	GCA_014696265.1	533	Bacteria>Terrabacteria_group>Cyanobacteria/Melainabacteria_group>Cyanobacteria>Oscillatoriophycideae	Planktothrix_sp._FACHB-1375	DGQHR-domain-containing-protein-[Planktothrix-sp.-FACHB-1375]	2692856
WP_190461118.1	DndB-ParB-nuclease	GCF_014696265.1	533	Bacteria>Terrabacteria_group>Cyanobacteria/Melainabacteria_group>Cyanobacteria>Oscillatoriophycideae	Planktothrix_sp._FACHB-1375	DGQHR-domain-containing-protein-[Planktothrix-sp.-FACHB-1375]	2692856
MBD2179702.1	DndB-ParB-nuclease	GCA_014696265.1	351	Bacteria>Terrabacteria_group>Cyanobacteria/Melainabacteria_group>Cyanobacteria>Oscillatoriophycideae	Planktothrix_sp._FACHB-1375	DNA-sulfur-modification-protein-DndB-[Planktothrix-sp.-FACHB-1375]	2692856
MBD2179703.1	DndB-ParB-nuclease	GCA_014696265.1	393	Bacteria>Terrabacteria_group>Cyanobacteria/Melainabacteria_group>Cyanobacteria>Oscillatoriophycideae	Planktothrix_sp._FACHB-1375	DNA-sulfur-modification-protein-DndB-[Planktothrix-sp.-FACHB-1375]	2692856
WP_190461125.1	DndB-ParB-nuclease	GCF_014696265.1	393	Bacteria>Terrabacteria_group>Cyanobacteria/Melainabacteria_group>Cyanobacteria>Oscillatoriophycideae	Planktothrix_sp._FACHB-1375	DNA-sulfur-modification-protein-DndB-[Planktothrix-sp.-FACHB-1375]	2692856
WP_079681451.1	DndB-ParB-nuclease	GCF_900009135.1	519	Bacteria>Terrabacteria_group>Cyanobacteria/Melainabacteria_group>Cyanobacteria>Oscillatoriophycideae	Planktothrix_sp._PCC_11201	DGQHR-domain-containing-protein-[Planktothrix-sp.-PCC-11201]	1729650
WP_079681452.1	DndB-ParB-nuclease	GCF_900009135.1	392	Bacteria>Terrabacteria_group>Cyanobacteria/Melainabacteria_group>Cyanobacteria>Oscillatoriophycideae	Planktothrix_sp._PCC_11201	DNA-sulfur-modification-protein-DndB-[Planktothrix-sp.-PCC-11201]	1729650
HAN73190.1	DndB-ParB-nuclease	GCA_003456035.1	393	Bacteria>Terrabacteria_group>Cyanobacteria/Melainabacteria_group>Cyanobacteria>Oscillatoriophycideae	Planktothrix_sp._UBA8402	hypothetical-protein-[Planktothrix-sp.-UBA8402]	2055759
WP_072717061.1	DndB-ParB-nuclease	GCF_900009145.1	524	Bacteria>Terrabacteria_group>Cyanobacteria/Melainabacteria_group>Cyanobacteria>Oscillatoriophycideae	Planktothrix_tepida	DGQHR-domain-containing-protein-[Planktothrix-tepida]	1678309
CUR35921.1	DndB-ParB-nuclease	GCA_900009145.1	524	Bacteria>Terrabacteria_group>Cyanobacteria/Melainabacteria_group>Cyanobacteria>Oscillatoriophycideae	Planktothrix_tepida_PCC_9214	conserved-hypothetical-protein-[Planktothrix-tepida-PCC-9214]	671072
MBE9211903.1	DndB-ParB-nuclease	GCA_015207665.1	525	Bacteria>Terrabacteria_group>Cyanobacteria/Melainabacteria_group>Cyanobacteria>Oscillatoriophycideae	Plectonema_cf._radiosum_LEGE_06105	DGQHR-domain-containing-protein-[Plectonema-cf.-radiosum-LEGE-06105]	945769
WP_193917327.1	DndB-ParB-nuclease	GCF_015207665.1	525	Bacteria>Terrabacteria_group>Cyanobacteria/Melainabacteria_group>Cyanobacteria>Oscillatoriophycideae	Plectonema_radiosum	DGQHR-domain-containing-protein-[Plectonema-radiosum]	945768
WP_012595683.1	DndB-ParB-nuclease	GCF_000021805.1	534	Bacteria>Terrabacteria_group>Cyanobacteria/Melainabacteria_group>Cyanobacteria>Oscillatoriophycideae	Rippkaea_orientalis	DGQHR-domain-containing-protein-[Rippkaea-orientalis]	2546366
WP_012595682.1	DndB-ParB-nuclease	GCF_000021805.1	422	Bacteria>Terrabacteria_group>Cyanobacteria/Melainabacteria_group>Cyanobacteria>Oscillatoriophycideae	Rippkaea_orientalis	DNA-sulfur-modification-protein-DndB-[Rippkaea-orientalis]	2546366
OJJ25017.1	DndB-ParB-nuclease	GCA_001890975.1	399	Bacteria>Terrabacteria_group>Cyanobacteria/Melainabacteria_group>Cyanobacteria>Oscillatoriophycideae	Roseofilum_reptotaenium_AO1-A	hypothetical-protein-BI308_13325-[Roseofilum-reptotaenium-AO1-A]	1925591
MBP0007613.1	DndB-ParB-nuclease	GCA_017746785.1	399	Bacteria>Terrabacteria_group>Cyanobacteria/Melainabacteria_group>Cyanobacteria>Oscillatoriophycideae	Roseofilum_sp._Belize_Diploria	DNA-sulfur-modification-protein-DndB-[Roseofilum-sp.-Belize-Diploria]	2821501
MBP0013415.1	DndB-ParB-nuclease	GCA_017746835.1	399	Bacteria>Terrabacteria_group>Cyanobacteria/Melainabacteria_group>Cyanobacteria>Oscillatoriophycideae	Roseofilum_sp._SID3	DNA-sulfur-modification-protein-DndB-[Roseofilum-sp.-SID3]	2821499
NER26240.1	DndB-ParB-nuclease	GCA_010672885.1	391	Bacteria>Terrabacteria_group>Cyanobacteria/Melainabacteria_group>Cyanobacteria>Oscillatoriophycideae	Symploca_sp._SIO1C4	DGQHR-domain-containing-protein-[Symploca-sp.-SIO1C4]	2607765
NER26241.1	DndB-ParB-nuclease	GCA_010672885.1	533	Bacteria>Terrabacteria_group>Cyanobacteria/Melainabacteria_group>Cyanobacteria>Oscillatoriophycideae	Symploca_sp._SIO1C4	DGQHR-domain-containing-protein-[Symploca-sp.-SIO1C4]	2607765
NET03666.1	DndB-ParB-nuclease	GCA_010672535.1	533	Bacteria>Terrabacteria_group>Cyanobacteria/Melainabacteria_group>Cyanobacteria>Oscillatoriophycideae	Symploca_sp._SIO2B6	DGQHR-domain-containing-protein-[Symploca-sp.-SIO2B6]	2607785
NET03665.1	DndB-ParB-nuclease	GCA_010672535.1	213	Bacteria>Terrabacteria_group>Cyanobacteria/Melainabacteria_group>Cyanobacteria>Oscillatoriophycideae	Symploca_sp._SIO2B6	hypothetical-protein-[Symploca-sp.-SIO2B6]	2607785
NEO97367.1	DndB-ParB-nuclease	GCA_010692645.1	391	Bacteria>Terrabacteria_group>Cyanobacteria/Melainabacteria_group>Cyanobacteria>Oscillatoriophycideae	Symploca_sp._SIO2E9	DGQHR-domain-containing-protein-[Symploca-sp.-SIO2E9]	2607812
NEO97368.1	DndB-ParB-nuclease	GCA_010692645.1	533	Bacteria>Terrabacteria_group>Cyanobacteria/Melainabacteria_group>Cyanobacteria>Oscillatoriophycideae	Symploca_sp._SIO2E9	DGQHR-domain-containing-protein-[Symploca-sp.-SIO2E9]	2607812
NEO31645.1	DndB-ParB-nuclease	GCA_010692425.1	533	Bacteria>Terrabacteria_group>Cyanobacteria/Melainabacteria_group>Cyanobacteria>Oscillatoriophycideae	Symploca_sp._SIO3C6	DGQHR-domain-containing-protein-[Symploca-sp.-SIO3C6]	2607803
NEO31644.1	DndB-ParB-nuclease	GCA_010692425.1	213	Bacteria>Terrabacteria_group>Cyanobacteria/Melainabacteria_group>Cyanobacteria>Oscillatoriophycideae	Symploca_sp._SIO3C6	hypothetical-protein-[Symploca-sp.-SIO3C6]	2607803
MBW4547995.1	DndB-ParB-nuclease	GCA_019358875.1	345	Bacteria>Terrabacteria_group>Cyanobacteria/Melainabacteria_group>Cyanobacteria>Oscillatoriophycideae	Symplocastrum_torsivum_CPER-KK1	DNA-sulfur-modification-protein-DndB-[Symplocastrum-torsivum-CPER-KK1]	450513
MBW4547998.1	DndB-ParB-nuclease	GCA_019358875.1	519	Bacteria>Terrabacteria_group>Cyanobacteria/Melainabacteria_group>Cyanobacteria>Oscillatoriophycideae	Symplocastrum_torsivum_CPER-KK1	hypothetical-protein-[Symplocastrum-torsivum-CPER-KK1]	450513
MBS9772435.1	DndB-ParB-nuclease	GCA_018457135.1	534	Bacteria>Terrabacteria_group>Cyanobacteria/Melainabacteria_group>Cyanobacteria>Oscillatoriophycideae	Trichodesmium_erythraeum_GBRTRLIN201	DGQHR-domain-containing-protein-[Trichodesmium-erythraeum-GBRTRLIN201]	1045867
WP_096830155.1	DndB-ParB-nuclease	GCF_002412335.2	530	Bacteria>Terrabacteria_group>Cyanobacteria/Melainabacteria_group>Cyanobacteria>Oscillatoriophycideae	Tychonema_bourrellyi	DGQHR-domain-containing-protein-[Tychonema-bourrellyi]	54313
WP_096830156.1	DndB-ParB-nuclease	GCF_002412335.2	379	Bacteria>Terrabacteria_group>Cyanobacteria/Melainabacteria_group>Cyanobacteria>Oscillatoriophycideae	Tychonema_bourrellyi	DNA-sulfur-modification-protein-DndB-[Tychonema-bourrellyi]	54313
WP_096830157.1	DndB-ParB-nuclease	GCF_002412335.2	392	Bacteria>Terrabacteria_group>Cyanobacteria/Melainabacteria_group>Cyanobacteria>Oscillatoriophycideae	Tychonema_bourrellyi	DNA-sulfur-modification-protein-DndB-[Tychonema-bourrellyi]	54313
WP_236505271.1	DndB-ParB-nuclease	GCF_021648855.1	457	Bacteria>Terrabacteria_group>Cyanobacteria/Melainabacteria_group>Cyanobacteria>Oscillatoriophycideae	Tychonema_sp._BBK16	DGQHR-domain-containing-protein-[Tychonema-sp.-BBK16]	2699888
WP_236505273.1	DndB-ParB-nuclease	GCF_021648855.1	530	Bacteria>Terrabacteria_group>Cyanobacteria/Melainabacteria_group>Cyanobacteria>Oscillatoriophycideae	Tychonema_sp._BBK16	DGQHR-domain-containing-protein-[Tychonema-sp.-BBK16]	2699888
WP_236505272.1	DndB-ParB-nuclease	GCF_021648855.1	82	Bacteria>Terrabacteria_group>Cyanobacteria/Melainabacteria_group>Cyanobacteria>Oscillatoriophycideae	Tychonema_sp._BBK16	hypothetical-protein-[Tychonema-sp.-BBK16]	2699888
MBE9095929.1	DndB-ParB-nuclease	GCA_015207285.1	530	Bacteria>Terrabacteria_group>Cyanobacteria/Melainabacteria_group>Cyanobacteria>Oscillatoriophycideae	Tychonema_sp._LEGE_07203	DGQHR-domain-containing-protein-[Tychonema-sp.-LEGE-07203]	1828671
WP_194063785.1	DndB-ParB-nuclease	GCF_015207285.1	530	Bacteria>Terrabacteria_group>Cyanobacteria/Melainabacteria_group>Cyanobacteria>Oscillatoriophycideae	Tychonema_sp._LEGE_07203	DGQHR-domain-containing-protein-[Tychonema-sp.-LEGE-07203]	1828671
WP_190435016.1	DndB-ParB-nuclease	GCF_014695285.1	533	Bacteria>Terrabacteria_group>Cyanobacteria/Melainabacteria_group>Cyanobacteria>Oscillatoriophycideae	unclassified_Coleofasciculus	MULTISPECIES:-DGQHR-domain-containing-protein-[unclassified-Coleofasciculus]	2692782
WP_190534471.1	DndB-ParB-nuclease	GCF_014695825.1	533	Bacteria>Terrabacteria_group>Cyanobacteria/Melainabacteria_group>Cyanobacteria>Oscillatoriophycideae	unclassified_Coleofasciculus	MULTISPECIES:-DGQHR-domain-containing-protein-[unclassified-Coleofasciculus]	2692782
WP_193876316.1	DndB-ParB-nuclease	GCF_015207445.1	532	Bacteria>Terrabacteria_group>Cyanobacteria/Melainabacteria_group>Cyanobacteria>Oscillatoriophycideae	unclassified_Coleofasciculus	MULTISPECIES:-DGQHR-domain-containing-protein-[unclassified-Coleofasciculus]	2692782
WP_190435021.1	DndB-ParB-nuclease	GCF_014695285.1	362	Bacteria>Terrabacteria_group>Cyanobacteria/Melainabacteria_group>Cyanobacteria>Oscillatoriophycideae	unclassified_Coleofasciculus	MULTISPECIES:-DNA-sulfur-modification-protein-DndB-[unclassified-Coleofasciculus]	2692782
WP_190435024.1	DndB-ParB-nuclease	GCF_014695285.1	391	Bacteria>Terrabacteria_group>Cyanobacteria/Melainabacteria_group>Cyanobacteria>Oscillatoriophycideae	unclassified_Coleofasciculus	MULTISPECIES:-DNA-sulfur-modification-protein-DndB-[unclassified-Coleofasciculus]	2692782
WP_193876314.1	DndB-ParB-nuclease	GCF_015207445.1	365	Bacteria>Terrabacteria_group>Cyanobacteria/Melainabacteria_group>Cyanobacteria>Oscillatoriophycideae	unclassified_Coleofasciculus	MULTISPECIES:-DNA-sulfur-modification-protein-DndB-[unclassified-Coleofasciculus]	2692782
WP_069969724.1	DndB-ParB-nuclease	GCF_014696935.1	532	Bacteria>Terrabacteria_group>Cyanobacteria/Melainabacteria_group>Cyanobacteria>Oscillatoriophycideae	unclassified_Desertifilum	MULTISPECIES:-DGQHR-domain-containing-protein-[unclassified-Desertifilum]	2621682
WP_069969723.1	DndB-ParB-nuclease	GCF_014696935.1	391	Bacteria>Terrabacteria_group>Cyanobacteria/Melainabacteria_group>Cyanobacteria>Oscillatoriophycideae	unclassified_Desertifilum	MULTISPECIES:-DNA-sulfur-modification-protein-DndB-[unclassified-Desertifilum]	2621682
WP_231636878.1	DndB-ParB-nuclease	GCF_014698175.1	384	Bacteria>Terrabacteria_group>Cyanobacteria/Melainabacteria_group>Cyanobacteria>Oscillatoriophycideae	unclassified_Planktothricoides	DNA-sulfur-modification-protein-DndB-[Planktothricoides-sp.-SR001]	2621081
WP_054468546.1	DndB-ParB-nuclease	GCF_014698175.1	533	Bacteria>Terrabacteria_group>Cyanobacteria/Melainabacteria_group>Cyanobacteria>Oscillatoriophycideae	unclassified_Planktothricoides	MULTISPECIES:-DGQHR-domain-containing-protein-[unclassified-Planktothricoides]	2621081
WP_054468545.1	DndB-ParB-nuclease	GCF_014698175.1	729	Bacteria>Terrabacteria_group>Cyanobacteria/Melainabacteria_group>Cyanobacteria>Oscillatoriophycideae	unclassified_Planktothricoides	MULTISPECIES:-hypothetical-protein-[unclassified-Planktothricoides]	2621081
WP_199315064.1	DndB-ParB-nuclease	GCF_014696265.1	354	Bacteria>Terrabacteria_group>Cyanobacteria/Melainabacteria_group>Cyanobacteria>Oscillatoriophycideae	unclassified_Planktothrix	MULTISPECIES:-DNA-sulfur-modification-protein-DndB-[unclassified-Planktothrix]	2648599
WP_194008800.1	DndB-ParB-nuclease	GCF_015207345.1	530	Bacteria>Terrabacteria_group>Cyanobacteria/Melainabacteria_group>Cyanobacteria>Oscillatoriophycideae	unclassified_Tychonema	MULTISPECIES:-DGQHR-domain-containing-protein-[unclassified-Tychonema]	2642144
WP_194008799.1	DndB-ParB-nuclease	GCF_015207345.1	370	Bacteria>Terrabacteria_group>Cyanobacteria/Melainabacteria_group>Cyanobacteria>Oscillatoriophycideae	unclassified_Tychonema	MULTISPECIES:-DNA-sulfur-modification-protein-DndB-[unclassified-Tychonema]	2642144
WP_106234250.1	DndB-ParB-nuclease	GCF_003003995.1	418	Bacteria>Terrabacteria_group>Cyanobacteria/Melainabacteria_group>Cyanobacteria>Pleurocapsales	Pleurocapsa_sp._CCALA_161	DNA-sulfur-modification-protein-DndB-[Pleurocapsa-sp.-CCALA-161]	2107688
NJK54448.1	DndB-ParB-nuclease	GCA_012030825.1	328	Bacteria>Terrabacteria_group>Cyanobacteria/Melainabacteria_group>Cyanobacteria>Pleurocapsales	Pleurocapsa_sp._SU_5_0	DGQHR-domain-containing-protein-[Pleurocapsa-sp.-SU_5_0]	2720480
WP_015193715.1	DndB-ParB-nuclease	GCF_000317575.1	573	Bacteria>Terrabacteria_group>Cyanobacteria/Melainabacteria_group>Cyanobacteria>Pleurocapsales	Stanieria_cyanosphaera	hypothetical-protein-[Stanieria-cyanosphaera]	102116
WP_088892398.1	DndB-ParB-nuclease	GCF_002215035.1	358	Bacteria>Terrabacteria_group>Cyanobacteria/Melainabacteria_group>Cyanobacteria>Pseudanabaenales	Leptolyngbya_ohadii	DNA-sulfur-modification-protein-DndB-[Leptolyngbya-ohadii]	1962290
WP_035991462.1	DndB-ParB-nuclease	GCF_000763385.1	359	Bacteria>Terrabacteria_group>Cyanobacteria/Melainabacteria_group>Cyanobacteria>Pseudanabaenales	Leptolyngbya_sp._KIOST-1	DNA-sulfur-modification-protein-DndB-[Leptolyngbya-sp.-KIOST-1]	1229172
WP_035991458.1	DndB-ParB-nuclease	GCF_000763385.1	650	Bacteria>Terrabacteria_group>Cyanobacteria/Melainabacteria_group>Cyanobacteria>Pseudanabaenales	Leptolyngbya_sp._KIOST-1	hypothetical-protein-[Leptolyngbya-sp.-KIOST-1]	1229172
WP_068393504.1	DndB-ParB-nuclease	GCF_001548435.1	358	Bacteria>Terrabacteria_group>Cyanobacteria/Melainabacteria_group>Cyanobacteria>Pseudanabaenales	Leptolyngbya_sp._NIES-3755	DNA-sulfur-modification-protein-DndB-[Leptolyngbya-sp.-NIES-3755]	1752064
WP_068387849.1	DndB-ParB-nuclease	GCF_001548435.1	582	Bacteria>Terrabacteria_group>Cyanobacteria/Melainabacteria_group>Cyanobacteria>Pseudanabaenales	Leptolyngbya_sp._NIES-3755	hypothetical-protein-[Leptolyngbya-sp.-NIES-3755]	1752064
MBD1876720.1	DndB-ParB-nuclease	GCA_014695395.1	358	Bacteria>Terrabacteria_group>Cyanobacteria/Melainabacteria_group>Cyanobacteria>Pseudanabaenales	Nodosilinea_sp._FACHB-131	DNA-sulfur-modification-protein-DndB-[Nodosilinea-sp.-FACHB-131]	2692832
WP_190755562.1	DndB-ParB-nuclease	GCF_014695395.1	358	Bacteria>Terrabacteria_group>Cyanobacteria/Melainabacteria_group>Cyanobacteria>Pseudanabaenales	Nodosilinea_sp._FACHB-131	DNA-sulfur-modification-protein-DndB-[Nodosilinea-sp.-FACHB-131]	2692832
NUN66660.1	DndB-ParB-nuclease	GCA_013361095.1	415	Bacteria>Terrabacteria_group>Cyanobacteria/Melainabacteria_group>Cyanobacteria>Pseudanabaenales	Pseudanabaena_biceps	DGQHR-domain-containing-protein-[Pseudanabaena-biceps]	927669
WP_071590138.1	DndB-ParB-nuclease	GCF_000332215.1	409	Bacteria>Terrabacteria_group>Cyanobacteria/Melainabacteria_group>Cyanobacteria>Pseudanabaenales	Pseudanabaena_biceps	DGQHR-domain-containing-protein-[Pseudanabaena-biceps]	927669
WP_175358421.1	DndB-ParB-nuclease	-	415	Bacteria>Terrabacteria_group>Cyanobacteria/Melainabacteria_group>Cyanobacteria>Pseudanabaenales	Pseudanabaena_biceps	DGQHR-domain-containing-protein-[Pseudanabaena-biceps]	927669
WP_175358429.1	DndB-ParB-nuclease	-	526	Bacteria>Terrabacteria_group>Cyanobacteria/Melainabacteria_group>Cyanobacteria>Pseudanabaenales	Pseudanabaena_biceps	DGQHR-domain-containing-protein-[Pseudanabaena-biceps]	927669
NUN66669.1	DndB-ParB-nuclease	GCA_013361095.1	526	Bacteria>Terrabacteria_group>Cyanobacteria/Melainabacteria_group>Cyanobacteria>Pseudanabaenales	Pseudanabaena_biceps	hypothetical-protein-[Pseudanabaena-biceps]	927669
ELS32759.1	DndB-ParB-nuclease	GCA_000332215.1	386	Bacteria>Terrabacteria_group>Cyanobacteria/Melainabacteria_group>Cyanobacteria>Pseudanabaenales	Pseudanabaena_biceps_PCC_7429	DGQHR-domain-protein-[Pseudanabaena-biceps-PCC-7429]	927668
WP_103666896.1	DndB-ParB-nuclease	GCF_002914585.1	325	Bacteria>Terrabacteria_group>Cyanobacteria/Melainabacteria_group>Cyanobacteria>Pseudanabaenales	Pseudanabaena_sp._BC1403	DGQHR-domain-containing-protein-[Pseudanabaena-sp.-BC1403]	2043171
MBD2261074.1	DndB-ParB-nuclease	GCA_014696715.1	359	Bacteria>Terrabacteria_group>Cyanobacteria/Melainabacteria_group>Cyanobacteria>Pseudanabaenales	Pseudanabaena_sp._FACHB-2040	DNA-sulfur-modification-protein-DndB-[Pseudanabaena-sp.-FACHB-2040]	2692859
WP_190488491.1	DndB-ParB-nuclease	GCF_014696715.1	359	Bacteria>Terrabacteria_group>Cyanobacteria/Melainabacteria_group>Cyanobacteria>Pseudanabaenales	Pseudanabaena_sp._FACHB-2040	DNA-sulfur-modification-protein-DndB-[Pseudanabaena-sp.-FACHB-2040]	2692859

Accession	Annotation	GCA	Length	Phyletic.spread	Strain.name	Defline	Species.Taxid
MBD2261075.1	DndB-ParB-nuclease	GCA_014696715.1	746	Bacteria>Terrabacteria_group>Cyanobacteria/Melainabacteria_group>Cyanobacteria>Pseudanabaenales	Pseudanabaena_sp._FACHB-2040	hypothetical-protein-[Pseudanabaena-sp.-FACHB-2040]	2692859
WP_190488493.1	DndB-ParB-nuclease	GCF_014696715.1	746	Bacteria>Terrabacteria_group>Cyanobacteria/Melainabacteria_group>Cyanobacteria>Pseudanabaenales	Pseudanabaena_sp._FACHB-2040	hypothetical-protein-[Pseudanabaena-sp.-FACHB-2040]	2692859
MCA6603889.1	DndB-ParB-nuclease	GCA_020405035.1	397	Bacteria>Terrabacteria_group>Cyanobacteria/Melainabacteria_group>Cyanobacteria>Pseudanabaenales	Pseudanabaena_sp._M007S1SP1A06QC	DGQHR-domain-containing-protein-[Pseudanabaena-sp.-M007S1SP1A06QC]	2771232
MCA6590888.1	DndB-ParB-nuclease	GCA_020404945.1	517	Bacteria>Terrabacteria_group>Cyanobacteria/Melainabacteria_group>Cyanobacteria>Pseudanabaenales	Pseudanabaena_sp._M38BS1SP1A06MG	hypothetical-protein-[Pseudanabaena-sp.-M38BS1SP1A06MG]	2771238
MCA6572550.1	DndB-ParB-nuclease	GCA_020404105.1	517	Bacteria>Terrabacteria_group>Cyanobacteria/Melainabacteria_group>Cyanobacteria>Pseudanabaenales	Pseudanabaena_sp._M53BS1SP1A06MG	hypothetical-protein-[Pseudanabaena-sp.-M53BS1SP1A06MG]	2771239
MCA6602056.1	DndB-ParB-nuclease	GCA_020404965.1	517	Bacteria>Terrabacteria_group>Cyanobacteria/Melainabacteria_group>Cyanobacteria>Pseudanabaenales	Pseudanabaena_sp._M57BS1SP1A06MG	hypothetical-protein-[Pseudanabaena-sp.-M57BS1SP1A06MG]	2771237
NJN24385.1	DndB-ParB-nuclease	GCA_012032835.1	333	Bacteria>Terrabacteria_group>Cyanobacteria/Melainabacteria_group>Cyanobacteria>Synechococcales	Acaryochloridaceae_cyanobacterium_RL_2_7	DGQHR-domain-containing-protein-[Acaryochloridaceae-cyanobacterium-RL_2_7]	2720424
WP_039781824.1	DndB-ParB-nuclease	GCF_000238775.2	359	Bacteria>Terrabacteria_group>Cyanobacteria/Melainabacteria_group>Cyanobacteria>Synechococcales	Acaryochloris_sp._CCMEE_5410	DNA-sulfur-modification-protein-DndB-[Acaryochloris-sp.-CCMEE-5410]	310037
WP_236096064.1	DndB-ParB-nuclease	-	394	Bacteria>Terrabacteria_group>Cyanobacteria/Melainabacteria_group>Cyanobacteria>Synechococcales	Aphanocapsa_montana	DGQHR-domain-containing-protein-[Aphanocapsa-montana]	327565
PSB02517.1	DndB-ParB-nuclease	GCA_003003775.1	661	Bacteria>Terrabacteria_group>Cyanobacteria/Melainabacteria_group>Cyanobacteria>Synechococcales	Merismopedia_glauca_CCAP_1448/3	hypothetical-protein-C7B64_12700-[Merismopedia-glauca-CCAP-1448/3]	1296344
WP_106289029.1	DndB-ParB-nuclease	GCF_003003775.1	661	Bacteria>Terrabacteria_group>Cyanobacteria/Melainabacteria_group>Cyanobacteria>Synechococcales	Merismopedia_glauca	hypothetical-protein-[Merismopedia-glauca]	292586
WP_009634304.1	DndB-ParB-nuclease	GCF_000332075.2	538	Bacteria>Terrabacteria_group>Cyanobacteria/Melainabacteria_group>Cyanobacteria>Synechococcales	Synechocystis_sp._PCC_7509	hypothetical-protein-[Synechocystis-sp.-PCC-7509]	927677
WP_199298349.1	DndB-ParB-nuclease	GCF_014695915.1	222	Bacteria>Terrabacteria_group>Cyanobacteria/Melainabacteria_group>Cyanobacteria>Synechococcales	Trichocoleus_sp._FACHB-40	DGQHR-domain-containing-protein-[Trichocoleus-sp.-FACHB-40]	2692870
WP_190550702.1	DndB-ParB-nuclease	GCF_014695845.1	389	Bacteria>Terrabacteria_group>Cyanobacteria/Melainabacteria_group>Cyanobacteria>Synechococcales	Trichocoleus_sp._FACHB-591	DGQHR-domain-containing-protein-[Trichocoleus-sp.-FACHB-591]	2692872
WP_190550709.1	DndB-ParB-nuclease	GCF_014695845.1	352	Bacteria>Terrabacteria_group>Cyanobacteria/Melainabacteria_group>Cyanobacteria>Synechococcales	Trichocoleus_sp._FACHB-591	DNA-sulfur-modification-protein-DndB-[Trichocoleus-sp.-FACHB-591]	2692872
MBD1932014.1	DndB-ParB-nuclease	GCA_014695675.1	532	Bacteria>Terrabacteria_group>Cyanobacteria/Melainabacteria_group>Cyanobacteria>Synechococcales	Trichocoleus_sp._FACHB-69	DGQHR-domain-containing-protein-[Trichocoleus-sp.-FACHB-69]	2692874
MBD1932016.1	DndB-ParB-nuclease	GCA_014695675.1	387	Bacteria>Terrabacteria_group>Cyanobacteria/Melainabacteria_group>Cyanobacteria>Synechococcales	Trichocoleus_sp._FACHB-69	DGQHR-domain-containing-protein-[Trichocoleus-sp.-FACHB-69]	2692874
WP_190453820.1	DndB-ParB-nuclease	GCF_014695675.1	532	Bacteria>Terrabacteria_group>Cyanobacteria/Melainabacteria_group>Cyanobacteria>Synechococcales	Trichocoleus_sp._FACHB-69	DGQHR-domain-containing-protein-[Trichocoleus-sp.-FACHB-69]	2692874
WP_190453827.1	DndB-ParB-nuclease	-	387	Bacteria>Terrabacteria_group>Cyanobacteria/Melainabacteria_group>Cyanobacteria>Synechococcales	Trichocoleus_sp._FACHB-69	DGQHR-domain-containing-protein-[Trichocoleus-sp.-FACHB-69]	2692874
MBD2065614.1	DndB-ParB-nuclease	GCA_014695815.1	532	Bacteria>Terrabacteria_group>Cyanobacteria/Melainabacteria_group>Cyanobacteria>Synechococcales	Trichocoleus_sp._FACHB-6	DGQHR-domain-containing-protein-[Trichocoleus-sp.-FACHB-6]	2692873
MBD2065616.1	DndB-ParB-nuclease	GCA_014695815.1	387	Bacteria>Terrabacteria_group>Cyanobacteria/Melainabacteria_group>Cyanobacteria>Synechococcales	Trichocoleus_sp._FACHB-6	DGQHR-domain-containing-protein-[Trichocoleus-sp.-FACHB-6]	2692873
MBD1930201.1	DndB-ParB-nuclease	GCA_014695505.1	532	Bacteria>Terrabacteria_group>Cyanobacteria/Melainabacteria_group>Cyanobacteria>Synechococcales	Trichocoleus_sp._FACHB-90	DGQHR-domain-containing-protein-[Trichocoleus-sp.-FACHB-90]	2692876
WP_190445535.1	DndB-ParB-nuclease	GCF_014695505.1	532	Bacteria>Terrabacteria_group>Cyanobacteria/Melainabacteria_group>Cyanobacteria>Synechococcales	Trichocoleus_sp._FACHB-90	DGQHR-domain-containing-protein-[Trichocoleus-sp.-FACHB-90]	2692876
MBD1835179.1	DndB-ParB-nuclease	GCA_014695275.1	532	Bacteria>Terrabacteria_group>Cyanobacteria/Melainabacteria_group>Cyanobacteria>unclassified_Cyanobacteria	Cyanobacteria_bacterium_FACHB-472	DGQHR-domain-containing-protein-[Cyanobacteria-bacterium-FACHB-472]	2692764
MBD1850968.1	DndB-ParB-nuclease	GCA_014695325.1	376	Bacteria>Terrabacteria_group>Cyanobacteria/Melainabacteria_group>Cyanobacteria>unclassified_Cyanobacteria	Cyanobacteria_bacterium_FACHB-502	DGQHR-domain-containing-protein-[Cyanobacteria-bacterium-FACHB-502]	2692765
MBD1850962.1	DndB-ParB-nuclease	GCA_014695325.1	358	Bacteria>Terrabacteria_group>Cyanobacteria/Melainabacteria_group>Cyanobacteria>unclassified_Cyanobacteria	Cyanobacteria_bacterium_FACHB-502	DNA-sulfur-modification-protein-DndB-[Cyanobacteria-bacterium-FACHB-502]	2692765
MBD1850961.1	DndB-ParB-nuclease	GCA_014695325.1	749	Bacteria>Terrabacteria_group>Cyanobacteria/Melainabacteria_group>Cyanobacteria>unclassified_Cyanobacteria	Cyanobacteria_bacterium_FACHB-502	hypothetical-protein-[Cyanobacteria-bacterium-FACHB-502]	2692765
MBU6346203.1	DndB-ParB-nuclease	GCA_018969285.1	517	Bacteria>Terrabacteria_group>Cyanobacteria/Melainabacteria_group>Cyanobacteria>unclassified_Cyanobacteria	Cyanobacteria_bacterium_REEB494	DGQHR-domain-containing-protein-[Cyanobacteria-bacterium-REEB494]	2800107
HCF29984.1	DndB-ParB-nuclease	GCA_003534575.1	667	Bacteria>Terrabacteria_group>Cyanobacteria/Melainabacteria_group>Cyanobacteria>unclassified_Cyanobacteria	Cyanobacteria_bacterium_UBA11049	hypothetical-protein-[Cyanobacteria-bacterium-UBA11049]	2055787
HBL10036.1	DndB-ParB-nuclease	GCA_003504865.1	533	Bacteria>Terrabacteria_group>Cyanobacteria/Melainabacteria_group>Cyanobacteria>unclassified_Cyanobacteria	Cyanobacteria_bacterium_UBA11162	hypothetical-protein-[Cyanobacteria-bacterium-UBA11162]	2055781
HBE21259.1	DndB-ParB-nuclease	GCA_003501765.1	536	Bacteria>Terrabacteria_group>Cyanobacteria/Melainabacteria_group>Cyanobacteria>unclassified_Cyanobacteria	Cyanobacteria_bacterium_UBA11367	hypothetical-protein-[Cyanobacteria-bacterium-UBA11367]	2055774
HBE21261.1	DndB-ParB-nuclease	GCA_003501765.1	391	Bacteria>Terrabacteria_group>Cyanobacteria/Melainabacteria_group>Cyanobacteria>unclassified_Cyanobacteria	Cyanobacteria_bacterium_UBA11367	hypothetical-protein-[Cyanobacteria-bacterium-UBA11367]	2055774
HBE33410.1	DndB-ParB-nuclease	GCA_003501785.1	531	Bacteria>Terrabacteria_group>Cyanobacteria/Melainabacteria_group>Cyanobacteria>unclassified_Cyanobacteria	Cyanobacteria_bacterium_UBA11368	hypothetical-protein-[Cyanobacteria-bacterium-UBA11368]	2055773
HBE33411.1	DndB-ParB-nuclease	GCA_003501785.1	391	Bacteria>Terrabacteria_group>Cyanobacteria/Melainabacteria_group>Cyanobacteria>unclassified_Cyanobacteria	Cyanobacteria_bacterium_UBA11368	hypothetical-protein-[Cyanobacteria-bacterium-UBA11368]	2055773
HAZ49384.1	DndB-ParB-nuclease	GCA_003486305.1	391	Bacteria>Terrabacteria_group>Cyanobacteria/Melainabacteria_group>Cyanobacteria>unclassified_Cyanobacteria	Cyanobacteria_bacterium_UBA11371	hypothetical-protein-[Cyanobacteria-bacterium-UBA11371]	2055770
HAZ49385.1	DndB-ParB-nuclease	GCA_003486305.1	531	Bacteria>Terrabacteria_group>Cyanobacteria/Melainabacteria_group>Cyanobacteria>unclassified_Cyanobacteria	Cyanobacteria_bacterium_UBA11371	hypothetical-protein-[Cyanobacteria-bacterium-UBA11371]	2055770
HAX77612.1	DndB-ParB-nuclease	GCA_003486675.1	534	Bacteria>Terrabacteria_group>Cyanobacteria/Melainabacteria_group>Cyanobacteria>unclassified_Cyanobacteria	Cyanobacteria_bacterium_UBA11372	hypothetical-protein-[Cyanobacteria-bacterium-UBA11372]	2055768
HBQ98598.1	DndB-ParB-nuclease	GCA_003535175.1	399	Bacteria>Terrabacteria_group>Cyanobacteria/Melainabacteria_group>Cyanobacteria>unclassified_Cyanobacteria	Cyanobacteria_bacterium_UBA11691	hypothetical-protein-[Cyanobacteria-bacterium-UBA11691]	2055778
HAG80003.1	DndB-ParB-nuclease	GCA_003450835.1	365	Bacteria>Terrabacteria_group>Cyanobacteria/Melainabacteria_group>Cyanobacteria>unclassified_Cyanobacteria	Cyanobacteria_bacterium_UBA12227	DNA-sulfur-modification-protein-DndB-[Cyanobacteria-bacterium-UBA12227]	2055765
HAG80004.1	DndB-ParB-nuclease	GCA_003450835.1	533	Bacteria>Terrabacteria_group>Cyanobacteria/Melainabacteria_group>Cyanobacteria>unclassified_Cyanobacteria	Cyanobacteria_bacterium_UBA12227	hypothetical-protein-[Cyanobacteria-bacterium-UBA12227]	2055765
HAJ62217.1	DndB-ParB-nuclease	GCA_003454535.1	532	Bacteria>Terrabacteria_group>Cyanobacteria/Melainabacteria_group>Cyanobacteria>unclassified_Cyanobacteria	Cyanobacteria_bacterium_UBA8543	hypothetical-protein-[Cyanobacteria-bacterium-UBA8543]	2055766
HBB35597.1	DndB-ParB-nuclease	GCA_003488275.1	542	Bacteria>Terrabacteria_group>Cyanobacteria/Melainabacteria_group>Cyanobacteria>unclassified_Cyanobacteria	Cyanobacteria_bacterium_UBA9273	hypothetical-protein-[Cyanobacteria-bacterium-UBA9273]	2055791
OWY65644.1	DndB-ParB-nuclease	GCA_002213405.1	538	Bacteria>Terrabacteria_group>Cyanobacteria/Melainabacteria_group>Cyanobacteria>unclassified_Cyanobacteria	cyanobacterium_TDX16	hypothetical-protein-B7486_40985-[cyanobacterium-TDX16]	1503470
PSB22822.1	DndB-ParB-nuclease	GCA_003003615.1	355	Bacteria>Terrabacteria_group>Cyanobacteria/Melainabacteria_group>Cyanobacteria>unclassified_Cyanobacteria	filamentous_cyanobacterium_CCP2	DNA-sulfur-modification-protein-DndB-[filamentous-cyanobacterium-CCP2]	2107700
PSB22820.1	DndB-ParB-nuclease	GCA_003003615.1	678	Bacteria>Terrabacteria_group>Cyanobacteria/Melainabacteria_group>Cyanobacteria>unclassified_Cyanobacteria	filamentous_cyanobacterium_CCP2	hypothetical-protein-C7B76_02585-[filamentous-cyanobacterium-CCP2]	2107700
PSB13849.1	DndB-ParB-nuclease	GCA_003003615.1	395	Bacteria>Terrabacteria_group>Cyanobacteria/Melainabacteria_group>Cyanobacteria>unclassified_Cyanobacteria	filamentous_cyanobacterium_CCP2	hypothetical-protein-C7B76_18390-[filamentous-cyanobacterium-CCP2]	2107700
PSN10279.1	DndB-ParB-nuclease	GCA_003017855.1	261	Bacteria>Terrabacteria_group>Cyanobacteria/Melainabacteria_group>Cyanobacteria>unclassified_Cyanobacteria	filamentous_cyanobacterium_CCT1	DNA-sulfur-modification-protein-DndB,-partial-[filamentous-cyanobacterium-CCT1]	2107702
PSB14497.1	DndB-ParB-nuclease	GCA_003003715.1	262	Bacteria>Terrabacteria_group>Cyanobacteria/Melainabacteria_group>Cyanobacteria>unclassified_Cyanobacteria	filamentous_cyanobacterium_Phorm_46	hypothetical-protein-C7B69_19010,-partial-[filamentous-cyanobacterium-Phorm-46]	2107705
WP_191300568.1	DndB-ParB-nuclease	GCF_014653295.1	397	Bacteria>Terrabacteria_group>Deinococcus-Thermus>Deinococci>Deinococcales	Deinococcus_indicus	DGQHR-domain-containing-protein-[Deinococcus-indicus]	223556
WP_191300558.1	DndB-ParB-nuclease	GCF_014653295.1	374	Bacteria>Terrabacteria_group>Deinococcus-Thermus>Deinococci>Deinococcales	Deinococcus_indicus	DNA-sulfur-modification-protein-DndB-[Deinococcus-indicus]	223556
GHG32600.1	DndB-ParB-nuclease	GCA_014653295.1	374	Bacteria>Terrabacteria_group>Deinococcus-Thermus>Deinococci>Deinococcales	Deinococcus_indicus	hypothetical-protein-GCM10017784_27660-[Deinococcus-indicus]	223556
GHG32627.1	DndB-ParB-nuclease	GCA_014653295.1	397	Bacteria>Terrabacteria_group>Deinococcus-Thermus>Deinococci>Deinococcales	Deinococcus_indicus	hypothetical-protein-GCM10017784_27710-[Deinococcus-indicus]	223556
WP_110886483.1	DndB-ParB-nuclease	GCF_003217515.1	545	Bacteria>Terrabacteria_group>Deinococcus-Thermus>Deinococci>Deinococcales	Deinococcus_yavapaiensis	DGQHR-domain-containing-protein-[Deinococcus-yavapaiensis]	309889
WP_110886490.1	DndB-ParB-nuclease	GCF_003217515.1	529	Bacteria>Terrabacteria_group>Deinococcus-Thermus>Deinococci>Deinococcales	Deinococcus_yavapaiensis	hypothetical-protein-[Deinococcus-yavapaiensis]	309889
PYE54086.1	DndB-ParB-nuclease	GCA_003217515.1	545	Bacteria>Terrabacteria_group>Deinococcus-Thermus>Deinococci>Deinococcales	Deinococcus_yavapaiensis_KR-236	DGQHR-domain-containing-protein-[Deinococcus-yavapaiensis-KR-236]	694435
PYE54094.1	DndB-ParB-nuclease	GCA_003217515.1	529	Bacteria>Terrabacteria_group>Deinococcus-Thermus>Deinococci>Deinococcales	Deinococcus_yavapaiensis_KR-236	DGQHR-domain-containing-protein-[Deinococcus-yavapaiensis-KR-236]	694435
WP_188207538.1	DndB-ParB-nuclease	GCF_014595945.1	366	Bacteria>Terrabacteria_group>Firmicutes>Bacilli>Bacillales	Alkalibacillus_aidingensis	DNA-sulfur-modification-protein-DndB-[Alkalibacillus-aidingensis]	2747607
WP_224880500.1	DndB-ParB-nuclease	GCF_020171805.1	368	Bacteria>Terrabacteria_group>Firmicutes>Bacilli>Bacillales	Alkalihalobacillus_algicola	DNA-sulfur-modification-protein-DndB-[Alkalihalobacillus-algicola]	225844
WP_034625561.1	DndB-ParB-nuclease	GCF_000787375.1	364	Bacteria>Terrabacteria_group>Firmicutes>Bacilli>Bacillales	Alkalihalobacillus_okhensis	DNA-sulfur-modification-protein-DndB-[Alkalihalobacillus-okhensis]	333138
WP_059104577.1	DndB-ParB-nuclease	GCF_001484965.1	366	Bacteria>Terrabacteria_group>Firmicutes>Bacilli>Bacillales	Alkalihalobacillus_shacheensis	DNA-sulfur-modification-protein-DndB-[Alkalihalobacillus-shacheensis]	1649580
WP_227938997.1	DndB-ParB-nuclease	GCF_020734105.1	364	Bacteria>Terrabacteria_group>Firmicutes>Bacilli>Bacillales	Alkalihalobacillus_sp._TRPH29	DNA-sulfur-modification-protein-DndB-[Alkalihalobacillus-sp.-TRPH29]	2879466
WP_088102991.1	DndB-ParB-nuclease	GCF_002156385.1	364	Bacteria>Terrabacteria_group>Firmicutes>Bacilli>Bacillales	Alkalihalobacillus_urbisdiaboli	DNA-sulfur-modification-protein-DndB-[Alkalihalobacillus-urbisdiaboli]	1960589
WP_144089338.1	DndB-ParB-nuclease	GCF_007559425.1	368	Bacteria>Terrabacteria_group>Firmicutes>Bacilli>Bacillales	Allobacillus_salarius	DNA-sulfur-modification-protein-DndB-[Allobacillus-sp.-SKP4-8]	1955272
WP_212369050.1	DndB-ParB-nuclease	GCF_018138385.1	368	Bacteria>Terrabacteria_group>Firmicutes>Bacilli>Bacillales	Allobacillus_saliphilus	DNA-sulfur-modification-protein-DndB-[Allobacillus-sp.-SKP8-2]	2912308
KIV56065.1	DndB-ParB-nuclease	GCA_000878885.1	368	Bacteria>Terrabacteria_group>Firmicutes>Bacilli>Bacillales	Aneurinibacillus_migulanus	DNA-sulfur-modification-protein-DndB-[Aneurinibacillus-migulanus]	47500
WP_043070627.1	DndB-ParB-nuclease	GCF_000878885.1	368	Bacteria>Terrabacteria_group>Firmicutes>Bacilli>Bacillales	Aneurinibacillus_migulanus	DNA-sulfur-modification-protein-DndB-[Aneurinibacillus-migulanus]	47500
AST07067.1	DndB-ParB-nuclease	GCA_002243705.1	364	Bacteria>Terrabacteria_group>Firmicutes>Bacilli>Bacillales	Anoxybacillus_flavithermus	DNA-sulfur-modification-protein-DndB-[Anoxybacillus-flavithermus]	33934
WP_003395745.1	DndB-ParB-nuclease	GCF_000353425.1	364	Bacteria>Terrabacteria_group>Firmicutes>Bacilli>Bacillales	Anoxybacillus_flavithermus	DNA-sulfur-modification-protein-DndB-[Anoxybacillus-flavithermus]	33934
WP_192953798.1	DndB-ParB-nuclease	GCF_014890455.1	364	Bacteria>Terrabacteria_group>Firmicutes>Bacilli>Bacillales	Anoxybacillus_flavithermus	DNA-sulfur-modification-protein-DndB-[Anoxybacillus-flavithermus]	33934
WP_035049630.1	DndB-ParB-nuclease	GCF_000753875.1	364	Bacteria>Terrabacteria_group>Firmicutes>Bacilli>Bacillales	Anoxybacillus	MULTISPECIES:-DNA-sulfur-modification-protein-DndB-[Anoxybacillus]	150247
WP_171334985.1	DndB-ParB-nuclease	GCF_013096955.1	364	Bacteria>Terrabacteria_group>Firmicutes>Bacilli>Bacillales	Anoxybacillus_sp._EFIL	DNA-sulfur-modification-protein-DndB-[Anoxybacillus-sp.-EFIL]	2508869
KFZ42270.1	DndB-ParB-nuclease	GCA_000753875.1	364	Bacteria>Terrabacteria_group>Firmicutes>Bacilli>Bacillales	Anoxybacillus_sp._KU2-6(11)	DNA-sulfur-modification-protein-DndB-[Anoxybacillus-sp.-KU2-6(11)]	1535751
KAB7707120.1	DndB-ParB-nuclease	GCA_009183655.1	366	Bacteria>Terrabacteria_group>Firmicutes>Bacilli>Bacillales	Bacillus_aerolatus	DNA-sulfur-modification-protein-DndB-[Bacillus-aerolatus]	2653354
WP_152151062.1	DndB-ParB-nuclease	GCF_009183655.1	366	Bacteria>Terrabacteria_group>Firmicutes>Bacilli>Bacillales	Bacillus_aerolatus	DNA-sulfur-modification-protein-DndB-[Bacillus-aerolatus]	2653354
MCC2345184.1	DndB-ParB-nuclease	GCA_020686345.1	364	Bacteria>Terrabacteria_group>Firmicutes>Bacilli>Bacillales	Bacillus_anthracis	DNA-sulfur-modification-protein-DndB-[Bacillus-anthracis]	1392
WP_226542336.1	DndB-ParB-nuclease	GCF_020524035.1	364	Bacteria>Terrabacteria_group>Firmicutes>Bacilli>Bacillales	Bacillus_carboniphilus	DNA-sulfur-modification-protein-DndB-[Bacillus-carboniphilus]	86663
WP_098529188.1	DndB-ParB-nuclease	GCF_002565525.1	388	Bacteria>Terrabacteria_group>Firmicutes>Bacilli>Bacillales	Bacillus_cereus	DGQHR-domain-containing-protein-[Bacillus-cereus]	1396
MBK4743761.1	DndB-ParB-nuclease	GCA_016632305.1	364	Bacteria>Terrabacteria_group>Firmicutes>Bacilli>Bacillales	Bacillus_cereus	DNA-sulfur-modification-protein-DndB-[Bacillus-cereus]	1396
PEQ46989.1	DndB-ParB-nuclease	GCA_002558315.1	364	Bacteria>Terrabacteria_group>Firmicutes>Bacilli>Bacillales	Bacillus_cereus	DNA-sulfur-modification-protein-DndB-[Bacillus-cereus]	1396
PFM97292.1	DndB-ParB-nuclease	GCA_002565765.1	364	Bacteria>Terrabacteria_group>Firmicutes>Bacilli>Bacillales	Bacillus_cereus	DNA-sulfur-modification-protein-DndB-[Bacillus-cereus]	1396
PFN25092.1	DndB-ParB-nuclease	GCA_002565815.1	364	Bacteria>Terrabacteria_group>Firmicutes>Bacilli>Bacillales	Bacillus_cereus	DNA-sulfur-modification-protein-DndB-[Bacillus-cereus]	1396
WP_098139394.1	DndB-ParB-nuclease	GCF_002555205.1	364	Bacteria>Terrabacteria_group>Firmicutes>Bacilli>Bacillales	Bacillus_cereus	DNA-sulfur-modification-protein-DndB-[Bacillus-cereus]	1396
WP_098448193.1	DndB-ParB-nuclease	GCF_002564555.1	364	Bacteria>Terrabacteria_group>Firmicutes>Bacilli>Bacillales	Bacillus_cereus	DNA-sulfur-modification-protein-DndB-[Bacillus-cereus]	1396
WP_098492091.1	DndB-ParB-nuclease	GCF_002565455.1	364	Bacteria>Terrabacteria_group>Firmicutes>Bacilli>Bacillales	Bacillus_cereus	DNA-sulfur-modification-protein-DndB-[Bacillus-cereus]	1396
WP_098529212.1	DndB-ParB-nuclease	GCF_002565525.1	364	Bacteria>Terrabacteria_group>Firmicutes>Bacilli>Bacillales	Bacillus_cereus	DNA-sulfur-modification-protein-DndB-[Bacillus-cereus]	1396
WP_098540559.1	DndB-ParB-nuclease	GCF_002565815.1	364	Bacteria>Terrabacteria_group>Firmicutes>Bacilli>Bacillales	Bacillus_cereus	DNA-sulfur-modification-protein-DndB-[Bacillus-cereus]	1396
WP_098850029.1	DndB-ParB-nuclease	GCF_002582825.1	364	Bacteria>Terrabacteria_group>Firmicutes>Bacilli>Bacillales	Bacillus_cereus	DNA-sulfur-modification-protein-DndB-[Bacillus-cereus]	1396
WP_098213323.1	DndB-ParB-nuclease	GCF_002560525.1	385	Bacteria>Terrabacteria_group>Firmicutes>Bacilli>Bacillales	Bacillus_cereus_group	MULTISPECIES:-DGQHR-domain-containing-protein-[Bacillus-cereus-group]	86661
WP_000340086.1	DndB-ParB-nuclease	GCF_000161155.1	364	Bacteria>Terrabacteria_group>Firmicutes>Bacilli>Bacillales	Bacillus_cereus_group	MULTISPECIES:-DNA-sulfur-modification-protein-DndB-[Bacillus-cereus-group]	86661
WP_070184203.1	DndB-ParB-nuclease	GCF_001757745.1	364	Bacteria>Terrabacteria_group>Firmicutes>Bacilli>Bacillales	Bacillus_cereus_group	MULTISPECIES:-DNA-sulfur-modification-protein-DndB-[Bacillus-cereus-group]	86661
WP_144476270.1	DndB-ParB-nuclease	GCF_016632305.1	364	Bacteria>Terrabacteria_group>Firmicutes>Bacilli>Bacillales	Bacillus_cereus_group	MULTISPECIES:-DNA-sulfur-modification-protein-DndB-[Bacillus-cereus-group]	86661
EEL22919.1	DndB-ParB-nuclease	GCA_000161155.1	364	Bacteria>Terrabacteria_group>Firmicutes>Bacilli>Bacillales	Bacillus_cereus_Rock1-3	DNA-sulfur-modification-protein-DndB-[Bacillus-cereus-Rock1-3]	526981
WP_248138599.1	DndB-ParB-nuclease	GCF_023148655.1	367	Bacteria>Terrabacteria_group>Firmicutes>Bacilli>Bacillales	Bacillus_infantis	DNA-sulfur-modification-protein-DndB-[Bacillus-infantis]	324767
WP_243388501.1	DndB-ParB-nuclease	GCF_022809095.1	408	Bacteria>Terrabacteria_group>Firmicutes>Bacilli>Bacillales	Bacillus_kexueae	DGQHR-domain-containing-protein-[Bacillus-kexueae]	2078952
WP_000384435.1	DndB-ParB-nuclease	GCF_000011625.1	364	Bacteria>Terrabacteria_group>Firmicutes>Bacilli>Bacillales	Bacillus	MULTISPECIES:-DNA-sulfur-modification-protein-DndB-[Bacillus]	1386
WP_000384436.1	DndB-ParB-nuclease	GCF_000293565.1	364	Bacteria>Terrabacteria_group>Firmicutes>Bacilli>Bacillales	Bacillus	MULTISPECIES:-DNA-sulfur-modification-protein-DndB-[Bacillus]	1386
WP_137075227.1	DndB-ParB-nuclease	GCF_005217355.1	364	Bacteria>Terrabacteria_group>Firmicutes>Bacilli>Bacillales	Bacillus	MULTISPECIES:-DNA-sulfur-modification-protein-DndB-[Bacillus]	1386
QWI75851.1	DndB-ParB-nuclease	GCA_018741985.1	365	Bacteria>Terrabacteria_group>Firmicutes>Bacilli>Bacillales	Bacillus_mycoides	DNA-sulfur-modification-protein-DndB-[Bacillus-mycoides]	1405
WP_215580745.1	DndB-ParB-nuclease	GCF_018736705.1	365	Bacteria>Terrabacteria_group>Firmicutes>Bacilli>Bacillales	Bacillus_mycoides	DNA-sulfur-modification-protein-DndB-[Bacillus-mycoides]	1405
WP_150909077.1	DndB-ParB-nuclease	GCF_008764035.1	364	Bacteria>Terrabacteria_group>Firmicutes>Bacilli>Bacillales	Bacillus_pumilus	DNA-sulfur-modification-protein-DndB-[Bacillus-pumilus]	1408
MBY7111612.1	DndB-ParB-nuclease	GCA_019837545.1	364	Bacteria>Terrabacteria_group>Firmicutes>Bacilli>Bacillales	Bacillus_sp._17RED48	DNA-sulfur-modification-protein-DndB-[Bacillus-sp.-17RED48]	2778093
WP_222655120.1	DndB-ParB-nuclease	GCF_019837545.1	364	Bacteria>Terrabacteria_group>Firmicutes>Bacilli>Bacillales	Bacillus_sp._17RED48	DNA-sulfur-modification-protein-DndB-[Bacillus-sp.-17RED48]	2778093
VXB17003.1	DndB-ParB-nuclease	GCA_902506085.1	365	Bacteria>Terrabacteria_group>Firmicutes>Bacilli>Bacillales	Bacillus_sp._349Y	DNA-sulfur-modification-protein-DndB-[Bacillus-sp.-349Y]	2653176
WP_095714459.1	DndB-ParB-nuclease	GCF_002289525.1	364	Bacteria>Terrabacteria_group>Firmicutes>Bacilli>Bacillales	Bacillus_sp._7705b	DNA-sulfur-modification-protein-DndB-[Bacillus-sp.-7705b]	2028568
WP_098118552.1	DndB-ParB-nuclease	GCF_002556365.1	371	Bacteria>Terrabacteria_group>Firmicutes>Bacilli>Bacillales	Bacillus_sp._AFS002410	DNA-sulfur-modification-protein-DndB-[Bacillus-sp.-AFS002410]	2033481
WP_098353862.1	DndB-ParB-nuclease	GCF_002561455.1	354	Bacteria>Terrabacteria_group>Firmicutes>Bacilli>Bacillales	Bacillus_sp._AFS015802	DGQHR-domain-containing-protein-[Bacillus-sp.-AFS015802]	2033486
WP_098353857.1	DndB-ParB-nuclease	GCF_002561455.1	365	Bacteria>Terrabacteria_group>Firmicutes>Bacilli>Bacillales	Bacillus_sp._AFS015802	DNA-sulfur-modification-protein-DndB-[Bacillus-sp.-AFS015802]	2033486
WP_149237989.1	DndB-ParB-nuclease	GCF_008368845.1	364	Bacteria>Terrabacteria_group>Firmicutes>Bacilli>Bacillales	Bacillus_sp._BB51/4	DNA-sulfur-modification-protein-DndB-[Bacillus-sp.-BB51/4]	2217819
TCN11069.1	DndB-ParB-nuclease	GCA_004345465.1	365	Bacteria>Terrabacteria_group>Firmicutes>Bacilli>Bacillales	Bacillus_sp._BK006	DNA-sulfur-modification-protein-DndB-[Bacillus-sp.-BK006]	2512183
WP_043931892.1	DndB-ParB-nuclease	GCF_000613125.1	365	Bacteria>Terrabacteria_group>Firmicutes>Bacilli>Bacillales	Bacillus_sp._EB01	DNA-sulfur-modification-protein-DndB-[Bacillus-sp.-EB01]	1347086
WP_053599591.1	DndB-ParB-nuclease	GCF_001278805.1	365	Bacteria>Terrabacteria_group>Firmicutes>Bacilli>Bacillales	Bacillus_sp._FJAT-18017	DNA-sulfur-modification-protein-DndB-[Bacillus-sp.-FJAT-18017]	1705566
KQL33516.1	DndB-ParB-nuclease	GCA_001420605.1	371	Bacteria>Terrabacteria_group>Firmicutes>Bacilli>Bacillales	Bacillus_sp._FJAT-25509	DNA-sulfur-modification-protein-DndB-[Bacillus-sp.-FJAT-25509]	1712029
WP_213122441.1	DndB-ParB-nuclease	GCF_018343525.1	365	Bacteria>Terrabacteria_group>Firmicutes>Bacilli>Bacillales	Bacillus_sp._FJAT-49754	DNA-sulfur-modification-protein-DndB-[Bacillus-sp.-FJAT-49754]	2833581
WP_026584148.1	DndB-ParB-nuclease	GCF_000518885.1	365	Bacteria>Terrabacteria_group>Firmicutes>Bacilli>Bacillales	Bacillus_sp._J33	DNA-sulfur-modification-protein-DndB-[Bacillus-sp.-J33]	935836
WP_102345890.1	DndB-ParB-nuclease	GCF_900240995.1	365	Bacteria>Terrabacteria_group>Firmicutes>Bacilli>Bacillales	Bacillus_sp._Marseille-P3661	DNA-sulfur-modification-protein-DndB-[Bacillus-sp.-Marseille-P3661]	1936234
OAT86005.1	DndB-ParB-nuclease	GCA_001655735.1	365	Bacteria>Terrabacteria_group>Firmicutes>Bacilli>Bacillales	Bacillus_sp._MKU004	DNA-sulfur-modification-protein-DndB-[Bacillus-sp.-MKU004]	1849370
OAT85999.1	DndB-ParB-nuclease	GCA_001655735.1	419	Bacteria>Terrabacteria_group>Firmicutes>Bacilli>Bacillales	Bacillus_sp._MKU004	hypothetical-protein-A6P54_17795-[Bacillus-sp.-MKU004]	1849370
RFB11806.1	DndB-ParB-nuclease	GCA_003399505.1	364	Bacteria>Terrabacteria_group>Firmicutes>Bacilli>Bacillales	Bacillus_sp._OE	DNA-sulfur-modification-protein-DndB-[Bacillus-sp.-OE]	2293320
WP_224597140.1	DndB-ParB-nuclease	GCF_020165985.1	367	Bacteria>Terrabacteria_group>Firmicutes>Bacilli>Bacillales	Bacillus_sp._RAR_GA_16	DNA-sulfur-modification-protein-DndB-[Bacillus-sp.-RAR_GA_16]	2876774
NMH73564.1	DndB-ParB-nuclease	GCA_012911845.1	365	Bacteria>Terrabacteria_group>Firmicutes>Bacilli>Bacillales	Bacillus_sp._RO2	DNA-sulfur-modification-protein-DndB-[Bacillus-sp.-RO2]	2723913
WP_169291160.1	DndB-ParB-nuclease	GCF_012911845.1	365	Bacteria>Terrabacteria_group>Firmicutes>Bacilli>Bacillales	Bacillus_sp._RO2	DNA-sulfur-modification-protein-DndB-[Bacillus-sp.-RO2]	2723913
QHT48496.1	DndB-ParB-nuclease	GCA_000469135.2	365	Bacteria>Terrabacteria_group>Firmicutes>Bacilli>Bacillales	Bacillus_sp._SB49	DNA-sulfur-modification-protein-DndB-[Bacillus-sp.-SB49]	1071080
WP_026577669.1	DndB-ParB-nuclease	GCF_000469135.2	365	Bacteria>Terrabacteria_group>Firmicutes>Bacilli>Bacillales	Bacillus_sp._SB49	DNA-sulfur-modification-protein-DndB-[Bacillus-sp.-SB49]	1071080
WP_119708127.1	DndB-ParB-nuclease	GCF_003586445.1	368	Bacteria>Terrabacteria_group>Firmicutes>Bacilli>Bacillales	Bacillus_sp._Y1	DNA-sulfur-modification-protein-DndB-[Bacillus-sp.-Y1]	352858
WP_069837233.1	DndB-ParB-nuclease	GCF_001747445.1	364	Bacteria>Terrabacteria_group>Firmicutes>Bacilli>Bacillales	Bacillus_subtilis	DNA-sulfur-modification-protein-DndB-[Bacillus-subtilis]	1423

Accession	Annotation	GCA	Length	Phyletic.spread	Strain.name	Defline	Species.Taxid
WP_103031520.1	DndB-ParB-nuclease	GCF_002893805.1	364	Bacteria>Terrabacteria_group>Firmicutes>Bacilli>Bacillales	Bacillus_subtilis	DNA-sulfur-modification-protein-DndB-[Bacillus-subtilis]	1423
WP_070184199.1	DndB-ParB-nuclease	GCF_001757745.1	385	Bacteria>Terrabacteria_group>Firmicutes>Bacilli>Bacillales	Bacillus_thuringiensis	DGQHR-domain-containing-protein-[Bacillus-thuringiensis]	1428
ALQ66685.1	DndB-ParB-nuclease	GCA_001455345.1	364	Bacteria>Terrabacteria_group>Firmicutes>Bacilli>Bacillales	Bacillus_thuringiensis	DNA-sulfur-modification-protein-DndB-[Bacillus-thuringiensis]	1428
PFJ44430.1	DndB-ParB-nuclease	GCA_002566135.1	364	Bacteria>Terrabacteria_group>Firmicutes>Bacilli>Bacillales	Bacillus_thuringiensis	DNA-sulfur-modification-protein-DndB-[Bacillus-thuringiensis]	1428
PFN50598.1	DndB-ParB-nuclease	GCA_002565105.1	364	Bacteria>Terrabacteria_group>Firmicutes>Bacilli>Bacillales	Bacillus_thuringiensis	DNA-sulfur-modification-protein-DndB-[Bacillus-thuringiensis]	1428
PFV26121.1	DndB-ParB-nuclease	GCA_002570925.1	364	Bacteria>Terrabacteria_group>Firmicutes>Bacilli>Bacillales	Bacillus_thuringiensis	DNA-sulfur-modification-protein-DndB-[Bacillus-thuringiensis]	1428
WP_000384437.1	DndB-ParB-nuclease	GCF_000161655.1	364	Bacteria>Terrabacteria_group>Firmicutes>Bacilli>Bacillales	Bacillus_thuringiensis	DNA-sulfur-modification-protein-DndB-[Bacillus-thuringiensis]	1428
WP_098516462.1	DndB-ParB-nuclease	GCF_002566135.1	364	Bacteria>Terrabacteria_group>Firmicutes>Bacilli>Bacillales	Bacillus_thuringiensis	DNA-sulfur-modification-protein-DndB-[Bacillus-thuringiensis]	1428
WP_098675166.1	DndB-ParB-nuclease	GCF_002576925.1	364	Bacteria>Terrabacteria_group>Firmicutes>Bacilli>Bacillales	Bacillus_thuringiensis	DNA-sulfur-modification-protein-DndB-[Bacillus-thuringiensis]	1428
OUA58856.1	DndB-ParB-nuclease	GCA_002147655.1	364	Bacteria>Terrabacteria_group>Firmicutes>Bacilli>Bacillales	Bacillus_thuringiensis_serovar_aizawai	DNA-sulfur-modification-protein-DndB-[Bacillus-thuringiensis-serovar-aizawai]	1433
OUA03849.1	DndB-ParB-nuclease	GCA_002147505.1	364	Bacteria>Terrabacteria_group>Firmicutes>Bacilli>Bacillales	Bacillus_thuringiensis_serovar_finitimus	DNA-sulfur-modification-protein-DndB-[Bacillus-thuringiensis-serovar-finitimus]	29337
WP_224993393.1	DndB-ParB-nuclease	GCF_020172005.1	386	Bacteria>Terrabacteria_group>Firmicutes>Bacilli>Bacillales	Bacillus_toyonensis	DGQHR-domain-containing-protein-[Bacillus-toyonensis]	155322
PHG68930.1	DndB-ParB-nuclease	GCA_002589375.1	364	Bacteria>Terrabacteria_group>Firmicutes>Bacilli>Bacillales	Bacillus_toyonensis	DNA-sulfur-modification-protein-DndB-[Bacillus-toyonensis]	155322
WP_098908845.1	DndB-ParB-nuclease	GCF_002583715.1	364	Bacteria>Terrabacteria_group>Firmicutes>Bacilli>Bacillales	Bacillus_toyonensis	DNA-sulfur-modification-protein-DndB-[Bacillus-toyonensis]	155322
WP_098954257.1	DndB-ParB-nuclease	GCF_002589375.1	364	Bacteria>Terrabacteria_group>Firmicutes>Bacilli>Bacillales	Bacillus_toyonensis	DNA-sulfur-modification-protein-DndB-[Bacillus-toyonensis]	155322
WP_224993410.1	DndB-ParB-nuclease	GCF_020172005.1	364	Bacteria>Terrabacteria_group>Firmicutes>Bacilli>Bacillales	Bacillus_toyonensis	DNA-sulfur-modification-protein-DndB-[Bacillus-toyonensis]	155322
EJV69670.1	DndB-ParB-nuclease	GCA_000293565.1	364	Bacteria>Terrabacteria_group>Firmicutes>Bacilli>Bacillales	Bacillus_wiedmannii	DNA-sulfur-modification-protein-DndB-[Bacillus-wiedmannii]	1890302
PEO17481.1	DndB-ParB-nuclease	GCA_002556915.1	364	Bacteria>Terrabacteria_group>Firmicutes>Bacilli>Bacillales	Bacillus_wiedmannii	DNA-sulfur-modification-protein-DndB-[Bacillus-wiedmannii]	1890302
WP_097994018.1	DndB-ParB-nuclease	GCF_002556405.1	364	Bacteria>Terrabacteria_group>Firmicutes>Bacilli>Bacillales	Bacillus_wiedmannii	DNA-sulfur-modification-protein-DndB-[Bacillus-wiedmannii]	1890302
WP_213421292.1	DndB-ParB-nuclease	GCF_018390575.1	367	Bacteria>Terrabacteria_group>Firmicutes>Bacilli>Bacillales	Bhargavaea_massiliensis	DNA-sulfur-modification-protein-DndB-[Bhargavaea-sp.-Marseille-Q1000]	2697500
WP_122953198.1	DndB-ParB-nuclease	GCF_006540025.1	362	Bacteria>Terrabacteria_group>Firmicutes>Bacilli>Bacillales	Brevibacillus_agri	DNA-sulfur-modification-protein-DndB-[Brevibacillus-agri]	51101
WP_218015228.1	DndB-ParB-nuclease	GCF_006170445.1	363	Bacteria>Terrabacteria_group>Firmicutes>Bacilli>Bacillales	Brevibacillus_sp._CHY01	DNA-sulfur-modification-protein-DndB-[Brevibacillus-sp.-CHY01]	1844116
HBZ83217.1	DndB-ParB-nuclease	GCA_003521185.1	362	Bacteria>Terrabacteria_group>Firmicutes>Bacilli>Bacillales	Brevibacillus_sp.	DNA-sulfur-modification-protein-DndB-[Brevibacillus-sp.]	1882945
WP_066465819.1	DndB-ParB-nuclease	GCF_001700325.1	354	Bacteria>Terrabacteria_group>Firmicutes>Bacilli>Bacillales	Caryophanon_latum	DNA-sulfur-modification-protein-DndB-[Caryophanon-latum]	33977
RIE01533.1	DndB-ParB-nuclease	GCA_003570905.1	366	Bacteria>Terrabacteria_group>Firmicutes>Bacilli>Bacillales	Cohnella_faecalis	DNA-sulfur-modification-protein-DndB-[Cohnella-faecalis]	2315694
WP_035333447.1	DndB-ParB-nuclease	GCF_000565285.1	365	Bacteria>Terrabacteria_group>Firmicutes>Bacilli>Bacillales	Cytobacillus_firmus	DNA-sulfur-modification-protein-DndB-[Cytobacillus-firmus]	1399
WP_058763155.1	DndB-ParB-nuclease	GCF_001482445.1	366	Bacteria>Terrabacteria_group>Firmicutes>Bacilli>Bacillales	Exiguobacterium_chiriqhucha	DNA-sulfur-modification-protein-DndB-[Exiguobacterium-chiriqhucha]	1385984
WP_075642727.1	DndB-ParB-nuclease	GCF_018618985.1	367	Bacteria>Terrabacteria_group>Firmicutes>Bacilli>Bacillales	Exiguobacterium	MULTISPECIES:-DNA-sulfur-modification-protein-DndB-[Exiguobacterium]	33986
VWX38605.1	DndB-ParB-nuclease	GCA_902506315.1	367	Bacteria>Terrabacteria_group>Firmicutes>Bacilli>Bacillales	Exiguobacterium_oxidotolerans	DNA-sulfur-modification-protein-DndB-[Exiguobacterium-oxidotolerans]	223958
WP_034809828.1	DndB-ParB-nuclease	GCF_000696055.1	366	Bacteria>Terrabacteria_group>Firmicutes>Bacilli>Bacillales	Exiguobacterium_sp._AB2	DNA-sulfur-modification-protein-DndB-[Exiguobacterium-sp.-AB2]	1484479
WP_128122846.1	DndB-ParB-nuclease	GCF_003467445.1	366	Bacteria>Terrabacteria_group>Firmicutes>Bacilli>Bacillales	Exiguobacterium_sp._AM39-5BH	DNA-sulfur-modification-protein-DndB-[Exiguobacterium-sp.-AM39-5BH]	2292355
ACQ71509.1	DndB-ParB-nuclease	GCA_000023045.1	366	Bacteria>Terrabacteria_group>Firmicutes>Bacilli>Bacillales	Exiguobacterium_sp._AT1b	DNA-sulfur-modification-protein-DndB-[Exiguobacterium-sp.-AT1b]	360911
WP_211781518.1	DndB-ParB-nuclease	GCF_018255795.1	366	Bacteria>Terrabacteria_group>Firmicutes>Bacilli>Bacillales	Exiguobacterium_sp._PFWT01	DNA-sulfur-modification-protein-DndB-[Exiguobacterium-sp.-PFWT01]	2829816
WP_114596993.1	DndB-ParB-nuclease	GCF_003339585.1	367	Bacteria>Terrabacteria_group>Firmicutes>Bacilli>Bacillales	Exiguobacterium_sp._RIT594	DNA-sulfur-modification-protein-DndB-[Exiguobacterium-sp.-RIT594]	2282449
WP_214789484.1	DndB-ParB-nuclease	GCF_018617485.1	365	Bacteria>Terrabacteria_group>Firmicutes>Bacilli>Bacillales	Exiguobacterium_sp._s124	DNA-sulfur-modification-protein-DndB-[Exiguobacterium-sp.-s124]	2751268
WP_131503322.1	DndB-ParB-nuclease	GCF_018618775.1	367	Bacteria>Terrabacteria_group>Firmicutes>Bacilli>Bacillales	Exiguobacterium_sp._s138	DNA-sulfur-modification-protein-DndB-[Exiguobacterium-sp.-s138]	2751202
WP_016510894.1	DndB-ParB-nuclease	-	366	Bacteria>Terrabacteria_group>Firmicutes>Bacilli>Bacillales	Exiguobacterium_sp._S17	DNA-sulfur-modification-protein-DndB-[Exiguobacterium-sp.-S17]	856854
WP_214782482.1	DndB-ParB-nuclease	GCF_018617295.1	366	Bacteria>Terrabacteria_group>Firmicutes>Bacilli>Bacillales	Exiguobacterium_sp._S3	DNA-sulfur-modification-protein-DndB-[Exiguobacterium-sp.-S3]	483245
WP_131443475.1	DndB-ParB-nuclease	GCF_004337195.1	366	Bacteria>Terrabacteria_group>Firmicutes>Bacilli>Bacillales	Exiguobacterium_sp._SH0S7	DNA-sulfur-modification-protein-DndB-[Exiguobacterium-sp.-SH0S7]	2510951
WP_131457460.1	DndB-ParB-nuclease	GCF_004337045.1	366	Bacteria>Terrabacteria_group>Firmicutes>Bacilli>Bacillales	Exiguobacterium_sp._SH5S4	DNA-sulfur-modification-protein-DndB-[Exiguobacterium-sp.-SH5S4]	2510961
WP_131433395.1	DndB-ParB-nuclease	GCF_004337025.1	366	Bacteria>Terrabacteria_group>Firmicutes>Bacilli>Bacillales	Exiguobacterium_sp._SL-10	DNA-sulfur-modification-protein-DndB-[Exiguobacterium-sp.-SL-10]	2510962
WP_222462160.1	DndB-ParB-nuclease	GCF_019801215.1	366	Bacteria>Terrabacteria_group>Firmicutes>Bacilli>Bacillales	Fictibacillus_nanhaiensis	DNA-sulfur-modification-protein-DndB-[Fictibacillus-nanhaiensis]	742169
WP_099353727.1	DndB-ParB-nuclease	GCF_002734285.1	366	Bacteria>Terrabacteria_group>Firmicutes>Bacilli>Bacillales	Fredinandcohnia_onubensis	DNA-sulfur-modification-protein-DndB-[Fredinandcohnia-onubensis]	1571209
ODG91503.1	DndB-ParB-nuclease	GCA_001712755.1	371	Bacteria>Terrabacteria_group>Firmicutes>Bacilli>Bacillales	Gottfriedia_luciferensis	DNA-sulfur-modification-protein-DndB-[Gottfriedia-luciferensis]	178774
WP_069034263.1	DndB-ParB-nuclease	GCF_001712755.1	371	Bacteria>Terrabacteria_group>Firmicutes>Bacilli>Bacillales	Gottfriedia_luciferensis	DNA-sulfur-modification-protein-DndB-[Gottfriedia-luciferensis]	178774
WP_146813152.1	DndB-ParB-nuclease	GCF_007991335.1	365	Bacteria>Terrabacteria_group>Firmicutes>Bacilli>Bacillales	Halobacillus_faecis	DNA-sulfur-modification-protein-DndB-[Halobacillus-faecis]	360184
MYL22007.1	DndB-ParB-nuclease	GCA_009856285.1	365	Bacteria>Terrabacteria_group>Firmicutes>Bacilli>Bacillales	Halobacillus_litoralis	DNA-sulfur-modification-protein-DndB-[Halobacillus-litoralis]	45668
WP_160839924.1	DndB-ParB-nuclease	GCF_009856285.1	365	Bacteria>Terrabacteria_group>Firmicutes>Bacilli>Bacillales	Halobacillus_litoralis	DNA-sulfur-modification-protein-DndB-[Halobacillus-litoralis]	45668
WP_079478870.1	DndB-ParB-nuclease	GCF_900166575.1	365	Bacteria>Terrabacteria_group>Firmicutes>Bacilli>Bacillales	Halobacillus_salinus	DNA-sulfur-modification-protein-DndB-[Halobacillus-salinus]	192814
WP_089830409.1	DndB-ParB-nuclease	GCF_900115605.1	365	Bacteria>Terrabacteria_group>Firmicutes>Bacilli>Bacillales	Halolactibacillus_halophilus	DNA-sulfur-modification-protein-DndB-[Halolactibacillus-halophilus]	306540
ATY86444.1	DndB-ParB-nuclease	GCA_002804065.1	373	Bacteria>Terrabacteria_group>Firmicutes>Bacilli>Bacillales	Kyrpidia_spormannii	DNA-sulfur-modification-protein-DndB-[Kyrpidia-spormannii]	2055160
WP_198592134.1	DndB-ParB-nuclease	GCF_002804065.1	370	Bacteria>Terrabacteria_group>Firmicutes>Bacilli>Bacillales	Kyrpidia_spormannii	DNA-sulfur-modification-protein-DndB-[Kyrpidia-spormannii]	2055160
WP_112116588.1	DndB-ParB-nuclease	GCF_900445455.1	428	Bacteria>Terrabacteria_group>Firmicutes>Bacilli>Bacillales	Lysinibacillus_capsici	DGQHR-domain-containing-protein-[Lysinibacillus-capsici]	2115968
WP_225340335.1	DndB-ParB-nuclease	GCF_020217405.1	331	Bacteria>Terrabacteria_group>Firmicutes>Bacilli>Bacillales	Lysinibacillus_capsici	DGQHR-domain-containing-protein-[Lysinibacillus-capsici]	2115968
WP_112116576.1	DndB-ParB-nuclease	GCF_900445455.1	350	Bacteria>Terrabacteria_group>Firmicutes>Bacilli>Bacillales	Lysinibacillus_capsici	DNA-sulfur-modification-protein-DndB-[Lysinibacillus-capsici]	2115968
WP_193832993.1	DndB-ParB-nuclease	GCF_015161405.1	350	Bacteria>Terrabacteria_group>Firmicutes>Bacilli>Bacillales	Lysinibacillus_fusiformis	DNA-sulfur-modification-protein-DndB-[Lysinibacillus-fusiformis]	28031
UDK97508.1	DndB-ParB-nuclease	GCA_020546525.1	331	Bacteria>Terrabacteria_group>Firmicutes>Bacilli>Bacillales	Lysinibacillus_sphaericus	DGQHR-domain-containing-protein-[Lysinibacillus-sphaericus]	1421
WP_229387045.1	DndB-ParB-nuclease	GCF_020546525.1	331	Bacteria>Terrabacteria_group>Firmicutes>Bacilli>Bacillales	Lysinibacillus_sphaericus	DGQHR-domain-containing-protein-[Lysinibacillus-sphaericus]	1421
WP_064205172.1	DndB-ParB-nuclease	GCF_015234955.1	579	Bacteria>Terrabacteria_group>Firmicutes>Bacilli>Bacillales	Mammaliicoccus_lentus	AIPR-family-protein-[Mammaliicoccus-lentus]	42858
WP_079476235.1	DndB-ParB-nuclease	GCF_900166605.1	366	Bacteria>Terrabacteria_group>Firmicutes>Bacilli>Bacillales	Marinococcus_halophilus	DNA-sulfur-modification-protein-DndB-[Marinococcus-halophilus]	1371
WP_046522702.1	DndB-ParB-nuclease	GCF_000986785.1	384	Bacteria>Terrabacteria_group>Firmicutes>Bacilli>Bacillales	Mesobacillus_campisalis	DGQHR-domain-containing-protein-[Mesobacillus-campisalis]	1408103
WP_046522707.1	DndB-ParB-nuclease	GCF_000986785.1	365	Bacteria>Terrabacteria_group>Firmicutes>Bacilli>Bacillales	Mesobacillus_campisalis	DNA-sulfur-modification-protein-DndB-[Mesobacillus-campisalis]	1408103
WP_066227733.1	DndB-ParB-nuclease	GCF_001591625.1	364	Bacteria>Terrabacteria_group>Firmicutes>Bacilli>Bacillales	Metabacillus_fastidiosus	DNA-sulfur-modification-protein-DndB-[Metabacillus-fastidiosus]	1458
WP_154308347.1	DndB-ParB-nuclease	GCF_009674805.1	365	Bacteria>Terrabacteria_group>Firmicutes>Bacilli>Bacillales	Metabacillus_lacus	DNA-sulfur-modification-protein-DndB-[Bacillus-lacus]	1983721
WP_144568035.1	DndB-ParB-nuclease	GCF_007828895.1	377	Bacteria>Terrabacteria_group>Firmicutes>Bacilli>Bacillales	Neobacillus_bataviensis	DGQHR-domain-containing-protein-[Neobacillus-bataviensis]	220685
WP_144568021.1	DndB-ParB-nuclease	GCF_007828895.1	365	Bacteria>Terrabacteria_group>Firmicutes>Bacilli>Bacillales	Neobacillus_bataviensis	DNA-sulfur-modification-protein-DndB-[Neobacillus-bataviensis]	220685
WP_235744143.1	DndB-ParB-nuclease	GCF_021560175.1	365	Bacteria>Terrabacteria_group>Firmicutes>Bacilli>Bacillales	Neobacillus_drentensis	DNA-sulfur-modification-protein-DndB-[Neobacillus-drentensis]	220684
QAV26936.1	DndB-ParB-nuclease	GCA_004116995.1	364	Bacteria>Terrabacteria_group>Firmicutes>Bacilli>Bacillales	Neobacillus_thermocopriae	DNA-sulfur-modification-protein-DndB-[Neobacillus-thermocopriae]	1215031
WP_133386541.1	DndB-ParB-nuclease	GCF_004358835.1	429	Bacteria>Terrabacteria_group>Firmicutes>Bacilli>Bacillales	Paenibacillus_amylolyticus	DGQHR-domain-containing-protein-[Paenibacillus-amylolyticus]	1451
WP_076332994.1	DndB-ParB-nuclease	GCF_001956055.1	366	Bacteria>Terrabacteria_group>Firmicutes>Bacilli>Bacillales	Paenibacillus_amylolyticus	DNA-sulfur-modification-protein-DndB-[Paenibacillus-amylolyticus]	1451
WP_127547357.1	DndB-ParB-nuclease	GCF_004001025.1	366	Bacteria>Terrabacteria_group>Firmicutes>Bacilli>Bacillales	Paenibacillus_amylolyticus	DNA-sulfur-modification-protein-DndB-[Paenibacillus-amylolyticus]	1451
WP_133386545.1	DndB-ParB-nuclease	GCF_004358835.1	366	Bacteria>Terrabacteria_group>Firmicutes>Bacilli>Bacillales	Paenibacillus_amylolyticus	DNA-sulfur-modification-protein-DndB-[Paenibacillus-amylolyticus]	1451
WP_134757392.1	DndB-ParB-nuclease	GCF_004514475.2	363	Bacteria>Terrabacteria_group>Firmicutes>Bacilli>Bacillales	Paenibacillus_athensensis	DNA-sulfur-modification-protein-DndB-[Paenibacillus-athensensis]	1967502
WP_216515415.1	DndB-ParB-nuclease	GCF_018917465.1	367	Bacteria>Terrabacteria_group>Firmicutes>Bacilli>Bacillales	Paenibacillus_barcinonensis	DNA-sulfur-modification-protein-DndB-[Paenibacillus-barcinonensis]	198119
WP_095267901.1	DndB-ParB-nuclease	GCF_002272015.1	364	Bacteria>Terrabacteria_group>Firmicutes>Bacilli>Bacillales	Paenibacillus_campinasensis	DNA-sulfur-modification-protein-DndB-[Paenibacillus-campinasensis]	66347
WP_135556455.1	DndB-ParB-nuclease	GCF_004764705.1	365	Bacteria>Terrabacteria_group>Firmicutes>Bacilli>Bacillales	Paenibacillus_cymbidii	DNA-sulfur-modification-protein-DndB-[Paenibacillus-cymbidii]	1639034
WP_046228169.1	DndB-ParB-nuclease	GCF_000971985.1	384	Bacteria>Terrabacteria_group>Firmicutes>Bacilli>Bacillales	Paenibacillus_dauci	DGQHR-domain-containing-protein-[Paenibacillus-dauci]	1567106
WP_046228175.1	DndB-ParB-nuclease	GCF_000971985.1	366	Bacteria>Terrabacteria_group>Firmicutes>Bacilli>Bacillales	Paenibacillus_dauci	DNA-sulfur-modification-protein-DndB-[Paenibacillus-dauci]	1567106
WP_127484301.1	DndB-ParB-nuclease	GCF_004000785.1	361	Bacteria>Terrabacteria_group>Firmicutes>Bacilli>Bacillales	Paenibacillus_ehimensis	DNA-sulfur-modification-protein-DndB-[Paenibacillus-ehimensis]	79264
WP_163853506.1	DndB-ParB-nuclease	GCF_010894335.1	369	Bacteria>Terrabacteria_group>Firmicutes>Bacilli>Bacillales	Paenibacillus_elgii	DNA-sulfur-modification-protein-DndB-[Paenibacillus-elgii]	189691
WP_152396588.1	DndB-ParB-nuclease	GCF_009363075.1	363	Bacteria>Terrabacteria_group>Firmicutes>Bacilli>Bacillales	Paenibacillus_guangzhouensis	DNA-sulfur-modification-protein-DndB-[Paenibacillus-guangzhouensis]	1473112
WP_013920774.1	DndB-ParB-nuclease	GCF_000218915.1	365	Bacteria>Terrabacteria_group>Firmicutes>Bacilli>Bacillales	Paenibacillus_mucilaginosus	DNA-sulfur-modification-protein-DndB-[Paenibacillus-mucilaginosus]	61624
WP_076212773.1	DndB-ParB-nuclease	GCF_001954655.1	365	Bacteria>Terrabacteria_group>Firmicutes>Bacilli>Bacillales	Paenibacillus_odorifer	DNA-sulfur-modification-protein-DndB-[Paenibacillus-odorifer]	189426
WP_141695512.1	DndB-ParB-nuclease	GCF_017589695.1	367	Bacteria>Terrabacteria_group>Firmicutes>Bacilli>Bacillales	Paenibacillus_polymyxa	DNA-sulfur-modification-protein-DndB-[Paenibacillus-polymyxa]	1406
WP_111149652.1	DndB-ParB-nuclease	GCF_003233845.1	362	Bacteria>Terrabacteria_group>Firmicutes>Bacilli>Bacillales	Paenibacillus_sambharensis	DNA-sulfur-modification-protein-DndB-[Paenibacillus-sambharensis]	1803190
WP_137221273.1	DndB-ParB-nuclease	GCF_005233705.1	359	Bacteria>Terrabacteria_group>Firmicutes>Bacilli>Bacillales	Paenibacillus_sp._CFBP13512	DNA-sulfur-modification-protein-DndB-[Paenibacillus-sp.-CFBP13512]	2184007
WP_192132716.1	DndB-ParB-nuclease	GCF_014842675.1	366	Bacteria>Terrabacteria_group>Firmicutes>Bacilli>Bacillales	Paenibacillus_sp._CFBP_13594	DNA-sulfur-modification-protein-DndB-[Paenibacillus-sp.-CFBP-13594]	2774037
WP_200965459.1	DndB-ParB-nuclease	GCF_015472005.1	360	Bacteria>Terrabacteria_group>Firmicutes>Bacilli>Bacillales	Paenibacillus_sp._DA-C8	DNA-sulfur-modification-protein-DndB-[Paenibacillus-sp.-DA-C8]	2749268
WP_236420991.1	DndB-ParB-nuclease	GCF_927798175.1	366	Bacteria>Terrabacteria_group>Firmicutes>Bacilli>Bacillales	Paenibacillus_sp._JJ-223	DNA-sulfur-modification-protein-DndB-[Paenibacillus-sp.-JJ-223]	2905647
WP_056694690.1	DndB-ParB-nuclease	GCF_001426865.1	366	Bacteria>Terrabacteria_group>Firmicutes>Bacilli>Bacillales	Paenibacillus_sp._Root52	DNA-sulfur-modification-protein-DndB-[Paenibacillus-sp.-Root52]	1736552
WP_220030863.1	DndB-ParB-nuclease	GCF_019454085.1	366	Bacteria>Terrabacteria_group>Firmicutes>Bacilli>Bacillales	Paenibacillus_sp._S25	DNA-sulfur-modification-protein-DndB-[Paenibacillus-sp.-S25]	2823905
WP_216791710.1	DndB-ParB-nuclease	-	365	Bacteria>Terrabacteria_group>Firmicutes>Bacilli>Bacillales	Paenibacillus_sp._SM_69	DNA-sulfur-modification-protein-DndB-[Paenibacillus-sp.-SM-69]	2594886
WP_057311485.1	DndB-ParB-nuclease	GCF_001428105.1	363	Bacteria>Terrabacteria_group>Firmicutes>Bacilli>Bacillales	Paenibacillus_sp._Soil766	DGQHR-domain-containing-protein-[Paenibacillus-sp.-Soil766]	1736404
WP_057311489.1	DndB-ParB-nuclease	GCF_001428105.1	362	Bacteria>Terrabacteria_group>Firmicutes>Bacilli>Bacillales	Paenibacillus_sp._Soil766	DNA-sulfur-modification-protein-DndB-[Paenibacillus-sp.-Soil766]	1736404
WP_220350271.1	DndB-ParB-nuclease	GCF_019550795.1	366	Bacteria>Terrabacteria_group>Firmicutes>Bacilli>Bacillales	Paenibacillus_sp._sptzw28	DGQHR-domain-containing-protein-[Paenibacillus-sp.-sptzw28]	715179
WP_220350286.1	DndB-ParB-nuclease	GCF_019550795.1	365	Bacteria>Terrabacteria_group>Firmicutes>Bacilli>Bacillales	Paenibacillus_sp._sptzw28	DNA-sulfur-modification-protein-DndB-[Paenibacillus-sp.-sptzw28]	715179
WP_235123019.1	DndB-ParB-nuclease	GCF_021513275.1	366	Bacteria>Terrabacteria_group>Firmicutes>Bacilli>Bacillales	Paenibacillus_sp._YPD9-1	DNA-sulfur-modification-protein-DndB-[Paenibacillus-sp.-YPD9-1]	2908839
WP_166244017.1	DndB-ParB-nuclease	GCF_011421635.1	362	Bacteria>Terrabacteria_group>Firmicutes>Bacilli>Bacillales	Paenibacillus_turpanensis	DNA-sulfur-modification-protein-DndB-[Paenibacillus-turpanensis]	2689078
WP_213654331.1	DndB-ParB-nuclease	GCF_018403325.1	450	Bacteria>Terrabacteria_group>Firmicutes>Bacilli>Bacillales	Paenibacillus_vini	DGQHR-domain-containing-protein-[Paenibacillus-vini]	1476024
WP_213654336.1	DndB-ParB-nuclease	GCF_018403325.1	364	Bacteria>Terrabacteria_group>Firmicutes>Bacilli>Bacillales	Paenibacillus_vini	DNA-sulfur-modification-protein-DndB-[Paenibacillus-vini]	1476024
WP_154892855.1	DndB-ParB-nuclease	GCF_007679725.1	366	Bacteria>Terrabacteria_group>Firmicutes>Bacilli>Bacillales	Paenibacillus_xylanexedens	DNA-sulfur-modification-protein-DndB-[Paenibacillus-xylanexedens]	528191
WP_043905159.1	DndB-ParB-nuclease	GCF_000632515.1	364	Bacteria>Terrabacteria_group>Firmicutes>Bacilli>Bacillales	Parageobacillus_genomosp._1	DNA-sulfur-modification-protein-DndB-[Parageobacillus-genomosp.-1]	1295642
WP_204539499.1	DndB-ParB-nuclease	GCF_016909175.1	365	Bacteria>Terrabacteria_group>Firmicutes>Bacilli>Bacillales	Peribacillus_deserti	DNA-sulfur-modification-protein-DndB-[Peribacillus-deserti]	673318
WP_127778144.1	DndB-ParB-nuclease	GCF_004006475.1	381	Bacteria>Terrabacteria_group>Firmicutes>Bacilli>Bacillales	Peribacillus_frigoritolerans	DGQHR-domain-containing-protein-[[Brevibacterium]	450367
WP_127778149.1	DndB-ParB-nuclease	GCF_004006475.1	366	Bacteria>Terrabacteria_group>Firmicutes>Bacilli>Bacillales	Peribacillus_frigoritolerans	DNA-sulfur-modification-protein-DndB-[[Brevibacterium]	450367
MBX9955707.1	DndB-ParB-nuclease	GCA_019748875.1	366	Bacteria>Terrabacteria_group>Firmicutes>Bacilli>Bacillales	Peribacillus_simplex	DNA-sulfur-modification-protein-DndB-[Peribacillus-simplex]	1478
WP_144528281.1	DndB-ParB-nuclease	GCF_007680885.1	366	Bacteria>Terrabacteria_group>Firmicutes>Bacilli>Bacillales	Peribacillus_simplex	DNA-sulfur-modification-protein-DndB-[Peribacillus-simplex]	1478
WP_053168284.1	DndB-ParB-nuclease	GCF_001270075.1	365	Bacteria>Terrabacteria_group>Firmicutes>Bacilli>Bacillales	Planococcus_glaciei	DNA-sulfur-modification-protein-DndB-[Planococcus-glaciei]	459472
WP_135501962.1	DndB-ParB-nuclease	GCF_014202615.1	367	Bacteria>Terrabacteria_group>Firmicutes>Bacilli>Bacillales	Planococcus_koreensis	DNA-sulfur-modification-protein-DndB-[Planomicrobium-koreense]	112331
WP_069576770.1	DndB-ParB-nuclease	GCF_001730005.1	367	Bacteria>Terrabacteria_group>Firmicutes>Bacilli>Bacillales	Planococcus_maritimus	DNA-sulfur-modification-protein-DndB-[Planococcus-maritimus]	192421
WP_084243802.1	DndB-ParB-nuclease	GCF_900176575.1	367	Bacteria>Terrabacteria_group>Firmicutes>Bacilli>Bacillales	Planomicrobium_okeanokoites	DNA-sulfur-modification-protein-DndB-[Planomicrobium-okeanokoites]	244
WP_188652385.1	DndB-ParB-nuclease	GCF_014642405.1	365	Bacteria>Terrabacteria_group>Firmicutes>Bacilli>Bacillales	Pontibacillus_salipaludis	DNA-sulfur-modification-protein-DndB-[Pontibacillus-salipaludis]	1697394
GGD08336.1	DndB-ParB-nuclease	GCA_014642405.1	365	Bacteria>Terrabacteria_group>Firmicutes>Bacilli>Bacillales	Pontibacillus_salipaludis	hypothetical-protein-GCM10011389_14880-[Pontibacillus-salipaludis]	1697394
WP_193426779.1	DndB-ParB-nuclease	GCF_014932885.1	423	Bacteria>Terrabacteria_group>Firmicutes>Bacilli>Bacillales	Priestia_aryabhattai	DGQHR-domain-containing-protein-[Priestia-aryabhattai]	412384
MBE5103322.1	DndB-ParB-nuclease	GCA_014932885.1	365	Bacteria>Terrabacteria_group>Firmicutes>Bacilli>Bacillales	Priestia_aryabhattai	DNA-sulfur-modification-protein-DndB-[Priestia-aryabhattai]	412384
WP_193426772.1	DndB-ParB-nuclease	GCF_014932885.1	365	Bacteria>Terrabacteria_group>Firmicutes>Bacilli>Bacillales	Priestia_aryabhattai	DNA-sulfur-modification-protein-DndB-[Priestia-aryabhattai]	412384
QLK09348.1	DndB-ParB-nuclease	GCA_013458535.1	365	Bacteria>Terrabacteria_group>Firmicutes>Bacilli>Bacillales	Priestia_megaterium	DNA-sulfur-modification-protein-DndB-[Priestia-megaterium]	1404
WP_075422633.1	DndB-ParB-nuclease	GCF_001937775.1	365	Bacteria>Terrabacteria_group>Firmicutes>Bacilli>Bacillales	Priestia_megaterium	DNA-sulfur-modification-protein-DndB-[Priestia-megaterium]	1404
WP_098383121.1	DndB-ParB-nuclease	GCF_002562195.1	365	Bacteria>Terrabacteria_group>Firmicutes>Bacilli>Bacillales	Priestia_megaterium	DNA-sulfur-modification-protein-DndB-[Priestia-megaterium]	1404
WP_098804137.1	DndB-ParB-nuclease	GCF_009935415.1	365	Bacteria>Terrabacteria_group>Firmicutes>Bacilli>Bacillales	Priestia_megaterium	DNA-sulfur-modification-protein-DndB-[Priestia-megaterium]	1404
WP_180896545.1	DndB-ParB-nuclease	GCF_013458535.1	365	Bacteria>Terrabacteria_group>Firmicutes>Bacilli>Bacillales	Priestia_megaterium	DNA-sulfur-modification-protein-DndB-[Priestia-megaterium]	1404
WP_098524922.1	DndB-ParB-nuclease	GCF_024169105.1	365	Bacteria>Terrabacteria_group>Firmicutes>Bacilli>Bacillales	Priestia_megaterium	MULTISPECIES:-DNA-sulfur-modification-protein-DndB-[Bacillaceae]	1404
WP_033579258.1	DndB-ParB-nuclease	GCF_015582655.1	365	Bacteria>Terrabacteria_group>Firmicutes>Bacilli>Bacillales	Priestia	MULTISPECIES:-DNA-sulfur-modification-protein-DndB-[Priestia]	2800373
WP_160919827.1	DndB-ParB-nuclease	GCF_009856375.1	368	Bacteria>Terrabacteria_group>Firmicutes>Bacilli>Bacillales	Pseudalkalibacillus_hwajinpoensis	DNA-sulfur-modification-protein-DndB-[Alkalihalobacillus-hwajinpoensis]	208199
WP_226611976.1	DndB-ParB-nuclease	GCF_020524205.1	368	Bacteria>Terrabacteria_group>Firmicutes>Bacilli>Bacillales	Pseudalkalibacillus_hwajinpoensis	DNA-sulfur-modification-protein-DndB-[Alkalihalobacillus-hwajinpoensis]	208199

Accession	Annotation	GCA	Length	Phyletic.spread	Strain.name	Defline	Species.Taxid
WP_159129320.1	DndB-ParB-nuclease	GCF_009806575.1	365	Bacteria>Terrabacteria_group>Firmicutes>Bacilli>Bacillales	Rossellomorea_marisflavi	DNA-sulfur-modification-protein-DndB-[Rossellomorea-marisflavi]	189381
WP_148796762.1	DndB-ParB-nuclease	GCF_008124485.1	365	Bacteria>Terrabacteria_group>Firmicutes>Bacilli>Bacillales	Rossellomorea_marisflavi	MULTISPECIES:-DNA-sulfur-modification-protein-DndB-[Bacillaceae]	189381
WP_060674960.1	DndB-ParB-nuclease	GCF_001305855.1	365	Bacteria>Terrabacteria_group>Firmicutes>Bacilli>Bacillales	Rossellomorea_vietnamensis	DNA-sulfur-modification-protein-DndB-[Rossellomorea-vietnamensis]	218284
NGZ74982.1	DndB-ParB-nuclease	GCA_011090185.1	365	Bacteria>Terrabacteria_group>Firmicutes>Bacilli>Bacillales	Saccharibacillus_alkalitolerans	DNA-sulfur-modification-protein-DndB-[Saccharibacillus-alkalitolerans]	2705290
WP_166273306.1	DndB-ParB-nuclease	GCF_011090185.1	365	Bacteria>Terrabacteria_group>Firmicutes>Bacilli>Bacillales	Saccharibacillus_alkalitolerans	DNA-sulfur-modification-protein-DndB-[Saccharibacillus-alkalitolerans]	2705290
WP_172251711.1	DndB-ParB-nuclease	GCF_013185225.1	365	Bacteria>Terrabacteria_group>Firmicutes>Bacilli>Bacillales	Saccharibacillus_deserti	DNA-sulfur-modification-protein-DndB-[Saccharibacillus-deserti]	1634444
OWA34253.1	DndB-ParB-nuclease	GCA_002189105.1	365	Bacteria>Terrabacteria_group>Firmicutes>Bacilli>Bacillales	Saccharibacillus_sp._O16	DNA-sulfur-modification-protein-DndB-[Saccharibacillus-sp.-O16]	1978120
WP_076558521.1	DndB-ParB-nuclease	GCF_900156645.1	365	Bacteria>Terrabacteria_group>Firmicutes>Bacilli>Bacillales	Salimicrobium_flavidum	DNA-sulfur-modification-protein-DndB-[Salimicrobium-flavidum]	570947
AOM83523.1	DndB-ParB-nuclease	GCA_001721685.1	364	Bacteria>Terrabacteria_group>Firmicutes>Bacilli>Bacillales	Salisediminibacterium_beveridgei	DNA-sulfur-modification-protein-DndB-[Bacillus-beveridgei]	632773
WP_069365494.1	DndB-ParB-nuclease	GCF_001721685.1	364	Bacteria>Terrabacteria_group>Firmicutes>Bacilli>Bacillales	Salisediminibacterium_beveridgei	DNA-sulfur-modification-protein-DndB-[Bacillus-beveridgei]	632773
WP_077622437.1	DndB-ParB-nuclease	GCF_900156915.1	366	Bacteria>Terrabacteria_group>Firmicutes>Bacilli>Bacillales	Sediminibacillus_massiliensis	DNA-sulfur-modification-protein-DndB-[Sediminibacillus-massiliensis]	1926277
WP_008405466.1	DndB-ParB-nuclease	GCF_000298255.1	348	Bacteria>Terrabacteria_group>Firmicutes>Bacilli>Bacillales	Solibacillus_isronensis	DNA-sulfur-modification-protein-DndB-[Solibacillus-isronensis]	412383
ARF14007.1	DndB-ParB-nuclease	GCA_002081995.1	363	Bacteria>Terrabacteria_group>Firmicutes>Bacilli>Bacillales	Sporosarcina_ureae	DNA-sulfur-modification-protein-DndB-[Sporosarcina-ureae]	1571
WP_029055188.1	DndB-ParB-nuclease	GCF_000425545.1	363	Bacteria>Terrabacteria_group>Firmicutes>Bacilli>Bacillales	Sporosarcina_ureae	DNA-sulfur-modification-protein-DndB-[Sporosarcina-ureae]	1571
WP_170061573.1	DndB-ParB-nuclease	GCF_002884435.1	379	Bacteria>Terrabacteria_group>Firmicutes>Bacilli>Bacillales	Terribacillus_goriensis	DGQHR-domain-containing-protein-[Terribacillus-goriensis]	386490
WP_102706499.1	DndB-ParB-nuclease	GCF_002884435.1	375	Bacteria>Terrabacteria_group>Firmicutes>Bacilli>Bacillales	Terribacillus_goriensis	DNA-sulfur-modification-protein-DndB-[Terribacillus-goriensis]	386490
WP_184662908.1	DndB-ParB-nuclease	GCF_014202575.1	364	Bacteria>Terrabacteria_group>Firmicutes>Bacilli>Bacillales	Texcoconibacillus_texcoconensis	DNA-sulfur-modification-protein-DndB-[Texcoconibacillus-texcoconensis]	1095777
WP_062444804.1	DndB-ParB-nuclease	GCF_014635125.1	408	Bacteria>Terrabacteria_group>Firmicutes>Bacilli>Bacillales	Thalassobacillus_devorans	DGQHR-domain-containing-protein-[Thalassobacillus-devorans]	279813
WP_062444830.1	DndB-ParB-nuclease	GCF_014635125.1	366	Bacteria>Terrabacteria_group>Firmicutes>Bacilli>Bacillales	Thalassobacillus_devorans	DNA-sulfur-modification-protein-DndB-[Thalassobacillus-devorans]	279813
WP_132947153.1	DndB-ParB-nuclease	GCF_004341205.1	368	Bacteria>Terrabacteria_group>Firmicutes>Bacilli>Bacillales	Thermolongibacillus_altinsuensis	DNA-sulfur-modification-protein-DndB-[Thermolongibacillus-altinsuensis]	575256
WP_085524477.1	DndB-ParB-nuclease	GCF_900178005.1	367	Bacteria>Terrabacteria_group>Firmicutes>Bacilli>Bacillales	Tuberibacillus_sp._Marseille-P3662	DNA-sulfur-modification-protein-DndB-[Tuberibacillus-sp.-Marseille-P3662]	1965358
WP_098357577.1	DndB-ParB-nuclease	GCF_002561435.1	364	Bacteria>Terrabacteria_group>Firmicutes>Bacilli>Bacillales	unclassified_Bacillus_(in:_Bacteria)	MULTISPECIES:-DNA-sulfur-modification-protein-DndB-[unclassified-Bacillus-(in:-Bacteria)]	185979
WP_214691173.1	DndB-ParB-nuclease	GCF_018617255.1	416	Bacteria>Terrabacteria_group>Firmicutes>Bacilli>Bacillales	unclassified_Exiguobacterium	MULTISPECIES:-DGQHR-domain-containing-protein-[unclassified-Exiguobacterium]	2644629
WP_015881068.1	DndB-ParB-nuclease	GCF_000023045.1	366	Bacteria>Terrabacteria_group>Firmicutes>Bacilli>Bacillales	unclassified_Exiguobacterium	MULTISPECIES:-DNA-sulfur-modification-protein-DndB-[unclassified-Exiguobacterium]	2644629
WP_159174141.1	DndB-ParB-nuclease	GCF_018618695.1	367	Bacteria>Terrabacteria_group>Firmicutes>Bacilli>Bacillales	unclassified_Exiguobacterium	MULTISPECIES:-DNA-sulfur-modification-protein-DndB-[unclassified-Exiguobacterium]	2644629
WP_214691167.1	DndB-ParB-nuclease	GCF_018617255.1	359	Bacteria>Terrabacteria_group>Firmicutes>Bacilli>Bacillales	unclassified_Exiguobacterium	MULTISPECIES:-DNA-sulfur-modification-protein-DndB-[unclassified-Exiguobacterium]	2644629
WP_214763754.1	DndB-ParB-nuclease	GCF_018618015.1	366	Bacteria>Terrabacteria_group>Firmicutes>Bacilli>Bacillales	unclassified_Exiguobacterium	MULTISPECIES:-DNA-sulfur-modification-protein-DndB-[unclassified-Exiguobacterium]	2644629
WP_214798987.1	DndB-ParB-nuclease	GCF_018618175.1	366	Bacteria>Terrabacteria_group>Firmicutes>Bacilli>Bacillales	unclassified_Exiguobacterium	MULTISPECIES:-DNA-sulfur-modification-protein-DndB-[unclassified-Exiguobacterium]	2644629
WP_214882883.1	DndB-ParB-nuclease	GCF_018617815.1	366	Bacteria>Terrabacteria_group>Firmicutes>Bacilli>Bacillales	unclassified_Exiguobacterium	MULTISPECIES:-DNA-sulfur-modification-protein-DndB-[unclassified-Exiguobacterium]	2644629
WP_214817547.1	DndB-ParB-nuclease	GCF_018617095.1	366	Bacteria>Terrabacteria_group>Firmicutes>Bacilli>Bacillales	unclassified_Exiguobacterium	unnamed-protein-product]	2644629
CDQ34590.1	DndB-ParB-nuclease	GCA_000723865.1	734	Bacteria>Terrabacteria_group>Firmicutes>Bacilli>Bacillales	Virgibacillus_halodenitrificans	DGQHR-domain-protein-[Virgibacillus-halodenitrificans]	1482
CDQ34594.1	DndB-ParB-nuclease	GCA_000723865.1	368	Bacteria>Terrabacteria_group>Firmicutes>Bacilli>Bacillales	Virgibacillus_halodenitrificans	DNA-sulfur-modification-protein-DndB-[Virgibacillus-halodenitrificans]	1482
MBQ4633983.1	DndB-ParB-nuclease	GCA_017415285.1	350	Bacteria>Terrabacteria_group>Firmicutes>Bacilli>unclassified_Bacilli	Bacilli_bacterium	DNA-sulfur-modification-protein-DndB-[Bacilli-bacterium]	1903720
WP_010249537.1	DndB-ParB-nuclease	GCF_000179595.2	358	Bacteria>Terrabacteria_group>Firmicutes>Clostridia>Eubacteriales	Acetivibrio_cellulolyticus	DNA-sulfur-modification-protein-DndB-[Acetivibrio-cellulolyticus]	35830
WP_066540123.1	DndB-ParB-nuclease	GCF_002201475.1	347	Bacteria>Terrabacteria_group>Firmicutes>Clostridia>Eubacteriales	Acutalibacter_muris	DNA-sulfur-modification-protein-DndB-[Acutalibacter-muris]	1796620
WP_114299108.1	DndB-ParB-nuclease	-	368	Bacteria>Terrabacteria_group>Firmicutes>Clostridia>Eubacteriales	Anaerobacterium_chartisolvens	DNA-sulfur-modification-protein-DndB-[Anaerobacterium-chartisolvens]	1297424
WP_073590724.1	DndB-ParB-nuclease	GCF_900143645.1	355	Bacteria>Terrabacteria_group>Firmicutes>Clostridia>Eubacteriales	Anaerocolumna_xylanovorans	DNA-sulfur-modification-protein-DndB-[Anaerocolumna-xylanovorans]	100134
WP_087241283.1	DndB-ParB-nuclease	GCF_002160025.1	344	Bacteria>Terrabacteria_group>Firmicutes>Clostridia>Eubacteriales	Anaeromassilibacillus_sp._An200	DNA-sulfur-modification-protein-DndB-[Anaeromassilibacillus-sp.-An200]	1965587
WP_095130879.1	DndB-ParB-nuclease	GCF_002270055.1	363	Bacteria>Terrabacteria_group>Firmicutes>Clostridia>Eubacteriales	Anaeromicrobium_sediminis	DNA-sulfur-modification-protein-DndB-[Anaeromicrobium-sediminis]	1478221
MBM6829705.1	DndB-ParB-nuclease	GCA_016902535.1	347	Bacteria>Terrabacteria_group>Firmicutes>Clostridia>Eubacteriales	Anaerotignum_lactatifermentans	DNA-sulfur-modification-protein-DndB-[Anaerotignum-lactatifermentans]	160404
WP_205133862.1	DndB-ParB-nuclease	GCF_016901915.1	347	Bacteria>Terrabacteria_group>Firmicutes>Clostridia>Eubacteriales	Anaerotignum_lactatifermentans	DNA-sulfur-modification-protein-DndB-[Anaerotignum-lactatifermentans]	160404
WP_087216845.1	DndB-ParB-nuclease	GCF_002159815.1	344	Bacteria>Terrabacteria_group>Firmicutes>Clostridia>Eubacteriales	Anaerotruncus_colihominis	DNA-sulfur-modification-protein-DndB-[Anaerotruncus-colihominis]	169435
WP_101552890.1	DndB-ParB-nuclease	GCF_900199635.1	355	Bacteria>Terrabacteria_group>Firmicutes>Clostridia>Eubacteriales	Anaerotruncus_massiliensis	DNA-sulfur-modification-protein-DndB-[Anaerotruncus-massiliensis]	1673720
WP_089282265.1	DndB-ParB-nuclease	GCF_900188145.1	363	Bacteria>Terrabacteria_group>Firmicutes>Clostridia>Eubacteriales	Anaerovirgula_multivorans	DNA-sulfur-modification-protein-DndB-[Anaerovirgula-multivorans]	312168
MBR2258732.1	DndB-ParB-nuclease	GCA_017475035.1	357	Bacteria>Terrabacteria_group>Firmicutes>Clostridia>Eubacteriales	Blautia_sp.	DNA-sulfur-modification-protein-DndB-[Blautia-sp.]	1955243
WP_035792360.1	DndB-ParB-nuclease	GCF_000424165.1	353	Bacteria>Terrabacteria_group>Firmicutes>Clostridia>Eubacteriales	Butyrivibrio_sp._AE3006	DNA-sulfur-modification-protein-DndB-[Butyrivibrio-sp.-AE3006]	1280673
MBO6241880.1	DndB-ParB-nuclease	GCA_017635265.1	440	Bacteria>Terrabacteria_group>Firmicutes>Clostridia>Eubacteriales	Butyrivibrio_sp.	DGQHR-domain-containing-protein-[Butyrivibrio-sp.]	28121
MBE5838081.1	DndB-ParB-nuclease	GCA_015056685.1	352	Bacteria>Terrabacteria_group>Firmicutes>Clostridia>Eubacteriales	Butyrivibrio_sp.	DNA-sulfur-modification-protein-DndB-[Butyrivibrio-sp.]	28121
MBP3195814.1	DndB-ParB-nuclease	GCA_017934005.1	354	Bacteria>Terrabacteria_group>Firmicutes>Clostridia>Eubacteriales	Butyrivibrio_sp.	DNA-sulfur-modification-protein-DndB-[Butyrivibrio-sp.]	28121
MBP3818097.1	DndB-ParB-nuclease	GCA_017940925.1	357	Bacteria>Terrabacteria_group>Firmicutes>Clostridia>Eubacteriales	Butyrivibrio_sp.	DNA-sulfur-modification-protein-DndB-[Butyrivibrio-sp.]	28121
WP_103868248.1	DndB-ParB-nuclease	GCF_900108105.1	353	Bacteria>Terrabacteria_group>Firmicutes>Clostridia>Eubacteriales	Butyrivibrio_sp._Su6	DNA-sulfur-modification-protein-DndB-[Butyrivibrio-sp.-Su6]	1520810
HJA72176.1	DndB-ParB-nuclease	GCA_019118365.1	337	Bacteria>Terrabacteria_group>Firmicutes>Clostridia>Eubacteriales	Candidatus_Lachnoclostridium_stercoravium	DNA-sulfur-modification-protein-DndB-[Candidatus-Lachnoclostridium-stercoravium]	2838633
HJD05340.1	DndB-ParB-nuclease	GCA_019120295.1	350	Bacteria>Terrabacteria_group>Firmicutes>Clostridia>Eubacteriales	Candidatus_Onthoplasma_faecipullorum	DNA-sulfur-modification-protein-DndB-[Candidatus-Onthoplasma-faecipullorum]	2840882
MBU3840488.1	DndB-ParB-nuclease	GCA_018883985.1	355	Bacteria>Terrabacteria_group>Firmicutes>Clostridia>Eubacteriales	Candidatus_Ruminococcus_intestinipullorum	DNA-sulfur-modification-protein-DndB-[Candidatus-Ruminococcus-intestinipullorum]	2838750
WP_183776578.1	DndB-ParB-nuclease	GCF_014202115.1	356	Bacteria>Terrabacteria_group>Firmicutes>Clostridia>Eubacteriales	Catenibacillus_scindens	DNA-sulfur-modification-protein-DndB-[Catenibacillus-scindens]	673271
WP_023353465.1	DndB-ParB-nuclease	GCF_000160035.2	360	Bacteria>Terrabacteria_group>Firmicutes>Clostridia>Eubacteriales	Catonella_morbi	DNA-sulfur-modification-protein-DndB-[Catonella-morbi]	43997
MBE6023512.1	DndB-ParB-nuclease	GCA_015058045.1	367	Bacteria>Terrabacteria_group>Firmicutes>Clostridia>Eubacteriales	Cellulosilyticum_sp.	DNA-sulfur-modification-protein-DndB-[Cellulosilyticum-sp.]	1926876
WP_127065290.1	DndB-ParB-nuclease	GCF_003990395.1	367	Bacteria>Terrabacteria_group>Firmicutes>Clostridia>Eubacteriales	Cellulosilyticum_sp._WCF-2	DNA-sulfur-modification-protein-DndB-[Cellulosilyticum-sp.-WCF-2]	2497860
NLD50192.1	DndB-ParB-nuclease	GCA_012513545.1	119	Bacteria>Terrabacteria_group>Firmicutes>Clostridia>Eubacteriales	Clostridiaceae_bacterium	hypothetical-protein-[Clostridiaceae-bacterium]	1898204
NLD50193.1	DndB-ParB-nuclease	GCA_012513545.1	298	Bacteria>Terrabacteria_group>Firmicutes>Clostridia>Eubacteriales	Clostridiaceae_bacterium	hypothetical-protein-[Clostridiaceae-bacterium]	1898204
OJV65163.1	DndB-ParB-nuclease	GCA_001897605.1	360	Bacteria>Terrabacteria_group>Firmicutes>Clostridia>Eubacteriales	Clostridiales_bacterium_38-18	hypothetical-protein-BGO41_00965-[Clostridiales-bacterium-38-18]	1895746
OJU18687.1	DndB-ParB-nuclease	GCA_001896915.1	351	Bacteria>Terrabacteria_group>Firmicutes>Clostridia>Eubacteriales	Clostridiales_bacterium_43-6	hypothetical-protein-BGN88_09450-[Clostridiales-bacterium-43-6]	1895747
MBS6250205.1	DndB-ParB-nuclease	GCA_018374915.1	423	Bacteria>Terrabacteria_group>Firmicutes>Clostridia>Eubacteriales	Clostridiales_bacterium	DGQHR-domain-containing-protein-[Clostridiales-bacterium]	1898207
MCC8050821.1	DndB-ParB-nuclease	GCA_020859985.1	360	Bacteria>Terrabacteria_group>Firmicutes>Clostridia>Eubacteriales	Clostridiales_bacterium	DNA-sulfur-modification-protein-DndB-[Clostridiales-bacterium]	1898207
NLL31712.1	DndB-ParB-nuclease	GCA_012520205.1	361	Bacteria>Terrabacteria_group>Firmicutes>Clostridia>Eubacteriales	Clostridiales_bacterium	DNA-sulfur-modification-protein-DndB-[Clostridiales-bacterium]	1898207
QTE70305.1	DndB-ParB-nuclease	GCA_017569305.1	337	Bacteria>Terrabacteria_group>Firmicutes>Clostridia>Eubacteriales	Clostridiales_bacterium_FE2011	DNA-sulfur-modification-protein-DndB-[Clostridiales-bacterium-FE2011]	2806438
ERI94634.1	DndB-ParB-nuclease	GCA_000466585.1	362	Bacteria>Terrabacteria_group>Firmicutes>Clostridia>Eubacteriales	Clostridiales_bacterium_oral_taxon_876_str._F0540	DNA-sulfur-modification-protein-DndB-[Clostridiales-bacterium-oral-taxon-876-str.-F0540]	1321778
WP_211144277.1	DndB-ParB-nuclease	GCF_016316925.1	361	Bacteria>Terrabacteria_group>Firmicutes>Clostridia>Eubacteriales	Clostridium_aciditolerans	DNA-sulfur-modification-protein-DndB-[Clostridium-aciditolerans]	339861
WP_211144271.1	DndB-ParB-nuclease	GCF_016316925.1	335	Bacteria>Terrabacteria_group>Firmicutes>Clostridia>Eubacteriales	Clostridium_aciditolerans	hypothetical-protein-[Clostridium-aciditolerans]	339861
WP_224168025.1	DndB-ParB-nuclease	GCF_900461085.1	363	Bacteria>Terrabacteria_group>Firmicutes>Clostridia>Eubacteriales	Clostridium_baratii	DNA-sulfur-modification-protein-DndB-[Clostridium-baratii]	1561
NOW04432.1	DndB-ParB-nuclease	GCA_013149485.1	361	Bacteria>Terrabacteria_group>Firmicutes>Clostridia>Eubacteriales	Clostridium_beijerinckii	DNA-sulfur-modification-protein-DndB-[Clostridium-beijerinckii]	1520
NOW91086.1	DndB-ParB-nuclease	GCA_013149505.1	361	Bacteria>Terrabacteria_group>Firmicutes>Clostridia>Eubacteriales	Clostridium_beijerinckii	DNA-sulfur-modification-protein-DndB-[Clostridium-beijerinckii]	1520
WP_041896610.1	DndB-ParB-nuclease	GCF_000833105.2	356	Bacteria>Terrabacteria_group>Firmicutes>Clostridia>Eubacteriales	Clostridium_beijerinckii	DNA-sulfur-modification-protein-DndB-[Clostridium-beijerinckii]	1520
WP_173713794.1	DndB-ParB-nuclease	GCF_013294155.1	361	Bacteria>Terrabacteria_group>Firmicutes>Clostridia>Eubacteriales	Clostridium_beijerinckii	DNA-sulfur-modification-protein-DndB-[Clostridium-beijerinckii]	1520
NFL33965.1	DndB-ParB-nuclease	GCA_011017025.1	358	Bacteria>Terrabacteria_group>Firmicutes>Clostridia>Eubacteriales	Clostridium_botulinum	DGQHR-domain-containing-protein-[Clostridium-botulinum]	1491
AVP62358.1	DndB-ParB-nuclease	GCA_003013655.1	361	Bacteria>Terrabacteria_group>Firmicutes>Clostridia>Eubacteriales	Clostridium_botulinum	DNA-sulfur-modification-protein-DndB-[Clostridium-botulinum]	1491
AVP65977.1	DndB-ParB-nuclease	GCA_003013615.1	361	Bacteria>Terrabacteria_group>Firmicutes>Clostridia>Eubacteriales	Clostridium_botulinum	DNA-sulfur-modification-protein-DndB-[Clostridium-botulinum]	1491
NFK79230.1	DndB-ParB-nuclease	GCA_011015635.1	361	Bacteria>Terrabacteria_group>Firmicutes>Clostridia>Eubacteriales	Clostridium_botulinum	DNA-sulfur-modification-protein-DndB-[Clostridium-botulinum]	1491
NFL33960.1	DndB-ParB-nuclease	GCA_011017025.1	362	Bacteria>Terrabacteria_group>Firmicutes>Clostridia>Eubacteriales	Clostridium_botulinum	DNA-sulfur-modification-protein-DndB-[Clostridium-botulinum]	1491
NFN99388.1	DndB-ParB-nuclease	GCA_011016295.1	362	Bacteria>Terrabacteria_group>Firmicutes>Clostridia>Eubacteriales	Clostridium_botulinum	DNA-sulfur-modification-protein-DndB-[Clostridium-botulinum]	1491
NFO14264.1	DndB-ParB-nuclease	GCA_011016315.1	362	Bacteria>Terrabacteria_group>Firmicutes>Clostridia>Eubacteriales	Clostridium_botulinum	DNA-sulfur-modification-protein-DndB-[Clostridium-botulinum]	1491
NFT93576.1	DndB-ParB-nuclease	GCA_011021465.1	362	Bacteria>Terrabacteria_group>Firmicutes>Clostridia>Eubacteriales	Clostridium_botulinum	DNA-sulfur-modification-protein-DndB-[Clostridium-botulinum]	1491
WP_013726257.1	DndB-ParB-nuclease	GCF_000204565.1	361	Bacteria>Terrabacteria_group>Firmicutes>Clostridia>Eubacteriales	Clostridium_botulinum	DNA-sulfur-modification-protein-DndB-[Clostridium-botulinum]	1491
WP_076175539.1	DndB-ParB-nuclease	GCF_000711055.1	361	Bacteria>Terrabacteria_group>Firmicutes>Clostridia>Eubacteriales	Clostridium_botulinum	DNA-sulfur-modification-protein-DndB-[Clostridium-botulinum]	1491
WP_148347239.1	DndB-ParB-nuclease	GCF_902362255.1	361	Bacteria>Terrabacteria_group>Firmicutes>Clostridia>Eubacteriales	Clostridium_butyricum	DNA-sulfur-modification-protein-DndB-[Clostridium-butyricum]	1492
ETI90590.1	DndB-ParB-nuclease	GCA_000508465.1	361	Bacteria>Terrabacteria_group>Firmicutes>Clostridia>Eubacteriales	Clostridium_butyricum_DORA_1	DNA-sulfur-modification-protein-DndB-[Clostridium-butyricum-DORA_1]	1403941
WP_099335651.1	DndB-ParB-nuclease	GCF_902386485.1	361	Bacteria>Terrabacteria_group>Firmicutes>Clostridia>Eubacteriales	Clostridium_cadaveris	DNA-sulfur-modification-protein-DndB-[Clostridium-cadaveris]	1529
WP_105176696.1	DndB-ParB-nuclease	GCF_900290265.1	362	Bacteria>Terrabacteria_group>Firmicutes>Clostridia>Eubacteriales	Clostridium_cagae	DNA-sulfur-modification-protein-DndB-[Clostridium-cagae]	2080751
WP_160359686.1	DndB-ParB-nuclease	GCF_009767885.1	361	Bacteria>Terrabacteria_group>Firmicutes>Clostridia>Eubacteriales	Clostridium_chromiireducens	DNA-sulfur-modification-protein-DndB-[Clostridium-chromiireducens]	225345
ENY94438.1	DndB-ParB-nuclease	GCA_000371405.1	344	Bacteria>Terrabacteria_group>Firmicutes>Clostridia>Eubacteriales	[Clostridium]_clostridioforme_CM201	DNA-sulfur-modification-protein-DndB-[[Clostridium]	999410
WP_012449433.1	DndB-ParB-nuclease	GCF_000020285.1	362	Bacteria>Terrabacteria_group>Firmicutes>Clostridia>Eubacteriales	Clostridium	DNA-sulfur-modification-protein-DndB-[Clostridium-botulinum]	1485
WP_170841297.1	DndB-ParB-nuclease	GCF_900111235.1	428	Bacteria>Terrabacteria_group>Firmicutes>Clostridia>Eubacteriales	[Clostridium]_fimetarium	DGQHR-domain-containing-protein-[[Clostridium]	99656
WP_013238123.1	DndB-ParB-nuclease	GCF_000143685.1	361	Bacteria>Terrabacteria_group>Firmicutes>Clostridia>Eubacteriales	Clostridium_ljungdahlii	DNA-sulfur-modification-protein-DndB-[Clostridium-ljungdahlii]	1538
WP_013238117.1	DndB-ParB-nuclease	GCF_000143685.1	339	Bacteria>Terrabacteria_group>Firmicutes>Clostridia>Eubacteriales	Clostridium_ljungdahlii	hypothetical-protein-[Clostridium-ljungdahlii]	1538
WP_039284243.1	DndB-ParB-nuclease	GCF_902386705.1	361	Bacteria>Terrabacteria_group>Firmicutes>Clostridia>Eubacteriales	Clostridium	MULTISPECIES:-DNA-sulfur-modification-protein-DndB-[Clostridium]	1485
WP_055291299.1	DndB-ParB-nuclease	GCF_001405575.1	359	Bacteria>Terrabacteria_group>Firmicutes>Clostridia>Eubacteriales	Clostridium	MULTISPECIES:-DNA-sulfur-modification-protein-DndB-[Clostridium]	1485
WP_058295424.1	DndB-ParB-nuclease	GCF_001458595.1	365	Bacteria>Terrabacteria_group>Firmicutes>Clostridia>Eubacteriales	Clostridium_neonatale	DNA-sulfur-modification-protein-DndB-[Clostridium-neonatale]	137838
STB12651.1	DndB-ParB-nuclease	GCA_900461285.1	333	Bacteria>Terrabacteria_group>Firmicutes>Clostridia>Eubacteriales	Clostridium_novyi	DGQHR-domain-[Clostridium-novyi]	1542
STB12637.1	DndB-ParB-nuclease	GCA_900461285.1	366	Bacteria>Terrabacteria_group>Firmicutes>Clostridia>Eubacteriales	Clostridium_novyi	DNA-sulfur-modification-protein-DndB-[Clostridium-novyi]	1542
WP_039252020.1	DndB-ParB-nuclease	GCF_000768605.1	361	Bacteria>Terrabacteria_group>Firmicutes>Clostridia>Eubacteriales	Clostridium_novyi	DNA-sulfur-modification-protein-DndB-[Clostridium-novyi]	1542
WP_099314200.1	DndB-ParB-nuclease	GCF_900217205.1	363	Bacteria>Terrabacteria_group>Firmicutes>Clostridia>Eubacteriales	Clostridium_paraputrificum	DNA-sulfur-modification-protein-DndB-[Clostridium-paraputrificum]	29363
EHK2406049.1	DndB-ParB-nuclease	GCA_018012095.1	335	Bacteria>Terrabacteria_group>Firmicutes>Clostridia>Eubacteriales	Clostridium_perfringens	DGQHR-domain-containing-protein-[Clostridium-perfringens]	1502
WP_164807203.1	DndB-ParB-nuclease	GCF_011062885.1	333	Bacteria>Terrabacteria_group>Firmicutes>Clostridia>Eubacteriales	Clostridium_perfringens	DGQHR-domain-containing-protein-[Clostridium-perfringens]	1502
WP_164817002.1	DndB-ParB-nuclease	GCF_011062525.1	328	Bacteria>Terrabacteria_group>Firmicutes>Clostridia>Eubacteriales	Clostridium_perfringens	DGQHR-domain-containing-protein-[Clostridium-perfringens]	1502
EHA1007701.1	DndB-ParB-nuclease	GCA_016610805.1	366	Bacteria>Terrabacteria_group>Firmicutes>Clostridia>Eubacteriales	Clostridium_perfringens	DNA-sulfur-modification-protein-DndB-[Clostridium-perfringens]	1502
EHK2327209.1	DndB-ParB-nuclease	GCA_018011795.1	366	Bacteria>Terrabacteria_group>Firmicutes>Clostridia>Eubacteriales	Clostridium_perfringens	DNA-sulfur-modification-protein-DndB-[Clostridium-perfringens]	1502
EHK2348190.1	DndB-ParB-nuclease	GCA_018011935.1	366	Bacteria>Terrabacteria_group>Firmicutes>Clostridia>Eubacteriales	Clostridium_perfringens	DNA-sulfur-modification-protein-DndB-[Clostridium-perfringens]	1502
EHK2406055.1	DndB-ParB-nuclease	GCA_018012095.1	366	Bacteria>Terrabacteria_group>Firmicutes>Clostridia>Eubacteriales	Clostridium_perfringens	DNA-sulfur-modification-protein-DndB-[Clostridium-perfringens]	1502
EHR0218645.1	DndB-ParB-nuclease	GCA_018992235.1	366	Bacteria>Terrabacteria_group>Firmicutes>Clostridia>Eubacteriales	Clostridium_perfringens	DNA-sulfur-modification-protein-DndB-[Clostridium-perfringens]	1502
HAT4209679.1	DndB-ParB-nuclease	GCA_015931985.1	366	Bacteria>Terrabacteria_group>Firmicutes>Clostridia>Eubacteriales	Clostridium_perfringens	DNA-sulfur-modification-protein-DndB-[Clostridium-perfringens]	1502
WP_003471754.1	DndB-ParB-nuclease	GCF_000171215.1	366	Bacteria>Terrabacteria_group>Firmicutes>Clostridia>Eubacteriales	Clostridium_perfringens	DNA-sulfur-modification-protein-DndB-[Clostridium-perfringens]	1502
WP_061428133.1	DndB-ParB-nuclease	GCF_902459515.1	366	Bacteria>Terrabacteria_group>Firmicutes>Clostridia>Eubacteriales	Clostridium_perfringens	DNA-sulfur-modification-protein-DndB-[Clostridium-perfringens]	1502
WP_070956854.1	DndB-ParB-nuclease	GCF_001854085.1	366	Bacteria>Terrabacteria_group>Firmicutes>Clostridia>Eubacteriales	Clostridium_perfringens	DNA-sulfur-modification-protein-DndB-[Clostridium-perfringens]	1502
WP_150540972.1	DndB-ParB-nuclease	GCF_902459455.1	366	Bacteria>Terrabacteria_group>Firmicutes>Clostridia>Eubacteriales	Clostridium_perfringens	DNA-sulfur-modification-protein-DndB-[Clostridium-perfringens]	1502
WP_164807208.1	DndB-ParB-nuclease	GCF_020138715.1	366	Bacteria>Terrabacteria_group>Firmicutes>Clostridia>Eubacteriales	Clostridium_perfringens	DNA-sulfur-modification-protein-DndB-[Clostridium-perfringens]	1502
WP_221365360.1	DndB-ParB-nuclease	GCF_019711035.1	366	Bacteria>Terrabacteria_group>Firmicutes>Clostridia>Eubacteriales	Clostridium_perfringens	DNA-sulfur-modification-protein-DndB-[Clostridium-perfringens]	1502
WP_061428147.1	DndB-ParB-nuclease	GCF_001579785.1	331	Bacteria>Terrabacteria_group>Firmicutes>Clostridia>Eubacteriales	Clostridium_perfringens	hypothetical-protein-[Clostridium-perfringens]	1502
WP_224397281.1	DndB-ParB-nuclease	GCF_020138655.1	335	Bacteria>Terrabacteria_group>Firmicutes>Clostridia>Eubacteriales	Clostridium_perfringens	hypothetical-protein-[Clostridium-perfringens]	1502
HAT4133841.1	DndB-ParB-nuclease	GCA_015931425.1	366	Bacteria>Terrabacteria_group>Firmicutes>Clostridia>Eubacteriales	Clostridium_perfringens	TPA:-DNA-sulfur-modification-protein-DndB-[Clostridium-perfringens]	1502
HAT4149723.1	DndB-ParB-nuclease	GCA_015931485.1	366	Bacteria>Terrabacteria_group>Firmicutes>Clostridia>Eubacteriales	Clostridium_perfringens	TPA:-DNA-sulfur-modification-protein-DndB-[Clostridium-perfringens]	1502
WP_115641085.1	DndB-ParB-nuclease	GCF_900461105.1	361	Bacteria>Terrabacteria_group>Firmicutes>Clostridia>Eubacteriales	Clostridium_putrefaciens	DNA-sulfur-modification-protein-DndB-[Clostridium-putrefaciens]	99675
MBE6055190.1	DndB-ParB-nuclease	GCA_015058425.1	363	Bacteria>Terrabacteria_group>Firmicutes>Clostridia>Eubacteriales	Clostridium_sartagoforme	DNA-sulfur-modification-protein-DndB-[Clostridium-sartagoforme]	84031
WP_181983916.1	DndB-ParB-nuclease	GCF_003461085.1	429	Bacteria>Terrabacteria_group>Firmicutes>Clostridia>Eubacteriales	Clostridium_sp._AF23-6LB	DGQHR-domain-containing-protein-[Clostridium-sp.-AF23-6LB]	2293005
WP_117464666.1	DndB-ParB-nuclease	GCF_003435855.1	340	Bacteria>Terrabacteria_group>Firmicutes>Clostridia>Eubacteriales	Clostridium_sp._AF46-12NS	DNA-sulfur-modification-protein-DndB-[Clostridium-sp.-AF46-12NS]	2293019
WP_118246196.1	DndB-ParB-nuclease	GCF_003464085.1	423	Bacteria>Terrabacteria_group>Firmicutes>Clostridia>Eubacteriales	Clostridium_sp._AM58-1XD	DGQHR-domain-containing-protein-[Clostridium-sp.-AM58-1XD]	2292307

Accession	Annotation	GCA	Length	Phyletic.spread	Strain.name	Defline	Species.Taxid
WP_176255645.1	DndB-ParB-nuclease	GCF_013282095.1	357	Bacteria>Terrabacteria_group>Firmicutes>Clostridia>Eubacteriales	Clostridium_sp._C5-48	DNA-sulfur-modification-protein-DndB-[Clostridium-sp.-C5-48]	2547410
CCZ05511.1	DndB-ParB-nuclease	GCA_000432475.1	357	Bacteria>Terrabacteria_group>Firmicutes>Clostridia>Eubacteriales	Clostridium_sp._CAG:127	dNA-sulfur-modification-protein-DndB-[Clostridium-sp.-CAG:127]	1262774
MBE6047489.1	DndB-ParB-nuclease	GCA_015058375.1	362	Bacteria>Terrabacteria_group>Firmicutes>Clostridia>Eubacteriales	Clostridium_sp.	DNA-sulfur-modification-protein-DndB-[Clostridium-sp.]	1506
MBE6050179.1	DndB-ParB-nuclease	GCA_015058405.1	361	Bacteria>Terrabacteria_group>Firmicutes>Clostridia>Eubacteriales	Clostridium_sp.	DNA-sulfur-modification-protein-DndB-[Clostridium-sp.]	1506
MBS5125668.1	DndB-ParB-nuclease	GCA_018365735.1	362	Bacteria>Terrabacteria_group>Firmicutes>Clostridia>Eubacteriales	Clostridium_sp.	DNA-sulfur-modification-protein-DndB-[Clostridium-sp.]	1506
MBS6765673.1	DndB-ParB-nuclease	GCA_018379185.1	352	Bacteria>Terrabacteria_group>Firmicutes>Clostridia>Eubacteriales	Clostridium_sp.	DNA-sulfur-modification-protein-DndB-[Clostridium-sp.]	1506
WP_077613031.1	DndB-ParB-nuclease	GCF_900155545.1	357	Bacteria>Terrabacteria_group>Firmicutes>Clostridia>Eubacteriales	Clostridium_sp._Marseille-P2415	DNA-sulfur-modification-protein-DndB-[Clostridium-sp.-Marseille-P2415]	1805471
WP_215700284.1	DndB-ParB-nuclease	GCF_018785315.1	352	Bacteria>Terrabacteria_group>Firmicutes>Clostridia>Eubacteriales	Clostridium_sp._MCC353	DNA-sulfur-modification-protein-DndB-[Clostridium-sp.-MCC353]	2592646
WP_074366248.1	DndB-ParB-nuclease	GCF_900095445.1	357	Bacteria>Terrabacteria_group>Firmicutes>Clostridia>Eubacteriales	Clostridium_sp._N3C	DNA-sulfur-modification-protein-DndB-[Clostridium-sp.-N3C]	1776758
WP_186860393.1	DndB-ParB-nuclease	GCF_014287825.1	359	Bacteria>Terrabacteria_group>Firmicutes>Clostridia>Eubacteriales	Clostridium_sp._NSJ-6	DNA-sulfur-modification-protein-DndB-[Clostridium-sp.-NSJ-6]	2763036
WP_058007719.1	DndB-ParB-nuclease	GCF_001444635.1	361	Bacteria>Terrabacteria_group>Firmicutes>Clostridia>Eubacteriales	Clostridium_sporogenes	DNA-sulfur-modification-protein-DndB-[Clostridium-sporogenes]	1509
WP_058012577.1	DndB-ParB-nuclease	GCF_019933215.1	361	Bacteria>Terrabacteria_group>Firmicutes>Clostridia>Eubacteriales	Clostridium_sporogenes	DNA-sulfur-modification-protein-DndB-[Clostridium-sporogenes]	1509
WP_115652186.1	DndB-ParB-nuclease	GCF_011020555.1	361	Bacteria>Terrabacteria_group>Firmicutes>Clostridia>Eubacteriales	Clostridium_sporogenes	DNA-sulfur-modification-protein-DndB-[Clostridium-sporogenes]	1509
WP_163224229.1	DndB-ParB-nuclease	GCF_011019675.1	361	Bacteria>Terrabacteria_group>Firmicutes>Clostridia>Eubacteriales	Clostridium_sporogenes	DNA-sulfur-modification-protein-DndB-[Clostridium-sporogenes]	1509
WP_195344421.1	DndB-ParB-nuclease	GCF_015550435.1	423	Bacteria>Terrabacteria_group>Firmicutes>Clostridia>Eubacteriales	[Clostridium]_symbiosum	DGQHR-domain-containing-protein-[[Clostridium]	1512
AOR23761.1	DndB-ParB-nuclease	GCA_001735765.2	362	Bacteria>Terrabacteria_group>Firmicutes>Clostridia>Eubacteriales	Clostridium_taeniosporum	DNA-sulfur-modification-protein-DndB-[Clostridium-taeniosporum]	394958
WP_069679909.1	DndB-ParB-nuclease	GCF_001735765.2	362	Bacteria>Terrabacteria_group>Firmicutes>Clostridia>Eubacteriales	Clostridium_taeniosporum	DNA-sulfur-modification-protein-DndB-[Clostridium-taeniosporum]	394958
WP_129032068.1	DndB-ParB-nuclease	GCF_004115485.1	361	Bacteria>Terrabacteria_group>Firmicutes>Clostridia>Eubacteriales	Clostridium_tetani	DNA-sulfur-modification-protein-DndB-[Clostridium-tetani]	1513
WP_071143938.1	DndB-ParB-nuclease	GCF_900104635.1	358	Bacteria>Terrabacteria_group>Firmicutes>Clostridia>Eubacteriales	Coprococcus_phoceensis	MULTISPECIES:-DNA-sulfur-modification-protein-DndB-[Lachnospiraceae]	1870993
WP_204613498.1	DndB-ParB-nuclease	GCF_016908775.1	372	Bacteria>Terrabacteria_group>Firmicutes>Clostridia>Eubacteriales	Defluviitalea_raffinosedens	DNA-sulfur-modification-protein-DndB-[Defluviitalea-raffinosedens]	1450156
WP_195710847.1	DndB-ParB-nuclease	GCF_015594625.1	435	Bacteria>Terrabacteria_group>Firmicutes>Clostridia>Eubacteriales	Desulfallas_sp._Bu1-1	DGQHR-domain-containing-protein-[Desulfallas-sp.-Bu1-1]	2787620
WP_006717311.1	DndB-ParB-nuclease	GCF_000231405.2	351	Bacteria>Terrabacteria_group>Firmicutes>Clostridia>Eubacteriales	Desulfitobacterium_metallireducens	DNA-sulfur-modification-protein-DndB-[Desulfitobacterium-metallireducens]	142877
MCB8814718.1	DndB-ParB-nuclease	GCA_020595055.1	344	Bacteria>Terrabacteria_group>Firmicutes>Clostridia>Eubacteriales	Desulfosporosinus_sp._SRJS8	DNA-sulfur-modification-protein-DndB-[Desulfosporosinus-sp.-SRJS8]	2885154
WP_229246336.1	DndB-ParB-nuclease	GCF_020595055.1	344	Bacteria>Terrabacteria_group>Firmicutes>Clostridia>Eubacteriales	Desulfosporosinus_sp._SRJS8	DNA-sulfur-modification-protein-DndB-[Desulfosporosinus-sp.-SRJS8]	2885154
WP_028087363.1	DndB-ParB-nuclease	GCF_000424345.1	431	Bacteria>Terrabacteria_group>Firmicutes>Clostridia>Eubacteriales	Dorea_longicatena	DGQHR-domain-containing-protein-[Dorea-longicatena]	88431
WP_213039797.1	DndB-ParB-nuclease	-	344	Bacteria>Terrabacteria_group>Firmicutes>Clostridia>Eubacteriales	Dysosmobacter_sp._Marseille-Q4140	DNA-sulfur-modification-protein-DndB-[Dysosmobacter-sp.-Marseille-Q4140]	2830675
WP_083790261.1	DndB-ParB-nuclease	GCF_025149125.1	368	Bacteria>Terrabacteria_group>Firmicutes>Clostridia>Eubacteriales	Enterocloster_asparagiformis	DNA-sulfur-modification-protein-DndB-[Enterocloster-asparagiformis]	333367
WP_196024354.1	DndB-ParB-nuclease	GCF_015669475.1	424	Bacteria>Terrabacteria_group>Firmicutes>Clostridia>Eubacteriales	Enterocloster_clostridioformis	DGQHR-domain-containing-protein-[Enterocloster-clostridioformis]	1531
WP_002596280.1	DndB-ParB-nuclease	GCF_000371405.1	344	Bacteria>Terrabacteria_group>Firmicutes>Clostridia>Eubacteriales	Enterocloster_clostridioformis	DNA-sulfur-modification-protein-DndB-[Enterocloster-clostridioformis]	1531
ESE31406.1	DndB-ParB-nuclease	GCA_000488855.1	358	Bacteria>Terrabacteria_group>Firmicutes>Clostridia>Eubacteriales	Eubacterium_brachy_ATCC_33089	DNA-sulfur-modification-protein-DndB-[Eubacterium-brachy-ATCC-33089]	1321814
WP_208066139.1	DndB-ParB-nuclease	GCF_017566045.1	356	Bacteria>Terrabacteria_group>Firmicutes>Clostridia>Eubacteriales	Eubacterium_callanderi	DNA-sulfur-modification-protein-DndB-[Eubacterium-callanderi]	53442
WP_118518391.1	DndB-ParB-nuclease	GCF_902364445.2	356	Bacteria>Terrabacteria_group>Firmicutes>Clostridia>Eubacteriales	Eubacterium	MULTISPECIES:-DNA-sulfur-modification-protein-DndB-[Eubacterium]	1730
WP_004067182.1	DndB-ParB-nuclease	-	356	Bacteria>Terrabacteria_group>Firmicutes>Clostridia>Eubacteriales	Eubacterium_plexicaudatum	DNA-sulfur-modification-protein-DndB-[Eubacterium-plexicaudatum]	97253
WP_021739891.1	DndB-ParB-nuclease	GCF_902375155.1	356	Bacteria>Terrabacteria_group>Firmicutes>Clostridia>Eubacteriales	Eubacterium_ramulus	DNA-sulfur-modification-protein-DndB-[Eubacterium-ramulus]	39490
MBS6835846.1	DndB-ParB-nuclease	GCA_018379775.1	353	Bacteria>Terrabacteria_group>Firmicutes>Clostridia>Eubacteriales	[Eubacterium]_rectale	DNA-sulfur-modification-protein-DndB-[[Eubacterium]	39491
WP_072902611.1	DndB-ParB-nuclease	GCF_900142225.1	358	Bacteria>Terrabacteria_group>Firmicutes>Clostridia>Eubacteriales	Hathewaya_proteolytica	DNA-sulfur-modification-protein-DndB-[Hathewaya-proteolytica]	29365
WP_161260519.1	DndB-ParB-nuclease	GCF_016908025.1	366	Bacteria>Terrabacteria_group>Firmicutes>Clostridia>Eubacteriales	Heliomicrobium_gestii	DNA-sulfur-modification-protein-DndB-[Heliomicrobium-gestii]	2699
WP_154575157.1	DndB-ParB-nuclease	GCF_009695915.1	356	Bacteria>Terrabacteria_group>Firmicutes>Clostridia>Eubacteriales	Hornefia_butyriciproducens	DNA-sulfur-modification-protein-DndB-[Hornefia-butyriciproducens]	2652293
WP_195608301.1	DndB-ParB-nuclease	GCF_015560295.1	355	Bacteria>Terrabacteria_group>Firmicutes>Clostridia>Eubacteriales	Intestinimonas_butyriciproducens	DNA-sulfur-modification-protein-DndB-[Intestinimonas-butyriciproducens]	1297617
WP_204751846.1	DndB-ParB-nuclease	GCF_016901775.1	344	Bacteria>Terrabacteria_group>Firmicutes>Clostridia>Eubacteriales	Intestinimonas_butyriciproducens	DNA-sulfur-modification-protein-DndB-[Intestinimonas-butyriciproducens]	1297617
EGC74038.1	DndB-ParB-nuclease	GCA_000191805.1	357	Bacteria>Terrabacteria_group>Firmicutes>Clostridia>Eubacteriales	Lachnospiraceae_bacterium_6_1_37FAA	DNA-sulfur-modification-protein-DndB-[Lachnospiraceae-bacterium-6_1_37FAA]	658656
EGC75132.1	DndB-ParB-nuclease	GCA_000191805.1	355	Bacteria>Terrabacteria_group>Firmicutes>Clostridia>Eubacteriales	Lachnospiraceae_bacterium_6_1_37FAA	DNA-sulfur-modification-protein-DndB-[Lachnospiraceae-bacterium-6_1_37FAA]	658656
MBD5476835.1	DndB-ParB-nuclease	GCA_014804365.1	372	Bacteria>Terrabacteria_group>Firmicutes>Clostridia>Eubacteriales	Lachnospiraceae_bacterium	DGQHR-domain-containing-protein-[Lachnospiraceae-bacterium]	1898203
MBP3506895.1	DndB-ParB-nuclease	GCA_017937345.1	397	Bacteria>Terrabacteria_group>Firmicutes>Clostridia>Eubacteriales	Lachnospiraceae_bacterium	DGQHR-domain-containing-protein-[Lachnospiraceae-bacterium]	1898203
MBS1471356.1	DndB-ParB-nuclease	GCA_018264955.1	277	Bacteria>Terrabacteria_group>Firmicutes>Clostridia>Eubacteriales	Lachnospiraceae_bacterium	DGQHR-domain-containing-protein-[Lachnospiraceae-bacterium]	1898203
HCJ08604.1	DndB-ParB-nuclease	GCA_003530125.1	354	Bacteria>Terrabacteria_group>Firmicutes>Clostridia>Eubacteriales	Lachnospiraceae_bacterium	DNA-sulfur-modification-protein-DndB-[Lachnospiraceae-bacterium]	1898203
MBD5471459.1	DndB-ParB-nuclease	GCA_014804285.1	354	Bacteria>Terrabacteria_group>Firmicutes>Clostridia>Eubacteriales	Lachnospiraceae_bacterium	DNA-sulfur-modification-protein-DndB-[Lachnospiraceae-bacterium]	1898203
MBD5530081.1	DndB-ParB-nuclease	GCA_014804645.1	355	Bacteria>Terrabacteria_group>Firmicutes>Clostridia>Eubacteriales	Lachnospiraceae_bacterium	DNA-sulfur-modification-protein-DndB-[Lachnospiraceae-bacterium]	1898203
MBE5850723.1	DndB-ParB-nuclease	GCA_015056775.1	340	Bacteria>Terrabacteria_group>Firmicutes>Clostridia>Eubacteriales	Lachnospiraceae_bacterium	DNA-sulfur-modification-protein-DndB-[Lachnospiraceae-bacterium]	1898203
MBE5937207.1	DndB-ParB-nuclease	GCA_015057405.1	349	Bacteria>Terrabacteria_group>Firmicutes>Clostridia>Eubacteriales	Lachnospiraceae_bacterium	DNA-sulfur-modification-protein-DndB-[Lachnospiraceae-bacterium]	1898203
MBE5958721.1	DndB-ParB-nuclease	GCA_015057625.1	357	Bacteria>Terrabacteria_group>Firmicutes>Clostridia>Eubacteriales	Lachnospiraceae_bacterium	DNA-sulfur-modification-protein-DndB-[Lachnospiraceae-bacterium]	1898203
MBP3505199.1	DndB-ParB-nuclease	GCA_017937345.1	357	Bacteria>Terrabacteria_group>Firmicutes>Clostridia>Eubacteriales	Lachnospiraceae_bacterium	DNA-sulfur-modification-protein-DndB-[Lachnospiraceae-bacterium]	1898203
MBQ3165687.1	DndB-ParB-nuclease	GCA_017397345.1	355	Bacteria>Terrabacteria_group>Firmicutes>Clostridia>Eubacteriales	Lachnospiraceae_bacterium	DNA-sulfur-modification-protein-DndB-[Lachnospiraceae-bacterium]	1898203
MBR2949459.1	DndB-ParB-nuclease	GCA_017500935.1	356	Bacteria>Terrabacteria_group>Firmicutes>Clostridia>Eubacteriales	Lachnospiraceae_bacterium	DNA-sulfur-modification-protein-DndB-[Lachnospiraceae-bacterium]	1898203
MBR6223841.1	DndB-ParB-nuclease	GCA_017540605.1	354	Bacteria>Terrabacteria_group>Firmicutes>Clostridia>Eubacteriales	Lachnospiraceae_bacterium	DNA-sulfur-modification-protein-DndB-[Lachnospiraceae-bacterium]	1898203
MBR6642129.1	DndB-ParB-nuclease	GCA_017520385.1	350	Bacteria>Terrabacteria_group>Firmicutes>Clostridia>Eubacteriales	Lachnospiraceae_bacterium	DNA-sulfur-modification-protein-DndB-[Lachnospiraceae-bacterium]	1898203
MBS6922319.1	DndB-ParB-nuclease	GCA_018380715.1	356	Bacteria>Terrabacteria_group>Firmicutes>Clostridia>Eubacteriales	Lachnospiraceae_bacterium	DNA-sulfur-modification-protein-DndB-[Lachnospiraceae-bacterium]	1898203
NBJ01284.1	DndB-ParB-nuclease	GCA_009911175.1	355	Bacteria>Terrabacteria_group>Firmicutes>Clostridia>Eubacteriales	Lachnospiraceae_bacterium	DNA-sulfur-modification-protein-DndB-[Lachnospiraceae-bacterium]	1898203
PWL93724.1	DndB-ParB-nuclease	GCA_003150445.1	356	Bacteria>Terrabacteria_group>Firmicutes>Clostridia>Eubacteriales	Lachnospiraceae_bacterium	DNA-sulfur-modification-protein-DndB-[Lachnospiraceae-bacterium]	1898203
GFI65390.1	DndB-ParB-nuclease	GCA_011958345.1	340	Bacteria>Terrabacteria_group>Firmicutes>Clostridia>Eubacteriales	Lachnospiraceae_bacterium	hypothetical-protein-IMSAG185_00989-[Lachnospiraceae-bacterium]	1898203
GFI65391.1	DndB-ParB-nuclease	GCA_011958345.1	430	Bacteria>Terrabacteria_group>Firmicutes>Clostridia>Eubacteriales	Lachnospiraceae_bacterium	hypothetical-protein-IMSAG185_00990-[Lachnospiraceae-bacterium]	1898203
GFI70617.1	DndB-ParB-nuclease	GCA_011958585.1	349	Bacteria>Terrabacteria_group>Firmicutes>Clostridia>Eubacteriales	Lachnospiraceae_bacterium	hypothetical-protein-IMSAG249_02446-[Lachnospiraceae-bacterium]	1898203
GFH97086.1	DndB-ParB-nuclease	GCA_011959105.1	358	Bacteria>Terrabacteria_group>Firmicutes>Clostridia>Eubacteriales	Lachnospiraceae_bacterium	hypothetical-protein-IMSAGC003_03655-[Lachnospiraceae-bacterium]	1898203
PTL28738.1	DndB-ParB-nuclease	GCA_003043955.1	340	Bacteria>Terrabacteria_group>Firmicutes>Clostridia>Eubacteriales	Lachnospiraceae_bacterium_oral_taxon_096	DNA-sulfur-modification-protein-DndB-[Lachnospiraceae-bacterium-oral-taxon-096]	712982
SFT61171.1	DndB-ParB-nuclease	GCA_900116395.1	356	Bacteria>Terrabacteria_group>Firmicutes>Clostridia>Eubacteriales	Lachnospiraceae_bacterium_XBD2001	DNA-sulfur-modification-protein-DndB-[Lachnospiraceae-bacterium-XBD2001]	1520820
OLA16401.1	DndB-ParB-nuclease	GCA_001916475.1	424	Bacteria>Terrabacteria_group>Firmicutes>Clostridia>Eubacteriales	Lachnospira_eligens	hypothetical-protein-BHW24_04580-[Lachnospira-eligens]	39485
WP_097006579.1	DndB-ParB-nuclease	GCF_900205965.1	356	Bacteria>Terrabacteria_group>Firmicutes>Clostridia>Eubacteriales	Lacrimispora_amygdalina	DNA-sulfur-modification-protein-DndB-[Lacrimispora-amygdalina]	253257
WP_100306406.1	DndB-ParB-nuclease	GCF_002797975.1	357	Bacteria>Terrabacteria_group>Firmicutes>Clostridia>Eubacteriales	Lacrimispora_celerecrescens	DNA-sulfur-modification-protein-DndB-[Lacrimispora-celerecrescens]	29354
WP_079492852.1	DndB-ParB-nuclease	GCF_900167445.1	357	Bacteria>Terrabacteria_group>Firmicutes>Clostridia>Eubacteriales	Maledivibacter_halophilus	DNA-sulfur-modification-protein-DndB-[Maledivibacter-halophilus]	36842
WP_215640143.1	DndB-ParB-nuclease	GCF_018783965.1	355	Bacteria>Terrabacteria_group>Firmicutes>Clostridia>Eubacteriales	Mediterraneibacter_faecis	DNA-sulfur-modification-protein-DndB-[Mediterraneibacter-faecis]	592978
MCF2569354.1	DndB-ParB-nuclease	GCA_021530965.1	355	Bacteria>Terrabacteria_group>Firmicutes>Clostridia>Eubacteriales	Mediterraneibacter_glycyrrhizinilyticus	DNA-sulfur-modification-protein-DndB-[Mediterraneibacter-glycyrrhizinilyticus]	342942
WP_054705690.1	DndB-ParB-nuclease	GCF_001311035.1	354	Bacteria>Terrabacteria_group>Firmicutes>Clostridia>Eubacteriales	Mediterraneibacter_glycyrrhizinilyticus	DNA-sulfur-modification-protein-DndB-[Mediterraneibacter-glycyrrhizinilyticus]	342942
WP_179012287.1	DndB-ParB-nuclease	GCF_013394775.1	359	Bacteria>Terrabacteria_group>Firmicutes>Clostridia>Eubacteriales	Oribacterium_sp._oral_taxon_102	DNA-sulfur-modification-protein-DndB-[Oribacterium-sp.-oral-taxon-102]	671214
MUU10526.1	DndB-ParB-nuclease	GCA_009739395.1	344	Bacteria>Terrabacteria_group>Firmicutes>Clostridia>Eubacteriales	Oscillibacter_sp.	DNA-sulfur-modification-protein-DndB-[Oscillibacter-sp.]	1945593
MBQ8012213.1	DndB-ParB-nuclease	GCA_017468645.1	356	Bacteria>Terrabacteria_group>Firmicutes>Clostridia>Eubacteriales	Oscillospiraceae_bacterium	DNA-sulfur-modification-protein-DndB-[Oscillospiraceae-bacterium]	2485925
MBQ9168106.1	DndB-ParB-nuclease	GCA_017463645.1	337	Bacteria>Terrabacteria_group>Firmicutes>Clostridia>Eubacteriales	Oscillospiraceae_bacterium	DNA-sulfur-modification-protein-DndB-[Oscillospiraceae-bacterium]	2485925
WP_084234534.1	DndB-ParB-nuclease	GCF_900176335.1	356	Bacteria>Terrabacteria_group>Firmicutes>Clostridia>Eubacteriales	Papillibacter_cinnamivorans	DNA-sulfur-modification-protein-DndB-[Papillibacter-cinnamivorans]	100176
KJS03668.1	DndB-ParB-nuclease	GCA_000961585.1	359	Bacteria>Terrabacteria_group>Firmicutes>Clostridia>Eubacteriales	Peptococcaceae_bacterium_BRH_c4a	hypothetical-protein-VR68_00745-[Peptococcaceae-bacterium-BRH_c4a]	1629716
WP_211802392.1	DndB-ParB-nuclease	GCF_018128025.1	357	Bacteria>Terrabacteria_group>Firmicutes>Clostridia>Eubacteriales	Proteiniclasticum_sediminis	DNA-sulfur-modification-protein-DndB-[Proteiniclasticum-sp.-BAD-10]	2804028
WP_193536501.1	DndB-ParB-nuclease	GCF_014982855.1	344	Bacteria>Terrabacteria_group>Firmicutes>Clostridia>Eubacteriales	Pseudoflavonifractor_gallinarum	DNA-sulfur-modification-protein-DndB-[Pseudoflavonifractor-gallinarum]	2779352
WP_161845264.1	DndB-ParB-nuclease	GCF_009935845.1	410	Bacteria>Terrabacteria_group>Firmicutes>Clostridia>Eubacteriales	Pseudoflavonifractor_sp._524-17	DGQHR-domain-containing-protein-[Pseudoflavonifractor-sp.-524-17]	2304577
WP_118642002.1	DndB-ParB-nuclease	GCF_003478995.1	355	Bacteria>Terrabacteria_group>Firmicutes>Clostridia>Eubacteriales	Pseudoflavonifractor_sp._AF19-9AC	DNA-sulfur-modification-protein-DndB-[Pseudoflavonifractor-sp.-AF19-9AC]	2292244
WP_215662667.1	DndB-ParB-nuclease	GCF_018784025.1	363	Bacteria>Terrabacteria_group>Firmicutes>Clostridia>Eubacteriales	Roseburia_hominis	DGQHR-domain-containing-protein-[Roseburia-hominis]	301301
WP_021922311.1	DndB-ParB-nuclease	GCF_001406855.1	363	Bacteria>Terrabacteria_group>Firmicutes>Clostridia>Eubacteriales	Roseburia_inulinivorans	DGQHR-domain-containing-protein-[Roseburia-inulinivorans]	360807
HCQ07193.1	DndB-ParB-nuclease	GCA_003525405.1	356	Bacteria>Terrabacteria_group>Firmicutes>Clostridia>Eubacteriales	Roseburia_sp.	DNA-sulfur-modification-protein-DndB-[Roseburia-sp.]	2049040
MBD5555486.1	DndB-ParB-nuclease	GCA_014804775.1	338	Bacteria>Terrabacteria_group>Firmicutes>Clostridia>Eubacteriales	Roseburia_sp.	DNA-sulfur-modification-protein-DndB-[Roseburia-sp.]	2049040
MBS5230489.1	DndB-ParB-nuclease	GCA_018366635.1	356	Bacteria>Terrabacteria_group>Firmicutes>Clostridia>Eubacteriales	Roseburia_sp.	DNA-sulfur-modification-protein-DndB-[Roseburia-sp.]	2049040
WP_186899190.1	DndB-ParB-nuclease	GCF_014287515.2	356	Bacteria>Terrabacteria_group>Firmicutes>Clostridia>Eubacteriales	Roseburia_sp._NSJ-69	DNA-sulfur-modification-protein-DndB-[Roseburia-sp.-NSJ-69]	2763062
RHT74337.1	DndB-ParB-nuclease	GCA_003480625.1	344	Bacteria>Terrabacteria_group>Firmicutes>Clostridia>Eubacteriales	Ruminococcaceae_bacterium_AM28-23LB	DNA-sulfur-modification-protein-DndB-[Ruminococcaceae-bacterium-AM28-23LB]	2292269
MBD8913299.1	DndB-ParB-nuclease	GCA_014848475.1	353	Bacteria>Terrabacteria_group>Firmicutes>Clostridia>Eubacteriales	Ruminococcus_bicirculans	DNA-sulfur-modification-protein-DndB-[Ruminococcus-bicirculans]	1160721
WP_109727346.1	DndB-ParB-nuclease	GCF_003149325.1	357	Bacteria>Terrabacteria_group>Firmicutes>Clostridia>Eubacteriales	Ruminococcus_flavefaciens	DNA-sulfur-modification-protein-DndB-[Ruminococcus-flavefaciens]	1265
WP_101878788.1	DndB-ParB-nuclease	GCF_002865425.1	358	Bacteria>Terrabacteria_group>Firmicutes>Clostridia>Eubacteriales	[Ruminococcus]_gnavus	DNA-sulfur-modification-protein-DndB-[[Ruminococcus]	33038
MBD9049686.1	DndB-ParB-nuclease	GCA_014848155.1	421	Bacteria>Terrabacteria_group>Firmicutes>Clostridia>Eubacteriales	Ruminococcus_sp.	DGQHR-domain-containing-protein-[Ruminococcus-sp.]	41978
MBQ6154126.1	DndB-ParB-nuclease	GCA_017431505.1	356	Bacteria>Terrabacteria_group>Firmicutes>Clostridia>Eubacteriales	Ruminococcus_sp.	DNA-sulfur-modification-protein-DndB-[Ruminococcus-sp.]	41978
WP_155236667.1	DndB-ParB-nuclease	GCF_009718155.1	356	Bacteria>Terrabacteria_group>Firmicutes>Clostridia>Eubacteriales	[Ruminococcus]_torques	DNA-sulfur-modification-protein-DndB-[[Ruminococcus]	33039
WP_058723689.1	DndB-ParB-nuclease	GCF_001481375.1	358	Bacteria>Terrabacteria_group>Firmicutes>Clostridia>Eubacteriales	Ruthenibacterium_lactatiformans	DNA-sulfur-modification-protein-DndB-[Ruthenibacterium-lactatiformans]	1550024
WP_125128691.1	DndB-ParB-nuclease	GCF_003885045.1	355	Bacteria>Terrabacteria_group>Firmicutes>Clostridia>Eubacteriales	Schaedlerella_arabinosiphila	DNA-sulfur-modification-protein-DndB-[Schaedlerella-arabinosiphila]	2044587
WP_221920263.1	DndB-ParB-nuclease	GCF_019754295.1	355	Bacteria>Terrabacteria_group>Firmicutes>Clostridia>Eubacteriales	Sellimonas_caecigallum	DNA-sulfur-modification-protein-DndB-[Sellimonas-caecigallum]	2592333
MBS6584128.1	DndB-ParB-nuclease	GCA_018377715.1	354	Bacteria>Terrabacteria_group>Firmicutes>Clostridia>Eubacteriales	Subdoligranulum_sp.	DNA-sulfur-modification-protein-DndB-[Subdoligranulum-sp.]	2053618
NLU49260.1	DndB-ParB-nuclease	GCA_012719535.1	389	Bacteria>Terrabacteria_group>Firmicutes>Clostridia>Eubacteriales	Syntrophomonadaceae_bacterium	DNA-sulfur-modification-protein-DndB-[Syntrophomonadaceae-bacterium]	2093811
NLG32053.1	DndB-ParB-nuclease	GCA_012515715.1	305	Bacteria>Terrabacteria_group>Firmicutes>Clostridia>Eubacteriales	Syntrophomonadaceae_bacterium	hypothetical-protein-[Syntrophomonadaceae-bacterium]	2093811
WP_118594465.1	DndB-ParB-nuclease	GCF_003479105.1	356	Bacteria>Terrabacteria_group>Firmicutes>Clostridia>Eubacteriales	unclassified_Blautia	MULTISPECIES:-DNA-sulfur-modification-protein-DndB-[unclassified-Blautia]	2648079
WP_117464665.1	DndB-ParB-nuclease	GCF_003435925.1	430	Bacteria>Terrabacteria_group>Firmicutes>Clostridia>Eubacteriales	unclassified_Clostridium	MULTISPECIES:-DGQHR-domain-containing-protein-[unclassified-Clostridium]	2614128
WP_221701306.1	DndB-ParB-nuclease	GCF_019734175.1	359	Bacteria>Terrabacteria_group>Firmicutes>Clostridia>Eubacteriales	unclassified_Clostridium	MULTISPECIES:-DNA-sulfur-modification-protein-DndB-[unclassified-Clostridium]	2614128
SCG94116.1	DndB-ParB-nuclease	GCA_900066565.1	353	Bacteria>Terrabacteria_group>Firmicutes>Clostridia>Eubacteriales	uncultured_Clostridium_sp.	DNA-sulfur-modification-protein-DndB-[uncultured-Clostridium-sp.]	59620
SCH53972.1	DndB-ParB-nuclease	GCA_900066575.1	351	Bacteria>Terrabacteria_group>Firmicutes>Clostridia>Eubacteriales	uncultured_Clostridium_sp.	DNA-sulfur-modification-protein-DndB-[uncultured-Clostridium-sp.]	59620
SCH61697.1	DndB-ParB-nuclease	GCA_900066525.1	356	Bacteria>Terrabacteria_group>Firmicutes>Clostridia>Eubacteriales	uncultured_Clostridium_sp.	DNA-sulfur-modification-protein-DndB-[uncultured-Clostridium-sp.]	59620
SCJ63525.1	DndB-ParB-nuclease	GCA_900066995.1	354	Bacteria>Terrabacteria_group>Firmicutes>Clostridia>Eubacteriales	uncultured_Ruminococcus_sp.	DNA-sulfur-modification-protein-DndB-[uncultured-Ruminococcus-sp.]	165186
MBP3401451.1	DndB-ParB-nuclease	GCA_017936125.1	83	Bacteria>Terrabacteria_group>Firmicutes>Clostridia>unclassified_Clostridia	Clostridia_bacterium	DNA-sulfur-modification-protein-DndB-[Clostridia-bacterium]	2044939
MBQ3496013.1	DndB-ParB-nuclease	GCA_017399185.1	353	Bacteria>Terrabacteria_group>Firmicutes>Clostridia>unclassified_Clostridia	Clostridia_bacterium	DNA-sulfur-modification-protein-DndB-[Clostridia-bacterium]	2044939
MBR2906466.1	DndB-ParB-nuclease	GCA_017501345.1	351	Bacteria>Terrabacteria_group>Firmicutes>Clostridia>unclassified_Clostridia	Clostridia_bacterium	DNA-sulfur-modification-protein-DndB-[Clostridia-bacterium]	2044939
MBR3885707.1	DndB-ParB-nuclease	GCA_017522705.1	357	Bacteria>Terrabacteria_group>Firmicutes>Clostridia>unclassified_Clostridia	Clostridia_bacterium	DNA-sulfur-modification-protein-DndB-[Clostridia-bacterium]	2044939
CDD71430.1	DndB-ParB-nuclease	GCA_000435775.1	358	Bacteria>Terrabacteria_group>Firmicutes>environmental_samples	Firmicutes_bacterium_CAG:270	dNA-sulfur-modification-protein-DndB-[Firmicutes-bacterium-CAG:270]	1263014
MBS6182769.1	DndB-ParB-nuclease	GCA_018374395.1	358	Bacteria>Terrabacteria_group>Firmicutes>Erysipelotrichia>Erysipelotrichales	Erysipelotrichaceae_bacterium	DNA-sulfur-modification-protein-DndB-[Erysipelotrichaceae-bacterium]	2049044
WP_183373715.1	DndB-ParB-nuclease	GCF_014202635.1	356	Bacteria>Terrabacteria_group>Firmicutes>Erysipelotrichia>Erysipelotrichales	Faecalicoccus_acidiformans	DNA-sulfur-modification-protein-DndB-[Faecalicoccus-acidiformans]	915173
WP_125119173.1	DndB-ParB-nuclease	GCF_003925875.1	357	Bacteria>Terrabacteria_group>Firmicutes>Erysipelotrichia>Erysipelotrichales	Intestinibaculum_porci	DNA-sulfur-modification-protein-DndB-[Intestinibaculum-porci]	2487118
WP_029072270.1	DndB-ParB-nuclease	GCF_000621925.1	361	Bacteria>Terrabacteria_group>Firmicutes>Erysipelotrichia>Erysipelotrichales	Kandleria_vitulina	DNA-sulfur-modification-protein-DndB-[Kandleria-vitulina]	1630
WP_154504217.1	DndB-ParB-nuclease	GCF_009696165.1	357	Bacteria>Terrabacteria_group>Firmicutes>Erysipelotrichia>Erysipelotrichales	Stecheria_intestinalis	DNA-sulfur-modification-protein-DndB-[Stecheria-intestinalis]	2606630
WP_196598964.1	DndB-ParB-nuclease	GCF_015732285.1	357	Bacteria>Terrabacteria_group>Firmicutes>Negativicutes>Selenomonadales	Pectinatus_frisingensis	DNA-sulfur-modification-protein-DndB-[Pectinatus-frisingensis]	865
GBG56072.1	DndB-ParB-nuclease	GCA_005381385.1	344	Bacteria>Terrabacteria_group>Firmicutes>Negativicutes>Selenomonadales	Sporomusaceae_bacterium_FL31	hypothetical-protein-SPFL3101_01354-[Sporomusaceae-bacterium-FL31]	2163554
WP_144351263.1	DndB-ParB-nuclease	GCF_007641255.1	364	Bacteria>Terrabacteria_group>Firmicutes>Negativicutes>Selenomonadales	Sporomusa_termitida	DNA-sulfur-modification-protein-DndB-[Sporomusa-termitida]	2377
HHX67359.1	DndB-ParB-nuclease	GCA_012841535.1	349	Bacteria>Terrabacteria_group>Firmicutes>Tissierellia>Tissierellales	Gallicola_sp.	DNA-sulfur-modification-protein-DndB-[Gallicola-sp.]	2683910
WP_179238653.1	DndB-ParB-nuclease	GCF_013403245.1	356	Bacteria>Terrabacteria_group>Firmicutes>Tissierellia>Tissierellia_incertae_sedis	Sedimentibacter_hydroxybenzoicus	DNA-sulfur-modification-protein-DndB-[Sedimentibacter-hydroxybenzoicus]	29345
NLK65420.1	DndB-ParB-nuclease	GCA_012519555.1	342	Bacteria>Terrabacteria_group>Firmicutes>Tissierellia>unclassified_Tissierellia	Tissierellia_bacterium	DNA-sulfur-modification-protein-DndB-[Tissierellia-bacterium]	2049431
ESL13566.1	DndB-ParB-nuclease	-	354	Bacteria>Terrabacteria_group>Firmicutes>unclassified_Firmicutes_sensu_stricto	Firmicutes_bacterium_ASF500	DNA-sulfur-modification-protein-DndB-[Firmicutes-bacterium-ASF500]	1378168

Accession	Annotation	GCA	Length	Phyletic.spread	Strain.name	Defline	Species.Taxid
KAF0197157.1	DndB-ParB-nuclease	GCA_009773495.1	366	Bacteria>Terrabacteria_group>Firmicutes>unclassified_Firmicutes_sensu_stricto	Firmicutes_bacterium	DGQHR-domain-containing-protein-[Firmicutes-bacterium]	1879010
MBS6206996.1	DndB-ParB-nuclease	GCA_018374595.1	354	Bacteria>Terrabacteria_group>Firmicutes>unclassified_Firmicutes_sensu_stricto	Firmicutes_bacterium	DNA-sulfur-modification-protein-DndB-[Firmicutes-bacterium]	1879010
MBP2656984.1	DndB-ParB-nuclease	GCA_017889665.1	359	Bacteria>Terrabacteria_group>Firmicutes>unclassified_Firmicutes_sensu_stricto	Firmicutes_bacterium	hypothetical-protein-[Firmicutes-bacterium]	1879010
MBO8161732.1	DndB-ParB-nuclease	GCA_017656345.1	362	Bacteria>Thermotogae>Thermotogae>Thermotogales>Fervidobacteriaceae	Thermosipho_sp._(in:_Bacteria)	DNA-sulfur-modification-protein-DndB-[Thermosipho-sp.-(in:-Bacteria)]	1968895
NBK94359.1	DndB-ParB-nuclease	GCA_009917555.1	349	Bacteria>unclassified_Bacteria	bacterium_1XD21-13	DNA-sulfur-modification-protein-DndB-[bacterium-1XD21-13]	2320888
RKJ47909.1	DndB-ParB-nuclease	GCA_003612585.1	357	Bacteria>unclassified_Bacteria	bacterium_1XD42-54	DNA-sulfur-modification-protein-DndB-[bacterium-1XD42-54]	2320110
NBJ82815.1	DndB-ParB-nuclease	GCA_009917545.1	427	Bacteria>unclassified_Bacteria	bacterium_1XD42-76	DGQHR-domain-containing-protein-[bacterium-1XD42-76]	2320881
RKI76487.1	DndB-ParB-nuclease	GCA_003611805.1	423	Bacteria>unclassified_Bacteria	bacterium_1xD42-87	DGQHR-domain-containing-protein-[bacterium-1xD42-87]	2320113
MBN4082977.1	DndB-ParB-nuclease	GCA_017116745.1	423	Bacteria>unclassified_Bacteria	bacterium_AH-315-A23	DGQHR-domain-containing-protein-[bacterium-AH-315-A23]	2814497
MBI1293690.1	DndB-ParB-nuclease	GCA_016124945.1	364	Bacteria>unclassified_Bacteria	bacterium	DNA-sulfur-modification-protein-DndB-[bacterium]	1869227
MBQ4122642.1	DndB-ParB-nuclease	GCA_017403285.1	356	Bacteria>unclassified_Bacteria	bacterium	DNA-sulfur-modification-protein-DndB-[bacterium]	1869227
MBT4763467.1	DndB-ParB-nuclease	GCA_018663345.1	356	Bacteria>unclassified_Bacteria	bacterium	DNA-sulfur-modification-protein-DndB-[bacterium]	1869227
HFS07928.1	DndB-ParB-nuclease	-	532	-	-	DGQHR-domain-containing-protein-[Anabaena-sp.]	-
HGY24402.1	DndB-ParB-nuclease	-	437	-	-	DGQHR-domain-containing-protein-[Candidatus-Bathyarchaeota-archaeon]	-
HGF99964.1	DndB-ParB-nuclease	-	525	-	-	DGQHR-domain-containing-protein-[Planktothricoides-sp.-SpSt-374]	-
HGF99965.1	DndB-ParB-nuclease	-	391	-	-	DGQHR-domain-containing-protein-[Planktothricoides-sp.-SpSt-374]	-
MBB1133319.1	DndB-ParB-nuclease	-	357	-	-	DGQHR-domain-containing-protein-[Pseudomonas-aeruginosa]	-
WP_210338363.1	DndB-ParB-nuclease	GCF_014852035.1	402	-	-	DNA-phosphorothioation-associated-DGQHR-protein-1-[Ensifer-sp.-ENS03]	-
MBP2140045.1	DndB-ParB-nuclease	-	376	-	-	DNA-sulfur-modification-protein-DndB-[Acidovorax-sp.-BE320]	-
MBR7553630.1	DndB-ParB-nuclease	GCA_018138385.1	368	-	-	DNA-sulfur-modification-protein-DndB-[Allobacillus-sp.-SKP8-2]	1955273
MBE0254611.1	DndB-ParB-nuclease	GCA_014856545.1	365	-	-	DNA-sulfur-modification-protein-DndB-[Bacillus-sp.-SBJS01]	2596923
WP_213402858.1	DndB-ParB-nuclease	-	364	-	-	DNA-sulfur-modification-protein-DndB-[Bacillus-sp.-TSO2]	716759
EIL2720856.1	DndB-ParB-nuclease	-	361	-	-	DNA-sulfur-modification-protein-DndB-[Citrobacter-koseri]	-
MBM7629496.1	DndB-ParB-nuclease	-	362	-	-	DNA-sulfur-modification-protein-DndB-[Cohnella-boryungensis]	-
HGW42320.1	DndB-ParB-nuclease	-	370	-	-	DNA-sulfur-modification-protein-DndB-[Hyphomicrobiales-bacterium]	-
MBE6935436.1	DndB-ParB-nuclease	GCA_015067705.1	355	-	-	DNA-sulfur-modification-protein-DndB-[Oscillospiraceae-bacterium]	1898205
MBE6959608.1	DndB-ParB-nuclease	GCA_015067945.1	354	-	-	DNA-sulfur-modification-protein-DndB-[Oscillospiraceae-bacterium]	1898205
NYH31563.1	DndB-ParB-nuclease	-	375	-	-	DNA-sulfur-modification-protein-DndB-[Paraburkholderia-bryophila]	-
NVM86142.1	DndB-ParB-nuclease	-	420	-	-	DNA-sulfur-modification-protein-DndB-[Rugamonas-sp.-SG757]	-
NVM86146.1	DndB-ParB-nuclease	-	366	-	-	DNA-sulfur-modification-protein-DndB-[Rugamonas-sp.-SG757]	-
MBB5946817.1	DndB-ParB-nuclease	-	357	-	-	DNA-sulfur-modification-protein-DndB-[Sphingobium-sp.-B1D3D]	-
HGT45528.1	DndB-ParB-nuclease	-	363	-	-	DNA-sulfur-modification-protein-DndB-[Verrucomicrobia-subdivision-3-bacterium]	-
EGR5369540.1	DndB-ParB-nuclease	-	364	-	-	DNA-sulfur-modification-protein-DndB-[Vibrio-parahaemolyticus]	-
MBB5879674.1	DndB-ParB-nuclease	-	414	-	-	DNA-sulfur-modification-protein-DndB-[Xanthomonas-arboricola]	-
MBD2792560.1	DndB-ParB-nuclease	-	362	-	-	DNA-sulfur-modification-protein-DndB-[Xenorhabdus-sp.-CUL]	-
HBW73408.1	DndB-ParB-nuclease	GCA_003514385.1	423	-	-	DndB-[Oscillospiraceae-bacterium]	1898205
NYH31570.1	DndB-ParB-nuclease	-	575	-	-	hypothetical-protein-[Paraburkholderia-bryophila]	-

Accession	Annotation	GCA	Length	Phyletic.spread	Strain.name	Defline	Species.Taxid
OLS18893.1	PAPS-reductase	GCA_001940645.1	511	Archaea>Asgard_group>Candidatus_Heimdallarchaeota	Candidatus_Heimdallarchaeota_archaeon_LC_3	hypothetical-protein-HeimC3_48310-[Candidatus-Heimdallarchaeota-archaeon-LC_3]	1841598
MBD3343367.1	PAPS-reductase	GCA_014729315.1	423	Archaea>Asgard_group>Candidatus_Lokiarchaeota>unclassified_Lokiarchaeota	Candidatus_Lokiarchaeota_archaeon	DNA-phosphorothioation-system-sulfurtransferase-DndC-[Candidatus-Lokiarchaeota-archaeon]	2053489
HII62663.1	PAPS-reductase	GCA_013330815.1	518	Archaea>Candidatus_Thermoplasmatota>Candidatus_Poseidoniia>Candidatus_Poseidoniales>Candidatus_Poseidoniaceae	Candidatus_Poseidoniaceae_archaeon	DNA-phosphorothioation-system-sulfurtransferase-DndC-[Candidatus-Poseidoniaceae-archaeon]	2666346
HIG20140.1	PAPS-reductase	GCA_012959435.1	298	Archaea>Candidatus_Thermoplasmatota>Candidatus_Poseidoniia>Candidatus_Poseidoniales>unclassified_Candidatus_Poseidoniales	Candidatus_Poseidoniales_archaeon	DNA-phosphorothioation-system-sulfurtransferase-DndC-[Candidatus-Poseidoniales-archaeon]	2163009
DAC17226.1	PAPS-reductase	GCA_003672705.1	518	Archaea>Candidatus_Thermoplasmatota>Candidatus_Poseidoniia>Candidatus_Poseidoniales>unclassified_Candidatus_Poseidoniales	Candidatus_Poseidoniales_archaeon	TPA:-DNA-phosphorothioation-system-sulfurtransferase-DndC-[Candidatus-Poseidoniales-archaeon]	2163009
MBQ3685660.1	PAPS-reductase	GCA_017412185.1	432	Archaea>Candidatus_Thermoplasmatota>Thermoplasmata>Methanomassiliicoccales>Candidatus_Methanomethylophilaceae	Candidatus_Methanomethylophilaceae_archaeon	DNA-phosphorothioation-system-sulfurtransferase-DndC-[Candidatus-Methanomethylophilaceae-archaeon]	2517205
AMH94605.1	PAPS-reductase	GCA_001560915.1	432	Archaea>Candidatus_Thermoplasmatota>Thermoplasmata>unclassified_Thermoplasmata	methanogenic_archaeon_ISO4-H5	sulfurtransferase-DndC-[methanogenic-archaeon-ISO4-H5]	1495144
MBN2881438.1	PAPS-reductase	GCA_016938695.1	445	Archaea>DPANN_group>Candidatus_Woesearchaeota	Candidatus_Woesearchaeota_archaeon	DNA-phosphorothioation-system-sulfurtransferase-DndC-[Candidatus-Woesearchaeota-archaeon]	2026803
MBT3813915.1	PAPS-reductase	GCA_018654495.1	469	Archaea>DPANN_group>Candidatus_Woesearchaeota	Candidatus_Woesearchaeota_archaeon	DNA-phosphorothioation-system-sulfurtransferase-DndC-[Candidatus-Woesearchaeota-archaeon]	2026803
MBT4110877.1	PAPS-reductase	GCA_018650945.1	469	Archaea>DPANN_group>Candidatus_Woesearchaeota	Candidatus_Woesearchaeota_archaeon	DNA-phosphorothioation-system-sulfurtransferase-DndC-[Candidatus-Woesearchaeota-archaeon]	2026803
MBT7556383.1	PAPS-reductase	GCA_018674815.1	385	Archaea>DPANN_group>Candidatus_Woesearchaeota	Candidatus_Woesearchaeota_archaeon	DNA-phosphorothioation-system-sulfurtransferase-DndC-[Candidatus-Woesearchaeota-archaeon]	2026803
RJQ15773.1	PAPS-reductase	GCA_003599145.1	465	Archaea>DPANN_group>Candidatus_Woesearchaeota	Candidatus_Woesearchaeota_archaeon	DNA-phosphorothioation-system-sulfurtransferase-DndC-[Candidatus-Woesearchaeota-archaeon]	2026803
MBL7116872.1	PAPS-reductase	GCA_016783785.1	334	Archaea>DPANN_group>Nanoarchaeota>unclassified_Nanoarchaeota	Nanoarchaeota_archaeon	phosphoadenosine-phosphosulfate-reductase-family-protein-[Nanoarchaeota-archaeon]	2026764
RLI77165.1	PAPS-reductase	GCA_003662995.1	454	Archaea>Euryarchaeota>Archaeoglobi>Archaeoglobales>unclassified_Archaeoglobales	Archaeoglobales_archaeon	DNA-phosphorothioation-system-sulfurtransferase-DndC-[Archaeoglobales-archaeon]	2250258
AIF04273.1	PAPS-reductase	-	375	Archaea>Euryarchaeota>environmental_samples	uncultured_marine_group_II/III_euryarchaeote_KM3_173_A11	3'-phosphoadenosine-5'-phosphosulfate-sulfotransferase-(PAPS-reductase)/FAD-synthetase-and(...)-[uncultured-marine-group-II/III-euryarchaeote-KM3_173_A11]	1457930
MBS7256901.1	PAPS-reductase	GCA_018385235.1	581	Archaea>Euryarchaeota>Methanomada_group>Methanobacteria>Methanobacteriales	Methanobrevibacter_sp.	phosphoadenosine-phosphosulfate-reductase-family-protein-[Methanobrevibacter-sp.]	66852
WP_220127027.1	PAPS-reductase	GCF_013761225.1	480	Archaea>Euryarchaeota>Methanomada_group>Methanococci>Methanococcales	Methanococcus_maripaludis	DNA-phosphorothioation-system-sulfurtransferase-DndC-[Methanococcus-maripaludis]	39152
NMA30224.1	PAPS-reductase	GCA_012799835.1	476	Archaea>Euryarchaeota>Stenosarchaea_group>Candidatus_Methanofastidiosa>unclassified_Candidatus_Methanofastidiosa	Candidatus_Methanofastidiosa_archaeon	DNA-phosphorothioation-system-sulfurtransferase-DndC-[Candidatus-Methanofastidiosa-archaeon]	2099674
NMC77230.1	PAPS-reductase	GCA_012797645.1	476	Archaea>Euryarchaeota>Stenosarchaea_group>Candidatus_Methanofastidiosa>unclassified_Candidatus_Methanofastidiosa	Candidatus_Methanofastidiosa_archaeon	DNA-phosphorothioation-system-sulfurtransferase-DndC-[Candidatus-Methanofastidiosa-archaeon]	2099674
WP_083252391.1	PAPS-reductase	GCF_001723155.1	504	Archaea>Euryarchaeota>Stenosarchaea_group>Halobacteria>Halobacteriales	Haladaptatus_sp._W1	DNA-phosphorothioation-system-sulfurtransferase-DndC-[Haladaptatus-sp.-W1]	1897478
QCC51447.1	PAPS-reductase	GCA_004799665.1	497	Archaea>Euryarchaeota>Stenosarchaea_group>Halobacteria>Halobacteriales	Halapricum_salinum	DNA-phosphorothioation-system-sulfurtransferase-DndC-[Halapricum-salinum]	1457250
WP_049994942.1	PAPS-reductase	GCF_000755225.1	497	Archaea>Euryarchaeota>Stenosarchaea_group>Halobacteria>Halobacteriales	Halapricum_salinum	DNA-phosphorothioation-system-sulfurtransferase-DndC-[Halapricum-salinum]	1457250
WP_221621764.1	PAPS-reductase	GCF_003862495.1	487	Archaea>Euryarchaeota>Stenosarchaea_group>Halobacteria>Halobacteriales	Halocatena_pleomorpha	DNA-phosphorothioation-system-sulfurtransferase-DndC-[Halomarina-oriensis]	1785090
WP_170094359.1	PAPS-reductase	GCF_012726095.1	500	Archaea>Euryarchaeota>Stenosarchaea_group>Halobacteria>Halobacteriales	Halomicrobium_mukohataei	DNA-phosphorothioation-system-sulfurtransferase-DndC-[Halomicrobium-mukohataei]	57705
WP_227261703.1	PAPS-reductase	GCF_020614395.1	493	Archaea>Euryarchaeota>Stenosarchaea_group>Halobacteria>Halobacteriales	Salarchaeum_japonicum	DNA-phosphorothioation-system-sulfurtransferase-DndC-[Salarchaeum-japonicum]	555573
WP_179268599.1	PAPS-reductase	GCF_013402875.1	495	Archaea>Euryarchaeota>Stenosarchaea_group>Halobacteria>Haloferacales	Halobaculum_salinum	DNA-phosphorothioation-system-sulfurtransferase-DndC-[Halobaculum-salinum]	2743089
WP_103992761.1	PAPS-reductase	GCF_004799685.1	493	Archaea>Euryarchaeota>Stenosarchaea_group>Halobacteria>Haloferacales	Halobellus_limi	DNA-phosphorothioation-system-sulfurtransferase-DndC-[Halobellus-limi]	699433
WP_185191435.1	PAPS-reductase	GCF_014212355.1	501	Archaea>Euryarchaeota>Stenosarchaea_group>Halobacteria>Haloferacales	Halobellus_ruber	DNA-phosphorothioation-system-sulfurtransferase-DndC-[Halobellus-ruber]	2761102
WP_121571074.1	PAPS-reductase	GCF_003665925.1	493	Archaea>Euryarchaeota>Stenosarchaea_group>Halobacteria>Haloferacales	Halobellus_sp._Atlit-38R	DNA-phosphorothioation-system-sulfurtransferase-DndC-[Halobellus-sp.-Atlit-38R]	2282131
WP_211609285.1	PAPS-reductase	GCF_900110465.1	501	Archaea>Euryarchaeota>Stenosarchaea_group>Halobacteria>Haloferacales	Halogranum_amylolyticum	DNA-phosphorothioation-system-sulfurtransferase-DndC-[Halogranum-amylolyticum]	660520
WP_121921657.1	PAPS-reductase	GCF_003856835.1	494	Archaea>Euryarchaeota>Stenosarchaea_group>Halobacteria>Haloferacales	Haloplanus_aerogenes	DNA-phosphorothioation-system-sulfurtransferase-DndC-[Haloplanus-aerogenes]	660522
RMB12939.1	PAPS-reductase	GCA_003688715.1	494	Archaea>Euryarchaeota>Stenosarchaea_group>Halobacteria>Haloferacales	Haloplanus_aerogenes	DNA-sulfur-modification-protein-DndC-[Haloplanus-aerogenes]	660522
WP_123619672.1	PAPS-reductase	GCF_003781945.1	493	Archaea>Euryarchaeota>Stenosarchaea_group>Halobacteria>Haloferacales	Halorubrum	MULTISPECIES:-DNA-phosphorothioation-system-sulfurtransferase-DndC-[Halorubrum]	56688
WP_144922257.1	PAPS-reductase	GCF_007671685.1	501	Archaea>Euryarchaeota>Stenosarchaea_group>Halobacteria>Haloferacales	Halorubrum_salsamenti	DNA-phosphorothioation-system-sulfurtransferase-DndC-[Halorubrum-salsamenti]	2583990
TKX87930.1	PAPS-reductase	GCA_005435045.1	501	Archaea>Euryarchaeota>Stenosarchaea_group>Halobacteria>Haloferacales	Halorubrum_sp._SS5	DNA-phosphorothioation-system-sulfurtransferase-DndC-[Halorubrum-sp.-SS5]	2518118
WP_207290880.1	PAPS-reductase	GCF_017352155.2	496	Archaea>Euryarchaeota>Stenosarchaea_group>Halobacteria>Natrialbales	Haloterrigena_alkaliphila	DNA-phosphorothioation-system-sulfurtransferase-DndC-[Haloterrigena-sp.-KZCA68]	2816475
WP_233274305.1	PAPS-reductase	GCF_000710605.1	497	Archaea>Euryarchaeota>Stenosarchaea_group>Halobacteria>Natrialbales	Haloterrigena_jeotgali	DNA-phosphorothioation-system-sulfurtransferase-DndC-[Haloterrigena-jeotgali]	413811
WP_136350881.1	PAPS-reductase	GCF_000337715.1	501	Archaea>Euryarchaeota>Stenosarchaea_group>Halobacteria>Natrialbales	Natronorubrum_bangense	DNA-phosphorothioation-system-sulfurtransferase-DndC-[Natronorubrum-bangense]	61858
ELY49717.1	PAPS-reductase	GCA_000337715.1	482	Archaea>Euryarchaeota>Stenosarchaea_group>Halobacteria>Natrialbales	Natronorubrum_bangense_JCM_10635	hypothetical-protein-C494_06870-[Natronorubrum-bangense-JCM-10635]	1227500
WP_160067777.1	PAPS-reductase	GCF_009834785.1	493	Archaea>Euryarchaeota>Stenosarchaea_group>Halobacteria>Natrialbales	Natronorubrum_halalkaliphilum	DNA-phosphorothioation-system-sulfurtransferase-DndC-[Natronorubrum-halalkaliphilum]	2691917
WP_141465264.1	PAPS-reductase	-	482	Archaea>Euryarchaeota>Stenosarchaea_group>Halobacteria>Natrialbales	Salinadaptatus_halalkaliphilus	DNA-phosphorothioation-system-sulfurtransferase-DndC-[Salinadaptatus-halalkaliphilus]	2419781
MBT8508694.1	PAPS-reductase	GCA_018687785.1	454	Archaea>Euryarchaeota>Stenosarchaea_group>Methanomicrobia>Methanomicrobiales	Methanomicrobiaceae_archaeon_CYW5	hypothetical-protein-[Methanomicrobiaceae-archaeon-CYW5]	1789227
PKL63559.1	PAPS-reductase	GCA_002839645.1	473	Archaea>Euryarchaeota>Stenosarchaea_group>Methanomicrobia>Methanomicrobiales	Methanomicrobiales_archaeon_HGW-Methanomicrobiales-2	DNA-phosphorothioation-system-sulfurtransferase-DndC-[Methanomicrobiales-archaeon-HGW-Methanomicrobiales-2]	2013818
WP_011991367.1	PAPS-reductase	GCF_000017625.1	487	Archaea>Euryarchaeota>Stenosarchaea_group>Methanomicrobia>Methanomicrobiales	Methanoregula_boonei	DNA-phosphorothioation-system-sulfurtransferase-DndC-[Methanoregula-boonei]	358766
OPX62559.1	PAPS-reductase	GCA_002067035.1	487	Archaea>Euryarchaeota>Stenosarchaea_group>Methanomicrobia>Methanomicrobiales	Methanoregula_sp._PtaB.Bin085	hypothetical-protein-A4E33_02308-[Methanoregula-sp.-PtaB.Bin085]	1811680
OPY36397.1	PAPS-reductase	GCA_002067895.1	464	Archaea>Euryarchaeota>Stenosarchaea_group>Methanomicrobia>Methanomicrobiales	Methanoregula_sp._PtaU1.Bin051	hypothetical-protein-A4E35_01936-[Methanoregula-sp.-PtaU1.Bin051]	1811682
WP_012618018.1	PAPS-reductase	GCF_000021965.1	486	Archaea>Euryarchaeota>Stenosarchaea_group>Methanomicrobia>Methanomicrobiales	Methanosphaerula_palustris	DNA-phosphorothioation-system-sulfurtransferase-DndC-[Methanosphaerula-palustris]	475088
ACL16699.1	PAPS-reductase	GCA_000021965.1	486	Archaea>Euryarchaeota>Stenosarchaea_group>Methanomicrobia>Methanomicrobiales	Methanosphaerula_palustris_E1-9c	sulfurtransferase-DndC-[Methanosphaerula-palustris-E1-9c]	521011
PXF61847.1	PAPS-reductase	GCA_003194445.1	458	Archaea>Euryarchaeota>Stenosarchaea_group>Methanomicrobia>Methanosarcinales	ANME-2_cluster_archaeon	DNA-phosphorothioation-system-sulfurtransferase-DndC-[ANME-2-cluster-archaeon]	2056317
NOR46499.1	PAPS-reductase	GCA_013139685.1	476	Archaea>Euryarchaeota>Stenosarchaea_group>Methanomicrobia>Methanosarcinales	Methanosarcinaceae_archaeon	DNA-phosphorothioation-system-sulfurtransferase-DndC-[Methanosarcinaceae-archaeon]	1945595
MCD4798386.1	PAPS-reductase	GCA_021108115.1	476	Archaea>Euryarchaeota>Stenosarchaea_group>Methanomicrobia>Methanosarcinales	Methanosarcinales_archaeon	DNA-phosphorothioation-system-sulfurtransferase-DndC-[Methanosarcinales-archaeon]	2250255
RLG24036.1	PAPS-reductase	GCA_003661045.1	477	Archaea>Euryarchaeota>Stenosarchaea_group>Methanomicrobia>Methanosarcinales	Methanosarcinales_archaeon	DNA-phosphorothioation-system-sulfurtransferase-DndC-[Methanosarcinales-archaeon]	2250255
WP_050035348.1	PAPS-reductase	GCF_000979335.1	479	Archaea>Euryarchaeota>Stenosarchaea_group>Methanomicrobia>Methanosarcinales	Methanosarcina_mazei	DNA-phosphorothioation-system-sulfurtransferase-DndC-[Methanosarcina-mazei]	2209
RQW79896.1	PAPS-reductase	GCA_003857025.1	475	Archaea>Euryarchaeota>Stenosarchaea_group>Methanomicrobia>Methanotrichales	Methanothrix_sp.	DNA-phosphorothioation-system-sulfurtransferase-DndC-[Methanothrix-sp.]	90426
MBT04146.1	PAPS-reductase	GCA_002727485.1	369	Archaea>Euryarchaeota>unclassified_Euryarchaeota	Euryarchaeota_archaeon	DNA-phosphorothioation-system-sulfurtransferase-DndC-[Euryarchaeota-archaeon]	2026739
MBT4406330.1	PAPS-reductase	GCA_018653665.1	375	Archaea>Euryarchaeota>unclassified_Euryarchaeota	Euryarchaeota_archaeon	DNA-phosphorothioation-system-sulfurtransferase-DndC-[Euryarchaeota-archaeon]	2026739
MBT7960395.1	PAPS-reductase	GCA_018676255.1	371	Archaea>Euryarchaeota>unclassified_Euryarchaeota	Euryarchaeota_archaeon	DNA-phosphorothioation-system-sulfurtransferase-DndC-[Euryarchaeota-archaeon]	2026739
MBT5122242.1	PAPS-reductase	GCA_018660715.1	210	Archaea>Euryarchaeota>unclassified_Euryarchaeota	Euryarchaeota_archaeon	hypothetical-protein-[Euryarchaeota-archaeon]	2026739
MBT5454434.1	PAPS-reductase	GCA_018665145.1	210	Archaea>Euryarchaeota>unclassified_Euryarchaeota	Euryarchaeota_archaeon	hypothetical-protein-[Euryarchaeota-archaeon]	2026739
MBT4060136.1	PAPS-reductase	GCA_018650285.1	375	Archaea>Euryarchaeota>unclassified_Euryarchaeota	Euryarchaeota_archaeon	phosphoadenosine-phosphosulfate-reductase-family-protein-[Euryarchaeota-archaeon]	2026739
AIF05632.1	PAPS-reductase	-	513	Archaea>TACK_group>Thaumarchaeota>environmental_samples	uncultured_marine_thaumarchaeote_KM3_186_C08	3'-phosphoadenosine-5'-phosphosulfate-sulfotransferase-(PAPS-reductase)/FAD-synthetase-and(...)-[uncultured-marine-thaumarchaeote-KM3_186_C08]	1456070
ARS64882.1	PAPS-reductase	GCA_002156965.1	515	Archaea>TACK_group>Thaumarchaeota>Nitrosopumilales>Nitrosopumilaceae	Candidatus_Nitrosomarinus_catalina	3'-phosphoadenosine-5'-phosphosulfate-sulfotransferase-DndC-[Candidatus-Nitrosomarinus-catalina]	1898749
WP_086907926.1	PAPS-reductase	GCF_002156965.1	515	Archaea>TACK_group>Thaumarchaeota>Nitrosopumilales>Nitrosopumilaceae	Candidatus_Nitrosomarinus_catalina	DNA-phosphorothioation-system-sulfurtransferase-DndC-[Candidatus-Nitrosomarinus-catalina]	1898749
EIJ65010.1	PAPS-reductase	GCA_000242875.3	319	Archaea>TACK_group>Thaumarchaeota>Nitrosopumilales>Nitrosopumilaceae	Candidatus_Nitrosopumilus_salaria_BD31	putative-sulfurtransferase-DndC-[Candidatus-Nitrosopumilus-salaria-BD31]	859350
WP_008301604.1	PAPS-reductase	GCF_000242875.2	319	Archaea>TACK_group>Thaumarchaeota>Nitrosopumilales>Nitrosopumilaceae	Candidatus_Nitrosopumilus_salaria	DNA-phosphorothioation-system-sulfurtransferase-DndC-[Candidatus-Nitrosopumilus-salaria]	1170320
PHY08231.1	PAPS-reductase	GCA_002737445.1	483	Archaea>TACK_group>Thaumarchaeota>Nitrosopumilales>Nitrosopumilaceae	Nitrosarchaeum_sp.	DNA-phosphorothioation-system-sulfurtransferase-DndC-[Nitrosarchaeum-sp.]	2026886
PHY08247.1	PAPS-reductase	GCA_002737445.1	476	Archaea>TACK_group>Thaumarchaeota>Nitrosopumilales>Nitrosopumilaceae	Nitrosarchaeum_sp.	DNA-phosphorothioation-system-sulfurtransferase-DndC-[Nitrosarchaeum-sp.]	2026886
VDK13108.1	PAPS-reductase	-	472	Archaea>TACK_group>Thaumarchaeota>Nitrosopumilales>Nitrosopumilaceae	Nitrosopumilaceae_archaeon	3'-phosphoadenosine-5'-phosphosulfate-sulfotransferase-(PAPS-reductase)/FAD-synthetase-and(...)-[Nitrosopumilaceae-archaeon]	2202732
MCE2498898.1	PAPS-reductase	GCA_021296175.1	532	Archaea>TACK_group>Thaumarchaeota>Nitrosopumilales>Nitrosopumilaceae	Nitrosopumilaceae_archaeon	DNA-phosphorothioation-system-sulfurtransferase-DndC-[Nitrosopumilaceae-archaeon]	2202732
MCE2508328.1	PAPS-reductase	GCA_021296165.1	501	Archaea>TACK_group>Thaumarchaeota>Nitrosopumilales>Nitrosopumilaceae	Nitrosopumilaceae_archaeon	DNA-phosphorothioation-system-sulfurtransferase-DndC-[Nitrosopumilaceae-archaeon]	2202732
KAF6243625.1	PAPS-reductase	GCA_014078545.1	506	Archaea>TACK_group>Thaumarchaeota>Nitrosopumilales>Nitrosopumilaceae	Nitrosopumilus_sp._b1	DNA-phosphorothioation-system-sulfurtransferase-DndC-[Nitrosopumilus-sp.-b1]	2109907
WP_182133243.1	PAPS-reductase	GCF_014078545.1	506	Archaea>TACK_group>Thaumarchaeota>Nitrosopumilales>Nitrosopumilaceae	Nitrosopumilus_sp._b1	DNA-phosphorothioation-system-sulfurtransferase-DndC-[Nitrosopumilus-sp.-b1]	2109907
MBC8501892.1	PAPS-reductase	GCA_014384445.1	513	Archaea>TACK_group>Thaumarchaeota>Nitrosopumilales>Nitrosopumilaceae	Nitrosopumilus_sp.	DNA-phosphorothioation-system-sulfurtransferase-DndC-[Nitrosopumilus-sp.]	2024843
MBC8502145.1	PAPS-reductase	GCA_014384445.1	476	Archaea>TACK_group>Thaumarchaeota>Nitrosopumilales>Nitrosopumilaceae	Nitrosopumilus_sp.	DNA-phosphorothioation-system-sulfurtransferase-DndC-[Nitrosopumilus-sp.]	2024843
MBL7001905.1	PAPS-reductase	GCA_016784625.1	502	Archaea>TACK_group>Thaumarchaeota>Nitrosopumilales>Nitrosopumilaceae	Nitrosopumilus_sp.	DNA-phosphorothioation-system-sulfurtransferase-DndC-[Nitrosopumilus-sp.]	2024843
NRA06466.1	PAPS-reductase	GCA_013215285.1	476	Archaea>TACK_group>Thaumarchaeota>Nitrosopumilales>Nitrosopumilaceae	Nitrosopumilus_sp.	DNA-phosphorothioation-system-sulfurtransferase-DndC-[Nitrosopumilus-sp.]	2024843
HIO32166.1	PAPS-reductase	GCA_015663895.1	476	Archaea>TACK_group>Thaumarchaeota>Thaumarchaeota_incertae_sedis>Candidatus_Nitrosopelagicus	Candidatus_Nitrosopelagicus_sp.	DNA-phosphorothioation-system-sulfurtransferase-DndC-[Candidatus-Nitrosopelagicus-sp.]	2026808
MBC8250875.1	PAPS-reductase	GCA_014381285.1	475	Archaea>TACK_group>Thaumarchaeota>Thaumarchaeota_incertae_sedis>Candidatus_Nitrosopelagicus	Candidatus_Nitrosopelagicus_sp.	DNA-phosphorothioation-system-sulfurtransferase-DndC-[Candidatus-Nitrosopelagicus-sp.]	2026808
MBT3761276.1	PAPS-reductase	GCA_018654855.1	546	Archaea>TACK_group>Thaumarchaeota>Thaumarchaeota_incertae_sedis>Candidatus_Nitrosopelagicus	Candidatus_Nitrosopelagicus_sp.	DNA-phosphorothioation-system-sulfurtransferase-DndC-[Candidatus-Nitrosopelagicus-sp.]	2026808
MBT4326512.1	PAPS-reductase	GCA_018652445.1	538	Archaea>TACK_group>Thaumarchaeota>Thaumarchaeota_incertae_sedis>Candidatus_Nitrosopelagicus	Candidatus_Nitrosopelagicus_sp.	DNA-phosphorothioation-system-sulfurtransferase-DndC-[Candidatus-Nitrosopelagicus-sp.]	2026808
MBT7252159.1	PAPS-reductase	GCA_018691945.1	258	Archaea>TACK_group>Thaumarchaeota>Thaumarchaeota_incertae_sedis>Candidatus_Nitrosopelagicus	Candidatus_Nitrosopelagicus_sp.	DNA-phosphorothioation-system-sulfurtransferase-DndC-[Candidatus-Nitrosopelagicus-sp.]	2026808
MBI1658936.1	PAPS-reductase	GCA_016126025.1	501	Archaea>TACK_group>Thaumarchaeota>unclassified_Thaumarchaeota	Thaumarchaeota_archaeon	DNA-phosphorothioation-system-sulfurtransferase-DndC-[Thaumarchaeota-archaeon]	2026795
MBT4176040.1	PAPS-reductase	GCA_018651685.1	482	Archaea>TACK_group>Thaumarchaeota>unclassified_Thaumarchaeota	Thaumarchaeota_archaeon	DNA-phosphorothioation-system-sulfurtransferase-DndC-[Thaumarchaeota-archaeon]	2026795
MBT6646241.1	PAPS-reductase	GCA_018697615.1	487	Archaea>TACK_group>Thaumarchaeota>unclassified_Thaumarchaeota	Thaumarchaeota_archaeon	DNA-phosphorothioation-system-sulfurtransferase-DndC-[Thaumarchaeota-archaeon]	2026795
RZD40204.1	PAPS-reductase	GCA_004195415.1	476	Archaea>TACK_group>Thaumarchaeota>unclassified_Thaumarchaeota	Thaumarchaeota_archaeon	DNA-phosphorothioation-system-sulfurtransferase-DndC-[Thaumarchaeota-archaeon]	2026795
RZD43272.1	PAPS-reductase	GCA_004195415.1	482	Archaea>TACK_group>Thaumarchaeota>unclassified_Thaumarchaeota	Thaumarchaeota_archaeon	DNA-phosphorothioation-system-sulfurtransferase-DndC-[Thaumarchaeota-archaeon]	2026795
MBS1260672.1	PAPS-reductase	GCA_018263495.1	475	Archaea>TACK_group>Thaumarchaeota>unclassified_Thaumarchaeota	Thaumarchaeota_archaeon	hypothetical-protein-[Thaumarchaeota-archaeon]	2026795
RNJ72392.1	PAPS-reductase	GCA_003724275.1	386	Archaea>TACK_group>Thaumarchaeota>unclassified_Thaumarchaeota	Thaumarchaeota_archaeon_S13	DNA-phosphorothioation-system-sulfurtransferase-DndC-[Thaumarchaeota-archaeon-S13]	2478557
RNJ74509.1	PAPS-reductase	GCA_003724175.1	471	Archaea>TACK_group>Thaumarchaeota>unclassified_Thaumarchaeota	Thaumarchaeota_archaeon_S15	DNA-phosphorothioation-system-sulfurtransferase-DndC-[Thaumarchaeota-archaeon-S15]	2478559
MBT4352522.1	PAPS-reductase	GCA_018653265.1	484	Archaea>unclassified_Archaea	archaeon	DNA-phosphorothioation-system-sulfurtransferase-DndC-[archaeon]	1906665
MBT4648403.1	PAPS-reductase	GCA_018657865.1	484	Archaea>unclassified_Archaea	archaeon	DNA-phosphorothioation-system-sulfurtransferase-DndC-[archaeon]	1906665
MBT7706579.1	PAPS-reductase	GCA_018676575.1	269	Archaea>unclassified_Archaea	archaeon	DNA-phosphorothioation-system-sulfurtransferase-DndC-[archaeon]	1906665
ACO31707.1	PAPS-reductase	GCA_000022565.1	512	Bacteria>Acidobacteria>Acidobacteriia>Acidobacteriales>Acidobacteriaceae	Acidobacterium_capsulatum_ATCC_51196	putative-sulfurtransferase-DndC-[Acidobacterium-capsulatum-ATCC-51196]	240015
WP_015898228.1	PAPS-reductase	GCF_000022565.1	512	Bacteria>Acidobacteria>Acidobacteriia>Acidobacteriales>Acidobacteriaceae	Acidobacterium_capsulatum	DNA-phosphorothioation-system-sulfurtransferase-DndC-[Acidobacterium-capsulatum]	33075
PYP82209.1	PAPS-reductase	GCA_003222245.1	485	Bacteria>Acidobacteria>Blastocatellia>unclassified_Blastocatellia	Blastocatellia_bacterium_AA13	DNA-phosphorothioation-system-sulfurtransferase-DndC-[Blastocatellia-bacterium-AA13]	2169413
PYP85887.1	PAPS-reductase	GCA_003222245.1	438	Bacteria>Acidobacteria>Blastocatellia>unclassified_Blastocatellia	Blastocatellia_bacterium_AA13	DNA-phosphorothioation-system-sulfurtransferase-DndC-[Blastocatellia-bacterium-AA13]	2169413
MBX7220554.1	PAPS-reductase	GCA_019695185.1	475	Bacteria>Acidobacteria>Blastocatellia>unclassified_Blastocatellia	Blastocatellia_bacterium	DNA-phosphorothioation-system-sulfurtransferase-DndC-[Blastocatellia-bacterium]	2052146
MYB19203.1	PAPS-reductase	GCA_009839125.1	280	Bacteria>Acidobacteria>Holophagae>Holophagales>unclassified_Holophagales	Holophagales_bacterium	phosphoadenosine-phosphosulfate-reductase-family-protein-[Holophagales-bacterium]	2478486
MBE3109161.1	PAPS-reductase	GCA_014894605.1	467	Bacteria>Acidobacteria>unclassified_Acidobacteria	Acidobacteria_bacterium	DNA-phosphorothioation-system-sulfurtransferase-DndC-[Acidobacteria-bacterium]	1978231
MBI3649441.1	PAPS-reductase	GCA_016201275.1	457	Bacteria>Acidobacteria>unclassified_Acidobacteria	Acidobacteria_bacterium	DNA-phosphorothioation-system-sulfurtransferase-DndC-[Acidobacteria-bacterium]	1978231
MBW4039111.1	PAPS-reductase	GCA_019351975.1	472	Bacteria>Acidobacteria>unclassified_Acidobacteria	Acidobacteria_bacterium	DNA-phosphorothioation-system-sulfurtransferase-DndC-[Acidobacteria-bacterium]	1978231
MYI75602.1	PAPS-reductase	GCA_009845485.1	504	Bacteria>Acidobacteria>unclassified_Acidobacteria	Acidobacteria_bacterium	DNA-phosphorothioation-system-sulfurtransferase-DndC-[Acidobacteria-bacterium]	1978231
PSH04059.1	PAPS-reductase	GCA_003010405.1	481	Bacteria>Acidobacteria>unclassified_Acidobacteria	Acidobacteria_bacterium	DNA-phosphorothioation-system-sulfurtransferase-DndC-[Acidobacteria-bacterium]	1978231
MXY25145.1	PAPS-reductase	GCA_009838525.1	279	Bacteria>Acidobacteria>unclassified_Acidobacteria	Acidobacteria_bacterium	phosphoadenosine-phosphosulfate-reductase-family-protein-[Acidobacteria-bacterium]	1978231
WP_200674547.1	PAPS-reductase	GCF_016617615.1	476	Bacteria>Aquificae>Aquificae>Aquificales>Hydrogenothermaceae	Persephonella_atlantica	DNA-phosphorothioation-system-sulfurtransferase-DndC-[Persephonella-atlantica]	2699429
WP_132527592.1	PAPS-reductase	GCF_004339575.1	478	Bacteria>Aquificae>Aquificae>Desulfurobacteriales>Desulfurobacteriaceae	Phorcysia_thermohydrogeniphila	DNA-phosphorothioation-system-sulfurtransferase-DndC-[Phorcysia-thermohydrogeniphila]	936138
TCK02902.1	PAPS-reductase	GCA_004339575.1	478	Bacteria>Aquificae>Aquificae>Desulfurobacteriales>Desulfurobacteriaceae	Phorcysia_thermohydrogeniphila	DNA-sulfur-modification-protein-DndC-[Phorcysia-thermohydrogeniphila]	936138
HEB13731.1	PAPS-reductase	GCA_011049675.1	269	Bacteria>Bacteria_incertae_sedis>Bacteria_candidate_phyla>candidate_division_CPR3	candidate_division_CPR3_bacterium	DNA-phosphorothioation-system-sulfurtransferase-DndC-[candidate-division-CPR3-bacterium]	2268181
MBL7074948.1	PAPS-reductase	GCA_016783505.1	452	Bacteria>Bacteria_incertae_sedis>Bacteria_candidate_phyla>candidate_division_KSB1>unclassified_candidate_division_KSB1	candidate_division_KSB1_bacterium	DNA-phosphorothioation-system-sulfurtransferase-DndC-[candidate-division-KSB1-bacterium]	2172550
MBN1969158.1	PAPS-reductase	GCA_016931755.1	465	Bacteria>Bacteria_incertae_sedis>Bacteria_candidate_phyla>Candidatus_Delongbacteria	Candidatus_Delongbacteria_bacterium	DNA-phosphorothioation-system-sulfurtransferase-DndC-[Candidatus-Delongbacteria-bacterium]	2044594
MBN2836537.1	PAPS-reductase	GCA_016938275.1	482	Bacteria>Bacteria_incertae_sedis>Bacteria_candidate_phyla>Candidatus_Delongbacteria	Candidatus_Delongbacteria_bacterium	DNA-phosphorothioation-system-sulfurtransferase-DndC-[Candidatus-Delongbacteria-bacterium]	2044594
PJB29749.1	PAPS-reductase	GCA_002790015.1	465	Bacteria>Bacteria_incertae_sedis>Bacteria_candidate_phyla>Candidatus_Desantisbacteria	Candidatus_Desantisbacteria_bacterium_CG_4_9_14_3_um_filter_40_11	DNA-phosphorothioation-system-sulfurtransferase-DndC-[Candidatus-Desantisbacteria-bacterium-CG_4_9_14_3_um_filter_40_11]	1974546
MBI1925915.1	PAPS-reductase	GCA_016178185.1	473	Bacteria>Bacteria_incertae_sedis>Bacteria_candidate_phyla>Candidatus_Poribacteria>unclassified_Candidatus_Poribacteria	Candidatus_Poribacteria_bacterium	DNA-phosphorothioation-system-sulfurtransferase-DndC-[Candidatus-Poribacteria-bacterium]	2026781
MYC76377.1	PAPS-reductase	GCA_009841255.1	469	Bacteria>Bacteria_incertae_sedis>Bacteria_candidate_phyla>Candidatus_Poribacteria>unclassified_Candidatus_Poribacteria	Candidatus_Poribacteria_bacterium	DNA-phosphorothioation-system-sulfurtransferase-DndC-[Candidatus-Poribacteria-bacterium]	2026781
MYE91173.1	PAPS-reductase	GCA_009840785.1	483	Bacteria>Bacteria_incertae_sedis>Bacteria_candidate_phyla>Candidatus_Poribacteria>unclassified_Candidatus_Poribacteria	Candidatus_Poribacteria_bacterium	DNA-phosphorothioation-system-sulfurtransferase-DndC-[Candidatus-Poribacteria-bacterium]	2026781
RKU24490.1	PAPS-reductase	GCA_003635255.1	469	Bacteria>Bacteria_incertae_sedis>Bacteria_candidate_phyla>Candidatus_Poribacteria>unclassified_Candidatus_Poribacteria	Candidatus_Poribacteria_bacterium	DNA-phosphorothioation-system-sulfurtransferase-DndC-[Candidatus-Poribacteria-bacterium]	2026781
RKU28220.1	PAPS-reductase	GCA_003635315.1	470	Bacteria>Bacteria_incertae_sedis>Bacteria_candidate_phyla>Candidatus_Poribacteria>unclassified_Candidatus_Poribacteria	Candidatus_Poribacteria_bacterium	DNA-phosphorothioation-system-sulfurtransferase-DndC-[Candidatus-Poribacteria-bacterium]	2026781
RKU15343.1	PAPS-reductase	GCA_003635185.1	272	Bacteria>Bacteria_incertae_sedis>Bacteria_candidate_phyla>Candidatus_Poribacteria>unclassified_Candidatus_Poribacteria	Candidatus_Poribacteria_bacterium	phosphoadenosine-phosphosulfate-reductase-[Candidatus-Poribacteria-bacterium]	2026781
RKU19318.1	PAPS-reductase	GCA_003635205.1	193	Bacteria>Bacteria_incertae_sedis>Bacteria_candidate_phyla>Candidatus_Poribacteria>unclassified_Candidatus_Poribacteria	Candidatus_Poribacteria_bacterium	phosphoadenosine-phosphosulfate-reductase,-partial-[Candidatus-Poribacteria-bacterium]	2026781
MBI3825594.1	PAPS-reductase	GCA_016200085.1	467	Bacteria>Bacteria_incertae_sedis>Bacteria_candidate_phyla>Candidatus_Rokubacteria>unclassified_Candidatus_Rokubacteria	Candidatus_Rokubacteria_bacterium	DNA-phosphorothioation-system-sulfurtransferase-DndC-[Candidatus-Rokubacteria-bacterium]	2053607
MBR6122301.1	PAPS-reductase	GCA_017541625.1	567	Bacteria>Bacteria_incertae_sedis>Bacteria_candidate_phyla>Candidatus_Saccharibacteria>unclassified_Candidatus_Saccharibacteria	Candidatus_Saccharibacteria_bacterium	phosphoadenosine-phosphosulfate-reductase-family-protein-[Candidatus-Saccharibacteria-bacterium]	2026720
MBR6122367.1	PAPS-reductase	GCA_017541625.1	579	Bacteria>Bacteria_incertae_sedis>Bacteria_candidate_phyla>Candidatus_Saccharibacteria>unclassified_Candidatus_Saccharibacteria	Candidatus_Saccharibacteria_bacterium	phosphoadenosine-phosphosulfate-reductase-family-protein-[Candidatus-Saccharibacteria-bacterium]	2026720
NJK50581.1	PAPS-reductase	GCA_012031035.1	527	Bacteria>Bacteria_incertae_sedis>Bacteria_candidate_phyla>Patescibacteria_group>Candidatus_Gracilibacteria	Candidatus_Gracilibacteria_bacterium	DNA-phosphorothioation-system-sulfurtransferase-DndC-[Candidatus-Gracilibacteria-bacterium]	2044595
RJQ25127.1	PAPS-reductase	GCA_003599675.1	571	Bacteria>Bacteria_incertae_sedis>Bacteria_candidate_phyla>Patescibacteria_group>Parcubacteria_group	Candidatus_Parcubacteria_bacterium	phosphoadenosine-phosphosulfate-reductase-[Candidatus-Parcubacteria-bacterium]	2762014
MBE0567700.1	PAPS-reductase	GCA_014859985.1	507	Bacteria>Candidatus_Krumholzbacteriota>Candidatus_Krumholzbacteria>unclassified_Candidatus_Krumholzbacteria	Candidatus_Krumholzbacteria_bacterium	DNA-phosphorothioation-system-sulfurtransferase-DndC-[Krumholzbacteria-bacterium]	2774603

Accession	Annotation	GCA	Length	Phyletic.spread	Strain.name	Defline	Species.Taxid
MXX96745.1	PAPS-reductase	GCA_009838195.1	311	Bacteria>FCB_group>Bacteroidetes/Chlorobi_group>Bacteroidetes>Bacteroidetes_Order_II._Incertae_sedis	Rhodothermaceae_bacterium	phosphoadenosine-phosphosulfate-reductase-family-protein-[Rhodothermaceae-bacterium]	2026787
WP_118838212.1	PAPS-reductase	GCF_003491185.1	468	Bacteria>FCB_group>Bacteroidetes/Chlorobi_group>Bacteroidetes>Bacteroidetes_Order_II._Incertae_sedis	Salinibacter_ruber	DNA-phosphorothioation-system-sulfurtransferase-DndC-[Salinibacter-ruber]	146919
KAA3144774.1	PAPS-reductase	GCA_008421245.1	445	Bacteria>FCB_group>Bacteroidetes/Chlorobi_group>Bacteroidetes>Bacteroidia	Alistipes_indistinctus	DNA-phosphorothioation-system-sulfurtransferase-DndC-[Alistipes-indistinctus]	626932
WP_195976871.1	PAPS-reductase	GCF_015668835.1	368	Bacteria>FCB_group>Bacteroidetes/Chlorobi_group>Bacteroidetes>Bacteroidia	Alistipes_onderdonkii	phosphoadenosine-phosphosulfate-reductase-[Alistipes-onderdonkii]	328813
OKY93720.1	PAPS-reductase	GCA_001915335.1	444	Bacteria>FCB_group>Bacteroidetes/Chlorobi_group>Bacteroidetes>Bacteroidia	Alistipes_putredinis	sulfurtransferase-[Alistipes-putredinis]	28117
WP_019150955.1	PAPS-reductase	GCF_902374845.1	446	Bacteria>FCB_group>Bacteroidetes/Chlorobi_group>Bacteroidetes>Bacteroidia	Alistipes_senegalensis	DNA-phosphorothioation-system-sulfurtransferase-DndC-[Alistipes-senegalensis]	1288121
WP_195498338.1	PAPS-reductase	GCF_015554845.1	446	Bacteria>FCB_group>Bacteroidetes/Chlorobi_group>Bacteroidetes>Bacteroidia	Alistipes_senegalensis	DNA-phosphorothioation-system-sulfurtransferase-DndC-[Alistipes-senegalensis]	1288121
WP_196016489.1	PAPS-reductase	GCF_015666955.1	444	Bacteria>FCB_group>Bacteroidetes/Chlorobi_group>Bacteroidetes>Bacteroidia	Alistipes_senegalensis	DNA-phosphorothioation-system-sulfurtransferase-DndC-[Alistipes-senegalensis]	1288121
MBP3481999.1	PAPS-reductase	GCA_017937125.1	443	Bacteria>FCB_group>Bacteroidetes/Chlorobi_group>Bacteroidetes>Bacteroidia	Alistipes_sp.	DNA-phosphorothioation-system-sulfurtransferase-DndC-[Alistipes-sp.]	1872444
MBQ3186735.1	PAPS-reductase	GCA_017398045.1	446	Bacteria>FCB_group>Bacteroidetes/Chlorobi_group>Bacteroidetes>Bacteroidia	Alistipes_sp.	DNA-phosphorothioation-system-sulfurtransferase-DndC-[Alistipes-sp.]	1872444
MBQ7952640.1	PAPS-reductase	GCA_017468275.1	368	Bacteria>FCB_group>Bacteroidetes/Chlorobi_group>Bacteroidetes>Bacteroidia	Alistipes_sp.	phosphoadenosine-phosphosulfate-reductase-[Alistipes-sp.]	1872444
NBJ06605.1	PAPS-reductase	GCA_009911575.1	447	Bacteria>FCB_group>Bacteroidetes/Chlorobi_group>Bacteroidetes>Bacteroidia	Alistipes_sp._Z76	DNA-phosphorothioation-system-sulfurtransferase-DndC-[Alistipes-sp.-Z76]	2304565
WP_124931867.1	PAPS-reductase	GCF_003859795.1	368	Bacteria>FCB_group>Bacteroidetes/Chlorobi_group>Bacteroidetes>Bacteroidia	Alloprevotella_sp._OH1205_COT-284	phosphoadenosine-phosphosulfate-reductase-[Alloprevotella-sp.-OH1205_COT-284]	2491043
WP_237785528.1	PAPS-reductase	-	446	Bacteria>FCB_group>Bacteroidetes/Chlorobi_group>Bacteroidetes>Bacteroidia	Alloprevotella_tannerae	DNA-phosphorothioation-system-sulfurtransferase-DndC-[Alloprevotella-tannerae]	76122
WP_006254209.1	PAPS-reductase	GCF_000159995.1	369	Bacteria>FCB_group>Bacteroidetes/Chlorobi_group>Bacteroidetes>Bacteroidia	Alloprevotella_tannerae	phosphoadenosine-phosphosulfate-reductase-[Alloprevotella-tannerae]	76122
MBO5083819.1	PAPS-reductase	GCA_017622495.1	443	Bacteria>FCB_group>Bacteroidetes/Chlorobi_group>Bacteroidetes>Bacteroidia	Bacteroidaceae_bacterium	DNA-phosphorothioation-system-sulfurtransferase-DndC-[Bacteroidaceae-bacterium]	2212467
MBO7417136.1	PAPS-reductase	GCA_017649545.1	445	Bacteria>FCB_group>Bacteroidetes/Chlorobi_group>Bacteroidetes>Bacteroidia	Bacteroidaceae_bacterium	DNA-phosphorothioation-system-sulfurtransferase-DndC-[Bacteroidaceae-bacterium]	2212467
MBR0432130.1	PAPS-reductase	GCA_017429525.1	450	Bacteria>FCB_group>Bacteroidetes/Chlorobi_group>Bacteroidetes>Bacteroidia	Bacteroidaceae_bacterium	DNA-phosphorothioation-system-sulfurtransferase-DndC-[Bacteroidaceae-bacterium]	2212467
MBR2416723.1	PAPS-reductase	GCA_017503645.1	442	Bacteria>FCB_group>Bacteroidetes/Chlorobi_group>Bacteroidetes>Bacteroidia	Bacteroidaceae_bacterium	DNA-phosphorothioation-system-sulfurtransferase-DndC-[Bacteroidaceae-bacterium]	2212467
MBO5135437.1	PAPS-reductase	GCA_017622315.1	369	Bacteria>FCB_group>Bacteroidetes/Chlorobi_group>Bacteroidetes>Bacteroidia	Bacteroidaceae_bacterium	phosphoadenosine-phosphosulfate-reductase-[Bacteroidaceae-bacterium]	2212467
MBQ8360777.1	PAPS-reductase	GCA_017451645.1	368	Bacteria>FCB_group>Bacteroidetes/Chlorobi_group>Bacteroidetes>Bacteroidia	Bacteroidaceae_bacterium	phosphoadenosine-phosphosulfate-reductase-[Bacteroidaceae-bacterium]	2212467
MBR0183522.1	PAPS-reductase	GCA_017448405.1	366	Bacteria>FCB_group>Bacteroidetes/Chlorobi_group>Bacteroidetes>Bacteroidia	Bacteroidaceae_bacterium	phosphoadenosine-phosphosulfate-reductase-[Bacteroidaceae-bacterium]	2212467
MBR1665845.1	PAPS-reductase	GCA_017470405.1	367	Bacteria>FCB_group>Bacteroidetes/Chlorobi_group>Bacteroidetes>Bacteroidia	Bacteroidaceae_bacterium	phosphoadenosine-phosphosulfate-reductase-[Bacteroidaceae-bacterium]	2212467
MBR5575898.1	PAPS-reductase	GCA_017558255.1	368	Bacteria>FCB_group>Bacteroidetes/Chlorobi_group>Bacteroidetes>Bacteroidia	Bacteroidaceae_bacterium	phosphoadenosine-phosphosulfate-reductase-[Bacteroidaceae-bacterium]	2212467
MBQ7361760.1	PAPS-reductase	GCA_017452965.1	839	Bacteria>FCB_group>Bacteroidetes/Chlorobi_group>Bacteroidetes>Bacteroidia	Bacteroidaceae_bacterium	phosphoadenosine-phosphosulfate-reductase-family-protein-[Bacteroidaceae-bacterium]	2212467
WP_015532016.1	PAPS-reductase	GCF_000210075.1	443	Bacteria>FCB_group>Bacteroidetes/Chlorobi_group>Bacteroidetes>Bacteroidia	Bacteroidaceae	MULTISPECIES:-DNA-phosphorothioation-system-sulfurtransferase-DndC-[Bacteroidaceae]	815
WP_016271523.1	PAPS-reductase	GCF_012976275.1	446	Bacteria>FCB_group>Bacteroidetes/Chlorobi_group>Bacteroidetes>Bacteroidia	Bacteroidaceae	MULTISPECIES:-DNA-phosphorothioation-system-sulfurtransferase-DndC-[Bacteroidaceae]	815
WP_055256793.1	PAPS-reductase	GCF_001405515.1	443	Bacteria>FCB_group>Bacteroidetes/Chlorobi_group>Bacteroidetes>Bacteroidia	Bacteroidaceae	MULTISPECIES:-DNA-phosphorothioation-system-sulfurtransferase-DndC-[Bacteroidaceae]	815
WP_143866348.1	PAPS-reductase	GCF_009193325.2	445	Bacteria>FCB_group>Bacteroidetes/Chlorobi_group>Bacteroidetes>Bacteroidia	Bacteroidaceae	MULTISPECIES:-DNA-phosphorothioation-system-sulfurtransferase-DndC-[Bacteroidaceae]	815
OKY99812.1	PAPS-reductase	GCA_001915345.1	443	Bacteria>FCB_group>Bacteroidetes/Chlorobi_group>Bacteroidetes>Bacteroidia	Bacteroidales_bacterium_43_36	sulfurtransferase-[Bacteroidales-bacterium-43_36]	1897038
OAV73669.1	PAPS-reductase	GCA_001657625.1	444	Bacteria>FCB_group>Bacteroidetes/Chlorobi_group>Bacteroidetes>Bacteroidia	Bacteroidales_bacterium_Barb6	hypothetical-protein-Barb6_00047-[Bacteroidales-bacterium-Barb6]	1633201
HBN05094.1	PAPS-reductase	GCA_003503875.1	411	Bacteria>FCB_group>Bacteroidetes/Chlorobi_group>Bacteroidetes>Bacteroidia	Bacteroidales_bacterium	DNA-phosphorothioation-system-sulfurtransferase-DndC-[Bacteroidales-bacterium]	2030927
HBN05553.1	PAPS-reductase	GCA_003503875.1	447	Bacteria>FCB_group>Bacteroidetes/Chlorobi_group>Bacteroidetes>Bacteroidia	Bacteroidales_bacterium	DNA-phosphorothioation-system-sulfurtransferase-DndC-[Bacteroidales-bacterium]	2030927
MBE6312338.1	PAPS-reductase	GCA_015061145.1	444	Bacteria>FCB_group>Bacteroidetes/Chlorobi_group>Bacteroidetes>Bacteroidia	Bacteroidales_bacterium	DNA-phosphorothioation-system-sulfurtransferase-DndC-[Bacteroidales-bacterium]	2030927
MBI9055109.1	PAPS-reductase	GCA_016342685.1	450	Bacteria>FCB_group>Bacteroidetes/Chlorobi_group>Bacteroidetes>Bacteroidia	Bacteroidales_bacterium	DNA-phosphorothioation-system-sulfurtransferase-DndC-[Bacteroidales-bacterium]	2030927
MBK7625656.1	PAPS-reductase	GCA_016709865.1	447	Bacteria>FCB_group>Bacteroidetes/Chlorobi_group>Bacteroidetes>Bacteroidia	Bacteroidales_bacterium	DNA-phosphorothioation-system-sulfurtransferase-DndC-[Bacteroidales-bacterium]	2030927
MBN2891872.1	PAPS-reductase	GCA_016938685.1	400	Bacteria>FCB_group>Bacteroidetes/Chlorobi_group>Bacteroidetes>Bacteroidia	Bacteroidales_bacterium	DNA-phosphorothioation-system-sulfurtransferase-DndC-[Bacteroidales-bacterium]	2030927
MBO4739885.1	PAPS-reductase	GCA_017618435.1	446	Bacteria>FCB_group>Bacteroidetes/Chlorobi_group>Bacteroidetes>Bacteroidia	Bacteroidales_bacterium	DNA-phosphorothioation-system-sulfurtransferase-DndC-[Bacteroidales-bacterium]	2030927
MBO7276351.1	PAPS-reductase	GCA_017647785.1	445	Bacteria>FCB_group>Bacteroidetes/Chlorobi_group>Bacteroidetes>Bacteroidia	Bacteroidales_bacterium	DNA-phosphorothioation-system-sulfurtransferase-DndC-[Bacteroidales-bacterium]	2030927
MBO7278970.1	PAPS-reductase	GCA_017647795.1	443	Bacteria>FCB_group>Bacteroidetes/Chlorobi_group>Bacteroidetes>Bacteroidia	Bacteroidales_bacterium	DNA-phosphorothioation-system-sulfurtransferase-DndC-[Bacteroidales-bacterium]	2030927
MBQ4408861.1	PAPS-reductase	GCA_017395345.1	435	Bacteria>FCB_group>Bacteroidetes/Chlorobi_group>Bacteroidetes>Bacteroidia	Bacteroidales_bacterium	DNA-phosphorothioation-system-sulfurtransferase-DndC-[Bacteroidales-bacterium]	2030927
MBQ5891036.1	PAPS-reductase	GCA_017381455.1	445	Bacteria>FCB_group>Bacteroidetes/Chlorobi_group>Bacteroidetes>Bacteroidia	Bacteroidales_bacterium	DNA-phosphorothioation-system-sulfurtransferase-DndC-[Bacteroidales-bacterium]	2030927
MBQ6878928.1	PAPS-reductase	GCA_017437125.1	443	Bacteria>FCB_group>Bacteroidetes/Chlorobi_group>Bacteroidetes>Bacteroidia	Bacteroidales_bacterium	DNA-phosphorothioation-system-sulfurtransferase-DndC-[Bacteroidales-bacterium]	2030927
MBQ9213667.1	PAPS-reductase	GCA_017459065.1	435	Bacteria>FCB_group>Bacteroidetes/Chlorobi_group>Bacteroidetes>Bacteroidia	Bacteroidales_bacterium	DNA-phosphorothioation-system-sulfurtransferase-DndC-[Bacteroidales-bacterium]	2030927
MBR1792967.1	PAPS-reductase	GCA_017480145.1	445	Bacteria>FCB_group>Bacteroidetes/Chlorobi_group>Bacteroidetes>Bacteroidia	Bacteroidales_bacterium	DNA-phosphorothioation-system-sulfurtransferase-DndC-[Bacteroidales-bacterium]	2030927
MBR4326123.1	PAPS-reductase	GCA_017542615.1	435	Bacteria>FCB_group>Bacteroidetes/Chlorobi_group>Bacteroidetes>Bacteroidia	Bacteroidales_bacterium	DNA-phosphorothioation-system-sulfurtransferase-DndC-[Bacteroidales-bacterium]	2030927
MBR5660105.1	PAPS-reductase	GCA_017548985.1	443	Bacteria>FCB_group>Bacteroidetes/Chlorobi_group>Bacteroidetes>Bacteroidia	Bacteroidales_bacterium	DNA-phosphorothioation-system-sulfurtransferase-DndC-[Bacteroidales-bacterium]	2030927
NLJ20471.1	PAPS-reductase	GCA_012517575.1	445	Bacteria>FCB_group>Bacteroidetes/Chlorobi_group>Bacteroidetes>Bacteroidia	Bacteroidales_bacterium	DNA-phosphorothioation-system-sulfurtransferase-DndC-[Bacteroidales-bacterium]	2030927
TGX84253.1	PAPS-reductase	GCA_004792655.1	446	Bacteria>FCB_group>Bacteroidetes/Chlorobi_group>Bacteroidetes>Bacteroidia	Bacteroidales_bacterium	DNA-phosphorothioation-system-sulfurtransferase-DndC-[Bacteroidales-bacterium]	2030927
MCD4666139.1	PAPS-reductase	GCA_021107235.1	466	Bacteria>FCB_group>Bacteroidetes/Chlorobi_group>Bacteroidetes>Bacteroidia	Bacteroidales_bacterium	DNA-phosphorothioation-system-sulfurtransferase-DndC,-partial-[Bacteroidales-bacterium]	2030927
SDF39110.1	PAPS-reductase	GCA_900102385.1	368	Bacteria>FCB_group>Bacteroidetes/Chlorobi_group>Bacteroidetes>Bacteroidia	Bacteroidales_bacterium_KHT7	hypothetical-protein-SAMN05216518_10681-[Bacteroidales-bacterium-KHT7]	1855373
HBG69684.1	PAPS-reductase	GCA_003499605.1	365	Bacteria>FCB_group>Bacteroidetes/Chlorobi_group>Bacteroidetes>Bacteroidia	Bacteroidales_bacterium	phosphoadenosine-phosphosulfate-reductase-[Bacteroidales-bacterium]	2030927
MBD5201895.1	PAPS-reductase	GCA_014800585.1	370	Bacteria>FCB_group>Bacteroidetes/Chlorobi_group>Bacteroidetes>Bacteroidia	Bacteroidales_bacterium	phosphoadenosine-phosphosulfate-reductase-[Bacteroidales-bacterium]	2030927
MBD5217067.1	PAPS-reductase	GCA_014800685.1	370	Bacteria>FCB_group>Bacteroidetes/Chlorobi_group>Bacteroidetes>Bacteroidia	Bacteroidales_bacterium	phosphoadenosine-phosphosulfate-reductase-[Bacteroidales-bacterium]	2030927
MBO4574633.1	PAPS-reductase	GCA_017616735.1	368	Bacteria>FCB_group>Bacteroidetes/Chlorobi_group>Bacteroidetes>Bacteroidia	Bacteroidales_bacterium	phosphoadenosine-phosphosulfate-reductase-[Bacteroidales-bacterium]	2030927
MBO5941838.1	PAPS-reductase	GCA_017631875.1	369	Bacteria>FCB_group>Bacteroidetes/Chlorobi_group>Bacteroidetes>Bacteroidia	Bacteroidales_bacterium	phosphoadenosine-phosphosulfate-reductase-[Bacteroidales-bacterium]	2030927
MBO6248517.1	PAPS-reductase	GCA_017635375.1	365	Bacteria>FCB_group>Bacteroidetes/Chlorobi_group>Bacteroidetes>Bacteroidia	Bacteroidales_bacterium	phosphoadenosine-phosphosulfate-reductase-[Bacteroidales-bacterium]	2030927
MBO7224710.1	PAPS-reductase	GCA_017647065.1	367	Bacteria>FCB_group>Bacteroidetes/Chlorobi_group>Bacteroidetes>Bacteroidia	Bacteroidales_bacterium	phosphoadenosine-phosphosulfate-reductase-[Bacteroidales-bacterium]	2030927
MBO7494835.1	PAPS-reductase	GCA_017650065.1	368	Bacteria>FCB_group>Bacteroidetes/Chlorobi_group>Bacteroidetes>Bacteroidia	Bacteroidales_bacterium	phosphoadenosine-phosphosulfate-reductase-[Bacteroidales-bacterium]	2030927
MBP5369449.1	PAPS-reductase	GCA_017960885.1	363	Bacteria>FCB_group>Bacteroidetes/Chlorobi_group>Bacteroidetes>Bacteroidia	Bacteroidales_bacterium	phosphoadenosine-phosphosulfate-reductase-[Bacteroidales-bacterium]	2030927
MBP5503081.1	PAPS-reductase	GCA_017962175.1	363	Bacteria>FCB_group>Bacteroidetes/Chlorobi_group>Bacteroidetes>Bacteroidia	Bacteroidales_bacterium	phosphoadenosine-phosphosulfate-reductase-[Bacteroidales-bacterium]	2030927
MBQ0029665.1	PAPS-reductase	GCA_018064855.1	368	Bacteria>FCB_group>Bacteroidetes/Chlorobi_group>Bacteroidetes>Bacteroidia	Bacteroidales_bacterium	phosphoadenosine-phosphosulfate-reductase-[Bacteroidales-bacterium]	2030927
MBQ0123959.1	PAPS-reductase	GCA_018065895.1	367	Bacteria>FCB_group>Bacteroidetes/Chlorobi_group>Bacteroidetes>Bacteroidia	Bacteroidales_bacterium	phosphoadenosine-phosphosulfate-reductase-[Bacteroidales-bacterium]	2030927
MBQ2187214.1	PAPS-reductase	GCA_017425595.1	366	Bacteria>FCB_group>Bacteroidetes/Chlorobi_group>Bacteroidetes>Bacteroidia	Bacteroidales_bacterium	phosphoadenosine-phosphosulfate-reductase-[Bacteroidales-bacterium]	2030927
MBQ2395587.1	PAPS-reductase	GCA_017424325.1	366	Bacteria>FCB_group>Bacteroidetes/Chlorobi_group>Bacteroidetes>Bacteroidia	Bacteroidales_bacterium	phosphoadenosine-phosphosulfate-reductase-[Bacteroidales-bacterium]	2030927
MBQ3251416.1	PAPS-reductase	GCA_017415715.1	368	Bacteria>FCB_group>Bacteroidetes/Chlorobi_group>Bacteroidetes>Bacteroidia	Bacteroidales_bacterium	phosphoadenosine-phosphosulfate-reductase-[Bacteroidales-bacterium]	2030927
MBQ4441137.1	PAPS-reductase	GCA_017394765.1	367	Bacteria>FCB_group>Bacteroidetes/Chlorobi_group>Bacteroidetes>Bacteroidia	Bacteroidales_bacterium	phosphoadenosine-phosphosulfate-reductase-[Bacteroidales-bacterium]	2030927
MBQ5975286.1	PAPS-reductase	GCA_017439105.1	366	Bacteria>FCB_group>Bacteroidetes/Chlorobi_group>Bacteroidetes>Bacteroidia	Bacteroidales_bacterium	phosphoadenosine-phosphosulfate-reductase-[Bacteroidales-bacterium]	2030927
MBQ6175777.1	PAPS-reductase	GCA_017429205.1	367	Bacteria>FCB_group>Bacteroidetes/Chlorobi_group>Bacteroidetes>Bacteroidia	Bacteroidales_bacterium	phosphoadenosine-phosphosulfate-reductase-[Bacteroidales-bacterium]	2030927
MBQ6305727.1	PAPS-reductase	GCA_017428285.1	366	Bacteria>FCB_group>Bacteroidetes/Chlorobi_group>Bacteroidetes>Bacteroidia	Bacteroidales_bacterium	phosphoadenosine-phosphosulfate-reductase-[Bacteroidales-bacterium]	2030927
MBR3571769.1	PAPS-reductase	GCA_017527345.1	368	Bacteria>FCB_group>Bacteroidetes/Chlorobi_group>Bacteroidetes>Bacteroidia	Bacteroidales_bacterium	phosphoadenosine-phosphosulfate-reductase-[Bacteroidales-bacterium]	2030927
MBR3784121.1	PAPS-reductase	GCA_017522105.1	366	Bacteria>FCB_group>Bacteroidetes/Chlorobi_group>Bacteroidetes>Bacteroidia	Bacteroidales_bacterium	phosphoadenosine-phosphosulfate-reductase-[Bacteroidales-bacterium]	2030927
MBR4647917.1	PAPS-reductase	GCA_017525245.1	366	Bacteria>FCB_group>Bacteroidetes/Chlorobi_group>Bacteroidetes>Bacteroidia	Bacteroidales_bacterium	phosphoadenosine-phosphosulfate-reductase-[Bacteroidales-bacterium]	2030927
MBR4690160.1	PAPS-reductase	GCA_017535565.1	365	Bacteria>FCB_group>Bacteroidetes/Chlorobi_group>Bacteroidetes>Bacteroidia	Bacteroidales_bacterium	phosphoadenosine-phosphosulfate-reductase-[Bacteroidales-bacterium]	2030927
MBR5082446.1	PAPS-reductase	GCA_017548325.1	366	Bacteria>FCB_group>Bacteroidetes/Chlorobi_group>Bacteroidetes>Bacteroidia	Bacteroidales_bacterium	phosphoadenosine-phosphosulfate-reductase-[Bacteroidales-bacterium]	2030927
MBR5211198.1	PAPS-reductase	GCA_017549505.1	369	Bacteria>FCB_group>Bacteroidetes/Chlorobi_group>Bacteroidetes>Bacteroidia	Bacteroidales_bacterium	phosphoadenosine-phosphosulfate-reductase-[Bacteroidales-bacterium]	2030927
MBR5353959.1	PAPS-reductase	GCA_017550405.1	366	Bacteria>FCB_group>Bacteroidetes/Chlorobi_group>Bacteroidetes>Bacteroidia	Bacteroidales_bacterium	phosphoadenosine-phosphosulfate-reductase-[Bacteroidales-bacterium]	2030927
MBR6178528.1	PAPS-reductase	GCA_017540625.1	363	Bacteria>FCB_group>Bacteroidetes/Chlorobi_group>Bacteroidetes>Bacteroidia	Bacteroidales_bacterium	phosphoadenosine-phosphosulfate-reductase-[Bacteroidales-bacterium]	2030927
MCF0210704.1	PAPS-reductase	GCA_021443125.1	365	Bacteria>FCB_group>Bacteroidetes/Chlorobi_group>Bacteroidetes>Bacteroidia	Bacteroidales_bacterium	phosphoadenosine-phosphosulfate-reductase-[Bacteroidales-bacterium]	2030927
MBQ0053430.1	PAPS-reductase	GCA_018065035.1	841	Bacteria>FCB_group>Bacteroidetes/Chlorobi_group>Bacteroidetes>Bacteroidia	Bacteroidales_bacterium	phosphoadenosine-phosphosulfate-reductase-family-protein-[Bacteroidales-bacterium]	2030927
WP_087251527.1	PAPS-reductase	GCF_002159645.1	443	Bacteria>FCB_group>Bacteroidetes/Chlorobi_group>Bacteroidetes>Bacteroidia	Bacteroidales	MULTISPECIES:-DNA-phosphorothioation-system-sulfurtransferase-DndC-[Bacteroidales]	171549
WP_025018537.1	PAPS-reductase	GCF_020885715.1	368	Bacteria>FCB_group>Bacteroidetes/Chlorobi_group>Bacteroidetes>Bacteroidia	Bacteroidales	MULTISPECIES:-hypothetical-protein-[Bacteroidaceae]	171549
WP_007855533.1	PAPS-reductase	GCF_001640865.1	471	Bacteria>FCB_group>Bacteroidetes/Chlorobi_group>Bacteroidetes>Bacteroidia	Bacteroidales	MULTISPECIES:-hypothetical-protein-[Bacteroidales]	171549
WP_021663293.1	PAPS-reductase	GCF_000613725.1	369	Bacteria>FCB_group>Bacteroidetes/Chlorobi_group>Bacteroidetes>Bacteroidia	Bacteroidales	MULTISPECIES:-hypothetical-protein-[Bacteroidales]	171549
WP_022185885.1	PAPS-reductase	GCF_002257605.1	367	Bacteria>FCB_group>Bacteroidetes/Chlorobi_group>Bacteroidetes>Bacteroidia	Bacteroidales	MULTISPECIES:-hypothetical-protein-[Bacteroidales]	171549
WP_227086414.1	PAPS-reductase	GCF_015556515.1	242	Bacteria>FCB_group>Bacteroidetes/Chlorobi_group>Bacteroidetes>Bacteroidia	Bacteroidales	MULTISPECIES:-hypothetical-protein-[Bacteroidales]	171549
WP_094421736.1	PAPS-reductase	GCF_002251455.1	366	Bacteria>FCB_group>Bacteroidetes/Chlorobi_group>Bacteroidetes>Bacteroidia	Bacteroidales	MULTISPECIES:-phosphoadenosine-phosphosulfate-reductase-[Bacteroidales]	171549
WP_027291706.1	PAPS-reductase	GCF_024792195.1	368	Bacteria>FCB_group>Bacteroidetes/Chlorobi_group>Bacteroidetes>Bacteroidia	Bacteroidales	MULTISPECIES:-phosphoadenosine-phosphosulfate-reductase-family-protein-[Bacteroidales]	171549
WP_148372259.1	PAPS-reductase	GCF_902363195.1	444	Bacteria>FCB_group>Bacteroidetes/Chlorobi_group>Bacteroidetes>Bacteroidia	Bacteroides_bouchesdurhonensis	DNA-phosphorothioation-system-sulfurtransferase-DndC-[Bacteroides-bouchesdurhonensis]	1841855
WP_055256756.1	PAPS-reductase	GCF_020091545.1	365	Bacteria>FCB_group>Bacteroidetes/Chlorobi_group>Bacteroidetes>Bacteroidia	Bacteroides_caccae	hypothetical-protein-[Bacteroides-caccae]	47678
WP_217713901.1	PAPS-reductase	GCF_019128795.1	368	Bacteria>FCB_group>Bacteroidetes/Chlorobi_group>Bacteroidetes>Bacteroidia	Bacteroides_cellulosilyticus	phosphoadenosine-phosphosulfate-reductase-family-protein-[Bacteroides-cellulosilyticus]	246787
WP_229116274.1	PAPS-reductase	GCF_015560045.1	366	Bacteria>FCB_group>Bacteroidetes/Chlorobi_group>Bacteroidetes>Bacteroidia	Bacteroides_clarus	phosphoadenosine-phosphosulfate-reductase-[Bacteroides-clarus]	626929
CDA71877.1	PAPS-reductase	GCA_000435315.1	369	Bacteria>FCB_group>Bacteroidetes/Chlorobi_group>Bacteroidetes>Bacteroidia	Bacteroides_coprocola_CAG:162	putative-uncharacterized-protein-[Bacteroides-coprocola-CAG:162]	1263040
WP_225450176.1	PAPS-reductase	GCF_021411755.1	443	Bacteria>FCB_group>Bacteroidetes/Chlorobi_group>Bacteroidetes>Bacteroidia	Bacteroides	DNA-phosphorothioation-system-sulfurtransferase-DndC-[Bacteroides-xylanisolvens]	816
WP_229101912.1	PAPS-reductase	-	406	Bacteria>FCB_group>Bacteroidetes/Chlorobi_group>Bacteroidetes>Bacteroidia	Bacteroides_finegoldii	DNA-phosphorothioation-system-sulfurtransferase-DndC-[Bacteroides-finegoldii]	338188
WP_008772054.1	PAPS-reductase	GCF_023654605.1	448	Bacteria>FCB_group>Bacteroidetes/Chlorobi_group>Bacteroidetes>Bacteroidia	Bacteroides_fragilis	DNA-phosphorothioation-system-sulfurtransferase-DndC-[Bacteroides-fragilis]	817
WP_032571936.1	PAPS-reductase	GCF_013867505.1	448	Bacteria>FCB_group>Bacteroidetes/Chlorobi_group>Bacteroidetes>Bacteroidia	Bacteroides_fragilis	DNA-phosphorothioation-system-sulfurtransferase-DndC-[Bacteroides-fragilis]	817
KAA4738563.1	PAPS-reductase	GCA_008569225.1	367	Bacteria>FCB_group>Bacteroidetes/Chlorobi_group>Bacteroidetes>Bacteroidia	Bacteroides_fragilis	phosphoadenosine-phosphosulfate-reductase-[Bacteroides-fragilis]	817
MCE8916096.1	PAPS-reductase	GCA_021410485.1	367	Bacteria>FCB_group>Bacteroidetes/Chlorobi_group>Bacteroidetes>Bacteroidia	Bacteroides_fragilis	phosphoadenosine-phosphosulfate-reductase-[Bacteroides-fragilis]	817
THC64596.1	PAPS-reductase	GCA_004798515.1	368	Bacteria>FCB_group>Bacteroidetes/Chlorobi_group>Bacteroidetes>Bacteroidia	Bacteroides_fragilis	phosphoadenosine-phosphosulfate-reductase-[Bacteroides-fragilis]	817
WP_032561801.1	PAPS-reductase	GCF_000598645.1	367	Bacteria>FCB_group>Bacteroidetes/Chlorobi_group>Bacteroidetes>Bacteroidia	Bacteroides_fragilis	phosphoadenosine-phosphosulfate-reductase-[Bacteroides-fragilis]	817
WP_227086415.1	PAPS-reductase	GCF_020558865.1	167	Bacteria>FCB_group>Bacteroidetes/Chlorobi_group>Bacteroidetes>Bacteroidia	Bacteroides_fragilis	phosphoadenosine-phosphosulfate-reductase-family-protein-[Bacteroides-fragilis]	817
EXZ41700.1	PAPS-reductase	GCA_000598825.1	448	Bacteria>FCB_group>Bacteroidetes/Chlorobi_group>Bacteroidetes>Bacteroidia	Bacteroides_fragilis_str._2-F-2_4	putative-sulfurtransferase-DndC-[Bacteroides-fragilis-str.-2-F-2-4]	1339280
EXZ74876.1	PAPS-reductase	GCA_000599185.2	448	Bacteria>FCB_group>Bacteroidetes/Chlorobi_group>Bacteroidetes>Bacteroidia	Bacteroides_fragilis_str._3976T8	putative-sulfurtransferase-DndC-[Bacteroides-fragilis-str.-3976T8]	1339314
WP_195546713.1	PAPS-reductase	GCF_015556115.1	443	Bacteria>FCB_group>Bacteroidetes/Chlorobi_group>Bacteroidetes>Bacteroidia	Bacteroides_intestinalis	DNA-phosphorothioation-system-sulfurtransferase-DndC-[Bacteroides-intestinalis]	329854
WP_204432320.1	PAPS-reductase	GCF_016899905.1	369	Bacteria>FCB_group>Bacteroidetes/Chlorobi_group>Bacteroidetes>Bacteroidia	Bacteroides_mediterraneensis	phosphoadenosine-phosphosulfate-reductase-family-protein-[Bacteroides-mediterraneensis]	1841856
WP_007766197.1	PAPS-reductase	GCF_000269545.1	443	Bacteria>FCB_group>Bacteroidetes/Chlorobi_group>Bacteroidetes>Bacteroidia	Bacteroides	MULTISPECIES:-DNA-phosphorothioation-system-sulfurtransferase-DndC-[Bacteroides]	816
WP_010537098.1	PAPS-reductase	GCF_000226135.1	446	Bacteria>FCB_group>Bacteroidetes/Chlorobi_group>Bacteroidetes>Bacteroidia	Bacteroides	MULTISPECIES:-DNA-phosphorothioation-system-sulfurtransferase-DndC-[Bacteroides]	816
WP_125042720.1	PAPS-reductase	GCF_003865075.1	446	Bacteria>FCB_group>Bacteroidetes/Chlorobi_group>Bacteroidetes>Bacteroidia	Bacteroides	MULTISPECIES:-DNA-phosphorothioation-system-sulfurtransferase-DndC-[Bacteroides]	816
WP_156730620.1	PAPS-reductase	GCF_015554205.1	446	Bacteria>FCB_group>Bacteroidetes/Chlorobi_group>Bacteroidetes>Bacteroidia	Bacteroides	MULTISPECIES:-DNA-phosphorothioation-system-sulfurtransferase-DndC-[Bacteroides]	816
WP_217290259.1	PAPS-reductase	GCF_019127935.1	444	Bacteria>FCB_group>Bacteroidetes/Chlorobi_group>Bacteroidetes>Bacteroidia	Bacteroides	MULTISPECIES:-DNA-phosphorothioation-system-sulfurtransferase-DndC-[Bacteroides]	816
WP_004323944.1	PAPS-reductase	GCF_013361195.1	366	Bacteria>FCB_group>Bacteroidetes/Chlorobi_group>Bacteroidetes>Bacteroidia	Bacteroides	MULTISPECIES:-hypothetical-protein-[Bacteroides]	816
WP_128129254.1	PAPS-reductase	GCF_006742345.1	471	Bacteria>FCB_group>Bacteroidetes/Chlorobi_group>Bacteroidetes>Bacteroidia	Bacteroides	MULTISPECIES:-hypothetical-protein-[Bacteroides]	816
WP_195571200.1	PAPS-reductase	GCF_015557365.1	471	Bacteria>FCB_group>Bacteroidetes/Chlorobi_group>Bacteroidetes>Bacteroidia	Bacteroides_nordii	hypothetical-protein-[Bacteroides-nordii]	291645
WP_229102635.1	PAPS-reductase	GCF_015554685.1	446	Bacteria>FCB_group>Bacteroidetes/Chlorobi_group>Bacteroidetes>Bacteroidia	Bacteroides_ovatus	DNA-phosphorothioation-system-sulfurtransferase-DndC-[Bacteroides-ovatus]	28116
WP_195332935.1	PAPS-reductase	GCF_015547585.1	443	Bacteria>FCB_group>Bacteroidetes/Chlorobi_group>Bacteroidetes>Bacteroidia	Bacteroides_salyersiae	DNA-phosphorothioation-system-sulfurtransferase-DndC-[Bacteroides-salyersiae]	291644
WP_211459214.1	PAPS-reductase	GCF_018291945.1	447	Bacteria>FCB_group>Bacteroidetes/Chlorobi_group>Bacteroidetes>Bacteroidia	Bacteroides_salyersiae	DNA-phosphorothioation-system-sulfurtransferase-DndC-[Bacteroides-salyersiae]	291644
WP_217738694.1	PAPS-reductase	GCF_019132055.1	446	Bacteria>FCB_group>Bacteroidetes/Chlorobi_group>Bacteroidetes>Bacteroidia	Bacteroides_salyersiae	DNA-phosphorothioation-system-sulfurtransferase-DndC-[Bacteroides-salyersiae]	291644
EFI05425.1	PAPS-reductase	GCA_000162515.1	367	Bacteria>FCB_group>Bacteroidetes/Chlorobi_group>Bacteroidetes>Bacteroidia	Bacteroides_sp._1_1_14	conserved-hypothetical-protein-[Bacteroides-sp.-1_1_14]	469585
EEY84701.1	PAPS-reductase	GCA_000162175.1	448	Bacteria>FCB_group>Bacteroidetes/Chlorobi_group>Bacteroidetes>Bacteroidia	Bacteroides_sp._2_1_33B	putative-sulfurtransferase-DndC-[Bacteroides-sp.-2_1_33B]	469589
WP_117578944.1	PAPS-reductase	GCF_024759205.1	448	Bacteria>FCB_group>Bacteroidetes/Chlorobi_group>Bacteroidetes>Bacteroidia	Bacteroides_sp._AM23-12	DNA-phosphorothioation-system-sulfurtransferase-DndC-[Bacteroides-sp.-AM23-12]	2292942
MBD5344278.1	PAPS-reductase	GCA_014802225.1	447	Bacteria>FCB_group>Bacteroidetes/Chlorobi_group>Bacteroidetes>Bacteroidia	Bacteroides_sp.	DNA-phosphorothioation-system-sulfurtransferase-DndC-[Bacteroides-sp.]	29523
MBD5363013.1	PAPS-reductase	GCA_014802395.1	447	Bacteria>FCB_group>Bacteroidetes/Chlorobi_group>Bacteroidetes>Bacteroidia	Bacteroides_sp.	DNA-phosphorothioation-system-sulfurtransferase-DndC-[Bacteroides-sp.]	29523
MCD8031129.1	PAPS-reductase	GCA_021202275.1	291	Bacteria>FCB_group>Bacteroidetes/Chlorobi_group>Bacteroidetes>Bacteroidia	Bacteroides_sp.	DNA-phosphorothioation-system-sulfurtransferase-DndC-[Bacteroides-sp.]	29523
MBD5276839.1	PAPS-reductase	GCA_014801485.1	287	Bacteria>FCB_group>Bacteroidetes/Chlorobi_group>Bacteroidetes>Bacteroidia	Bacteroides_sp.	phosphoadenosine-phosphosulfate-reductase-[Bacteroides-sp.]	29523

Accession	Annotation	GCA	Length	Phyletic.spread	Strain.name	Defline	Species.Taxid
MBD5289893.1	PAPS-reductase	GCA_014801515.1	370	Bacteria>FCB_group>Bacteroidetes/Chlorobi_group>Bacteroidetes>Bacteroidia	Bacteroides_sp.	phosphoadenosine-phosphosulfate-reductase-[Bacteroides-sp.]	29523
MBD5358486.1	PAPS-reductase	GCA_014802265.1	370	Bacteria>FCB_group>Bacteroidetes/Chlorobi_group>Bacteroidetes>Bacteroidia	Bacteroides_sp.	phosphoadenosine-phosphosulfate-reductase-[Bacteroides-sp.]	29523
MBD5376837.1	PAPS-reductase	GCA_014802445.1	287	Bacteria>FCB_group>Bacteroidetes/Chlorobi_group>Bacteroidetes>Bacteroidia	Bacteroides_sp.	phosphoadenosine-phosphosulfate-reductase-[Bacteroides-sp.]	29523
MBE6279680.1	PAPS-reductase	GCA_015060785.1	367	Bacteria>FCB_group>Bacteroidetes/Chlorobi_group>Bacteroidetes>Bacteroidia	Bacteroides_sp.	phosphoadenosine-phosphosulfate-reductase-[Bacteroides-sp.]	29523
WP_072067704.1	PAPS-reductase	GCF_001049535.1	446	Bacteria>FCB_group>Bacteroidetes/Chlorobi_group>Bacteroidetes>Bacteroidia	Bacteroides_thetaiotaomicron	DNA-phosphorothioation-system-sulfurtransferase-DndC-[Bacteroides-thetaiotaomicron]	818
WP_196993445.1	PAPS-reductase	GCF_015831785.1	443	Bacteria>FCB_group>Bacteroidetes/Chlorobi_group>Bacteroidetes>Bacteroidia	Bacteroides_thetaiotaomicron	DNA-phosphorothioation-system-sulfurtransferase-DndC-[Bacteroides-thetaiotaomicron]	818
WP_008760268.1	PAPS-reductase	GCF_024758865.1	367	Bacteria>FCB_group>Bacteroidetes/Chlorobi_group>Bacteroidetes>Bacteroidia	Bacteroides_thetaiotaomicron	phosphoadenosine-phosphosulfate-reductase-[Bacteroides-thetaiotaomicron]	818
WP_075318140.1	PAPS-reductase	GCF_900130135.1	446	Bacteria>FCB_group>Bacteroidetes/Chlorobi_group>Bacteroidetes>Bacteroidia	Bacteroides_togonis	DNA-phosphorothioation-system-sulfurtransferase-DndC-[Bacteroides-togonis]	1917883
RGY00484.1	PAPS-reductase	GCA_003463375.1	446	Bacteria>FCB_group>Bacteroidetes/Chlorobi_group>Bacteroidetes>Bacteroidia	Bacteroides_uniformis	DNA-phosphorothioation-system-sulfurtransferase-DndC-[Bacteroides-uniformis]	820
WP_227186521.1	PAPS-reductase	GCF_020558575.1	443	Bacteria>FCB_group>Bacteroidetes/Chlorobi_group>Bacteroidetes>Bacteroidia	Bacteroides_uniformis	DNA-phosphorothioation-system-sulfurtransferase-DndC-[Bacteroides-uniformis]	820
GKH13851.1	PAPS-reductase	GCA_022834995.1	368	Bacteria>FCB_group>Bacteroidetes/Chlorobi_group>Bacteroidetes>Bacteroidia	Bacteroides_uniformis	hypothetical-protein-CE91St12_20610-[Bacteroides-uniformis]	820
WP_151882839.1	PAPS-reductase	GCF_009020755.1	368	Bacteria>FCB_group>Bacteroidetes/Chlorobi_group>Bacteroidetes>Bacteroidia	Bacteroides_uniformis	phosphoadenosine-phosphosulfate-reductase-[Bacteroides-uniformis]	820
WP_244074566.1	PAPS-reductase	GCF_022834995.1	368	Bacteria>FCB_group>Bacteroidetes/Chlorobi_group>Bacteroidetes>Bacteroidia	Bacteroides_uniformis	phosphoadenosine-phosphosulfate-reductase-[Bacteroides-uniformis]	820
WP_234152863.1	PAPS-reductase	GCF_021412865.1	398	Bacteria>FCB_group>Bacteroidetes/Chlorobi_group>Bacteroidetes>Bacteroidia	Bacteroides_xylanisolvens	DNA-phosphorothioation-system-sulfurtransferase-DndC-[Bacteroides-xylanisolvens]	371601
HIP34163.1	PAPS-reductase	GCA_015662855.1	415	Bacteria>FCB_group>Bacteroidetes/Chlorobi_group>Bacteroidetes>Bacteroidia	Bacteroidia_bacterium	DNA-phosphorothioation-system-sulfurtransferase-DndC-[Bacteroidia-bacterium]	2044936
MBI5541385.1	PAPS-reductase	GCA_016218155.1	451	Bacteria>FCB_group>Bacteroidetes/Chlorobi_group>Bacteroidetes>Bacteroidia	Bacteroidia_bacterium	DNA-phosphorothioation-system-sulfurtransferase-DndC-[Bacteroidia-bacterium]	2044936
WP_195491302.1	PAPS-reductase	GCF_015555135.1	446	Bacteria>FCB_group>Bacteroidetes/Chlorobi_group>Bacteroidetes>Bacteroidia	Barnesiella_intestinihominis	DNA-phosphorothioation-system-sulfurtransferase-DndC-[Barnesiella-intestinihominis]	487174
WP_204457528.1	PAPS-reductase	GCF_905219685.1	370	Bacteria>FCB_group>Bacteroidetes/Chlorobi_group>Bacteroidetes>Bacteroidia	Barnesiella_sp._CU968	phosphoadenosine-phosphosulfate-reductase-[Barnesiella-sp.-CU968]	2780099
WP_124317918.1	PAPS-reductase	GCF_003851945.2	451	Bacteria>FCB_group>Bacteroidetes/Chlorobi_group>Bacteroidetes>Bacteroidia	Butyricimonas_faecihominis	DNA-phosphorothioation-system-sulfurtransferase-DndC-[Butyricimonas-faecihominis]	1472416
WP_065220185.1	PAPS-reductase	GCF_902376195.1	443	Bacteria>FCB_group>Bacteroidetes/Chlorobi_group>Bacteroidetes>Bacteroidia	Butyricimonas	MULTISPECIES:-DNA-phosphorothioation-system-sulfurtransferase-DndC-[Butyricimonas]	574697
WP_087420356.1	PAPS-reductase	GCF_002161485.1	443	Bacteria>FCB_group>Bacteroidetes/Chlorobi_group>Bacteroidetes>Bacteroidia	Butyricimonas_sp._An62	DNA-phosphorothioation-system-sulfurtransferase-DndC-[Butyricimonas-sp.-An62]	1965649
HIX20994.1	PAPS-reductase	GCA_019114485.1	471	Bacteria>FCB_group>Bacteroidetes/Chlorobi_group>Bacteroidetes>Bacteroidia	Candidatus_Parabacteroides_faecavium	hypothetical-protein-[Candidatus-Parabacteroides-faecavium]	2838720
MBU3808935.1	PAPS-reductase	GCA_018884025.1	368	Bacteria>FCB_group>Bacteroidetes/Chlorobi_group>Bacteroidetes>Bacteroidia	Candidatus_Phocaeicola_faecipullorum	phosphoadenosine-phosphosulfate-reductase-[Candidatus-Phocaeicola-faecipullorum]	2838733
HIZ05180.1	PAPS-reductase	GCA_019116145.1	443	Bacteria>FCB_group>Bacteroidetes/Chlorobi_group>Bacteroidetes>Bacteroidia	Candidatus_Phocaeicola_gallistercoris	DNA-phosphorothioation-system-sulfurtransferase-DndC-[Candidatus-Phocaeicola-gallistercoris]	2838735
WP_163348020.1	PAPS-reductase	GCF_010448835.1	454	Bacteria>FCB_group>Bacteroidetes/Chlorobi_group>Bacteroidetes>Bacteroidia	Draconibacterium_halophilum	DNA-phosphorothioation-system-sulfurtransferase-DndC-[Draconibacterium-halophilum]	2706887
WP_185144275.1	PAPS-reductase	-	451	Bacteria>FCB_group>Bacteroidetes/Chlorobi_group>Bacteroidetes>Bacteroidia	Dysgonomonas_alginatilytica	DNA-phosphorothioation-system-sulfurtransferase-DndC-[Dysgonomonas-alginatilytica]	1605892
WP_163358252.1	PAPS-reductase	GCF_010501075.1	445	Bacteria>FCB_group>Bacteroidetes/Chlorobi_group>Bacteroidetes>Bacteroidia	Dysgonomonas_sp._25	DNA-phosphorothioation-system-sulfurtransferase-DndC-[Dysgonomonas-sp.-25]	2302933
NDV79934.1	PAPS-reductase	GCA_010501105.1	442	Bacteria>FCB_group>Bacteroidetes/Chlorobi_group>Bacteroidetes>Bacteroidia	Dysgonomonas_sp._511	DNA-phosphorothioation-system-sulfurtransferase-DndC-[Dysgonomonas-sp.-511]	2302930
WP_163273964.1	PAPS-reductase	GCF_010501105.1	442	Bacteria>FCB_group>Bacteroidetes/Chlorobi_group>Bacteroidetes>Bacteroidia	Dysgonomonas_sp._511	DNA-phosphorothioation-system-sulfurtransferase-DndC-[Dysgonomonas-sp.-511]	2302930
WP_194225818.1	PAPS-reductase	GCF_015234155.1	452	Bacteria>FCB_group>Bacteroidetes/Chlorobi_group>Bacteroidetes>Bacteroidia	Dysgonomonas_sp._GY75	DNA-phosphorothioation-system-sulfurtransferase-DndC-[Dysgonomonas-sp.-GY75]	2780419
CEA15225.1	PAPS-reductase	GCA_000953535.1	446	Bacteria>FCB_group>Bacteroidetes/Chlorobi_group>Bacteroidetes>Bacteroidia	Fermentimonas_caenicola	putative-sulfurtransferase-DndC-[Fermentimonas-caenicola]	1562970
GAO29924.1	PAPS-reductase	GCA_000974365.1	367	Bacteria>FCB_group>Bacteroidetes/Chlorobi_group>Bacteroidetes>Bacteroidia	Geofilum_rubicundum_JCM_15548	phosphoadenylyl-sulfate-reductase-[Geofilum-rubicundum-JCM-15548]	1236989
MBE6343695.1	PAPS-reductase	GCA_015061365.1	368	Bacteria>FCB_group>Bacteroidetes/Chlorobi_group>Bacteroidetes>Bacteroidia	Lentimicrobiaceae_bacterium	phosphoadenosine-phosphosulfate-reductase-[Lentimicrobiaceae-bacterium]	2026756
OCW94616.1	PAPS-reductase	GCA_001704385.2	368	Bacteria>FCB_group>Bacteroidetes/Chlorobi_group>Bacteroidetes>Bacteroidia	Macellibacteroides_sp._HH-ZS	phosphoadenosine-phosphosulfate-reductase-[Macellibacteroides-sp.-HH-ZS]	1857568
WP_211196506.1	PAPS-reductase	-	493	Bacteria>FCB_group>Bacteroidetes/Chlorobi_group>Bacteroidetes>Bacteroidia	Marinifilum_sp._JC120	DNA-phosphorothioation-system-sulfurtransferase-DndC-[Marinifilum-sp.-JC120]	2302941
MBI9062562.1	PAPS-reductase	GCA_016342595.1	393	Bacteria>FCB_group>Bacteroidetes/Chlorobi_group>Bacteroidetes>Bacteroidia	Marinilabiliaceae_bacterium	phosphoadenosine-phosphosulfate-reductase-family-protein-[Marinilabiliaceae-bacterium]	2053574
MVZ89007.1	PAPS-reductase	GCA_009768845.1	510	Bacteria>FCB_group>Bacteroidetes/Chlorobi_group>Bacteroidetes>Bacteroidia	Microbacter_sp._ANSKLAB05	DNA-phosphorothioation-system-sulfurtransferase-DndC-[Microbacter-sp.-ANSKLAB05]	2684407
GFI51911.1	PAPS-reductase	GCA_011960975.1	447	Bacteria>FCB_group>Bacteroidetes/Chlorobi_group>Bacteroidetes>Bacteroidia	Muribaculaceae_bacterium	hypothetical-protein-IMSAGC021_00202-[Muribaculaceae-bacterium]	2498093
RXE72919.1	PAPS-reductase	GCA_004102785.1	368	Bacteria>FCB_group>Bacteroidetes/Chlorobi_group>Bacteroidetes>Bacteroidia	Muribaculaceae_bacterium_Isolate-013_(NCI)	phosphoadenosine-phosphosulfate-reductase-[Muribaculaceae-bacterium-Isolate-013-(NCI)]	2489218
ROS84213.1	PAPS-reductase	GCA_003833075.1	370	Bacteria>FCB_group>Bacteroidetes/Chlorobi_group>Bacteroidetes>Bacteroidia	Muribaculaceae_bacterium_Isolate-036_(Harlan)	phosphoadenosine-phosphosulfate-reductase-[Muribaculaceae-bacterium-Isolate-036-(Harlan)]	2486460
ROT09371.1	PAPS-reductase	GCA_003762615.1	839	Bacteria>FCB_group>Bacteroidetes/Chlorobi_group>Bacteroidetes>Bacteroidia	Muribaculaceae_bacterium_Isolate-037_(Harlan)	phosphoadenosine-phosphosulfate-reductase-[Muribaculaceae-bacterium-Isolate-037-(Harlan)]	2486461
ROT09909.1	PAPS-reductase	GCA_003762615.1	368	Bacteria>FCB_group>Bacteroidetes/Chlorobi_group>Bacteroidetes>Bacteroidia	Muribaculaceae_bacterium_Isolate-037_(Harlan)	phosphoadenosine-phosphosulfate-reductase-[Muribaculaceae-bacterium-Isolate-037-(Harlan)]	2486461
ROT13595.1	PAPS-reductase	GCA_003762665.1	445	Bacteria>FCB_group>Bacteroidetes/Chlorobi_group>Bacteroidetes>Bacteroidia	Muribaculaceae_bacterium_Isolate-102_(HZI)	DNA-phosphorothioation-system-sulfurtransferase-DndC-[Muribaculaceae-bacterium-Isolate-102-(HZI)]	2486470
MBO6251941.1	PAPS-reductase	GCA_017635355.1	842	Bacteria>FCB_group>Bacteroidetes/Chlorobi_group>Bacteroidetes>Bacteroidia	Muribaculaceae_bacterium	phosphoadenosine-phosphosulfate-reductase-family-protein-[Muribaculaceae-bacterium]	2498093
MBR6489765.1	PAPS-reductase	GCA_017538045.1	842	Bacteria>FCB_group>Bacteroidetes/Chlorobi_group>Bacteroidetes>Bacteroidia	Muribaculaceae_bacterium	phosphoadenosine-phosphosulfate-reductase-family-protein-[Muribaculaceae-bacterium]	2498093
MBJ2184673.1	PAPS-reductase	GCA_016405265.1	368	Bacteria>FCB_group>Bacteroidetes/Chlorobi_group>Bacteroidetes>Bacteroidia	Muribaculaceae_bacterium	phosphoadenosine-phosphosulfate-reductase-[Muribaculaceae-bacterium]	2498093
MCC8174480.1	PAPS-reductase	GCA_020860925.1	518	Bacteria>FCB_group>Bacteroidetes/Chlorobi_group>Bacteroidetes>Bacteroidia	Odoribacter_sp.	DNA-phosphorothioation-system-sulfurtransferase-DndC-[Odoribacter-sp.]	1965233
WP_195305595.1	PAPS-reductase	GCF_015560605.1	368	Bacteria>FCB_group>Bacteroidetes/Chlorobi_group>Bacteroidetes>Bacteroidia	Odoribacter_splanchnicus	phosphoadenosine-phosphosulfate-reductase-family-protein-[Odoribacter-splanchnicus]	28118
MBO5086229.1	PAPS-reductase	GCA_017622455.1	444	Bacteria>FCB_group>Bacteroidetes/Chlorobi_group>Bacteroidetes>Bacteroidia	Paludibacteraceae_bacterium	DNA-phosphorothioation-system-sulfurtransferase-DndC-[Paludibacteraceae-bacterium]	2748031
MBP8967354.1	PAPS-reductase	GCA_018056365.1	445	Bacteria>FCB_group>Bacteroidetes/Chlorobi_group>Bacteroidetes>Bacteroidia	Paludibacteraceae_bacterium	DNA-phosphorothioation-system-sulfurtransferase-DndC-[Paludibacteraceae-bacterium]	2748031
MBR6105397.1	PAPS-reductase	GCA_017542385.1	368	Bacteria>FCB_group>Bacteroidetes/Chlorobi_group>Bacteroidetes>Bacteroidia	Paludibacteraceae_bacterium	phosphoadenosine-phosphosulfate-reductase-[Paludibacteraceae-bacterium]	2748031
MBR6110692.1	PAPS-reductase	GCA_017541855.1	369	Bacteria>FCB_group>Bacteroidetes/Chlorobi_group>Bacteroidetes>Bacteroidia	Paludibacteraceae_bacterium	phosphoadenosine-phosphosulfate-reductase-[Paludibacteraceae-bacterium]	2748031
WP_122268469.1	PAPS-reductase	GCF_015548725.1	472	Bacteria>FCB_group>Bacteroidetes/Chlorobi_group>Bacteroidetes>Bacteroidia	Parabacteroides_distasonis	hypothetical-protein-[Parabacteroides-distasonis]	823
WP_229099856.1	PAPS-reductase	GCF_015556515.1	196	Bacteria>FCB_group>Bacteroidetes/Chlorobi_group>Bacteroidetes>Bacteroidia	Parabacteroides_distasonis	phosphoadenosine-phosphosulfate-reductase-family-protein-[Parabacteroides-distasonis]	823
MCF2604827.1	PAPS-reductase	GCA_021531385.1	366	Bacteria>FCB_group>Bacteroidetes/Chlorobi_group>Bacteroidetes>Bacteroidia	Parabacteroides_distasonis	phosphoadenosine-phosphosulfate-reductase-[Parabacteroides-distasonis]	823
EKN06313.1	PAPS-reductase	GCA_000307395.1	446	Bacteria>FCB_group>Bacteroidetes/Chlorobi_group>Bacteroidetes>Bacteroidia	Parabacteroides_goldsteinii_CL02T12C30	hypothetical-protein-HMPREF1076_05276-[Parabacteroides-goldsteinii-CL02T12C30]	999418
WP_007659347.1	PAPS-reductase	GCF_000307395.1	446	Bacteria>FCB_group>Bacteroidetes/Chlorobi_group>Bacteroidetes>Bacteroidia	Parabacteroides_goldsteinii	DNA-phosphorothioation-system-sulfurtransferase-DndC-[Parabacteroides-goldsteinii]	328812
WP_234201958.1	PAPS-reductase	GCF_021411685.1	368	Bacteria>FCB_group>Bacteroidetes/Chlorobi_group>Bacteroidetes>Bacteroidia	Parabacteroides_merdae	phosphoadenosine-phosphosulfate-reductase-[Parabacteroides-merdae]	46503
WP_129734879.1	PAPS-reductase	GCF_014647375.1	444	Bacteria>FCB_group>Bacteroidetes/Chlorobi_group>Bacteroidetes>Bacteroidia	Parabacteroides	MULTISPECIES:-DNA-phosphorothioation-system-sulfurtransferase-DndC-[Parabacteroides]	375288
RHR53290.1	PAPS-reductase	GCA_003479205.1	472	Bacteria>FCB_group>Bacteroidetes/Chlorobi_group>Bacteroidetes>Bacteroidia	Parabacteroides_sp._AF17-28	hypothetical-protein-DWW90_16615-[Parabacteroides-sp.-AF17-28]	2292241
WP_122345776.1	PAPS-reductase	GCF_003479205.1	472	Bacteria>FCB_group>Bacteroidetes/Chlorobi_group>Bacteroidetes>Bacteroidia	Parabacteroides_sp._AF17-28	hypothetical-protein-[Parabacteroides-sp.-AF17-28]	2292241
WP_008630105.1	PAPS-reductase	GCF_902374365.1	444	Bacteria>FCB_group>Bacteroidetes/Chlorobi_group>Bacteroidetes>Bacteroidia	Paraprevotella_xylaniphila	DNA-phosphorothioation-system-sulfurtransferase-DndC-[Paraprevotella-xylaniphila]	454155
WP_204482002.1	PAPS-reductase	GCF_016901555.1	446	Bacteria>FCB_group>Bacteroidetes/Chlorobi_group>Bacteroidetes>Bacteroidia	Phocaeicola_coprocola	DNA-phosphorothioation-system-sulfurtransferase-DndC-[Phocaeicola-coprocola]	310298
MBM6671933.1	PAPS-reductase	GCA_016900015.1	367	Bacteria>FCB_group>Bacteroidetes/Chlorobi_group>Bacteroidetes>Bacteroidia	Phocaeicola_coprophilus	phosphoadenosine-phosphosulfate-reductase-[Phocaeicola-coprophilus]	387090
WP_230328777.1	PAPS-reductase	GCF_015670245.1	173	Bacteria>FCB_group>Bacteroidetes/Chlorobi_group>Bacteroidetes>Bacteroidia	Phocaeicola_massiliensis	phosphoadenosine-phosphosulfate-reductase-family-protein-[Phocaeicola-massiliensis]	204516
WP_118166161.1	PAPS-reductase	GCF_003469295.1	369	Bacteria>FCB_group>Bacteroidetes/Chlorobi_group>Bacteroidetes>Bacteroidia	Phocaeicola_plebeius	phosphoadenosine-phosphosulfate-reductase-family-protein-[Phocaeicola-plebeius]	310297
KAB6443624.1	PAPS-reductase	GCA_009102885.1	445	Bacteria>FCB_group>Bacteroidetes/Chlorobi_group>Bacteroidetes>Bacteroidia	Phocaeicola_vulgatus	DNA-phosphorothioation-system-sulfurtransferase-DndC-[Phocaeicola-vulgatus]	821
BDC07561.1	PAPS-reductase	GCA_020885715.1	368	Bacteria>FCB_group>Bacteroidetes/Chlorobi_group>Bacteroidetes>Bacteroidia	Phocaeicola_vulgatus	hypothetical-protein-GAIMETA21S03_34440-[Phocaeicola-vulgatus]	821
KAB3683746.1	PAPS-reductase	GCA_009017905.1	471	Bacteria>FCB_group>Bacteroidetes/Chlorobi_group>Bacteroidetes>Bacteroidia	Phocaeicola_vulgatus	hypothetical-protein-GAT18_00805-[Phocaeicola-vulgatus]	821
WP_229508500.1	PAPS-reductase	GCF_020885715.1	368	Bacteria>FCB_group>Bacteroidetes/Chlorobi_group>Bacteroidetes>Bacteroidia	Phocaeicola_vulgatus	phosphoadenosine-phosphosulfate-reductase-[Phocaeicola-vulgatus]	821
WP_229537762.1	PAPS-reductase	GCF_020827675.1	365	Bacteria>FCB_group>Bacteroidetes/Chlorobi_group>Bacteroidetes>Bacteroidia	Phocaeicola_vulgatus	phosphoadenosine-phosphosulfate-reductase-[Phocaeicola-vulgatus]	821
KDS46036.1	PAPS-reductase	GCA_000699865.1	471	Bacteria>FCB_group>Bacteroidetes/Chlorobi_group>Bacteroidetes>Bacteroidia	Phocaeicola_vulgatus_str._3975_RP4	hypothetical-protein-M099_3751-[Phocaeicola-vulgatus-str.-3975-RP4]	1339352
WP_061157028.1	PAPS-reductase	GCF_021406655.1	368	Bacteria>FCB_group>Bacteroidetes/Chlorobi_group>Bacteroidetes>Bacteroidia	Porphyromonas_gingivalis	hypothetical-protein-[Porphyromonas-gingivalis]	837
WP_238583191.1	PAPS-reductase	GCF_001297745.1	282	Bacteria>FCB_group>Bacteroidetes/Chlorobi_group>Bacteroidetes>Bacteroidia	Porphyromonas_gingivalis	phosphoadenosine-phosphosulfate-reductase,-partial-[Porphyromonas-gingivalis]	837
WP_232724665.1	PAPS-reductase	GCF_000177355.1	445	Bacteria>FCB_group>Bacteroidetes/Chlorobi_group>Bacteroidetes>Bacteroidia	Prevotella_amnii	DNA-phosphorothioation-system-sulfurtransferase-DndC-[Prevotella-amnii]	419005
WP_004335930.1	PAPS-reductase	GCF_000177315.1	446	Bacteria>FCB_group>Bacteroidetes/Chlorobi_group>Bacteroidetes>Bacteroidia	Prevotella_bivia	DNA-phosphorothioation-system-sulfurtransferase-DndC-[Prevotella-bivia]	28125
WP_027449533.1	PAPS-reductase	GCF_000621825.1	368	Bacteria>FCB_group>Bacteroidetes/Chlorobi_group>Bacteroidetes>Bacteroidia	Prevotella_brevis	phosphoadenosine-phosphosulfate-reductase-[Prevotella-brevis]	83231
WP_006281442.1	PAPS-reductase	GCF_000179055.1	367	Bacteria>FCB_group>Bacteroidetes/Chlorobi_group>Bacteroidetes>Bacteroidia	Prevotella_bryantii	hypothetical-protein-[Prevotella-bryantii]	77095
MBF1078917.1	PAPS-reductase	GCA_015256965.1	531	Bacteria>FCB_group>Bacteroidetes/Chlorobi_group>Bacteroidetes>Bacteroidia	Prevotellaceae_bacterium	phosphoadenosine-phosphosulfate-reductase-family-protein-[Prevotellaceae-bacterium]	2049047
MCB9017731.1	PAPS-reductase	GCA_020636675.1	368	Bacteria>FCB_group>Bacteroidetes/Chlorobi_group>Bacteroidetes>Bacteroidia	Prevotellaceae_bacterium	phosphoadenosine-phosphosulfate-reductase-[Prevotellaceae-bacterium]	2049047
WP_217326674.1	PAPS-reductase	-	261	Bacteria>FCB_group>Bacteroidetes/Chlorobi_group>Bacteroidetes>Bacteroidia	Prevotella_copri	phosphoadenosine-phosphosulfate-reductase-family-protein-[Prevotella-copri]	165179
RGS45643.1	PAPS-reductase	GCA_003459305.1	366	Bacteria>FCB_group>Bacteroidetes/Chlorobi_group>Bacteroidetes>Bacteroidia	Prevotella_copri	phosphoadenosine-phosphosulfate-reductase-[Prevotella-copri]	165179
WP_117727068.1	PAPS-reductase	GCF_019127575.1	366	Bacteria>FCB_group>Bacteroidetes/Chlorobi_group>Bacteroidetes>Bacteroidia	Prevotella_copri	phosphoadenosine-phosphosulfate-reductase-[Prevotella-copri]	165179
WP_118086061.1	PAPS-reductase	GCF_003459205.1	366	Bacteria>FCB_group>Bacteroidetes/Chlorobi_group>Bacteroidetes>Bacteroidia	Prevotella_copri	phosphoadenosine-phosphosulfate-reductase-[Prevotella-copri]	165179
WP_203049670.1	PAPS-reductase	GCF_016803255.1	366	Bacteria>FCB_group>Bacteroidetes/Chlorobi_group>Bacteroidetes>Bacteroidia	Prevotella_copri	phosphoadenosine-phosphosulfate-reductase-[Prevotella-copri]	165179
WP_203054982.1	PAPS-reductase	GCF_019249795.1	366	Bacteria>FCB_group>Bacteroidetes/Chlorobi_group>Bacteroidetes>Bacteroidia	Prevotella_copri	phosphoadenosine-phosphosulfate-reductase-[Prevotella-copri]	165179
WP_233401482.1	PAPS-reductase	GCF_021347545.1	366	Bacteria>FCB_group>Bacteroidetes/Chlorobi_group>Bacteroidetes>Bacteroidia	Prevotella_copri	phosphoadenosine-phosphosulfate-reductase-[Prevotella-copri]	165179
WP_218415924.1	PAPS-reductase	GCF_019249755.1	246	Bacteria>FCB_group>Bacteroidetes/Chlorobi_group>Bacteroidetes>Bacteroidia	Prevotella_copri	phosphoadenylyl-sulfate-reductase-[Prevotella-copri]	165179
WP_025002862.1	PAPS-reductase	GCF_000614065.1	367	Bacteria>FCB_group>Bacteroidetes/Chlorobi_group>Bacteroidetes>Bacteroidia	Prevotella_dentasini	phosphoadenosine-phosphosulfate-reductase-[Prevotella-dentasini]	589537
WP_036868671.1	PAPS-reductase	GCF_000759075.1	445	Bacteria>FCB_group>Bacteroidetes/Chlorobi_group>Bacteroidetes>Bacteroidia	Prevotella_histicola	DNA-phosphorothioation-system-sulfurtransferase-DndC-[Prevotella-histicola]	470565
WP_100020435.1	PAPS-reductase	GCF_002763785.1	446	Bacteria>FCB_group>Bacteroidetes/Chlorobi_group>Bacteroidetes>Bacteroidia	Prevotella_intermedia	DNA-phosphorothioation-system-sulfurtransferase-DndC-[Prevotella-intermedia]	28131
WP_097549243.1	PAPS-reductase	GCF_002763535.1	367	Bacteria>FCB_group>Bacteroidetes/Chlorobi_group>Bacteroidetes>Bacteroidia	Prevotella_intermedia	phosphoadenosine-phosphosulfate-reductase-[Prevotella-intermedia]	28131
MBF1430158.1	PAPS-reductase	GCA_015262365.1	450	Bacteria>FCB_group>Bacteroidetes/Chlorobi_group>Bacteroidetes>Bacteroidia	Prevotella_melaninogenica	DNA-phosphorothioation-system-sulfurtransferase-DndC-[Prevotella-melaninogenica]	28132
WP_211812667.1	PAPS-reductase	GCF_018127885.1	446	Bacteria>FCB_group>Bacteroidetes/Chlorobi_group>Bacteroidetes>Bacteroidia	Prevotella_melaninogenica	DNA-phosphorothioation-system-sulfurtransferase-DndC-[Prevotella-melaninogenica]	28132
WP_018919963.1	PAPS-reductase	GCF_000477535.1	369	Bacteria>FCB_group>Bacteroidetes/Chlorobi_group>Bacteroidetes>Bacteroidia	Prevotella	MULTISPECIES:-phosphoadenosine-phosphosulfate-reductase-[Prevotella]	838
WP_004368863.1	PAPS-reductase	GCF_902375085.1	367	Bacteria>FCB_group>Bacteroidetes/Chlorobi_group>Bacteroidetes>Bacteroidia	Prevotella_oralis	hypothetical-protein-[Prevotella-oralis]	28134
WP_181020755.1	PAPS-reductase	-	450	Bacteria>FCB_group>Bacteroidetes/Chlorobi_group>Bacteroidetes>Bacteroidia	Prevotella_ruminicola	DNA-phosphorothioation-system-sulfurtransferase-DndC-[Prevotella-ruminicola]	839
WP_073047781.1	PAPS-reductase	GCF_900112275.1	833	Bacteria>FCB_group>Bacteroidetes/Chlorobi_group>Bacteroidetes>Bacteroidia	Prevotella_ruminicola	phosphoadenosine-phosphosulfate-reductase-family-protein-[Prevotella-ruminicola]	839
WP_013063290.1	PAPS-reductase	GCF_000025925.1	367	Bacteria>FCB_group>Bacteroidetes/Chlorobi_group>Bacteroidetes>Bacteroidia	Prevotella_ruminicola	phosphoadenosine-phosphosulfate-reductase-[Prevotella-ruminicola]	839
WP_009162849.1	PAPS-reductase	GCF_000318195.2	367	Bacteria>FCB_group>Bacteroidetes/Chlorobi_group>Bacteroidetes>Bacteroidia	Prevotella_saccharolytica	hypothetical-protein-[Prevotella-saccharolytica]	633701
WP_094880695.1	PAPS-reductase	GCF_002265625.1	365	Bacteria>FCB_group>Bacteroidetes/Chlorobi_group>Bacteroidetes>Bacteroidia	Prevotella_sp._885	phosphoadenosine-phosphosulfate-reductase-[Prevotella-sp.-885]	2022527
WP_028910791.1	PAPS-reductase	GCF_000424185.1	368	Bacteria>FCB_group>Bacteroidetes/Chlorobi_group>Bacteroidetes>Bacteroidia	Prevotella_sp._AGR2160	phosphoadenosine-phosphosulfate-reductase-[Prevotella-sp.-AGR2160]	1280674
CDB05295.1	PAPS-reductase	GCA_000434975.1	365	Bacteria>FCB_group>Bacteroidetes/Chlorobi_group>Bacteroidetes>Bacteroidia	Prevotella_sp._CAG:520	phosphoadenosine-phosphosulfate-reductase-[Prevotella-sp.-CAG:520]	1262929
CDD19965.1	PAPS-reductase	GCA_000436035.1	366	Bacteria>FCB_group>Bacteroidetes/Chlorobi_group>Bacteroidetes>Bacteroidia	Prevotella_sp._CAG:732	conserved-domain-protein-[Prevotella-sp.-CAG:732]	1262934
MBF1617810.1	PAPS-reductase	GCA_015260405.1	445	Bacteria>FCB_group>Bacteroidetes/Chlorobi_group>Bacteroidetes>Bacteroidia	Prevotella_sp.	DNA-phosphorothioation-system-sulfurtransferase-DndC-[Prevotella-sp.]	59823
MBQ2673648.1	PAPS-reductase	GCA_017406665.1	464	Bacteria>FCB_group>Bacteroidetes/Chlorobi_group>Bacteroidetes>Bacteroidia	Prevotella_sp.	DNA-phosphorothioation-system-sulfurtransferase-DndC-[Prevotella-sp.]	59823
MBQ2951243.1	PAPS-reductase	GCA_017410365.1	445	Bacteria>FCB_group>Bacteroidetes/Chlorobi_group>Bacteroidetes>Bacteroidia	Prevotella_sp.	DNA-phosphorothioation-system-sulfurtransferase-DndC-[Prevotella-sp.]	59823
MBQ3700198.1	PAPS-reductase	GCA_017412705.1	443	Bacteria>FCB_group>Bacteroidetes/Chlorobi_group>Bacteroidetes>Bacteroidia	Prevotella_sp.	DNA-phosphorothioation-system-sulfurtransferase-DndC-[Prevotella-sp.]	59823
MBQ9669624.1	PAPS-reductase	GCA_017449585.1	445	Bacteria>FCB_group>Bacteroidetes/Chlorobi_group>Bacteroidetes>Bacteroidia	Prevotella_sp.	DNA-phosphorothioation-system-sulfurtransferase-DndC-[Prevotella-sp.]	59823
MBR4239068.1	PAPS-reductase	GCA_017541945.1	447	Bacteria>FCB_group>Bacteroidetes/Chlorobi_group>Bacteroidetes>Bacteroidia	Prevotella_sp.	DNA-phosphorothioation-system-sulfurtransferase-DndC-[Prevotella-sp.]	59823
MCD8288454.1	PAPS-reductase	GCA_021204685.1	448	Bacteria>FCB_group>Bacteroidetes/Chlorobi_group>Bacteroidetes>Bacteroidia	Prevotella_sp.	DNA-phosphorothioation-system-sulfurtransferase-DndC-[Prevotella-sp.]	59823
WP_237819035.1	PAPS-reductase	GCF_022024015.1	366	Bacteria>FCB_group>Bacteroidetes/Chlorobi_group>Bacteroidetes>Bacteroidia	Prevotella_sp._E9-3	phosphoadenosine-phosphosulfate-reductase-[Prevotella-sp.-E9-3]	2913621
WP_104368838.1	PAPS-reductase	GCF_002933775.1	446	Bacteria>FCB_group>Bacteroidetes/Chlorobi_group>Bacteroidetes>Bacteroidia	Prevotella_sp._MGM1	DNA-phosphorothioation-system-sulfurtransferase-DndC-[Prevotella-sp.-MGM1]	2033405
WP_125235392.1	PAPS-reductase	GCF_003932845.1	451	Bacteria>FCB_group>Bacteroidetes/Chlorobi_group>Bacteroidetes>Bacteroidia	Prevotella_sp._OH937_COT-195	DNA-phosphorothioation-system-sulfurtransferase-DndC-[Prevotella-sp.-OH937_COT-195]	2491051
WP_107620591.1	PAPS-reductase	GCF_003043945.1	446	Bacteria>FCB_group>Bacteroidetes/Chlorobi_group>Bacteroidetes>Bacteroidia	Prevotella_sp._oral_taxon_376	DNA-phosphorothioation-system-sulfurtransferase-DndC-[Prevotella-sp.-oral-taxon-376]	712466
WP_094382985.1	PAPS-reductase	GCF_002251435.1	366	Bacteria>FCB_group>Bacteroidetes/Chlorobi_group>Bacteroidetes>Bacteroidia	Prevotella_sp._P2-180	phosphoadenosine-phosphosulfate-reductase-[Prevotella-sp.-P2-180]	2024224
WP_094389585.1	PAPS-reductase	GCF_002251345.1	445	Bacteria>FCB_group>Bacteroidetes/Chlorobi_group>Bacteroidetes>Bacteroidia	Prevotella_sp._P4-119	DNA-phosphorothioation-system-sulfurtransferase-DndC-[Prevotella-sp.-P4-119]	2024218
WP_028902858.1	PAPS-reductase	GCF_000619985.1	833	Bacteria>FCB_group>Bacteroidetes/Chlorobi_group>Bacteroidetes>Bacteroidia	Prevotella_sp._P6B4	phosphoadenosine-phosphosulfate-reductase-family-protein-[Prevotella-sp.-P6B4]	1410614
WP_028903764.1	PAPS-reductase	GCF_000619985.1	367	Bacteria>FCB_group>Bacteroidetes/Chlorobi_group>Bacteroidetes>Bacteroidia	Prevotella_sp._P6B4	phosphoadenosine-phosphosulfate-reductase-[Prevotella-sp.-P6B4]	1410614
MBR3078189.1	PAPS-reductase	GCA_017509495.1	833	Bacteria>FCB_group>Bacteroidetes/Chlorobi_group>Bacteroidetes>Bacteroidia	Prevotella_sp.	phosphoadenosine-phosphosulfate-reductase-family-protein-[Prevotella-sp.]	59823
MBD9299269.1	PAPS-reductase	GCA_014848905.1	366	Bacteria>FCB_group>Bacteroidetes/Chlorobi_group>Bacteroidetes>Bacteroidia	Prevotella_sp.	phosphoadenosine-phosphosulfate-reductase-[Prevotella-sp.]	59823
MBE6249405.1	PAPS-reductase	GCA_015060535.1	367	Bacteria>FCB_group>Bacteroidetes/Chlorobi_group>Bacteroidetes>Bacteroidia	Prevotella_sp.	phosphoadenosine-phosphosulfate-reductase-[Prevotella-sp.]	59823
MBO4851538.1	PAPS-reductase	GCA_017619755.1	366	Bacteria>FCB_group>Bacteroidetes/Chlorobi_group>Bacteroidetes>Bacteroidia	Prevotella_sp.	phosphoadenosine-phosphosulfate-reductase-[Prevotella-sp.]	59823
MBP3246938.1	PAPS-reductase	GCA_017934505.1	367	Bacteria>FCB_group>Bacteroidetes/Chlorobi_group>Bacteroidetes>Bacteroidia	Prevotella_sp.	phosphoadenosine-phosphosulfate-reductase-[Prevotella-sp.]	59823
MBR1395056.1	PAPS-reductase	GCA_017471465.1	367	Bacteria>FCB_group>Bacteroidetes/Chlorobi_group>Bacteroidetes>Bacteroidia	Prevotella_sp.	phosphoadenosine-phosphosulfate-reductase-[Prevotella-sp.]	59823

Accession	Annotation	GCA	Length	Phyletic.spread	Strain.name	Defline	Species.Taxid
MBR1654890.1	PAPS-reductase	GCA_017470445.1	367	Bacteria>FCB_group>Bacteroidetes/Chlorobi_group>Bacteroidetes>Bacteroidia	Prevotella_sp.	phosphoadenosine-phosphosulfate-reductase-[Prevotella-sp.]	59823
MBR2202897.1	PAPS-reductase	GCA_017478025.1	366	Bacteria>FCB_group>Bacteroidetes/Chlorobi_group>Bacteroidetes>Bacteroidia	Prevotella_sp.	phosphoadenosine-phosphosulfate-reductase-[Prevotella-sp.]	59823
MBR6591988.1	PAPS-reductase	GCA_017521045.1	368	Bacteria>FCB_group>Bacteroidetes/Chlorobi_group>Bacteroidetes>Bacteroidia	Prevotella_sp.	phosphoadenosine-phosphosulfate-reductase-[Prevotella-sp.]	59823
HAH76809.1	PAPS-reductase	GCA_003450395.1	365	Bacteria>FCB_group>Bacteroidetes/Chlorobi_group>Bacteroidetes>Bacteroidia	Prevotella_stercorea	phosphoadenosine-phosphosulfate-reductase-[Prevotella-stercorea]	363265
MBN1769435.1	PAPS-reductase	GCA_016930365.1	448	Bacteria>FCB_group>Bacteroidetes/Chlorobi_group>Bacteroidetes>Bacteroidia	Prolixibacteraceae_bacterium	DNA-phosphorothioation-system-sulfurtransferase-DndC-[Prolixibacteraceae-bacterium]	2053594
MBP7508130.1	PAPS-reductase	GCA_017998095.1	466	Bacteria>FCB_group>Bacteroidetes/Chlorobi_group>Bacteroidetes>Bacteroidia	Prolixibacteraceae_bacterium	DNA-phosphorothioation-system-sulfurtransferase-DndC-[Prolixibacteraceae-bacterium]	2053594
MBW8334339.1	PAPS-reductase	GCA_019459485.1	444	Bacteria>FCB_group>Bacteroidetes/Chlorobi_group>Bacteroidetes>Bacteroidia	Prolixibacteraceae_bacterium	DNA-phosphorothioation-system-sulfurtransferase-DndC-[Prolixibacteraceae-bacterium]	2053594
MBP7508519.1	PAPS-reductase	GCA_017998095.1	551	Bacteria>FCB_group>Bacteroidetes/Chlorobi_group>Bacteroidetes>Bacteroidia	Prolixibacteraceae_bacterium	phosphoadenosine-phosphosulfate-reductase-family-protein-[Prolixibacteraceae-bacterium]	2053594
MBP7508680.1	PAPS-reductase	GCA_017998095.1	573	Bacteria>FCB_group>Bacteroidetes/Chlorobi_group>Bacteroidetes>Bacteroidia	Prolixibacteraceae_bacterium	phosphoadenosine-phosphosulfate-reductase-family-protein-[Prolixibacteraceae-bacterium]	2053594
QZE15892.1	PAPS-reductase	GCA_019720755.1	389	Bacteria>FCB_group>Bacteroidetes/Chlorobi_group>Bacteroidetes>Bacteroidia	Prolixibacteraceae_bacterium	phosphoadenosine-phosphosulfate-reductase-family-protein-[Prolixibacteraceae-bacterium]	2053594
MBZ4652251.1	PAPS-reductase	GCA_020054905.1	366	Bacteria>FCB_group>Bacteroidetes/Chlorobi_group>Bacteroidetes>Bacteroidia	Proteiniphilum_sp.	sulfurtransferase-DndC-[Proteiniphilum-sp.]	1926877
MBS1323838.1	PAPS-reductase	GCA_018266035.1	444	Bacteria>FCB_group>Bacteroidetes/Chlorobi_group>Bacteroidetes>Bacteroidia	Rikenellaceae_bacterium	DNA-phosphorothioation-system-sulfurtransferase-DndC-[Rikenellaceae-bacterium]	2049048
SUE34431.1	PAPS-reductase	GCA_900455755.1	368	Bacteria>FCB_group>Bacteroidetes/Chlorobi_group>Bacteroidetes>Bacteroidia	Rikenella_microfusus	PUA-domain-(predicted-RNA-binding-domain)-[Rikenella-microfusus]	28139
MBR5167612.1	PAPS-reductase	GCA_017557605.1	429	Bacteria>FCB_group>Bacteroidetes/Chlorobi_group>Bacteroidetes>Bacteroidia	Salinivirgaceae_bacterium	DNA-phosphorothioation-system-sulfurtransferase-DndC-[Salinivirgaceae-bacterium]	2053305
MBR2196508.1	PAPS-reductase	GCA_017474765.1	367	Bacteria>FCB_group>Bacteroidetes/Chlorobi_group>Bacteroidetes>Bacteroidia	Salinivirgaceae_bacterium	phosphoadenosine-phosphosulfate-reductase-[Salinivirgaceae-bacterium]	2053305
MBR6082080.1	PAPS-reductase	GCA_017542785.1	367	Bacteria>FCB_group>Bacteroidetes/Chlorobi_group>Bacteroidetes>Bacteroidia	Salinivirgaceae_bacterium	phosphoadenosine-phosphosulfate-reductase-[Salinivirgaceae-bacterium]	2053305
MCD7932572.1	PAPS-reductase	GCA_021201455.1	242	Bacteria>FCB_group>Bacteroidetes/Chlorobi_group>Bacteroidetes>Bacteroidia	Tannerellaceae_bacterium	phosphoadenosine-phosphosulfate-reductase-family-protein-[Tannerellaceae-bacterium]	2785930
WP_075588644.1	PAPS-reductase	GCF_001938785.1	446	Bacteria>FCB_group>Bacteroidetes/Chlorobi_group>Bacteroidetes>Bacteroidia	Tannerella_forsythia	DNA-phosphorothioation-system-sulfurtransferase-DndC-[Tannerella-forsythia]	28112
WP_060828468.1	PAPS-reductase	GCF_001547875.1	368	Bacteria>FCB_group>Bacteroidetes/Chlorobi_group>Bacteroidetes>Bacteroidia	Tannerella_forsythia	phosphoadenosine-phosphosulfate-reductase-[Tannerella-forsythia]	28112
WP_087421099.1	PAPS-reductase	GCF_015560935.1	444	Bacteria>FCB_group>Bacteroidetes/Chlorobi_group>Bacteroidetes>Bacteroidia	unclassified_Butyricimonas	MULTISPECIES:-DNA-phosphorothioation-system-sulfurtransferase-DndC-[unclassified-Butyricimonas]	2637652
WP_136408919.1	PAPS-reductase	GCF_004803935.1	447	Bacteria>FCB_group>Bacteroidetes/Chlorobi_group>Bacteroidetes>Bacteroidia	unclassified_Duncaniella	MULTISPECIES:-DNA-phosphorothioation-system-sulfurtransferase-DndC-[unclassified-Duncaniella]	2649562
WP_172178624.1	PAPS-reductase	GCF_013166575.1	446	Bacteria>FCB_group>Bacteroidetes/Chlorobi_group>Bacteroidetes>Bacteroidia	unclassified_Prevotella	MULTISPECIES:-DNA-phosphorothioation-system-sulfurtransferase-DndC-[unclassified-Prevotella]	2638335
VBB44798.1	PAPS-reductase	GCA_900498215.1	443	Bacteria>FCB_group>Bacteroidetes/Chlorobi_group>Bacteroidetes>Bacteroidia	uncultured_Paludibacter_sp.	putative-sulfurtransferase-DndC-[uncultured-Paludibacter-sp.]	497635
MBK6483957.1	PAPS-reductase	GCA_016704325.1	367	Bacteria>FCB_group>Bacteroidetes/Chlorobi_group>Bacteroidetes>Chitinophagia	Chitinophagaceae_bacterium	phosphoadenosine-phosphosulfate-reductase-family-protein-[Chitinophagaceae-bacterium]	1869212
MBK8609278.1	PAPS-reductase	GCA_016710745.1	368	Bacteria>FCB_group>Bacteroidetes/Chlorobi_group>Bacteroidetes>Chitinophagia	Chitinophagaceae_bacterium	phosphoadenosine-phosphosulfate-reductase-family-protein-[Chitinophagaceae-bacterium]	1869212
WP_078669518.1	PAPS-reductase	GCF_900167135.1	439	Bacteria>FCB_group>Bacteroidetes/Chlorobi_group>Bacteroidetes>Chitinophagia	Chitinophaga_eiseniae	DNA-phosphorothioation-system-sulfurtransferase-DndC-[Chitinophaga-eiseniae]	634771
MBX7224970.1	PAPS-reductase	GCA_019694955.1	367	Bacteria>FCB_group>Bacteroidetes/Chlorobi_group>Bacteroidetes>Chitinophagia	Chitinophagales_bacterium	phosphoadenosine-phosphosulfate-reductase-family-protein-[Chitinophagales-bacterium]	2448779
NOT92471.1	PAPS-reductase	GCA_013141115.1	367	Bacteria>FCB_group>Bacteroidetes/Chlorobi_group>Bacteroidetes>Chitinophagia	Ferruginibacter_sp.	phosphoadenosine-phosphosulfate-reductase-family-protein-[Ferruginibacter-sp.]	1940288
NOT93842.1	PAPS-reductase	GCA_013141115.1	368	Bacteria>FCB_group>Bacteroidetes/Chlorobi_group>Bacteroidetes>Chitinophagia	Ferruginibacter_sp.	phosphoadenosine-phosphosulfate-reductase-family-protein-[Ferruginibacter-sp.]	1940288
WP_076378670.1	PAPS-reductase	GCF_002355595.1	368	Bacteria>FCB_group>Bacteroidetes/Chlorobi_group>Bacteroidetes>Chitinophagia	Filimonas_lacunae	phosphoadenosine-phosphosulfate-reductase-family-protein-[Filimonas-lacunae]	477680
WP_131850663.1	PAPS-reductase	GCF_004343705.1	423	Bacteria>FCB_group>Bacteroidetes/Chlorobi_group>Bacteroidetes>Chitinophagia	Flaviaesturariibacter_aridisoli	DNA-phosphorothioation-system-sulfurtransferase-DndC-[Flaviaesturariibacter-aridisoli]	2545761
MBL7682029.1	PAPS-reductase	GCA_016786615.1	437	Bacteria>FCB_group>Bacteroidetes/Chlorobi_group>Bacteroidetes>Chitinophagia	Flavipsychrobacter_sp.	DNA-phosphorothioation-system-sulfurtransferase-DndC-[Flavipsychrobacter-sp.]	2307286
MBL7689988.1	PAPS-reductase	GCA_016786565.1	425	Bacteria>FCB_group>Bacteroidetes/Chlorobi_group>Bacteroidetes>Chitinophagia	Flavipsychrobacter_sp.	DNA-phosphorothioation-system-sulfurtransferase-DndC-[Flavipsychrobacter-sp.]	2307286
NTS40781.1	PAPS-reductase	GCA_013327885.1	368	Bacteria>FCB_group>Bacteroidetes/Chlorobi_group>Bacteroidetes>Chitinophagia	Flavisolibacter_longurius	phosphoadenosine-phosphosulfate-reductase-family-protein-[Flavisolibacter-longurius]	2740583
WP_090657717.1	PAPS-reductase	GCF_900115755.1	440	Bacteria>FCB_group>Bacteroidetes/Chlorobi_group>Bacteroidetes>Chitinophagia	Parafilimonas_terrae	DNA-phosphorothioation-system-sulfurtransferase-DndC-[Parafilimonas-terrae]	1465490
WP_026765131.1	PAPS-reductase	GCF_000511175.1	367	Bacteria>FCB_group>Bacteroidetes/Chlorobi_group>Bacteroidetes>Chitinophagia	Sediminibacterium_salmoneum	phosphoadenosine-phosphosulfate-reductase-family-protein-[Sediminibacterium-salmoneum]	426421
WP_106522856.1	PAPS-reductase	GCF_003014535.1	447	Bacteria>FCB_group>Bacteroidetes/Chlorobi_group>Bacteroidetes>Chitinophagia	Taibaiella_chishuiensis	DNA-phosphorothioation-system-sulfurtransferase-DndC-[Taibaiella-chishuiensis]	1434707
PZX54034.1	PAPS-reductase	GCA_003254055.1	374	Bacteria>FCB_group>Bacteroidetes/Chlorobi_group>Bacteroidetes>Cytophagia	Algoriphagus_chordae	phosphoadenosine-phosphosulfate-reductase-family-protein-[Algoriphagus-chordae]	237019
WP_111317452.1	PAPS-reductase	GCF_003254055.1	374	Bacteria>FCB_group>Bacteroidetes/Chlorobi_group>Bacteroidetes>Cytophagia	Algoriphagus_chordae	phosphoadenosine-phosphosulfate-reductase-family-protein-[Algoriphagus-chordae]	237019
WP_111672685.1	PAPS-reductase	GCF_003259255.1	440	Bacteria>FCB_group>Bacteroidetes/Chlorobi_group>Bacteroidetes>Cytophagia	Algoriphagus_litoralis	DNA-phosphorothioation-system-sulfurtransferase-DndC-[Algoriphagus-litoralis]	2202829
WP_113923488.1	PAPS-reductase	GCF_003316845.1	367	Bacteria>FCB_group>Bacteroidetes/Chlorobi_group>Bacteroidetes>Cytophagia	Aquiflexum_aquatile	phosphoadenosine-phosphosulfate-reductase-family-protein-[Aquiflexum-aquatile]	2249427
WP_109745132.1	PAPS-reductase	GCF_003148625.1	457	Bacteria>FCB_group>Bacteroidetes/Chlorobi_group>Bacteroidetes>Cytophagia	Arcicella_aurantiaca	DNA-phosphorothioation-system-sulfurtransferase-DndC-[Arcicella-aurantiaca]	591202
MBC7408610.1	PAPS-reductase	GCA_014376355.1	442	Bacteria>FCB_group>Bacteroidetes/Chlorobi_group>Bacteroidetes>Cytophagia	Arcicella_sp.	DNA-phosphorothioation-system-sulfurtransferase-DndC-[Arcicella-sp.]	1872628
WP_132121434.1	PAPS-reductase	GCF_004348825.1	445	Bacteria>FCB_group>Bacteroidetes/Chlorobi_group>Bacteroidetes>Cytophagia	Arundinibacter_roseus	DNA-phosphorothioation-system-sulfurtransferase-DndC-[Arundinibacter-roseus]	2070510
WP_014771610.1	PAPS-reductase	GCF_000265405.1	431	Bacteria>FCB_group>Bacteroidetes/Chlorobi_group>Bacteroidetes>Cytophagia	Belliella_baltica	DNA-phosphorothioation-system-sulfurtransferase-DndC-[Belliella-baltica]	232259
PGH40558.1	PAPS-reductase	GCA_002573685.1	368	Bacteria>FCB_group>Bacteroidetes/Chlorobi_group>Bacteroidetes>Cytophagia	Candidatus_Nephrothrix_sp._EaCA	phosphoadenosine-phosphosulfate-reductase-[Candidatus-Nephrothrix-sp.-EaCA]	2044729
WP_119755251.1	PAPS-reductase	GCF_003589925.1	367	Bacteria>FCB_group>Bacteroidetes/Chlorobi_group>Bacteroidetes>Cytophagia	Chryseolinea_soli	phosphoadenosine-phosphosulfate-reductase-family-protein-[Chryseolinea-soli]	2321403
WP_127123873.1	PAPS-reductase	GCF_003990915.1	367	Bacteria>FCB_group>Bacteroidetes/Chlorobi_group>Bacteroidetes>Cytophagia	Chryseotalea_sanaruensis	phosphoadenosine-phosphosulfate-reductase-family-protein-[Chryseotalea-sanaruensis]	2482724
NJN26653.1	PAPS-reductase	GCA_012033595.1	442	Bacteria>FCB_group>Bacteroidetes/Chlorobi_group>Bacteroidetes>Cytophagia	Cyclobacteriaceae_bacterium	DNA-phosphorothioation-system-sulfurtransferase-DndC-[Cyclobacteriaceae-bacterium]	2099675
MBX7090400.1	PAPS-reductase	GCA_019694875.1	368	Bacteria>FCB_group>Bacteroidetes/Chlorobi_group>Bacteroidetes>Cytophagia	Cyclobacteriaceae_bacterium	phosphoadenosine-phosphosulfate-reductase-family-protein-[Cyclobacteriaceae-bacterium]	2099675
WP_166144210.1	PAPS-reductase	GCF_011290685.1	367	Bacteria>FCB_group>Bacteroidetes/Chlorobi_group>Bacteroidetes>Cytophagia	Cyclobacterium_plantarum	phosphoadenosine-phosphosulfate-reductase-family-protein-[Cyclobacterium-plantarum]	2716263
MBI1231179.1	PAPS-reductase	GCA_016124425.1	439	Bacteria>FCB_group>Bacteroidetes/Chlorobi_group>Bacteroidetes>Cytophagia	Cytophagales_bacterium	DNA-phosphorothioation-system-sulfurtransferase-DndC-[Cytophagales-bacterium]	2053541
TAG40986.1	PAPS-reductase	GCA_004293645.1	441	Bacteria>FCB_group>Bacteroidetes/Chlorobi_group>Bacteroidetes>Cytophagia	Cytophagia_bacterium	DNA-phosphorothioation-system-sulfurtransferase-DndC-[Cytophagia-bacterium]	2026730
MBT1712318.1	PAPS-reductase	GCA_018598175.1	366	Bacteria>FCB_group>Bacteroidetes/Chlorobi_group>Bacteroidetes>Cytophagia	Dawidia_cretensis	phosphoadenosine-phosphosulfate-reductase-family-protein-[Fulvivirgaceae-bacterium-PWU5]	2782350
WP_115833040.1	PAPS-reductase	GCF_003383615.1	367	Bacteria>FCB_group>Bacteroidetes/Chlorobi_group>Bacteroidetes>Cytophagia	Dyadobacter_luteus	phosphoadenosine-phosphosulfate-reductase-family-protein-[Dyadobacter-luteus]	2259619
WP_138365725.1	PAPS-reductase	GCF_005860805.1	368	Bacteria>FCB_group>Bacteroidetes/Chlorobi_group>Bacteroidetes>Cytophagia	Dyadobacter_luticola	phosphoadenosine-phosphosulfate-reductase-family-protein-[Dyadobacter-luticola]	1979387
WP_149639895.1	PAPS-reductase	GCF_008369915.1	447	Bacteria>FCB_group>Bacteroidetes/Chlorobi_group>Bacteroidetes>Cytophagia	Dyadobacter_sp._UC_10	DNA-phosphorothioation-system-sulfurtransferase-DndC-[Dyadobacter-sp.-UC-10]	2605428
WP_215222602.1	PAPS-reductase	GCF_018642165.1	435	Bacteria>FCB_group>Bacteroidetes/Chlorobi_group>Bacteroidetes>Cytophagia	Echinicola_shivajiensis	DNA-phosphorothioation-system-sulfurtransferase-DndC-[Echinicola-shivajiensis]	1035916
AFK04124.1	PAPS-reductase	GCA_000263195.1	372	Bacteria>FCB_group>Bacteroidetes/Chlorobi_group>Bacteroidetes>Cytophagia	Emticicia_oligotrophica_DSM_17448	phosphoadenosine-phosphosulfate-reductase-[Emticicia-oligotrophica-DSM-17448]	929562
WP_207337247.1	PAPS-reductase	GCF_017353255.1	372	Bacteria>FCB_group>Bacteroidetes/Chlorobi_group>Bacteroidetes>Cytophagia	Fibrella_aquatilis	phosphoadenosine-phosphosulfate-reductase-family-protein-[Fibrella-sp.-HMF5036]	2817059
MAE83941.1	PAPS-reductase	GCA_002687205.1	379	Bacteria>FCB_group>Bacteroidetes/Chlorobi_group>Bacteroidetes>Cytophagia	Flammeovirgaceae_bacterium	phosphoadenosine-phosphosulfate-reductase-[Flammeovirgaceae-bacterium]	2026740
WP_091516211.1	PAPS-reductase	GCF_900112255.1	368	Bacteria>FCB_group>Bacteroidetes/Chlorobi_group>Bacteroidetes>Cytophagia	Flexibacter_flexilis	phosphoadenosine-phosphosulfate-reductase-family-protein-[Flexibacter-flexilis]	998
WP_143401568.1	PAPS-reductase	GCF_007097395.1	440	Bacteria>FCB_group>Bacteroidetes/Chlorobi_group>Bacteroidetes>Cytophagia	Fulvivirga_sp._M361	DNA-phosphorothioation-system-sulfurtransferase-DndC-[Fulvivirga-sp.-M361]	2594266
WP_135431164.1	PAPS-reductase	GCF_004745825.1	451	Bacteria>FCB_group>Bacteroidetes/Chlorobi_group>Bacteroidetes>Cytophagia	Hymenobacter_fodinae	DNA-phosphorothioation-system-sulfurtransferase-DndC-[Hymenobacter-sp.-92R-1]	2510796
WP_206986788.1	PAPS-reductase	GCF_017313265.1	462	Bacteria>FCB_group>Bacteroidetes/Chlorobi_group>Bacteroidetes>Cytophagia	Hymenobacter	MULTISPECIES:-DNA-phosphorothioation-system-sulfurtransferase-DndC-[Hymenobacter]	89966
WP_208178319.1	PAPS-reductase	GCF_017571495.1	453	Bacteria>FCB_group>Bacteroidetes/Chlorobi_group>Bacteroidetes>Cytophagia	Hymenobacter_negativus	DNA-phosphorothioation-system-sulfurtransferase-DndC-[Hymenobacter-negativus]	2795026
WP_119654946.1	PAPS-reductase	GCF_003583925.1	367	Bacteria>FCB_group>Bacteroidetes/Chlorobi_group>Bacteroidetes>Cytophagia	Hymenobacter_rubripertinctus	phosphoadenosine-phosphosulfate-reductase-family-protein-[Hymenobacter-rubripertinctus]	2029981
WP_068195607.1	PAPS-reductase	GCF_001507645.1	367	Bacteria>FCB_group>Bacteroidetes/Chlorobi_group>Bacteroidetes>Cytophagia	Hymenobacter_sedentarius	phosphoadenosine-phosphosulfate-reductase-family-protein-[Hymenobacter-sedentarius]	1411621
WP_068339787.1	PAPS-reductase	GCF_001596155.1	442	Bacteria>FCB_group>Bacteroidetes/Chlorobi_group>Bacteroidetes>Cytophagia	Hymenobacter_sp._PAMC_26554	DNA-phosphorothioation-system-sulfurtransferase-DndC-[Hymenobacter-sp.-PAMC-26554]	1484116
EON76972.1	PAPS-reductase	GCA_000390185.1	439	Bacteria>FCB_group>Bacteroidetes/Chlorobi_group>Bacteroidetes>Cytophagia	Lunatimonas_lonarensis	3’-phosphoadenosine-5’-phosphosulfate-sulfurtransferase-DndC-[Lunatimonas-lonarensis]	1232681
WP_010854651.1	PAPS-reductase	GCF_000390185.1	439	Bacteria>FCB_group>Bacteroidetes/Chlorobi_group>Bacteroidetes>Cytophagia	Lunatimonas_lonarensis	DNA-phosphorothioation-system-sulfurtransferase-DndC-[Lunatimonas-lonarensis]	1232681
WP_045113326.1	PAPS-reductase	GCF_000169175.1	468	Bacteria>FCB_group>Bacteroidetes/Chlorobi_group>Bacteroidetes>Cytophagia	Microscilla_marina	DNA-phosphorothioation-system-sulfurtransferase-DndC-[Microscilla-marina]	1027
WP_053337438.1	PAPS-reductase	GCF_000169175.1	468	Bacteria>FCB_group>Bacteroidetes/Chlorobi_group>Bacteroidetes>Cytophagia	Microscilla_marina	DNA-phosphorothioation-system-sulfurtransferase-DndC-[Microscilla-marina]	1027
WP_187065420.1	PAPS-reductase	GCF_014306105.1	445	Bacteria>FCB_group>Bacteroidetes/Chlorobi_group>Bacteroidetes>Cytophagia	Pontibacter_cellulosilyticus	DNA-phosphorothioation-system-sulfurtransferase-DndC-[Pontibacter-sp.-SD6]	1720253
WP_076666401.1	PAPS-reductase	GCF_900156345.1	445	Bacteria>FCB_group>Bacteroidetes/Chlorobi_group>Bacteroidetes>Cytophagia	Pontibacter_indicus	DNA-phosphorothioation-system-sulfurtransferase-DndC-[Pontibacter-indicus]	1317125
WP_229957491.1	PAPS-reductase	GCF_020907275.1	366	Bacteria>FCB_group>Bacteroidetes/Chlorobi_group>Bacteroidetes>Cytophagia	Pontibacter_silvestris	phosphoadenosine-phosphosulfate-reductase-family-protein-[Pontibacter-silvestris]	2305183
QHT72092.1	PAPS-reductase	GCA_010119975.1	431	Bacteria>FCB_group>Bacteroidetes/Chlorobi_group>Bacteroidetes>Cytophagia	Rhodocytophaga_rosea	DNA-phosphorothioation-system-sulfurtransferase-DndC-[Rhodocytophaga-rosea]	2704465
WP_232064505.1	PAPS-reductase	GCF_010119975.1	458	Bacteria>FCB_group>Bacteroidetes/Chlorobi_group>Bacteroidetes>Cytophagia	Rhodocytophaga_rosea	DNA-phosphorothioation-system-sulfurtransferase-DndC-[Rhodocytophaga-rosea]	2704465
WP_062588827.1	PAPS-reductase	GCF_001593005.1	435	Bacteria>FCB_group>Bacteroidetes/Chlorobi_group>Bacteroidetes>Cytophagia	Roseivirga_ehrenbergii	DNA-phosphorothioation-system-sulfurtransferase-DndC-[Roseivirga-ehrenbergii]	279360
WP_069843540.1	PAPS-reductase	GCF_001747095.1	438	Bacteria>FCB_group>Bacteroidetes/Chlorobi_group>Bacteroidetes>Cytophagia	Roseivirga_sp._4D4	DNA-phosphorothioation-system-sulfurtransferase-DndC-[Roseivirga-sp.-4D4]	1889784
WP_181308783.1	PAPS-reductase	GCF_013623775.1	368	Bacteria>FCB_group>Bacteroidetes/Chlorobi_group>Bacteroidetes>Cytophagia	Rufibacter_sp._XAAS-G3-1	phosphoadenosine-phosphosulfate-reductase-family-protein-[Rufibacter-sp.-XAAS-G3-1]	2729134
WP_122933605.1	PAPS-reductase	GCF_003711225.1	368	Bacteria>FCB_group>Bacteroidetes/Chlorobi_group>Bacteroidetes>Cytophagia	Runella_sp._SP2	phosphoadenosine-phosphosulfate-reductase-family-protein-[Runella-sp.-SP2]	2268026
MBO2012584.1	PAPS-reductase	GCA_017571495.1	453	Bacteria>FCB_group>Bacteroidetes/Chlorobi_group>Bacteroidetes>Cytophagia	Siccationidurans_soli	DNA-phosphorothioation-system-sulfurtransferase-DndC-[Siccationidurans-soli]	373890
WP_097126503.1	PAPS-reductase	GCF_900230225.1	446	Bacteria>FCB_group>Bacteroidetes/Chlorobi_group>Bacteroidetes>Cytophagia	Spirosoma_fluviale	DNA-phosphorothioation-system-sulfurtransferase-DndC-[Spirosoma-fluviale]	1597977
WP_020597293.1	PAPS-reductase	GCF_000374025.1	447	Bacteria>FCB_group>Bacteroidetes/Chlorobi_group>Bacteroidetes>Cytophagia	Spirosoma_panaciterrae	DNA-phosphorothioation-system-sulfurtransferase-DndC-[Spirosoma-panaciterrae]	496058
WP_171738710.1	PAPS-reductase	GCF_013127955.1	446	Bacteria>FCB_group>Bacteroidetes/Chlorobi_group>Bacteroidetes>Cytophagia	Spirosoma_taeanense	DNA-phosphorothioation-system-sulfurtransferase-DndC-[Spirosoma-taeanense]	2735870
WP_186738170.1	PAPS-reductase	GCF_014282275.1	446	Bacteria>FCB_group>Bacteroidetes/Chlorobi_group>Bacteroidetes>Cytophagia	Spirosoma_utsteinense	DNA-phosphorothioation-system-sulfurtransferase-DndC-[Spirosoma-utsteinense]	2585773
WP_166918548.1	PAPS-reductase	GCF_011761635.1	446	Bacteria>FCB_group>Bacteroidetes/Chlorobi_group>Bacteroidetes>Cytophagia	Thermonema_lapsum	DNA-phosphorothioation-system-sulfurtransferase-DndC-[Thermonema-lapsum]	28195
WP_051633173.1	PAPS-reductase	GCF_000426825.1	449	Bacteria>FCB_group>Bacteroidetes/Chlorobi_group>Bacteroidetes>Cytophagia	Thermonema_rossianum	DNA-phosphorothioation-system-sulfurtransferase-DndC-[Thermonema-rossianum]	55505
WP_202335806.1	PAPS-reductase	GCF_016757735.1	367	Bacteria>FCB_group>Bacteroidetes/Chlorobi_group>Bacteroidetes>Flavobacteriia	Aequorivita	MULTISPECIES:-phosphoadenosine-phosphosulfate-reductase-family-protein-[unclassified-Aequorivita]	153265
WP_218937250.1	PAPS-reductase	GCF_006346335.1	455	Bacteria>FCB_group>Bacteroidetes/Chlorobi_group>Bacteroidetes>Flavobacteriia	Aequorivita_sinensis	DNA-phosphorothioation-system-sulfurtransferase-DndC-[Aequorivita-sinensis]	1382458
PHR11297.1	PAPS-reductase	GCA_002733915.1	442	Bacteria>FCB_group>Bacteroidetes/Chlorobi_group>Bacteroidetes>Flavobacteriia	Aequorivita_sp.	DNA-phosphorothioation-system-sulfurtransferase-DndC-[Aequorivita-sp.]	1872390
WP_105247685.1	PAPS-reductase	GCF_002964975.1	447	Bacteria>FCB_group>Bacteroidetes/Chlorobi_group>Bacteroidetes>Flavobacteriia	Apibacter_adventoris	DNA-phosphorothioation-system-sulfurtransferase-DndC-[Apibacter-adventoris]	1679466
MBQ0737611.1	PAPS-reductase	GCA_018066725.1	452	Bacteria>FCB_group>Bacteroidetes/Chlorobi_group>Bacteroidetes>Flavobacteriia	Aquimarina_celericrescens	DNA-phosphorothioation-system-sulfurtransferase-DndC-[Aquimarina-celericrescens]	1964542
WP_174412242.1	PAPS-reductase	GCF_000520995.1	454	Bacteria>FCB_group>Bacteroidetes/Chlorobi_group>Bacteroidetes>Flavobacteriia	Aquimarina_macrocephali	DNA-phosphorothioation-system-sulfurtransferase-DndC-[Aquimarina-macrocephali]	666563
WP_126160989.1	PAPS-reductase	GCF_003957295.1	367	Bacteria>FCB_group>Bacteroidetes/Chlorobi_group>Bacteroidetes>Flavobacteriia	Arenibacter_aquaticus	phosphoadenosine-phosphosulfate-reductase-family-protein-[Arenibacter-aquaticus]	2489054
WP_219052749.1	PAPS-reductase	GCF_019312585.1	445	Bacteria>FCB_group>Bacteroidetes/Chlorobi_group>Bacteroidetes>Flavobacteriia	Aureisphaera_sp._CAU_1614	DNA-phosphorothioation-system-sulfurtransferase-DndC-[Aureisphaera-sp.-CAU-1614]	2857106
WP_119058308.1	PAPS-reductase	GCF_900538215.1	446	Bacteria>FCB_group>Bacteroidetes/Chlorobi_group>Bacteroidetes>Flavobacteriia	Candidatus_Ornithobacterium_hominis	DNA-phosphorothioation-system-sulfurtransferase-DndC-[Candidatus-Ornithobacterium-hominis]	2497989
WP_221407925.1	PAPS-reductase	-	442	Bacteria>FCB_group>Bacteroidetes/Chlorobi_group>Bacteroidetes>Flavobacteriia	Candidatus_Ornithobacterium_hominis	DNA-phosphorothioation-system-sulfurtransferase-DndC-[Candidatus-Ornithobacterium-hominis]	2497989
AEK23166.1	PAPS-reductase	GCA_000220625.1	330	Bacteria>FCB_group>Bacteroidetes/Chlorobi_group>Bacteroidetes>Flavobacteriia	Capnocytophaga_canimorsus_Cc5	hypothetical-protein-Ccan_10500-[Capnocytophaga-canimorsus-Cc5]	860228
CEN48484.1	PAPS-reductase	GCA_000947275.1	446	Bacteria>FCB_group>Bacteroidetes/Chlorobi_group>Bacteroidetes>Flavobacteriia	Capnocytophaga_canimorsus	conserved-hypothetical-protein-[Capnocytophaga-canimorsus]	28188
WP_044729637.1	PAPS-reductase	GCF_000947275.1	446	Bacteria>FCB_group>Bacteroidetes/Chlorobi_group>Bacteroidetes>Flavobacteriia	Capnocytophaga_canimorsus	DNA-phosphorothioation-system-sulfurtransferase-DndC-[Capnocytophaga-canimorsus]	28188
WP_109861978.1	PAPS-reductase	GCF_003146745.1	446	Bacteria>FCB_group>Bacteroidetes/Chlorobi_group>Bacteroidetes>Flavobacteriia	Capnocytophaga_canimorsus	DNA-phosphorothioation-system-sulfurtransferase-DndC-[Capnocytophaga-canimorsus]	28188
WP_212895342.1	PAPS-reductase	GCF_018332635.1	446	Bacteria>FCB_group>Bacteroidetes/Chlorobi_group>Bacteroidetes>Flavobacteriia	Capnocytophaga_canis	DNA-phosphorothioation-system-sulfurtransferase-DndC-[Capnocytophaga-canis]	1848903
MBM0660372.1	PAPS-reductase	GCA_016806995.1	447	Bacteria>FCB_group>Bacteroidetes/Chlorobi_group>Bacteroidetes>Flavobacteriia	Capnocytophaga_genosp._AHN8471	DNA-phosphorothioation-system-sulfurtransferase-DndC-[Capnocytophaga-genosp.-AHN8471]	327574
WP_203084771.1	PAPS-reductase	GCF_016806995.1	447	Bacteria>FCB_group>Bacteroidetes/Chlorobi_group>Bacteroidetes>Flavobacteriia	Capnocytophaga_genosp._AHN8471	DNA-phosphorothioation-system-sulfurtransferase-DndC-[Capnocytophaga-genosp.-AHN8471]	327574
GIJ94076.1	PAPS-reductase	GCA_016863695.1	299	Bacteria>FCB_group>Bacteroidetes/Chlorobi_group>Bacteroidetes>Flavobacteriia	Capnocytophaga_stomatis	hypothetical-protein-CAPN002_12940-[Capnocytophaga-stomatis]	1848904
WP_170932116.1	PAPS-reductase	GCF_900197635.1	452	Bacteria>FCB_group>Bacteroidetes/Chlorobi_group>Bacteroidetes>Flavobacteriia	Cellulophaga_lytica	DNA-phosphorothioation-system-sulfurtransferase-DndC-[Cellulophaga-lytica]	979
WP_136521961.1	PAPS-reductase	GCF_004916905.1	447	Bacteria>FCB_group>Bacteroidetes/Chlorobi_group>Bacteroidetes>Flavobacteriia	Chryseobacterium_candidae	DNA-phosphorothioation-system-sulfurtransferase-DndC-[Chryseobacterium-candidae]	1978493
WP_123876812.1	PAPS-reductase	GCF_003815695.1	446	Bacteria>FCB_group>Bacteroidetes/Chlorobi_group>Bacteroidetes>Flavobacteriia	Chryseobacterium_carnipullorum	DNA-phosphorothioation-system-sulfurtransferase-DndC-[Chryseobacterium-carnipullorum]	1124835
WP_191736972.1	PAPS-reductase	GCF_014837145.1	372	Bacteria>FCB_group>Bacteroidetes/Chlorobi_group>Bacteroidetes>Flavobacteriia	Chryseobacterium_caseinilyticum	phosphoadenosine-phosphosulfate-reductase-family-protein-[Chryseobacterium-caseinilyticum]	2771428
WP_066690410.1	PAPS-reductase	GCF_900142615.1	373	Bacteria>FCB_group>Bacteroidetes/Chlorobi_group>Bacteroidetes>Flavobacteriia	Chryseobacterium_contaminans	phosphoadenosine-phosphosulfate-reductase-family-protein-[Chryseobacterium-contaminans]	1423959
WP_105683034.1	PAPS-reductase	GCF_002979665.1	374	Bacteria>FCB_group>Bacteroidetes/Chlorobi_group>Bacteroidetes>Flavobacteriia	Chryseobacterium_culicis	phosphoadenosine-phosphosulfate-reductase-family-protein-[Chryseobacterium-culicis]	680127
WP_027385161.1	PAPS-reductase	GCF_000430845.1	372	Bacteria>FCB_group>Bacteroidetes/Chlorobi_group>Bacteroidetes>Flavobacteriia	Chryseobacterium_gregarium	phosphoadenosine-phosphosulfate-reductase-family-protein-[Chryseobacterium-gregarium]	456299
WP_079243042.1	PAPS-reductase	GCF_002025665.1	446	Bacteria>FCB_group>Bacteroidetes/Chlorobi_group>Bacteroidetes>Flavobacteriia	Chryseobacterium_indologenes	DNA-phosphorothioation-system-sulfurtransferase-DndC-[Chryseobacterium-indologenes]	253
WP_130868037.1	PAPS-reductase	GCF_900683405.1	368	Bacteria>FCB_group>Bacteroidetes/Chlorobi_group>Bacteroidetes>Flavobacteriia	Chryseobacterium_indologenes	phosphoadenosine-phosphosulfate-reductase-family-protein-[Chryseobacterium-indologenes]	253
WP_111959898.1	PAPS-reductase	GCF_007991435.1	368	Bacteria>FCB_group>Bacteroidetes/Chlorobi_group>Bacteroidetes>Flavobacteriia	Chryseobacterium_lathyri	phosphoadenosine-phosphosulfate-reductase-family-protein-[Chryseobacterium-lathyri]	395933
WP_115972599.1	PAPS-reductase	GCF_003385515.1	446	Bacteria>FCB_group>Bacteroidetes/Chlorobi_group>Bacteroidetes>Flavobacteriia	Chryseobacterium_pennae	DNA-phosphorothioation-system-sulfurtransferase-DndC-[Chryseobacterium-pennae]	2258962
WP_115971825.1	PAPS-reductase	GCF_003385515.1	368	Bacteria>FCB_group>Bacteroidetes/Chlorobi_group>Bacteroidetes>Flavobacteriia	Chryseobacterium_pennae	phosphoadenosine-phosphosulfate-reductase-family-protein-[Chryseobacterium-pennae]	2258962
RZJ90737.1	PAPS-reductase	GCA_004210875.1	441	Bacteria>FCB_group>Bacteroidetes/Chlorobi_group>Bacteroidetes>Flavobacteriia	Chryseobacterium_sp.	DNA-phosphorothioation-system-sulfurtransferase-DndC-[Chryseobacterium-sp.]	1871047
WP_180565395.1	PAPS-reductase	GCF_903969135.1	454	Bacteria>FCB_group>Bacteroidetes/Chlorobi_group>Bacteroidetes>Flavobacteriia	Chryseobacterium_sp._JV274	DNA-phosphorothioation-system-sulfurtransferase-DndC-[Chryseobacterium-sp.-JV274]	1932669
WP_179285911.1	PAPS-reductase	GCF_002205795.1	453	Bacteria>FCB_group>Bacteroidetes/Chlorobi_group>Bacteroidetes>Flavobacteriia	Chryseobacterium_sp._VAUSW3	DNA-phosphorothioation-system-sulfurtransferase-DndC-[Chryseobacterium-sp.-VAUSW3]	2010998
WP_106658989.1	PAPS-reductase	GCF_002843175.1	367	Bacteria>FCB_group>Bacteroidetes/Chlorobi_group>Bacteroidetes>Flavobacteriia	Confluentibacter_flavum	phosphoadenosine-phosphosulfate-reductase-family-protein-[Confluentibacter-flavum]	1909700
WP_179241061.1	PAPS-reductase	GCF_013402795.1	451	Bacteria>FCB_group>Bacteroidetes/Chlorobi_group>Bacteroidetes>Flavobacteriia	Costertonia_aggregata	DNA-phosphorothioation-system-sulfurtransferase-DndC-[Costertonia-aggregata]	343403
WP_209065952.1	PAPS-reductase	GCF_011882165.1	453	Bacteria>FCB_group>Bacteroidetes/Chlorobi_group>Bacteroidetes>Flavobacteriia	Croceivirga_sp._JEA036	DNA-phosphorothioation-system-sulfurtransferase-DndC-[Croceivirga-sp.-JEA036]	2721162

Accession	Annotation	GCA	Length	Phyletic.spread	Strain.name	Defline	Species.Taxid
MBT04105.1	PAPS-reductase	GCA_002727735.1	305	Bacteria>FCB_group>Bacteroidetes/Chlorobi_group>Bacteroidetes>Flavobacteriia	Crocinitomicaceae_bacterium	hypothetical-protein-[Crocinitomicaceae-bacterium]	2026728
WP_185116449.1	PAPS-reductase	GCF_002023405.1	454	Bacteria>FCB_group>Bacteroidetes/Chlorobi_group>Bacteroidetes>Flavobacteriia	Elizabethkingia_ursingii	DNA-phosphorothioation-system-sulfurtransferase-DndC-[Elizabethkingia-ursingii]	1756150
RYH74849.1	PAPS-reductase	GCA_004151365.1	451	Bacteria>FCB_group>Bacteroidetes/Chlorobi_group>Bacteroidetes>Flavobacteriia	Flavobacteriaceae_bacterium_144Ye	DNA-phosphorothioation-system-sulfurtransferase-DndC-[Flavobacteriaceae-bacterium-144Ye]	2510794
ACU07674.1	PAPS-reductase	GCA_000023725.1	453	Bacteria>FCB_group>Bacteroidetes/Chlorobi_group>Bacteroidetes>Flavobacteriia	Flavobacteriaceae_bacterium_3519-10	hypothetical-protein-FIC_01226-[Flavobacteriaceae-bacterium-3519-10]	531844
MXV38345.1	PAPS-reductase	GCA_009835005.1	451	Bacteria>FCB_group>Bacteroidetes/Chlorobi_group>Bacteroidetes>Flavobacteriia	Flavobacteriaceae_bacterium_Ap0902	DNA-phosphorothioation-system-sulfurtransferase-DndC-[Flavobacteriaceae-bacterium-Ap0902]	2684397
MBD3724544.1	PAPS-reductase	GCA_014763515.1	442	Bacteria>FCB_group>Bacteroidetes/Chlorobi_group>Bacteroidetes>Flavobacteriia	Flavobacteriaceae_bacterium	DNA-phosphorothioation-system-sulfurtransferase-DndC-[Flavobacteriaceae-bacterium]	1871037
UPT70974.1	PAPS-reductase	GCA_023213215.1	453	Bacteria>FCB_group>Bacteroidetes/Chlorobi_group>Bacteroidetes>Flavobacteriia	Flavobacteriaceae_bacterium	DNA-phosphorothioation-system-sulfurtransferase-DndC-[Flavobacteriaceae-bacterium]	1871037
MAR42616.1	PAPS-reductase	GCA_002701825.1	305	Bacteria>FCB_group>Bacteroidetes/Chlorobi_group>Bacteroidetes>Flavobacteriia	Flavobacteriaceae_bacterium	hypothetical-protein-[Flavobacteriaceae-bacterium]	1871037
MAV13445.1	PAPS-reductase	GCA_002703065.1	309	Bacteria>FCB_group>Bacteroidetes/Chlorobi_group>Bacteroidetes>Flavobacteriia	Flavobacteriaceae_bacterium	hypothetical-protein-[Flavobacteriaceae-bacterium]	1871037
MBL4662529.1	PAPS-reductase	GCA_016763715.1	366	Bacteria>FCB_group>Bacteroidetes/Chlorobi_group>Bacteroidetes>Flavobacteriia	Flavobacteriaceae_bacterium	phosphoadenosine-phosphosulfate-reductase-family-protein-[Flavobacteriaceae-bacterium]	1871037
MAB47211.1	PAPS-reductase	GCA_002682935.1	375	Bacteria>FCB_group>Bacteroidetes/Chlorobi_group>Bacteroidetes>Flavobacteriia	Flavobacteriaceae_bacterium	phosphoadenosine-phosphosulfate-reductase-[Flavobacteriaceae-bacterium]	1871037
MAD96781.1	PAPS-reductase	GCA_002686575.1	367	Bacteria>FCB_group>Bacteroidetes/Chlorobi_group>Bacteroidetes>Flavobacteriia	Flavobacteriaceae_bacterium	phosphoadenosine-phosphosulfate-reductase-[Flavobacteriaceae-bacterium]	1871037
MBF90820.1	PAPS-reductase	GCA_002715345.1	369	Bacteria>FCB_group>Bacteroidetes/Chlorobi_group>Bacteroidetes>Flavobacteriia	Flavobacteriales_bacterium	DNA-phosphorothioation-system-sulfurtransferase-DndC-[Flavobacteriales-bacterium]	2021391
MBK8707889.1	PAPS-reductase	GCA_016715385.1	440	Bacteria>FCB_group>Bacteroidetes/Chlorobi_group>Bacteroidetes>Flavobacteriia	Flavobacteriales_bacterium	DNA-phosphorothioation-system-sulfurtransferase-DndC-[Flavobacteriales-bacterium]	2021391
MBS4039684.1	PAPS-reductase	GCA_018335925.1	439	Bacteria>FCB_group>Bacteroidetes/Chlorobi_group>Bacteroidetes>Flavobacteriia	Flavobacteriales_bacterium	DNA-phosphorothioation-system-sulfurtransferase-DndC-[Flavobacteriales-bacterium]	2021391
MBK6473783.1	PAPS-reductase	GCA_016704485.1	376	Bacteria>FCB_group>Bacteroidetes/Chlorobi_group>Bacteroidetes>Flavobacteriia	Flavobacteriales_bacterium	phosphoadenosine-phosphosulfate-reductase-family-protein-[Flavobacteriales-bacterium]	2021391
HAQ71014.1	PAPS-reductase	GCA_003477125.1	375	Bacteria>FCB_group>Bacteroidetes/Chlorobi_group>Bacteroidetes>Flavobacteriia	Flavobacteriales_bacterium	phosphoadenosine-phosphosulfate-reductase-[Flavobacteriales-bacterium]	2021391
MBH1960131.1	PAPS-reductase	GCA_015999765.1	454	Bacteria>FCB_group>Bacteroidetes/Chlorobi_group>Bacteroidetes>Flavobacteriia	Flavobacteriia_bacterium	DNA-phosphorothioation-system-sulfurtransferase-DndC-[Flavobacteriia-bacterium]	2044941
NCO64661.1	PAPS-reductase	GCA_009993745.1	451	Bacteria>FCB_group>Bacteroidetes/Chlorobi_group>Bacteroidetes>Flavobacteriia	Flavobacteriia_bacterium	DNA-phosphorothioation-system-sulfurtransferase-DndC-[Flavobacteriia-bacterium]	2044941
WP_072959295.1	PAPS-reductase	GCF_002217195.1	375	Bacteria>FCB_group>Bacteroidetes/Chlorobi_group>Bacteroidetes>Flavobacteriia	Flavobacterium_aquidurense	phosphoadenosine-phosphosulfate-reductase-family-protein-[Flavobacterium-aquidurense]	362413
WP_208863515.1	PAPS-reductase	GCF_002222055.1	454	Bacteria>FCB_group>Bacteroidetes/Chlorobi_group>Bacteroidetes>Flavobacteriia	Flavobacterium_araucananum	DNA-phosphorothioation-system-sulfurtransferase-DndC-[Flavobacterium-araucananum]	946678
WP_097554527.1	PAPS-reductase	GCF_002530755.1	367	Bacteria>FCB_group>Bacteroidetes/Chlorobi_group>Bacteroidetes>Flavobacteriia	Flavobacterium_branchiophilum	phosphoadenosine-phosphosulfate-reductase-family-protein-[Flavobacterium-branchiophilum]	55197
WP_196239572.1	PAPS-reductase	GCF_003202435.1	454	Bacteria>FCB_group>Bacteroidetes/Chlorobi_group>Bacteroidetes>Flavobacteriia	Flavobacterium_cheongpyeongense	DNA-phosphorothioation-system-sulfurtransferase-DndC-[Flavobacterium-cheongpyeongense]	2212651
WP_068843556.1	PAPS-reductase	GCF_900142685.1	368	Bacteria>FCB_group>Bacteroidetes/Chlorobi_group>Bacteroidetes>Flavobacteriia	Flavobacterium_chilense	phosphoadenosine-phosphosulfate-reductase-family-protein-[Flavobacterium-chilense]	946677
WP_223052473.1	PAPS-reductase	GCF_019891545.1	452	Bacteria>FCB_group>Bacteroidetes/Chlorobi_group>Bacteroidetes>Flavobacteriia	Flavobacterium_coralii	DNA-phosphorothioation-system-sulfurtransferase-DndC-[Flavobacterium-sp.-D11R37]	2838017
ESU22604.1	PAPS-reductase	GCA_000498495.1	454	Bacteria>FCB_group>Bacteroidetes/Chlorobi_group>Bacteroidetes>Flavobacteriia	Flavobacterium_enshiense_DK69	hypothetical-protein-FEDK69T_18570-[Flavobacterium-enshiense-DK69]	1107311
WP_023573841.1	PAPS-reductase	GCF_000498495.1	454	Bacteria>FCB_group>Bacteroidetes/Chlorobi_group>Bacteroidetes>Flavobacteriia	Flavobacterium_enshiense	DNA-phosphorothioation-system-sulfurtransferase-DndC-[Flavobacterium-enshiense]	1341165
WP_114754037.1	PAPS-reductase	GCF_003350545.1	375	Bacteria>FCB_group>Bacteroidetes/Chlorobi_group>Bacteroidetes>Flavobacteriia	Flavobacterium_glaciei	phosphoadenosine-phosphosulfate-reductase-family-protein-[Flavobacterium-glaciei]	386300
WP_035626398.1	PAPS-reductase	-	440	Bacteria>FCB_group>Bacteroidetes/Chlorobi_group>Bacteroidetes>Flavobacteriia	Flavobacterium_hydatis	DNA-phosphorothioation-system-sulfurtransferase-DndC-[Flavobacterium-hydatis]	991
WP_205625904.1	PAPS-reductase	GCF_000737695.1	454	Bacteria>FCB_group>Bacteroidetes/Chlorobi_group>Bacteroidetes>Flavobacteriia	Flavobacterium_hydatis	DNA-phosphorothioation-system-sulfurtransferase-DndC-[Flavobacterium-hydatis]	991
WP_110347440.1	PAPS-reductase	GCF_003202405.1	439	Bacteria>FCB_group>Bacteroidetes/Chlorobi_group>Bacteroidetes>Flavobacteriia	Flavobacterium_hydrophilum	DNA-phosphorothioation-system-sulfurtransferase-DndC-[Flavobacterium-hydrophilum]	2211445
WP_014388834.1	PAPS-reductase	GCF_000455605.1	440	Bacteria>FCB_group>Bacteroidetes/Chlorobi_group>Bacteroidetes>Flavobacteriia	Flavobacterium_indicum	DNA-phosphorothioation-system-sulfurtransferase-DndC-[Flavobacterium-indicum]	312277
WP_188726543.1	PAPS-reductase	GCF_014644395.1	454	Bacteria>FCB_group>Bacteroidetes/Chlorobi_group>Bacteroidetes>Flavobacteriia	Flavobacterium_limi	DNA-phosphorothioation-system-sulfurtransferase-DndC-[Flavobacterium-limi]	2045105
WP_187656012.1	PAPS-reductase	GCF_014385475.1	442	Bacteria>FCB_group>Bacteroidetes/Chlorobi_group>Bacteroidetes>Flavobacteriia	Flavobacterium_macrobrachii	DNA-phosphorothioation-system-sulfurtransferase-DndC-[Flavobacterium-macrobrachii]	591204
WP_135219756.1	PAPS-reductase	GCF_014876705.1	446	Bacteria>FCB_group>Bacteroidetes/Chlorobi_group>Bacteroidetes>Flavobacteriia	Flavobacterium	MULTISPECIES:-DNA-phosphorothioation-system-sulfurtransferase-DndC-[unclassified-Flavobacterium]	237
WP_243230462.1	PAPS-reductase	GCF_022802975.1	454	Bacteria>FCB_group>Bacteroidetes/Chlorobi_group>Bacteroidetes>Flavobacteriia	Flavobacterium_pectinovorum	DNA-phosphorothioation-system-sulfurtransferase-DndC-[Flavobacterium-pectinovorum]	29533
WP_113637051.1	PAPS-reductase	GCF_003312425.1	439	Bacteria>FCB_group>Bacteroidetes/Chlorobi_group>Bacteroidetes>Flavobacteriia	Flavobacterium_psychrolimnae	DNA-phosphorothioation-system-sulfurtransferase-DndC-[Flavobacterium-psychrolimnae]	249351
WP_179213523.1	PAPS-reductase	GCF_900186475.1	454	Bacteria>FCB_group>Bacteroidetes/Chlorobi_group>Bacteroidetes>Flavobacteriia	Flavobacterium_psychrophilum	DNA-phosphorothioation-system-sulfurtransferase-DndC-[Flavobacterium-psychrophilum]	96345
WP_179214502.1	PAPS-reductase	GCF_900186495.1	454	Bacteria>FCB_group>Bacteroidetes/Chlorobi_group>Bacteroidetes>Flavobacteriia	Flavobacterium_psychrophilum	DNA-phosphorothioation-system-sulfurtransferase-DndC-[Flavobacterium-psychrophilum]	96345
WP_144257340.1	PAPS-reductase	GCF_007097245.1	345	Bacteria>FCB_group>Bacteroidetes/Chlorobi_group>Bacteroidetes>Flavobacteriia	Flavobacterium_restrictum	DNA-phosphorothioation-system-sulfurtransferase-DndC-[Flavobacterium-sp.-LB1R34]	2594428
KAF2516328.1	PAPS-reductase	GCA_004634195.1	446	Bacteria>FCB_group>Bacteroidetes/Chlorobi_group>Bacteroidetes>Flavobacteriia	Flavobacterium_salilacus_subsp._salilacus	DNA-phosphorothioation-system-sulfurtransferase-DndC-[Flavobacterium-sp.-SaA2.12]	2545633
WP_023575224.1	PAPS-reductase	GCF_000498515.1	451	Bacteria>FCB_group>Bacteroidetes/Chlorobi_group>Bacteroidetes>Flavobacteriia	Flavobacterium_saliperosum	DNA-phosphorothioation-system-sulfurtransferase-DndC-[Flavobacterium-saliperosum]	329186
ESU27664.1	PAPS-reductase	GCA_000498515.1	451	Bacteria>FCB_group>Bacteroidetes/Chlorobi_group>Bacteroidetes>Flavobacteriia	Flavobacterium_saliperosum_S13	hypothetical-protein-FSS13T_01370-[Flavobacterium-saliperosum-S13]	1341155
WP_121314162.1	PAPS-reductase	GCF_003634615.1	374	Bacteria>FCB_group>Bacteroidetes/Chlorobi_group>Bacteroidetes>Flavobacteriia	Flavobacterium_sp._102	phosphoadenosine-phosphosulfate-reductase-family-protein-[Flavobacterium-sp.-102]	2135623
WP_200894159.1	PAPS-reductase	GCF_000967805.1	452	Bacteria>FCB_group>Bacteroidetes/Chlorobi_group>Bacteroidetes>Flavobacteriia	Flavobacterium_sp._316	DNA-phosphorothioation-system-sulfurtransferase-DndC-[Flavobacterium-sp.-316]	1603293
WP_211064957.1	PAPS-reductase	GCF_018080005.1	453	Bacteria>FCB_group>Bacteroidetes/Chlorobi_group>Bacteroidetes>Flavobacteriia	Flavobacterium_sp._CS20	DNA-phosphorothioation-system-sulfurtransferase-DndC-[Flavobacterium-sp.-CS20]	2775246
MBF01205.1	PAPS-reductase	GCA_002714575.1	446	Bacteria>FCB_group>Bacteroidetes/Chlorobi_group>Bacteroidetes>Flavobacteriia	Flavobacterium_sp.	DNA-phosphorothioation-system-sulfurtransferase-DndC-[Flavobacterium-sp.]	239
WP_056250033.1	PAPS-reductase	GCF_001422725.1	375	Bacteria>FCB_group>Bacteroidetes/Chlorobi_group>Bacteroidetes>Flavobacteriia	Flavobacterium_sp._Leaf82	phosphoadenosine-phosphosulfate-reductase-family-protein-[Flavobacterium-sp.-Leaf82]	1736238
WP_198528497.1	PAPS-reductase	GCF_001427905.1	454	Bacteria>FCB_group>Bacteroidetes/Chlorobi_group>Bacteroidetes>Flavobacteriia	Flavobacterium_sp._Root420	DNA-phosphorothioation-system-sulfurtransferase-DndC-[Flavobacterium-sp.-Root420]	1736533
WP_202390764.1	PAPS-reductase	GCF_009826935.1	457	Bacteria>FCB_group>Bacteroidetes/Chlorobi_group>Bacteroidetes>Flavobacteriia	Flavobacterium_sp._Sd200	DNA-phosphorothioation-system-sulfurtransferase-DndC-[Flavobacterium-sp.-Sd200]	2692211
WP_220641802.1	PAPS-reductase	-	454	Bacteria>FCB_group>Bacteroidetes/Chlorobi_group>Bacteroidetes>Flavobacteriia	Flavobacterium_sp._WSW3-B6	DNA-phosphorothioation-system-sulfurtransferase-DndC-[Flavobacterium-sp.-WSW3-B6]	2856519
WP_026977602.1	PAPS-reductase	GCF_000425485.1	375	Bacteria>FCB_group>Bacteroidetes/Chlorobi_group>Bacteroidetes>Flavobacteriia	Flavobacterium_tegetincola	phosphoadenosine-phosphosulfate-reductase-family-protein-[Flavobacterium-tegetincola]	150172
MBL1280772.1	PAPS-reductase	GCA_002734065.2	454	Bacteria>FCB_group>Bacteroidetes/Chlorobi_group>Bacteroidetes>Flavobacteriia	Fluviicola_sp.	DNA-phosphorothioation-system-sulfurtransferase-DndC-[Fluviicola-sp.]	1917219
PHR45459.1	PAPS-reductase	GCA_002734085.1	442	Bacteria>FCB_group>Bacteroidetes/Chlorobi_group>Bacteroidetes>Flavobacteriia	Fluviicola_sp.	DNA-phosphorothioation-system-sulfurtransferase-DndC-[Fluviicola-sp.]	1917219
WP_172441768.1	PAPS-reductase	GCF_002900225.1	454	Bacteria>FCB_group>Bacteroidetes/Chlorobi_group>Bacteroidetes>Flavobacteriia	Formosa_algae	DNA-phosphorothioation-system-sulfurtransferase-DndC-[Formosa-algae]	225843
WP_057781520.1	PAPS-reductase	GCF_017872935.1	375	Bacteria>FCB_group>Bacteroidetes/Chlorobi_group>Bacteroidetes>Flavobacteriia	Formosa_algae	phosphoadenosine-phosphosulfate-reductase-family-protein-[Formosa-algae]	225843
WP_130962953.1	PAPS-reductase	GCF_004310325.1	367	Bacteria>FCB_group>Bacteroidetes/Chlorobi_group>Bacteroidetes>Flavobacteriia	Hyunsoonleella_flava	phosphoadenosine-phosphosulfate-reductase-family-protein-[Hyunsoonleella-sp.-T58]	2527939
WP_130937543.1	PAPS-reductase	GCF_014636335.1	367	Bacteria>FCB_group>Bacteroidetes/Chlorobi_group>Bacteroidetes>Flavobacteriia	Hyunsoonleella_pacifica	phosphoadenosine-phosphosulfate-reductase-family-protein-[Hyunsoonleella-pacifica]	1080224
WP_007096428.1	PAPS-reductase	GCF_000154725.1	445	Bacteria>FCB_group>Bacteroidetes/Chlorobi_group>Bacteroidetes>Flavobacteriia	Kordia_algicida	DNA-phosphorothioation-system-sulfurtransferase-DndC-[Kordia-algicida]	221066
WP_160129422.1	PAPS-reductase	GCF_009901525.1	451	Bacteria>FCB_group>Bacteroidetes/Chlorobi_group>Bacteroidetes>Flavobacteriia	Kordia_antarctica	DNA-phosphorothioation-system-sulfurtransferase-DndC-[Kordia-antarctica]	1218801
MBQ50901.1	PAPS-reductase	GCA_002727005.1	451	Bacteria>FCB_group>Bacteroidetes/Chlorobi_group>Bacteroidetes>Flavobacteriia	Leeuwenhoekiella_sp.	DNA-phosphorothioation-system-sulfurtransferase-DndC-[Leeuwenhoekiella-sp.]	1977054
MBF8984108.1	PAPS-reductase	GCA_015686845.1	247	Bacteria>FCB_group>Bacteroidetes/Chlorobi_group>Bacteroidetes>Flavobacteriia	Lutibacter_sp._B2	phosphoadenosine-phosphosulfate-reductase-family-protein-[Lutibacter-sp.-B2]	2789328
WP_196119051.1	PAPS-reductase	-	247	Bacteria>FCB_group>Bacteroidetes/Chlorobi_group>Bacteroidetes>Flavobacteriia	Lutibacter_sp._B2	phosphoadenosine-phosphosulfate-reductase-family-protein-[Lutibacter-sp.-B2]	2789328
WP_230387203.1	PAPS-reductase	-	618	Bacteria>FCB_group>Bacteroidetes/Chlorobi_group>Bacteroidetes>Flavobacteriia	Lutibacter_sp._B2	phosphoadenosine-phosphosulfate-reductase-family-protein-[Lutibacter-sp.-B2]	2789328
WP_053976583.1	PAPS-reductase	GCF_001280435.1	375	Bacteria>FCB_group>Bacteroidetes/Chlorobi_group>Bacteroidetes>Flavobacteriia	Mangrovimonas_xylaniphaga	phosphoadenosine-phosphosulfate-reductase-family-protein-[Mangrovimonas-xylaniphaga]	1645915
WP_209435197.1	PAPS-reductase	GCF_000744105.1	454	Bacteria>FCB_group>Bacteroidetes/Chlorobi_group>Bacteroidetes>Flavobacteriia	Maribacter_forsetii	DNA-phosphorothioation-system-sulfurtransferase-DndC-[Maribacter-forsetii]	444515
HAF77441.1	PAPS-reductase	GCA_003447055.1	442	Bacteria>FCB_group>Bacteroidetes/Chlorobi_group>Bacteroidetes>Flavobacteriia	Maribacter_sp.	DNA-phosphorothioation-system-sulfurtransferase-DndC-[Maribacter-sp.]	1897614
PVW16571.1	PAPS-reductase	GCA_003095375.1	449	Bacteria>FCB_group>Bacteroidetes/Chlorobi_group>Bacteroidetes>Flavobacteriia	Marixanthomonas_spongiae	DNA-phosphorothioation-system-sulfurtransferase-DndC-[Marixanthomonas-sp.-HN-E44]	2174845
WP_206074000.1	PAPS-reductase	GCF_003095375.1	452	Bacteria>FCB_group>Bacteroidetes/Chlorobi_group>Bacteroidetes>Flavobacteriia	Marixanthomonas_spongiae	DNA-phosphorothioation-system-sulfurtransferase-DndC-[Marixanthomonas-sp.-HN-E44]	2174845
WP_189394875.1	PAPS-reductase	GCF_000423405.1	452	Bacteria>FCB_group>Bacteroidetes/Chlorobi_group>Bacteroidetes>Flavobacteriia	Mesonia_mobilis	DNA-phosphorothioation-system-sulfurtransferase-DndC-[Mesonia-mobilis]	369791
WP_207036608.1	PAPS-reductase	GCF_017313785.1	452	Bacteria>FCB_group>Bacteroidetes/Chlorobi_group>Bacteroidetes>Flavobacteriia	Muricauda_aurea	DNA-phosphorothioation-system-sulfurtransferase-DndC-[Muricauda-ruestringensis]	2915619
WP_136468586.1	PAPS-reductase	GCF_004804315.1	452	Bacteria>FCB_group>Bacteroidetes/Chlorobi_group>Bacteroidetes>Flavobacteriia	Muricauda_onchidii	DNA-phosphorothioation-system-sulfurtransferase-DndC-[Flagellimonas-sp.-XY-359]	2562684
WP_207025787.1	PAPS-reductase	GCF_017313275.1	439	Bacteria>FCB_group>Bacteroidetes/Chlorobi_group>Bacteroidetes>Flavobacteriia	Muricauda_profundi	DNA-phosphorothioation-system-sulfurtransferase-DndC-[Muricauda-lutimaris]	2915620
WP_169932168.1	PAPS-reductase	GCF_012974865.1	367	Bacteria>FCB_group>Bacteroidetes/Chlorobi_group>Bacteroidetes>Flavobacteriia	Nonlabens_sp._Ci31	phosphoadenosine-phosphosulfate-reductase-family-protein-[Nonlabens-sp.-Ci31]	2608253
WP_198551039.1	PAPS-reductase	GCF_002836085.1	454	Bacteria>FCB_group>Bacteroidetes/Chlorobi_group>Bacteroidetes>Flavobacteriia	Olleya_sp._1-3	DNA-phosphorothioation-system-sulfurtransferase-DndC-[Olleya-sp.-1-3]	2058323
WP_068449091.1	PAPS-reductase	GCF_001640115.1	375	Bacteria>FCB_group>Bacteroidetes/Chlorobi_group>Bacteroidetes>Flavobacteriia	Polaribacter_atrinae	phosphoadenosine-phosphosulfate-reductase-family-protein-[Polaribacter-atrinae]	1333662
WP_201288841.1	PAPS-reductase	GCF_009832745.1	454	Bacteria>FCB_group>Bacteroidetes/Chlorobi_group>Bacteroidetes>Flavobacteriia	Polaribacter_septentrionalilitoris	DNA-phosphorothioation-system-sulfurtransferase-DndC-[Polaribacter-sp.-ANORD1]	2494657
WP_026776290.1	PAPS-reductase	GCF_000687935.1	448	Bacteria>FCB_group>Bacteroidetes/Chlorobi_group>Bacteroidetes>Flavobacteriia	Polaribacter_sp._Hel_I_88	DNA-phosphorothioation-system-sulfurtransferase-DndC-[Polaribacter-sp.-Hel_I_88]	1250006
WP_147117801.1	PAPS-reductase	GCF_007997065.1	375	Bacteria>FCB_group>Bacteroidetes/Chlorobi_group>Bacteroidetes>Flavobacteriia	Polaribacter_sp._IC063	phosphoadenosine-phosphosulfate-reductase-family-protein-[Polaribacter-sp.-IC063]	57031
WP_172824410.1	PAPS-reductase	GCF_900105145.1	454	Bacteria>FCB_group>Bacteroidetes/Chlorobi_group>Bacteroidetes>Flavobacteriia	Polaribacter_sp._KT25b	DNA-phosphorothioation-system-sulfurtransferase-DndC-[Polaribacter-sp.-KT25b]	1855336
WP_200796295.1	PAPS-reductase	GCF_900129035.1	453	Bacteria>FCB_group>Bacteroidetes/Chlorobi_group>Bacteroidetes>Flavobacteriia	Psychroflexus_salarius	DNA-phosphorothioation-system-sulfurtransferase-DndC-[Psychroflexus-salarius]	1155689
WP_224462215.1	PAPS-reductase	GCF_020164595.1	453	Bacteria>FCB_group>Bacteroidetes/Chlorobi_group>Bacteroidetes>Flavobacteriia	Psychroflexus_sp._CAK8W	DNA-phosphorothioation-system-sulfurtransferase-DndC-[Psychroflexus-sp.-CAK8W]	2873596
WP_224463026.1	PAPS-reductase	GCF_020164535.1	453	Bacteria>FCB_group>Bacteroidetes/Chlorobi_group>Bacteroidetes>Flavobacteriia	Psychroflexus_sp._CCL10W	DNA-phosphorothioation-system-sulfurtransferase-DndC-[Psychroflexus-sp.-CCL10W]	2873598
WP_093026440.1	PAPS-reductase	GCF_900116665.1	438	Bacteria>FCB_group>Bacteroidetes/Chlorobi_group>Bacteroidetes>Flavobacteriia	Pustulibacterium_marinum	DNA-phosphorothioation-system-sulfurtransferase-DndC-[Pustulibacterium-marinum]	1224947
WP_199398992.1	PAPS-reductase	GCF_003234935.1	455	Bacteria>FCB_group>Bacteroidetes/Chlorobi_group>Bacteroidetes>Flavobacteriia	Putridiphycobacter_roseus	DNA-phosphorothioation-system-sulfurtransferase-DndC-[Putridiphycobacter-roseus]	2219161
WP_004920339.1	PAPS-reductase	GCF_000183155.1	442	Bacteria>FCB_group>Bacteroidetes/Chlorobi_group>Bacteroidetes>Flavobacteriia	Riemerella_anatipestifer	DNA-phosphorothioation-system-sulfurtransferase-DndC-[Riemerella-anatipestifer]	34085
WP_154137481.1	PAPS-reductase	GCF_009670965.2	453	Bacteria>FCB_group>Bacteroidetes/Chlorobi_group>Bacteroidetes>Flavobacteriia	Riemerella_anatipestifer	DNA-phosphorothioation-system-sulfurtransferase-DndC-[Riemerella-anatipestifer]	34085
WP_224470001.1	PAPS-reductase	GCF_020164555.1	455	Bacteria>FCB_group>Bacteroidetes/Chlorobi_group>Bacteroidetes>Flavobacteriia	Salegentibacter_lacus	DNA-phosphorothioation-system-sulfurtransferase-DndC-[Salegentibacter-sp.-LM13S]	2873599
MBI6115996.1	PAPS-reductase	GCA_016236915.1	451	Bacteria>FCB_group>Bacteroidetes/Chlorobi_group>Bacteroidetes>Flavobacteriia	Salegentibacter_maritimus	DNA-phosphorothioation-system-sulfurtransferase-DndC-[Salegentibacter-maritimus]	2794347
WP_198627754.1	PAPS-reductase	GCF_016236915.1	451	Bacteria>FCB_group>Bacteroidetes/Chlorobi_group>Bacteroidetes>Flavobacteriia	Salegentibacter_maritimus	DNA-phosphorothioation-system-sulfurtransferase-DndC-[Salegentibacter-maritimus]	2794347
WP_208109640.1	PAPS-reductase	GCF_004362395.1	454	Bacteria>FCB_group>Bacteroidetes/Chlorobi_group>Bacteroidetes>Flavobacteriia	Salegentibacter_sp._24	DNA-phosphorothioation-system-sulfurtransferase-DndC-[Salegentibacter-sp.-24]	2183986
KAB1065610.1	PAPS-reductase	GCA_008806325.1	379	Bacteria>FCB_group>Bacteroidetes/Chlorobi_group>Bacteroidetes>Flavobacteriia	Salibacter_halophilus	phosphoadenosine-phosphosulfate-reductase-family-protein-[Salibacter-halophilus]	1803916
WP_151166434.1	PAPS-reductase	GCF_008806325.1	379	Bacteria>FCB_group>Bacteroidetes/Chlorobi_group>Bacteroidetes>Flavobacteriia	Salibacter_halophilus	phosphoadenosine-phosphosulfate-reductase-family-protein-[Salibacter-halophilus]	1803916
WP_186525871.1	PAPS-reductase	GCF_004358095.2	454	Bacteria>FCB_group>Bacteroidetes/Chlorobi_group>Bacteroidetes>Flavobacteriia	Seonamhaeicola_sp._W255	DNA-phosphorothioation-system-sulfurtransferase-DndC-[Seonamhaeicola-sp.-W255]	2528206
WP_175545844.1	PAPS-reductase	GCF_900119185.1	452	Bacteria>FCB_group>Bacteroidetes/Chlorobi_group>Bacteroidetes>Flavobacteriia	Sinomicrobium_oceani	DNA-phosphorothioation-system-sulfurtransferase-DndC-[Sinomicrobium-oceani]	1150368
WP_044630959.1	PAPS-reductase	GCF_000943565.1	444	Bacteria>FCB_group>Bacteroidetes/Chlorobi_group>Bacteroidetes>Flavobacteriia	Tamlana_sedimentorum	DNA-phosphorothioation-system-sulfurtransferase-DndC-[Tamlana-sedimentorum]	1435349
KJD36964.1	PAPS-reductase	GCA_000943565.1	444	Bacteria>FCB_group>Bacteroidetes/Chlorobi_group>Bacteroidetes>Flavobacteriia	Tamlana_sedimentorum	hypothetical-protein-PW52_00445-[Tamlana-sedimentorum]	1435349
WP_101907978.1	PAPS-reductase	GCF_018616275.1	375	Bacteria>FCB_group>Bacteroidetes/Chlorobi_group>Bacteroidetes>Flavobacteriia	Tenacibaculum_dicentrarchi	phosphoadenosine-phosphosulfate-reductase-family-protein-[Tenacibaculum-dicentrarchi]	669041
WP_214984766.1	PAPS-reductase	GCF_018616355.1	375	Bacteria>FCB_group>Bacteroidetes/Chlorobi_group>Bacteroidetes>Flavobacteriia	Tenacibaculum_dicentrarchi	phosphoadenosine-phosphosulfate-reductase-family-protein-[Tenacibaculum-dicentrarchi]	669041
WP_232163702.1	PAPS-reductase	GCF_022380985.1	375	Bacteria>FCB_group>Bacteroidetes/Chlorobi_group>Bacteroidetes>Flavobacteriia	Tenacibaculum_dicentrarchi	phosphoadenosine-phosphosulfate-reductase-family-protein-[Tenacibaculum-dicentrarchi]	669041
WP_232124681.1	PAPS-reductase	GCF_022382115.1	375	Bacteria>FCB_group>Bacteroidetes/Chlorobi_group>Bacteroidetes>Flavobacteriia	Tenacibaculum	MULTISPECIES:-phosphoadenosine-phosphosulfate-reductase-family-protein-[Tenacibaculum]	104267
WP_193706321.1	PAPS-reductase	GCF_015143465.1	375	Bacteria>FCB_group>Bacteroidetes/Chlorobi_group>Bacteroidetes>Flavobacteriia	Tenacibaculum_piscium	phosphoadenosine-phosphosulfate-reductase-family-protein-[Tenacibaculum-piscium]	1458515
WP_233884049.1	PAPS-reductase	GCF_021390725.1	375	Bacteria>FCB_group>Bacteroidetes/Chlorobi_group>Bacteroidetes>Flavobacteriia	Tenacibaculum_piscium	phosphoadenosine-phosphosulfate-reductase-family-protein-[Tenacibaculum-piscium]	1458515
WP_197017689.1	PAPS-reductase	GCF_000518405.1	454	Bacteria>FCB_group>Bacteroidetes/Chlorobi_group>Bacteroidetes>Flavobacteriia	Tenacibaculum_sp._47A_GOM-205m	DNA-phosphorothioation-system-sulfurtransferase-DndC-[Tenacibaculum-sp.-47A_GOM-205m]	1380384
GFD73168.1	PAPS-reductase	GCA_009811435.1	541	Bacteria>FCB_group>Bacteroidetes/Chlorobi_group>Bacteroidetes>Flavobacteriia	Tenacibaculum_sp._KUL113	hypothetical-protein-KUL113_25880-[Tenacibaculum-sp.-KUL113]	2480800
WP_199735319.1	PAPS-reductase	GCF_003633785.1	454	Bacteria>FCB_group>Bacteroidetes/Chlorobi_group>Bacteroidetes>Flavobacteriia	unclassified_Flavobacterium	MULTISPECIES:-DNA-phosphorothioation-system-sulfurtransferase-DndC-[unclassified-Flavobacterium]	196869
MBS7332834.1	PAPS-reductase	GCA_018384435.1	454	Bacteria>FCB_group>Bacteroidetes/Chlorobi_group>Bacteroidetes>Flavobacteriia	Weeksellaceae_bacterium	DNA-phosphorothioation-system-sulfurtransferase-DndC-[Weeksellaceae-bacterium]	2823802
MBV7440224.1	PAPS-reductase	GCA_019218445.1	449	Bacteria>FCB_group>Bacteroidetes/Chlorobi_group>Bacteroidetes>Flavobacteriia	Weeksellaceae_bacterium_TAE3-ERU29	DNA-phosphorothioation-system-sulfurtransferase-DndC-[Weeksellaceae-bacterium-TAE3-ERU29]	2856344
WP_195870343.1	PAPS-reductase	GCF_015645385.1	454	Bacteria>FCB_group>Bacteroidetes/Chlorobi_group>Bacteroidetes>Flavobacteriia	Winogradskyella_marina	DNA-phosphorothioation-system-sulfurtransferase-DndC-[Winogradskyella-marina]	2785530
MCA0151719.1	PAPS-reductase	GCA_020166345.1	375	Bacteria>FCB_group>Bacteroidetes/Chlorobi_group>Bacteroidetes>Flavobacteriia	Winogradskyella_sp._2Y89	phosphoadenosine-phosphosulfate-reductase-family-protein-[Winogradskyella-sp.-2Y89]	2877122
WP_224476691.1	PAPS-reductase	GCF_020166345.1	375	Bacteria>FCB_group>Bacteroidetes/Chlorobi_group>Bacteroidetes>Flavobacteriia	Winogradskyella_sp._2Y89	phosphoadenosine-phosphosulfate-reductase-family-protein-[Winogradskyella-sp.-2Y89]	2877122
MBL86570.1	PAPS-reductase	GCA_002722255.1	442	Bacteria>FCB_group>Bacteroidetes/Chlorobi_group>Bacteroidetes>Flavobacteriia	Winogradskyella_sp.	DNA-phosphorothioation-system-sulfurtransferase-DndC-[Winogradskyella-sp.]	1883156
WP_218547146.1	PAPS-reductase	GCF_019203985.1	453	Bacteria>FCB_group>Bacteroidetes/Chlorobi_group>Bacteroidetes>Flavobacteriia	Winogradskyella_sp._WHY3	DNA-phosphorothioation-system-sulfurtransferase-DndC-[Winogradskyella-sp.-WHY3]	2828330
WP_242937764.1	PAPS-reductase	GCF_022760175.1	367	Bacteria>FCB_group>Bacteroidetes/Chlorobi_group>Bacteroidetes>Flavobacteriia	Zhouia_spongiae	phosphoadenosine-phosphosulfate-reductase-family-protein-[Zhouia-spongiae]	2202721
WP_215438275.1	PAPS-reductase	GCF_018728565.1	454	Bacteria>FCB_group>Bacteroidetes/Chlorobi_group>Bacteroidetes>Flavobacteriia	Zobellia_russellii	DNA-phosphorothioation-system-sulfurtransferase-DndC-[Zobellia-russellii]	248907
WP_013766333.1	PAPS-reductase	GCF_000212735.1	436	Bacteria>FCB_group>Bacteroidetes/Chlorobi_group>Bacteroidetes>Saprospiria	Haliscomenobacter_hydrossis	DNA-phosphorothioation-system-sulfurtransferase-DndC-[Haliscomenobacter-hydrossis]	2350
MCB9310858.1	PAPS-reductase	GCA_020636195.1	423	Bacteria>FCB_group>Bacteroidetes/Chlorobi_group>Bacteroidetes>Saprospiria	Lewinellaceae_bacterium	DNA-phosphorothioation-system-sulfurtransferase-DndC-[Lewinellaceae-bacterium]	2760807
MCB9082104.1	PAPS-reductase	GCA_020636445.1	365	Bacteria>FCB_group>Bacteroidetes/Chlorobi_group>Bacteroidetes>Saprospiria	Lewinellaceae_bacterium	phosphoadenosine-phosphosulfate-reductase-family-protein-[Lewinellaceae-bacterium]	2760807
CAH1001253.1	PAPS-reductase	GCA_923081025.1	371	Bacteria>FCB_group>Bacteroidetes/Chlorobi_group>Bacteroidetes>Saprospiria	Lewinella_maritima	hypothetical-protein-LEM8419_02152-[Lewinella-maritima]	1383882
WP_238751101.1	PAPS-reductase	GCF_923081025.1	371	Bacteria>FCB_group>Bacteroidetes/Chlorobi_group>Bacteroidetes>Saprospiria	Lewinella_maritima	phosphoadenosine-phosphosulfate-reductase-family-protein-[Lewinella-maritima]	1383882
WP_044217707.1	PAPS-reductase	GCF_000759025.1	376	Bacteria>FCB_group>Bacteroidetes/Chlorobi_group>Bacteroidetes>Saprospiria	Phaeodactylibacter_xiamenensis	phosphoadenosine-phosphosulfate-reductase-family-protein-[Phaeodactylibacter-xiamenensis]	1524460
MBK6352115.1	PAPS-reductase	GCA_016705435.1	441	Bacteria>FCB_group>Bacteroidetes/Chlorobi_group>Bacteroidetes>Saprospiria	Saprospiraceae_bacterium	DNA-phosphorothioation-system-sulfurtransferase-DndC-[Saprospiraceae-bacterium]	2202734
MBK6572847.1	PAPS-reductase	GCA_016704015.1	441	Bacteria>FCB_group>Bacteroidetes/Chlorobi_group>Bacteroidetes>Saprospiria	Saprospiraceae_bacterium	DNA-phosphorothioation-system-sulfurtransferase-DndC-[Saprospiraceae-bacterium]	2202734
MBK8405861.1	PAPS-reductase	GCA_016711565.1	441	Bacteria>FCB_group>Bacteroidetes/Chlorobi_group>Bacteroidetes>Saprospiria	Saprospiraceae_bacterium	DNA-phosphorothioation-system-sulfurtransferase-DndC-[Saprospiraceae-bacterium]	2202734
MBL7823168.1	PAPS-reductase	GCA_016787185.1	441	Bacteria>FCB_group>Bacteroidetes/Chlorobi_group>Bacteroidetes>Saprospiria	Saprospiraceae_bacterium	DNA-phosphorothioation-system-sulfurtransferase-DndC-[Saprospiraceae-bacterium]	2202734

Accession	Annotation	GCA	Length	Phyletic.spread	Strain.name	Defline	Species.Taxid
MBP6235344.1	PAPS-reductase	GCA_017986015.1	441	Bacteria>FCB_group>Bacteroidetes/Chlorobi_group>Bacteroidetes>Saprospiria	Saprospiraceae_bacterium	DNA-phosphorothioation-system-sulfurtransferase-DndC-[Saprospiraceae-bacterium]	2202734
MBV6440607.1	PAPS-reductase	GCA_019187225.1	454	Bacteria>FCB_group>Bacteroidetes/Chlorobi_group>Bacteroidetes>Saprospiria	Saprospiraceae_bacterium	hypothetical-protein-[Saprospiraceae-bacterium]	2202734
MBK6498242.1	PAPS-reductase	GCA_016704395.1	366	Bacteria>FCB_group>Bacteroidetes/Chlorobi_group>Bacteroidetes>Saprospiria	Saprospiraceae_bacterium	phosphoadenosine-phosphosulfate-reductase-family-protein-[Saprospiraceae-bacterium]	2202734
MBK6665665.1	PAPS-reductase	GCA_016703485.1	366	Bacteria>FCB_group>Bacteroidetes/Chlorobi_group>Bacteroidetes>Saprospiria	Saprospiraceae_bacterium	phosphoadenosine-phosphosulfate-reductase-family-protein-[Saprospiraceae-bacterium]	2202734
MBK8828948.1	PAPS-reductase	GCA_016714525.1	366	Bacteria>FCB_group>Bacteroidetes/Chlorobi_group>Bacteroidetes>Saprospiria	Saprospiraceae_bacterium	phosphoadenosine-phosphosulfate-reductase-family-protein-[Saprospiraceae-bacterium]	2202734
MBK9582421.1	PAPS-reductase	GCA_016718065.1	366	Bacteria>FCB_group>Bacteroidetes/Chlorobi_group>Bacteroidetes>Saprospiria	Saprospiraceae_bacterium	phosphoadenosine-phosphosulfate-reductase-family-protein-[Saprospiraceae-bacterium]	2202734
MCB0522403.1	PAPS-reductase	GCA_020436325.1	365	Bacteria>FCB_group>Bacteroidetes/Chlorobi_group>Bacteroidetes>Saprospiria	Saprospiraceae_bacterium	phosphoadenosine-phosphosulfate-reductase-family-protein-[Saprospiraceae-bacterium]	2202734
MCB0706407.1	PAPS-reductase	GCA_020437105.1	370	Bacteria>FCB_group>Bacteroidetes/Chlorobi_group>Bacteroidetes>Saprospiria	Saprospiraceae_bacterium	phosphoadenosine-phosphosulfate-reductase-family-protein-[Saprospiraceae-bacterium]	2202734
WP_016196023.1	PAPS-reductase	GCF_000403135.1	371	Bacteria>FCB_group>Bacteroidetes/Chlorobi_group>Bacteroidetes>Sphingobacteriia	Arcticibacter_svalbardensis	phosphoadenosine-phosphosulfate-reductase-family-protein-[Arcticibacter-svalbardensis]	1288027
WP_129569817.1	PAPS-reductase	GCF_017569325.1	439	Bacteria>FCB_group>Bacteroidetes/Chlorobi_group>Bacteroidetes>Sphingobacteriia	Mucilaginibacter	MULTISPECIES:-DNA-phosphorothioation-system-sulfurtransferase-DndC-[Mucilaginibacter]	423349
RAV55863.1	PAPS-reductase	GCA_003286445.1	439	Bacteria>FCB_group>Bacteroidetes/Chlorobi_group>Bacteroidetes>Sphingobacteriia	Mucilaginibacter_rubeus	DNA-phosphorothioation-system-sulfurtransferase-DndC-[Mucilaginibacter-rubeus]	2027860
WP_162431197.1	PAPS-reductase	GCF_010093045.1	435	Bacteria>FCB_group>Bacteroidetes/Chlorobi_group>Bacteroidetes>Sphingobacteriia	Mucilaginibacter_sp._14171R-50	DNA-phosphorothioation-system-sulfurtransferase-DndC-[Mucilaginibacter-sp.-14171R-50]	2703789
MBV8388976.1	PAPS-reductase	GCA_019235595.1	465	Bacteria>FCB_group>Bacteroidetes/Chlorobi_group>Bacteroidetes>Sphingobacteriia	Mucilaginibacter_sp.	DNA-phosphorothioation-system-sulfurtransferase-DndC-[Mucilaginibacter-sp.]	1882438
WP_090466111.1	PAPS-reductase	GCF_900103125.1	441	Bacteria>FCB_group>Bacteroidetes/Chlorobi_group>Bacteroidetes>Sphingobacteriia	Mucilaginibacter_sp._OK268	DNA-phosphorothioation-system-sulfurtransferase-DndC-[Mucilaginibacter-sp.-OK268]	1881048
WP_172335594.1	PAPS-reductase	GCF_013149275.1	438	Bacteria>FCB_group>Bacteroidetes/Chlorobi_group>Bacteroidetes>Sphingobacteriia	Mucilaginibacter_sp._SG564	DNA-phosphorothioation-system-sulfurtransferase-DndC-[Mucilaginibacter-sp.-SG564]	2587022
WP_228949109.1	PAPS-reductase	GCF_020880975.1	439	Bacteria>FCB_group>Bacteroidetes/Chlorobi_group>Bacteroidetes>Sphingobacteriia	Mucilaginibacter_sp._UR6-1	DNA-phosphorothioation-system-sulfurtransferase-DndC-[Mucilaginibacter-sp.-UR6-1]	1435643
WP_129875607.1	PAPS-reductase	GCF_004168255.1	437	Bacteria>FCB_group>Bacteroidetes/Chlorobi_group>Bacteroidetes>Sphingobacteriia	Mucilaginibacter_terrigena	DNA-phosphorothioation-system-sulfurtransferase-DndC-[Mucilaginibacter-terrigena]	2492395
WP_094570627.1	PAPS-reductase	GCF_002257585.1	441	Bacteria>FCB_group>Bacteroidetes/Chlorobi_group>Bacteroidetes>Sphingobacteriia	Mucilaginibacter_xinganensis	DNA-phosphorothioation-system-sulfurtransferase-DndC-[Mucilaginibacter-xinganensis]	1234841
ASU34254.1	PAPS-reductase	GCA_002257585.1	441	Bacteria>FCB_group>Bacteroidetes/Chlorobi_group>Bacteroidetes>Sphingobacteriia	Mucilaginibacter_xinganensis	DNA-sulfur-modification-protein-DndC-[Mucilaginibacter-xinganensis]	1234841
WP_188753158.1	PAPS-reductase	GCF_014642075.1	447	Bacteria>FCB_group>Bacteroidetes/Chlorobi_group>Bacteroidetes>Sphingobacteriia	Parapedobacter_defluvii	DNA-phosphorothioation-system-sulfurtransferase-DndC-[Parapedobacter-defluvii]	2045106
WP_079717015.1	PAPS-reductase	GCF_900168055.1	433	Bacteria>FCB_group>Bacteroidetes/Chlorobi_group>Bacteroidetes>Sphingobacteriia	Parapedobacter_luteus	DNA-phosphorothioation-system-sulfurtransferase-DndC-[Parapedobacter-luteus]	623280
WP_182952641.1	PAPS-reductase	GCF_014172405.1	441	Bacteria>FCB_group>Bacteroidetes/Chlorobi_group>Bacteroidetes>Sphingobacteriia	Pedobacter_gandavensis	DNA-phosphorothioation-system-sulfurtransferase-DndC-[Pedobacter-gandavensis]	2679963
WP_057930418.1	PAPS-reductase	GCF_001442625.1	434	Bacteria>FCB_group>Bacteroidetes/Chlorobi_group>Bacteroidetes>Sphingobacteriia	Pedobacter_ginsenosidimutans	DNA-phosphorothioation-system-sulfurtransferase-DndC-[Pedobacter-ginsenosidimutans]	687842
KRT17789.1	PAPS-reductase	GCA_001442625.1	434	Bacteria>FCB_group>Bacteroidetes/Chlorobi_group>Bacteroidetes>Sphingobacteriia	Pedobacter_ginsenosidimutans	sulfurtransferase-[Pedobacter-ginsenosidimutans]	687842
WP_138723727.1	PAPS-reductase	GCF_005925325.1	441	Bacteria>FCB_group>Bacteroidetes/Chlorobi_group>Bacteroidetes>Sphingobacteriia	Pedobacter_helvus	DNA-phosphorothioation-system-sulfurtransferase-DndC-[Pedobacter-helvus]	2563444
WP_133576849.1	PAPS-reductase	GCF_004362715.1	441	Bacteria>FCB_group>Bacteroidetes/Chlorobi_group>Bacteroidetes>Sphingobacteriia	Pedobacter_metabolipauper	DNA-phosphorothioation-system-sulfurtransferase-DndC-[Pedobacter-metabolipauper]	425513
WP_131527693.1	PAPS-reductase	GCF_004331735.1	440	Bacteria>FCB_group>Bacteroidetes/Chlorobi_group>Bacteroidetes>Sphingobacteriia	Pedobacter_psychrodurus	DNA-phosphorothioation-system-sulfurtransferase-DndC-[Pedobacter-psychrodurus]	2530456
WP_131595911.1	PAPS-reductase	GCF_004331695.1	441	Bacteria>FCB_group>Bacteroidetes/Chlorobi_group>Bacteroidetes>Sphingobacteriia	Pedobacter_psychroterrae	DNA-phosphorothioation-system-sulfurtransferase-DndC-[Pedobacter-psychroterrae]	2530453
WP_090886041.1	PAPS-reductase	GCF_900111155.1	440	Bacteria>FCB_group>Bacteroidetes/Chlorobi_group>Bacteroidetes>Sphingobacteriia	Pedobacter_rhizosphaerae	DNA-phosphorothioation-system-sulfurtransferase-DndC-[Pedobacter-rhizosphaerae]	390241
CAH0137772.1	PAPS-reductase	GCA_918698245.1	435	Bacteria>FCB_group>Bacteroidetes/Chlorobi_group>Bacteroidetes>Sphingobacteriia	Pedobacter_sp._Bi126	hypothetical-protein-SRABI126_00210-[Pedobacter-sp.-Bi126]	2822349
WP_219076556.1	PAPS-reductase	GCF_019317285.1	371	Bacteria>FCB_group>Bacteroidetes/Chlorobi_group>Bacteroidetes>Sphingobacteriia	Pedobacter_sp._D749	phosphoadenosine-phosphosulfate-reductase-family-protein-[Pedobacter-sp.-D749]	2856523
WP_193421911.1	PAPS-reductase	GCF_014947265.1	442	Bacteria>FCB_group>Bacteroidetes/Chlorobi_group>Bacteroidetes>Sphingobacteriia	Pedobacter_sp._MR2016-19	DNA-phosphorothioation-system-sulfurtransferase-DndC-[Pedobacter-sp.-MR2016-19]	2780089
WP_129715549.1	PAPS-reductase	GCF_004138205.1	439	Bacteria>FCB_group>Bacteroidetes/Chlorobi_group>Bacteroidetes>Sphingobacteriia	Pedobacter_sp._SYP-B3415	DNA-phosphorothioation-system-sulfurtransferase-DndC-[Pedobacter-sp.-SYP-B3415]	2496641
WP_090987184.1	PAPS-reductase	GCF_900111825.1	441	Bacteria>FCB_group>Bacteroidetes/Chlorobi_group>Bacteroidetes>Sphingobacteriia	Pedobacter_suwonensis	DNA-phosphorothioation-system-sulfurtransferase-DndC-[Pedobacter-suwonensis]	332999
WP_090497053.1	PAPS-reductase	GCF_900100705.1	435	Bacteria>FCB_group>Bacteroidetes/Chlorobi_group>Bacteroidetes>Sphingobacteriia	Pedobacter_terrae	DNA-phosphorothioation-system-sulfurtransferase-DndC-[Pedobacter-terrae]	405671
WP_138727951.1	PAPS-reductase	GCF_005925345.1	368	Bacteria>FCB_group>Bacteroidetes/Chlorobi_group>Bacteroidetes>Sphingobacteriia	Pedobacter_ureilyticus	phosphoadenosine-phosphosulfate-reductase-family-protein-[Pedobacter-ureilyticus]	1393051
WP_120183302.1	PAPS-reductase	GCF_003609575.1	439	Bacteria>FCB_group>Bacteroidetes/Chlorobi_group>Bacteroidetes>Sphingobacteriia	Pelobium_manganitolerans	DNA-phosphorothioation-system-sulfurtransferase-DndC-[Pelobium-manganitolerans]	1842495
MBK9457850.1	PAPS-reductase	GCA_016718845.1	445	Bacteria>FCB_group>Bacteroidetes/Chlorobi_group>Bacteroidetes>Sphingobacteriia	Sphingobacteriales_bacterium	DNA-phosphorothioation-system-sulfurtransferase-DndC-[Sphingobacteriales-bacterium]	2044944
MBK9458116.1	PAPS-reductase	GCA_016718845.1	367	Bacteria>FCB_group>Bacteroidetes/Chlorobi_group>Bacteroidetes>Sphingobacteriia	Sphingobacteriales_bacterium	phosphoadenosine-phosphosulfate-reductase-family-protein-[Sphingobacteriales-bacterium]	2044944
OYZ02007.1	PAPS-reductase	GCA_002281575.1	438	Bacteria>FCB_group>Bacteroidetes/Chlorobi_group>Bacteroidetes>Sphingobacteriia	Sphingobacteriia_bacterium_28-36-52	sulfurtransferase-[Sphingobacteriia-bacterium-28-36-52]	1970573
WP_103905138.1	PAPS-reductase	GCF_011046555.1	372	Bacteria>FCB_group>Bacteroidetes/Chlorobi_group>Bacteroidetes>Sphingobacteriia	Sphingobacterium_lactis	phosphoadenosine-phosphosulfate-reductase-family-protein-[Sphingobacterium-lactis]	797291
WP_187444081.1	PAPS-reductase	GCF_011043555.1	454	Bacteria>FCB_group>Bacteroidetes/Chlorobi_group>Bacteroidetes>Sphingobacteriia	Sphingobacterium	MULTISPECIES:-DNA-phosphorothioation-system-sulfurtransferase-DndC-[Sphingobacterium]	28453
WP_079640697.1	PAPS-reductase	GCF_900168125.1	453	Bacteria>FCB_group>Bacteroidetes/Chlorobi_group>Bacteroidetes>Sphingobacteriia	Sphingobacterium_nematocida	DNA-phosphorothioation-system-sulfurtransferase-DndC-[Sphingobacterium-nematocida]	1513896
WP_182498387.1	PAPS-reductase	GCF_014644335.1	435	Bacteria>FCB_group>Bacteroidetes/Chlorobi_group>Bacteroidetes>Sphingobacteriia	Sphingobacterium_soli	DNA-phosphorothioation-system-sulfurtransferase-DndC-[Sphingobacterium-soli]	1914757
GGE18159.1	PAPS-reductase	GCA_014644335.1	435	Bacteria>FCB_group>Bacteroidetes/Chlorobi_group>Bacteroidetes>Sphingobacteriia	Sphingobacterium_soli	hypothetical-protein-GCM10011516_14740-[Sphingobacterium-soli]	1914757
WP_066751080.1	PAPS-reductase	GCF_001586775.1	452	Bacteria>FCB_group>Bacteroidetes/Chlorobi_group>Bacteroidetes>Sphingobacteriia	Sphingobacterium_sp._CFCC_11742	DNA-phosphorothioation-system-sulfurtransferase-DndC-[Sphingobacterium-sp.-CFCC-11742]	1775560
WP_088161326.1	PAPS-reductase	GCF_002196555.1	369	Bacteria>FCB_group>Bacteroidetes/Chlorobi_group>Bacteroidetes>Sphingobacteriia	Sphingobacterium_sp._G1-14	phosphoadenosine-phosphosulfate-reductase-family-protein-[Sphingobacterium-sp.-G1-14]	2003121
TCR11093.1	PAPS-reductase	GCA_004342495.1	369	Bacteria>FCB_group>Bacteroidetes/Chlorobi_group>Bacteroidetes>Sphingobacteriia	Sphingobacterium_sp._JUb78	phosphoadenosine-phosphosulfate-reductase-family-protein-[Sphingobacterium-sp.-JUb78]	2485111
WP_132771225.1	PAPS-reductase	GCF_004342495.1	369	Bacteria>FCB_group>Bacteroidetes/Chlorobi_group>Bacteroidetes>Sphingobacteriia	Sphingobacterium_sp._JUb78	phosphoadenosine-phosphosulfate-reductase-family-protein-[Sphingobacterium-sp.-JUb78]	2485111
WP_230144105.1	PAPS-reductase	GCF_918697995.1	435	Bacteria>FCB_group>Bacteroidetes/Chlorobi_group>Bacteroidetes>Sphingobacteriia	unclassified_Pedobacter	MULTISPECIES:-DNA-phosphorothioation-system-sulfurtransferase-DndC-[unclassified-Pedobacter]	2628915
OQA97722.1	PAPS-reductase	GCA_002069975.1	443	Bacteria>FCB_group>Bacteroidetes/Chlorobi_group>Bacteroidetes>unclassified_Bacteroidetes	Bacteroidetes_bacterium_ADurb.Bin217	hypothetical-protein-BWY22_01169-[Bacteroidetes-bacterium-ADurb.Bin217]	1852809
KAB2892740.1	PAPS-reductase	GCA_008933715.1	378	Bacteria>FCB_group>Bacteroidetes/Chlorobi_group>Bacteroidetes>unclassified_Bacteroidetes	Bacteroidetes_bacterium	DNA-phosphorothioation-system-sulfurtransferase-DndC-[Bacteroidetes-bacterium]	1898104
MBK6338776.1	PAPS-reductase	GCA_016705125.1	441	Bacteria>FCB_group>Bacteroidetes/Chlorobi_group>Bacteroidetes>unclassified_Bacteroidetes	Bacteroidetes_bacterium	DNA-phosphorothioation-system-sulfurtransferase-DndC-[Bacteroidetes-bacterium]	1898104
MBK7908787.1	PAPS-reductase	GCA_016713765.1	441	Bacteria>FCB_group>Bacteroidetes/Chlorobi_group>Bacteroidetes>unclassified_Bacteroidetes	Bacteroidetes_bacterium	DNA-phosphorothioation-system-sulfurtransferase-DndC-[Bacteroidetes-bacterium]	1898104
MBL0080073.1	PAPS-reductase	GCA_016721275.1	442	Bacteria>FCB_group>Bacteroidetes/Chlorobi_group>Bacteroidetes>unclassified_Bacteroidetes	Bacteroidetes_bacterium	DNA-phosphorothioation-system-sulfurtransferase-DndC-[Bacteroidetes-bacterium]	1898104
MBL0310162.1	PAPS-reductase	GCA_016722375.1	439	Bacteria>FCB_group>Bacteroidetes/Chlorobi_group>Bacteroidetes>unclassified_Bacteroidetes	Bacteroidetes_bacterium	DNA-phosphorothioation-system-sulfurtransferase-DndC-[Bacteroidetes-bacterium]	1898104
MBN9483654.1	PAPS-reductase	GCA_017308155.1	436	Bacteria>FCB_group>Bacteroidetes/Chlorobi_group>Bacteroidetes>unclassified_Bacteroidetes	Bacteroidetes_bacterium	DNA-phosphorothioation-system-sulfurtransferase-DndC-[Bacteroidetes-bacterium]	1898104
MBU0489730.1	PAPS-reductase	GCA_018812245.1	444	Bacteria>FCB_group>Bacteroidetes/Chlorobi_group>Bacteroidetes>unclassified_Bacteroidetes	Bacteroidetes_bacterium	DNA-phosphorothioation-system-sulfurtransferase-DndC-[Bacteroidetes-bacterium]	1898104
OFX44496.1	PAPS-reductase	GCA_001769045.1	448	Bacteria>FCB_group>Bacteroidetes/Chlorobi_group>Bacteroidetes>unclassified_Bacteroidetes	Bacteroidetes_bacterium_GWA2_33_15	sulfurtransferase-[Bacteroidetes-bacterium-GWA2_33_15]	1797317
OFX87862.1	PAPS-reductase	GCA_001768405.1	443	Bacteria>FCB_group>Bacteroidetes/Chlorobi_group>Bacteroidetes>unclassified_Bacteroidetes	Bacteroidetes_bacterium_GWE2_32_14	sulfurtransferase-[Bacteroidetes-bacterium-GWE2_32_14]	1797332
OFY07327.1	PAPS-reductase	GCA_001769115.1	365	Bacteria>FCB_group>Bacteroidetes/Chlorobi_group>Bacteroidetes>unclassified_Bacteroidetes	Bacteroidetes_bacterium_GWE2_42_42	phosphoadenosine-phosphosulfate-reductase-[Bacteroidetes-bacterium-GWE2_42_42]	1797339
TAE69635.1	PAPS-reductase	GCA_004293265.1	375	Bacteria>FCB_group>Bacteroidetes/Chlorobi_group>Bacteroidetes>unclassified_Bacteroidetes	Bacteroidetes_bacterium	phosphoadenosine-phosphosulfate-reductase-[Bacteroidetes-bacterium]	1898104
MBK8330505.1	PAPS-reductase	GCA_016711825.1	366	Bacteria>FCB_group>Bacteroidetes/Chlorobi_group>Bacteroidetes>unclassified_Bacteroidetes	Bacteroidetes_bacterium	phosphoadenosine-phosphosulfate-reductase-family-protein-[Bacteroidetes-bacterium]	1898104
MBK8489230.1	PAPS-reductase	GCA_016711065.1	368	Bacteria>FCB_group>Bacteroidetes/Chlorobi_group>Bacteroidetes>unclassified_Bacteroidetes	Bacteroidetes_bacterium	phosphoadenosine-phosphosulfate-reductase-family-protein-[Bacteroidetes-bacterium]	1898104
MBM3454036.1	PAPS-reductase	GCA_016868905.1	367	Bacteria>FCB_group>Bacteroidetes/Chlorobi_group>Bacteroidetes>unclassified_Bacteroidetes	Bacteroidetes_bacterium	phosphoadenosine-phosphosulfate-reductase-family-protein-[Bacteroidetes-bacterium]	1898104
MBS1758866.1	PAPS-reductase	GCA_018268055.1	368	Bacteria>FCB_group>Bacteroidetes/Chlorobi_group>Bacteroidetes>unclassified_Bacteroidetes	Bacteroidetes_bacterium	phosphoadenosine-phosphosulfate-reductase-family-protein-[Bacteroidetes-bacterium]	1898104
MCB0536685.1	PAPS-reductase	GCA_020436205.1	367	Bacteria>FCB_group>Bacteroidetes/Chlorobi_group>Bacteroidetes>unclassified_Bacteroidetes	Bacteroidetes_bacterium	phosphoadenosine-phosphosulfate-reductase-family-protein-[Bacteroidetes-bacterium]	1898104
MBV5330005.1	PAPS-reductase	GCA_019162945.1	516	Bacteria>FCB_group>Bacteroidetes/Chlorobi_group>Chlorobi>Chlorobia	Chlorobium_sp.	DNA-phosphorothioation-system-sulfurtransferase-DndC-[Chlorobium-sp.]	1095
MBV5329171.1	PAPS-reductase	GCA_019162945.1	235	Bacteria>FCB_group>Bacteroidetes/Chlorobi_group>Chlorobi>Chlorobia	Chlorobium_sp.	hypothetical-protein-[Chlorobium-sp.]	1095
MBV5329172.1	PAPS-reductase	GCA_019162945.1	178	Bacteria>FCB_group>Bacteroidetes/Chlorobi_group>Chlorobi>Chlorobia	Chlorobium_sp.	phosphoadenosine-phosphosulfate-reductase-family-protein-[Chlorobium-sp.]	1095
MBN8544916.1	PAPS-reductase	GCA_017302895.1	437	Bacteria>FCB_group>Bacteroidetes/Chlorobi_group>Ignavibacteriae>Ignavibacteria	Ignavibacteria_bacterium	DNA-phosphorothioation-system-sulfurtransferase-DndC-[Ignavibacteria-bacterium]	2053306
NPV11874.1	PAPS-reductase	GCA_013177855.1	450	Bacteria>FCB_group>Bacteroidetes/Chlorobi_group>Ignavibacteriae>Ignavibacteria	Ignavibacteria_bacterium	DNA-phosphorothioation-system-sulfurtransferase-DndC-[Ignavibacteria-bacterium]	2053306
MBW7841604.1	PAPS-reductase	GCA_019455015.1	438	Bacteria>FCB_group>Bacteroidetes/Chlorobi_group>Ignavibacteriae>Ignavibacteria	Ignavibacterium_sp.	DNA-phosphorothioation-system-sulfurtransferase-DndC-[Ignavibacterium-sp.]	2651167
WP_022836018.1	PAPS-reductase	GCF_000419585.1	293	Bacteria>FCB_group>Bacteroidetes/Chlorobi_group>Rhodothermaeota>Rhodothermia	Salisaeta_longa	phosphoadenosine-phosphosulfate-reductase-family-protein-[Salisaeta-longa]	503170
KOY84527.1	PAPS-reductase	GCA_001281695.1	367	Bacteria>FCB_group>Bacteroidetes/Chlorobi_group>unclassified_Bacteroidetes/Chlorobi_group	bacterium_336/3	phosphoadenosine-phosphosulfate-reductase-[bacterium-336/3]	1664068
MBL7648117.1	PAPS-reductase	GCA_016791475.1	328	Bacteria>FCB_group>Candidatus_Hydrogenedentes>unclassified_Candidatus_Hydrogenedentes	Candidatus_Hydrogenedentes_bacterium	phosphoadenosine-phosphosulfate-reductase-family-protein-[Candidatus-Hydrogenedentes-bacterium]	2030809
MBT4129398.1	PAPS-reductase	GCA_018650795.1	285	Bacteria>FCB_group>Candidatus_Marinimicrobia	Candidatus_Marinimicrobia_bacterium	phosphoadenosine-phosphosulfate-reductase-family-protein-[Candidatus-Marinimicrobia-bacterium]	2026760
MBT4948013.1	PAPS-reductase	GCA_018659105.1	285	Bacteria>FCB_group>Candidatus_Marinimicrobia	Candidatus_Marinimicrobia_bacterium	phosphoadenosine-phosphosulfate-reductase-family-protein-[Candidatus-Marinimicrobia-bacterium]	2026760
MBO5533294.1	PAPS-reductase	GCA_017626945.1	579	Bacteria>FCB_group>Fibrobacteres>Fibrobacteria>Fibrobacterales	Fibrobacter_sp.	phosphoadenosine-phosphosulfate-reductase-family-protein-[Fibrobacter-sp.]	35828
MBO7059481.1	PAPS-reductase	GCA_017642335.1	579	Bacteria>FCB_group>Fibrobacteres>Fibrobacteria>Fibrobacterales	Fibrobacter_sp.	phosphoadenosine-phosphosulfate-reductase-family-protein-[Fibrobacter-sp.]	35828
MBQ9224763.1	PAPS-reductase	GCA_017470355.1	579	Bacteria>FCB_group>Fibrobacteres>Fibrobacteria>Fibrobacterales	Fibrobacter_sp.	phosphoadenosine-phosphosulfate-reductase-family-protein-[Fibrobacter-sp.]	35828
WP_014546338.1	PAPS-reductase	GCF_000024665.1	579	Bacteria>FCB_group>Fibrobacteres>Fibrobacteria>Fibrobacterales	Fibrobacter_succinogenes	phosphoadenosine-phosphosulfate-reductase-family-protein-[Fibrobacter-succinogenes]	833
ADL26678.1	PAPS-reductase	GCA_000146505.1	579	Bacteria>FCB_group>Fibrobacteres>Fibrobacteria>Fibrobacterales	Fibrobacter_succinogenes_subsp._succinogenes_S85	phosphoadenosine-phosphosulfate-reductase-domain-protein-[Fibrobacter-succinogenes-subsp.-succinogenes-S85]	59374
MBT4187591.1	PAPS-reductase	GCA_018651425.1	522	Bacteria>FCB_group>Gemmatimonadetes>Gemmatimonadetes>Gemmatimonadales	Gemmatimonadales_bacterium	DNA-phosphorothioation-system-sulfurtransferase-DndC-[Gemmatimonadales-bacterium]	2448054
MXW05561.1	PAPS-reductase	GCA_009835325.1	513	Bacteria>FCB_group>Gemmatimonadetes>unclassified_Gemmatimonadetes	Gemmatimonadetes_bacterium	DNA-phosphorothioation-system-sulfurtransferase-DndC-[Gemmatimonadetes-bacterium]	2026742
MXX38512.1	PAPS-reductase	GCA_009835785.1	459	Bacteria>FCB_group>Gemmatimonadetes>unclassified_Gemmatimonadetes	Gemmatimonadetes_bacterium	DNA-phosphorothioation-system-sulfurtransferase-DndC-[Gemmatimonadetes-bacterium]	2026742
MXY84770.1	PAPS-reductase	GCA_009837645.1	459	Bacteria>FCB_group>Gemmatimonadetes>unclassified_Gemmatimonadetes	Gemmatimonadetes_bacterium	DNA-phosphorothioation-system-sulfurtransferase-DndC-[Gemmatimonadetes-bacterium]	2026742
MYB66983.1	PAPS-reductase	GCA_009840005.1	459	Bacteria>FCB_group>Gemmatimonadetes>unclassified_Gemmatimonadetes	Gemmatimonadetes_bacterium	DNA-phosphorothioation-system-sulfurtransferase-DndC-[Gemmatimonadetes-bacterium]	2026742
MBA4157583.1	PAPS-reductase	GCA_013820915.1	270	Bacteria>FCB_group>Gemmatimonadetes>unclassified_Gemmatimonadetes	Gemmatimonadetes_bacterium	phosphoadenosine-phosphosulfate-reductase-family-protein-[Gemmatimonadetes-bacterium]	2026742
MYB71145.1	PAPS-reductase	GCA_009840005.1	282	Bacteria>FCB_group>Gemmatimonadetes>unclassified_Gemmatimonadetes	Gemmatimonadetes_bacterium	phosphoadenosine-phosphosulfate-reductase-family-protein-[Gemmatimonadetes-bacterium]	2026742
MBN2837858.1	PAPS-reductase	GCA_016938425.1	471	Bacteria>Fusobacteria>Fusobacteriia>Fusobacteriales>Fusobacteriaceae	Fusobacteriaceae_bacterium	DNA-phosphorothioation-system-sulfurtransferase-DndC-[Fusobacteriaceae-bacterium]	2053555
WP_204689410.1	PAPS-reductase	GCF_016902555.1	448	Bacteria>Fusobacteria>Fusobacteriia>Fusobacteriales>Fusobacteriaceae	Fusobacterium_mortiferum	phosphoadenosine-phosphosulfate-reductase-family-protein-[Fusobacterium-mortiferum]	850
WP_175613344.1	PAPS-reductase	GCF_903181465.1	438	Bacteria>Fusobacteria>Fusobacteriia>Fusobacteriales>Fusobacteriaceae	Fusobacterium_mortiferum	phosphoadenosine-phosphosulfate-reductase-family-protein,-partial-[Fusobacterium-mortiferum]	850
WP_005959448.1	PAPS-reductase	GCF_000262225.1	573	Bacteria>Fusobacteria>Fusobacteriia>Fusobacteriales>Fusobacteriaceae	Fusobacterium_necrophorum	phosphoadenosine-phosphosulfate-reductase-family-protein-[Fusobacterium-necrophorum]	859
WP_106895172.1	PAPS-reductase	GCF_003019715.1	573	Bacteria>Fusobacteria>Fusobacteriia>Fusobacteriales>Fusobacteriaceae	Fusobacterium_necrophorum	phosphoadenosine-phosphosulfate-reductase-family-protein-[Fusobacterium-necrophorum]	859
WP_011016392.1	PAPS-reductase	GCF_003019295.1	574	Bacteria>Fusobacteria>Fusobacteriia>Fusobacteriales>Fusobacteriaceae	Fusobacterium_nucleatum	phosphoadenosine-phosphosulfate-reductase-family-protein-[Fusobacterium-nucleatum]	851
WP_170185954.1	PAPS-reductase	GCF_012843705.1	572	Bacteria>Fusobacteria>Fusobacteriia>Fusobacteriales>Fusobacteriaceae	Fusobacterium_sp._FSA-380-WT-3A	phosphoadenosine-phosphosulfate-reductase-family-protein-[Fusobacterium-sp.-FSA-380-WT-3A]	2725304
WP_081982637.1	PAPS-reductase	GCF_000800875.1	547	Bacteria>Fusobacteria>Fusobacteriia>Fusobacteriales>Fusobacteriaceae	unclassified_Cetobacterium	MULTISPECIES:-phosphoadenosine-phosphosulfate-reductase-family-protein-[unclassified-Cetobacterium]	2630983
HIL26299.1	PAPS-reductase	GCA_015660105.1	508	Bacteria>Nitrospinae/Tectomicrobia_group>Nitrospinae>Nitrospinia>Nitrospinales	Nitrospinaceae_bacterium	DNA-phosphorothioation-system-sulfurtransferase-DndC-[Nitrospinaceae-bacterium]	2358456
MBC8285043.1	PAPS-reductase	GCA_014382485.1	521	Bacteria>Nitrospinae/Tectomicrobia_group>Nitrospinae>unclassified_Nitrospinae	Nitrospinae_bacterium	DNA-phosphorothioation-system-sulfurtransferase-DndC-[Nitrospinae-bacterium]	2026769
WP_202882116.1	PAPS-reductase	GCF_012184425.1	465	Bacteria>Nitrospirae>Nitrospira>Candidatus_Troglogloeales>Candidatus_Manganitrophaceae	Candidatus_Manganitrophus_noduliformans	DNA-phosphorothioation-system-sulfurtransferase-DndC-[Candidatus-Manganitrophus-noduliformans]	2606439
RJQ43051.1	PAPS-reductase	GCA_003599425.1	329	Bacteria>Nitrospirae>Nitrospira>Nitrospirales>Nitrospiraceae	Nitrospiraceae_bacterium	MAG:-DNA-phosphorothioation-system-sulfurtransferase-DndC-[Nitrospiraceae-bacterium]	2026770
MBF0488888.1	PAPS-reductase	GCA_015233045.1	365	Bacteria>Nitrospirae>unclassified_Nitrospirae	Nitrospirae_bacterium	DNA-phosphorothioation-system-sulfurtransferase-DndC-[Nitrospirae-bacterium]	2026887
MBF0566741.1	PAPS-reductase	GCA_015233615.1	452	Bacteria>Nitrospirae>unclassified_Nitrospirae	Nitrospirae_bacterium	DNA-phosphorothioation-system-sulfurtransferase-DndC-[Nitrospirae-bacterium]	2026887
MBI2058416.1	PAPS-reductase	GCA_016180645.1	431	Bacteria>Nitrospirae>unclassified_Nitrospirae	Nitrospirae_bacterium	DNA-phosphorothioation-system-sulfurtransferase-DndC-[Nitrospirae-bacterium]	2026887
MBI4849887.1	PAPS-reductase	GCA_016213945.1	245	Bacteria>Nitrospirae>unclassified_Nitrospirae	Nitrospirae_bacterium	DNA-phosphorothioation-system-sulfurtransferase-DndC-[Nitrospirae-bacterium]	2026887
MBI4849886.1	PAPS-reductase	GCA_016213945.1	136	Bacteria>Nitrospirae>unclassified_Nitrospirae	Nitrospirae_bacterium	hypothetical-protein-[Nitrospirae-bacterium]	2026887
MBE7562009.1	PAPS-reductase	GCA_015100135.1	485	Bacteria>Proteobacteria>Acidithiobacillia>Acidithiobacillales>Acidithiobacillaceae	Acidithiobacillus_sp._HP-6	DNA-phosphorothioation-system-sulfurtransferase-DndC-[Acidithiobacillus-sp.-HP-6]	2697655
WP_193649873.1	PAPS-reductase	GCF_015100135.1	485	Bacteria>Proteobacteria>Acidithiobacillia>Acidithiobacillales>Acidithiobacillaceae	unclassified_Acidithiobacillus	MULTISPECIES:-DNA-phosphorothioation-system-sulfurtransferase-DndC-[unclassified-Acidithiobacillus]	2614800
WP_023458139.1	PAPS-reductase	GCF_000495815.1	378	Bacteria>Proteobacteria>Alphaproteobacteria>Caulobacterales>Caulobacteraceae	Asticcacaulis_sp._AC466	DNA-phosphorothioation-system-sulfurtransferase-DndC-[Asticcacaulis-sp.-AC466]	1282362
ESQ84197.1	PAPS-reductase	GCA_000495815.1	378	Bacteria>Proteobacteria>Alphaproteobacteria>Caulobacterales>Caulobacteraceae	Asticcacaulis_sp._AC466	hypothetical-protein-AEAC466_10660-[Asticcacaulis-sp.-AC466]	1282362
WP_168045873.1	PAPS-reductase	GCF_011927945.1	275	Bacteria>Proteobacteria>Alphaproteobacteria>Caulobacterales>Caulobacteraceae	Brevundimonas_alba	phosphoadenosine-phosphosulfate-reductase-family-protein-[Brevundimonas-alba]	74314
WP_125255816.1	PAPS-reductase	GCF_003934285.1	275	Bacteria>Proteobacteria>Alphaproteobacteria>Caulobacterales>Caulobacteraceae	Brevundimonas_fluminis	phosphoadenosine-phosphosulfate-reductase-family-protein-[Brevundimonas-fluminis]	2487274
QTC90324.1	PAPS-reductase	GCA_017487405.1	487	Bacteria>Proteobacteria>Alphaproteobacteria>Caulobacterales>Caulobacteraceae	Brevundimonas_goettingensis	DNA-phosphorothioation-system-sulfurtransferase-DndC-[Brevundimonas-goettingensis]	2774190
WP_207868743.1	PAPS-reductase	GCF_017487405.1	487	Bacteria>Proteobacteria>Alphaproteobacteria>Caulobacterales>Caulobacteraceae	Brevundimonas_goettingensis	DNA-phosphorothioation-system-sulfurtransferase-DndC-[Brevundimonas-goettingensis]	2774190
WP_183214008.1	PAPS-reductase	GCF_014199945.1	518	Bacteria>Proteobacteria>Alphaproteobacteria>Caulobacterales>Caulobacteraceae	Brevundimonas_variabilis	DNA-phosphorothioation-system-sulfurtransferase-DndC-[Brevundimonas-variabilis]	74312
WP_007665261.1	PAPS-reductase	GCF_000281955.1	524	Bacteria>Proteobacteria>Alphaproteobacteria>Caulobacterales>Caulobacteraceae	Caulobacter_sp._AP07	DNA-phosphorothioation-system-sulfurtransferase-DndC-[Caulobacter-sp.-AP07]	1144304
MBP7703729.1	PAPS-reductase	GCA_017999465.1	275	Bacteria>Proteobacteria>Alphaproteobacteria>Caulobacterales>Caulobacteraceae	Caulobacter_sp.	phosphoadenosine-phosphosulfate-reductase-family-protein-[Caulobacter-sp.]	78
WP_188519108.1	PAPS-reductase	-	275	Bacteria>Proteobacteria>Alphaproteobacteria>Hyphomicrobiales>Alsobacteraceae	Alsobacter_metallidurans	phosphoadenosine-phosphosulfate-reductase-family-protein-[Alsobacter-metallidurans]	340221
SDO86101.1	PAPS-reductase	GCA_900104035.1	379	Bacteria>Proteobacteria>Alphaproteobacteria>Hyphomicrobiales>Aurantimonadaceae	Aureimonas_jatrophae	DNA-sulfur-modification-protein-DndC-[Aureimonas-jatrophae]	1166073
WP_082652779.1	PAPS-reductase	GCF_001463705.1	379	Bacteria>Proteobacteria>Alphaproteobacteria>Hyphomicrobiales>Aurantimonadaceae	Aureimonas_sp._AU12	DNA-phosphorothioation-system-sulfurtransferase-DndC-[Aureimonas-sp.-AU12]	1638161
WP_082640107.1	PAPS-reductase	GCF_001463725.1	379	Bacteria>Proteobacteria>Alphaproteobacteria>Hyphomicrobiales>Aurantimonadaceae	Aureimonas_sp._AU20	DNA-phosphorothioation-system-sulfurtransferase-DndC-[Aureimonas-sp.-AU20]	1349819
WP_117412168.1	PAPS-reductase	GCF_003432385.1	384	Bacteria>Proteobacteria>Alphaproteobacteria>Hyphomicrobiales>Breoghaniaceae	Breoghania_sp._L-A4	DNA-phosphorothioation-system-sulfurtransferase-DndC-[Breoghania-sp.-L-A4]	2304600
WP_236854463.1	PAPS-reductase	GCF_001652485.1	350	Bacteria>Proteobacteria>Alphaproteobacteria>Hyphomicrobiales>Brucellaceae	Brucella_pseudogrignonensis	DNA-phosphorothioation-system-sulfurtransferase-DndC-[Brucella-pseudogrignonensis]	419475
WP_237853934.1	PAPS-reductase	GCF_022024335.1	278	Bacteria>Proteobacteria>Alphaproteobacteria>Hyphomicrobiales>Brucellaceae	Brucella_pseudogrignonensis	phosphoadenosine-phosphosulfate-reductase-family-protein-[Brucella-pseudogrignonensis]	419475

Accession	Annotation	GCA	Length	Phyletic.spread	Strain.name	Defline	Species.Taxid
WP_121539138.1	PAPS-reductase	GCF_003664555.1	378	Bacteria>Proteobacteria>Alphaproteobacteria>Hyphomicrobiales>Brucellaceae	[Ochrobactrum]_soli	DNA-phosphorothioation-system-sulfurtransferase-DndC-[[Ochrobactrum]	2448455
WP_235935498.1	PAPS-reductase	GCF_011058215.1	287	Bacteria>Proteobacteria>Alphaproteobacteria>Hyphomicrobiales>Devosiaceae	Devosia_aurantiaca	DNA-phosphorothioation-system-sulfurtransferase-DndC-[Devosia-aurantiaca]	2714858
WP_157290573.1	PAPS-reductase	GCF_009758415.1	278	Bacteria>Proteobacteria>Alphaproteobacteria>Hyphomicrobiales>Devosiaceae	Devosia_marina	phosphoadenosine-phosphosulfate-reductase-family-protein-[Devosia-marina]	2683198
WP_088343788.1	PAPS-reductase	GCF_002198685.1	515	Bacteria>Proteobacteria>Alphaproteobacteria>Hyphomicrobiales>Hyphomicrobiaceae	Rhodomicrobium	MULTISPECIES:-DNA-phosphorothioation-system-sulfurtransferase-DndC-[Rhodomicrobium]	1068
CAA2136840.1	PAPS-reductase	-	498	Bacteria>Proteobacteria>Alphaproteobacteria>Hyphomicrobiales>Methylobacteriaceae	Methylobacterium_bullatum	hypothetical-protein-MBLL_00363-[Methylobacterium-bullatum]	570505
WP_058619946.1	PAPS-reductase	GCF_001476615.1	495	Bacteria>Proteobacteria>Alphaproteobacteria>Hyphomicrobiales>Methylobacteriaceae	Methylobacterium_indicum	DNA-phosphorothioation-system-sulfurtransferase-DndC-[Methylobacterium-indicum]	1775910
WP_010682104.1	PAPS-reductase	GCF_000364445.2	369	Bacteria>Proteobacteria>Alphaproteobacteria>Hyphomicrobiales>Methylobacteriaceae	Methylobacterium_mesophilicum	DNA-phosphorothioation-system-sulfurtransferase-DndC-[Methylobacterium-mesophilicum]	39956
WP_127729010.1	PAPS-reductase	GCF_004004555.2	523	Bacteria>Proteobacteria>Alphaproteobacteria>Hyphomicrobiales>Methylobacteriaceae	Methylobacterium_oryzihabitans	DNA-phosphorothioation-system-sulfurtransferase-DndC-[Methylobacterium-sp.-TER-1]	2499852
WP_093567328.1	PAPS-reductase	GCF_900116815.1	277	Bacteria>Proteobacteria>Alphaproteobacteria>Hyphomicrobiales>Methylobacteriaceae	Methylobacterium_sp._174MFSha1.1	phosphoadenosine-phosphosulfate-reductase-family-protein-[Methylobacterium-sp.-174MFSha1.1]	1502749
SFU78195.1	PAPS-reductase	GCA_900116815.1	277	Bacteria>Proteobacteria>Alphaproteobacteria>Hyphomicrobiales>Methylobacteriaceae	Methylobacterium_sp._174MFSha1.1	Phosphoadenosine-phosphosulfate-reductase-family-protein-[Methylobacterium-sp.-174MFSha1.1]	1502749
MBP33149.1	PAPS-reductase	GCA_002727035.1	526	Bacteria>Proteobacteria>Alphaproteobacteria>Hyphomicrobiales>Methylobacteriaceae	Methylobacterium_sp.	DNA-phosphorothioation-system-sulfurtransferase-DndC-[Methylobacterium-sp.]	409
WP_058189970.1	PAPS-reductase	GCF_001455965.1	369	Bacteria>Proteobacteria>Alphaproteobacteria>Hyphomicrobiales>Methylobacteriaceae	Methylobacterium_sp._GXS13	DNA-phosphorothioation-system-sulfurtransferase-DndC-[Methylobacterium-sp.-GXS13]	1730094
KQQ17383.1	PAPS-reductase	GCA_001423385.1	369	Bacteria>Proteobacteria>Alphaproteobacteria>Hyphomicrobiales>Methylobacteriaceae	Methylobacterium_sp._Leaf121	sulfurtransferase-[Methylobacterium-sp.-Leaf121]	1736262
WP_012754231.1	PAPS-reductase	GCF_000022685.1	369	Bacteria>Proteobacteria>Alphaproteobacteria>Hyphomicrobiales>Methylobacteriaceae	Methylorubrum_extorquens	DNA-phosphorothioation-system-sulfurtransferase-DndC-[Methylorubrum-extorquens]	408
WP_238508664.1	PAPS-reductase	GCF_022214735.1	499	Bacteria>Proteobacteria>Alphaproteobacteria>Hyphomicrobiales>Methylobacteriaceae	Methylorubrum_extorquens	DNA-phosphorothioation-system-sulfurtransferase-DndC-[Methylorubrum-extorquens]	408
WP_247523770.1	PAPS-reductase	GCF_023073955.1	488	Bacteria>Proteobacteria>Alphaproteobacteria>Hyphomicrobiales>Nitrobacteraceae	Bradyrhizobium_sp._145	DNA-phosphorothioation-system-sulfurtransferase-DndC-[Bradyrhizobium-sp.-145]	2782621
WP_247531341.1	PAPS-reductase	GCF_023073775.1	488	Bacteria>Proteobacteria>Alphaproteobacteria>Hyphomicrobiales>Nitrobacteraceae	Bradyrhizobium_sp._162	DNA-phosphorothioation-system-sulfurtransferase-DndC-[Bradyrhizobium-sp.-162]	2782635
WP_208069158.1	PAPS-reductase	GCF_017565685.3	497	Bacteria>Proteobacteria>Alphaproteobacteria>Hyphomicrobiales>Nitrobacteraceae	Bradyrhizobium_sp._38S5	DNA-phosphorothioation-system-sulfurtransferase-DndC-[Bradyrhizobium-sp.-38S5]	2819348
MBR1285375.1	PAPS-reductase	GCA_018130125.1	547	Bacteria>Proteobacteria>Alphaproteobacteria>Hyphomicrobiales>Nitrobacteraceae	Bradyrhizobium_sp._AUGA_SZCCT0177	DNA-phosphorothioation-system-sulfurtransferase-DndC-[Bradyrhizobium-sp.-AUGA-SZCCT0177]	2807665
WP_212267800.1	PAPS-reductase	GCF_018130125.1	547	Bacteria>Proteobacteria>Alphaproteobacteria>Hyphomicrobiales>Nitrobacteraceae	Bradyrhizobium_sp._AUGA_SZCCT0177	DNA-phosphorothioation-system-sulfurtransferase-DndC-[Bradyrhizobium-sp.-AUGA-SZCCT0177]	2807665
WP_247356727.1	PAPS-reductase	GCF_023100905.1	488	Bacteria>Proteobacteria>Alphaproteobacteria>Hyphomicrobiales>Nitrobacteraceae	unclassified_Bradyrhizobium	MULTISPECIES:-DNA-phosphorothioation-system-sulfurtransferase-DndC-[unclassified-Bradyrhizobium]	2631580
WP_011578999.1	PAPS-reductase	GCF_011317445.1	495	Bacteria>Proteobacteria>Alphaproteobacteria>Hyphomicrobiales>Phyllobacteriaceae	Chelativorans	MULTISPECIES:-DNA-phosphorothioation-system-sulfurtransferase-DndC-[Chelativorans]	449972
WP_006204722.1	PAPS-reductase	-	378	Bacteria>Proteobacteria>Alphaproteobacteria>Hyphomicrobiales>Phyllobacteriaceae	Mesorhizobium_amorphae	DNA-phosphorothioation-system-sulfurtransferase-DndC-[Mesorhizobium-amorphae]	71433
WP_192355956.1	PAPS-reductase	GCF_014843885.1	281	Bacteria>Proteobacteria>Alphaproteobacteria>Hyphomicrobiales>Phyllobacteriaceae	Mesorhizobium_mediterraneum	phosphoadenosine-phosphosulfate-reductase-family-protein-[Mesorhizobium-mediterraneum]	43617
WP_095795932.1	PAPS-reductase	GCF_002294705.1	495	Bacteria>Proteobacteria>Alphaproteobacteria>Hyphomicrobiales>Phyllobacteriaceae	Mesorhizobium	MULTISPECIES:-DNA-phosphorothioation-system-sulfurtransferase-DndC-[Mesorhizobium]	68287
WP_141841103.1	PAPS-reductase	-	494	Bacteria>Proteobacteria>Alphaproteobacteria>Hyphomicrobiales>Phyllobacteriaceae	Mesorhizobium_sp._B2-4-15	DNA-phosphorothioation-system-sulfurtransferase-DndC-[Mesorhizobium-sp.-B2-4-15]	2589934
WP_119819810.1	PAPS-reductase	GCF_003590645.1	494	Bacteria>Proteobacteria>Alphaproteobacteria>Hyphomicrobiales>Phyllobacteriaceae	Mesorhizobium_sp._DCY119	DNA-phosphorothioation-system-sulfurtransferase-DndC-[Mesorhizobium-sp.-DCY119]	2108445
RWA59262.1	PAPS-reductase	GCA_004016595.1	376	Bacteria>Proteobacteria>Alphaproteobacteria>Hyphomicrobiales>Phyllobacteriaceae	Mesorhizobium_sp.	DNA-phosphorothioation-system-sulfurtransferase-DndC-[Mesorhizobium-sp.]	1871066
RWA97445.1	PAPS-reductase	GCA_004016835.1	379	Bacteria>Proteobacteria>Alphaproteobacteria>Hyphomicrobiales>Phyllobacteriaceae	Mesorhizobium_sp.	DNA-phosphorothioation-system-sulfurtransferase-DndC-[Mesorhizobium-sp.]	1871066
RWD27537.1	PAPS-reductase	GCA_004017075.1	377	Bacteria>Proteobacteria>Alphaproteobacteria>Hyphomicrobiales>Phyllobacteriaceae	Mesorhizobium_sp.	DNA-phosphorothioation-system-sulfurtransferase-DndC-[Mesorhizobium-sp.]	1871066
RWL89316.1	PAPS-reductase	GCA_004020305.1	495	Bacteria>Proteobacteria>Alphaproteobacteria>Hyphomicrobiales>Phyllobacteriaceae	Mesorhizobium_sp.	DNA-phosphorothioation-system-sulfurtransferase-DndC-[Mesorhizobium-sp.]	1871066
RWN94627.1	PAPS-reductase	GCA_004021525.1	495	Bacteria>Proteobacteria>Alphaproteobacteria>Hyphomicrobiales>Phyllobacteriaceae	Mesorhizobium_sp.	DNA-phosphorothioation-system-sulfurtransferase-DndC-[Mesorhizobium-sp.]	1871066
RWO22206.1	PAPS-reductase	GCA_004021465.1	495	Bacteria>Proteobacteria>Alphaproteobacteria>Hyphomicrobiales>Phyllobacteriaceae	Mesorhizobium_sp.	DNA-phosphorothioation-system-sulfurtransferase-DndC-[Mesorhizobium-sp.]	1871066
RWQ46781.1	PAPS-reductase	GCA_004021735.1	495	Bacteria>Proteobacteria>Alphaproteobacteria>Hyphomicrobiales>Phyllobacteriaceae	Mesorhizobium_sp.	DNA-phosphorothioation-system-sulfurtransferase-DndC-[Mesorhizobium-sp.]	1871066
TIM30553.1	PAPS-reductase	GCA_004961485.1	376	Bacteria>Proteobacteria>Alphaproteobacteria>Hyphomicrobiales>Phyllobacteriaceae	Mesorhizobium_sp.	DNA-phosphorothioation-system-sulfurtransferase-DndC-[Mesorhizobium-sp.]	1871066
TIN32545.1	PAPS-reductase	GCA_004961445.1	495	Bacteria>Proteobacteria>Alphaproteobacteria>Hyphomicrobiales>Phyllobacteriaceae	Mesorhizobium_sp.	DNA-phosphorothioation-system-sulfurtransferase-DndC-[Mesorhizobium-sp.]	1871066
TIP92998.1	PAPS-reductase	GCA_004962305.1	495	Bacteria>Proteobacteria>Alphaproteobacteria>Hyphomicrobiales>Phyllobacteriaceae	Mesorhizobium_sp.	DNA-phosphorothioation-system-sulfurtransferase-DndC-[Mesorhizobium-sp.]	1871066
TIW10978.1	PAPS-reductase	GCA_005063115.1	376	Bacteria>Proteobacteria>Alphaproteobacteria>Hyphomicrobiales>Phyllobacteriaceae	Mesorhizobium_sp.	DNA-phosphorothioation-system-sulfurtransferase-DndC-[Mesorhizobium-sp.]	1871066
RWP03129.1	PAPS-reductase	GCA_004021085.1	278	Bacteria>Proteobacteria>Alphaproteobacteria>Hyphomicrobiales>Phyllobacteriaceae	Mesorhizobium_sp.	hypothetical-protein-EOQ99_21710-[Mesorhizobium-sp.]	1871066
RWQ12714.1	PAPS-reductase	GCA_004021905.1	275	Bacteria>Proteobacteria>Alphaproteobacteria>Hyphomicrobiales>Phyllobacteriaceae	Mesorhizobium_sp.	hypothetical-protein-EOR92_32900-[Mesorhizobium-sp.]	1871066
WP_201415355.1	PAPS-reductase	GCF_016591715.1	374	Bacteria>Proteobacteria>Alphaproteobacteria>Hyphomicrobiales>Phyllobacteriaceae	Mesorhizobium_sp._J8	DNA-phosphorothioation-system-sulfurtransferase-DndC-[Mesorhizobium-sp.-J8]	2777475
WP_071071678.1	PAPS-reductase	GCF_001854795.1	522	Bacteria>Proteobacteria>Alphaproteobacteria>Hyphomicrobiales>Phyllobacteriaceae	Mesorhizobium_sp._LCM_4577	DNA-phosphorothioation-system-sulfurtransferase-DndC-[Mesorhizobium-sp.-LCM-4577]	1848288
WP_128371610.1	PAPS-reductase	GCF_004023325.1	376	Bacteria>Proteobacteria>Alphaproteobacteria>Hyphomicrobiales>Phyllobacteriaceae	Mesorhizobium_sp._M1A.F.Ca.ET.072.01.1.1	DNA-phosphorothioation-system-sulfurtransferase-DndC-[Mesorhizobium-sp.-M1A.F.Ca.ET.072.01.1.1]	2496753
WP_127221119.1	PAPS-reductase	GCF_003998155.1	376	Bacteria>Proteobacteria>Alphaproteobacteria>Hyphomicrobiales>Phyllobacteriaceae	Mesorhizobium_sp._M1E.F.Ca.ET.063.01.1.1	DNA-phosphorothioation-system-sulfurtransferase-DndC-[Mesorhizobium-sp.-M1E.F.Ca.ET.063.01.1.1]	2496750
WP_127281906.1	PAPS-reductase	GCF_003998955.1	281	Bacteria>Proteobacteria>Alphaproteobacteria>Hyphomicrobiales>Phyllobacteriaceae	Mesorhizobium_sp._M4B.F.Ca.ET.017.02.2.1	phosphoadenosine-phosphosulfate-reductase-family-protein-[Mesorhizobium-sp.-M4B.F.Ca.ET.017.02.2.1]	2496649
WP_149801934.1	PAPS-reductase	GCF_900115245.1	278	Bacteria>Proteobacteria>Alphaproteobacteria>Hyphomicrobiales>Phyllobacteriaceae	Mesorhizobium_sp._NFR06	phosphoadenosine-phosphosulfate-reductase-family-protein-[Mesorhizobium-sp.-NFR06]	1566290
WP_036259481.1	PAPS-reductase	GCF_000427725.1	495	Bacteria>Proteobacteria>Alphaproteobacteria>Hyphomicrobiales>Phyllobacteriaceae	Mesorhizobium_sp._WSM3224	DNA-phosphorothioation-system-sulfurtransferase-DndC-[Mesorhizobium-sp.-WSM3224]	1040986
WP_124998088.1	PAPS-reductase	GCF_003863365.1	384	Bacteria>Proteobacteria>Alphaproteobacteria>Hyphomicrobiales>Phyllobacteriaceae	Mesorhizobium_tamadayense	DNA-phosphorothioation-system-sulfurtransferase-DndC-[Mesorhizobium-tamadayense]	425306
WP_095517565.1	PAPS-reductase	GCF_002284535.1	379	Bacteria>Proteobacteria>Alphaproteobacteria>Hyphomicrobiales>Phyllobacteriaceae	Mesorhizobium_wenxiniae	DNA-phosphorothioation-system-sulfurtransferase-DndC-[Mesorhizobium-wenxiniae]	2014805
WP_175276875.1	PAPS-reductase	GCF_013358405.1	384	Bacteria>Proteobacteria>Alphaproteobacteria>Hyphomicrobiales>Phyllobacteriaceae	Oricola_thermophila	DNA-phosphorothioation-system-sulfurtransferase-DndC-[Oricola-thermophila]	2742145
WP_235981908.1	PAPS-reductase	GCF_015904235.1	500	Bacteria>Proteobacteria>Alphaproteobacteria>Hyphomicrobiales>Pleomorphomonadaceae	Methylobrevis_albus	DNA-phosphorothioation-system-sulfurtransferase-DndC-[Methylobrevis-albus]	2793297
WP_059755075.1	PAPS-reductase	GCF_003610845.1	492	Bacteria>Proteobacteria>Alphaproteobacteria>Hyphomicrobiales>Rhizobiaceae	Agrobacterium_deltaense	MULTISPECIES:-DNA-phosphorothioation-system-sulfurtransferase-DndC-[Agrobacterium]	1183412
WP_037171394.1	PAPS-reductase	GCF_005145045.1	511	Bacteria>Proteobacteria>Alphaproteobacteria>Hyphomicrobiales>Rhizobiaceae	Agrobacterium_larrymoorei	DNA-phosphorothioation-system-sulfurtransferase-DndC-[Agrobacterium-larrymoorei]	160699
WP_174163590.1	PAPS-reductase	GCF_013321135.1	281	Bacteria>Proteobacteria>Alphaproteobacteria>Hyphomicrobiales>Rhizobiaceae	Agrobacterium_rhizogenes	phosphoadenosine-phosphosulfate-reductase-family-protein-[Agrobacterium-rhizogenes]	359
WP_019566302.1	PAPS-reductase	GCF_000381165.1	493	Bacteria>Proteobacteria>Alphaproteobacteria>Hyphomicrobiales>Rhizobiaceae	Agrobacterium_sp._10MFCol1.1	DNA-phosphorothioation-system-sulfurtransferase-DndC-[Agrobacterium-sp.-10MFCol1.1]	1150775
KVK54338.1	PAPS-reductase	GCA_001526505.1	493	Bacteria>Proteobacteria>Alphaproteobacteria>Hyphomicrobiales>Rhizobiaceae	Agrobacterium_sp._D14	hypothetical-protein-L901_18375-[Agrobacterium-sp.-D14]	1336743
WP_174056001.1	PAPS-reductase	GCF_013317565.1	361	Bacteria>Proteobacteria>Alphaproteobacteria>Hyphomicrobiales>Rhizobiaceae	Agrobacterium_tumefaciens	DNA-phosphorothioation-system-sulfurtransferase-DndC-[Agrobacterium-tumefaciens]	358
WP_236742501.1	PAPS-reductase	GCF_001975795.1	257	Bacteria>Proteobacteria>Alphaproteobacteria>Hyphomicrobiales>Rhizobiaceae	Agrobacterium_tumefaciens	hypothetical-protein-[Agrobacterium-tumefaciens]	358
AMD60418.1	PAPS-reductase	GCA_001551895.1	379	Bacteria>Proteobacteria>Alphaproteobacteria>Hyphomicrobiales>Rhizobiaceae	Agrobacterium_tumefaciens	sulfurtransferase-[Agrobacterium-tumefaciens]	358
WP_156584786.1	PAPS-reductase	GCF_009743995.1	379	Bacteria>Proteobacteria>Alphaproteobacteria>Hyphomicrobiales>Rhizobiaceae	Agrobacterium_vitis	DNA-phosphorothioation-system-sulfurtransferase-DndC-[Agrobacterium-vitis]	373
WP_192439530.1	PAPS-reductase	GCF_014852035.1	379	Bacteria>Proteobacteria>Alphaproteobacteria>Hyphomicrobiales>Rhizobiaceae	Ensifer_sp._ENS03	DNA-phosphorothioation-system-sulfurtransferase-DndC-[Ensifer-sp.-ENS03]	2769283
WP_052637084.1	PAPS-reductase	GCF_000967425.1	282	Bacteria>Proteobacteria>Alphaproteobacteria>Hyphomicrobiales>Rhizobiaceae	Pseudorhizobium_banfieldiae	phosphoadenosine-phosphosulfate-reductase-family-protein-[Pseudorhizobium-banfieldiae]	1125847
MAM13639.1	PAPS-reductase	GCA_002695375.1	379	Bacteria>Proteobacteria>Alphaproteobacteria>Hyphomicrobiales>Rhizobiaceae	Rhizobiaceae_bacterium	DNA-phosphorothioation-system-sulfurtransferase-DndC-[Rhizobiaceae-bacterium]	1913961
NNU46284.1	PAPS-reductase	GCA_013087625.1	279	Bacteria>Proteobacteria>Alphaproteobacteria>Hyphomicrobiales>Rhizobiaceae	Rhizobium_changzhiense	phosphoadenosine-phosphosulfate-reductase-family-protein-[Rhizobium-changzhiense]	2692317
WP_171601353.1	PAPS-reductase	GCF_013087625.1	279	Bacteria>Proteobacteria>Alphaproteobacteria>Hyphomicrobiales>Rhizobiaceae	Rhizobium_changzhiense	phosphoadenosine-phosphosulfate-reductase-family-protein-[Rhizobium-changzhiense]	2692317
WP_004679897.1	PAPS-reductase	GCF_000194195.1	493	Bacteria>Proteobacteria>Alphaproteobacteria>Hyphomicrobiales>Rhizobiaceae	Rhizobium_etli	MULTISPECIES:-DNA-phosphorothioation-system-sulfurtransferase-DndC-[Rhizobium]	29449
WP_193446021.1	PAPS-reductase	GCF_013087605.1	279	Bacteria>Proteobacteria>Alphaproteobacteria>Hyphomicrobiales>Rhizobiaceae	Rhizobium_indigoferae	phosphoadenosine-phosphosulfate-reductase-family-protein-[Rhizobium-indigoferae]	158891
WP_168337897.1	PAPS-reductase	-	376	Bacteria>Proteobacteria>Alphaproteobacteria>Hyphomicrobiales>Rhizobiaceae	Rhizobium_laguerreae	DNA-phosphorothioation-system-sulfurtransferase-DndC-[Rhizobium-laguerreae]	1076926
WP_025398076.1	PAPS-reductase	GCF_000517605.1	493	Bacteria>Proteobacteria>Alphaproteobacteria>Hyphomicrobiales>Rhizobiaceae	Rhizobium_leguminosarum	DNA-phosphorothioation-system-sulfurtransferase-DndC-[Rhizobium-leguminosarum]	384
WP_130787524.1	PAPS-reductase	GCF_004306655.1	494	Bacteria>Proteobacteria>Alphaproteobacteria>Hyphomicrobiales>Rhizobiaceae	Rhizobium_leguminosarum	DNA-phosphorothioation-system-sulfurtransferase-DndC-[Rhizobium-leguminosarum]	384
WP_131702496.1	PAPS-reductase	GCF_004330525.1	493	Bacteria>Proteobacteria>Alphaproteobacteria>Hyphomicrobiales>Rhizobiaceae	Rhizobium_leguminosarum	DNA-phosphorothioation-system-sulfurtransferase-DndC-[Rhizobium-leguminosarum]	384
WP_168266327.1	PAPS-reductase	GCF_012276225.1	379	Bacteria>Proteobacteria>Alphaproteobacteria>Hyphomicrobiales>Rhizobiaceae	Rhizobium_leguminosarum	DNA-phosphorothioation-system-sulfurtransferase-DndC-[Rhizobium-leguminosarum]	384
WP_222071943.1	PAPS-reductase	GCF_019778265.1	493	Bacteria>Proteobacteria>Alphaproteobacteria>Hyphomicrobiales>Rhizobiaceae	Rhizobium_leguminosarum	DNA-phosphorothioation-system-sulfurtransferase-DndC-[Rhizobium-leguminosarum]	384
WP_222381171.1	PAPS-reductase	GCF_019799505.1	279	Bacteria>Proteobacteria>Alphaproteobacteria>Hyphomicrobiales>Rhizobiaceae	Rhizobium_leguminosarum	phosphoadenosine-phosphosulfate-reductase-family-protein-[Rhizobium-leguminosarum]	384
WP_081687994.1	PAPS-reductase	GCF_000427925.1	524	Bacteria>Proteobacteria>Alphaproteobacteria>Hyphomicrobiales>Rhizobiaceae	Rhizobium_mesoamericanum	DNA-phosphorothioation-system-sulfurtransferase-DndC-[Rhizobium-mesoamericanum]	1079800
WP_081279326.1	PAPS-reductase	GCF_001664225.1	390	Bacteria>Proteobacteria>Alphaproteobacteria>Hyphomicrobiales>Rhizobiaceae	Rhizobium_phaseoli	DNA-phosphorothioation-system-sulfurtransferase-DndC-[Rhizobium-phaseoli]	396
WP_081282820.1	PAPS-reductase	GCF_001664285.1	379	Bacteria>Proteobacteria>Alphaproteobacteria>Hyphomicrobiales>Rhizobiaceae	Rhizobium_phaseoli	DNA-phosphorothioation-system-sulfurtransferase-DndC-[Rhizobium-phaseoli]	396
WP_209914626.1	PAPS-reductase	GCF_017877395.1	493	Bacteria>Proteobacteria>Alphaproteobacteria>Hyphomicrobiales>Rhizobiaceae	Rhizobium_pusense	DNA-phosphorothioation-system-sulfurtransferase-DndC-[Rhizobium-pusense]	648995
WP_220367277.1	PAPS-reductase	GCF_019511305.1	376	Bacteria>Proteobacteria>Alphaproteobacteria>Hyphomicrobiales>Rhizobiaceae	Rhizobium_pusense	DNA-phosphorothioation-system-sulfurtransferase-DndC-[Rhizobium-pusense]	648995
MBP2614109.1	PAPS-reductase	GCA_017877395.1	493	Bacteria>Proteobacteria>Alphaproteobacteria>Hyphomicrobiales>Rhizobiaceae	Rhizobium_pusense	DNA-sulfur-modification-protein-DndC-[Rhizobium-pusense]	648995
SOD50252.1	PAPS-reductase	GCA_900215635.1	526	Bacteria>Proteobacteria>Alphaproteobacteria>Hyphomicrobiales>Rhizobiaceae	Rhizobium_sp._AN6A	DNA-sulfur-modification-protein-DndC-[Rhizobium-sp.-AN6A]	1841611
TKV70610.1	PAPS-reductase	GCA_005298145.1	491	Bacteria>Proteobacteria>Alphaproteobacteria>Hyphomicrobiales>Rhizobiaceae	Rhizobium_sp._AU243	DNA-phosphorothioation-system-sulfurtransferase-DndC-[Rhizobium-sp.-AU243]	2303425
KQM32601.1	PAPS-reductase	GCA_001421235.1	359	Bacteria>Proteobacteria>Alphaproteobacteria>Hyphomicrobiales>Rhizobiaceae	Rhizobium_sp._Leaf202	sulfurtransferase-[Rhizobium-sp.-Leaf202]	1735675
WP_085780251.1	PAPS-reductase	GCF_002117485.1	493	Bacteria>Proteobacteria>Alphaproteobacteria>Hyphomicrobiales>Rhizobiaceae	Rhizobium_sp._NXC14	DNA-phosphorothioation-system-sulfurtransferase-DndC-[Rhizobium-sp.-NXC14]	1981173
WP_132714632.1	PAPS-reductase	GCF_004341455.1	376	Bacteria>Proteobacteria>Alphaproteobacteria>Hyphomicrobiales>Rhizobiaceae	Rhizobium_sp._PP-CC-2G-626	DNA-phosphorothioation-system-sulfurtransferase-DndC-[Rhizobium-sp.-PP-CC-2G-626]	2135646
TCP80777.1	PAPS-reductase	GCA_004341455.1	376	Bacteria>Proteobacteria>Alphaproteobacteria>Hyphomicrobiales>Rhizobiaceae	Rhizobium_sp._PP-CC-2G-626	DNA-sulfur-modification-protein-DndC-[Rhizobium-sp.-PP-CC-2G-626]	2135646
KQY41331.1	PAPS-reductase	GCA_001427205.1	379	Bacteria>Proteobacteria>Alphaproteobacteria>Hyphomicrobiales>Rhizobiaceae	Rhizobium_sp._Root491	sulfurtransferase-[Rhizobium-sp.-Root491]	1736548
PDT43970.1	PAPS-reductase	GCA_002531965.1	376	Bacteria>Proteobacteria>Alphaproteobacteria>Hyphomicrobiales>Rhizobiaceae	Sinorhizobium_fredii	DNA-phosphorothioation-system-sulfurtransferase-DndC-[Sinorhizobium-fredii]	380
WP_097588014.1	PAPS-reductase	GCF_002531965.1	376	Bacteria>Proteobacteria>Alphaproteobacteria>Hyphomicrobiales>Rhizobiaceae	Sinorhizobium_fredii	DNA-phosphorothioation-system-sulfurtransferase-DndC-[Sinorhizobium-fredii]	380
WP_127630864.1	PAPS-reductase	GCF_004002725.1	279	Bacteria>Proteobacteria>Alphaproteobacteria>Hyphomicrobiales>Rhizobiaceae	Sinorhizobium_medicae	phosphoadenosine-phosphosulfate-reductase-family-protein-[Sinorhizobium-medicae]	110321
WP_127672678.1	PAPS-reductase	GCF_004004675.1	376	Bacteria>Proteobacteria>Alphaproteobacteria>Hyphomicrobiales>Rhizobiaceae	Sinorhizobium_meliloti	DNA-phosphorothioation-system-sulfurtransferase-DndC-[Sinorhizobium-meliloti]	382
WP_201446060.1	PAPS-reductase	GCF_000375585.1	520	Bacteria>Proteobacteria>Alphaproteobacteria>Hyphomicrobiales>Rhizobiaceae	Sinorhizobium_meliloti	DNA-phosphorothioation-system-sulfurtransferase-DndC-[Sinorhizobium-meliloti]	382
WP_065785423.1	PAPS-reductase	GCF_001695905.1	376	Bacteria>Proteobacteria>Alphaproteobacteria>Hyphomicrobiales>Rhizobiaceae	unclassified_Ensifer	MULTISPECIES:-DNA-phosphorothioation-system-sulfurtransferase-DndC-[unclassified-Ensifer]	2633371
WP_200342395.1	PAPS-reductase	GCF_016583645.1	520	Bacteria>Proteobacteria>Alphaproteobacteria>Hyphomicrobiales>Rhodovibrionaceae	Rhodovibrio_sodomensis	DNA-phosphorothioation-system-sulfurtransferase-DndC-[Rhodovibrio-sodomensis]	1088
MBG6162806.1	PAPS-reductase	GCA_015752055.1	278	Bacteria>Proteobacteria>Alphaproteobacteria>Hyphomicrobiales>Stappiaceae	Labrenzia_sp._EL_195	3’-phosphoadenosine-5’-phosphosulfate-sulfotransferase-(PAPS-reductase)/FAD-synthetase-[Labrenzia-sp.-EL_195]	2787743
[truncated: 11,576,027 more chars]
