# Supplementary material for: Reappraisal of the DNA phosphorothioate modification machinery: uncovering neglected functional modalities and identification of new counter-invader defense systems
Source: Nucleic Acids Res. 2024 Jan 2;52(3):1005–26. doi: 10.1093/nar/gkad1213 (PMC10853773; doi:10.1093/nar/gkad1213)
Supplement: gkad1213_Supplemental_Files [file Supplementary_Dataset_3.pdf.pdf › Supplementary_Dataset_3.pdf.pdf]

**Supplementary_Dataset_3_Alignment_Files_Index**


1. DndE reference alignment
   - i. DndE Clade 1 reference alignment……………………..1-8
   - ii. DndE Clade 2 reference alignment……………………..9-14
2. DndD and CxC ABC ATPase reference alignment
   - i. DndD ABC reference alignment………………………..15-20
   - ii. CxC ABC reference alignment………………………….21-26
3. DndF reference alignment
   - i. Canonical DndF reference alignment……….………….27
   - ii. HEPN+DndF reference alignment………………………..28
   - iii. NERD+iPK+PK+DndF reference alignment…………….29
   - iv. wHTH+NERD+iPK+PK+DndF reference alignment……30
   - v. wHTH+DndF reference alignment……………………….31
   - vi. GNAT+DndF reference alignment……………………….32
   - vii. ZnR+DndF reference alignment…………………………33
4. DndG reference alignment………………………….34-36
5. DndH reference alignment………………………….37-39

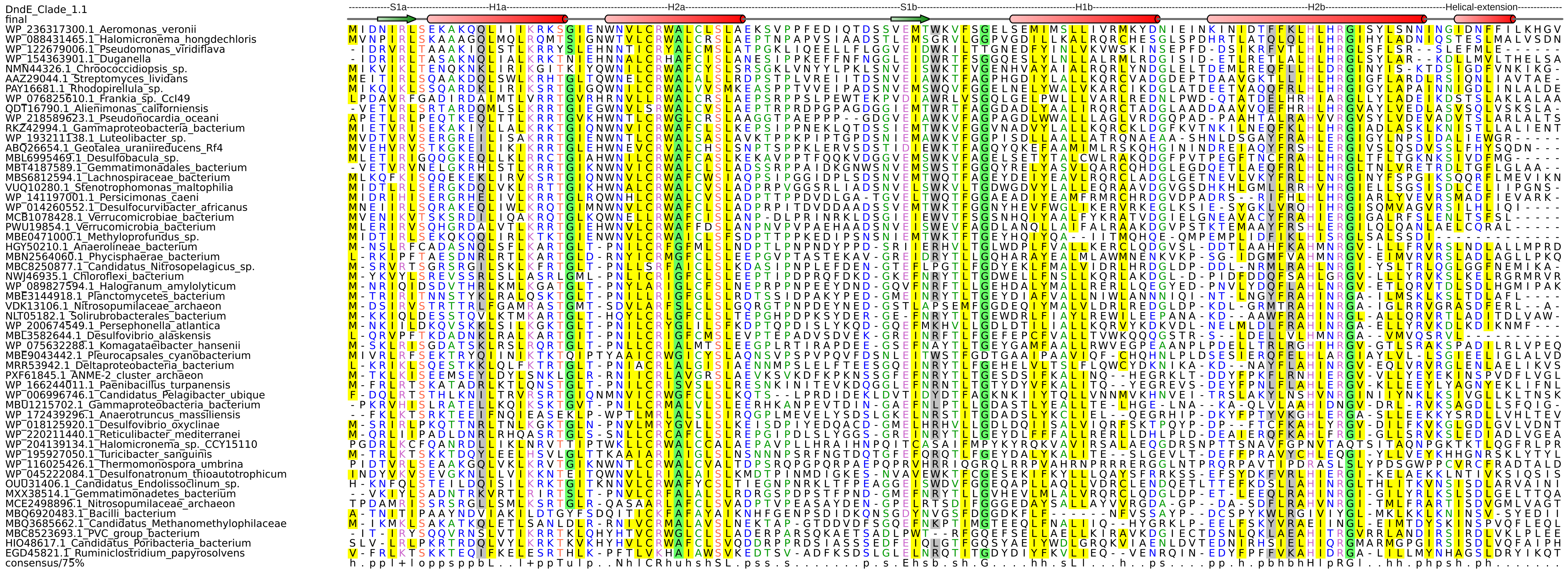
DndE_Clade_1.1
final
S1a
H1a
H2a
S1b
H1b
H2b
Helical-extension
WP_236317300.1_Aeromonas_veronii
WP_088431465.1_Halomicronema_hongdechloris
WP_122679006.1_Pseudomonas_viridiflava
WP_154363901.1_Duganella
NMN44326.1_Chroococcidiopsis_sp.
AAZ29044.1_Streptomyces_lividans
PAY16681.1_Rhodopirellula_sp.
WP_076825610.1_Frankia_sp._CcI49
QDT16790.1_Alienimonas_californiensis
WP_218589623.1_Pseudonocardia_oceani
RKZ42994.1_Gammaproteobacteria_bacterium
WP_193211138.1_Luteolibacter_sp.
ABQ26654.1_Geotalea_uraniireducens_Rf4
MBL6995469.1_Desulfobacula_sp.
MBT4187589.1_Gemmatimonadales_bacterium
MBS6812594.1_Lachnospiraceae_bacterium
VUQ10280.1_Stenotrophomonas_maltophilia
WP_141197001.1_Persicimonas_caeni
WP_014260552.1_Desulfocurvibacter_africanus
MCB1078428.1_Verrucomicrobiae_bacterium
PWU19854.1_Verrucomicrobia_bacterium
MBE0471000.1_Methyloprofundus_sp.
HGY50210.1_Anaerolineae_bacterium
MBN2564060.1_Phycisphaerae_bacterium
MBC8250877.1_Candidatus_Nitrosopelagicus_sp.
NWJ46935.1_Chloroflexi_bacterium
WP_089827594.1_Halogranum_amylolyticum
MBE3144918.1_Planctomycetes_bacterium
VDK13106.1_Nitrosopumilaceae_archaeon
NLT05182.1_Solirubrobacterales_bacterium
WP_200674549.1_Persephonella_atlantica
MBL3582644.1_Desulfovibrio_alaskensis
WP_075632288.1_Komagataeibacter_hansenii
MBE9043442.1_Pleurocapsales_cyanobacterium
MRR53942.1_Deltaproteobacteria_bacterium
PXF61845.1_ANME-2_cluster_archaeon
WP_166244011.1_Paenibacillus_turpanensis
WP_006996746.1_Candidatus_Pelagibacter_ubique
MBU1215702.1_Gammaproteobacteria_bacterium
WP_172439296.1_Anaerotruncus_massiliensis
WP_018125920.1_Desulfovibrio_oxyclinae
WP_220211440.1_Reticulibacter_mediterranei
WP_204139134.1_Halomicronema_sp._CCY15110
WP_195927050.1_Turicibacter_sanguinis
WP_116025426.1_Thermomonospora_umbrina
WP_045222084.1_Desulfonatronum_thioautotrophicum
OUU31406.1_Candidatus_Endolissoclinum_sp.
MXX38514.1_Gemmatimonadetes_bacterium
MCE2498896.1_Nitrosopumilaceae_archaeon
MBQ6920483.1_Bacilli_bacterium
MBQ3685662.1_Candidatus_Methanomethylophilaceae
MBC8523693.1_PVC_group_bacterium
HIO48617.1_Candidatus_Poribacteria_bacterium
EGD45821.1_Ruminiclostridium_papyrosolvens
consensus/75%
h.ppl+loppspppbL..l+ppTulp..NhlCRhuhshSL.pss.s.......p.s.Ehsb.sh.G....hh.sLl...h..ps....pp.h.pbhbhHlpRGl...hh.phpsh.ph......

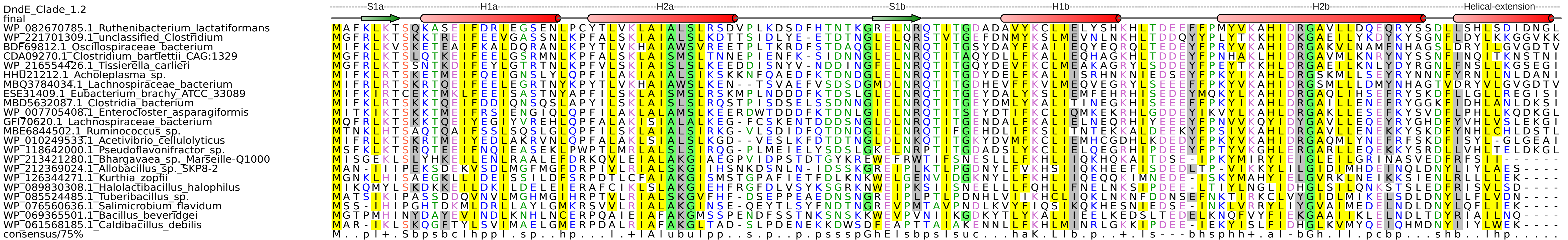
DndE_Clade_1.2
final
S1a
H1a
H2a
S1b
H1b
H2b
Helical-extension
WP_082670785.1_Ruthenibacterium_lactatiformans
WP_221701309.1_unclassified_Clostridium
BDF69812.1_Oscillospiraceae_bacterium
CDA09270.1_Clostridium_bartlettii_CAG:1329
WP_216554426.1_Tissierella_carlieri
HHU21212.1_Acholeplasma_sp.
MBQ3784034.1_Lachnospiraceae_bacterium
ESE31409.1_Eubacterium_brachy_ATCC_33089
MBD5632087.1_Clostridia_bacterium
WP_007705408.1_Enterocloster_asparagiformis
GFI70620.1_Lachnospiraceae_bacterium
MBE6844502.1_Ruminococcus_sp.
WP_010249533.1_Acetivibrio_cellulolyticus
WP_118642000.1_Pseudoflavonifractor_sp.
WP_213421280.1_Bhargavaea_sp._Marseille-Q1000
WP_212369024.1_Allobacillus_sp._SKP8-2
WP_126344271.1_Kurthia_zopfii
WP_089830308.1_Halolactibacillus_halophilus
WP_085524485.1_Tuberibacillus_sp.
WP_076560636.1_Salimicrobium_flavidum
WP_069365501.1_Bacillus_beveridgei
WP_061568185.1_Caldibacillus_debilis
consensus/75%

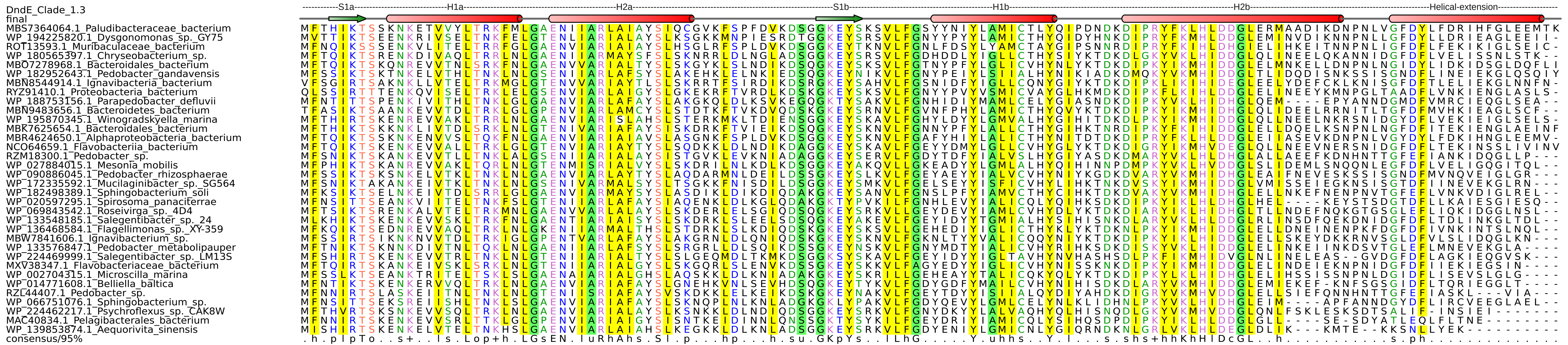
DndE_Clade_1.3
final
S1a
H1a
H2a
S1b
H1b
H2b
Helical-extension
MBS7364064.1_Paludibacteraceae_bacterium
WP_194225820.1_Dysgonomonas_sp._GY75
ROT13593.1_Muribaculaceae_bacterium
WP_180565397.1_Chryseobacterium_sp.
MBO7278968.1_Bacteroidales_bacterium
WP_182952643.1_Pedobacter_gandavensis
MBN8544914.1_Ignavibacteria_bacterium
RYZ91410.1_Proteobacteria_bacterium
WP_188753156.1_Parapedobacter_defluvii
MBN9483656.1_Bacteroidetes_bacterium
WP_195870345.1_Winogradskyella_marina
MBK7625654.1_Bacteroidales_bacterium
MBR4624650.1_Alphaproteobacteria_bacterium
NCO64659.1_Flavobacteriia_bacterium
RZM18300.1_Pedobacter_sp.
WP_027884015.1_Mesonia_mobilis
WP_090886045.1_Pedobacter_rhizosphaerae
WP_172335592.1_Mucilaginibacter_sp._SG564
WP_182498389.1_Sphingobacterium_soli
WP_020597295.1_Spirosoma_panaciterrae
WP_069843542.1_Roseivirga_sp._4D4
WP_133548185.1_Salegentibacter_sp._24
WP_136468584.1_Flagellimonas_sp._XY-359
MBW7841606.1_Ignavibacterium_sp.
WP_133576847.1_Pedobacter_metabolipauper
WP_224469999.1_Salegentibacter_sp._LM13S
MXV38347.1_Flavobacteriaceae_bacterium
WP_002704315.1_Microscilla_marina
WP_014771608.1_Belliella_baltica
RZL44407.1_Pedobacter_sp.
WP_066751076.1_Sphingobacterium_sp.
WP_224462217.1_Psychroflexus_sp._CAK8W
MAC40834.1_Pelagibacterales_bacterium
WP_139853874.1_Aequorivita_sinensis
consensus/95%

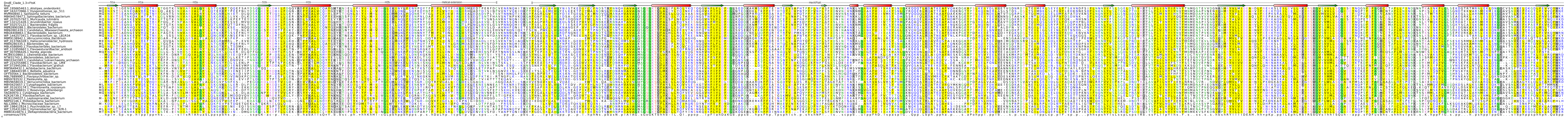

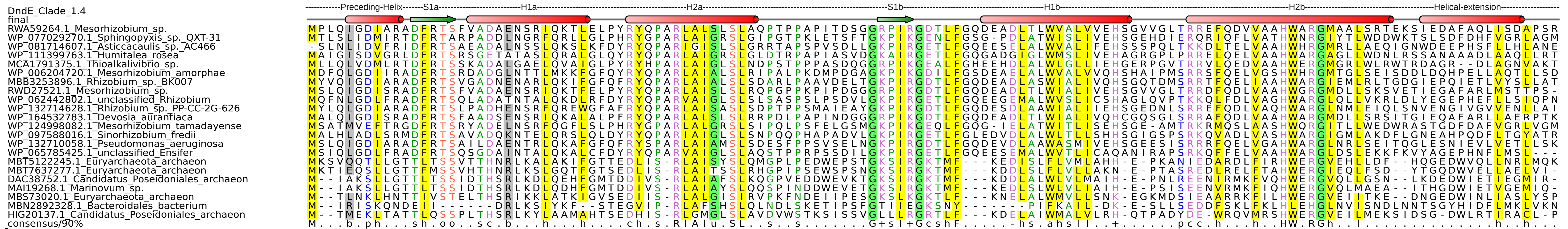
DndE_Clade_1.4
final
Preceding-Helix
S1a
H1a
H2a
S1b
H1b
H2b
Helical-extension
RWA59264.1_Mesorhizobium_sp.
WP_077029270.1_Sphingopyxis_sp._QXT-31
WP_081714607.1_Asticcacaulis_sp._AC466
WP_111399763.1_Humitalea_rosea
MCA1791375.1_Thioalkalivibrio_sp.
WP_006204720.1_Mesorhizobium_amorphae
MBB3253896.1_Rhizobium_sp._BK007
RWD27521.1_Mesorhizobium_sp.
WP_062442802.1_unclassified_Rhizobium
WP_132714628.1_Rhizobium_sp._PP-CC-2G-626
WP_164532783.1_Devosia_aurantiaca
WP_124998082.1_Mesorhizobium_tamadayense
WP_097588016.1_Sinorhizobium_fredii
WP_132710058.1_Pseudomonas_aeruginosa
WP_065785425.1_unclassified_Ensifer
MBT5122245.1_Euryarchaeota_archaeon
MBT7637277.1_Euryarchaeota_archaeon
DAC38752.1_Candidatus_Poseidoniales_archaeon
MAI19268.1_Marinovum_sp.
MBS73020.1_Euryarchaeota_archaeon
MBN2892328.1_Bacteroidales_bacterium
HIG20137.1_Candidatus_Poseidoniales_archaeon
consensus/90%

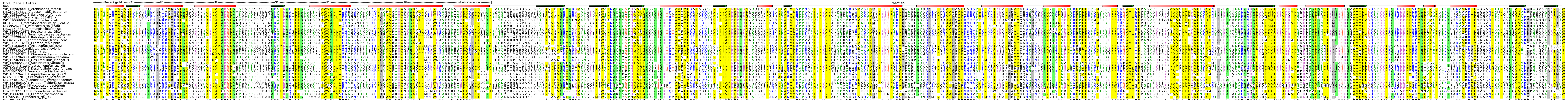
DndE_Clade_1.4+FtsK
final
Preceding-Helix
WP_156969001.1_Arenimonas_metalli
MBC6405082.1_Rhodospirillales_bacterium
WP_093412627.1_Salipiger_profundus
SDG06591.1_Dyella_sp._333MFSha
WP_010666007.1_Acetobacter_aceti
KQQ17380.1_Methylobacterium_sp._Leaf121
MBD9526219.1_Paracoccus_sp._PAR01
MBC7160884.1_Immundisolibacter_sp.
WP_226614268.1_Roseicella_sp._GB24
MCB1885299.1_Geminicoccaceae_bacterium
WP_037299490.1_Rubritepida_flocculans
MBB4126715.1_Xanthomonas_translucens
WP_211211320.1_Elioraea_tepidiphila
WP_041836056.1_Acidovorax_sp._JS42
HJA75297.1_Candidatus_Desulfovibrio
MBS3904664.1_Simkania_sp.
WP_081541824.1_Chromobacterium_violaceum
WP_213378400.1_Allochromatium_tepidum
WP_157469888.1_Desulfobulbus_elongatus
WP_148665470.1_Sulfurifustis_variabilis
VFK24947.1_Candidatus_Kentron_sp._MB
WP_209818705.1_Desulfovibrio_desulfuricans
MBM3861552.1_Verrucomicrobia_bacterium
WP_165226411.1_Aquisphaera_sp._JC669
MBP7830370.1_Kiritimatiellae_bacterium
MBL7648115.1_Candidatus_Hydrogenedentes
WP_132450307.1_Paraburkholderia_sp._BL8N3
MBL8680161.1_Myxococcales_bacterium
MBP8808960.1_Kofleriaceae_bacterium
HDY33132.1_Armatimonadetes_bacterium
WP_198669010.1_Elioraea_thermophila
OTE96534.1_Crenothrix_sp._D3
consensus/75%

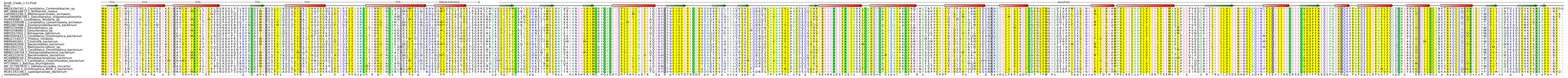

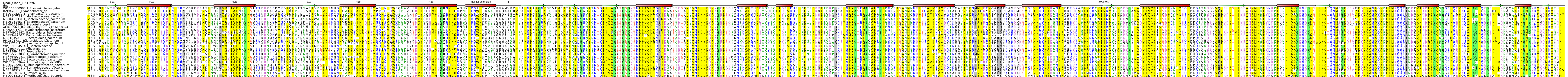
DndE_Clade_1.6+FtsK
final
WP_118300988.1_Phocaeicola_vulgatus
RZJ94781.1_Hymenobacter_sp.
MBD1208272.1_Ignavibacteria_bacterium
MBR5117122.1_Muribaculaceae_bacterium
MBO4451331.1_Bacteroidaceae_bacterium
MBQ6751482.1_Bacteroidaceae_bacterium
MBM0128968.1_Prevotella_copri
AEI46556.1_Runella_slithyformis_DSM_19594
MAM29317.1_Flavobacteriaceae_bacterium
MBP7497614.1_Bacteroidales_bacterium
MBP5344730.1_Bacteroidales_bacterium
MBR1835098.1_Bacteroidales_bacterium
RMG69578.1_Bacteroidetes_bacterium
TXF75218.1_Chryseobacterium_sp._legu1
WP_171034514.1_Bacteroidaceae
MBP8934701.1_Prevotella_sp.
MBR1389429.1_Prevotella_sp.
WP_122203035.1_Parabacteroides_merdae
MBK7640796.1_Bacteroidetes_bacterium
MBR5199622.1_Bacteroidales_bacterium
WP_114069642.1_Runella_sp._HYN0085
MBQ8722288.1_Paludibacteraceae_bacterium
MCC5946645.1_Bernardetiaceae_bacterium
MBR6103723.1_Paludibacteraceae_bacterium
MBO4850132.1_Prevotella_sp.
MBQ9218150.1_Muribaculaceae_bacterium
consensus/80%

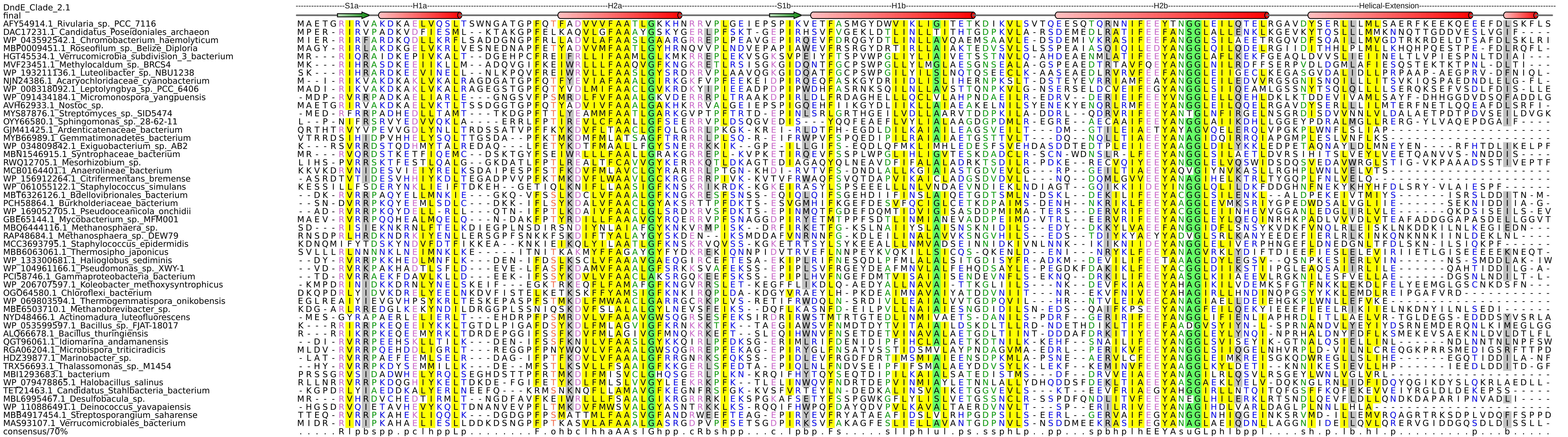
DndE_Clade_2.1
final
S1a
H1a
H2a
S1b
H1b
H2b
Helical-Extension
AFY54914.1_Rivularia_sp._PCC_7116
DAC17231.1_Candidatus_Poseidoniales_archaeon
WP_043592542.1_Chromobacterium_haemolyticum
MBP0009451.1_Roseofilum_sp._Belize_Diploria
HGT45534.1_Verrucomicrobia_subdivision_3_bacterium
MVF23451.1_Methylocaldum_sp._BRCS4
WP_193211136.1_Luteolibacter_sp._NBU1238
NJN24386.1_Acaryochloridaceae_cyanobacterium
WP_008318092.1_Leptolyngbya_sp._PCC_6406
WP_091434184.1_Micromonospora_yangpuensis
AVH62933.1_Nostoc_sp.
MYS87876.1_Streptomyces_sp._SID5474
OYY66580.1_Sphingomonas_sp._28-62-11
GJM41425.1_Ardenticatenaceae_bacterium
MYB66989.1_Gemmatimonadetes_bacterium
WP_034809842.1_Exiguobacterium_sp._AB2
MBN1546915.1_Syntrophaceae_bacterium
RWQ12705.1_Mesorhizobium_sp.
MCB0164401.1_Anaerolineae_bacterium
WP_156912264.1_Citrifermentans_bremense
WP_061055122.1_Staphylococcus_simulans
MBT6326126.1_Bdellovibrionales_bacterium
PCH58864.1_Burkholderiaceae_bacterium
WP_169052705.1_Pseudooceanicola_onchidii
GBE65144.1_Mycobacterium_sp._MFM001
MBQ6444116.1_Methanosphaera_sp.
RAP48684.1_Methanosphaera_sp._DEW79
MCC3693795.1_Staphylococcus_epidermidis
MBB6063061.1_Thermosipho_japonicus
WP_133300681.1_Halioglobus_sediminis
WP_104961166.1_Pseudomonas_sp._XWY-1
PCI58746.1_Gammaproteobacteria_bacterium
WP_206707597.1_Koleobacter_methoxysyntrophicus
OGO64580.1_Chloroflexi_bacterium
WP_069803594.1_Thermogemmatispora_onikobensis
MBE6503710.1_Methanobrevibacter_sp.
NYD48466.1_Actinomadura_luteofluorescens
WP_053599597.1_Bacillus_sp._FJAT-18017
ALQ66678.1_Bacillus_thuringiensis
QGT96061.1_Idiomarina_andamanensis
RGA06204.1_Microbispora_triticiradicis
HDZ39877.1_Marinobacter_sp.
TRX56693.1_Thalassomonas_sp._M1454
MBI1293683.1_bacterium
WP_079478865.1_Halobacillus_salinus
TET21463.1_Candidatus_Stahlbacteria_bacterium
MBL6995467.1_Desulfobacula_sp.
WP_110886491.1_Deinococcus_yavapaiensis
MBB4917454.1_Streptosporangium_saharense
MAS93107.1_Verrucomicrobiales_bacterium
consensus/70%

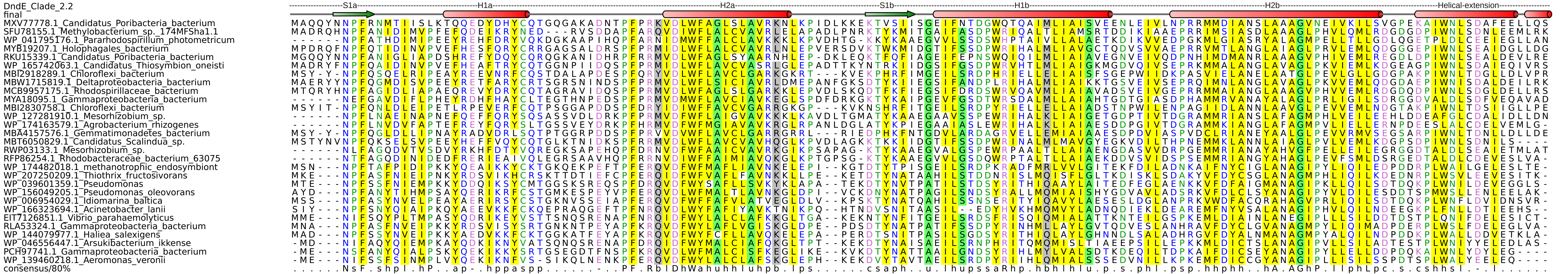
DndE_Clade_2.2
final
S1a
H1a
H2a
S1b
H1b
H2b
Helical-extension
MXV77778.1_Candidatus_Poribacteria_bacterium
SFU78155.1_Methylobacterium_sp._174MFSha1.1
WP_041795176.1_Pararhodospirillum_photometricum
MYB19207.1_Holophagales_bacterium
RKU15339.1_Candidatus_Poribacteria_bacterium
WP_165742063.1_Candidatus_Thiosymbion_oneisti
MBI2918289.1_Chloroflexi_bacterium
MBW1715819.1_Deltaproteobacteria_bacterium
MCB9957175.1_Rhodospirillaceae_bacterium
MYA18095.1_Gammaproteobacteria_bacterium
MBI2830758.1_Chloroflexi_bacterium
WP_127281910.1_Mesorhizobium_sp.
WP_174163579.1_Agrobacterium_rhizogenes
MBA4157576.1_Gemmatimonadetes_bacterium
MBT6050829.1_Candidatus_Scalindua_sp.
RWP03133.1_Mesorhizobium_sp.
RFP86254.1_Rhodobacteraceae_bacterium_63075
WP_174482018.1_methanotrophic_endosymbiont
WP_207250209.1_Thiothrix_fructosivorans
WP_039601359.1_Pseudomonas
WP_156049205.1_Pseudomonas_oleovorans
WP_006954029.1_Idiomarina_baltica
WP_166323694.1_Acinetobacter_lanii
EIT7126851.1_Vibrio_parahaemolyticus
RLA53324.1_Gammaproteobacteria_bacterium
WP_144079977.1_Haliea_salexigens
WP_046556447.1_Arsukibacterium_ikkense
PCH97741.1_Gammaproteobacteria_bacterium
WP_139460218.1_Aeromonas_veronii
consensus/80%

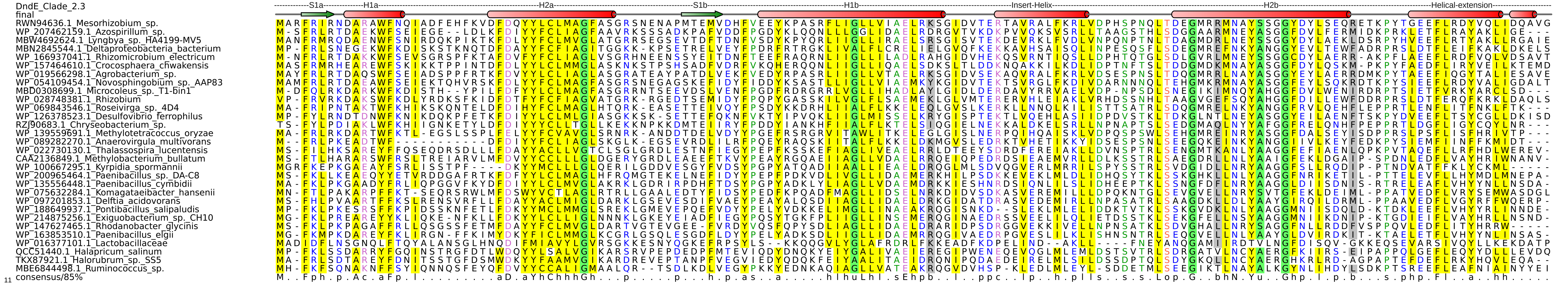

DndE_Clade_2.3
final
S1a
H1a
H2a
S1b
H1b
Insert-Helix
H2b
Helical-extension
RWN94636.1_Mesorhizobium_sp.
WP_207462159.1_Azospirillum_sp.
MBW4692624.1_Lyngbya_sp._HA4199-MV5
MBN2845544.1_Deltaproteobacteria_bacterium
WP_166937041.1_Rhizomicrobium_electricum
WP_157464610.1_Crocosphaera_chwakensis
WP_019566298.1_Agrobacterium_sp.
WP_054109454.1_Novosphingobium_sp._AAP83
MBD0308699.1_Microcoleus_sp._T1-bin1
WP_028748381.1_Rhizobium
WP_069843546.1_Roseivirga_sp._4D4
WP_126378523.1_Desulfovibrio_ferrophilus
RZJ90683.1_Chryseobacterium_sp.
WP_139559691.1_Methylotetracoccus_oryzae
WP_089282270.1_Anaerovirgula_multivorans
WP_022730130.1_Thalassospira_lucentensis
CAA2136849.1_Methylobacterium_bullatum
WP_100667295.1_Kyrpidia_spormannii
WP_200965464.1_Paenibacillus_sp._DA-C8
WP_135556448.1_Paenibacillus_cymbidii
WP_075632284.1_Komagataeibacter_hansenii
WP_097201853.1_Delftia_acidovorans
WP_188649937.1_Pontibacillus_salipaludis
WP_214875256.1_Exiguobacterium_sp._CH10
WP_147627465.1_Rhodanobacter_glycinis
WP_163853510.1_Paenibacillus_elgii
WP_016377101.1_Lactobacillaceae
QCC51440.1_Halapricum_salinum
TKX87921.1_Halorubrum_sp._SS5
MBE6844498.1_Ruminococcus_sp.
consensus/85%

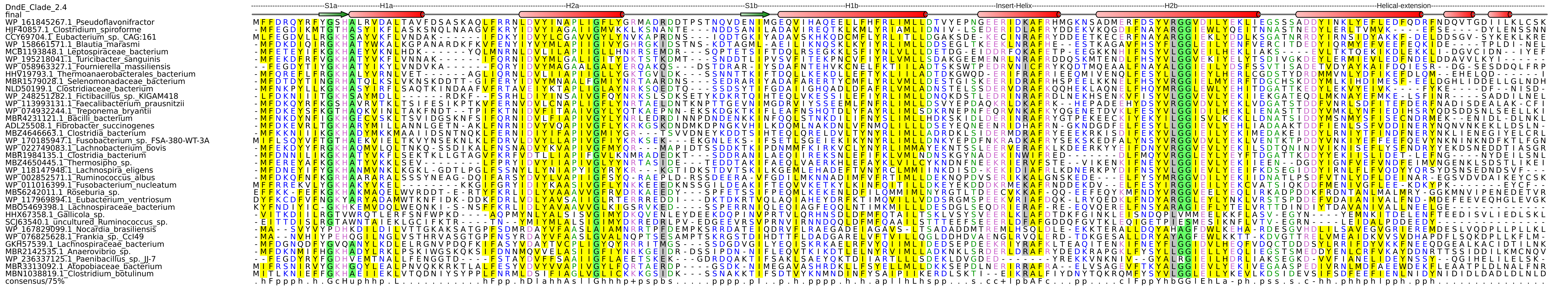
DndE_Clade_2.4
final
S1a
H1a
H2a
S1b
H1b
Insert-Helix
H2b
Helical-extension
WP_161845267.1_Pseudoflavonifractor
HJF40857.1_Clostridium_spiroforme
CCY69704.1_Eubacterium_sp._CAG:161
WP_158661571.1_Blautia_marasmi
MCB1193848.1_Leptospiraceae_bacterium
WP_195218041.1_Turicibacter_sanguinis
WP_058963327.1_Fournierella_massiliensis
HHV19793.1_Thermoanaerobacterales_bacterium
MBR1579028.1_Selenomonadaceae_bacterium
NLD50199.1_Clostridiaceae_bacterium
WP_248251282.1_Fictibacillus_sp._KIGAM418
WP_113993131.1_Faecalibacterium_prausnitzii
WP_074932244.1_Treponema_bryantii
MBR4231121.1_Bacilli_bacterium
ADL25508.1_Fibrobacter_succinogenes
MBZ4646663.1_Clostridia_bacterium
WP_170185947.1_Fusobacterium_sp._FSA-380-WT-3A
WP_022749083.1_Lachnobacterium_bovis
MBR1984135.1_Clostridia_bacterium
MBZ4650445.1_Thermosipho_sp.
WP_118147948.1_Lachnospira_eligens
WP_002852571.1_Ruminococcus_albus
WP_011016399.1_Fusobacterium_nucleatum
MBS6242011.1_Roseburia_sp.
WP_117969894.1_Eubacterium_ventriosum
MBO5469398.1_Lachnospiraceae_bacterium
HHX67358.1_Gallicola_sp.
SCJ63540.1_uncultured_Ruminococcus_sp.
WP_167829099.1_Nocardia_brasiliensis
WP_076825628.1_Frankia_sp._Ccl49
GKH57539.1_Lachnospiraceae_bacterium
MBR2142535.1_Anaerovibrio_sp.
WP_236337125.1_Paenibacillus_sp._JJ-7
MBR3313092.1_Atopobiaceae_bacterium
MBN1038819.1_Clostridium_botulinum
consensus/75%

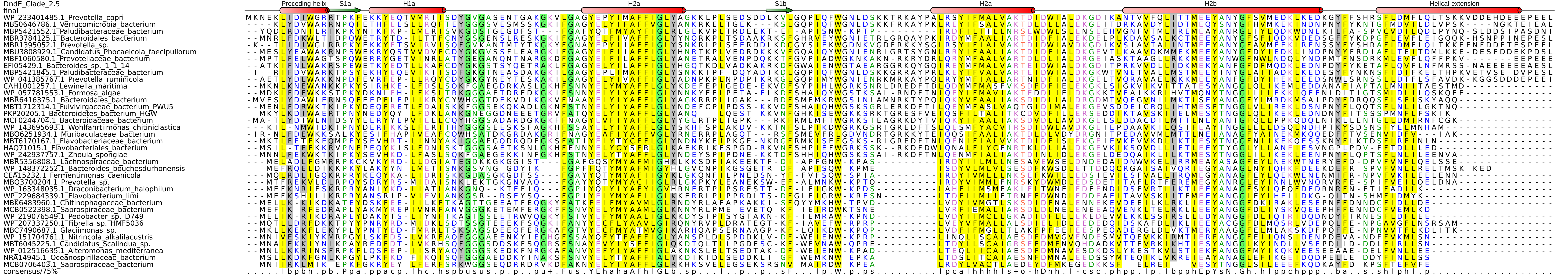
DndE_Clade_2.5
final
Preceding-helix
S1a
H1a
H2a
S1b
H2a
H2b
Helical-extension
WP_233401485.1_Prevotella_copri
MBS0646786.1_Verrucomicrobia_bacterium
MBP5421552.1_Paludibacteraceae_bacterium
MBR3784125.1_Bacteroidales_bacterium
MBR1395052.1_Prevotella_sp.
MBU3808929.1_Candidatus_Phocaeicola_faecipullorum
MBF1060580.1_Prevotellaceae_bacterium
EFI05429.1_Bacteroides_sp._1_1_14
MBP5421845.1_Paludibacteraceae_bacterium
WP_041385767.1_Prevotella_ruminicola
CAH1001257.1_Lewinella_maritima
WP_057781553.1_Formosa_algae
MBR6416375.1_Bacteroidales_bacterium
MBT1712314.1_Fulvivirgaceae_bacterium_PWU5
PKP20205.1_Bacteroidetes_bacterium_HGW
MCF0244704.1_Bacteroidaceae_bacterium
WP_143695693.1_Wohlfahrtiimonas_chitiniclastica
MBO6251934.1_Muribaculaceae_bacterium
MBT6170167.1_Flavobacteriaceae_bacterium
HAQ71015.1_Flavobacteriales_bacterium
WP_242937757.1_Zhouia_spongiae
MBR5356808.1_Lachnospiraceae_bacterium
WP_148372252.1_Bacteroides_bouchesdurhonensis
CEA15232.1_Fermentimonas_caenicola
MBQ3700204.1_Prevotella_sp.
WP_163348035.1_Draconibacterium_halophilum
WP_229684339.1_Flavobacterium_limi
MBK6483960.1_Chitinophagaceae_bacterium
MCB0522398.1_Saprospiraceae_bacterium
WP_219076549.1_Pedobacter_sp._D749
WP_207337250.1_Fibrella_sp._HMF5036
MBC7490687.1_Glaciimonas_sp.
WP_151704761.1_Nitrincola_alkalilacustris
MBT6045225.1_Candidatus_Scalindua_sp.
WP_012516635.1_Alteromonas_mediterranea
NRA14945.1_Oceanospirillaceae_bacterium
MCB0706403.1_Saprospiraceae_bacterium
consensus/75%

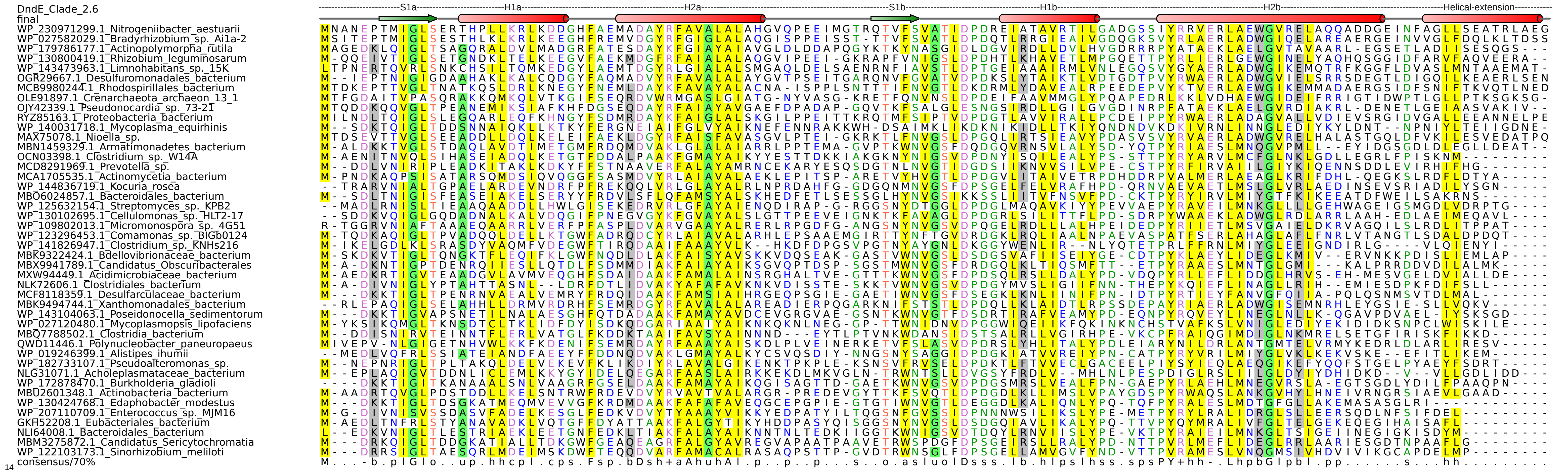
DndE_Clade_2.6
final
S1a
H1a
H2a
S1b
H1b
H2b
Helical-extension
WP_230971299.1_Nitrogeniibacter_aestuarii
WP_027582029.1_Bradyrhizobium_sp._Ai1a-2
WP_179786177.1_Actinopolymorpha_rutila
WP_130800419.1_Rhizobium_leguminosarum
WP_143473963.1_Limnohabitans_sp._15K
OGR29667.1_Desulfuromonadales_bacterium
MCB9980244.1_Rhodospirillales_bacterium
OLE91897.1_Crenarchaeota_archaeon_13_1
OJY42339.1_Pseudonocardia_sp._73-21
RYZ85163.1_Proteobacteria_bacterium
WP_140031718.1_Mycoplasma_equirhinis
MAX75078.1_Nioella_sp.
MBN1459329.1_Armatimonadetes_bacterium
OCN03398.1_Clostridium_sp._W14A
MCD8291969.1_Prevotella_sp.
MCA1705535.1_Actinomycetia_bacterium
WP_144836719.1_Kocuria_rosea
MBO6024857.1_Bacteroidales_bacterium
WP_125632154.1_Streptomyces_sp._KPB2
WP_130102695.1_Cellulomonas_sp._HLT2-17
WP_109802013.1_Micromonospora_sp._4G51
WP_123296453.1_Comamonas_sp._BIGb0124
WP_141826947.1_Clostridium_sp._KNHs216
MBK9322424.1_Bdellovibrionaceae_bacterium
MBX9941789.1_Candidatus_Obscuribacterales
MXW94449.1_Acidimicrobiaceae_bacterium
NLK72606.1_Clostridiales_bacterium
MCF8118359.1_Desulfarculaceae_bacterium
MBK9494744.1_Xanthomonadales_bacterium
WP_143104063.1_Poseidonocella_sedimentorum
WP_027120480.1_Mycoplasmopsis_lipofaciens
MBQ7788502.1_Clostridia_bacterium
QWD11446.1_Polynucleobacter_paneuropaeus
WP_019246399.1_Alistipes_ihumii
WP_182733107.1_Pseudoalteromonas_sp.
NLG31071.1_Acholeplasmataceae_bacterium
WP_172878470.1_Burkholderia_gladioli
MBU2601348.1_Actinobacteria_bacterium
WP_130424768.1_Edaphobacter_modestus
WP_207110709.1_Enterococcus_sp._MJM16
GKH52208.1_Eubacteriales_bacterium
NLI64008.1_Bacteroidales_bacterium
MBM3275872.1_Candidatus_Sericytochromatia
WP_122103173.1_Sinorhizobium_meliloti
consensus/70%

DndD-ABC associated with clade 1.1 DndE

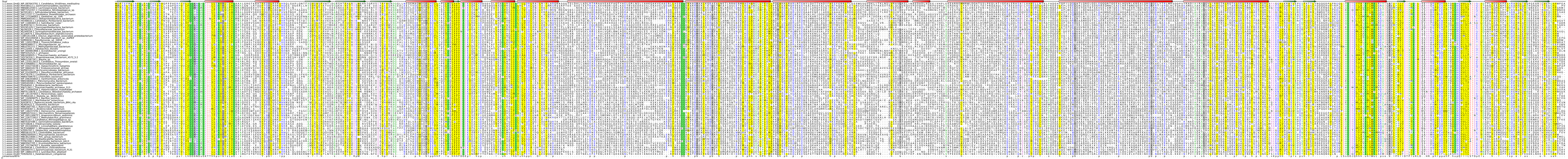

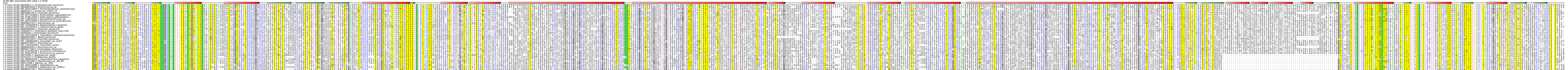
DndD-ABC associated with clade 1.2 DndE
final
1.2.assoc.DndD_BDF69811.1_Oscillospiraceae_bacterium
1.2.assoc.DndD_MBQ6943026.1_Ruminococcus_sp.
1.2.assoc.DndD_WP_058723687.1_Ruthenibacterium_lactatiformans
1.2.assoc.DndD_NBK94357.1_bacterium_1XD21-13
1.2.assoc.DndD_MBR3885705.1_Clostridia_bacterium
1.2.assoc.DndD_WP_007705406.1_Enterocloster_asparagiformis
1.2.assoc.DndD_WP_204613509.1_Defluviitalea_raffinosedens
1.2.assoc.DndD_WP_118642001.1_Pseudoflavonifractor_sp.
1.2.assoc.DndD_WP_087414569.1_Butyricicoccus_pullicaecorum
1.2.assoc.DndD_MBZ4662722.1_Caloramator_sp.
1.2.assoc.DndD_WP_079492854.1_Maledivibacter_halophilus
1.2.assoc.DndD_WP_161260521.1_Heliomicrobium_gestii
1.2.assoc.DndD_MBR2906464.1_Clostridia_bacterium
1.2.assoc.DndD_CDA09271.1_Clostridium_bartlettii_CAG:1329
1.2.assoc.DndD_ERI94636.1_Clostridiales_bacterium
1.2.assoc.DndD_WP_055070045.1_Clostridium_massiliamazoniense
1.2.assoc.DndD_MBQ4633985.1_Bacilli_bacterium
1.2.assoc.DndD_MBP3401449.1_Clostridia_bacterium
1.2.assoc.DndD_WP_158096446.1_Gemmiger_sp._An87
1.2.assoc.DndD_MBQ2673647.1_Prevotella_sp.
1.2.assoc.DndD_WP_216554428.1_Tissierella_carlieri
1.2.assoc.DndD_MBE6844503.1_Ruminococcus_sp.
1.2.assoc.DndD_MBS6677493.1_Clostridiales_bacterium
1.2.assoc.DndD_WP_010249534.1_Acetivibrio_cellulolyticus
1.2.assoc.DndD_WP_009244796.1_Ruminococcus_gnavus
1.2.assoc.DndD_ARF14005.1_Sporosarcina_ureae
1.2.assoc.DndD_KYD22017.1_Caldibacillus_debilis
1.2.assoc.DndD_WP_089830318.1_Halolactibacillus_halophilus
1.2.assoc.DndD_MBU7315389.1_Paenibacillus_sp._SM_69
1.2.assoc.DndD_NMH73566.1_Bacillus_sp._RO2
1.2.assoc.DndD_WP_085524486.1_Tuberibacillus_sp.
1.2.assoc.DndD_WP_212369044.1_Allobacillus_sp._SKP8-2
1.2.assoc.DndD_MBO8161740.1_Thermosipho_sp.
consensus/90%

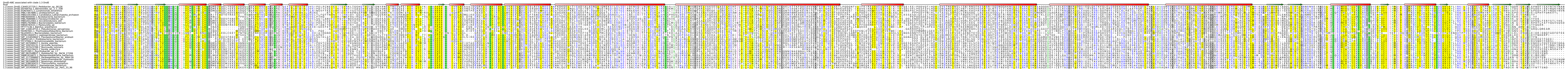
DndD-ABC associated with clade 1.3 DndE
final
1.3.assoc.DndD_CAH0137729.1_Pedobacter_sp._Bi126
1.3.assoc.DndD_EEY84702.1_Bacteroides_sp._2_1_33B
1.3.assoc.DndD_EON76971.1_Lunatimonas_lonarensis
1.3.assoc.DndD_MBC7367886.1_Undibacterium_sp.
1.3.assoc.DndD_MBD3343366.1_Candidatus_Lokiarchaeota_archaeon
1.3.assoc.DndD_MBI3649440.1_Acidobacteria_bacterium
1.3.assoc.DndD_MBL7689989.1_Flavipsychrobacter_sp.
1.3.assoc.DndD_MBO2012583.1_Siccationidurans_soli
1.3.assoc.DndD_MBR1792968.1_Bacteroidales_bacterium
1.3.assoc.DndD_MBS9783531.1_Pasteurella_sp.
1.3.assoc.DndD_PFY77136.1_Bacillus_wiedmannii
1.3.assoc.DndD_WP_116814715.1_Pseudomonas_aeruginosa
1.3.assoc.DndD_RKX67841.1_Thermodesulfobacteria_bacterium
1.3.assoc.DndD_NLJ20055.1_Bacteroidales_bacterium
1.3.assoc.DndD_MCB1194023.1_Leptospiraceae_bacterium
1.3.assoc.DndD_WP_131850662.1_Flaviaesturariibacter_aridisoli
1.3.assoc.DndD_NBP02145.1_Proteobacteria_bacterium
1.3.assoc.DndD_PIY37064.1_Armatimonadetes_bacterium
1.3.assoc.DndD_WP_007096427.1_Kordia_algicida
1.3.assoc.DndD_WP_109745131.1_Arcicella_aurantiaca
1.3.assoc.DndD_WP_207025789.1_Muricauda_lutimaris
1.3.assoc.DndD_WP_160129421.1_Kordia_antarctica
1.3.assoc.DndD_PJX21285.1_Psychrobacter_sp._L7
1.3.assoc.DndD_WP_062548657.1_Pedobacter_sp._PACM_27299
1.3.assoc.DndD_MBW1616871.1_Deltaproteobacteria_bacterium
1.3.assoc.DndD_WP_217789860.1_Parasegetibacter_sp._MAH-26
1.3.assoc.DndD_WP_013766332.1_Haliscomenobacter_hydrossis
1.3.assoc.DndD_WP_062588829.1_Roseivirga_ehrenbergii
1.3.assoc.DndD_WP_038030423.1_Thermonema_rossianum
1.3.assoc.DndD_MCB9310859.1_Lewinellaceae_bacterium
1.3.assoc.DndD_WP_101450655.1_Polaribacter_sp._Hel1_33_96
consensus/85%

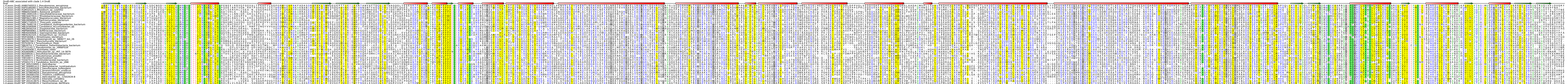
DndD-ABC associated with clade 1.4 DndE
final
1.4.assoc.DndD_KAB0740553.1_Pseudomonas_aeruginosa
1.4.assoc.DndD_KAF0189260.1_Desulfobulbaceae_bacterium
1.4.assoc.DndD_KQM32525.1_Rhizobium_sp._Leaf202
1.4.assoc.DndD_MBB0025848.1_Ralstonia_pickettii
1.4.assoc.DndD_MBC2695339.1_Desulfobacteraceae_bacterium
1.4.assoc.DndD_MBC6407914.1_Rhodobacteraceae_bacterium
1.4.assoc.DndD_MBF0621385.1_Magnetococcales_bacterium
1.4.assoc.DndD_MBI2808680.1_Planctomycetes_bacterium
1.4.assoc.DndD_MBK1716525.1_Thiocystis_violacea
1.4.assoc.DndD_MBL7648116.1_Candidatus_Hydrogenedentes_bacterium
1.4.assoc.DndD_MBL8390946.1_Candidatus_Accumulibacter_sp.
1.4.assoc.DndD_MBL8456254.1_Zoogloeaceae_bacterium
1.4.assoc.DndD_MBP8808961.1_Kofleriaceae_bacterium
1.4.assoc.DndD_MBV6440606.1_Saprospiraceae_bacterium
1.4.assoc.DndD_MBY6262325.1_Azospirillum_sp._412522
1.4.assoc.DndD_MSP34068.1_Rickettsiales_bacterium
1.4.assoc.DndD_MYG28479.1_Boseongicola_sp._SB0677_bin_26
1.4.assoc.DndD_NBO92768.1_Planctomycetia_bacterium
1.4.assoc.DndD_PDT43974.1_Sinorhizobium_fredii
1.4.assoc.DndD_PJB29752.1_Candidatus_Desantisbacteria_bacterium
1.4.assoc.DndD_PTT12142.1_Pseudomonas_sp._HMWF034
1.4.assoc.DndD_PZW40408.1_Humitalea_rosea
1.4.assoc.DndD_RDU60088.1_Helicobacter_sp._MIT_14-3879
1.4.assoc.DndD_RJQ58934.1_Desulfobacteraceae_bacterium
1.4.assoc.DndD_RLU09077.1_Pseudomonas_prosekii
1.4.assoc.DndD_TIW10977.1_Mesorhizobium_sp.
1.4.assoc.DndD_TXH47572.1_Burkholderiaceae_bacterium
1.4.assoc.DndD_VFK65173.1_Candidatus_Kentron_sp._UNK
1.4.assoc.DndD_WP_005219445.1_Helicobacter
1.4.assoc.DndD_WP_006891413.1_Methylobacter_tundripaludum
1.4.assoc.DndD_WP_015402928.1_Desulfocapsa_sulfexigens
1.4.assoc.DndD_WP_053939525.1_Amantichitinum_ursilacus
1.4.assoc.DndD_WP_079854659.1_Pseudomonas_aeruginosa
1.4.assoc.DndD_WP_093065250.1_Thiothrix_caldifontis
1.4.assoc.DndD_WP_095226498.1_Helicobacter_sp._12S02634-8
1.4.assoc.DndD_WP_153716245.1_Eikenella_corrodens
1.4.assoc.DndD_WP_201413875.1_Mesorhizobium_sp._J8
1.4.assoc.DndD_WP_235658035.1_Xanthomonas_perforans
consensus/80%

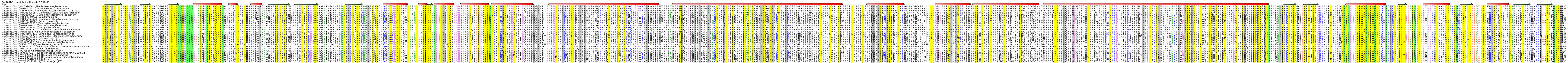
DndD-ABC associated with clade 1.5 DndE
final
1.5.assoc.DndD_HCO92856.1_Phycisphaerales_bacterium
1.5.assoc.DndD_HDR46165.1_Geoalkalibacter_subterraneus
1.5.assoc.DndD_KFB76726.1_Candidatus_Accumulibacter_sp._SK-02
1.5.assoc.DndD_MBD3339595.1_Candidatus_Lokiarchaeota_archaeon
1.5.assoc.DndD_MBD3807687.1_Epsilonproteobacteria_bacterium
1.5.assoc.DndD_MBF0234499.1_Desulfamplus_sp.
1.5.assoc.DndD_MBF0595632.1_Candidatus_Omnitrophica_bacterium
1.5.assoc.DndD_MBG2714038.1_Proteus_mirabilis
1.5.assoc.DndD_MBK8956063.1_Saprospiraceae_bacterium
1.5.assoc.DndD_MBR6091901.1_Bacteroidales_bacterium
1.5.assoc.DndD_MBS3952332.1_Methylomicrobium_sp.
1.5.assoc.DndD_MBU2541731.1_Candidatus_Omnitrophica_bacterium
1.5.assoc.DndD_MBW6485177.1_Syntrophobacterales_bacterium
1.5.assoc.DndD_MBZ4194743.1_Candidatus_Contendobacter_sp.
1.5.assoc.DndD_MCB5279572.1_Candidatus_Cloacimonetes_bacterium
1.5.assoc.DndD_MCC5465771.1_Pelosinus_sp._Bkl1
1.5.assoc.DndD_MCC6933030.1_Deltaproteobacteria_bacterium
1.5.assoc.DndD_NCA87337.1_Gammaproteobacteria_bacterium
1.5.assoc.DndD_NPV11873.1_Ignavibacteria_bacterium
1.5.assoc.DndD_OGX26181.1_Omnitrophica_WOR_2_bacterium_GWF2_38_59
1.5.assoc.DndD_PFT19404.1_Bacillus_thuringiensis
1.5.assoc.DndD_PQP85507.1_Paenibacillus_sp._AR247
1.5.assoc.DndD_RIA09372.1_Flavobacteriaceae_bacterium_MAR_2010_72
1.5.assoc.DndD_WP_020589863.1_Desulfobacter_curvatus
1.5.assoc.DndD_WP_045222648.1_Desulfonatronum_thioautotrophicum
1.5.assoc.DndD_WP_066618669.1_Rufibacter_roseus
1.5.assoc.DndD_WP_093191181.1_Thiocapsa_sp._KS1
consensus/100%

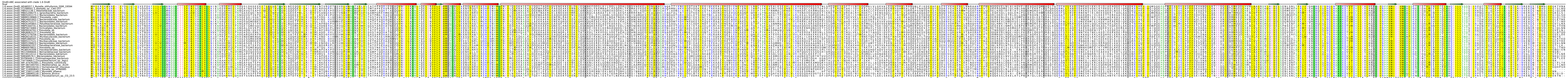
DndD-ABC associated with clade 1.6 DndE
final
1.6.assoc.DndD_AEI46557.1_Runella_slithyformis_DSM_19594
1.6.assoc.DndD_CCZ99501.1_Alistipes_sp._CAG:157
1.6.assoc.DndD_HBH21139.1_Rikenellaceae_bacterium
1.6.assoc.DndD_MBD1208271.1_Ignavibacteria_bacterium
1.6.assoc.DndD_MBD5201173.1_Bacteroidales_bacterium
1.6.assoc.DndD_MBM0128969.1_Prevotella_copri
1.6.assoc.DndD_MBO4641603.1_Bacteroidaceae_bacterium
1.6.assoc.DndD_MBO4751577.1_Bacteroidales_bacterium
1.6.assoc.DndD_MBO5345803.1_Paludibacteraceae_bacterium
1.6.assoc.DndD_MBP5344731.1_Bacteroidales_bacterium
1.6.assoc.DndD_MBP5673069.1_Victivallales_bacterium
1.6.assoc.DndD_MBP8934702.1_Prevotella_sp.
1.6.assoc.DndD_MBQ6063127.1_Prevotella_sp.
1.6.assoc.DndD_MBQ7279258.1_Bacteroidales_bacterium
1.6.assoc.DndD_MBQ9218151.1_Muribaculaceae_bacterium
1.6.assoc.DndD_MBR1880597.1_Prevotella_sp.
1.6.assoc.DndD_MBR5117121.1_Muribaculaceae_bacterium
1.6.assoc.DndD_MBR5199623.1_Bacteroidales_bacterium
1.6.assoc.DndD_MBR6041557.1_Paludibacteraceae_bacterium
1.6.assoc.DndD_MBR6979822.1_Prevotella_sp.
1.6.assoc.DndD_MCB9191508.1_Flavobacteriales_bacterium
1.6.assoc.DndD_MCC5946644.1_Bernardetiaceae_bacterium
1.6.assoc.DndD_MCC8114112.1_Bacteroidales_bacterium
1.6.assoc.DndD_RMG69579.1_Bacteroidetes_bacterium
1.6.assoc.DndD_RYF90449.1_Chitinophagaceae_bacterium
1.6.assoc.DndD_TXF74969.1_Chryseobacterium_sp._legu1
1.6.assoc.DndD_WP_074761591.1_Prevotella_ruminicola
1.6.assoc.DndD_WP_087340754.1_Mediterranea_sp._An20
1.6.assoc.DndD_WP_089334216.1_Hymenobacter_mucosus
1.6.assoc.DndD_WP_114069643.1_Runella_sp._HYN0085
1.6.assoc.DndD_WP_158211148.1_Myroides_phaeus
1.6.assoc.DndD_WP_188465130.1_Bizionia_arctica
1.6.assoc.DndD_WP_209548599.1_Flavobacterium_sp._CG_23.5
consensus/100%

CxC-ABC associated with clade 2.1 DndE
final

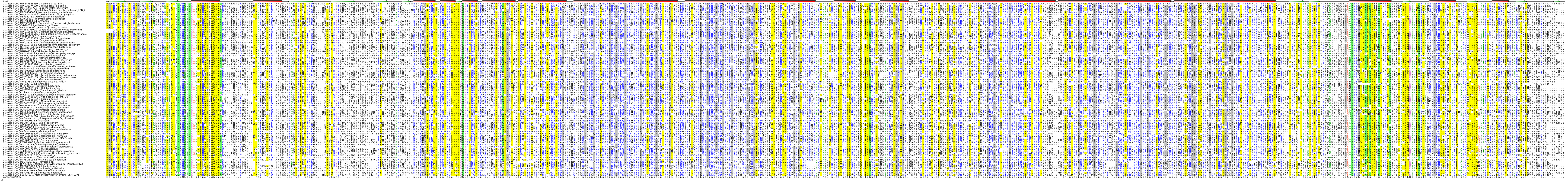

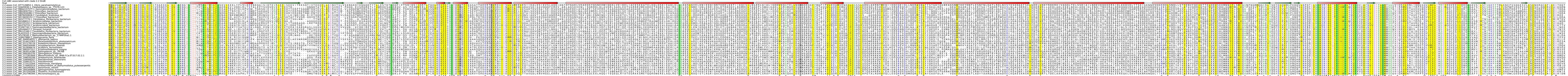
CxC-ABC associated with clade 2.2 DndE
final
2.2.assoc.CxC_EIT7126852.1_Vibrio_parahaemolyticus
2.2.assoc.CxC_GHJ45930.1_Catellatospora_sp._TT07R-123
2.2.assoc.CxC_MBA4157577.1_Gemmatimonadetes_bacterium
2.2.assoc.CxC_MBI2830759.1_Chloroflexi_bacterium
2.2.assoc.CxC_MBI2918288.1_Chloroflexi_bacterium
2.2.assoc.CxC_MBT6050828.1_Candidatus_Scalindua_sp.
2.2.assoc.CxC_MCC6630152.1_Chloroflexi_bacterium
2.2.assoc.CxC_MXV77779.1_Candidatus_Poribacteria_bacterium
2.2.assoc.CxC_MXV88012.1_Acidimicrobiales_bacterium
2.2.assoc.CxC_MXY25152.1_Acidobacteria_bacterium
2.2.assoc.CxC_MXZ76733.1_Acidimicrobiia_bacterium
2.2.assoc.CxC_PCH97742.1_Gammaproteobacteria_bacterium
2.2.assoc.CxC_QDV48457.1_Gimesia_fumaroli
2.2.assoc.CxC_RKU15340.1_Candidatus_Poribacteria_bacterium
2.2.assoc.CxC_RLA53325.1_Gammaproteobacteria_bacterium
2.2.assoc.CxC_SFU78165.1_Methylobacterium_sp._174MFSha1.1
2.2.assoc.CxC_UOR07888.1_Qipengyuania_flava
2.2.assoc.CxC_WP_006954031.1_Idiomarina_baltica
2.2.assoc.CxC_WP_014415363.1_Pararhodospirillum_photometricum
2.2.assoc.CxC_WP_037278119.1_Rubellimicrobium_mesophilum
2.2.assoc.CxC_WP_046556448.1_Arsukibacterium_ikkense
2.2.assoc.CxC_WP_052606674.1_Acidithrix_ferrooxidans
2.2.assoc.CxC_WP_090207327.1_Ectothiorhodospira_magna
2.2.assoc.CxC_WP_093014188.1_Sphingobium_sp._YR768
2.2.assoc.CxC_WP_099185376.1_Sphingobium_fuliginis
2.2.assoc.CxC_WP_127281909.1_Mesorhizobium_sp._M4B.F.Ca.ET.017.02.2.1
2.2.assoc.CxC_WP_148026208.1_Streptomyces_lavendulae
2.2.assoc.CxC_WP_156049207.1_Pseudomonas_oleovorans
2.2.assoc.CxC_WP_159453275.1_Pseudomonas
2.2.assoc.CxC_WP_172515304.1_Halomonas_meridiana
2.2.assoc.CxC_WP_174482019.1_endosymbiont_of_Bathymodiolus_puteoserpentis
2.2.assoc.CxC_WP_180349362.1_Aeromonas_veronii
2.2.assoc.CxC_WP_184160869.1_Sphingomonas_kyeonggiensis
2.2.assoc.CxC_WP_207250208.1_Thiothrix_fructosivorans
2.2.assoc.CxC_WP_242796394.1_Micromonospora_sp.
consensus/100%

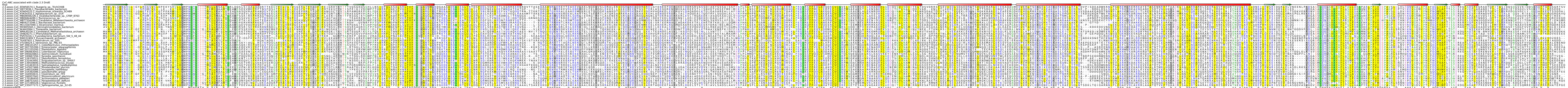
CxC-ABC associated with clade 2.3 DndE
final
2.3.assoc.CxC_EEW56574.1_Ruegeria_sp._TrichCH4B
2.3.assoc.CxC_HIE73036.1_Flavobacteriales_bacterium
2.3.assoc.CxC_KDA04225.1_Hyphomonas_oceanitis_SCH89
2.3.assoc.CxC_KVK54332.1_Agrobacterium_sp._D14
2.3.assoc.CxC_MBD8566226.1_Oxalobacteraceae_sp._CFBP_8763
2.3.assoc.CxC_MBE6844499.1_Ruminococcus_sp.
2.3.assoc.CxC_MBT7556386.1_Candidatus_Woesearchaeota_archaeon
2.3.assoc.CxC_MBY6273346.1_Bacillaceae_bacterium
2.3.assoc.CxC_MCC4307090.1_Alcanivorax_marinus
2.3.assoc.CxC_MXW05565.1_Gemmatimonadetes_bacterium
2.3.assoc.CxC_NLW42064.1_Tissierellia_bacterium
2.3.assoc.CxC_NMA30226.1_Candidatus_Methanofastidiosa_archaeon
2.3.assoc.CxC_OHB26271.1_Phenylobacterium_sp.
2.3.assoc.CxC_PSP25416.1_Cyanobacteria_bacterium_SW_5_48_44
2.3.assoc.CxC_RMG31909.1_Euryarchaeota_archaeon
2.3.assoc.CxC_TKX87932.1_Halorubrum_sp._SS5
2.3.assoc.CxC_VTU38524.1_Variovorax_sp._PBL-E5
2.3.assoc.CxC_WP_006101257.1_Coleofasciculus_chthonoplastes
2.3.assoc.CxC_WP_007705399.1_Enterocloster_asparagiformis
2.3.assoc.CxC_WP_027255200.1_Planktothrix_agardhii
2.3.assoc.CxC_WP_090063043.1_Celeribacter_neptunius
2.3.assoc.CxC_WP_095820863.1_Salimicrobium_humidisoli
2.3.assoc.CxC_WP_100667294.1_Kyrpidia_spormannii
2.3.assoc.CxC_WP_126378521.1_Desulfovibrio_ferrophilus
2.3.assoc.CxC_WP_131443491.1_Exiguobacterium_sp._SH0S7
2.3.assoc.CxC_WP_139559690.1_Methylotetracoccus_oryzae
2.3.assoc.CxC_WP_141463019.1_Salinadaptatus_halalkaliphilus
2.3.assoc.CxC_WP_147627466.1_Rhodanobacter_glycinis
2.3.assoc.CxC_WP_148510066.1_Hungatella_hathewayi
2.3.assoc.CxC_WP_154392096.1_pseudomallei_group
2.3.assoc.CxC_WP_164950631.1_Clostridium_sp._FP2
2.3.assoc.CxC_WP_166937042.1_Rhizomicrobium_electricum
2.3.assoc.CxC_WP_170180450.1_Actinomadura_pelletieri
2.3.assoc.CxC_WP_188753163.1_Parapedobacter_defluvii
2.3.assoc.CxC_WP_227289328.1_Vagococcus_fluvialis
2.3.assoc.CxC_WP_235077172.1_Sphingomonas_sp._S2-65
consensus/85%

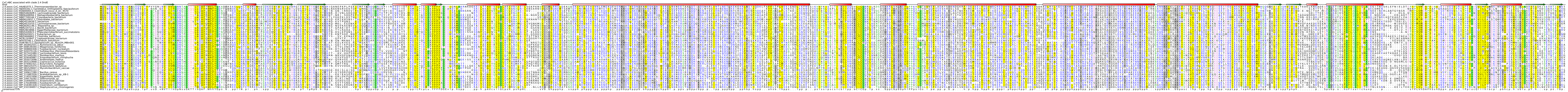
CxC-ABC associated with clade 2.4 DndE
final

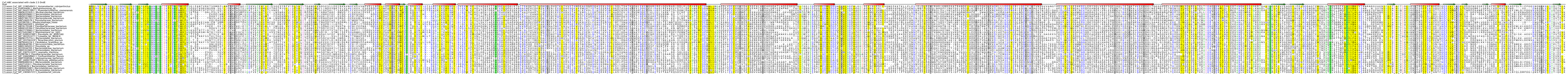
CxC-ABC associated with clade 2.5 DndE
final
2.5.assoc.CxC_WP_119654943.1_Hymenobacter_rubripertinctus
2.5.assoc.CxC_PVW16408.1_Marixanthomonas_sp.
2.5.assoc.CxC_WP_044217697.1_Phaeodactylibacter_xiamenensis
2.5.assoc.CxC_MBO6251935.1_Muribaculaceae_bacterium
2.5.assoc.CxC_MBQ0029662.1_Bacteroidales_bacterium
2.5.assoc.CxC_MBQ0123962.1_Bacteroidales_bacterium
2.5.assoc.CxC_MBQ7361753.1_Bacteroidaceae_bacterium
2.5.assoc.CxC_MBF1060579.1_Prevotellaceae_bacterium
2.5.assoc.CxC_QHT68700.1_Rhodocytophaga_rosea
2.5.assoc.CxC_MBP3482005.1_Alistipes_sp.
2.5.assoc.CxC_MBT6170168.1_Flavobacteriaceae_bacterium
2.5.assoc.CxC_MBR6105394.1_Paludibacteraceae_bacterium
2.5.assoc.CxC_MBO7278965.1_Bacteroidales_bacterium
2.5.assoc.CxC_MCC5452867.1_Rheinheimera_sp._UJ51
2.5.assoc.CxC_WP_028910788.1_Prevotella_sp._AGR2160
2.5.assoc.CxC_WP_182251577.1_Colwellia_sp._BRX8-9
2.5.assoc.CxC_MBQ6175774.1_Bacteroidales_bacterium
2.5.assoc.CxC_WP_193706319.1_Tenacibaculum_piscium
2.5.assoc.CxC_MBT6045224.1_Candidatus_Scalindua_sp.
2.5.assoc.CxC_MBS0646785.1_Verrucomicrobia_bacterium
2.5.assoc.CxC_MBR1395053.1_Prevotella_sp.
2.5.assoc.CxC_MCF0244705.1_Bacteroidaceae_bacterium
2.5.assoc.CxC_MBR5353965.1_Bacteroidales_bacterium
2.5.assoc.CxC_WP_238751103.1_Lewinella_maritima
2.5.assoc.CxC_PIZ08784.1_Flavobacteriaceae_bacterium
2.5.assoc.CxC_WP_106658982.1_Confluentibacter_flavum
2.5.assoc.CxC_MBK7058596.1_Leptospiraceae_bacterium
2.5.assoc.CxC_WP_168927508.1_Nitrincola_alkalilacustris
2.5.assoc.CxC_MBK8489224.1_Bacteroidetes_bacterium
2.5.assoc.CxC_HAQ71016.1_Flavobacteriales_bacterium
2.5.assoc.CxC_TAE69639.1_Bacteroidetes_bacterium
2.5.assoc.CxC_MBI9062568.1_Marinilabiliaceae_bacterium
2.5.assoc.CxC_MCA0235876.1_Bacteroidetes_bacterium
2.5.assoc.CxC_WP_138365721.1_Dyadobacter_luticola
consensus/90%

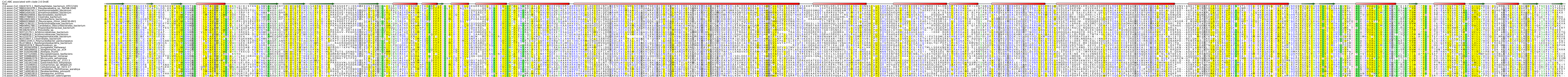
CxC-ABC associated with clade 2.6 DndE
final
2.6.assoc.CxC_EAV47472.1_Methylophilales_bacterium_HTCC2181
2.6.assoc.CxC_MBD2261078.1_Pseudanabaena_sp._FACHB-2040
2.6.assoc.CxC_MBK9494745.1_Xanthomonadales_bacterium
2.6.assoc.CxC_MBN1459330.1_Armatimonadetes_bacterium
2.6.assoc.CxC_MBO6024858.1_Bacteroidales_bacterium
2.6.assoc.CxC_MBQ7788503.1_Clostridia_bacterium
2.6.assoc.CxC_MBU2601349.1_Actinobacteria_bacterium
2.6.assoc.CxC_MBW4536682.1_Pleurocapsa_minor_HA4230-MV1
2.6.assoc.CxC_MBX7136151.1_Fimbriimonadaceae_bacterium
2.6.assoc.CxC_MBX9941788.1_Candidatus_Obscuribacterales_bacterium
2.6.assoc.CxC_MCC6497155.1_Propionibacteriaceae_bacterium
2.6.assoc.CxC_MCD8291970.1_Prevotella_sp.
2.6.assoc.CxC_MXY12179.1_Acidimicrobiaceae_bacterium
2.6.assoc.CxC_MYA85818.1_Acidimicrobiaceae_bacterium
2.6.assoc.CxC_NLG31070.1_Acholeplasmataceae_bacterium
2.6.assoc.CxC_NLK72607.1_Clostridiales_bacterium
2.6.assoc.CxC_QWD11445.1_Polynucleobacter_paneuropaeus
2.6.assoc.CxC_RLA73804.1_Epsilonproteobacteria_bacterium
2.6.assoc.CxC_RWG01078.1_Mesorhizobium_sp.
2.6.assoc.CxC_WP_002603058.1_Hungatella_hathewayi
2.6.assoc.CxC_WP_009188452.1_Streptomyces_sp._e14
2.6.assoc.CxC_WP_019246398.1_Alistipes_ihumii
2.6.assoc.CxC_WP_027120481.1_Mycoplasmopsis_lipofaciens
2.6.assoc.CxC_WP_028884893.1_Teredinibacter_turnerae
2.6.assoc.CxC_WP_046662379.1_Microcystis_aeruginosa
2.6.assoc.CxC_WP_093491749.1_Streptomyces_sp._2112.3
2.6.assoc.CxC_WP_102342200.1_Galactobacillus_timonensis
2.6.assoc.CxC_WP_123296452.1_Comamonas_sp._BIGb0124
2.6.assoc.CxC_WP_130102694.1_Cellulomonas_sp._HLT2-17
2.6.assoc.CxC_WP_148136049.1_Candidatus_Formimonas_warabiya
2.6.assoc.CxC_WP_149918019.1_Parabacteroides_johnsonii
2.6.assoc.CxC_WP_203652815.1_Demequina_activiva
2.6.assoc.CxC_WP_235228001.1_Oscillibacter_valericigenes
consensus/80%

P-loop-Kinase + Helical-(Lid)-domain + α+β domain (Canonical DndF)

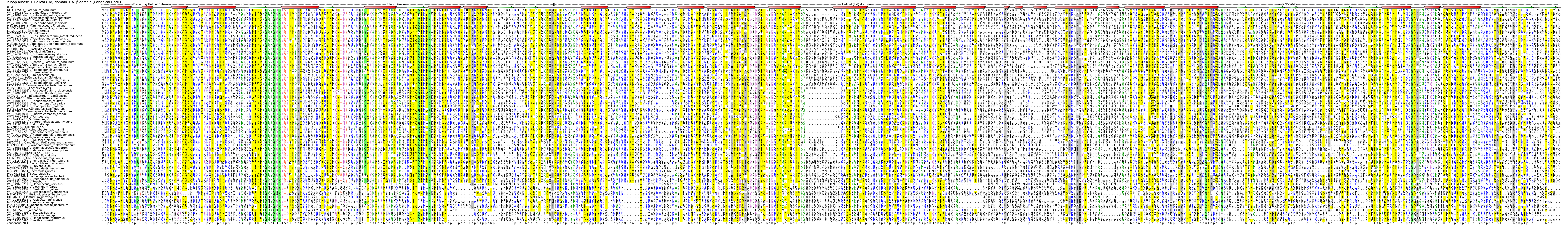

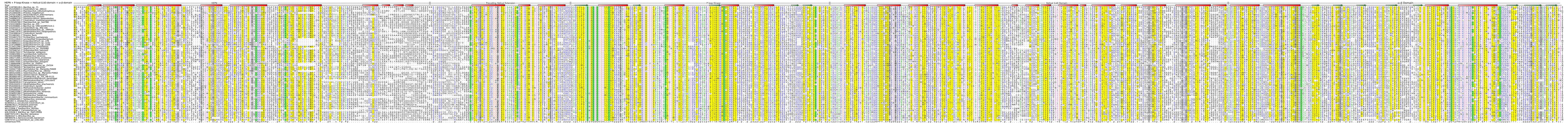
HEPN + P-loop-Kinase + Helical-(Lid)-domain + α+β-domain
final
HEPN
Preceding Helical Extension
P-loop Kinase
Helical (Lid) Domain
α+β Domain

NERD + PKinase(Inactive) + PKinase(Active) + P-loop-Kinase + Helical-(Lid)-domain + $\alpha+\beta$-domain

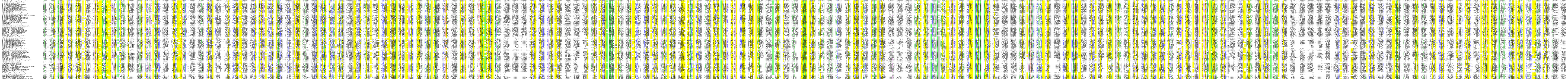

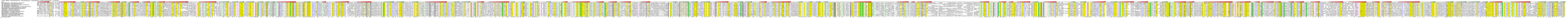
wHTH+ NERD + PKinase(Inactive) + PKinase(Active) + P-loop-Kinase + Helical-(Lid)-domain + α+β-domain
final
WP_008514337.1_Dethiobacter_alkaliphilus
WP_244084474.1_Desulforhabdus_sp.
WP_206707460.1_Koleobacter_methoxysyntrophicus
WP_163338765.1_Desulfopila_sp.
WP_072867803.1_Desulfofundulus_thermosubterraneus
WP_015050782.1_Thermacetogenium_phaeum
OQB76594.1_Deltaproteobacteria_bacterium
OPL12258.1_Firmicutes_bacterium
NLM38753.1_Firmicutes_bacterium
MCF7708072.1_Verrucomicrobia_bacterium
MCD5401678.1_candidate_division
MBT3376948.1_Lentisphaerae_bacterium
HHX42347.1_Armatimonadetes_bacterium
HHV07442.1_Firmicutes_bacterium
AEG14183.1_Desulfofundulus_kuznetsovii
consensus/100%

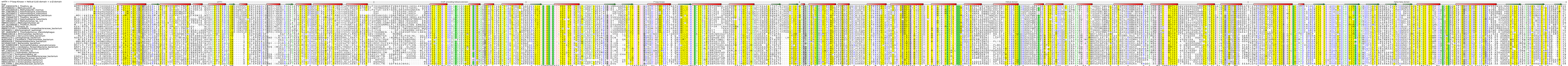
wHTH + P-loop-Kinase + Helical-(Lid)-domain + α+β-domain
final
WP_236501676.1_Thiothrix_sp.
MCF7988202.1_Methylovulum_sp.
WP_020160010.1_Methylobacter_marinus
WP_238639625.1_Halorhodospira_halochloris
WP_162549411.1_Halorhodospira_halochloris
MBU2569721.1_Gammaproteobacteria_bacterium
WP_156946702.1_Thiothrix_lacustris
WP_238631702.1_Halorhodospira_halochloris
WP_196436507.1_Methylomonas_sp._LL1
MBE0472024.1_Methyloprofundus_sp.
WP_162596646.1_Thiofilum_flexile
QVL49769.1_Thiocapsa_sp.
MBK8753705.1_Candidatus_Competibacteraceae_bacterium
SDY40803.1_Allochromatium_warmingii
WP_164654367.1_Thiorhodococcus_mannitoliphagus
MBQ6338418.1_Kiritimatiellae_bacterium
MCC5851092.1_Verrucomicrobia_bacterium
NMA47279.1_Lentisphaerae_bacterium
NLW19095.1_Candidatus_Cloacimonetes_bacterium
MRR06439.1_Deltaproteobacteria_bacterium
MCK9482318.1_Bacteroidia_bacterium
WP_028893208.1_Syntrophorhabdus_aromaticivorans
MBT4917030.1_Candidatus_Peregrinibacteria_bacterium
MBA3016879.1_Desulfobacteraceae_bacterium
WP_176015023.1_Victivallis_sp.
OGO79372.1_Clostridiales_bacterium
OPY77734.1_Syntrophorhabdus_sp.
VEN73292.1_uncultured_Desulfobacteraceae_bacterium
RPH52307.1_Desulfobacteraceae_bacterium
MBR4258821.1_Kiritimatiellae_bacterium
MBR4189797.1_Kiritimatiellae_bacterium
HHL33999.1_Desulfobulbaceae_bacterium
consensus/90%

GNAT + P-loop-Kinase + Helical-(Lid)-domain + α+β-domain final

```
MBR3362218.1_Lachnospiraceae_bacterium          MLNKEIPFRPRLEGDFRVRFYNAVSEIGEETTSMEIEQIATREINWVEDDCSFNLGQRKKYRAVWMLFRDLIRASWKACYRDGVL [illegible]
WP_236343992.1_Paenibacillus_sp.                MLNRTISFRPKLEGQFRMRFYNAVSLIQETTSIANIEQIVDDEVSWVENDCLVNMAQRKKYRAVWLLFRDLIRASWKACYREGVL [illegible]
WP_227111828.1_Flavonifractor_plautii           MINREIPFRPRLEGEFRVRFYNAASEITEKTPTLTIARIAEREIEWVEKDCQYNIEQRKKYRAVWFLFRDLIRASWKACYRNGVI [illegible]
WP_201069153.1_Thiorhodovibrio_winogradskyi     ---MIVPIRPPLDGACAERFLAFSRCLARTSAESAREALLADEIGWARDALSSATFAFDTYEAAVRVLADLHRLGWQVRESGGIE [illegible]
WP_195457371.1_Flavonifractor_plautii           MLNREIPFRPRLEGDFRIRFYNAVSRITENTTLADIENIADEEIKWVTSECTFNLDQRKKYRAVWFLFRDLIHASWKALYRDGVL [illegible]
WP_184337298.1_Prosthecobacter_vanneervenii     ---MNITIHPALDGIAAERLRDFAIRL-ADGNEGAVQEFVKSEAAWVSRTKALNGQ-SCSYEASARLLADLRLLKWKVRADSGIE [illegible]
WP_132379986.1_Muricomes_intestini              MLNREIPFRPKLEGDFRIRFYNAVSSITEQTSLFEIEQIANDEIRWVQDECTYNLEQRKKYRAIWMLFRDLTRASWKACYRDGVL [illegible]
WP_086001533.1_Plesiocystis_pacifica            -MAQTVRYSPKIKKAAYTKLTGMVRA------ADGPLQLQASLIDFAE------GARSKRQRAAALVLADLVRQGWDVHVRDKEI [illegible]
WP_062286127.1_Moorella_mulderi                 -MSDFIPFRPNLQGPFLERFLSKAVELCNSEPRTTVDSIAINEYRWATETVLVNPRQRRIYRAVWLFLRDLLRAGWTCRWYDSNI [illegible]
RZO60483.1_Sandaracinaceae_bacterium            GSEEILRFAPKVRADRYQEMVALVAE------GATGVALQRQLLAYAEMVSRSEGTAGESHRAAALVLVDLLRQGWDVFLDGGAI [illegible]
OQB13591.1_Firmicutes_bacterium                 MLNREIPFRPKLEGDFRIRFYNAVSVINEETSPLEIEKISNNEIMWVENVCTYNLGQRKKYRAVWMLFRDLTRASWKACYREGVL [illegible]
MYH44924.1_Acidimicrobiaceae_bacterium          LE---RRFRPTLSFGYQQDLLALVQDLDGRPEIVRIRSSIDTAETHERGVHPDDIEPTIVYLASLRVLRDLTLQGWVAGADDDGV [illegible]
MYD24497.1_Gemmatimonadetes_bacterium           LE---RRFRPRLSFGFQQDLLALAQDLEGRLEVSRIRSSIDTAETHERGVPPDSLGPTMVFLASLRVLRDLTLQGWIAGADCDGI [illegible]
MCD7785256.1_Oscillospiraceae_bacterium         MTNRTIPFRPKLEGEFRTRFYTVVQQINEDTKTSELEEIIAQEVHWTEHECTVNLVQRKKYRAVWMLLRDLMRASWKACYREGVL [illegible]
MCA9607806.1_Myxococcales_bacterium             LDDQPVHFAPKIRGDRYEQLVDLVEE------GGDTLALQRRLVAYAEVIAREDGDAGAHERAAVLVLADLLRQGWEVFLDGGSI [illegible]
MBZ4195022.1_Candidatus_Contendobacter_sp.      ---MIVPIDPPLQGIAIDRFHALAKALTHSQREGDLQVLLAEDLAWAHAHLDPETNAFVAYEAAARVLVDLVRLGWQAREEGGIE [illegible]
MBW8380978.1_Youngiibacter_sp.                  MINREIPFRPRLEGNFRIRFYNAVSAINDNTSVIDIEKIANQEIDWVENECAYNLVQRKKYRAIWLLFRDLIRASWRACYREGVL [illegible]
MBS4007144.1_Clostridium_sp.                    MINRTIPYRPKLEGVFRKRFYATAAIINDT----NLEDLISRELKWVEEECDFNIKQRRKYRAVWLLLRDLVRASWNATFRSGVL [illegible]
MBR4653016.1_Kiritimatiellae_bacterium          QFPRIVPFIPNLEGVYNQRFYSLVSSWRHGENQDIHVAALEKEVSDLEKSASQKTEQYIRYRAILLLLRDLLTTTWAANFEYGQL [illegible]
MBQ6952697.1_Bacteroidales_bacterium            NLIKEVAFSPKLEGLFRTRFYEKAQLLHDIDST---DSISKEEVQWVENECTTNLRQRKKIYRAVWFLLRDLVKAHWVPRFRDGKL [illegible]
MBP2018538.1_Symbiobacterium_terraclitae        -MS-FYPYRPKLNGTFLERFHAKTFQLCQSAPSVEIRRIALEEYEWATHEVMINPQQRRIYRASWLLLRDLLVAGWSCRWFDGEI [illegible]
MBL9155175.1_Verrucomicrobiales_bacterium       ---MHIEIHPNVEGIAAERLLDFAASLAADTSSARLPALVEQEAAWARRTPHLNGQ-TKAYEASMRLLADLRALKWRVRADRGIE [illegible]
MBF0370482.1_Magnetococcales_bacterium          WHPMIVPIRANLEGSAADRFLALARTLARAPKEEDRMALLASELEWASSHISLEDQ-RLAFEAAVRVMFDLERLGWQVREEGGVE [illegible]
KWX82524.1_Paenibacillus_riograndensis          MLNRAIPFRPRLEGMFRVRFYDAVSSINENTPLTYIEHLVEKEIFWVEKECLVNLIQRKKYRAIWLLLRDLVRASWRACFREGVL [illegible]
KLR61496.1_Actinobacteria_bacterium             LE---RRFRPRLSFGYLQDLHALVQHLADRFEVHLIRSCIDTAEAWDLGVGSDDLAPTIVYLASLRVLRDLTLQGWEAGVDDDGV [illegible]
CAA9344327.1_uncultured_Chloroflexia_bacterium  PETSIRRFRPVLTLNYQRELHSLARHVQGLKPARVLADQIAKVSDYASGLKRRDVDPTARYVATLQVLRDLLQQGWELRSDDEGL [illegible]
consensus/100%                                  .......h.s.lp.....ph...s.................p.................A.hbhh.DL....W......... [illegible]
```

ZnR + P-loop-Kinase + Helical-(Lid)-domain + α+β-domain

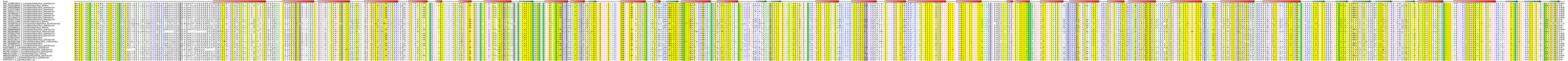

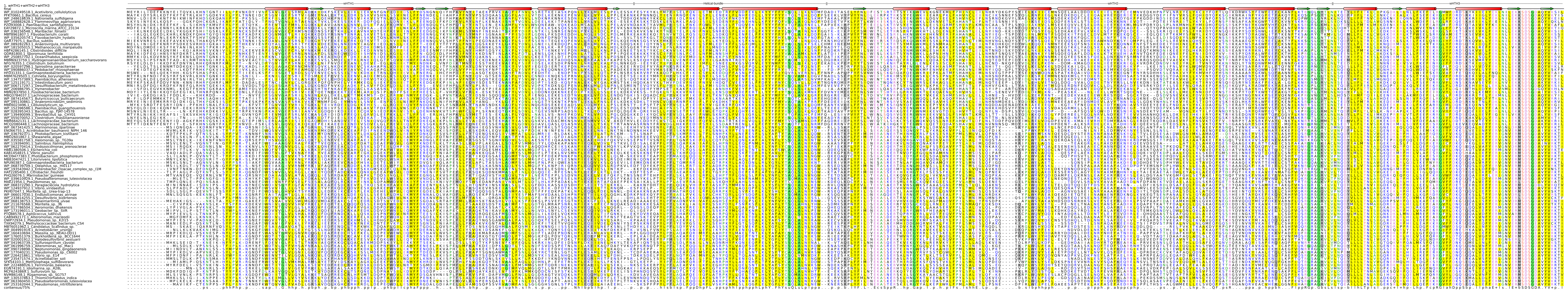
1. wHTH1+wHTH2+wHTH3
final
wHTH1
Helical bundle
wHTH2
wHTH3
consensus/75%

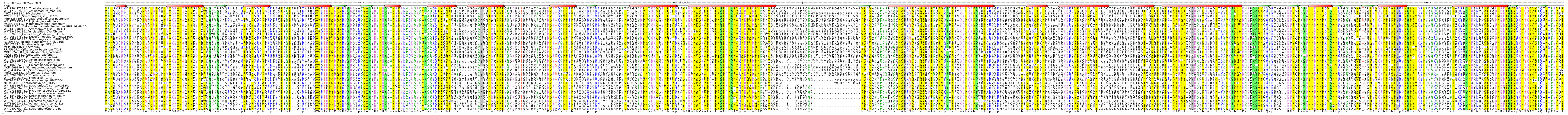

2. wHTH1+wHTH2+wHTH3
final
WP_156427528.1_Thiohalocapsa_sp._ML1
WP_177287693.1_Actinomadura_madurae
MBZ2184883.1_Bryobacter_sp.
MYT21751.1_Streptomyces_sp._SID7760
MBM4257408.1_Deltaproteobacteria_bacterium
WP_127779522.1_Lujinxingia_sediminis
MCA9214811.1_Planctomycetales_bacterium
OGP93186.1_Deltaproteobacteria_bacterium_RBG_16_48_10

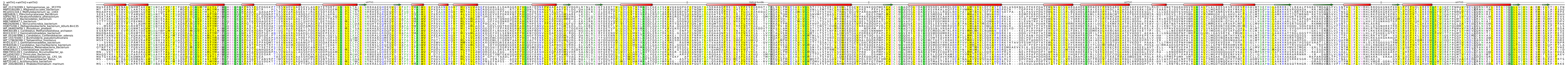
3. wHTH1+wHTH2+wHTH3
final
WP_212742099.1_Sphingomonas_sp._3F27F9
MBF0160289.1_Magnetococcales_bacterium
MCJ2031318.1_Methylobacterium_sp._J-043
HBJ34826.1_Planctomycetaceae_bacterium
SDF81782.1_Paraburkholderia_phenazinium
HCA80931.1_Bacteroidetes_bacterium
MBE7489047.1_bacterium
MBI2926046.1_Verrucomicrobia_bacterium
OQB75208.1_Deltaproteobacteria_bacterium_ADurb.Bin135
WP_064829458.1_Rhizobium_phaseoli
NMC60189.1_Candidatus_Methanofastidiosa_archaeon
MXX13157.1_Gemmatimonadetes_bacterium
WP_051497896.1_Candidatus_Contendobacter_odensis
WP_155755067.1_Burkholderia_pseudomultivorans
WP_179636398.1_Sphaerotilus_montanus
MYD13237.1_Gemmatimonadetes_bacterium
MYB40548.1_Candidatus_Saccharibacteria_bacterium
RTL43634.1_Candidatus_Melainabacteria_bacterium
NMW20620.1_Chlorobiaceae_bacterium
MBK7955139.1_Candidatus_Accumulibacter_sp.
MCL6562302.1_Firmicutes_bacterium
OYT73418.1_Chloracidobacterium_sp._CP2_5A
WP_138085267.1_Phragmitibacter_flavus
NBT25140.1_Actinomycetia_bacterium
WP_200286169.1_Rhabdochromatium_marinum
consensus/100%

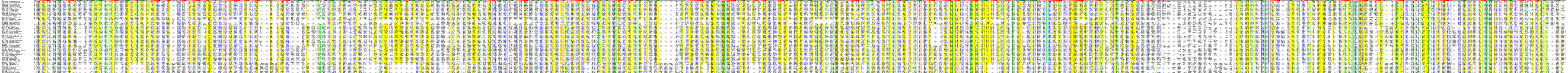
iSF2/Swi2-Helicase+HTH+Beta-sandwich+Beta-sandwich+TRD+MTase(TRD-insert)+PDDEXK-REase+HerA/FtsK-ATPase
iSF2/Swi2-helicase
final
consensus/70%

iSF2/Swi2-Helicase+HTH+TRD+MTase(TRD-insert)+PDDEXK-REase+HerA/FtsK-ATPase

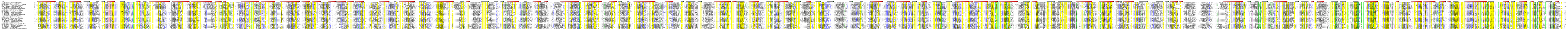

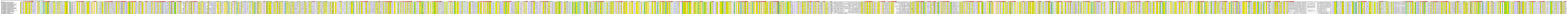
iSF2/Swi2-Helicase+HTH+Beta-sandwich+Beta-sandwich+TRD+MTase(TRD-insert)+PDDEXK-REase+Sigma-HTH+HerA/FtsK-ATPase
final
WP_069582521.1_Aliivibrio_fischeri
WP_179897467.1_Pantoea_sp._WMus005
KZY78560.1_Oleiphilus_sp._HI0068
WP_171041044.1_Pseudoalteromonas_aurantia
WP_010380876.1_Pseudoalteromonas_rubra
WP_187501329.1_Pantoea_agglomerans
WP_232735484.1_Pseudoalteromonas_sp._3J6
WP_165902219.1_Vibrio_crassostreae
WP_165926642.1_Vibrio_crassostreae
WP_169044072.1_Pseudoalteromonas_arctica
WP_088208772.1_Enterobacter_cancerogenus
WP_126495433.1_Enterobacter_hormaechei
WP_071922656.1_Enterobacter_kobei
WP_181416034.1_Gilliamella_apicola
WP_219233675.1_Shewanella_sp._NKUCC01_JLK
WP_172397937.1_Gilliamella_apicola
WP_182440111.1_Colwellia_sp._RSH04
MBL4798492.1_Oleispira_sp.
WP_197412121.1_Colwellia_sp._TT2012
WP_063668659.1_Aliivibrio_fischeri
consensus/100%
